# Formal Single Atom Editing of the Glycosylated Natural Product Fidaxomicin Improves Acid Stability and Retains Antibiotic Activity

**DOI:** 10.1021/jacsau.4c00206

**Published:** 2024-05-21

**Authors:** Isabella Ferrara, Gleb A. Chesnokov, Silvia Dittmann, Olivier Blacque, Susanne Sievers, Karl Gademann

**Affiliations:** † Department of Chemistry, University of Zurich, Winterthurerstrasse 190, 8057 Zürich, Switzerland; ‡ Department for Microbial Physiology and Molecular Biology, Institute of Microbiology, Center for Functional Genomics of Microbes, University of Greifswald, Felix-Hausdorff-Strasse 8, 17489 Greifswald, Germany

**Keywords:** semi synthesis, natural products, single atom exchange, carbohydrates, glycomimetics, thioglycosides

## Abstract

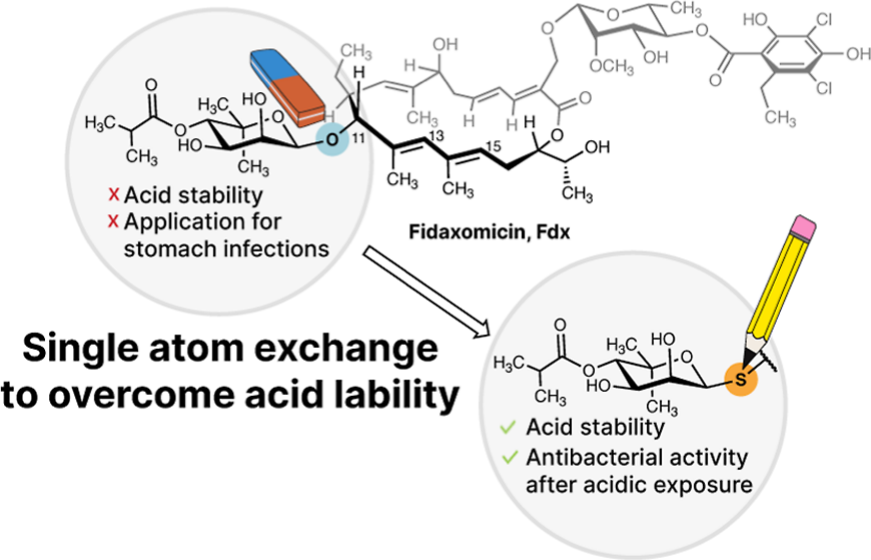

Fidaxomicin (Fdx) constitutes a glycosylated natural product with excellent antibacterial activity against various Gram-positive bacteria but is approved only for *Clostridioides difficile* infections. Poor water solubility and acid lability preclude its use for other infections. Herein, we describe our strategy to overcome the acid lability by introducing acid-stable S-linked glycosides. We describe the direct, diastereoselective modification of unprotected Fdx without the need to avoid air or moisture. Using our newly established approach, Fdx was converted to the single atom exchanged analogue S-Fdx, in which the acid labile O-glycosidic bond to the noviose sugar was replaced by the acid stable S-glycosidic bond. Studies of the antibacterial activity of a structurally diverse set of thioglycoside derivatives revealed high potency of acyl derivatives of S-Fdx against *Clostridioides difficile* (MIC range: 0.12–4 μg/mL) and excellent potency against *Clostridium perfringens* (MIC range: 0.06–0.5 μg/mL).

## Introduction

**Figure 1:**
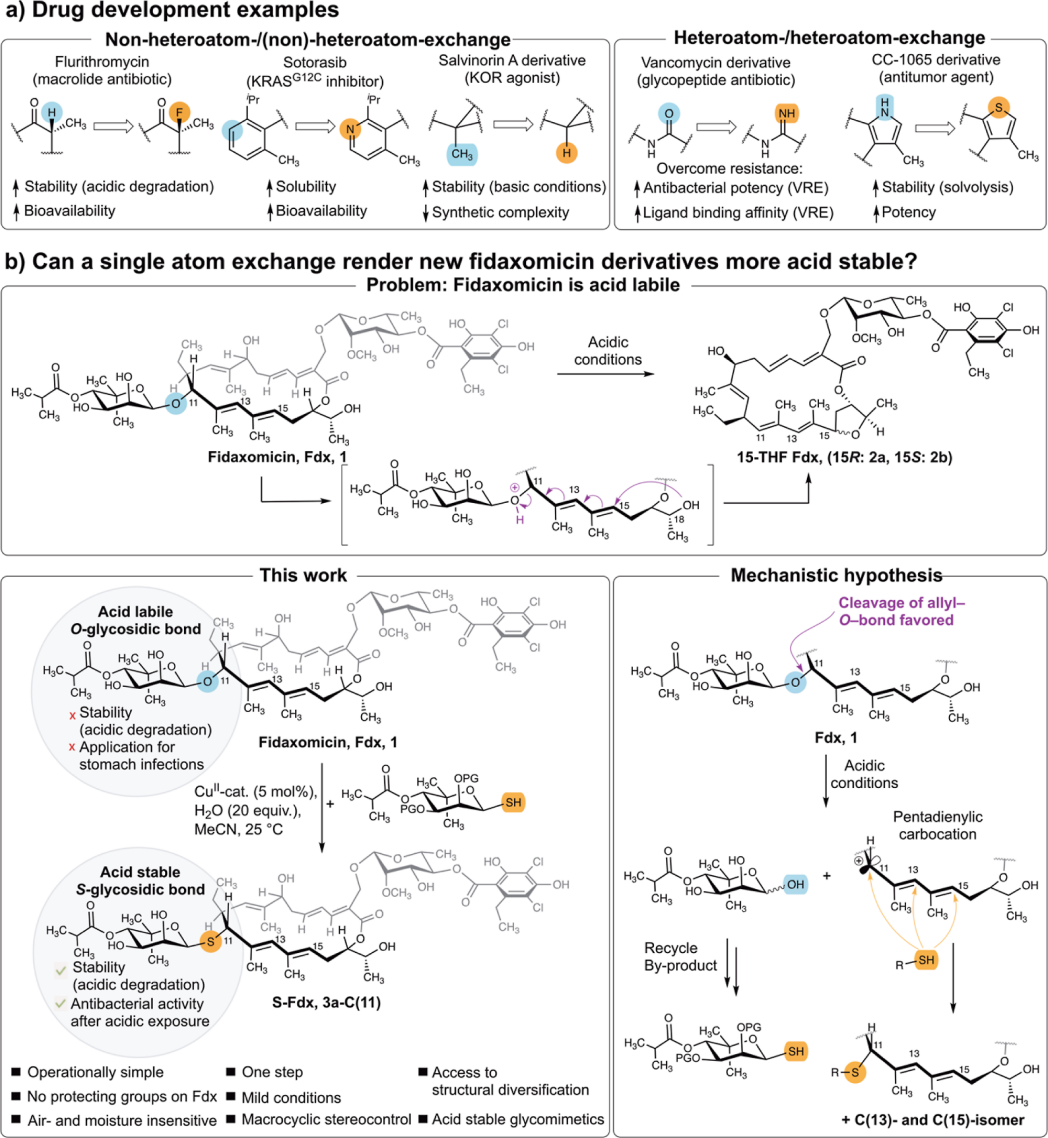
Effect of single atom exchanges in biologically active compounds. (a) Selected examples from drug development with formal single atom exchanges of nonheteroatoms (left) or heteroatoms (right). Note that the examples also include modifications of more than one atom (e.g., CH_3_/H). (b) Overview of the acid lability of fidaxomicin (Fdx, **1**), as suggested by Hattori et al.^18^ (top), and our strategy to perform a single atom O-/S-exchange in Fdx to access acid stable derivatives (bottom). KRAS^G12C^: Kirsten rat sarcoma viral oncogene with the G12C mutation. KOR is a k-opioid receptor. VRE Vancomycin-resistant enterococci. Protecting group (PG) (acetonide or acetyl).

Exchanging a single atom in a molecule can significantly impact its biological and chemical characteristics.^1,2^ Systematic investigation on the effect of a single atom swap enables us to understand the structure–activity relationship (SAR) of biologically active molecules in detail and allows precise structure-based design in a medicinal chemistry context.^1^ Selected examples from the field of drug development display the plethora of applications for single atom modifications and their beneficial effect on the stability (e.g., flurithromycin,^3^ CC-1065 analogue,^1^ and salvinorin A derivative),^4^ solubility (e.g., sotorasib),^5,6^ biological activity (e.g., vancomycin derivatives^1^ and CC-1065 analogue),^1^ or synthetic complexity (e.g., salvinorin A derivative)^4^ (Figure 1a).^1^ As single-atom exchanges can exert tremendous impact on the physicochemical properties of molecules, methods to enable precise, late-stage single atom molecular editing are highly sought-after.^2,6^ In this work, we present our strategy for a formal single atom O-/S-exchange in the natural product antibiotic fidaxomicin (Fdx, **1**) to overcome its acid lability with the ultimate goal to extend its application portfolio.

The glycosylated macrolactone Fdx (also known as lipiarmycin A3 or tiacumicin B)^7^ was approved in 2011 for the treatment of *Clostridioides difficile* (*C. diff.*) infections (CDI) that constitute one of the most common nosocomial infections.^8−13^ Alarmingly, the repertoire of therapeutic options for CDI is very limited,^8,9,13,14^ and the first Fdx resistant clinical *C. diff.* isolates have been reported.^15^ Furthermore, Fdx has promising in vitro activities against *Clostridium perfringens* (*C. perfringens*), *Staphylococcus aureus*, and *Mycobacterium tuberculosis*.^7,9,14,16^ However, the clinical use is restricted to CDI, mainly due to unfavorable physicochemical properties such as poor aqueous solubility^7,17^ and lack of acid stability.^17,18^ After oral administration, Fdx acts locally in the intestine. While local, intestinal application does not require high solubility and acid stability, these are of critical importance to extend the application portfolio beyond CDI. As such, acid stability is a necessity for gastric application, especially when using gastroretentive dosage forms, but it also impacts the bioavailability with the membrane permeability of Fdx being higher in the low pH of the stomach.^19^ Taken together, the emerging resistance development in CDI, with potential larger applications for other pathogens, however, limited by poor acid stability and solubility, all provides a strong rationale for the development of second generation analogues. Therefore, we aim at synthesizing Fdx derivatives via chemical modifications with the main objective to improve its properties.

Bacterial infections of the stomach are rare as the harsh acidic environment constitutes a protective barrier.^20^ Therefore, infections are commonly associated with prior weakening of the protective measures, for example, by gastroduodenal surgery or immunocompromised states.^20−23^*C. perfringens* exploits these predisposing circumstances and possesses a very high virulence due to its fast growth.^21,22^ An infection with this Gram-positive, anaerobic, and gas-forming bacterium can manifest itself in emphysematous gastritis or the wound infection gas gangrene, among other pathophysiological conditions.^20−24^ While the incidence rate is relatively low, both diseases are associated with high lethality.^21,23,24^ Treatment options involve antibiotic therapy and surgical intervention.^23−25^ The development of new therapies for *C. perfringens* infections is crucial given the development of resistances by *C. perfringens* against currently used antibiotics (clindamycin, penicillin, metronidazole, etc.)^26−29^ and the prevalence of penicillin allergies. With the promising in vitro activity of Fdx,^16^ the synthesis of acid-stable glycomimetics constitutes a viable strategy to extend the portfolio of treatment options against *C. perfringens*.

The biological activity of Fdx is based on its binding to a unique binding site of the bacterial RNA polymerase, thereby interfering with the transcription initiation.^30−32^ The noviose-derived sugar at C(11) of Fdx is indispensable for the biological activity.^7,30^ However, the O-glycosidic bond to the noviose moiety causes Fdx to be acid labile.^18^ The O-atom readily undergoes protonation, rendering the noviose a good leaving group. Under acidic conditions, the products of a formal intramolecular S_N_2″-attack of the C(18)–OH at C(15) were observed (**2**, Figure 1b).^18^ In presence of nucleophilic solvents (i.e., MeOH), the corresponding MeOH adduct was reported.^18^ As S-atoms are less basic than their O-counterparts, we envisaged that a single atom exchange in the O-glycosidic bond could overcome the acid lability (Figure 1b).^33−36^ To access the O-/S-exchanged product, we made use of the acid-mediated reactivity toward nucleophiles to introduce thiols. Despite initially an acid-mediated S_N_2″-type reaction of Fdx was suggested,^18^ we envisioned an S_N_1-type mechanism to be feasible. While the prevalent mechanism for acid-mediated heterolysis of the C–O bond in O-glycosidic linkages involves the formation of an oxocarbenium ion,^36,37^ we suggest the allylic nature of the aglycone in Fdx to favor the formation of a carbenium ion at the aglycone. Further, the formation of the oxocarbenium ion is disfavored by the presence of an electron-withdrawing β C–O bond,^38^ rendering its formation relatively slow.^36,37,39^ For aglycones that can form a stabilized carbocation upon aglycone-O-bond fission, faster hydrolysis rates were observed.^39,40^ We therefore suggest the formation of a pentadienylic carbocation following C–O-bond fission in Fdx under acidic conditions. Herein, we present our strategy to intercept acidic degradation by trapping the proposed carbocation with thiol nucleophiles (Figure 1b). A 1-thionoviose derivative as the nucleophile gave access to the single atom exchanged S-analogue of Fdx (S-Fdx, **3a-C(11)**) in a single, operationally simple step. Moreover, the intrinsic high nucleophilicity of thiols allowed the reactions to be conducted directly on unprotected Fdx, effectively competing with intramolecular reactions with OH-groups. This strategy presents a way to access structurally diverse thioglycoside derivatives of Fdx, while also introducing a more acid stable S-glycosidic bond.

## Results and Discussion

### Proof of Concept

**Figure 2:**
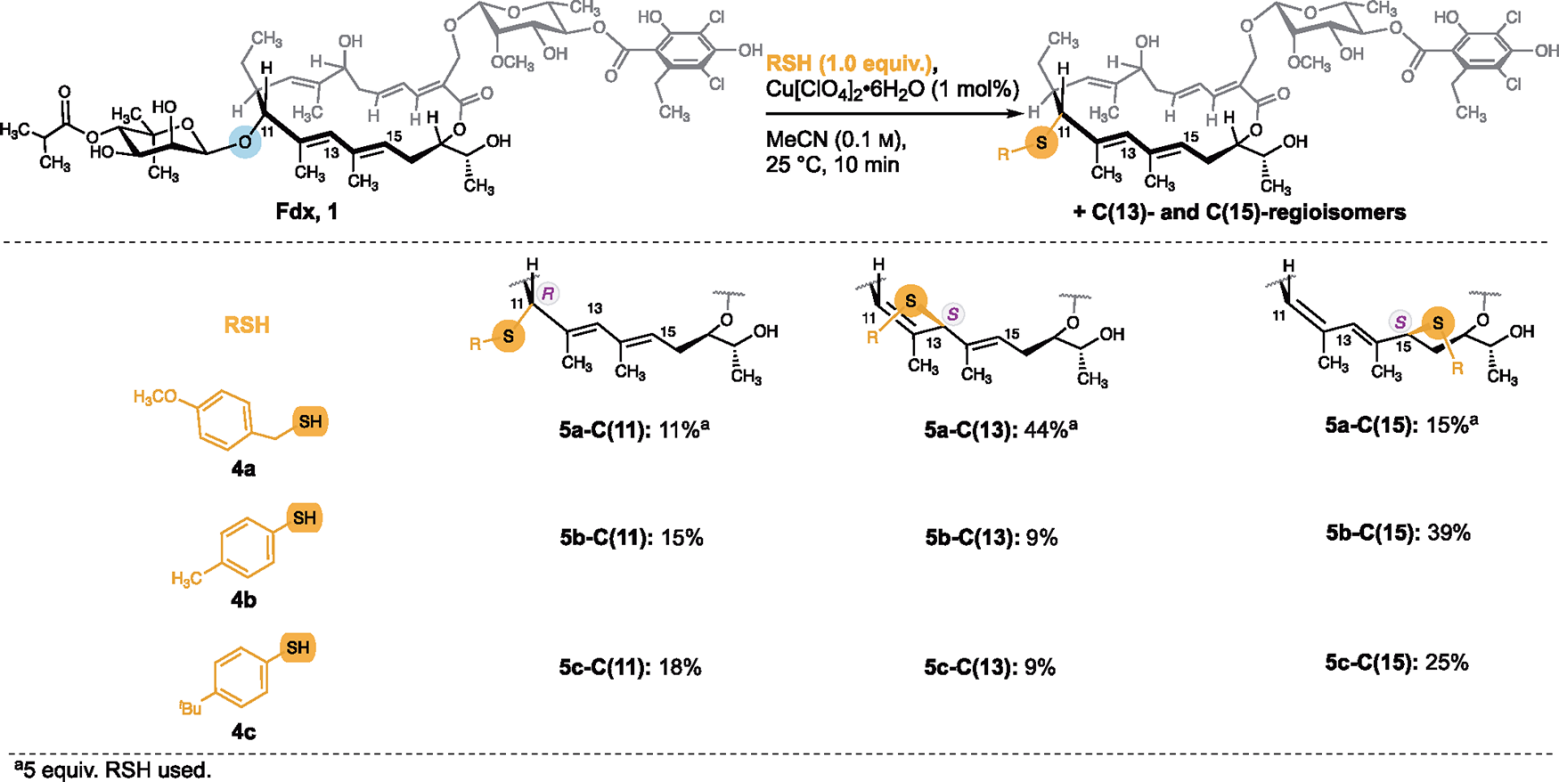
Reactivity of Fdx toward thiol nucleophiles using benzylic and aromatic thiol model substrates.

We started out to investigate the reactivity of Fdx toward thiol nucleophiles under Lewis acidic (LA) conditions using benzylic and aromatic thiol nucleophiles as model substrates. Despite we aimed at synthesizing S-Fdx by using a 1-thionoviose derivative, 1-thiosugars do not constitute the ideal substrates to study the feasibility of introducing thiol nucleophiles in all three regioisomeric positions. The commercial availability of 1-thiosugars is scarce, demanding prior synthesis. Further, potential epimerization of the anomeric thiol could complicate the analysis of the reaction outcome. Therefore, as a proof of concept we studied the reactivity of Fdx with 4-methoxybenzyl mercaptan (**4a**). Preliminary screening of reaction conditions was carried out (see Tables S1–S3) which indeed revealed the formation of the three regioisomeric products **5a-C(11)**, **5a-C(13)**, and **5a-C(15)** (Figure 2). Copper(II) perchlorate (Cu[ClO_4_]_2_·6H_2_O) at an LA loading of 1 mol % in MeCN resulted in the complete conversion of Fdx within 10 min at 25 °C. To employ the conditions for thiosugars, we aimed at keeping the reaction time short to avoid potential epimerization at elevated reaction times under LA conditions. While initial screens were performed using an excess of thiol (5.0 equiv) to compete with intramolecular side reactions, stoichiometric amounts were chosen in later experiments for reasons of operational simplicity and to avoid the use of excess amounts of precious 1-thiosugar. These conditions were successfully applied for aromatic thiols as nucleophiles (**5b**–**c**, Figure 2). The absolute configuration was assigned by 1- and 2-D NMR analysis and corroborated by DP4+ calculations (see SI_DP4+).^41^ The reactions proceeded with high diastereoselectivity at the newly formed stereogenic centers, providing solely the epimer corresponding to an attack at the outer face of the π-system. The diastereoselectivity is likely caused by macrocyclic stereocontrol. Importantly, for the C(11)-isomer, retention of configuration (R) was obtained compared to Fdx. With the promising result that thiols can be introduced diastereoselectively in the C(11)-, C(13)-, and C(15)- positions, we turned our attention on 1-thiosugars. Using the conditions specified above with 1-thio-β-d-glucose sodium salt did not lead to product formation (see Table S4).

However, the per-O-acetylated 1-thio-β-d-glucose **6a** could be successfully employed in the reaction (see Tables S5 and S6), although separation of the regioisomers proved troublesome. Next, we investigated the configurational stability of the per-O-acylated 1-thiosugars under the reaction conditions. Treating 1-thiosugar **6a** with 1 mol % Cu[ClO_4_]_2_·6H_2_O in MeCN did not lead to anomeric epimerization on the reaction time scale, also not after addition of acetic acid to mimic the acidic conditions in the presence of Fdx (p*K*_a_ 6.8^17^) (see Figures S1 and S2). Thus, introducing the β-anomer of 1-thiosugars allows us to control the configuration of the glycosidic linkage, which proved challenging to access for glycosylation of the aglycone.^42^ Therefore, we next aimed at synthesizing a per-O-protected 1-thio-β-d-noviose derivative.

### Synthesis of 1-Thionoviose Derivative by Recycling the Reaction Byproduct

**Figure 3:**
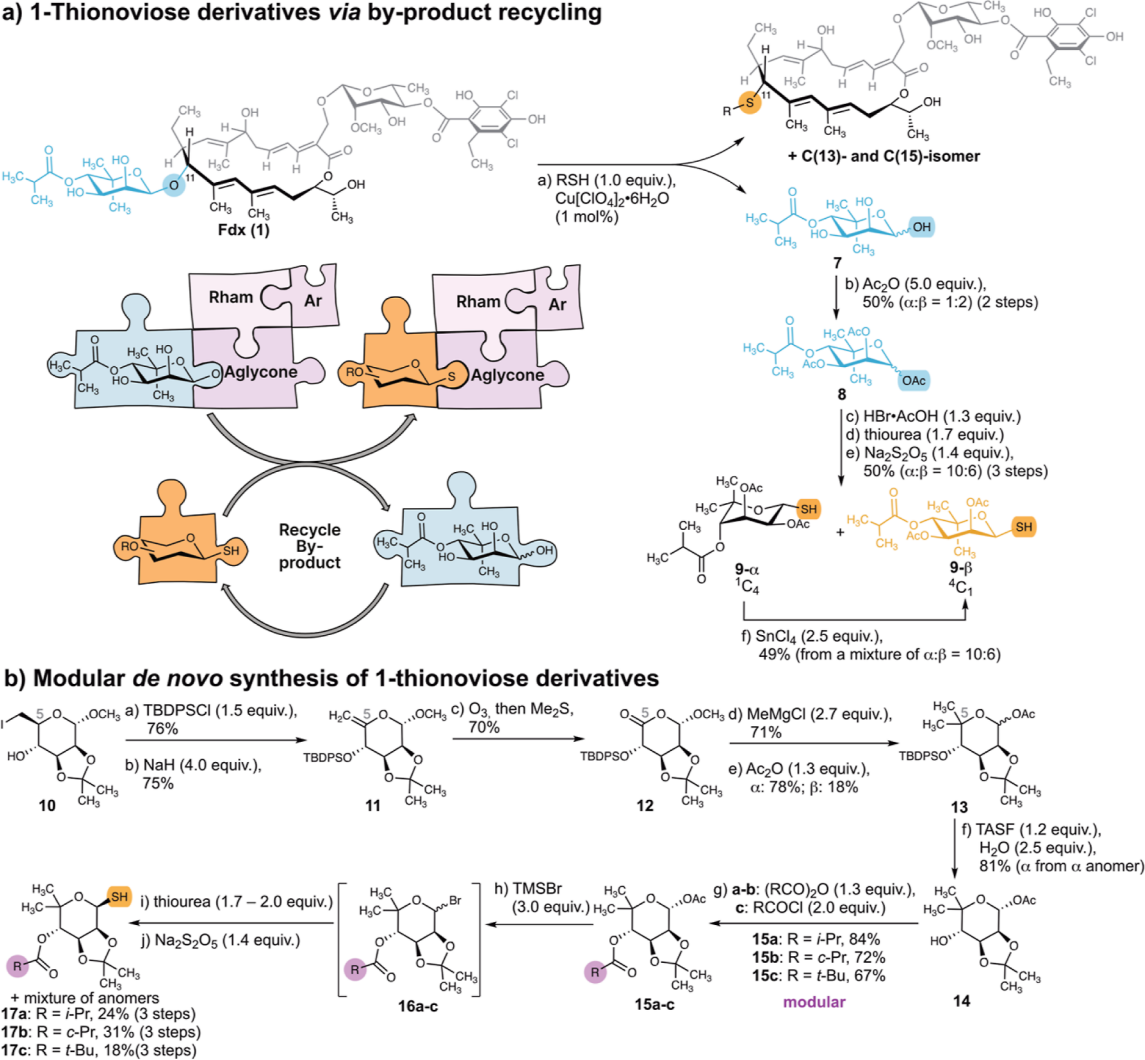
Strategies to access 1-thionoviose. (a) Isolation and functionalization of the noviose byproduct. Reagents and conditions: (a) Cu[ClO_4_]_2_·6H_2_O (1 mol %), RSH (1.0 equiv), and MeCN (0.1–0.5 M), 25 °C, 10–30 min; (b) Ac_2_O (5.0 equiv), pyridine (0.4 M), 0 to 25 °C, 20 h, 50% (α/β = 1:2) (2 steps); (c) HBr·AcOH (1.3 equiv), CH_2_Cl_2_ (0.4 M), 0 °C, 1.5 h; (d) thiourea (1.7 equiv), acetone (0.2 M), 70 °C, 2 h; (e) Na_2_S_2_O_5_ (1.4 equiv), CH_2_Cl_2_/H_2_O (3:2, 0.1 M), 70 °C, 2 h, 50% (α/β = 10:6) (3 steps); (f) SnCl_4_ (2.5 equiv), CH_2_Cl_2_ (0.3 M), 25 °C, 21 h, 49% (from a mixture α/β = 10:6). Rham Rhamnose. Ar Dichloro-homoorsellinate. (b) Modular de novo synthesis of thionoviose derivatives. Reagents and conditions: (a) TBDPSCl (1.5 equiv), Im (2.0 equiv), CH_2_Cl_2_ (0.2 M), 25 °C, 19 h, 76%; (b) NaH (4.0 equiv), THF/DMF 2:1 (0.03 M), 0 to 25 °C, 17 h, 75%; (c) O_3_, CH_2_Cl_2_/MeOH 1:1 (0.04 M), −78 °C, 30 min, then Me_2_S, −78 to 25 °C, 1 h, 70%; (d) MeMgCl (2.7 equiv), TMEDA (3.3 equiv), THF (0.03 M), −78 to 25 °C, 4.5 h, 71%; (e) Ac_2_O (1.3 equiv), pyridine (0.4 M), 0 to 25 °C, 17 h, α: 78%, β: 18%; (f) TASF (1.2 equiv), H_2_O (2.5 equiv), DMF (0.1 M), 25 °C, 7 h, 81% (α from α anomer); (g) pyridine (0.4 M), 0 to 25 °C, 24 h; (a) (*i*-PrCO)_2_O (1.3 equiv), 84%; (b) (*c*-PrCO)_2_O (1.3 equiv), 72%; (c) *t*-BuCOCl (2.0 equiv), 67%; (h) TMSBr (3.0 equiv), CH_2_Cl_2_ (0.3–0.4 M), 0 °C, 7.0–7.5 h; (i) thiourea (1.7–2.0 equiv), acetone, 60 °C, 15–16 h; and (j) Na_2_S_2_O_5_ (1.4 equiv), CH_2_Cl_2_/H_2_O 3:2, 60 °C, 3 h; (a) 24% β, 9%(α + β) (3 steps), (b) 31% β, 15%(α + β) (3 steps), and (c) 18% β, 18%(α + β) (3 steps).

To study the effect of exchanging an O- for a S-glycosidic linkage on the chemical and biological properties of Fdx derivatives, we envisaged the synthesis of S-Fdx from a 1-thionoviose derivative. Noviose is not commercially available. However, the byproduct of the LA-catalyzed reaction of Fdx with thiols is the noviose moiety that is cleaved off from the natural product (Figures 1b and 3a). Isolation of 4-*O*-isobutyryl noviose **7** from the aqueous phase, followed by acetylation, afforded per-*O*-acylated noviose **8** as a mixture of anomers (α/β = 1:2). The acylated pyranose **8** was then reused to access the 1-thiosugar, analogous to previously reported strategies.^43^ Noteworthy, the intermediately formed glycosyl bromide proved to be highly air and moisture sensitive. Due to the limited stability, the freshly prepared noviosyl bromide was directly treated with thiourea, followed by reduction with sodium metabisulfite to provide the per-O-acylated 1-thionoviose **9** in a yield of 50% (over 3 steps) as an inseparable anomeric mixture (α/β = 10:6). The mixture was subjected to SnCl_4_ mediated epimerization^43^ to provide 1-β-thionoviose. Following the substitution reaction with Fdx, basic methanolysis to deprotect the OAc groups, however, also resulted in the removal of the isobutyrate moiety. While reusing the noviose byproduct presents an elegant strategy to access 1-thionoviose derivatives, orthogonal protecting groups are necessary to selectively free the *syn*-diol leaving the 4-*O*-isobutyrate intact.

### De Novo Synthesis of 1-Thionoviose Derivatives

We next aimed at developing a de novo synthesis of 1-thionoviose derivatives starting from commercially available α-d-methoxy mannose. In addition, orthogonal protection groups provide access to a modular route to synthesize 4-*O*-acyl derivatives of thionoviose. This is of particular interest as the 4-*O*-isobutyryl moiety of noviose contributes significantly to the biological activity.^9^ The key challenge starting from α-d-methoxy mannose is to introduce the *gem*-dimethyl group at C(5), which we envisioned to perform via a Grignard reaction of the lactone, as reported by Hedberg et al.^44^ We decided to use an acetonide functionality for orthogonal protection of the 2,3-*syn* diol, as this can be readily removed under the LA conditions of the substitution reaction at Fdx. We initiated our synthesis with the preparation of the literature known iodide **10**(45−48) (Figure 3b). *tert*-Butyldiphenylsilyl (TBDPS) protection of the C(4)–OH group proceeded smoothly in 76% yield. Elimination of the iodide afforded *exo*-methylene **11** in a yield of 75%. Ozonolysis of the olefin **11** provided the lactone **12** in a yield of 70% which sets the starting point for the key Grignard reaction. The Grignard reaction with 2.7 equiv of methylmagnesium chloride (MeMgCl) gave access to the C-skeleton of the targeted noviose derivative in 71% yield. Next, the anomeric OH-group of the hemiacetal was acetylated to afford compound **13** in a yield of 96% and a diastereoselectivity of ca. 8:2 (α/β). Due to the acid and base sensitivity of compound **13**, the TBDPS deprotection proved challenging. Classical conditions such as tetrabutylammonium fluoride or HF·pyridine did not yield satisfactory results. However, the tris(dimethylamino)sulfonium difluorotrimethylsilicate (TASF) reagent, in the presence of water, allowed us to carry out the TBDPS deprotection under mild conditions with a yield of 81%. The C(4)–OH group was then acylated to provide the 4-*O*-isobutyryl-, 4-*O*-cyclopropanoyl-, and 4-*O*-pivaloyl-noviose derivatives **15a**–**c**. Finally, the anomeric acetate was converted to the thiol in a sequence similar to that used before. However, due to the acid-sensitive nature of the acetonides **15a**–**c**, bromotrimethyl silane (TMSBr) was used to form the glycosyl bromide. The freshly prepared air- and moisture-sensitive glycosyl bromide was then subjected to substitution with thiourea and subsequent reduction using sodium metabisulfite. Thereby, the acetonide-protected 4-*O*-acyl-1-thionoviose derivatives **17a**–**c** were accessed as anomeric mixtures enriched in the targeted β-anomer. Separation by column chromatography afforded the β-anomers **17a**–**c** (X-ray structures available) in yields ranging from 18 to 31% over 3 steps along with a mixture of anomeric epimers.

### Synthesis of S-Fdx Derivatives

**Figure 4:**
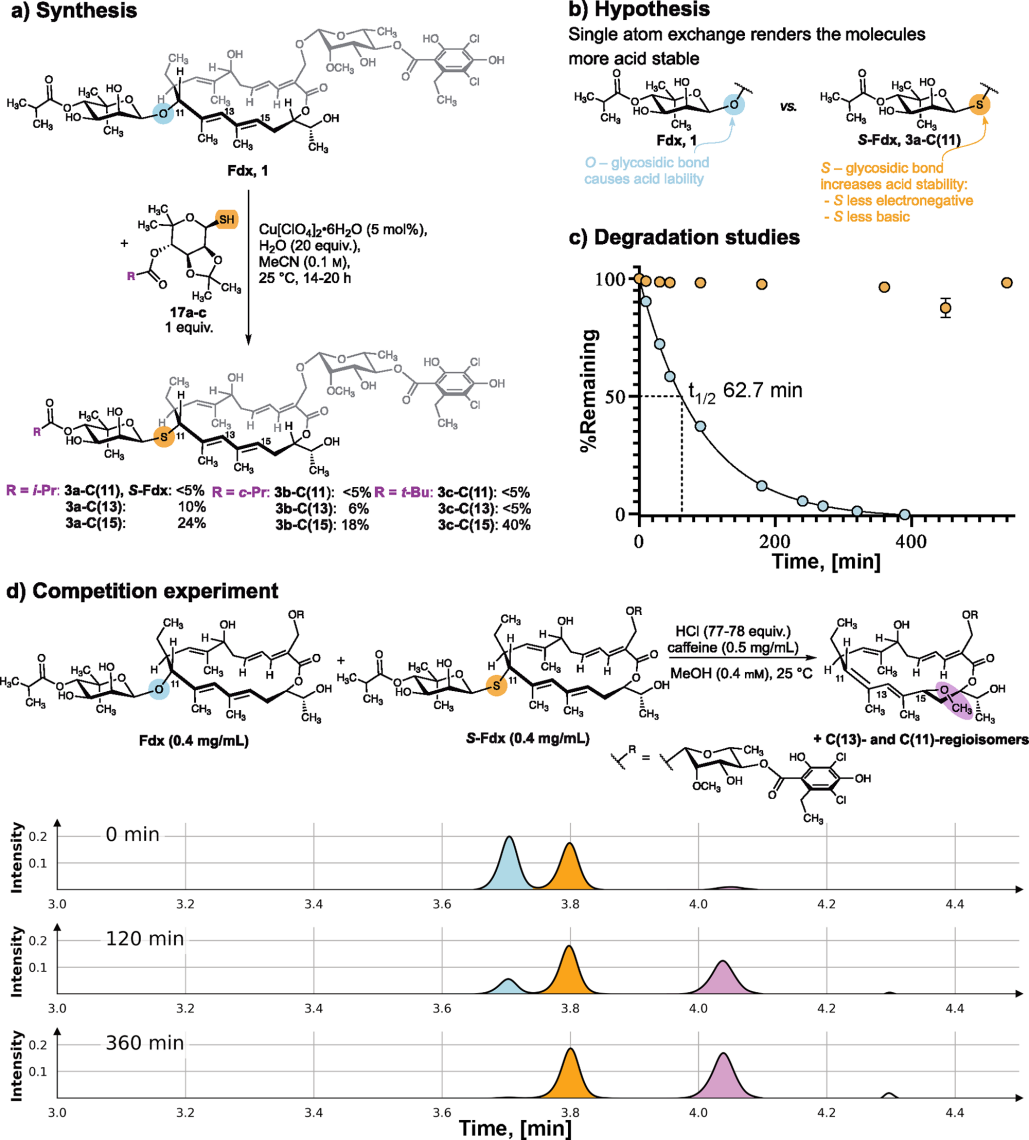
(a) Semi synthesis of the single atom exchanged analogue S-Fdx and its 4-*O*-acyl derivatives. (b–d) Effect of the S-/O-exchange on the acid stability: (b) Hypothesis. (c) Acid-mediated degradation of Fdx (cyan) and S-Fdx (orange): Fdx/S-Fdx (0.5 mg/mL) was treated with HCl (68 equiv) in MeOH (0.5 mM) at 25 °C in the presence of caffeine (0.5 mg/mL) as an internal standard (IS). The percentage of remaining analyte was plotted over time. The half-life *t*_1/2_(Fdx) of 62.7 min was determined by nonlinear regression to a one phase exponential decay. Data refer to mean ± SD (*n* = 2–4). Note: most error bars are too small to be displayed. (d) Competition experiments reveal Fdx to degrade faster than S-Fdx under acidic conditions: (top) A mixture of Fdx (0.4 mg/mL) and S-Fdx (0.4 mg/mL) in MeOH was subjected to HCl (77–78 equiv) in the presence of caffeine (IS), and the mixture was stirred at 25 °C. (bottom) UV-chromatogram at 270 nm of UHPLC–MS analyses performed at the indicated time points. *t*_R_(Fdx): 3.7 min; *t*_R_(S-Fdx): 3.8 min; and t_R_(degradation products): 4.0 min.

With the acetonide-protected thionoviose derivatives **17a**–**c** in hand, we turned our attention to the semi synthesis of the single atom exchanged Fdx analogue, S-Fdx (**3a-C(11)**), and the corresponding 4-*O*-acyl derivatives. To perform the substitution reaction and the acetonide deprotection in a single reaction, water was added, the LA loading was increased to 5 mol %, and an elevated reaction time of 14–20 h was applied (Figure 4a). Purification via preparative RP-HPLC afforded S-Fdx in a yield of 3% along with the C(13)–(10%) and the C(15)-isomer (24%) (Figure 4a). Separation of the regioisomers of Fdx derivatives with similar retention times proved difficult and often required several runs of repurification. While cyclopropanoyl was introduced at C(3)–OH of the noviose moiety previously using Shimada catalyst with promising results, the introduction of a pivaloyl group could not be achieved using this method, also not with 1 equiv of catalyst at 50 °C.^49^ Therefore, compound **3c-C(11)** is the first Fdx derivative with a pivaloyl substituent on the noviose moiety, allowing us to gain further SAR information. Comparison of the ^1^H NMR spectra of Fdx^18^ and S-Fdx displays significant shifts of signals in proximity of the site of the O-/S-exchange, whereas signals far away largely display similar shifts (see Figure S3). The coupling constants of signals in direct proximity of the exchanged heteroatom on both, the aglycone and the noviose moiety, are highly similar, indicating a similar conformation in solution (S-Fdx: ^3^*J*_H10–H11_ = 10.9 Hz, ^3^*J*_H1″–H2″_ = 1.3 Hz; Fdx: ^3^*J*_H10–H11_ ≈ 9.7 Hz, ^3^*J*_H1″–H2″_ = 1.3 Hz^18^).

### Stability Studies of Fdx vs S-Fdx: The Impact of a Single Atom

O-glycosides are hydrolyzed under acidic conditions more readily than their S-counterparts.^33,34^ This is primarily caused by the lower basicity of S compared to O in acidic aqueous media favoring protonation of O,^33−36^ and the lower nucleofugacity of RSH versus ROH.^36^ The degradation kinetics of Fdx in MeOH in the presence of HCl have been studied previously.^18^ To test our hypothesis, that a single atom exchange would render Fdx derivatives more acid stable (Figure 4b), we performed analogous acidic degradation studies in MeOH. In the presence of 68 equiv HCl in MeOH at 25 °C, Fdx undergoes acid-mediated degradation with a half-life of 62.7 min (Figure 4c), providing primarily one new peak in the UHPLC–MS chromatogram, with the mass corresponding to the MeOH adduct^18^ (*m*/*z*: [M + NH_4_^+^]: 858, [M + Na^+^]: 863). NMR analysis of HCl-mediated degradation of Fdx in MeOD-*d*_4_ revealed the formation of at least three new species, likely corresponding to regioisomers of the MeOH adduct (see Figure S4). In contrast, S-Fdx was found to be stable over a time frame of 540 min (Figure 4c). This finding was confirmed by NMR studies in MeOD-*d*_4_ (Figure S4).

For direct comparability, a competition experiment was performed by subjecting a mixture of Fdx and S-Fdx in MeOH to acidic conditions. A much faster degradation of Fdx in comparison to S-Fdx was observed (Figures 4d and S5). These results confirm the initial hypothesis that replacing the O- for a S-glycosidic linkage renders Fdx derivatives less amenable to acid-mediated reactions with nucleophiles.

### Acidic Degradation and Its Impact on Antibacterial Activity

**Figure 5:**
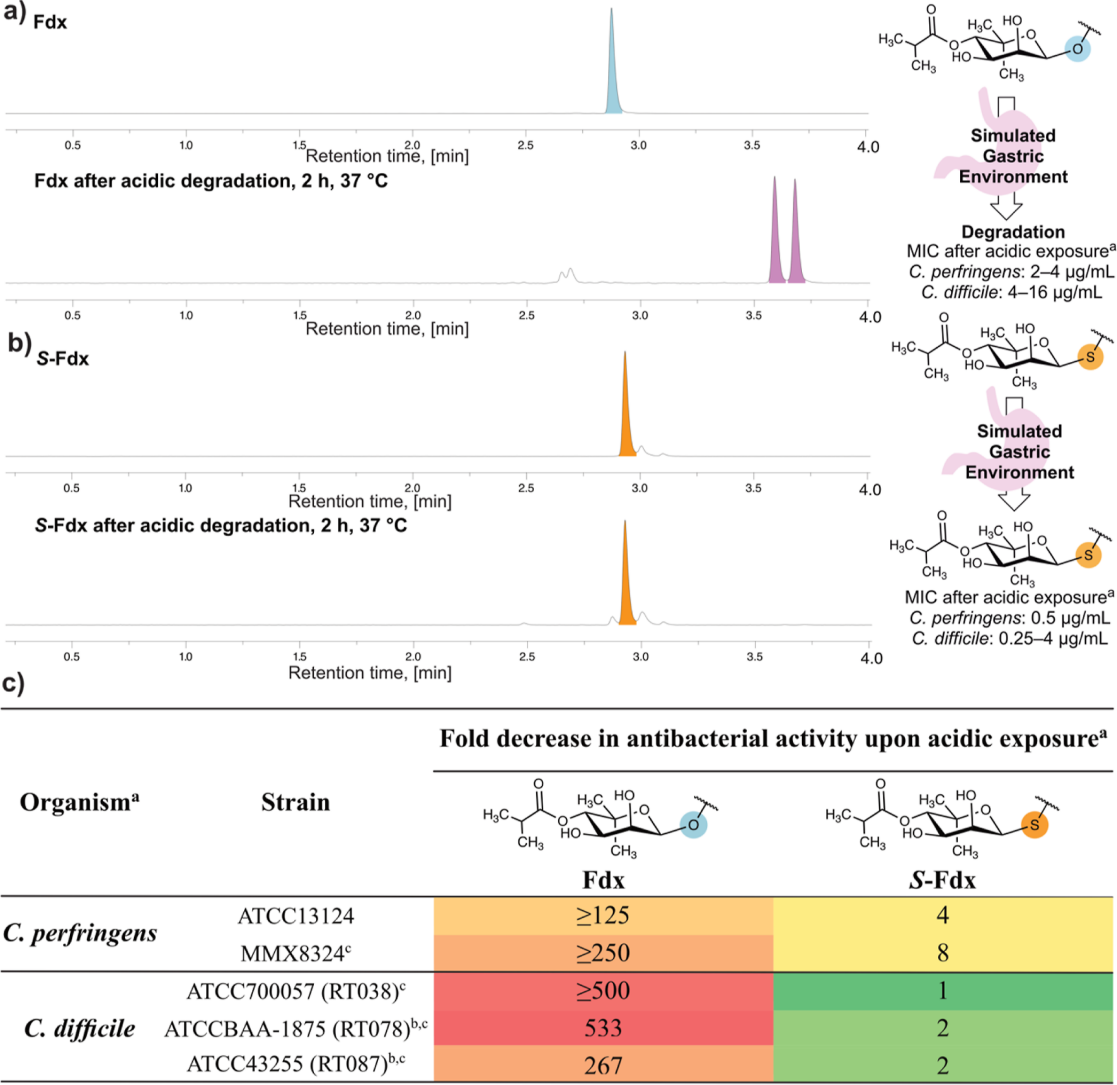
Acidic degradation and its impact on the antibacterial activity. (a,b) UV chromatogram at 270 nm of UHPLC-MS analysis of Fdx (a) and S-Fdx (b) before and after exposure to acidified SGFsp (simulated gastric fluid without pepsin) (pH 0)/MeCN 1:1 at 37 °C for 2 h at a concentration of 0.3 mg/mL. (c) Fold increase in minimum inhibitory concentration (MIC) values compared to without pre-exposure to acidic conditions. ^a^MIC was determined by Microbiologics via broth microdilution assay. ^b^Toxigenic. ^c^Clindamycin nonsusceptible or intermediate susceptible. RT Ribotype.

Fdx was reported to be stable in a pH range of 4–6.^17^ At lower pH, acidic degradation renders the molecule instable.^17,18,50^ With the increased stability of our S-glycosidic analogue toward acid-mediated nucleophilic substitution, we next wanted to study the effect of exposure to acidic conditions as they are encountered in gastric fluid. Despite the poor water solubility of Fdx,^17^ the solubility and thus the exposure to the acidic conditions are significantly impacted by the presence of solubility enhancers. In the currently used film-coated tablet Dificlir, Fdx is supplied along with microcrystalline cellulose and sodium starch glycollate,^9^ which can enhance the solubility or dissolution rate. To mimic physiological conditions in a simplified setting, the conditions were chosen close to simulated gastric fluid without pepsin (SGFsp, pH 1.2).^51^ MeCN was used as a cosolvent to account for the poor water solubility of Fdx/S-Fdx at low pH.^17^ The addition of MeCN however decreases the acid strength,^52,53^ requiring the pH to be adjusted. Fdx/S-Fdx was exposed to acidic conditions at 37 °C for 2 h before the solutions were neutralized, and the antibacterial activity was investigated. We observed full degradation of Fdx within 2 h under acidic conditions, simulating the acidic environment in the stomach (Figures 5a and S6). The acidic exposure significantly diminished the antibacterial activity of Fdx. An increase in the minimum inhibitory concentration (MIC) of Fdx of more than 250-fold against *C. diff.* and 125-fold increase against *C. perfringens* (see Figure 5c and Table S7) was found when comparing the antibacterial activity of Fdx with and without pre-exposure to the acidic environment. In contrast, S-Fdx remained intact (Figure 5b) upon exposure to the same conditions and largely maintained its activity (1- to 4-fold increase against *C. diff.*; 4- to 8-fold increase against *C. perfringens*, see Figure 5c and Table S7). These findings clearly demonstrate the superior acid stability of S-Fdx brought about by exchanging a single atom in the molecule. The improved acid stability along with the promising activity against *C. perfringens* even after treatment under acidic conditions (MIC: 0.5 μg/mL, see Figure 5b) renders S-Fdx derivatives promising to extend the application portfolio of Fdx to stomach infections.

### Synthesis of Thioglycoside Derivatives of Fdx

**Figure 6:**
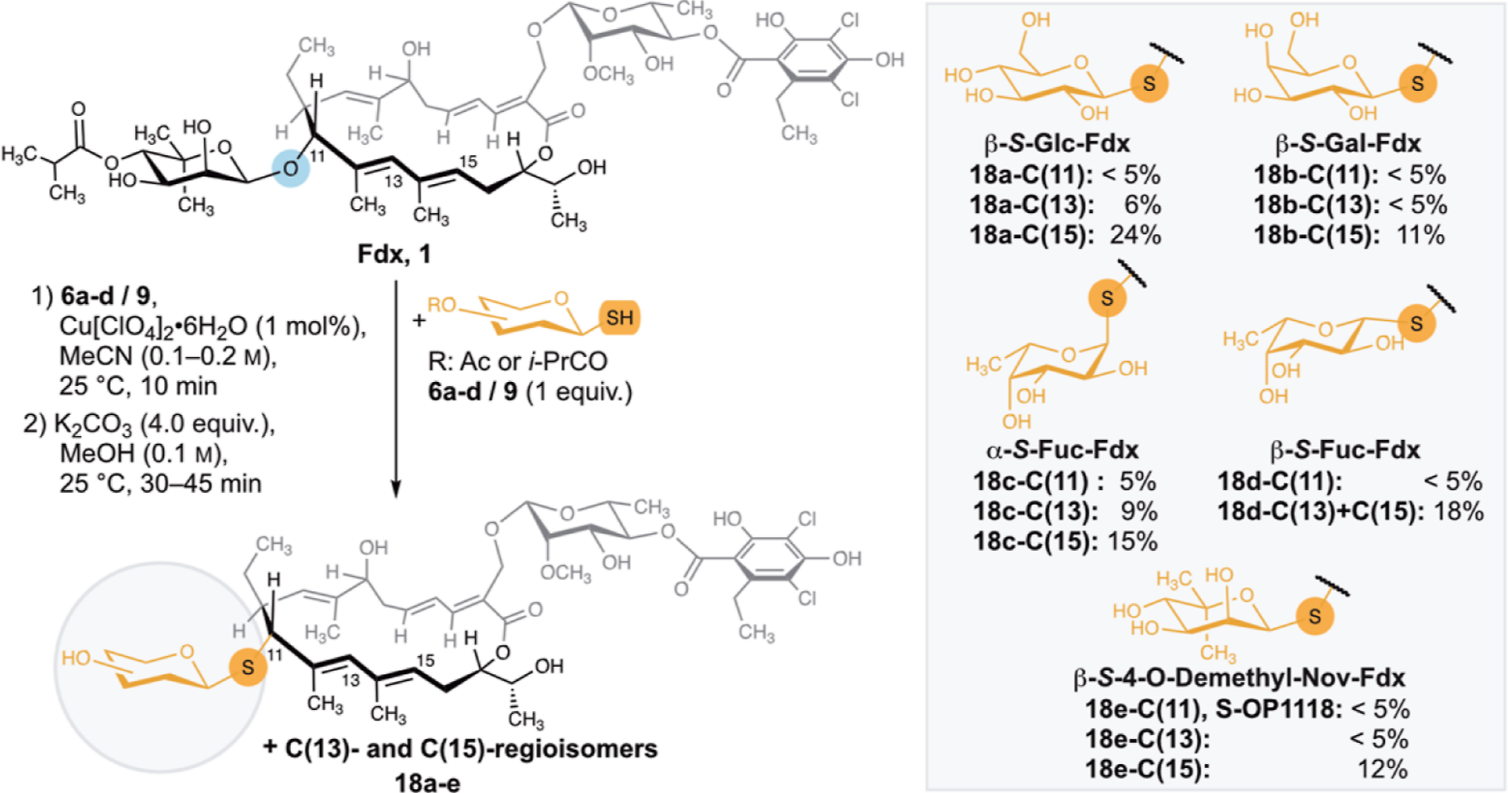
2-Step protocol for the synthesis of thioglycoside derivatives of Fdx. Yields refer to isolated yields over two steps.

Having demonstrated S-glycosidic Fdx derivatives to be more acid stable, we next wanted to extend the scope of Fdx thioglycosides. We envisioned our method for introducing S-based modifications to provide access to structural diversification. As per-O-acetylated 1-thioglucose **6a** proved to be a suitable nucleophile in our procedure, we envisaged a two-step protocol for glycodiversification. Because separation of the acetylated regioisomers proved difficult, we opted to introduce per-O-acylated 1-thiosugars, followed by deprotection under basic methanolysis and finally separation via preparative RP-HPLC. We selected various per-O-acylated 1-thiosugars (**6a**–**d**, **9**), comprising a hydrophilic CH_2_OAc unit or hydrophobic methyl groups at C(5) of the pyranose. After full conversion of Fdx under LA conditions in the first reaction step, remaining LA was removed by an aqueous workup to afford the acyl-protected thioglycoside derivatives along with side products from intramolecular reaction with the C(18)–OH. The acylated intermediates were then subjected to basic methanolysis to provide after separation the three regioisomeric products of the thioglycoside derivatives **18a**–**e** (Figure 6). Noteworthy, using the per-O-acylated 1-thionoviose **9-β** in the newly established two-step protocol gave access to the single atom exchanged analogue of OP-1118 (**18e-C(11)**, S**-OP1118**), the main metabolite of Fdx.^9^ For the thioglycosides **18a**–**e**, we observed a product distribution favoring the C(15)-isomer (11–24%), followed by the C(13)-isomer (<5–9%) and the C(11)-isomer (≤5%) (Figure 6). The product distribution in all synthesized Fdx derivatives suggests the C(15)-position to be more readily accessible or also that in addition to the suggested S_N_1 type mechanism, a competing S_N_2″ reaction could be operational.

### Antibacterial Activity of Thioglycoside Derivatives

For the thioglycosides **18a**–**e**, the antibacterial activity was characterized by determining the MIC against *C. diff.* (ATCCBAA-1382, Table S8). Unsurprisingly, for the C(13)- and C(15)-regioisomers, comprising a structurally very distinct macrocyclic core, no antibacterial activity was observed. Unfortunately, no susceptibility toward the C(11)-derivatives **18a–d-C(11)** was observed. This emphasizes the role of the noviose moiety for the biological activity of Fdx. The O-/S-exchange from Fdx to S-Fdx and from OP-1118 to S-OP1118 resulted in a decreased activity, however, to a lesser extent for OP-1118 (16-fold vs 63-fold, Table S8). This is likely caused to a large extent by the loss in hydrogen bond acceptor strength going from O to S, as the glycosidic O-atom was previously reported to be involved in hydrogen bonding interactions.^30^ However, as the effect is not the same for Fdx and OP-1118, likely the hydrogen bonding interaction of the isobutyryl-moiety^30^ is also affected by the O-/S-exchange. Interestingly, the loss of activity comparing Fdx to S-Fdx is of similar magnitude as that of Fdx to OP-1118. This suggests that modification of the ester moiety in the 4-position of the noviose constitutes a promising strategy to recover some activity lost upon O-/S-exchange. Thus, we examined the effect of first modifications of the acyl group. The antibacterial activity of the acyl derivatives of S-Fdx was assessed by means of MIC against *C. diff.* and *C. perfringens* (Tables 1 and S9). MIC determination was performed by the broth microdilution method.^54,55^ Exchanging a single atom from Fdx to S-Fdx resulted in a decreased activity of 0.12–2 μg/mL against *C. diff.* and 0.06–0.12 μg/mL against *C. perfringens*. A slightly decreased activity was observed for acyl derivatives **3b-C(11)** and **3c-C(11)**. In comparison to the nonacylated S-OP1118 (Table S8), esterification with lipophilic alkyl carbonyl derivatives clearly lead to an increase in antibacterial potency, however, not to an extent that makes up for the loss of activity upon O-/S-exchange. Further modification of the ester is necessary to obtain more SAR information.

**Table 1:**
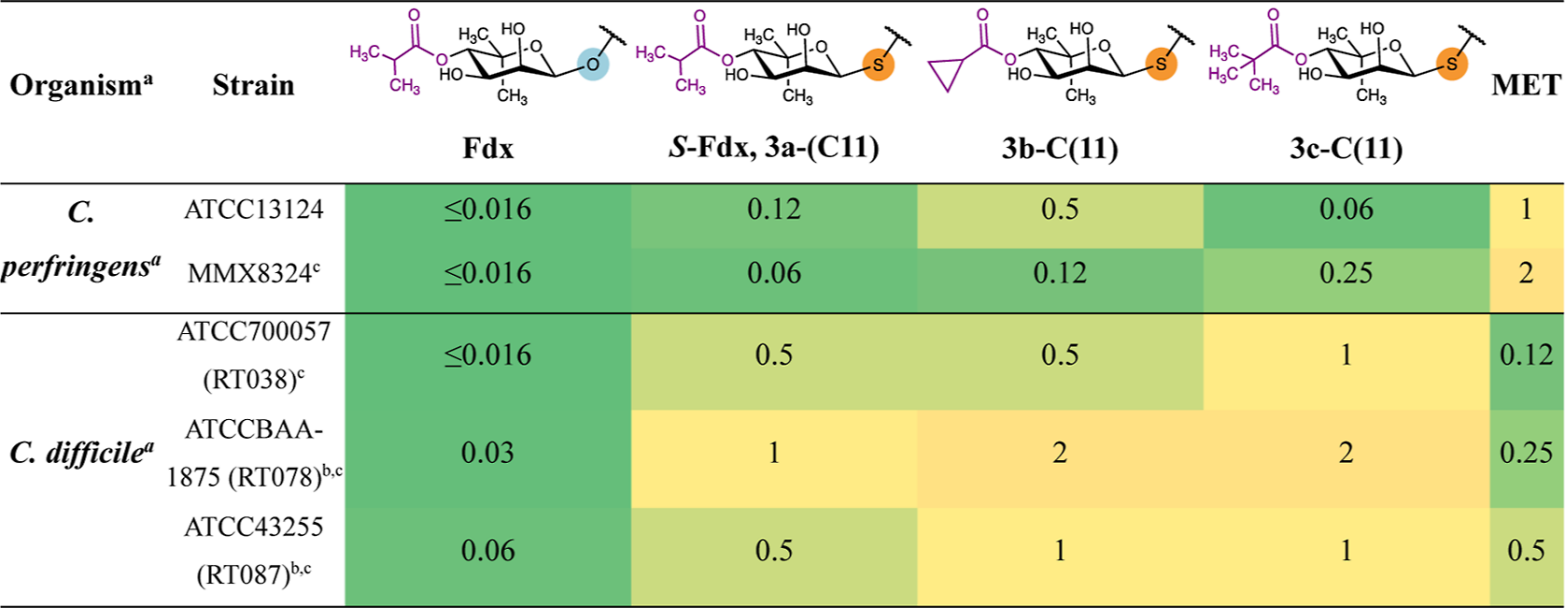
MIC Values of S-Fdx Derivatives in μg/mL

a MIC determined by Microbiologics via broth microdilution assay.

b Toxigenic.

c Clindamycin nonsusceptible or intermediate susceptible. RT Ribotype. MET Metronidazole.

Gratifyingly, all S-Fdx derivatives were found highly active against *C. perfringens* with MIC values ranging from 0.06 to 0.5 μg/mL. In comparison, the currently used metronidazole (MET)^24,27,29,56^ had a MIC range of 1 to 2 μg/mL against the investigated *C. perfringens* isolates. Noteworthy, excellent activity was observed for the clindamycin resistant *C. perfringens* isolate.

### Mechanistic Studies

**Figure 7:**
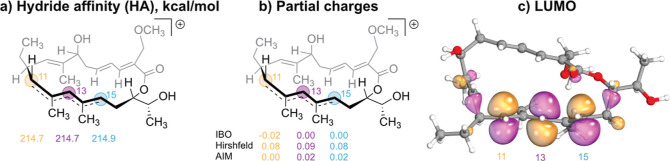
(a) HA values; (b) partial charges; and (c) LUMO of the suggested carbocation intermediate, computed at the r^2^SCAN-3c//ωB97M-V/def2-TZVPP level of theory.

A better understanding of the reaction mechanism might pave the way for future experiments to shift the product distribution toward the C(11)-isomer. As discussed, initial proposals on the acid-mediated reactivity of Fdx toward nucleophiles suggested an S_N_2″-mechanism.^18^ As in this work, three regioisomers C(11), C(13), and C(15) were obtained, and the reaction does not proceed via a S_N_2″ mechanism solely. Further, retention of configuration was observed for the C(11)-isomer. Thus, a direct S_N_2-type mechanism was ruled out. To exclude that the formal S_N_2″ product rearranges to the other regioisomers, the isolated C(15)-isomer was resubjected to the reaction conditions in the absence of a thiol. On the reaction time scale, no conversion of the C(15)-isomer was observed (Figure S7). Further, the reactivity of the C(15)-isomer toward a thiol nucleophile under the reaction conditions was assessed. On the reaction time scale, no conversion was observed (Table S10). Thus, initial formation of the C(15)-isomer, followed by subsequent S_N_2′ or S_N_2″-reactions with thiol can be excluded. In absence of a thiol, an intramolecular reaction of Fdx to the previously described 15S-THF derivative (**2b**)^18^ was observed (see Supporting Information). To exclude an initial intramolecular reaction, followed by an opening of the derivative **2b** by S_N_2′ or S_N_2″-type attack by the thiol, the derivative **2b** was subjected to the reaction conditions. Neither the 15S-derivative **2b** nor the 15*R*-derivative **2a** underwent significant reaction on the reaction time scale (Table S10). The reaction was also not affected by the presence of butylated hydroxytoluene (BHT), rendering a radical mechanism unlikely (Figure S8). Because all of the experimental data suggest an S_N_1 mechanism as the probable mode of operation in the reaction under consideration, we decided to analyze the system with density functional theory (DFT) simulation. The goal of the simulation was to determine the most stable conformer of the forming carbocation together with its electronic properties, which in turn might give us deeper understanding of the observed regio- and stereoselectivity, potentially providing means of altering the selectivity. For the sake of computation efficiency, the rhamnose side chain was substituted with a methyl group. The conformational space was explored with the GFN2-xTB semiempirical method.^57^ The generated structures were further refined using the r^2^SCAN-3c method,^58^ and the energies together with electronic properties of the found structures were computed at the ωB97M-V/def2-TZVPP level of theory (Figure 7).^59^ It was found that in the most stable conformer of the carbocation, the plane of the pentadienylic system is almost perpendicular to the plane of the macrocycle, ensuring sterical hindrance of one of the faces of the carbocation. Therefore, only the outer face of the π-system is reactive to nucleophiles (inner-annular stereocontrol). This result supports the experimental outcomes with only one set of epimers observed for each possible position of attack (C(11), C(13), and C(15)). To better understand the regioselectivity, hydride affinity (HA) values of the positions C(11), C(13), and C(15) were calculated together with partial charges. Interestingly, unlike in the pentadienyl carbocation, in the studied system, all three HA were found to be almost equal (214.7, 214.7, 214.9 kcal/mol for positions C(11), C(13), and C(15), respectively) within the calculation error. This suggests ca. 1:1:1 regioselectivity in the substitution reaction. Similar conclusions can be drawn based on partial charge values. Thus, IBO^60^-based partial charges were determined to be −0.02, 0.00, and 0.00 for positions C(11), C(13), and C(15); QTAIM-based partial charges: 0.00, 0.02, and 0.02; Hirshfeld partial charges: 0.08, 0.09, and 0.08. Also, analysis of the LUMO orbital of the carbocation provides the same outcome. The distribution of the orbital over atoms C(11), C(13), and C(15) is almost even, indicating no orbital control. So, both electronic and thermodynamic properties of the suggested carbocation intermediate suggest ca. 1:1:1 regioselectivity, which within a computational error largely agrees with the experimental results. Thus, an S_N_1 kinetic model for the mechanism of the substitution reaction in Fdx is plausible and fits the experimental results. The small variations in the regioselectivity of the substitution can be attributed to, on one hand, different steric encumbrance at the positions C(11), C(13), and C(15) (e.g., Et group at C(10)) and, on the other hand, energetically different effects of the substitution on the conformational energy of the Fdx macrocycle, which is dependent on the nucleophile.

## Conclusions

Despite the promising activities of Fdx against many Gram-positive bacteria, among them *C. perfringens*,^9,16^ poor solubility and acid lability prevent its use for infections outside the intestine. The acid lability is attributed to the labile bis-allylic O-glycosidic noviose moiety at C(11) of Fdx.^18^ Therefore, we envisaged a single atom exchange of O- for a S-glycosidic linkage to constitute a promising strategy to increase the acid stability. Herein, we exploit the acid-mediated reactivity of Fdx toward nucleophiles^18^ to introduce more acid stable S-based modifications. The high nucleophilicity of thiols also enables the modification of Fdx in the presence of reactive OH–functionalities. In this work, we developed an operationally simple protocol for the direct introduction of 1-thiosugars into the Fdx skeleton under substitution of the C(11)-noviose, giving access to C(11)-, C(13)-, and C(15)-thioglycoside derivatives of Fdx. Advantages of the strategy are its insensitivity to air and moisture and its applicability for the direct modification of unprotected Fdx. While the regioselectivity so far could not be manipulated toward the C(11)-regioisomer, the C(11)-product is formed diastereoselectively with retention of configuration compared to Fdx. Furthermore, we present the first synthesis of 1-thionoviose derivatives allowing us to access the single atom exchanged derivative S-Fdx. With the O- and S-glycosidic analogues of Fdx in hand, we assessed the direct effect of the single atom exchange on the acid stability and the antibacterial activity of Fdx. We found the O-/S-exchange to significantly enhance the acid stability of Fdx, as confirmed by methanolic degradation studies. Moreover, forced acidic degradation studies of both analogues revealed full degradation of Fdx concomitant with a significant loss of biological activity, while S-Fdx proved stable under the same conditions mostly maintaining the antibacterial activity. With the modular nature of our newly established 1-thionoviose synthesis, 4-O-acyl derivatives of S-Fdx were accessed. Despite the O-/S-exchange is overall associated with a decrease in the antibacterial activities against *C. diff.* and *C. perfringens*, the S-Fdx derivatives still possess high potency against *C. diff.* with MIC ranges of 0.12–4 μg/mL and excellent potency against *C. perfringens* with MIC ranges of 0.06–0.5 μg/mL. The new Fdx derivatives provide valuable SAR information and pave the way to extend the application portfolio of Fdx derivatives beyond local, intestinal infections.

## Materials and Methods

Detailed information on experimental procedures, the compound characterization, crystallographic data, and DP4+ results are provided in the Supporting Information files.

## Chemical Synthesis and Characterization of Compounds

Unless indicated differently, all chemicals used were of reagent grade, purchased from commercial sources, and used as received. All solvents used in the reactions were obtained from commercial sources and used as received. Fidaxomicin (CAS: 873857-62-6) was purchased from BOC Sciences (98%, lot: B21S04071) and from Biosynth Carbosynth (lot: 310151901). OP-1118 for antibacterial testing was provided by Erik Jung (University of Zurich). Reactions were carried out under inert atmosphere (N_2_) using flame-dried glassware and anhydrous solvents, if not indicated otherwise. Reactions of Fdx with thiol nucleophiles were performed without any precautions to avoid air or moisture, and solvents used were not anhydrous. Detailed information on the reaction conditions, experimental procedures, instruments used, and the characterization of all newly synthesized compounds including 1- and 2-D NMR spectral data can be found in the Supporting Information.

## X-ray Crystallography

Details on the X-ray crystal-structure determination and the crystallographic data of compounds **13**, **14**, **15b**, **17a**, **17b**, **17c** can be found in the Supporting Information, and supplementary crystallographic data were deposited at CCDC.

## Computations

### DP4+ Calculations

DFT calculations were carried out using the ORCA 5.0.1 (**5b-C(11)**, **5b-C(13)**, **5b-C(15)**) or the ORCA 5.0.3 (**18e-C(11)** (full molecule and simplified molecule), **18e-C(13)**, **18e-C(15)**) package^61−64^ following the DP4+ protocol developed by Grimblat, Zanardi and Sarotti.^41^^1^H and ^13^C magnetic shielding tensors (σ) were computed using gauge including atomic orbital (GIAO) single-point NMR calculations at the B3LYP/6-31G(d) level of theory with the CPCM^65,66^ implicit solvation model (solvent: acetone).^41^ DP4+ probabilities were calculated from the unscaled Boltzmann averaged NMR chemical shifts (TMS as a reference: σ_C_(TMS): 190.0976; σ_H_(TMS): 32.148) using the Excel spreadsheet provided by Grimblat, Zanardi, and Sarotti.^41^ For simplification purposes, the rhamnose-dichlorohomoorsellinate moiety was substituted for a methyl group. For S-OP1118 [**18e-C(11)**], the calculations were performed for the full molecule, and the simplified variant (rhamnose-dichlorohomoorsellinate replaced by methyl) with both leading to similar results. For detailed procedures and the experimental data obtained, refer to the SI_DP4+ file.

### DFT Simulations

For the calculation of the properties of the carbocation of Fdx, the following protocol was applied. Conformational search was performed similarly to the DP4+ protocol mentioned above. Geometry optimizations were performed at the r^2^SCAN-3c level of theory.^58^ Numerical frequency calculations were performed at the same level of theory to characterize all stationary points. The electronic energy was further refined through single point calculations at the ωB97M-V level of theory.^59^ All the atoms were described with the def2-TZVPP basis set. Thermochemical properties were calculated using qRRHO approximation.^67^ Hydride affinities were calculated as Gibbs energies of the corresponding hydride addition reactions. The partial charges were calculated with Multiwfn software.^68^ The molecular orbitals were visualized with IboView software.

### Acidic Degradation Studies

#### Acidic Methanolysis

Detailed experimental procedures, calibration curves, and processed data for the acidic methanolysis studies can be found in the Supporting Information.

#### Fdx vs S-Fdx Acidic Methanolysis Competition Experiment

Solutions of caffeine in MeOH (5 mg/mL), of Fdx in MeOH (1 mg/mL), and of S-Fdx in MeOH (1 mg/mL) were prepared. Then, in a 1.5 mL screw-cap vial equipped with a stirring bar, 164.5 μL of the S-Fdx solution (1 mg/mL in MeOH) was mixed with 164.5 μL of the Fdx solution (1 mg/mL in MeOH), followed by addition of 37 μL of caffeine solution (5 mg/mL). To the solution was then added 3.95 μL of HCl (3 M solution in MeOH, freshly opened ampule, *c*_final_: 0.032 M). The reaction mixture was stirred at 25 °C. At the indicated time points, 5 μL of the reaction mixture was taken and diluted in 45 μL MeOH, and the sample was subjected to UHPLC–MS analysis, using the following LC time program (time–% B): 0.00 min −5%, 0.50 min −5%, 0.55 min −30%, 3.50 min −60%, 3.55 min −100%. The retention times of the analytes and methanolysis degradation products (mixture of regioisomers; *m*/*z*: [M + NH_4_^+^]: 858, [M + Na^+^]: 863) are as follows: Fdx–*t*_R_: 3.7 min; S-Fdx—*t*_R_: 3.8 min; degradation products—*t*_R_: 4.0 min; caffeine: *t*_R_: 1.3 min.

### Acidic Hydrolysis and Acidic Degradation in a Simplified Simulated Gastric Environment

#### Solvent II

2× simulated gastric fluid without pepsin (SGFsp) was prepared as reported in the literature^69^ but adjusting the pH to 0. 0.4 g of NaCl was dissolved in 85 mL of Milli-Q water, followed by addition of 8.5 mL of HCl (32%). The pH was then adjusted to 0, and the volume was diluted to 100 mL with Milli-Q water.

Fdx (2.8 μmol, 3.0 mg, 1.0 equiv) or S-Fdx (2.8 μmol, 3.0 mg, 1.0 equiv) was dissolved in MeCN (5 mL), followed by addition of solvent II (5 mL). The solution was stirred at 37 °C for 2 h before being cooled to 0 °C and neutralized at 0 °C with NaOH (1 m). Then, the solvent was evaporated in vacuo at 40 °C, and the residual solid was suspended in acetone and filtered [4 mm syringe filter, PTFE (hydrophilic), pore size: 0.22 μm, BGB Analytik AG], and the solvent was removed by N_2_ blowdown evaporation to provide the product of the degradation experiment as a colorless solid (Fdx: 2.76 mg; S-Fdx: 2.67 mg). The solids were used for characterization of the antibacterial activity via broth microdilution assays^54,55^ by Microbiologics (for assay procedure see Supporting Information). A small sample of the neutralized mixture was subjected to UHPLC–MS analysis (UHPLC–MS B). For Fdx, the experiment was performed twice, and the solid obtained after nitrogen blowdown was analyzed via ^1^H NMR analysis.

### MIC Determination

MIC determination against *C. difficile* strain ATCCBAA-1382 was carried out using the broth microdilution assay, as previously described.^70^ MICs reported herein refer to the concentration of the test compound suppressing growth to OD_600_ < 0.15. A detailed assay protocol can be found in the Supporting Information.

MIC determination against a panel of ten *C. difficile* (including Toxin A/B-producing strains) and two *C. perfringens* strains was performed as a paid service by Microbiologics (site: Microbiologics, INC (formerly: Micromyx, LLC), 4717 Campus Drive, Kalamazoo, MI, USA 49008). The MIC assay method followed the procedures described by CLSI for each group of organisms^54,55^ using the broth microdilution method spanning a testing range of 0.016–16 μg/mL. MIC values were read where visible growth of the organism was inhibited. A detailed assay protocol can be found in the Supporting Information.

## Data Availability

Additional data, such as the coordinate files for conformers of both epimers of the compounds **5b-C(11)**, **5b-C(13)**, **5b-C(15)**, **18e-C(11)** (simplified and full molecule), **18e-C(13)**, **18e-C(15),** and NMR data (raw data and additional 2-D NMR data), were deposited at zenodo and can be obtained free of charge via DOI: 10.5281/zenodo.10618934.

## Supporting Information Available

Experimental procedures, compound characterization, crystallographic data, and NMR spectra of new compounds (PDF)
Detailed procedures and results of DP4+ calculations (PDF)
Crystallographic data of compound **13** (CCDC 2333101) (CIF)
CheckCIF/PLATON report of compound **13** (CCDC 2333101) (PDF)
Crystallographic data of compound **14** (CCDC 2333102) (CIF)
CheckCIF/PLATON report of compound **14** (CCDC 2333102) (PDF)
Crystallographic data of compound **15b** (CCDC 2333103) (CIF)
CheckCIF/PLATON report of compound **15b** (CCDC 2333103) (PDF)
Crystallographic data of compound **17a** (CCDC 2333104) (CIF)
CheckCIF/PLATON report of compound **17a** (CCDC 2333104) (PDF)
Crystallographic data of compound **17b** (CCDC 2333105) (CIF)
CheckCIF/PLATON report of compound **17b** (CCDC 2333105) (PDF)
Crystallographic data of compound **17c** (CCDC 2333106) (CIF)
CheckCIF/PLATON report of compound **17c** (CCDC 2333106) (PDF)

## Supplementary Material

au4c00206_si_002.pdf

au4c00206_si_003.cif

au4c00206_si_004.pdf

au4c00206_si_005.cif

au4c00206_si_006.pdf

au4c00206_si_007.cif

au4c00206_si_008.pdf

au4c00206_si_009.cif

au4c00206_si_010.pdf

au4c00206_si_011.cif

au4c00206_si_012.pdf

au4c00206_si_013.cif
