# Supplementary material for: Formal Single Atom Editing of the Glycosylated Natural Product Fidaxomicin Improves Acid Stability and Retains Antibiotic Activity
Source: JACS Au. 2024 May 21;4(6):2267–80. doi: 10.1021/jacsau.4c00206 (PMC11200244; doi:10.1021/jacsau.4c00206)
Supplement: Supplementary file 1 — au4c00206_si_001.pdf [file au4c00206_si_001.pdf]

Supporting Information

Formal Single Atom Editing of the Glycosylated Natural Product Fidaxomicin Improves Acid Stability and Retains Antibiotic Activity

Isabella Ferrara[a], Gleb A. Chesnokov[a], Silvia Dittmann[b], Olivier Blacque[a], Susanne Sievers[b], and Karl Gademann*[a]

[a]Department of Chemistry, University of Zurich, Winterthurerstrasse 190, 8057 Zürich, Switzerland.

[b]Department for Microbial Physiology and Molecular Biology, Institute of Microbiology, Center for Functional Genomics of Microbes, University of Greifswald, Felix-Hausdorff-Strasse 8, 17489 Greifswald, Germany.

*Correspondence to Karl Gademann. E-Mail: karl.gademann@chem.uzh.ch

Table of contents

Supplementary tables 2
Supplementary figures 8
Supplementary materials and methods 15
General 15
- Configurational stability of 1-thioglycosides 17
- Control experiments for mechanistic studies 17
- Acid stability experiments 19
- Broth microdilution MIC assay against *C. difficile* strain ATCCBAA-1382 22
- Broth microdilution MIC assay against a panel of *C. difficile* and *C. perfringens* 22

Characterization of compounds 25
- Characterization of Fdx derivatives 25
- General procedures 25
- Fdx derivatives from proof of concept studies 26
- Thioglycoside derivatives of Fdx 33
- 4''-*O*-acyl-*S*-Fdx derivatives 44
- Isolation and functionalization of noviose 52
- *De novo* synthesis of thionoviose derivatives 56
- Synthesis of literature known compounds 66

Crystallographic data 69
Supplementary references 73
Spectra 74
- Spectral data for Fdx derivatives from proof of concept studies 75
- Spectral data for thioglycoside derivatives of Fdx 138
- Spectral data for 4''-*O*-acyl-*S*-Fdx derivatives 244
- Spectral data for noviose derivatives 307
- Spectral data for literature known compounds 360

Supplementary tables

Table S 1: LA screening with 4-methoxybenzyl mercaptan 4a

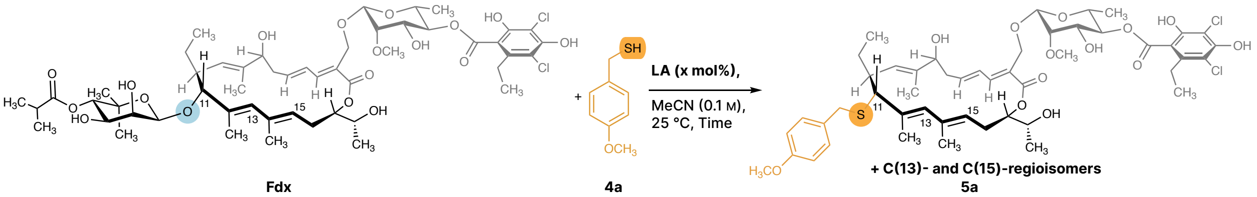

Entry	LA	LA loading [mol%]	Time	Fdx conversion[a]	Product formation C(11):C(13):C(15)[a,c]
1	none	-	67 h	not complete[b]	16:59:25
2	$Yb(OTf)_3$	1	1 h	full conversion	17:59:24
3	$CeCl_3$	10[d]	2 h	traces	7:65:28
4	$ZnCl_2$	1	2 h	traces	11:81:8
5	LiI	1	24 h	traces	12:63:25
6	$CuBr_2$	1	2 h	>95%	15:56:29
7	$CuCl_2$	1	24 h	not complete[b]	13:58:29
8	$Cu[ClO_4]_2$•6 H_2O	1	10 min	full conversion	16:60:24
9	$Cu[ClO_4]_2$•6 H_2O	10[e]	10 min	full conversion	17:55:28
10	$Cu[ClO_4]_2$•6 H_2O	100[d]	10 min	full conversion	16:52:32

[a]Analyzed *via* UHPLC-MS analysis at 270 nm. Reactions were performed following **General procedure for screening 4-methoxy-benzylmercaptan 4a**. [b]Majority of Fdx converted. [c]Due to the similar retention times of the regioisomeric products and thus, partial overlap of the integrals the ratio is not accurate but can serve as a rough estimate for the product distribution. [d]Fdx and LA were directly suspended in MeCN, before addition of thiol. [e]10 mM solution of LA in MeCN was used.

Table S 2: Solvent screening with 4-methoxybenzyl mercaptan 4a

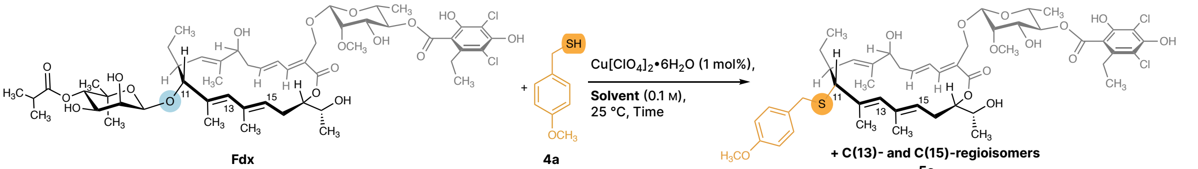

Entry	Solvent	Time	Fdx conversion[a]	Product formation C(11):C(13):C(15)[a,c]
1	MeCN	10 min	full conversion	16:60:24
2	THF	2 h	full conversion	16:57:27
3	DMF	10 min	no conversion	-
4	CH_2Cl_2/MeCN 9:1	2 h	not complete[b]	16:61:23

[a]Analyzed *via* UHPLC-MS analysis at 270 nm. Reactions were performed following **General procedure for screening 4-methoxy-benzylmercaptan 4a**. [b]Majority of Fdx converted. [c]Due to the similar retention times of the regioisomeric products and thus, partial overlap of the integrals the ratio is not accurate but can serve as a rough estimate for the product distribution.

Table S 3: Studying the impact of the thiol stoichiometry with 4-methoxybenzyl mercaptan 4a

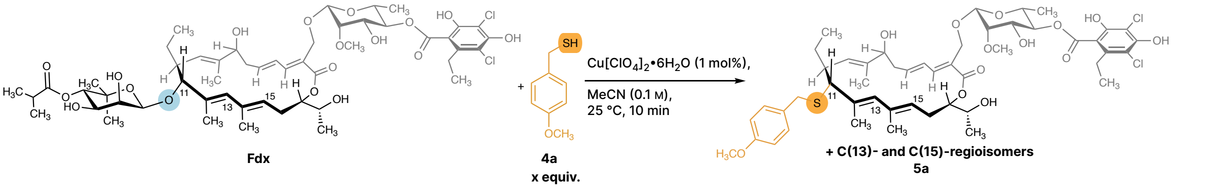

Entry	x equiv.	Fdx conversion[a]	Product formation
			C(11):C(13):C(15)[a,b]
1	1	full conversion	13:49:38
2	2	full conversion	13:53:34
3	5	full conversion	16:60:24
4	10	full conversion	18:63:20
5	20	full conversion	20:64:16

[a]Analyzed *via* UHPLC-MS analysis at 270 nm. Reactions were performed following **General procedure for screening 4-methoxy-benzylmercaptan 4a**. [b]Due to the similar retention times of the regioisomeric products and thus, partial overlap of the integrals the ratio is not accurate but can serve as a rough estimate for the product distribution.

Table S 4: Testing the reactivity of Fdx towards 1-thio-β-D glucose sodium salt

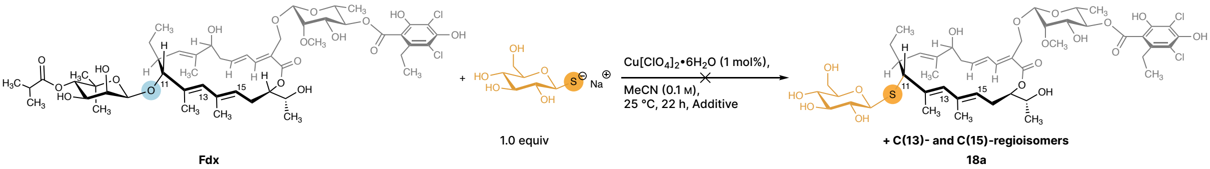

Entry	Additive	Fdx conversion[a]	Product formation[a]
1	none	traces[b]	-
2	acetic acid (1.0 equiv.)	no conversion	-
3	H_2O (20.0 equiv.)	no conversion	-

[a]Analyzed *via* UHPLC-MS at 270 nm. Reactions were performed on a 10 mg (Fdx) scale following the **General procedure for screening thiosugars**. [b]THF-Fdx side product formation observed.

Table S 5: Solvent screening with 1-thiosugar 6a

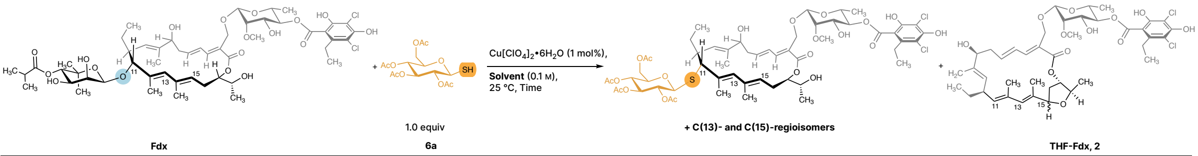

Entry	Solvent	Time	Fdx conversion[a]	Fdx:Product[a,b]:THF-Fdx[a]
1	MeCN	10 min	full conversion	0:76:24
2	THF	1 h	full conversion	0:18:82
3	EtOAc	21 h	full conversion	0:39:61
4	acetone	1 h	full conversion	0:53:47
5	DMF	21 h	traces	99:0:1
6	DMSO	21 h	no conversion	100:0:0

[a]Analyzed *via* UHPLC-MS at 270 nm. Reactions were performed on a 5 mg (Fdx) scale following the **General procedure for screening thiosugars**. [b]Due to the similar retention times of the regioisomeric products, the ratio of regioisomers was not determined. Product refers to regioisomeric mixture.

Table S 6: Brønsted acid (HA) screening with 1-thiosugar 6a

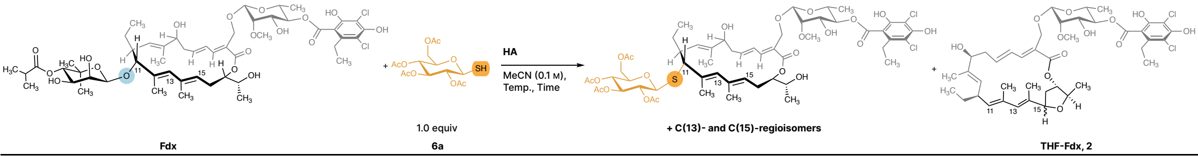

Entry	HA	LA loading	Temp.	Time	Fdx conversion[a]	Product[a,b]:THF-Fdx[a]
1	FA	1 mol%[c]	25 °C	4 h	no conversion	-
2	TFA	1 mol%[c]	25 °C	4 h	no conversion	-
3	TFA	1 equiv.	25 °C	4 h	full conversion	86:14
4	TFA	10 equiv.	25 °C	10 min	full conversion	85:15
5	TFA	1 equiv.	60 °C	10 min	full conversion	68:32

[a]Analyzed *via* UHPLC-MS at 270 nm. Reactions were performed on a 20 mg (Fdx) scale following the **General procedure for screening thiosugars**. [b]Due to the similar retention times of the regioisomeric products, the ratio of regioisomers was not determined. Product refers to regioisomeric mixture. [c]A 1x stock solution of HA in MeCN was used.

Table S 7: Acidic degradation and its impact on the biological activity

Organism[a]	Strain	Fdx 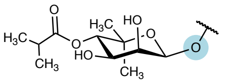			*S*-Fdx 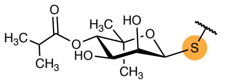			MET
		no acidic exposure	after acidic exposure	Fold decrease in activity	no acidic exposure	after acidic exposure	Fold decrease in activity	
C. perfringens	ATCC13124	≤0.016	2	≥125	0.12	0.5	4	1
	MMX8324[c]	≤0.016	4	≥250	0.06	0.5	8	2
C. difficile	ATCC700057 (RT038)[c]	≤0.016	8	≥500	0.5	0.5	1	0.12
	ATCCBAA-1805 (RT027)[b,c]	0.12	>16	>133	2	4	2	2
	ATCC43255 (RT087)[b,c]	0.06	16	267	0.5	1	2	0.5
	ATCC9689 (RT001)[b]	≤0.016	4	≥250	0.12	0.25	2	0.25
	ATCCBAA-1875 (RT078)[b,c]	0.03	16	533	1	2	2	0.25
	NCTC13366 (RT027)[c]	0.12	>16	>133	2	2	1	1
	MMX8260 (RT017)[c]	0.03	16	533	0.25	1	4	0.25
	MMX8264 (RT027)[c]	0.12	>16	>133	2	4	2	2
	MMX8282 (RT017)[c]	0.03	16	533	0.25	1	4	0.25
	MMX8290 (RT078)[c]	0.03	16	533	1	2	2	0.5

Minimum inhibitory concentrations (MIC) values with or without pre-exposure to acidic conditions in µg/mL. [a]MIC determined by Microbiologics *via* broth microdilution assay. [b]toxigenic. [c]Clindamycin non- or intermediate susceptible. RT Ribotype. MET Metronidazole.

Table S 8: MIC values against *C. difficile* isolate ATCCBAA-1382 in µg/mL

regioisomer	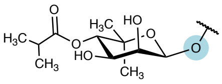 Fdx **1**	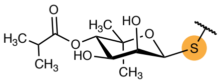 *S*-Fdx **3a**	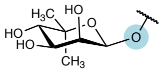 OP-1118	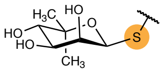 *S*-OP1118 **18e**
C(11)	0.008-0.016[c]	0.5-1[c]	0.5[c]	8[a]
C(13)	-	32[c]	-	>32[a]
C(15)	-	16[c]	-	>32[a]
regioisomer	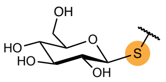 β-*S*-Glc-Fdx **18a**	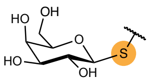 β-*S*-Gal-Fdx **18b**	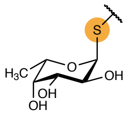 α-*S*-Fuc-Fdx **18c**	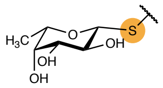 β-*S*-Fuc-Fdx **18d**
C(11)	>32[b]	>32[a]	>32[a]	>32[a]
C(13)	-	>32[a]	>32[a]	>32[a,d]
C(15)	-	>32[a]	>32[a]	>32[a,d]

Each compound was tested in one to three biological replicates, each of them in two technical replicates:
[a] 1 biological replicate, [b] 2 biological replicates, [c] ≥3 biological replicates.
[d] Mixture of C(13) and C(15)-regioisomers tested.

Table S 9: Antibacterial activity of *S*-Fdx derivatives

**Organism**[a]	**Strain**	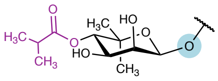 **Fdx, 1**	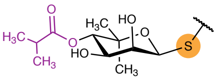 ***S*-Fdx, 3a-C(11)**	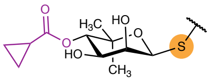 **3b-C(11)**	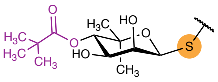 **3c-C(11)**	**MET**
C. perfringens	ATCC13124	≤0.016	0.12	0.5	0.06	1
	MMX8324[c]	≤0.016	0.06	0.12	0.25	2
C. difficile	ATCC700057 (RT038)[c]	≤0.016	0.5	0.5	1	0.12
	ATCCBAA-1805 (RT027)[b,c]	0.12	2	2	2	2
	ATCC43255 (RT087)[b,c]	0.06	0.5	1	1	0.5
	ATCC9689 (RT001)[b]	≤0.016	0.12	0.12	0.25	0.25
	ATCCBAA-1875 (RT078)[b,c]	0.03	1	2	2	0.25
	NCTC13366 (RT027)[c]	0.12	2	2	4	1
	MMX8260 (RT017)[c]	0.03	0.25	1	1	0.25
	MMX8264 (RT027)[c]	0.12	2	4	4	2
	MMX8282 (RT017)[c]	0.03	0.25	1	1	0.25
	MMX8290 (RT078)[c]	0.03	1	1	2	0.5

MIC values in µg/mL. [a]MIC determined by Microbiologics *via* broth microdilution assay. [b]toxigenic. [c]Clindamycin non- or intermediate susceptible. RT Ribotype. MET Metronidazole.

Table S 10: Control experiments for mechanistic studies

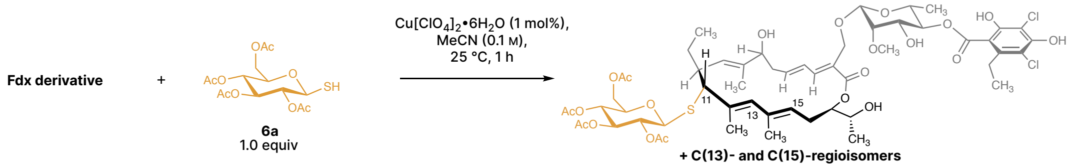

Entry	Fdx derivative	Deviation from conditions above	Conversion[a]	Product formation[a]
1	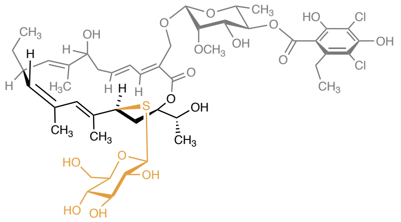 **18a-C(15)**	none	-	-
2	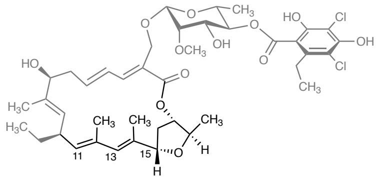 15*S*-THF Fdx, **2b**	none	-	-
3	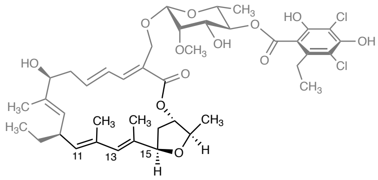 15*R*-THF Fdx, **2a**	1.5 h	Minor amount	traces[b]

[a]Analyzed *via* UHPLC-MS analysis at 270 nm. [b]Primarily starting material after 1.5 h. Formation of multiple new species that were not isolated. After 10 min, only traces of conversion were observed.

Supplementary figures

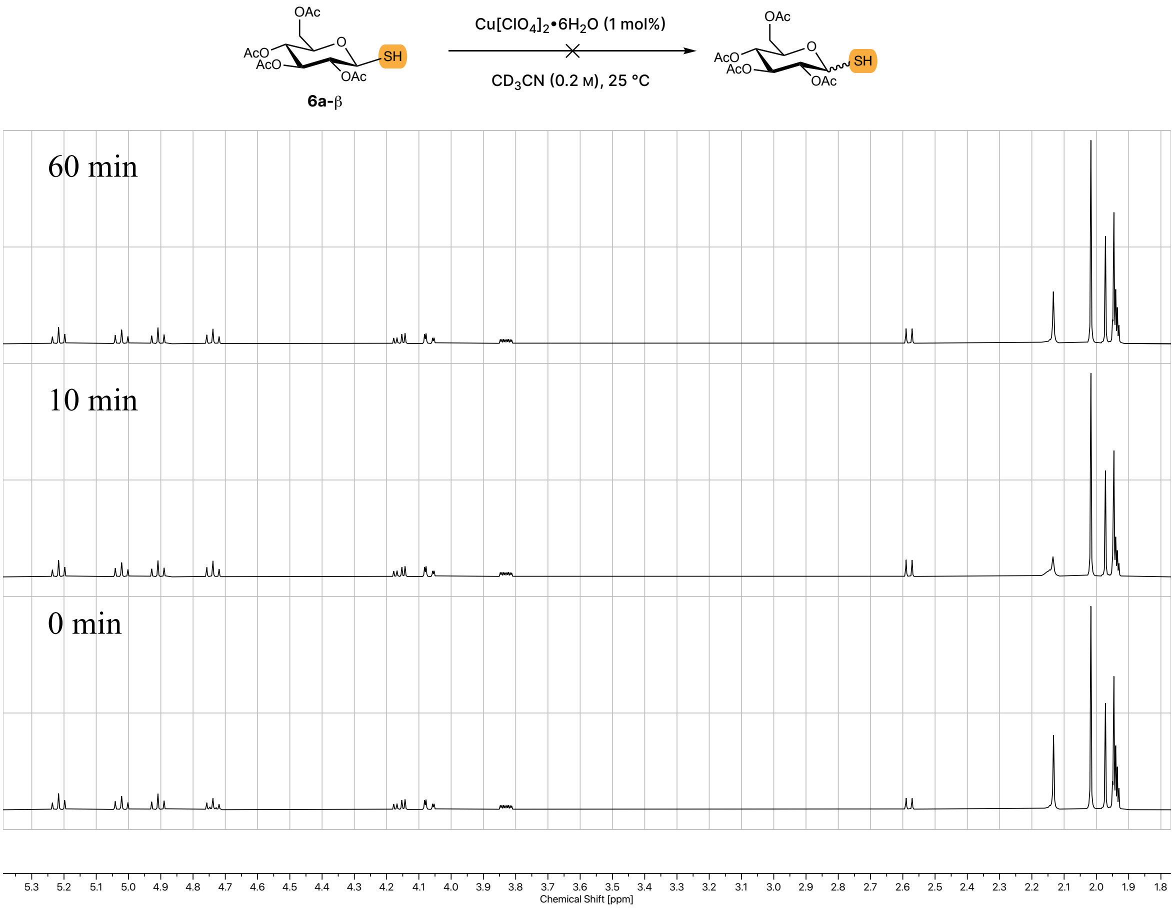

Figure S 1: ^{1}H NMR analysis of the configurational stability of the anomeric center of 1-thio-β-D-glucose tetra-*O*-acetate 6a in MeCN-d_3 subjected to 1 mol% $Cu[ClO_4]_2 \cdot 6\ H_2O$ for the indicated time period.

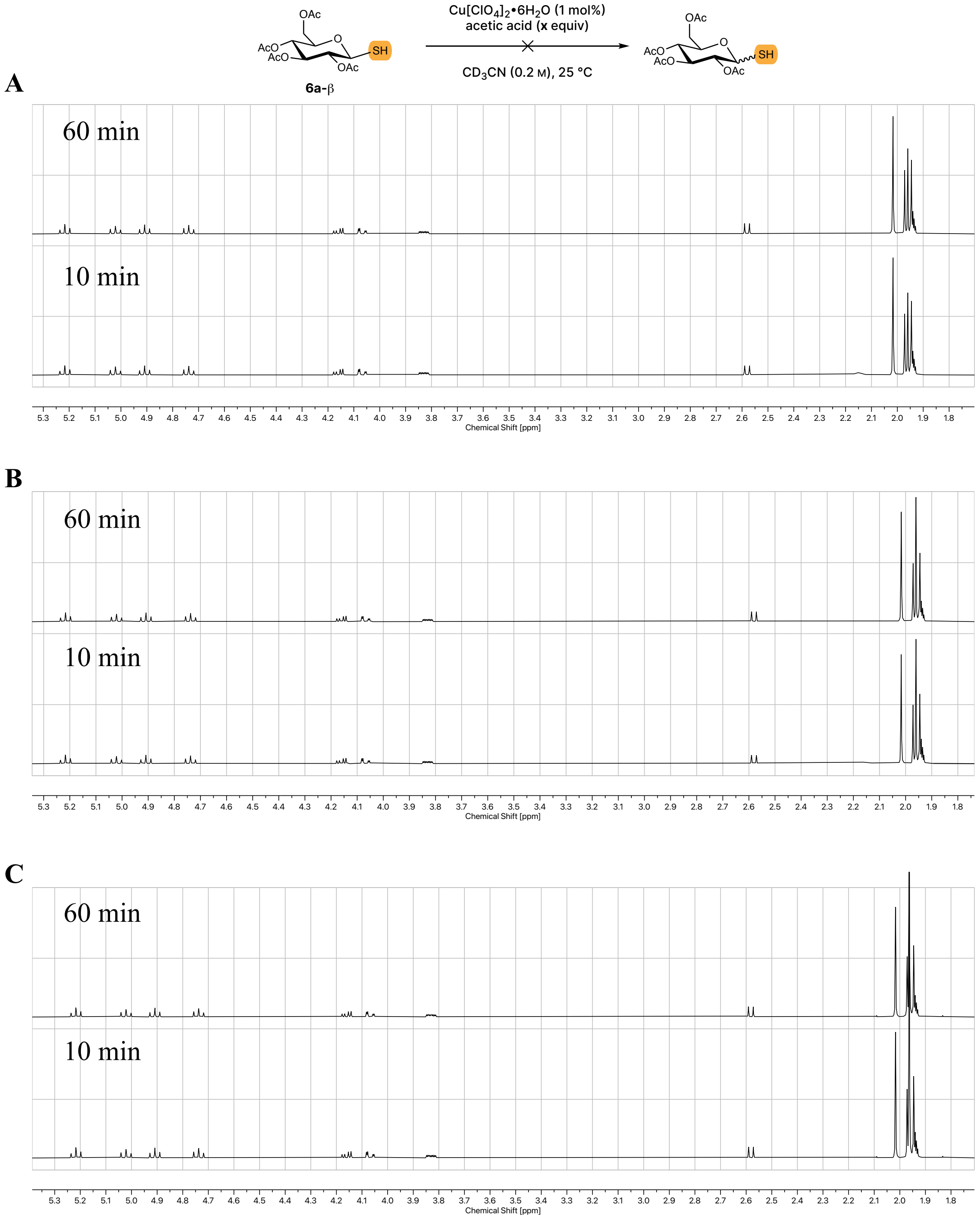

Figure S 2: ^{1}H NMR analysis of the configurational stability of the anomeric center of 1-thio-β-D-glucose tetra-*O*-acetate 6a in MeCN-d_3 subjected to 1 mol% $Cu[ClO_4]_2$•6 H_2O in the presence of (**A**) 1 equiv. / (**B**) 2 equiv. / (**C**) 5 equiv. of acetic acid, for the indicated time period.

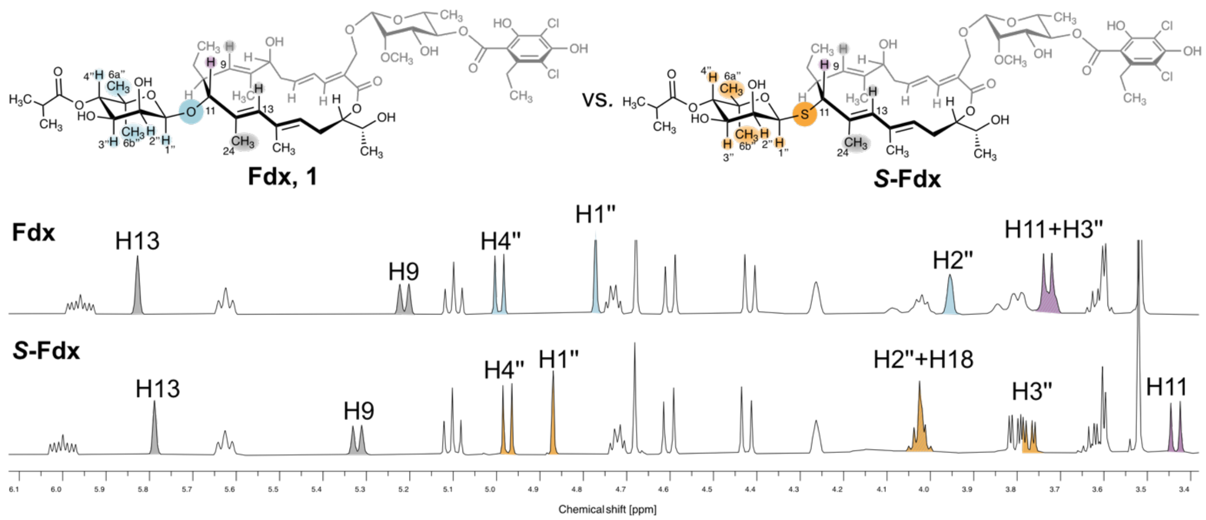

Figure S 3: Comparison of the ^{1}H NMR signals of Fdx and *S*-Fdx in acetone-d_6 in the region of 3.4 to 6.1 ppm

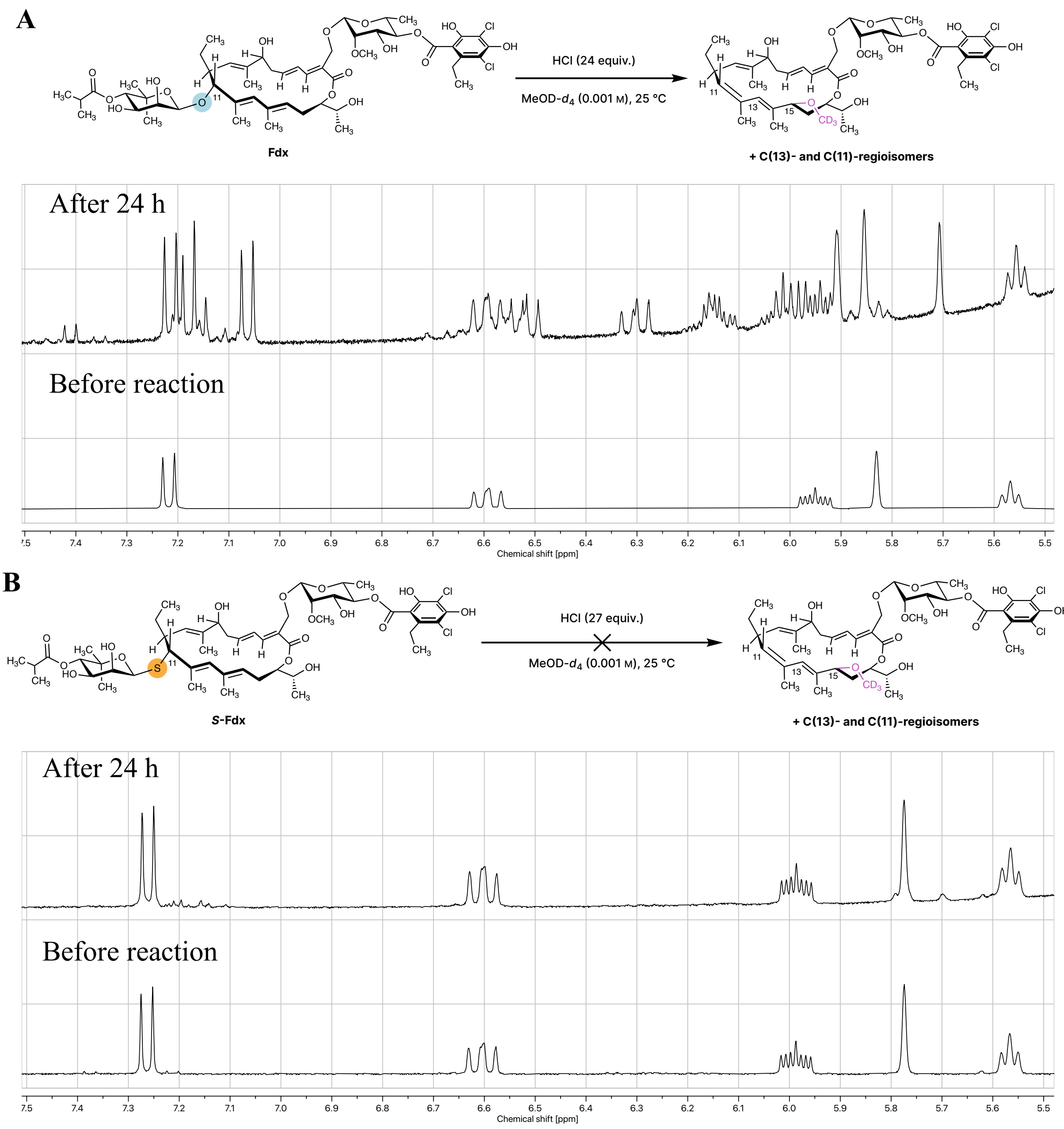

Figure S 4: NMR studies on the acid-mediated degradation of Fdx (A)/*S*-Fdx (B): Fdx/*S*-Fdx was treated with HCl (24-27 equiv.) in MeOD-*d₄* (0.001 M) at 25 °C for 24 h, after which time an ^{1}H NMR analysis was performed. **A**: In the case of Fdx, a complex mixture, displaying at least three new species (likely CD_3OD adducts) was observed in the NMR spectrum. **B**: For S-Fdx, only traces of degradation were observed after 24 h. NMR spectra were measured in MeOD-d_4.

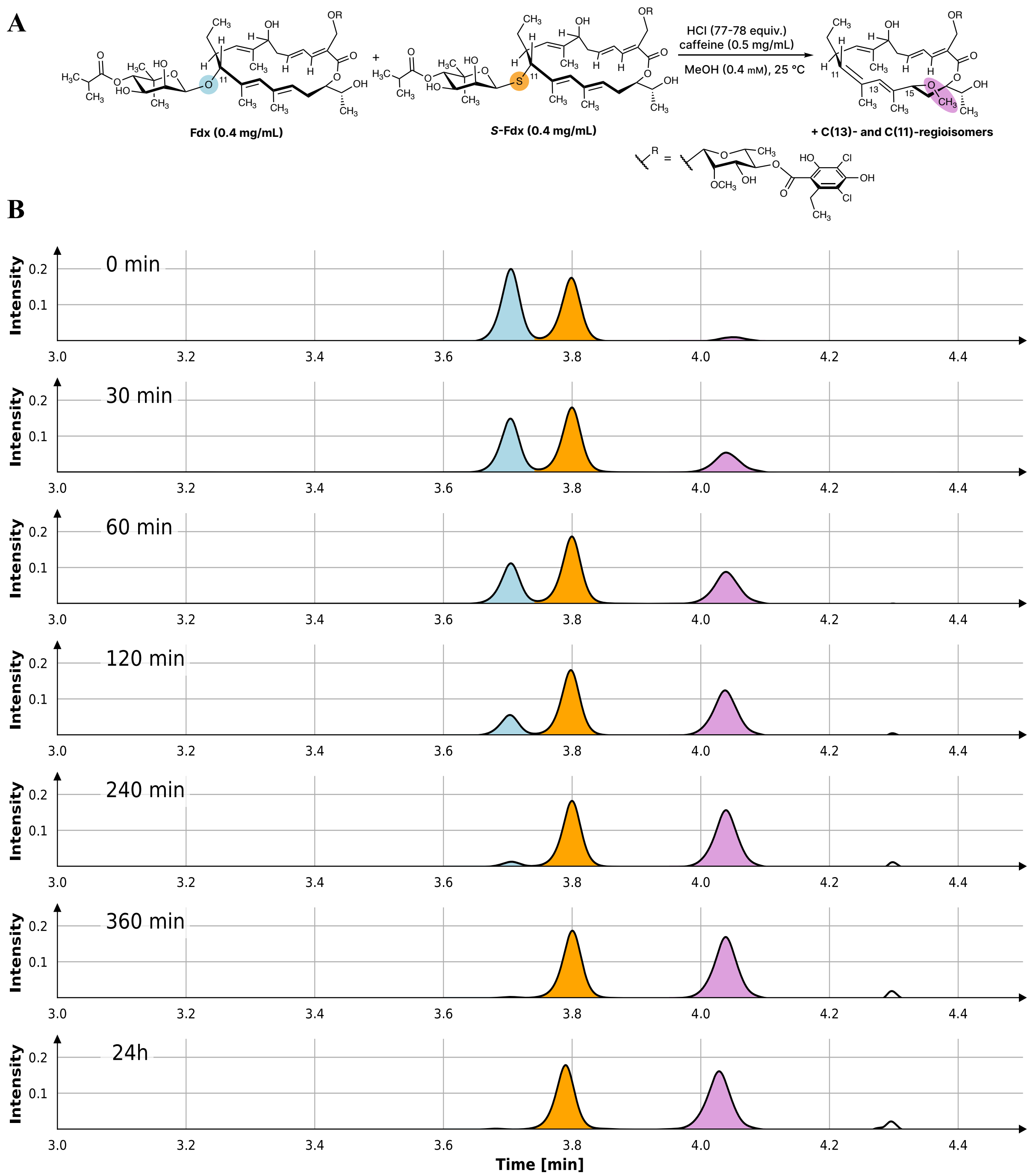

Figure S 5: Competition experiments reveal Fdx to degrade faster than *S*-Fdx under acidic conditions: (A) A mixture of Fdx (0.4 mg/mL) and *S*-Fdx (0.4 mg/mL) in MeOH was subjected to HCl (77-78 equiv.) in the presence of caffeine as an internal standard, and the mixture stirred at 25 °C. **(B)** Chromatogram of the UV peaks at 270 nm of UHPLC-MS analyses performed at the indicated time points. t_R(Fdx): 3.7 min; t_R(*S*-Fdx): 3.8 min; t_R(degradation products): 4.0 min.

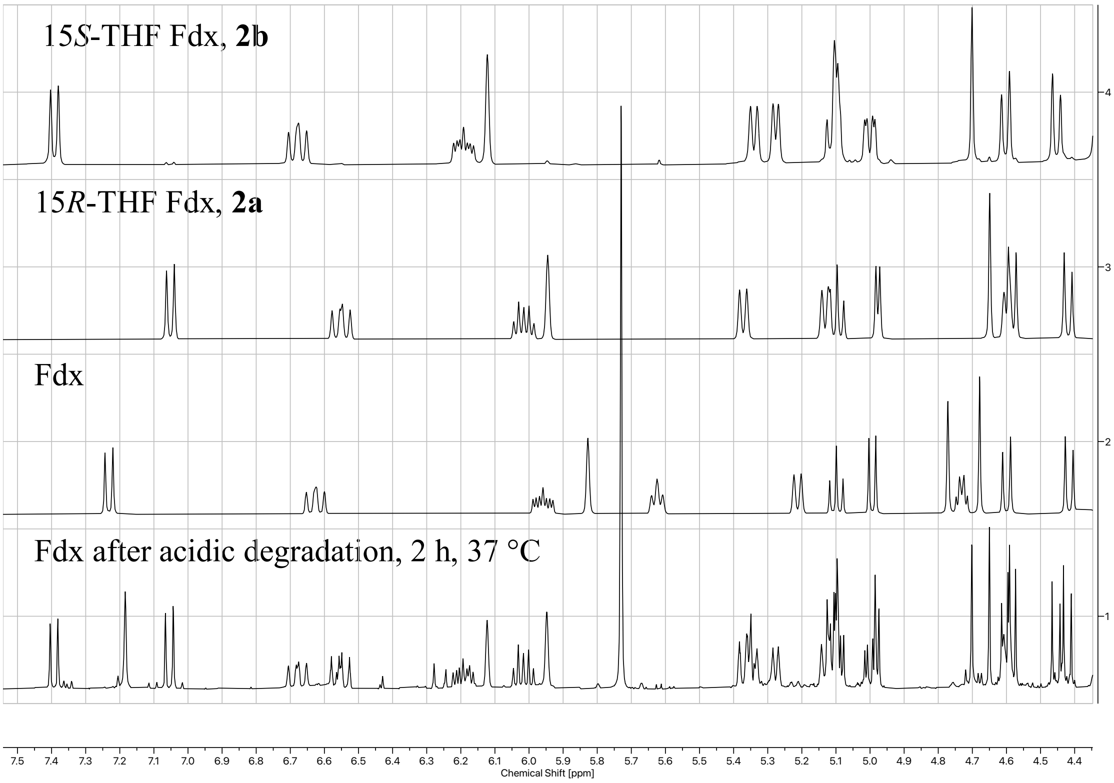

Figure S 6: Acidic degradation studies of Fdx: Fdx was exposed to acidified SGFsp (simulated gastric fluid without pepsin) (pH 0)/MeCN 1:1 at 37 °C for 2 h at a concentration of 0.3 mg/mL. ^{1}H NMR analysis in acetone-d_6 of the degradation product after 2 h reveals full degradation of Fdx to primarily the 15*S*-THF Fdx **2b** and 15*R*-THF Fdx **2a**.

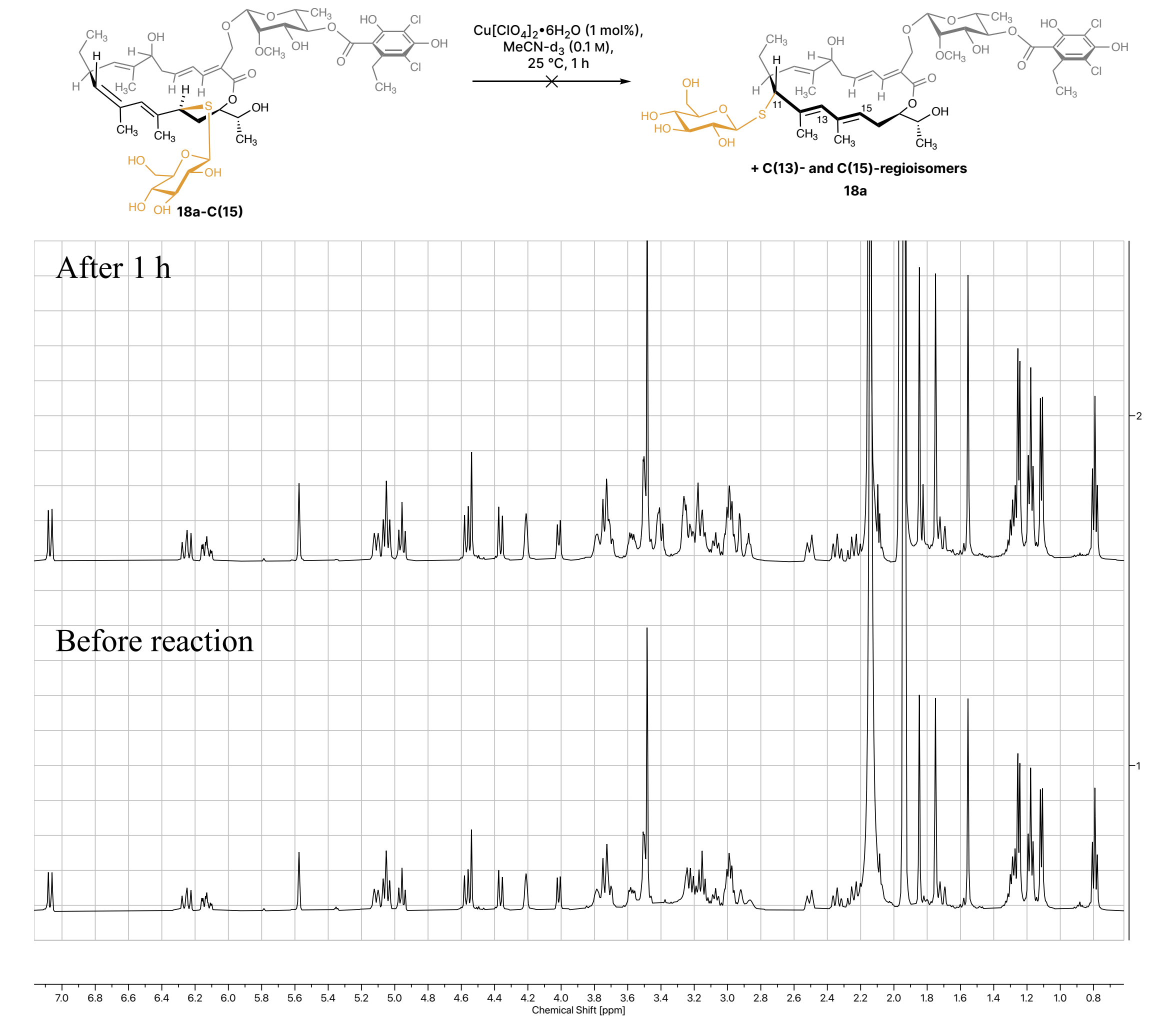

Figure S 7: C(15)-*S*-Glc Fdx (18a-C(15)) does not rearrange under reaction conditions. ^{1}H NMR spectra were recorded in MeCN-d_3.

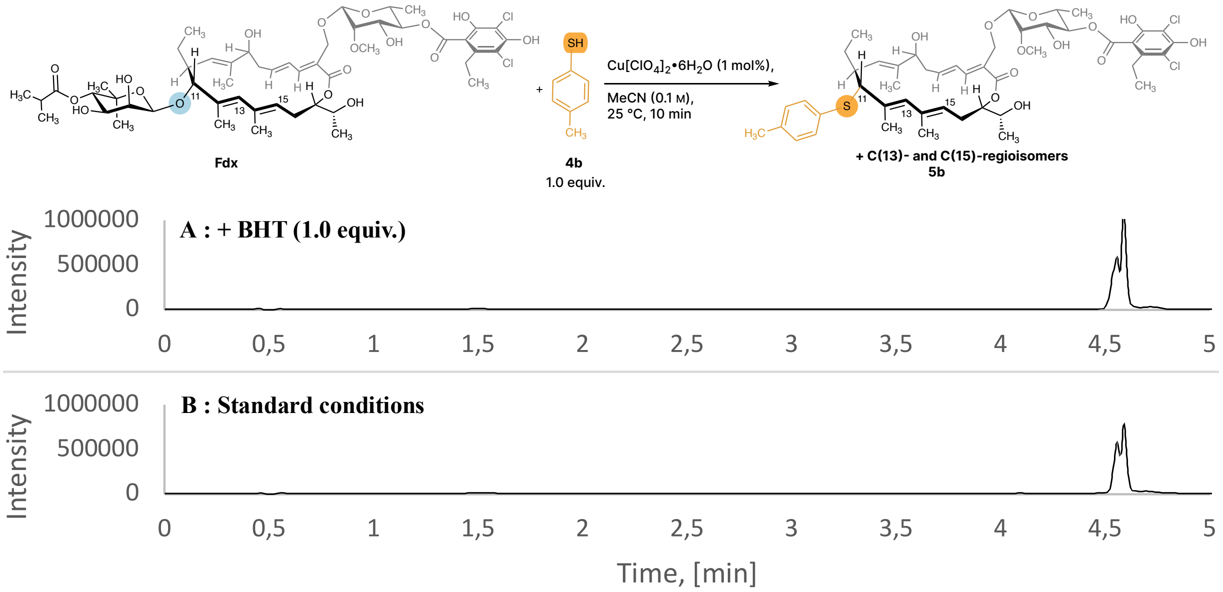

Figure S 8: UHPLC-MS chromatograms at 270 nm in the presence (A) or absence (B) of butylated hydroxytoluene (BHT, 1.0 equiv.)

Supplementary materials and methods

General

Reactions were monitored *via* UHPLC-Ms or TLC (Silica gel 60 coated alumina plates, Merck® 60 F254, 20x20 cm, visualization by UV absorbance at 254 nm, or staining with aqueous $KMnO_4$-solution, or 10% H_2SO_4 in EtOH). Removal of solvents *in vacuo* was performed using a rotary evaporator at 40 °C water bath temperature unless noted otherwise. Purification by flash column chromatography was carried out on silica gel (0.040-0.063 mm, Merck), or, where indicated, fine pore silica (Silica gel 60, 0.015-0.040 mm, No: 1.15111.1000, Merck). **Nuclear magnetic resonance spectra (NMR):** ^1H NMR, and ^{13}C NMR spectra were recorded on the instruments Bruker Avance-500 (500 MHz, 126 MHz), or Bruker Avance-400 (400 MHz, 101 MHz) in the indicated deuterated solvent at 25 °C. 2-D NMR spectra (COSY, TOCSY, HSQC, HMBC, NOESY), DEPT-90, and DEPT-135 spectra were measured on a Bruker Avance-500 instrument (500 MHz, 126 MHz) in the indicated solvents at 25 °C. The spectra were analyzed using MestReNova *v14.1.2-25024.*[1] The chemical shift (δ) in ppm of the reference solvent peak was set to the value reported by Fulmer *et al.*[2] Compound characterization and atom assignment was performed by 1- and 2-D NMR analysis (spectra attached). For all the assignments, C3''' *versus* C5''', and C2''' *versus* C6''', could not be unambiguously assigned. However, based on the computed ^{13}C chemical shifts for *S*-OP1118 (11-desnoviosyl-11-thio-(4''-desbutyryl)-β-D-noviosyl fidaxomicin) (see DP4+ calculation file, Supplementary Information_DP4+), the expected chemical shift for C3''' should be higher than the one for C5''', and the shift for C6''' higher than for C2'''. Thus, C3''' and C6'''were assigned to the higher chemical shift, respectively. **UHPLC-MS:** Ultra-high performance liquid chromatography mass spectrometry (UHPLC-MS) was used to monitor reactions and analyze experiments. If not indicated differently, measurements were run on an Ultimate 3000 LC system (Thermo Fisher Scientific) coupled to a triple quadrupole Quantum Ultra EMR MS (Thermo Fisher Scientific) on a reversed-phase column (Kinetex® EVO C18; 1.7 µm; 100 Å, 50 x 2.1 mm; Phenomenex). The equipment of the LC comprised an HPG-3400RS pump, a WPS-3000TRS autosampler, a TCC-3000RS column oven and a Vanquish DAD detector (all Thermo Fisher Scientific). The following solvent system was used: H_2O + 0.1% HCOOH (A), MeCN + 0.1% HCOOH (B). Samples were prepared in MeOH at a final concentration of ≤ 100 µg/mL, filtered (4 mm syringe filter, PTFE (hydrophilic), pore size: 0.22 µm, obtained from BGB Analytik AG), and injected at an injection volume of 1 µL. Measurements were run with a flow rate of 0.4 mL/min. The equipment of the LC comprised an H-ESI II ion source (source temperature: 250 °C, capillary temperature: 270 °C, capillary voltage: 3.5 kV) and datasets were obtained at resolution 0.7 on Q3 in centroid mode. Where indicated, **UHPLC-MS B** was used: Measurements were run on a Synapt G2 HR-ESI-QTOF-MS (Waters, Milford, USA) fitted with an electrospray ion source (ESI), coupled to an Aquity UPLC (Waters, Milford, USA). Samples were run on a reversed-phase column (Waters Acquity BEH C18; 1.7 µm; 50 x 2.1 mm). The equipment of the LC comprised a column manager, a sample manager, a binary solvent manager, and an eλ diode array detector (all Waters Aquity Ultra Performance LC). The following solvent system was used: H_2O + 0.04% HCOOH + 0.02% TFA (A), MeCN + 0.04% HCOOH + 0.02% TFA (B).Sample preparation and injection volume were used as described above. UV spectra were monitored over a range of 190 – 300 nm. Runs were performed using a linear gradient of 10-95% B over 3 min (then 2 min 95% B). The ESI was used in positive ionization mode (source temperature: 120 °C, capillary voltage: 3.0 kV, sampling cone 40 V, extraction cone 4 V, cone gas (N_2) 4 L/h, desolvation gas (N_2) 800 L/min, mass analyzer in resolution mode: mass range 100 – 2000 *m/z* with a scan rate of 1 Hz). Calibration of the mass was performed to an accuracy of under 2 ppm (50-2500 *m/z*) using a 5 mM aqueous solution of sodium formate and the lock masses of caffeine (*m/z* 195.0882,

0.7 ng/mL) and leucine-enkephalin (*m/z* 556.2771, 2 ng/mL). **High resolution electrospray ionization mass spectrometry (HRMS): On-flow injection:** High resolution mass spectra were measured on a QExactive MS instrument with a heated ESI source (ThermoFisher Scientific, Bremen, Germany) following separation on the connected Dionex Ultimate 3000 UHPLC system (ThermoFischer Scientifics, Germering, Germany). Samples were prepared in the indicated solvent system (50 µg/mL) and injected with an *XRS* auto-sampler (*CTC*, Zwingen, Switzerland) (injection volume: 1 µL; flow rate: 120 µL/min). Ion source parameters: spray voltage: 3.0 kV, capillary temperature: 280 °C, sheath gas: 30 L/min, aux gas: 8 L/min, s-lens RF level: 55.0, aux gas temperature: 250 °C. Full scan MS was performed in alternating alternating (+)/(−)-ESI mode (mass ranges: 80–1200 *m/z*, 133–2000 *m/z*, or 200–3000 *m/z,* resolution: 70000 (full width half-maximum), automatic gain control target: 3×10^6, maximum allowed ion transfer time: 30 ms). Calibration of the mass was performed to an accuracy of less than 2 ppm using *Pierce*® ESI calibration solutions (*ThermoFisher Scientific*, Rockford, USA) and the lock masses of frequently encountered erucamide (*m/z* 338.34174, (+)-ESI) and palmitic acid (*m/z* 255.23295, (−)-ESI). **Infrared spectra (IR)** were acquired on a *SpectrumTwo* FT-IR spectrometer (Perkin-Elmer) with a *Specac Golden Gate*™ attenuated total reflection (ATR) device. **Preparative high performance liquid chromatography (Prep HPLC):** Purification *via* preparative HPLC was performed on a *Prominence* modular HPLC system (*Shimadzu*) coupled to an *SPD-20A* UV/Vis detector (*Shimadzu*). Purification conditions were first evaluated using the analytical instrument with a reversed-phase (RP) column (*Gemini NX* C18, 3 µm, 10 Å, 150 mm × 4.6 mm). Suitable conditions were then used on the preparative HPLC on a RP column (*Gemini NX* C18, 5 µm, 110 Å, 250 mm × 21.2 mm). The Equipment of the LC comprised a a *CBM-20A* system controller, *LC-20A* solvent delivery unit, a *DGU-20A* degassing unit, and a *FRC-10A* fraction collector (all *Shimadzu*). Solvents and conditions are listed in the individual experiments. Samples were filtered over a Discovery® DSC-18 SPE cartridge prior to purification by prep-HPLC. **Specific optical rotations** $[\alpha]_D^T$ were acquired on a *Jasco P-2000 Polarimeter* at the temperature indicated. Analyte solutions were measured in MeOH (c is given in g/100 mL). The dimension for $[\alpha]_D^T$ is ° cm^3 dm^{-1} g^{-1}. **Melting points** were measured on a B-545 melting point instrument (Büchi) using a temperature gradient of 1 °C/min.

General procedure for the screening of conditions

*General procedure for screening 4-methoxy-benzylmercaptan **4a***

Fdx (4.73 µmol, 5.00 mg, 1.0 equiv.) was suspended in the indicated solvent (0.1 M) and a solution of LA (1 mM in the indicated solvent, 1 mol%), followed by addition of 4-methoxy-benzylmercaptan **4a** (1.0–20.0 equiv.). The mixture was stirred at 25 °C for the indicated time. The reactions were monitored via UHPLC-MS analysis and data processing was performed with the Thermo Xcalibur 3.0.41 Qual Browser software.[3]

General procedure for screening thiosugars

Fdx (1.0 equiv.) and 1-thio-β-D-glucose sodium salt/ 1-thio-β-D-glucose tetraacetate **6a** (1.0 equiv.) were suspended in the indicated solvent (0.1 M) followed by addition of $Cu[ClO_4]_2$•6 H_2O (10 mM in the indicated solvent, 1 mol%) or the indicated Brønsted acid (HA), and the corresponding additive, where indicated. The mixture was stirred at 25 °C for the indicated time. The reactions were monitored *via* UHPLC-MS analysis (UHPLC-MS B) and data processing was performed with the MestReNova MS plugin.[1] The HA screening was analyzed *via* UHPLC-MS analysis (LC time program (time – % B): 0.00 min – 5%, 0.50 min – 5%, 0.55 min – 30%, 3.50 min – 70%, 3.55 min – 100%) and data analysis was performed with the Thermo Xcalibur 3.0.41 Qual Browser software.[3]

Configurational stability of 1-thioglycosides

Under the standard reaction conditions

The stability of the anomeric configuration of 1-thioglycosides in the presence of 1 mol% $Cu[ClO_4]_2$•6 H_2O in MeCN was studied over time. A 0.002 M solution of $Cu[ClO_4]_2$•6 H_2O in MeCN-*d₃* was prepared by dissolving 1.41 mg $Cu[ClO_4]_2$•6 H_2O in 2 mL MeCN-d_3. 1-Thio-β-D-glucose tetraacetate **6a** (95 µmol, 34.6 mg, 1.0 equiv.) was dissolved in a solution of $Cu[ClO_4]_2$•6 H_2O (0.002 M in MeCN-d_3, 500 µL, 1 mol%). The mixture was stirred at 25 °C for 1 h. The reaction was monitored *via* ^{1}H NMR analysis after 0 min, 10 min and 60 min by diluting 50 µL of the reaction mixture into 450 µL MeCN-d_3.

In the presence of acetic acid

To mimic the acidic environment in the presence of Fdx (pK_a approx. 6.8[4]), the stability of the anomeric configuration of 1-thioglycosides in the presence of 1 mol% $Cu[ClO_4]_2$•6 H_2O in MeCN was studied over time in the presence of varying equivalents of acetic acid. A 0.002 M solution of $Cu[ClO_4]_2$•6 H_2O in MeCN-d_3 was prepared by dissolving 1.41 mg $Cu[ClO_4]_2$•6 H_2O in 2 mL MeCN-d_3. 1-Thio-β-D-glucose tetraacetate **6a** (18.9 µmol, 6.9 mg, 1.0 equiv.) was dissolved in a solution of $Cu[ClO_4]_2$•6 H_2O (0.002 M in MeCN-d_3, 100 µL, 1 mol%), followed by addition of acetic acid (**A**: 1.1 µL, 18.9 µmol, 1 equiv. **B**: 2.2 µL, 37.8 µmol, 2 equiv. **C**: 5.5 µL, 94.5 µmol, 5 equiv.). The mixture was stirred at 25 °C for 1 h. The reaction was monitored *via* ^{1}H NMR analysis after 10 min and 60 min by diluting 50 µL of the reaction mixture into 450 µL MeCN-d_3.

Control experiments for mechanistic studies

LA mediated reactivity of Fdx in the absence of thiol nucleophile

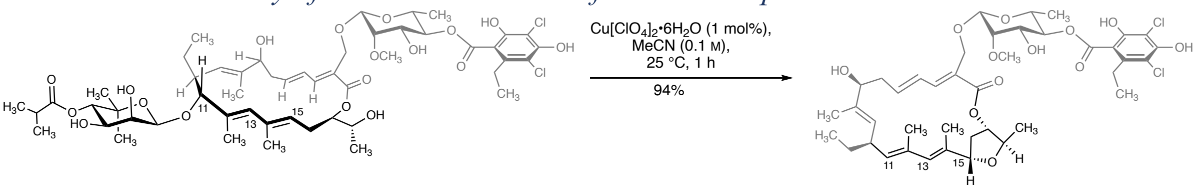

Fdx **15*S*-THF Fdx**

Fdx (47.3 µmol, 50 mg, 1.0 equiv.) was suspended in MeCN (451 µL, 0.1 M), followed by addition of a solution of $Cu[ClO_4]_2$•6 H_2O (10 mM in MeCN, 47.3 µL, 1 mol%). The mixture was stirred at 25 °C for 1 h. The reaction mixture was then diluted with CH_2Cl_2 (10 mL), and water (10 mL). The layers were separated, the aqueous phase was extracted with CH_2Cl_2 (3 x 10 mL), and the combined organic phase washed with brine (1x 30 mL), dried over anhydrous $MgSO_4$, filtered, and concentrated *in vacuo* to provide the intramolecular degradation product 15*S*-THF Fdx **2b**[5] as a colorless solid in a yield of 94% (44.5 µmol, 36 mg). The NMR spectroscopic data is consistent with previously reported data.[5]

^{1}H NMR (500 MHz, acetone-d_6): δ (ppm) 7.39 (d, *J*=11.3 Hz, 1H), 6.68 (dd, *J*=15.1, 11.4 Hz, 1H), 6.19 (ddd, *J*=14.8, 9.3, 5.1 Hz, 1H), 6.12 (s, 1H), 5.34 (d, *J*=9.9 Hz, 1H), 5.28 (d, *J*=7.9 Hz, 1H), 5.14 – 5.07 (m, 2H), 5.00 (dd, *J*=11.6, 3.8 Hz, 1H), 4.70 (s, 1H), 4.60 (d, *J*=11.6 Hz, 1H), 4.45 (d, *J*=11.8 Hz, 1H), 4.34 (s, 1H), 4.07 (q, *J*=6.6 Hz, 1H), 3.83 (dd, *J*=10.1, 3.5 Hz, 1H), 3.64 – 3.57 (m, 2H), 3.53 (s, 3H), 3.25 – 3.15 (m, 1H), 3.00 (q, *J*=7.3 Hz, 2H), 2.76 – 2.70 (m, 1H), 2.50 (ddd, *J*=14.4, 9.3, 3.6 Hz, 1H), 2.17 – 2.09 (m, 1H), 1.91 (s, 3H), 1.75 (s, 3H), 1.73 – 1.66 (m, 1H), 1.69 (s, 3H), 1.42 (dt, *J*=14.1, 7.1 Hz, 1H), 1.38 – 1.33 (m, 1H), 1.31 (d, *J*=6.3 Hz, 3H), 1.23 – 1.19 (m, 6H), 0.86 (t, *J*=7.4 Hz, 3H). **^{13}C NMR** (126 MHz, acetone-d_6) δ (ppm) 169.5, 166.8, 155.9, 153.8, 146.8, 145.3, 142.6, 136.1, 136.0, 134.7, 133.6, 133.1, 127.8, 127.1, 125.0, 114.5, 110.7, 108.2, 101.6, 81.7, 80.8, 80.2, 77.6, 77.2, 72.9, 72.3, 70.6, 63.0, 61.7, 39.6, 36.6, 35.8, 30.5, 26.2, 20.6, 18.5, 18.2, 18.1, 14.7, 14.4, 11.9. **HRMS** ESI(+) (MeOH) calculated for $C_{41}H_{54}O_{12}Cl_2Na^+$ $[M+Na]^+$: 831.28845, found: 831.28847.

Stability of the C(15)–isomer/ formal S_N2'' product under the reaction conditions

To exclude a mechanism in which the formal S_N2''-product, the C(15) isomer undergoes a rearrangement to provide the other regioisomers (C(11)- and C(13)-isomer), the stability of the isolated C(15) isomer of *S*-Glc-Fdx (**18a-(C15)**) under the reaction conditions in the absence of thiol nucleophile was studied. C(15)–*S*-Glc-Fdx **18a-(C15)** (2.98 µmol, 3.0 mg, 1.0 equiv.) was suspended in MeCN-d_3 (28 µL, 0.1 M), followed by addition of a solution of $Cu[ClO_4]_2$•6 H_2O (10 mM in MeCN, 2.98 µL, 1 mol%). The mixture was stirred at 25 °C for 1 h. After 1 h, the mixture was diluted into MeCN-d_3 (400 µL) and analyzed *via* UHPLC-MS and ^{1}H NMR analysis.

Reactivity of the C(15)–isomer / formal S_N2'' product towards thiol nucleophiles under the reaction conditions

To study whether the C(15)–isomer could undergo a subsequent reaction with the thiol nucleophile, the stability of the isolated C(15)–isomer of *S*-Glc-Fdx under the reaction conditions was studied. C(15)-*S*-Glc-Fdx **18a-C(15)** (12.3 µmol, 12.4 mg, 1.0 equiv.) and 1-thio-β-D-glucose tetraacetate **6a** (12.3 µmol, 4.5 mg, 1.0 equiv.) were suspended in MeCN (117.7 µL, 0.1 M), followed by addition of a solution of $Cu[ClO_4]_2$•6 H_2O (10 mM in MeCN, 12.3 µL, 1 mol%). The mixture was stirred at 25 °C for 1 h. The reaction was monitored *via* UHPLC-MS analysis.

15S-THF Fdx derivative is not a reaction intermediate

15*S*-THF Fdx **2b** (12.3 µmol, 10.0 mg, 1.0 equiv.) and 1-thio-β-D-glucose tetraacetate **6a** (12.3 µmol, 4.5 mg, 1.0 equiv.) were dissolved in MeCN (117.7 µL, 0.1 M), followed by addition of a solution of $Cu[ClO_4]_2$•6 H_2O (10 mM in MeCN, 12.3 µL, 1 mol%). The mixture was stirred at 25 °C for 1 h. The reaction was monitored *via* UHPLC-MS analysis.

The 15R-THF Fdx derivative is not a reaction intermediate

15*R*-THF Fdx **2a** (12.3 µmol, 10.0 mg, 1.0 equiv.) and 1-thio-β-D-glucose tetraacetate **6a** (12.3 µmol, 4.5 mg, 1.0 equiv.) were dissolved in MeCN (117.7 µL, 0.1 M), followed by addition of a solution of $Cu[ClO_4]_2$•6 H_2O (10 mM in MeCN, 12.3 µL, 1 mol%). The mixture was stirred at 25 °C for 1.5 h. The reaction was monitored *via* UHPLC-MS analysis.

Reaction in presence of radical trapping agent BHT

Fdx (18.9 µmol, 20.0 mg, 1.0 equiv.), and 4-methylbenzenethiol **4b** (18.9 µmol, 2.4 mg, 1.0 equiv.), were suspended in a solution of $Cu[ClO_4]_2$•6 H_2O (1 mM in MeCN, 189 µL, 1 mol%) in the presence (A) or absence (B) of butylated hydroxytoluene (18.9 µmol, 4.2 mg, 1.0 equiv.), followed by addition of MeCN (11 µL, 0.1 M). The mixture was stirred at 25 °C for 10 min. The reaction was monitored *via* UHPLC-MS analysis using the following LC time program (time – % B): 0.00 min – 5%, 0.50 min – 5%, 0.55 min – 30%, 3.50 min – 70%, 3.55 min – 100%.

Acid stability experiments

Acidic methanolysis

The experiments were analyzed by UHPLC-MS, using the following LC time program (time – % B) : 0.00 min – 5%, 0.50 min – 5%, 4.50 min – 95%, 4.55 min – 100%:

The retention times of the analytes and methanolysis degradation products (mixture of regioisomers; m/z: $[M+NH_4^+]$: 858, $[M+Na^+]$: 863) were as follows: Fdx - t_R: 3.7 min; *S*-Fdx – t_R: 3.7 - 3.8 min; degradation products - t_R: 3.9 - 4.0 min; caffeine: t_R: 1.4 - 1.5 min.

<u>Calibration curve for quantification:</u>

For quantification, a calibration curve was generated for both analytes, Fdx, and *S*-Fdx, independently, in the presence of caffeine as an internal standard. Quantification was performed by integrating the UV peaks of Fdx/*S*-Fdx and the peak of caffeine at an absorbance of 270 nm.

Solvent I: A solution of caffeine in MeOH (50 µg/mL) was prepared and used as solvent for the calibration curves.

A stock solution of Fdx/*S*-Fdx was prepared in solvent I at a concentration of 1 mg/mL, and then diluted 10-fold into solvent I to access a concentration of 100 µg/mL. A 2-fold serial dilution of Fdx/*S*-Fdx ranging from 100 µg/mL to 3.125 µg/mL was then prepared in solvent I. The samples were then subjected to UHPLC-MS analysis and the peaks at 270 nm integrated using the Thermo Xcalibur 3.0.41 Qual Browser software by Thermo Fisher Scientific.[3] Further analysis was carried out in GraphPad Prism *version* 10.1.2.[6] For both analytes, each sample was measured three times. The ratio of the integrals of the analyte and caffeine was plotted against the concentration of analyte (mean±S.D., n=3). A simple linear regression was performed without any constraints for both standard curves providing the following best-linear fit equations (Goodness of Fit: $R^2 \geq 0.9996$):

Fdx: $y = 0.007437x + 0.007070$ (Equation Fdx)

***S*-Fdx**: $y = 0.008056x + 0.008993$ (Equation S-Fdx)

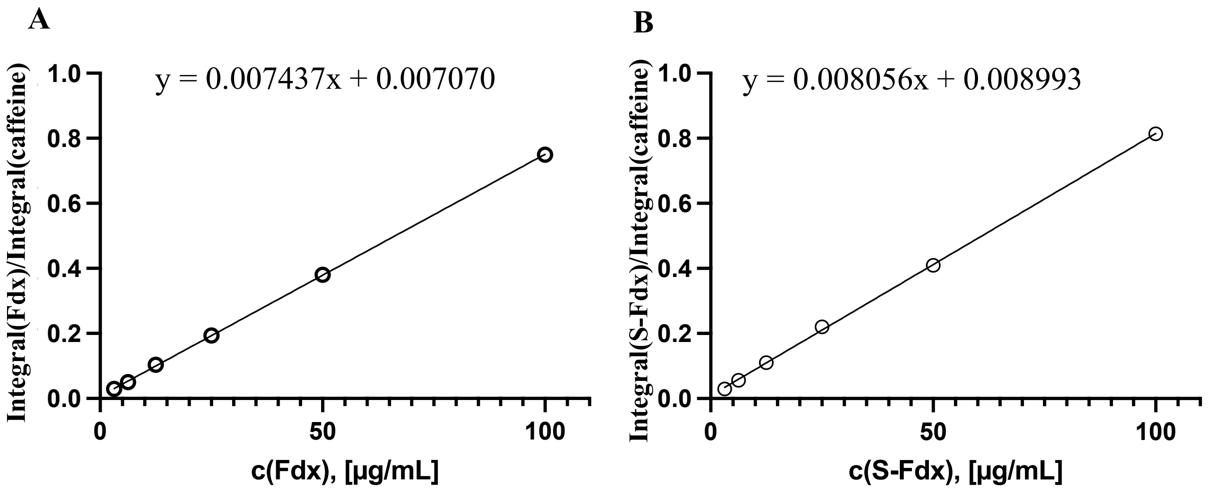

Figure 1: Standard curve of Fdx (A)/ *S*-Fdx (B) in the presence of caffeine as an internal standard. Data refers to mean±S.D. (n=3). Note: Error bars are too small to be displayed.

Table 1: Fdx calibration curve

**c (Fdx), [µg/mL]**	**Integral (Fdx)/Integral (caffeine) (mean, n=3)**	**S.D. (n=3)**
100.00	0.7509	0.0030
50.00	0.3787	0.0049
25.00	0.1936	0.0039
12.50	0.1014	0.0054
6.25	0.0518	0.0021
3.125	0.0273	0.0016

Table 2: *S*-Fdx calibration curve

c (*S*-Fdx), [µg/mL]	Integral (*S*-Fdx)/Integral (caffeine) (mean, n=3)	S.D. (n=3)
100.00	0.8102	0.0050
50.00	0.4084	0.0031
25.00	0.2175	0.0023
12.50	0.1112	0.0018
6.25	0.0578	0.0025
3.125	0.0297	0.0007

<u>Degradation of Fdx with HCl in MeOH</u>

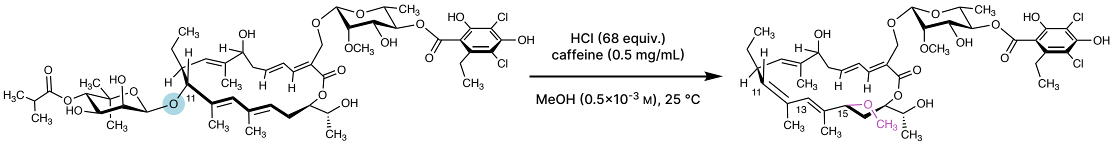

Solutions of caffeine in MeOH (1 mg/mL) and of Fdx in MeOH (1 mg/mL) were prepared. Then, in a 1.5 mL screw-cap vial equipped with a stirring bar 198 µL of the Fdx solution (1 mg/mL in MeOH) was diluted in 198 µL of caffeine solution (1 mg/mL). To the solution was then added 4.27 µL HCl (3 M solution in MeOH, freshly opened ampule, c_{final}: 0.032 M). The reaction mixture was stirred at 25 °C. At the indicated time points 5 µL of the reaction mixture were taken and diluted in 45 µL MeOH, and the sample subjected to UHPLC-MS analysis (each sample was measured twice).

<u>Degradation of *S*-Fdx with HCl in MeOH</u>

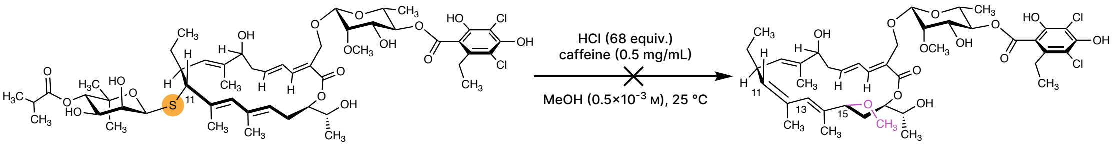

Solutions of caffeine in MeOH (1 mg/mL) and of *S*-Fdx in MeOH (1 mg/mL) were prepared. Then, in a 1.5 mL screw-cap vial equipped with a stirring bar 198 µL of the *S*-Fdx solution (1 mg/mL in MeOH) was diluted in 198 µL of caffeine solution (1 mg/mL). To the solution was then added 4.27 µL HCl (3 M solution in MeOH, freshly opened ampule, c_{final}: 0.032 M). The reaction mixture was stirred at 25 °C. At the indicated time points 5 µL of the reaction mixture were taken and diluted in 45 µL MeOH, and the sample subjected to UHPLC-MS analysis. The experiment was repeated two times. During each experiment the samples taken at the indicated time points were each measured twice.

The data of the degradation experiments of Fdx and *S*-Fdx with HCl in MeOH were analyzed with the Thermo Xcalibur 3.0.41 Qual Browser software by Thermo Fisher Scientific[3] to determine the ratio of the integrals of the absorbance peaks at 270 nm of the analyte and the internal standard caffeine. From these ratios, the concentration of analyte in the sample was calculated using the respective equation (Equation Fdx or equation *S*-Fdx). The concentration calculated at t = 0 min, was then set to 100% analyte remaining, respectively. Following concentrations were subtracted from the concentration at time point 0 min, and the remaining percentage of analyte calculated. These calculations were performed in Microsoft Excel *version* 16.79.1.[7] The percentage of analyte remaining was plotted over time in GraphPad Prism *version* 10.1.2.[6] For Fdx, the half-life was determined in GraphPad Prism *version* 10.1.2[6] by fitting (non-linear regression) to a standard one phase exponential decay equation.

Table 3: Degradation of Fdx in MeOH over time

Time, [min]	Integral (Fdx)/Integral (caffeine) (mean, n=2)	c (Fdx), [µg/mL] (mean, n=2)	% Remaining (mean, n=2)	S.D. [% remaining], (n=2)
0	0.4234	55.9801	100.00	0.00
10	0.3828	50.5282	90.26	0.48
30	0.3075	40.3931	72.16	0.30
45	0.2500	32.6713	58.36	0.04
90	0.1619	20.8225	37.20	0.49
180	0.0562	6.6119	11.81	0.12
240	0.0296	3.0357	5.42	0.50
270	0.0206	1.8152	3.24	0.24
320	0.0116	0.6132	1.10	0.23
390	0.0058	-0.1685	-0.30	0.39

Table 4: Degradation of *S*-Fdx in MeOH over time

Time, [min]	Integral (*S*-Fdx)/Integral (caffeine) (mean, n=2-4)	c (*S*-Fdx), [µg/mL] (mean, n=2-4)	% Remaining (mean, n=2-4)	S.D. [% remaining], (n=2-4)
0	0.3722	45.0831	100.00	0.00
10	0.3682	44.5940	98.91	0.75
30	0.3670	44.4398	98.58	0.96
45	0.3631	43.9513	98.34	1.11
90	0.3658	44.2859	98.23	0.43
180	0.3637	44.0244	97.66	0.90
360	0.3561	43.0854	96.40	0.66
450	0.3299	39.8340	87.60	4.11
540	0.3627	43.9043	98.23	1.48

NMR studies in MeOH-d_4 after 24h reveal a complex degradation mixture of Fdx:

Fdx (0.61 µmol, 0.65 mg, 1.0 equiv) was dissolved in MeOD-d_4 (440.2 µL, 0.001 M) and treated with HCl (3 M solution in MeOH, freshly opened ampule, 4.8 µL, 14.4 µmol, 24 equiv., c_{final}: 0.032 M). The solution was stirred at 25 °C for 24 h, before an ^{1}H NMR analysis of the mixture was performed.

S-Fdx (0.54 µmol, 0.58 mg, 1.0 equiv) was dissolved in MeOD-d_4 (440.2 µL, 0.001 M) and treated with HCl (3 M solution in MeOH, freshly opened ampule, 4.8 µL, 14.4 µmol, 27 equiv., c_{final}: 0.032 M). The solution was stirred at 25 °C for 24 h, before an ^{1}H NMR analysis of the mixture was performed.

Broth microdilution MIC assay against *C. difficile* strain ATCCBAA-1382

BHI I: Brain Heart Infusion (Oxoid; CM 1135) supplemented with 5 g/L yeast extract (Roth No. 2363.3) and 0.1% taurocholate (Roth No. 8149.3); (germination medium)
BHI II: Brain Heart Infusion (Oxoid; CM 1135) supplemented with 5 g/L yeast extract (Roth No. 2363.3) and 0.1% cysteine (Thermo Scientific; J63745.22); (pre-culture medium)
BHI III: Brain Heart Infusion (Oxoid; CM 1135) supplemented with 5 g/L yeast extract (Roth No. 2363.3) and 0.4% cysteine (Thermo Scientific; J63745.22), pH 7.2; (test medium)

Culture preparation:
8 mL of BHI I were inoculated with 200 µL of a spore suspension of *C. difficile* 630 (DSM No. 27543; ATCC No. BAA-1382). The liquid germination culture was incubated overnight at 37 °C in an anaerobic workstation (95% N2, 5% H2; Whitley A35 HEPA by Meintrup DWS). 10 µL of the germination culture were used further to inoculate 5 mL of freshly prepared BHI II. A 1:5 dilution series of the liquid culture was prepared (five dilution steps). The serial dilutions of this liquid pre-culture were incubated overnight at 37 °C under anaerobic conditions. Next day, a dilution of the pre-culture exhibiting an $OD_{600} \leq 2$ was chosen for inoculation of the test medium and diluted to an OD_{600} of 0.1 with BHI III. 100 µL of the OD_{600} adjusted pre-culture was used in the assay protocol:

Assay protocol:
A 96-well microtiter plate (TPP Switzerland; Nr.92096) was prepared as follows: Stock solutions of the test compounds were prepared in DMSO (Sigma; D8418; for molecular biology) at a concentration of 10 mg/mL. The stock solutions were then diluted in BHI III to a concentration of 64 µg/mL. 200 µL of the solutions were dispensed in the first well of a row in the 96 well plate, respectively. 2-fold serial dilutions in BHI III spanning a range of 64 µg/mL to 0.125 µg/mL were prepared over the 96 well plate, by diluting 100 µL of the higher concentration into 100 µL BHI III, respectively. To the serial dilutions was then added the same volume of the freshly prepared overnight culture (V_{final}: 200 µL; testing range: 32 µg/mL – 0.0625 µg/mL). The negative control consisted of BHI III only (200 µL). The organism growth control was performed in the absence of test compound (100 µL BHI III + 100 µL overnight culture). The 96 well plates were incubated overnight at 37 °C under anaerobic conditions. The OD_{600} was determined using a plate reader (BioTek; SynergyMx) (positive control: final OD_{600}: 0.6 – 0.7). The concentration of the test compound suppressing growth to $OD_{600} < 0.15$ was reported as MIC. Each compound was tested in one to three biological replicates, each of them in two technical replicates. For Fdx a concentration range of 2–0.004 µg/mL was tested.

Broth microdilution MIC assay against a panel of *C. difficile* and *C. perfringens*

MIC determinations against a panel of ten *C. difficile* (including Toxin A/B-producing strains) and two *C. perfringens* strains were performed as a paid service by Microbiologics (site: Microbiologics, INC (formerly: Micromyx, LLC), 4717 Campus Drive, Kalamazoo, MI, USA 49008). Susceptibility testing was conducted following guidelines per the Clinical and Laboratory Standards Institute (CLSI),[8,9] with the exception that broth microdilution was used for anaerobes (for non-Bacteroides spp.; the reference method is agar dilution).[8,9] Experiments were carried out in singlicate. The test compounds were provided in solid form (1–4 mg) and stored at -20 °C before testing. Stock solutions of the compounds in DMSO were prepared at the day of the assay at 101x the final testing concentration. The testing range for the compounds was 0.016-16 µg/mL. Stock solutions of comparator drugs were prepared at 101X the final testing concentration using solvents recommended by CLSI.[8,9] The following comparator compounds were provided by Microbiologics and the source specifications, drug diluent conditions, and testing range was as indicated:

Comparator drug	Supplier	Cat. No./ Lot No.	Testing range [µg/mL]	Solvent/Diluent
Metronidazole	Sigma	M3761-5G/ MKCJ4156	0.03–32	DMSO/H_2O
Fidaxomicin	Merck	F-109/ SEB1301001885	0.016–16	DMSO/H_2O
Clindamycin	Sigma	C5269-100MG/ 021M1533	0.008–8	H_2O/H_2O

Test organisms:

Test organisms consisted of isolates from the American Type Culture Collection (ATCC; Manassas, VA), the National Collection of Type Cultures (NCTC; Salisbury, UK) and the Microbiologics Repository (MMX; Kalamazoo, MI). Upon initial receipt at Microbiologics, the organisms were sub-cultured onto an appropriate agar medium and incubated under atmospheric conditions required for growth. Anaerobic organisms were handled in a Bactron II anaerobic chamber (Shel Lab, Cornelius, WA). Following incubation for 18–24 h at 35°C in the appropriate atmosphere, colonies were harvested from these plates and cell suspensions were prepared and frozen at -80°C with a cryoprotectant. Prior to testing, isolates were streaked from frozen vials onto Trypticase Soy Agar with 5% sheep blood (Remel; Lenexa, KS; Lot No. 693244) or Supplemented Brucella Agar (SBA; Becton Dickinson [BD]; Sparks, MD; Lot No. 3254602), and inoculated plates were incubated at 35°C overnight. The anaerobic bacteria Bacteroides fragilis (*B. fragilis)* ATCC25285, and *C. difficile* ATCC700057 were tested as quality controls.

Test Media

Organisms were tested in the appropriate media according to CLSI guidelines.[8,9] The medium for anaerobes consisted of pre-reduced Brucella Broth (BD; Lot No. 1327525) supplemented with 5 µg/mL hemin (Sigma; Lot No. SLBD8813V), 1 µg/mL Vitamin K1 (Sigma; Lot No. MKCB9432), and 5% LHB (Supplemented Brucella broth; SBB).

Broth Microdilution MIC Assay

The MIC assay method followed the procedures described by CLSI for each group of organisms[8,9] and employed automated liquid handlers (Multidrop Combi, Thermo Scientific, Waltham, MA; Biomek 3000, and Biomek FX, Beckman Coulter; Fullerton, CA) to conduct serial dilutions and liquid transfers. The wells in columns 2 through 12 in standard 96-well microdilution plates (Costar 3795) were filled with 150 µL of the appropriate diluent. These would become the "mother plates" from which "daughter", or test plates, would be prepared. The drugs (300 µL at 101x the desired top concentration in the test plates) were dispensed into the appropriate well in column 1 of each mother plate. The Biomek 3000 was used to make serial two-fold dilutions through column 11 in the "mother plate". The wells of column 12 contained no drug and served as the organism growth control wells.

Rows A through H of the daughter plates were loaded with 190 µL per well of the appropriate test medium using the Multidrop Combi. The daughter plates were prepared using the Biomek FX which transferred 2 µL of drug solution from each well of a mother plate to the corresponding well of the daughter plate in a single step.

A standardized inoculum of each organism was prepared per CLSI.[8,9] Colonies were picked from the streak plates and a suspension was prepared to equal a 0.5 McFarland (1 to $2x10^8$ CFU/mL for all organisms except *C. difficile*, for which a 0.5 McFarland is approximately 1 to 4 x 10^7 CFU/mL). Suspensions were then diluted 1:10.

A MatrixTM Multichannel Electronic Pipette (Thermo Scientific; Waltham, MA) was used to inoculate anaerobes in the anaerobic chamber, and the final target concentration for *C. difficile* was 5 x 10^4 CFU/mL. Inoculated plates were then covered with a sterile lid. Plates inoculated with anaerobes were stacked in a BD GasPak EZ Anaerobe Container System and incubated at 35°C for 46 to 48 hr.

After incubation, plates were viewed from the bottom using a plate viewer. An un-inoculated solubility control plate was observed for evidence of drug precipitation and contamination. MIC values were read where visible growth of the organism was inhibited.

Table 5: MIC values of *S*-Fdx derivatives against a panel of *C. difficile* and *C. perfringens* including QC strains and clindamycin susceptibility data

Organism[a]	Strain	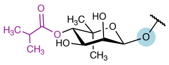 Fdx, 1	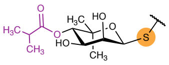 *S*-Fdx, 3a-C(11)	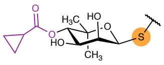 3b-C(11)	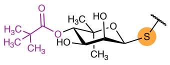 3c-C(11)	MET	CLI	
							MIC	SIR
B. fragilis	ATCC 25285 (QC)	>16	>16	>16	>16	1	1	S
C. perfringens	ATCC13124	≤0.016	0.12	0.5	0.06	1	0.06	S
	MMX8324[c]	≤0.016	0.06	0.12	0.25	2	>8	R
C. difficile	ATCC700057 (RT038, QC)[c]	≤0.016	0.5	0.5	1	0.12	4	I
	ATCCBAA-1805 (RT027)[b,c]	0.12	2	2	2	2	4	I
	ATCC43255 (RT087)[b,c]	0.06	0.5	1	1	0.5	4	I
	ATCC9689 (RT001)[b]	≤0.016	0.12	0.12	0.25	0.25	0.12	S
	ATCCBAA-1875 (RT078)[b,c]	0.03	1	2	2	0.25	4	I
	NCTC13366 (RT027)[c]	0.12	2	2	4	1	4	I
	MMX8260 (RT017)[c]	0.03	0.25	1	1	0.25	>8	R
	MMX8264 (RT027)[c]	0.12	2	4	4	2	4	I
	MMX8282 (RT017)[c]	0.03	0.25	1	1	0.25	>8	R
	MMX8290 (RT078)[c]	0.03	1	1	2	0.5	8	R

MIC values in µg/mL. [a]MIC determined by Microbiologics *via* broth microdilution assay. [b]toxigenic. [c]Clindamycin non- or intermediate susceptible. QC Quality control. RT Ribotype. MET Metronidazole. CLI Clindamycin. S susceptible, I intermediate, R resistant.

Characterization of compounds

Characterization of Fdx derivatives

General procedures

MeCN used was of analytical grade and no precautions were taken to avoid air or moisture for the reactions in General procedure A-C. A 10 mM-solution of copper(II)perchlorate in MeCN was prepared by dissolving copper(II)perchlorate hexahydrate ($Cu[ClO_4]_2$•6 H_2O, 19 mg) in MeCN (5 mL). The resulting solution was further diluted 10-fold into MeCN to obtain a 1 mM-solution of $Cu[ClO_4]_2$•6 H_2O in MeCN. For larger amounts of crude (>200 mg) or difficult separations, the crude was split on multiple runs for purification by RP-HPLC and the corresponding peaks of the runs combined for analysis or repurification. For regioisomers with very similar retention times, repurification was repeated under the indicated conditions for repurification until NMR analysis confirmed a single regioisomer.

General procedure A: Proof of concept studies

Fdx (**1**, 1.0 equiv.), and the thiol nucleophile (if solid) (1.0 equiv.) were suspended in a solution of $Cu[ClO_4]_2$•6 H_2O (1 mM in MeCN, 1 mol%), followed by addition of MeCN. To the suspension was added the respective thiol (if liquid) (1.0 – 5.0 equiv.) and the mixture stirred at 25 °C for 10 min. The reaction was monitored by UHPLC-MS/UV. After complete conversion of the starting material, the reaction mixture was diluted with CH_2Cl_2 (10 mL) and water (10 mL). The aqueous phase was extracted with CH_2Cl_2 (3×10 mL), the combined organic phase washed with brine (1×40 mL), dried over anhydrous $MgSO_4$, filtered, and concentrated *in vacuo*. Excess amounts of thiol were separated from the crude material by column chromatography on silica using CH_2Cl_2 as the eluent. The product containing mixture was then eluted from the column using EtOAc followed by removal of the solvent *in vacuo*. The residue was resuspended in MeCN and prepurified over a Supelco® Discovery® DSC-18 SPE tube (1 g). Further purification was performed by preparative RP-HPLC (Gemini NX C18, 5 µ, 110 Å, 250 mm × 21.2 mm; solvent A: H_2O + 0.1% HCOOH, solvent B: MeCN + 0.1% HCOOH; 20 mL/min) using the conditions specified in the individual experiment.

General procedure B: Synthesis of Fdx thioglycoside derivatives

Fdx (**1**, 1.0 equiv.), and the 1-thiosugar **6a-d**/**9** (1.0 equiv.) were suspended in MeCN (c_{final}: 0.1 - 0.2 M), followed by addition of a solution of $Cu[ClO_4]_2$•6 H_2O (10 mM in MeCN, 1 mol%) or where indicated the suspension was prepared in a solution of $Cu[ClO_4]_2$•6 H_2O (1 mM in MeCN, 1 mol%), followed by addition of MeCN, and the mixture stirred at 25 °C for 10 min. The reaction was monitored by UHPLC-MS. After complete conversion of the starting material, the reaction mixture was diluted with CH_2Cl_2 (10–20 mL) and water (10–20 mL). The aqueous phase was extracted with CH_2Cl_2 (3× 10‒20 mL), the combined organic phase washed with brine (1× 40‒80 mL), dried over anhydrous $MgSO_4$, filtered, and concentrated *in vacuo*. The aqueous phase was concentrated *in vacuo* separately. The crude material derived from the organic phase was dissolved in MeOH (0.1 M). Potassium carbonate (4 equiv.) was added to the solution and the mixture stirred at 25 °C. The reaction was monitored via UHPLC-MS analysis. After complete conversion of the starting material, the reaction was diluted with MeOH (10 mL) or MeCN (10 mL) (as indicated), and the mixture filtered over a Supelco® Discovery® DSC-18 SPE tube (2 g) to provide after concentration *in vacuo* the crude material. Further purification was performed by preparative RP-HPLC (Gemini NX C18, 5 µ, 110 Å, 250 mm × 21.2 mm; solvent A: H_2O + 0.1% HCOOH, solvent B: MeCN + 0.1% HCOOH; (or

where indicated MeOH + 0.1% HCOOH); 20 $^{mL}/_{min}$) using the conditions specified in the individual experiment.

General procedure C: Synthesis of 4''-O-acyl S-Fdx derivatives

Fdx (**1**, 1.0 equiv.) and 2,3-di-*O*-isopropylidene-4-*O*-acyl-1-β-thio-D-noviose **17a-c** (1.0 equiv.) were suspended in MeCN (0.1 M), followed by addition of water (20.0 equiv.) and then, a solution of Cu[ClO_4]$_2$•6 H_2O (10 mM in MeCN, 5 mol%). The mixture was stirred at 25 °C for 14 to 20 h. The reaction was monitored by UHPLC-MS. After complete conversion of the intermediately formed acetonide protected intermediate, the reaction mixture was diluted with CH_2Cl_2 (20–40 mL), and water (20–40 mL). The layers were separated, the aqueous phase was extracted with CH_2Cl_2 (3×20–40 mL), and the combined organic phase washed with brine (1×60-100 mL), dried over anhydrous $MgSO_4$, filtered, and concentrated *in vacuo*. The crude material was dissolved in MeCN and filtered over a Supelco® Discovery® DSC-18 SPE tube (0.5–1 g) to provide after concentration *in vacuo* the crude material for further purification by preparative RP-HPLC (Gemini NX C18, 5 µ, 110 Å, 250 mm × 21.2 mm; solvent A: H_2O + 0.1% HCOOH, solvent B: MeCN + 0.1% HCOOH; 18 $^{mL}/_{min}$) using the conditions specified in the individual experiment.

Fdx derivatives from proof of concept studies

11-Desnoviosyl-xy-p-methoxybenzylsulfide fidaxomicin (5a)

11-Desnoviosyl-xy-*p*-methoxybenzylsulfide fidaxomicin (**5a**) was synthesized from Fdx (**1**, 189 µmol, 200 mg, 1.0 equiv.) and 4-methoxy-benzylmercaptan (**4a**, 945 µmol, 132 µL, 5.0 equiv.) in a solution of Cu[ClO_4]$_2$•6 H_2O (1 mM in MeCN, 1892 µL, 1 mol%) and MeCN (108 µL) following General procedure A. The crude material was obtained as a pale yellow oil. Purification was performed by preparative RP-HPLC using a linear gradient of 40-60% B over 120 min (LC time program (time - %B): 0 min - 40%, 15 min – 40%, 135 min - 60%, 145 min – 70%, 146 min - 100%, 156 min - 100%). Product containing fractions were combined separately and concentrated *in vacuo*.

11-Desnoviosyl-11-*p*-methoxybenzylsulfide fidaxomicin (**5a-C(11)**) was obtained as a colorless solid in a yield of 11% (21.6 µmol, 20.8 mg, t_R = 127.0 min).

11-Desnoviosyl-13-*p*-methoxybenzylsulfide fidaxomicin (**5a-C(13)**) was obtained as a colorless solid in a yield of 44% (82.2 µmol, 79.2 mg, t_R = 115.0 min).

11-Desnoviosyl-15-*p*-methoxybenzylsulfide fidaxomicin (**5a-C(15)**) was obtained as a colorless solid in a yield of 15% (28.8 µmol, 27.8 mg, t_R = 135.0 min).

11-Desnoviosyl-11-p-methoxybenzylsulfide fidaxomicin (5a-C(11))

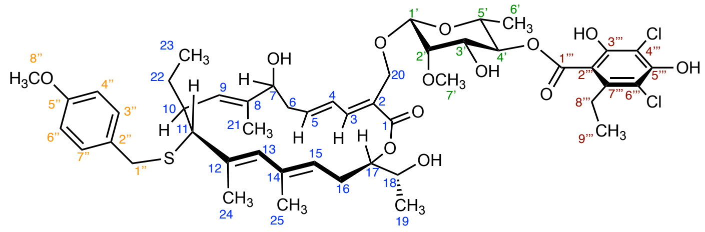

^{1}H NMR (400 MHz, acetone-d$_6$) δ (ppm) 7.29 – 7.22 (m, 3H, H3, H3'', H7''), 6.90 – 6.82 (m, 2H, H4'', H6''), 6.69 – 6.57 (m, 1H, H4), 5.98 (ddd, *J* = 14.7, 9.6, 4.6 Hz, 1H, H5), 5.65 (s, 1H, H13), 5.63 (d, *J* = 9.2 Hz, 1H, H15), 5.27 (dt, *J* = 10.1, 1.5 Hz, 1H, H9), 5.10 (t, *J* = 9.7 Hz, 1H, H4'), 4.74 (dt, *J* = 6.9, 4.7 Hz, 1H, H17), 4.68 (d, *J* = 0.8 Hz, 1H, H1'), 4.60 (d, *J* = 11.5 Hz, 1H, H20a), 4.42 (d, *J* = 11.5 Hz, 1H, H20b), 4.25 (d, *J* = 4.0 Hz, 1H, H7), 4.04 (p, *J* = 6.4 Hz, 1H, H18), 3.80 (dd, *J* = 9.9, 3.4 Hz, 1H, H3'), 3.77 (s, 3H, H8''), 3.66 – 3.59 (m, 2H,), 3.57 (d, *J* = 9.1 Hz, 2H), 3.52 (s, 3H, H7'), 3.08 (d, *J* = 11.0 Hz, 1H, H11), 3.00 (q, *J* = 7.4 Hz, 2H, H8'''), 2.79 – 2.72 (m, 1H, H16a), 2.74 – 2.64 (m, 1H, H6a), 2.56 – 2.40 (m, 3H, H10, H16b, H6b), 1.96 – 1.89 (m, 1H, H22a), 1.88 (d, *J* = 1.2 Hz, 3H, H24), 1.72 (d, *J* = 1.4 Hz, 3H, H25), 1.65 – 1.62 (m, 3H, H21), 1.31 (d, *J* = 6.2 Hz, 3H, H6'),

1.29 – 1.21 (m, 1H, H22b), 1.21 (t, *J* = 7.3 Hz, 2H, H9’’’), 1.19 (d, *J* = 6.2 Hz, 3H, H19), 0.72 (t, *J* = 7.4 Hz, 3H, H23). **^{13}C NMR** (101 MHz, acetone-d$_6$) δ (ppm) 169.5 (C1’’’), 167.7 (C1), 159.5 (C5’’), 155.9 (C3’’’), 153.8 (C5’’’), 145.5 (C3), 143.5 (C5), 142.7 (C7’’’), 136.5 (C8), 136.1 (C14), 134.4 (C12), 133.2 (C13), 131.6 (C2’’), 130.9 (C3’’, C7’’), 128.2 (C4), 126.2 (C9), 125.6 (C15), 125.2 (C2), 114.5 (C6’’’), 114.5 (C4’’, C6’’), 110.6 (C2’’’), 108.2 (C4’’’), 101.7 (C1’), 81.6 (C2’), 78.2 (C17), 77.6 (C4’), 72.9 (C7), 72.3 (C3’), 70.6 (C5’), 67.6 (C18), 63.3 (C20), 61.7 (C7’), 60.3 (C11), 55.5 (C8’’), 40.7 (C10), 37.2 (C6), 35.3 (C1’’), 28.4 (C16), 27.4 (C22), 26.2 (C8’’’), 20.7 (C19), 18.2 (C6’), 17.6 (C25), 15.1 (C21), 14.4 (C9’’’), 13.7 (C24), 10.6 (C23). **HRMS** ESI(+) (MeOH/CHCl$_3$ = 3:2) calculated for $C_{49}H_{64}O_{13}Cl_2NaS^+$ [M+Na]$^+$: 985.33369, found: 985.33395. **Specific Rotation** $[\alpha]_D^{24°C}$ = +84.5 (c = 0.38, MeOH). **FT-IR** (acetone-*d$_6$*): ν (cm^{-1}) 3407w, 2969w, 2934w, 1698m, 1609w, 1589w, 1511m, 1455w, 1405w, 1379m, 1313m, 1246s, 1213m, 1198m, 1179m, 1143m, 1112m, 1089m, 1067s, 1022s, 901w, 856w, 801w, 761w, 735w, 694w, 529w.

11-Desnoviosyl-13-p-methoxybenzylsulfide fidaxomicin (5a-C(13))

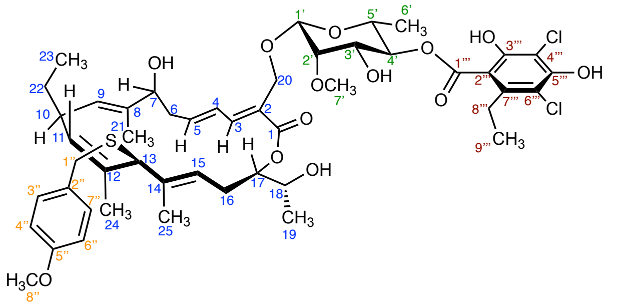

^{1}H NMR (acetone-*d$_6$*, 500 MHz): δ (ppm) 7.28 – 7.22 (m, 2H, H3’’, H7’’), 7.20 (d, *J*=11.4 Hz, 1H, H3), 6.88 – 6.84 (m, 2H, H4’’, H6’’), 6.64 – 6.54 (m, 1H, H4), 6.05 (ddd, *J*=15.1, 9.7, 5.6 Hz, 1H, H5), 5.71 (ddt, *J*=10.3, 6.2, 1.4 Hz, 1H, H15), 5.35 – 5.29 (m, 2H, H9, H11), 5.10 (t, *J*=9.7 Hz, 1H, H4’), 4.83 (dt, *J*=9.0, 3.3 Hz, 1H, H17), 4.61 (d, *J*=11.5 Hz, 1H, H20a), 4.60 (s, 1H, H1’), 4.48 (d, *J*=11.5 Hz, 1H, H20b), 4.33 – 4.26 (m, 1H, H7), 3.94 – 3.86 (m, 1H, H18), 3.77 (s, 3H, H8’’), 3.77 – 3.73 (m, 1H, H3’), 3.70 (s, 1H, H13), 3.68 – 3.61 (m, 1H, H5’), 3.60 – 3.54 (m, 1H, H2’), 3.56 (d, *J*=3.9 Hz, 2H, H1’’), 3.51 (s, 3H, H7’), 3.24 – 3.16 (m, 1H, H10), 3.01 (q, *J*=7.4 Hz, 2H, H8’’’), 2.72 (dddd, *J*=14.4, 5.6, 3.8, 1.6 Hz, 1H, H6a), 2.61 – 2.54 (m, 1H, H16a), 2.54 – 2.46 (m, 1H, H16b), 2.48 – 2.40 (m, 1H, H6b), 1.71 (d, *J*=1.3 Hz, 3H, H21), 1.62 (d, *J*=1.2 Hz, 3H, H24), 1.57 – 1.47 (m, 1H, H22a), 1.47 (s, 3H, H25), 1.45 – 1.35 (m, 1H, H22b), 1.31 (d, *J*=6.1 Hz, 3H, H6’), 1.22 (t, *J*=7.4 Hz, 3H, H9’’’), 1.15 (d, *J*=6.1 Hz, 3H, H19), 0.93 (t, *J*=7.3 Hz, 3H, H23); **^{13}C NMR** (126 MHz, acetone-*d$_6$*) δ (ppm) 169.6 (C1’’’), 166.6 (C1), 159.6 (C5’’), 156.2 (C3’’’), 154.0 (C5’’’), 144.9 (C3), 143.4 (C5), 142.9 (C7’’’), 137.2 (C14), 134.7 (C11), 134.3 (C8 or C12), 134.3 (C12 or C8), 131.4 (C2’’), 130.9 (C3’’, C7’’), 127.9 (C4), 127.0 (C9), 125.2 (C2), 121.4 (C15), 114.6 (C6’’’), 114.6 (C4’’, C6’’), 110.3 (C2’’’), 108.2 (C4’’’), 100.8 (C1’), 81.6 (C2’), 77.9 (C17), 77.7 (C4’), 72.8 (C7), 72.3 (C3’), 70.6 (C5’), 66.6 (C18), 62.6 (C20), 62.1 (C13), 61.6 (C7’), 55.5 (C8’’), 39.3 (C10), 37.6 (C6), 35.4 (C1’’), 30.1 (C22), 29.0 (C16), 26.3 (C8’’’), 21.2 (C19), 18.4 (C6’), 17.0 (C25), 14.7 (C21), 14.4 (C9’’’), 12.3 (C23), 12.1 (C24); **HRMS** ESI(−) (MeOH/CHCl$_3$ = 3:2) calculated for $C_{49}H_{63}O_{13}Cl_2S^-$ [M−H]$^-$: 961.33719, found: 961.33684. **R*$_f$*** (pentane/acetone = 1:1) = 0.8. **Specific Rotation** $[\alpha]_D^{24°C}$ = -148.1 (c = 0.34, MeOH). **FT-IR** (acetone-*d$_6$*): ν (cm^{-1}) 3406w, 2963m, 2933m, 1696s, 1644m, 1610w, 1587w, 1511m, 1454m, 1405m, 1374m, 1313m, 1243s, 1197s, 1175s, 1144m, 1112m, 1089s, 1065s, 1022s, 985m, 902w, 871w, 832w, 800w, 761m, 737w, 693w, 543w, 477w.

11-Desnoviosyl-15-p-methoxybenzylsulfide fidaxomicin (5a-C(15))

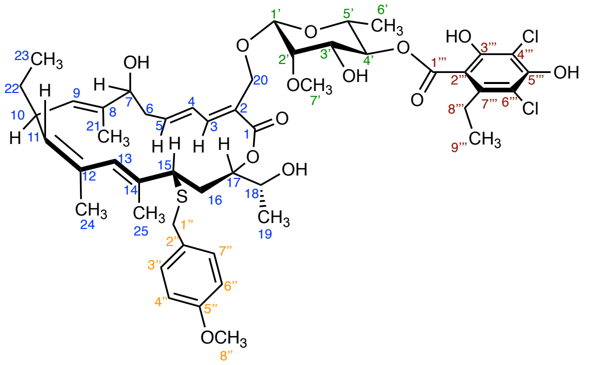

^{1}H NMR (400 MHz, acetone-d$_6$) δ (ppm) 7.23 – 7.17 (m, 2H, H3'', H7''), 7.09 (d, J = 11.2 Hz, 1H, H3), 6.88 – 6.82 (m, 2H, H4'', H6''), 6.32 (ddd, J = 15.1, 11.3, 1.3 Hz, 1H, H4), 6.18 (ddd, J = 15.0, 10.9, 4.0 Hz, 1H, H5), 5.48 (d, J = 1.5 Hz, 1H, H13), 5.19 (dt, J = 10.5, 1.4 Hz, 1H, H9), 5.11 (t, J = 9.7 Hz, 1H, H4'), 5.09 – 5.02 (m, 1H, H11), 5.06 – 4.99 (m, 1H, H17), 4.65 (d, J = 0.8 Hz, 1H, H1'), 4.61 (d, J = 10.9 Hz, 1H, H20a), 4.44 (d, J = 10.9 Hz, 1H, H20b), 4.30 (t, J = 3.5 Hz, 1H, H7), 3.82 (dd, J = 9.9, 3.4 Hz, 1H, H3'), 3.81 – 3.77 (m, 1H, H18), 3.78 (s, 3H, H8''), 3.60 – 3.53 (m, 2H, H2', H5'), 3.51 (s, 3H, H7'), 3.47 (s, 2H, H1''), 3.30 (dd, J = 10.9, 1.6 Hz, 1H, H15), 3.13 (tdd, J = 10.1, 8.5, 6.7 Hz, 1H, H10), 2.99 (q, J = 7.3 Hz, 2H, H8'''), 2.63 – 2.53 (m, 1H, H6b), 2.38 (ddd, J = 13.5, 10.9, 2.7 Hz, 1H, H6a), 2.22 (dt, J = 14.5, 11.3 Hz, 1H, H16a), 1.89 (d, J = 1.1 Hz, 3H, H25), 1.81 (d, J = 1.2 Hz, 3H, H24), 1.75 (ddd, J = 14.6, 2.7, 1.6 Hz, 1H, H16b), 1.59 (d, J = 1.4 Hz, 3H, H21), 1.41 – 1.35 (m, 1H, H22a), 1.33 (d, J = 6.2 Hz, 3H, H6'), 1.32 – 1.26 (m, 1H, H22b), 1.19 (t, J = 7.4 Hz, 3H, H9'''), 1.13 (d, J = 6.5 Hz, 3H, H19), 0.83 (t, J = 7.4 Hz, 3H, H23). **^{13}C NMR** (101 MHz, acetone-d$_6$) δ (ppm) 169.4 (C1'''), 167.5 (C1), 159.5 (C5''), 155.8 (C3'''), 153.9 (C5'''), 144.9 (C3), 143.2 (C5), 142.6 (C7'''), 137.7 (C11), 134.0 (C8), 133.8 (C13), 132.3 (C14), 132.1 (), 131.6 (C2''), 130.8 (C3'', C7''), 127.1 (C4), 127.0 (C9), 125.2 (C2), 114.6 (C6'''), 114.5 (C4'', C6''), 110.6 (C2'''), 108.3 (C4'''), 100.0 (C1'), 81.6 (C2'), 78.0 (C17), 77.5 (C4'), 73.9 (C7), 72.3 (C3'), 70.8 (C5'), 69.7 (C18), 61.9 (), 61.8 (), 55.5 (C8''), 54.4 (C15), 39.5 (C10), 38.4 (C6), 35.0 (C1''), 31.3 (C16), 30.5 (), 30.3 (C22), 26.2 (C8'''), 19.1 (C19), 18.0 (C6'), 17.1 (C24), 15.5 (C21), 14.3 (C9'''), 13.6 (C25), 12.0 (C23). **HRMS** ESI(+) (MeOH/$CHCl_3$ = 3:2) calculated for $C_{49}H_{64}O_{13}Cl_2NaS^+$ $[M+Na]^+$: 985.33369, found: 985.33391. **Specific Rotation** $[\alpha]_D^{24°C}$ = – 10.8 (c = 0.30, MeOH). **FT-IR** (acetone-d_6): ν (cm^{-1}) 3426w, 2963w, 2934w, 2873w, 1698m, 1640w, 1610w, 1588w, 1511m, 1455w, 1442w, 1405w, 1372w, 1312m, 1300m, 1238s, 1198m, 1176m, 1144w, 1107m, 1091m, 1070s, 1022s, 988w, 917w, 900w, 857w, 824w, 801w, 761w, 736w, 693w, 676w, 581w, 531w, 510w, 485w, 478w.

11-Desnoviosyl-xy-p-tolylsulfide fidaxomicin (5b)

11-Desnoviosyl-xy-*p*-tolylsulfide fidaxomicin (**5b**) was synthesized from Fdx (**1**, 189 µmol, 200 mg, 1.0 equiv.) and 4-methylbenzenethiol (**4b**, 189 µmol, 23.5 mg, 1.0 equiv.) in a solution of $Cu[ClO_4]_2$•6 H_2O (1 mM in MeCN, 1892 µL, 1 mol%) and MeCN (108 µL) following General procedure A. However, no column chromatography on silica was performed. The crude material was obtained as a pale yellow solid. Purification was performed by preparative RP-HPLC using a linear gradient of 45-65% B over 120 min (LC time program (time - %B): 0 min - 45%, 15 min – 45% , 135 min - 65%, 155 min - 75%, 156 min - 100%, 166 min - 100%). Product containing fractions were combined separately and the C15 isomer was repurified by preparative RP-HPLC using the same conditions.

11-Desnoviosyl-11-*p*-tolylsulfide fidaxomicin (**5b-C(11)**) was obtained as a colorless solid in a yield of 15% (27.7 µmol, 25.9 mg, t_R = 99.2 min)

11-Desnoviosyl-13-*p*-tolylsulfide fidaxomicin (**5b-C(13)**) was obtained as a colorless solid in a yield of 9% (16.3 µmol, 15.2 mg, t_R = 94.8 min) and a purity of approx. 92% (determined from ^{1}H NMR spectrum by comparing the integral of a 1H signal to the sum of the integrals of this signal and the respective signal of the impurity.).

11-Desnoviosyl-15-*p*-tolylsulfide fidaxomicin (**5b-C(15)**) was obtained as a colorless solid in a yield of 39% (73.0 µmol, 68.2 mg, t_R = 112.4 min).

11-Desnoviosyl-11-p-tolylsulfide fidaxomicin (5b-C(11))

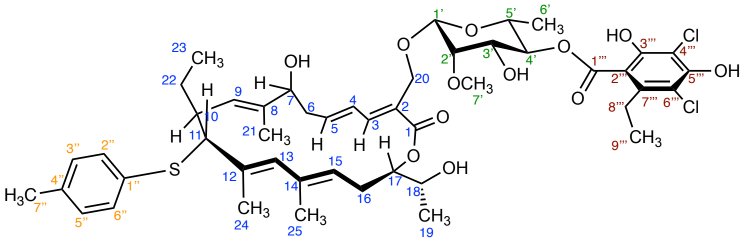

^{1}H NMR (500 MHz, acetone-d_6) δ (ppm) 7.32 – 7.27 (m, 2H, H2'', H6''), 7.21 (d, J = 11.4 Hz, 1H, H3), 7.10 (d, J = 7.8 Hz, 2H, H3'', H5''), 6.66 – 6.57 (m, 1H, H4), 5.98 (ddd, J = 14.7, 9.5, 4.7 Hz, 1H, H5), 5.34 (dt, J = 10.1, 1.6 Hz, 1H, H9), 5.31 (s, 1H, H13), 5.22 (t, J = 8.2 Hz, 1H, H15), 5.09 (t, J = 9.7 Hz, 1H, H4'), 4.69 (dd, J = 7.0, 4.6 Hz, 1H, H17), 4.66 (s, 1H, H1'), 4.58 (d, J = 11.5 Hz, 1H, H20a), 4.40 (d, J = 11.5 Hz, 1H, H20b), 4.27 (t, J = 3.7 Hz, 1H, H7), 3.97 (p, J = 6.4 Hz, 1H, H18), 3.79 (dd, J = 9.9, 3.4 Hz, 1H, H3'), 3.65 – 3.59 (m, 1H, H5'), 3.58 (d, J = 3.5 Hz, 1H, H2'), 3.51 (s, 3H, H7'), 3.50 (d, J = 6.8 Hz, 1H, H11), 3.00 (q, J = 7.3 Hz, 2H, H8'''), 2.71 (ddt, J = 15.5, 5.0, 2.4 Hz, 1H, H6b (H_{Re})), 2.65 – 2.57 (m, 2H, H16a (H_{Si}), H10), 2.50 (ddd, J = 14.9, 9.5, 4.3 Hz, 1H, H6a (H_{Si})), 2.34 (ddd, J = 14.1, 9.4, 4.6 Hz, 1H, H16b, (H_{Re})), 2.28 (s, 3H, H7''), 2.19 – 2.11 (m, 1H, H22a (H_{Re})), 1.84 (d, J = 1.3 Hz, 3H, H24), 1.68 (d, J = 1.2 Hz, 3H, H21), 1.56 (d, J = 1.3 Hz, 3H, H25), 1.46 (dt, J = 13.5, 7.6 Hz, 1H, H22b (H_{Si})), 1.30 (d, J = 6.2 Hz, 3H, H6'), 1.21 (t, J = 7.3 Hz, 3H, H9'''), 1.16 (d, J = 6.2 Hz, 3H, H19), 0.89 (t, J = 7.4 Hz, 3H, H23). **^{13}C NMR** (126 MHz, acetone-d_6) δ (ppm) 169.5 (C1'''), 167.6 (C1), 155.9 (C3'''), 153.8 (C5'''), 145.5 (C3), 143.6 (C5), 142.7 (C7'''), 138.1 (C4''), 136.7 (C8), 135.9 (C14), 135.0 (C2'', C6''), 134.0 (C12), 133.4 (C13), 132.9 (C1''), 130.1 (C3'', C5''), 128.1 (C4), 126.0 (C9), 125.2 (C15), 125.2 (C2), 114.5 (C6'''), 110.6 (C2'''), 108.2 (C4'''), 101.7 (C1'), 81.6 (C2'), 78.0 (C17), 77.6 (C4'), 72.9 (C7), 72.3 (C3'), 70.6 (C5'), 67.5 (C18), 66.7 (C11), 63.3 (C20), 61.7 (C7'), 41.2 (C10), 37.2 (C6), 28.3 (C16), 27.8 (C22), 26.2 (C8'''), 21.1 (C7''), 20.7 (C19), 18.2 (C6'), 17.4 (C25), 15.1 (C21), 14.4 (C9'''), 13.7 (C24), 10.8 (C23). **HRMS** ESI(+) (MeOH/$CHCl_3$ = 3:2) calculated for $C_{48}H_{62}O_{12}Cl_2NaS$ $[M+Na]^+$: 955.32312, found: 955.32288. **R_f** (pentane/acetone = 1:1) = 0.9. **Specific Rotation** $[\alpha]_D^{27°C}$ = +146.1 (c=0.22, MeOH). **FT-IR** (acetone-d_6): ν (cm^{-1}) 3406w, 2972w, 2933w, 2875w, 1696s, 1590w, 1491w, 1455w, 1404w, 1379m, 1313s, 1245s, 1212s, 1143m, 1113m, 1089s, 1067s, 1021s, 901w, 857w, 810w, 762w, 736w, 696w, 490w.

11-Desnoviosyl-13-p-tolylsulfide fidaxomicin (5b-C(13))

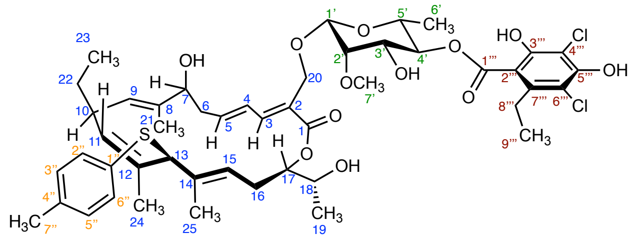

^{1}H NMR (500 MHz, acetone-d_6) δ (ppm) 7.27 (d, J = 8.0 Hz, 2H, H2'', H6''), 7.20 (d, J = 11.4 Hz, 1H, H3), 7.12 (d, J = 7.8 Hz, 2H, H3'', H5''), 6.58 (dd, J = 15.0, 11.4 Hz, 1H, H4), 6.02 (ddd, J = 15.1, 9.6, 5.6 Hz, 1H, H5), 5.88 – 5.80 (m, 1H, H15), 5.22 (dt, J = 9.9, 1.6 Hz, 1H, H9), 5.09 (t, J = 9.8 Hz, 1H, H4'), 4.99 – 4.93 (m, 1H, H11), 4.84 (dt, J = 9.1, 3.4 Hz, 1H, H17), 4.61 (d, J = 11.6 Hz, 1H, H20a), 4.60 (s, 1H, H1'), 4.48 (d, J = 11.5 Hz, 1H, H20b), 4.25 (s, 1H, H7), 4.13 (s, 1H, H13), 3.98 – 3.88 (m, 1H, H18), 3.75 (dd, J = 9.9, 3.4 Hz, 1H, H3'), 3.68 – 3.59 (m, 1H, H5'), 3.56 (d, J = 3.4 Hz, 1H, H2'), 3.51 (s, 3H, H7'), 3.00 (q, J = 7.3 Hz, 2H, H8'''), 2.87 (dt, J = 9.8, 7.3 Hz, 1H, H10), 2.73 – 2.65 (m, 1H, H6b (H_{Re})), 2.64 – 2.57 (m, 1H, H16a (H_{Si})), 2.53 (ddd, J = 14.2, 10.2, 3.6 Hz, 1H, H16b (H_{Re})), 2.42 (ddd, J = 14.0, 9.7, 3.4 Hz, 1H, H6a (H_{Si})), 2.28 (s, 3H, H7''), 1.64 (s, 3H, H21), 1.59 (s, 3H, H24), 1.53 (s, 3H, H25), 1.30 (d, J = 6.2 Hz, 3H, H6'), 1.21 (t, J = 7.3 Hz, 3H, H9'''), 1.17 (d, J = 6.1 Hz, 3H, H19), 1.14 – 1.03 (m, 2H, H22), 0.52 (t, J = 7.4 Hz, 3H, H23). **^{13}C NMR** (126 MHz, acetone-d_6) δ (ppm) 169.6 (C1'''), 166.7 (C1), 156.1 (C3'''), 154.0 (C5'''), 145.0 (C3), 143.5 (C5), 142.8 (C7'''), 137.9 (C4''), 137.2 (C14), 135.0 (C11), 134.3 (C2'', C6''), 133.9 (C8), 133.3 (C12), 132.7 (C1''),

130.3 (C3”, C5”), 127.9 (C4), 127.1 (C9), 125.2 (C2), 121.5 (C15), 114.6 (C6’”), 110.4 (C2’”), 108.2 (C4’”), 100.8 (C1’), 81.6 (C2’), 77.9 (C17), 77.6 (C4’), 72.8 (C7), 72.3 (C3’), 70.6 (C5’), 66.9 (C13), 66.7 (C18), 62.6 (C20), 61.6 (C7’), 39.1 (C10), 37.6 (C6), 29.7 (C22), 29.1 (C16), 26.3 (C8’”), 21.2 (C19), 21.0 (C7”), 18.3 (C6’), 17.1 (C25), 14.6 (C21), 14.4 (C9’”), 12.0 (C24), 11.7 (C23). **HRMS** ESI(+) (MeOH/$CHCl_3$ = 3:2) calculated for $C_{48}H_{62}O_{12}Cl_2NaS^+$ $[M+Na]^+$: 955.32312, found: 955.32173. $\mathbf{R_f}$ (pentane/acetone = 1:1) = 0.9. **Specific Rotation** $[\alpha]_D^{27°C}$ = -22.5 (c=0.24, MeOH). **FT-IR** (acetone-d_6): ν (cm^{-1}) 3406w, 2969w, 2932w, 2874w, 1697s, 1656w, 1590w, 1492w, 1454w, 1404w, 1376m, 1312m, 1243s, 1197m, 1143w, 1113m, 1089m, 1065s, 1021s, 986m, 903w, 872w, 858w, 809w, 761w, 736w, 693w, 635w, 581w, 499w.

11-Desnoviosyl-15-p-tolylsulfide fidaxomicin (5b-C(15))

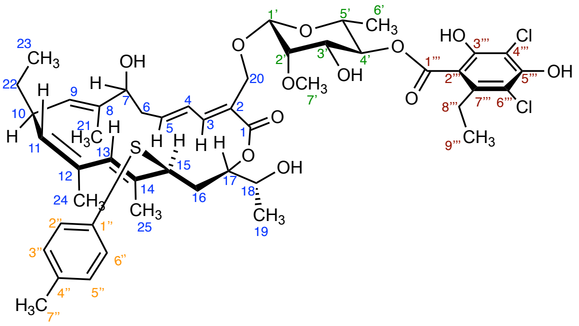

^{1}H NMR (500 MHz, acetone-d_6) δ (ppm) 7.22 (d, J = 7.9 Hz, 2H, H2”, H6”), 7.10 (d, J = 7.8 Hz, 2H, H3”, H5”), 7.04 (d, J = 11.4 Hz, 1H, H3), 6.35 – 6.24 (m, 1H, H4), 6.14 (ddd, J = 15.1, 11.0, 4.1 Hz, 1H, H5), 5.15 – 5.08 (m, 3H, H9, H17, H4’), 5.06 (s, 1H, H13), 4.64 (s, 1H, H1’), 4.61 (d, J = 9.5 Hz, 1H, H11), 4.59 (d, J = 10.9 Hz, 1H, H20b), 4.42 (d, J = 10.9 Hz, 1H, H20a), 4.26 (t, J = 3.4 Hz, 1H, H7), 3.87 – 3.78 (m, 2H, H18, H3’), 3.65 (dd, J = 11.1, 1.5 Hz, 1H, H15), 3.58 (d, J = 3.4 Hz, 1H, H2’), 3.57 – 3.52 (m, 1H, H5’), 3.51 (s, 3H, H7’), 3.03-2.95 (m, 3H, H10, H8’”), 2.59 – 2.51 (m, 1H, H6a (H_{Si})), 2.37 (ddd, J = 13.8, 11.0, 2.7 Hz, 1H, H6b (H_{Re})), 2.33 – 2.24 (m, 1H, H16a (H_{Si})), 2.28 (s, 3H, H7”), 1.96 (dt, J = 15.0, 2.0 Hz, 1H, H16b (H_{Re})), 1.86 (s, 3H, H25), 1.63 (s, 3H, H24), 1.55 (s, 3H, H21), 1.32 (d, J = 6.2 Hz, 3H, 6’), 1.24 – 1.20 (m, 2H, H22a (H_{Re}), H22b (H_{Si})), 1.19 (t, J = 7.4 Hz, 3H, H9’”), 1.15 (d, J = 6.4 Hz, 3H, H19), 0.76 (t, J = 7.3 Hz, 3H, H23). **^{13}C NMR** (126 MHz, acetone-d_6) δ (ppm) 169.4 (C1’”), 167.6 (C1), 155.8 (C3’”), 153.9 (C5’”), 144.8 (C3), 143.1 (C5), 142.6 (C7’”), 138.4 (C4”), 137.2 (C11), 135.4 (C2”, C6”), 134.2 (C13), 133.8 (C8), 132.1 (C1”), 131.9 (C12), 131.3 (C14), 130.1 (C3”, C5”), 127.0 (C4), 126.9 (C9), 125.2 (C2), 114.6 (C6’”), 110.6 (C2’”), 108.2 (C4’”), 100.0 (C1’), 81.6 (C2’), 77.9 (C17), 77.5 (C4’), 73.7 (C7), 72.3 (C3’), 70.8 (C5’), 69.7 (C18), 61.9 (C20), 61.8 (C7’), 60.1 (C15), 39.4 (C10), 38.4 (C6), 31.1 (C16), 29.6 (C22), 26.2 (C8’”), 21.1 (C7”), 19.1 (C19), 18.0 (C6’), 17.0 (C24), 15.4 (C21), 14.4 (C9’”), 13.9 (C25), 11.9 (C23). **HRMS** ESI(+) (MeOH/$CHCl_3$ = 3:2) calculated for $C_{48}H_{62}O_{12}Cl_2NaS^+$ $[M+Na]^+$: 955.32312, found: 955.32390. $\mathbf{R_f}$ (pentane/acetone = 1:1) = 0.9. **Specific Rotation** $[\alpha]_D^{27°C}$ = +94.1 (c=0.66, MeOH). **FT-IR** (acetone-d_6): ν (cm^{-1}) 3437w, 2964w, 2932w, 2873w, 1698m, 1639m, 1591w, 1492w, 1405w, 1377m, 1311m, 1237s, 1198m, 1179m, 1144m, 1091m, 1071s, 1022s, 916w, 856w, 809w, 762w, 736w, 677w, 646w, 581w, 530w, 488w.

11-Desnoviosyl-xy-p-tert-butylbenzenesulfide fidaxomicin (5c)

11-Desnoviosyl-xy-*p*-*tert*-butylbenzenesulfide fidaxomicin (**5c**) was synthesized from Fdx (**1**, 142 µmol, 150 mg, 1.0 equiv.) and 4-*tert*-butylbenzenethiol (**4c**, 142 µmol, 23.9 µL, 1.0 equiv.) in a solution of $Cu[ClO_4]_2$•6 H_2O (1 mM in MeCN, 1419 µL, 1 mol%) and MeCN (81 µL) following General procedure A. The crude material was obtained as a pale yellow solid.

However, the crude material was separated from residual thiol, using an eluent of acetone/pentane = 4:6. Further purification was performed by preparative RP-HPLC using a linear gradient of 55-75% B over 120 min (LC time program (time - %B): 0 min - 55%, 15 min – 55%, 135 min - 75%, 155 min - 75%, 156 min - 100%, 166 min - 100%. Product containing fractions were combined separately:

11-Desnoviosyl-11-*p*-*tert*-butylbenzenesulfide fidaxomicin **(5c-C(11))** was obtained as a colorless solid in a yield of 18% (26.1 µmol, 25.5 mg, t_R =91.2 min).

11-Desnoviosyl-13-*p*-*tert*-butylbenzenesulfide fidaxomicin (**5c-C(13)**) was obtained as a colorless solid in a yield of 9% (13.4 µmol, 13.1 mg, t_R =86.7 min).
11-Desnoviosyl-15-*p*-*tert*-butylbenzenesulfide fidaxomicin (**5c-C(15)**) was obtained as a colorless solid in a yield of 25% (35.8 µmol, 34.9 mg, t_R =99.5 min) and a purity of approx. 92% (determined from ^{1}H NMR spectrum by comparing the integral of a 1H signal to the sum of the integrals of this signal and the respective signal of the impurity).

11-Desnoviosyl-11-p-tert-butylbenzenesulfide fidaxomicin (5c-C(11))

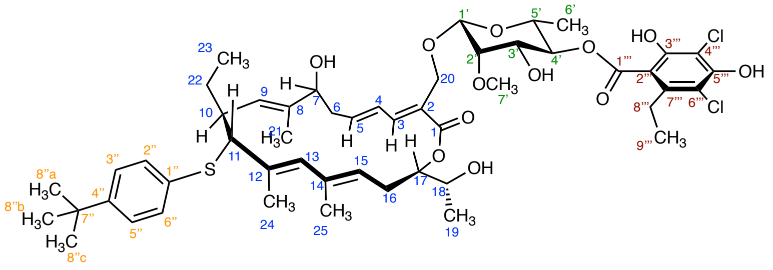

^{1}H NMR (500 MHz, acetone-d_6,): δ (ppm) 7.32 (s, 4H, H2'', H3'', H5'', H6''), 7.17 (d, *J*=11.5 Hz, 1H, H3), 6.66 – 6.57 (m, 1H, H4), 5.96 (ddd, *J*=14.7, 9.5, 4.7 Hz, 1H, H5), 5.33 (dt, *J*=10.3, 1.5 Hz, 1H, H9), 5.28 (s, 1H, H13), 5.12 – 5.05 (m, 2H, H15, H4'), 4.66 (s, 1H, H1'), 4.69 – 4.62 (m, 1H, H17), 4.58 (d, *J*=11.5 Hz, 1H, H20a), 4.39 (d, *J*=11.4 Hz, 1H, H20b), 4.27 (t, *J*=4.2 Hz, 1H, H7), 3.94 (p, *J*=6.3 Hz, 1H, H18), 3.79 (dd, *J*=9.9, 3.4 Hz, 1H, H3'), 3.64 – 3.57 (m, 2H, H2', H5'), 3.56 (d, *J*=11.0 Hz, 1H, H11), 3.50 (s, 3H, H7'), 2.99 (q, *J*=7.3 Hz, 2H, H8'''), 2.71 (ddt, *J*=15.5, 4.9, 2.4 Hz, 1H, H6a), 2.67 – 2.55 (m, 2H, H10, H16a), 2.50 (ddd, *J*=15.0, 9.6, 4.3 Hz, 1H, H6b), 2.30 (ddd, *J*=14.0, 9.1, 4.5 Hz, 1H, H16b), 2.12 (tdd, *J*=11.6, 5.6, 1.8 Hz, 1H, H22a), 1.81 (d, *J*=1.2 Hz, 3H, H24), 1.68 (s, 3H, H21), 1.56 (d, *J*=1.4 Hz, 3H, H25), 1.48 – 1.38 (m, 1H, H22b), 1.30 (d, *J*=6.3 Hz, 3H, H6'), 1.28 (s, 9H, H8''a, H8''b, H8''c), 1.21 (t, *J*=7.4 Hz, 3H, H9'''), 1.14 (d, *J*=6.3 Hz, 3H, H19), 0.89 (t, *J*=7.4 Hz, 3H, H23). **^{13}C NMR** (126 MHz, acetone-d_6) δ (ppm) 169.5 (C1'''), 167.7 (C1), 155.9 (C3'''), 153.9 (C5'''), 151.2 (C4''), 145.3 (C3), 143.4 (C5), 142.6 (C7'''), 136.6 (C8), 135.8 (C14), 134.7 (C2'', C6''), 133.9 (C13), 133.6 (C12), 132.9 (C1''), 128.1 (C4), 126.4 (C3'', C5''), 126.0 (C9), 125.4 (C15), 125.3 (C2), 114.6 (C6'''), 110.6 (C2'''), 108.2 (C4'''), 101.7 (C1'), 81.6 (C2'), 78.1 (C17), 77.6 (C4'), 72.9 (C7), 72.3 (C3'), 70.6 (C5'), 67.6 (C18), 66.5 (C11), 63.3 (C20), 61.7 (C7'), 41.4 (C10), 37.2 (C6), 35.1 (C7''), 31.6 (C8''a, C8''b, C8''c), 28.3 (C16), 27.7 (C22), 26.2 (C8'''), 20.6 (C19), 18.2 (C6'), 17.3 (C25), 15.2 (C21), 14.4 (C9'''), 13.6 (C24), 10.9 (C23). **HRMS** ESI(+) (MeOH/$CHCl_3$ = 3:2) calculated for $C_{51}H_{68}O_{12}Cl_2NaS^+$ $[M+Na]^+$: 997.37007, found: 997.36965. **R_f** (pentane/acetone = 7:3) = 0.3. **Specific Rotation** $[\alpha]_D^{27°C}$ = +106.1 (c = 0.83, MeOH). **FT-IR** (acetone-d_6): ν (cm^{-1}) 3386w, 2964w, 2934w, 2873w, 1697s, 1590w, 1489w, 1456w, 1380w, 1312m, 1245s, 1212m, 1198m, 1143w, 1113m, 1089m, 1066s, 1021s, 986w, 900w, 828w, 800w, 761w, 734w, 694w, 557w.

11-Desnoviosyl-13-p-tert-butylbenzenesulfide fidaxomicin (5c-C(13))

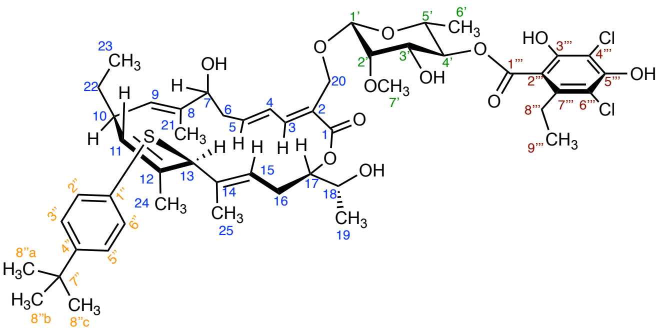

^{1}H NMR (500 MHz, acetone-d_6) δ (ppm) 7.38 – 7.33 (m, 2H, H3'', H5''), 7.33 – 7.29 (m, 2H, H2'', H6''), 7.20 (d, *J* = 11.4 Hz, 1H, H3), 6.58 (dd, *J* = 15.0, 11.4 Hz, 1H, H4), 6.02 (ddd, *J* = 15.1, 9.6, 5.6 Hz, 1H, H5), 5.86 – 5.79 (m, 1H, H15), 5.21 (dt, *J* = 9.7, 1.6 Hz, 1H, H9), 5.10 (t, *J* = 9.7 Hz, 1H, H4'), 4.96 – 4.90 (m, 1H, H11), 4.87 – 4.82 (m, 1H, H17), 4.61 (d, *J* = 11.5 Hz, 1H, H20a), 4.60 (s, 1H, H1'), 4.48 (d, *J* = 11.5 Hz, 1H, H20b), 4.25 (t, *J* = 3.9 Hz, 1H, H7), 4.15 (s, 1H, H13), 3.99 – 3.90 (m, 1H, H18), 3.76 (dd, *J* = 9.9, 3.4 Hz, 1H, H3'), 3.64 (dq, *J* = 9.5, 6.1 Hz, 1H, H5'), 3.57 (d, *J* = 3.4 Hz, 1H, H2'), 3.52 (s, 3H, H7'), 3.00 (q, *J* = 7.4 Hz, 2H, H8'''), 2.90 – 2.81 (m, 1H,

H10), 2.74 – 2.65 (m, 1H, H6a), 2.64 – 2.58 (m, 2H, H16a), 2.58 – 2.51 (m, 1H, H16b), 2.42 (ddd, *J* = 13.9, 9.7, 3.4 Hz, 1H, H6b), 1.64 (s, 3H, H21), 1.59 (s, 3H, H24), 1.54 (s, 3H, H25), 1.30 (d, *J* = 6.4 Hz, 3H, H6’), 1.29 (s, 9H, H8”a, H8”b, H8”c), 1.21 (t, *J* = 7.4 Hz, 3H, H9’’’), 1.18 (d, *J* = 6.1 Hz, 3H, H19), 1.11 – 0.97 (m, 2H, H22a, H22b), 0.49 (t, *J* = 7.4 Hz, 3H, H23). **^{13}C NMR** (126 MHz, acetone-d$_6$) δ (ppm) 169.7 (C1’’’), 166.7 (C1), 156.2 (C3’’’), 154.1 (C5’’’), 151.1 (C4”), 145.0 (C3), 143.5 (C5), 142.8 (C7’’’), 137.3 (C14), 135.0 (C11), 134.3 (C2”, C6”), 133.9 (C8), 133.2 (C12), 132.7 (C1”), 127.9 (C4), 127.1 (C9), 126.6 (C3”, C5”), 125.2 (C2), 121.4 (C15), 114.6 (C6’’’), 110.2 (C2’’’), 108.2 (C4’’’), 100.8 (C1’), 81.7 (C2’), 78.0 (C17), 77.6 (C4’), 72.8 (C7), 72.4 (C3’), 70.6 (C5’), 66.9 (C13), 66.7 (C18), 62.6 (C20), 61.7 (C7’), 39.0 (C10), 37.6 (C6), 35.0 (C7”), 31.6 (C8”a, C8”b, C8”c), 29.8 (C22), 29.1 (C16), 26.3 (C8’’’), 21.3 (C19), 18.3 (C6’), 17.1 (C25), 14.6 (C21), 14.4 (C9’’’), 12.0 (C23), 11.9 (C24). **HRMS** ESI(+) (MeOH/CHCl$_3$ = 3:2) calculated for $C_{51}H_{68}O_{12}Cl_2NaS^+$ [M+Na]$^+$: 997.37007, found: 997.36876. **R*f*** (pentane/acetone = 7:3) = 0.3. **Specific Rotation** $[\alpha]_D^{27°C}$ = – 128.9 (c=0.24, MeOH). **FT-IR** (acetone-*d$_6$*): ν (cm^{-1}) 3479w, 2964w, 2933w, 2872w, 1697s, 1644m, 1590w, 1489w, 1455w, 1402m, 1378m, 1312m, 1243s, 1197s, 1143m, 1113s, 1089s, 1065s, 1021s, 985m, 902w, 872w, 859w, 831w, 800w, 761w, 737w, 556w.

11-Desnoviosyl-15-p-tert-butylbenzenesulfide fidaxomicin (5c-C(15))

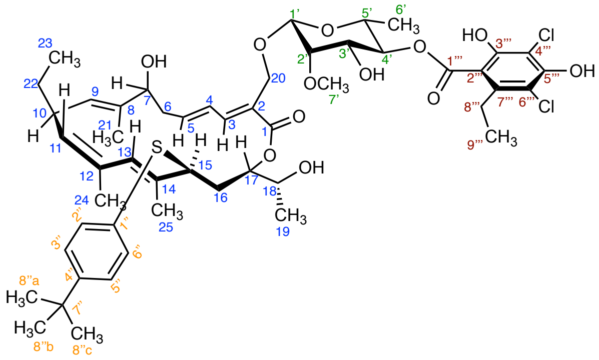

^{1}H NMR(500 MHz, acetone-*d$_6$*): δ (ppm) 7.37 – 7.30 (m, 2H, H3”, H5”), 7.27 (d, *J*=8.5 Hz, 2H, H2”, H6”), 7.05 (d, *J*=11.4 Hz, 1H, H3), 6.30 (ddd, *J*=14.9, 11.4, 1.4 Hz, 1H, H4), 6.14 (ddd, *J*=15.2, 11.0, 4.2 Hz, 1H, H5), 5.15 – 5.07 (m, 4H, H9, H13, H17, H4’), 4.66 (d, *J*=9.4 Hz, 1H, H11), 4.63 (s, 1H, H1’), 4.59 (d, *J*=11.0 Hz, 1H, H20a), 4.42 (d, *J*=11.0 Hz, 1H, H20b), 4.26 (t, *J*=3.4 Hz, 1H, H7), 3.85 – 3.79 (m, 2H, H18, H3’), 3.68 (dd, *J*=10.9, 1.6 Hz, 1H, H15), 3.58 (d, *J*=3.5 Hz, 1H, H2’) 3.56 – 3.53 (m, 1H, H5’), 3.51 (s, 3H, H7’), 3.02 – 2.95 (m, 3H, H10, H8’’’), 2.55 (dt, *J*=13.9, 3.6 Hz, 1H, H6a), 2.41 – 2.32 (m, 1H, H6b), 2.34 – 2.25 (m, 1H, H16a), 1.98 (ddd, *J*=14.7, 2.9, 1.6 Hz, 1H, H16b), 1.88 (s, 3H, H25), 1.64 (d, *J*=1.3 Hz, 3H, H24), 1.54 (d, *J*=1.4 Hz, 3H, H21), 1.32 (d, *J*=6.2 Hz, 3H, H6’), 1.29 (s, 9H, H8”a-c), 1.26 – 1.13 (m, 2H, H22), 1.19 (t, *J*=7.2 Hz, 3H, H9’’’), 1.15 (d, *J*=6.4 Hz, 3H, H19), 0.76 (t, *J*=7.4 Hz, 3H, H23). **^{13}C NMR** (126 MHz, acetone-d$_6$) δ (ppm) 169.4 (C1’’’), 167.6 (C1), 155.8 (C3’’’), 154.0 (C5’’’), 151.5 (C4”), 144.8 (C3), 143.2 (C5), 142.5 (C7’’’), 137.0 (C11), 134.9 (C2”, C6”), 133.9 (C13), 133.8 (C8), 132.1 (C1”), 131.9 (C12), 131.8 (C14), 127.0 (C4), 126.9 (C9), 126.3 (C3”, C5”), 125.2 (C2), 114.6 (C6’’’), 110.6 (C2’’’), 108.3 (C4’’’), 100.0 (C1’), 81.6 (C2’), 78.0 (C17), 77.4 (C4’), 73.7 (C7), 72.3 (C3’), 70.8 (C5’), 69.7 (C18), 61.9 (C20), 61.8 (C7’), 59.8 (C15), 39.4 (C10), 38.4 (C6), 35.1 (C7”), 31.6 (C8”a-c), 31.3 (C16), 29.7 (C22), 26.2 (C8’’’), 19.1 (C19), 18.0 (C6’), 17.1 (C24), 15.4 (C21), 14.3 (C9’’’), 14.1 (C25), 12.2 (C23). **HRMS** ESI(+) (MeOH/CHCl$_3$ = 3:2) calculated for $C_{51}H_{68}O_{12}Cl_2NaS^+$ [M+Na]$^+$: 997.37007, found: 997.37034. **R*f*** (pentane/acetone = 7:3) = 0.29. **Specific Rotation** $[\alpha]_D^{27°C}$ = +70.1 (c=0.39, MeOH). **FT-IR** (acetone-*d$_6$*): ν (cm^{-1}) 3417w, 2963w, 2934w, 2872w, 1698m, 1655w, 1639w, 1590w, 1489w, 1456w, 1403w, 1376w, 1311m, 1292w, 1236s, 1198s, 11790m, 1143w, 1110m, 1090s, 1069s, 1022s, 916w, 899w, 856w, 828w, 800w, 762w, 736w, 724w, 693w, 677w, 558w, 530w, 478w.

Thioglycoside derivatives of Fdx

11-Desnoviosyl-xy-thio-β-D-glucosyl fidaxomicin (18a)

11-Desnoviosyl-xy-thio-β-D-glucosyl fidaxomicin (**18a**) was synthesized from Fdx (**1**, 189 µmol, 200 mg, 1.0 equiv.) and 1-thio-β-D-glucose-tetra-*O*-acetate (**6a**, 189 µmol, 68.9 mg, 1.0 equiv.) in a solution of $Cu[ClO_4]_2$•6 H_2O (1 mM in MeCN, 1892 µL, 1 mol%) and MeCN (108 µL, c_{final}: 0.1 M) following General procedure B. The crude material derived from the organic phase was dissolved in MeOH (1985 µL, 0.1 M). Potassium carbonate (756 µmol, 104 mg, 4.0 equiv.) was added to the solution and the mixture stirred at 25 °C. After 30 min, the reaction was diluted with MeOH (10 mL), and the mixture filtered over a Supelco® Discovery® DSC-18 SPE tube (2 g) to provide after concentration *in vacuo* the crude material as a pale brown solid. Further purification was performed by preparative RP-HPLC using an isocratic eluent of 60% of MeOH+0.1% HCOOH in water+0.1% HCOOH (LC time program (time - %B): 0 min - 60%, 135 min - 60%. C13 isomer containing fractions were combined separately and repurified using the same conditions. C11 isomer containing fractions were combined separately and repurified by preparative RP-HPLC using an isocratic eluent of 35% of MeCN+0.1% HCOOH in water+0.1% HCOOH with otherwise unchanged conditions. Product containing fractions were combined separately and concentrated *in vacuo* at 44 °C (water bath):

11-Desnoviosyl-11-thio-β-D-glucosyl fidaxomicin (**18a-C(11)**) was obtained as a colorless solid in a yield of 2 % (3.8 µmol, 3.8 mg, t_R = 32.0 min (35% MeCN+0.1% HCOOH in water+0.1% HCOOH)).

11-Desnoviosyl-13-thio-β-D-glucosyl fidaxomicin (**18a-C(13)**) was obtained as a colorless solid in a yield of 6 % (10.7 µmol , 10.8 mg, t_R = 28.9 min).

11-Desnoviosyl-15-thio-β-D-glucoyl fidaxomicin (**18a-C(15)**) was obtained as a colorless solid in a yield of 24 % (45.0 µmol , 45.3 mg, t_R = 35.0 min).

11-Desnoviosyl-11-thio-β-D-glucosyl fidaxomicin (18a-C(11))

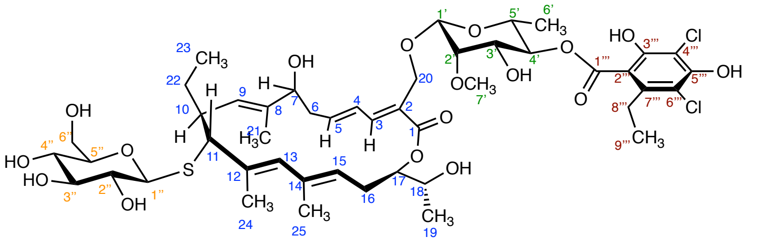

^{1}H NMR(500 MHz, acetone-d_6): δ (ppm) 7.29 (d, *J*=11.4 Hz, 1H, H3), 6.68 – 6.60 (m, 1H, H4), 6.00 (ddd, *J*=14.7, 9.7, 4.5 Hz, 1H, H5), 5.80 (s, 1H, H13), 5.74 – 5.67 (m, 1H, H15), 5.35 (dt, *J*=10.1, 1.6 Hz, 1H, H9), 5.10 (t, *J*=9.7 Hz, 1H, H4’), 4.73 – 4.69 (m, 1H, H17), 4.67 (d, *J*=0.8 Hz, 1H, H1’), 4.60 (d, *J*=11.6 Hz, 1H, H20a), 4.43 (d, *J*=11.5 Hz, 1H, H20b), 4.29 – 4.26 (m, 1H, H7), 4.26 (d, *J*=9.9 Hz, 1H, H1’’), 4.04 – 3.97 (m, 1H, H18), 3.84 (dd, *J*=11.9, 3.1 Hz, 1H, H6a’’), 3.80 (dd, *J*=9.9, 3.4 Hz, 1H, H3’), 3.67 – 3.56 (m, 3H, H2’, H5’, H6b’’), 3.55 – 3.52 (m, 1H, H11), 3.52 (s, 3H, H7’), 3.37 – 3.27 (m, 2H, H3’’, H4’’), 3.25 – 3.17 (m, 2H, H2’’, H5’’), 3.01 (qd, *J*=7.4, 1.8 Hz, 2H, H8’’’), 2.75 – 2.64 (m, 2H, H6a, H16a), 2.60 – 2.41 (m, 3H, H10, H6b, H16b), 2.00 – 1.93 (m, 1H, H22a), 1.91 (d, *J*=1.3 Hz, 3H, H24), 1.66 (d, *J*=1.4 Hz, 3H, H25), 1.65 (d, *J*=1.3 Hz, 3H, H21), 1.37 – 1.27 (m, 1H, H22b), 1.31 (d, *J*=6.2 Hz, 3H, H6’), 1.22 (t, *J*=7.4 Hz, 3H, H9’’’), 1.15 (d, *J*=6.2 Hz, 3H, H19), 0.83 (t, *J*=7.4 Hz, 3H, H23). **^{13}C NMR** (126 MHz, acetone-d_6) δ (ppm) 169.6 (C1’’’), 167.6 (C1), 156.1 (C3’’’), 154.3 (C5’’’), 145.8 (C3), 143.8 (C5), 142.7 (C7’’’), 136.6 (C8), 136.0 (C12), 136.0 (C14), 133.2 (C13), 128.0 (C4), 125.9 (C9), 125.5 (C15), 125.1 (C2), 114.8 (C6’’’), 110.2 (C2’’’), 108.3 (C4’’’), 101.6 (C1’), 87.7 (C1’’), 81.7 (C2’), 81.1 (C5’’), 79.6 (C3’’), 78.2 (C17), 77.6 (C4’), 74.4 (C2’’), 72.9 (C7), 72.4 (C3’), 72.0 (C4’’), 70.6 (C5’), 67.1 (C18), 63.7 (C6’’), 63.2 (C20), 62.5 (C11), 61.7 (C7’), 41.6 (C10), 37.1 (C6), 28.4 (C16), 27.6 (C22), 26.3 (C8’’’), 20.8 (C19), 18.2 (C6’), 17.5 (C25), 15.0 (C21), 14.4 (C9’’’), 14.0 (C24), 10.8 (C23). **HRMS** ESI(+)(MeOH) calculated for $C_{47}H_{66}O_{17}Cl_2NaS^+$

$[M+Na]^+$: 1027.32900, found: 1027.32984. **Specific Rotation** $[\alpha]_D^{24°C}$ = +55.2 (c = 0.57, MeOH). **FT-IR** (solid): ν (cm^{-1}) 3379m, 2972w, 2932w, 2876w, 1688m, 1683m, 1651w, 1645m, 1589w, 1435w, 1417w, 1378m, 1314m, 1242m, 1213m, 1199m, 1109m, 1088m, 1063s, 1020s, 900w, 873w, 762w, 737w, 695w, 575w, 527w.

11-Desnoviosyl-13-thio-β-D-glucosyl fidaxomicin (18a-C(13))

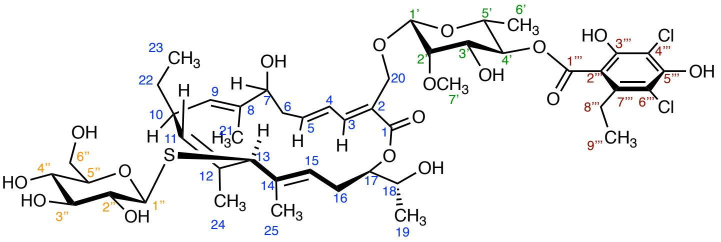

^{1}H NMR(500 MHz, acetone- d_6): δ (ppm) 7.20 (d, *J*=11.3 Hz, 1H, H3), 6.60 (dd, *J*=15.0, 11.4 Hz, 1H, H4), 6.06 (ddd, *J*=15.1, 9.6, 5.5 Hz, 1H, H5), 5.60 (ddt, *J*=7.8, 6.2, 3.1 Hz, 1H, H15), 5.43 (dd, *J*=7.9, 1.4 Hz, 1H, H11), 5.32 (dt, *J*=9.8, 1.6 Hz, 1H, H9), 5.10 (t, *J*=9.8 Hz, 1H, H4’), 4.83 (dt, *J*=9.1, 3.2 Hz, 1H, H17), 4.61 (d, *J*=11.5 Hz, 1H, H20a), 4.61 (s, 1H, H1’), 4.48 (d, *J*=11.6 Hz, 1H, H20b), 4.30 (s, 1H, H7), 4.27 (d, *J*=9.6 Hz, 1H, H1’’), 4.24 (s, 1H, H13), 3.92 – 3.87 (m, 1H, H18), 3.85 (dd, *J*=11.9, 2.7 Hz, 1H, H6’’a), 3.76 (dd, *J*=9.9, 3.4 Hz, 1H, H3’), 3.66 – 3.60 (m, 2H, , H6’’b, H5’), 3.56 (d, *J*=3.3 Hz, 1H, H2’), 3.51 (s, 3H, H7’), 3.40 – 3.28 (m, 3H, H2’’, H3’’, H4’’), 3.24 (ddd, *J*=9.4, 6.3, 2.8 Hz, 1H, H5’’), 3.21 – 3.16 (m, 1H, H10), 3.01 (q, *J*=7.4 Hz, 2H, H8’’’), 2.77 – 2.68 (m, 1H, H6a), 2.60 – 2.49 (m, 2H, H16a, H16b), 2.45 (ddd, *J*=14.0, 9.6, 3.4 Hz, 1H, H6b), 1.71 (s, 1H, H21), 1.58 (s, 3H, H24), 1.56 – 1.53 (m, 1H, H22a), 1.51 (s, 3H, H25), 1.39 (dt, *J*=13.3, 7.3 Hz, 1H, H22b), 1.31 (d, *J*=6.1 Hz, 3H, H6’), 1.22 (t, *J*=7.4 Hz, 3H, H9’’’), 1.16 (d, *J*=6.1 Hz, 3H, H19), 0.94 (t, *J*=7.4 Hz, 3H, H23). **^{13}C NMR** (126 MHz, acetone- d_6) δ (ppm) 169.7 (C1’’’), 166.6 (C1), 156.3 (C3’’’), 154.6 (C5’’’), 145.0 (C3), 143.5 (C5), 142.8 (C7’’’), 137.8 (C14), 135.2 (C11), 134.3 (C8), 133.5 (C12), 127.9 (C4), 127.0 (C9), 125.2 (C2), 121.1 (C15), 114.9 (C6’’’), 109.7 (C2’’’), 108.2 (C4’’’), 100.8 (C1’), 84.0 (C1’’), 81.7 (C2’), 81.5 (C5’’), 80.1 (C3’’), 77.9 (C17), 77.5 (C4’), 74.1 (C2’’), 72.8 (C7), 72.3 (C3’), 71.7 (C4’’), 70.7 (C5’), 66.6 (C18), 63.1 (C6’’), 62.6 (C20), 61.7 (C7’), 59.1 (C13), 39.1 (C10), 37.6 (C6), 30.3 (C22), 29.1 (C16), 26.4 (C8’’’), 21.2 (C19), 18.4 (C6’), 17.2 (C25), 14.7 (C21), 14.4 (C9’’’), 12.4 (C23), 11.7 (C24). **HRMS** ESI(+)(MeOH) calculated for $C_{47}H_{66}O_{17}Cl_2NaS^+$ $[M+Na]^+$: 1027.32900, found: 1027.32973. **Specific Rotation** $[\alpha]_D^{22°C}$ = –127.6 (c = 0.49, MeOH). **FT-IR** (acetone-d_6): ν (cm^{-1}) 3387m, 2965w, 2925w, 2875w, 1694s, 1644m, 1590w, 1453w, 1402w, 1372m, 1313m, 1246s, 1208m, 1110m, 1089s, 1066s, 1023s, 902w, 873w, 825w, 799w, 762w, 737w, 694w, 619w, 581w.

11-Desnoviosyl-15-thio-β-D-glucosyl fidaxomicin (18a-C(15))

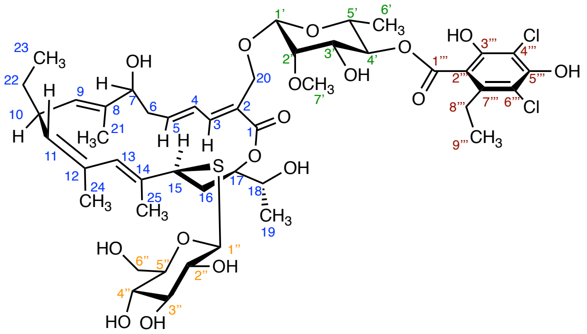

^{1}H NMR (500 MHz, acetone-d_6): δ (ppm) 7.11 (d, *J*=11.3 Hz, 1H, H3), 6.32 (ddd, *J*=15.1, 11.3, 1.5 Hz, 1H, H4), 6.20 (ddd, *J*=15.0, 11.1, 4.0 Hz, 1H, H5), 5.68 (s, 1H, H13), 5.23 – 5.06 (m, 4H, H9, H11, H17, H4’), 4.66 (s, 1H, H1’), 4.63 (d, *J*=11.0 Hz, 1H, H20a), 4.47 (d, *J*=10.9 Hz, 1H, H20b), 4.28 (t, *J*=3.3 Hz, 1H, H7), 4.14 – 4.08 (m, 1H, H1’’), 3.88 – 3.77 (m, 4H, H15, H18, H3’, H6’’a), 3.67 (dd, *J*=11.8, 5.7 Hz, 1H, H6’’b), 3.61 – 3.53 (m, 2H, H2’, H5’), 3.51 (s, 3H, H7’), 3.39 – 3.31 (m, 1H, H4’’), 3.31 – 3.22 (m, 2H, H2’’, H3’’), 3.15 – 3.03 (m, 2H, H10, H5’’), 2.98 (qd, *J*=7.4, 2.2 Hz, 2H, H8’’’), 2.58 (dtd, *J*=13.7, 4.2, 1.5 Hz, 1H, H6a), 2.38 (ddd, *J*=13.7, 11.0, 2.7 Hz, 1H, H6b), 2.25 (dt, *J*=14.6, 11.4 Hz, 1H, H16a), 1.88 (s, 3H, H25), 1.83 – 1.77 (m, 1H, H16b), 1.78 (d, *J*=1.2 Hz, 3H, H24), 1.58 (d, *J*=1.4 Hz, 3H, H21), 1.32 (d, *J*=6.2 Hz, 3H, H6’), 1.30 – 1.22 (m, 2H, H22a, H22b), 1.19 (t, *J*=7.4 Hz, 3H, H9’’’),

1.16 (d, *J*=6.5 Hz, 3H, H19), 0.79 (t, *J*=7.3 Hz, 3H, H23). **^{13}C NMR** (126 MHz, acetone-d_6) δ (ppm) 169.4 (C1’’’), 167.8 (C1), 155.7 (C3’’’), 154.1 (C5’’’), 144.9 (C3), 143.3 (C5), 142.5 (C7’’’), 138.0 (C11), 134.0 (C13), 133.8 (C8), 132.3 (C12), 131.1 (C14), 127.0 (C4), 126.9 (C9), 125.2 (C2), 114.7 (C6’’’), 110.3 (C2’’’), 108.1 (C4’’’), 100.0 (C1’), 84.4 (C1’’), 81.6 (C2’), 81.3 (C5’’), 79.9 (C3’’), 78.1 (C17), 77.4 (C4’), 73.7 (C7), 73.5 (C2’’), 72.2 (C3’), 71.4 (C4’’), 70.8 (C5’), 69.6 (C18), 62.9 (C6’’), 61.9 (C20), 61.8 (C7’), 52.6 (C15), 39.5 (C10), 38.3 (C6), 31.1 (C16), 29.7 (C22), 26.2 (C8’’’), 19.1 (C19), 17.9 (C6’), 17.1 (C24), 15.5 (C21), 14.3 (C9’’’), 13.3 (C25), 12.1 (C23). **HRMS** ESI(+)(MeOH) calculated for $C_{47}H_{66}O_{17}Cl_2NaS^+$ $[M+Na]^+$: 1027.32900, found: 1027.32940. **Specific Rotation** $[\alpha]_D^{24°C}$ = +34.6 (c = 0.65, MeOH). **FT-IR** (acetone-d_6): ν (cm^{-1}) 3387m, 2968w, 2931w, 2874w, 1694s, 1638m, 1589w, 1406m, 1372m, 1311m, 1239s, 1198m, 1179m, 1142m, 1090s, 1064s, 1021s, 917w, 900w, 872w, 857w, 762w, 736w, 676w, 617w, 580w, 531w, 478w.

11-Desnoviosyl-xy-thio-β-D-galactosyl fidaxomicin (18b)

11-Desnoviosyl-xy-thio-β-D-galactosyl fidaxomicin (**18b**) was synthesized from Fdx (**1**, 284 µmol, 300 mg, 1.0 equiv.) and 1-thio-β-D-galactose-tetra-*O*-acetate (**6b**, 284 µmol, 103 mg, 1.0 equiv.) in MeCN (1216 µL, 0.2 M) and a solution of $Cu[ClO_4]_2$•6 H_2O (10 mM in MeCN, 284 µL, 1 mol%) following General Procedure B. The crude material derived from the organic phase was dissolved in MeOH (2978 µL, 0.1 M). Potassium carbonate (1136 µmol, 157 mg, 4.0 equiv.) was added to the solution and the mixture stirred at 25 °C. After 30 min, the reaction was diluted with MeOH (10 mL), and the mixture filtered over a Supelco® Discovery® DSC-18 SPE tube (2 g) to provide after concentration *in vacuo* the crude material as a pale orange solid. Further purification was performed by preparative RP-HPLC using an isocratic eluent of 33% of MeCN+0.1% HCOOH in water+0.1% HCOOH (LC time program (time - %B): 0 min - 33%, 65 min - 33%. Product containing fractions were combined separately and repurified by preparative RP-HPLC using an isocratic eluent of 32% of MeCN+0.1% HCOOH in water+0.1% HCOOH. Product containing fractions were combined separately and concentrated *in vacuo* at 44 °C (water bath):

11-Desnoviosyl-11-thio-β-D-galactosyl fidaxomicin (**18b-C(11)**) was obtained as a colorless solid in a yield of 2% (4.3 µmol, 4.3 mg, t_R = 51.5 min (32%B)).

11-Desnoviosyl-13-thio-β-D-galactosyl Fidaxomicin (**18b-C(13)**) was obtained as a colorless solid in a yield of 4% (9.9 µmol, 10 mg, t_R = 41.2 min (32%B)).

11-Desnoviosyl-15-thio-β-D-galactosyl Fidaxomicin (**18b-C(15)**) was obtained as a colorless solid in a yield of 11% (30.6 µmol, 30.8 mg, t_R = 46.8 min (32%B)).

11-Desnoviosyl-11-thio-β-D-galactosyl fidaxomicin (18b-C(11))

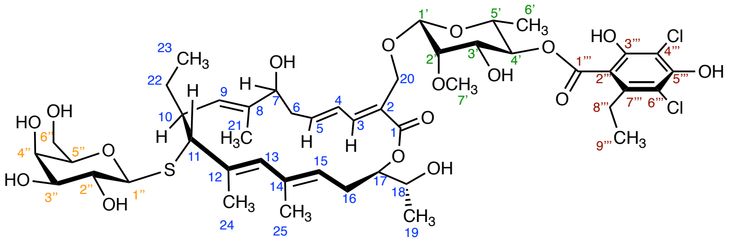

^{1}H NMR (500 MHz, acetone-d_6,): δ (ppm) 7.30 (d, *J*=11.4 Hz, 1H, H3), 6.69 – 6.60 (m, 1H, H4), 6.01 (ddd, *J*=14.5, 9.6, 4.4 Hz, 1H, H5), 5.79 (s, 1H, H13), 5.74 – 5.64 (m, 1H, H15), 5.36 (dt, *J*=10.1, 1.6 Hz, 1H, H9), 5.10 (t, *J*=9.7 Hz, 1H, H4’), 4.72 (dt, *J*=8.1, 4.2 Hz, 1H, H17), 4.67 (d, *J*=0.9 Hz, 1H, H1’), 4.60 (d, *J*=11.5 Hz, 1H, H20a), 4.43 (d, *J*=11.5 Hz, 1H, H20b), 4.28 (d, *J*=4.0 Hz, 1H, H7), 4.22 (d, *J*=9.8 Hz, 1H, H1’’), 4.03 – 3.95 (m, 1H, H18), 3.90 (dd, *J*=3.4, 1.3 Hz, 1H, H4’’), 3.83 – 3.69 (m, 3H, H3’, H6a’’, H6b’’), 3.65 – 3.59 (m, 2H, H2’, H5’), 3.57 (t, *J*=9.3 Hz, 1H, H2’’), 3.54 – 3.52 (m, 1H, H11), 3.52 (s, 3H, H7’), 3.48 – 3.41 (m, 2H, H3’’, H5’’), 3.00 (qd, *J*=7.4, 1.6 Hz, 2H, H8’’’), 2.75 – 2.63 (m, 2H, H16b, H6a), 2.60 – 2.42 (m, 3H, H10, H16a, H6b), 2.00 – 1.91 (m, 1H, H22b), 1.90 (d, *J*=1.2 Hz, 3H, H24), 1.64 (d, *J*=1.8 Hz, 6H, H21, H25), 1.38 – 1.26 (m, 1H, H22a,),

1.31 (d, *J*=6.2 Hz, 3H, H6’), 1.22 (t, *J*=7.4 Hz, 3H, H9’’’), 1.16 (d, *J*=6.2 Hz, 3H, H19), 0.83 (t, *J*=7.4 Hz, 3H, H23). **^{13}C NMR** (126 MHz, acetone-d_6) δ (ppm) 169.5 (C1’’’), 167.5 (C1), 156.0 (C3’’’), 154.2 (C5’’’), 145.9 (C3), 143.9 (C5), 142.7 (C7’’’), 136.5 (C8), 136.1 (C12), 136.0 (C14), 133.2 (C13), 128.0 (C4), 125.9 (C9), 125.2 (C15), 125.0 (C2), 114.7 (C6’’’), 110.3 (C2’’’), 108.2 (C4’’’), 101.6 (C1’), 88.1 (C1’’), 81.7 (C2’), 79.9 (C5’’), 78.1 (C17), 77.6 (C4’), 76.1 (C3’’), 72.9 (C7), 72.3 (C3’), 71.5 (C2’’), 70.6 (C5’), 69.8 (C4’’), 67.2 (C18), 63.2 (C20), 62.4 (C11, C6’’), 61.7 (C7’), 41.7 (C10), 37.1 (C6), 28.4 (C16), 27.5 (C22), 26.3 (C8’’’), 20.9 (C19), 18.2 (C6’), 17.5 (C25), 15.0 (C21), 14.4 (C9’’’), 14.0 (C24), 10.8 (C23). **HRMS** ESI(+)(MeOH) calculated for $C_{47}H_{66}O_{17}Cl_2NaS^+$ $[M+Na]^+$: 1027.32900, found: 1027.32912. **Specific Rotation** $[\alpha]_D^{25°C}$ = +58.3 (c = 0.37, MeOH). **FT-IR** (acetone-d_6): ν (cm^{-1}) 3405m, 2970w, 2932w, 2876w, 1695s, 1644w, 1589w, 1379w, 1361w, 1313m, 1247s, 1213m, 1199m, 1141w, 1114m, 1066s, 1023s, 902w, 865w, 807w, 762w, 736w, 696w, 581w, 529w, 498w.

11-Desnoviosyl-13-thio-β-D-galactosyl fidaxomicin (18b-C(13))

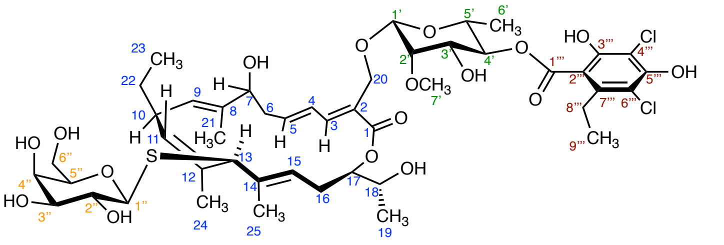

^{1}H NMR(acetone-d_6, 500 MHz): δ (ppm) 7.21 (d, *J*=11.4 Hz, 1H, H3), 6.60 (dd, *J*=15.0, 11.4 Hz, 1H, H4), 6.06 (ddd, *J*=15.1, 9.6, 5.5 Hz, 1H, H5), 5.64 – 5.57 (m, 1H, H15), 5.43 (dd, *J*=7.9, 1.5 Hz, 1H, H11), 5.32 (dt, *J*=9.9, 1.6 Hz, 1H, H9), 5.10 (t, *J*=9.7 Hz, 1H, H4’), 4.83 (dt, *J*=9.1, 3.2 Hz, 1H, H17), 4.61 (d, *J*=11.8 Hz, 1H, H20b), 4.61 (s, 1H, H1’), 4.49 (d, *J*=11.6 Hz, 1H, H20a), 4.30 (t, *J*=4.4 Hz, 1H, H7), 4.23 (d, *J*=9.8 Hz, 1H, H1’’), 4.22 (s, 1H, H13), 3.95 – 3.86 (m, 2H, H18, H4’’), 3.82 – 3.74 (m, 2H, H6a’’, H3’), 3.72 – 3.61 (m, 3H, H2’’, H6b’’, H5’), 3.57 (d, *J*=3.3 Hz, 1H, H2’), 3.52 (s, 3H, H7’), 3.50 – 3.42 (m, 2H, H3’’, H5’’), 3.18 (dq, *J*=9.7, 7.3 Hz, 1H, H10), 3.02 (q, *J*=7.4 Hz, 2H, H8’’’), 2.73 (dddd, *J*=14.4, 5.5, 3.7, 1.7 Hz, 1H, H6a), 2.60 – 2.41 (m, 3H, H6b, H16a, H16b), 1.71 (d, *J*=1.3 Hz, 3H, H21), 1.58 (d, *J*=1.2 Hz, 3H, H24), 1.57 – 1.48 (m, 1H, H22b), 1.51 (s, 3H, H25), 1.43 – 1.33 (m, 1H, H22a), 1.31 (d, *J*=6.1 Hz, 3H, H6’), 1.23 (t, *J*=7.4 Hz, 3H, H9’’’), 1.16 (d, *J*=6.0 Hz, 3H, H19), 0.93 (t, *J*=7.4 Hz, 3H, H23). **^{13}C NMR** (126 MHz, acetone-d_6) δ (ppm) 169.8 (C1’’’), 166.6 (C1), 156.4 (C3’’’), 154.6 (C5’’’), 145.0 (C3), 143.5 (C5), 142.8 (C7’’’), 137.8 (C12 or C14), 135.1 (C11), 134.2 (C8), 133.5 (C12 or C14), 127.9 (C4), 127.1 (C9), 125.2 (C2), 121.1 (C15), 114.9 (C6’’’), 109.7 (C2’’’), 108.2 (C4’’’), 100.8 (C1’), 84.4 (C1’’), 81.7 (C2’), 80.1 (C5’’), 77.9 (C17), 77.6 (C4’), 76.5 (C3’’), 72.8 (C7), 72.4 (C3’), 71.3 (C2’’), 70.7 (C5’), 70.3 (C4’’), 66.6 (C18), 62.7 (C6’’), 62.6 (C20), 61.7 (C7’), 59.1 (C13), 39.1 (C10), 37.6 (C6), 30.6 (C22), 29.1 (C16), 26.4 (C8’’’), 21.3 (C19), 18.4 (C6’), 17.2 (C25), 14.7 (C21), 14.4 (C9’’’), 12.4 (C23), 11.7 (C24). **HRMS** ESI(+)(MeOH) calculated for $C_{47}H_{66}O_{17}Cl_2NaS^+$ $[M+Na]^+$: 1027.32900, found: 1027.32951. **Specific Rotation** $[\alpha]_D^{25°C}$ = –136.6 (c = 0.47, MeOH). **FT-IR** (acetone-d_6): ν (cm^{-1}) 3401w, 2965w, 2931w, 2875w, 1695s, 1644w, 1589w, 1454w, 1405w, 1375m, 1313m, 1245s, 1208m, 1141m, 1113m, 1087s, 1064s, 1023s, 902w, 868w, 800w, 762w, 738w, 695w, 581w.

11-Desnoviosyl-15-thio-β-D-galactosyl fidaxomicin (18b-C(15))

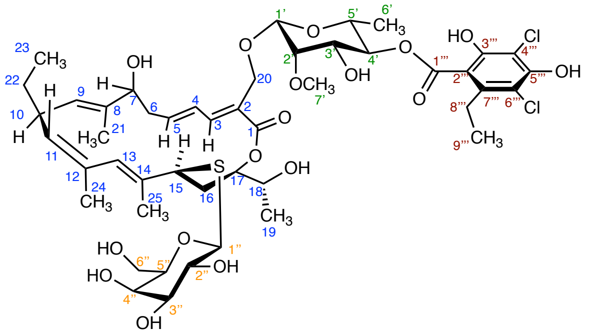

^{1}H NMR (500 MHz, acetone-d_6) δ (ppm) 7.11 (d, J = 11.4 Hz, 1H, H3), 6.33 (ddd, J = 15.1, 11.3, 1.5 Hz, 1H, H4), 6.20 (ddd, J = 15.1, 11.0, 4.0 Hz, 1H, H5), 5.67 (s, 1H, H13), 5.18 (dt, J = 10.5, 1.4 Hz, 1H, H9), 5.17 – 5.08 (m, 3H, H11, H17, H4'), 4.66 (s, 1H, H1'), 4.64 (d, J = 10.9 Hz, 1H, H20a), 4.47 (d, J = 10.9 Hz, 1H, H20b), 4.29 (d, J = 3.5 Hz, 1H, H7), 4.08 (d, J = 9.8 Hz, 1H, H1''), 3.90 (dd, J = 3.5, 1.1 Hz, 1H, H4''), 3.86 – 3.78 (m, 4H, H18, H15, H6a'', H3'), 3.75 (dd, J = 11.2, 5.4 Hz, 1H, H6b''), 3.63 – 3.54 (m, 3H, H2', H5', H2''), 3.52 (s, 3H, H7'), 3.39 – 3.33 (m, 2H, H3'', H5''), 3.13-3.03 (m, 1H, H10), 2.99 (qd, J = 7.3, 1.6 Hz, 2H, H8'''), 2.58 (dtd, J = 13.7, 4.2, 1.5 Hz, 1H, H6a), 2.39 (ddd, J = 13.6, 11.0, 2.6 Hz, 1H, H6b), 2.25 (dt, J = 14.6, 11.4 Hz, 1H, H16a), 1.88 (d, J = 1.1 Hz, 3H, H25), 1.85 – 1.79 (m, 1H, H16b), 1.78 (d, J = 1.2 Hz, 3H, H24), 1.59 (d, J = 1.5 Hz, 3H, H21), 1.33 (d, J = 6.2 Hz, 3H, H6'), 1.32 – 1.22 (m, 2H, H22a, H22b), 1.20 (t, J = 7.4 Hz, 3H, H9'''), 1.16 (d, J = 6.4 Hz, 3H, H19), 0.78 (t, J = 7.4 Hz, 3H, H23). **^{13}C NMR** (126 MHz, acetone-d_6) δ (ppm) 169.3 (C1'''), 167.7 (C1), 155.7 (C3'''), 153.9 (C5'''), 144.8 (C3), 143.2 (C5), 142.6 (C7'''), 137.8 (C11), 133.8 (C8), 133.8 (C13), 132.3 (C12), 131.3 (C14), 127.0 (C9), 127.0 (C4), 125.3 (C2), 114.6 (C6'''), 110.4 (C2'''), 108.1 (C4'''), 100.0 (C1'), 84.9 (C1''), 81.6 (C2'), 79.9 (C5''), 78.1 (C17), 77.4 (C4'), 76.4 (C3''), 73.7 (C7), 72.2 (C3'), 70.8 (C5'), 70.8 (C2''), 70.0 (C4''), 69.6 (C18), 62.4 (C6''), 61.9 (C20), 61.8 (C7'), 52.6 (C15), 39.5 (C10), 38.4 (C6), 31.1 (C16), 29.6 (C22), 26.2 (C8'''), 19.1 (C19), 17.9 (C6'), 17.1 (C24), 15.5 (C21), 14.3 (C9'''), 13.5 (C25), 12.0 (C23). **HRMS** ESI(+)(MeOH) calculated for $C_{47}H_{66}O_{17}Cl_2NaS^+$ $[M+Na]^+$: 1027.32900, found: 1027.32954. **Specific Rotation** $[\alpha]_D^{25°C}$ = +54.6 (c = 0.71, MeOH). **FT-IR** (acetone-d_6): ν (cm^{-1}) 3407w, 2963w, 2932w, 2873w, 1695s, 1638w, 1589w, 1405w, 1374m, 1311m, 1241s, 1198m, 1180m, 1142m, 1109m, 1088s, 1062s, 1022s, 916w, 867w, 762w, 737w, 694w, 531w, 480w.

11-Desnoviosyl-xy-thio-α-L-fucosyl fidaxomicin (18c)

11-Desnoviosyl-xy-thio-α-L-fucosyl fidaxomicin (**18c**) was synthesized from Fdx (**1**, 284 µmol, 300 mg, 1.0 equiv.) and 2,3,4-tri-*O*-acetyl-1-thio-α-L-fucose (**6c**, 284 µmol, 87 mg, 1.0 equiv.) in MeCN (1216 µL, 0.2 M) and a solution of $Cu[ClO_4]_2$•6 H_2O (10 mM in MeCN, 284 µL, 1 mol%) following General procedure B. The crude material derived from the organic phase was dissolved in MeOH (2978 µL, 0.1 M). Potassium carbonate (1136 µmol, 157 mg, 4.0 equiv.) was added to the solution and the mixture stirred at 25 °C. After 30 min, the reaction was diluted with MeOH (10 mL), and the mixture filtered over a Supelco® Discovery® DSC-18 SPE tube (2 g) to provide after concentration *in vacuo* the crude material as a pale orange solid. Further purification was performed by preparative RP-HPLC using a linear gradient of 30-43% B over 60 min (LC time program (time - %B): 0 min - 30%, 15 min - 30%; 75 min - 43%). Product containing fractions (C11 isomer) were combined separately and repurified by preparative RP-HPLC using the same eluent system but a linear gradient of 30-40% over 60 min (LC time program (time - %B): 0 min - 30%, 5 min – 30%, 365 min - 40%, 95 min - 40%). Product containing fractions (C13 and C15 isomer) were combined separately and repurified by preparative RP-HPLC using a linear gradient of 50-70% over 100 min (solvent A: H_2O + 0.1% HCOOH, solvent B: MeOH + 0.1% HCOOH; 20 $^{mL}/_{min}$; LC time program (time - %B): 0 min - 50%, 5 min – 50%, 105 min -70%). Product containing fractions were combined separately and concentrated *in vacuo* at 44 °C (water bath):

11-Desnoviosyl-11-thio-α-L-fucosyl fidaxomicin (**18c-C(11)**) was obtained as a colorless solid in a yield of 5% (15.4 µmol, 15.2 mg, t_R = 65.8 min (30-40%MeCN in 60 min)).

11-Desnoviosyl-13-thio-α-L-fucosyl fidaxomicin (**18c-C(13)**) was obtained as a colorless solid in a yield of 9% (26.3 µmol, 26 mg, t_R = 72.0 min (50-70%MeOH in 100 min)).
11-Desnoviosyl-15-thio-α-L-fucosyl fidaxomicin (**18c-C(15)**) was obtained as a colorless solid in a yield of 15% (41.4 µmol, 41 mg, t_R = 75.0 min (50-70%MeOH in 100 min)).

11-Desnoviosyl-11-thio-α-L-fucosyl fidaxomicin (18c-C(11))

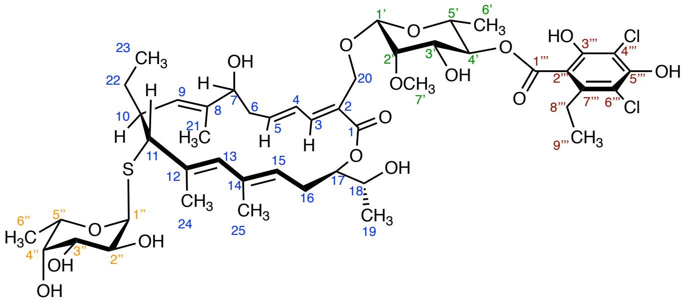

^{1}H NMR (500 MHz, acetone-d$_6$) δ (ppm) 7.29 (d, *J* = 11.5 Hz, 1H, H3), 6.69 – 6.59 (m, 1H, H4), 6.01 (ddd, *J* = 14.5, 9.3, 4.7 Hz, 1H, H5), 5.78 (s, 1H, H13), 5.63 – 5.57 (m, 1H, H15), 5.35 (dt, *J* = 10.2, 1.6 Hz, 1H, H9), 5.22 (d, *J* = 5.4 Hz, 1H, H1”), 5.10 (t, *J* = 9.7 Hz, 1H, H4’), 4.75 (dt, *J* = 7.2, 4.4 Hz, 1H, H17), 4.68 (d, *J* = 0.9 Hz, 1H, H1’), 4.60 (d, *J* = 11.5 Hz, 1H, H20a), 4.43 (d, *J* = 11.5 Hz, 1H, H20b), 4.28 (s, 1H, H7), 4.13 (qd, *J* = 6.5, 1.4 Hz, 1H, H5”), 4.07 – 3.97 (m, 1H, H18), 3.98 (dd, *J* = 9.9, 5.4 Hz, 1H, H2”), 3.81 (dd, *J* = 10.0, 3.5 Hz, 1H, H3’), 3.68 – 3.64 (m, 1H, H4”), 3.66 – 3.59 (m, 1H, H5’), 3.62 – 3.58 (m, 1H, H2’), 3.55 (dd, *J* = 10.0, 3.4 Hz, 1H, H3”), 3.52 (s, 3H, H7’), 3.37 (d, *J* = 10.9 Hz, 1H, H11), 3.00 (qd, *J* = 7.4, 0.8 Hz, 2H, H8’”), 2.76 – 2.64 (m, 2H, H6b, H16b), 2.57 (ddd, *J* = 11.1, 8.6, 2.9 Hz, 1H, H10), 2.55 – 2.42 (m, 2H, H6a, H16a), 2.10 – 2.04 (m, 1H, H22b), 1.83 (d, *J* = 1.4 Hz, 3H, H24), 1.66 (d, *J* = 1.4 Hz, 3H, H25), 1.65 (t, *J* = 0.9 Hz, 3H, H21), 1.38 – 1.27 (m, 1H, H22a), 1.31 (d, *J* = 6.1 Hz, 3H, H6’), 1.23 (t, *J* = 7.4 Hz, 3H, H9’”), 1.20 (d, *J* = 6.5 Hz, 3H, H6”), 1.17 (d, *J* = 6.1 Hz, 3H, H19), 0.84 (t, *J* = 7.4 Hz, 3H, H23). **^{13}C NMR** (126 MHz, acetone-d$_6$) δ (ppm) 169.5 (C1’”), 167.6 (C1), 155.9 (C3’”), 153.9 (C5’”), 145.7 (C3), 143.7 (C5), 142.7 (C7’”), 136.6 (C8), 136.1 (C12), 136.0 (C14), 132.3 (C13), 128.0 (C4), 126.0 (C9), 125.3 (C15), 125.1 (C2), 114.6 (C6’”), 110.6 (C2’”), 108.2 (C4’”), 101.7 (C1’), 88.7 (C1”), 81.7 (C2’), 78.0 (C17), 77.6 (C4’), 72.9 (C7), 72.8 (C3”), 72.7 (C4”), 72.3 (C3’), 70.6 (C5’), 69.8 (C2”), 68.5 (C5”), 67.5 (C18), 63.3 (C20), 62.1 (C11), 61.7 (C7’), 42.6 (C10), 37.1 (C6), 28.5 (C16), 27.6 (C22), 26.2 (C8’”), 20.8 (C19), 18.2 (C6’), 17.5 (C25), 16.9 (C6”), 15.1 (C21), 14.4 (C9’”), 14.0 (C24), 11.0 (C23). **HRMS** ESI(+)(MeOH) calculated for $C_{47}H_{66}O_{16}Cl_2NaS^+$ $[M+Na]^+$: 1011.33408, found: 1011.33461. **Specific Rotation** $[\alpha]_D^{23°C}$ = + 5.14 (c = 0.18). **FT-IR** (acetone-*d$_6$*): ν (cm^{-1}) 3400m, 2977w, 2931w, 2875w, 1691m, 1643w, 1589w, 1453w, 1403w, 1379w, 1313w, 1245s, 1214m, 1164w, 1144w, 1090s, 1063s, 1023m, 988w, 933w, 902w, 764w, 697w, 629w, 585w, 575w, 548w, 533w, 521w, 505w, 480w, 467w.

11-Desnoviosyl-13-thio-α-L-fucosyl fidaxomicin (18c-C(13))

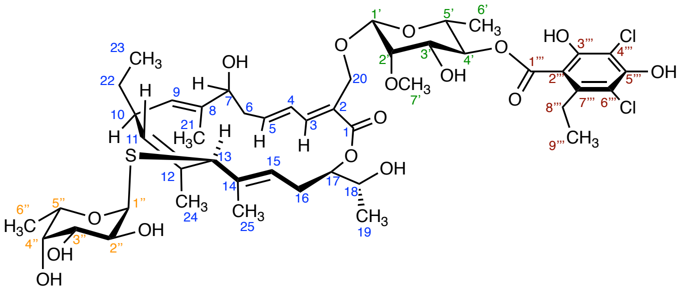

^{1}H NMR (acetone-*d$_6$*, 500 MHz): δ (ppm) 7.21 (d, *J*=11.6 Hz, 1H, H3), 6.60 (dd, *J*=15.0, 11.4 Hz, 1H, H4), 6.06 (ddd, *J*=15.1, 9.7, 5.5 Hz, 1H, H5), 5.77 – 5.69 (m, 1H, H15), 5.36 – 5.29 (m, 2H, H9, H11), 5.17 (d, *J*=5.6 Hz, 1H, H1”), 5.10 (t, *J*=9.7 Hz, 1H, H4’), 4.83 (dt, *J*=9.1, 3.3 Hz, 1H, H17), 4.61 (d, *J*=11.2 Hz, 1H, H20a), 4.60 (s, 1H, H1’), 4.49 (d, *J*=11.5 Hz, 1H, H20b), 4.29 (t, *J*=3.4 Hz, 1H, H7), 4.22 (qd, *J*=6.7, 1.2 Hz, 1H, H5”), 4.07 (dd, *J*=9.8, 5.6 Hz, 1H, H2”), 3.91 (dq, *J*=9.1, 6.1 Hz, 1H, H18), 3.83 (s, 1H, H13), 3.76 (dd, *J*=9.9, 3.4 Hz, 1H, H3’), 3.70 (dd, *J*=3.6, 1.4 Hz, 1H, H4”), 3.69 – 3.62 (m, 2H, H3”, H5’), 3.56 (d, *J*=3.3 Hz, 1H, H2’), 3.52 (s, 3H, H7’), 3.19 – 3.09 (m, 1H, H10), 3.02 (q, *J*=7.4

Hz, 2H, H8’’’), 2.72 (dddd, *J*=14.4, 5.5, 3.8, 1.7 Hz, 1H, H6a), 2.62 – 2.54 (m, 1H, H16a), 2.52 (ddd, *J*=14.2, 10.3, 3.6 Hz, 1H, H16b), 2.44 (ddd, *J*=13.6, 9.6, 3.3 Hz, 1H, H6b), 1.71 (d, *J*=1.0 Hz, 3H, H21), 1.59 (d, *J*=1.2 Hz, 3H, H24), 1.52 (s, 3H, H25), 1.47 – 1.33 (m, 2H, H22a, H22b), 1.31 (d, *J*=6.1 Hz, 3H, H6’), 1.23 (t, *J*=7.4 Hz, 3H, H9’’’), 1.21 (d, *J*=6.5 Hz, 3H, H6’’), 1.16 (d, *J*=6.1 Hz, 3H, H19), 0.91 (t, *J*=7.4 Hz, 3H, H23). **^{13}C NMR** (126 MHz, acetone-d$_6$) δ (ppm) 169.8 (C1’’’), 166.6 (C1), 156.4 (C3’’’), 154.4 (C5’’’), 145.0 (C3), 143.6 (C5), 142.9 (C7’’’), 137.9 (C14), 134.9 (C11), 134.0 (C8), 133.4 (C12), 127.9 (C4), 127.2 (C9), 125.1 (C2), 121.4 (C15), 114.8 (C6’’’), 109.8 (C2’’’), 108.2 (C4’’’), 100.7 (C1’), 84.8 (C1’’), 81.7 (C2’), 78.0 (C17), 77.6 (C4’), 72.9 (C7), 72.8 (C3’’), 72.7 (C4’’), 72.4 (C3’), 70.7 (C5’), 69.3 (C2’’), 67.4 (C5’’), 66.6 (C18), 62.6 (C20), 61.7 (C7’), 59.3 (C13), 39.3 (C10), 37.6 (C6), 30.6 (C22), 29.1 (C16), 26.4 (C8’’’), 21.3 (C19), 18.4 (C6’), 17.3 (C25), 16.8 (C6’’), 14.6 (C21), 14.4 (C9’’’), 12.3 (C23), 11.7 (C24). **HRMS** ESI(+)(MeOH) calculated for $C_{47}H_{66}O_{16}Cl_2NaS^+$ [M+Na]$^+$: 1011.33408, found: 1011.33334. **Specific Rotation** $[\alpha]_D^{23°C}$ = - 209.03 (c = 0.14). **FT-IR** (acetone-*d$_6$*): ν (cm^{-1}) 3414m, 2974w, 2932w, 2876w, 1695s, 1644w, 1590w, 1454w, 1404w, 1376m, 1313m, 1244s, 1209m, 1144w, 1090s, 1064s, 1023m, 988m, 933w, 903w, 873w, 833w, 799w, 763w, 736w, 697w, 633w, 581w.

11-Desnoviosyl-15-thio-α-L-fucosyl fidaxomicin (18c-C(15))

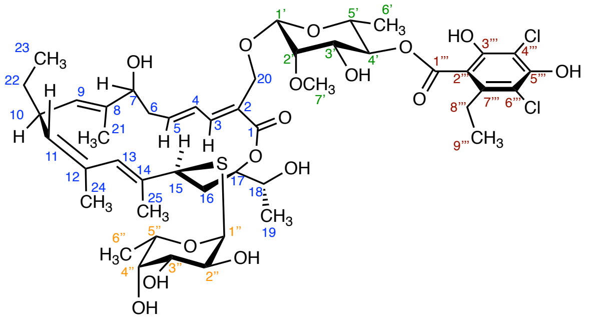

^{1}H NMR (acetone-*d$_6$*, 500 MHz): δ (ppm) 7.14 (d, *J*=11.3 Hz, 1H, H3), 6.34 (ddd, *J*=15.0, 11.4, 1.4 Hz, 1H, H4), 6.19 (ddd, *J*=15.1, 10.8, 4.4 Hz, 1H, H5), 5.63 (s, 1H, H13), 5.18 (dt, *J*=10.4, 1.4 Hz, 1H, H9), 5.16 – 5.08 (m, 1H, H17), 5.12 (t, *J*=9.7 Hz, 1H, H4’), 5.08 (d, *J*=5.6 Hz, 1H, H1’’), 5.05 (d, *J*=9.4 Hz, 1H, H11), 4.66 (s, 1H, H1’), 4.62 (d, *J*=11.0 Hz, 1H, H20a), 4.46 (d, *J*=10.9 Hz, 1H, H20b), 4.34 – 4.28 (m, 2H, H7, H5’’), 3.99 (dd, *J*=9.9, 5.6 Hz, 1H, H2’’), 3.84 (dd, *J*=10.0, 3.3 Hz, 1H, H3’), 3.83 – 3.79 (m, 1H, H18), 3.71 (dd, *J*=3.5, 1.4 Hz, 1H, H4’’), 3.61 – 3.53 (m, 3H, H3’’, H2’, H5’), 3.52 (s, 3H, H7’), 3.47 (dd, *J*=10.7, 1.9 Hz, 1H, H15), 3.15 – 3.06 (m, 1H, H10), 3.00 (qd, *J*=7.3, 1.5 Hz, 2H, H8’’’), 2.58 (dtd, *J*=13.6, 4.4, 1.5 Hz, 1H, H6a), 2.40 (ddd, *J*=13.6, 10.8, 2.7 Hz, 1H, H6b), 2.29 (dt, *J*=14.5, 11.1 Hz, 1H, H16a), 1.90 (d, *J*=1.1 Hz, 3H, H25), 1.90 (dt, *J*=14.7, 2.3 Hz, 1H, H16b), 1.79 (d, *J*=1.3 Hz, 3H, H24), 1.59 (d, *J*=1.4 Hz, 3H, H21), 1.33 (d, *J*=6.2 Hz, 3H, H6’), 1.38 – 1.18 (m, 2H, H22a, H22b), 1.27 (d, *J*=6.6 Hz, 3H, H6’’), 1.20 (t, *J*=7.4 Hz, 3H, H9’’’), 1.16 (d, *J*=6.4 Hz, 3H, H19), 0.79 (t, *J*=7.4 Hz, 3H, H23). **^{13}C NMR** (126 MHz, acetone-d$_6$) δ (ppm) 169.4 (C1’’’), 167.6 (C1), 155.9 (C3’’’), 154.1 (C5’’’), 144.8 (C3), 143.2 (C5), 142.6 (C7’’’), 137.6 (C11), 134.1 (C8), 133.2 (C13), 132.4 (C14), 132.2 (C12), 127.2 (C4), 127.1 (C9), 125.2 (C2), 114.6 (C6’’’), 110.4 (C2’’’), 108.2 (C4’’’), 100.1 (C1’), 85.0 (C1’’), 81.7 (C2’), 78.1 (C17), 77.5 (C4’), 73.9 (C7), 72.8 (C3’’), 72.7 (C4’’), 72.3 (C3’), 70.8 (C5’), 69.7 (C18), 69.2 (C2’’), 67.5 (C5’’), 62.0 (C20), 61.8 (C7’), 52.1 (C15), 39.6 (C10), 38.4 (C6), 32.2 (C16), 29.6 (C22), 26.2 (C8’’’), 19.2 (C19), 18.0 (C6’), 17.1 (C24), 16.9 (C6’’), 15.5 (C21), 14.4 (C9’’’), 13.9 (C25), 12.1 (C23). **HRMS** ESI(+)(MeOH) calculated for $C_{47}H_{66}O_{16}Cl_2NaS^+$ [M+Na]$^+$: 1011.33408, found: 1011.33464. **Specific Rotation** $[\alpha]_D^{23°C}$ = - 49.13 (c = 0.14). **FT-IR** (acetone-*d$_6$*): ν (cm^{-1}) 3408m, 2975w, 2932w, 2874w, 1695m, 1639m, 1589w, 1406w, 1377m, 1311m, 1239s, 1198m, 1162w, 1144m, 1089s, 1061s, 1022s, 988m, 933w, 916w, 872w, 858w, 828w, 763w, 737w, 697w, 631w, 480w.

11-Desnoviosyl-xy-thio-β-L-fucosyl fidaxomicin (18d)

11-Desnoviosyl-xy-thio-β-L-fucosyl fidaxomicin (**18d**) was synthesized from Fdx (**1**, 284 µmol, 300 mg, 1.0 equiv.) and 2,3,4-tri-*O*-acetyl-1-thio-β-L-fucose (**6d**, 284 µmol, 87 mg, 1.0 equiv.) in MeCN (1216 µL, 0.2 M) and a solution of $Cu[ClO_4]_2$•6 H_2O (10 mM in MeCN,

284 µL, 1 mol%) following General procedure B. The crude material derived from the organic phase was dissolved in MeOH (2978 µL, 0.1 M). Potassium carbonate (1136 µmol, 157 mg, 4.0 equiv.) was added to the solution and the mixture stirred at 25 °C. After 30 min, the reaction was diluted with MeOH (10 mL), and the mixture filtered over a Supelco® Discovery® DSC-18 SPE tube (2 g) to provide after concentration *in vacuo* the crude material as a pale orange solid. Further purification was performed by preparative RP-HPLC using an isocratic eluent of 36% B (LC time program (time - %B): 0 min - 36%, 65 min – 36%). Product containing fractions (C11 isomer) were combined separately and repurified by preparative RP-HPLC using an isocratic eluent of 34% B (LC time program (time - %B): 0 min - 34%, 65 min – 34%). Further purification of the C11 isomer proved unsuccessful. Product containing fractions (C13 and C15 isomer) were combined separately and repurified by preparative RP-HPLC using an isocratic eluent of 34% B (LC time program (time - %B): 0 min - 34%, 65 min – 34%). Product containing fractions were combined and concentrated *in vacuo* at 44 °C (water bath) to provide an in-separatable mixture of the C13 and C15 regioisomers:

11-Desnoviosyl-11-thio-β-L-fucosyl fidaxomicin (**18d-C(11)**) was obtained as a colorless solid with a purity of approx. 87% (determined based on the integrals of the H3 signal of the product and the most prominent impurity in the ^{1}H NMR spectrum) and in a yield of 2% (4.3 µmol, 4.3 mg, t_R = 39.5 min (34% B)).

11-Desnoviosyl-13/15-thio-β-L-fucosyl fidaxomicin (**18d-C(13)+C(15)**) was obtained as a colorless solid as a 1:3 mixture of the C13 and C15 regioisomers in a yield of 18% (50.4 µmol, 49.9 mg, t_R = 44.4 min (34% B))

11-Desnoviosyl-11-thio-β-L-fucosyl fidaxomicin (18d-C(11))

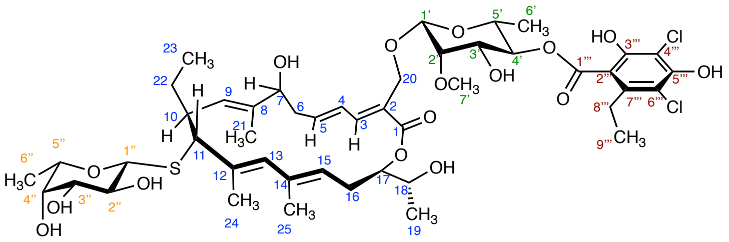

^{1}H NMR (500 MHz, acetone-d_6) δ (ppm) 7.24 (d, J = 11.4 Hz, 1H, H3), 6.64 (ddd, J = 15.5, 11.2, 1.6 Hz, 1H, H4), 6.00 (ddd, J = 14.7, 9.8, 4.5 Hz, 1H, H5), 5.72 (s, 1H, H13), 5.58 (t, J = 8.3 Hz, 1H, H15), 5.35 (dt, J = 10.1, 1.6 Hz, 1H, H9), 5.10 (t, J = 9.7 Hz, 1H, H4’), 4.72 (dt, J = 6.6, 4.9 Hz, 1H, H17), 4.68 (s, 1H, H1’), 4.61 (d, J = 11.5 Hz, 1H, H20a), 4.42 (d, J = 11.5 Hz, 1H, H20b), 4.26 (d, J = 4.1 Hz, 1H, H7), 4.17 (d, J = 9.7 Hz, 1H, H1”), 4.02 (p, J = 6.4 Hz, 1H, H18), 3.81 (dd, J = 9.9, 3.4 Hz, 1H, H3’), 3.67 – 3.56 (m, 5H, H11, H2’, H5’, H2”, H4”), 3.52 (s, 3H, H7’), 3.50 – 3.44 (m, 1H, H5”), 3.41 (dd, J = 9.0, 3.4 Hz, 1H, H3”), 3.01 (q, J = 7.3 Hz, 2H, H8’’’), 2.78 – 2.67 (m, 2H, H6a, H16a), 2.60 – 2.47 (m, 2H, H6b, H10), 2.43 (ddd, J = 13.9, 9.0, 4.5 Hz, 1H, H16b), 1.98 – 1.89 (m, 1H, H22a), 1.87 (s, 3H, H24), 1.75 (s, 3H, H25), 1.67 (s, 3H, H21), 1.38 – 1.27 (m, 1H, H22b), 1.31 (d, J = 6.1 Hz, 3H, H6’), 1.25 (d, J = 6.5 Hz, 3H, H6”), 1.22 (t, J = 7.4 Hz, 3H, H9’’’), 1.17 (d, J = 6.3 Hz, 3H, H19), 0.83 (t, J = 7.4 Hz, 3H, H23). **^{13}C NMR** (126 MHz, acetone-d_6) δ (ppm) 169.5 (C1’’’), 167.8 (C1), 156.0 (C3’’’), 154.0 (C5’’’), 145.4 (C3), 143.4 (C5), 142.7 (C7’’’), 136.6 (C8), 136.4 (C14), 133.8 (C12), 133.1 (C13), 128.3 (C4), 126.5 (C9), 125.7 (C15), 125.3 (C2), 114.6 (C6’’’), 110.5 (C2’’’), 108.2 (C4’’’), 101.7 (C1’), 84.4 (C1”), 81.6 (C2’), 78.2 (C17), 77.6 (C4’), 76.6 (C3”), 75.2 (C5”), 73.0 (C7), 72.6 (C4”), 72.3 (C3’), 71.2 (C2”), 70.6 (C5’), 67.6 (C18), 63.3 (C20), 61.7 (C7’), 58.1 (C11), 40.6 (C10), 37.3 (C6), 28.3 (C16), 27.4 (C22), 26.2 (C8’’’), 20.6 (C19), 18.2 (C6’), 17.7 (C25), 17.1 (C6”), 15.2 (C21), 14.4 (C9’’’), 13.4 (C24), 10.6 (C23). **HRMS** ESI(+)(MeOH) calculated for $C_{47}H_{66}O_{16}Cl_2NaS^+$ $[M+Na]^+$: 1011.33408, found: 1011.33513. **FT-IR** (acetone-d_6): ν (cm^{-1}) 3409m, 2975w, 2931w, 2875w, 1694m, 1643w, 1590w, 1454w, 1404w, 1380m, 1313m, 1246s, 1214m, 1199m, 1164w, 1144w, 1091m, 1065s, 1024m, 997w, 901w, 868w, 805w, 762w, 739w.

11-Desnoviosyl-13/15-thio-β-L-fucosyl fidaxomicin (mixture of regioisomers C13:C15 = 1:3, 18d-C(13)+C(15))

C(13) isomer

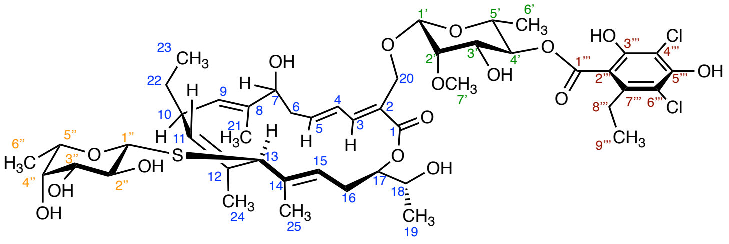

C(15) isomer

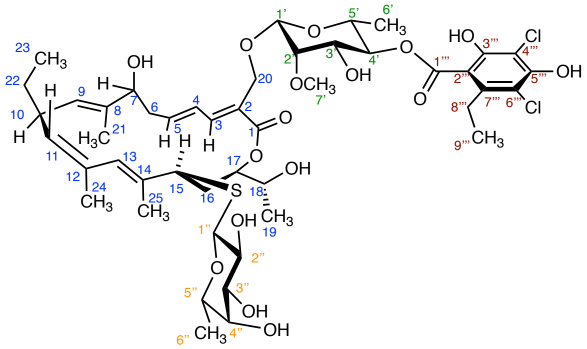

^{1}H NMR(500 MHz, acetone-d_6,): δ (ppm) 7.23 – 7.17 (m, 0.3H, C(13): H3), 7.13 (d, *J*=11.3 Hz, 1H, C(15): H3), 6.63 – 6.55 (m, 0.3H, C(13): H4), 6.34 (ddd, *J*=15.2, 11.3, 1.4 Hz, 1H, C(15): H4), 6.19 (ddd, *J*=15.0, 10.9, 4.2 Hz, 1H, C(15): H5), 6.05 (ddd, *J*=15.1, 9.7, 5.6 Hz, 0.3H, C(13): H5), 5.76 (ddt, *J*=10.0, 6.2, 1.3 Hz, 0.3H, C(13): H15), 5.70 (s, 1H, C(15): H13), 5.42 (dd, *J*=7.8, 1.5 Hz, 0.3H, C(13): H11), 5.32 (dt, *J*=9.9, 1.6 Hz, 0.3H, C(13): H9), 5.19 (dt, *J*=10.5, 1.4 Hz, 1H, C(15): H9), 5.14 – 5.06 (m, 3.3H, C(13): H4’, C(15): H11, H17, H4’,), 4.83 (dt, *J*=8.8, 3.5 Hz, 0.3H, C(13): H17), 4.66 (d, *J*=0.8 Hz, 1H, C(15): H1’), 4.62 (d, *J*=11.0 Hz, 1H, C(15): H20a), 4.64 – 4.58 (m, 0.6 H, C(13): H20a, H1’), 4.50 – 4.44 (m, 0.3H, C(13): H20b), 4.46 (d, *J*=11.1 Hz, 1H, C(15): H20b), 4.30 (t, *J*=3.4 Hz, 1.3H, C(13): H7, C(15): H7), 4.16 (d, *J*=9.6 Hz, 0.3H, C(13): H1”), 4.15 (d, *J*=9.1 Hz, 1H, C(15): H1”), 4.07 (s, 0.3H, C(13): H13), 3.96 – 3.89 (m, 0.3H, C(13): H18), 3.85 – 3.75 (m, 2.3H, C(13): H3’, C(15): H3’, H18), 3.67 – 3.54 (m, 4.9H, C(13): H2’, H5’, H4” C(15): H15, H2’, H5’, H4”), 3.54 – 3.50 (m, 4.2H, C(13): H7’, H2”, C(15): H7’), 3.49 – 3.41 (m, 3.6H, C(13): H3”, H5”, C(15): H2”, H3”, H5”), 3.16 – 3.06 (m, 1.3H, C(13): H10, C(15): H10), 3.04 – 2.97 (m, 2.6H, C(13): H8’’’, C(15): H8’’’), 2.73 (dddd, *J*=14.3, 5.2, 3.6, 1.5 Hz, 0.3H, C(13): H6a), 2.61 – 2.55 (m, 1.3H, C(13): H16a, C(15): H6a), 2.54 – 2.43 (m, 0.6H, C(13): H6b, H16b), 2.39 (ddd, *J*=13.6, 11.0, 2.7 Hz, 1H, C(15): H6b), 2.23 (dt, *J*=14.6, 11.2 Hz, 1H, C(15): H16a), 1.90 (d, *J*=1.1 Hz, 3H, C(15): H25), 1.89 – 1.85 (m, 1H, C(15): H16b), 1.79 (d, *J*=1.2 Hz, 3H, C(15): H24), 1.70 (t, *J*=0.9 Hz, 0.9H, C(13): H21), 1.62 (d, *J*=1.2 Hz, 0.9H, C(13): H24), 1.59 (d, *J*=1.3 Hz, 3H, C(15): H21), 1.55 – 1.45 (m, 0.3H, C(13): H22a), 1.50 (d, *J*=1.2 Hz, 0.9H, C(13): H25), 1.41 – 1.34 (m, 0.3H, C(13): H22b), 1.33 (d, *J*=6.2 Hz, 3H, C(15): H6’), 1.31 (d, *J*=6.1 Hz, 0.9H, C(13): H6’), 1.33 – 1.24 (m, 2H, C(15): H22a, H22b), 1.25 – 1.17 (m, 4.8H, C(13): H9’’’, H6” C(15): H9’’’), 1.17 – 1.15 (m, 3.9H, C(13): H19, C(15): H19), 1.14 (d, *J*=6.4 Hz, 3H, C(15): H6”), 0.91 (t, *J*=7.4 Hz, 0.9H, C(13): H23), 0.79 (t, *J*=7.4 Hz, 3H, C(15): H23). **^{13}C NMR** (126 MHz, acetone-d_6) δ (ppm) 169.7 (C(13): C1’’’), 169.4 (C(15): C1’’’), 167.6 (C(15): C1), 166.7 (C(13): C1), 156.2 (C(13): C3’’’), 155.8 (C(15): C3’’’), 154.0 (C(13): C5’’’), 153.8 (C(15): C5’’’), 145.0 (C(13): C3), 144.8 (C(15): C3), 143.5 (C(13): C5), 143.2 (C(15): C5), 142.9 (C(15): C7’’’), 142.6 (C(15): C7’’’), 137.1 (C(15): C11), 136.9 (C(13): C14), 135.9 (C(13): C12), 134.5 (C(15): C14), 134.3 (C(13): C8), 133.9 (C(15): C8), 133.7 (C(13): C11), 132.5 (C(15): C12, C13), 128.0 (C(13): C4), 127.1 (C(15): C4), 127.1 (C(15): C9), 127.1 (C(13): C9), 125.3 (C(15): C2), 125.2 (C(13): C2), 121.9 (C(13): C15), 114.6 (C(13): C6’’’), 114.5 (C(15): C6’’’), 110.7 (C(15):

C2'''), 110.4 (C(13): C2'''), 108.3 (C(15): C4'''), 108.2 (C(13): C4'''), 100.8 (C(13): C1'), 100.0 (C(15): C1'), 87.4 (C(15): C1''), 86.9 (C(13): C1''), 81.7 (C(13): C2', C(15): C2'), 78.1 (C(15): C17), 77.9 (C(13): C17), 77.7 (C(13): C4'), 77.5 (C(15): C4'), 76.5 (C(15): C3''), 76.4 (C(13): C3''), 75.4 (C(15): C5''), 75.3 (C(13): C5''), 73.8 (C(15): C7), 72.9 (C(13): C7), 72.6 (C(15): C4''), 72.5 (C(13): C4''), 72.4 (C(13): C3'), 72.3 (C(15): C3'), 71.8 (C(13): C2''), 71.1 (C(15): C2''), 70.8 (C(15): C5'), 70.6 (C(13): C5'), 69.7 (C(15): C18), 66.7 (C(13): C18), 62.7 (C(13): C13), 62.6 (C(13): C20), 61.9 (C(15): C20), 61.8 (C(15): C7'), 61.7 (C(13): C7'), 55.8 (C(15): C15), 39.6 (C(15): C10), 39.3 (C(13): C10), 38.4 (C(15): C6), 37.6 (C(13): C6), 31.6 (C(15): C16), 30.2 (C(13): C22), 29.8 (C(15): C22), 29.1 (C(13): C16), 26.3 (C(13): C8'''), 26.2 (C(15): C8'''), 21.2 (C(13): C19), 19.1 (C(15): C19), 18.4 (C(13): C6'), 18.0 (C(15): C6'), 17.3 (C(15): C24), 17.1 (C(15): C6''), 17.1 (C(13): C6''), 16.7 (C(13): C25), 15.5 (C(15): C21), 14.7 (C(13): C21), 14.7 (C(15): C25), 14.4 (C(13): C9'''), 14.4 (C(15): C9'''), 12.7 (C(13): C24), 12.4 (C(13): C23), 12.0 (C(15): C23). **HRMS** ESI(+)(MeOH) calculated for $C_{47}H_{66}O_{16}Cl_2NaS^+$ $[M+Na]^+$: 1011.33408, found: 1011.33217. **FT-IR** (acetone-d_6): ν (cm^{-1}) 3417m, 2976w, 2934w, 2874w, 1696m, 1640m, 1590w, 1454w, 1405w, 1379m, 1311m, 1241s, 1199m, 1164m, 1145w, 1092m, 1065s, 1024s, 996m, 916w, 865w, 802w, 762w, 739w, 674w.

11-Desnoviosyl-xy-thio-(4''-desbutyryl)-β-D-noviosyl fidaxomicin (18e)

11-Desnoviosyl-xy-thio-(4''-desbutyryl)-β-D-noviosyl fidaxomicin (**18e**) was synthesized from Fdx (**1**, 215 µmol, 227 mg, 1.0 equiv.) and 2,3-di-*O*-acetyl-4-*O*-isobutyryl-1-β-thio-D-noviose (**9**, 215 µmol, 75 mg, 1.0 equiv.) in MeCN (2058 µL, 0.1 M) and a solution of $Cu[ClO_4]_2•6\ H_2O$ (10 mM in MeCN, 215 µL, 1 mol%) following General procedure B. The crude material derived from the organic phase was dissolved in MeOH (2255 µL, 0.1 M). Potassium carbonate (860 µmol,119 mg, 4.0 equiv.) was added to the solution and the mixture stirred at 25 °C. After 45 min, the reaction was diluted with MeCN (10 mL) (precipitation), and the mixture filtered over a Supelco® Discovery® DSC-18 SPE tube (500 mg) and the column rinsed with another 20 mL of MeCN to provide after concentration *in vacuo* the crude material as a colorless to pale yellow solid. The remaining precipitate on the DSC-18 SPE tube was dissolved by rinsing the DSC-18 tube with 10 mL water and the aqueous phase then extracted with EtOAc (3×10 mL). The combined organic phase was washed with brine (1×30 mL), dried over anhydrous $MgSO_4$, filtered, and concentrated *in vacuo* to provide a colorless to pale rose solid. The combined crude material was purified by preparative RP-HPLC using an isocratic eluent of 37% B (LC time program (time - %B): 0 min - 37%, 65 min - 37%). Product containing fractions were combined separately and repurified by preparative RP-HPLC under the same conditions. Product containing fractions were combined separately and concentrated *in vacuo* at 44 °C (water bath):

11-Desnoviosyl-11-thio-(4''-desbutyryl)-β-D-noviosyl fidaxomicin (***S*-OP1118, 18e-C(11)**) was obtained as a colorless solid in a yield of 2% (3.3 µmol, 3.3 mg, t_R = 46.8 min).

11-Desnoviosyl-13-thio-(4''-desbutyryl)-β-D-noviosyl fidaxomicin (**18e-C(13)**) was obtained as a colorless solid with a purity of approx. 94% (determined from ^1H NMR spectrum by comparing the integral of a 1H signal to the sum of the integrals of this signal and the respective signal of the impurity) in a yield of 2% (3.8 µmol, 3.8 mg, t_R = 48.7 min).

11-Desnoviosyl-15-thio-(4''-desbutyryl)-β-D-noviosyl fidaxomicin (**18e-C(15)**) was obtained as a colorless solid in a yield of 12% (25 µmol, 25.1 mg, t_R = 43.2 min).

11-Desnoviosyl-11-thio-(4”-desbutyryl)-β-D-noviosyl fidaxomicin (S-OP1118, 18e-C(11))

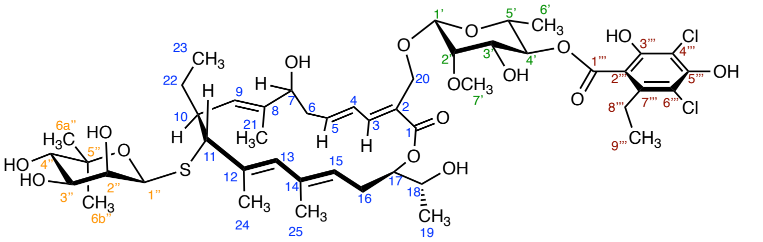

^{1}H NMR (500 MHz, acetone-d$_6$) δ (ppm) 7.28 (d, *J* = 11.7 Hz, 1H, H3), 6.64 (dddd, *J* = 14.7, 11.6, 2.1, 1.0 Hz, 1H, H4), 6.00 (ddd, *J* = 14.6, 9.6, 4.6 Hz, 1H, H5), 5.78 (s, 1H, H13), 5.67 – 5.58 (m, 1H, H15), 5.32 (dt, *J* = 10.1, 1.6 Hz, 1H, H9), 5.10 (t, *J* = 9.7 Hz, 1H, H4’), 4.81 (d, *J* = 1.4 Hz, 1H, H1”), 4.73 (dt, *J* = 6.8, 4.9 Hz, 1H, H17), 4.68 (d, *J* = 0.9 Hz, 1H, H1’), 4.60 (d, *J* = 11.5 Hz, 1H, H20a), 4.42 (d, *J* = 11.5 Hz, 1H, H20b), 4.28 – 4.21 (m, 1H, H7), 4.05 – 3.99 (m, 1H, H18), 3.93 (dd, *J* = 3.5, 1.3 Hz, 1H, H2”), 3.79 (dd, *J* = 9.9, 3.4 Hz, 1H, H3’), 3.66 – 3.57 (m, 2H, H2’, H5’), 3.55 (dd, *J* = 9.9, 3.5 Hz, 1H, H3”), 3.52 (s, 3H, H7’), 3.46 (d, *J* = 9.9 Hz, 1H, H4”), 3.41 (d, *J* = 10.8 Hz, 1H, H11), 3.08 – 2.94 (m, 2H, H8’’’), 2.79 – 2.66 (m, 2H, H16b (H_{Re}), H6b (H_{Re})), 2.55 – 2.39 (m, 3H, H16a (H_{Si}), H10, H6a (H_{Si})), 2.04 – 1.94 (m, 1H, H22a (H_{Re})), 1.87 (d, *J* = 1.2 Hz, 3H, H24), 1.72 (d, *J* = 1.4 Hz, 3H, H25), 1.64 (dd, *J* = 1.4, 0.7 Hz, 3H, H21), 1.35 – 1.28 (m, 1H, H22b (H_{Si})), 1.31 (d, *J* = 6.1 Hz, 3H, H6’), 1.25 – 1.18 (m, 6H, H6”a (equatorial), H9’’’), 1.18 (d, *J* = 6.3 Hz, 3H, H19), 1.09 (s, 3H, H6”b (axial)), 0.82 (t, *J* = 7.4 Hz, 3H, H23). **^{13}C NMR** (126 MHz, acetone-d$_6$) δ (ppm) 169.8 (C1’’’), 167.8 (C1), 156.4 (C3’’’ or C5’’’), 155.5 (C5’’’ or C3’’’), 145.6 (C3), 143.6 (C5), 142.6 (C7’’’), 136.5 (C8), 136.5 (C12), 136.1 (C14), 131.7 (C13), 128.2 (C4), 126.3 (C15), 126.2 (C9), 125.2 (C2), 115.3 (C6’’’), 108.7 (C2’’’), 108.1 (C4’’’), 101.7 (C1’), 81.7 (C2’), 81.3 (C1”), 78.3 (C17), 77.7 (C5”), 77.3 (C4’), 74.5 (C2”), 74.2 (C4”), 72.9 (C7), 72.3 (C3”), 72.3 (C3’), 70.7 (C5’), 67.6 (C18), 63.3 (C20), 62.8 (C11), 61.7 (C7’), 41.5 (C10), 37.2 (C6), 28.8 (C6”a (equatorial)), 28.4 (C16), 27.7 (C22), 26.3 (C8’’’), 20.7 (C19), 18.3 (C6’), 17.6 (C25), 16.7 (C6”b (axial)), 15.1 (C21), 14.4 (C9’’’), 14.3 (C24), 10.9 (C23). (*Note*: Signal reported for Fdx at 110.2 ppm[5] was not observed. But HMBC signal of H8’’’ with C-atoms at 142.6 ppm, 115.3 ppm, 108.7 ppm (C-signal has low intensity), and 14.4 ppm). **HRMS** ESI(+)(MeOH) calculated for $C_{48}H_{68}O_{16}Cl_2NaS^+$ [M+Na]$^+$: 1025.34973, found: 1025.35026. **Specific Rotation** $[\alpha]_D^{23°C}$ = +32.1(c = 0.06, MeOH). **FT-IR** (MeOH): ν (cm^{-1}) 3376m, 2921s, 2851m, 1693m, 1637m, 1578w, 1510w, 1459m, 1376m, 1312m, 1282m, 1253s, 1213m, 1198m, 1066s, 1023s, 951w, 793w, 761w, 692w.

11-Desnoviosyl-13-thio-(4”-desbutyryl)-β-D-noviosyl fidaxomicin (18e-C(13))

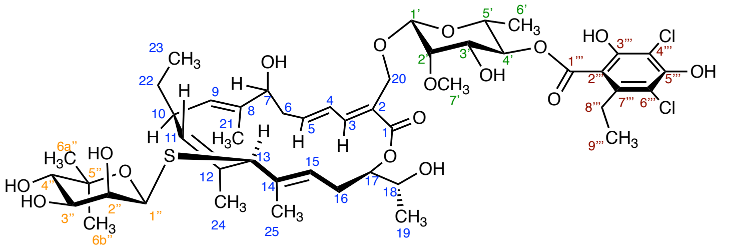

^{1}H NMR (500 MHz, acetone-d$_6$) δ (ppm) 7.20 (d, *J* = 11.3 Hz, 1H, H3), 6.60 (dd, *J* = 15.0, 11.4 Hz, 1H, H4), 6.06 (ddd, *J* = 15.1, 9.5, 5.6 Hz, 1H, H5), 5.64 – 5.57 (m, 1H, H15), 5.38 (dd, *J* = 7.8, 1.4 Hz, 1H, H11), 5.33 (dt, *J* = 9.9, 1.6 Hz, 1H, H9), 5.10 (t, *J* = 9.7 Hz, 1H, H4’), 4.83 (dt, *J* = 9.0, 3.3 Hz, 1H, H17), 4.73 (d, *J* = 1.5 Hz, 1H, H1”), 4.62 (d, *J* = 11.7 Hz, 1H, H20a), 4.61 (s, 1H, H1’), 4.49 (d, *J* = 11.5 Hz, 1H, H20b), 4.31 (s, 1H, H7), 3.92 – 3.84 (m, 1H, H18), 3.86 (s, 1H, H13), 3.82 – 3.74 (m, 2H, H2”, H3’), 3.69 – 3.60 (m, 1H, H5’), 3.59 – 3.53 (m, 3H, H3”, H4”, H2’), 3.52 (s, 3H, H7’), 3.24 – 3.16 (m, 1H, H10), 3.02 (q, *J* = 7.4 Hz, 2H, H8’’’), 2.77 – 2.70 (m, 1H, H6b (H_{Re})), 2.60 – 2.41 (m, 3H, H6a (H_{Si}), H16a (H_{Si}), H16b (H_{Re})), 1.71 (d, *J* = 1.3 Hz, 3H, H21), 1.58 (d, *J* = 1.1 Hz, 3H, H24), 1.58 – 1.52 (m, 1H, H22b (H_{Re})), 1.51 (s, 3H, H25), 1.41 – 1.33 (m, 1H, H22a (H_{Si})), 1.31 (d, *J* = 6.1 Hz, 3H, H6’), 1.24 (s, 3H, H6”a (equatorial)), 1.23 (t, *J* = 7.4 Hz, 3H, H9’’’), 1.21 (s, 3H, H6”b (axial)), 1.15 (d, *J* = 6.1 Hz, 3H, H19), 0.90 (t, *J* = 7.3 Hz, 3H, H23). **^{13}C NMR** (126 MHz, acetone-d$_6$) δ (ppm) 169.8 (C1’’’), 166.6 (C1), 156.4 (C3’’’), 154.6 (C5’’’), 144.9 (C3), 143.4 (C5), 142.8 (C7”), 137.6 (C14), 135.1 (C12), 134.6 (C8), 134.5 (C11), 127.9 (C4), 126.7 (C9),

125.2 (C2), 121.4 (C15), 114.9 (C6'''), 109.6 (C2'''), 108.2 (C4'''), 100.8 (C1'), 81.7 (C2'), 78.1 (C1''), 78.0 (C17), 77.8 (C5''), 77.6 (C4'), 74.4 (C4''), 74.2 (C2''), 72.8 (C7), 72.6 (C3''), 72.4 (C3'), 70.7 (C5'), 66.6 (C18), 62.6 (C20), 61.7 (C7'), 60.4 (C13), 39.1 (C10), 37.6 (C6), 30.2 (C22), 29.1 (C16), 28.8 (C6''a (equatorial)), 26.4 (C8'''), 21.3 (C19), 18.4 (C6'), 18.2 (C6''b (axial)), 17.2 (C25), 14.8 (C21), 14.4 (C9'''), 12.0 (C23), 12.0 (C24). **HRMS** ESI(+)(MeOH) calculated for $C_{48}H_{68}O_{16}Cl_2NaS^+$ $[M+Na]^+$: 1025.34973, found: 1025.34939. **Specific Rotation** $[\alpha]_D^{23°C}$ = -92.3 (c = 0.07, MeOH). **FT-IR** (MeOH): ν (cm^{-1}) 3389m, 2976w, 2925w, 2874w, 2853w, 1693w, 1641m, 1588w, 1511w, 1453w, 1375m, 1313m, 1282w, 1246s, 1203m, 1090m, 1066s, 1022m, 986w, 951w, 860w, 794w, 763w, 690w, 591w.

11-Desnoviosyl-15-thio-(4''-desbutyryl)- β-D-noviosyl fidaxomicin (18e-C(15))

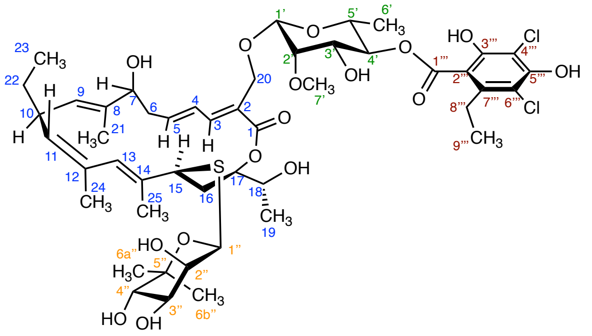

^{1}H NMR (acetone-d_6, 500 MHz): δ (ppm) 7.15 (d, *J*=11.2 Hz, 1H, H3), 6.32 (ddd, *J*=15.1, 11.3, 1.4 Hz, 1H, H4), 6.20 (ddd, *J*=15.0, 11.0, 4.0 Hz, 1H, H5), 5.62 (d, *J*=1.4 Hz, 1H, H13), 5.19 (dt, *J*=10.4, 1.4 Hz, 1H, H9), 5.16 – 5.11 (m, 1H, H17), 5.12 (t, *J*=9.7 Hz, 1H, H4'), 5.04 (d, *J*=9.5 Hz, 1H, H11), 4.66 (s, 1H, H1'), 4.65 (d, *J*=1.2 Hz, 1H, H1''), 4.62 (d, *J*=10.9 Hz, 1H, H20b), 4.45 (d, *J*=10.9 Hz, 1H, H20a), 4.32 – 4.28 (m, 1H, H7), 3.86 – 3.78 (m, 2H, H18, H3'), 3.74 (dd, *J*=3.3, 1.4 Hz, 1H, H2''), 3.60 – 3.52 (m, 3H, H15, H2', H5'), 3.52 (s, 3H, H7'), 3.52 (d, *J*=9.8 Hz, 1H, H4''), 3.48 (dd, *J*=9.9, 3.2 Hz, 1H, H3''), 3.16-3.06 (m, 1H, H10), 2.99 (qd, *J*=7.4, 2.0 Hz, 2H, H8'''), 2.59 (dtd, *J*=13.4, 4.2, 1.4 Hz, 1H, H6a (H_{Si})), 2.37 (ddd, *J*=13.5, 11.0, 2.7 Hz, 1H, H6b (H_{Re})), 2.23 (dt, *J*=14.6, 11.4 Hz, 1H, H16a (H_{Si})), 1.90 (d, *J*=1.1 Hz, 3H, H25), 1.82 (d, *J*=1.2 Hz, 3H, H24), 1.81 – 1.76 (m, 1H, H16b (H_{Re})), 1.58 (d, *J*=1.3 Hz, 3H, H21), 1.33 (d, *J*=6.2 Hz, 3H, H6'), 1.31 – 1.23 (m, 2H, H22a (H_{Re}), H22b (H_{Si})), 1.27 (s, 3H, H6''a (equatorial)), 1.20 (t, *J*=7.4 Hz, 3H, H9'''), 1.16 (d, *J*=6.5 Hz, 3H, H19), 1.14 (s, 3H, H6''b (axial)), 0.78 (t, *J*=7.4 Hz, 3H, H23). **^{13}C NMR** (126 MHz, acetone-d_6) δ (ppm) 169.3 (C1'''), 167.5 (C1), 155.7 (C3'''), 153.7 (C5'''), 144.9 (C3), 143.2 (C5), 142.6 (C7'''), 138.1 (C11), 134.0 (C13), 133.8 (C8), 132.7 (C14), 131.9 (C12), 127.2 (C4), 127.1 (C9), 125.2 (C2), 114.5 (C6'''), 110.7 (C2'''), 108.1 (C4'''), 100.1 (C1'), 81.6 (C2'), 78.1 (C17), 77.8 (C5''), 77.7 (C1''), 77.5 (C4'), 74.2 (C4''), 73.9 (C2''), 73.8 (C7), 72.4 (C3''), 72.2 (C3'), 70.8 (C5'), 69.6 (C18), 61.9 (C20), 61.8 (C7'), 53.5 (C15), 39.5 (C10), 38.3 (C6), 31.1 (C16), 29.8 (C22), 28.9 (C6''a (equatorial)), 26.2 (C8'''), 19.0 (C19), 18.0 (C6''b (axial), C6'), 17.0 (C24), 15.5 (C21), 14.3 (C9'''), 13.5 (C25), 11.9 (C23). **HRMS** ESI(+)(MeOH) calculated for $C_{48}H_{68}O_{16}Cl_2NaS^+$ $[M+Na]^+$: 1025.34973, found: 1025.35005. **Specific Rotation** $[\alpha]_D^{23°C}$ = +21.4(c = 0.36, MeOH). **FT-IR** (acetone-d_6): ν (cm^{-1}) 3421m, 2977w, 2931w, 2874w, 1696m, 1640w, 1590w, 1405w, 1376w, 1311m, 1240s, 1199m, 1181m, 1148w, 1091s, 1068s, 1023s, 951w, 857w, 762w, 680w.

4''-*O*-acyl-*S*-Fdx derivatives

11-Desnoviosyl-xy-thio-β-D-noviosyl fidaxomicin (3a)

11-Desnoviosyl-xy-thio-β-D-noviosyl fidaxomicin (**3a**) was synthesized after General procedure C from Fdx (**1**, 90 µmol, 95 mg, 1.0 equiv.), and (3aS,4S,7S,7aS)-4-mercapto-2,2,6,6-tetramethyltetrahydro-4H-[1,3]dioxolo[4,5-c]pyran-7-yl isobutyrate (**17a**, 90 µmol, 27 mg, 1.0 equiv.) in MeCN (864 µL, 0.1 M) using milliQ water (1.8 mmol, 33µL, 20 equiv.) and $Cu[ClO_4]_2$•6 H_2O (10 mM solution in MeCN, 450 µL, 5 mol%). The reaction mixture was stirred at 25 °C for 19 h. Purification of the crude material after aqueous workup and filtration was performed by preparative RP-HPLC using an isocratic eluent of 48% B (LC time program

(time - %B): 0 min - 48%, 110 min – 48%). Product containing fractions (C13 and C11 isomer) were combined separately and repurified by preparative RP-HPLC using the same conditions. Product containing fractions were combined separately and concentrated *in vacuo* at 44 °C (water bath):

11-Desnoviosyl-11-thio-β-D-noviosyl fidaxomicin (*S*-Fdx, **3a-C(11)**) was obtained as a colorless solid in a yield of 3% (2.7 µmol, 2.9 mg, t_R =43.3 min).

11-Desnoviosyl-13-thio-β-D-noviosyl fidaxomicin (**3a-C(13)**) was obtained as a colorless solid in a yield of 10% (9.3 µmol, 10 mg, t_R = 51.7 min).

11-Desnoviosyl-15-thio-β-D-noviosyl fidaxomicin (**3a-C(15)**) was obtained as a colorless solid in a yield of 24% (21.4 µmol, 23 mg, t_R = 41.8 min).

11-Desnoviosyl-11-thio-β-D-noviosyl fidaxomicin (S-Fdx, 3a-C(11))

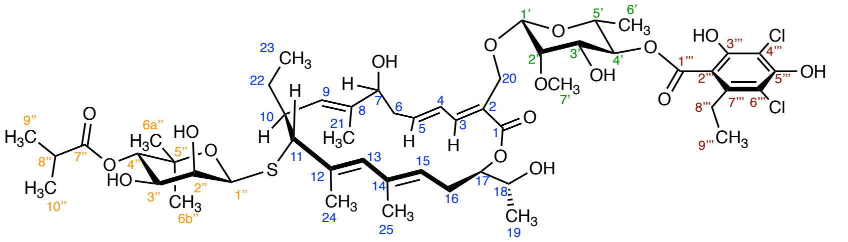

^{1}H NMR (500 MHz, acetone-d_6): δ (ppm) 7.28 (d, *J*=11.5 Hz, 1H, H3), 6.68 – 6.60 (m, 1H, H4), 6.00 (ddd, *J*=14.7, 9.7, 4.6 Hz, 1H, H5), 5.79 (s, 1H, H13), 5.63 (t, *J*=8.2 Hz, 1H, H15), 5.32 (dt, *J*=10.2, 1.6 Hz, 1H, H9), 5.10 (t, *J*=9.7 Hz, 1H, H4’), 4.97 (d, *J*=10.2 Hz, 1H, H4’’), 4.87 (d, *J*=1.3 Hz, 1H, H1’’), 4.72 (dt, *J*=6.8, 4.8 Hz, 1H, H17), 4.68 (d, *J*=0.8 Hz, 1H, H1’), 4.60 (d, *J*=11.5 Hz, 1H, H20a), 4.42 (d, *J*=11.5 Hz, 1H, H20b), 4.26 (s, 1H, H7), 4.06 – 3.99 (m, 2H, H18, H2’’), 3.80 (dd, *J*=9.9, 3.4 Hz, 1H, H3’), 3.77 (dd, *J*=10.2, 3.4 Hz, 1H, H3’’), 3.65 – 3.58 (m, 2H, H2’, H5’), 3.52 (s, 3H, H7’), 3.43 (d, *J*=10.9 Hz, 1H, H11), 3.01 (qd, *J*=7.4, 1.9 Hz, 2H, H8’’’), 2.77 – 2.67 (m, 2H, H6a, H16a), 2.56 (hept, *J*=7.1 Hz, 1H, H8’’), 2.55 – 2.39 (m, 3H, H6b, H10, H16b), 2.02 – 1.94 (m, 1H, H22a), 1.87 (d, *J*=1.2 Hz, 3H, H24), 1.72 (d, *J*=1.4 Hz, 3H, H25), 1.65 (d, *J*=1.3 Hz, 3H, H21), 1.31 (d, *J*=6.2 Hz, 3H, H6’), 1.35 – 1.26 (m, 1H, H22b), 1.22 (t, *J*=7.4 Hz, 3H, H9’’’), 1.18 (d, *J*=6.5 Hz, 3H, H19), 1.17 (s, 3H, H6b’’), 1.15 (d, *J*=7.0 Hz, 3H, H9’’), 1.13 (d, *J*=7.0 Hz, 3H, H10’’), 1.08 (s, 3H, H6a’’), 0.82 (t, *J*=7.4 Hz, 3H, H23). **^{13}C NMR** (126 MHz, acetone-d_6) δ (ppm) 176.8 (C7’’), 169.6 (C1’’’), 167.8 (C1), 156.2 (C3’’’), 154.6 (C5’’’), 145.6 (C3), 143.6 (C5), 142.6 (C7’’’), 136.6 (C8), 136.5 (C12), 136.1 (C14), 131.7 (C13), 128.2 (C4), 126.5 (C15), 126.1 (C9), 125.2 (C2), 114.9 (C6’’’), 109.9 (C2’’’), 108.3 (C4’’’), 101.7 (C1’), 81.7 (C2’), 81.4 (C1’’), 78.2 (C17), 77.5 (C4’), 76.5 (C5’’), 75.4 (C4’’), 74.9 (C2’’), 73.0 (C7), 72.4 (C3’), 70.7 (C5’), 70.4 (C3’’), 67.7 (C18), 63.3 (C20), 62.9 (C11), 61.7 (C7’), 41.4 (C10), 37.2 (C6), 34.8 (C8’’), 28.5 (C6a’’), 28.4 (C16), 27.7 (C22), 26.3 (C8’’’), 20.8 (C19), 19.4 (C9’’), 19.1 (C10’’), 18.2 (C6’), 17.7 (C6b’’), 17.6 (C25), 15.1 (C21), 14.4 (C9’’’), 14.3 (C24), 10.8 (C23). **HRMS** ESI(+)(MeOH) calculated for $C_{52}H_{74}O_{17}Cl_2NaS^+$ $[M+Na]^+$: 1095.39160, found: 1095.39151. **Specific Rotation** $[\alpha]_D^{24°C}$ = 4.90 (c = 0.23). **FT-IR** (acetone-d_6): ν (cm^{-1}) 3421w, 2976m, 2931m, 2876w, 1696m, 1643m, 1590w, 1455w, 1404w, 1384m, 1371m, 1313m, 1247s, 1199s, 1161m, 1114m, 1068s, 1024s, 958w, 901w, 858w, 765w, 692w.

11-Desnoviosyl-13-thio-β-D-noviosyl fidaxomicin (3a-C(13))

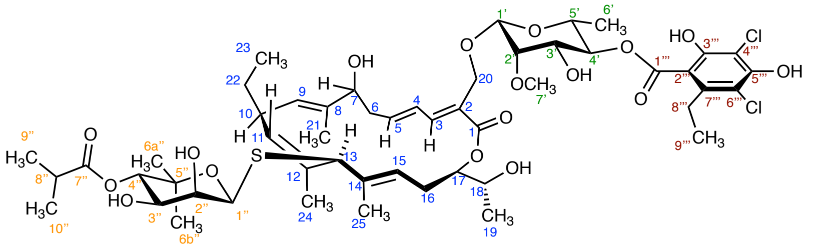

^{1}H NMR (500 MHz, acetone-d_6) δ (ppm) 7.20 (dd, *J* = 11.4, 0.8 Hz, 1H, H3), 6.60 (dd, *J* = 15.0, 11.4 Hz, 1H, H4), 6.06 (ddd, *J* = 15.1, 9.5, 5.6 Hz, 1H, H5), 5.62 (dd, *J* = 10.2, 6.3 Hz, 1H, H15), 5.40 (dd, *J* = 8.0, 1.4

Hz, 1H, H11), 5.32 (dt, *J* = 9.9, 1.6 Hz, 1H, H9), 5.10 (t, *J* = 9.7 Hz, 1H, H4'), 5.05 (d, *J* = 10.2 Hz, 1H, H4''), 4.83 (dt, *J* = 9.1, 3.3 Hz, 1H, H17), 4.79 (d, *J* = 1.4 Hz, 1H, H1''), 4.61 (d, *J* = 0.9 Hz, 1H, H1'), 4.61 (d, *J* = 11.4 Hz, 1H, H20a), 4.49 (d, *J* = 11.6 Hz, 1H, H20b), 4.31 (s, 1H, H7), 4.21 (d, *J* = 5.9 Hz, 1H, OH18), 4.16 (d, *J* = 5.5 Hz, 1H, OH2''), 3.94 – 3.85 (m, 2H, H18, H2''), 3.88 (s, 1H, H13), 3.82 – 3.74 (m, 2H, H3', H3''), 3.69 – 3.61 (m, 1H, H5'), 3.57 (d, *J* = 3.0 Hz, 1H, H2'), 3.52 (s, 3H, H7'), 3.25 – 3.17 (m, 1H, H10), 3.02 (q, *J* = 7.4 Hz, 2H, H8'''), 2.76 – 2.69 (m, 1H, H6a), 2.58 (hept, *J* = 7.0 Hz, 1H, H8''), 2.58 – 2.41 (m, 3H, H6b, H16a, H16b), 1.71 (dd, *J* = 1.3, 0.7 Hz, 3H, H21), 1.59 (d, *J* = 1.2 Hz, 3H, H24), 1.63 – 1.54 (m, 1H, H22a), 1.51 (s, 3H, H25), 1.41 – 1.34 (m, 1H, H22b), 1.31 (d, *J* = 6.2 Hz, 3H, H6'), 1.30 (s, 3H, H6b''), 1.23 (t, *J* = 7.4 Hz, 3H, H9'''), 1.16 (d, *J* = 7.1 Hz, 3H, H9''), 1.16 (d, *J* = 6.0 Hz, 3H, H19), 1.14 (d, *J* = 7.0 Hz, 3H, H10''), 1.13 (s, 3H, H6a''), 0.91 (t, *J* = 7.4 Hz, 3H, H23). **^{13}C NMR** (126 MHz, acetone-d_6) δ (ppm) 176.8 (C7''), 169.7 (C1'''), 166.6 (C1), 156.4 (C3'''), 154.4 (C5'''), 144.9 (C3), 143.4 (C5), 142.8 (C7'''), 137.5 (C14), 135.0 (C12), 134.7 (C11), 134.6 (C8), 128.0 (C4), 126.6 (C9), 125.3 (C2), 121.5 (C15), 114.8 (C6'''), 110.2 (C2'''), 108.2 (C4'''), 100.8 (C1'), 81.7 (C2'), 78.2 (C1''), 78.0 (C17), 77.6 (C4'), 76.6 (C5''), 75.4 (C4''), 74.3 (C2''), 72.8 (C7), 72.4 (C3'), 70.7 (C5'), 70.6 (C3''), 66.6 (C18), 62.7 (C20), 61.7 (C7'), 60.4 (C13), 39.1 (C10), 37.6 (C6), 34.8 (C8''), 30.2 (C22), 29.1 (C16), 28.6 (C6a''), 26.3 (C8'''), 21.3 (C19), 19.4 (C9''), 19.2 (C6b''), 19.1 (C10''), 18.4 (C6'), 17.2 (C25), 14.8 (C21), 14.4 (C9'''), 12.0 (C23), 12.0 (C24). **HRMS** ESI(+)(MeOH) calculated for $C_{52}H_{74}O_{17}Cl_2NaS^+$ $[M+Na]^+$: 1095.39160, found: 1095.39109. **Specific Rotation** $[\alpha]_D^{23°C}$ = - 143.37 (c = 0.42). **FT-IR** (solid): ν (cm^{-1}) 3423w, 2976w, 2934w, 2875w, 1702m, 1643w, 1589w, 1455w, 1406w, 1369m, 1313m, 1239s, 1196s, 1158m, 1111m, 1065s, 1021s, 957m, 901w, 859w, 831w, 798w, 790w, 762w, 737, 690, 531.

11-Desnoviosyl-15-thio-β-D-noviosyl fidaxomicin (3a-C(15))

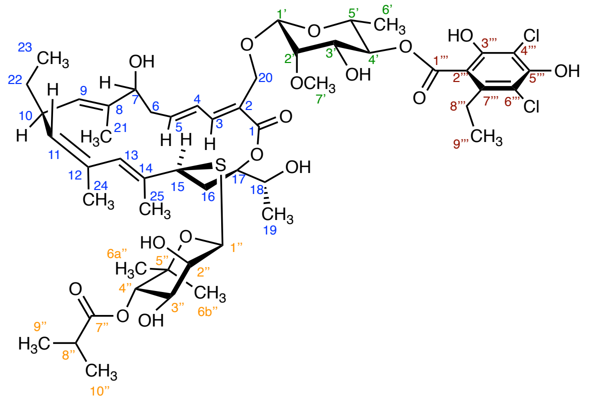

^{1}H NMR (500 MHz, acetone-d_6) δ (ppm) 7.15 (d, *J* = 11.2 Hz, 1H, H3), 6.32 (ddd, *J* = 15.1, 11.4, 1.4 Hz, 1H, H4), 6.20 (ddd, *J* = 15.1, 10.9, 4.1 Hz, 1H, H5), 5.63 (d, *J* = 1.4 Hz, 1H, H13), 5.19 (dt, *J* = 10.5, 1.4 Hz, 1H, H9), 5.17 – 5.11 (m, 1H, H17), 5.12 (t, *J* = 9.8 Hz, 1H, H4'), 5.05 (d, *J* = 9.5 Hz, 1H, H11), 5.02 (d, *J* = 10.2 Hz, 1H, H4''), 4.71 (d, *J* = 1.3 Hz, 1H, H1''), 4.67 (d, *J* = 0.8 Hz, 1H, H1'), 4.62 (d, *J* = 10.9 Hz, 1H, H20a), 4.45 (d, *J* = 10.8 Hz, 1H, H20b), 4.31 (t, *J* = 3.5 Hz, 1H, H7), 4.10 (d, *J* = 5.5 Hz, 1H, OH2''), 3.86 – 3.80 (m, 3H, H18, H2'', H3'), 3.70 (dd, *J* = 10.3, 3.5 Hz, 1H, H3''), 3.63 – 3.53 (m, 3H, H15, H2', H5'), 3.52 (s, 3H, H7'), 3.16 – 3.08 (m, 1H, H10), 3.00 (qd, *J* = 7.2, 1.0 Hz, 2H, H8'''), 2.63 – 2.55 (m, 1H, H6a), 2.57 (hept, *J* = 7.0 Hz, 1H, H8''), 2.37 (ddd, *J* = 13.5, 11.0, 2.7 Hz, 1H, H6b), 2.24 (dt, *J* = 14.6, 11.4 Hz, 1H, H16a), 1.91 (d, *J* = 1.1 Hz, 3H, H25), 1.83 (d, *J* = 1.2 Hz, 3H, H24), 1.83 – 1.78 (m, 1H, H16b), 1.58 (d, *J* = 1.4 Hz, 3H, H21), 1.33 (d, *J* = 6.2 Hz, 3H, H6'), 1.31 – 1.24 (m, 2H, H22a, H22b), 1.22 (s, 3H, H6b''), 1.20 (t, *J* = 7.4 Hz, 3H, H9'''), 1.17 (d, *J* = 6.5 Hz, 3H, H19), 1.16 (s, 3H, H6a''), 1.15 (d, *J* = 7.0 Hz, 3H, H9''), 1.13 (d, *J* = 7.0 Hz, 3H, H10''), 0.79 (t, *J* = 7.4 Hz, 3H, H23). **^{13}C NMR** (126 MHz, acetone-d_6) δ (ppm) 176.8 (C7''), 169.4 (C1'''), 167.5 (C1), 155.9 (C3'''), 153.9 (C5'''), 144.8 (C3), 143.2 (C5), 142.6 (C7'''), 138.2 (C11), 134.1 (C8), 133.9 (C13), 132.7 (C14), 132.0 (C12), 127.2 (C4), 127.1 (C9), 125.2 (C2), 114.6 (C6'''), 110.6 (C2'''), 108.2 (C4'''), 100.1 (C1'), 81.7 (C2'), 78.1 (C17), 77.9 (C1''), 77.5 (C4'), 76.6 (C5''), 75.3 (C4''), 74.2 (C2''), 74.0 (C7), 72.3 (C3'), 70.8 (C5'), 70.5 (C3''), 69.7 (C18), 61.9 (C20), 61.8 (C7'), 53.6 (C15), 39.5 (C10), 38.4 (C6), 34.7 (C8''), 31.1 (C16), 29.9 (C22), 28.6 (C6a''), 26.2 (C8'''), 19.4 (C9''), 19.2 (C10''), 19.1 (C19), 18.8 (C6b''), 18.0 (C6'),

17.0 (C24), 15.5 (C21), 14.4 (C9’’’), 13.6 (C25), 12.0 (C23). **HRMS** ESI(+)(MeOH) calculated for $C_{52}H_{74}O_{17}Cl_2NaS^+$ [M+Na]$^+$: 1095.39160, found: 1095.38989. **Specific Rotation** $[\alpha]_D^{23°C}$ = 7.11 (c = 0.97). **FT-IR** (solid): ν (cm^{-1}) 3422w, 2976w, 2934w, 2875w, 1706m, 1638w, 1590w, 1455w, 1406w, 1383m, 1370m, 1311w, 1234s, 1196m, 1156m, 1109m, 1088m, 1066s, 1021s, 956w, 917w, 855, 763w, 532w, 460w.

11-Desnoviosyl-xy-4’’-O-cyclopropanoyl-thio-β-D-noviosyl fidaxomicin (4’’-O-cyclopropanoyl-S-Fdx, 3b)

11-Desnoviosyl-xy-4’’-*O*-cyclopropanoyl-thio-β-D-noviosyl fidaxomicin (**3b**) was synthesized after General procedure C from Fdx (**1**, 175 µmol, 185 mg, 1.0 equiv.), and (3aS,4S,7S,7aS)-4-mercapto-2,2,6,6-tetramethyltetrahydro-4H-[1,3]dioxolo[4,5-c]pyran-7-yl cyclopropanecarboxylate (**17b**, 175 µmol, 53 mg, 1.0 equiv.) in MeCN (1.67 mL, 0.1 M) using milliQ water (3.51 mmol, 63 µL, 20 equiv.) and $Cu[ClO_4]_2$•6 H_2O (10 mM solution in MeCN, 876 µL, 5 mol%). The reaction mixture was stirred at 25 °C for 20 h. Purification of the crude material after aqueous work-up and filtration was performed by preparative RP-HPLC using an isocratic eluent of 46% B (LC time program (time - %B): 0 min – 46%, 110 min - 46%). Product containing fractions (C11 isomer) were combined separately and repurified by preparative RP-HPLC using the same conditions. Product containing fractions were combined separately and concentrated *in vacuo* at 44 °C (water bath):

11-Desnoviosyl-11-4’’-*O*-cyclopropanoyl-thio-β-D-noviosyl fidaxomicin (**3b-C(11)**) was obtained as a colorless solid in a yield of 3% (4.5 µmol, 4.8 mg, t_R = 47.3 min).

11-Desnoviosyl-13-4’’-*O*-cyclopropanoyl-thio-β-D-noviosyl fidaxomicin (**3b-C(13)**) was obtained as a colorless solid in a yield of 6% (10.9 µmol, 11.7 mg, t_R = 54.2 min).

11-Desnoviosyl-15-4’’-*O*-cyclopropanoyl-thio-β-D-noviosyl fidaxomicin (**3b-C(15)**) was obtained as a colorless solid in a yield of 18% (32.0 µmol, 34.3 mg, t_R = 43.8 min).

11-Desnoviosyl-11-4’’-O-cyclopropanoyl-thio-β-D-noviosyl fidaxomicin (4’’-O-cyclopropanoyl-S-Fdx , 3b-C(11))

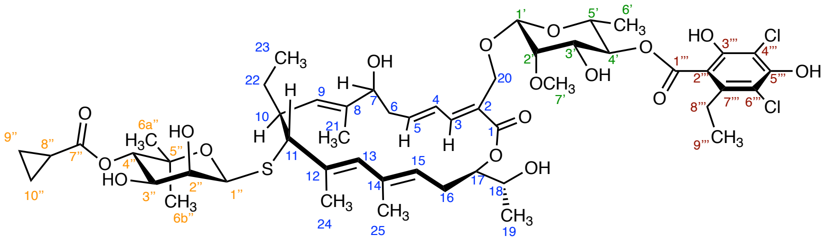

^{1}H NMR (500 MHz, acetone-d_6) δ (ppm) 7.28 (d, *J* = 11.5 Hz, 1H, H3), 6.69 – 6.59 (m, 1H, H4), 6.00 (ddd, *J* = 14.6, 9.7, 4.6 Hz, 1H, H5), 5.79 (s, 1H, H13), 5.63 (t, *J* = 8.2 Hz, 1H, H15), 5.32 (dt, *J* = 10.1, 1.6 Hz, 1H, H9), 5.10 (t, *J* = 9.7 Hz, 1H, H4’), 4.98 (d, *J* = 10.2 Hz, 1H, H4’’), 4.87 (d, *J* = 1.4 Hz, 1H, H1’’), 4.72 (dt, *J* = 6.9, 4.8 Hz, 1H, H17), 4.68 (s, 1H, H1’), 4.60 (d, *J* = 11.5 Hz, 1H, H20a), 4.42 (d, *J* = 11.5 Hz, 1H, H20b), 4.26 (t, *J* = 4.0 Hz, 1H, H7), 4.06 – 3.99 (m, 2H, H18, H2’’), 3.84 – 3.74 (m, 2H, H3’, H3’’), 3.67 – 3.57 (m, 2H, H2’, H5’), 3.52 (s, 3H, H7’), 3.44 (d, *J* = 10.9 Hz, 1H, H11), 3.01 (qd, *J* = 7.4, 2.0 Hz, 2H, H8’’’), 2.77 – 2.66 (m, 2H, H6a, H16a), 2.55 – 2.39 (m, 3H, H6b, H10, H16b), 2.02 – 1.95 (m, 1H, H22a), 1.87 (d, *J* = 1.1 Hz, 3H, H24), 1.72 (d, *J* = 1.3 Hz, 3H, H25), 1.65 (s, 3H, H21), 1.65 – 1.58 (m, 1H, H8’’), 1.31 (d, *J* = 6.2 Hz, 3H, H6’), 1.35 – 1.24 (m, 1H, H22b), 1.22 (t, *J* = 7.4 Hz, 3H, H9’’’), 1.18 (d, *J* = 6.2 Hz, 3H, H19), 1.17 (s, 3H, H6b’’), 1.08 (s, 3H, H6a’’), 0.91 – 0.85 (m, 4H, H9a’’, H9b’’, H10a’’, H10b’’), 0.82 (t, *J* = 7.4 Hz, 3H, H23). **^{13}C NMR** (126 MHz, acetone-d_6) δ (ppm) 174.8 (C7’’), 169.6 (C1’’’), 167.8 (C1), 156.1 (C3’’’), 154.4 (C5’’’), 145.6 (C3), 143.6 (C5), 142.6 (C7’’’), 136.6 (C12), 136.5 (C8), 136.1 (C14), 131.7 (C13), 128.2 (C4), 126.5 (C15), 126.1 (C9), 125.2 (C2), 114.8 (C6’’’), 110.1 (C2’’’), 108.2 (C4’’’), 101.7 (C1’), 81.7 (C2’), 81.4 (C1’’), 78.2 (C17), 77.6 (C4’), 76.5 (C5’’), 75.7 (C4’’), 74.9 (C2’’), 72.9 (C7), 72.3 (C3’), 70.7 (C5’), 70.4 (C3’’), 67.6 (C18), 63.3 (C20), 62.9 (C11), 61.7 (C7’), 41.4 (C10), 37.2

(C6), 28.5 (C6a”), 28.4 (C16), 27.7 (C22), 26.3 (C8’”), 20.8 (C19), 18.2 (C6’), 17.7 (C6b”), 17.6 (C25), 15.1 (C21), 14.4 (C9’”), 14.3 (C24), 13.5 (C8”), 10.8 (C23), 8.4 (C9” or C10”), 8.3 (C9” or C10”). **HRMS** ESI(+)(MeOH) calculated for $C_{52}H_{72}Cl_2O_{17}NaS^+$ $[M+Na]^+$: 1093.37595, found: 1093.37667. **FT-IR** (solid): ν (cm^{-1}) 3414w, 2976w, 2931w, 1700m, 1643m, 1589w, 1382m, 1313m, 1242m, 1199m, 1184m, 1111m, 1065s, 1021s, 933w, 897w, 853w, 799w, 762m, 737w, 692w, 526w, 509w.

11-Desnoviosyl-13-4”-O-cyclopropanoyl-thio-β-D-noviosyl fidaxomicin (3b-C(13))

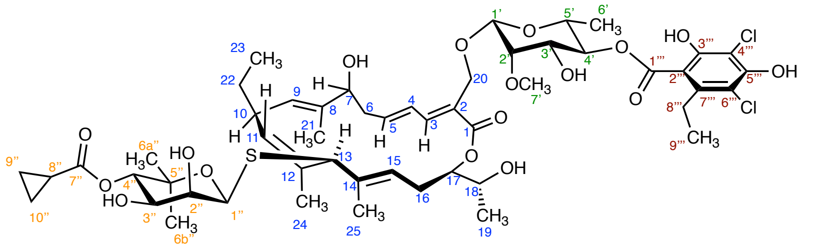

^{1}H NMR (500 MHz, acetone-d_6) δ (ppm) 7.20 (d, *J* = 11.4 Hz, 1H, H3), 6.60 (dd, *J* = 15.0, 11.4 Hz, 1H, H4), 6.06 (ddd, *J* = 15.0, 9.5, 5.6 Hz, 1H, H5), 5.64 – 5.58 (m, 1H, H15), 5.40 (dd, *J* = 7.8, 1.4 Hz, 1H, H11), 5.32 (dt, *J* = 10.0, 1.6 Hz, 1H, H9), 5.10 (t, *J* = 9.7 Hz, 1H, H4’), 5.05 (d, *J* = 10.2 Hz, 1H, H4”), 4.83 (dt, *J* = 9.0, 3.3 Hz, 1H, H17), 4.79 (d, *J* = 1.3 Hz, 1H, H1”), 4.62 – 4.59 (m, 2H, H20a, H1’), 4.49 (d, *J* = 11.5 Hz, 1H, H20b), 4.31 (d, *J* = 3.9 Hz, 1H, H7), 3.93 – 3.85 (m, 3H, H13, H18, H2”), 3.81 – 3.74 (m, 2H, H3’, H3”), 3.65 (dq, *J* = 9.6, 6.1 Hz, 1H, H5’), 3.57 (d, *J* = 3.4 Hz, 1H, H2’), 3.52 (s, 3H, H7’), 3.21 (dtd, *J* = 10.0, 7.9, 5.7 Hz, 1H, H10), 3.02 (q, *J* = 7.4 Hz, 2H, H8’”), 2.74 (dddd, *J* = 14.4, 5.4, 3.6, 1.6 Hz, 1H, H6a), 2.61 – 2.54 (m, 1H, H16a), 2.54 – 2.40 (m, 2H, H6b, H16b), 1.71 (d, *J* = 1.3 Hz, 3H, H21), 1.68 – 1.62 (m, 1H, H8”), 1.63 – 1.54 (m, 1H, H22a), 1.59 (d, *J* = 1.1 Hz, 3H, H24), 1.51 (s, 3H, H25), 1.42 – 1.34 (m, 1H, H22b), 1.31 (d, *J* = 6.1 Hz, 3H, H6’), 1.30 (s, 3H, H6b”), 1.23 (t, *J* = 7.3 Hz, 3H, H9’”), 1.15 (d, *J* = 6.1 Hz, 3H, H19), 1.13 (s, 3H, H6a”), 0.91 (t, *J* = 7.3 Hz, 3H, H23), 0.95 – 0.85 (m, 4H, H9a”, H9b”, H10a”, H10b”). **^{13}C NMR** (126 MHz, acetone-d_6) δ (ppm) 174.8 (C7”), 169.7 (C1’”), 166.7 (C1), 156.2 (C3’”), 154.3 (C5’”), 144.9 (C3), 143.4 (C5), 142.8 (C7’”), 137.5 (C14), 135.0 (C12), 134.7 (C11), 134.6 (C8), 127.9 (C4), 126.6 (C9), 125.3 (C2), 121.5 (C15), 114.7 (C6’”), 109.9 (C2’”), 108.1 (C4’”), 100.8 (C1’), 81.7 (C2’), 78.2 (C1”), 78.0 (C17), 77.6 (C4’), 76.5 (C5”), 75.6 (C4”), 74.2 (C2”), 72.7 (C7), 72.3 (C3’), 70.7 (C5’), 70.5 (C3”), 66.5 (C18), 62.7 (C20), 61.7 (C7’), 60.5 (C13), 39.1 (C10), 37.6 (C6), 30.2 (C22), 29.1 (C16), 28.6 (C6a”), 26.3 (C8’”), 21.2 (C19), 19.1 (C6b”), 18.4 (C6’), 17.2 (C25), 14.8 (C21), 14.4 (C9’”), 13.5 (C8”), 12.0 (C23), 12.0 (C24), 8.5 (C9” or C10”), 8.3 (C9” or C10”). **HRMS** ESI(+)(MeOH) calculated for $C_{52}H_{72}Cl_2O_{17}NaS^+$ $[M+Na]^+$: 1093.37595, found: 1093.37587. **Specific Rotation** $[\alpha]_D^{24°C}$ = –149.1 (c =0.62, MeOH). **FT-IR** (solid): ν (cm^{-1}) 3419w, 2979w, 2933w, 1699m, 1644w, 1590w, 1454w, 1383m, 1370m, 1312w, 1240m, 1199m, 1184m, 1110m, 1065s, 1022s, 933w, 900w, 858w, 828w, 798w, 762w, 738w, 691w, 426w.

11-Desnoviosyl-15-4''-O-cyclopropanoyl-thio-β-D-noviosyl fidaxomicin (3b-C(15))

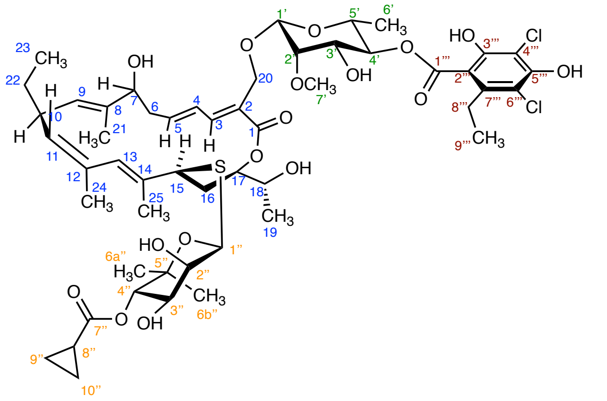

^{1}H NMR (500 MHz, acetone-d_6) δ (ppm) 7.15 (d, J = 11.3 Hz, 1H, H3), 6.32 (ddd, J = 15.2, 11.2, 1.3 Hz, 1H, H4), 6.20 (ddd, J = 15.1, 11.0, 4.0 Hz, 1H, H5), 5.63 (d, J = 1.5 Hz, 1H, H13), 5.19 (dt, J = 10.4, 1.4 Hz, 1H, H9), 5.18 – 5.11 (m, 1H, H17), 5.12 (t, J = 9.7 Hz, 1H, H4'), 5.05 (d, J = 9.5 Hz, 1H, H11), 5.02 (d, J = 10.2 Hz, 1H, H4''), 4.71 (d, J = 1.3 Hz, 1H, H1''), 4.67 (d, J = 0.8 Hz, 1H, H1'), 4.62 (d, J = 10.9 Hz, 1H, H20a), 4.45 (d, J = 10.8 Hz, 1H, H20b), 4.30 (s, 1H, H7), 3.86 – 3.79 (m, 3H, H18, H3', H2''), 3.70 (dd, J = 10.2, 3.5 Hz, 1H, H3''), 3.61 – 3.54 (m, 3H, H15, H2', H5'), 3.52 (s, 3H, H7'), 3.17 – 3.08 (m, 1H, H10), 3.05 – 2.94 (m, 2H, H8'''), 2.59 (ddd, J = 13.8, 6.1, 3.7 Hz, 1H, H6a), 2.37 (ddd, J = 13.6, 11.0, 2.6 Hz, 1H, H6b), 2.24 (dt, J = 14.7, 11.4 Hz, 1H, H16a), 1.91 (d, J = 1.1 Hz, 3H, H25), 1.83 (d, J = 1.2 Hz, 3H, H24), 1.80 (ddd, J = 14.6, 2.7, 1.6 Hz, 1H, H16b), 1.68 – 1.60 (m, 1H, H8''), 1.58 (d, J = 1.3 Hz, 3H, H21), 1.33 (d, J = 6.2 Hz, 3H, H6'), 1.32 – 1.23 (m, 2H, H22a, H22b), 1.22 (s, 3H, H6b''), 1.20 (t, J = 7.3 Hz, 3H, H9'''), 1.17 (d, J = 6.4 Hz, 3H, H19), 1.16 (s, 3H, H6a''), 0.91 – 0.84 (m, 4H, H9a'', H9b'', H10a'', H10b''), 0.80 (t, J = 7.4 Hz, 3H, H23). **^{13}C NMR** (126 MHz, acetone-d_6) δ (ppm) 174.8 (C7''), 169.4 (C1'''), 167.5 (C1), 155.8 (C3'''), 153.8 (C5'''), 144.9 (C3), 143.2 (C5), 142.6 (C7'''), 138.2 (C11), 134.1 (C8), 133.9 (C13), 132.7 (C14), 131.9 (C12), 127.2 (C4), 127.1 (C9), 125.2 (C2), 114.5 (C6'''), 110.7 (C2'''), 108.2 (C4'''), 100.1 (C1'), 81.7 (C2'), 78.1 (C17), 77.8 (C1''), 77.5 (C4'), 76.6 (C5''), 75.6 (C4''), 74.2 (C2''), 74.0 (C7), 72.3 (C3'), 70.8 (C5'), 70.5 (C3''), 69.7 (C18), 61.9 (C20), 61.8 (C7'), 53.6 (C15), 39.5 (C10), 38.4 (C6), 31.1 (C16), 29.8 (C22), 28.6 (C6a''), 26.2 (C8'''), 19.1 (C19), 18.8 (C6b''), 18.0 (C6'), 17.0 (C24), 15.5 (C21), 14.4 (C9'''), 13.6 (C25), 13.5 (C8''), 12.0 (C23), 8.5 (C9'' or C10''), 8.3 (C9'' or C10''). **HRMS** ESI(+)(MeOH) calculated for $C_{52}H_{72}Cl_2O_{17}NaS^+$ $[M+Na]^+$: 1093.37595, found: 1093.37527. **Specific Rotation** $[\alpha]_D^{24°C}$ = +3.5 (c = 0.46, MeOH). **FT-IR** (solid): ν (cm^{-1}) 3419w, 2978w, 2934w, 1712m, 1638m, 1590w, 1454w, 1383m, 1371m, 1311m, 1236m, 1199m, 1179m, 1109m, 1065s, 1022s, 933w, 916w, 898w, 854w, 762m, 737w, 691w, 431w, 411w.

11-Desnoviosyl-xy-4''-O-pivaloyl-thio-β-D-noviosyl fidaxomicin (4''-O-pivaloyl-S-Fdx, 3c)

11-Desnoviosyl-xy-4''-*O*-pivaloyl-thio-β-D-noviosyl fidaxomicin (**3c**) was synthesized after General procedure C from Fdx (**1**, 138 µmol, 146 mg, 1.0 equiv.), and (3aS,4S,7S,7aS)-4-mercapto-2,2,6,6-tetramethyltetrahydro-4H-[1,3]dioxolo[4,5-c]pyran-7-yl pivalate (**17c**, 138 µmol, 44 mg, 1.0 equiv.) in MeCN (1.31 mL, 0.1 M) using milliQ water (2.76 mmol, 50 µL, 20.0 equiv.) and $Cu[ClO_4]_2$•6 H_2O (10 mM solution in MeCN, 691 µL, 5 mol%). The reaction mixture was stirred at 25 °C for 14 h. Purification of the crude material after aqueous work-up and filtration was performed by preparative RP-HPLC using an isocratic eluent of 50% B (LC time program (time - %B): 0 min - 50%, 110 min – 50%). Product containing fractions (C11 and C15 isomer mixture) were combined separately and repurified by preparative RP-HPLC using the same conditions. Product containing fractions were combined separately and concentrated *in vacuo* at 44 °C (water bath):

11-Desnoviosyl-11-4''-*O*-pivaloyl-thio-β-D-noviosyl fidaxomicin (**3c-C(11)**) was obtained as a colorless solid in a yield of 1% (1.8 µmol, 1.9 mg, t_R = 50.0 min).

11-Desnoviosyl-13-4''-*O*- pivaloyl-thio-β-D-noviosyl fidaxomicin (**3c-C(13)**) was obtained as a colorless solid in a yield of 3% (4.5 µmol, 4.9 mg, t_R = 60.5 min).

11-Desnoviosyl-15-4''-*O*- pivaloyl thio-β-D-noviosyl fidaxomicin (**3c-C(15)**) was obtained as a colorless solid in a yield of 40% (55.2 µmol, 60.1 mg, t_R = 47.6 min).

11-Desnoviosyl-11-4''-O-pivaloyl-thio-β-D-noviosyl fidaxomicin (4''-O-pivaloyl-S-Fdx, 3c-C(11))

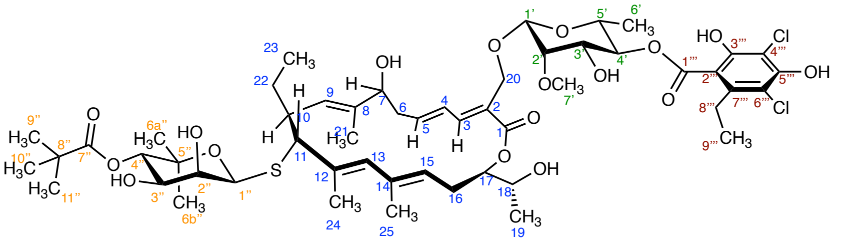

^{1}H NMR (500 MHz, acetone-d_6) δ (ppm) 7.28 (d, J = 11.4 Hz, 1H, H3), 6.69 – 6.60 (m, 1H, H4), 6.00 (ddd, J = 14.6, 9.7, 4.6 Hz, 1H, H5), 5.81 – 5.76 (m, 1H, H13), 5.66 – 5.60 (m, 1H, H15), 5.32 (dt, J = 10.3, 1.6 Hz, 1H, H9), 5.10 (t, J = 9.7 Hz, 1H, H4'), 4.95 (d, J = 10.2 Hz, 1H, H4''), 4.87 (d, J = 1.4 Hz, 1H, H1''), 4.72 (dt, J = 6.7, 4.8 Hz, 1H, H17), 4.68 (d, J = 0.8 Hz, 1H, H1'), 4.60 (d, J = 11.5 Hz, 1H, H20a), 4.42 (d, J = 11.5 Hz, 1H, H20b), 4.26 (s, 1H, H7), 4.07 – 3.99 (m, 2H, H18, H2''), 3.80 (dd, J = 9.9, 3.4 Hz, 1H, H3'), 3.77 (dd, J = 10.2, 3.5 Hz, 1H, H3''), 3.66 – 3.58 (m, 2H, H2', H5'), 3.52 (s, 3H, H7'), 3.44 (d, J = 10.9 Hz, 1H, H11), 3.01 (qd, J = 7.5, 2.0 Hz, 2H, H8'''), 2.77 – 2.66 (m, 2H, H6a, H16a), 2.55 – 2.39 (m, 3H, H6b, H10, H16b), 2.01 – 1.95 (m, 1H, H22a), 1.87 (d, J = 1.2 Hz, 3H, H24), 1.72 (d, J = 1.4 Hz, 3H, H25), 1.65 (d, J = 0.9 Hz, 3H, H21), 1.31 (d, J = 6.2 Hz, 3H, H6'), 1.32 – 1.27 (m, 1H, H22b), 1.22 (t, J = 7.4 Hz, 3H, H9'''), 1.19 (s, 9H, H9'', H10'', H11''), 1.19 – 1.17 (m, 6H, H19, H6b''), 1.08 (s, 3H, H6a''), 0.82 (t, J = 7.4 Hz, 3H, H23). **^{13}C NMR** (126 MHz, acetone-d_6) δ (ppm) 178.1 (C7''), 169.5 (C1'''), 167.8 (C1), 155.9 (C3'''), 153.9 (C5'''), 145.6 (C3), 143.6 (C5), 142.7 (C7'''), 136.6 (C12), 136.5 (C8), 136.1 (C14), 131.7 (C13), 128.2 (C4), 126.5 (C15), 126.1 (C9), 125.2 (C2), 114.5 (C6'''), 110.6 (C2'''), 108.2 (C4'''), 101.7 (C1'), 81.7 (C2'), 81.5 (C1''), 78.2 (C17), 77.6 (C4'), 76.5 (C5''), 75.4 (C4''), 75.0 (C2''), 73.0 (C7), 72.3 (C3'), 70.6 (C5'), 70.5 (C3''), 67.7 (C18), 63.3 (C20), 62.9 (C11), 61.7 (C7'), 41.4 (C10), 39.4 (C8''), 37.2 (C6), 28.5 (C6a''), 28.4 (C16), 27.7 (C22), 27.4 (C9'', C10'', C11''), 26.2 (C8'''), 20.8 (C19), 18.2 (C6'), 17.7 (C6b''), 17.6 (C25), 15.1 (C21), 14.4 (C9'''), 14.3 (C24), 10.8 (C23). **HRMS** ESI(+)(MeOH) calculated for $C_{53}H_{76}O_{17}Cl_2NaS^+$ $[M+Na]^+$: 1109.40725, found: 1109.40652. **FT-IR** (solid): ν (cm^{-1}) 3426w, 2963w, 2918w, 2875w, 2850w, 1705w, 1643w, 1590w, 1457w, 1398w, 1382w, 1370w, 1312w, 1284w, 1259m, 1214m, 1184m, 1160m, 1088m, 1064s, 1017s, 861w, 902w, 861w, 797s, 767m, 737w, 689w.

11-Desnoviosyl-13-4''-O-pivaloyl-thio-β-D-noviosyl fidaxomicin (3c-C(13))

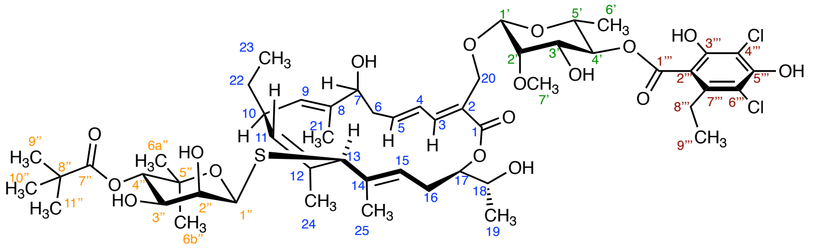

^{1}H NMR (500 MHz, acetone-d_6) δ (ppm) 7.23 – 7.17 (m, 1H, H3), 6.60 (dd, J = 15.2, 11.4 Hz, 1H, H4), 6.06 (ddd, J = 15.0, 9.5, 5.6 Hz, 1H, H5), 5.65 – 5.58 (m, 1H, H15), 5.41 (dd, J = 7.8, 1.4 Hz, 1H, H11), 5.32 (dt, J = 10.0, 1.6 Hz, 1H, H9), 5.10 (t, J = 9.7 Hz, 1H, H4'), 5.03 (d, J = 10.2 Hz, 1H, H4''), 4.83 (dt, J = 9.0, 3.2 Hz, 1H, H17), 4.80 (d, J = 1.3 Hz, 1H, H1''), 4.61 (s, 1H, H1'), 4.61 (d, J = 11.5 Hz, 1H, H20a), 4.49 (d, J = 11.6 Hz, 1H, H20b), 4.31 (s, 1H, H7), 3.93 – 3.86 (m, 3H, H13, H18, H2''), 3.81 – 3.75 (m, 2H, H3'', H3'), 3.69 – 3.61 (m, 1H, H5'), 3.57 (d, J = 3.4 Hz, 1H, H2'), 3.52 (s, 3H, H7'), 3.25 – 3.17 (m, 1H, H10), 3.02 (q, J = 7.4 Hz, 2H, H8'''), 2.74 (dddd, J = 14.4, 5.5, 3.7, 1.6 Hz, 1H, H6a), 2.60 – 2.55 (m, 1H, H16a), 2.54 – 2.42 (m, 2H, H6b, H16b), 1.71 (s, 1H, H21), 1.63 – 1.54 (m, 1H, H22a), 1.59 (s, 3H, H24), 1.51 (s, 3H, H25), 1.41 – 1.33 (m, 1H, H22b), 1.32 – 1.30 (m, 6H, H6', H6b''), 1.23 (t, J = 7.4 Hz, 3H, H9'''), 1.20 (s, 9H, H9'', H10'', H11''), 1.16 (d, J = 6.1 Hz, 3H, H19), 1.13 (s, 3H, H6a''), 0.91 (t, J = 7.4 Hz, 3H, H23). **^{13}C NMR** (126 MHz, acetone-d_6) δ (ppm) 178.0 (C7''), 169.7 (C1'''), 166.6 (C1), 156.3 (C3'''), 154.2 (C5'''), 144.9 (C3), 143.4 (C5), 142.8

(C7’’’), 137.5 (C14), 135.0 (C12), 134.7 (C11), 134.6 (C8), 128.0 (C4), 126.6 (C9), 125.3 (C2), 121.5 (C15), 114.7 (C6’’’), 110.0 (C2’’’), 108.2 (C4’’’), 100.8 (C1’), 81.7 (C2’), 78.2 (C1’’), 78.0 (C17), 77.6 (C4’), 76.6 (C5’’), 75.4 (C4’’), 74.4 (C2’’), 72.8 (C7), 72.4 (C3’), 70.7 (C5’), 70.7 (C3’’), 66.6 (C18), 62.7 (C20), 61.7 (C7’), 60.4 (C13), 39.4 (C8’’), 39.1 (C10), 37.6 (C6), 30.2 (C22), 29.1 (C16), 28.6 (C6a’’), 27.4 (C9’’, C10’’, C11’’), 26.3 (C8’’’), 21.2 (C19), 19.2 (C6b’’), 18.4 (C6’), 17.2 (C25), 14.8 (C21), 14.4 (C9’’’), 12.0 (C23), 12.0 (C24). **HRMS** ESI(+)(MeOH) calculated for $C_{53}H_{76}O_{17}Cl_2NaS^+$ $[M+Na]^+$: 1109.40725, found: 1109.40614. **Specific Rotation** $[\alpha]_D^{24°C}$ = –149.2 (c = 0.48, MeOH). **FT-IR** (solid): ν (cm^{-1}) 3417w, 2976w, 2934w, 2875w, 1700m, 1644w, 1591w, 1479w, 1456w, 1370m, 1312m, 1286m, 1240m, 1183m, 1161m, 1111m, 1066s, 1021s, 957w, 904w, 859w, 799w, 785w, 763w, 737w, 691w, 475w, 453w, 421w.

11-Desnoviosyl-15-4’’-O-pivaloyl-thio-β-D-noviosyl fidaxomicin (3c-C(15))

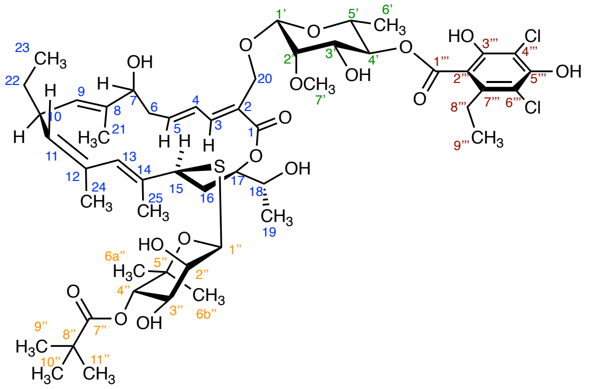

^{1}H NMR (500 MHz, acetone-d_6) δ (ppm) 7.16 (d, J = 11.3 Hz, 1H, H3), 6.32 (ddd, J = 15.1, 11.3, 1.3 Hz, 1H, H4), 6.20 (ddd, J = 15.0, 10.9, 4.1 Hz, 1H, H5), 5.64 (s, 1H, H13), 5.19 (dt, J = 10.5, 1.4 Hz, 1H, H9), 5.18 – 5.10 (m, 1H, H17), 5.12 (t, J = 9.8 Hz, 1H, H4’), 5.05 (d, J = 9.4 Hz, 1H, H11), 5.00 (d, J = 10.2 Hz, 1H, H4’’), 4.72 (d, J = 1.4 Hz, 1H, H1’’), 4.67 (s, 1H, H1’), 4.62 (d, J = 10.9 Hz, 1H, H20a), 4.45 (d, J = 10.8 Hz, 1H, H20b), 4.31 (t, J = 3.5 Hz, 1H, H7), 3.87 – 3.78 (m, 3H, H18, H3’, H2’’), 3.70 (dd, J = 10.2, 3.6 Hz, 1H, H3’’), 3.62 – 3.54 (m, 3H, H15, H2’, H5’), 3.52 (s, 3H, H7’), 3.12 (ddd, J = 10.1, 6.9, 3.0 Hz, 1H, H10), 2.99 (qd, J = 7.3, 1.5 Hz, 2H, H8’’’), 2.63 – 2.55 (m, 1H, H6a), 2.37 (ddd, J = 13.6, 11.0, 2.7 Hz, 1H, H6b), 2.24 (dt, J = 14.7, 11.4 Hz, 1H, H16a), 1.92 (d, J = 1.0 Hz, 3H, H25), 1.84 (d, J = 1.2 Hz, 3H, H24), 1.83 – 1.79 (m, 1H, H16b), 1.58 (d, J = 1.4 Hz, 3H, H21), 1.33 (d, J = 6.2 Hz, 3H, H6’), 1.31 – 1.24 (m, 2H, H22a, H22b), 1.22 (s, 3H, H6b’’), 1.21 – 1.18 (m, 3H, H9’’’), 1.19 (s, 9H, H9’’, H10’’, H11’’), 1.17 (d, J = 6.4 Hz, 3H, H19), 1.16 (s, 3H, H6a’’), 0.79 (t, J = 7.3 Hz, 3H, H23). **^{13}C NMR** (126 MHz, acetone-d_6) δ (ppm) 178.0 (C7’’), 169.4 (C1’’’), 167.5 (C1), 155.9 (C3’’’), 154.0 (C5’’’), 144.8 (C3), 143.2 (C5), 142.6 (C7’’’), 138.2 (C11), 134.1 (C8), 133.9 (C13), 132.7 (C14), 132.0 (C12), 127.2 (C4), 127.1 (C9), 125.2 (C2), 114.6 (C6’’’), 110.5 (C2’’’), 108.2 (C4’’’), 100.1 (C1’), 81.7 (C2’), 78.1 (C17), 77.9 (C1’’), 77.5 (C4’), 76.6 (C5’’), 75.3 (C4’’), 74.2 (C2’’), 73.9 (C7), 72.3 (C3’), 70.8 (C5’), 70.7 (C3’’), 69.7 (C18), 61.9 (C20), 61.8 (C7’), 53.7 (C15), 39.5 (C10), 39.3 (C8’’), 38.4 (C6), 31.1 (C16), 29.8 (C22), 28.6 (C6a’’), 27.4 (C9’’, C10’’, C11’’), 26.2 (C8’’’), 19.1 (C19), 18.9 (C6b’’), 18.0 (C6’), 17.0 (C24), 15.5 (C21), 14.4 (C9’’’), 13.6 (C25), 11.9 (C23). **HRMS** ESI(+)(MeOH) calculated for $C_{53}H_{76}O_{17}Cl_2NaS^+$ $[M+Na]^+$: 1109.40725, found: 1109.40629. **Specific Rotation** $[\alpha]_D^{25°C}$ = +8.7 (c = 0.47, MeOH). **FT-IR** (solid): ν (cm^{-1}) 3425w, 2977w, 2934w, 2874w, 1708m, 1639m, 1590w, 1479w, 1456w, 1399m, 1369m, 1311m, 1286m, 1234m, 1181m, 1158m, 1109m, 1090m, 1066s, 1022s, 956m, 914w, 856w, 783w, 762m, 736w, 690w, 531w, 422w.

Isolation and functionalization of noviose

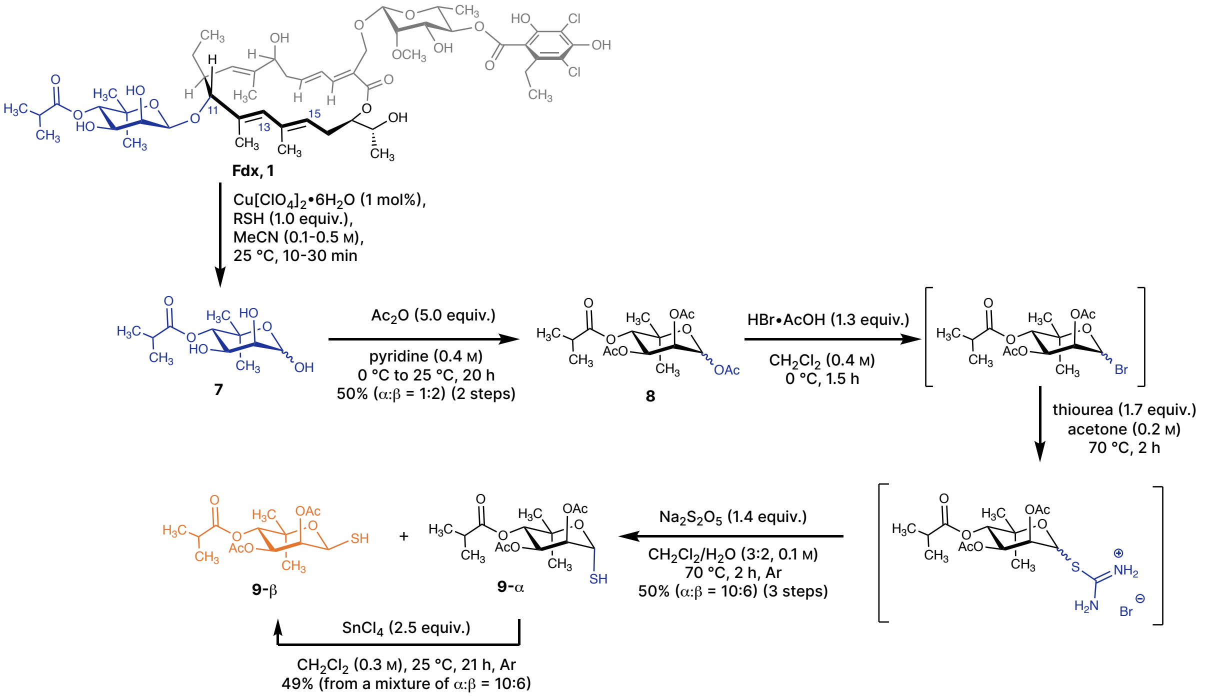

Figure 2: Overall scheme for the isolation and functionalization of the noviose by-product

Isolation and acetylation of noviose-derived sugar in pyridine

The crude 4-*O*-demethyl-4-*O*-isobutyryl-D-noviose by-product obtained from the aqueous phase of various reactions of Fdx with different thiol nucleophiles performed analogous to General procedure B (first reaction step) with minor modifications (MeCN (0.1–0.5 M), 10–30 min) were combined and used in the acetylation procedure performed after Doyle *et al.*[10]:
The crude 4-*O*-demethyl-4-*O*-isobutyryl-D-noviose (**7**, 400 mg, 1.61 mmol, 1.0 equiv., derived from 2.4 g Fdx with a yield of 71%) was dissolved in anhydrous pyridine (4.3 mL, 0.4 M), and the solution cooled to 0 °C and sparged with Ar for 10 min. At 0 °C, acetic anhydride (756 µL, 8.05 mmol, 5.0 equiv.) was added dropwise over 30 min. The mixture was allowed to slowly warm to 25 °C while stirring overnight. After 20 h, and complete conversion of the starting material, the reaction mixture was poured into ice-water (30 mL) and CH_2Cl_2 (30 mL) was added. The layers were separated, and the aqueous phase was extracted with CH_2Cl_2 (2×30 mL). The combined organic phase was washed with 1 M aqueous HCl solution (2×90 mL), saturated aqueous $NaHCO_3$ solution (1×90 mL) and water (1×90 mL). The organic phase was then dried over anhydrous $MgSO_4$, filtered, and concentrated *in vacuo* to provide the crude material as a brown oil. The crude material was purified *via* column chromatography on silica using an eluent of EtOAc/pentane = 2:8 to provide the α epimer (70.1 mg, 0.187 mmol) and β epimer (238.9 mg, 0.638 mmol) of the product **8** as colorless oils in a yield of 12% (α, 8% over 2 steps) and 40% (β, 28% over 2 steps), respectively.
Note: The isolation and acetylation in pyridine were repeated three times and the mixture of epimers (α:β = 1:2) was obtained as a colorless oil in a yield of 72% (average of three reactions, 50% over 2 steps) and used in the next step without separation of the epimers.

1,2,3-tri-O-acetyl-4-O-demethyl-4-O-isobutyryl-α-D-noviose (8-α)

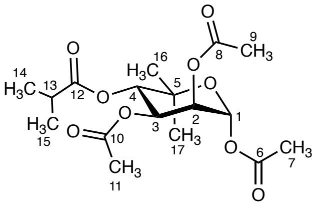

^{1}H NMR (acetone-*d*$_6$, 500 MHz): δ (ppm) 6.01 (d, *J*=2.4 Hz, 1H, H1), 5.43 (dd, *J*=10.3, 3.4 Hz, 1H, H3), 5.27 (d, *J*=10.3 Hz, 1H, H4), 5.22 (dd, *J*=3.4, 2.4 Hz, 1H, H2), 2.61 (hept, *J*=7.0 Hz, 1H, H13), 2.12 (s, 3H, H9), 2.12 (s, 3H, H7), 1.97 (s, 3H, H11), 1.39 (s, 3H, H17), 1.23 (s, 3H, H16), 1.15 (d, *J*=1.4 Hz, 3H, H14 or H15), 1.14 (d, *J*=1.5 Hz, 3H, H14 or H15). **^{13}C NMR** (126 MHz, acetone-*d*$_6$) δ (ppm) 176.1 (C12), 170.3 (C10), 170.3 (C8), 169.1 (C6), 91.7 (C1), 78.3 (C5), 71.6 (C4), 70.0 (C2), 67.2 (C3), 34.7 (C13), 28.6 (C16), 23.9 (C17), 21.1 (C7), 20.7 (C9), 20.6 (C11), 19.2 (C14 or C15), 19.0 (C14 or C15). **HRMS** ESI(+) (MeOH/$CHCl_3$ 3:2) calculated for $C_{17}H_{26}O_9Na^+$ [M+Na]$^+$: 397.14690, found: 397.14653. **R*f*** (pentane/EtOAc = 8:2) = 0.5. **Specific Rotation** $[\alpha]_D^{24°C}$ = +26.1 (c = 1.1, MeOH). **FT-IR** (acetone-*d*$_6$): ν (cm^{-1}) 2982w, 2939w, 1753s, 1471w, 1434w, 1372m, 1298w, 1225s, 1148s, 1073m, 1051m, 1022m, 964m, 912w, 860w, 790w, 762w, 693w, 601w, 564w, 514w.

1,2,3-tri-O-acetyl-4-O-demethyl-4-O-isobutyryl-β-D-noviose (8-β)

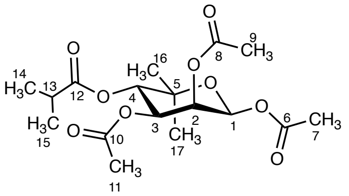

^{1}H NMR (acetone-*d*$_6$, 500 MHz): δ (ppm) 6.14 (d, *J*=1.5 Hz, 1H, H1), 5.44 (dd, *J*=3.4, 1.5 Hz, 1H, H2), 5.31 (dd, *J*=10.5, 3.4 Hz, 1H, H3), 5.18 (d, *J*=10.5 Hz, 1H, H4), 2.59 (hept, *J*=6.9 Hz, 1H, H13), 2.13 (s, 3H, H9), 2.02 (s, 3H, H7), 1.93 (s, 3H, H11), 1.42 (s, 3H, H17), 1.22 (s, 3H, H16), 1.15 – 1.11 (m, 6H, H14, H15). **^{13}C NMR** (126 MHz, acetone-*d*$_6$) δ (ppm) 176.2 (C12), 170.6 (C8), 170.0 (C10), 169.0 (C6), 87.9 (C1), 75.8 (C5), 71.8 (C4), 69.7 (C2), 69.3 (C3), 34.7 (C13), 28.3 (C16), 20.7 (C7), 20.6 (C9), 20.5 (C11), 19.2 (C14 or C15), 19.1 (C14 or C15), 19.0 (C17). **HRMS** ESI(+) (MeOH/$CHCl_3$ 3:2) calculated for $C_{17}H_{26}O_9Na^+$ [M+Na]$^+$: 397.14690, found: 397.14645. **R*f*** (pentane/EtOAc = 8:2) = 0.4. **FT-IR** (acetone-*d*$_6$): ν (cm^{-1}) 2982w, 2940w, 2880w, 1750s, 1471w, 1434w, 1371m, 1225s, 1168m, 1147m, 1090m, 1070m, 1041m, 971w, 951w, 918w, 880w, 754w, 602w, 518w.

3-step-protocoll for the synthesis of 2,3-di-O-acetyl4-O-demethyl-4-O-isobutyryl-1-thio-D-noviose (9): Mixture of anomers

The procedure was adapted from Doyle *et al.* with minor modifications.[10] A solution of 1,2,3-tri-*O*-acetyl-4-*O*-demethyl-4-*O*-isobutyryl-D-noviose **8** (390 mg, 1.04 mmol, 1.0 equiv., mixture of epimers (α:β = 1:2)) in anhydrous CH_2Cl_2 (2.7 mL, 0.4 M), was sparged with Ar for 2 min and then cooled to 0 °C. At 0 °C in the dark, HBr (33% in AcOH, 224 µL, 1.35 mmol, 1.3 equiv.) was added and the solution stirred at 0 °C for 1.5 h. The reaction was monitored *via* UHPLC-MS analysis. After full conversion of the starting material, the solvent was removed under reduced pressure (high vacuum) at 25 °C to provide a pale orange oil. Due to the limited stability, the crude glycosyl bromide was used immediately in the next step without purification.
The freshly prepared 2,3-di-*O*-acetyl-4-*O*-isobutyryl-D-noviopyranosyl bromide was dissolved in anhydrous acetone (6.6 mL). Thiourea (134 mg, 1.76 mmol, 1.7 equiv.) was added, and the mixture was sparged with Ar for 2 min, before it was heated to reflux (70 °C) and stirred under reflux for 2 h. After 2 h, the mixture was cooled to 25 °C and the solvent evaporated *in vacuo* at 40 °C water bath temperature to provide the crude thiouronium salt as a colorless solid (540 mg). The salt was used in the next step without purification.
The crude salt was suspended in a mixture of CH_2Cl_2 (6.6 mL) and water (4.3 mL, freshly degassed by sparging with Ar), and sodium metabisulfite (277 mg, 1.46 mmol, 1.4 equiv.) was added. The mixture was sparged with Ar for 5 min and then heated to reflux (70 °C) and stirred under reflux for 2 h. Then, the mixture was cooled to 25 °C, and the layers were separated. The aqueous phase was extracted with CH_2Cl_2 (3×15 mL), and the combined organic phase was washed with water (1×45 mL), dried over anhydrous $MgSO_4$, filtered, and the solvent removed

under reduced pressure at 40°C to provide the crude material as a colorless oil (298 mg). The crude was purified *via* column chromatography on silica (Silica gel 60, 0.015-0.040 mm) using an eluent of EtOAc/pentane = 15:85 to provide the product **9** as a mixture of epimers (α:β =1:0.6) as a colorless oil in a yield of 50% (181 mg, 0.52 mmol).
Note: For operational simplicity, the mixture of epimers with very similar R_f values was subjected to the epimerization step.

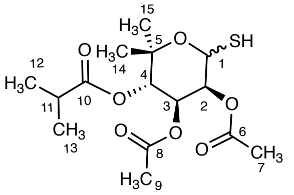

^{1}H NMR (acetone-d_6, 500 MHz): δ (ppm) 5.36 (dd, *J*=10.2, 1.5 Hz, 0.6H, H1β), 5.33 (dd, *J*=3.5, 1.5 Hz, 0.6H, H2β), 5.27 (dd, *J*=5.1, 3.4 Hz, 1H, H3α), 5.24 (t, *J*=8.1 Hz, 1H, H1α), 5.22 (dd, *J*=10.7, 3.5 Hz, 0.6H, H3β), 5.11 (d, *J*=10.8 Hz, 0.6H, H4β), 5.00 (dd, *J*=8.1, 3.4 Hz, 1H, H2α), 4.92 (d, *J*=5.0 Hz, 1H, H4α), 2.86 (d, *J*=10.2 Hz, 0.6H, SHβ), 2.72 (d, *J*=8.1 Hz, 1H, SHα), 2.66 (hept, *J*=7.1 Hz, 1H, H11α), 2.57 (hept, *J*=7.0 Hz, 0.6H, H11β), 2.19 (s, 1.8H, H7β), 2.10 (s, 3H, H9α), 2.00 (s, 3H, H7α), 1.89 (s, 1.8H, H9β), 1.47 (s, 3H, H14α), 1.38 (s, 1.8H, H15β), 1.25 (s, 3H, H15α), 1.19 (d, *J*=7.1 Hz, 3H, H12α or H13α), 1.18 (d, *J*=7.0 Hz, 3H, H12α or H13α), 1.18 (s, 1.8H, H14β), 1.15 – 1.08 (m, 3.6H, H12β, H13β). **^{13}C NMR** (126 MHz, acetone-d_6) δ (ppm) 176.2 (C10β), 175.8 (C10α), 170.8 (C6β), 170.1 (C8β), 170.0 (C6α), 169.9 (C8α), 77.5 (C5α), 77.5 (C5β), 73.6 (C2β), 72.8 (C4α), 72.4 (C2α), 72.4 (C1α), 71.8 (C1β), 71.4 (C4β), 70.2 (C3β), 68.3 (C3α), 34.7 (C11β), 34.7 (C11α), 28.4 (C14β), 26.0 (C15α), 24.3 (C14α), 20.7 (C9α), 20.7 (C7α), 20.5 (C9β), 20.5 (C7β), 19.3 (C12α or C13α), 19.2 (C12β or C13β), 19.1 (C12α or C13α), 19.1 (C12β or C13β), 18.3 (C15β). **HRMS** ESI(+) (MeOH/$CHCl_3$ 3:2) calculated for $C_{15}H_{24}O_7NaS^+$ $[M+Na]^+$: 371.11349, found: 371.11357. **R_f** (pentane/EtOAc = 85:15) = 0.3. **Specific Rotation** $[\alpha]_D^{24°C}$ = – 6.2 (c = 1.6, MeOH). **FT-IR** (acetone-d6): ν (cm-1) 2979w, 2938w, 2878w, 1748s, 1470w, 1434w, 1387w, 1371w, 1234s, 1219s, 1187w, 1149m, 1113w, 1067m, 976w, 926w.

Epimerization at the anomeric position of 1-thionoviose derivative 9

2,3-Di-O-acetyl-4-O-demethyl-4-O-isobutyryl-1-thio-β-D-noviose (9-β)

The procedure was adapted from Doyle *et al.* with minor modifications.[10] 2,3-di-*O*-acetyl-4-*O*-demethyl-4-*O*-isobutyryl-1-thio-D-noviose **9** (mixture of epimers (α:β =1:0.6), 245 mg, 0.70 mmol, 1.0 equiv.) was dissolved in anhydrous CH_2Cl_2 (2.7 mL, 0.3 M), and the solution sparged with Ar for 1 min. $SnCl_4$ (1 M solution in CH_2Cl_2, 1.8 mL, 1.76 mmol, 2.5 equiv.) was added dropwise at 25 °C and the reaction was stirred at 25 °C. After 21 h, the mixture was diluted with EtOAc (50 mL) and quenched with 1 M aqueous $KHSO_4$ solution (50 mL). The phases were separated, the aqueous phase extracted with EtOAc (3×50 mL), and the combined organic phase washed with saturated aqueous $NaHCO_3$ solution (2×150 mL) and brine (1×150 mL). The organic phase was then dried over anhydrous $MgSO_4$, filtered, and concentrated *in vacuo* to provide the crude product as an orange oil. The crude was purified *via* column chromatography on silica (Silica gel 60, 0.015-0.040 mm) using an eluent of EtOAc/pentane = 1:9. The major product, the **9** β epimer, was obtained as a colorless oil in a yield of 49% (121 mg, 0.347 mmol).
The **9** α epimer was obtained as a colorless oil in a yield of 10% (25 mg, 0.07 mmol) and a purity of approx. 85% (determined based on the integrals of the H4 signal of the α and the contaminating β epimer in the ^{1}H NMR spectrum).

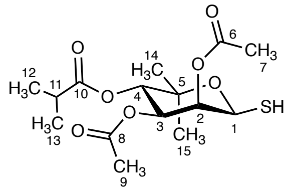

^{1}H NMR (500 MHz, acetone-d_6): δ (ppm) 5.36 (dd, *J*=10.2, 1.5 Hz, 1H, H1), 5.33 (dd, *J*=3.6, 1.4 Hz, 1H, H2), 5.22 (dd, *J*=10.7, 3.5 Hz, 1H, H3), 5.11 (d, *J*=10.8 Hz, 1H, H4), 2.86 (d, *J*=10.1 Hz, 1H, SH), 2.57 (hept, *J*=7.0 Hz, 1H, H11), 2.19 (s, 3H, H7), 1.89 (s, 3H, H9), 1.38 (s, 3H, H15), 1.18 (s, 3H, H14), 1.14 – 1.09 (m, 6H, H12, H13) **^{13}C NMR** (126 MHz, acetone-d_6) δ (ppm) 176.2 (C10), 170.8 (C6), 170.1 (C8), 77.5 (C5), 73.6 (C2), 71.8 (C1), 71.4 (C4), 70.2 (C3), 34.7 (C11), 28.4 (C14), 20.5 (C9), 20.5 (C7), 19.2 (C12 or C13), 19.1 (C12 or C13), 18.3 (C15). **HRMS** ESI(+) (MeOH/$CHCl_3$ 3:2) calculated for $C_{15}H_{24}O_7NaS^+$ $[M+Na]^+$: 371.11349, found: 371.11381. **R*f*** (EtOAc/pentane = 15:85) = 0.3. **Specific Rotation** $[\alpha]_D^{24°C} = -$ 65.3 (c = 0.4, MeOH). **FT-IR** (acetone-*d6*): ν (cm^{-1}) 2979w, 2938w, 2879w, 1742s, 1470w, 1433w, 1386w, 1370m, 1259m, 1231s, 1217s, 1186m, 1147m, 1106m, 1066s, 1039m, 974m, 935w, 923w, 908w, 863w, 842w, 827w, 801w, 779w, 710w, 672w, 629w, 601w, 593w, 523w, 486w.

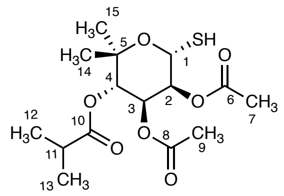

^{1}H NMR (500 MHz, acetone-d_6): δ (ppm) 5.27 (dd, *J*=5.3, 3.9 Hz, 1H, H3), 5.24 (t, *J*=8.2 Hz, 1H, H1), 5.00 (dd, *J*=8.1, 3.5 Hz, 1H, H2), 4.92 (d, *J*=5.0 Hz, 1H, H4), 2.72 (d, *J*=8.1 Hz, 1H, SH), 2.66 (hept, *J*=7.0 Hz, 1H, H11), 2.10 (s, 3H, H9), 2.00 (s, 3H, H7), 1.47 (s, 3H, H14), 1.25 (s, 3H, H15), 1.19 (d, *J*=7.0 Hz, 3H, H12 or H13), 1.18 (d, *J*=7.0 Hz, 3H, H12 or H13). **^{13}C NMR** (126 MHz, acetone-d_6) δ (ppm) 175.8 (C10), 170.0 (C6), 169.9 (C8), 77.5 (C5), 72.8 (C4), 72.4 (C1, C2), 68.3 (C3), 34.7 (C11), 26.0 (C15), 24.3 (C14), 20.7 (C9), 20.7 (C7), 19.3 (C12 or C13), 19.1 (C12 or C13). **R*f*** (EtOAc/pentane = 15:85) = 0.3. **FT-IR** (acetone-*d6*): ν (cm^{-1}) 2978w, 2938w, 2878w, 1746s, 1470w, 1434w, 1387w, 1370m, 1232s, 1216s, 1148m, 1116m, 1062m, 1023m, 987w, 975w, 929w, 894w, 879w, 857w, 802w, 760w, 710w, 626w, 601w, 536w.
Note: The high $^3J_{H1-H2}$ coupling constant suggests the structure to exist predominantly as the $^1C_4$ chair in solution.

De novo synthesis of thionoviose derivatives

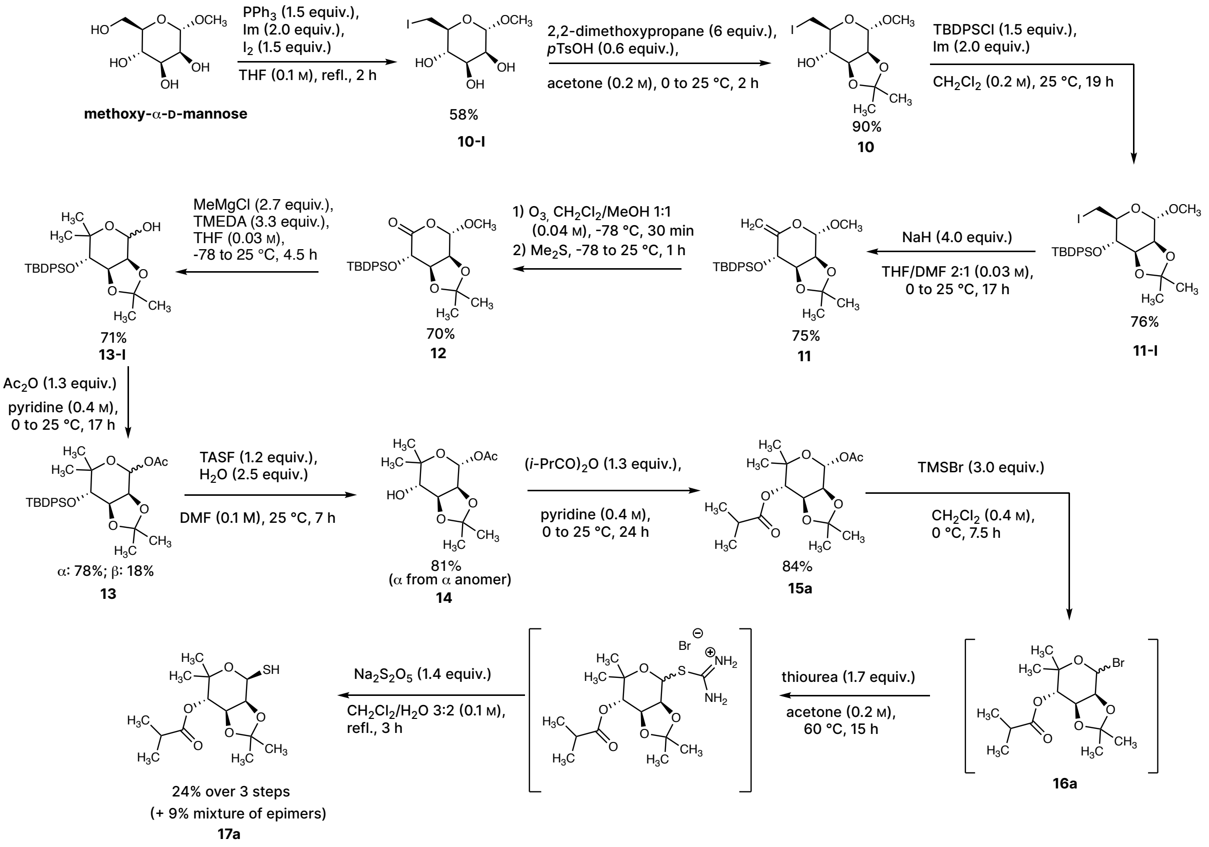

Figure 3: Overall scheme for the *de novo* synthesis of 2,3-*O*-acetonide protected 4-*O*-demethyl-4-*O*-isobutyryl thionoviose 17a

Methyl 6-deoxy-6-iodo-α-D-mannopyranoside (10-I)[11–13]

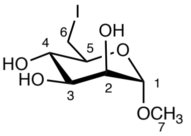

Methyl 6-deoxy-6-iodo-α-D-mannopyranoside **10-I** was synthesized after Skaanderup *et al.*[12] and Ionescu *et al.*[11] from methyl α-D-mannopyranoside (10.0 g, 51.5 mmol, 1.0 equiv.), imidazole (7.0 g, 103 mmol, 2.0 equiv.), and PPh_3 (20.5 g, 77.2 mmol, 1.5 equiv.) in anhydrous THF (400 mL). To the refluxing mixture was added dropwise under N_2 a solution of iodine (19.6 g, 77.2 mmol, 1.5 equiv.) in anhydrous THF (100 mL). After stirring at reflux for 2 h, the reaction mixture was allowed to cool to room temperature and the precipitate filtered off and washed with THF. The filtrate was concentrated *in vacuo* and the crude material purified *via* column chromatography using an eluent of $MeOH/CH_2Cl_2$=1:10 to provide the product as a colorless solid in a yield of 58% (9.05 g, 29.8 mmol). The NMR spectroscopic data is consistent with previously reported data.[12,13]

^{1}H NMR (500 MHz, D_2O) δ (ppm) 4.76 (d, *J* = 1.7 Hz, 1H, H1), 3.96 (dd, *J* = 3.5, 1.7 Hz, 1H, H2), 3.79 (dd, *J* = 9.6, 3.4 Hz, 1H, H3), 3.67 (dd, *J* = 11.1, 2.3 Hz, 1H, H6a), 3.60 (t, *J* = 9.4 Hz, 1H, H4), 3.52 – 3.46 (m, 1H, H5), 3.47 (s, 3H, H7), 3.40 (dd, *J* = 11.0, 7.2 Hz, 1H, H6b). **^{13}C NMR** (126 MHz, D_2O) δ (ppm) 101.8 (C1), 72.2 (C5), 71.3 (C4), 70.8 (C3), 70.6 (C2), 55.7 (C7), 6.9 (C6). **HRMS** ESI(+) (MeOH) calculated for $C_7H_{13}O_5INa^+$ [M+Na]$^+$: 326.96999, found: 326.96950.

Methyl 6-deoxy-6-iodo-2,3-O-isoproylidene-α-D-mannopyranoside[14,15] (10)

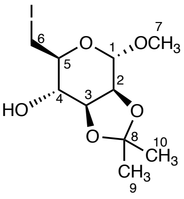

Methyl 6-deoxy-6-iodo-2,3-O-isoproylidene-α-D-mannopyranoside (**10**) was synthesized after Kumamoto *et al.*[15] from methyl 6-deoxy-6-iodo-α-D-mannopyranoside **10-I** (2.20 g, 7.24 mmol, 1.0 equiv.). To a solution of the iodo-sugar in acetone (technical grade, 42 mL, 0.2 M) was added 2,2-dimethoxypropane (5.34 mL, 43.4 mmol, 6.0 equiv.) and the mixture cooled to 0 °C. At 0 °C, pTsOH•H_2O (0.83 g, 4.34 mmol, 0.6 equiv.) was added and the mixture stirred at 0 °C for 5 min, before it was allowed to warm to 25 °C and stirred for another 2 h at 25 °C. After complete conversion of the starting material, NEt_3 (4.8 mL, 34.0 mmol, 4.7 equiv.) was added and the mixture concentrated by removing two-thirds of the volatiles *in vacuo*. The mixture was diluted with CH_2Cl_2 (40 mL) and saturated aqueous $NaHCO_3$ solution (80 mL). The phases were separated, and the aqueous phase extracted with CH_2Cl_2 (2×40 mL).The combined organic phase was washed with brine (1×120 mL), dried over anhydrous $MgSO_4$, filtered, and concentrated *in vacuo* to provide the crude product. Purification via column chromatography, using an eluent of CH_2Cl_2:MeOH = 95:5, afforded the product **10** as a colorless to pale yellow solid in a yield of 90% (2.24 g, 6.51 mmol). The NMR spectroscopic data is consistent with previously reported data.[14]

^{1}H NMR (400 MHz, $CDCl_3$) δ (ppm) 4.90 (s, 1H, H1), 4.12 – 4.06 (m, 2H, H2, H3), 3.56 (dd, *J* = 10.5, 2.3 Hz, 1H, H6a), 3.45 (s, 3H, H7), 3.51 – 3.35 (m, 2H, H4, H5), 3.29 (dd, *J* = 10.5, 6.9 Hz, 1H, H6b), 1.50 (s, 3H, H9 or H10), 1.32 (s, 3H, H9 or H10). **^{13}C NMR** (101 MHz, $CDCl_3$) δ (ppm) 109.9, 98.4, 78.6, 75.8, 73.1, 68.9, 55.6, 28.1, 26.2, 7.0. **HRMS** ESI(+) (MeOH) calculated for $C_{10}H_{17}O_5INa^+$ $[M+Na]^+$: 367.00129, found: 367.00150.

Methyl 4-O-tert-butyldiphenylsilyloxy-6-deoxy-6-iodo-2,3-O-isoproylidene-α-D-mannopyranoside (11-I)

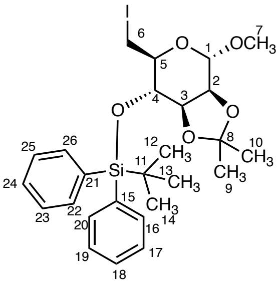

The procedure was adapted from Ghosal and Shaw with minor modifications.[16] To a solution of methyl 6-deoxy-6-iodo-2,3-O-isoproylidene-α-D-mannopyranoside **10** (2.20 g, 6.39 mmol, 1.0 equiv) in anhydrous CH_2Cl_2 (32 mL, 0.2 M) was added imidazole (0.87 g, 12.8 mmol, 2.0 euqiv.), followed by TBDPSCl (2.49 mL, 9.59 mmol, 1.5 equiv.). The reaction mixture was stirred at 25 °C for 19 h. After complete conversion of the starting material, water (60 mL) was added, and the phases separated. The aqueous layer was extracted with CH_2Cl_2 (2×60 mL), the combined organic phase dried over anhydrous $MgSO_4$, filtered, and concentrated *in vacuo* to afford the crude product as an orange oil. The crude product was purified by column chromatography using an eluent of EtOAc/pentane = 1:19. The product **11-I** was obtained as a colorless oil in a yield of 76% (2.82 g, 4.84 mmol).

^{1}H NMR (500 MHz, $CDCl_3$) δ (ppm) 7.72 – 7.68 (m, 2H, H16, H20), 7.66 – 7.63 (m, 2H, H22, H26), 7.47 – 7.32 (m, 6H, H17, H18, H19, H23, H24, H25), 4.83 (s, 1H, H1), 4.21 (t, *J*=6.2 Hz, 1H, H3), 4.06 (d, *J*=5.7 Hz, 1H, H2), 3.68 (td, *J*=9.4, 2.2 Hz, 1H, H5), 3.51 (s, 3H, H7), 3.49 (dd, *J*=10.4, 1.7 Hz, 1H, H6a), 3.39 (dd, *J*=9.6, 6.7 Hz, 1H, H4), 2.77 (t, *J*=9.8 Hz, 1H, H6b), 1.22 (s, 3H, H10), 1.06 (s, 9H, H12, H13, H14), 1.06 (s, 3H, H9). **^{13}C NMR** (126 MHz, $CDCl_3$) δ (ppm) 136.6 (C16, C20), 136.0 (C22, C26), 133.8 (C21), 132.5 (C15), 130.1 (C18), 129.9 (C24), 127.8 (C23, C25), 127.7 (C17, C19), 109.4 (C8), 98.4 (C1), 78.8 (C3), 75.9 (C2), 75.5 (C4), 70.9 (C5), 55.8 (C7), 27.5 (C9), 27.1 (C12, C13, C14), 26.4 (C10), 19.8 (C11), 6.7 (C6). **HRMS** ESI(+) (MeOH/$CHCl_3$ 3:2) calculated for $C_{26}H_{35}O_5INaSi^+$ $[M+Na]^+$: 605.11906, found: 605.11829. **R_f** (EtOAc/pentane = 1:15) = 0.6. **Specific Rotation** $[\alpha]_D^{24°C}$ = +3.7 (c = 1.2, MeOH). **FT-IR** (film): ν (cm^{-1}) 2986w, 2956w, 2932w, 2857w, 1472w, 1463w, 1428w, 1382w, 1371w, 1244w, 1219m, 1201w, 1168m, 1140m, 1111s, 1089s, 1060s, 1026s, 1006w, 999w, 975m, 960m, 940w, 928w, 853s, 821m, 775m, 740m, 701s, 689m, 658w, 634w, 621w, 610m, 501s, 486m, 471w.

Methyl 4-O-tert-butyldiphenylsilyloxy-6-deoxy-2,3-O-isoproylidene-α-D-manno-hex-5-enopyranoside (11)

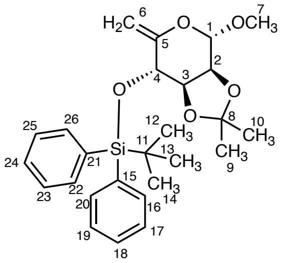

The procedure was adapted from Matzner *et al.*[17] and Chrétien[18] with minor modifications. NaH (60% in mineral oil, 48.1 mmol, 1.92 g, 4.0 equiv.) was suspended in anhydrous THF (40 mL) and stirred under Ar for 5 min. The solvent was then removed and anhydrous DMF (35 mL) and anhydrous THF (70 mL) were added, and the mixture cooled to 0 °C. A solution of methyl 4-*O*-*tert*-butyldiphenylsilyloxy-6-deoxy-6-iodo-2,3-*O*-isoproylidene-α-D-mannopyranoside **11-I** (12.0 mmol, 7.00 g, 1.0 equiv.) in a mixture of anhydrous DMF (85 mL) and anhydrous THF (175 mL) was then added slowly to the suspension at 0 °C. The mixture was stirred for 20 min at 0 °C, before it was allowed to warm to 25 °C and stirred for 17 h at 25 °C. After full conversion of the starting material, the mixture was cooled to 0 °C and the reaction carefully quenched by slow addition of ice-cold half saturated aqueous NH_4Cl solution (300 mL), followed by EtOAc (300 mL). The phases were separated, and the aqueous phase extracted with EtOAc (3×300 mL). The combined organic phase was dried over anhydrous $MgSO_4$, filtered, and concentrated *in vacuo* to provide the crude product. The crude was purified via column chromatography using an eluent of EtOAc/pentane = 1:18 to afford the product **11** as a colorless oil in a yield of 75% (4.10 g, 9.02 mmol).

^{1}H NMR (500 MHz, $CDCl_3$) δ (ppm) 7.74 – 7.67 (m, 4H, TBDPS-Ar-H), 7.45 – 7.39 (m, 2H, TBDPS-Ar-H), 7.38 – 7.33 (m, 4H, TBDPS-Ar-H), 4.78 (d, *J*=3.8 Hz, 1H, H1), 4.52 (t, *J*=0.9 Hz, 1H, H6a), 4.45 (t, *J*=1.0 Hz, 1H, H6b), 4.28 – 4.24 (m, 2H, H3, H4), 4.17 – 4.11 (m, 1H, H2), 3.50 (s, 3H, H7), 1.22 (s, 3H, H10), 1.20 (s, 3H, H9), 1.11 (s, 9H, H12, H13, H14). **^{13}C NMR** (126 MHz, $CDCl_3$) δ (ppm) 155.1 (C5), 136.3 (2xTBDPS-Ar-CH), 136.2 (2xTBDPS-Ar-CH), 133.6 (TBDPS-Ar-C), 133.5 (TBDPS-Ar-C), 129.8 (2xTBDPS-Ar-CH), 127.6 (2x TBDPS-Ar-CH), 127.5 (2xTBDPS-Ar-CH), 109.8 (C8), 100.6 (C1), 93.3 (C6), 77.9 (C3), 75.2 (C2), 71.2 (C4), 56.5 (C7), 27.1 (C12, C13, C14), 26.9 (C9), 25.1 (C10), 19.6 (C11).

HRMS ESI(+) (MeOH) calculated for $C_{26}H_{34}O_5NaSi^+$ $[M+Na]^+$: 477.20677, found: 477.20619. **R_f** (EtOAc/pentane = 1:15) = 0.5. **Specific Rotation** $[\alpha]_D^{23°C}$ = + 43.1 (c = 0.5, MeOH). **FT-IR** ($CDCl_3$): ν (cm^{-1}) 3072w, 3049w, 2988w, 2957w, 2932m, 2898w, 2858w, 1661w, 1473w, 1463w, 1428m, 1382m, 1372m, 1254w, 1245w, 1215m, 1198m, 1165m, 1137m, 1111s, 1097s, 1074s, 1038m, 1014m, 998m, 977m, 929w, 873m, 847m, 822m, 805m, 790w, 740m, 702s, 622w, 612m, 509m, 488m.

(4S,7S,7aR)-7-((tert-butyldiphenylsilyl)oxy)-4-methoxy-2,2-dimethyltetrahydro-6H-[1,3]dioxolo[4,5-c]pyran-6-one (12)

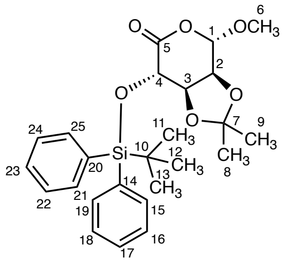

The procedure was adapted from Hedberg *et al.*[19] with minor modifications. Methyl 4-*O*-*tert*-butyldiphenylsilyloxy-6-deoxy-2,3-*O*-isoproylidene-α-D-manno-hex-5-enopyranoside **11** (8.91 mmol, 4.05 g, 1.0 equiv.) was dissolved in anhydrous CH_2Cl_2/MeOH (1:1, 200 mL, 0.04 M) and the mixture cooled to -78 °C while purging with N_2. At -78 °C, first O_2 was bubbled through the solution for 5 min, then ozone was bubbled through the solution for 30 min under constant stirring during which time the color of the solution first turned yellow and then dark blue. After a color change to dark blue was observed, the ozone supply was switched off, O_2 bubbled through for 15 min followed by N_2 for 10 min until the deep blue color had faded. At -78 °C was then added dimethyl sulfide (1960 µL, 26.7 mmol, 3.0 equiv.) and the mixture then allowed to slowly warm to 25 °C. After stirring at 25 °C for 1 h, the volatiles were removed *in vacuo* to provide the crude material. Purification by column chromatography using an eluent of EtOAc/pentane = 1:10 afforded the product **12** as a colorless oil in a yield of 70% (2.83 g, 6.19 mmol).

^{1}H NMR (500 MHz, $CDCl_3$) δ (ppm) 7.79 – 7.75 (m, 2H, TBDPS-Ar-H), 7.75 – 7.71 (m, 2H, TBDPS-Ar-H), 7.46 – 7.41 (m, 2H, TBDPS-Ar-H), 7.40 – 7.34 (m, 4H, TBDPS-Ar-H), 4.84 (d, *J* = 6.5 Hz, 1H, H1), 4.44 (dd, *J* = 8.2, 6.9 Hz, 1H, H3), 4.24 (d, *J* = 6.8 Hz, 1H, H4), 4.22 (dd, *J* = 8.3, 6.8 Hz, 1H, H2), 3.54 (s, 3H, H6), 1.34 (s, 3H, H8), 1.30 (s, 3H, H9), 1.13 (s, 9H, H11, H12, H13). **^{13}C NMR** (126 MHz, $CDCl_3$) δ (ppm) 168.0 (C5), 136.4 (2xTBDPS-Ar-CH), 136.4 (2xTBDPS-Ar-CH), 132.9 (TBDPS-Ar-C), 132.5 (TBDPS-Ar-C), 130.1 (TBDPS-Ar-CH), 130.0 (TBDPS-Ar-CH), 127.7 (2xTBDPS-Ar-CH), 127.6 (2xTBDPS-Ar-CH), 111.8 (C7), 102.3 (C1), 76.9 (C4), 75.0 (C2), 72.4 (C4), 57.8 (C6), 27.0 (C11, C12, C13), 26.6 (C8), 24.5 (C9), 19.7 (C10). **HRMS** ESI(+) (MeOH) calculated for $C_{25}H_{32}O_6NaSi^+$ $[M+Na]^+$: 479.18604, found: 479.18573. **R_f** (EtOAc/pentane = 1:9) = 0.4. **Specific Rotation** $[\alpha]_D^{24°C}$ = +63.7 (c = 0.9, MeOH). **FT-IR** ($CDCl_3$): ν (cm^{-1}) 2933w, 2898w, 2859w, 1773s, 1473w, 1463w, 1455w, 1428w, 1384w, 1376w, 1262w, 1235w, 1212m, 1196w, 1143s, 1114s, 1079s, 1030w, 997m, 976m, 935w, 897w, 872m, 851w, 822w, 742w, 723w, 703s, 621w, 612w, 512m, 486w.

(7S,7aR)-7-((tert-butyldiphenylsilyl)oxy)-2,2,6,6-tetramethyltetrahydro-4H-[1,3]dioxolo[4,5-c]pyran-4-ol (13-I)

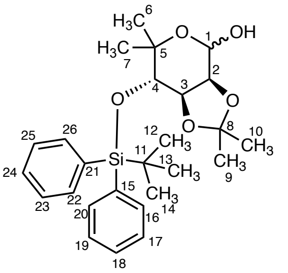

The procedure was adapted from Hedberg *et al.*[19] with minor modifications. To a solution of (4S,7S,7aR)-7-((tert-butyldiphenylsilyl)oxy)-4-methoxy-2,2-dimethyltetrahydro-6H-[1,3]dioxolo[4,5-c]pyran-6-one **12** (3.50 mmol, 1.60 g, 1.0 equiv.) in THF (110 mL, 0.03 M) at -78 °C was added slowly a pre-mixed (at 25 °C) solution of N,N,N",N"-tetramethylethylenediamine (TMEDA) (11.6 mmol, 1.75 mL, 3.3 equiv.) and methylmagnesium chloride (3 M solution in THF, 9.46 mmol, 3.15 mL, 2.7 equiv.) in THF (7 mL). The reaction mixture was stirred at -78 °C for 0.5 h, before it was allowed to warm to 25 °C and stirred at 25 °C for 4 h. After full conversion of the starting material, the mixture was cooled to 0 °C and then slowly quenched with water (120 mL). The aqueous phase was extracted with CH_2Cl_2 (3×120 mL). The combined organic phase was washed with 1 M solution of aqueous HCl (1×300 mL) and brine (1×300 mL), dried over anhydrous $MgSO_4$, filtered, and concentrated *in vacuo* to provide the crude material. The crude mixture was purified *via* column chromatography using an eluent of EtOAc/pentane = 15:85 to afford the product **13-I** as a colorless oil as a mixture of epimers (α:β = 1:0.45) in a yield of 71% (1.14 g, 2.50 mmol).

^{1}H NMR($CDCl_3$, 500 MHz) δ (ppm) (mixture of epimers α:β = 1:0.45) 7.73 – 7.62 (m, 5.8H, TBDPS-Ar-Hα+β), 7.46 – 7.40 (m, 2.9H, TBDPS-Ar-Hα+β), 7.39 – 7.33 (m, 5.8H, TBDPS-Ar-Hα+β), 5.13 (dd, *J*=10.8, 2.5 Hz, 1H, H1α), 4.95 (dd, *J*=6.3, 4.2 Hz, 0.45H, H1β), 4.29 (dd, *J*=7.2, 6.3 Hz, 0.45H, H3β), 4.23 (dd, *J*=6.8, 5.7 Hz, 1H, H3α), 4.14 (dd, *J*=6.7, 2.5 Hz, 1H, H2α), 4.06 (dd, *J*=7.2, 4.2 Hz, 0.45H, H2β), 3.77 (d, *J*=5.7 Hz, 1H, H4α), 3.69 (d, *J*=6.3 Hz, 0.45H, H4β), 3.36 (d, *J*=10.8 Hz, 1H, OH1α), 3.07 (d, *J*=6.3 Hz, 0.45H, OH1β), 1.37 (s, 1.35H, H7β), 1.28 (s, 3H, H7α), 1.19 (s, 3H, H6α), 1.18 (s, 1.35H, H6β), 1.18 (s, 3H, H9α), 1.16 (s, 1.35H, H9β), 1.15 (s, 3H, H10α),1.10 (s, 1.35H, H10β), 1.09 (s, 4.05H, H12β, H13β, H14β), 1.08 (s, 9H, H12α, H13α, H14α). **^{13}C NMR** (126 MHz, $CDCl_3$) δ (ppm) 136.4 (2xTBDPS-Ar-CH), 136.4 (2xTBDPS-Ar-CH), 136.3 (2xTBDPS-Ar-CH), 136.2 (2xTBDPS-Ar-CH), 133.5 (TBDPS-Ar-C), 133.5 (TBDPS-Ar-C), 133.5 (TBDPS-Ar-C), 133.3 (TBDPS-Ar-C), 130.0 (TBDPS-Ar-CH), 129.9 (TBDPS-Ar-CH), 129.9 (TBDPS-Ar-CH), 129.9 (TBDPS-Ar-CH), 127.7 (2xTBDPS-Ar-CH), 127.7 (2xTBDPS-Ar-CH), 127.6 (2xTBDPS-Ar-CH), 127.5 (2xTBDPS-Ar-CH), 110.0 (C8α), 109.1 (C8β), 92.7 (C1β), 88.7 (C1α), 77.4 (C3α), 76.7 (C5α), 76.6 (C2α), 76.4 (C5β), 76.4 (C3β), 76.0 (C4β), 75.1 (C4α), 74.2 (C2β), 28.8 (C6α), 27.5 (C6β), 27.3 (C12α, C13α, C14α), 27.3 (C12β, C13β, C14β), 26.8 (C9β), 26.6 (C9α), 25.6

(C10α), 25.4 (C7β), 24.8 (C10β), 21.9 (C7α), 19.8 (C11β), 19.8 (C11α). **HRMS** ESI(+) (MeOH) calculated for $C_{26}H_{36}O_5NaSi^+$ [M+Na]$^+$: 479.22242, found: 479.22266. $\mathbf{R_f}$ (EtOAc/pentane = 15:85) = 0.3. **Specific Rotation** $[\alpha]_D^{24°C}$ = +8.9 (c = 0.5, MeOH). **FT-IR** ($CDCl_3$): ν (cm^{-1}) 3431w, 2975w, 2933w, 2858w, 1472w, 1463w, 1428w, 1381w, 1371w, 1242w, 1215w, 1165w, 1111s, 1085m, 1073m, 1031w, 1007w, 970w, 879w, 854w, 822w, 806w, 740w, 702m, 690w, 622w, 612w, 508m, 488w.

(3aS,4R/S,7S,7aR)-7-((tert-butyldiphenylsilyl)oxy)-2,2,6,6-tetramethyltetrahydro-4H-[1,3]dioxolo[4,5-c]pyran-4-yl acetate (13)

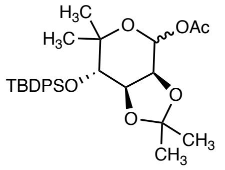

The procedure was adapted from Hedberg *et al.*[19] with minor modifications. To a solution of (7*S*,7a*R*)-7-((*tert*-butyldiphenylsilyl)oxy)-2,2,6,6-tetramethyltetrahydro-4H-[1,3]dioxolo[4,5-c]pyran-4-ol **13-I** (0.66 mmol, 300 mg, 1.0 equiv.) in anhydrous pyridine (2 mL, 0.4 M) at 0 °C was added dropwise acetic anhydride (0.85 mmol, 80 µL, 1.3 equiv.) and the solution then allowed to warm to 25 °C and stirred at 25 °C for 17 h. After full conversion of the starting material, toluene (3 mL) was added, and the solvent was then removed *in vacuo* and then co-evaporated with toluene (3×3 mL). The crude material was purified *via* column chromatography using an eluent of EtOAc/pentane = 1:15 to afford both anomeric epimers of the product **13** as colorless solids in a yield of 78% (α, 256 mg, 0.51 mmol) and 18% (β, 60 mg, 0.12 mmol), respectively.

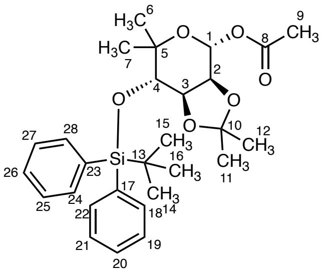

^{1}H NMR (500 MHz, $CDCl_3$) δ (ppm) 7.72 – 7.68 (m, 2H, TBDPS-Ar-H), 7.68 – 7.63 (m, 2H, TBDPS-Ar-H), 7.44 – 7.38 (m, 2H, TBDPS-Ar-H), 7.38 – 7.33 (m, 4H, TBDPS-Ar-H), 5.92 (d, *J*=4.0 Hz, 1H, H1), 4.26 (t, *J*=6.7 Hz, 1H, H3), 4.14 (dd, *J*=6.8, 4.0 Hz, 1H, H2), 3.64 (d, *J*=6.7 Hz, 1H, H4), 2.09 (s, 3H, H9), 1.35 (s, 3H, H6), 1.20 (s, 3H, H7), 1.10 (s, 3H, H11), 1.08 (s, 9H, H15, H16, H17), 1.07 (s, 3H, H12). **^{13}C NMR** (126 MHz, $CDCl_3$) δ (ppm) 169.6 (C8), 136.4 (2xTBDPS-Ar-CH), 136.2 (2xTBDPS-Ar-CH), 133.7 (TBDPS-Ar-C), 133.5 (TBDPS-Ar-C), 129.8 (TBDPS-Ar-CH), 129.8 (TBDPS-Ar-CH), 127.6 (2xTBDPS-Ar-CH), 127.5 (2xTBDPS-Ar-CH), 109.3 (C10), 91.5 (C1), 77.6 (C5), 77.0 (C3), 76.5 (C4), 75.1 (C2), 27.3 (C15, C16, C17), 27.0 (C12), 26.8 (C7), 25.3 (C11), 23.7 (C6), 21.5 (C9), 19.8 (C13). **HRMS** ESI(+) (MeOH) calculated for $C_{28}H_{38}O_6NaSi^+$ [M+Na]$^+$: 521.23299, found: 521.23270. $\mathbf{R_f}$ (EtOAc/pentane = 0.5:9.5) = 0.3. **Melting point** m_p 79.4±0.2 °C. **Specific Rotation** $[\alpha]_D^{24°C}$ = +35.2 (c = 0.5, MeOH). **FT-IR** ($CDCl_3$): ν (cm^{-1}) 2934w, 2858w, 1758m, 1473w, 1428w, 1371m, 1218m, 1175m, 1150m, 1112s, 1079m, 1042m, 1004m, 956m, 931m, 879w, 846w, 821w, 741w, 703m, 611w, 566w, 508m. The structure was confirmed by X-ray crystal structure analysis. Crystals were obtained from recovered starting material after TBDPS deprotection attempts. The crystals formed upon slow evaporation from EtOAc (over 3 days).

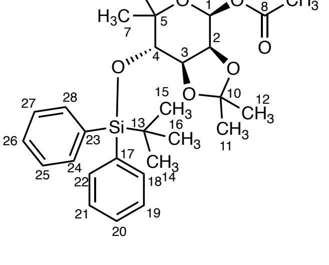

^{1}H NMR(500 MHz, $CDCl_3$) δ (ppm) 7.74 – 7.67 (m, 4H, TBDPS-Ar-H), 7.46 – 7.31 (m, 6H, TBDPS-Ar-H), 6.05 (d, *J*=3.4 Hz, 1H, H1), 4.33 (t, *J*=7.4 Hz, 1H, H3), 4.26 (dd, *J*=7.8, 3.4 Hz, 1H, H2), 3.96 (d, *J*=7.0 Hz, 1H, H4), 1.79 (s, 3H, H9), 1.30 (s, 3H, H6), 1.19 (s, 3H, H11), 1.15 (s, 3H, H12), 1.11 (s, 3H, H7), 1.09 (s, 9H, H15, H16, H17). **^{13}C NMR** (126 MHz, $CDCl_3$) δ (ppm) 169.5 (C8), 136.6 (2xTBDPS-Ar-CH), 136.5 (2xTBDPS-Ar-CH), 133.7 (TBDPS-Ar-C), 133.6 (TBDPS-Ar-C), 129.7 (TBDPS-Ar-CH), 129.6 (TBDPS-Ar-CH), 127.5 (2xTBDPS-Ar-CH), 127.2 (2xTBDPS-Ar-CH), 110.0 (C10), 89.9 (C1), 77.3 (C3), 76.2 (C4), 72.3 (C2), 28.9 (C7), 27.3 (C15, C16, C17), 26.3 (C11), 24.8 (C12), 23.1 (C6),

21.3 (C9), 19.7 (C13). **HRMS** ESI(+) (MeOH) calculated for $C_{28}H_{38}O_6NaSi^+$ $[M+Na]^+$: 521.23299, found: 521.23282. **R*f*** (EtOAc/pentane = 0.5:9.5) = 0.2. **Melting point** m_p 122.5±0.4 °C. **Specific Rotation** $[\alpha]_D^{24°C}$ = –31.3 (c = 0.6, MeOH). **FT-IR** ($CDCl_3$): ν (cm^{-1}) 2976w, 2933w, 2858w, 1752m, 1472w, 1463w, 1428w, 1372m, 1264w, 1233m, 1211m, 1162w, 1107s, 1081m, 1040m, 1008m, 988w, 971w, 933w, 906w, 833m, 741w, 702m, 690w, 611w, 509m, 487w.

(3aS,4R,7S,7aS)-7-hydroxy-2,2,6,6-tetramethyltetrahydro-4H-[1,3]dioxolo[4,5-c]pyran-4-yl acetate (14)

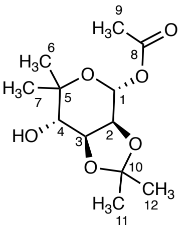

The procedure was adapted from Scheidt *et al.*[20] with modifications. The reaction was performed in a polypropylene tube (15 mL) equipped with a stirring bar. No precautions were taken to avoid air. To a solution of (3a*S*,4*R*,7*S*,7a*R*)-7-((*tert*-butyldiphenylsilyl)oxy)-2,2,6,6-tetramethyltetrahydro-4*H*-[1,3]dioxolo[4,5-*c*]pyran-4-yl acetate **13** (α-epimer, 0.74 mmol, 367 mg, 1.0 equiv.) in anhydrous DMF (5.8 mL, 0.1 M) at 25 °C was added milliQ water (1.84 mmol, 33 µL, 2.5 equiv.). To the solution was then added a solution of TASF (243 mg, 0.88 mmol, 1.2 equiv.) in anhydrous DMF (1.8 mL). The reaction was stirred at 25 °C for 7 h. After full conversion of the starting material, the solution was diluted into EtOAc (110 mL), and the organic phase washed with 1 M aqueous $KHSO_4$ solution (2×40 mL) and saturated aqueous $NaHCO_3$ solution (1×60 mL). The organic phase was dried over anhydrous $MgSO_4$, filtered, and concentrated *in vacuo* to provide the crude material as a colorless oil. The crude material was purified *via* column chromatography using an eluent of EtOAc/pentane = 3:7 to afford the product **14** as a single epimer (α) as a colorless solid in a yield of 81% (156 mg, 0.60 mmol). *Note*: Water should be added before TASF to avoid side product formation.

^{1}H NMR (500 MHz, $CDCl_3$) δ (ppm) 6.13 (d, *J* = 2.8 Hz, 1H, H1), 4.29 – 4.22 (m, 2H, H2, H3), 3.61 (d, *J* = 6.8 Hz, 1H, H4), 2.10 (s, 3H, H9), 1.52 (s, 3H, H11), 1.37 (s, 6H, H6, H12), 1.25 (s, 3H, H7). **^{13}C NMR** (126 MHz, $CDCl_3$) δ (ppm) 169.3 (C8), 110.1 (C10), 91.4 (C1), 77.0 (C5), 76.7 (C3), 75.4 (C2), 74.8 (C4), 27.8 (C11), 26.6 (C6), 25.7 (C12), 22.6 (C7), 21.4 (C9). **HRMS** ESI(+) (MeOH) calculated for $C_{12}H_{20}O_6Na^+$ $[M+Na]^+$: 283.11521, found: 283.11502. **R*f*** (EtOAc/pentane = 3:7) = 0.3. **Melting point** mp 92.5±0.3 °C. **Specific Rotation** $[\alpha]_D^{23°C}$ = + 12.4 (c = 1.5, MeOH). **FT-IR** (solid): ν (cm^{-1}) 3473w, 3441w, 2986w, 2938w, 2924w, 1752s, 1459w, 1442w, 1429w, 1384m, 1373s, 1236s, 1216s, 1194w, 1170s, 1143s, 1074s, 1037s, 1020s, 1005m, 985m, 956s, 926s, 902m, 873m, 848m, 815m, 792w, 657w, 606w, 568m, 512m. *Note*: When repeating the experiment on a larger scale (2.49 mmol, 1.24 g), the product was obtained as a colorless, crystalline solid, allowing its characterization by X-ray crystal structure analysis.

(3aS,4S,7S,7aS)-4-acetoxy-2,2,6,6-tetramethyltetrahydro-4H-[1,3]dioxolo[4,5-c]pyran-7-yl isobutyrate (15a)

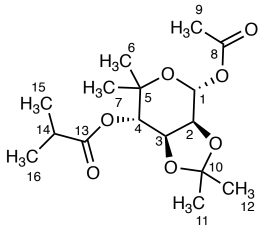

To a solution of (3a*S*,4*R*,7*S*,7a*S*)-7-hydroxy-2,2,6,6-tetramethyltetrahydro-4*H*-[1,3]dioxolo[4,5-*c*]pyran-4-yl acetate **14** (2.55 mmol, 665 mg, 1.0 equiv.) in anhydrous pyridine (6.4 mL, 0.4 M) at 0 °C was added dropwise isobutyric anhydride (3.32 mmol, 568 µL, 1.3 equiv.) and the solution then allowed to warm to 25 °C and stirred at 25 °C for 24 h. After full conversion of the starting material, toluene (6 mL) was added, and the solvent was removed *in vacuo* and then co-evaporated with toluene (3×6 mL). The crude material was purified *via* column chromatography using an eluent of EtOAc/pentane = 1:10. After concentration *in vacuo* the residue was taken up in EtOAc (100 mL) and the organic phase washed with saturated aqueous

$NaHCO_3$ solution (2×100 mL), dried over anhydrous MgSO4, filtered, and concentrated *in vacuo* to provide the product **15a** as a colorless solid in a yield of 84% (705 mg, 2.13 mmol). **^{1}H NMR** (500 MHz, CDCl3): δ (ppm) 6.15 (d, *J*=3.6 Hz, 1H, H1), 5.05 (d, *J*=6.4 Hz, 1H, H4), 4.26 (t, *J*=6.1 Hz, 1H, H3), 4.19 (dd, *J*=5.9, 3.6 Hz, 1H, H2), 2.62 (hept, *J*=7.0 Hz, 1H, H14), 2.11 (s, 3H, H9), 1.55 (s, 3H, H11), 1.35 (s, 3H, H12), 1.33 (s, 3H, H7), 1.24 (s, 3H, H6), 1.21 (d, *J*=7.0 Hz, 3H, H15), 1.20 (d, *J*=7.0 Hz, 3H, H16). **^{13}C NMR** (126 MHz, $CDCl_3$) δ (ppm) 176.1 (C13), 169.3 (C8), 110.2 (C10), 91.1 (C1), 76.2 (C5), 74.9 (C2), 74.7 (C3), 72.7 (C4), 34.2 (C14), 27.7 (C11), 26.2 (C12), 25.9 (C7), 23.8 (C6), 21.4 (C9), 19.2 (C15), 19.0 (C16). **HRMS** ESI(+) (MeOH + NaI) calculated for $C_{16}H_{26}O_7Na^+$ $[M+Na]^+$: 353.15707, found: 353.15694. **R*f*** (EtOAc/pentane = 2:8) = 0.7. **Melting point** m_p 66.8±0.9 °C. **Specific Rotation** $[\alpha]_D^{24°C}$ = + 14 (c = 0.7, MeOH). **FT-IR** ($CDCl_3$): ν (cm^{-1}) 2983w, 2938w, 1742s, 1470w, 1385m, 1372m, 1220s, 1200m, 1176m, 1147s, 1082m, 1045s, 1019m, 1004m, 987m, 953s, 874m, 853m, 817w, 789w, 752w, 605w, 573w, 553w, 511m.

(3aS,4R,7S,7aS)-4-Acetoxy-2,2,6,6-tetramethyltetrahydro-4H-[1,3]dioxolo[4,5-c]pyran-7-yl cyclopropanecarboxylate (15b)

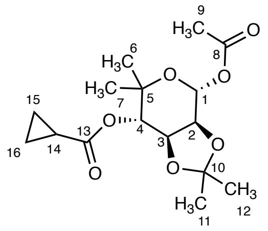

To a solution of (3a*S*,4*R*,7*S*,7a*S*)-7-hydroxy-2,2,6,6-tetramethyltetrahydro-4*H*-[1,3]dioxolo[4,5-*c*]pyran-4-yl acetate **14** (1.92 mmol, 500 mg, 1.0 equiv.) in anhydrous pyridine (4.8 mL, 0.4 M) at 0 °C was added dropwise cyclopropanecarboxylic acid anhydride (2.50 mmol, 346 µL, 1.3 equiv.) and the solution then allowed to warm to 25 °C and stirred at 25 °C for 24 h. Then, toluene (6 mL) was added, and the solvent was removed *in vacuo* and then co-evaporated with toluene (2×6 mL). The mixture was taken up in EtOAc (50 mL) and the organic phase washed with saturated aqueous $NaHCO_3$ solution (3×50 mL), dried over anhydrous $MgSO_4$, filtered, and concentrated *in vacuo* to provide a colorless oil. The crude material was purified *via* column chromatography using an eluent of EtOAc/pentane = 1:10 to provide after concentration *in vacuo* the product **15b** as a colorless, crystalline solid in a yield of 72% (456 mg, 1.39 mmol). **^{1}H NMR** (500 MHz, $CDCl_3$) δ (ppm) 6.13 (d, *J* = 3.8 Hz, 1H, H1), 5.07 (d, *J* = 6.1 Hz, 1H, H4), 4.29 (t, *J* = 6.0 Hz, 1H, H3), 4.20 (dd, *J* = 5.8, 3.9 Hz, 1H, H2), 2.12 (s, 3H, H9), 1.67 (tt, *J* = 8.3, 3.9 Hz, 1H, H14), 1.54 (s, 3H, H11), 1.35 (s, 3H, H12), 1.35 (s, 3H, H7), 1.25 (s, 3H, H6), 1.09 – 1.00 (m, 2H, H15a, H16a), 0.95 – 0.87 (m, 2H, H15b, H16b). **^{13}C NMR** (126 MHz, $CDCl_3$) δ (ppm) 174.0 (C13), 169.4 (C8), 110.3 (C10), 91.1 (C1), 76.2 (C5), 74.8 (C3), 74.7 (C2), 72.8 (C4), 27.7 (C11), 26.1 (C12), 25.7 (C7), 23.9 (C6), 21.4 (C9), 13.0 (C14), 9.1 (C15 or C16), 8.9 (C15 or C16). **HRMS** ESI(+) (MeOH) calculated for $C_{16}H_{24}O_7Na^+$ $[M+Na]^+$: 351.14142, found: 351.14153. **R*f*** (EtOAc/pentane = 1:9) = 0.2. **Melting point** m_p 91.2±0.4 °C. **Specific Rotation** $[\alpha]_D^{24°C}$ = + 12.8 (c = 0.5, MeOH). **FT-IR** (solid): ν (cm^{-1}) 2985w, 1747m, 1729s, 1458w, 1421w, 1387m, 1375m, 1327w, 1315w, 1307w, 1258w, 1243m, 1221m, 1201m, 1169s, 1148s, 1103w, 1079s, 1043s, 1022s, 1004m, 986m, 955s, 919m, 875m, 847m, 827w, 815m, 789m, 773w, 745w, 725w, 663w, 606w, 574w, 546w, 512m, 497w, 479w, 431w. The structure was confirmed by X-ray crystal structure analysis.

(3aS,4R,7S,7aS)-4-acetoxy-2,2,6,6-tetramethyltetrahydro-4H-[1,3]dioxolo[4,5-c]pyran-7-yl pivalate (15c)

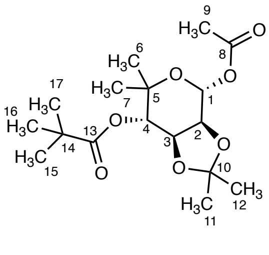

To a solution of (3a*S*,4*R*,7*S*,7a*S*)-7-hydroxy-2,2,6,6-tetramethyltetrahydro-4*H*-[1,3]dioxolo[4,5-*c*]pyran-4-yl acetate **14** (2.31 mmol, 600 mg, 1.0 equiv.) in anhydrous pyridine (6.0 mL, 0.4 M) at 0 °C was added dropwise pivaloyl chloride (4.61 mmol, 567 µL, 2.0 equiv.) and the solution then allowed to warm to 25 °C and stirred at 25 °C for 24 h. Then, the mixture was diluted with EtOAc (50 mL), and the organic phase washed with saturated aqueous $NaHCO_3$ solution

(4×50 mL), dried over anhydrous $MgSO_4$, filtered, and concentrated *in vacuo* to provide a colorless oil. The crude material was purified *via* column chromatography using an eluent of EtOAc/pentane = 1:9 to provide after concentration *in vacuo* the product **15c** as a colorless solid in a yield of 67% (530 mg, 1.54 mmol).

^{1}H NMR (500 MHz, $CDCl_3$): δ (ppm) 6.16 (d, *J*=3.6 Hz, 1H, H1), 5.04 (d, *J*=6.3 Hz, 1H, H4), 4.24 (t, *J*=6.1 Hz, 1H, H3), 4.17 (dd, *J*=5.8, 3.5 Hz, 1H, H2), 2.12 (s, 3H, H9), 1.55 (s, 3H, H11), 1.35 (s, 3H, H12), 1.33 (s, 3H, H7), 1.25 (s, 3H, H6), 1.24 (s, 9H, H15, H16, H17). **^{13}C NMR** (126 MHz, $CDCl_3$) δ (ppm) 177.4 (C13), 169.3 (C8), 110.2 (C10), 91.2 (C1), 76.1 (C5), 74.9 (C2), 74.6 (C3), 72.7 (C4), 39.1 (C14), 27.7 (C11), 27.3 (C15, C16, C17), 26.2 (C12), 26.1 (C7), 23.9 (C6), 21.4 (C9). **HRMS** ESI(+) (MeOH) calculated for $C_{17}H_{28}O_7Na^+$ $[M+Na]^+$: 367.17272, found: 367.17293. **R_f** (EtOAc/pentane = 1:9) = 0.4. **Melting point** m_p 74.7±0.4 °C. **Specific Rotation** $[\alpha]_D^{23\,°C}$ = +15.4 (c = 1.4, MeOH). **FT-IR** (solid): ν (cm^{-1}) 2982w, 2936w, 1750m, 1735m, 1480w, 1457w, 1388w, 1374m, 1280w, 1236m, 1221m, 1198m, 1146s, 1128m, 1080m, 1045m, 1029m, 1015m, 954s, 935m, 910w, 881m, 871m, 852m, 819m, 800w, 789w, 772w, 608w, 575w, 557w, 512m, 494w, 480w, 430w.

(3aS,4S,7S,7aS)-4-Mercapto-2,2,6,6-tetramethyltetrahydro-4H-[1,3]dioxolo[4,5-c]pyran-7-yl isobutyrate (17a)

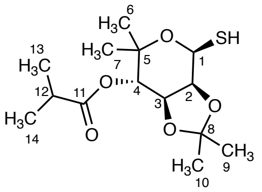

The procedure was adapted from Doyle *et al.* but using TMSBr for the acid and base sensitive starting material.[10] To a solution of (3a*S*,4*R*,7*S*,7a*S*)-4-acetoxy-2,2,6,6-tetramethyltetrahydro-4*H*-[1,3]dioxolo[4,5-*c*]pyran-7-yl isobutyrate **15a** (0.91 mmol, 300 mg, 1.0 equiv.) in anhydrous CH_2Cl_2 (2.3 mL, 0.4 M) at 0 °C in the dark was added dropwise TMSBr (2.72 mmol, 371 µL, 3.0 equiv.) and the mixture stirred at 0 °C for 7.5 h. The reaction was monitored *via* UHPLC-MS analysis. After 7.5 h, the solvent was removed under reduced pressure (high vacuum) at 25 °C. Due to the limited stability, the crude glycosyl bromide was used immediately in the next step without purification. The freshly prepared 2,3-*O*-isopropylidene-4-*O*-isobutyryl-D-noviopyranosyl bromide was dissolved in anhydrous acetone (5.9 mL). Thiourea (1.54 mmol, 118 mg, 1.7 equiv.) was added and the mixture was sparged with Ar for 2 min before it was heated to 60 °C and stirred at 60 °C under reflux for 15 h. After 15 h, the mixture was cooled to 25 °C and the solvent evaporated *in vacuo* at 40 °C water bath temperature to provide the crude thiouronium salt as a colorless to pale green solid. The salt was used in the next step without purification.

The crude salt was suspended in a mixture of CH_2Cl_2 (5.9 mL) and water (3.9 mL, degassed by sparging with Ar) and sodium metabisulfite (1.27 mmol, 242 mg, 1.4 equiv.) was added. The mixture was sparged with Ar for 5 min and then heated to reflux (60 °C) and stirred under reflux for 3 h. Then, the mixture was cooled to 25 °C and the layers were separated. The aqueous phase was extracted with CH_2Cl_2 (3×30 mL),the combined organic phase was washed with water (1×90 mL), dried over anhydrous magnesium sulfate, filtered, and the solvent removed under reduced pressure at 40°C to provide the crude material as a colorless oil. The crude was purified *via* column chromatography on fine pore silica (Silica gel 60; 0.015-0.040 mm) using an eluent of EtOAc/pentane = 5:95 to provide the product **17a** as a colorless, crystalline solid in a yield of 24% (0.21 mmol, 65 mg) over 3 steps along with a mixture of epimers (α:β = 1:1) as a colorless, crystalline solid in a yield of 9% over 3 steps (25 mg, 0.08 mmol).

^{1}H NMR (500 MHz, $CDCl_3$) δ (ppm) 5.08 – 5.04 (m, 1H, H4), 4.97 (dd, *J*=10.7, 1.7 Hz, 1H, H1), 4.24 – 4.18 (m, 2H, H2, H3), 2.87 (d, *J*=10.7 Hz, 1H, SH), 2.60 (hept, *J*=7.0 Hz, 1H, H12), 1.58 (s, 3H, H9), 1.37 (s, 3H, H10), 1.29 (s, 3H, H7), 1.20 (d, *J*=7.1 Hz, 3H, H13), 1.19 (s, 3H, H6), 1.19 (d, *J*=6.8 Hz, 3H, H14). **^{13}C NMR** (126 MHz, $CDCl_3$) δ (ppm) 175.9 (C11), 110.2 (C8), 76.8 (C5), 76.5 (C2), 75.3 (C3), 72.9 (C4), 71.0 (C1), 34.2 (C12), 28.1 (C7), 27.3 (C9), 26.3 (C10), 19.5 (C6), 19.2 (C13 or C14), 19.0 (C13 or C14). **HRMS** ESI(+) (MeOH) calculated for $C_{14}H_{24}O_5NaS^+$ $[M+Na]^+$: 327.12367, found: 327.12359. **R_f** (EtOAc/pentane =

5:95) = 0.4. **Melting point** m_p 77.5±0.8 °C. **Specific Rotation** $[\alpha]_D^{24°C}$ = – 25.3 (c = 0.6, MeOH). **FT-IR** (solid): ν (cm^{-1}) 2979w, 2927w, 2875w, 2854w, 1742s, 1465w, 1384m, 1369m, 1236m, 1219m, 1200m, 1185m, 1147s, 1118s, 1068s, 1037m, 1016m, 1001m, 969s, 944w, 934w, 924m, 905w, 864m, 841w, 823w, 803m, 786w, 761w, 675w, 509m, 426w. The X-ray crystal structural data for the β-epimer is reported in the Crystallographic data section. The crystal was obtained from a mixture of epimers (α:β = 10:90, assigned by ^{1}H NMR analysis) *via* slow evaporation from a solution in EtOAc.

(3aS,4S,7S,7aS)-4-Mercapto-2,2,6,6-tetramethyltetrahydro-4H-[1,3]dioxolo[4,5-c]pyran-7-yl cyclopropanecarboxylate (17b)

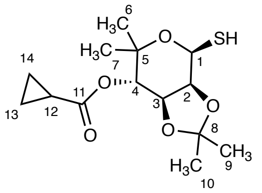

The procedure was adapted from Doyle *et al.* but using TMSBr for the acid and base sensitive starting material.[10] To a solution of (3a*S*,4*R*,7*S*,7a*S*)-4-Acetoxy-2,2,6,6-tetramethyltetrahydro-4*H*-[1,3]dioxolo[4,5-*c*]pyran-7-yl cyclopropanecarboxylate **15b** (1.31 mmol, 430 mg, 1.0 equiv.) in anhydrous CH_2Cl_2 (4.3 mL, 0.3 M) at 0 °C in the dark was added dropwise TMSBr (3.93 mmol, 535 µL, 3.0 equiv.) and the mixture stirred at 0 °C for 7 h. After 7 h, the solvent was removed under reduced pressure (high vacuum) at 0 °C over 1 h. Due to the limited stability, the crude glycosyl bromide was used immediately in the next step without purification.

To the freshly prepared 2,3-*O*-isopropylidene-4-*O*-cyclopropanecarbonyl-D-noviopyranosyl bromide was then added at 0 °C a solution of thiourea (2.62 mmol, 199 mg, 2.0 equiv.) in anhydrous acetone (8.6 mL). The mixture was sparged with Ar for 2 min before it was heated to 60 °C and stirred at 60 °C under reflux for 16 h. After 16 h, the mixture was cooled to 25 °C and the solvent evaporated *in vacuo* to provide the crude thiouronium salt as a colorless to pale blue solid. The salt was used in the next step without purification.

The crude salt was suspended in a mixture of CH_2Cl_2 (8.6 mL) and water (5.6 mL, degassed by sparging with Ar), and sodium metabisulfite (1.83 mmol, 349 mg, 1.4 equiv.) was added. The mixture was sparged with Ar for 5 min and then heated to reflux (60 °C), and stirred under reflux for 3 h. Then, the mixture was cooled to 25 °C, diluted with CH_2Cl_2 (50 mL) and water (50 mL), and the layers were separated. The aqueous phase was extracted with CH_2Cl_2 (3×50 mL), the combined organic phase was washed with water (1×200 mL), dried over anhydrous $MgSO_4$, filtered, and the solvent removed under reduced pressure to provide the crude material as a colorless solid. The crude was purified *via* column chromatography on fine pore silica (Silica gel 60; 0.015-0.040 mm) using an eluent of EtOAc/pentane = 1:10 to provide the product **17b** as a colorless, crystalline solid in a yield of 31% (0.40 mmol, 122 mg) over 3 steps along with a mixture of epimers (α:β = 6:10) as a colorless, crystalline solid in a yield of 15% over 3 steps (59 mg, 0.20 mmol).

^{1}H NMR(500 MHz, $CDCl_3$): δ (ppm) 5.09 – 5.04 (m, 1H, H4), 5.00 – 4.95 (m, 1H, H1), 4.26 – 4.21 (m, 2H, H2, H3), 2.86 (d, *J*=10.7 Hz, 1H, SH), 1.63 (tt, *J*=8.0, 4.6 Hz, 1H, H12), 1.57 (s, 3H, H9), 1.37 (s, 3H, H10), 1.29 (s, 3H, H7), 1.20 (s, 3H, H6), 1.07 – 0.98 (m, 2H, H13a, H14a), 0.94 – 0.87 (m, 2H, H13b, H14b). **^{13}C NMR** (126 MHz, $CDCl_3$) δ (ppm) 174.0 (C11), 110.2 (C8), 76.8 (C5), 76.5 (C2), 75.2 (C3), 73.2 (C4), 71.0 (C1), 28.1 (C7), 27.3 (C9), 26.3 (C10), 19.5 (C6), 13.0 (C12), 9.0 (C13 or C14), 8.8 (C13 or C14). **HRMS** ESI(+) (MeOH) calculated for $C_{14}H_{22}O_5NaS^+$ $[M+Na]^+$: 325.10802, found: 325.10806. **R_f** (EtOAc/pentane = 1:9) = 0.3. **Melting point** m_p 116.2±1.7 °C. **Specific Rotation** $[\alpha]_D^{22°C}$ =–35.1 (c = 0.7, MeOH). **FT-IR** (solid): ν (cm^{-1}) 2995w, 2979w, 2921w, 2853w, 1728s, 1454w, 1388m, 1372m, 1332w, 1242m, 1217m, 1200m, 1172s, 1124m, 1070s, 1035m, 1012m, 1001m, 973m, 950m, 929m, 865m, 846m, 827w, 802m, 780w, 754w, 741w, 674w, 666w, 626w, 511m, 459w, 442w. The X-ray crystal structural data for the β-epimer is reported in the Crystallographic data section. The crystal was obtained from a mixture of epimers (α:β = 6:94, assigned by ^{1}H NMR analysis) *via* slow evaporation from a solution in EtOAc.

(3aS,4S,7S,7aS)-4-Mercapto-2,2,6,6-tetramethyltetrahydro-4H-[1,3]dioxolo[4,5-c]pyran-7-yl pivalate (17c)

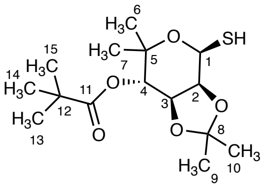

The procedure was adapted from Doyle *et al.* but using TMSBr for the acid and base sensitive starting material.[10] To a solution of (3a*S*,4*R*,7*S*,7a*S*)-4-acetoxy-2,2,6,6-tetramethyltetrahydro-4*H*-[1,3]dioxolo[4,5-*c*]pyran-7-yl pivalate **15c** (1.16 mmol, 400 mg, 1.0 equiv.) in anhydrous CH_2Cl_2 (3.8 mL, 0.3 M) at 0 °C in the dark was added dropwise TMSBr (3.48 mmol, 474 µL, 3.0 equiv.) and the mixture stirred at 0 °C for 7 h. After 7 h, the solvent was removed under reduced pressure (high vacuum) at 0 °C over 1 h. Due to the limited stability, the crude glycosyl bromide was used immediately in the next step without purification.

To the freshly prepared 2,3-*O*-isopropylidene-4-*O*-pivaloyl-D-noviopyranosyl bromide was then added at 0 °C a solution of thiourea (2.32 mmol, 177 mg, 2.0 equiv.) in anhydrous acetone (7.6 mL). The mixture was sparged with Ar for 2 min before it was heated to 60 °C and stirred at 60 °C under reflux for 16 h. After 16 h, the mixture was cooled to 25 °C and the solvent evaporated *in vacuo* to provide the crude thiouronium salt as a colorless to pale blue solid. The salt was used in the next step without purification.

The crude salt was suspended in a mixture of CH_2Cl_2 (7.6 mL) and water (5.0 mL, degassed by sparging with Ar), and sodium metabisulfite (1.63 mmol, 309 mg, 1.4 equiv.) was added. The mixture was sparged with Ar for 5 min and then heated to reflux (60 °C) and stirred under reflux for 3 h. Then, the mixture was cooled to 25 °C, diluted with CH_2Cl_2 (50 mL) and water (50 mL), and the layers were separated. The aqueous phase was extracted with CH_2Cl_2 (3×50 mL), the combined organic phase was washed with water (1×200 mL), dried over anhydrous $MgSO_4$, filtered, and the solvent removed *in vacuo* to provide the crude material as a colorless solid. The crude was purified *via* column chromatography on fine pore silica (Silica gel 60; 0.015-0.040 mm) using an eluent of EtOAc/pentane = 5:95 to provide the product **17c** as a colorless, crystalline solid in a yield of 18% (0.21 mmol, 68 mg) over 3 steps along with a mixture of epimers as a colorless, crystalline solid in a yield of 18% over 3 steps (0.20 mmol, 65 mg).

^{1}H NMR(500 MHz, $CDCl_3$): δ (ppm) 5.07 – 5.01 (m, 1H, H4), 4.98 – 4.92 (m, 1H, H1), 4.24 – 4.19 (m, 2H, H2, H3), 2.87 (d, *J*=10.7 Hz, 1H, SH), 1.58 (s, 3H, H9), 1.37 (s, 3H, H10), 1.30 (s, 3H, H7), 1.23 (s, 9H, H13, H14, H15), 1.19 (s, 3H, H6). **^{13}C NMR** (126 MHz, $CDCl_3$) δ (ppm) 177.2 (C11), 110.3 (C8), 76.9 (stacked with solvent signal, C5), 76.5 (C2), 75.2 (C3), 72.8 (C4), 71.1 (C1), 39.0 (C12), 28.2 (C7), 27.3 (C13, C14, C15), 27.2 (C9), 26.3 (C10), 19.8 (C6). **HRMS** ESI(+) (MeOH) calculated for $C_{15}H_{26}O_5NaS^+$ $[M+Na]^+$: 341.13932, found: 341.13958. **R*f*** (EtOAc/pentane = 1:9) = 0.5. **Melting point** m_p 60.5±0.5 °C. **Specific Rotation** $[\alpha]_D^{23\,°C}$ = –16.8 (c = 1.1, MeOH). **FT-IR** (solid): ν (cm^{-1}) 2980w, 2934w, 1724s, 1477w, 1457w, 1396w, 1379m, 1370m, 1300w, 1275m, 1238m, 1221m, 1197m, 1151s, 1127s, 1067s, 1035m, 1002m, 970m, 935w, 877m, 864m, 840w, 826w, 806w, 794m, 765w, 512w, 430w. The X-ray crystal structural data for the β-epimer is reported in the Crystallographic data section. The crystal was obtained from a mixture of epimers (α:β = 13:87, assigned by ^{1}H NMR analysis) *via* slow evaporation from a solution in EtOAc.

Synthesis of literature known compounds

1,2,3,4-Tetra-O-acetyl-α/β-L-fucopyranose[10,21,22]

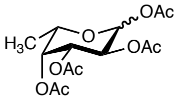

1,2,3,4-Tetra-*O*-acetyl-α-L-fucopyranose was synthesized after Doyle *et al.*[10] L-Fucose (18.3 mmol, 3.02 g, 1.0 equiv.) was dissolved in anhydrous pyridine (51 mL, 0.4 M), sparged with Ar for 5 min and then, cooled to 0 °C. At 0 °C, acetic anhydride (110 mmol, 10.3 mL, 6.0 equiv.) was added dropwise over 30 min. The mixture was allowed to slowly warm to 25 °C while stirring. After 24 h, the reaction mixture was poured onto an ice-water mixture (100 mL). CH_2Cl_2 (100 mL) was added, and the layers were separated. The aqueous layer was extracted with CH_2Cl_2 (1×100 mL), and the combined organic phase was washed with 1 M aqueous HCl solution (2×200 mL), saturated aqueous $NaHCO_3$ solution (1×200 mL), and water (1×200 mL). The organic phase was dried over anhydrous $MgSO_4$, filtered, and concentrated *in vacuo* to provide the crude material as a pale yellow oil. Purification *via* column chromatography using an eluent of pentane/EtOAc = 3:1 afforded the product after concentration *in vacuo* as a colorless oil in a yield of 85% (5.20 g, 15.6 mmol). The product was obtained as a mixture of epimers of α:β = 3:1 (assigned by integration in ^{1}H NMR spectrum (H1β: 5.66 (d, J = 8.3 Hz, 1H), H1α: 6.31 (d, J = 2.9 Hz, 1H); *Note*: Approx. 9% furanose formation was observed: H1β-furanose: 6.29 (d, *J*=4.7 Hz, 1H); H1α-furanose: 6.16 (s, 1H).[21]). The NMR spectroscopic data is consistent with previously reported data.[10,21,22] The mixture of epimers was used in the next step without further purification.

^{1}H NMR (400 MHz, $CDCl_3$) δ (ppm) 6.31 (d, *J* = 2.9 Hz, 1H, α), 5.66 (d, *J* = 8.3 Hz, 0.3H, β), 5.33 – 5.29 (m, 3H, α), 5.29 – 5.27 (m, 0.3H, β), 5.24 (dd, *J* = 3.5, 1.1 Hz, 0.3H, β), 5.05 (dd, *J* = 10.4, 3.5 Hz, 0.3H, β), 4.25 (q, *J* = 6.8 Hz, 1H, α), 3.99 – 3.89 (m, 0.3H, β), 2.16 (s, 0.9H, β), 2.15 (s, 3H, α), 2.12 (s, 3H, α), 2.09 (s, 0.9H, β), 2.01 (s, 0.9H, β), 1.99 (s, 3H, α), 1.98 (s, 3H, α), 1.97 (s, 0.9H, β), 1.20 (d, *J* = 6.5 Hz, 0.9H, β), 1.13 (d, *J* = 6.5 Hz, 3H, α). **^{13}C NMR** (126 MHz, $CDCl_3$) δ (ppm) 170.6α, 170.6β, 170.3α, 170.1β, 170.0α, 169.5β, 169.2β, 169.2α, 92.3β, 90.0α, 71.3β, 70.7α, 70.3β, 70.0β, 68.0β, 67.9α, 67.4α, 66.6α, 21.0α, 20.9β, 20.8α+β, 20.7β, 20.7α, 20.6α+β, 16.0α, 16.0β. **HRMS** ESI(+) (MeOH/$CHCl_3$ 3:2) calculated for $C_{14}H_{20}O_9Na^+$ $[M+Na]^+$: 355.09995, found: 355.10130.

2,3,4-Tri-O-acetyl-α-L-fucopyranosyl bromide[10]

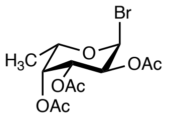

2,3,4-Tri-*O*-acetyl-α-L-fucopyranosyl bromide was synthesized after Doyle *et al.*[10] To a solution of 1,2,3,4-Tetra-*O*-acetyl-L-fucopyranose (mixture of epimers α:β = 3:1, 10.2 mmol, 3.39 g, 1.0 equiv.) in anhydrous CH_2Cl_2 (20 mL, 0.5 M) at 0 °C was added dropwise HBr (33% in AcOH, 30.6 mmol, 5.07 mL, 3.0 equiv.). The reaction mixture was allowed to warm to 25 °C while stirring for 3 h. After complete conversion of the starting material, the mixture was poured onto an ice-water mixture (100 mL), and the layers were separated. The aqueous phase was extracted with CH_2Cl_2 (3×100 mL), and the combined organic phase was washed with saturated aqueous $NaHCO_3$ solution (2×300 mL), and brine (1×300 mL), dried over anhydrous $MgSO_4$, filtered, and concentrated *in vacuo* to provide the product as a yellow oil in a yield of 95% (9.72 mmol, 3.43 g). The NMR spectroscopic data is consistent with previously reported data.[10]

^{1}H NMR (400 MHz, $CDCl_3$) δ (ppm) 6.68 (d, *J* = 3.9 Hz, 1H), 5.40 (dd, *J* = 10.6, 3.3 Hz, 1H), 5.35 (dd, *J* = 3.3, 1.3 Hz, 1H), 5.02 (dd, *J* = 10.5, 3.9 Hz, 1H), 4.40 (q, *J* = 6.6 Hz, 1H), 2.16 (s, 3H), 2.10 (s, 3H), 2.00 (s, 3H), 1.21 (d, *J* = 6.5 Hz, 3H). **^{13}C NMR** (101 MHz, $CDCl_3$) δ (ppm) 170.4, 170.3, 169.9, 89.4, 70.1, 69.9, 68.6, 68.0, 20.9, 20.8, 20.7, 15.7. **HRMS** ESI(+) (MeOH/$CHCl_3$ 3:2) calculated for $C_{12}H_{17}O_7^+$ $[M-Br^-]^+$: 273.09688, found: 273.09795.

2,3,4-Tri-O-acetyl-1-thio-α/β-L-fucopyranose (**6c**, **6d**)[10]

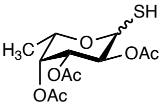

2,3,4-Tri-*O*-acetyl-1-thio-α/β-L-fucopyranose was synthesized after Doyle *et al.*[10] To a solution of 2,3,4-Tri-*O*-acetyl-α-L-fucopyranosyl bromide (8.60 mmol, 3.04 g, 1.0 equiv.) in anhydrous acetone (43.7 mL, 0.2 M) was added thiourea (14.6 mmol, 1.11 g, 1.7 equiv.). The mixture was sparged with Ar for 5 min and then heated to reflux (70 °C) and stirred under reflux for 14 h. After 14 h, the mixture was cooled to 25 °C and the solvent evaporated *in vacuo* to provide the crude thiouronium salt as a colorless solid. The salt was used in the next step without further purification. The crude salt was suspended in a mixture of CH_2Cl_2 (45 mL) and water (30 mL; degassed by sparging with Ar), and sodium metabisulfite (11.8 mmol, 2.25 g, 1.4 equiv.) was added. The mixture was sparged with Ar for 5 min and then heated to reflux (70 °C) and stirred under reflux for 2 h. The biphasic mixture was cooled to 25 °C, and the layers were separated. The aqueous phase was extracted with CH_2Cl_2 (3×30 mL) and the combined organic phase was washed with water (1×120 mL), dried over anhydrous $MgSO_4$, filtered, and concentrated *in vacuo* to provide the crude material as a yellow oil. The crude material was purified *via* column chromatography using an eluant of pentane/acetone = 8:2 to provide the α-anomer **6c** as a colorless oil in a yield of 7% (0.58 mmol, 177 mg) and the β-anomer **6d** as a colorless solid in a yield of 56% (4.81 mmol, 1.47 g). The NMR spectroscopic data is consistent with previously reported data.[10]

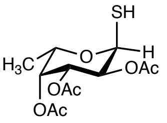

α

^{1}H NMR (400 MHz, $CDCl_3$) δ (ppm) 5.97 (t, *J* = 4.4 Hz, 1H), 5.30 (dd, *J* = 2.6, 1.3 Hz, 1H), 5.26 – 5.23 (m, 2H), 4.52 (q, *J* = 6.5 Hz, 1H), 2.16 (s, 3H), 2.08 (s, 3H), 2.00 (s, 3H), 1.78 (d, *J* = 5.1 Hz, 1H), 1.16 (d, *J* = 6.5 Hz, 3H). **^{13}C NMR** (101 MHz, $CDCl_3$) δ (ppm) 170.6, 170.2, 170.1, 77.8, 70.9, 68.1, 67.7, 65.6, 21.0, 20.8, 20.8, 16.0. **HRMS** ESI(+) (MeOH) calculated for $C_{12}H_{18}O_7NaS^+$ $[M+Na]^+$: 329.06654, found: 329.06678.

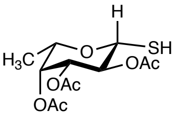

β

^{1}H NMR (400 MHz, $CDCl_3$) δ (ppm) 5.27 (dd, *J* = 3.5, 1.1 Hz, 1H), 5.15 (t, *J* = 9.8 Hz, 1H), 5.01 (dd, *J* = 10.1, 3.4 Hz, 1H), 4.49 (t, *J* = 9.8 Hz, 1H), 3.83 (qd, *J* = 6.4, 1.2 Hz, 1H), 2.32 (d, *J* = 9.9 Hz, 1H), 2.18 (s, 3H), 2.07 (s, 3H), 1.98 (s, 3H), 1.22 (d, *J* = 6.5 Hz, 3H). **^{13}C NMR** (101 MHz, $CDCl_3$) δ (ppm) 170.7, 170.2, 170.1, 79.0, 73.9, 72.1, 71.2, 70.5, 21.0, 20.8, 20.7, 16.5. **HRMS** ESI(+) (MeOH) calculated for $C_{12}H_{18}O_7NaS^+$ $[M+Na]^+$: 329.06654, found: 329.06691.

15R-THF Fdx (2a)[5]

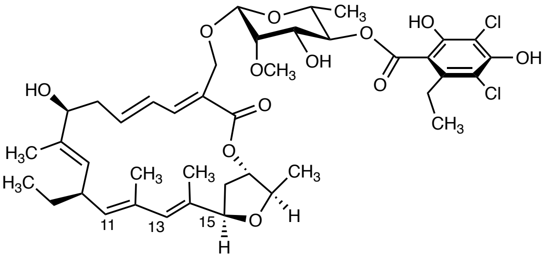

15R-THF Fdx **2a** was synthesized after Hattori *et al.*[5] To a solution of Fdx (94.5 µmol, 100 mg, 1.0 equiv.) in anhydrous CH_2Cl_2 (6 mL, 0.02 M) was added HCl (4 M solution in dioxane, 189 µmol, 47 µL, 2.0 equiv.) and the mixture stirred at 25 °C for 1 h. After complete conversion of the starting material, the reaction mixture was diluted with CH_2Cl_2 (30 mL) and brine (45 mL), and the aqueous phase extracted with CH_2Cl_2 (3×45 mL). The combined organic phase was dried over anhydrous $MgSO_4$, filtered, and concentrated *in vacuo* to provide the crude material. Purification was performed by preparative RP-HPLC (LC time program (time – %B) 0 min – 60%, 15 min – 60%, 75 min – 75%, 85 min – 75%, 86 min – 100, 96 min – 100% (Gemini, 250 mm × 21.2 mm, 5 µm, H_2O + 0.1% HCOOH (A) and MeCN + 0.1% HCOOH (B), flow rate of 20 mL/min) to provide the product as a colorless solid in a yield of 65% (0.06 mmol, 49.4 mg, t_R: 26.0 min). The NMR spectroscopic data is consistent with previously reported data.[5]

^{1}H NMR (500 MHz, acetone-d_6): δ (ppm) 7.05 (d, *J*=11.3 Hz, 1H), 6.55 (dd, *J*=15.1, 11.4 Hz, 1H), 6.01 (dt, *J*=14.8, 7.2 Hz, 1H), 5.94 (s, 1H), 5.37 (dt, *J*=10.5, 1.5 Hz, 1H), 5.13 (d, *J*=9.5 Hz, 1H), 5.10 (t, *J*=9.8 Hz, 1H), 4.98 (d, *J*=5.6 Hz, 1H), 4.65 (s, 1H), 4.60 (d, *J*=9.1 Hz, 1H),

4.58 (d, *J*=11.4 Hz, 1H), 4.42 (d, *J*=11.4 Hz, 1H), 4.28 (t, *J*=3.7 Hz, 1H), 4.23 (q, *J*=6.6 Hz, 1H), 3.82 (dd, *J*=9.9, 3.4 Hz, 1H), 3.62 – 3.54 (m, 2H), 3.51 (s, 3H), 3.23 (tt, *J*=9.7, 7.1 Hz, 1H), 3.00 (q, *J*=7.3 Hz, 2H), 2.62 (dddd, *J*=15.2, 7.1, 3.5, 1.4 Hz, 1H), 2.50 – 2.42 (m, 2H), 2.12 (dd, *J*=14.2, 1.7 Hz, 1H), 1.78 (s, 3H), 1.67 (s, 3H), 1.64 (s, 3H), 1.50 – 1.35 (m, 2H), 1.30 (d, *J*=6.2 Hz, 3H), 1.21 (t, *J*=7.3 Hz, 3H), 1.21 (d, *J*=6.6 Hz, 3H), 0.88 (t, *J*=7.4 Hz, 3H).
^{13}C NMR (126 MHz, acetone-d_6) δ (ppm) 169.5, 167.0, 155.9, 153.9, 145.7, 143.9, 142.6, 136.0, 133.5, 133.3, 131.1, 128.1, 127.5, 125.9, 125.0, 114.6, 110.5, 108.2, 101.5, 82.1, 81.8, 81.7, 80.3, 77.6, 72.9, 72.3, 70.6, 62.9, 61.7, 40.1, 38.1, 35.5, 29.5, 26.2, 20.0, 18.7, 18.2, 16.0, 15.2, 14.4, 12.3. **HRMS** ESI(+) (MeOH/$CHCl_3$ 3:2) calculated for $C_{41}H_{58}Cl_2NO_{12}^+$ $[M+NH_4]^+$: 826.33306, found: 826.33353.

Crystallographic data

Single crystal X-ray diffraction data of compounds **13**, **14**, **15b**, **17a**, **17b** and **17c** were collected at 160.0(1) K on a Rigaku OD Synergy/Hypix diffractometer **(13, 14, 15b, 17a**) or on a Rigaku OD Synergy/Pilatus diffractometer **(17b, 17c**) using the copper X-ray radiation (λ = 1.54184 Å) from a dual wavelength X-ray source and an Oxford Instruments Cryojet XL cooler. The selected suitable single crystals were mounted using polybutene oil on a flexible loop fixed on a goniometer head and immediately transferred to the diffractometer. Pre-experiments, data collections, data reductions and analytical absorption corrections[23] were performed with the program suite *CrysAlisPro*.[24] Using *Olex2*,[25] the structures were solved with the *SHELXT*[26] small molecule structure solution program and refined with the *SHELXL* program package[27] by full-matrix least-squares minimization on F^2. *PLATON*[28] was used to check the results of the X-ray analyses. The data collection parameters, refinement parameters and displacement ellipsoid plots are given in Tables **6-8**.Supplementary crystallographic data for this paper have been deposited at the Cambridge Crystallographic Data Center, under the deposition numbers CCDC 2333101 (**13**), 2333102 (**14**), 2333103 (**15b**), 2333104 (**17a**), 2333105 (**17b**), 2333106 (**17c**). These data can be obtained free of charge *via* www.ccdc.cam.ac.uk/structures.

The chiral compound **13** crystallized in the Sohncke space group $P2_1$. There are two independent molecules in the asymmetric unit. The structure was refined as a two-component twin: the component 2 is rotated by 180° around the reciprocal lattice vector: (7 0 9). The integral twin law (-0.007 0 0.996 0 -1 0 1.004 0 0.007) was handled by the refinement program with the *SHELXL* commands *TWIN* and *BASF*. The absolute configuration was reliably determined by the Flack parameter x = -0.011(6). The chiral compound **14** crystallized in the Sohncke space group $P2_12_12$. The absolute configuration was reliably determined by the Flack parameter x = 0.04(7). The chiral compound **15b** crystallized in the Sohncke space group $P2_1$. The absolute configuration was reliably determined by the Flack parameter x = 0.01(6). The chiral compound **17a** crystallized in the Sohncke space group $P2_1$. The absolute configuration was reliably determined by the Flack parameter x = -0.002(10). The C=O group is disordered over two sets of positions with site-occupancy factors of 0.280(17) and 0.720(17). Compound **17b** crystallized in the Sohncke space group $P2_12_12_1$. The absolute configuration was reliably determined by the Flack parameter x = -0.023(11). The chiral compound **17c** crystallized in the Sohncke space group $C2$. The absolute configuration was reliably determined by the Flack parameter x = 0.02(4). The crystals appeared to be flat plates giving rise to moderate to weak diffraction. The quality of diffraction was also affected by twinning, but a cut was not possible. Despite its relatively bad quality, especially in comparison with the other crystal structures reported herein, the crystal structure of **17c** is included in the manuscript for characterization purposes.

Table 6: Crystallographic data for compounds 13 and 14

	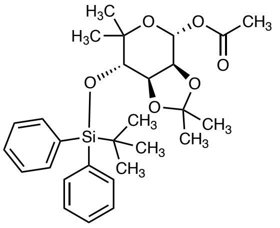 **13**	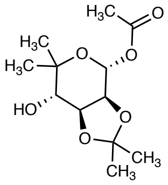 **14**
Molecular structure	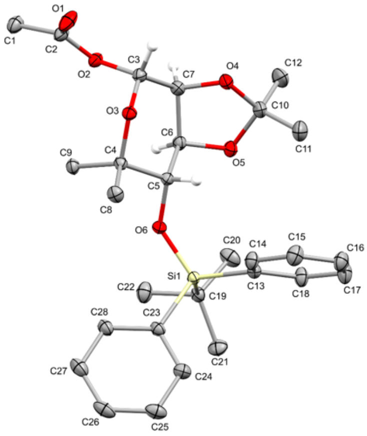	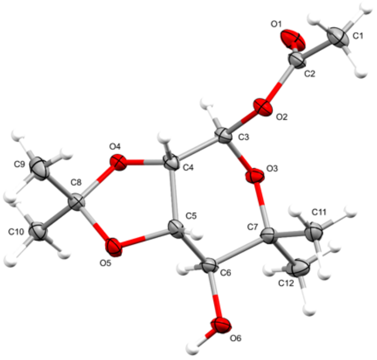
Empirical formula	$C_{28}H_{38}O_6Si$	$C_{12}H_{20}O_6$
Formula weight [g/mol]	498.67	260.28
Temperature [K]	160.0(1)	160.0(1)
Crystal system	monoclinic	orthorhombic
Space group	$P2_1$	$P2_12_12$
Unit cell parameters:		
a [Å]	9.98325(6)	18.3045(2)
b [Å]	28.91324(16)	12.9361(2)
c [Å]	10.00713(6)	5.64330(10)
α [°]	90	90
β [°]	105.9185(6)	90
γ [°]	90	90
Volume [Å^3]	2777.77(3)	1336.27(3)
Z	4	4
ρ_{calc} [g/cm^3]	1.192	1.294
μ [mm^{-1}]	1.056	0.873
F(000)	1072.0	560.0
Crystal size [mm^3]	0.28 × 0.22 × 0.12	0.17 × 0.09 × 0.03
Radiation	Cu Kα (λ = 1.54184)	Cu Kα (λ = 1.54184)
2Θ range for data collection [°]	6.114 to 154.702	8.37 to 154.56
Index ranges	$-12 \le h \le 12$, $-36 \le k \le 34$, $-12 \le l \le 12$	$-19 \le h \le 22$, $-16 \le k \le 16$, $-7 \le l \le 6$
Reflections collected	58429	19357
Independent reflections	11715 [R_{int} = 0.0297, R_{sigma} = 0.0182]	2825 [R_{int} = 0.0345, R_{sigma} = 0.0181]
Data/restraints/parameters	11715/1/649	2825/0/172
Goodness-of-fit on F^2	1.047	1.063
Final R indexes [I>=2σ (I)]	R_1 = 0.0275, wR_2 = 0.0760	R_1 = 0.0280, wR_2 = 0.0667
Final R indexes [all data]	R_1 = 0.0275, wR_2 = 0.0760	R_1 = 0.0310, wR_2 = 0.0703
Largest diff. peak/hole [e Å^{-3}]	0.41/-0.17	0.16/-0.16
Flack parameter	-0.011(6)	0.04(7)

Table 7: Crystallographic data for compounds 15b and 17a

	15b	17a
	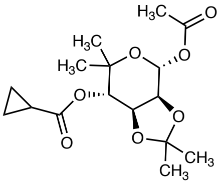	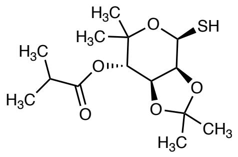
Molecular structure	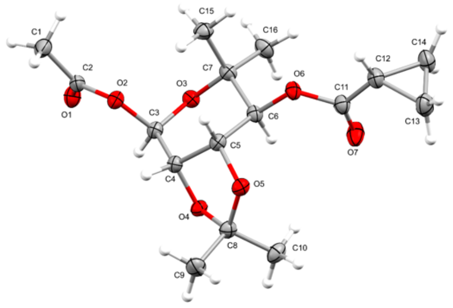	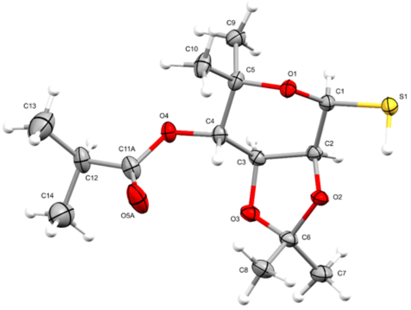
Empirical formula	$C_{16}H_{24}O_7$	$C_{14}H_{24}O_5S$
Formula weight [g/mol]	328.35	304.39
Temperature [K]	160.0(1)	160.0(1)
Crystal system	monoclinic	monoclinic
Space group	$P2_1$	$P2_1$
Unit cell parameters:		
a [Å]	5.66530(10)	8.1344(2)
b [Å]	8.53630(10)	5.46690(10)
c [Å]	17.77240(10)	18.7103(4)
α [°]	90	90
β [°]	92.8740(10)	98.803(2)
γ [°]	90	90
Volume [Å^3]	858.405(19)	822.24(3)
Z	2	2
ρ_{calc} [g/cm^3]	1.270	1.229
μ [mm^{-1}]	0.835	1.889
F(000)	352.0	328.0
Crystal size [mm^3]	0.21 × 0.13 × 0.07	0.24 × 0.05 × 0.02
Radiation	Cu Kα (λ = 1.54184)	Cu Kα (λ = 1.54184)
2Θ range for data collection [°]	4.978 to 148.882	4.78 to 148.682
Index ranges	$-7 \leq h \leq 7$, $-10 \leq k \leq 10$, $-22 \leq l \leq 19$	$-10 \leq h \leq 7$, $-6 \leq k \leq 6$, $-23 \leq l \leq 23$
Reflections collected	17973	19782
Independent reflections	3489 [R_{int} = 0.0264, R_{sigma} = 0.0166]	3338 [R_{int} = 0.0447, R_{sigma} = 0.0252]
Data/restraints/parameters	3489/1/214	3338/91/210
Goodness-of-fit on F^2	1.043	1.045
Final R indexes [I>=2σ (I)]	R_1 = 0.0267, wR_2 = 0.0713	R_1 = 0.0310, wR_2 = 0.0815
Final R indexes [all data]	R_1 = 0.0269, wR_2 = 0.0715	R_1 = 0.0322, wR_2 = 0.0822
Largest diff. peak/hole [e Å^{-3}]	0.15/-0.15	0.19/-0.23
Flack parameter	0.01(6)	-0.002(10)

Table 8: Crystallographic data for compounds 17b and 17c

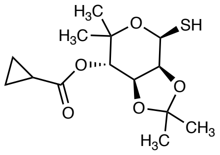

17b

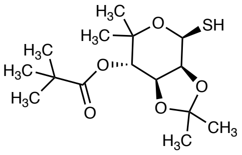

17c

Molecular structure

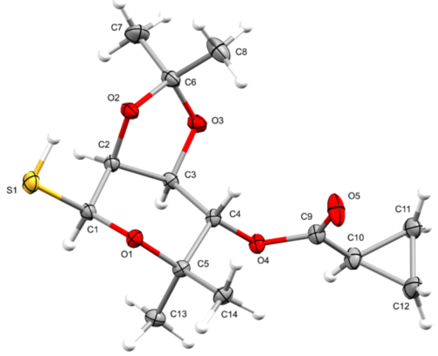

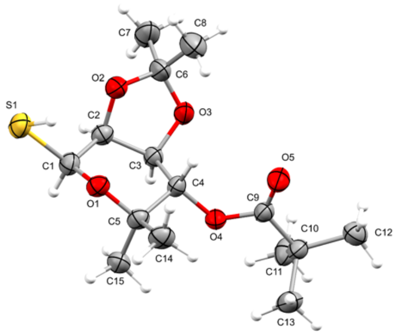

	17b	17c
Empirical formula	$C_{14}H_{22}O_5S$	$C_{15}H_{26}O_5S$
Formula weight [g/mol]	302.37	318.42
Temperature [K]	160.0(1)	160.0(1)
Crystal system	orthorhombic	monoclinic
Space group	$P2_12_12_1$	C2
Unit cell parameters:		
a [Å]	5.4779(2)	16.1776(9)
b [Å]	13.4791(4)	10.9250(5)
c [Å]	21.0555(6)	21.0953(14)
α [°]	90	90
β [°]	90	109.647(7)
γ [°]	90	90
Volume [Å^3]	1554.68(9)	3511.3(4)
Z	4	8
ρ_{calc} [g/cm^3]	1.292	1.205
μ [mm^{-1}]	1.997	1.790
F(000)	648.0	1376.0
Crystal size [mm^3]	0.23 × 0.11 × 0.03	0.22 × 0.08 × 0.04
Radiation	Cu Kα (λ = 1.54184)	Cu Kα (λ = 1.54184)
2Θ range for data collection [°]	7.788 to 152.834	4.448 to 136.496
Index ranges	$-6 \le h \le 6$, $-10 \le k \le 16$, $-26 \le l \le 26$	$-19 \le h \le 18$, $-13 \le k \le 12$, $-23 \le l \le 25$
Reflections collected	16048	30819
Independent reflections	3236 [R_{int} = 0.0450, R_{sigma} = 0.0320]	6365 [R_{int} = 0.0583, R_{sigma} = 0.0441]
Data/restraints/parameters	3236/0/189	6365/1/399
Goodness-of-fit on F^2	1.049	1.132
Final R indexes [I>=2σ (I)]	R_1 = 0.0355, wR_2 = 0.0873	R_1 = 0.1027, wR_2 = 0.2830
Final R indexes [all data]	R_1 = 0.0393, wR_2 = 0.0908	R_1 = 0.1356, wR_2 = 0.3082
Largest diff. peak/hole [e Å^{-3}]	0.32/-0.22	0.75/-0.25
Flack parameter	-0.023(11)	0.02(4)

Supplementary references

(1) Mestrelab Research S.L. MestReNova, **2020**.
(2) Fulmer, G. R.; Miller, A. J. M.; Sherden, N. H.; Gottlieb, H. E.; Nudelman, A.; Stoltz, B. M.; Bercaw, J. E.; Goldberg, K. I. NMR Chemical Shifts of Trace Impurities: Common Laboratory Solvents, Organics, and Gases in Deuterated Solvents Relevant to the Organometallic Chemist. *Organometallics* **2010**, *29* (9), 2176–2179. https://doi.org/10.1021/om100106e.
(3) Thermo Fisher Scientific Inc. Qual Browser. Thermo Xcalibur 3.0.41., **2013**.
(4) Selvi, B. A.; Hlaing, Y. CS.; Infante, K.; Kaner, M.; Gualano, M.; Patel, D.; Babayeva, M. Physicochemical Characterization, Solubilization, and Stabilization of a Macrolide Antibiotic. *J. Drug Deliv. Sci. Technol.* **2020**, *57*, 101755. https://doi.org/10.1016/j.jddst.2020.101755.
(5) Hattori, H.; Kaufmann, E.; Miyatake-Ondozabal, H.; Berg, R.; Gademann, K. Total Synthesis of Tiacumicin A. Total Synthesis, Relay Synthesis, and Degradation Studies of Fidaxomicin (Tiacumicin B, Lipiarmycin A3). *J. Org. Chem.* **2018**, *83* (13), 7180–7205. https://doi.org/10.1021/acs.joc.8b00101.
(6) GraphPad Software, LLC. Prism 10 for Windows 64-Bit, **2023**. https://www.graphpad.com.
(7) Microsoft Corporation. Microsoft Excel for Mac, **2023**. https://office.microsoft.com/excel.
(8) *CLSI. Methods for Antimicrobial Susceptibility Testing of Anaerobic Bacteria; 9th Ed. CLSI Standard M11.*; CLSI: 950 West Valley Road, Suite 2500, Wayne, Pennsylvania 19087 USA, **2018**.
(9) *CLSI. Performance Standards for Antimicrobial Susceptibility Testing. 33rd Ed. CLSI Supplement M100.*; CLSI: 950 West Valley Road, Suite 2500, Wayne, Pennsylvania 19087 USA, **2023**.
(10) Doyle, L. M.; O'Sullivan, S.; Di Salvo, C.; McKinney, M.; McArdle, P.; Murphy, P. V. Stereoselective Epimerizations of Glycosyl Thiols. *Org. Lett.* **2017**, *19* (21), 5802–5805. https://doi.org/10.1021/acs.orglett.7b02760.
(11) Ionescu, C.; Sippelli, S.; Toupet, L.; Barragan-Montero, V. New Mannose Derivatives: The Tetrazole Analogue of Mannose-6-Phosphate as Angiogenesis Inhibitor. *Bioorg. Med. Chem. Lett.* **2016**, *26* (2), 636–639. https://doi.org/10.1016/j.bmcl.2015.11.059.
(12) Skaanderup, P. R.; Poulsen, C. S.; Hyldtoft, L.; Jørgensen, M. R.; Madsen, R. Regioselective Conversion of Primary Alcohols into Iodides in Unprotected Methyl Furanosides and Pyranosides. *Synthesis* **2002**, *2002* (12), 1721–1727. https://doi.org/10.1055/s-2002-33641.
(13) Suthagar, K.; Fairbanks, A. J. A New Way to Do an Old Reaction: Highly Efficient Reduction of Organic Azides by Sodium Iodide in the Presence of Acidic Ion Exchange Resin. *Chem. Commun.* **2017**, *53* (4), 713–715. https://doi.org/10.1039/C6CC08574A.
(14) Traboni, S.; Bedini, E.; Giordano, M.; Iadonisi, A. One-Pot Synthesis of Orthogonally Protected Sugars through Sequential Base-Promoted/Acid-Catalyzed Steps: A Solvent-Free Approach with Self-Generation of a Catalytic Species. *Tetrahedron Lett.* **2019**, *60* (27), 1777–1780. https://doi.org/10.1016/j.tetlet.2019.05.066.
(15) Kumamoto, H.; Deguchi, K.; Wagata, T.; Furuya, Y.; Odanaka, Y.; Kitade, Y.; Tanaka, H. Radical-Mediated Stannylation of Vinyl Sulfones: Access to Novel 4′-Modified Neplanocin A Analogues. *Tetrahedron* **2009**, *65* (38), 8007–8013. https://doi.org/10.1016/j.tet.2009.07.039.
(16) Ghosal, P.; Shaw, A. K. A Chiron Approach to Aminocytitols by Petasis-Borono-Mannich Reaction: Formal Synthesis of (+)-Conduramine E and (−)-Conduramine E. *J. Org. Chem.* **2012**, *77* (17), 7627–7632. https://doi.org/10.1021/jo300804d.

(17) Matzner, D.; Schüller, A.; Seitz, T.; Wittmann, V.; Mayer, G. Fluoro-Carba-Sugars Are Glycomimetic Activators of the *glmS* Ribozyme. *Chem. - Eur. J.* **2017**, *23* (51), 12604–12612. https://doi.org/10.1002/chem.201702371.
(18) Chrétien, F. A Convenient Preparation of Hex-5,6-Enopyranosides. *Synth. Commun.* **1989**, *19* (5–6), 1015–1024. https://doi.org/10.1080/00397918908051023.
(19) Hedberg, C.; Estrup, M.; Eikeland, E. Z.; Jensen, H. H. Vinyl Grignard-Mediated Stereoselective Carbocyclization of Lactone Acetals. *J. Org. Chem.* **2018**, *83* (4), 2154–2165. https://doi.org/10.1021/acs.joc.7b03079.
(20) Scheidt, K. A.; Chen, H.; Follows, B. C.; Chemler, S. R.; Coffey, D. S.; Roush, W. R. Tris(Dimethylamino)Sulfonium Difluorotrimethylsilicate, a Mild Reagent for the Removal of Silicon Protecting Groups. *J. Org. Chem.* **1998**, *63* (19), 6436–6437. https://doi.org/10.1021/jo981215i.
(21) Ruttens, B.; Kováč, P. A Facile Synthesis of Armed and Disarmed Colitose Thioglycosides. *Synthesis* **2004**, *2004* (15), 2505–2508. https://doi.org/10.1055/s-2004-831206.
(22) Ichikawa, Y.; Sim, M. M.; Wong, C. H. Efficient Chemical Synthesis of GDP-Fucose. *J. Org. Chem.* **1992**, *57* (10), 2943–2946. https://doi.org/10.1021/jo00036a036.
(23) Clark, R. C.; Reid, J. S. The Analytical Calculation of Absorption in Multifaceted Crystals. *Acta Crystallogr. Sect. A* **1995**, *51* (6), 887–897. https://doi.org/10.1107/S0108767395007367.
(24) CrysAlisPro (Version 1.171.42.75a-100a), Rigaku Oxford Diffraction Ltd, Yarnton, Oxfordshire, England (**2022-2023**).
(25) Dolomanov, O. V.; Bourhis, L. J.; Gildea, R. J.; Howard, J. A. K.; Puschmann, H. OLEX2: A Complete Structure Solution, Refinement and Analysis Program. *J. Appl. Crystallogr.* **2009**, *42* (2), 339–341. https://doi.org/10.1107/S0021889808042726.
(26) Sheldrick, G. M. SHELXT – Integrated Space-Group and Crystal-Structure Determination. *Acta Crystallogr. Sect. A* **2015**, *71* (1), 3–8. https://doi.org/10.1107/S2053273314026370.
(27) Sheldrick, G. M. Crystal Structure Refinement with SHELXL. *Acta Crystallogr. Sect. C* **2015**, *71* (1), 3–8. https://doi.org/10.1107/S2053229614024218.
(28) Spek, A. L. Structure Validation in Chemical Crystallography. *Acta Crystallogr. Sect. D* **2009**, *65* (2), 148–155. https://doi.org/10.1107/S090744490804362X.

Spectra

Spectral data for Fdx derivatives from proof of concept studies

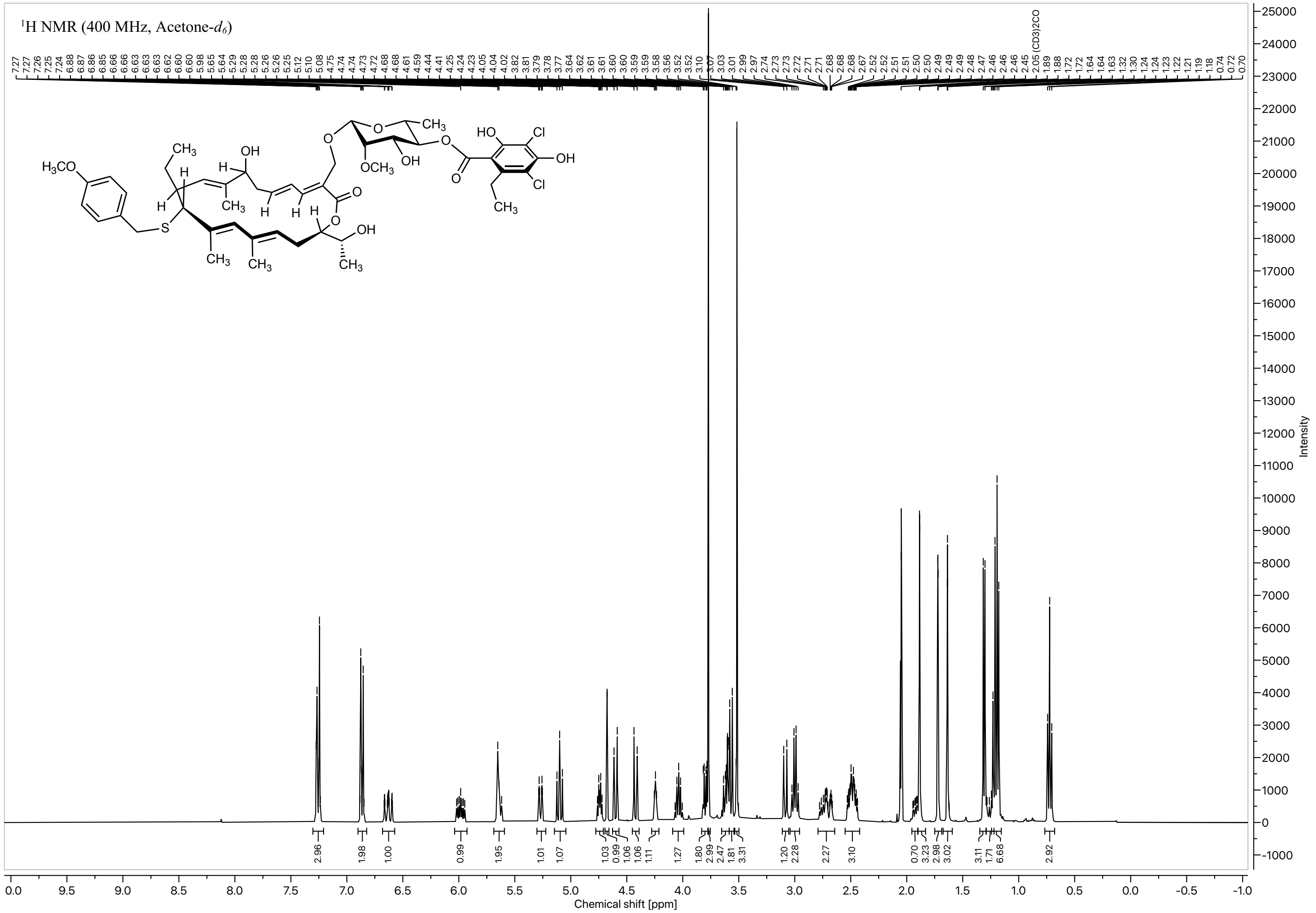

Figure 4: ^{1}H NMR spectrum of 11-desnoviosyl-11-*p*-methoxybenzylsulfide fidaxomicin (5a-C(11)) in acetone-d_6

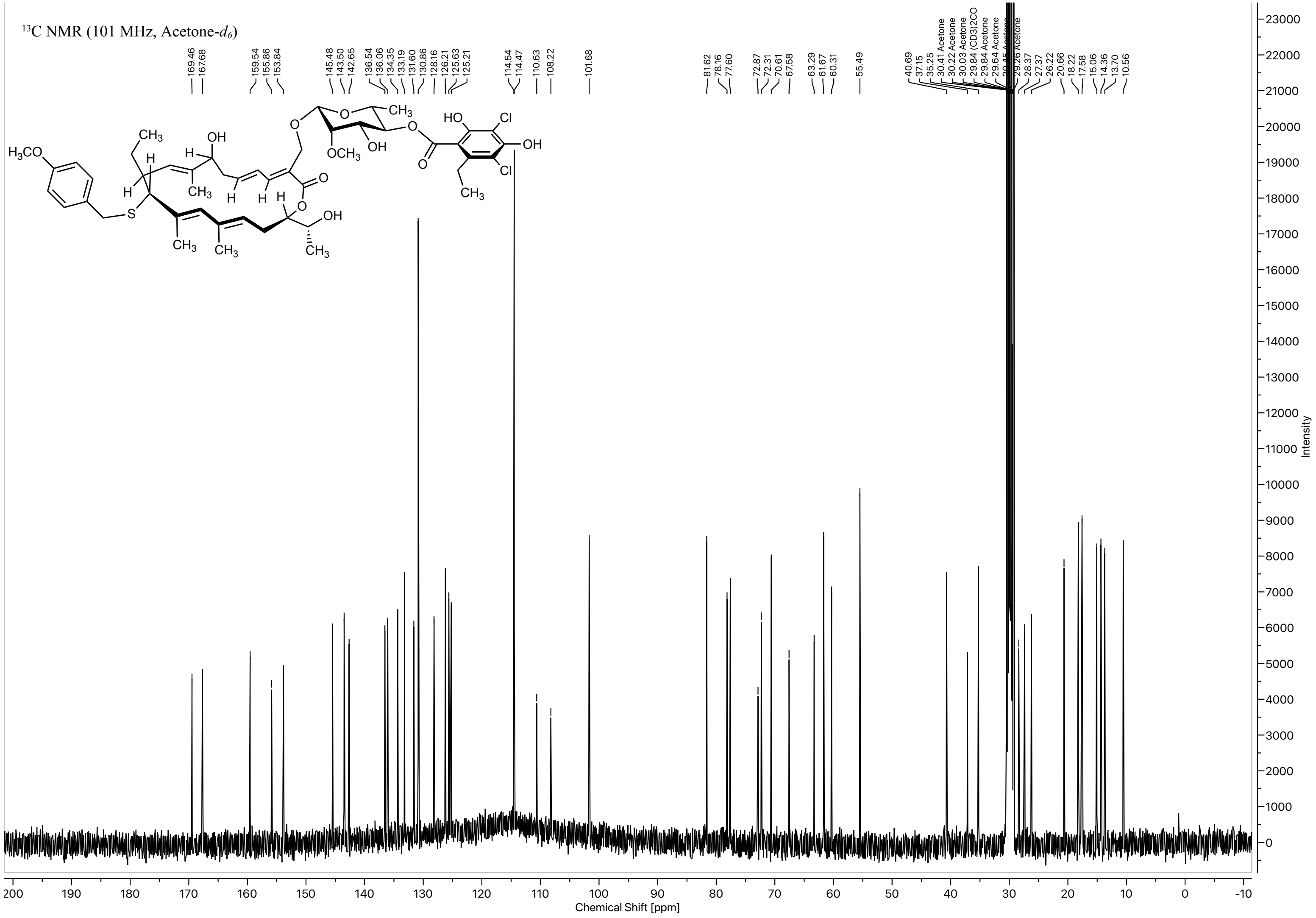

Figure 5: ^{13}C NMR spectrum of 11-desnoviosyl-11-*p*-methoxybenzylsulfide fidaxomicin (5a-C(11)) in acetone-d_6

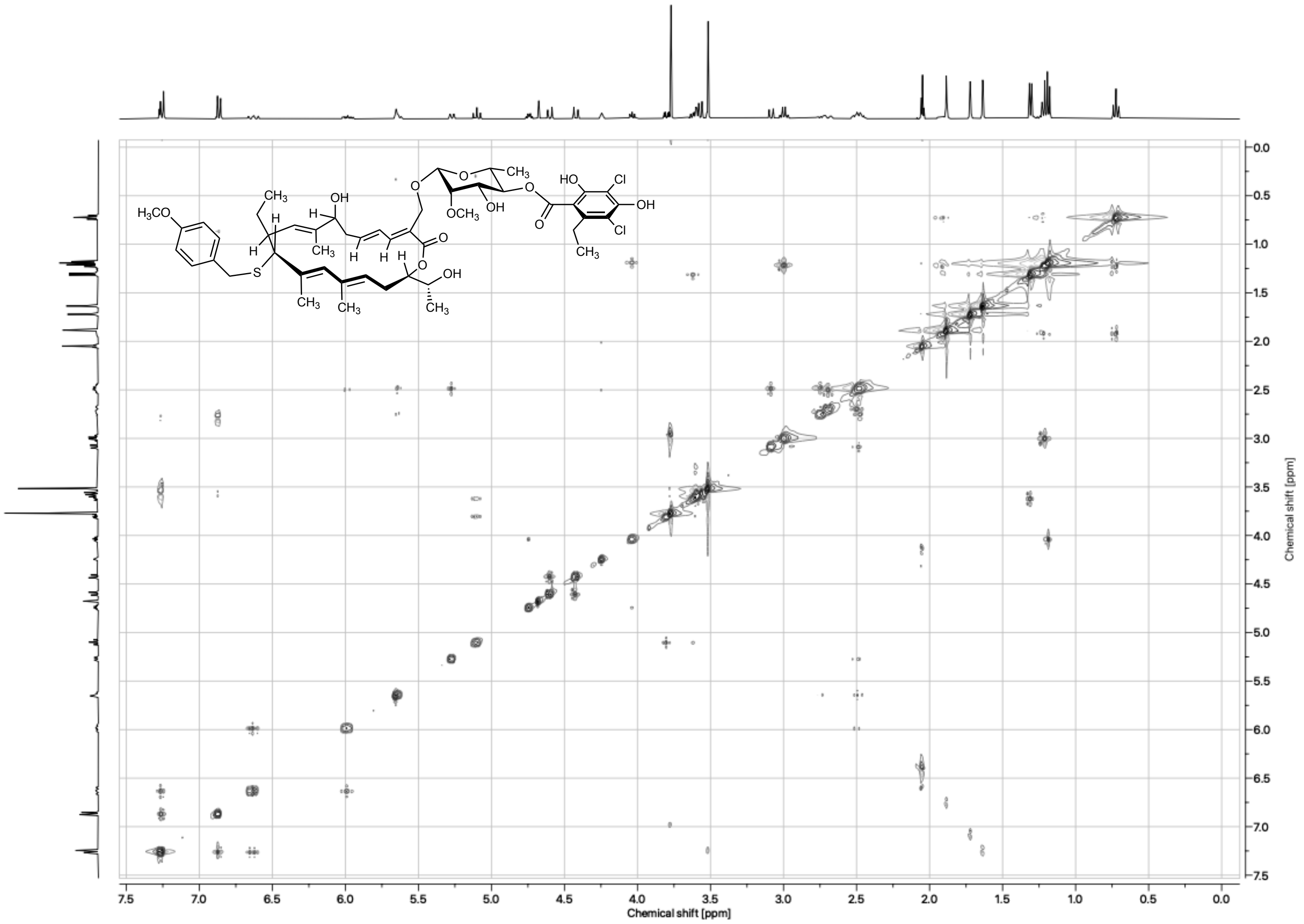

Figure 6: COSY spectrum of 11-desnoviosyl-11-*p*-methoxybenzylsulfide fidaxomicin (5a-C(11)) in acetone-d_6

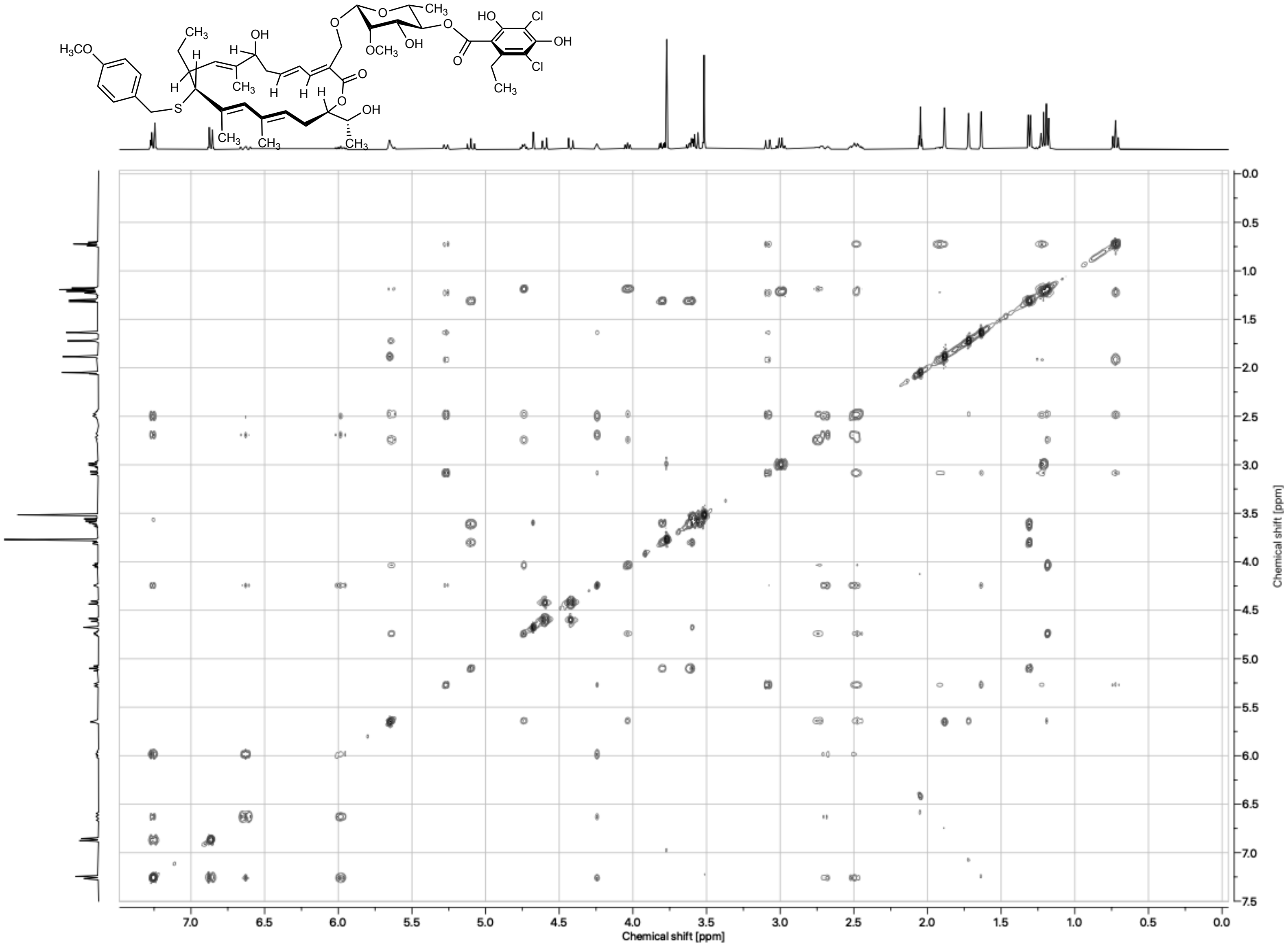

Figure 7: TOCSY spectrum of 11-desnoviosyl-11-*p*-methoxybenzylsulfide fidaxomicin (5a-C(11)) in acetone-d_6

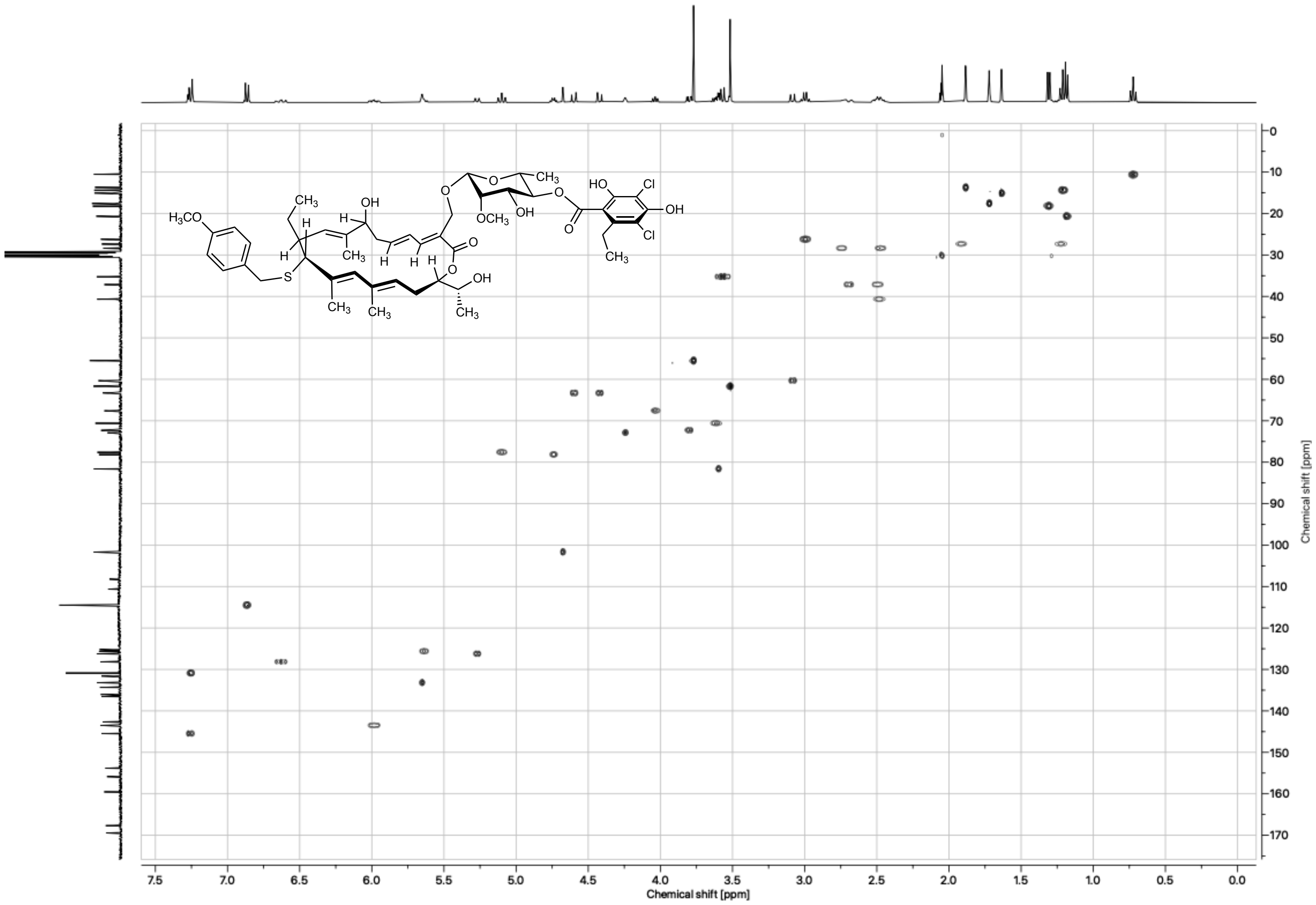

Figure 8: HSQC spectrum of 11-desnoviosyl-11-*p*-methoxybenzylsulfide fidaxomicin (5a-C(11)) in acetone-d_6

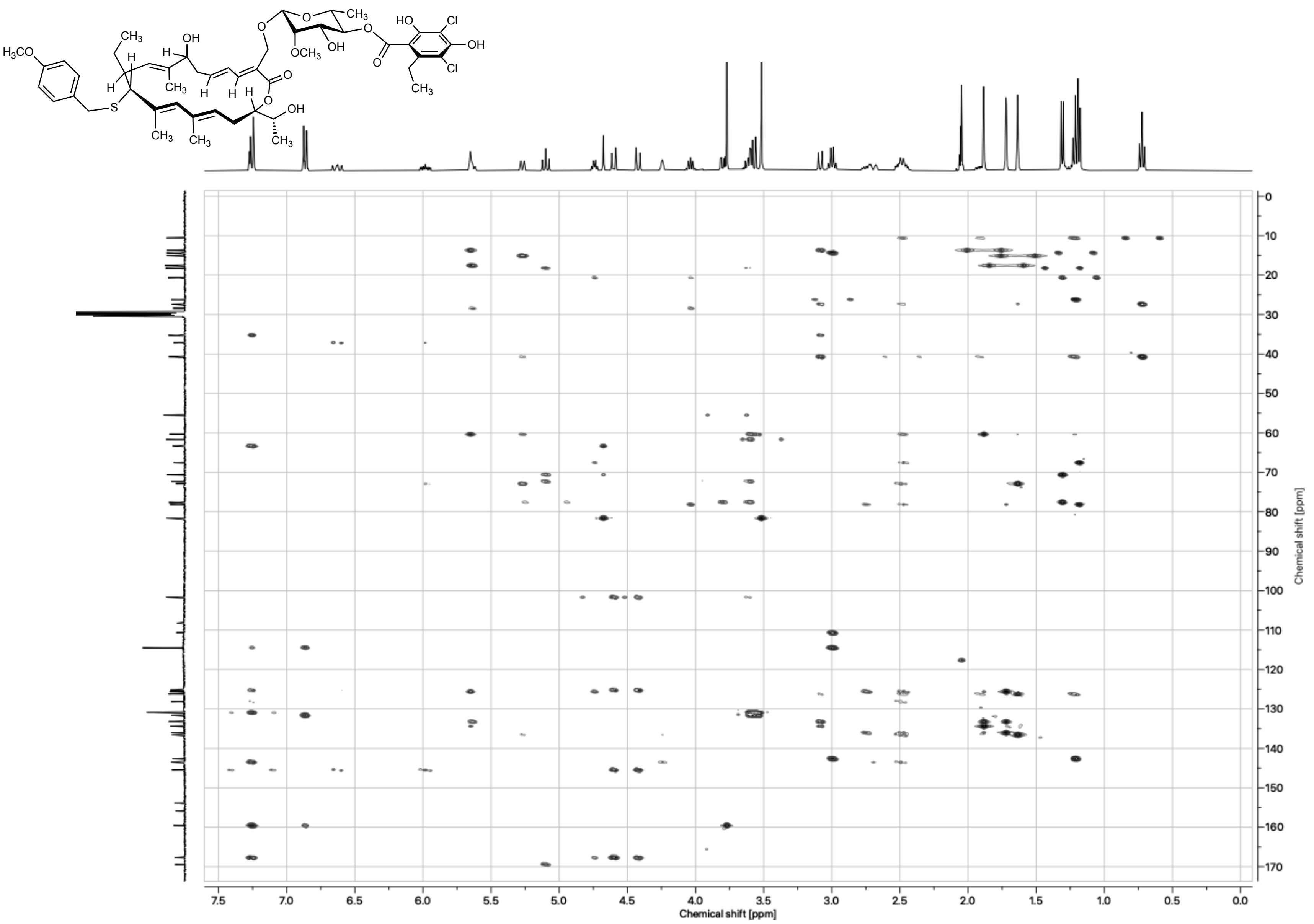

Figure 9: HMBC spectrum of 11-desnoviosyl-11-*p*-methoxybenzylsulfide fidaxomicin (5a-C(11)) in acetone-d_6

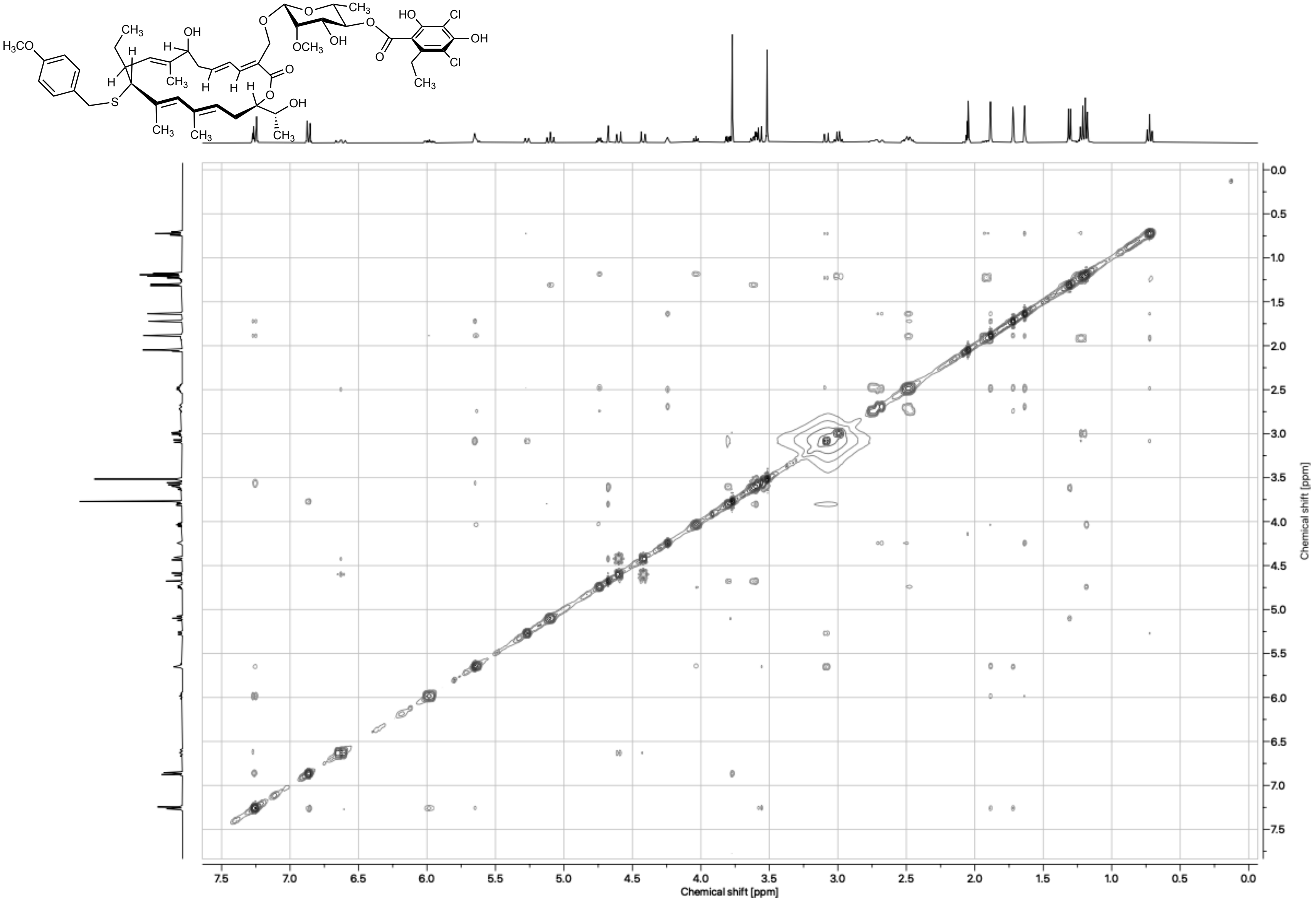

Figure 10: NOESY spectrum of 11-desnoviosyl-11-*p*-methoxybenzylsulfide fidaxomicin (5a-C(11)) in acetone-d_6

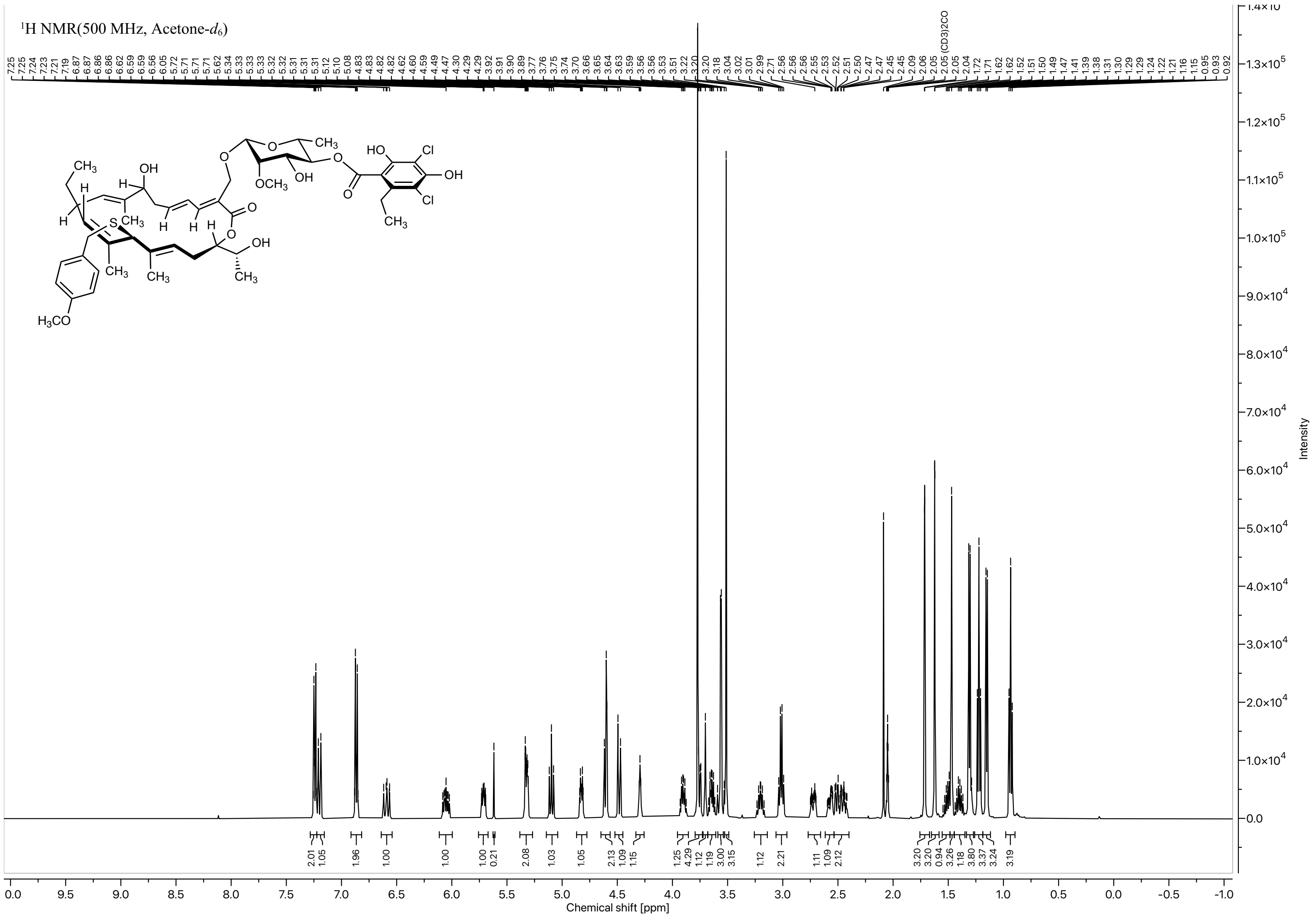

Figure 11: ^{1}H NMR spectrum of 11-desnoviosyl-13-*p*-methoxybenzylsulfide fidaxomicin (5a-C(13)) in acetone-d_6

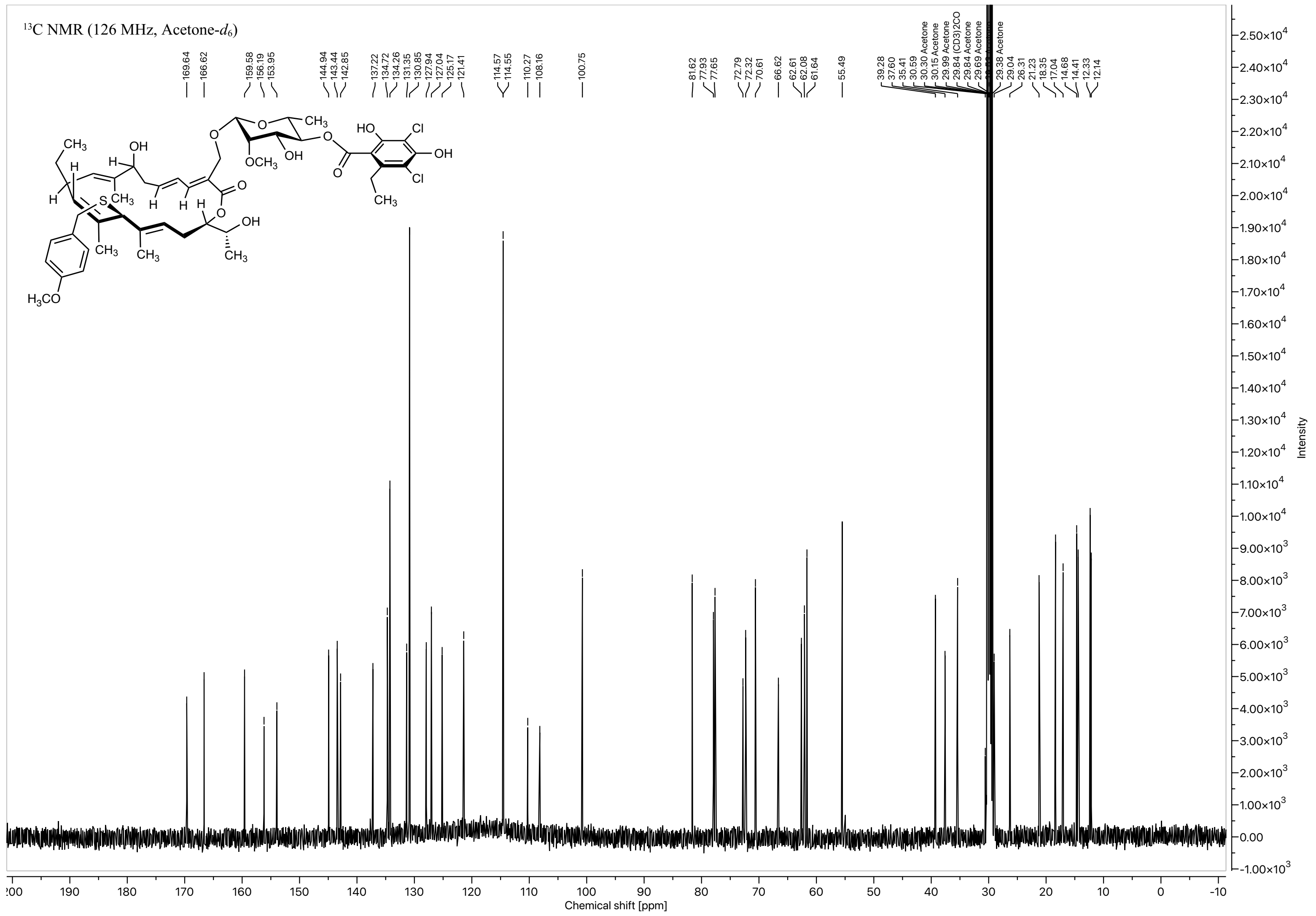

Figure 12: ^{13}C NMR spectrum of 11-desnoviosyl-13-*p*-methoxybenzylsulfide fidaxomicin (5a-C(13)) in acetone-d_6

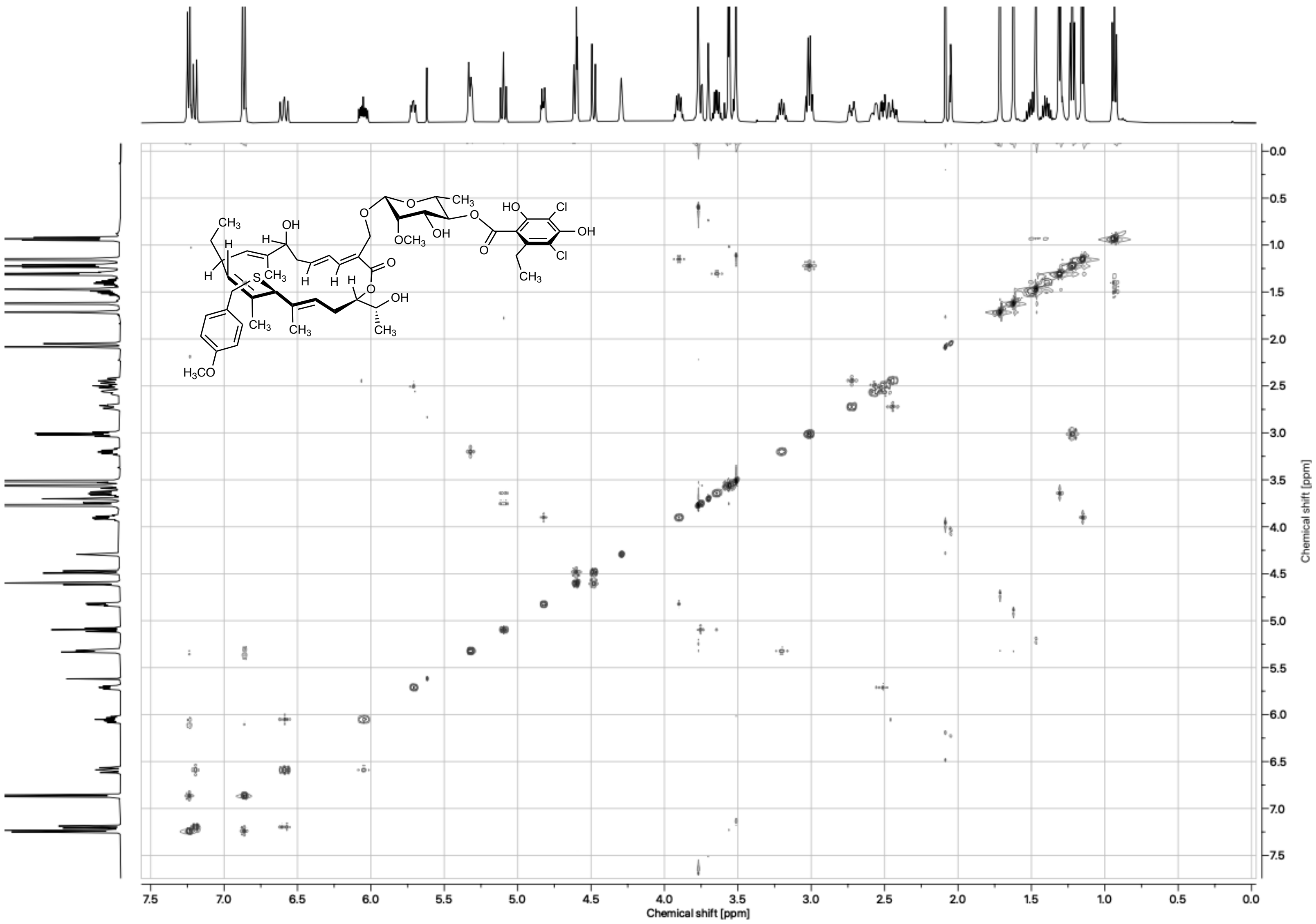

Figure 13: COSY spectrum of 11-desnoviosyl-13-*p*-methoxybenzylsulfide fidaxomicin (5a-C(13)) in acetone-d_6

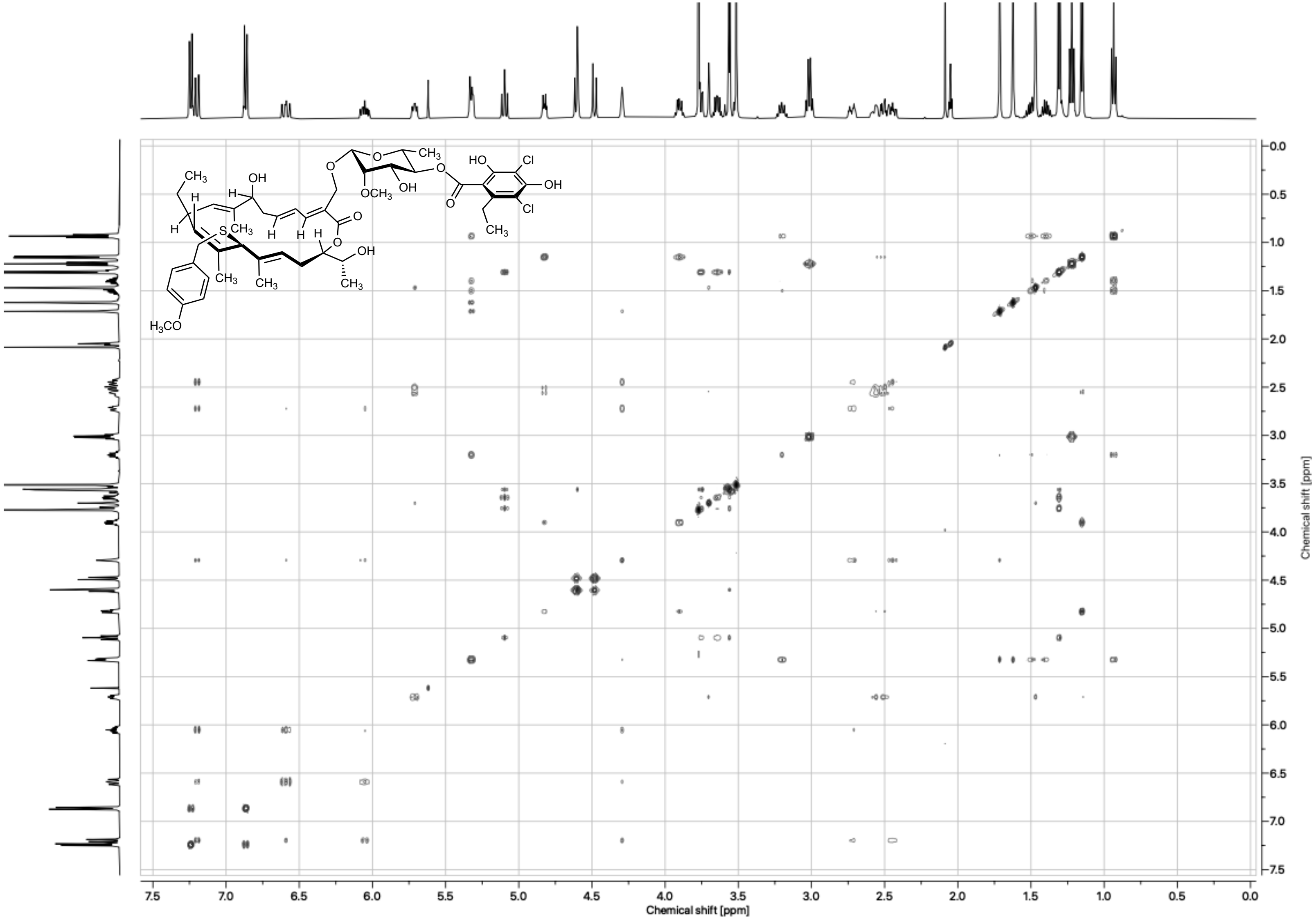

Figure 14: TOCSY spectrum of 11-desnoviosyl-13-*p*-methoxybenzylsulfide fidaxomicin (5a-C(13)) in acetone-d_6

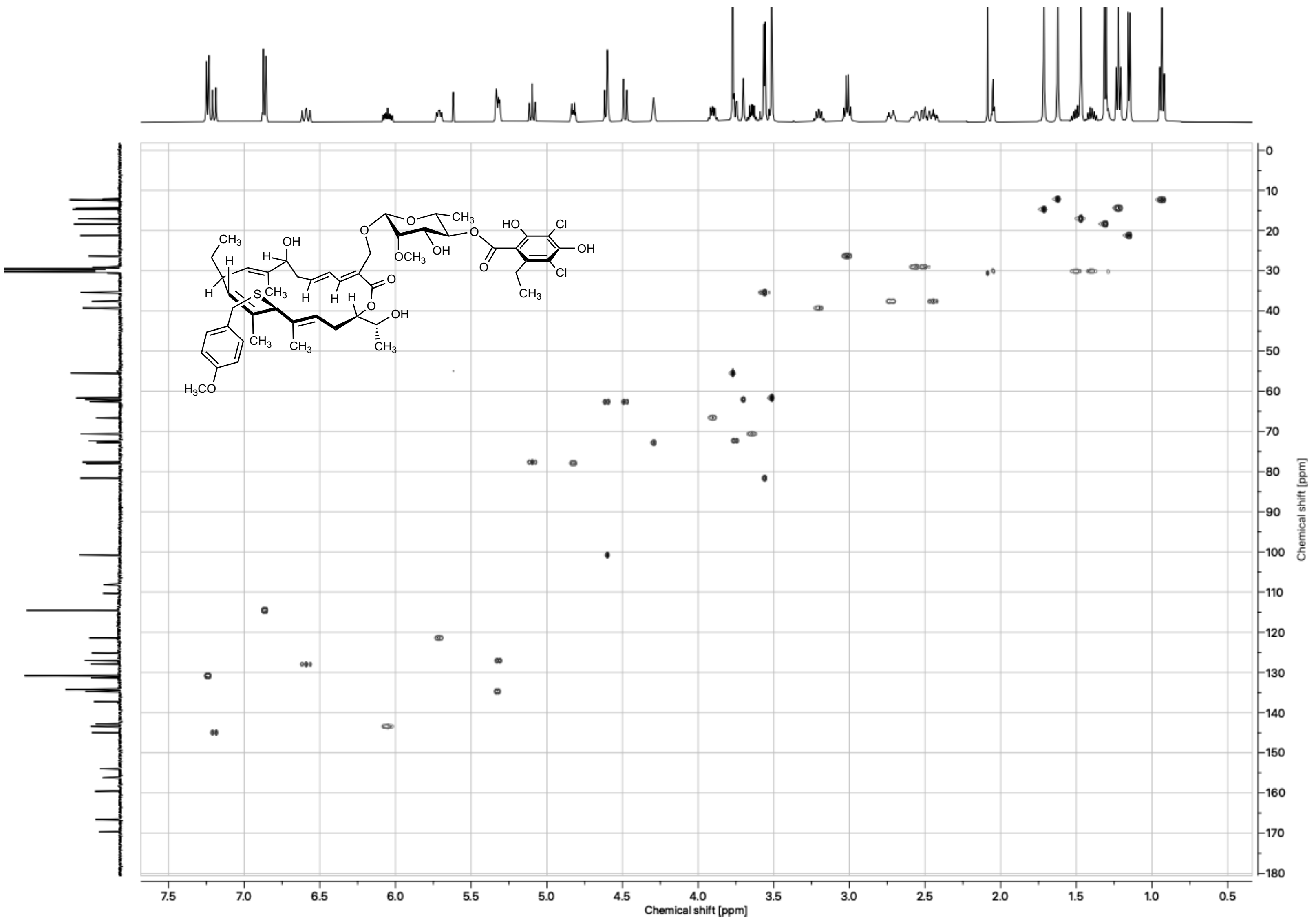

Figure 15: HSQC spectrum of 11-desnoviosyl-13-*p*-methoxybenzylsulfide fidaxomicin (5a-C(13)) in acetone-d_6

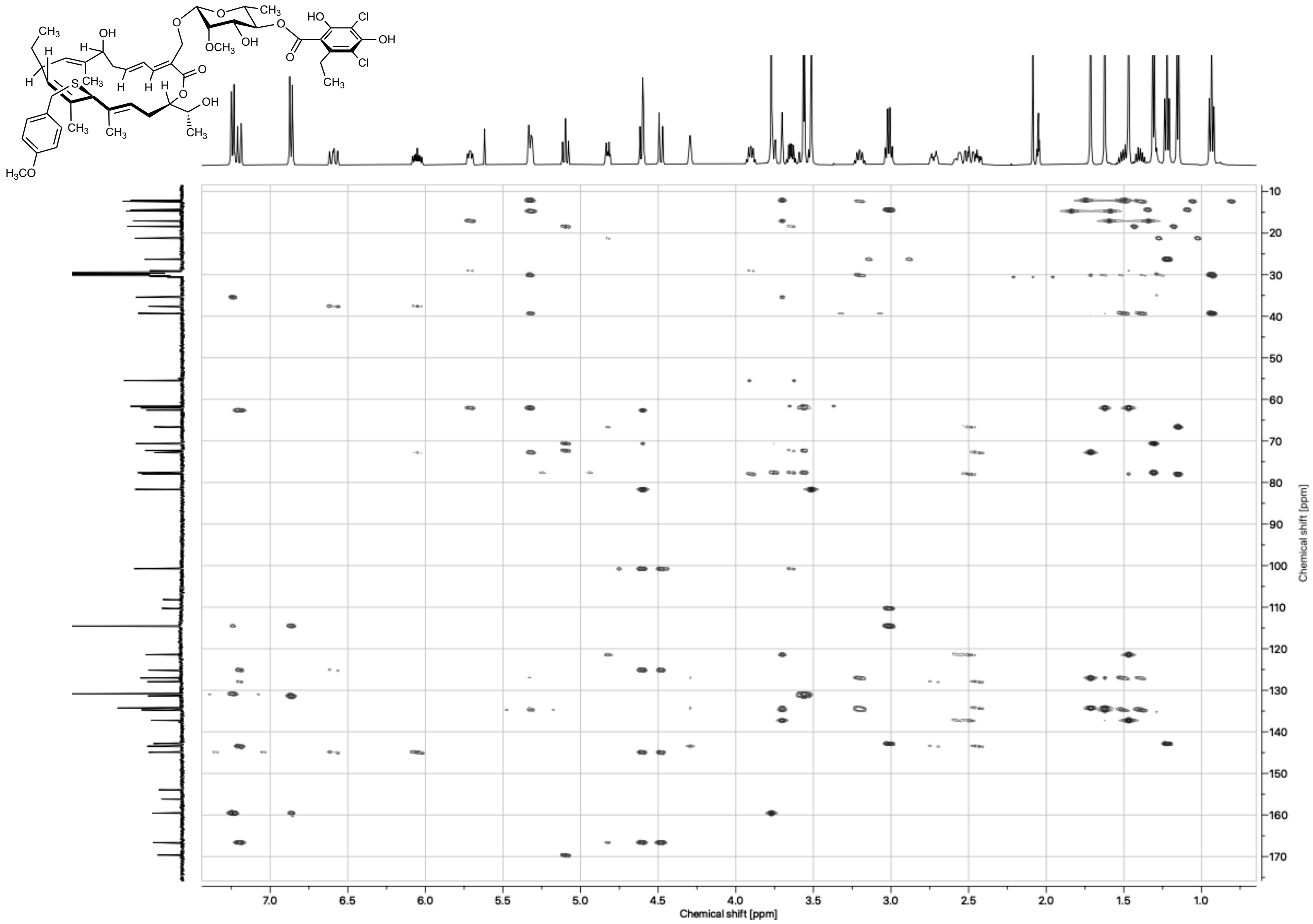

Figure 16: HMBC spectrum of 11-desnoviosyl-13-*p*-methoxybenzylsulfide fidaxomicin (5a-C(13)) in acetone-d_6

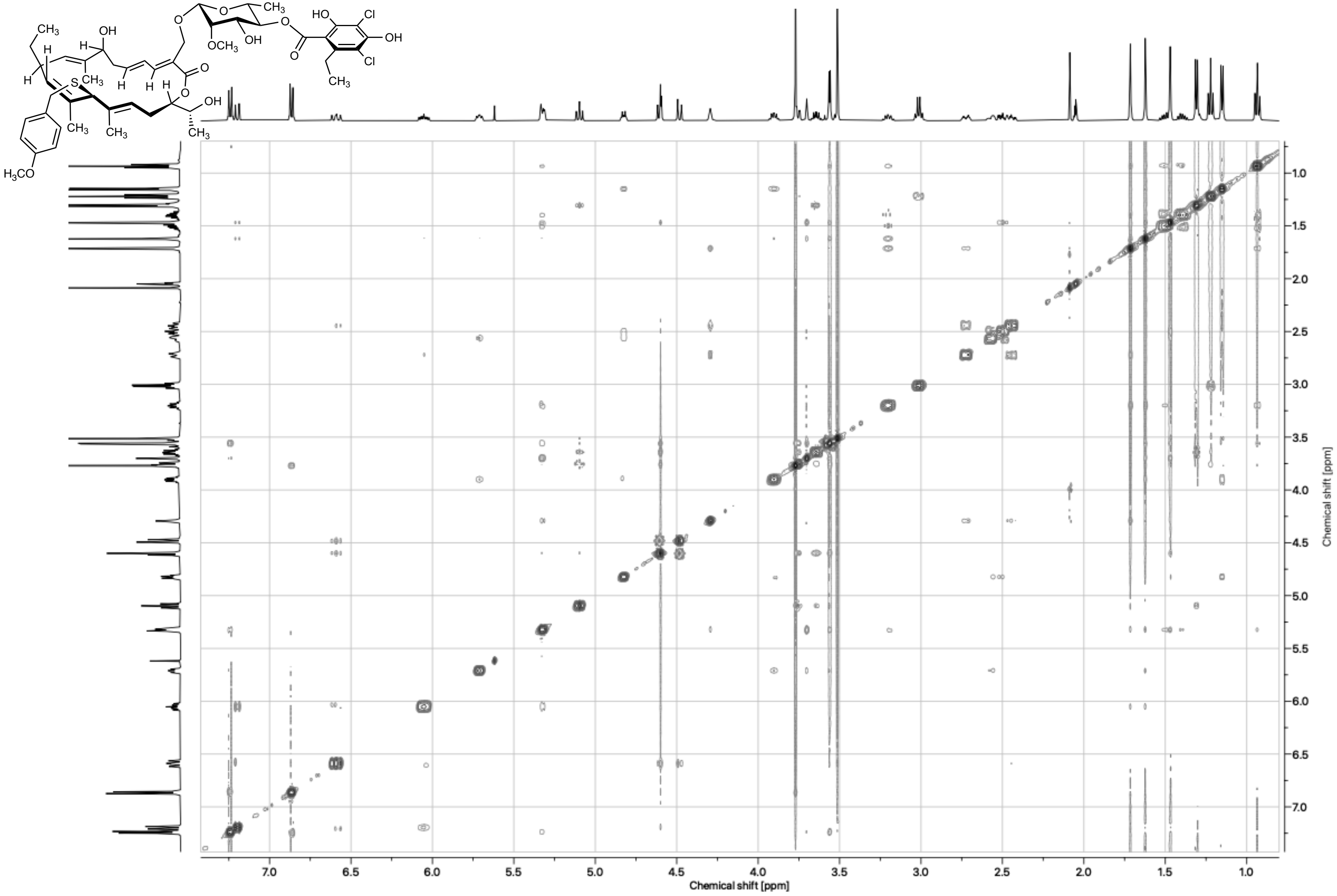

Figure 17: NOESY spectrum of 11-desnoviosyl-13-*p*-methoxybenzylsulfide fidaxomicin (5a-C(13)) in acetone-d_6

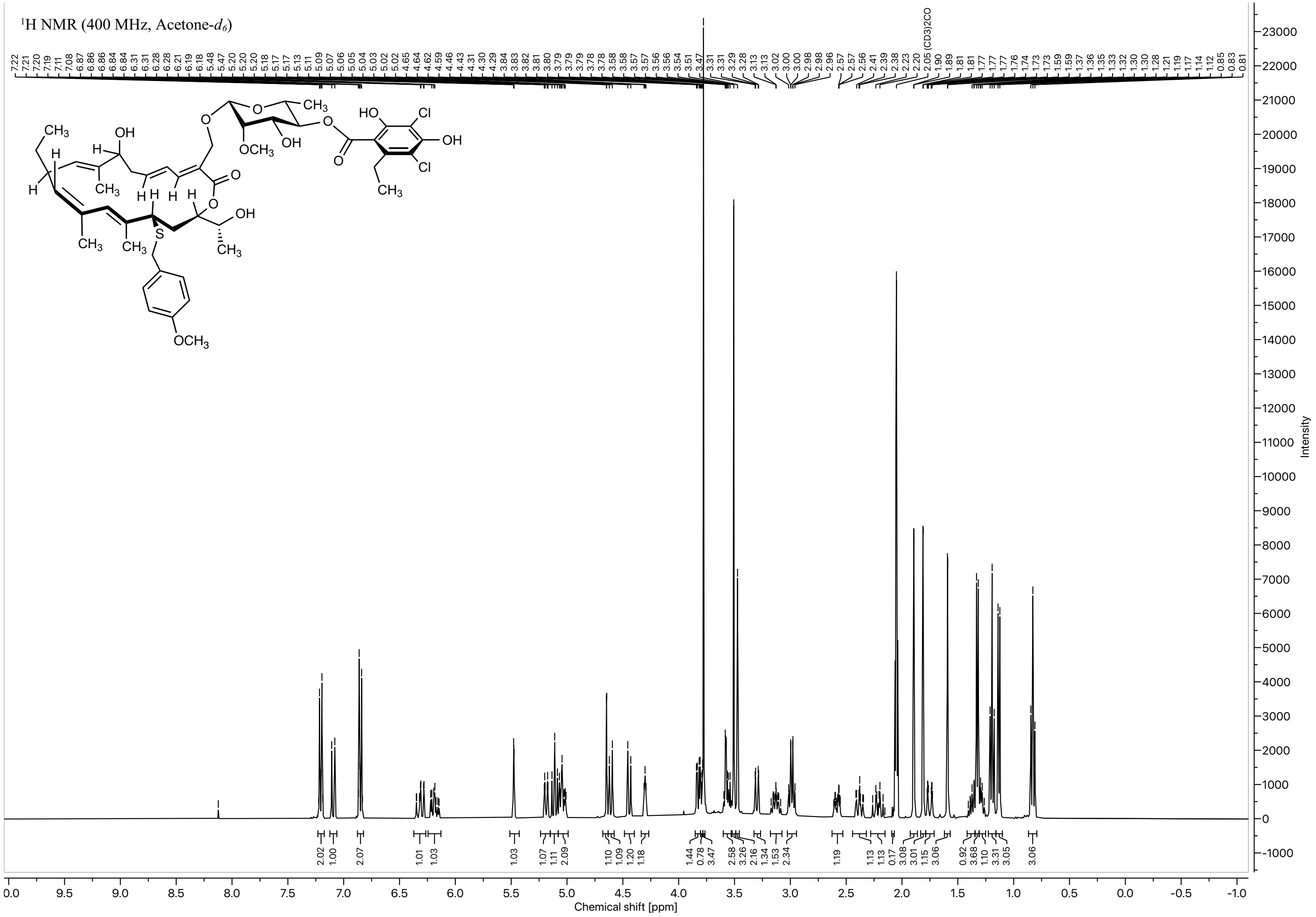

Figure 18: ^{1}H NMR spectrum of 11-desnoviosyl-15-*p*-methoxybenzylsulfide fidaxomicin (5a-C(15)) in acetone-d_6

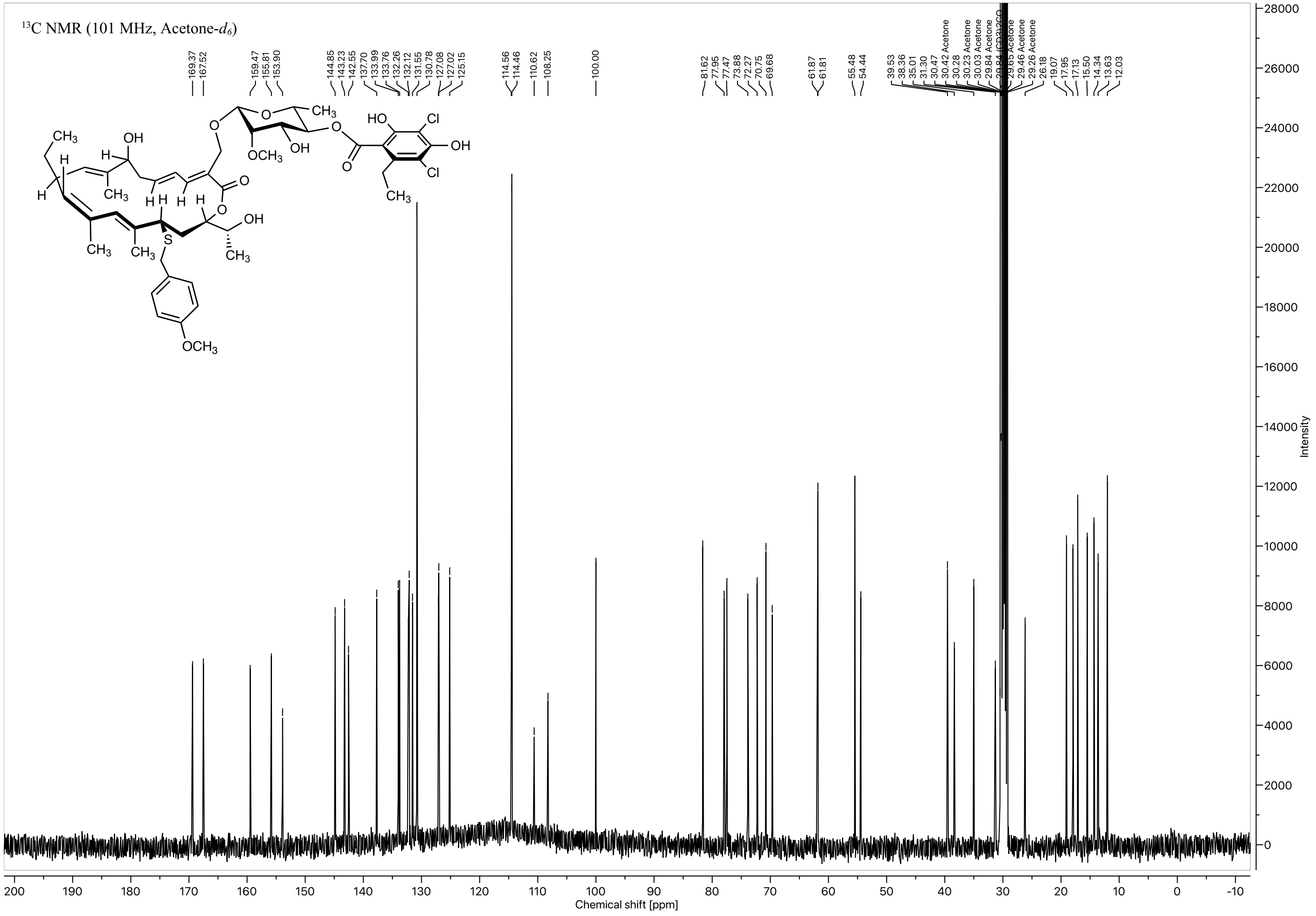

Figure 19: ^{13}C NMR spectrum of 11-desnoviosyl-15-*p*-methoxybenzylsulfide fidaxomicin (5a-C(15)) in acetone-d_6

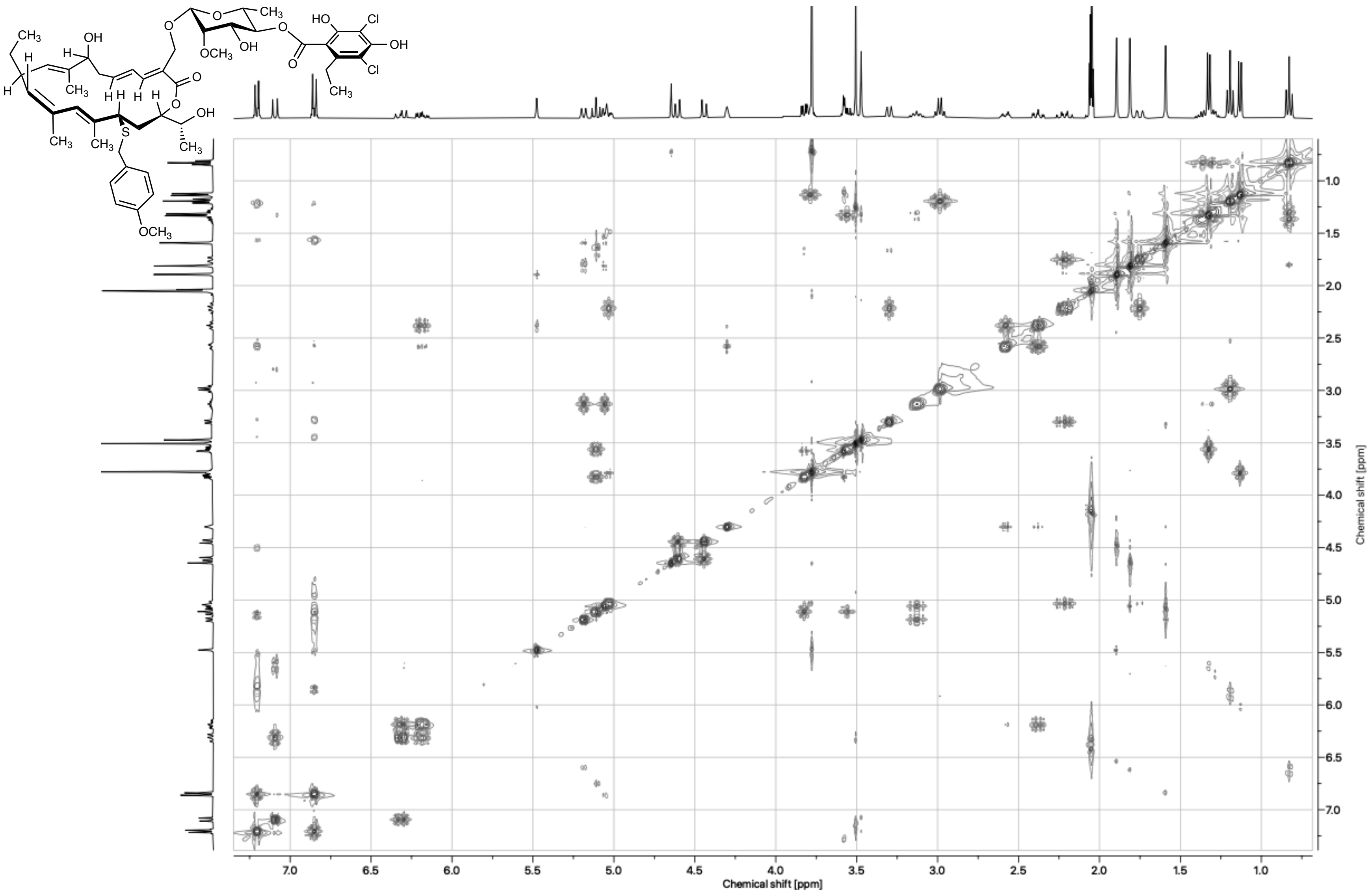

Figure 20: COSY spectrum of 11-desnoviosyl-15-*p*-methoxybenzylsulfide fidaxomicin (5a-C(15)) in acetone-d_6

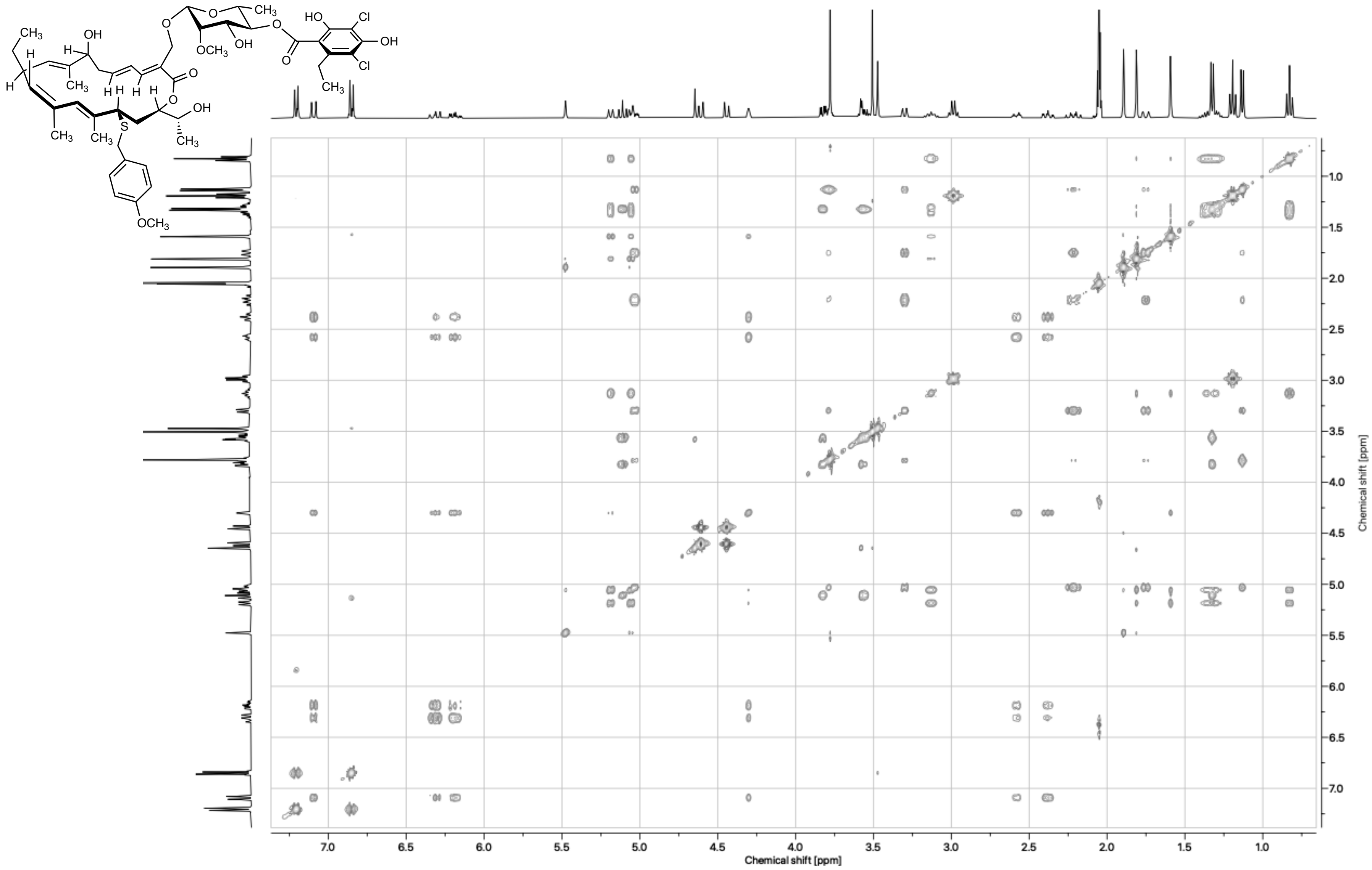

Figure 21: TOCSY spectrum of 11-desnoviosyl-15-*p*-methoxybenzylsulfide fidaxomicin (5a-C(15)) in acetone-d_6

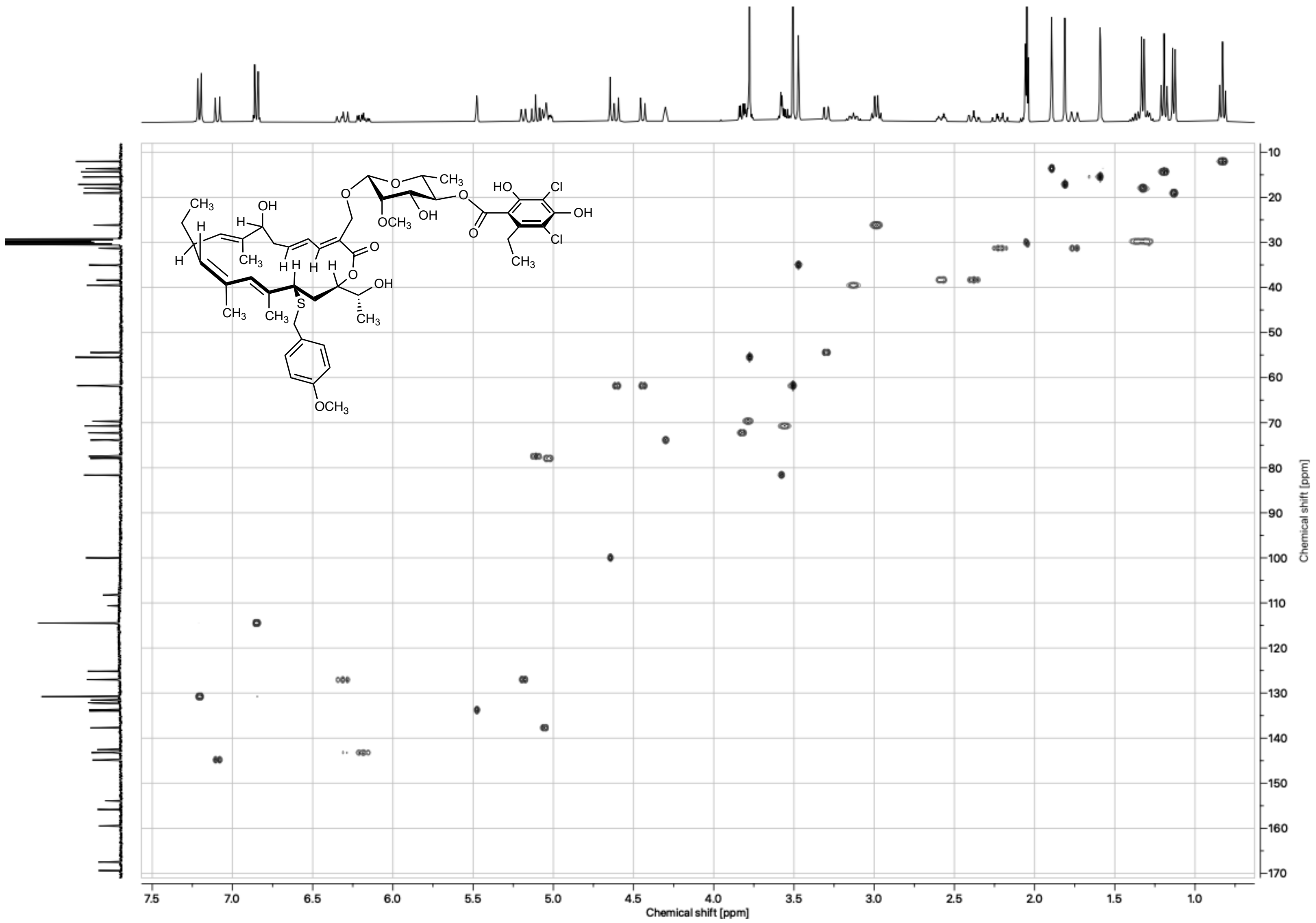

Figure 22: HSQC spectrum of 11-desnoviosyl-15-*p*-methoxybenzylsulfide fidaxomicin (5a-C(15)) in acetone-d_6

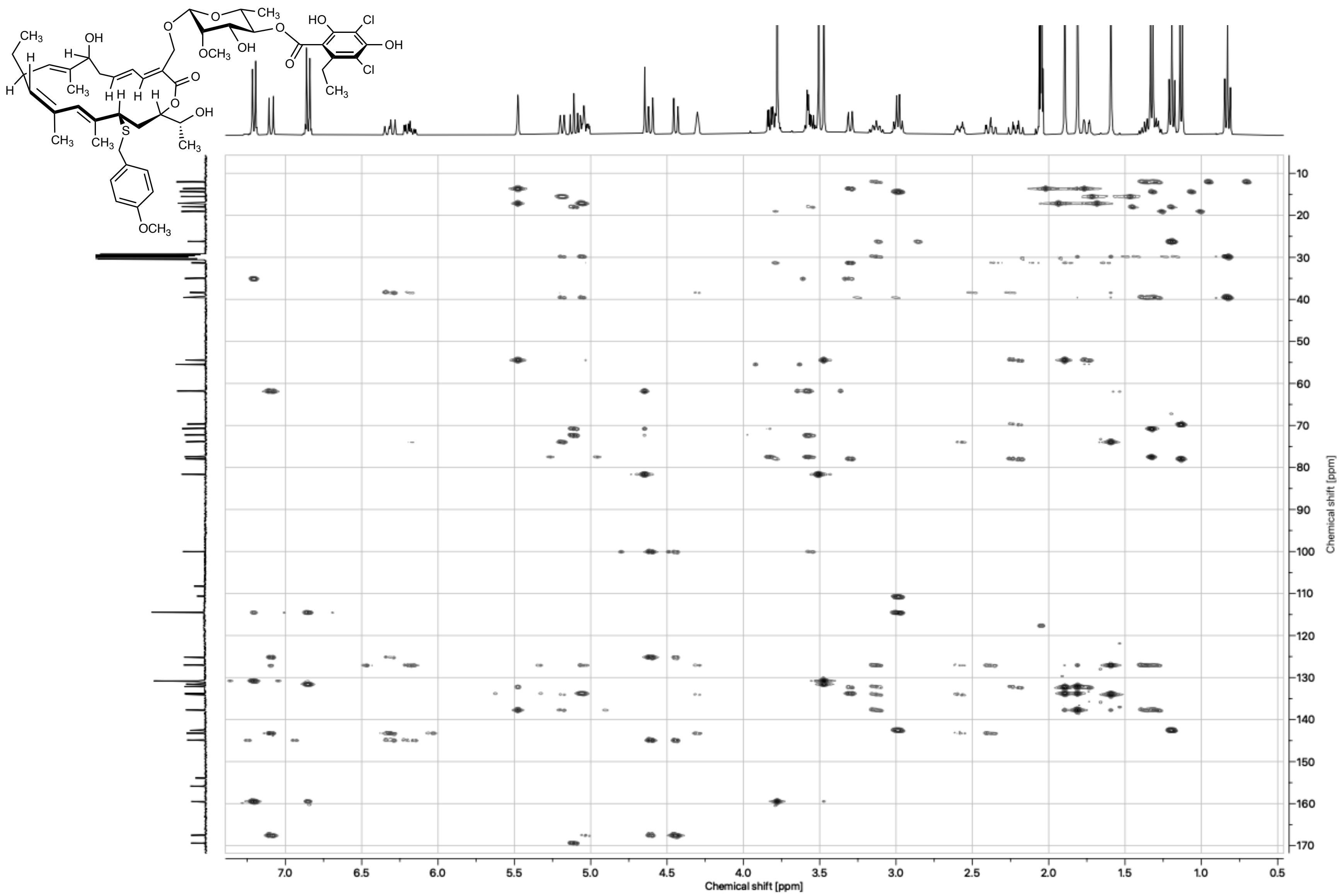

Figure 23: HMBC spectrum of 11-desnoviosyl-15-*p*-methoxybenzylsulfide fidaxomicin (5a-C(15)) in acetone-d_6

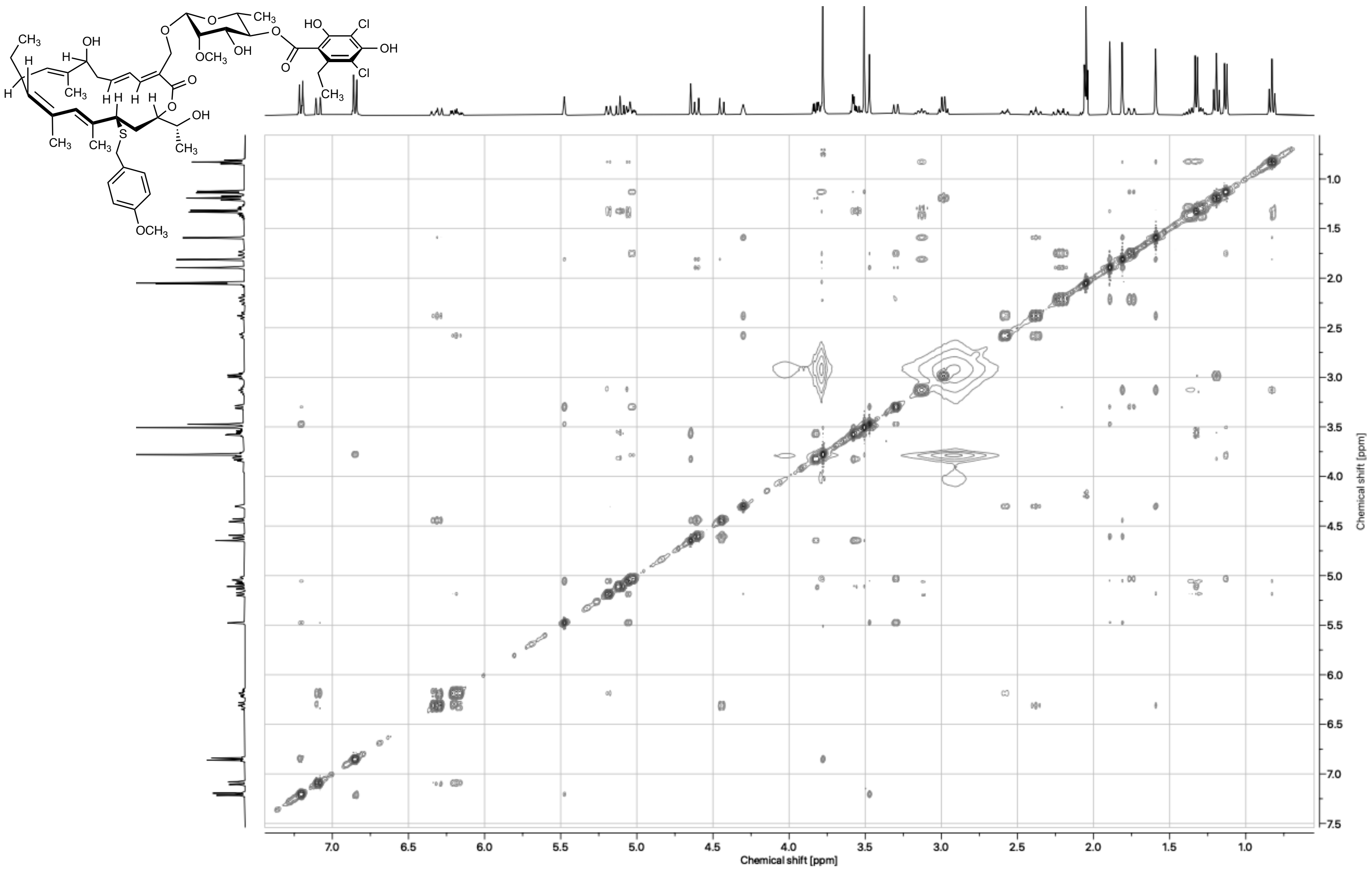

Figure 24: NOESY spectrum of 11-desnoviosyl-15-*p*-methoxybenzylsulfide fidaxomicin (5a-C(15)) in acetone-d_6

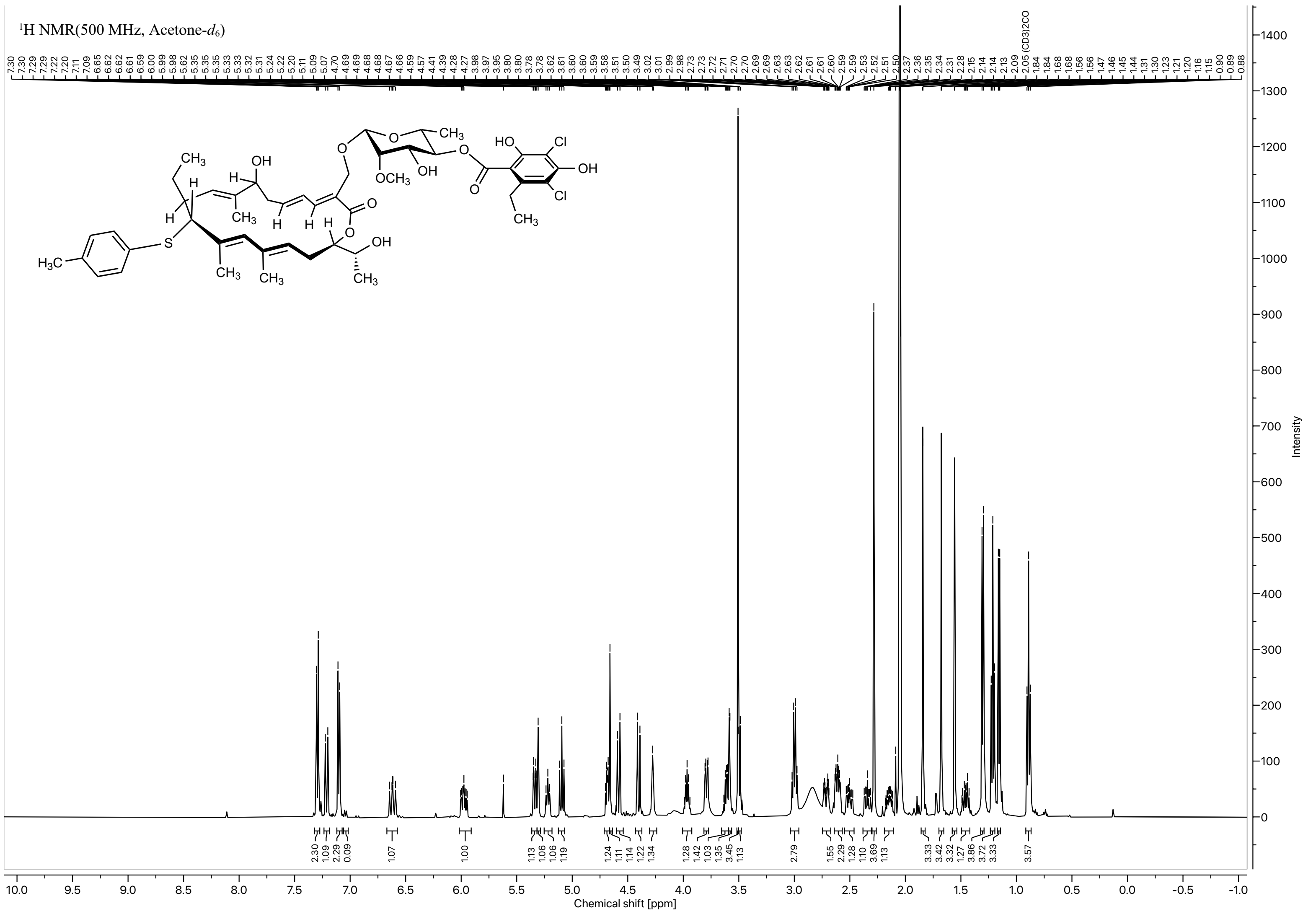

Figure 25: ^{1}H NMR spectrum of 11-desnoviosyl-11-*p*-tolylsulfide fidaxomicin (5b-C(11)) in acetone-d_6

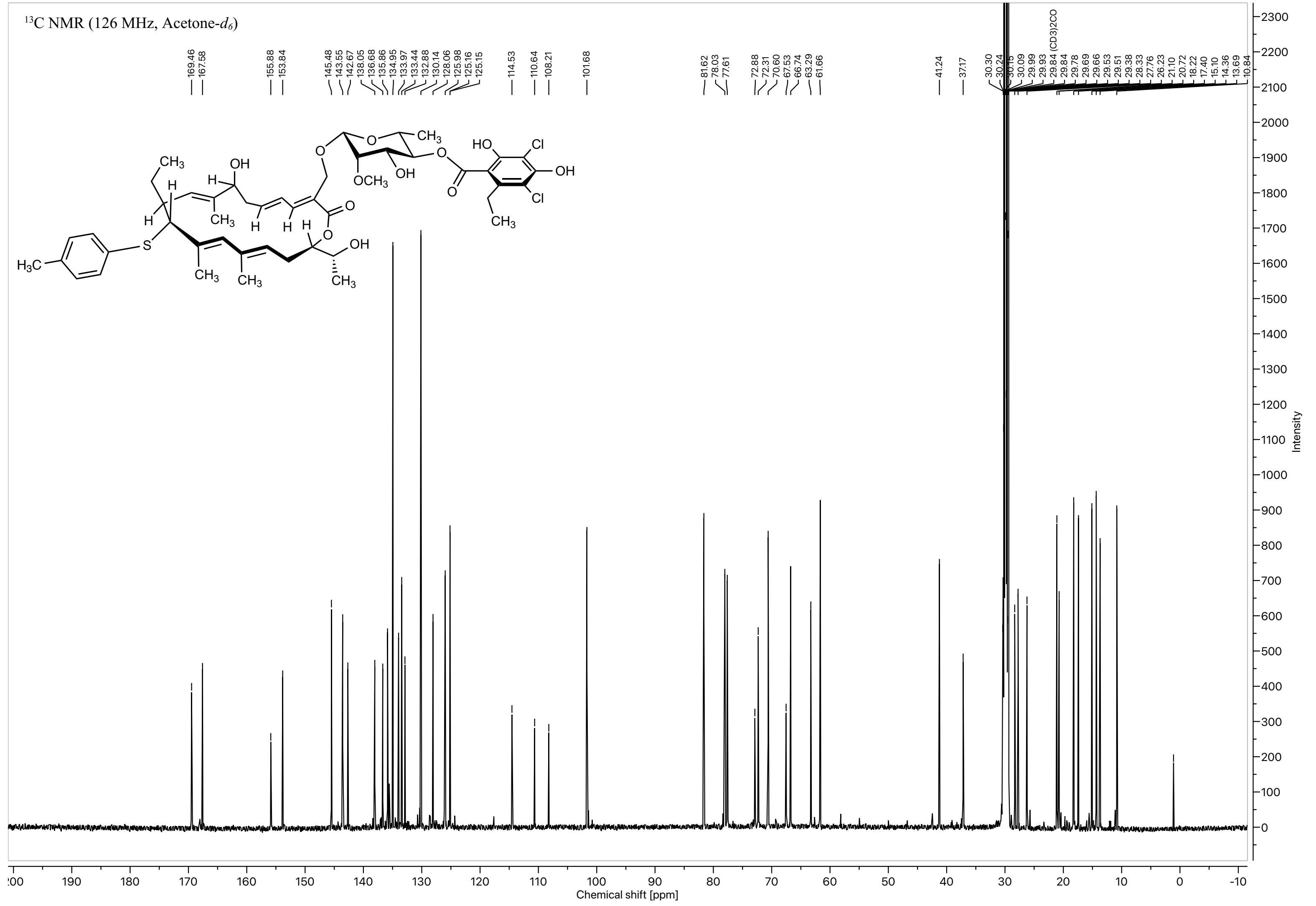

Figure 26: ^{13}C NMR spectrum of 11-desnoviosyl-11-*p*-tolylsulfide fidaxomicin (5b-C(11)) in acetone-d$_6$

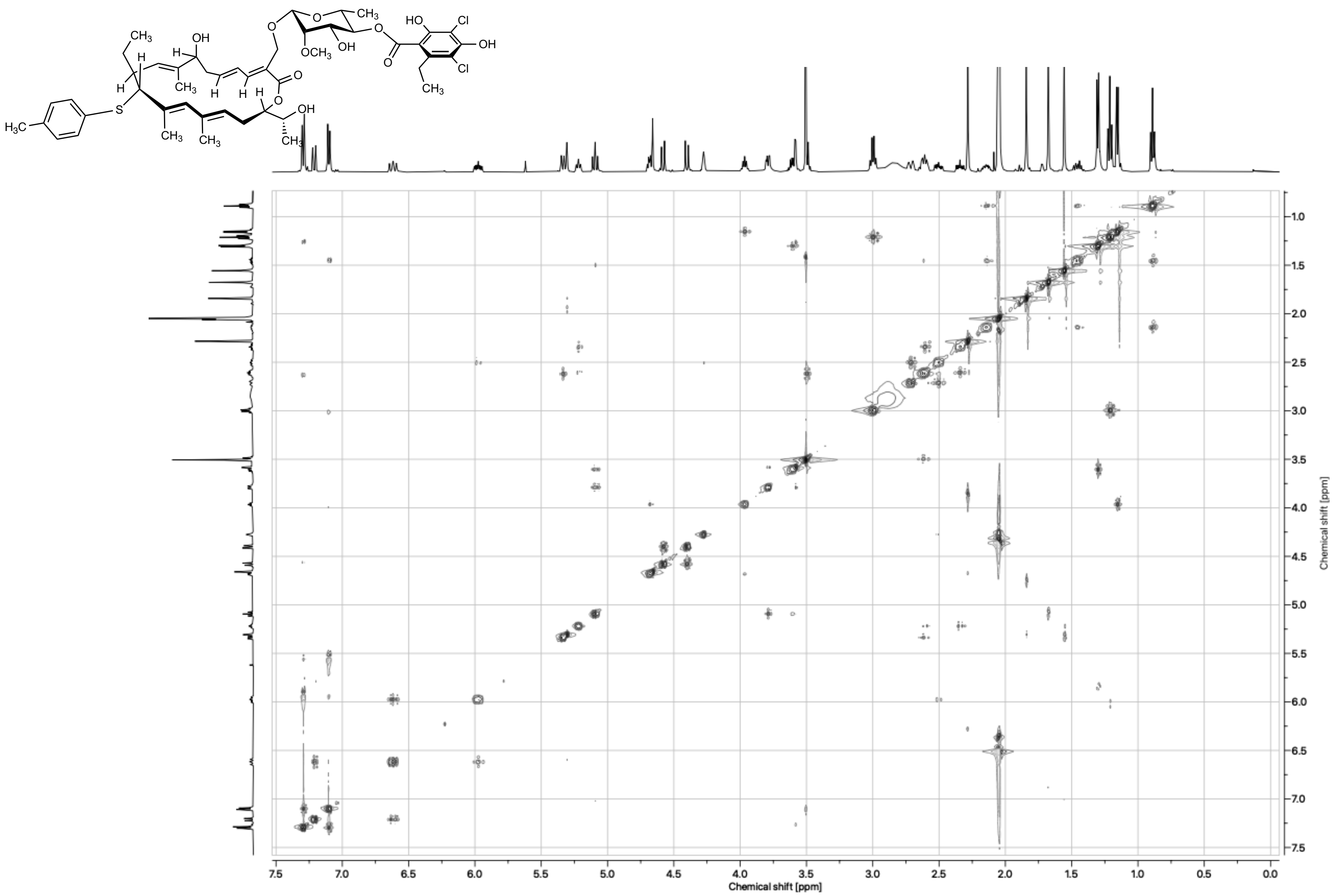

Figure 27: COSY spectrum of 11-desnoviosyl-11-*p*-tolylsulfide fidaxomicin (5b-C(11)) in acetone-d_6

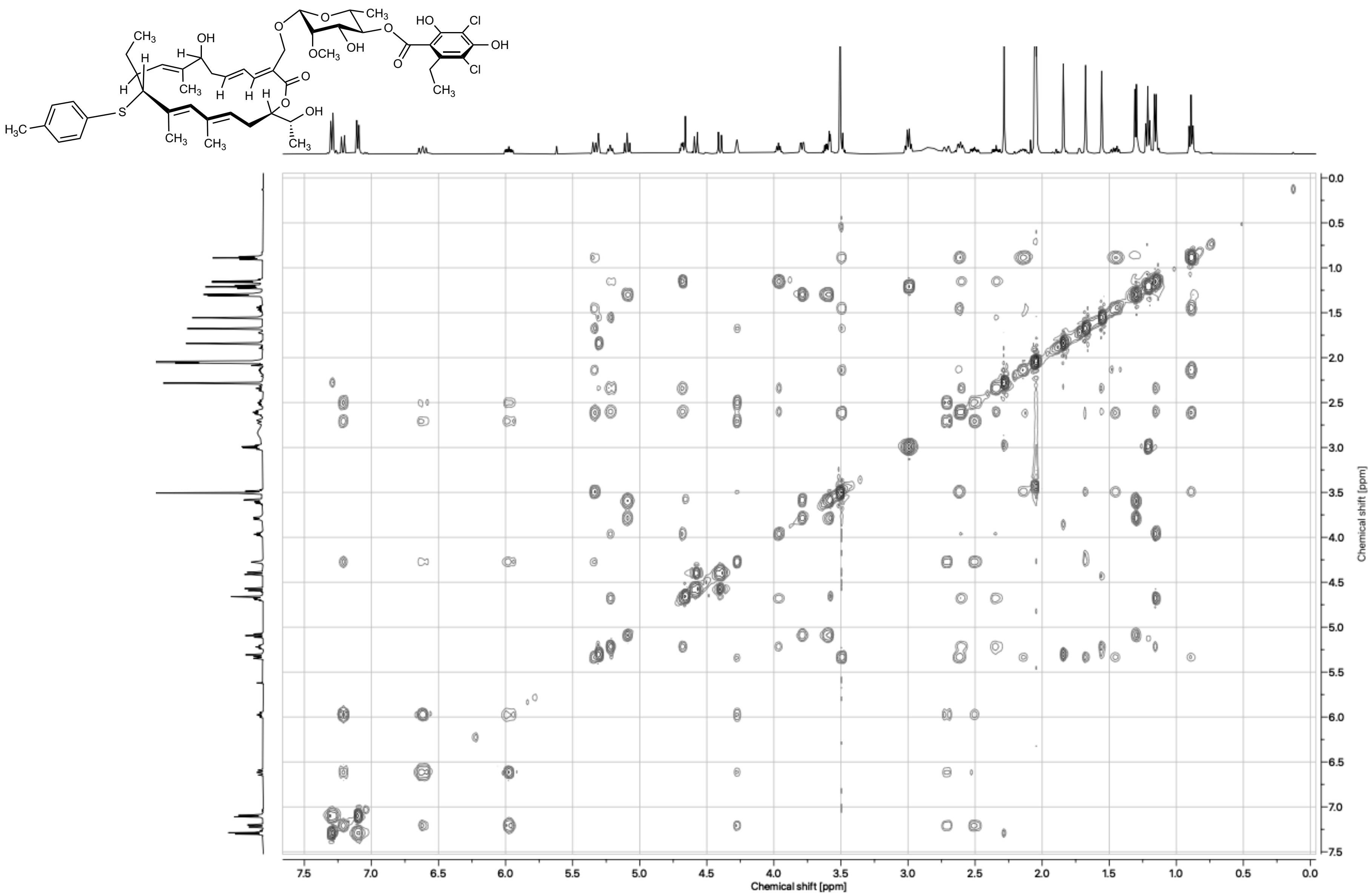

Figure 28: TOCSY spectrum of 11-desnoviosyl-11-*p*-tolylsulfide fidaxomicin (5b-C(11)) in acetone-d_6

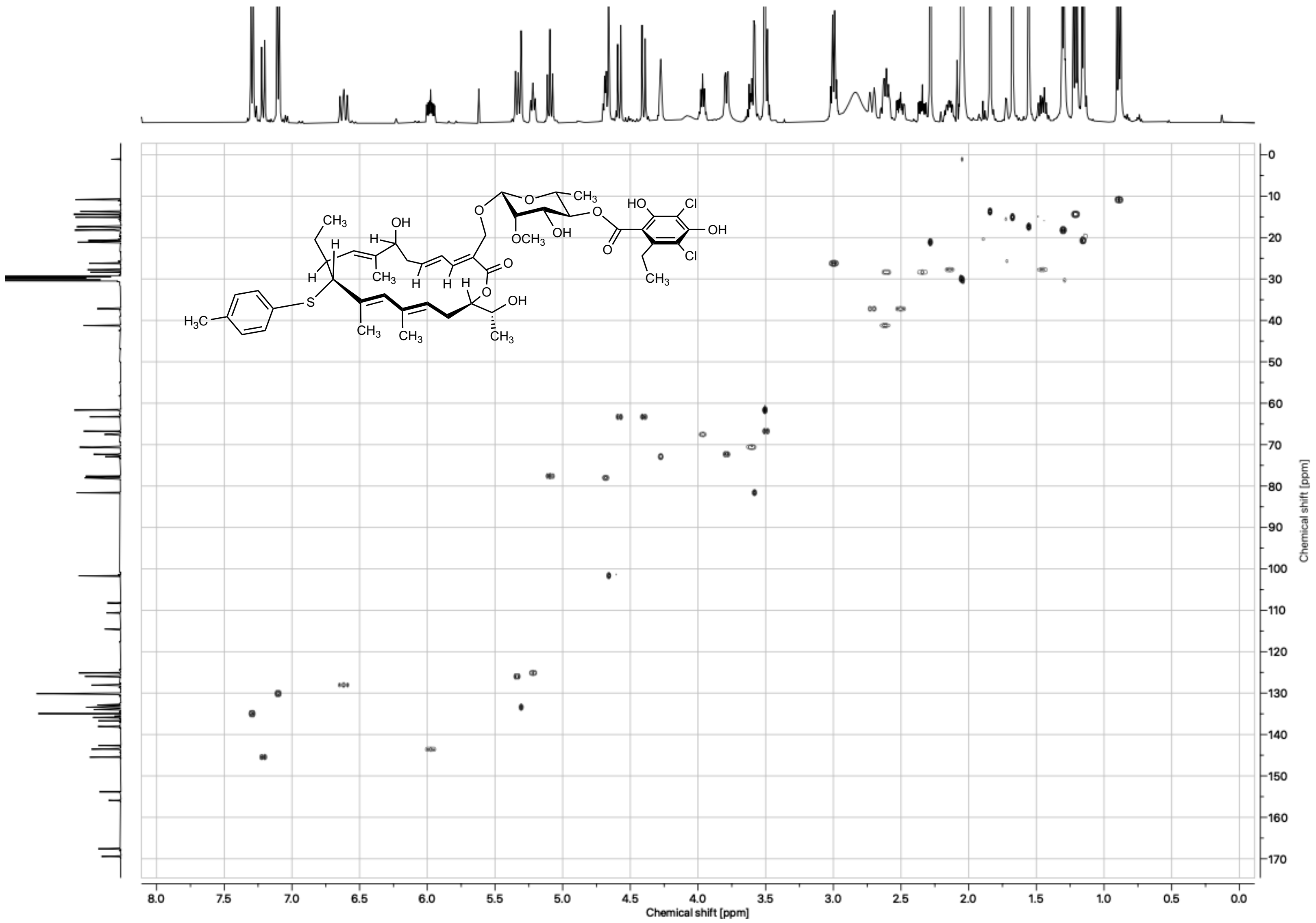

Figure 29: HSQC spectrum of 11-desnoviosyl-11-*p*-tolylsulfide fidaxomicin (5b-C(11)) in acetone-d_6

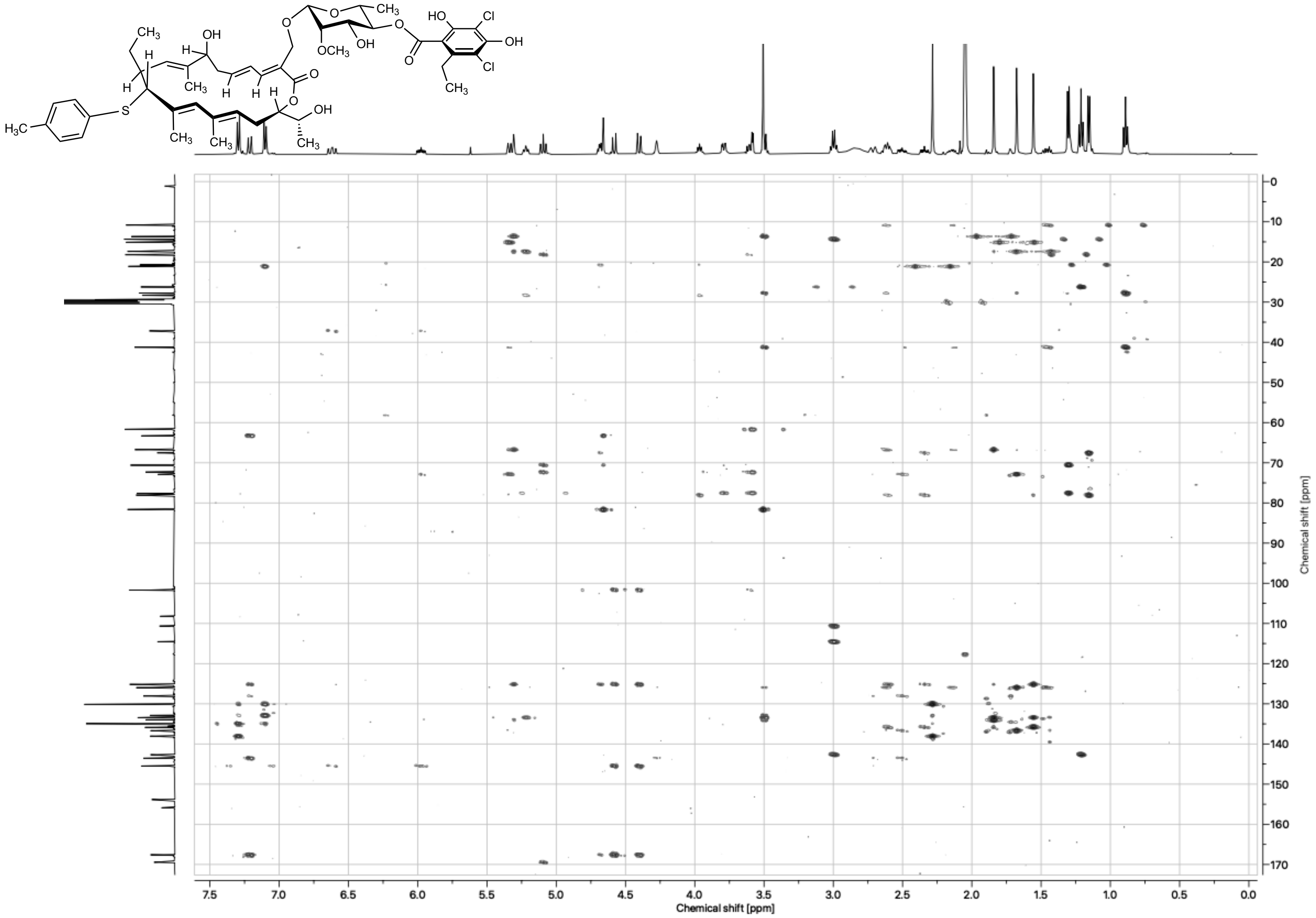

Figure 30: HMBC spectrum of 11-desnoviosyl-11-*p*-tolylsulfide fidaxomicin (5b-C(11)) in acetone-d_6

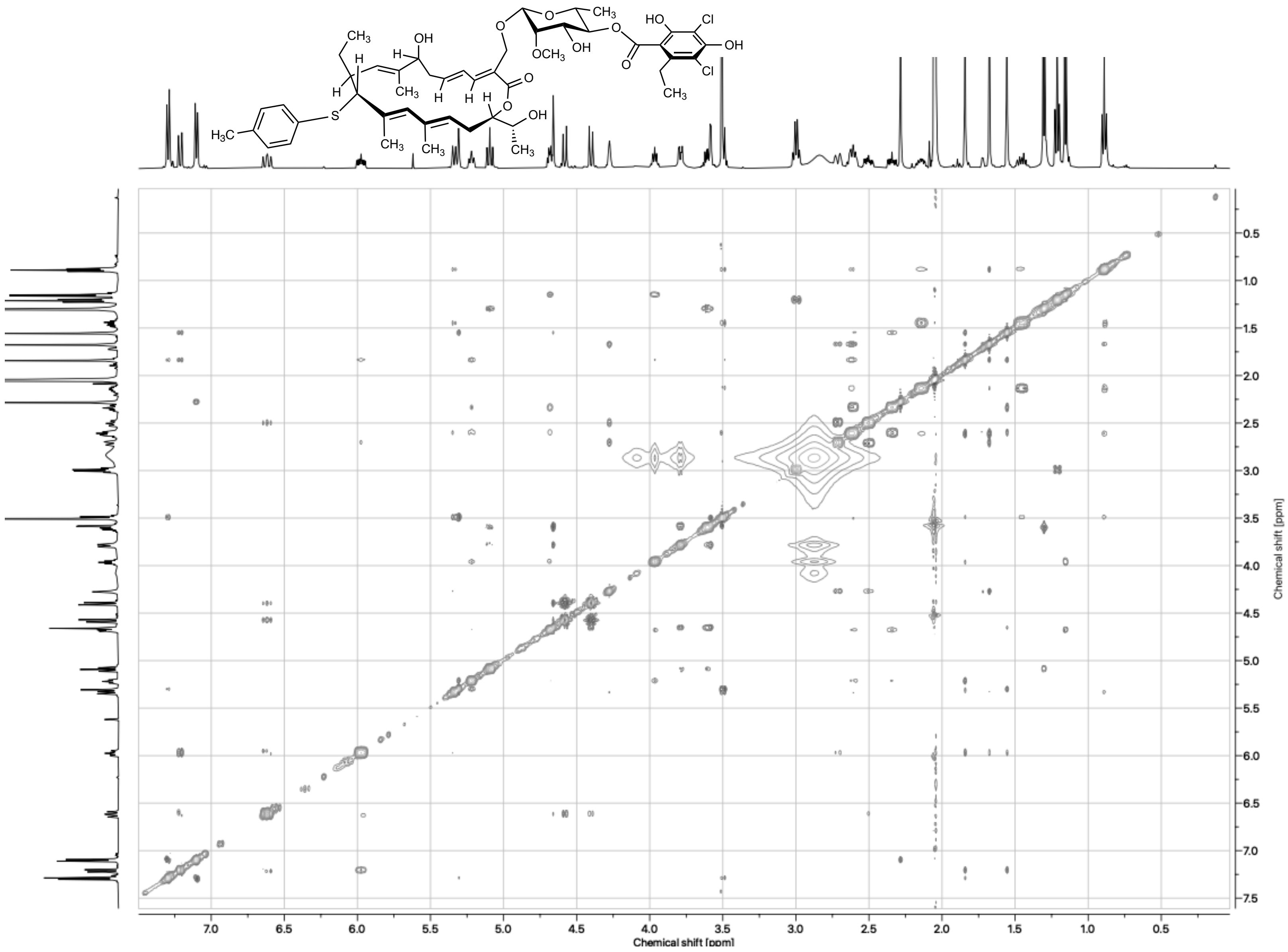

Figure 31: NOESY spectrum of 11-desnoviosyl-11-*p*-tolylsulfide fidaxomicin (5b-C(11)) in acetone-d_6

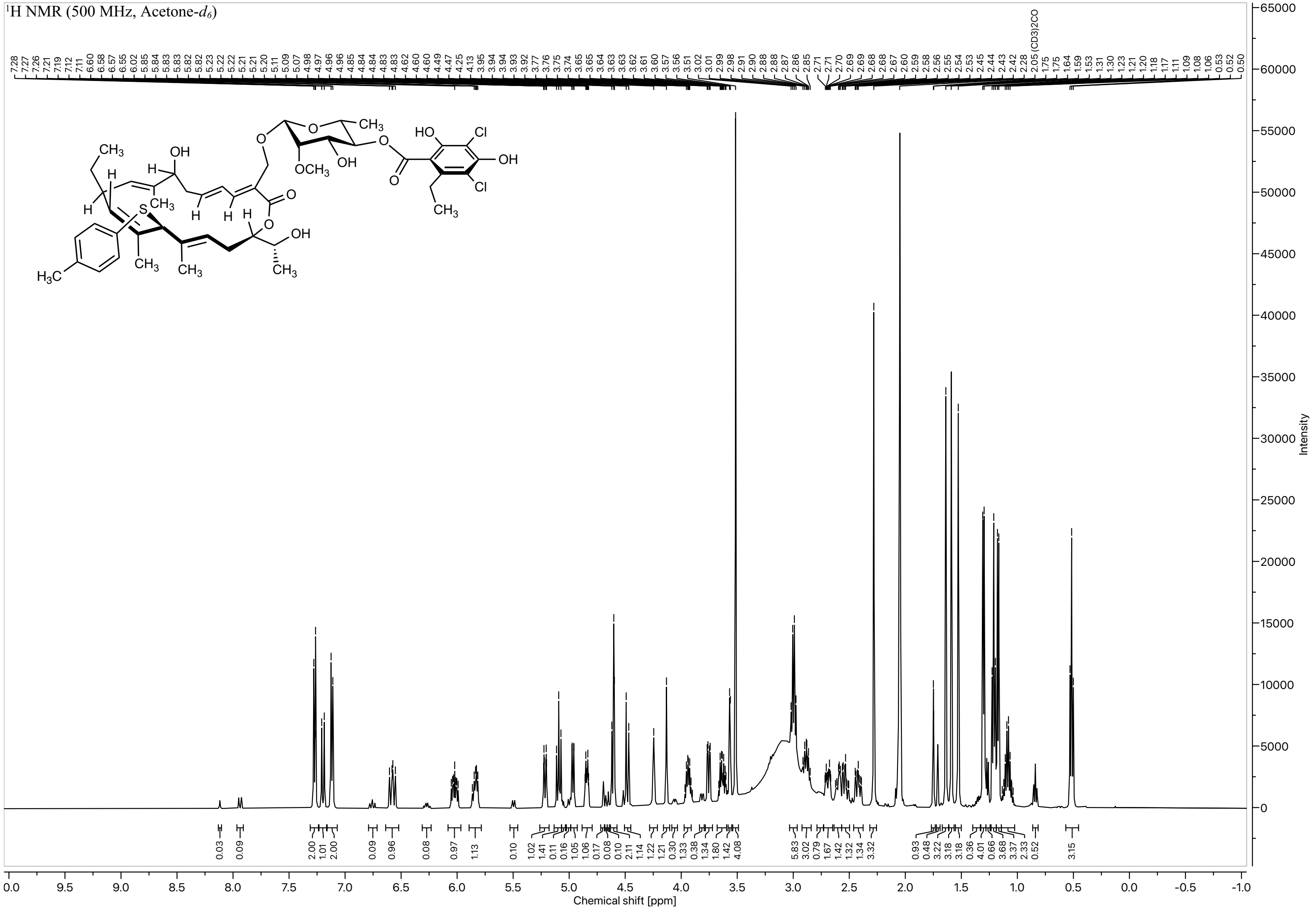

Figure 32: ^{1}H NMR spectrum of 11-desnoviosyl-13-*p*-tolylsulfide fidaxomicin (5b-C(13)) in acetone-d_6

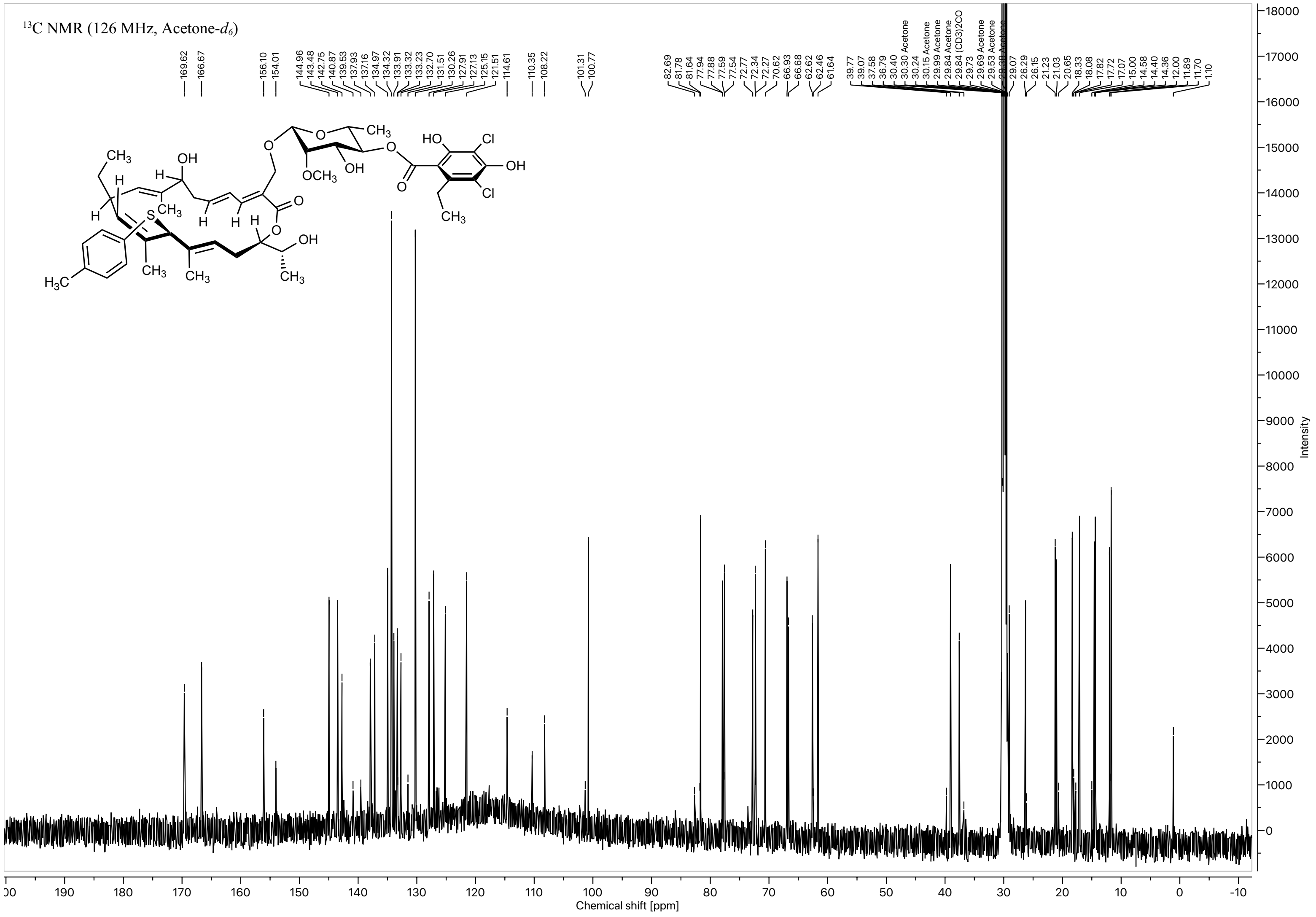

Figure 33: ^{13}C NMR spectrum of 11-desnoviosyl-13-*p*-tolylsulfide fidaxomicin (5b-C(13)) in acetone-d_6

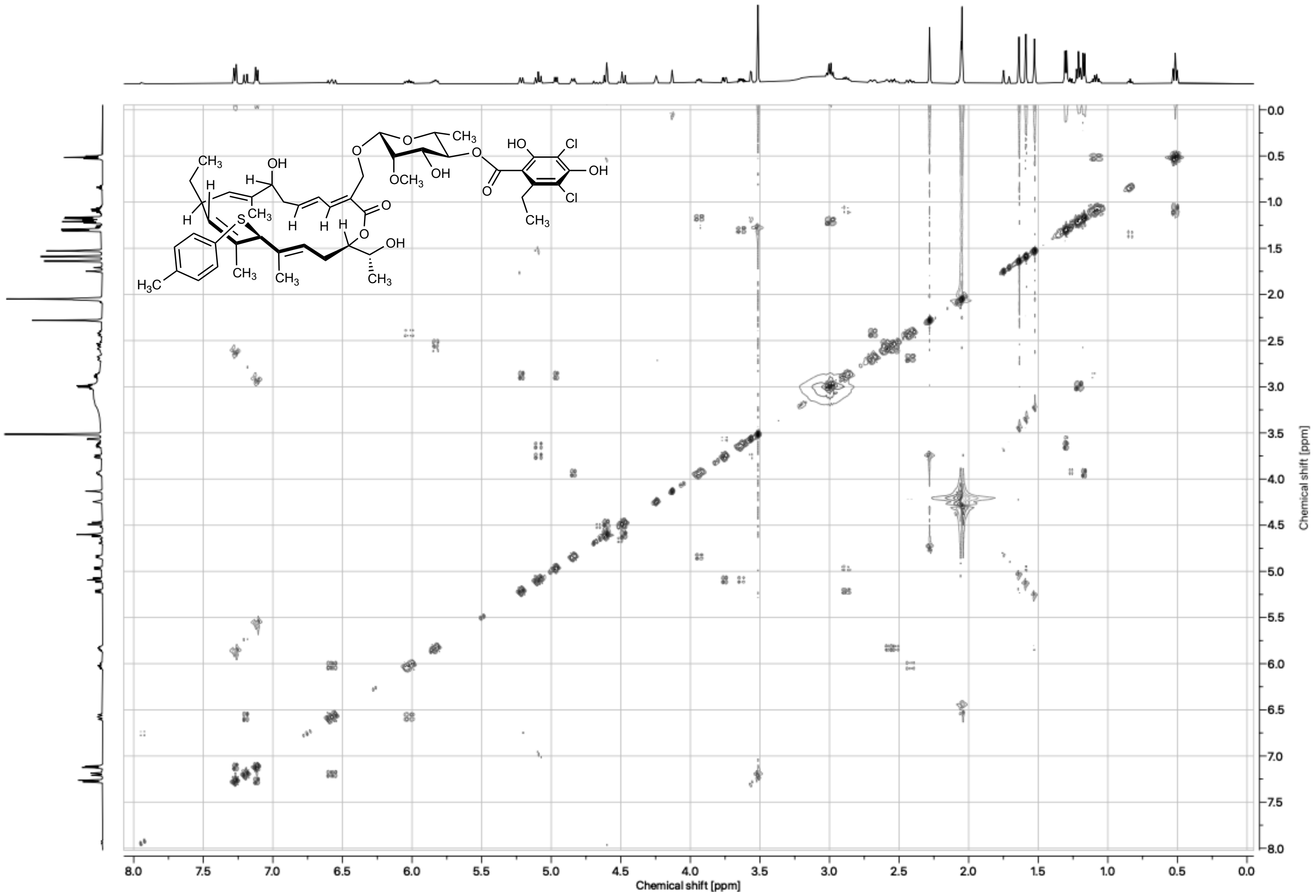

Figure 34: COSY spectrum of 11-desnoviosyl-13-*p*-tolylsulfide fidaxomicin (5b-C(13)) in acetone-d_6

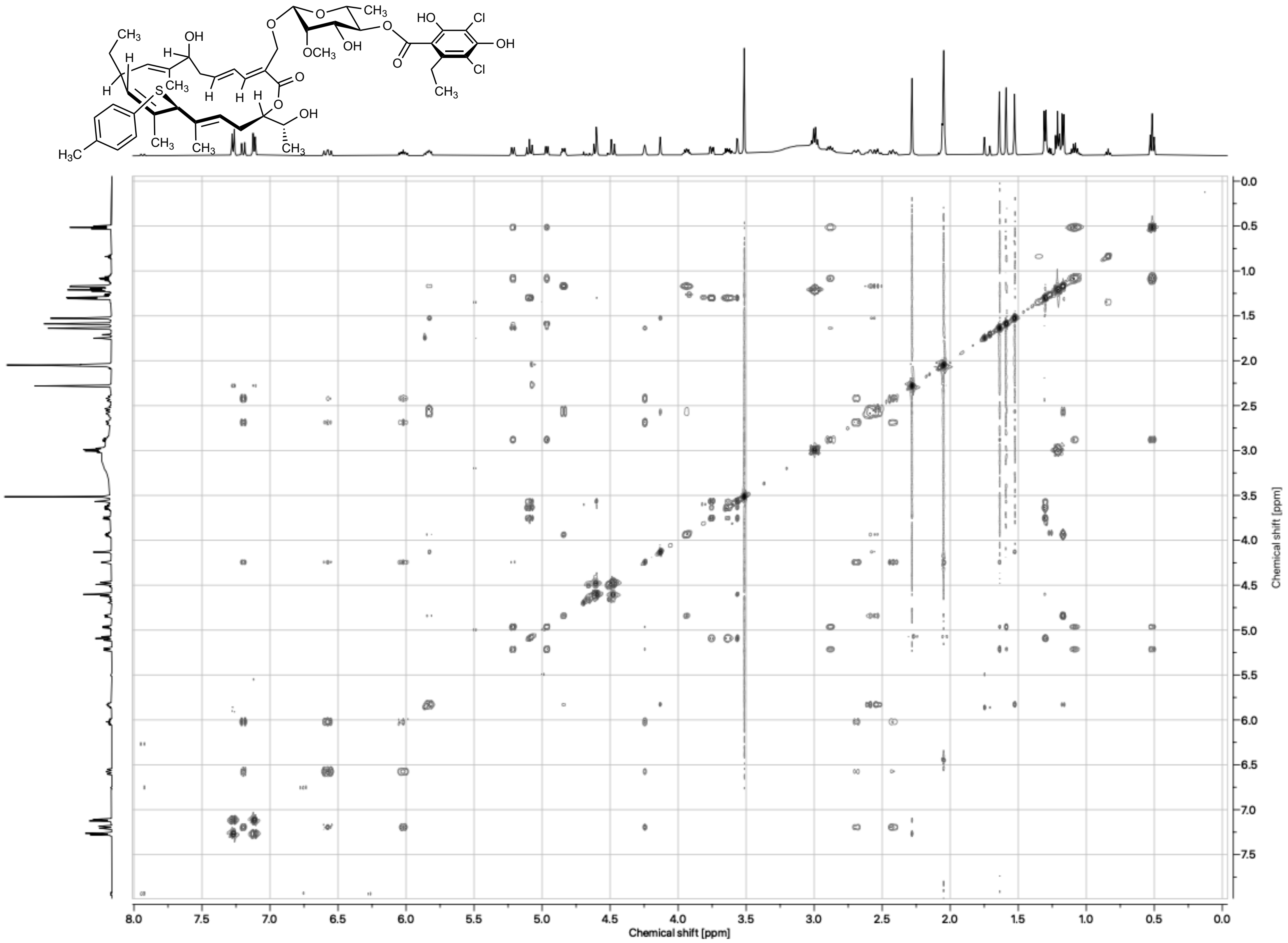

Figure 35: TOCSY spectrum of 11-desnoviosyl-13-*p*-tolylsulfide fidaxomicin (5b-C(13)) in acetone-d_6

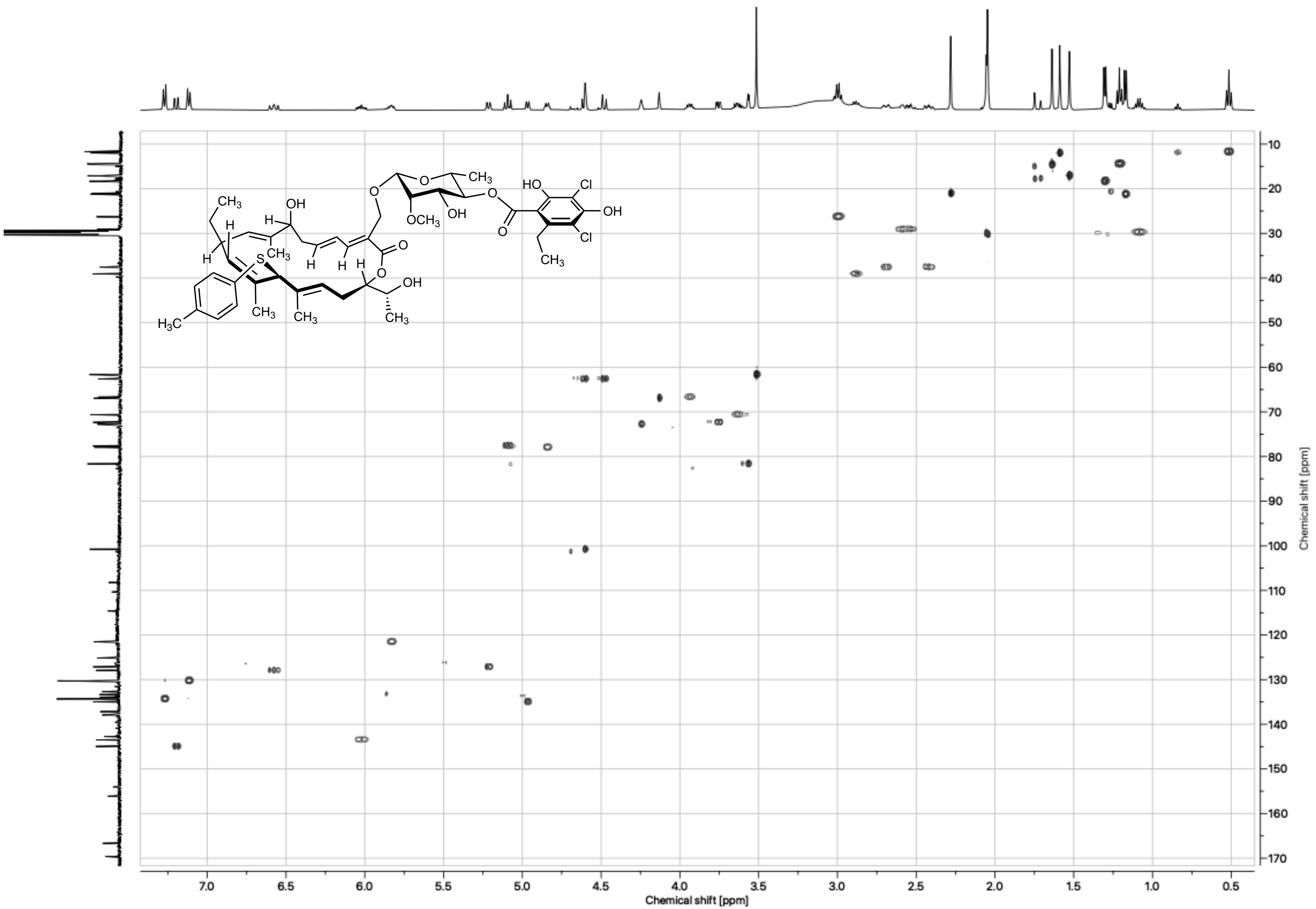

Figure 36: HSQC spectrum of 11-desnoviosyl-13-*p*-tolylsulfide fidaxomicin (5b-C(13)) in acetone-d_6

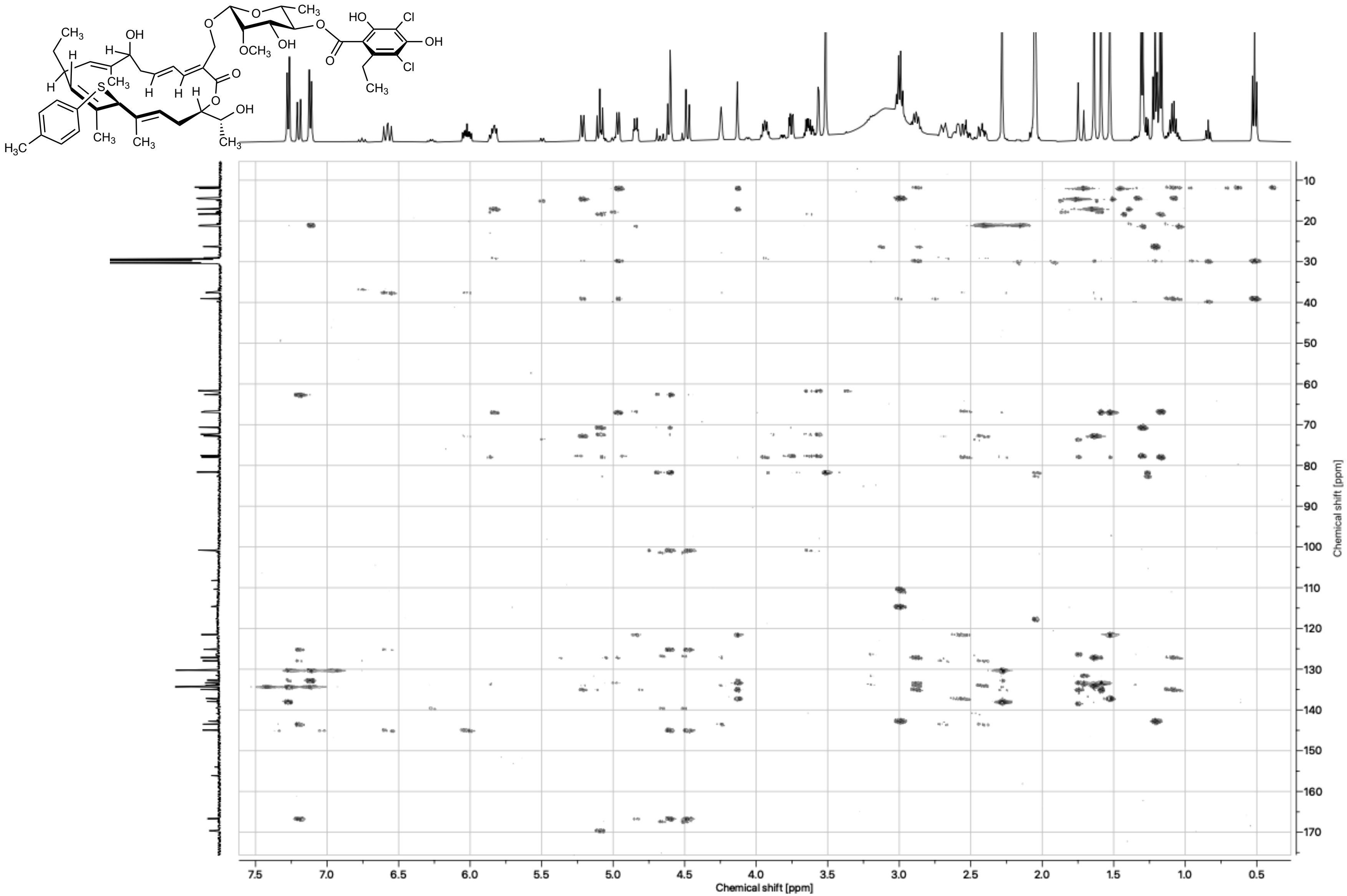

Figure 37: HMBC spectrum of 11-desnoviosyl-13-*p*-tolylsulfide fidaxomicin (5b-C(13)) in acetone-d_6

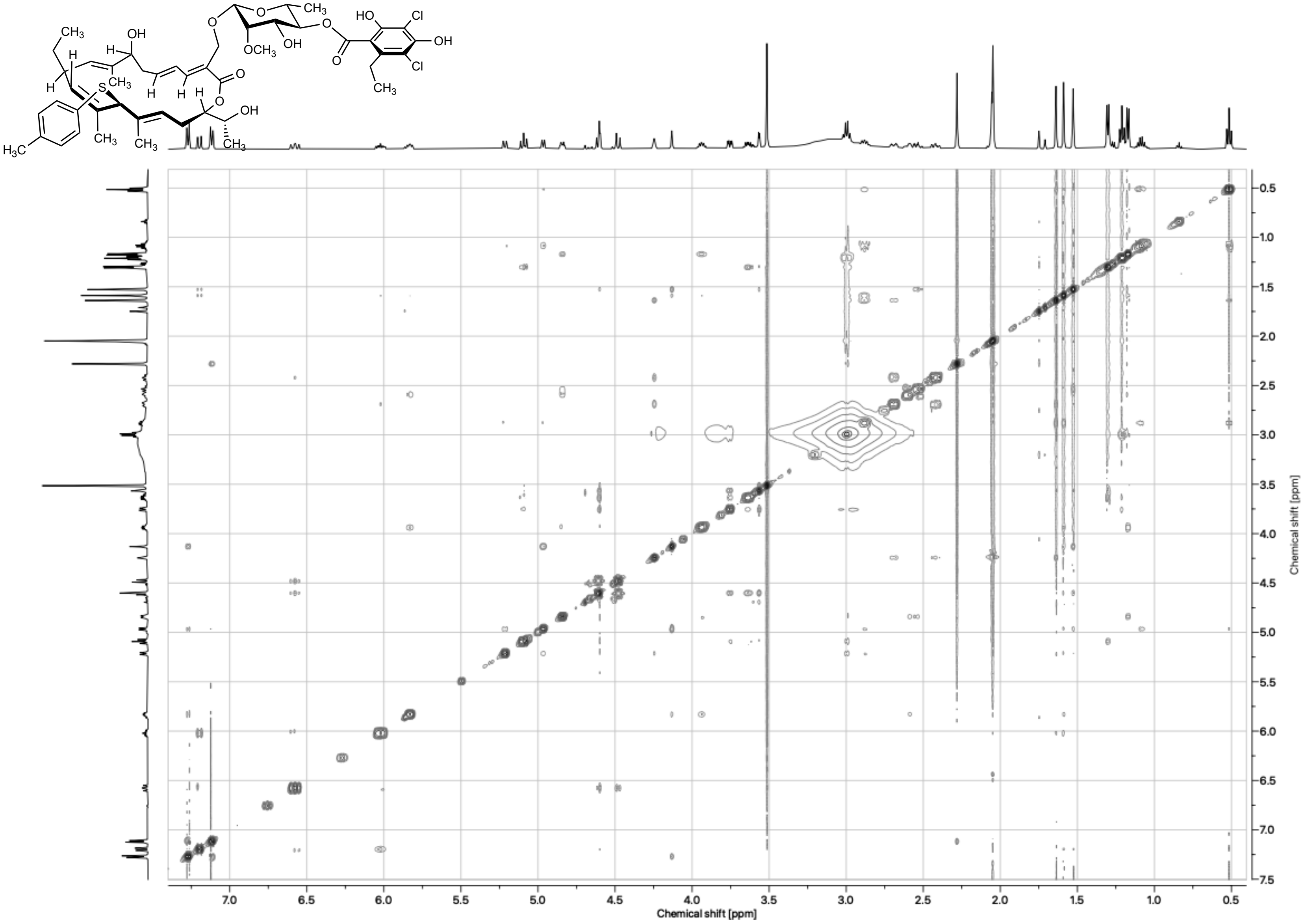

Figure 38: NOESY spectrum of 11-desnoviosyl-13-*p*-tolylsulfide fidaxomicin (5b-C(13)) in acetone-d_6

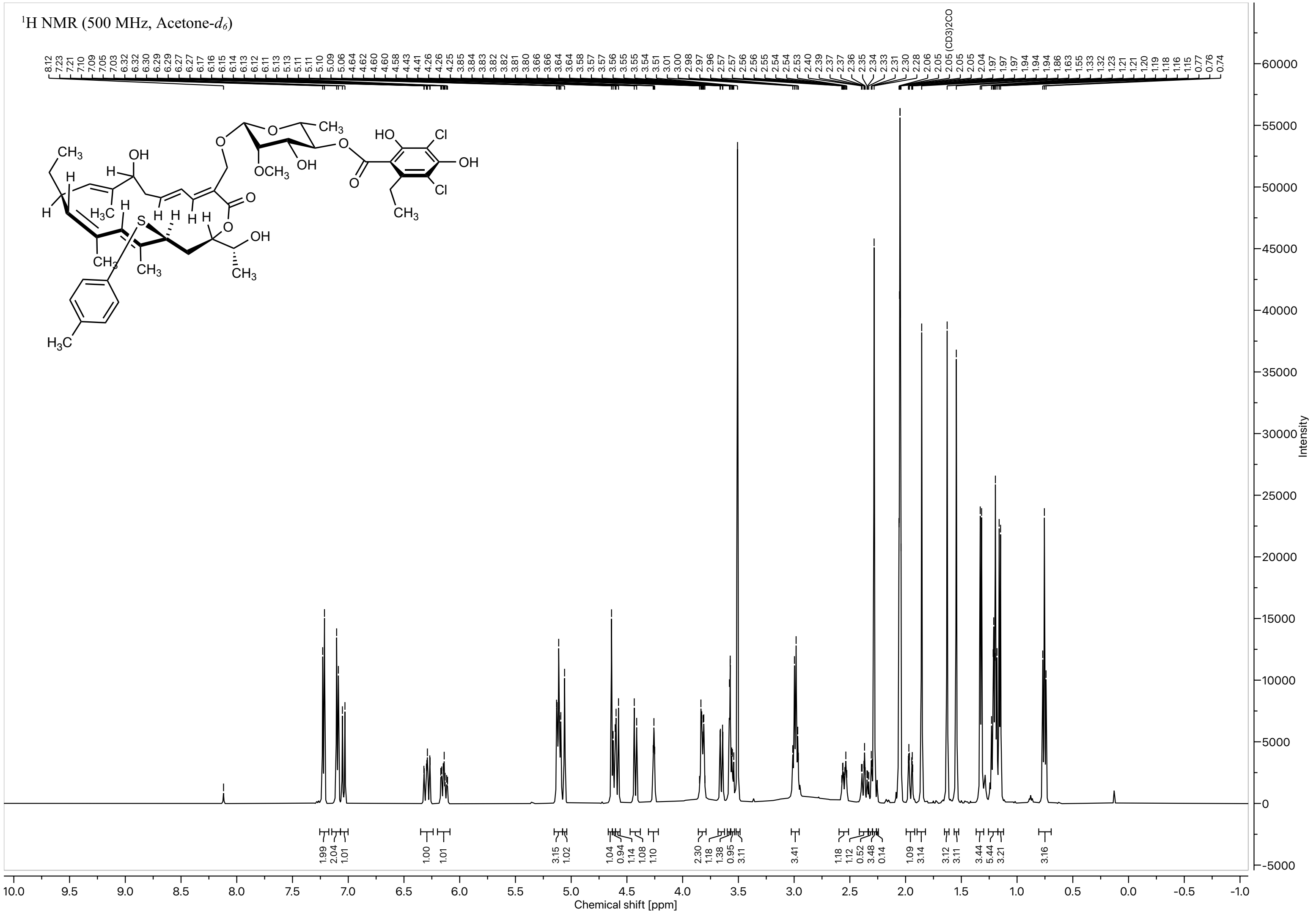

Figure 39: ^{1}H NMR spectrum of 11-desnoviosyl-15-*p*-tolylsulfide fidaxomicin (5b-C(15)) in acetone-d_6

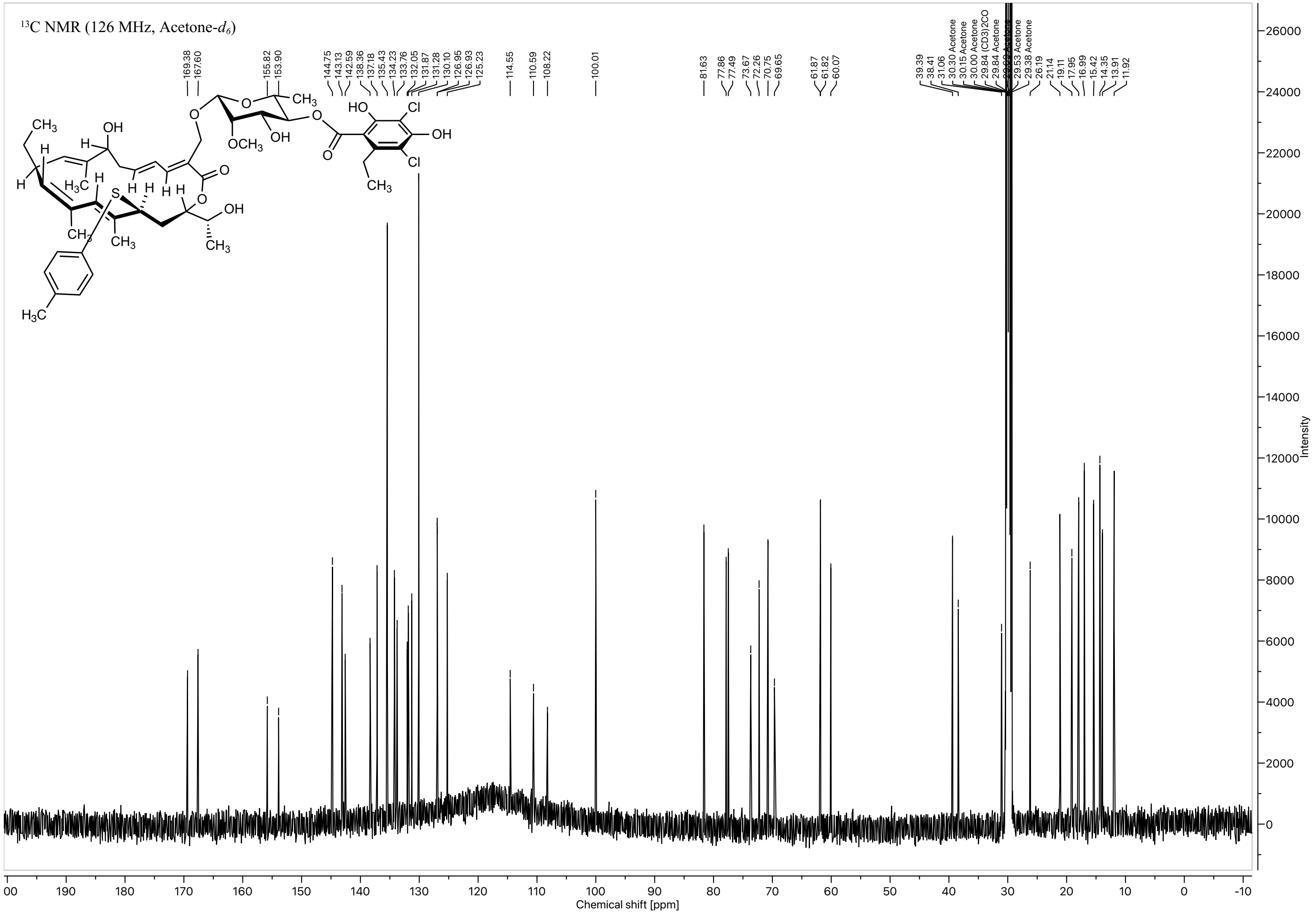

Figure 40: ^{13}C NMR spectrum of 11-desnoviosyl-15-*p*-tolylsulfide fidaxomicin (5b-C(15)) in acetone-d_6

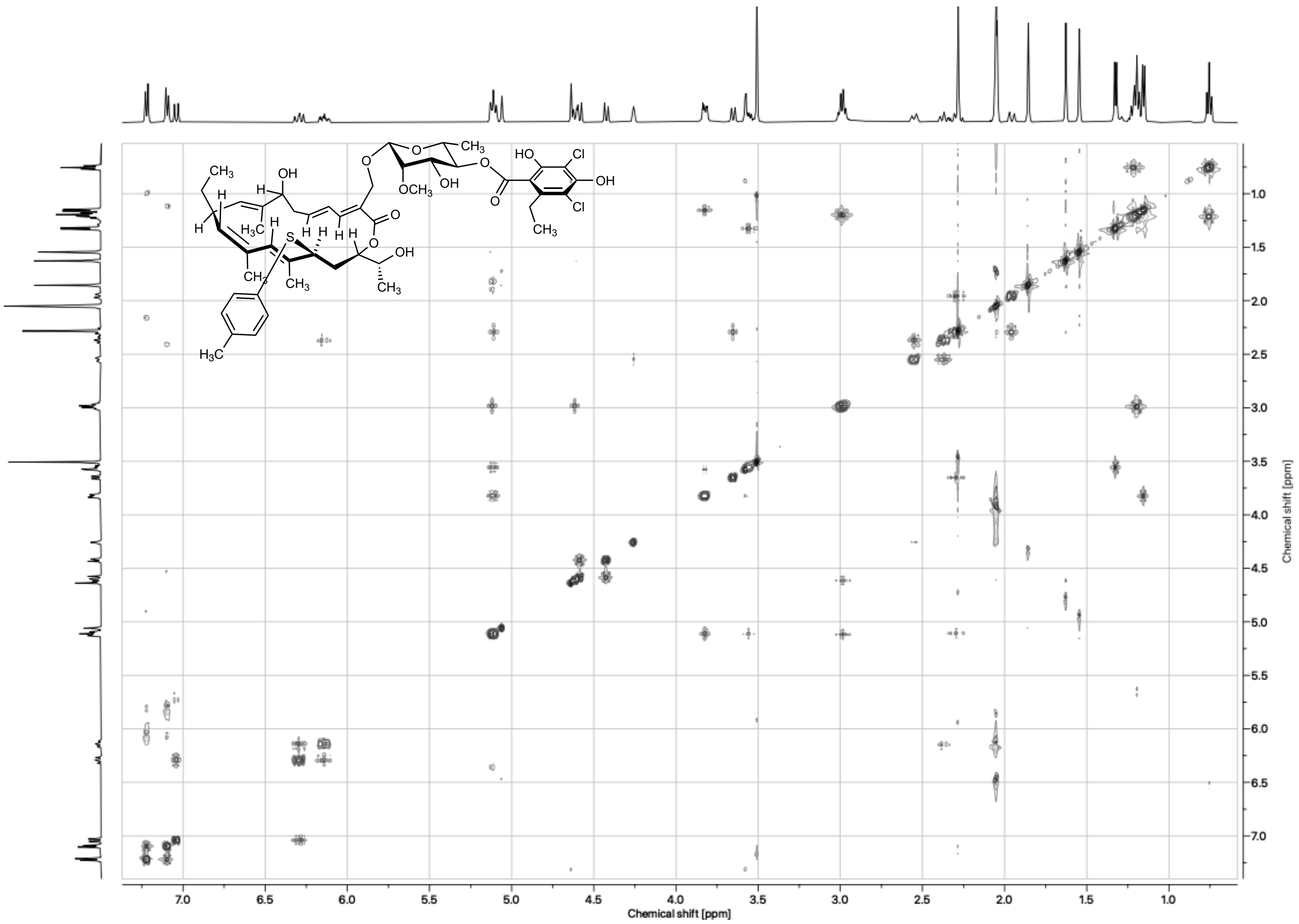

Figure 41: COSY spectrum of 11-desnoviosyl-15-*p*-tolylsulfide fidaxomicin (5b-C(15)) in acetone-d_6

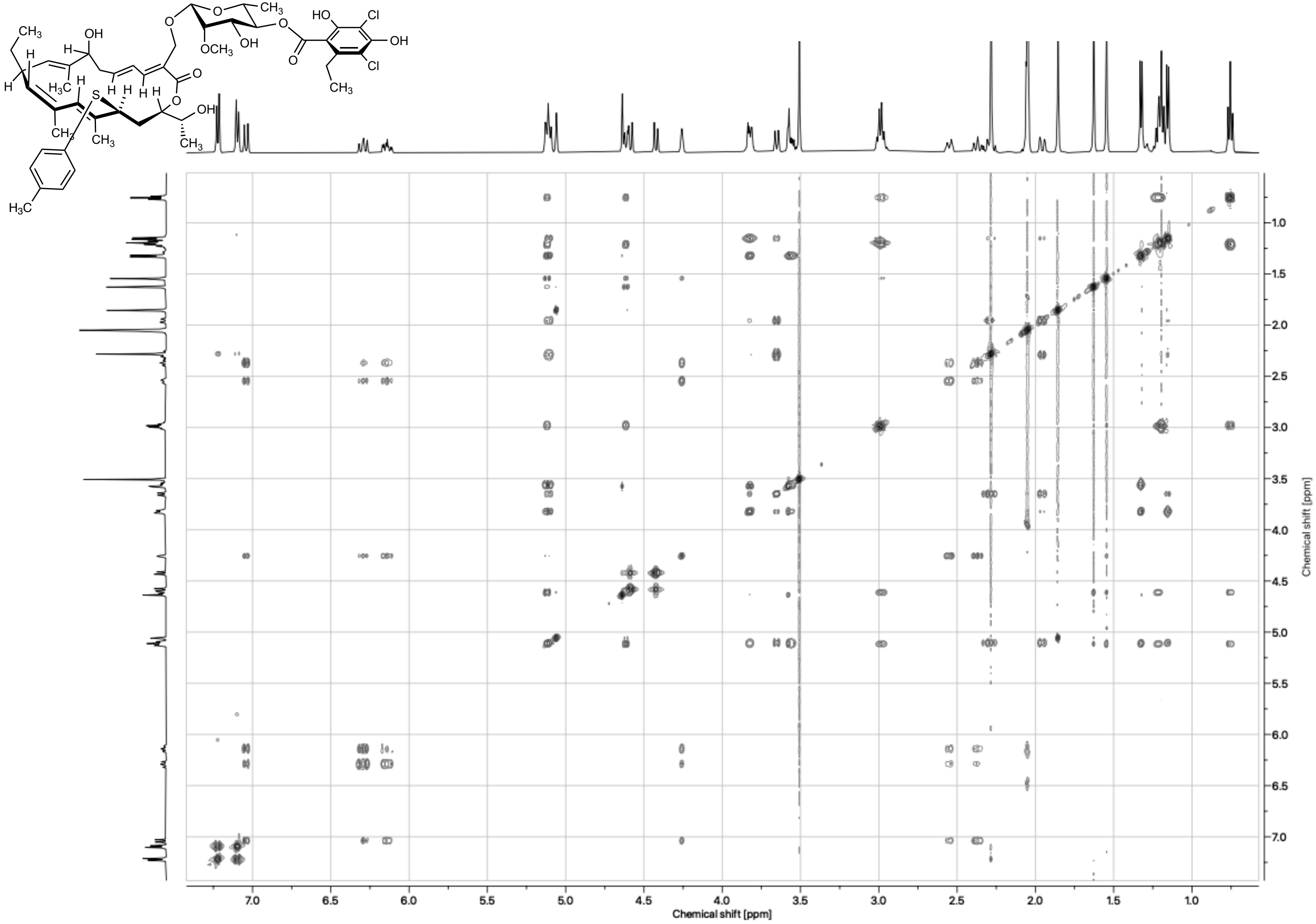

Figure 42: TOCSY spectrum of 11-desnoviosyl-15-*p*-tolylsulfide fidaxomicin (5b-C(15)) in acetone-d_6

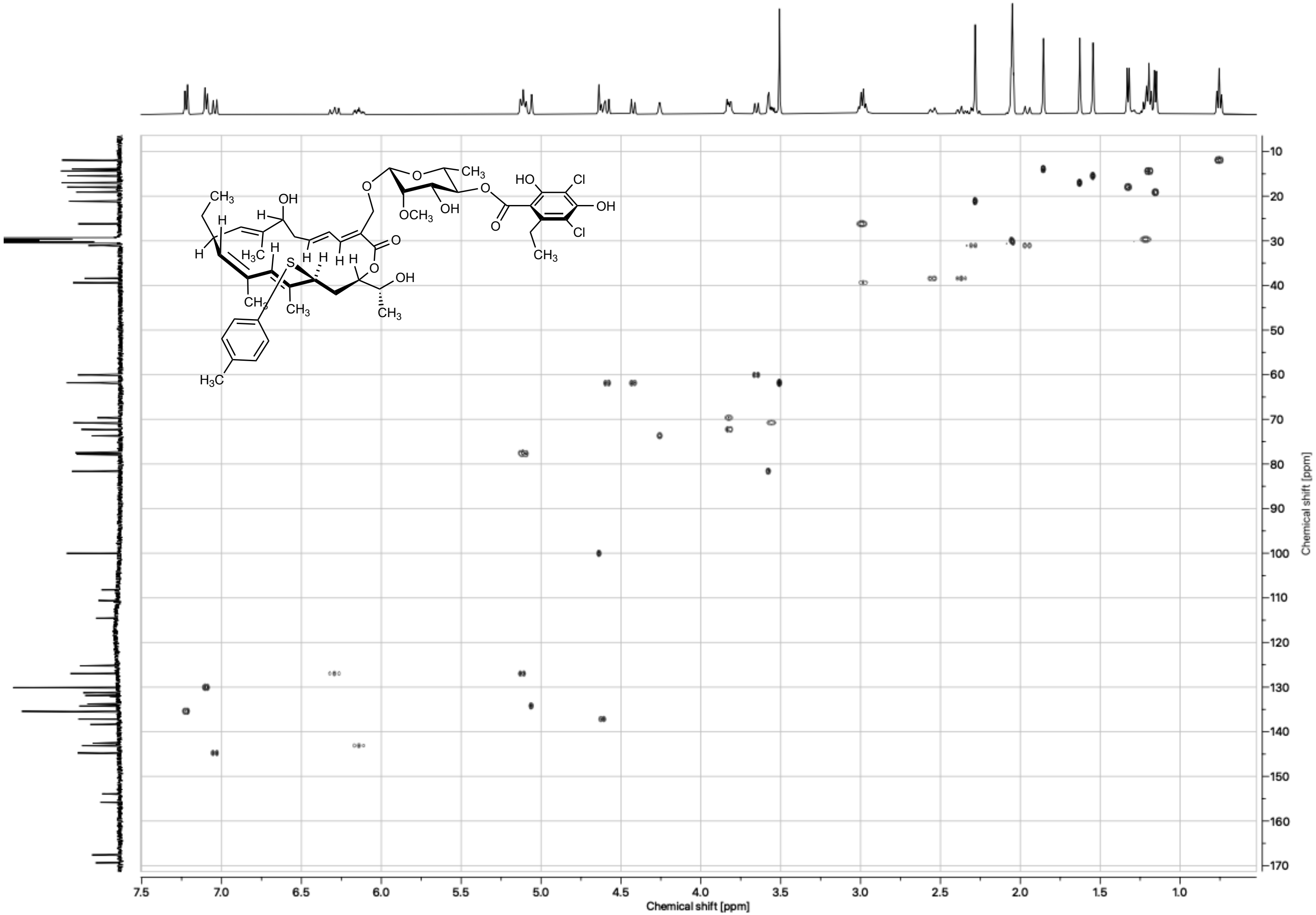

Figure 43: HSQC spectrum of 11-desnoviosyl-15-*p*-tolylsulfide fidaxomicin (5b-C(15)) in acetone-d_6

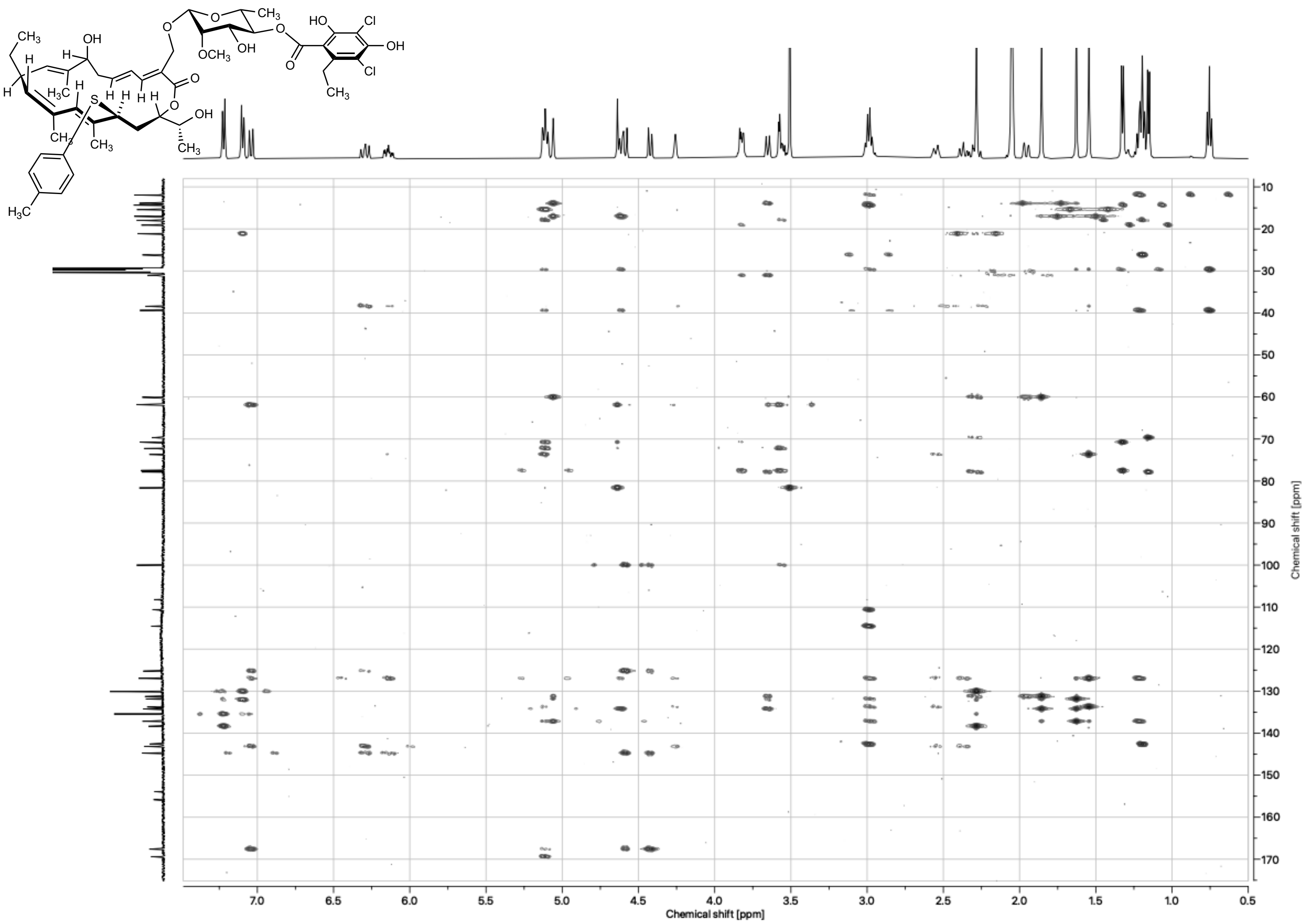

Figure 44: HMBC spectrum of 11-desnoviosyl-15-*p*-tolylsulfide fidaxomicin (5b-C(15)) in acetone-d_6

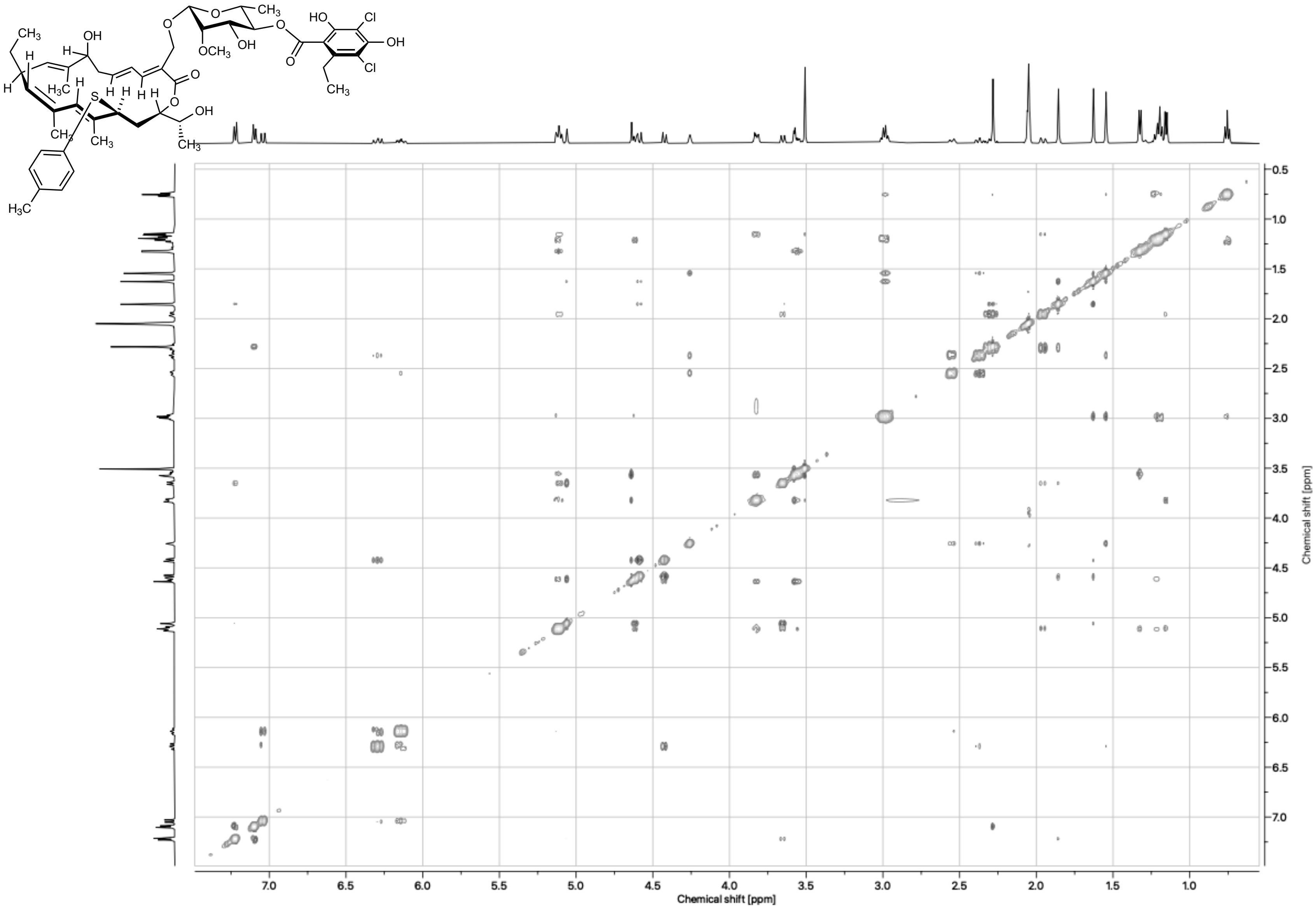

Figure 45: NOESY spectrum of 11-desnoviosyl-15-*p*-tolylsulfide fidaxomicin (5b-C(15)) in acetone-d_6

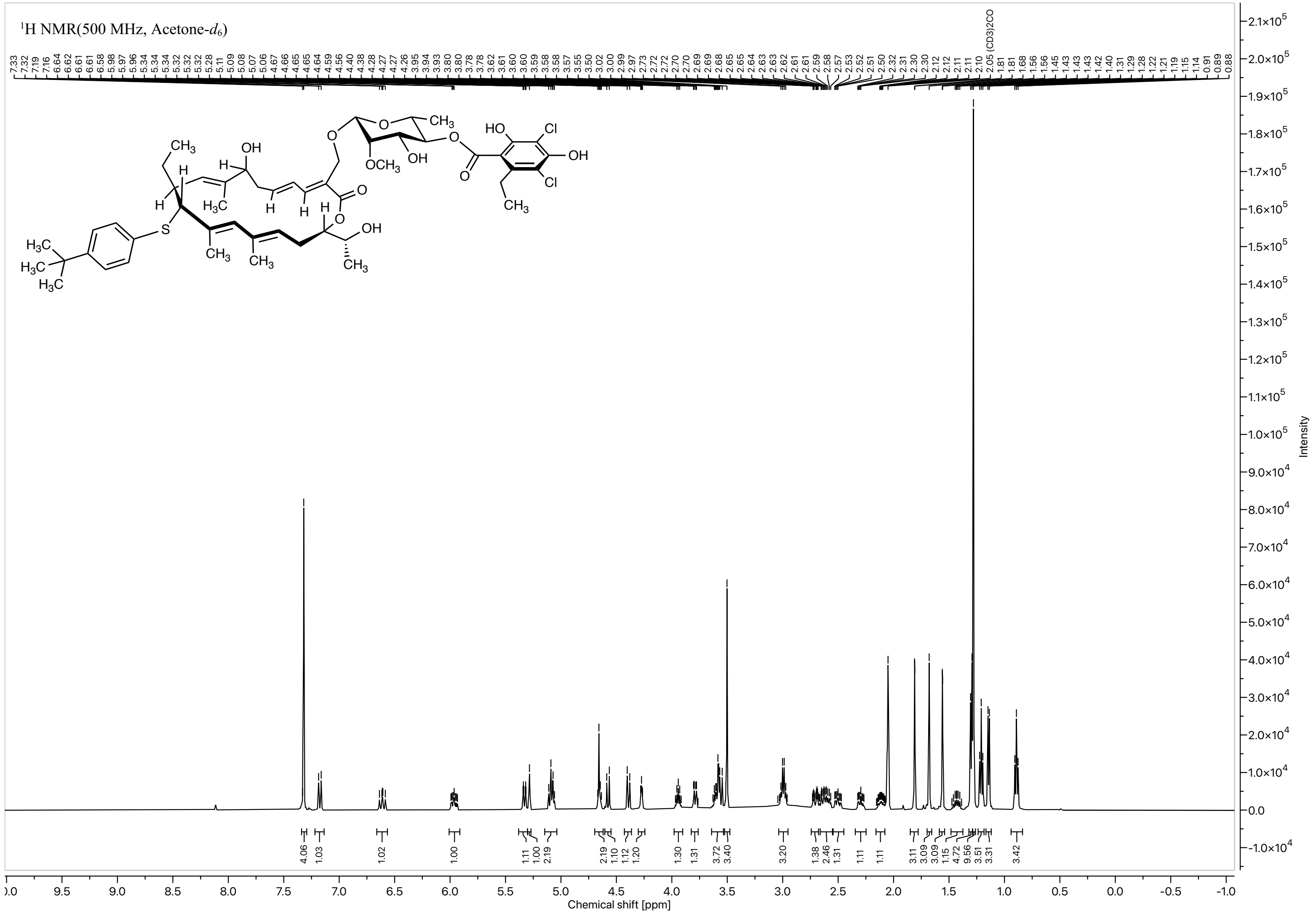

Figure 46: ^{1}H NMR spectrum of 11-desnoviosyl-11-*p*-*tert*-butylbenzenesulfide fidaxomicin (5c-C(11)) in acetone-d_6

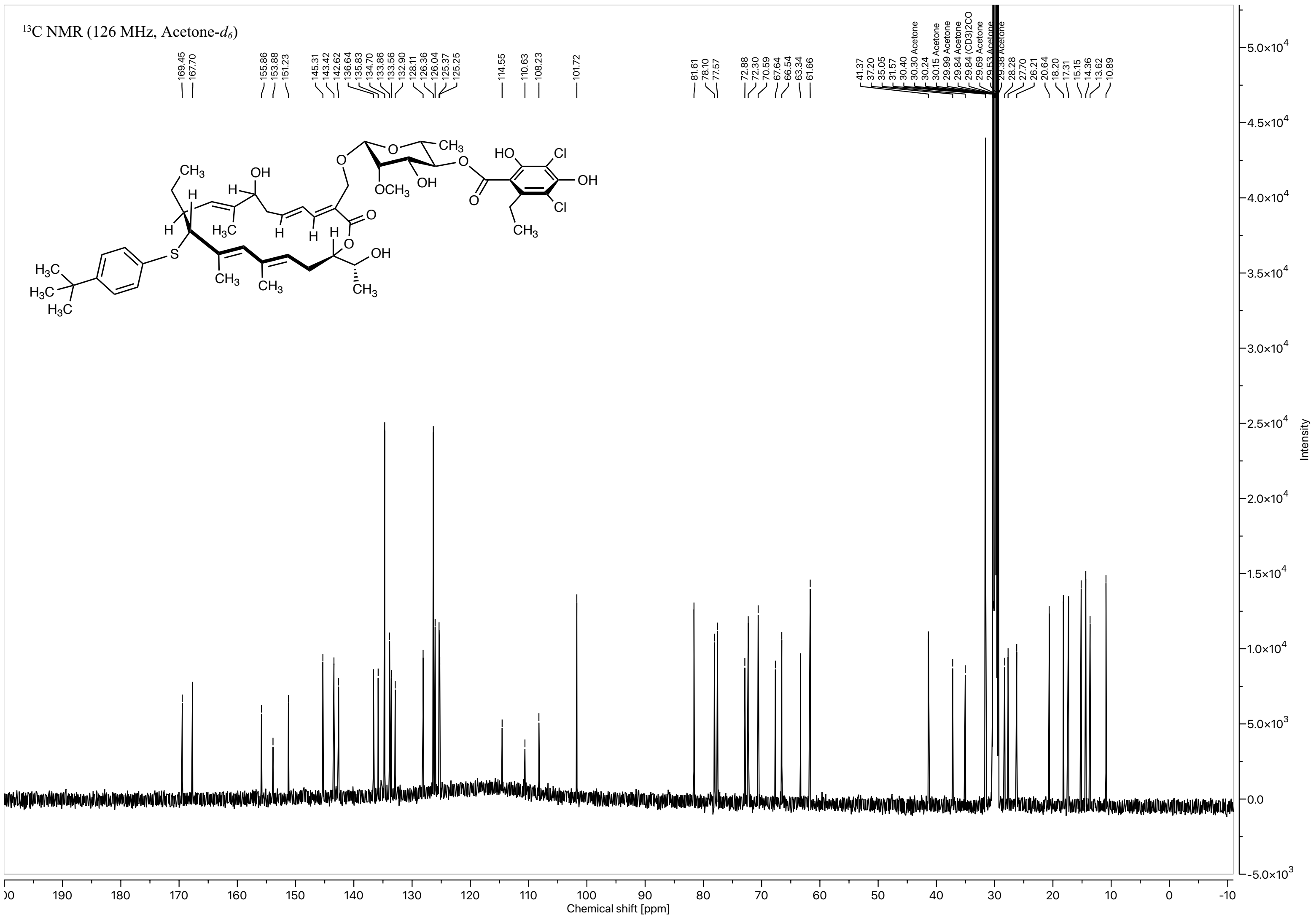

Figure 47: ^{13}C NMR spectrum of 11-desnoviosyl-11-*p*-*tert*-butylbenzenesulfide fidaxomicin (5c-C(11)) in acetone-d_6

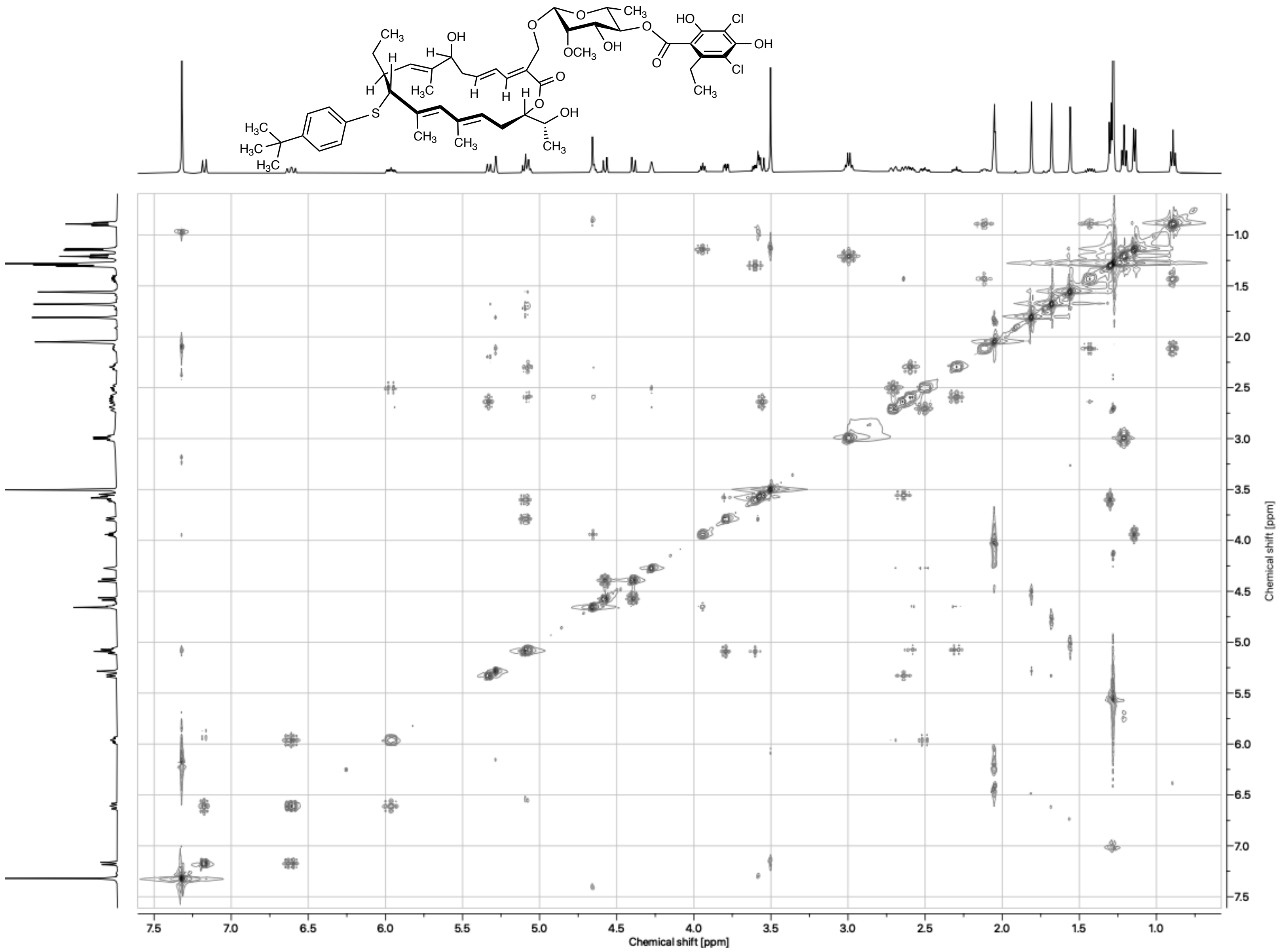

Figure 48: COSY spectrum of 11-desnoviosyl-11-*p*-*tert*-butylbenzenesulfide fidaxomicin (5c-C(11)) in acetone-d_6

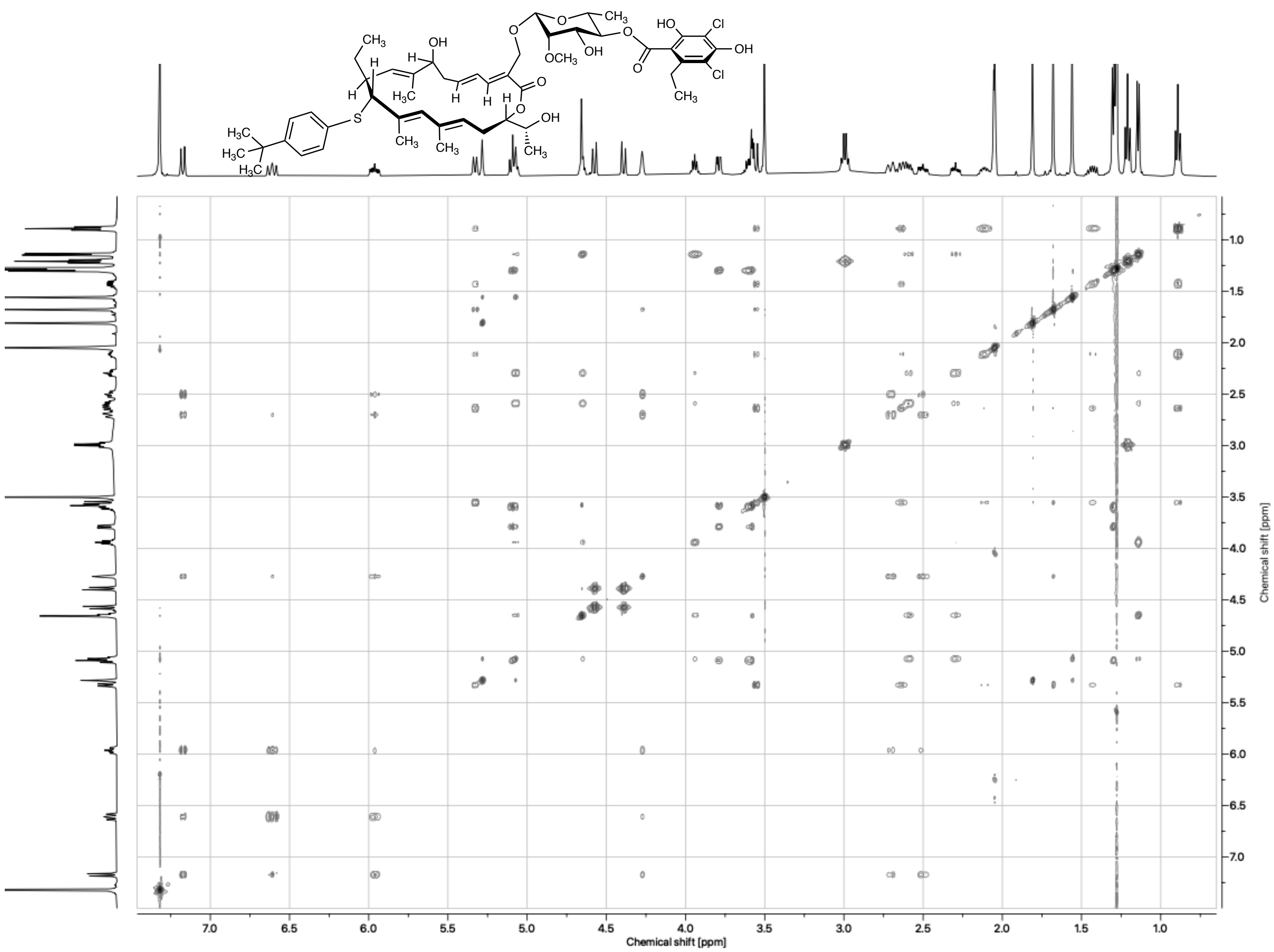

Figure 49: TOCSY spectrum of 11-desnoviosyl-11-*p-tert*-butylbenzenesulfide fidaxomicin (5c-C(11)) in acetone-d_6

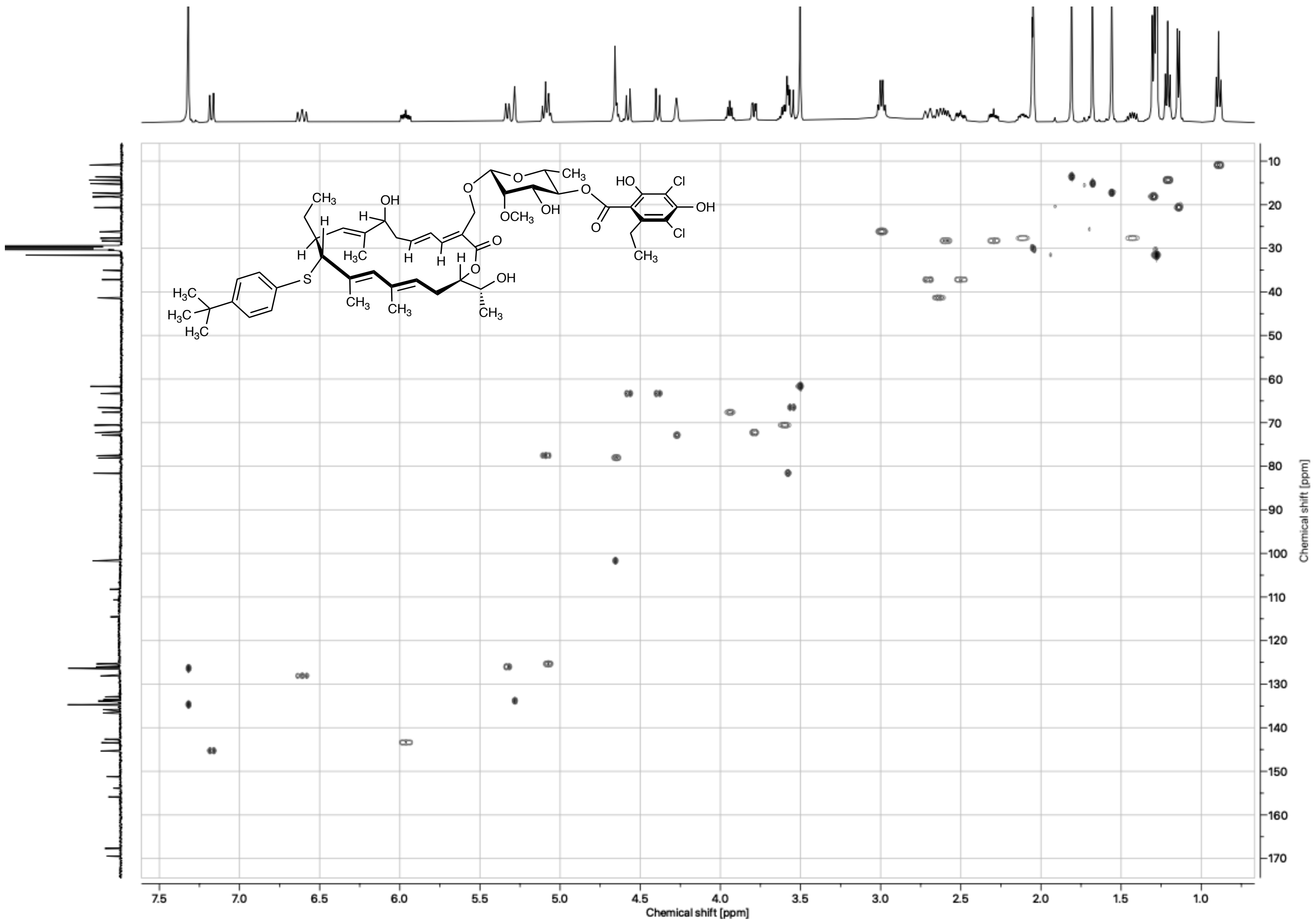

Figure 50: HSQC spectrum of 11-desnoviosyl-11-*p*-*tert*-butylbenzenesulfide fidaxomicin (5c-C(11)) in acetone-d_6

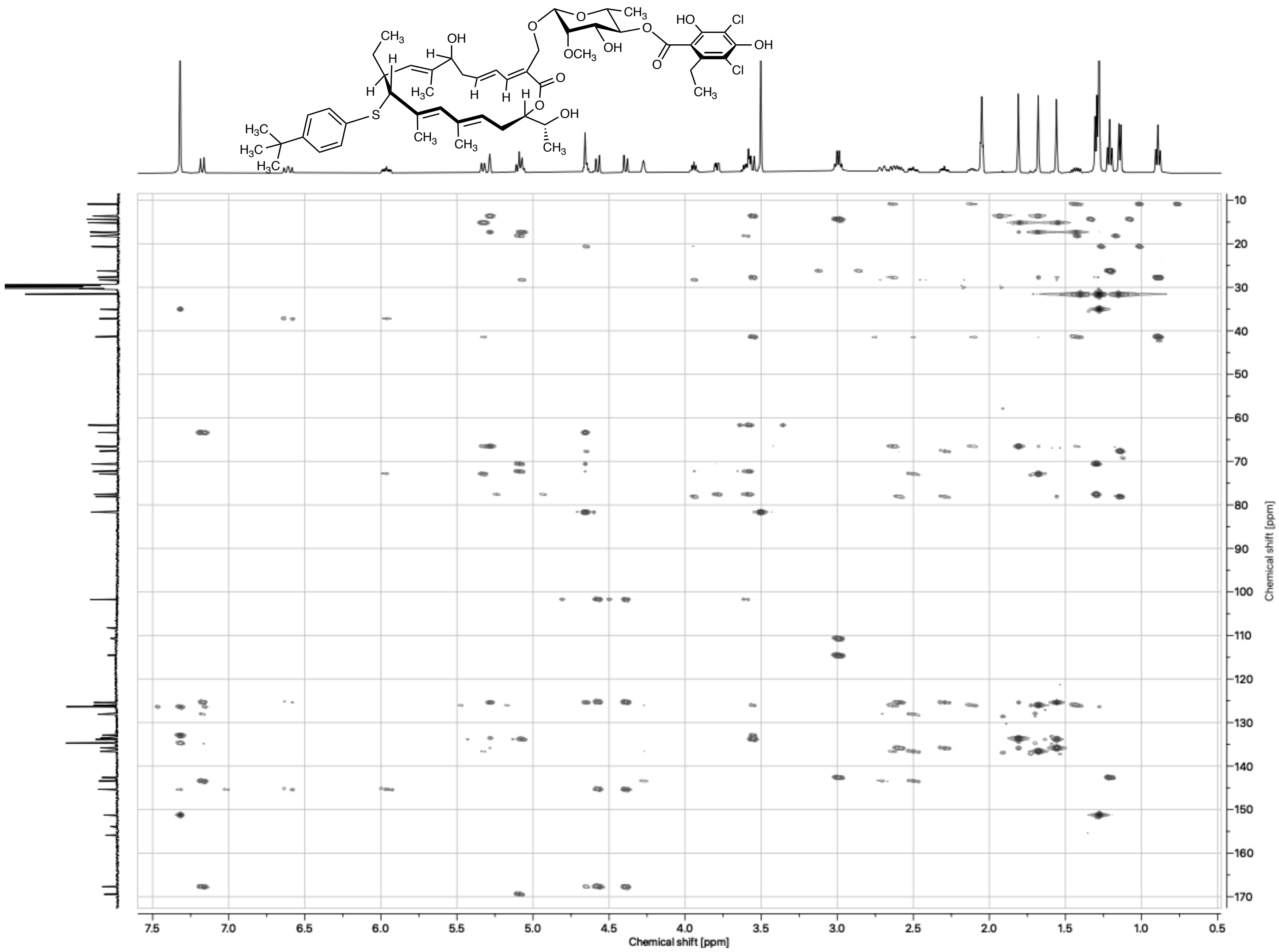

Figure 51: HMBC spectrum of 11-desnoviosyl-11-*p*-*tert*-butylbenzenesulfide fidaxomicin (5c-C(11)) in acetone-d_6

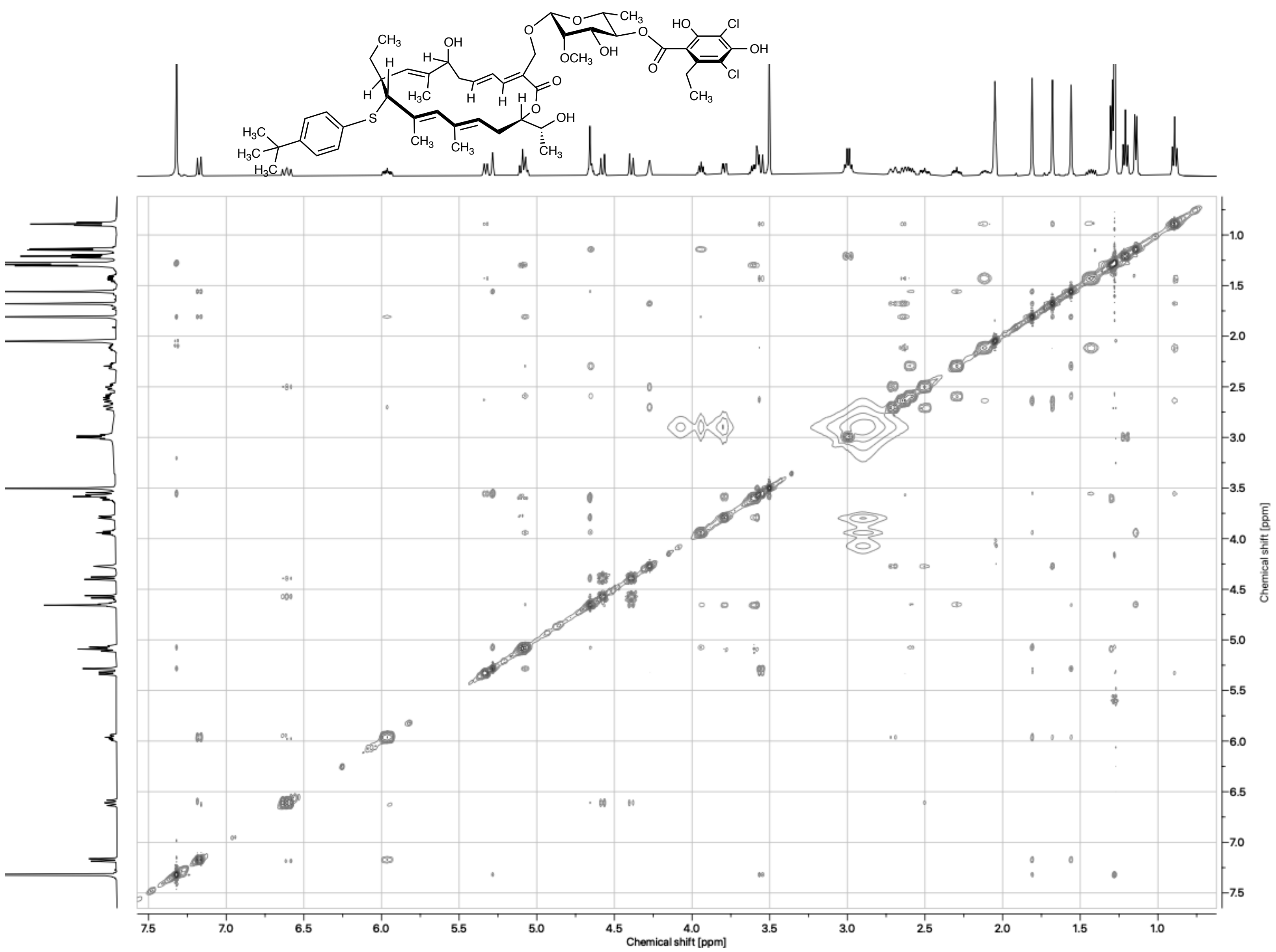

Figure 52: NOESY spectrum of 11-desnoviosyl-11-*p*-*tert*-butylbenzenesulfide fidaxomicin (5c-C(11)) in acetone-d_6

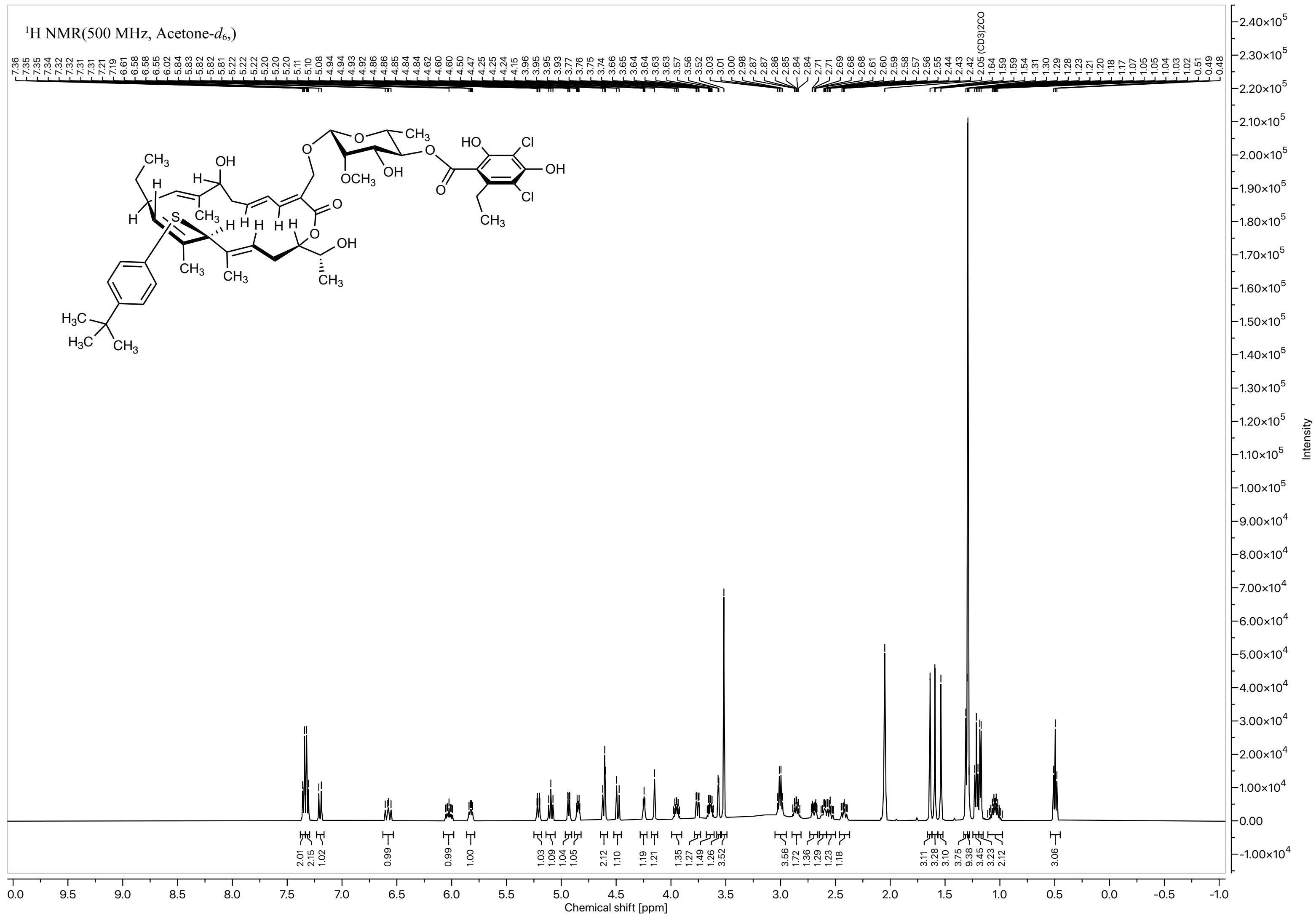

Figure 53: ^{1}H NMR spectrum of 11-desnoviosyl-13-*p*-*tert*-butylbenzenesulfide fidaxomicin (5c-C(13)) in acetone-d_6

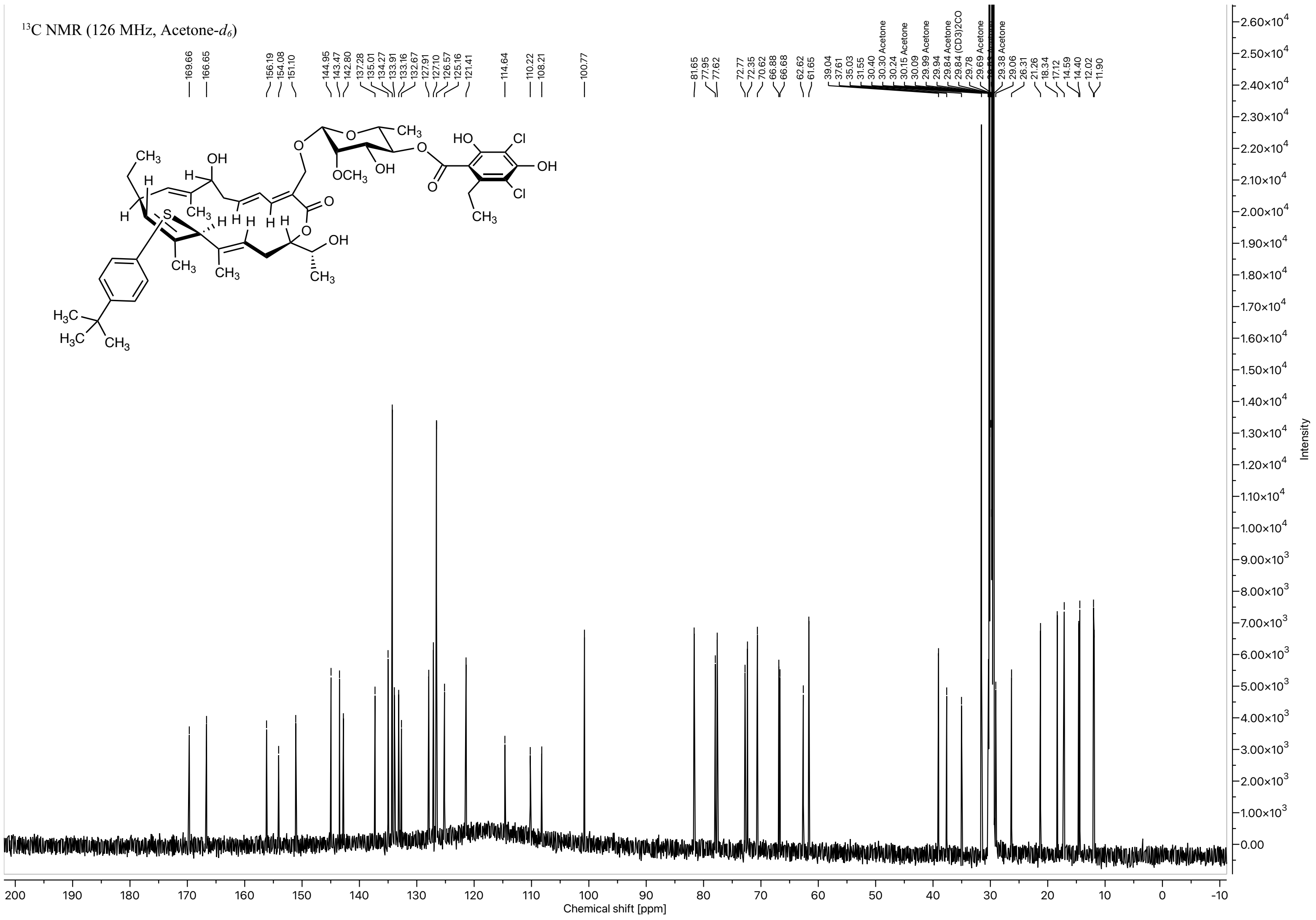

Figure 54: ^{13}C NMR spectrum of 11-desnoviosyl-13-*p*-*tert*-butylbenzenesulfide fidaxomicin (5c-C(13)) in acetone-d_6

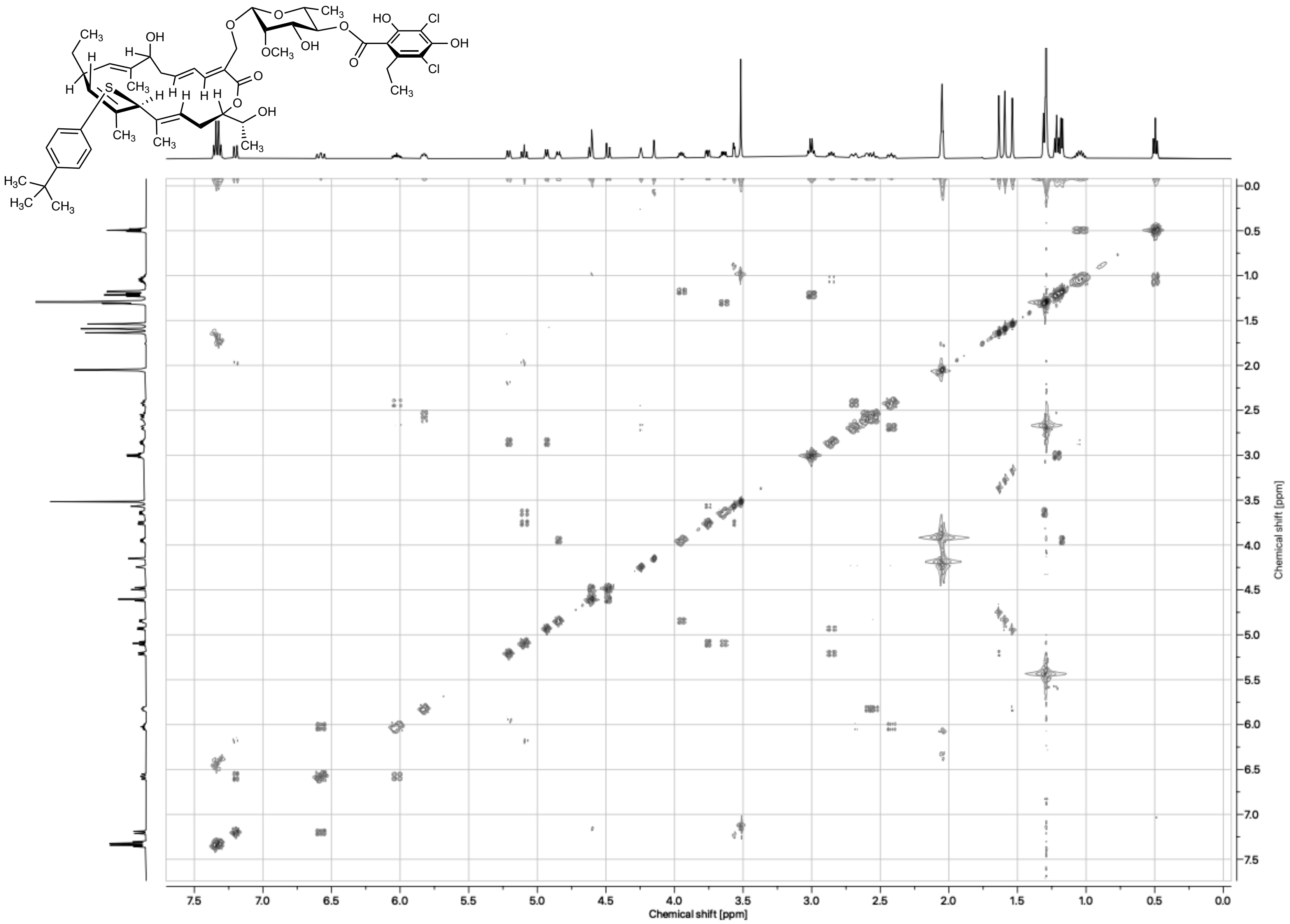

Figure 55: COSY spectrum of 11-desnoviosyl-13-*p*-*tert*-butylbenzenesulfide fidaxomicin (5c-C(13)) in acetone-d_6

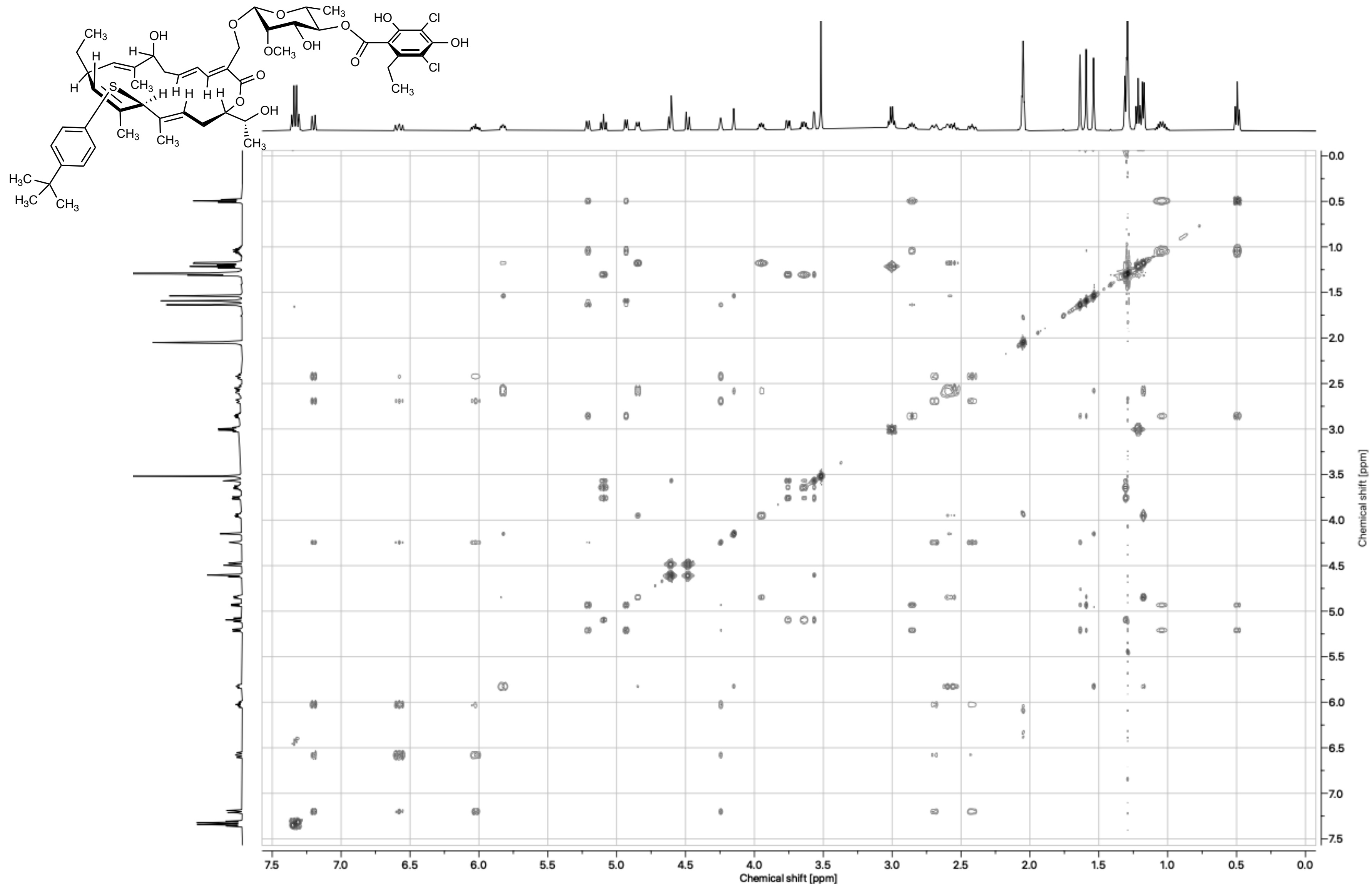

Figure 56: TOCSY spectrum of 11-desnoviosyl-13-*p*-*tert*-butylbenzenesulfide fidaxomicin (5c-C(13)) in acetone-d_6

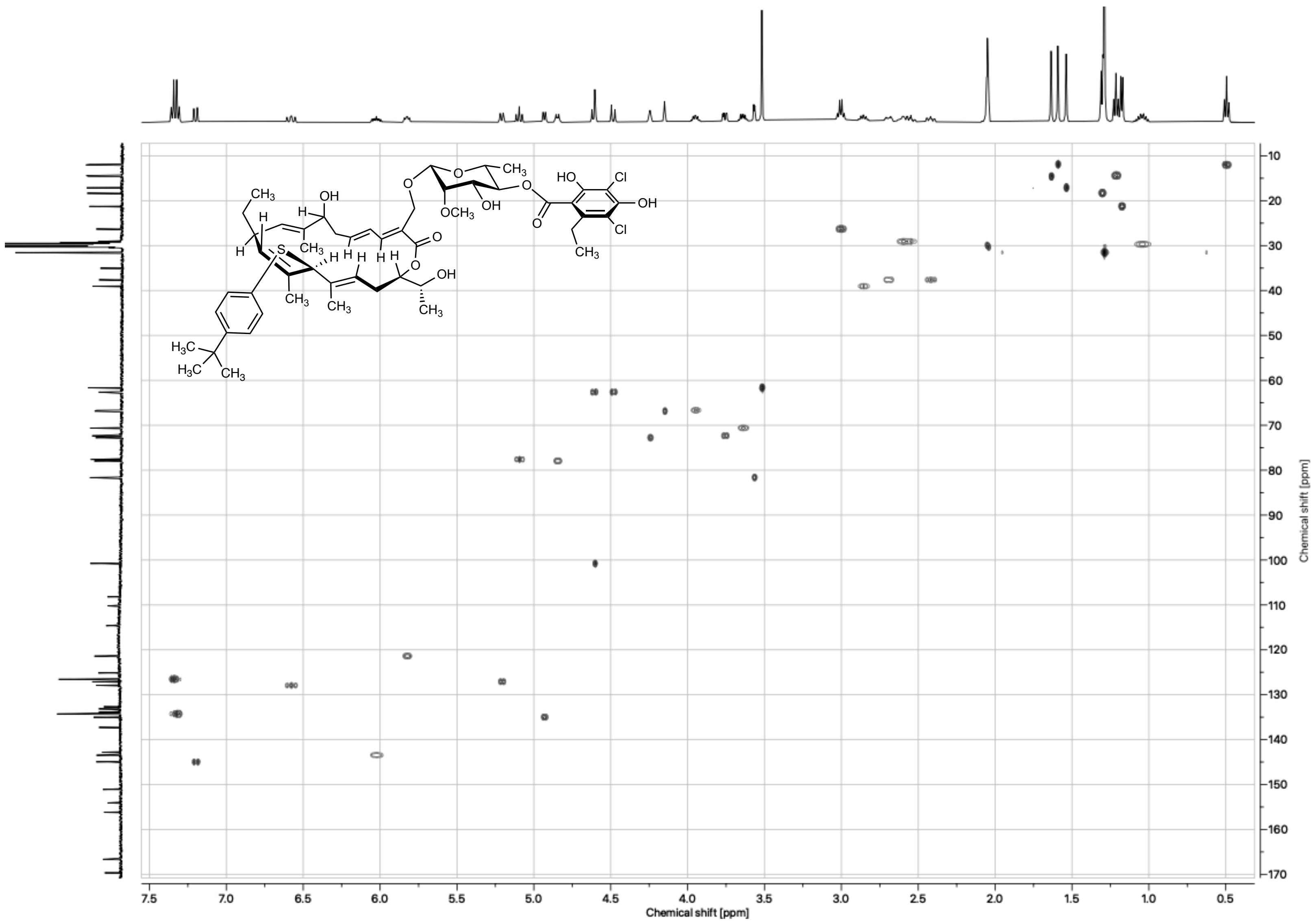

Figure 57: HSQC spectrum of 11-desnoviosyl-13-*p*-*tert*-butylbenzenesulfide fidaxomicin (5c-C(13)) in acetone-d_6

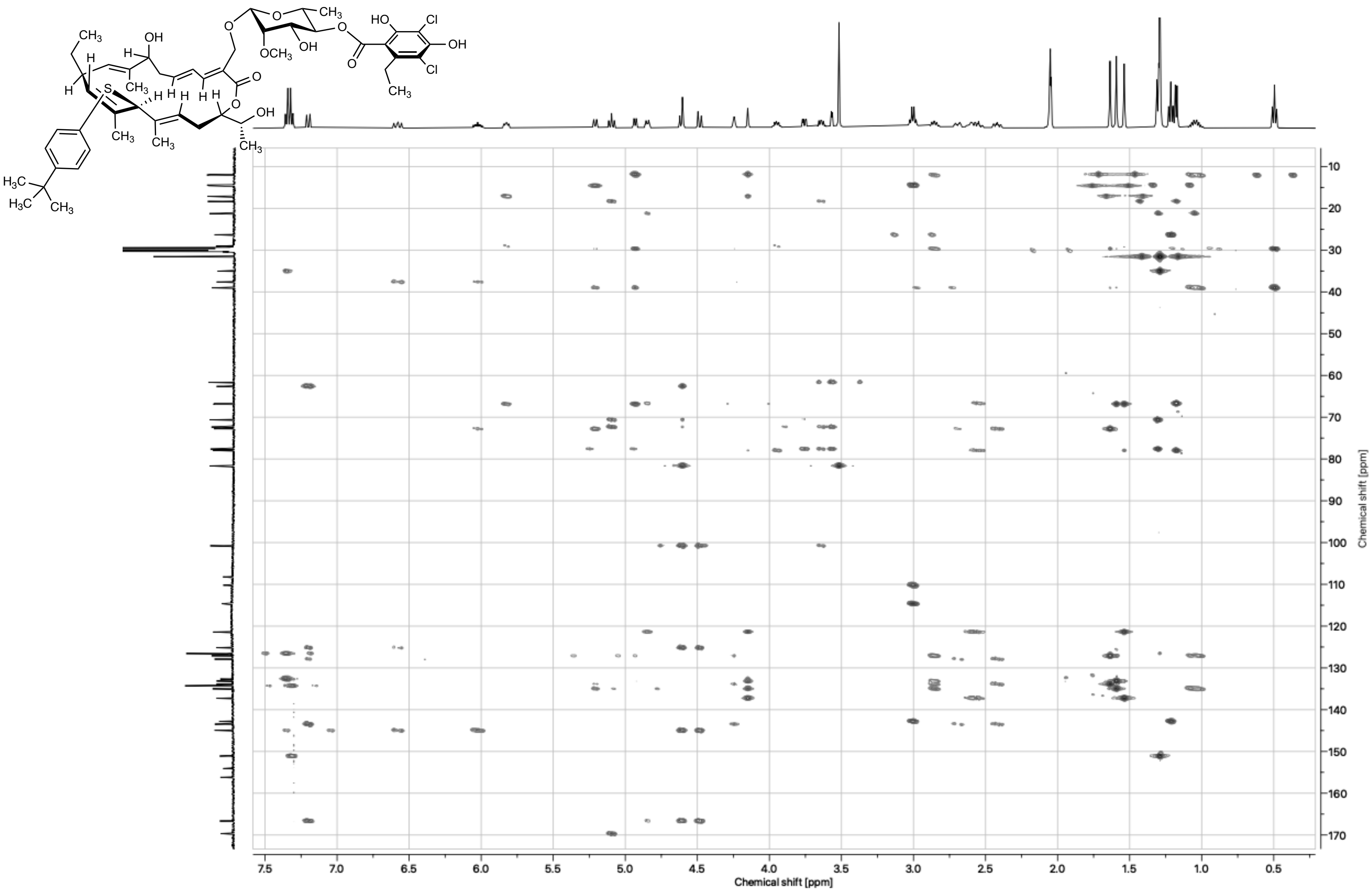

Figure 58: HMBC spectrum of 11-desnoviosyl-13-*p*-*tert*-butylbenzenesulfide fidaxomicin (5c-C(13)) in acetone-d_6

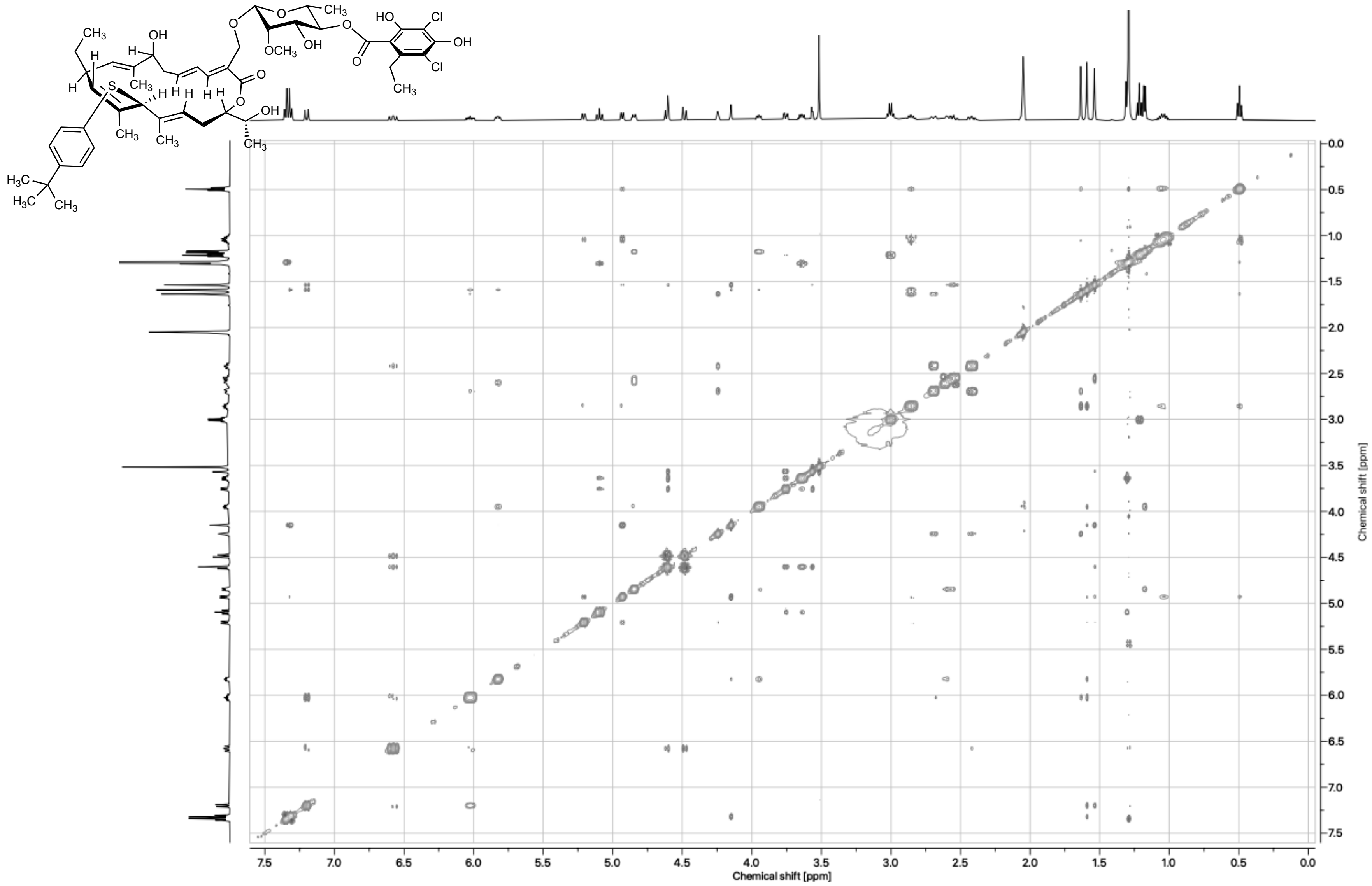

Figure 59: NOESY spectrum of 11-desnoviosyl-13-*p*-*tert*-butylbenzenesulfide fidaxomicin (5c-C(13)) in acetone-d_6

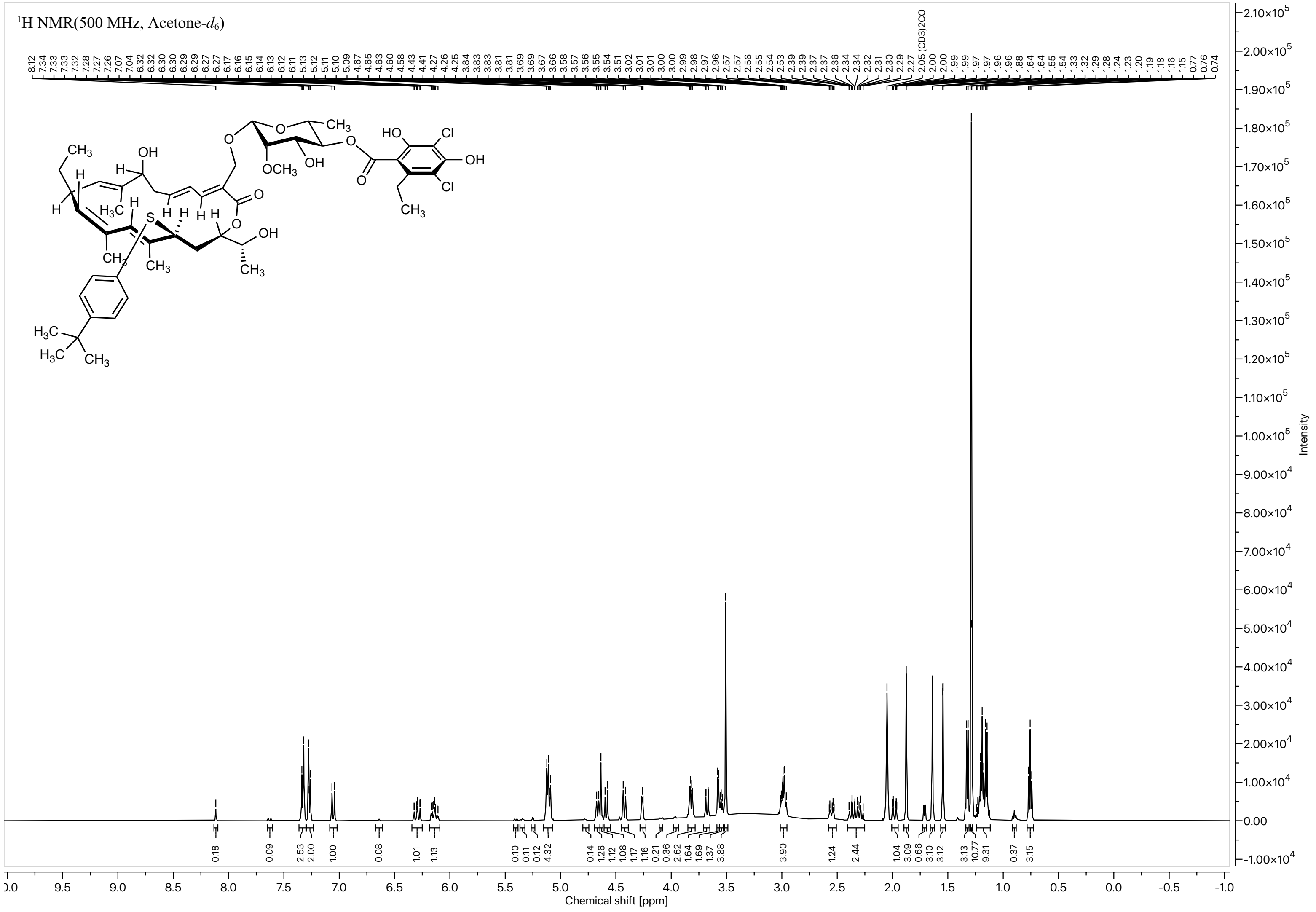

Figure 60: ^{1}H NMR spectrum of 11-desnoviosyl-15-*p*-*tert*-butylbenzenesulfide fidaxomicin (5c-C(15)) in acetone-d_6

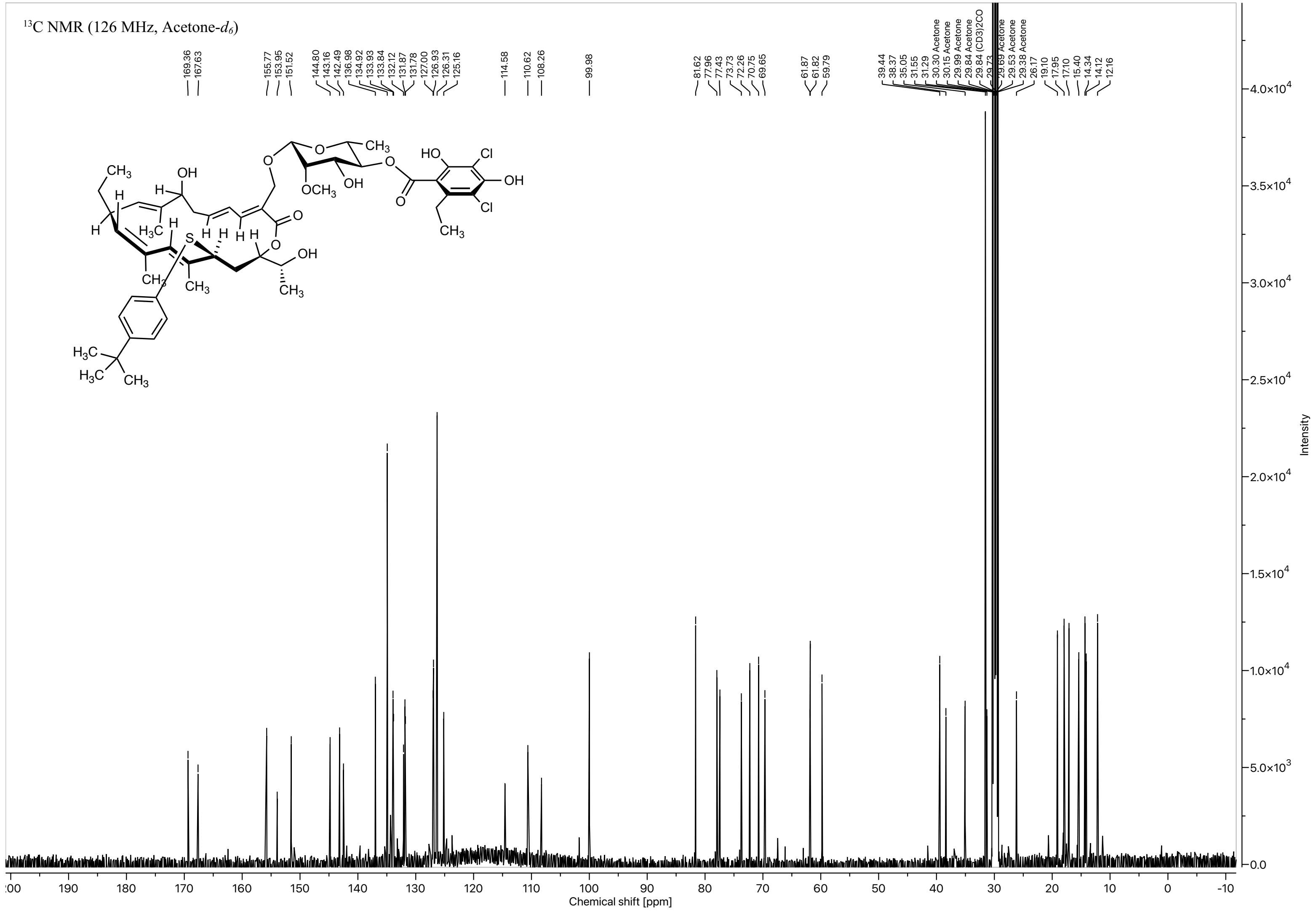

Figure 61: ^{13}C NMR spectrum of 11-desnoviosyl-15-*p*-*tert*-butylbenzenesulfide fidaxomicin (5c-C(15)) in acetone-d_6

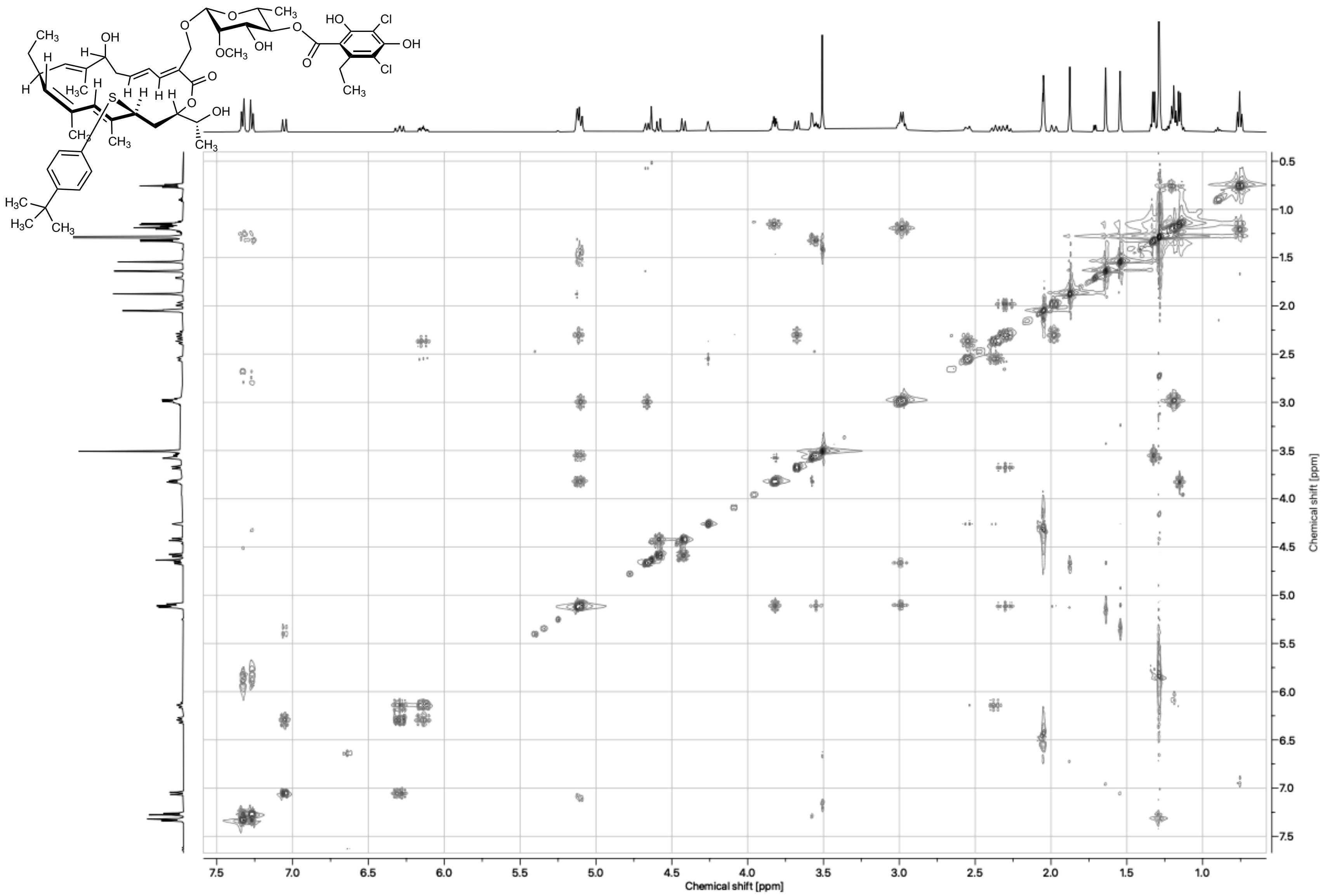

Figure 62: COSY spectrum of 11-desnoviosyl-15-*p*-*tert*-butylbenzenesulfide fidaxomicin (5c-C(15)) in acetone-d_6

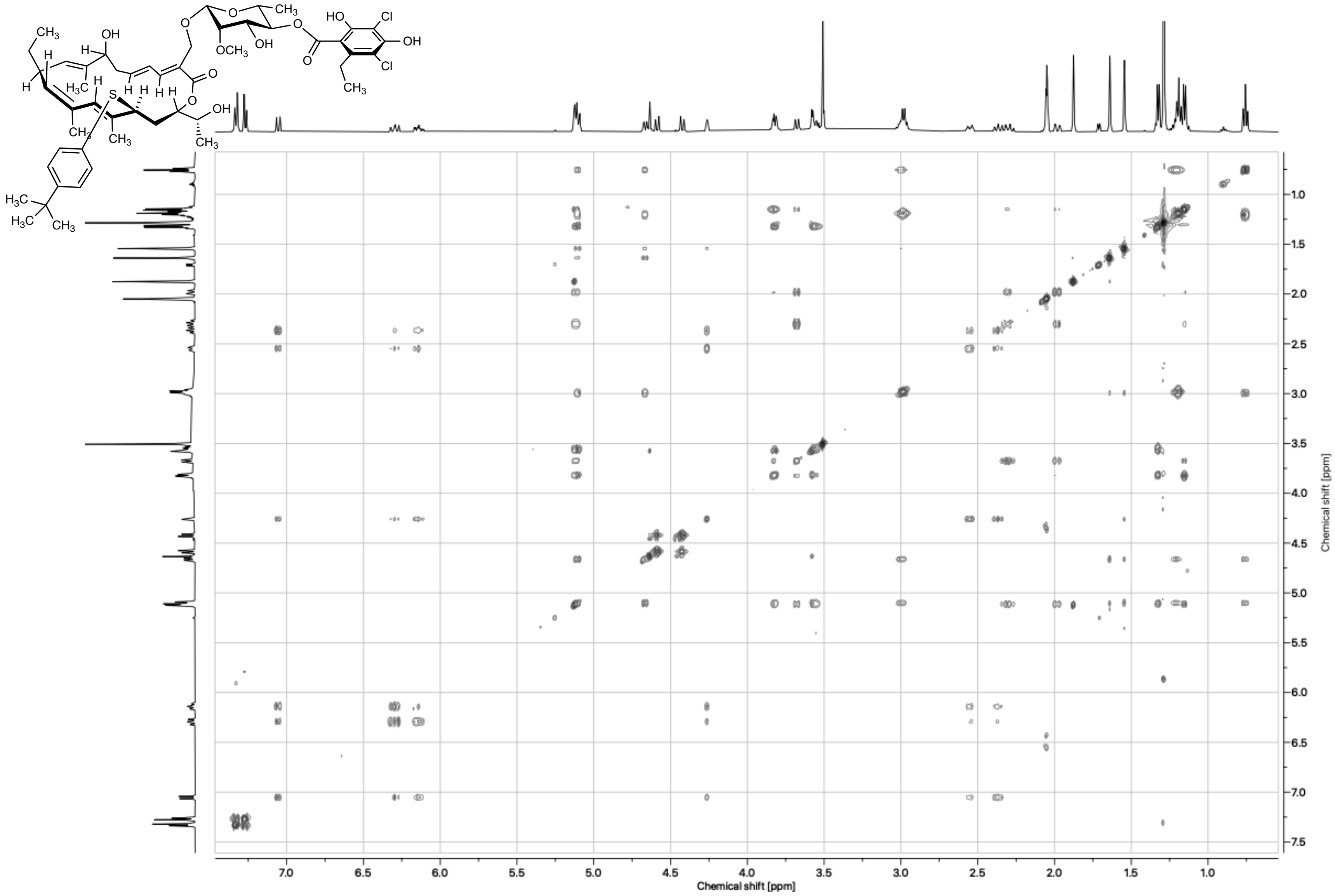

Figure 63: TOCSY spectrum of 11-desnoviosyl-15-*p*-*tert*-butylbenzenesulfide fidaxomicin (5c-C(15)) in acetone-d_6

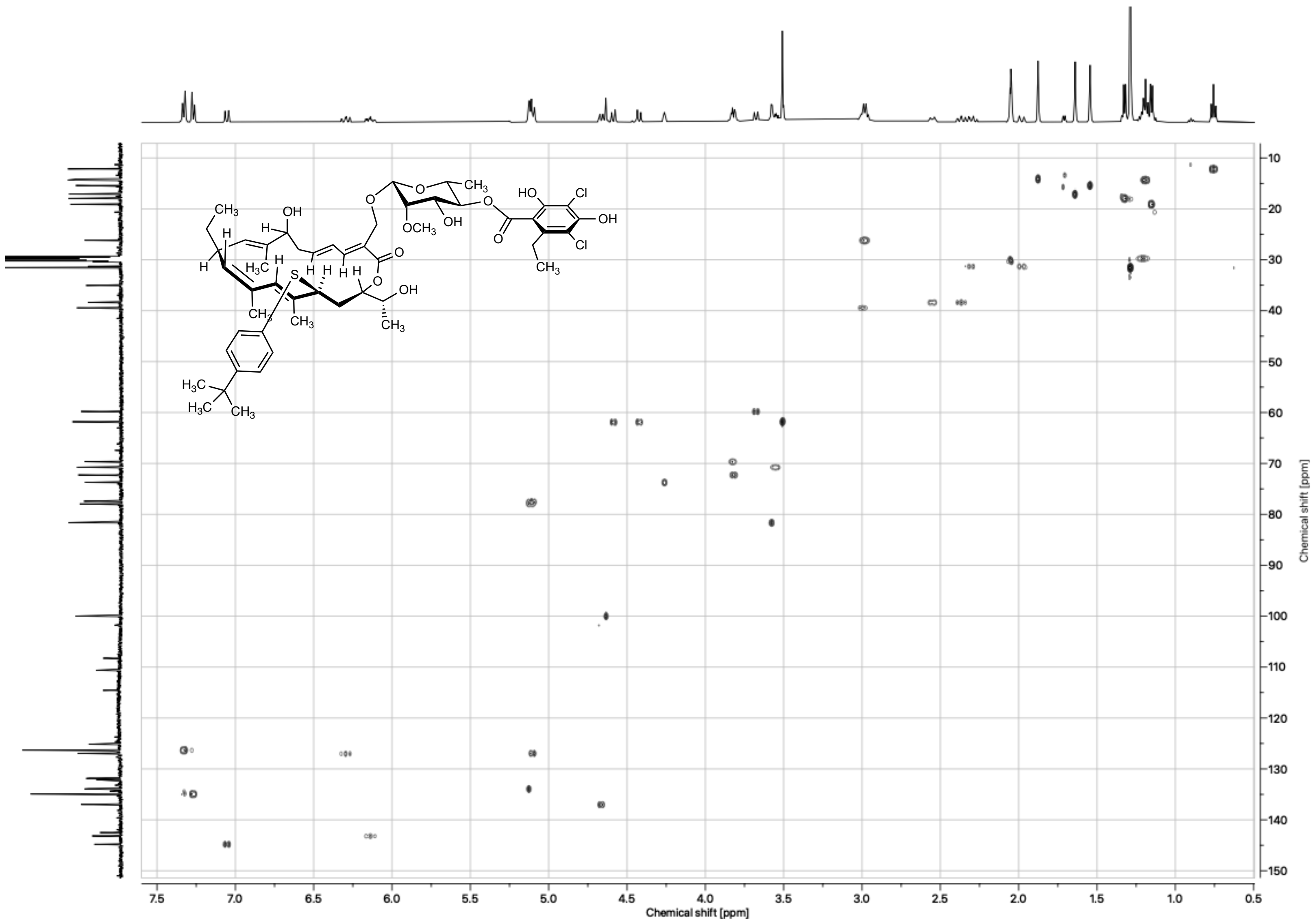

Figure 64: HSQC spectrum of 11-desnoviosyl-15-*p*-*tert*-butylbenzenesulfide fidaxomicin (5c-C(15)) in acetone-d_6

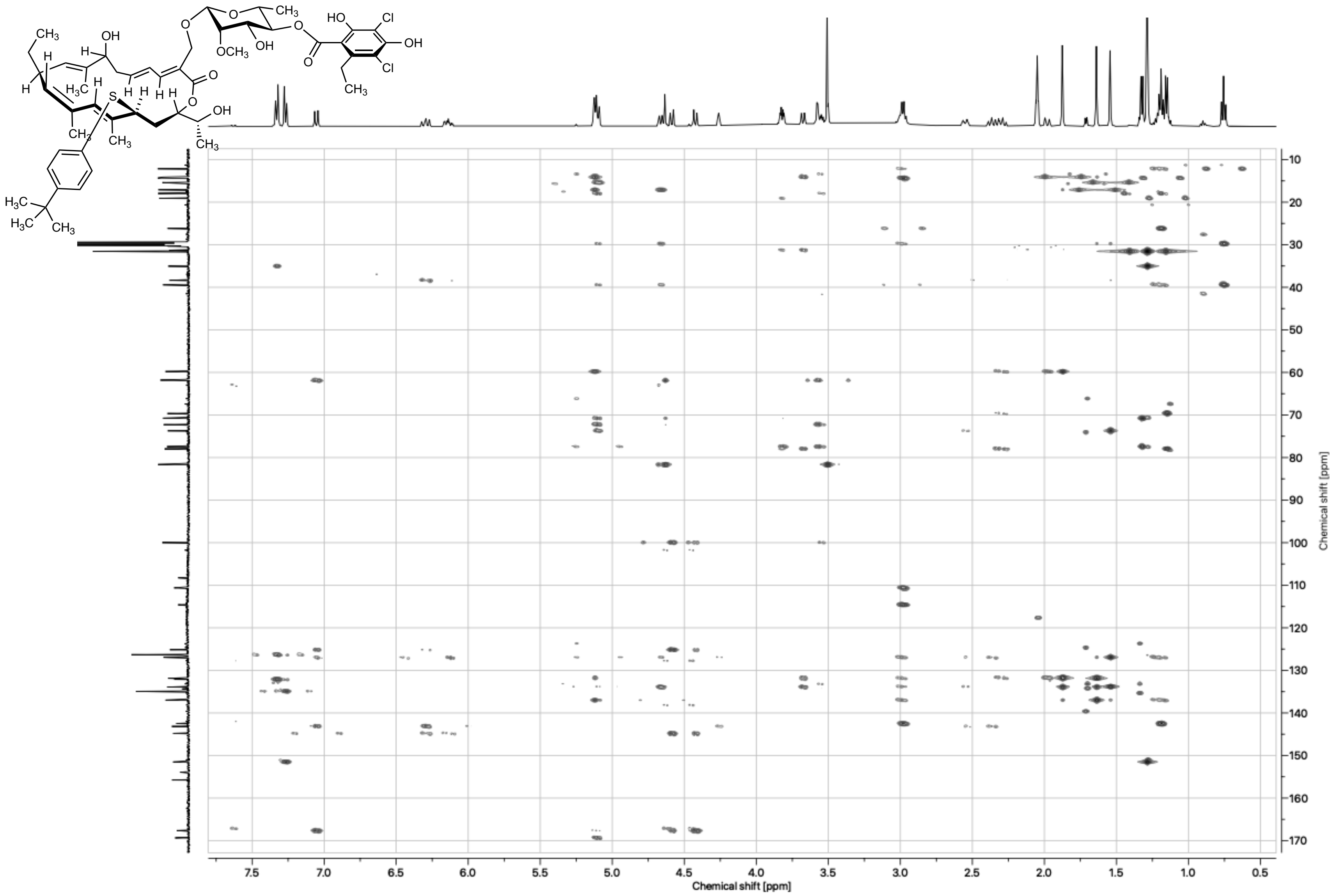

Figure 65: HMBC spectrum of 11-desnoviosyl-15-*p-tert*-butylbenzenesulfide fidaxomicin (5c-C(15)) in acetone-d_6

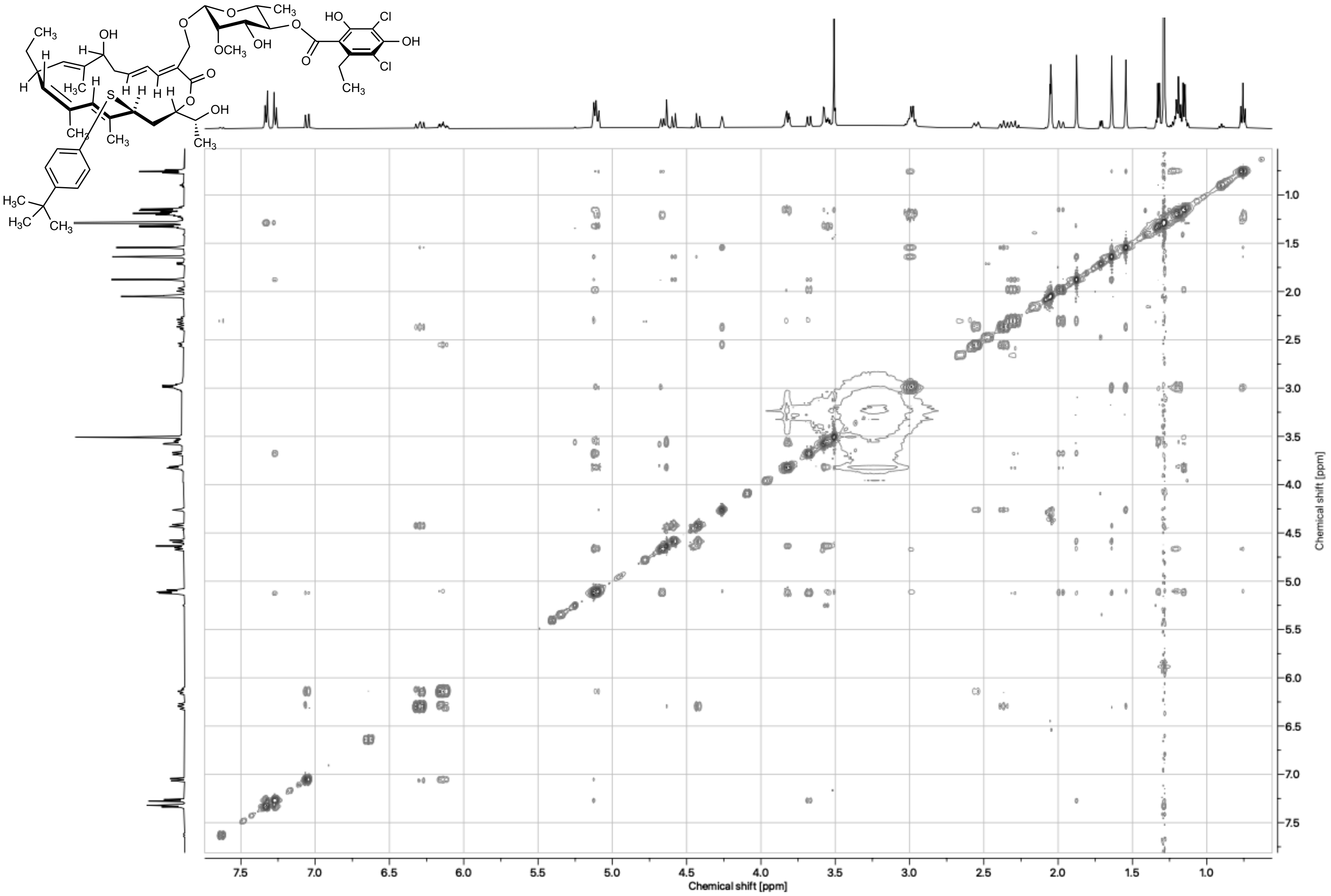

Figure 66: NOESY spectrum of 11-desnoviosyl-15-*p*-*tert*-butylbenzenesulfide fidaxomicin (5c-C(15)) in acetone-d_6

Spectral data for thioglycoside derivatives of Fdx

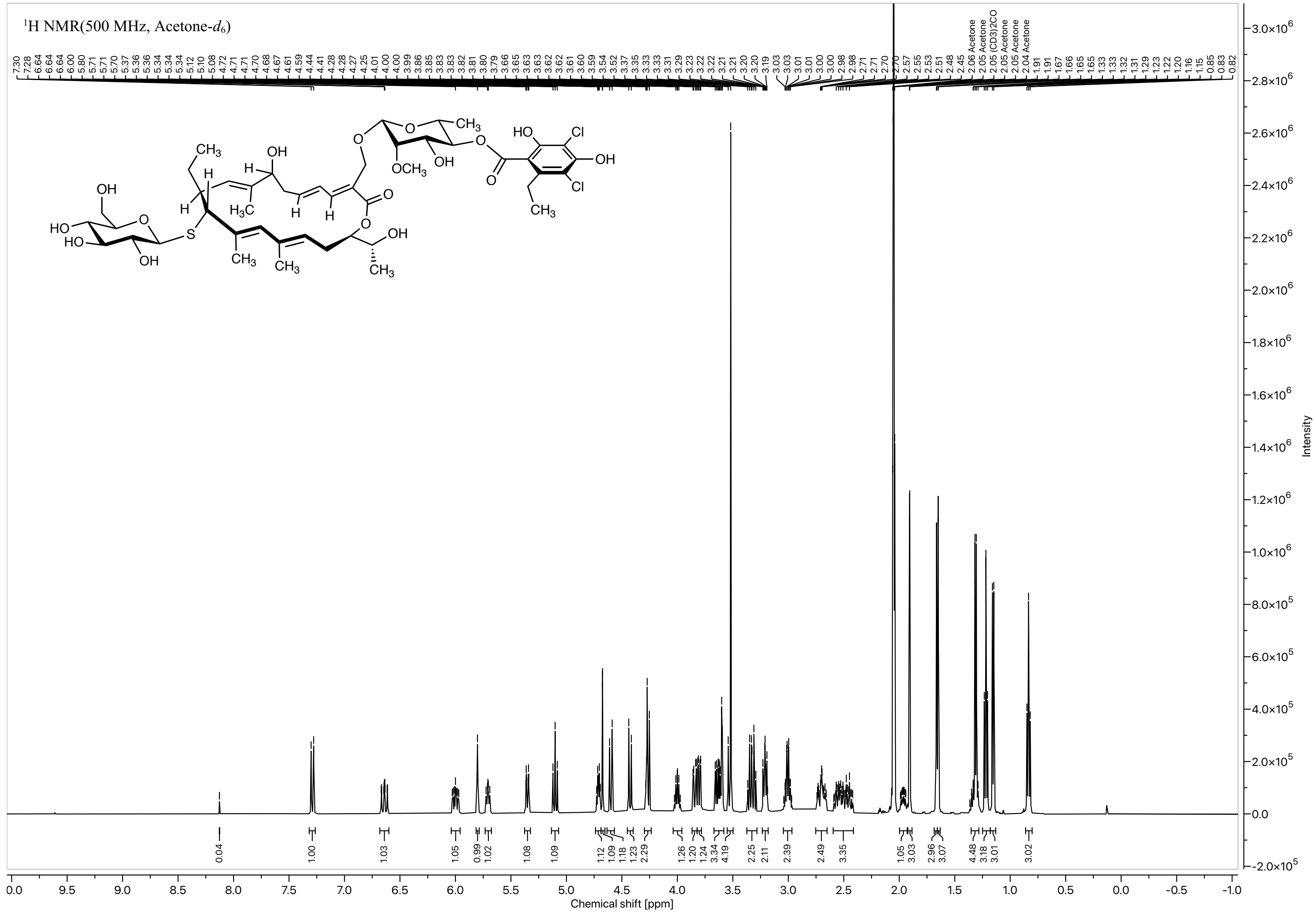

Figure 67: ^{1}H NMR spectrum of 11-desnoviosyl-11-thio-β-D-glucosyl fidaxomicin (18a-C(11)) in acetone-d_6

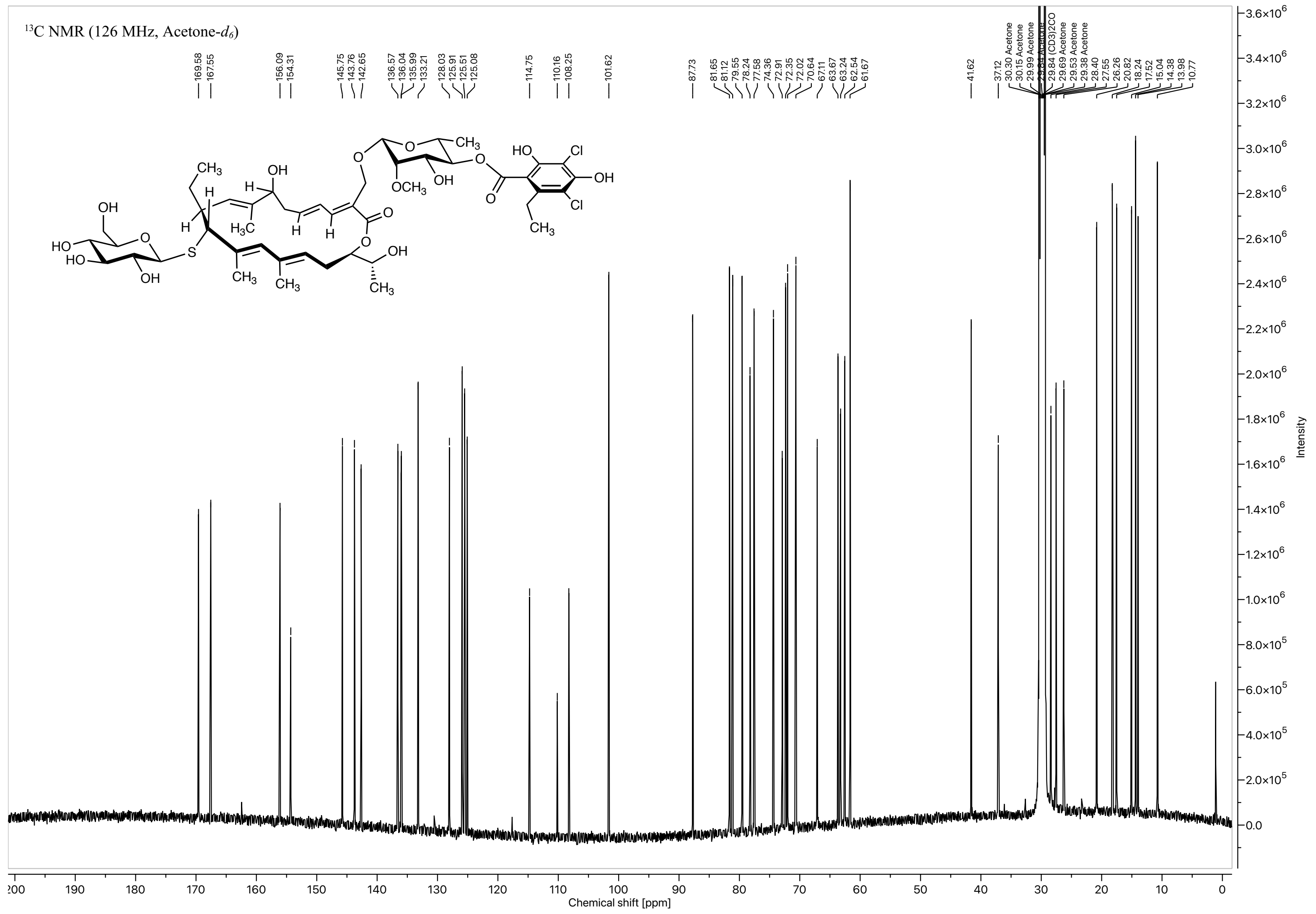

Figure 68: ^{13}C NMR spectrum of 11-desnoviosyl-11-thio-β-D-glucosyl fidaxomicin (18a-C(11)) in acetone-d_6

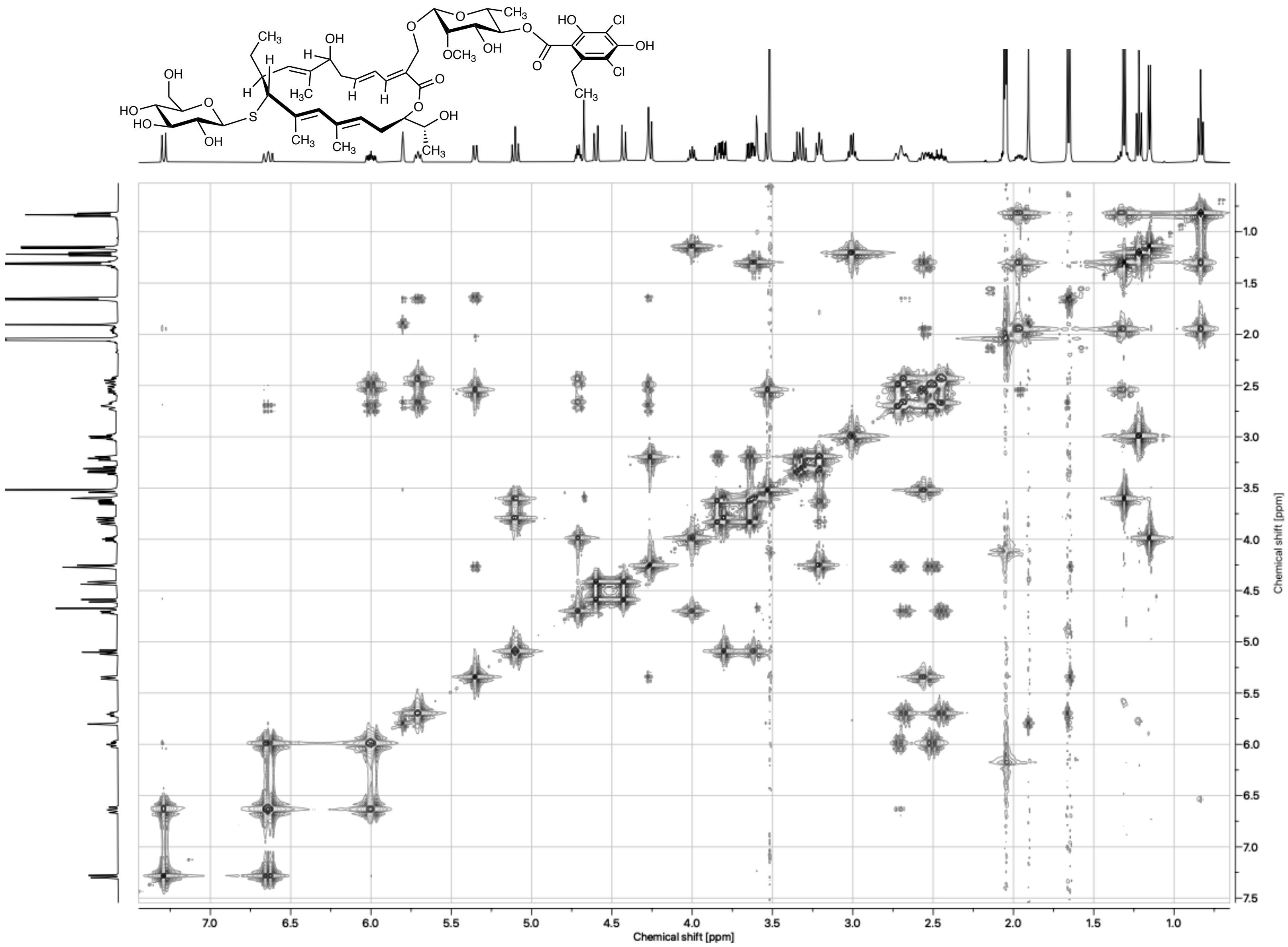

Figure 69: COSY spectrum of 11-desnoviosyl-11-thio-β-D-glucosyl fidaxomicin (18a-C(11)) in acetone-d_6

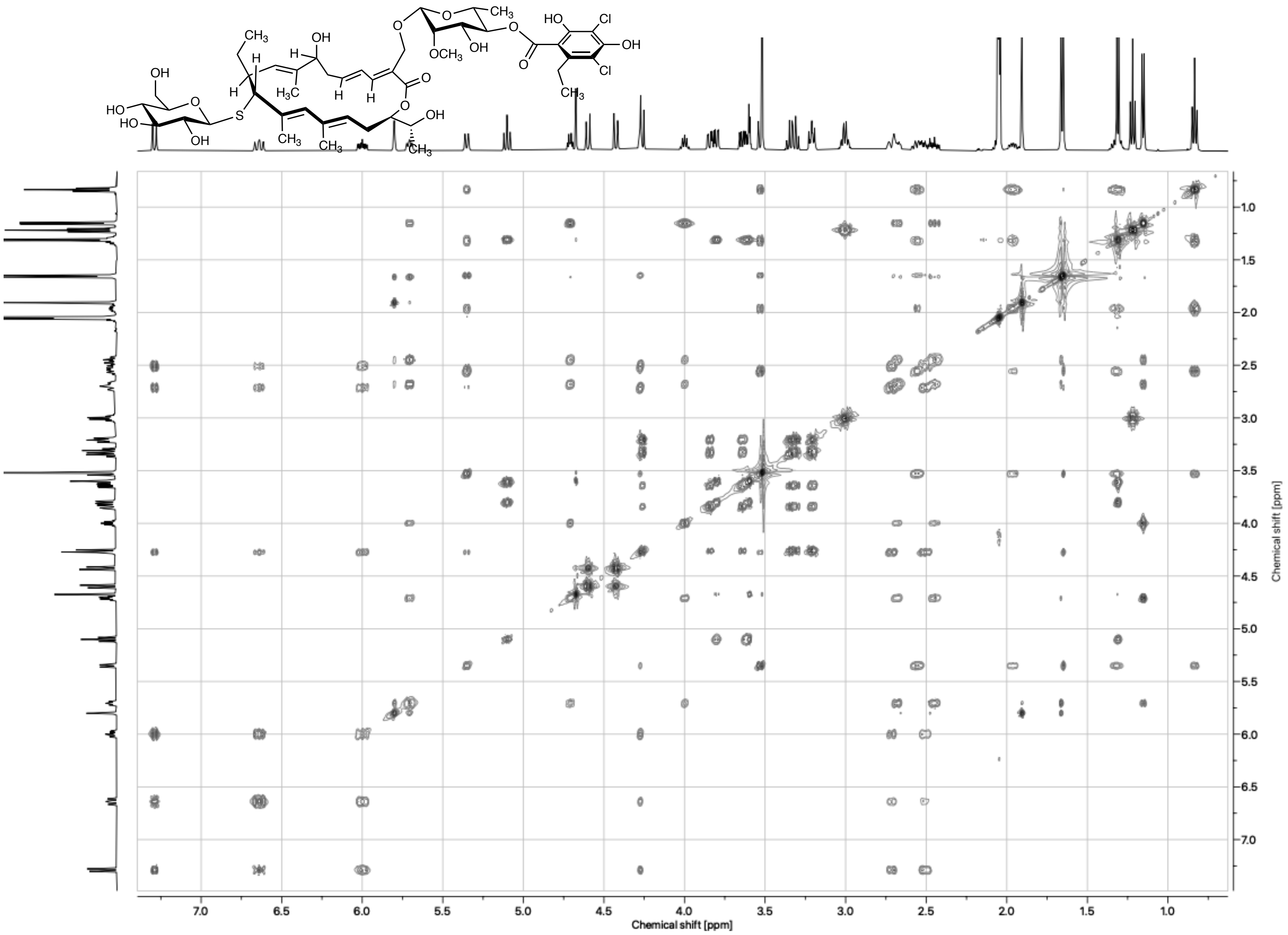

Figure 70: TOCSY spectrum of 11-desnoviosyl-11-thio-β-D-glucosyl fidaxomicin (18a-C(11)) in acetone-d_6

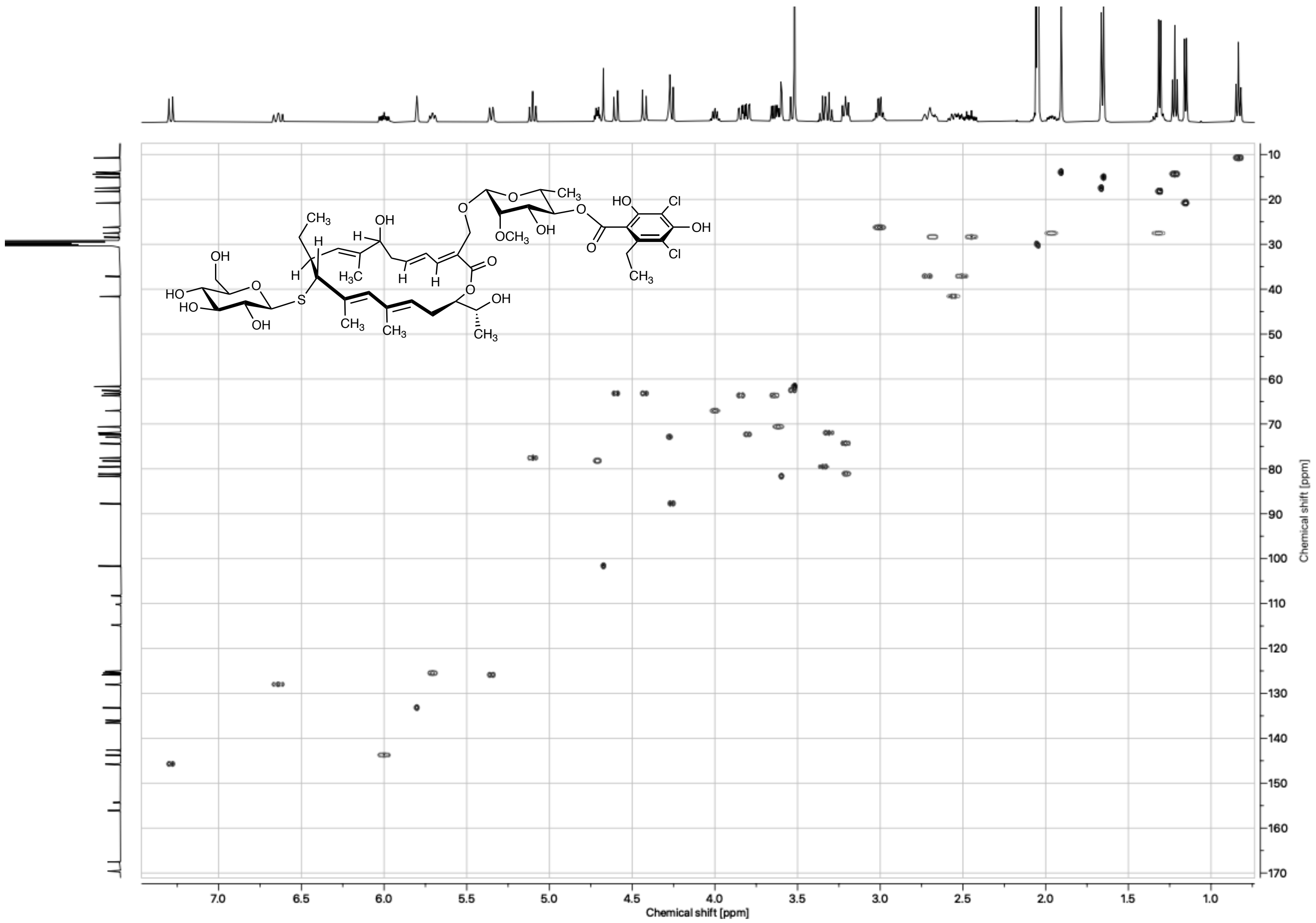

Figure 71: HSQC spectrum of 11-desnoviosyl-11-thio-β-D-glucosyl fidaxomicin (18a-C(11)) in acetone-d_6

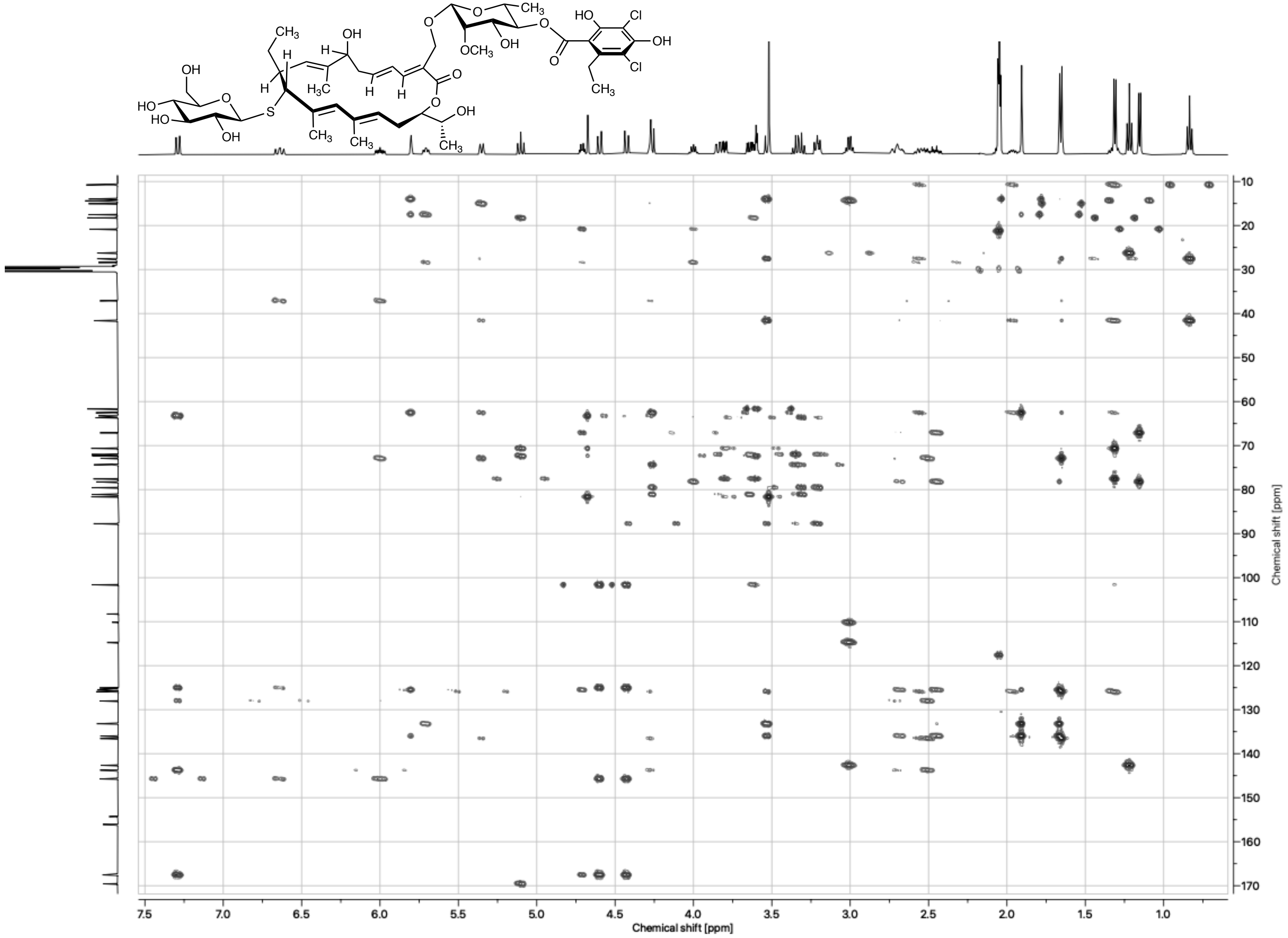

Figure 72: HMBC spectrum of 11-desnoviosyl-11-thio-β-D-glucosyl fidaxomicin (18a-C(11)) in acetone-d_6

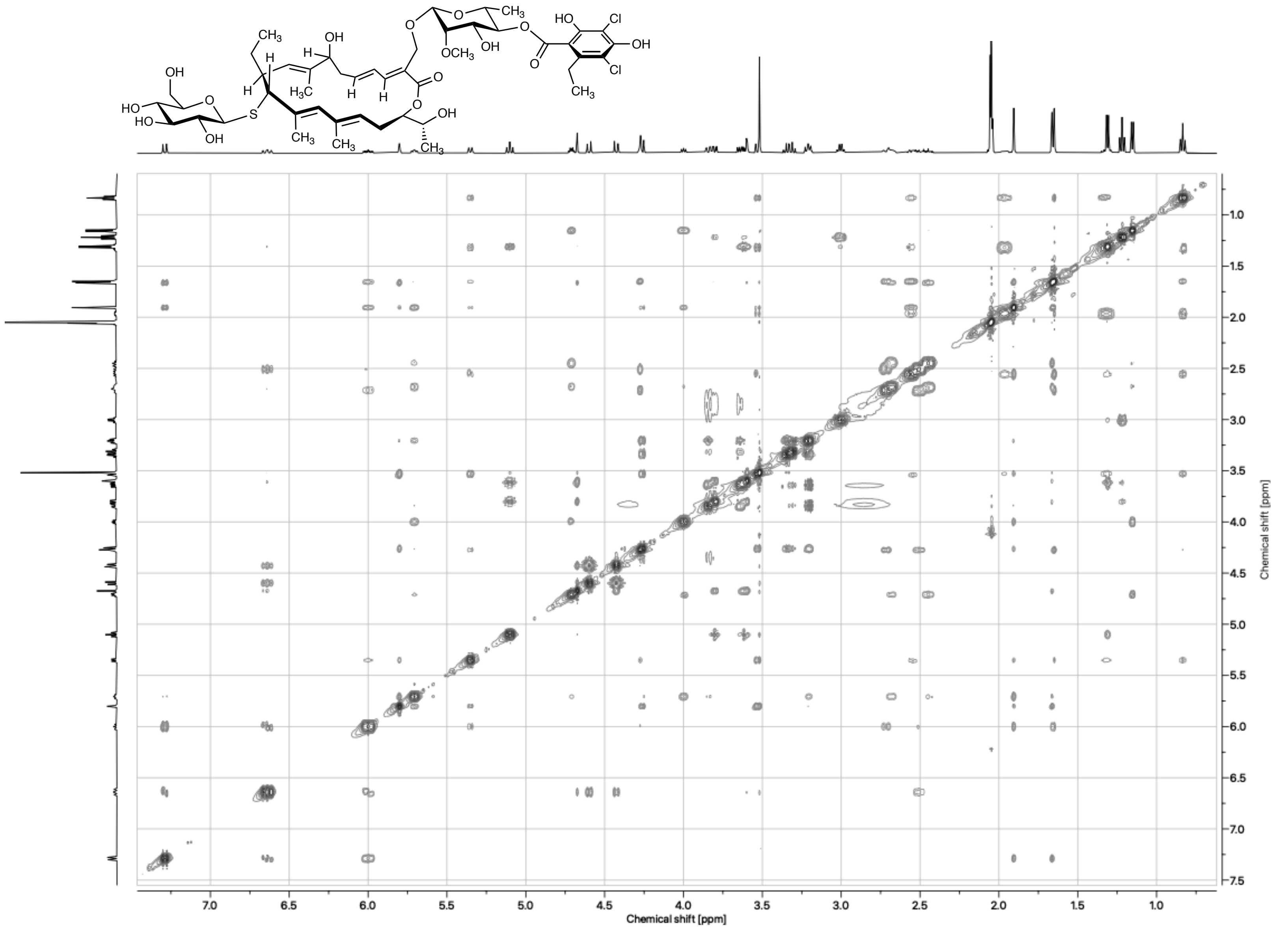

Figure 73: NOESY spectrum of 11-desnoviosyl-11-thio-β-D-glucosyl fidaxomicin (18a-C(11)) in acetone-d_6

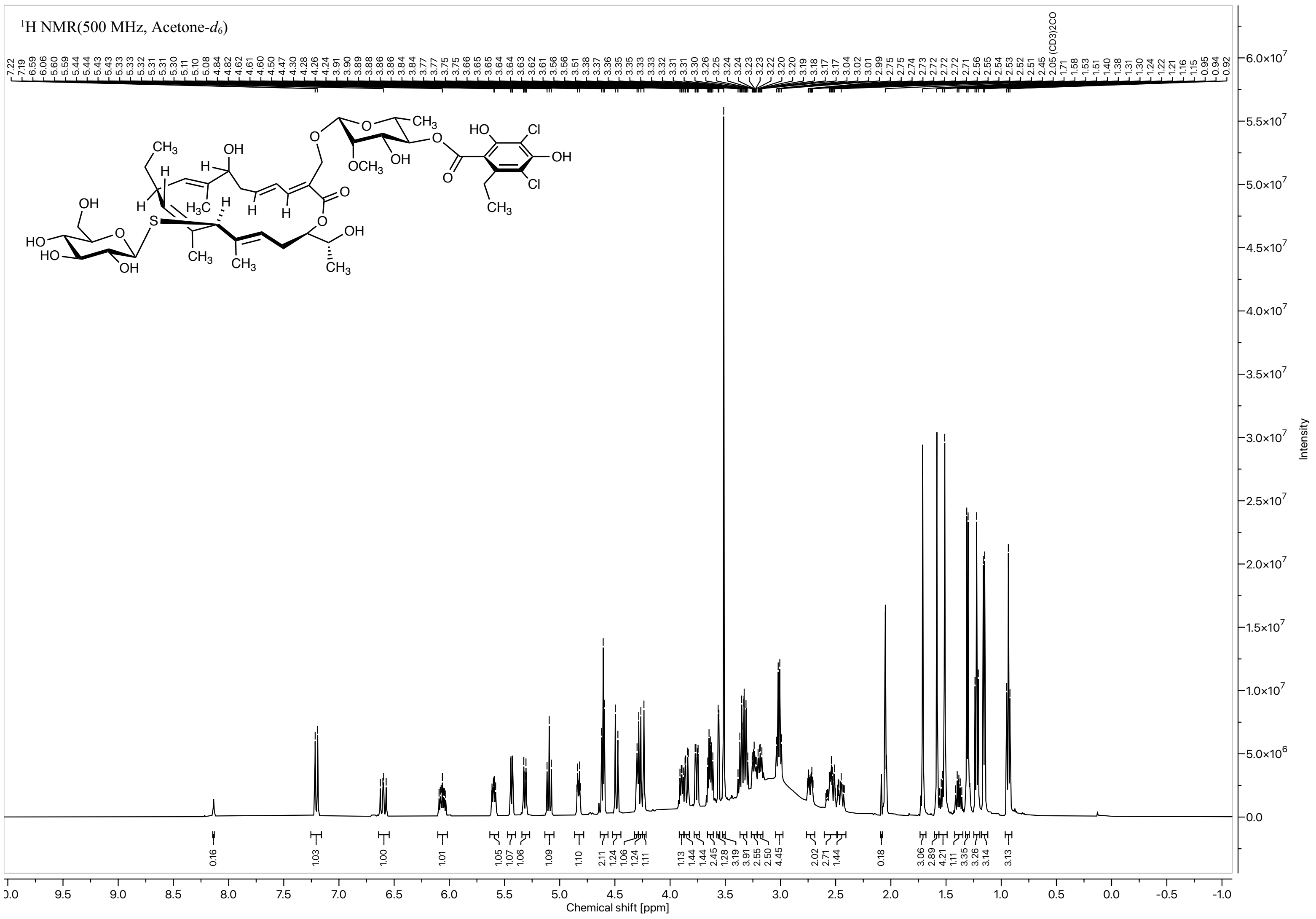

Figure 74: ^{1}H NMR spectrum of 11-desnoviosyl-13-thio-β-D-glucosyl fidaxomicin (18a-C(13)) in acetone-d_6

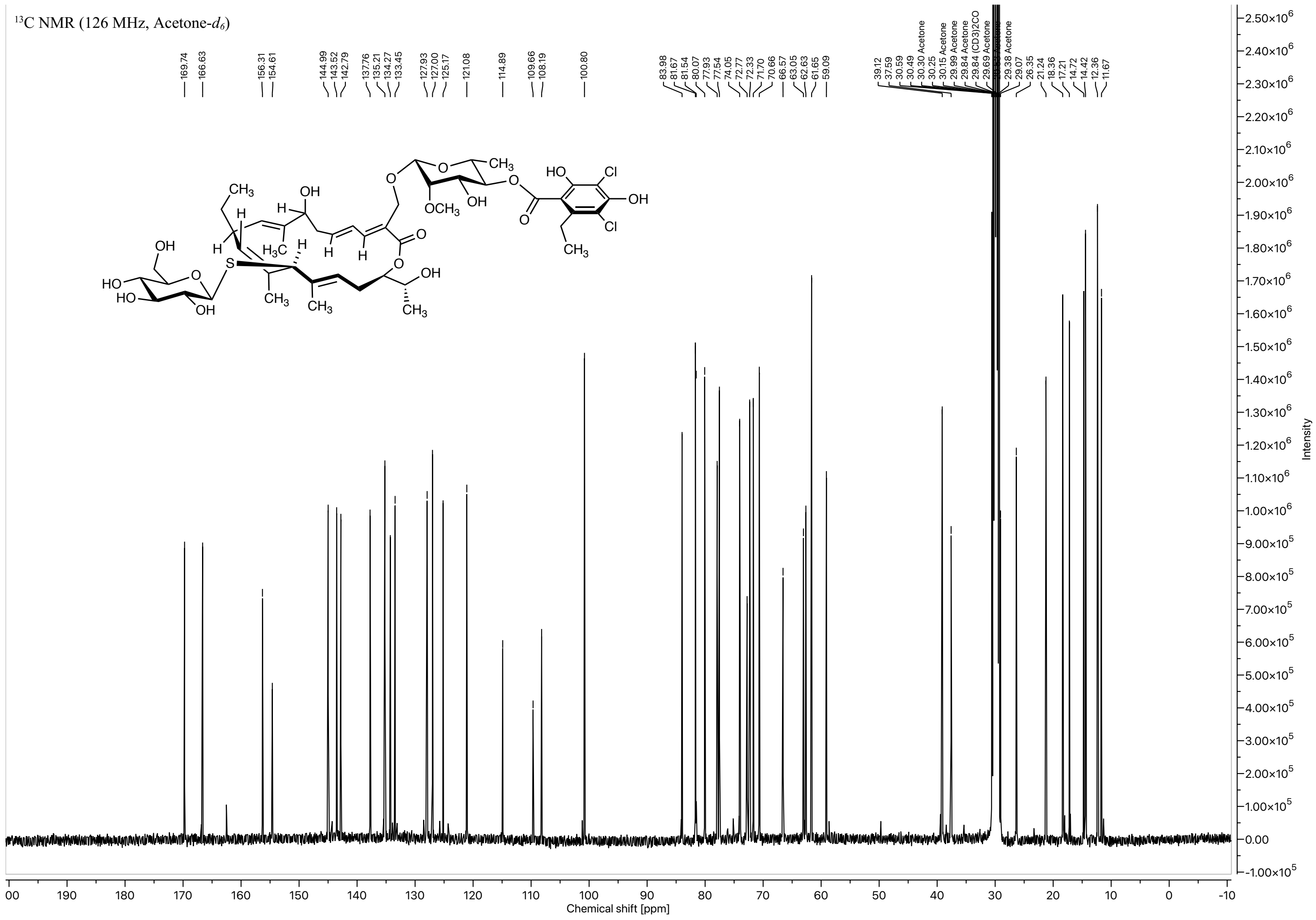

Figure 75: ^{13}C NMR spectrum of 11-desnoviosyl-13-thio-β-D-glucosyl fidaxomicin (18a-C(13)) in acetone-d_6

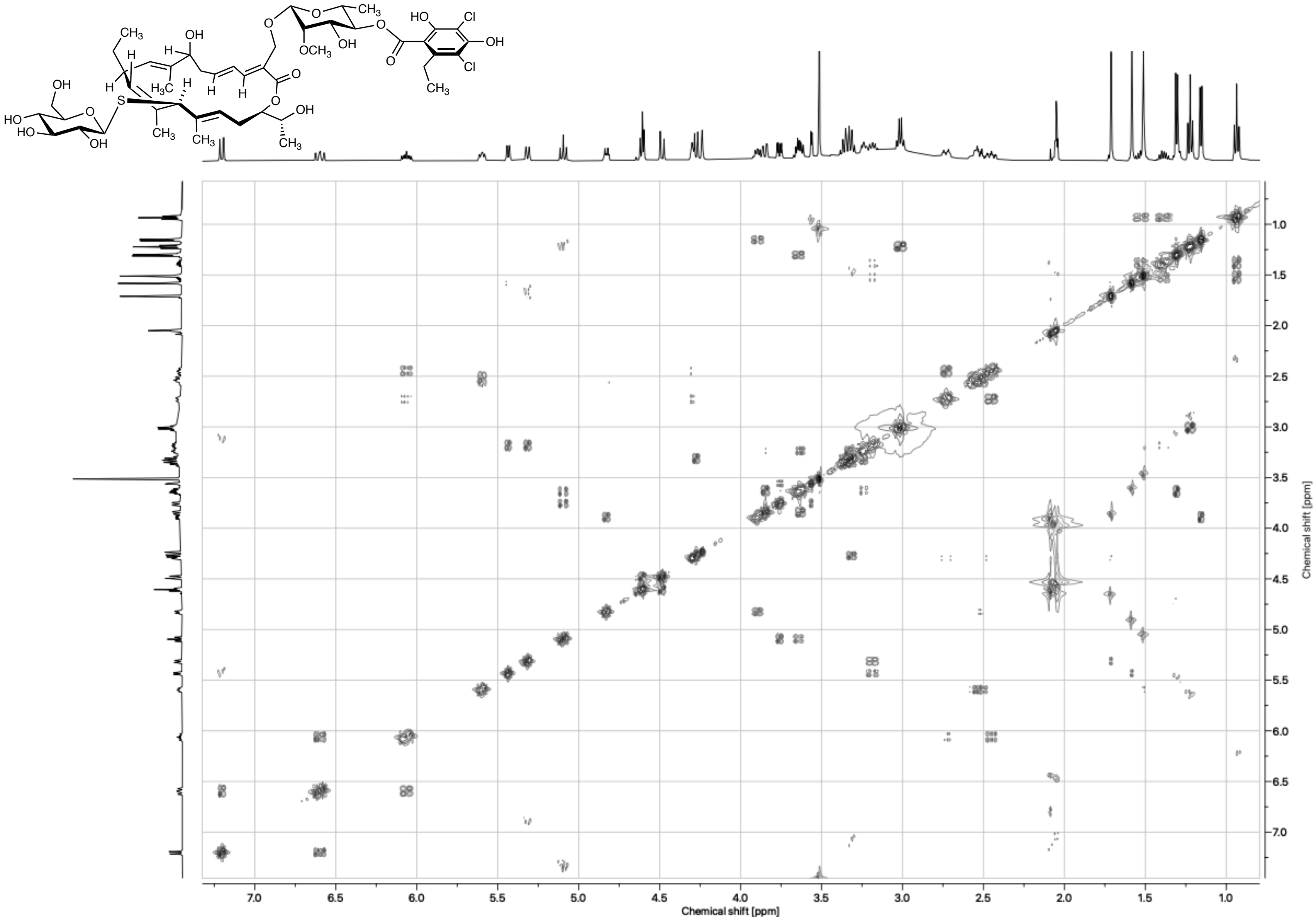

Figure 76: COSY spectrum of 11-desnoviosyl-13-thio-β-D-glucosyl fidaxomicin (18a-C(13)) in acetone-d_6

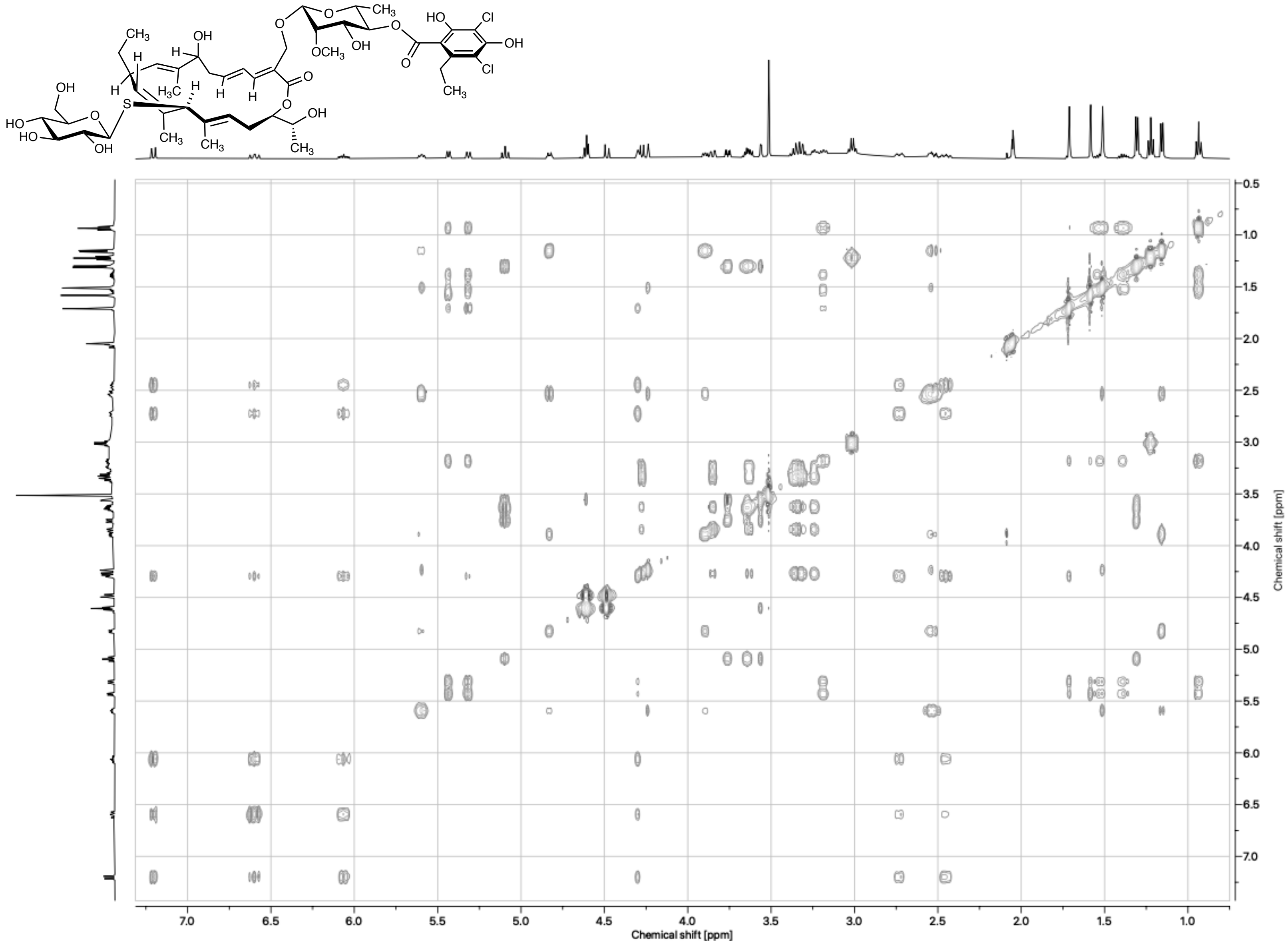

Figure 77: TOCSY spectrum of 11-desnoviosyl-13-thio-β-D-glucosyl fidaxomicin (18a-C(13)) in acetone-d_6

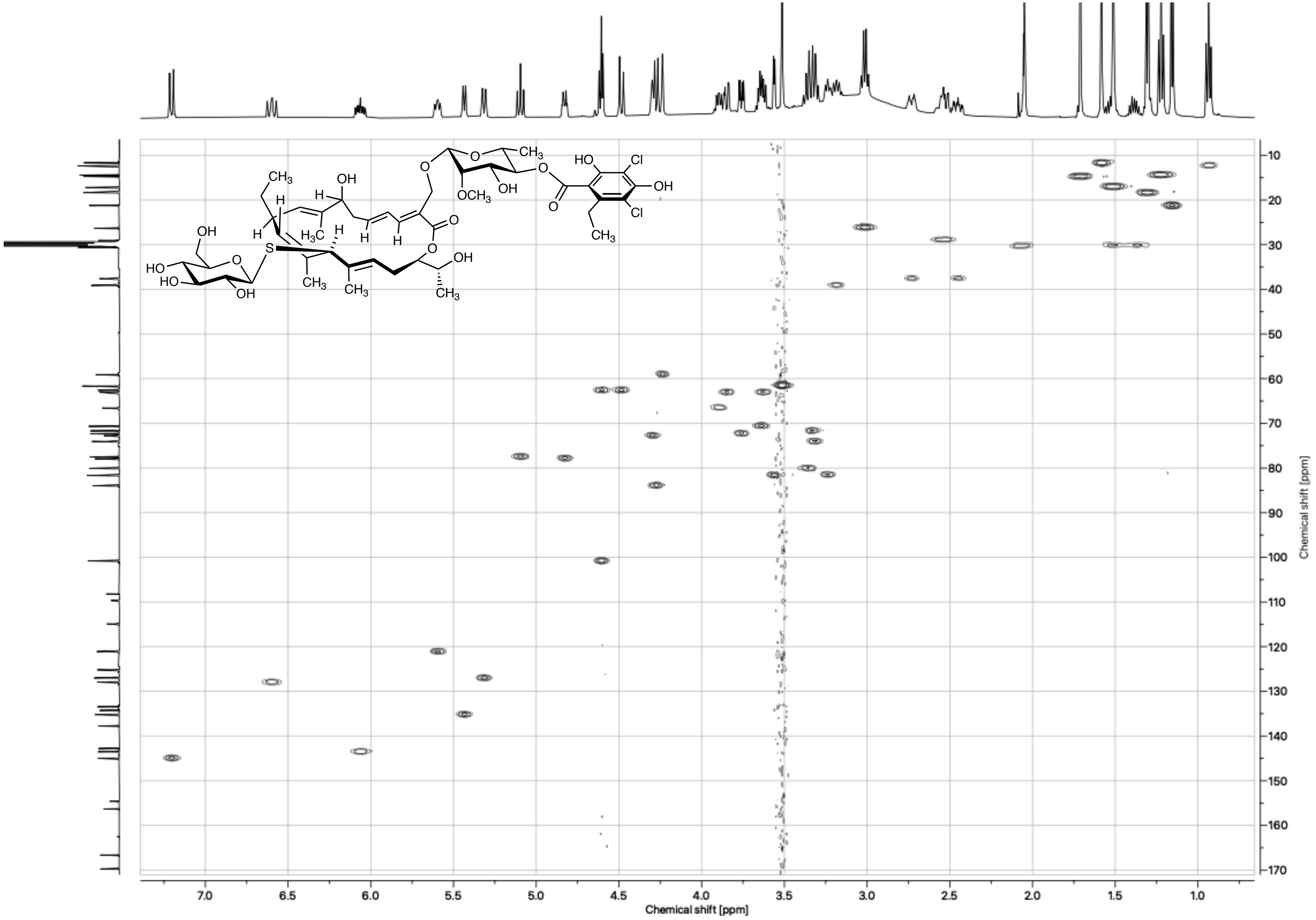

Figure 78: HSQC spectrum of 11-desnoviosyl-13-thio-β-D-glucosyl fidaxomicin (18a-C(13)) in acetone-d_6

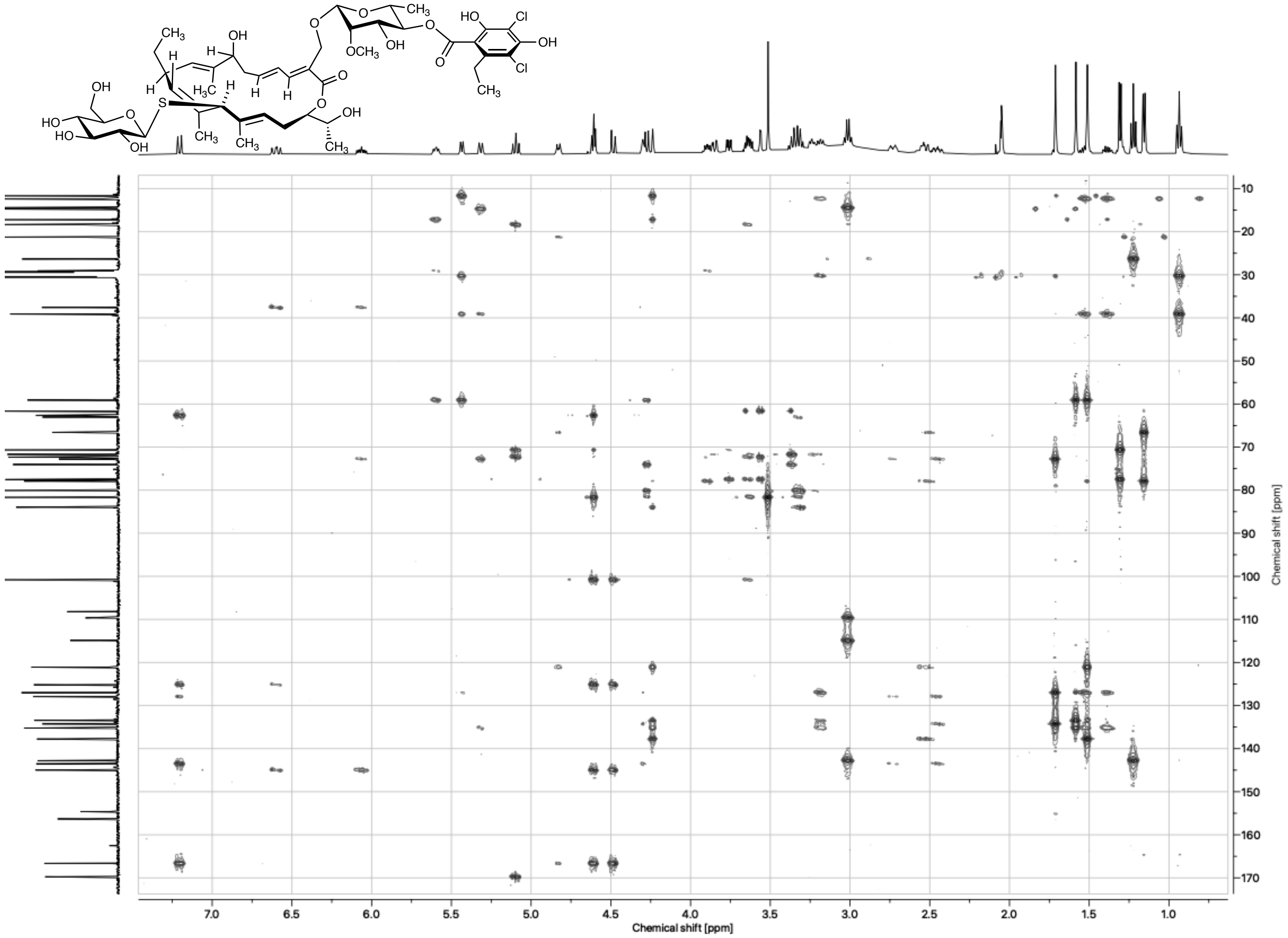

Figure 79: HMBC spectrum of 11-desnoviosyl-13-thio-β-D-glucosyl fidaxomicin (18a-C(13)) in acetone-d_6

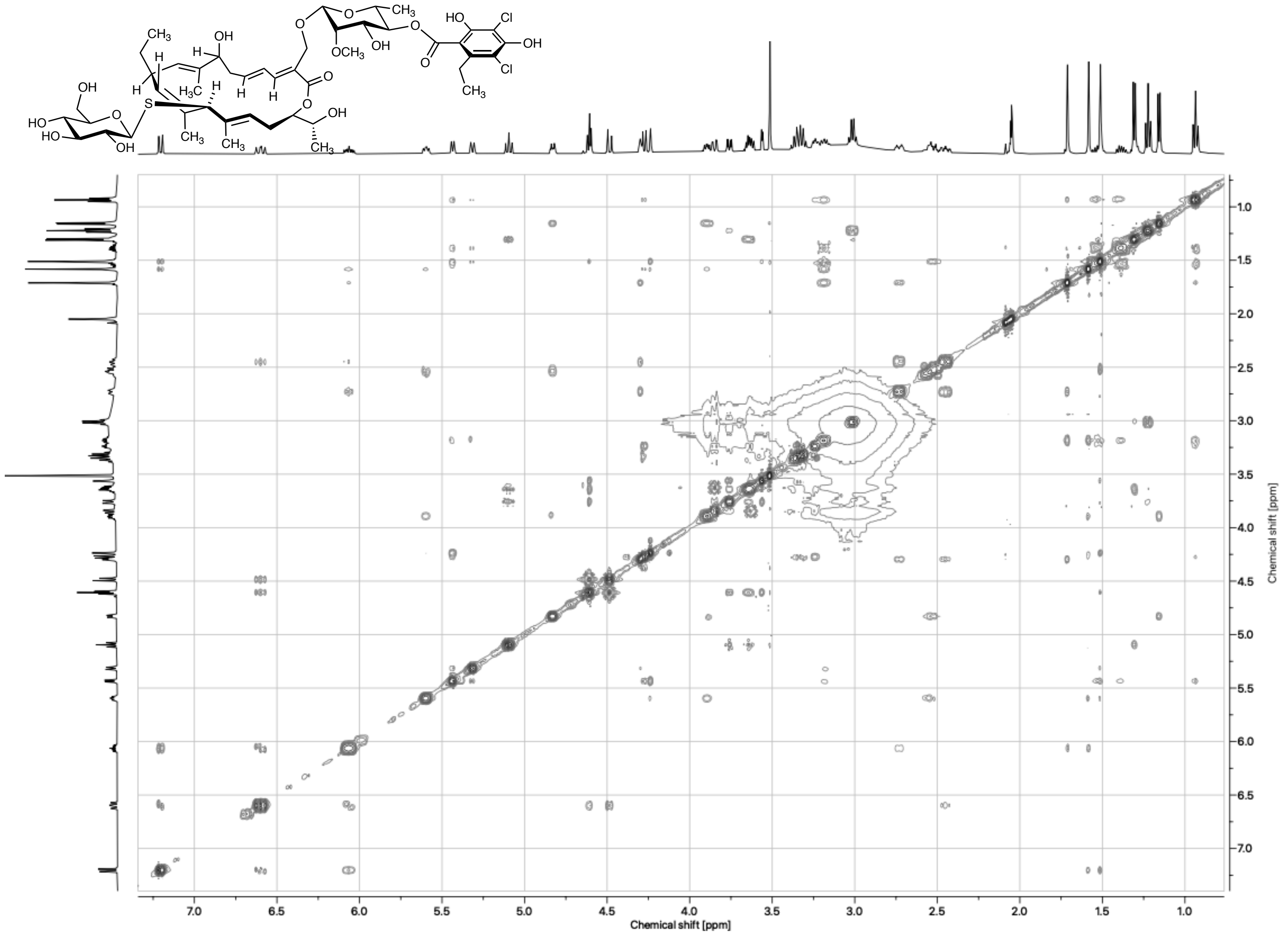

Figure 80: NOESY spectrum of 11-desnoviosyl-13-thio-β-D-glucosyl fidaxomicin (18a-C(13)) in acetone-d_6

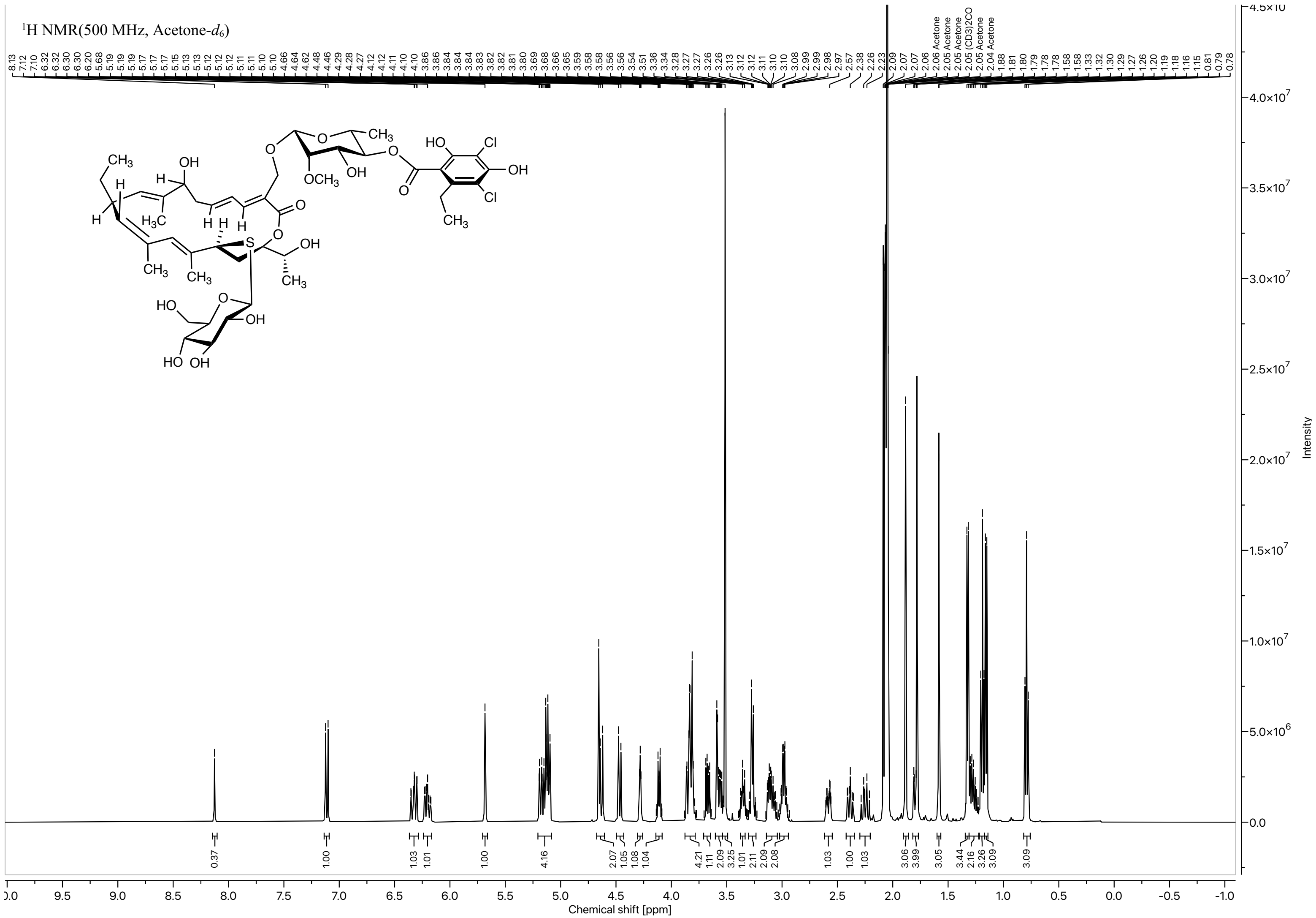

Figure 81: ^{1}H NMR spectrum of 11-desnoviosyl-15-thio-β-D-glucosyl fidaxomicin (18a-C(15)) in acetone-d_6

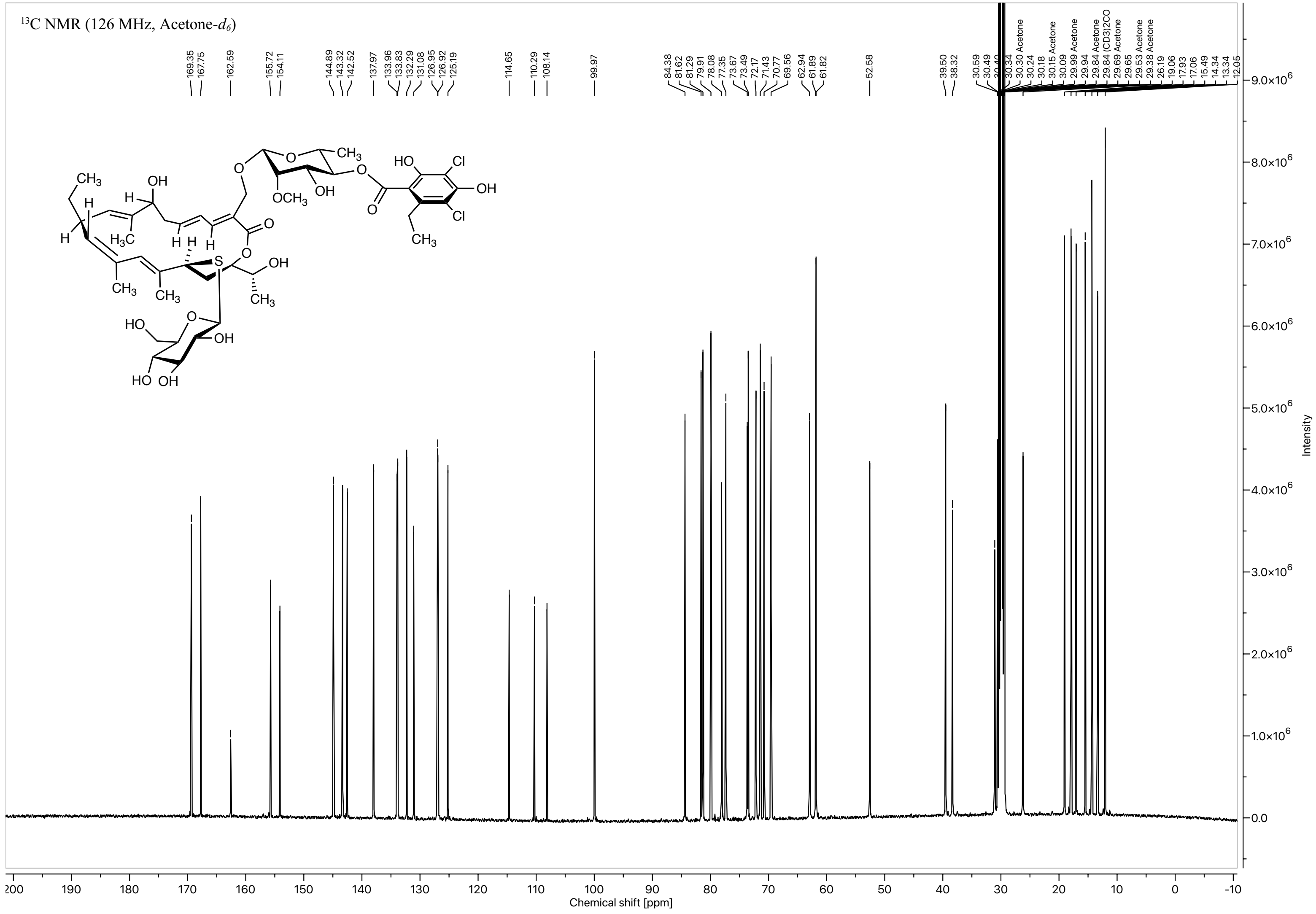

Figure 82: ^{13}C NMR spectrum of 11-desnoviosyl-15-thio-β-D-glucosyl fidaxomicin (18a-C(15)) in acetone-d_6

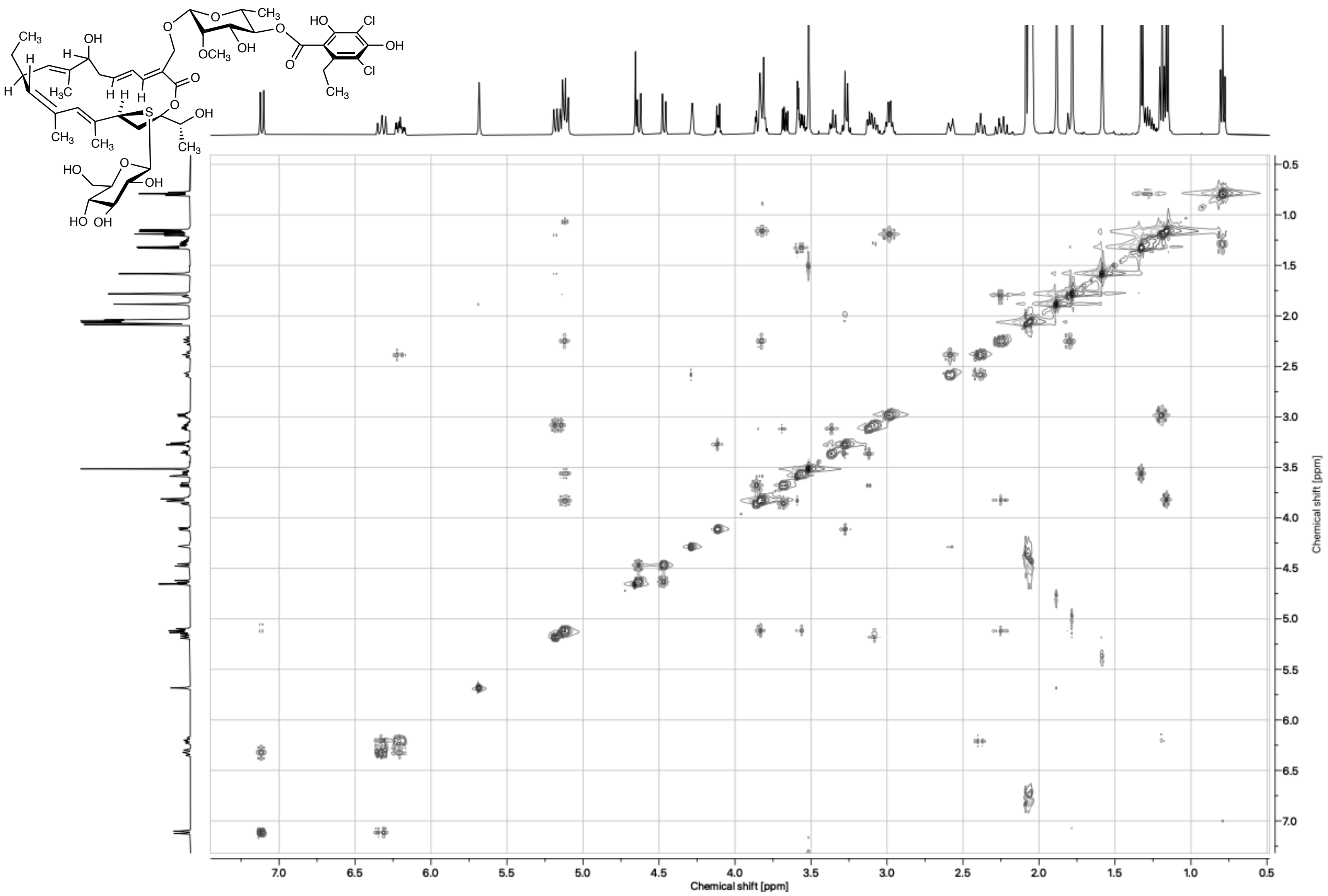

Figure 83: COSY spectrum of 11-desnoviosyl-15-thio-β-D-glucosyl fidaxomicin (18a-C(15)) in acetone-d_6

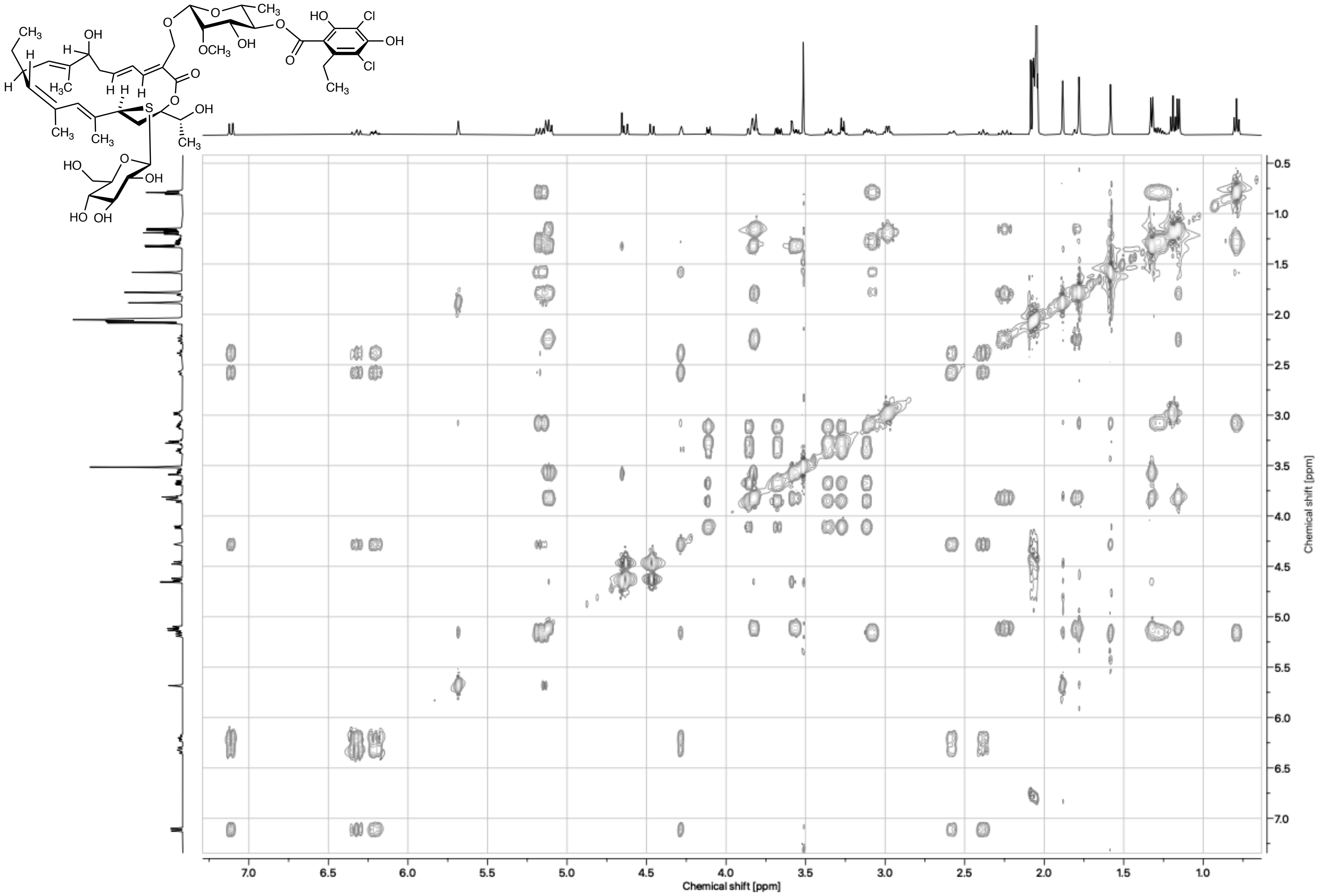

Figure 84: TOCSY spectrum of 11-desnoviosyl-15-thio-β-D-glucosyl fidaxomicin (18a-C(15)) in acetone-d_6

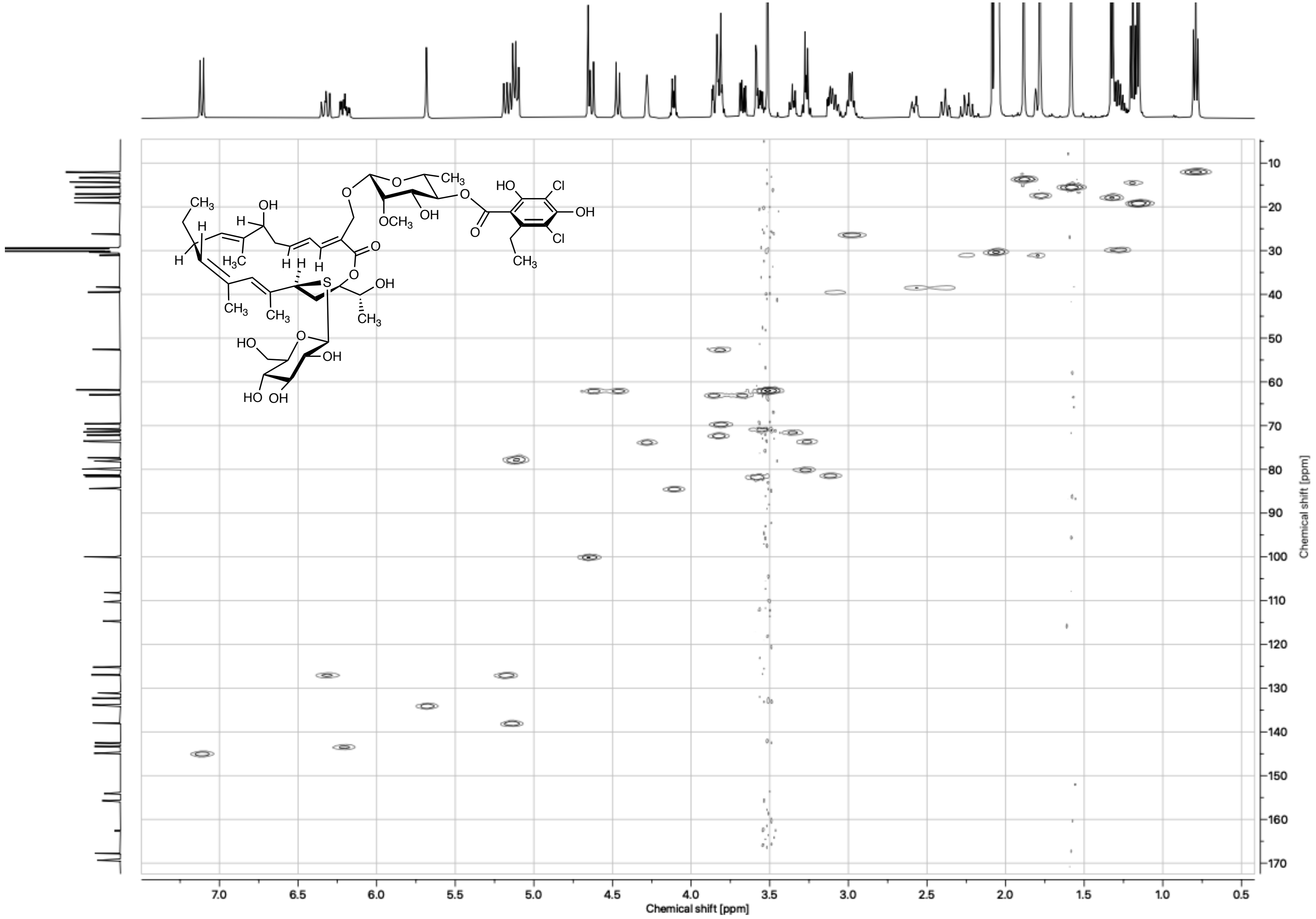

Figure 85: HSQC spectrum of 11-desnoviosyl-15-thio-β-D-glucosyl fidaxomicin (18a-C(15)) in acetone-d_6

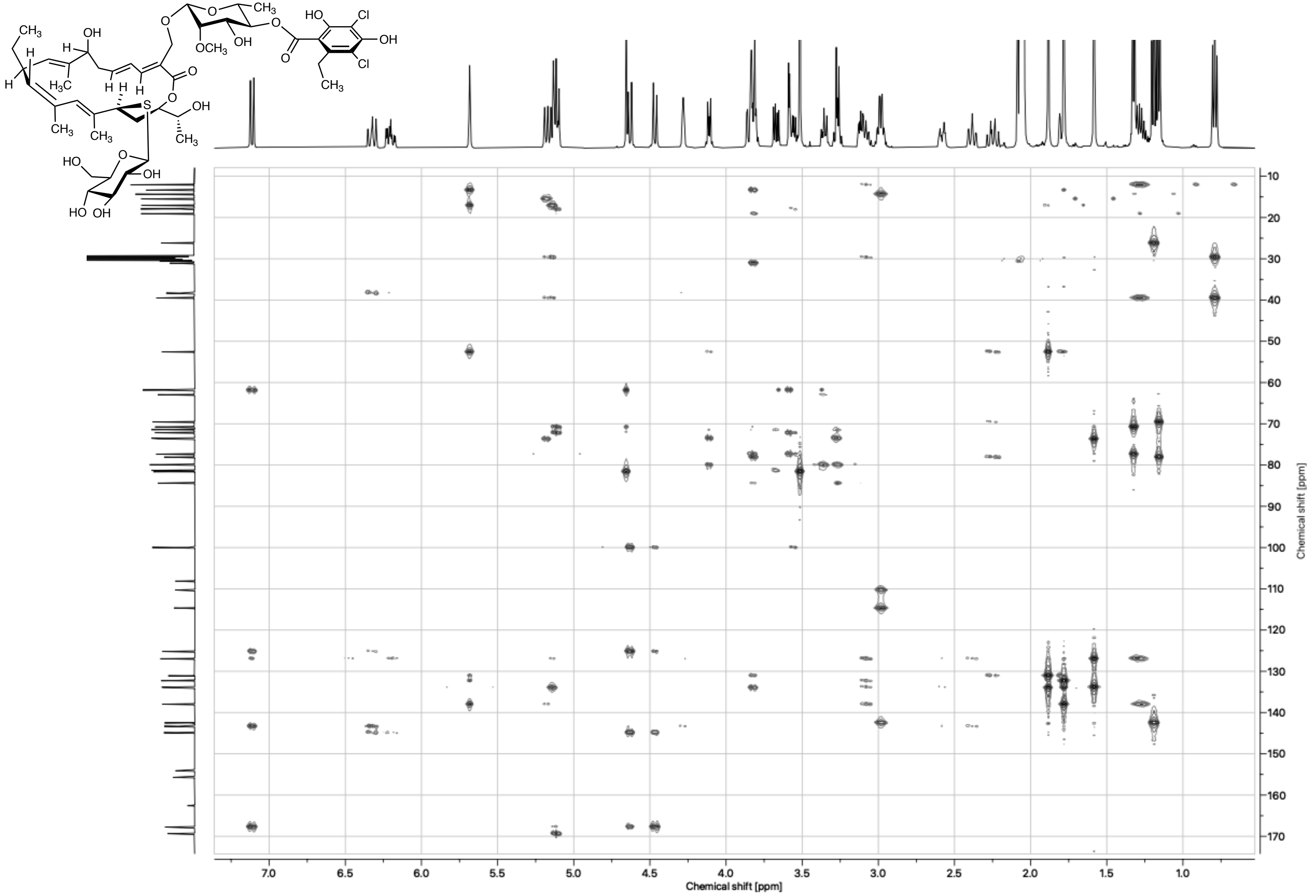

Figure 86: HMBC spectrum of 11-desnoviosyl-15-thio-β-D-glucosyl fidaxomicin (18a-C(15)) in acetone-d_6

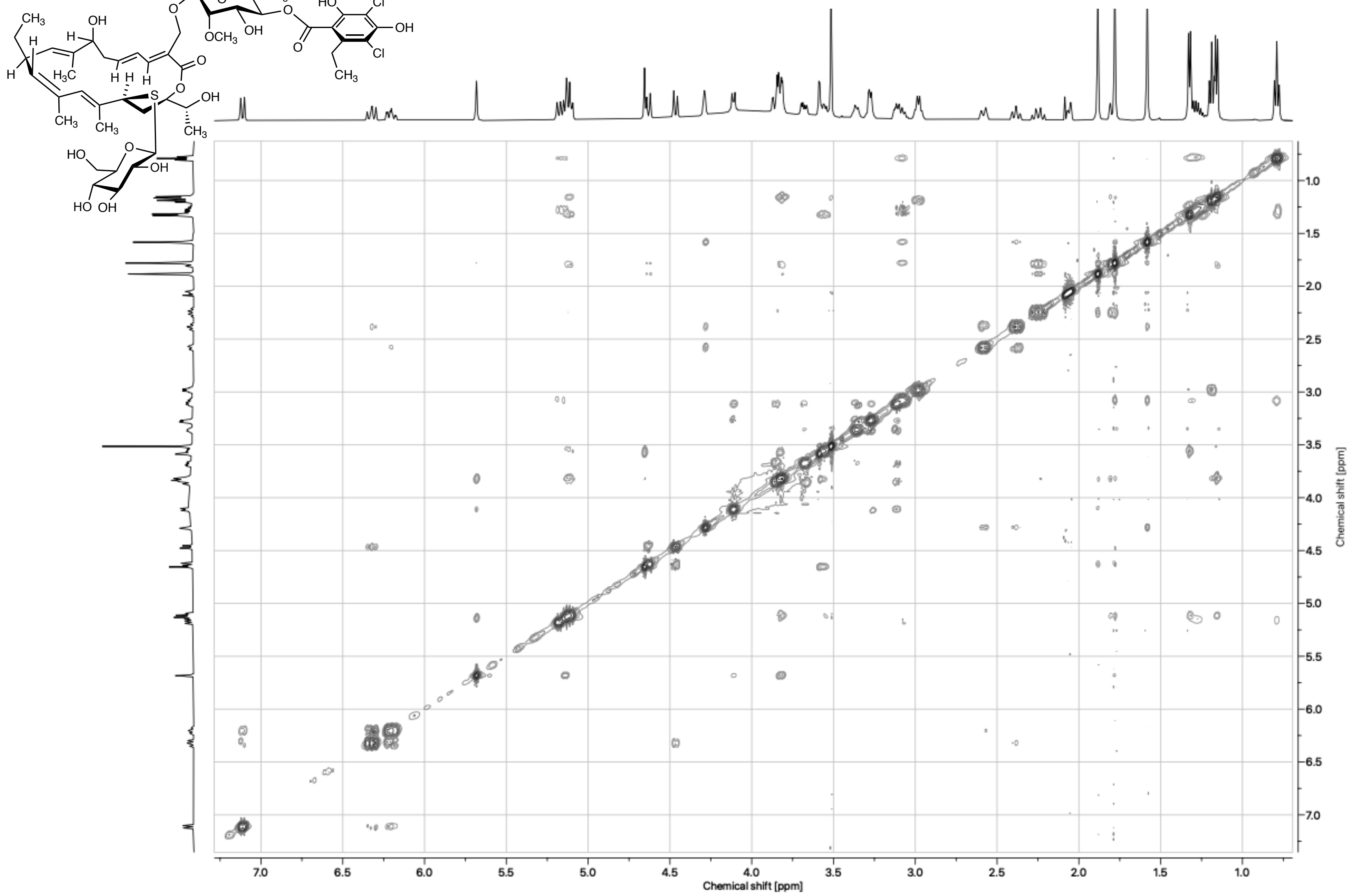

Figure 87: NOESY spectrum of 11-desnoviosyl-15-thio-β-D-glucosyl fidaxomicin (18a-C(15)) in acetone-d_6

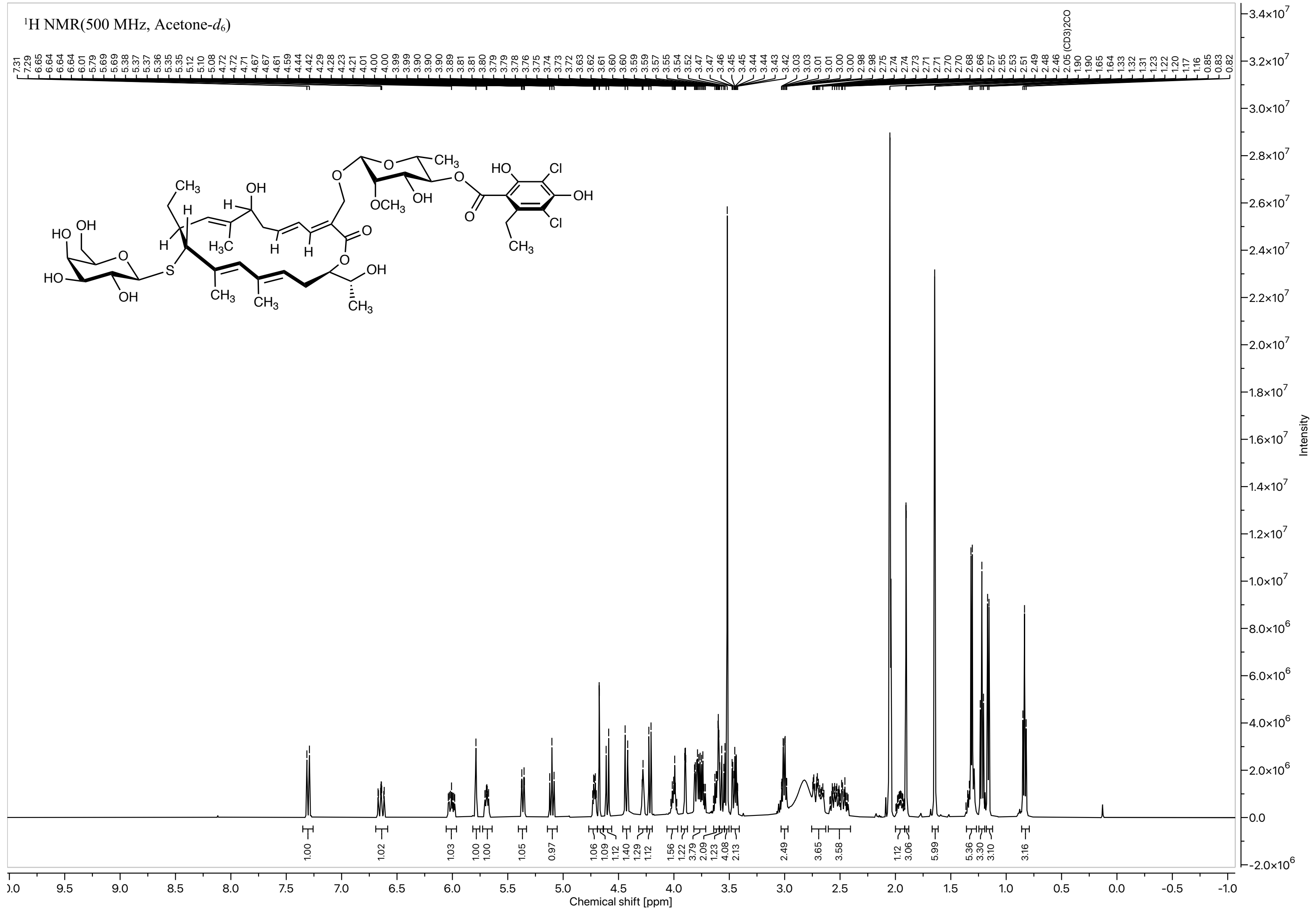

Figure 88: ^{1}H NMR spectrum of 11-desnoviosyl-11-thio-β-D-galactosyl fidaxomicin (18b-C(11)) in acetone-d_6

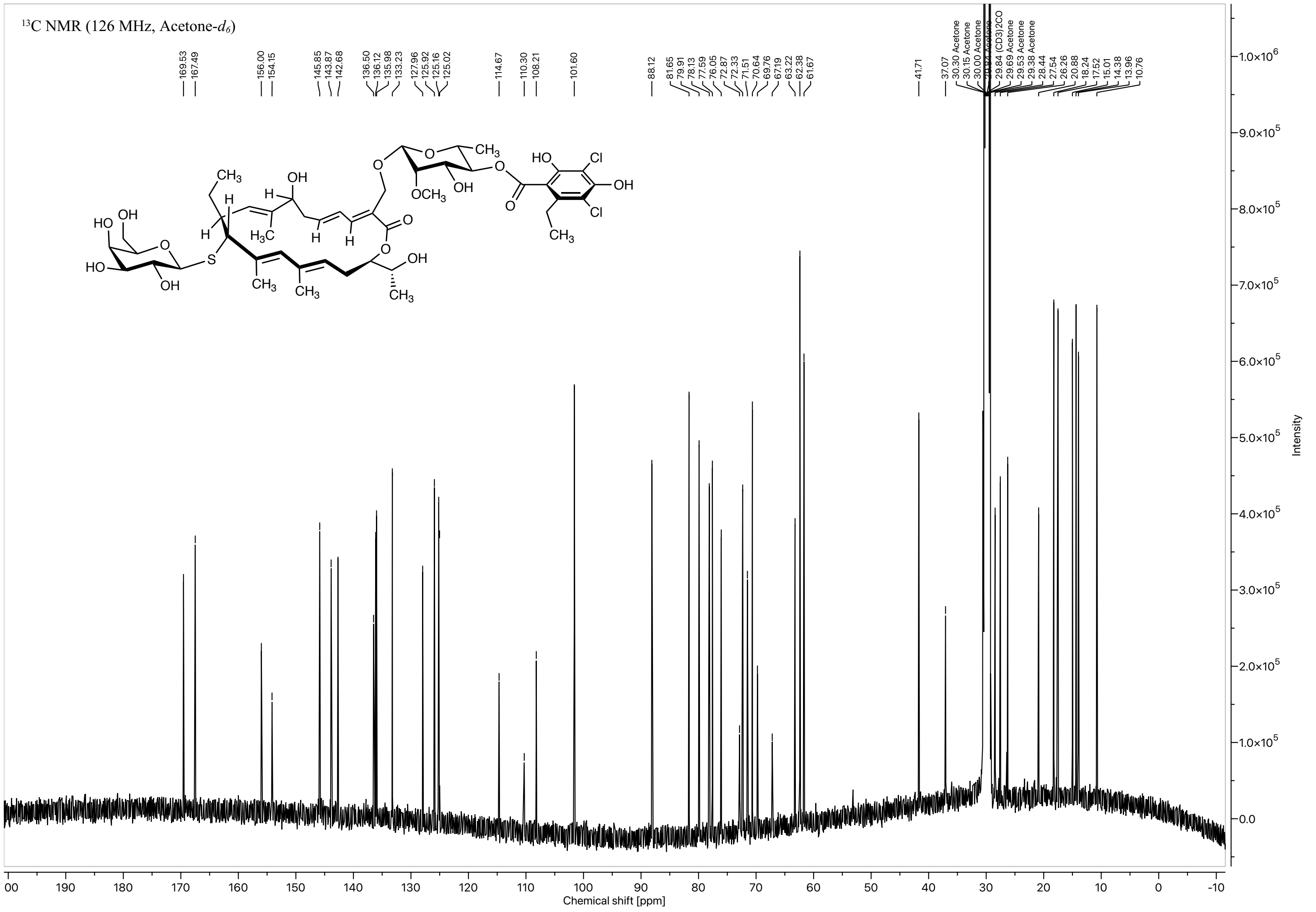

Figure 89: ^{13}C NMR spectrum of 11-desnoviosyl-11-thio-β-D-galactosyl fidaxomicin (18b-C(11)) in acetone-d$_6$

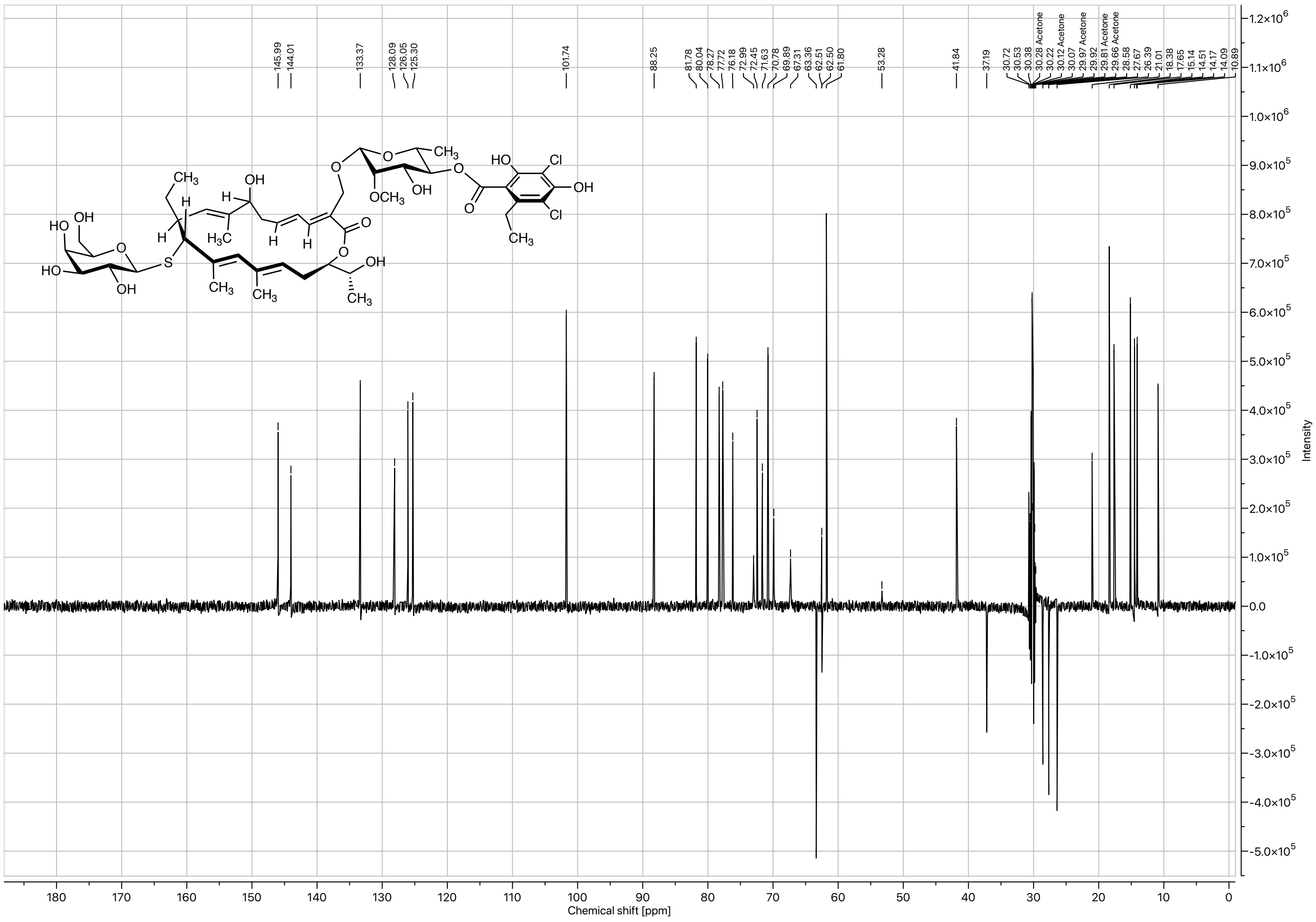

Figure 90: DEPT 135 spectrum of 11-desnoviosyl-11-thio-β-D-galactosyl fidaxomicin (18b-C(11)) in acetone-d_6

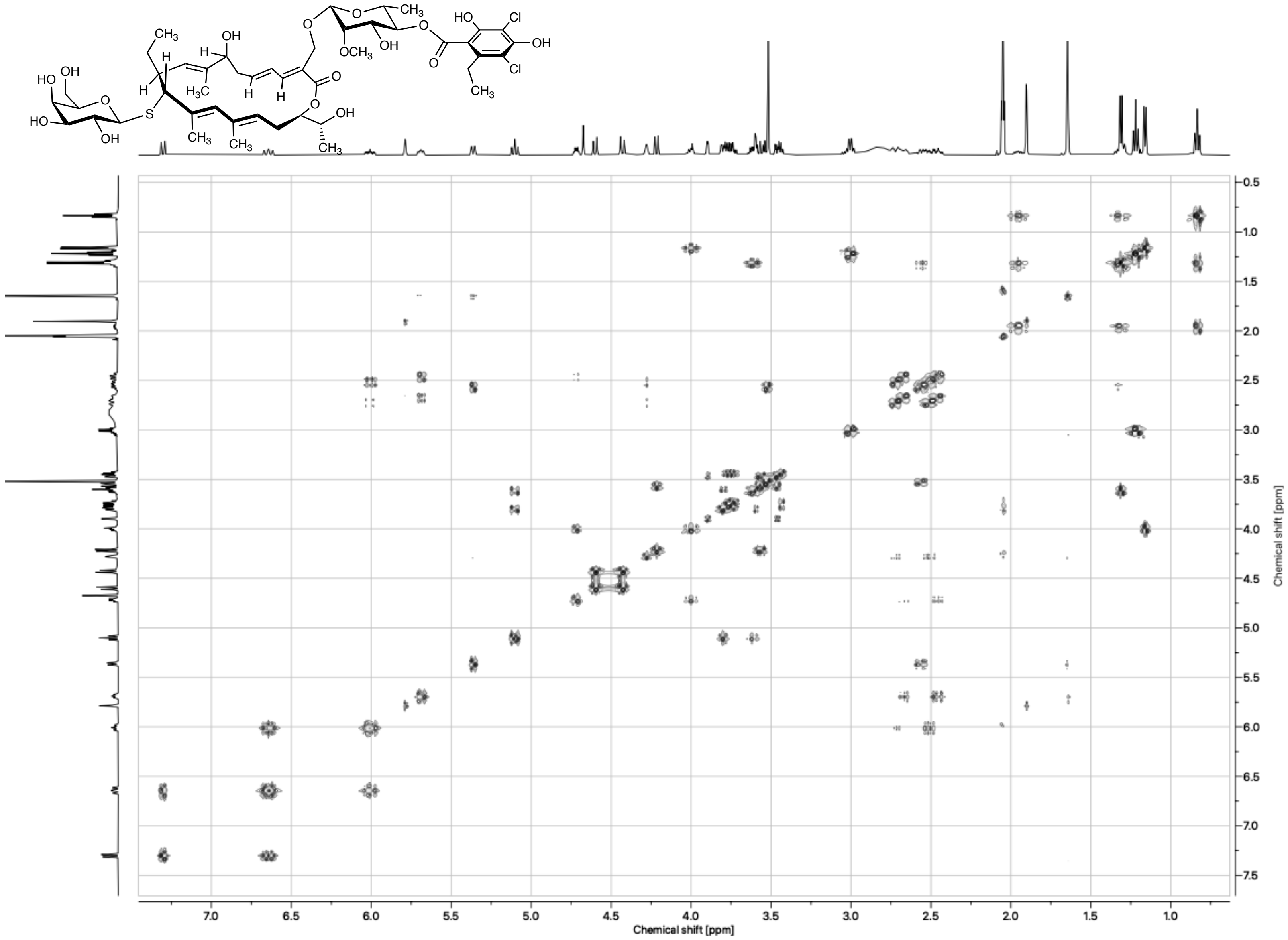

Figure 91: COSY spectrum of 11-desnoviosyl-11-thio-β-D-galactosyl fidaxomicin (18b-C(11)) in acetone-d_6

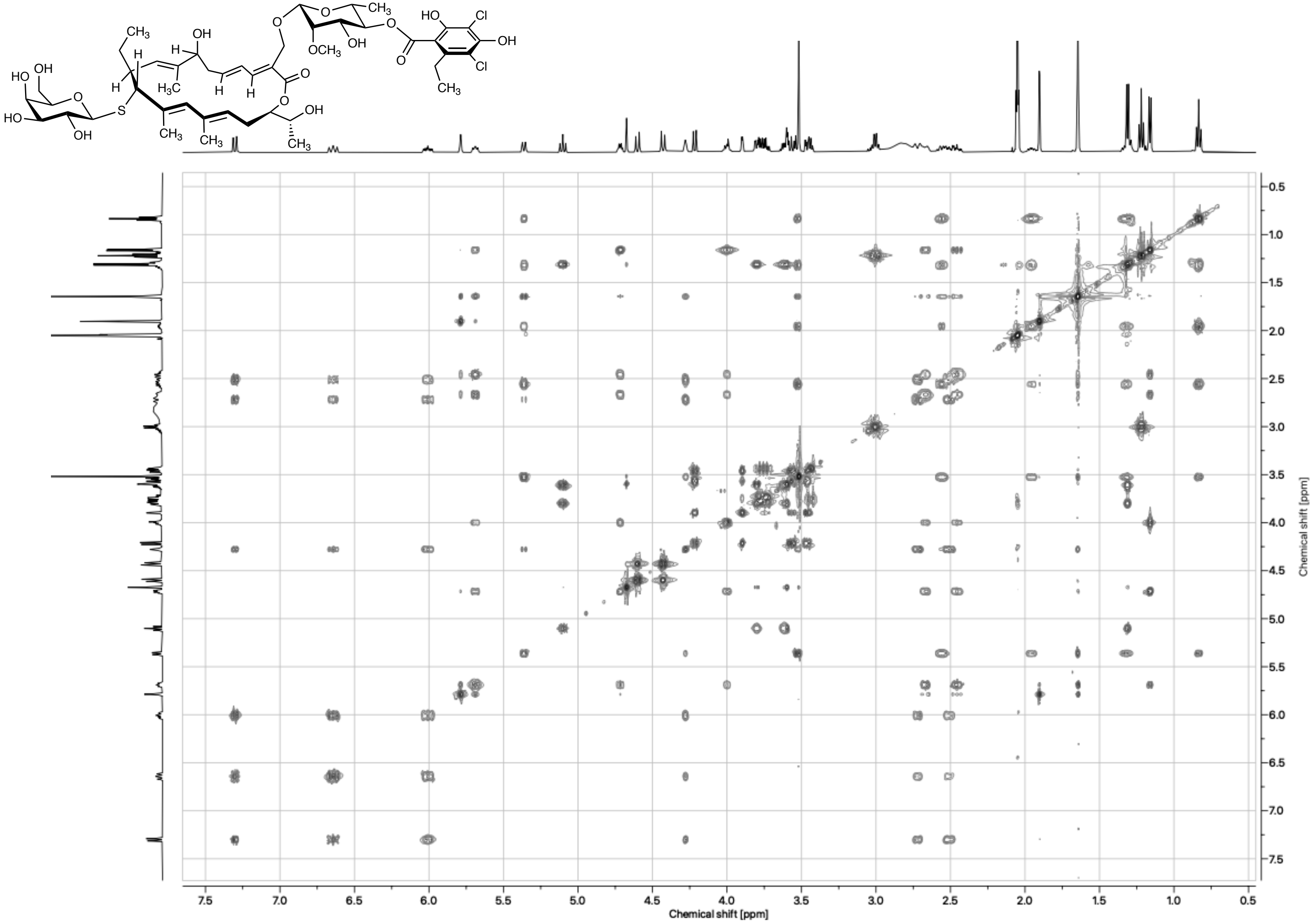

Figure 92: TOCSY spectrum of 11-desnoviosyl-11-thio-β-D-galactosyl fidaxomicin (18b-C(11)) in acetone-d_6

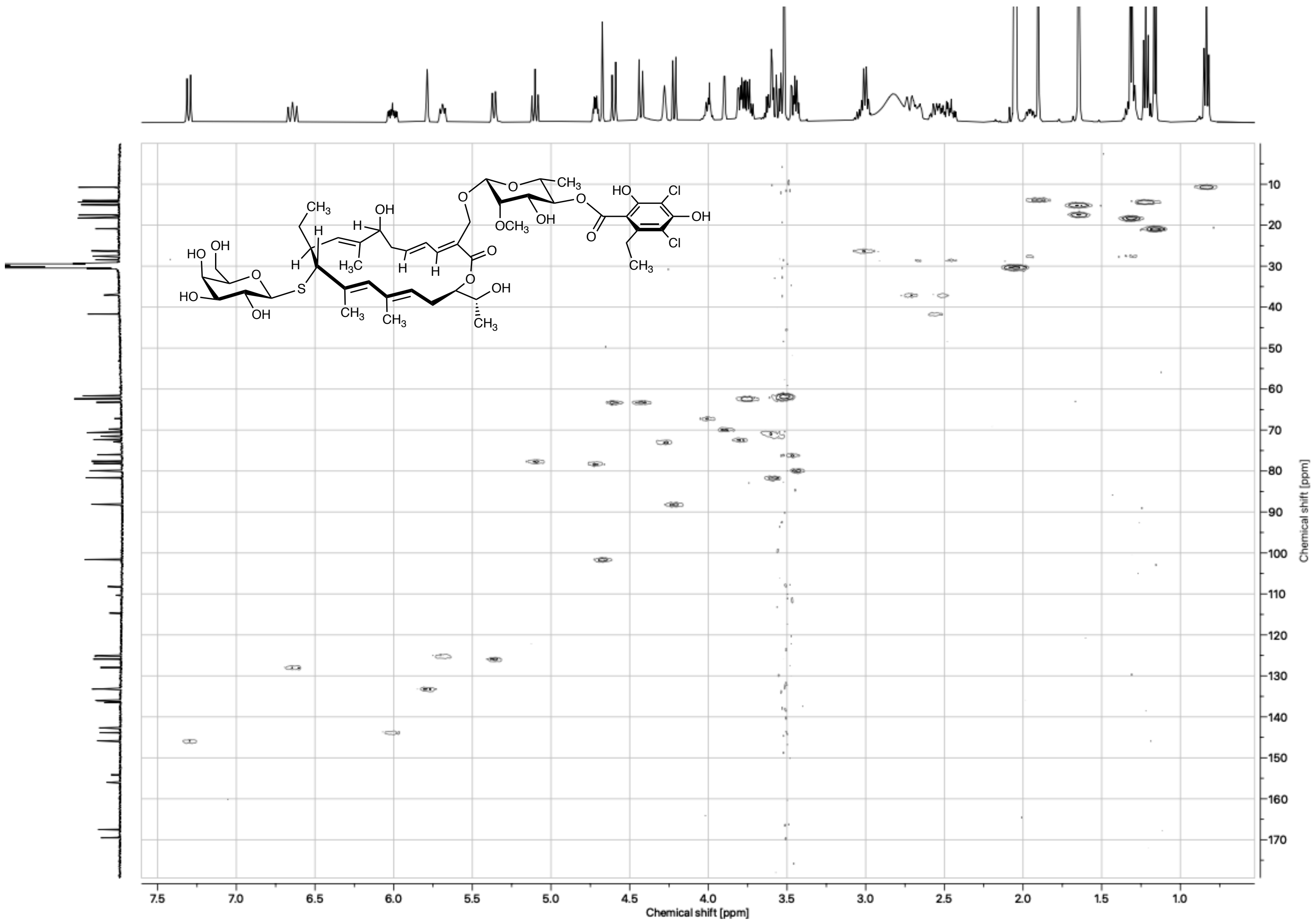

Figure 93: HSQC spectrum of 11-desnoviosyl-11-thio-β-D-galactosyl fidaxomicin (18b-C(11)) in acetone-d_6

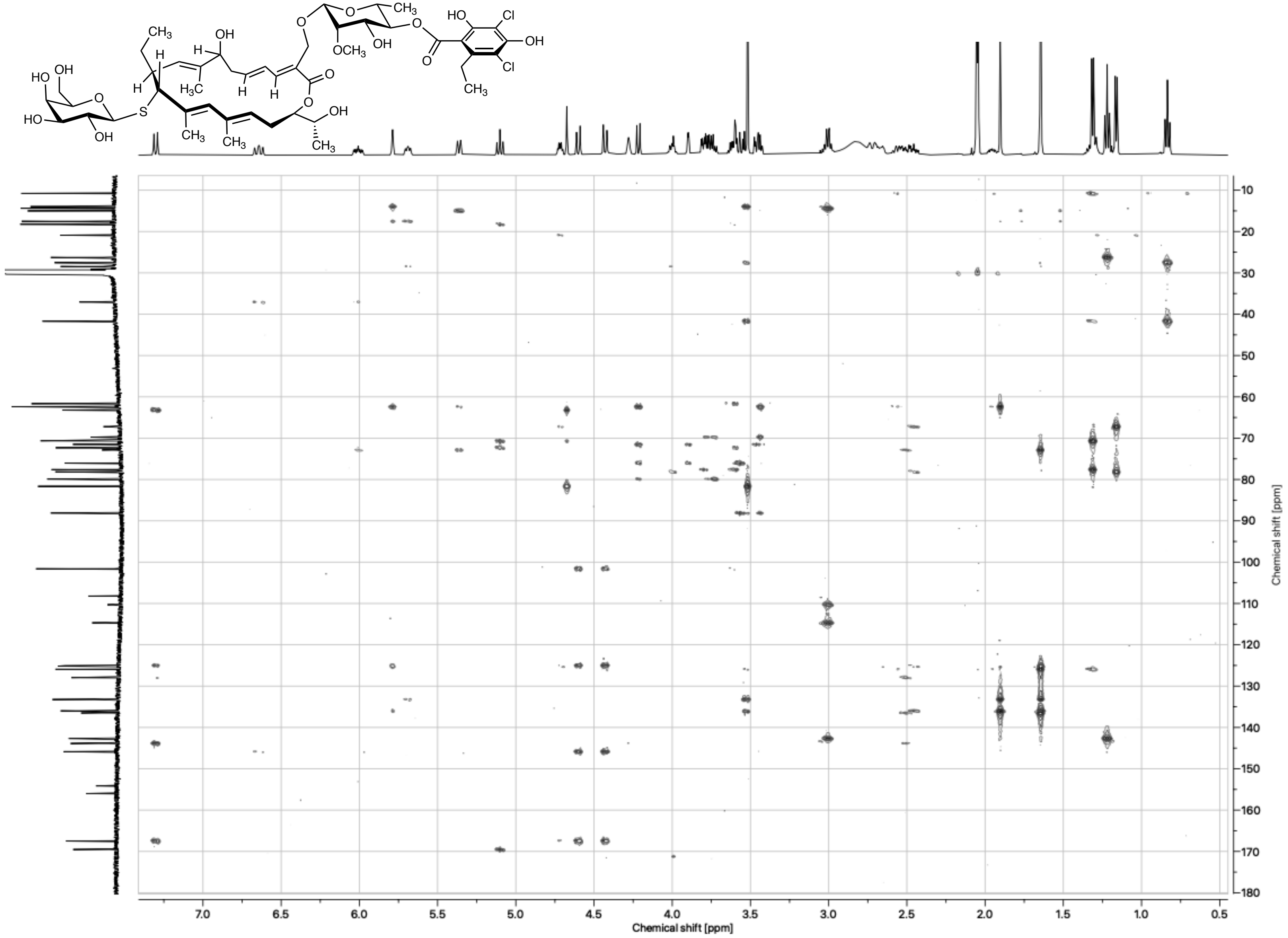

Figure 94: HMBC spectrum of 11-desnoviosyl-11-thio-β-D-galactosyl fidaxomicin (18b-C(11)) in acetone-d_6

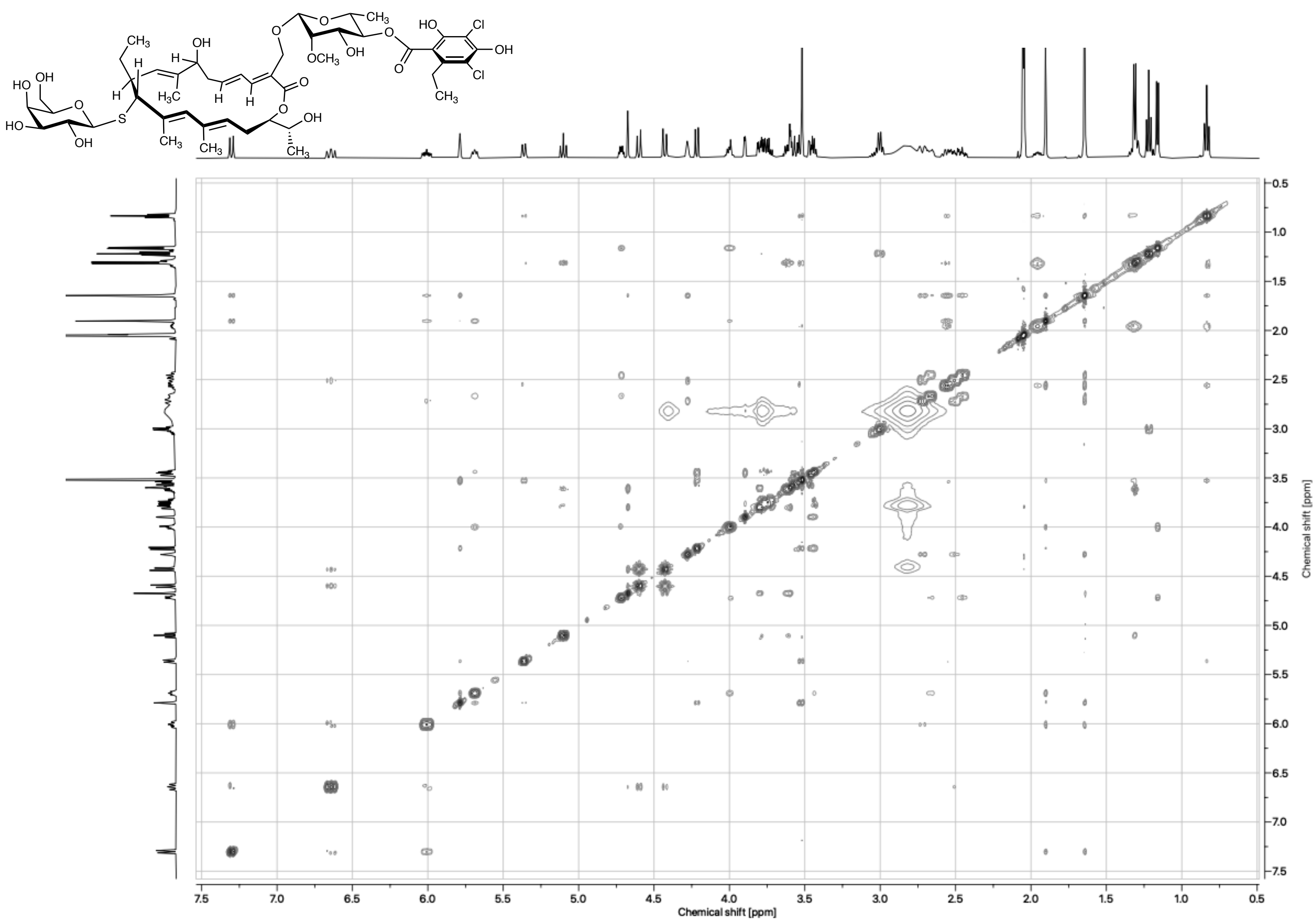

Figure 95: NOESY spectrum of 11-desnoviosyl-11-thio-β-D-galactosyl fidaxomicin (18b-C(11)) in acetone-d_6

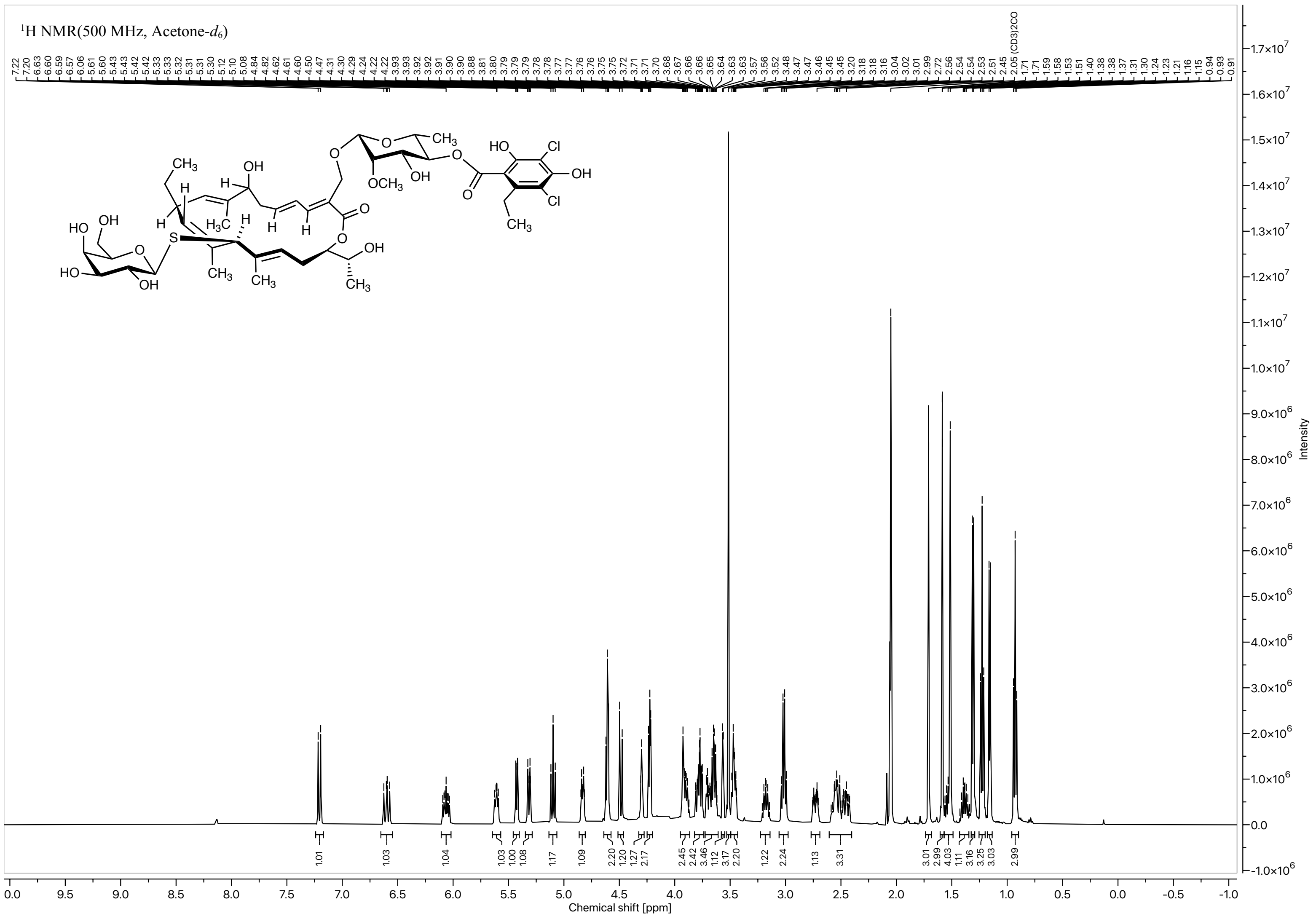

Figure 96: ^{1}H NMR spectrum of 11-desnoviosyl-13-thio-β-D-galactosyl fidaxomicin (18b-C(13)) in acetone-d_6

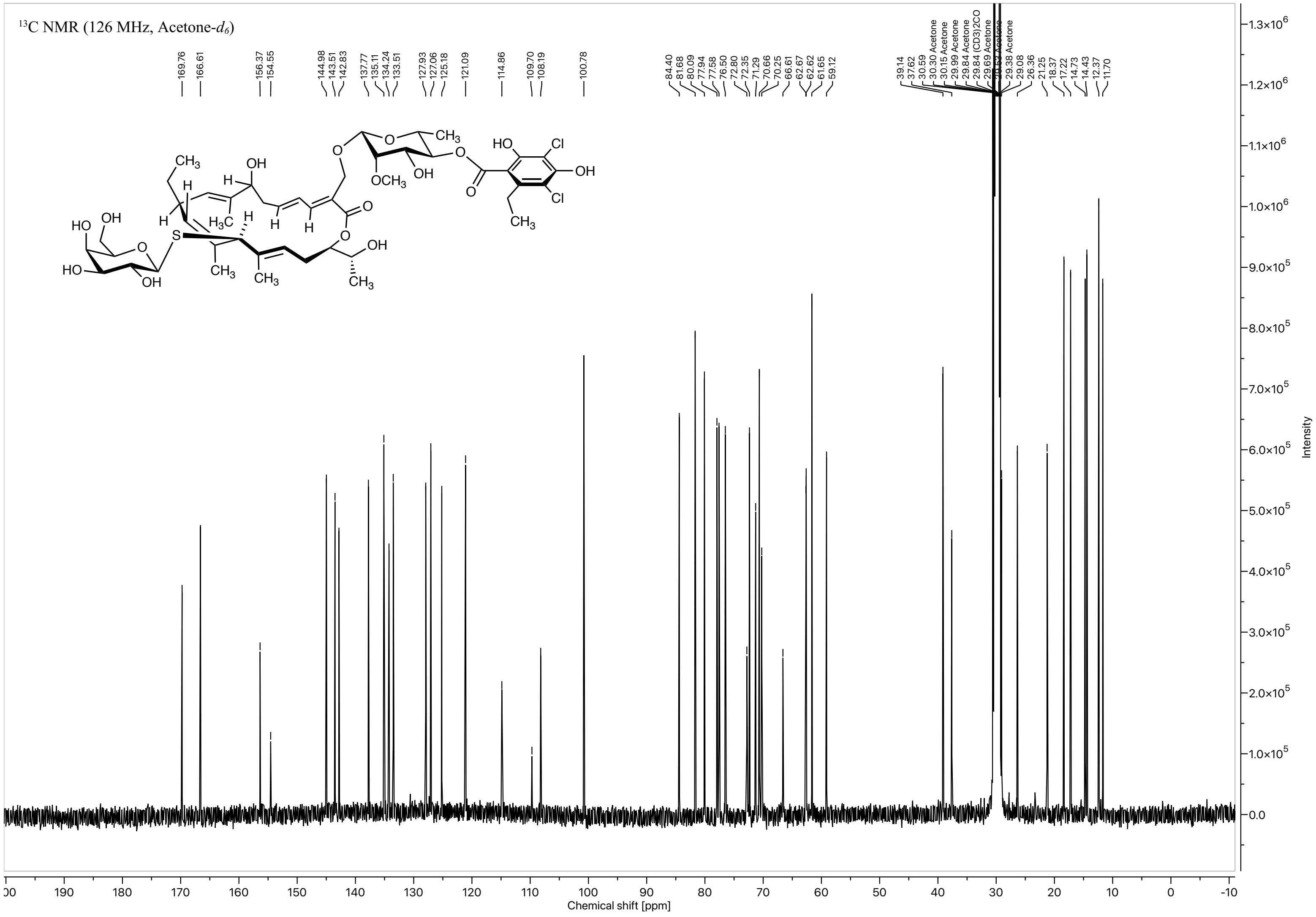

Figure 97: ^{13}C NMR spectrum of 11-desnoviosyl-13-thio-β-D-galactosyl fidaxomicin (18b-C(13)) in acetone-d_6

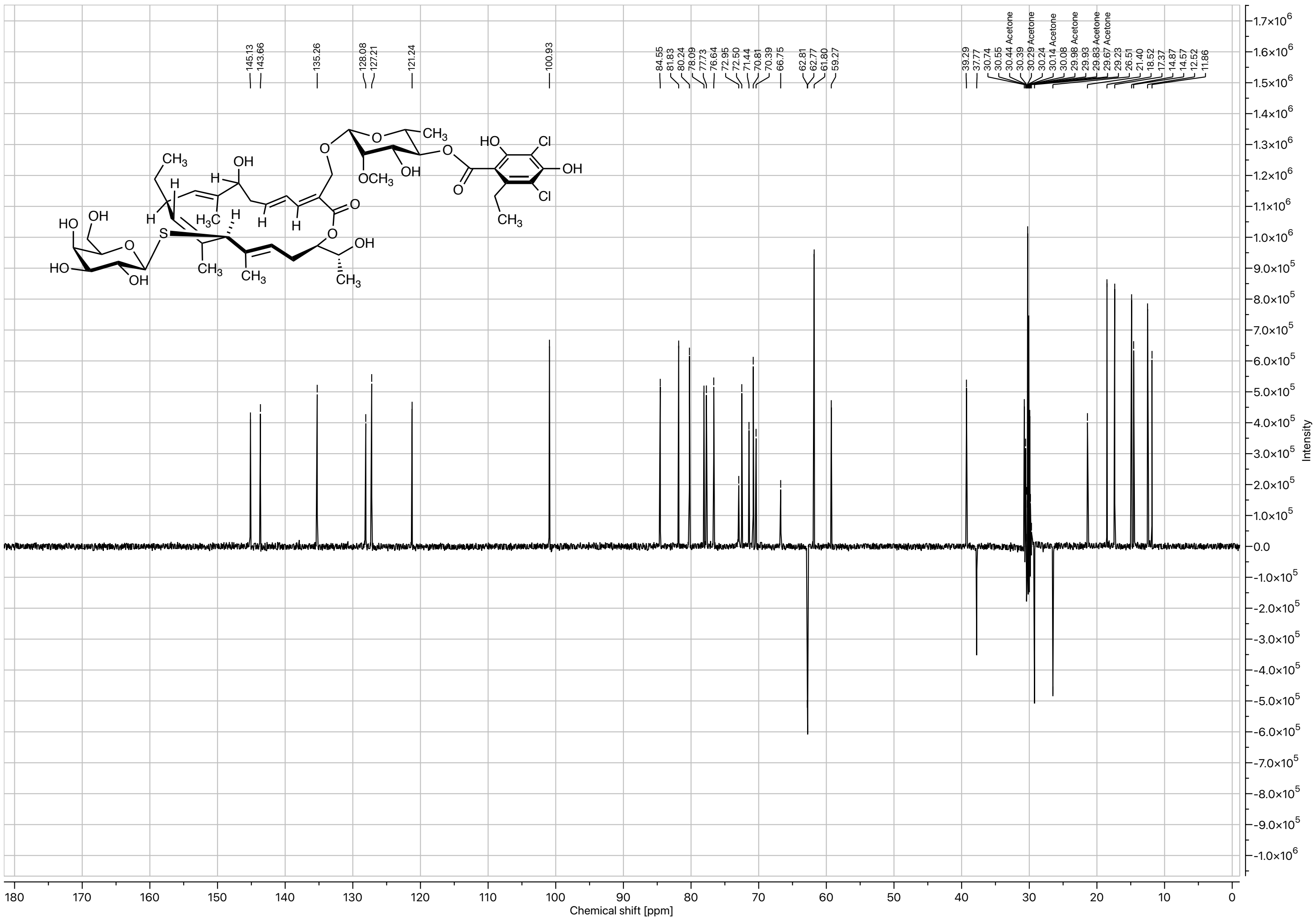

Figure 98: DEPT135 spectrum of 11-desnoviosyl-13-thio-β-D-galactosyl fidaxomicin (18b-C(13)) in acetone-d_6

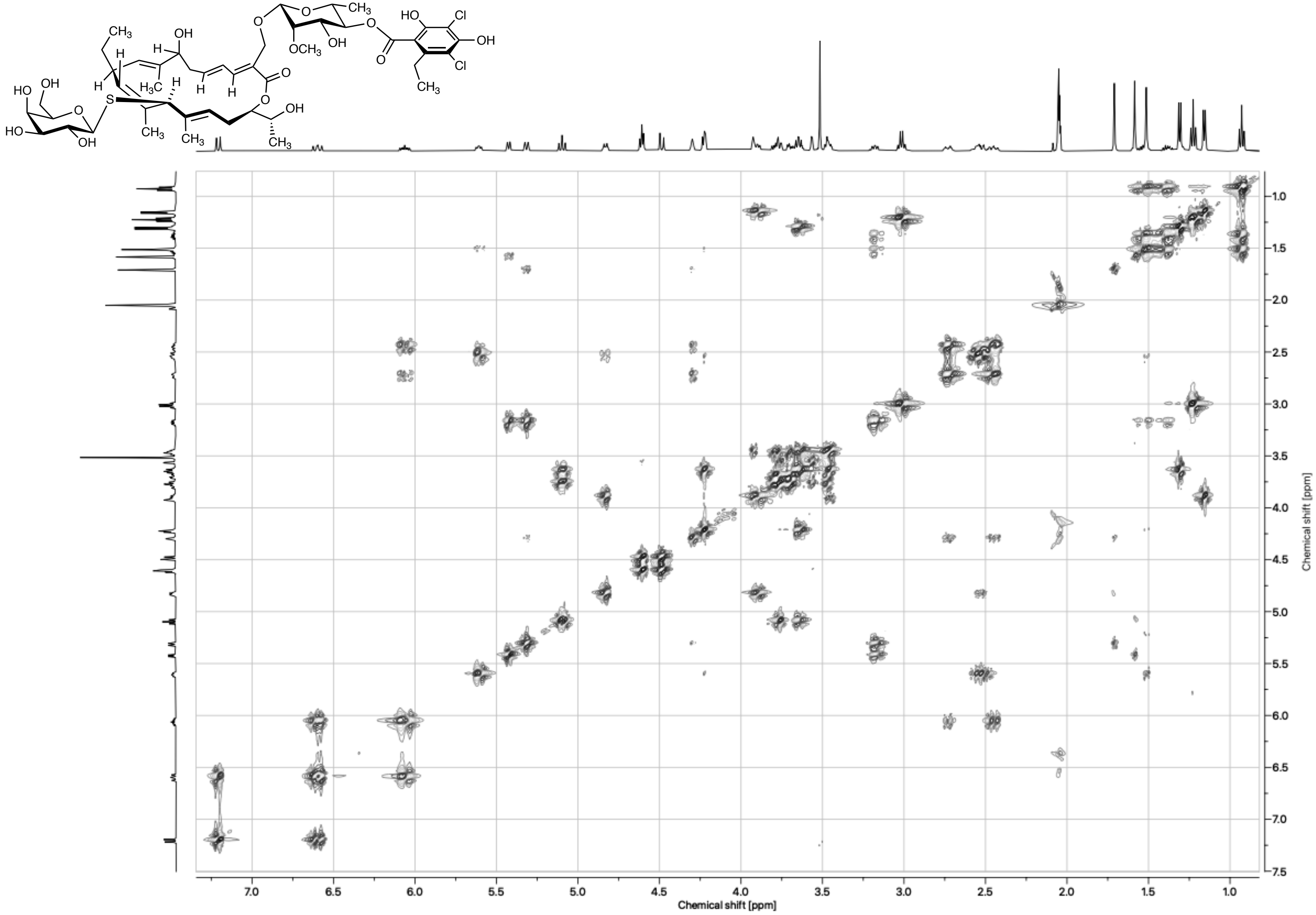

Figure 99: COSY spectrum of 11-desnoviosyl-13-thio-β-D-galactosyl fidaxomicin (18b-C(13)) in acetone-d_6

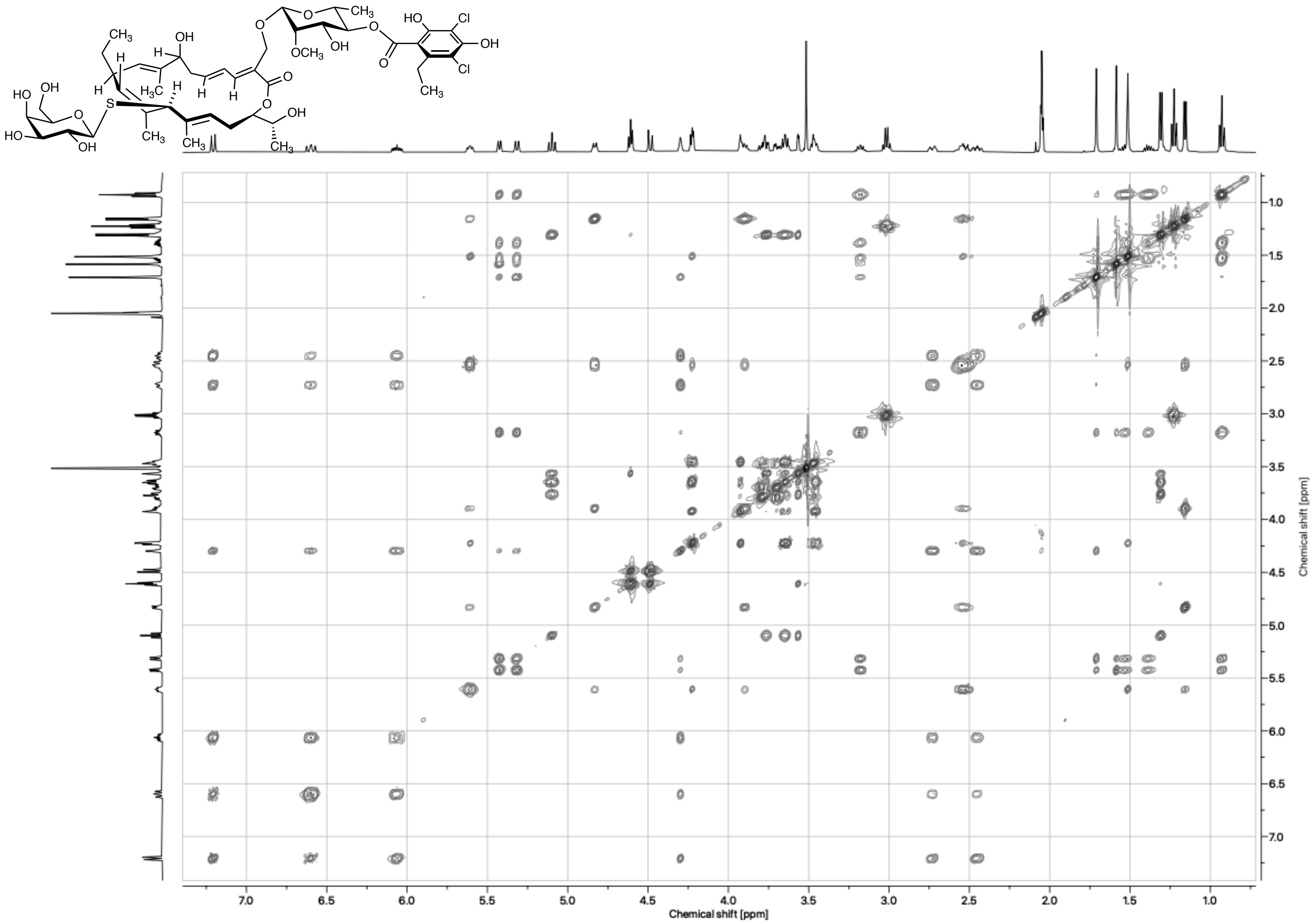

Figure 100: TOCSY spectrum of 11-desnoviosyl-13-thio-β-D-galactosyl fidaxomicin (18b-C(13)) in acetone-d_6

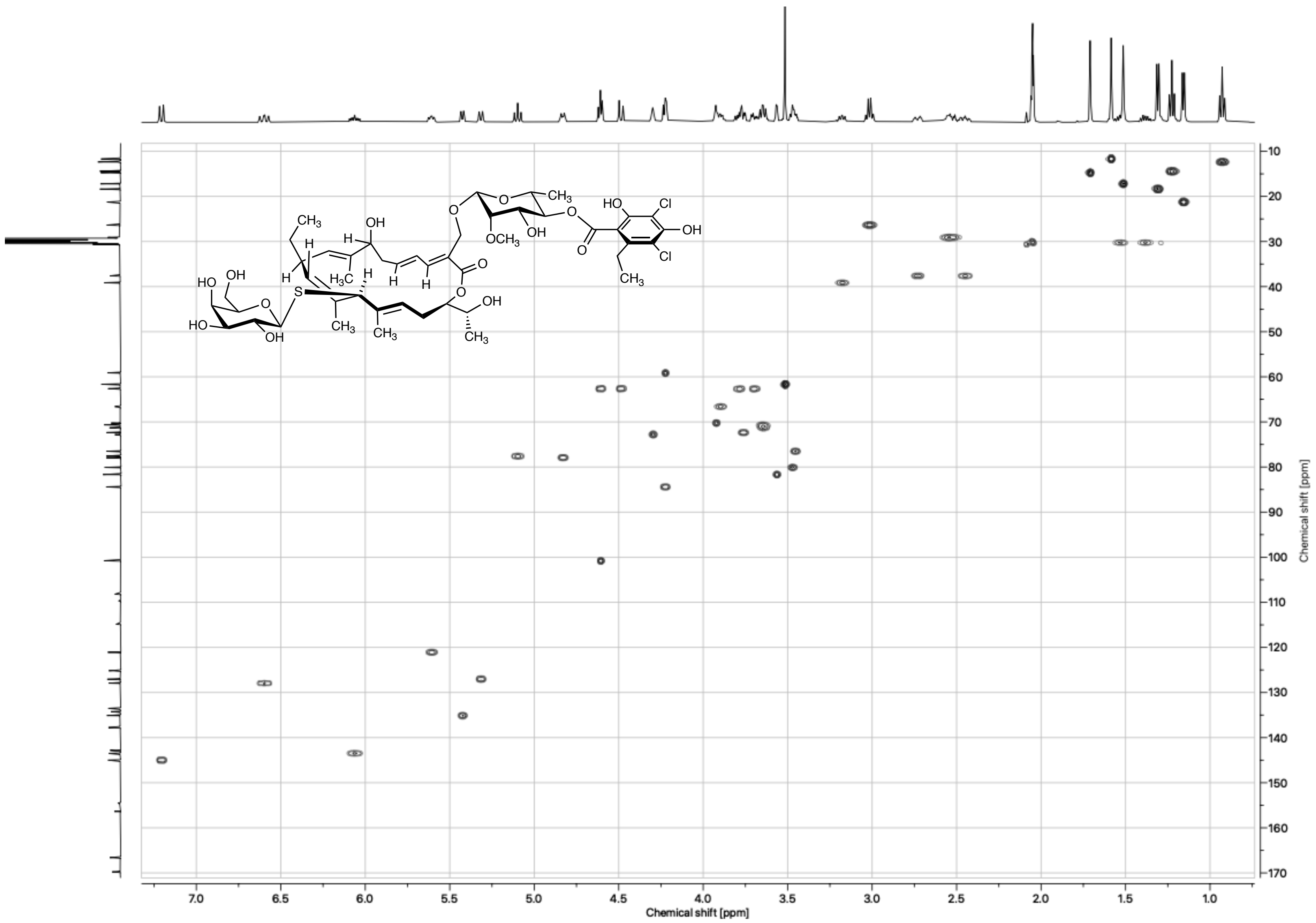

Figure 101: HSQC spectrum of 11-desnoviosyl-13-thio-β-D-galactosyl fidaxomicin (18b-C(13)) in acetone-d_6

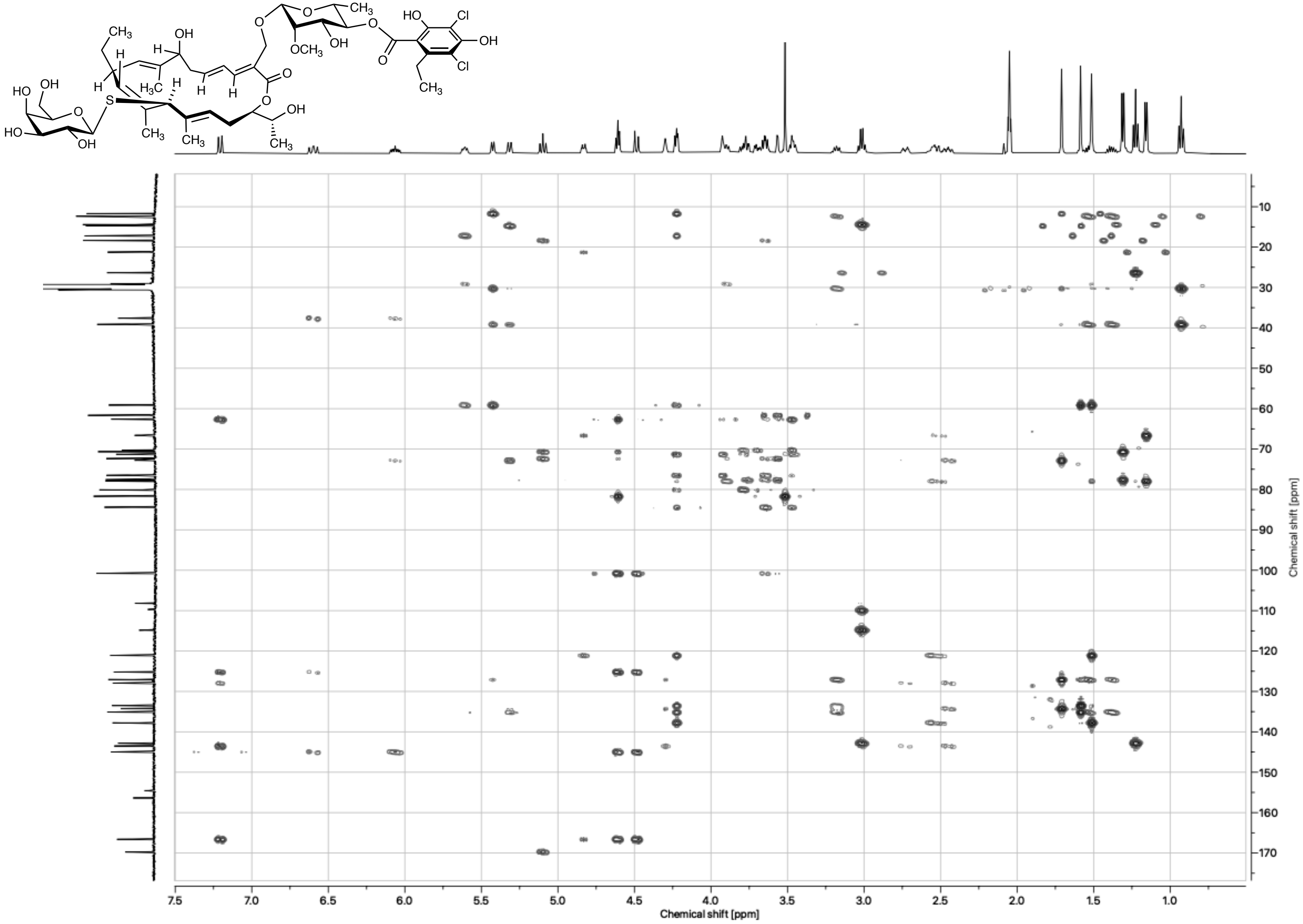

Figure 102: HMBC spectrum of 11-desnoviosyl-13-thio-β-D-galactosyl fidaxomicin (18b-C(13)) in acetone-d_6

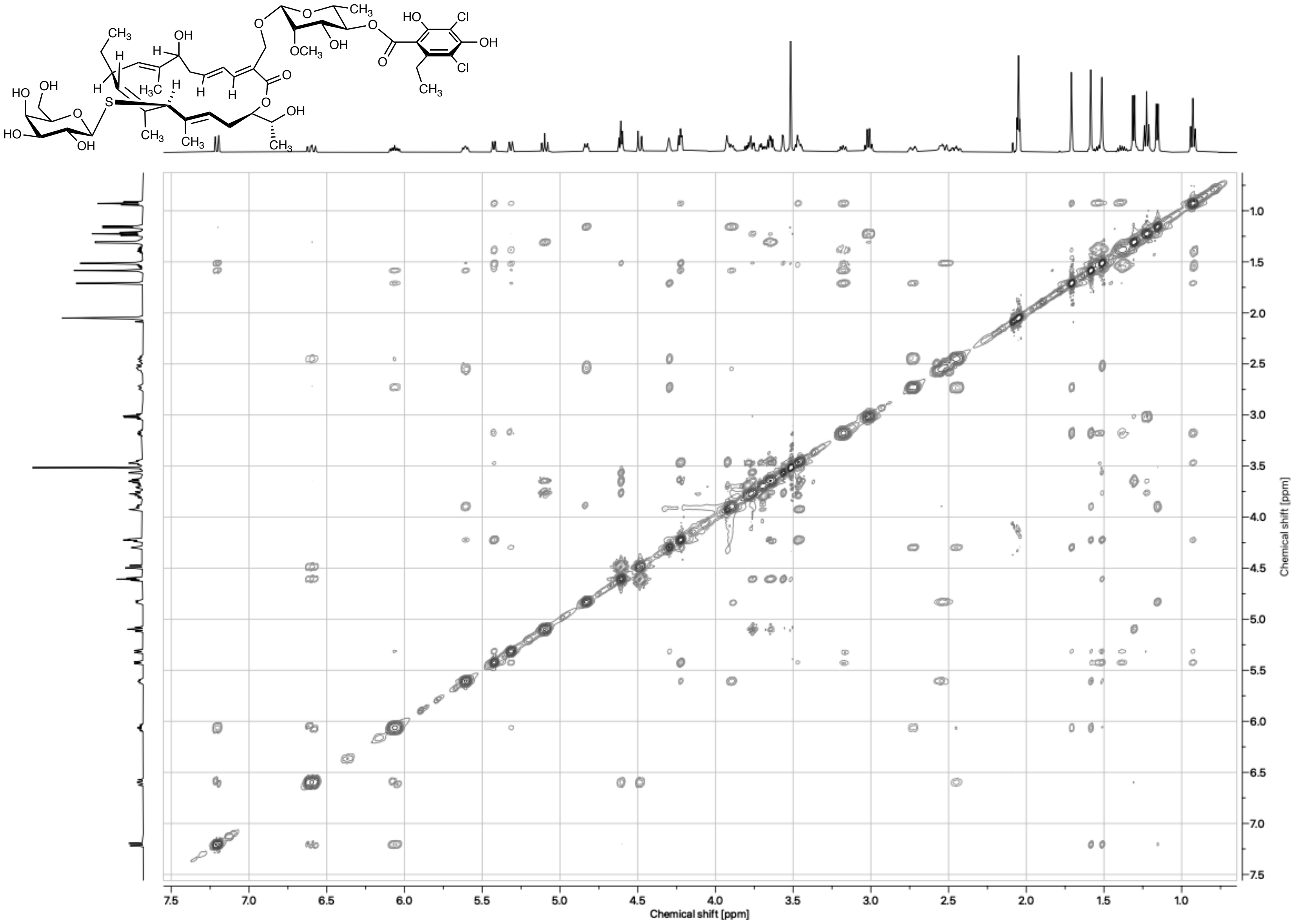

Figure 103: NOESY spectrum of 11-desnoviosyl-13-thio-β-D-galactosyl fidaxomicin (18b-C(13)) in acetone-d_6

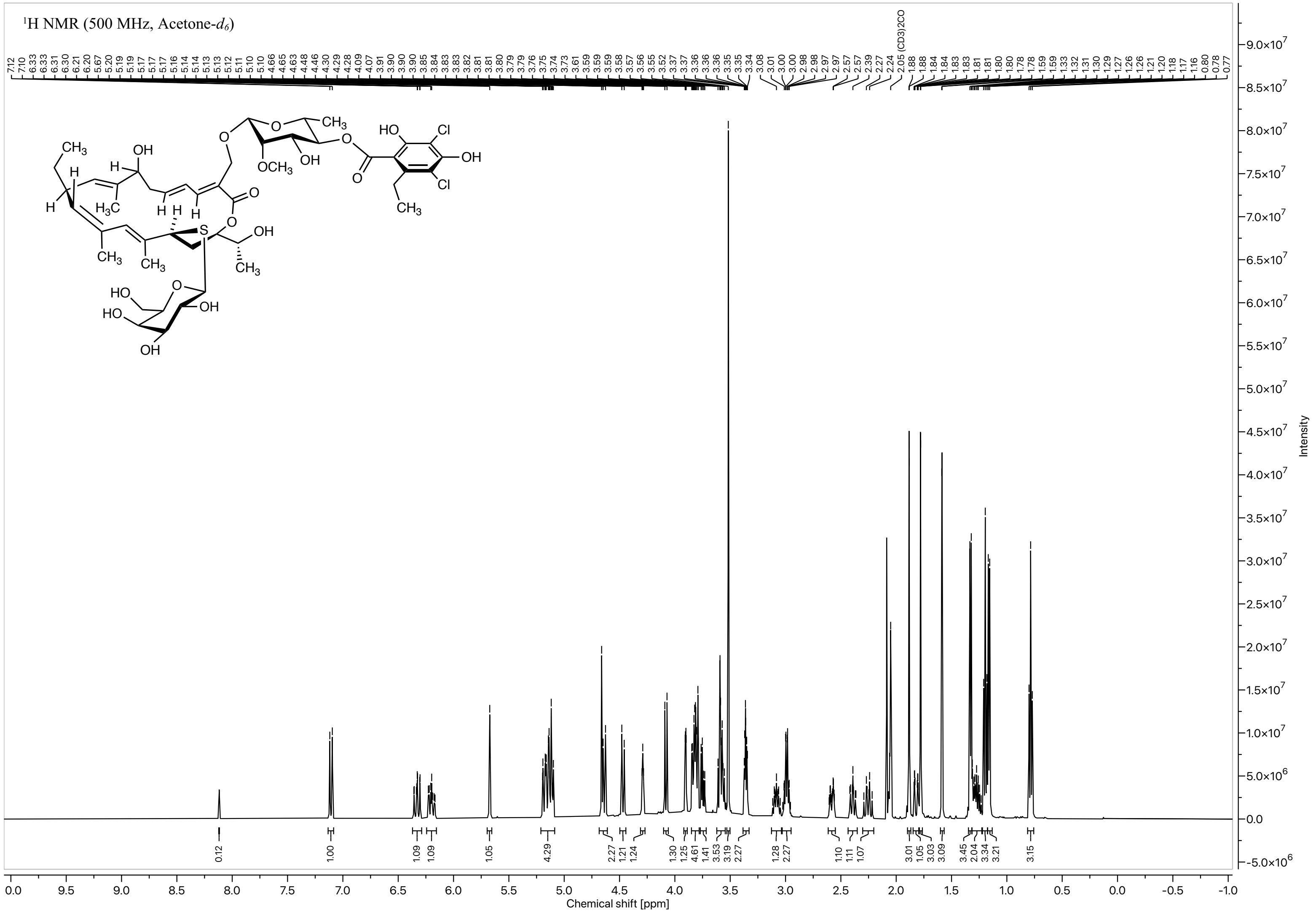

Figure 104: ^{1}H NMR spectrum of 11-desnoviosyl-15-thio-β-D-galactosyl fidaxomicin (18b-C(15)) in acetone-d_6

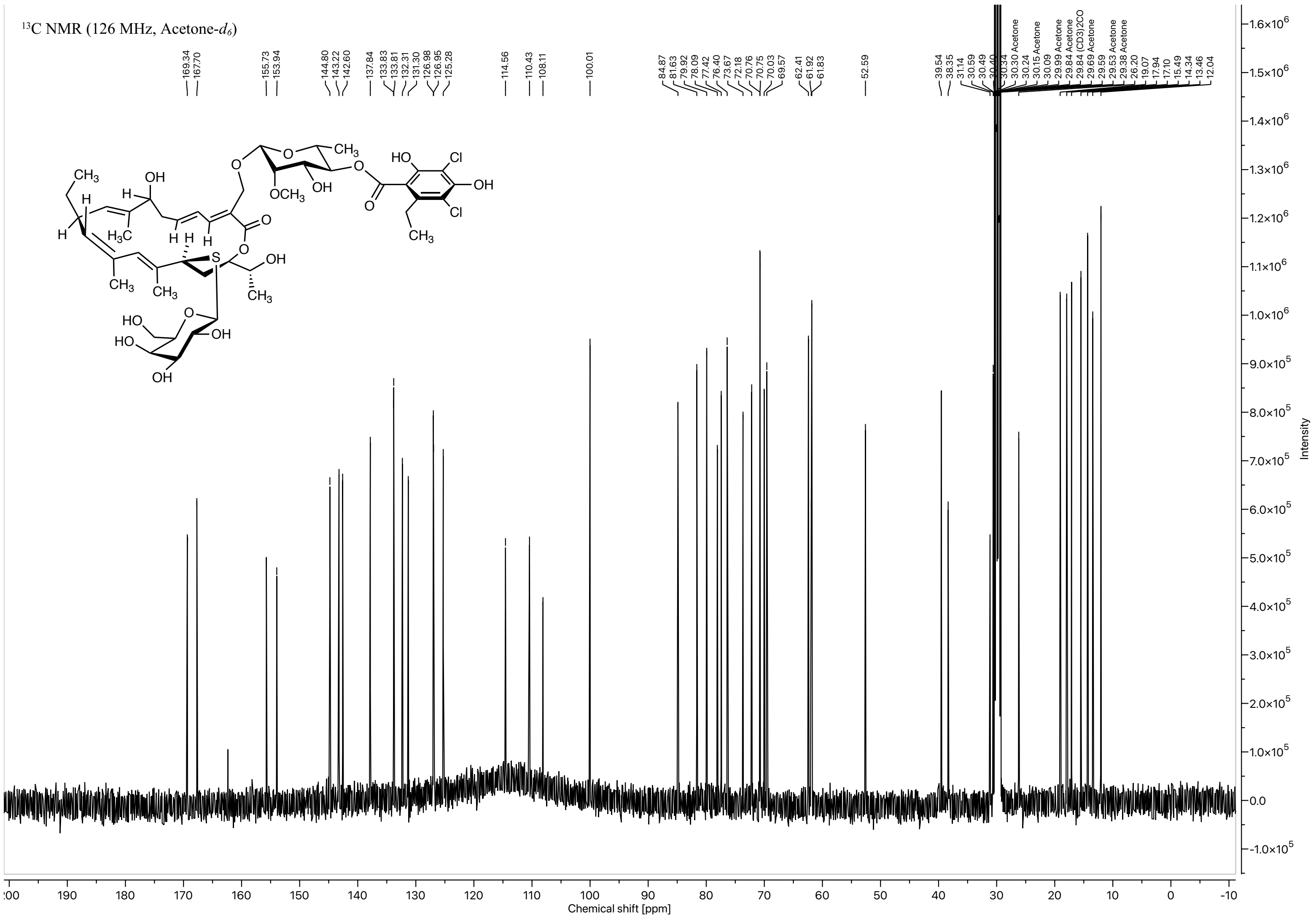

Figure 105: ^{13}C NMR spectrum of 11-desnoviosyl-15-thio-β-D-galactosyl fidaxomicin (18b-C(15)) in acetone-d_6

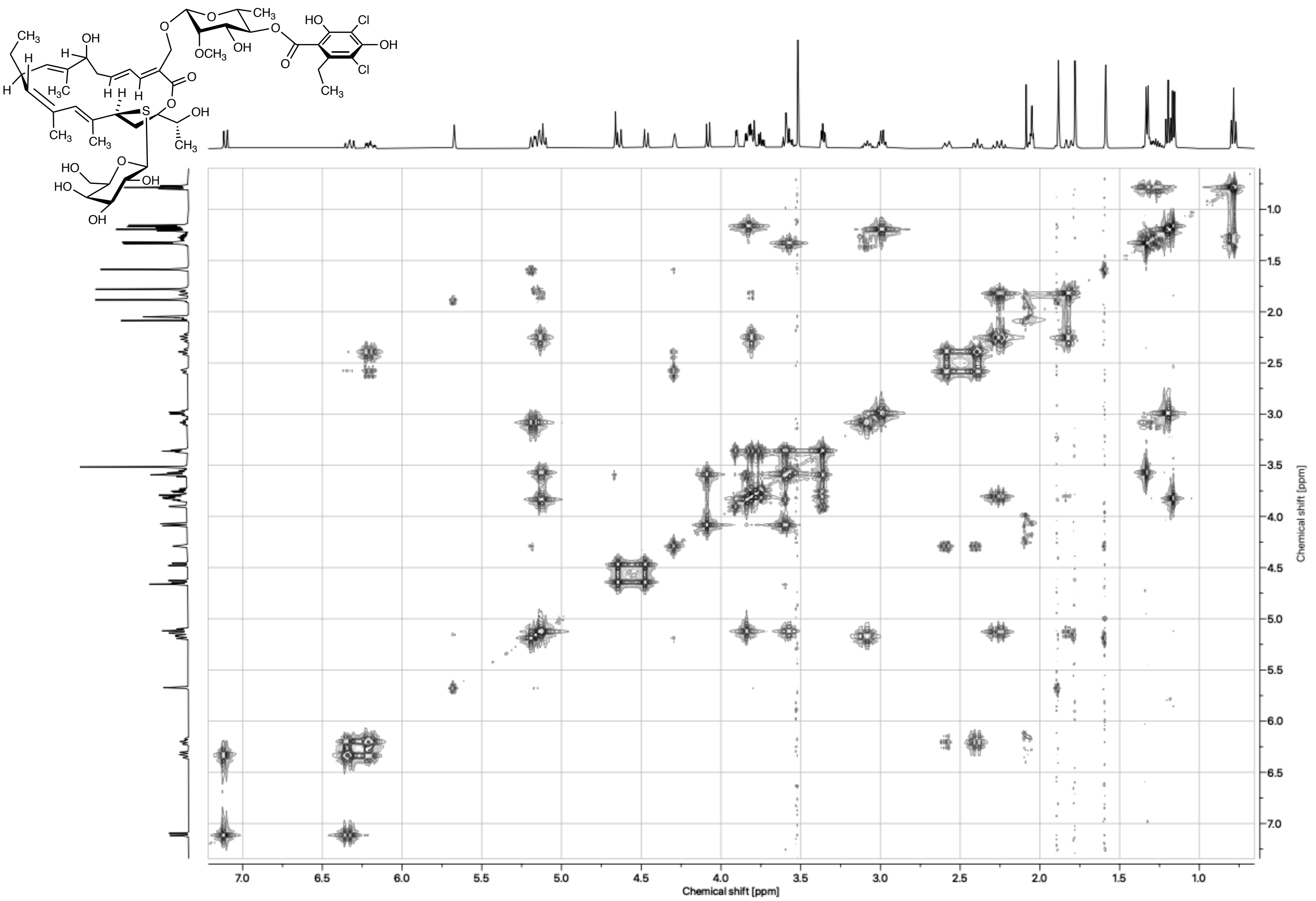

Figure 106: COSY spectrum of 11-desnoviosyl-15-thio-β-D-galactosyl fidaxomicin (18b-C(15)) in acetone-d_6

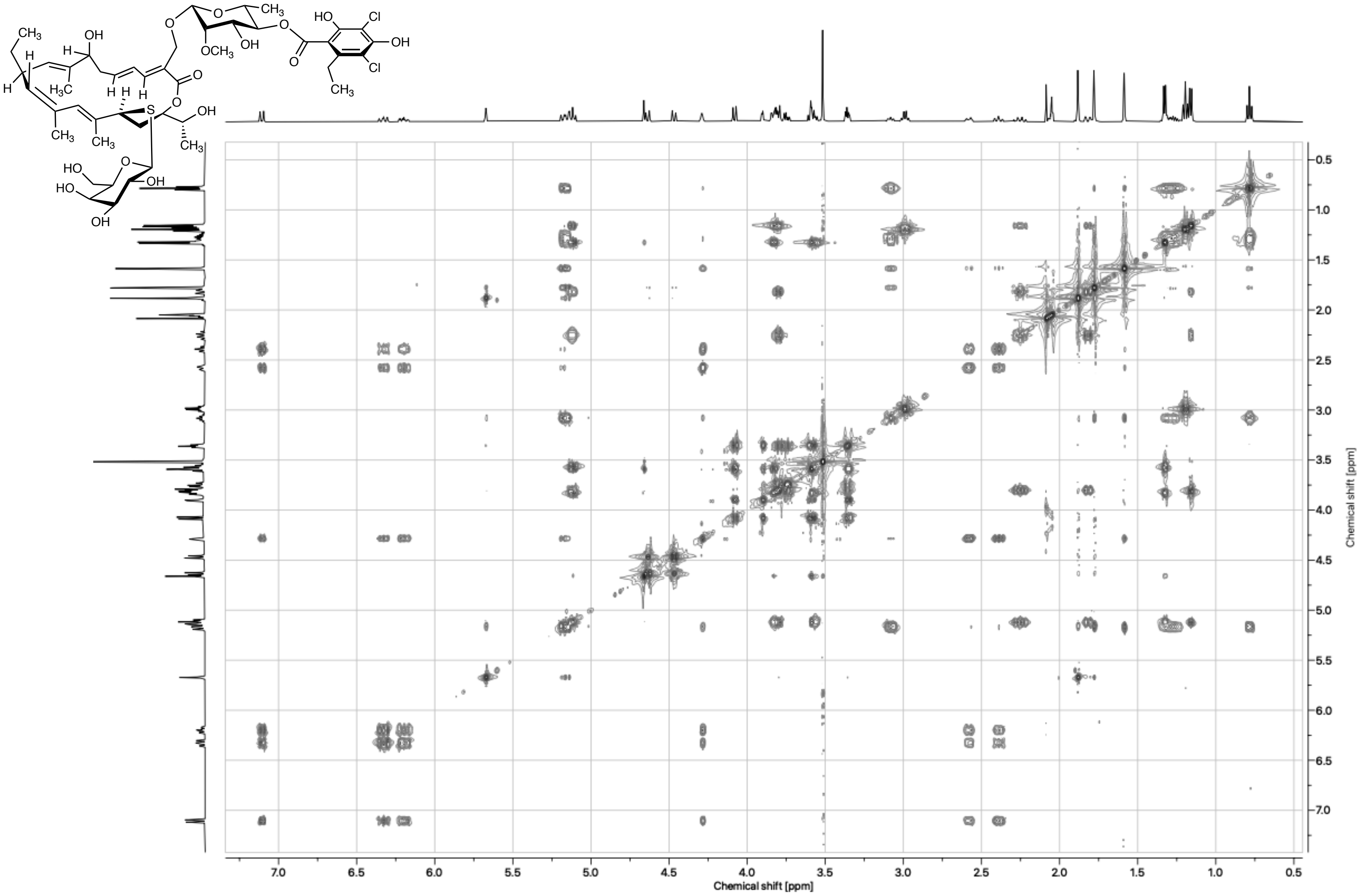

Figure 107: TOCSY spectrum of 11-desnoviosyl-15-thio-β-D-galactosyl fidaxomicin (18b-C(15)) in acetone-d_6

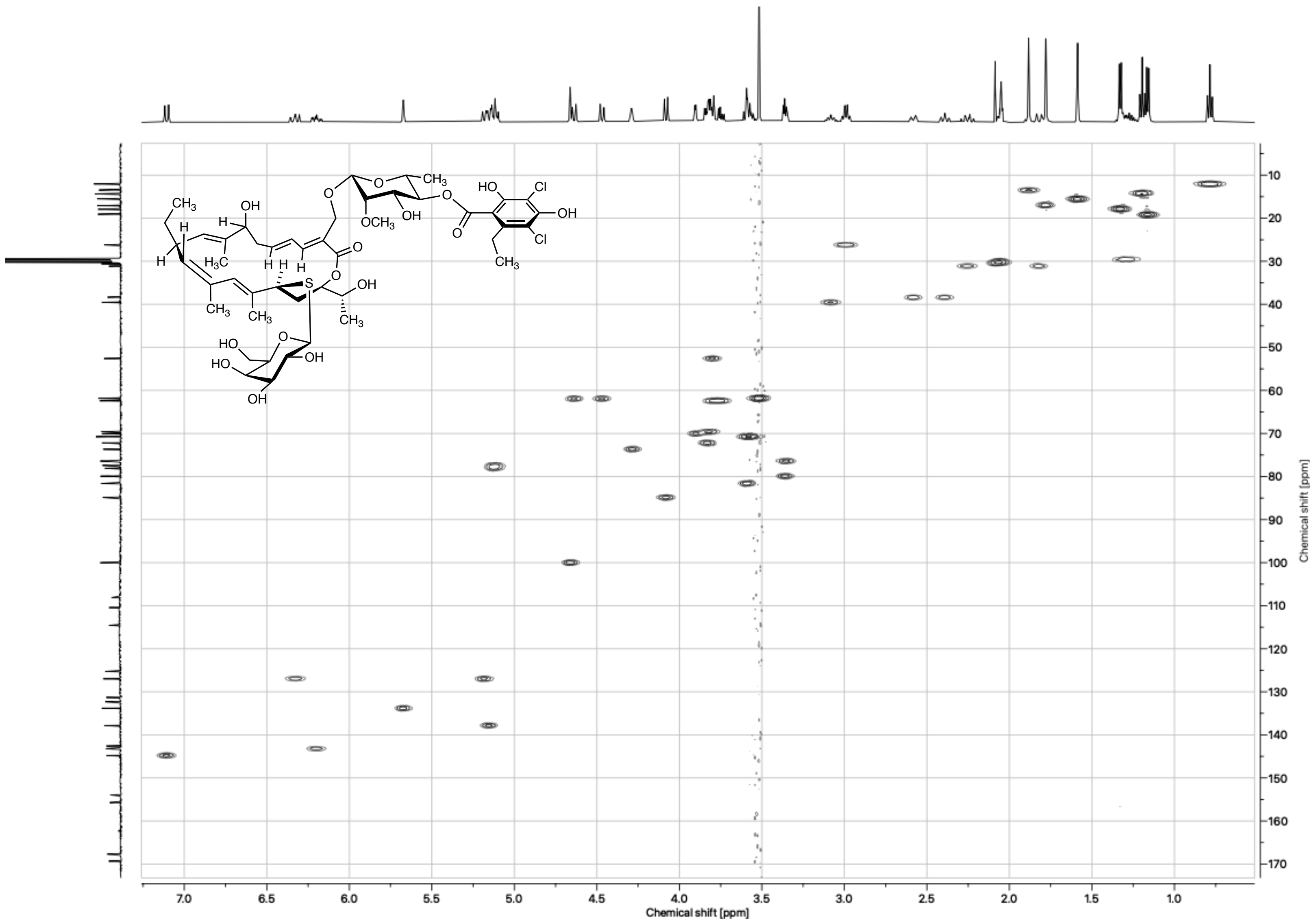

Figure 108: HSQC spectrum of 11-desnoviosyl-15-thio-β-D-galactosyl fidaxomicin (18b-C(15)) in acetone-d_6

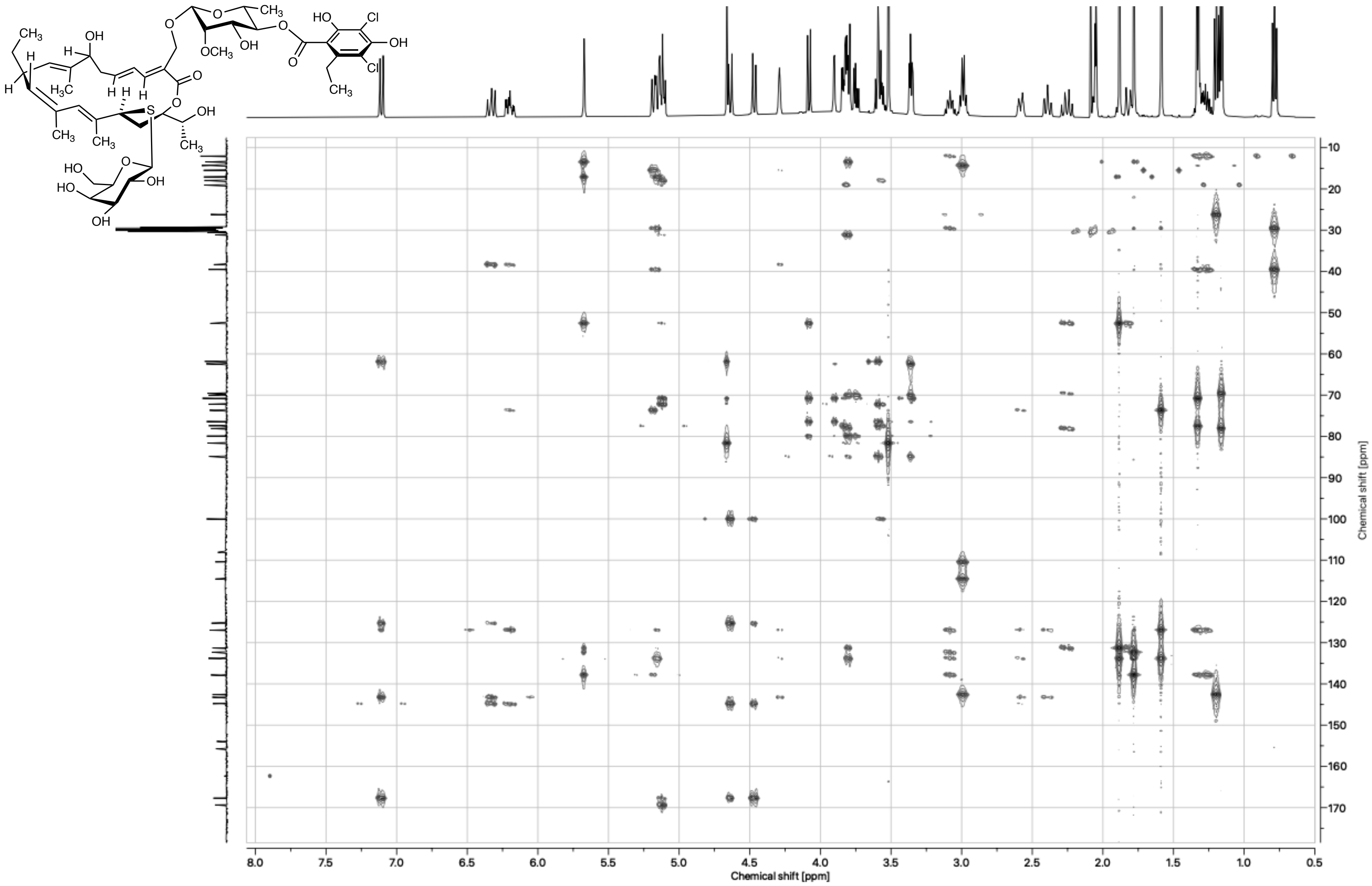

Figure 109: HMBC spectrum of 11-desnoviosyl-15-thio-β-D-galactosyl fidaxomicin (18b-C(15)) in acetone-d_6

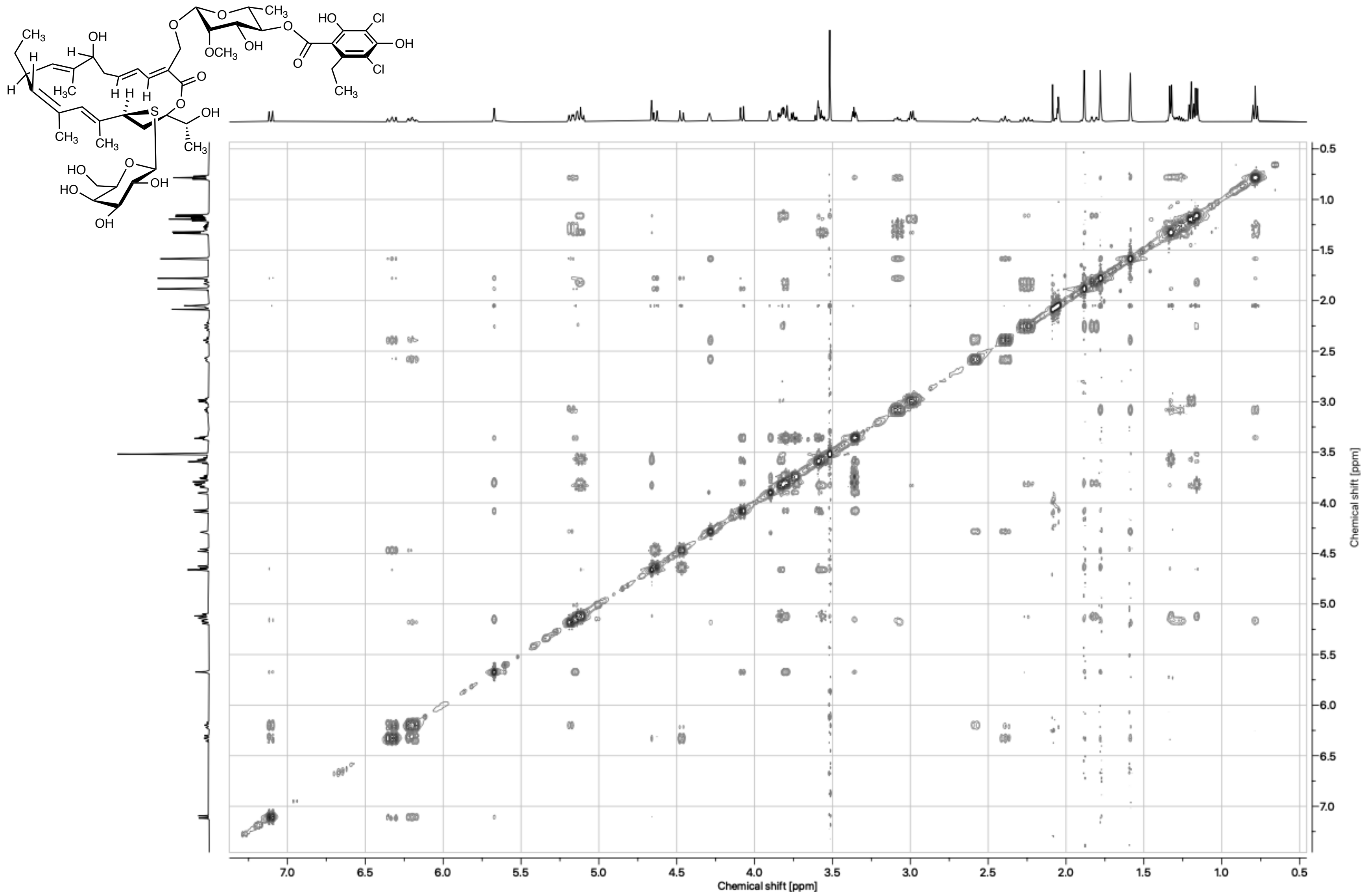

Figure 110: NOESY spectrum of 11-desnoviosyl-15-thio-β-D-galactosyl fidaxomicin (18b-C(15)) in acetone-d_6

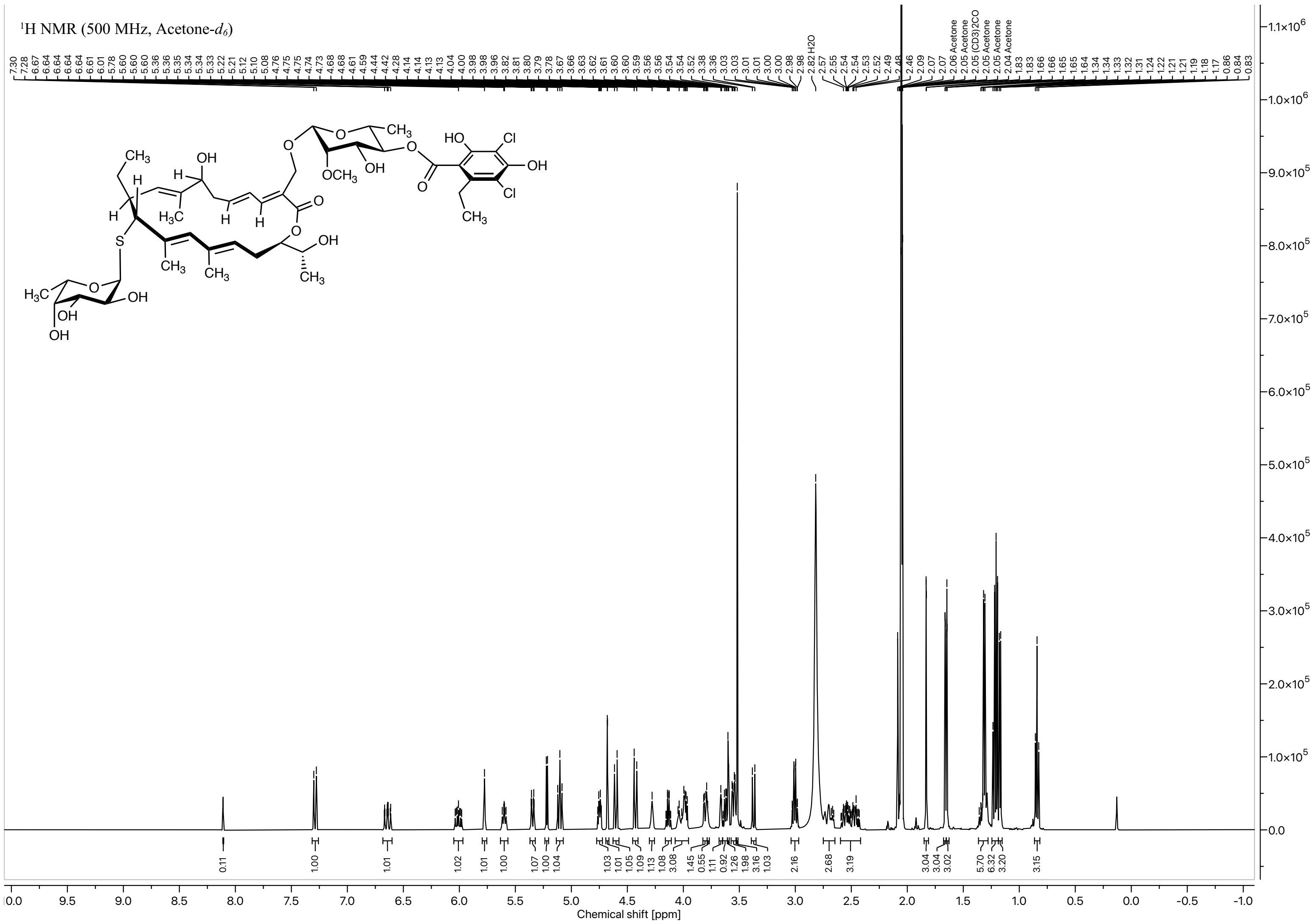

Figure 111: ^{1}H NMR spectrum of 11-desnoviosyl-11-thio-α-L-fucosyl fidaxomicin (18c-C(11)) in acetone-d_6

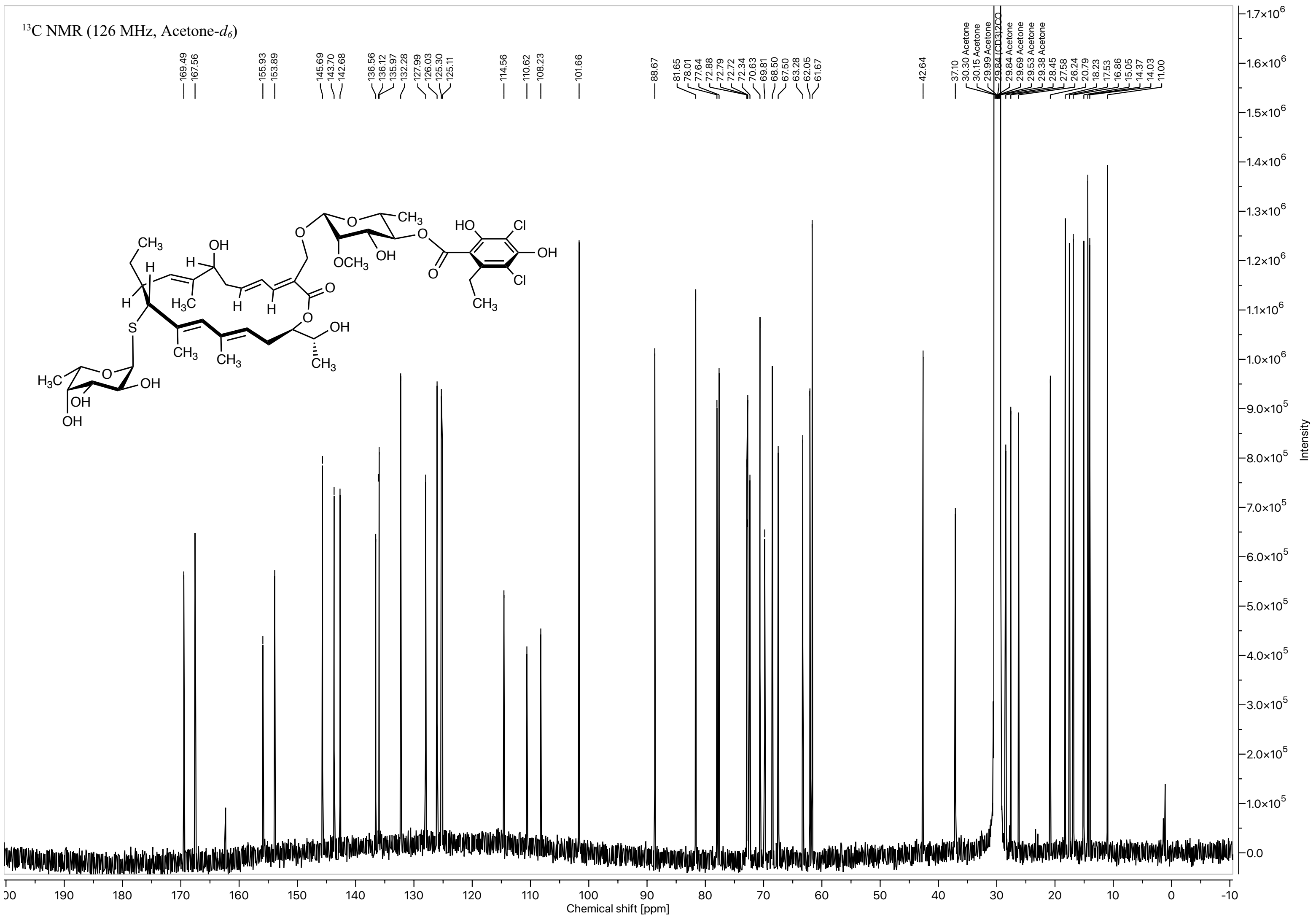

Figure 112: ^{13}C NMR spectrum of 11-desnoviosyl-11-thio-α-L-fucosyl fidaxomicin (18c-C(11)) in acetone-d_6

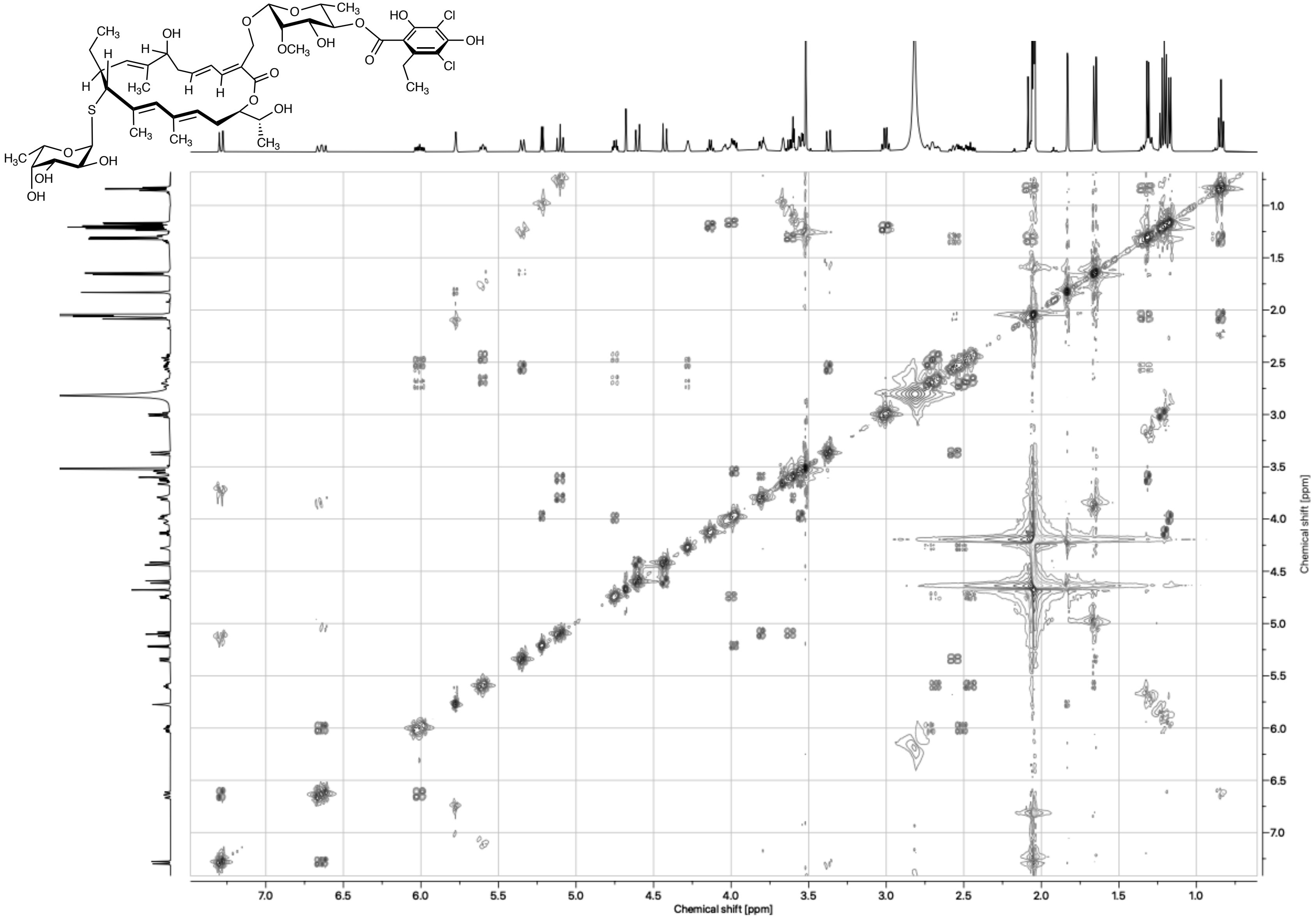

Figure 113: COSY spectrum of 11-desnoviosyl-11-thio-α-L-fucosyl fidaxomicin (18c-C(11)) in acetone-d_6

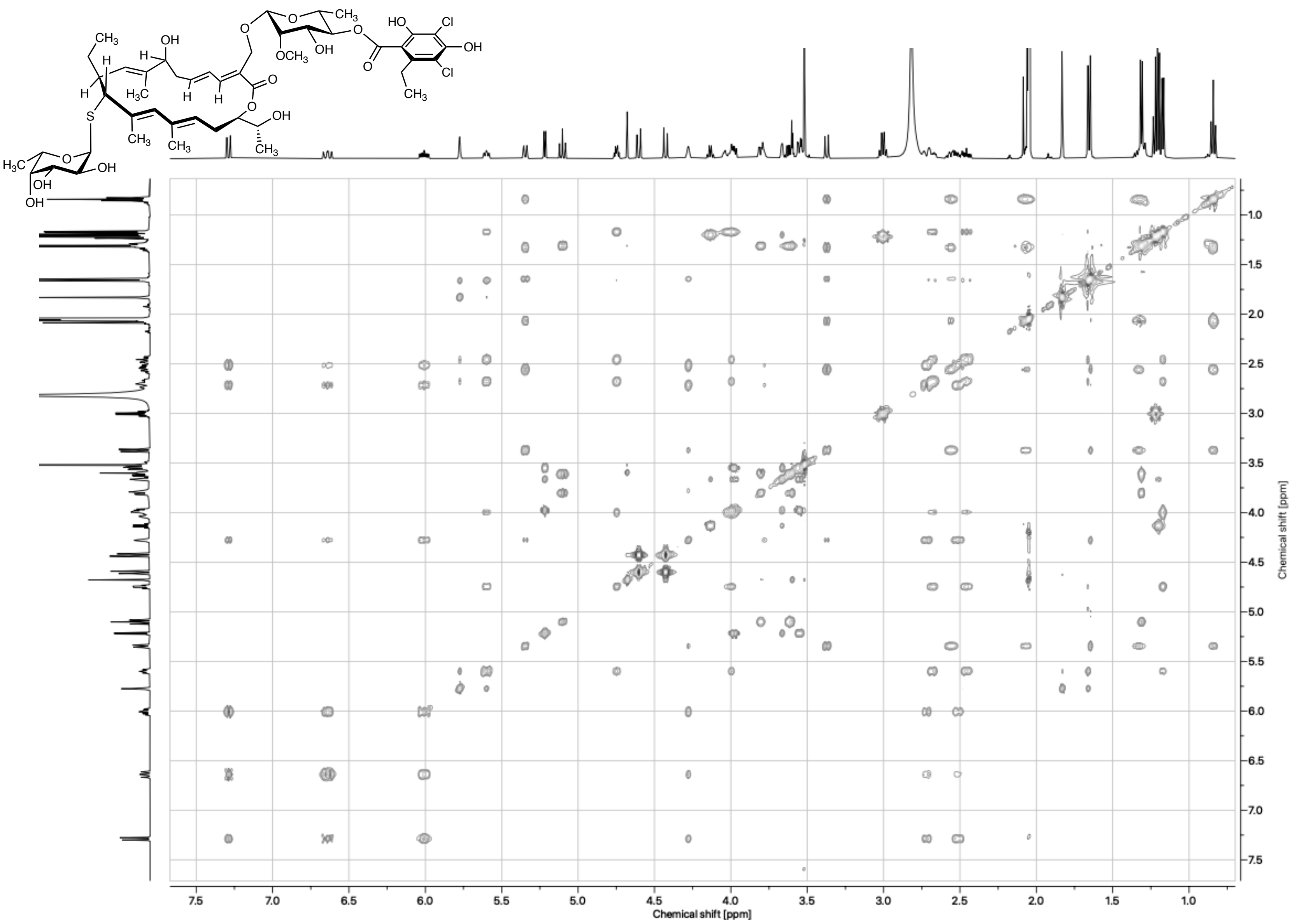

Figure 114: TOCSY spectrum of 11-desnoviosyl-11-thio-α-L-fucosyl fidaxomicin (18c-C(11)) in acetone-d_6

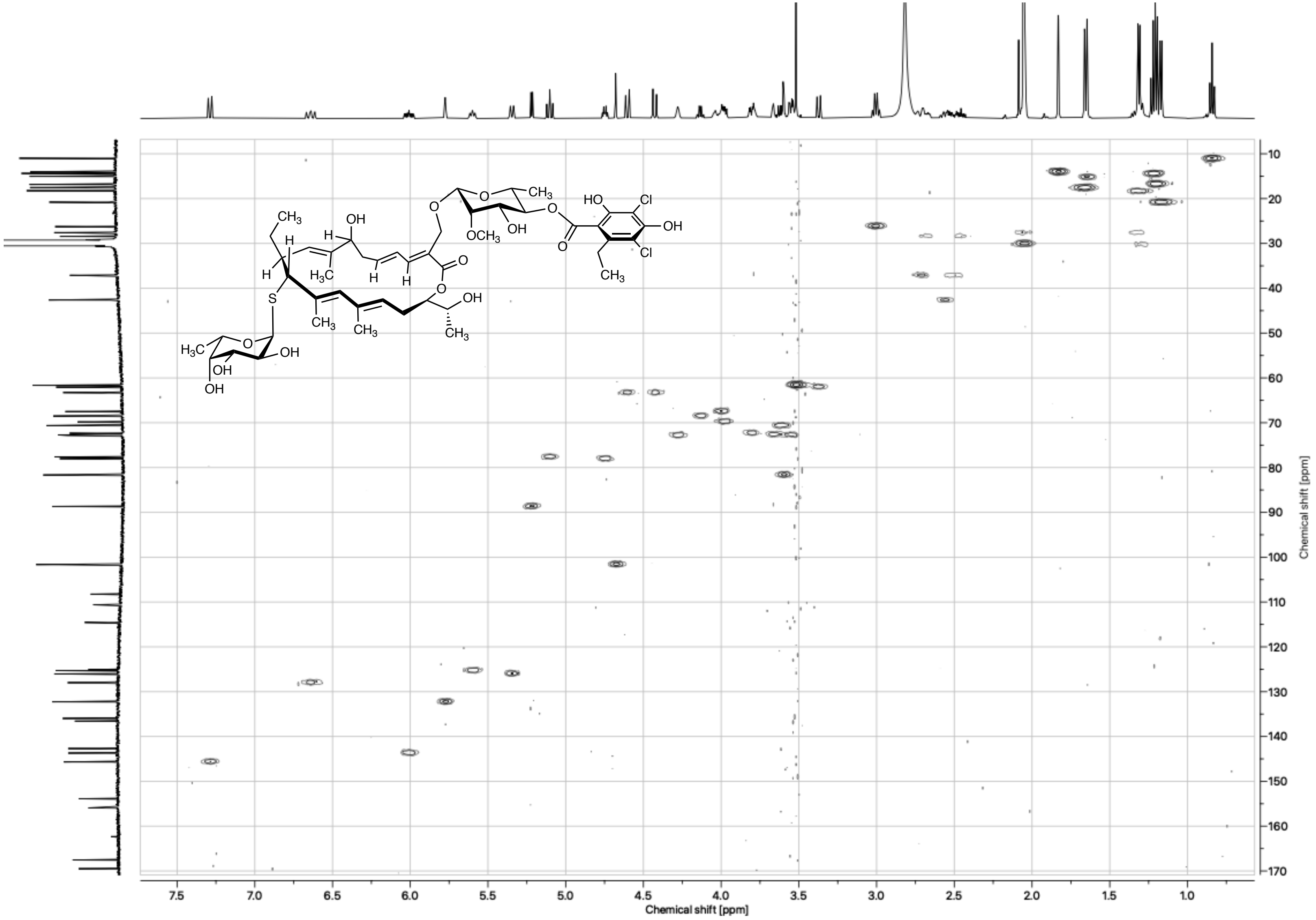

Figure 115. HSQC spectrum of 11-desnoviosyl-11-thio-α-L-fucosyl fidaxomicin (18c-C(11)) in acetone-d_6

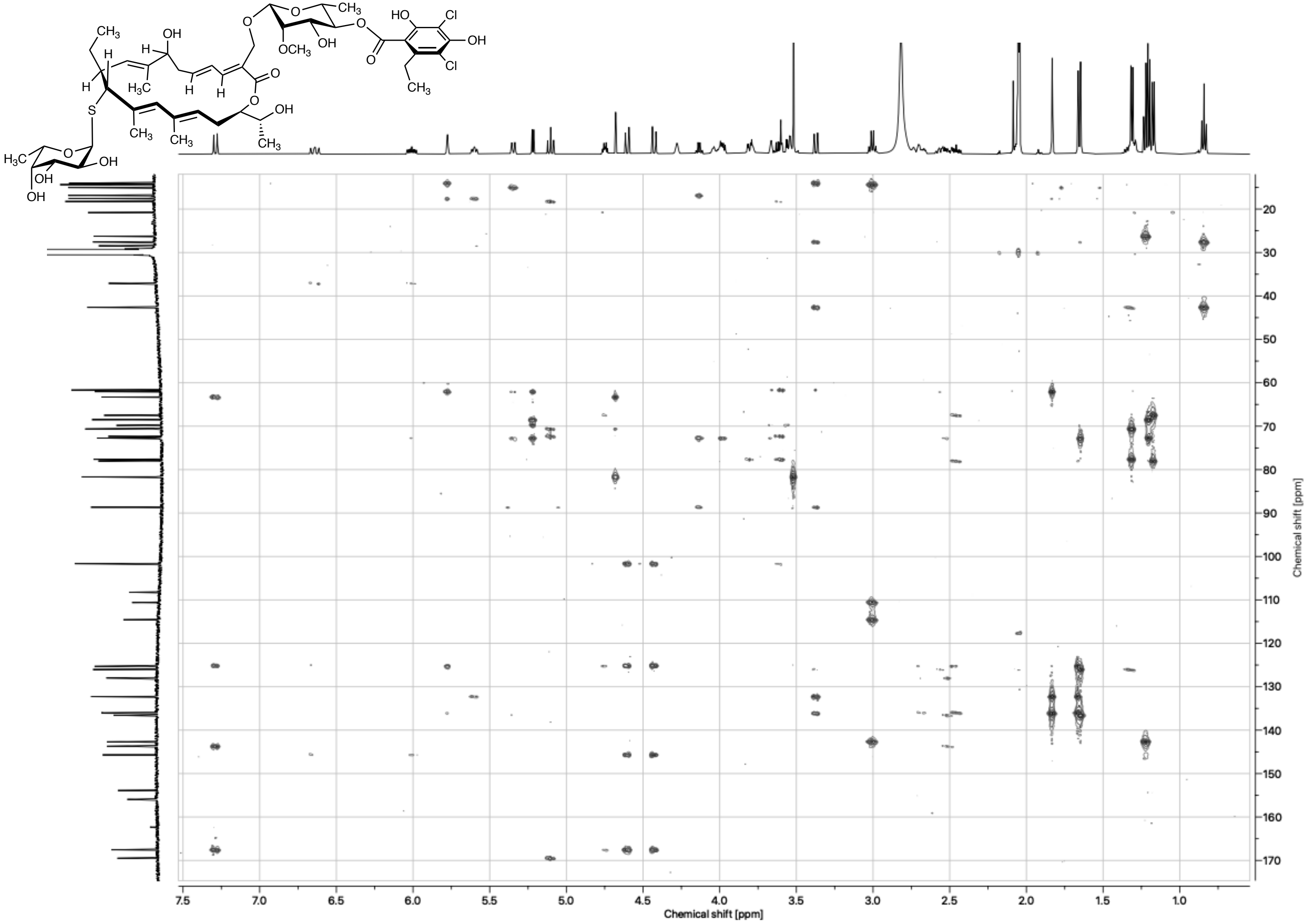

Figure 116: HMBC spectrum of 11-desnoviosyl-11-thio-α-L-fucosyl fidaxomicin (18c-C(11)) in acetone-d_6

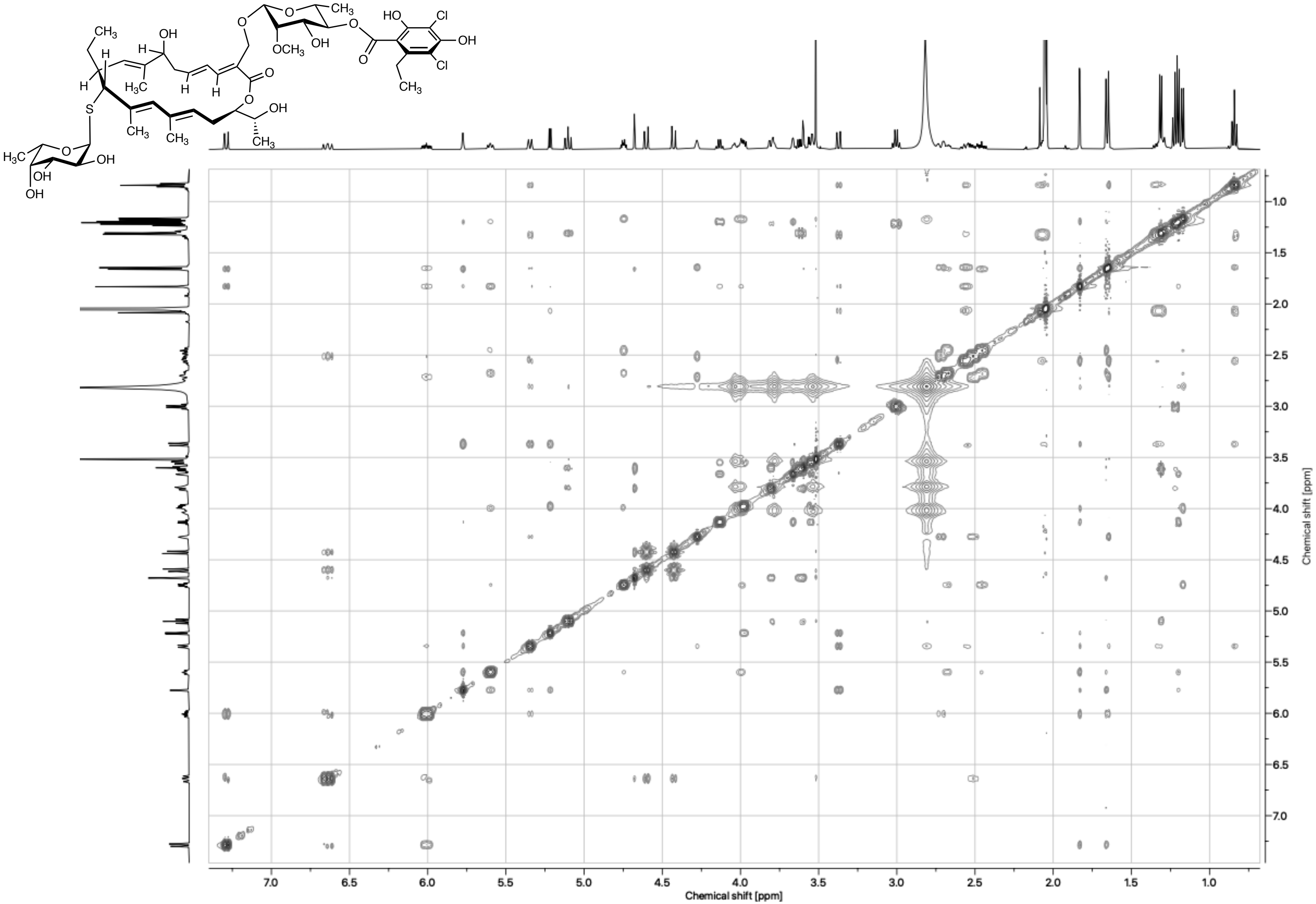

Figure 117: NOESY spectrum of 11-desnoviosyl-11-thio-α-L-fucosyl fidaxomicin (18c-C(11)) in acetone-d_6

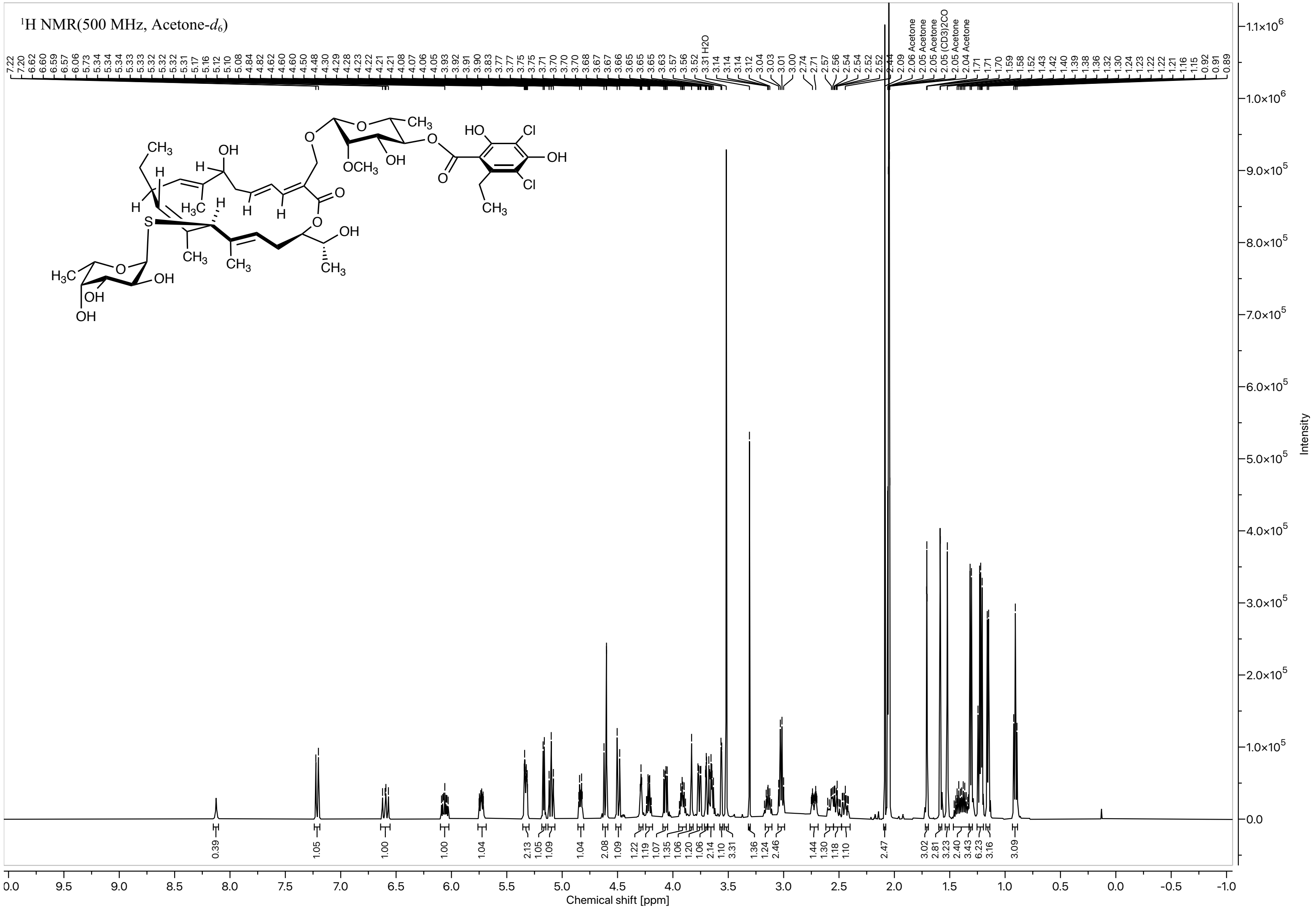

Figure 118: ^{1}H NMR spectrum of 11-desnoviosyl-13-thio-α-L-fucosyl fidaxomicin (18c-C(13)) in acetone-d_6

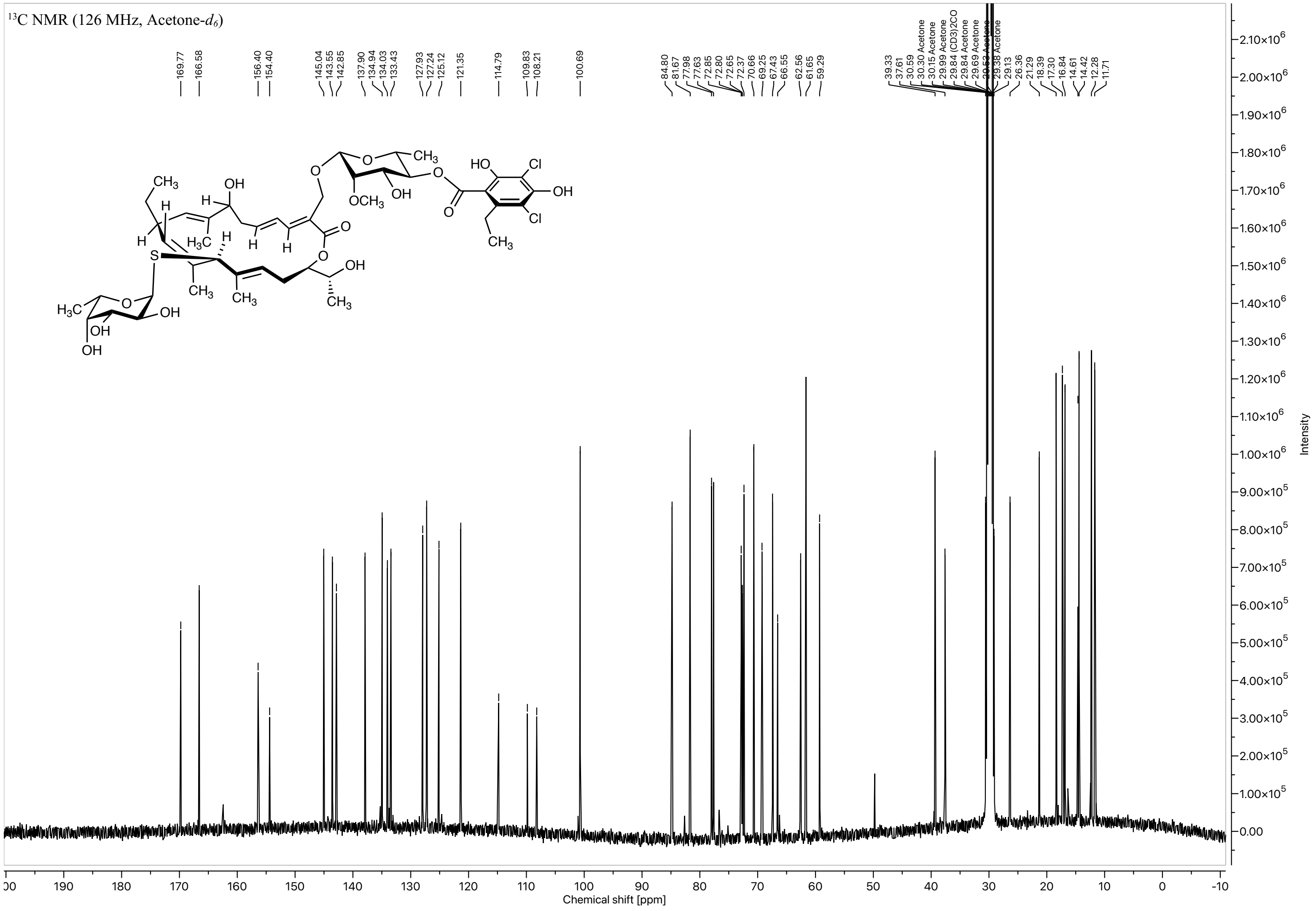

Figure 119: ^{13}C NMR spectrum of 11-desnoviosyl-13-thio-α-L-fucosyl fidaxomicin (18c-C(13)) in acetone-d_6

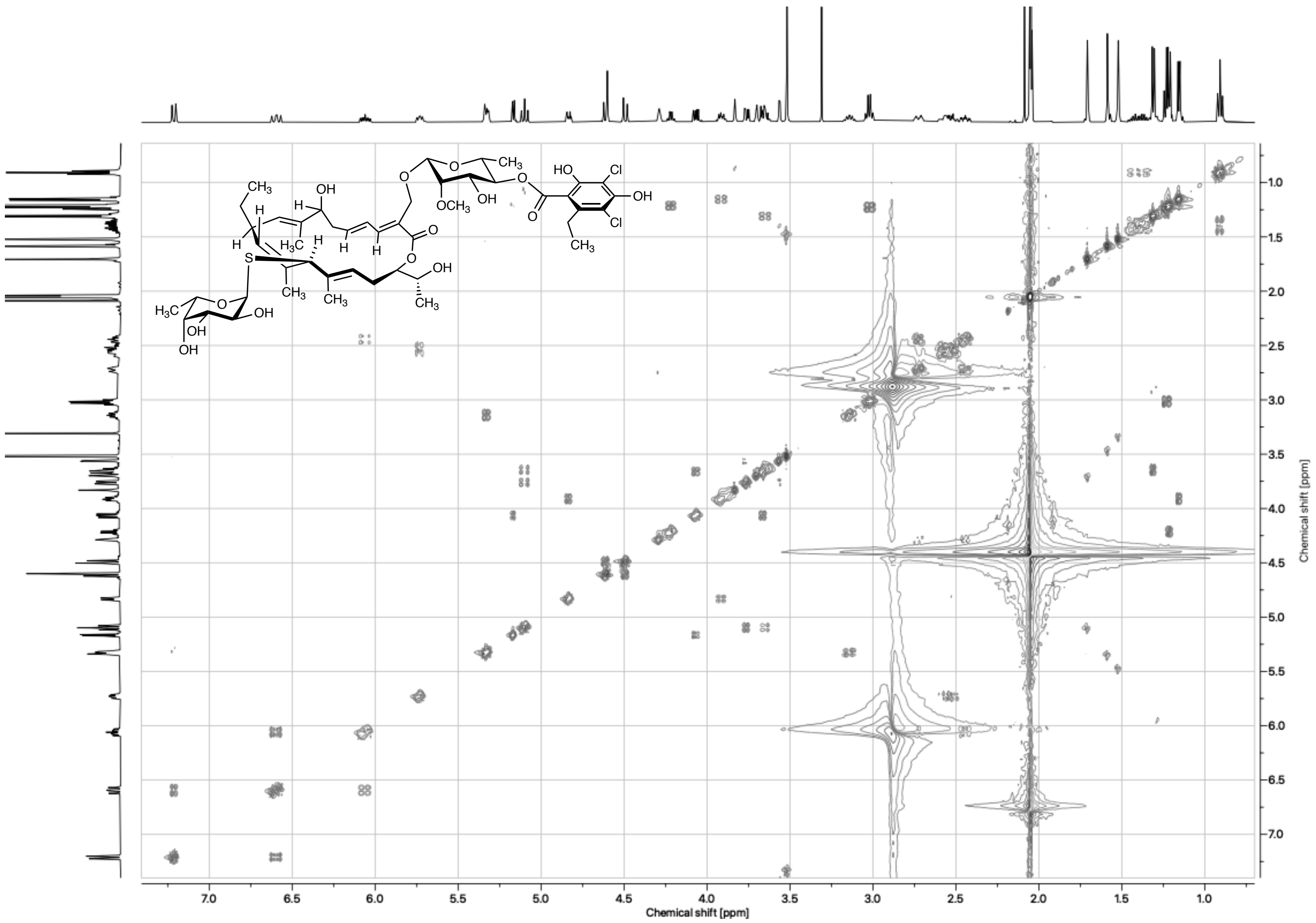

Figure 120: COSY spectrum of 11-desnoviosyl-13-thio-α-L-fucosyl fidaxomicin (18c-C(13)) in acetone-d_6

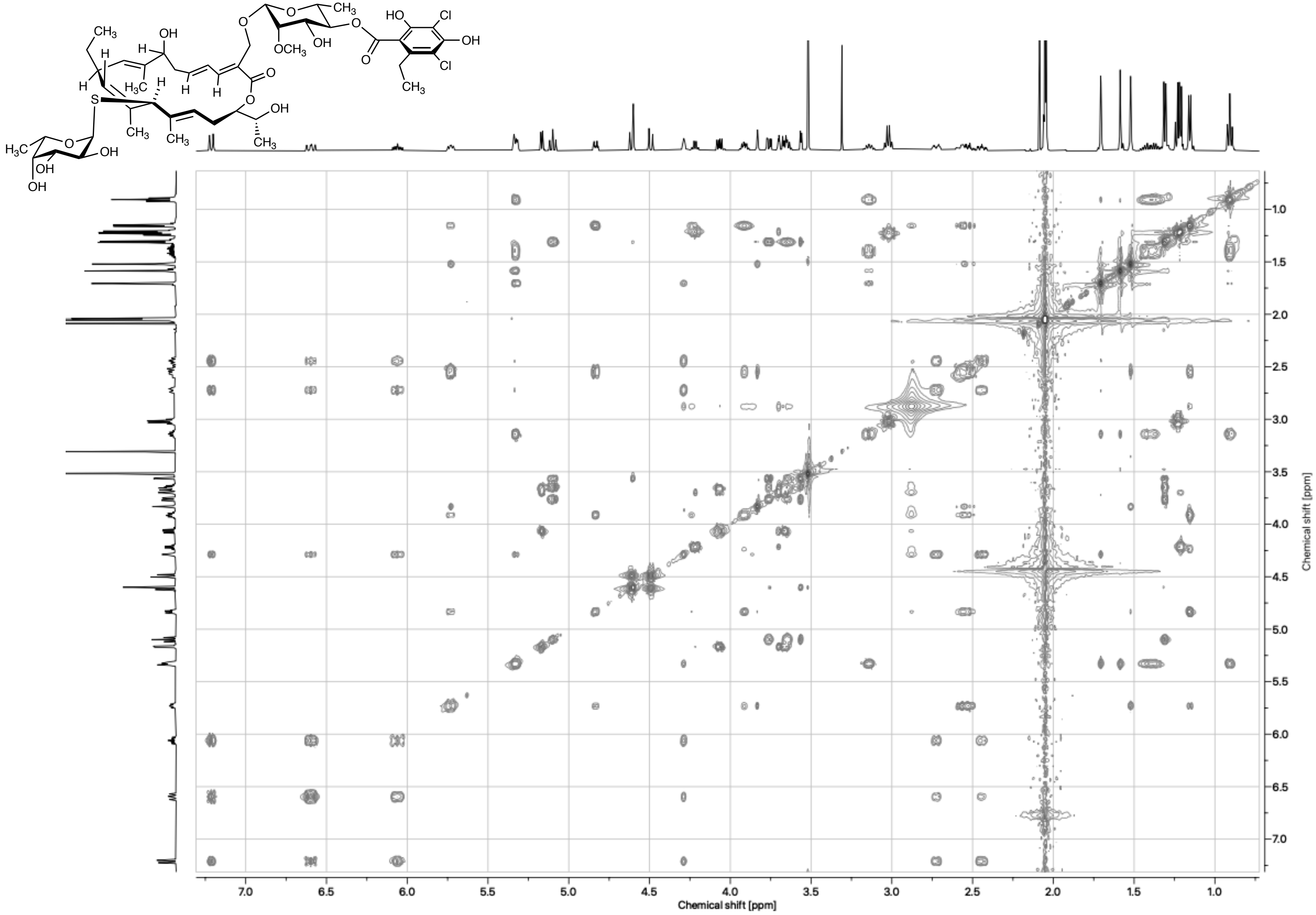

Figure 121: TOCSY spectrum of 11-desnoviosyl-13-thio−α-L-fucosyl fidaxomicin (18c-C(13)) in acetone-d_6

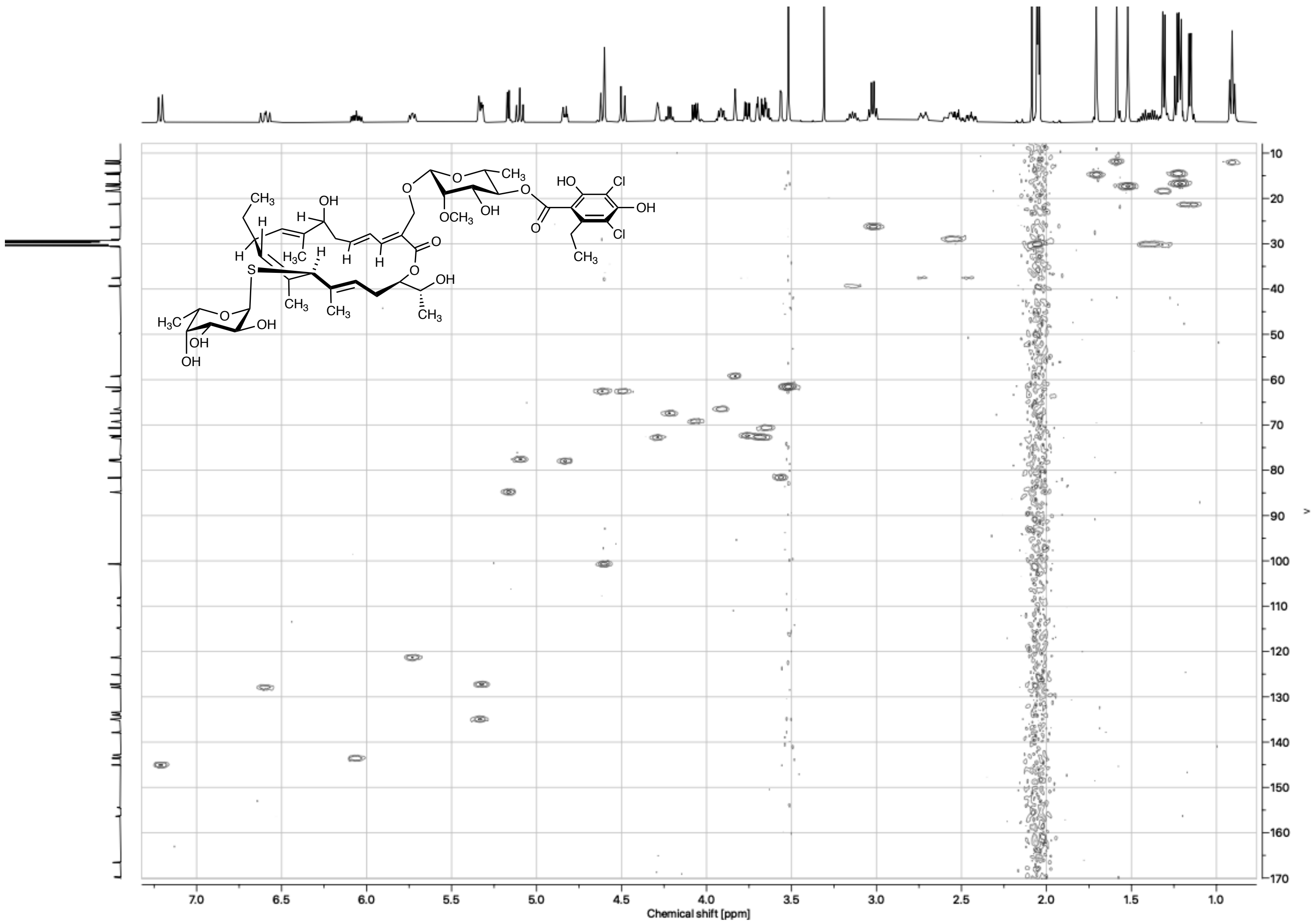

Figure 122: HSQC spectrum of 11-desnoviosyl-13-thio-α-L-fucosyl fidaxomicin (18c-C(13)) in acetone-d_6

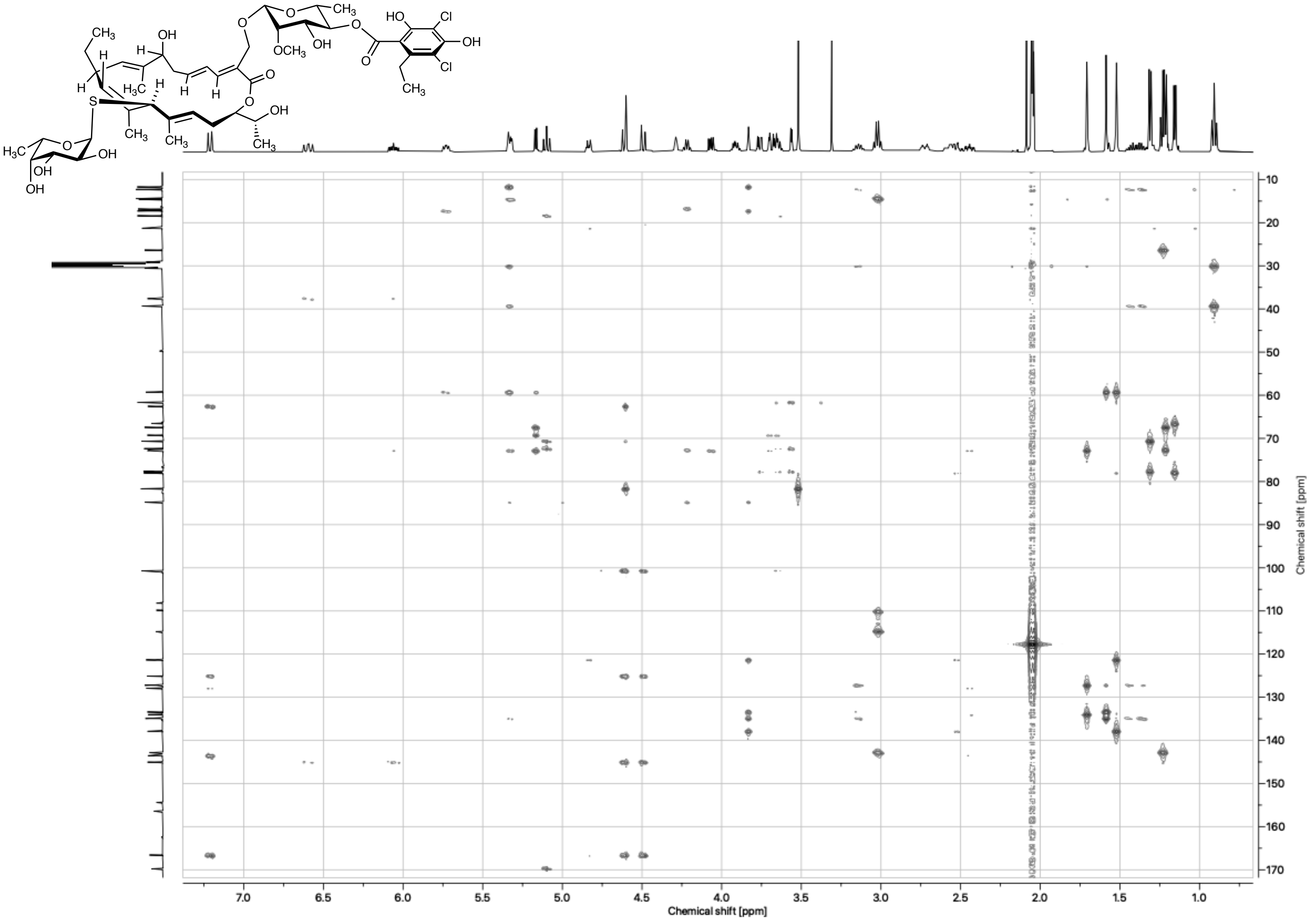

Figure 123: HMBC spectrum of 11-desnoviosyl-13-thio-α-L-fucosyl fidaxomicin (18c-C(13)) in acetone-d_6

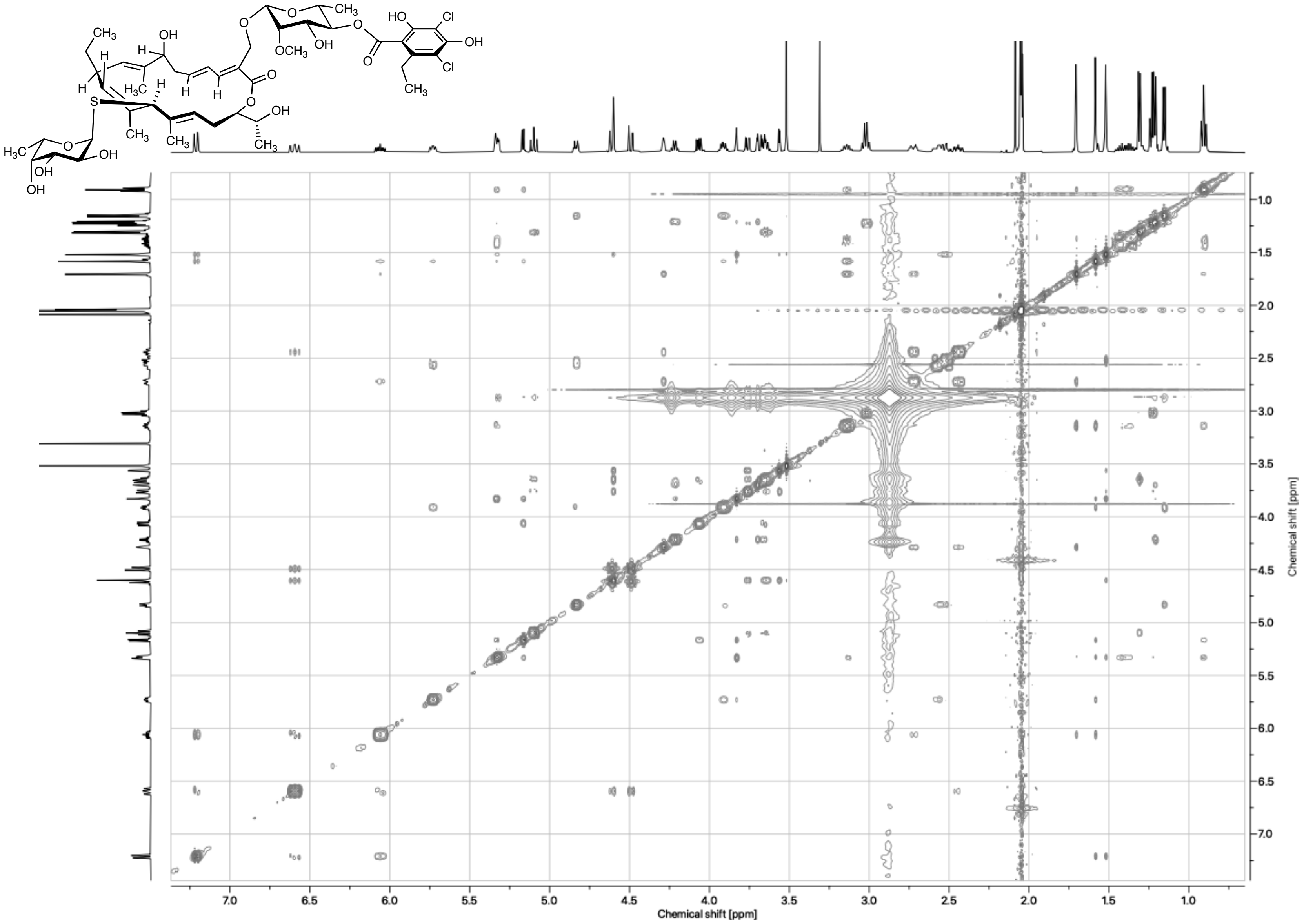

Figure 124: NOESY spectrum of 11-desnoviosyl-13-thio-α-L-fucosyl fidaxomicin (18c-C(13)) in acetone-d_6

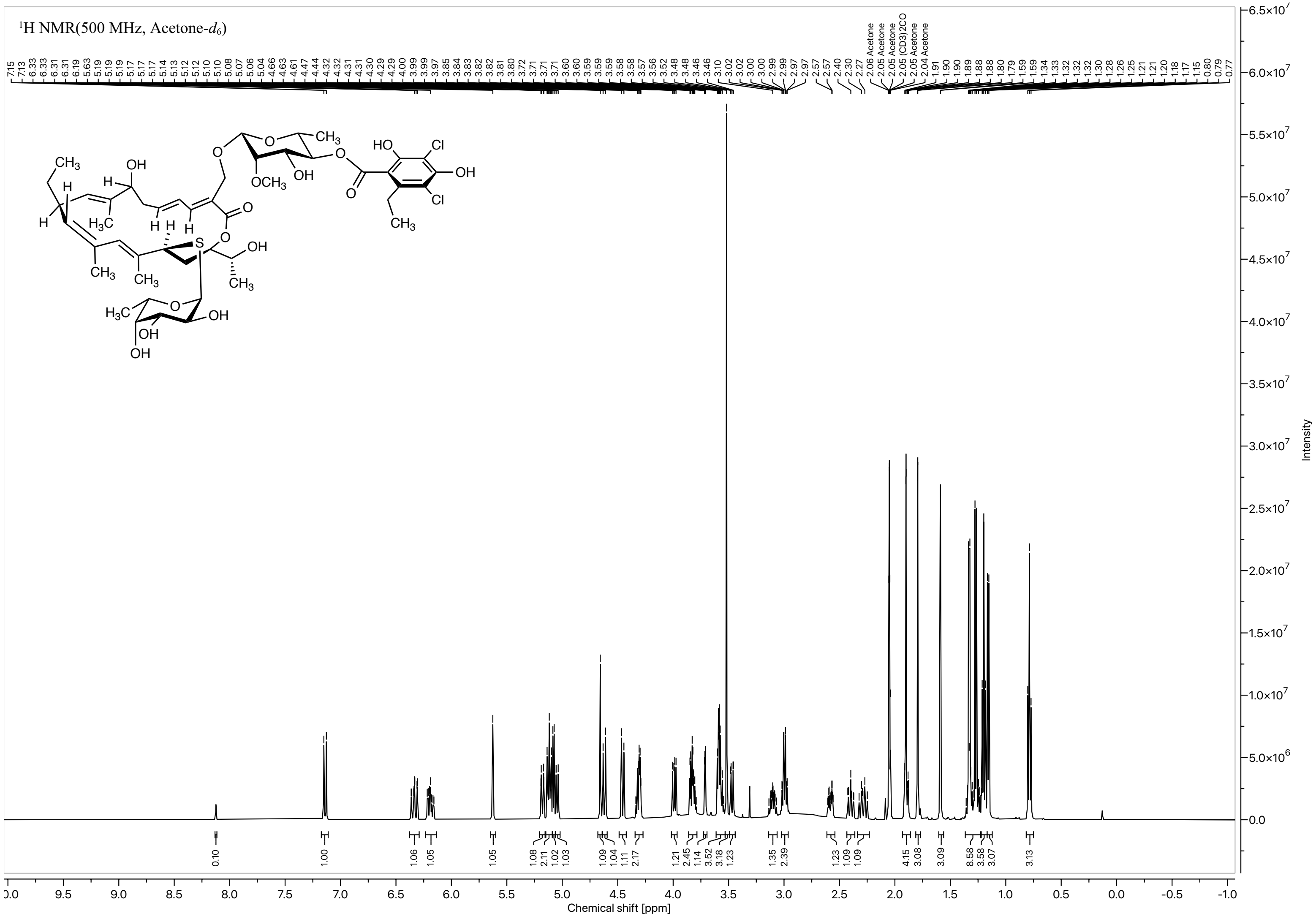

Figure 125: ^{1}H NMR spectrum of 11-desnoviosyl-15-thio-α-L-fucosyl fidaxomicin (18c-C(15)) in acetone-d_6

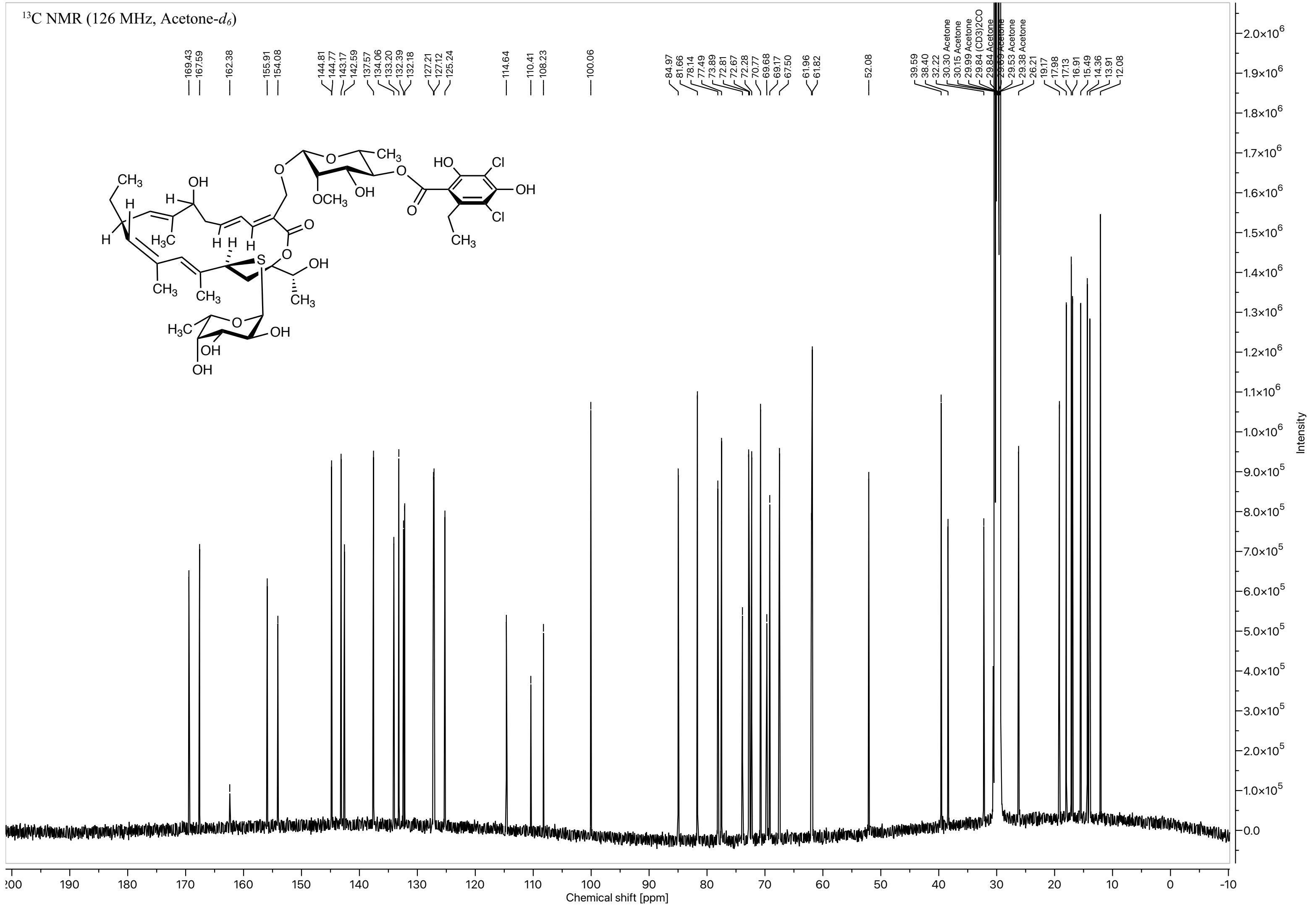

Figure 126: ^{13}C NMR spectrum of 11-desnoviosyl-15-thio-α-L-fucosyl fidaxomicin (18c-C(15)) in acetone-d_6

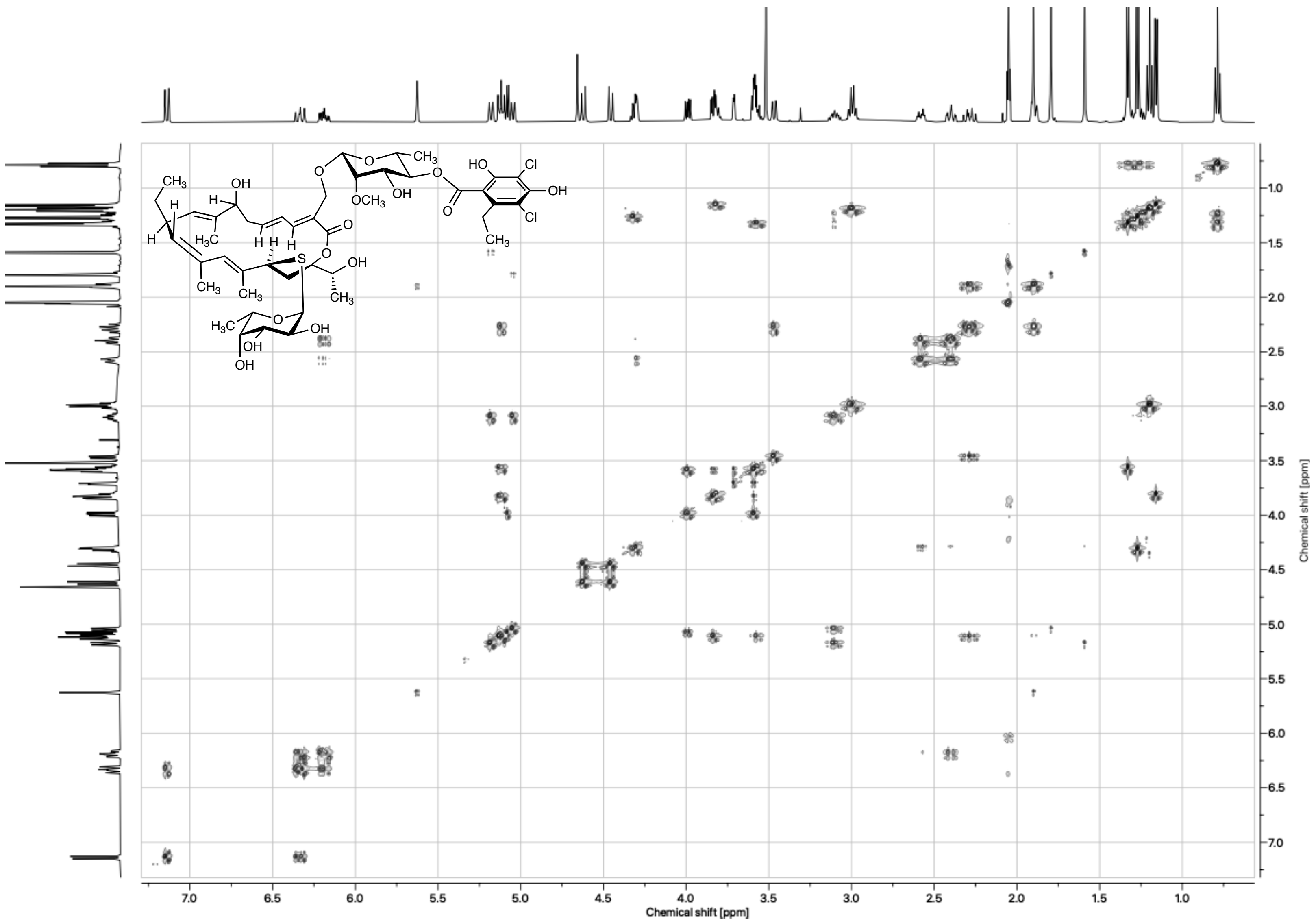

Figure 127: COSY spectrum of 11-desnoviosyl-15-thio- α-L-fucosyl fidaxomicin (18c-C(15)) in acetone-d_6

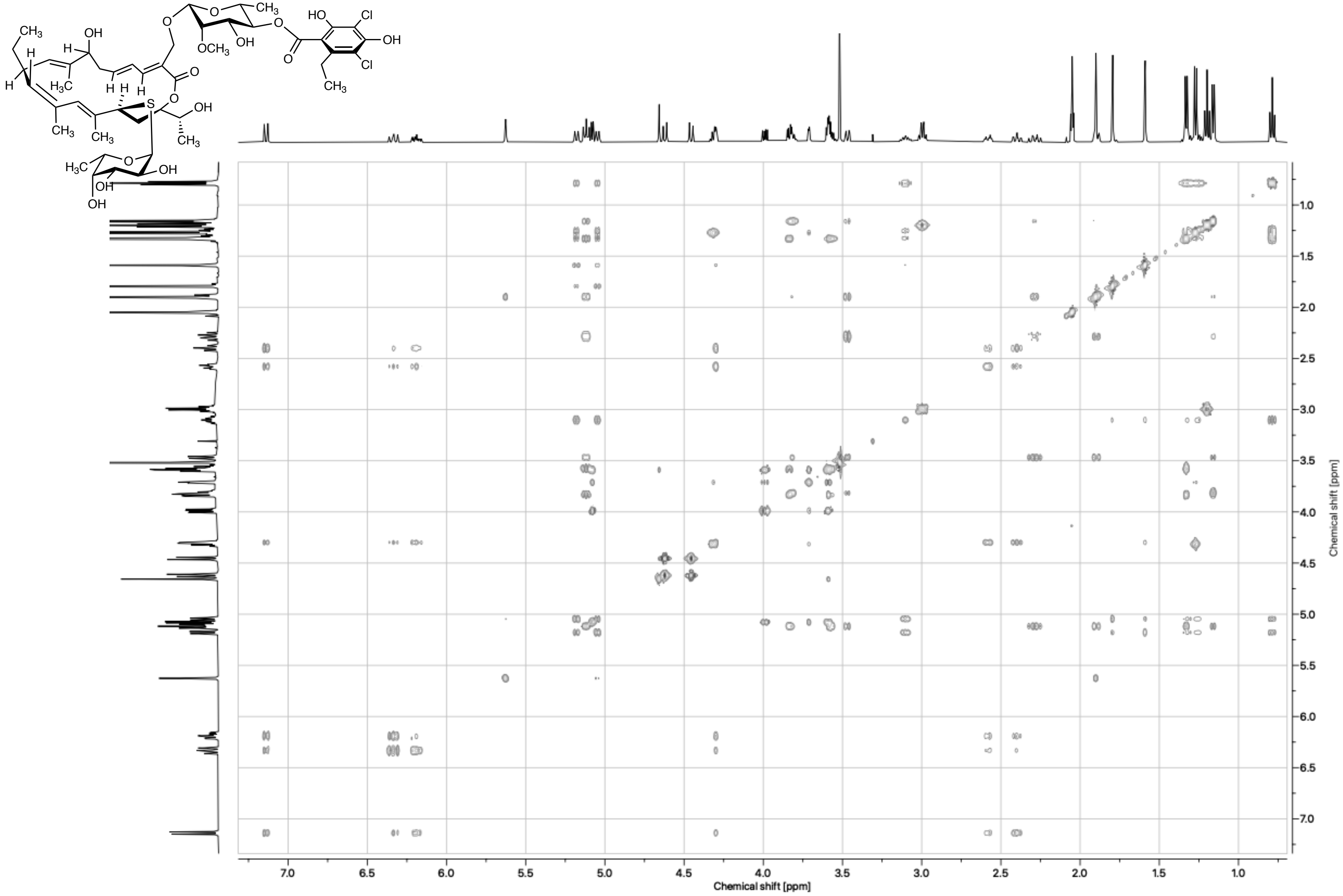

Figure 128: TOCSY spectrum of 11-desnoviosyl-15-thio- α-L-fucosyl fidaxomicin (18c-C(15)) in acetone-d_6

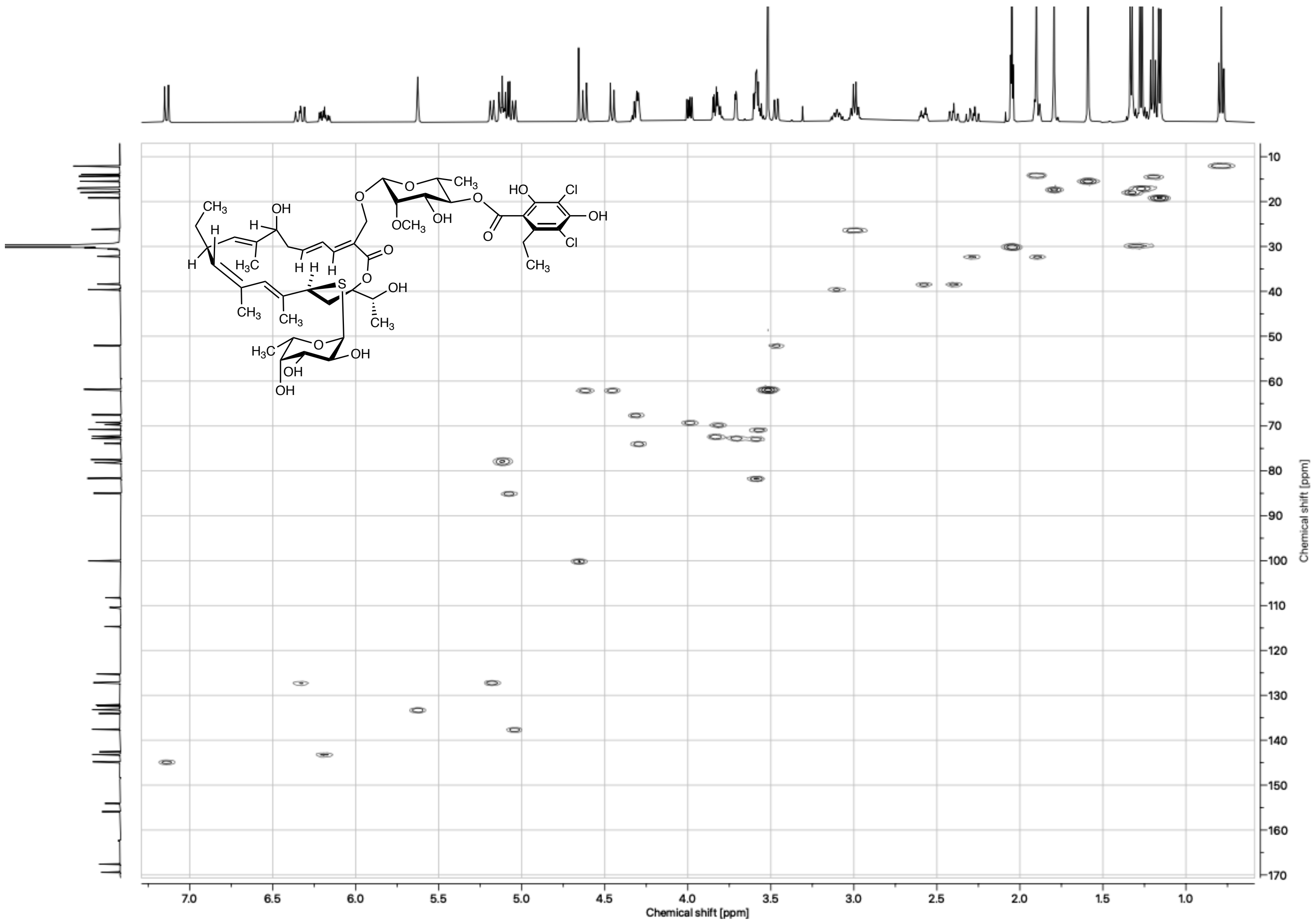

Figure 129: HSQC spectrum of 11-desnoviosyl-15-thio-α-L-fucosyl fidaxomicin (18c-C(15)) in acetone-d_6

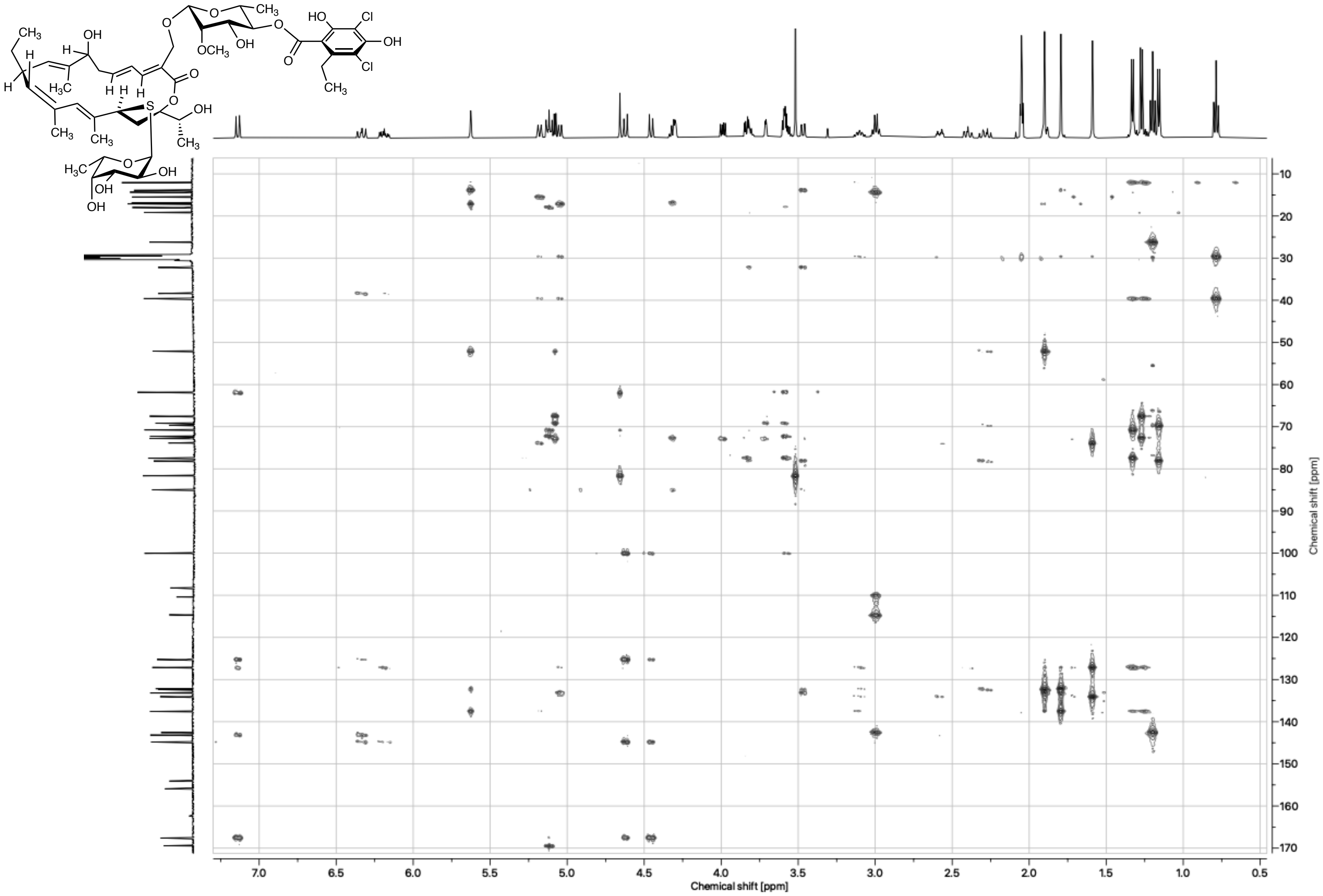

Figure 130: HMBC spectrum of 11-desnoviosyl-15-thio-α-L-fucosyl fidaxomicin (18c-C(15)) in acetone-d_6

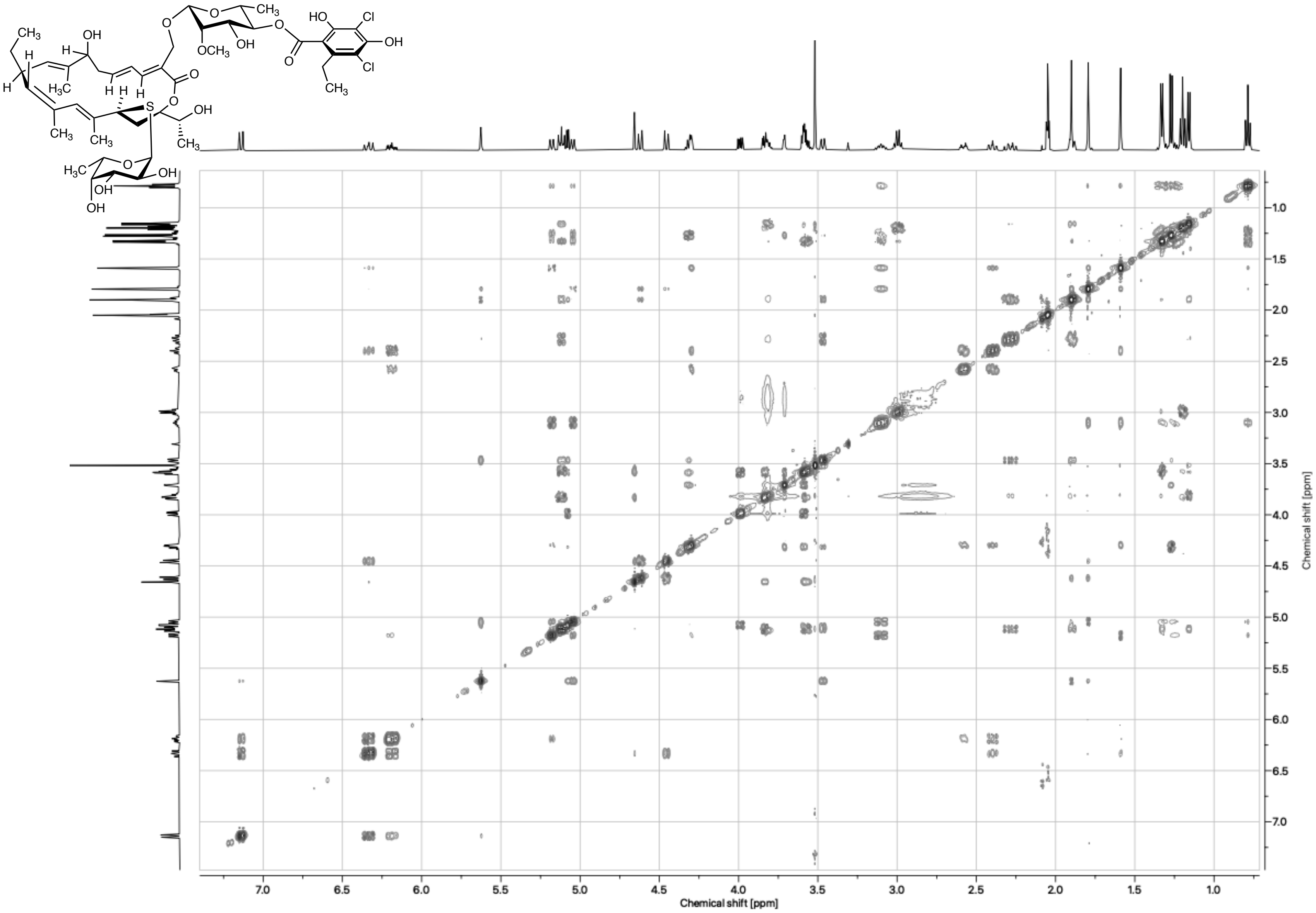

Figure 131: NOESY spectrum of 11-desnoviosyl-15-thio-α-L-fucosyl fidaxomicin (18c-C(15)) in acetone-d_6

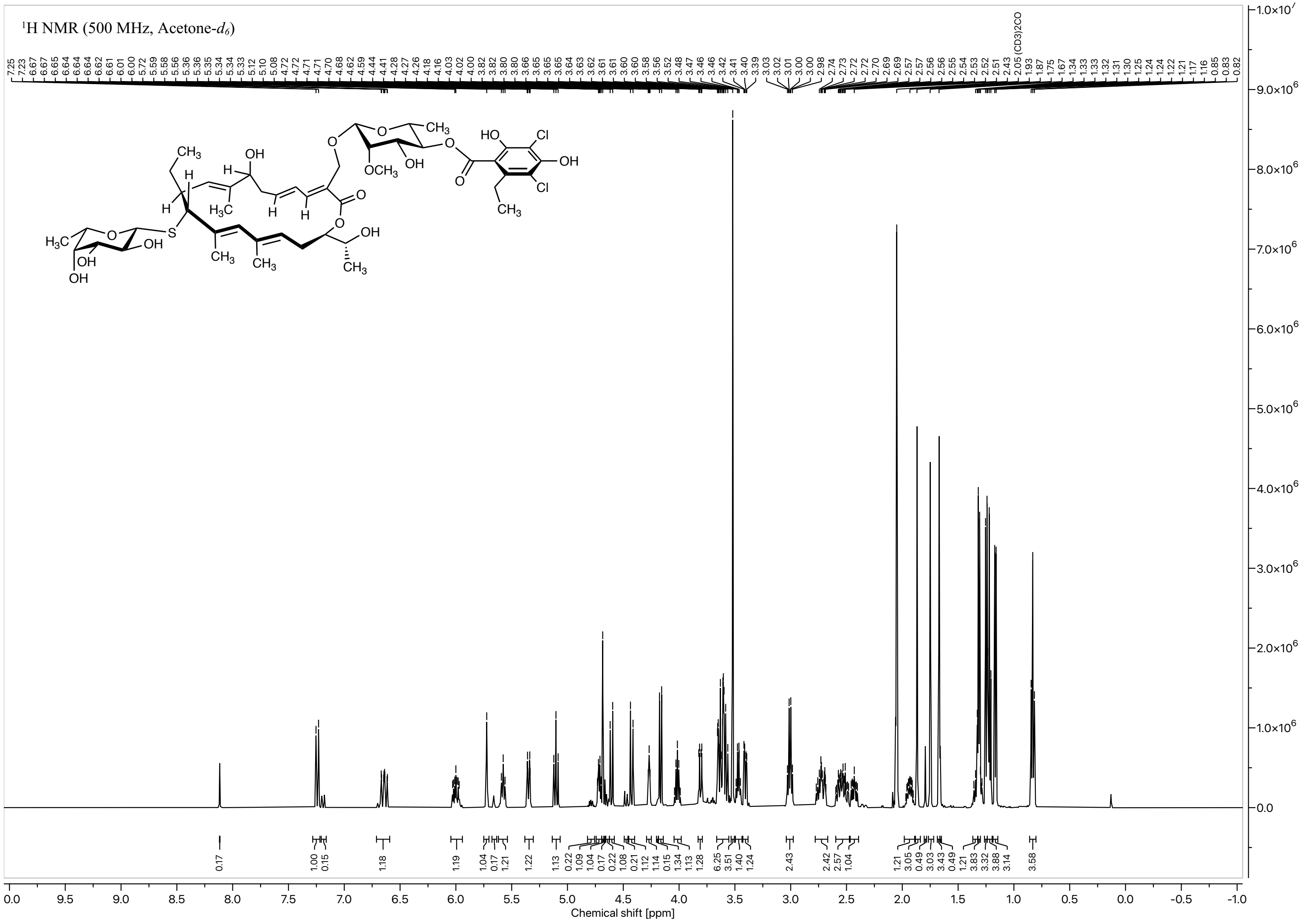

Figure 132: ^{1}H NMR spectrum of 11-desnoviosyl-11-thio-β-L-fucosyl fidaxomicin (18d-C(11)) in acetone-d_6

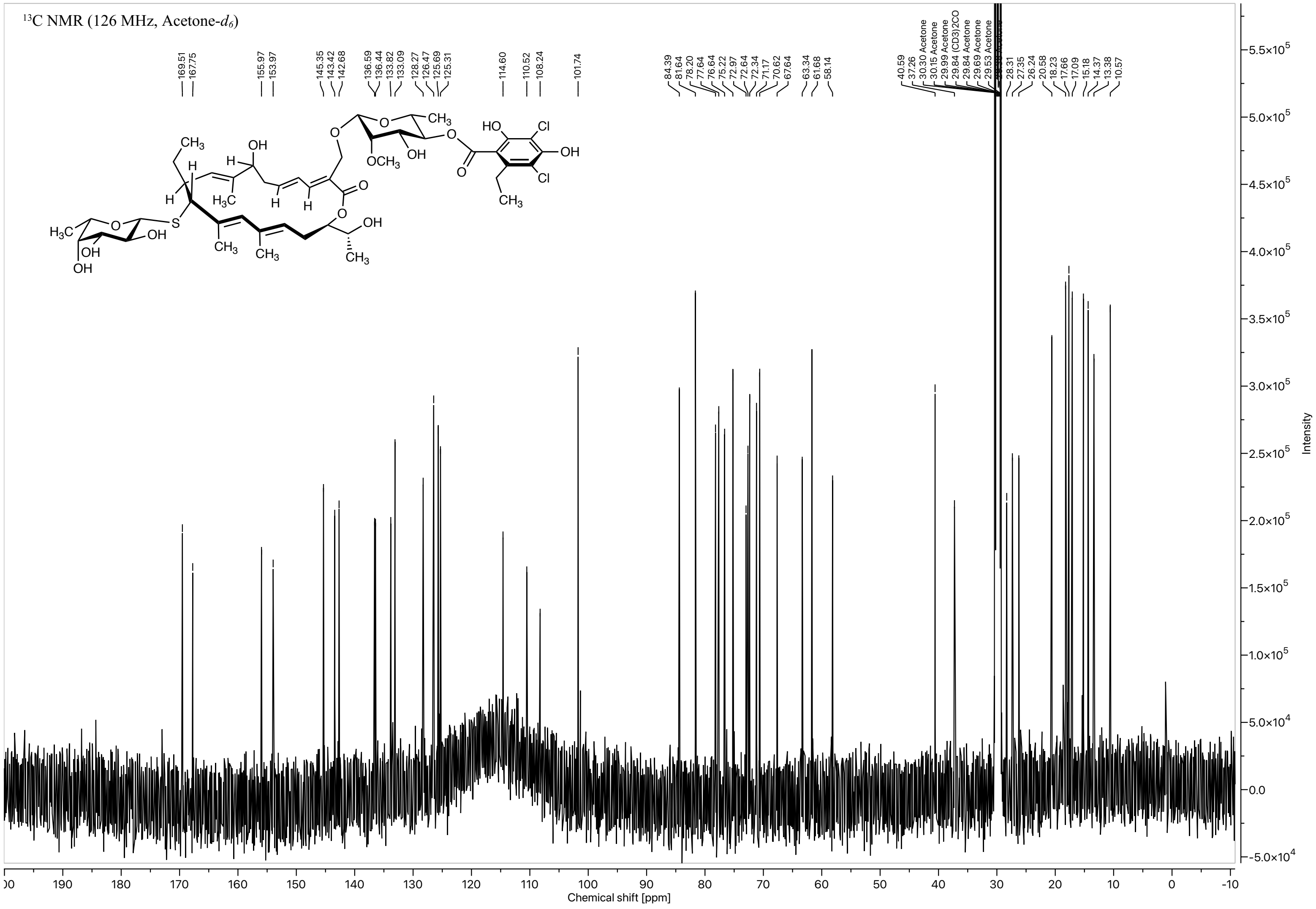

Figure 133: ^{13}C NMR spectrum of 11-desnoviosyl-11-thio-β-L-fucosyl fidaxomicin (18d-C(11)) in acetone-d_6

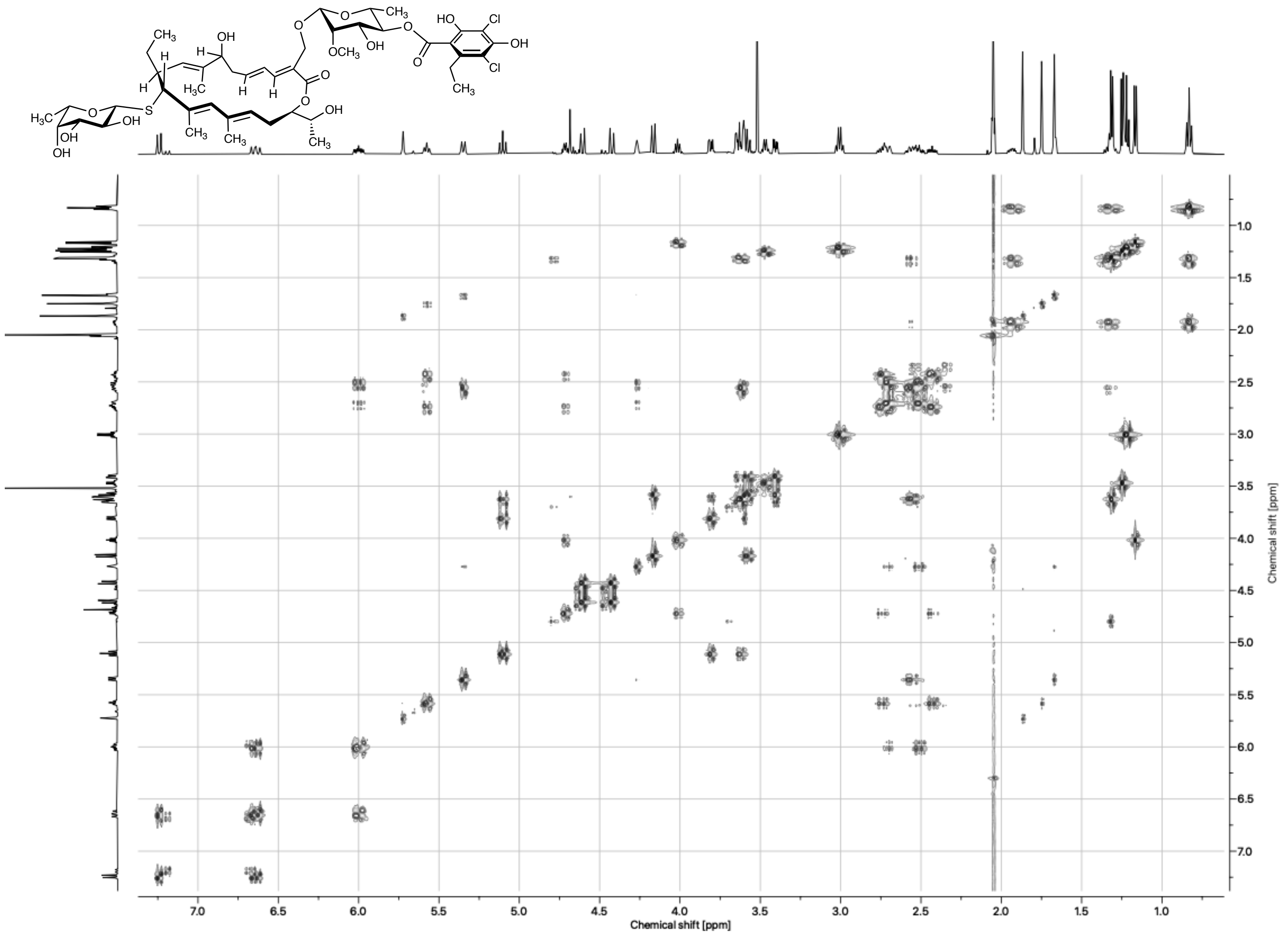

Figure 134: COSY spectrum of 11-desnoviosyl-11-thio-β-L-fucosyl fidaxomicin (18d-C(11)) in acetone-d_6

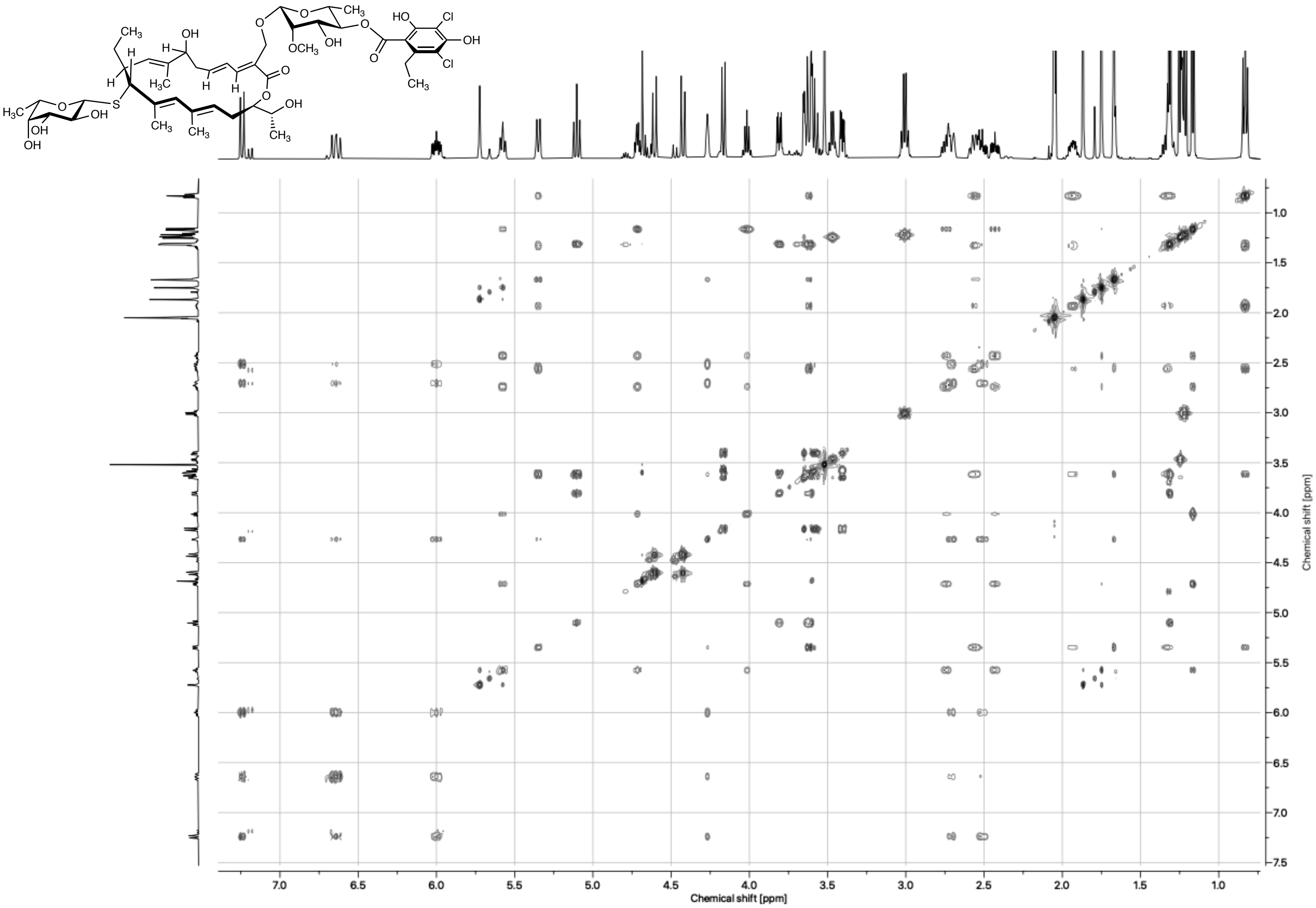

Figure 135: TOCSY spectrum of 11-desnoviosyl-11-thio-β-L-fucosyl fidaxomicin (18d-C(11)) in acetone-d_6

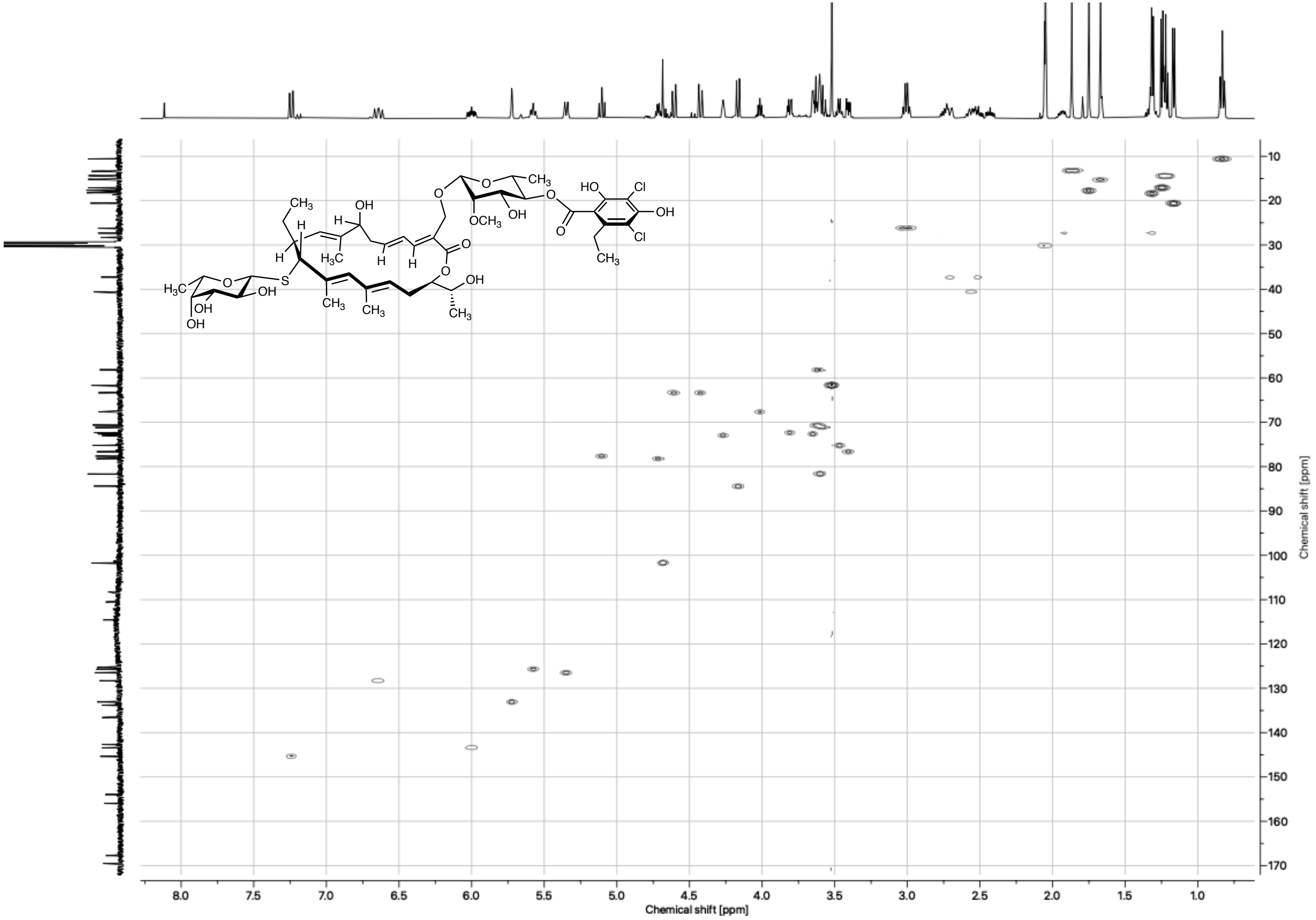

Figure 136: HSQC spectrum of 11-desnoviosyl-11-thio-β-L-fucosyl fidaxomicin (18d-C(11)) in acetone-d_6

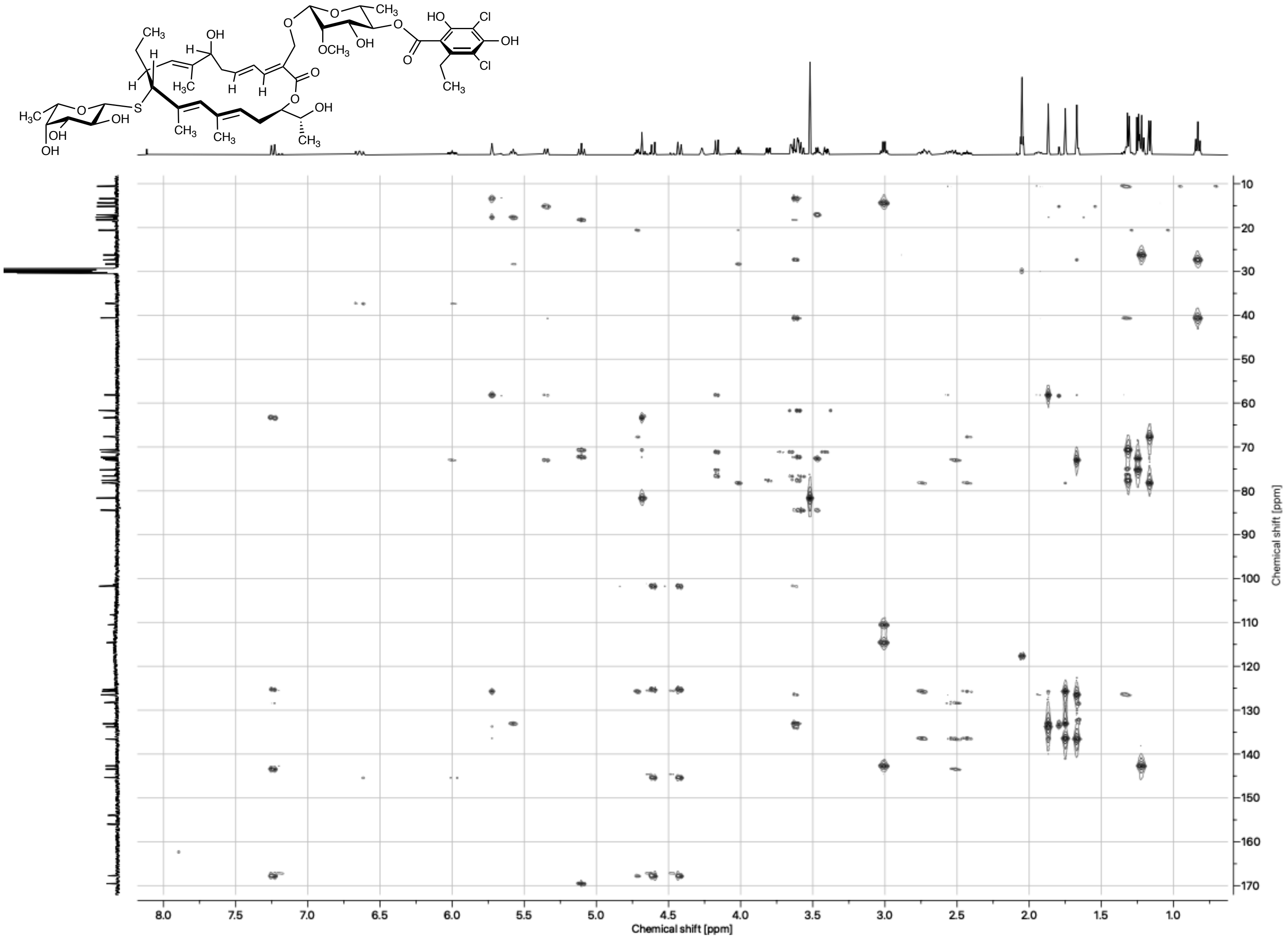

Figure 137: HMBC spectrum of 11-desnoviosyl-11-thio-β-L-fucosyl fidaxomicin (18d-C(11)) in acetone-d_6

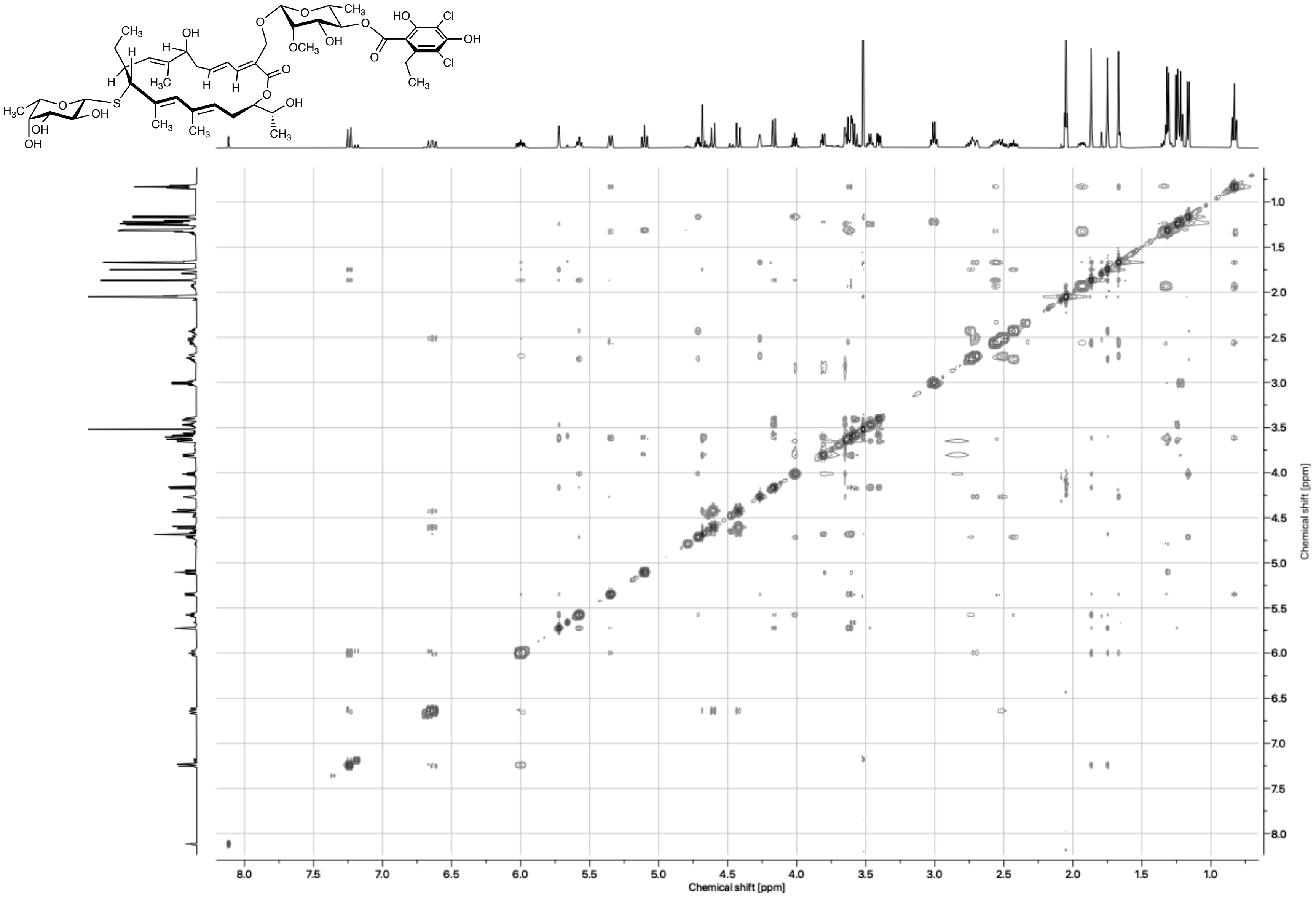

Figure 138: NOESY spectrum of 11-desnoviosyl-11-thio-β-L-fucosyl fidaxomicin (18d-C(11)) in acetone-d_6

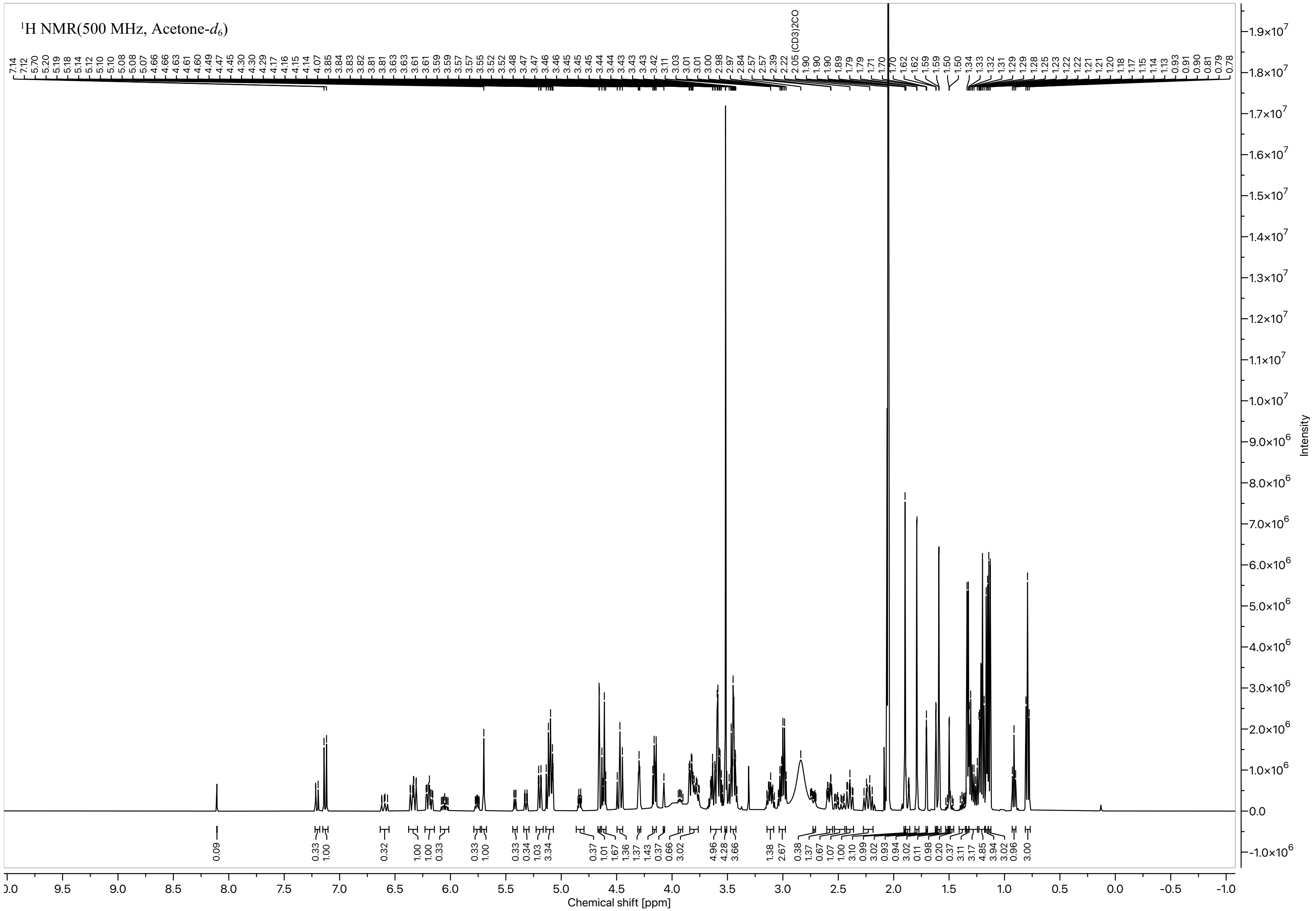

Figure 139: ^{1}H NMR spectrum of 11-desnoviosyl-13/15-thio-β-L-fucosyl fidaxomicin (18d-C(13)+C(15)) in acetone-d_6 (mixture of regioisomers)

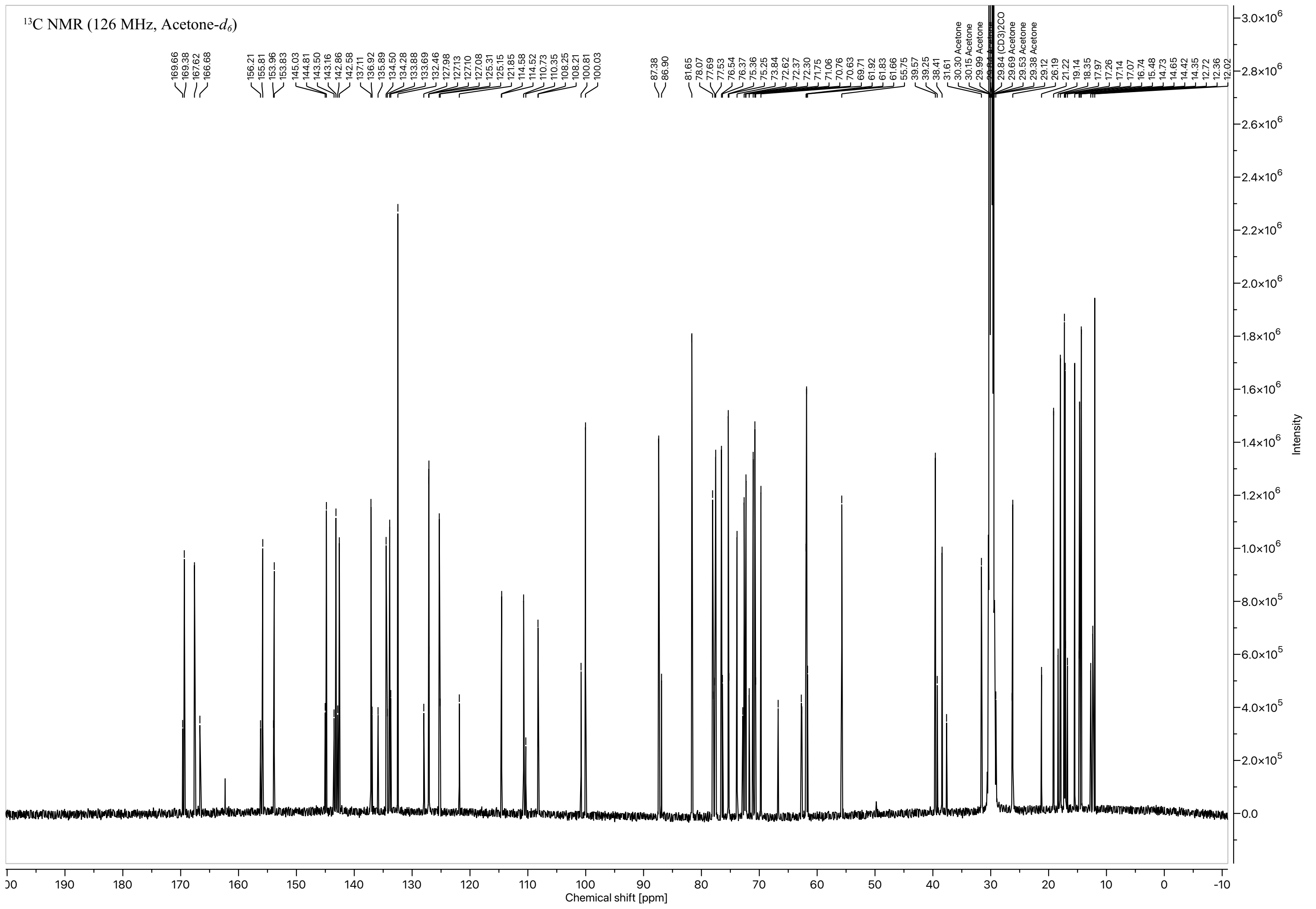

Figure 140: ^{13}C NMR spectrum of 11-desnoviosyl-13/15-thio-β-L-fucosyl fidaxomicin (18d-C(13)+C(15)) in acetone-d_6 (mixture of regioisomers)

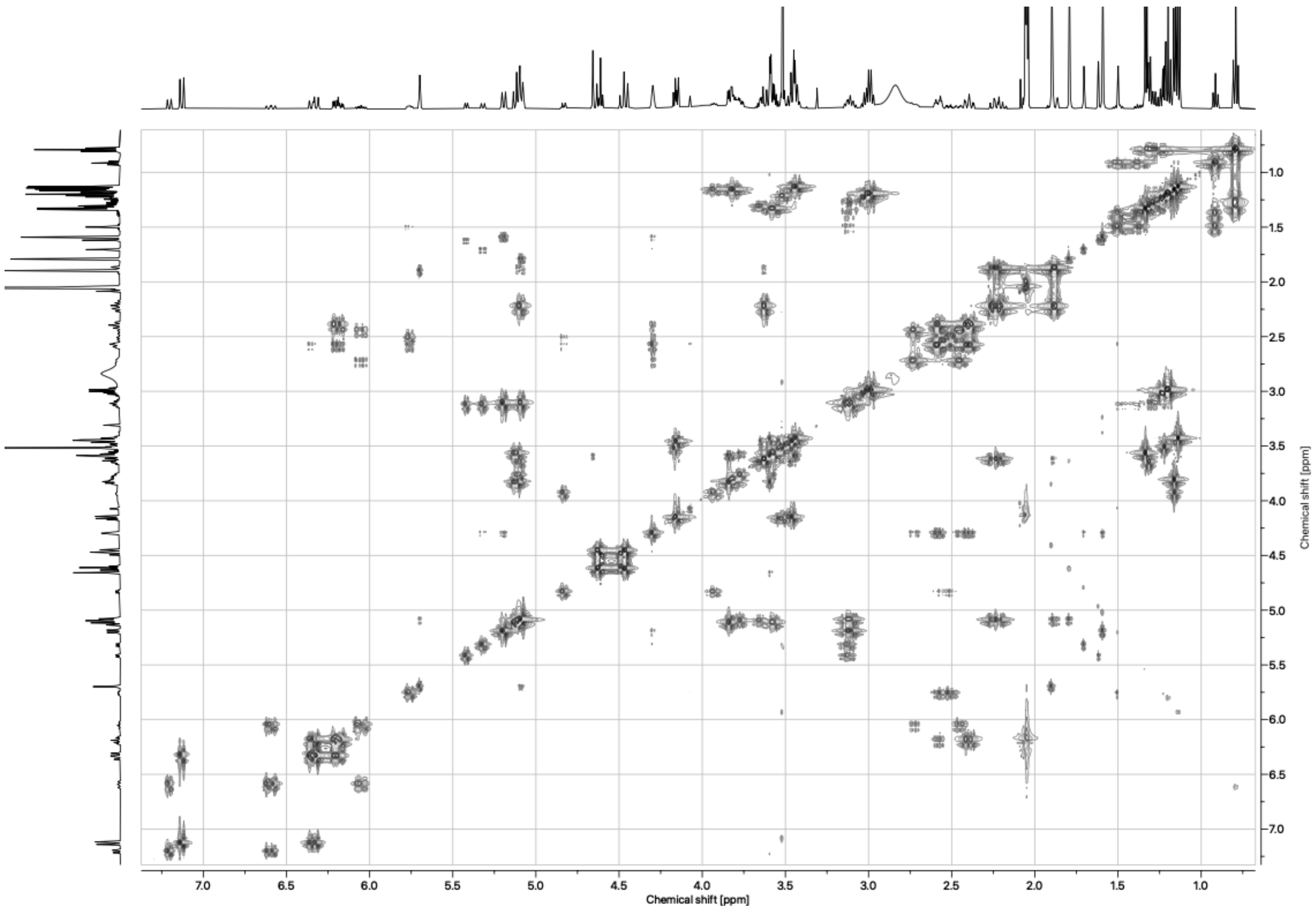

Figure 141: COSY spectrum of 11-desnoviosyl-13/15-thio-β-L-fucosyl fidaxomicin (18d-C(13)+C(15)) in acetone-d_6 (mixture of regioisomers)

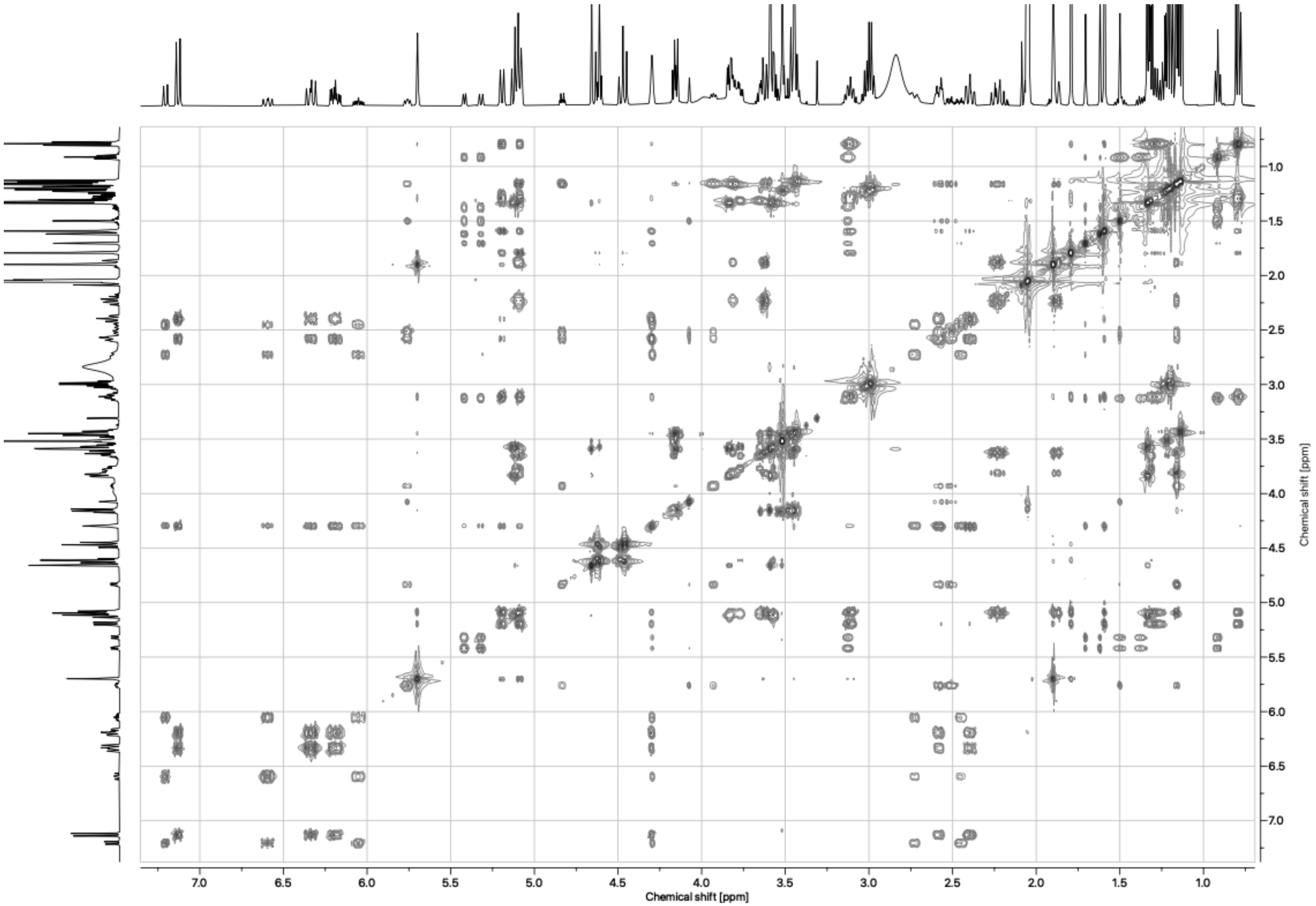

Figure 142: TOCSY spectrum of 11-desnoviosyl-13/15-thio-β-L-fucosyl fidaxomicin (18d-C(13)+C(15)) in acetone-d_6 (mixture of regioisomers)

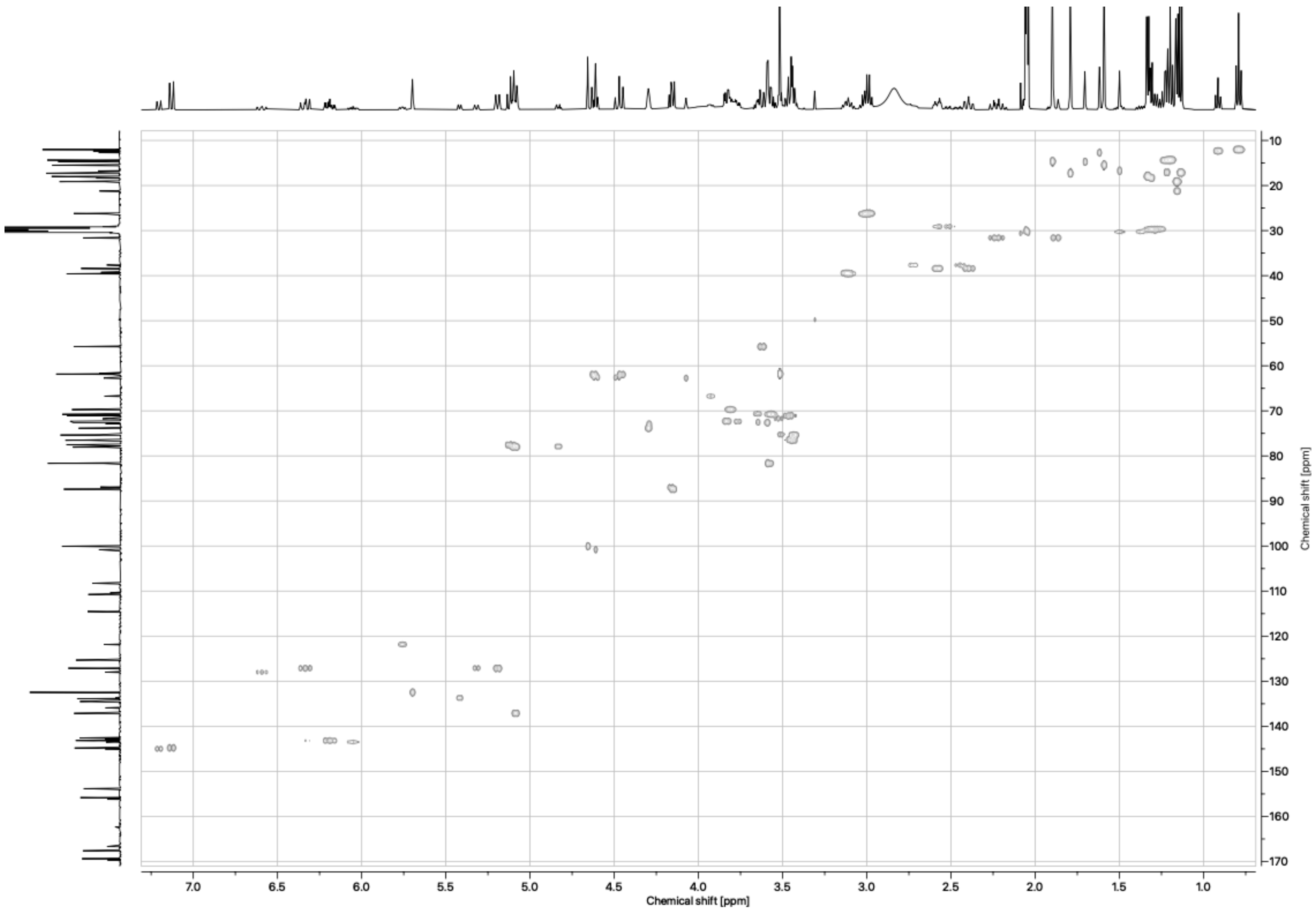

Figure 143: HSQC spectrum of 11-desnoviosyl-13/15-thio-β-L-fucosyl fidaxomicin (18d-C(13)+C(15)) in acetone-d_6 (mixture of regioisomers)

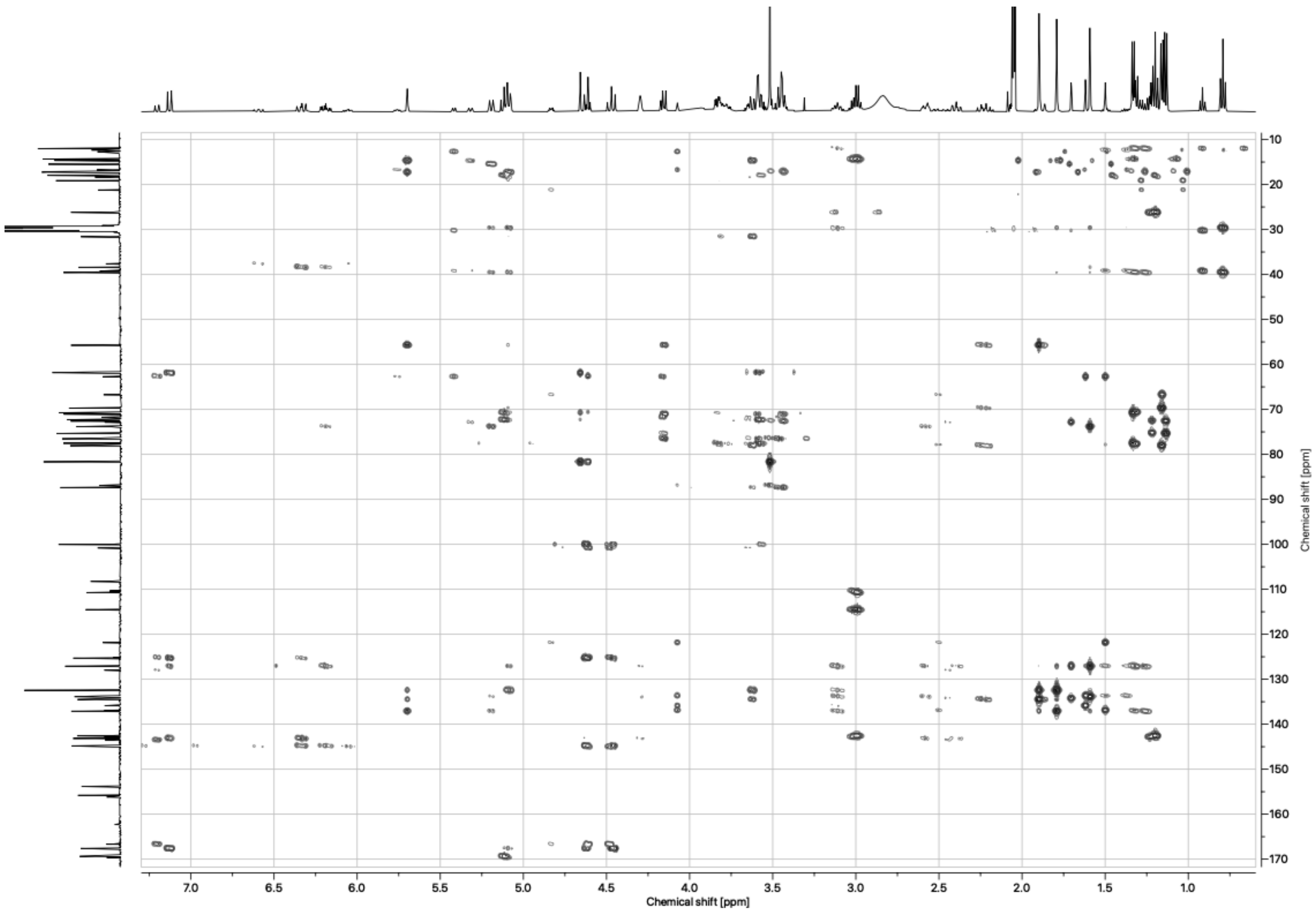

Figure 144: HMBC spectrum of 11-desnoviosyl-13/15-thio-β-L-fucosyl fidaxomicin (18d-C(13)+C(15)) in acetone-d_6 (mixture of regioisomers)

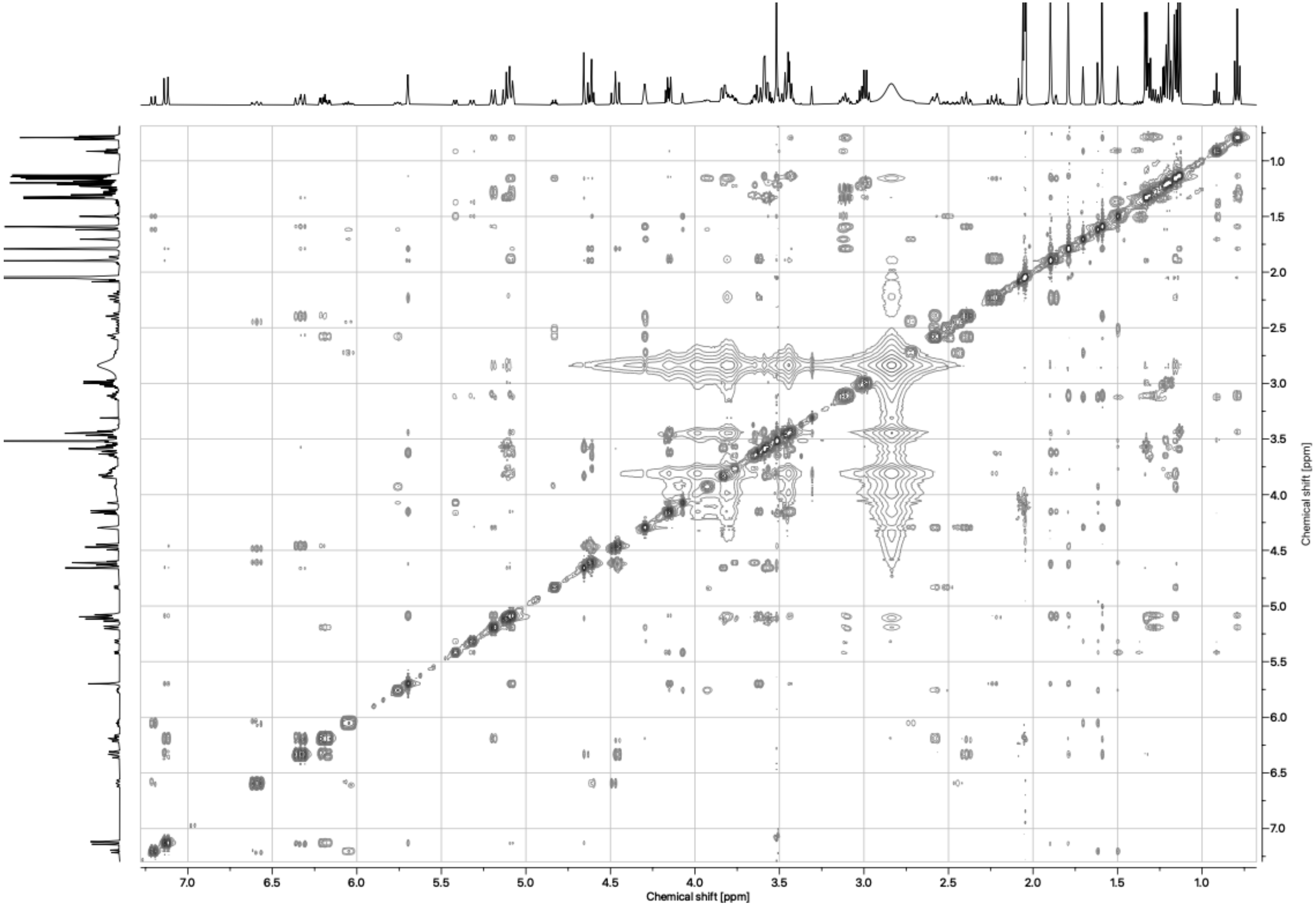

Figure 145: NOESY spectrum of 11-desnoviosyl-13/15-thio-β-L-fucosyl fidaxomicin (18d-C(13)+C(15)) in acetone-d_6 (mixture of regioisomers)

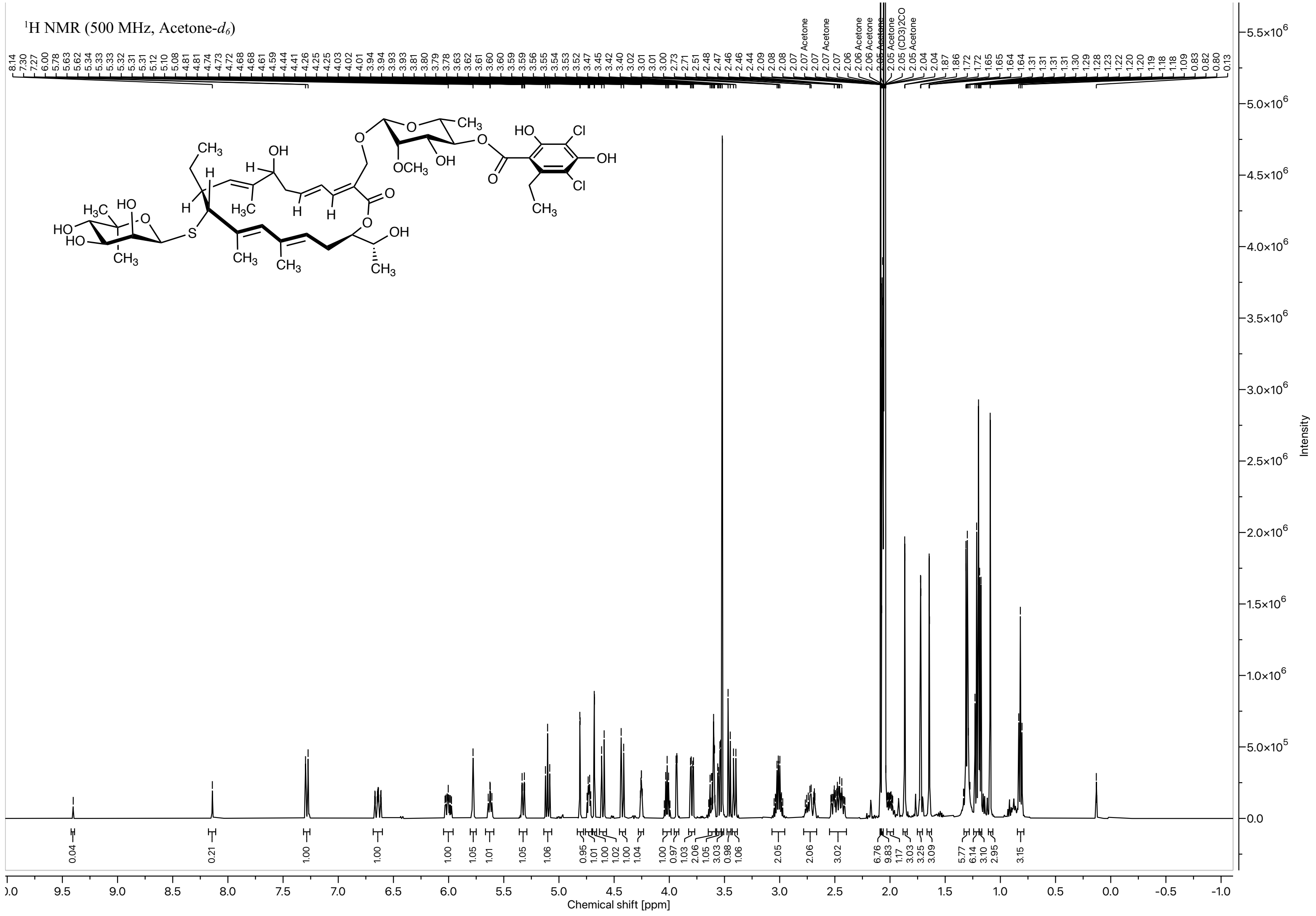

Figure 146: ^{1}H NMR spectrum of 11-desnoviosyl-11-thio-(4”-desbutyryl)-β-D-noviosyl fidaxomicin (S-OP1118, 18e-C(11)) in acetone-d_6

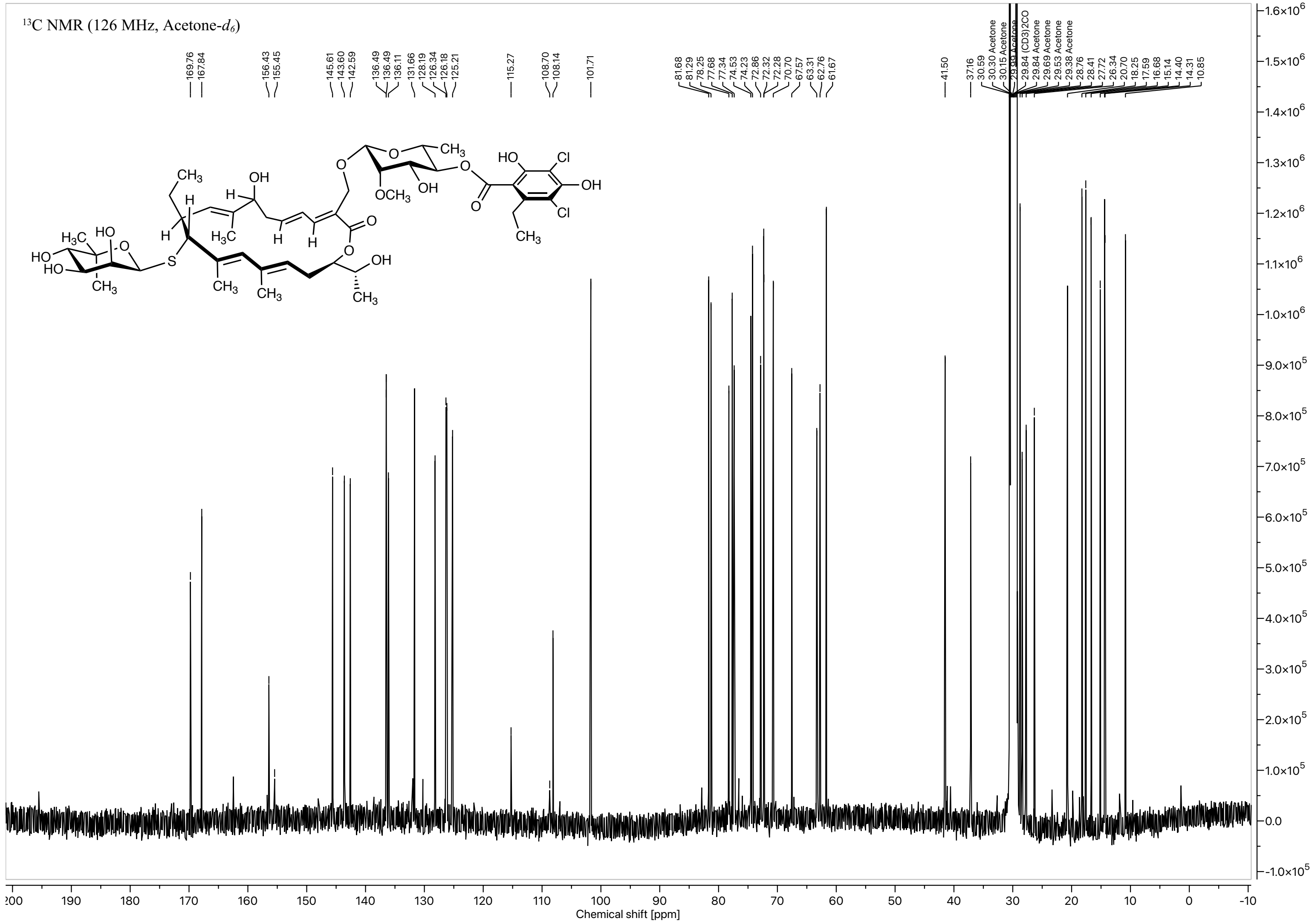

Figure 147: ^{13}C NMR spectrum of 11-desnoviosyl-11-thio-(4"-desbutyryl)-β-D-noviosyl fidaxomicin (S-OP1118, 18e-C(11)) in acetone-d_6

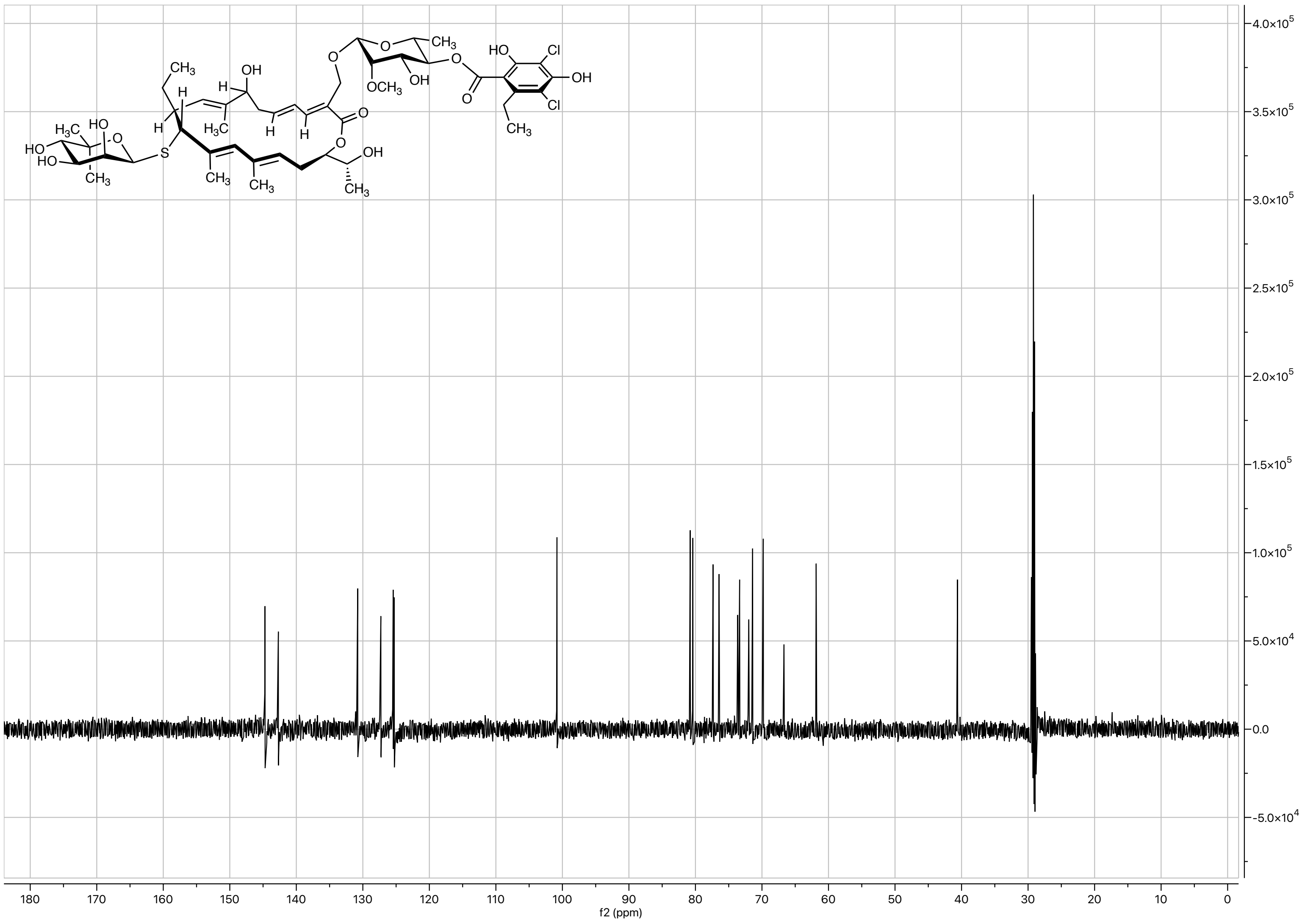

Figure 148: DEPT90 spectrum of 11-desnoviosyl-11-thio-(4”-desbutyryl)-β-D-noviosyl fidaxomicin (S-OP1118, 18e-C(11)) in acetone-d_6

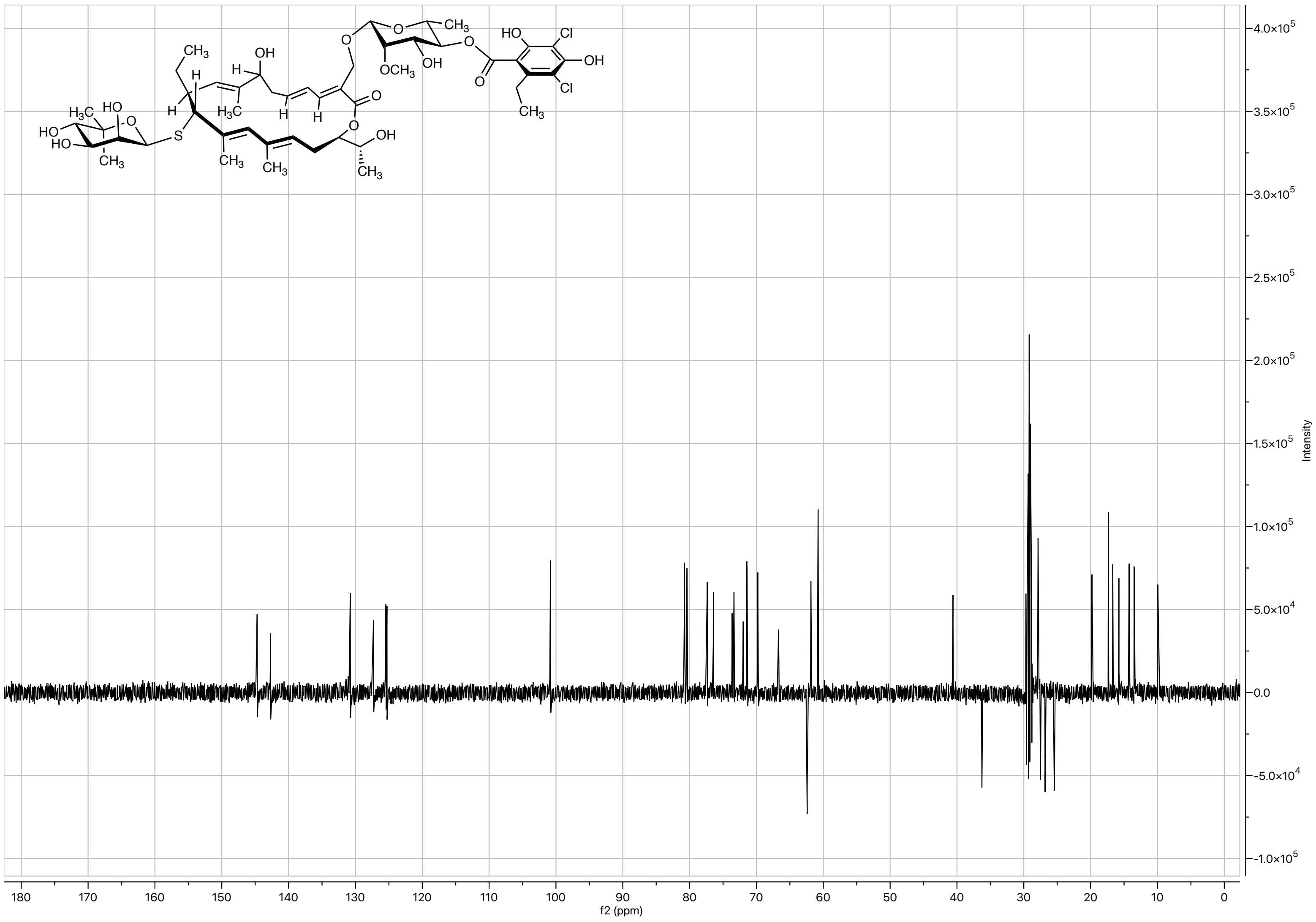

Figure 149: DEPT135 spectrum of 11-desnoviosyl-11-thio-(4”-desbutyryl)-β-D-noviosyl fidaxomicin (S-OP1118, 18e-C(11)) in acetone-d_6

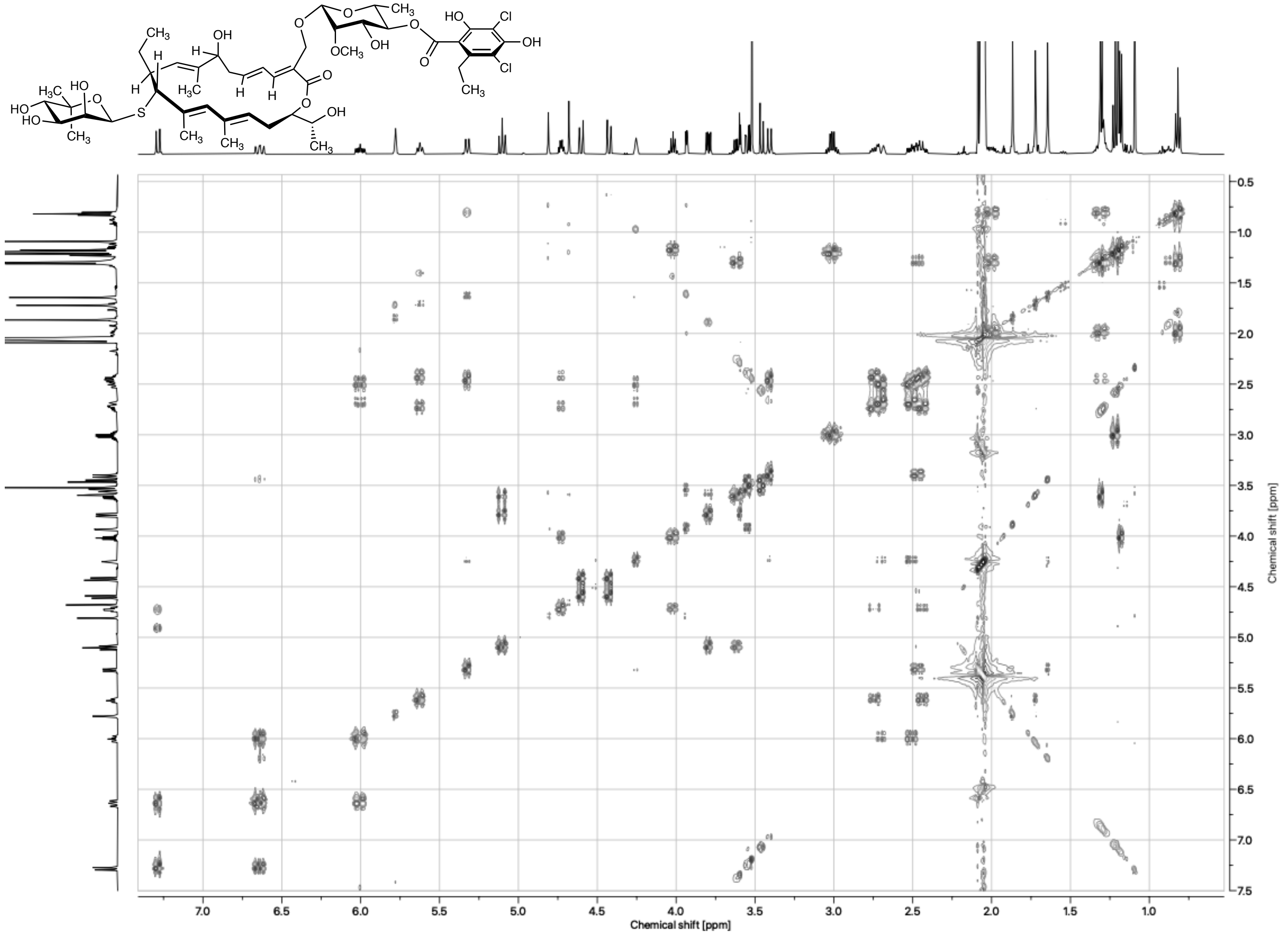

Figure 150: COSY spectrum of 11-desnoviosyl-11-thio-(4”-desbutyryl)-β-D-noviosyl fidaxomicin (S-OP1118, 18e-C(11)) in acetone-d_6

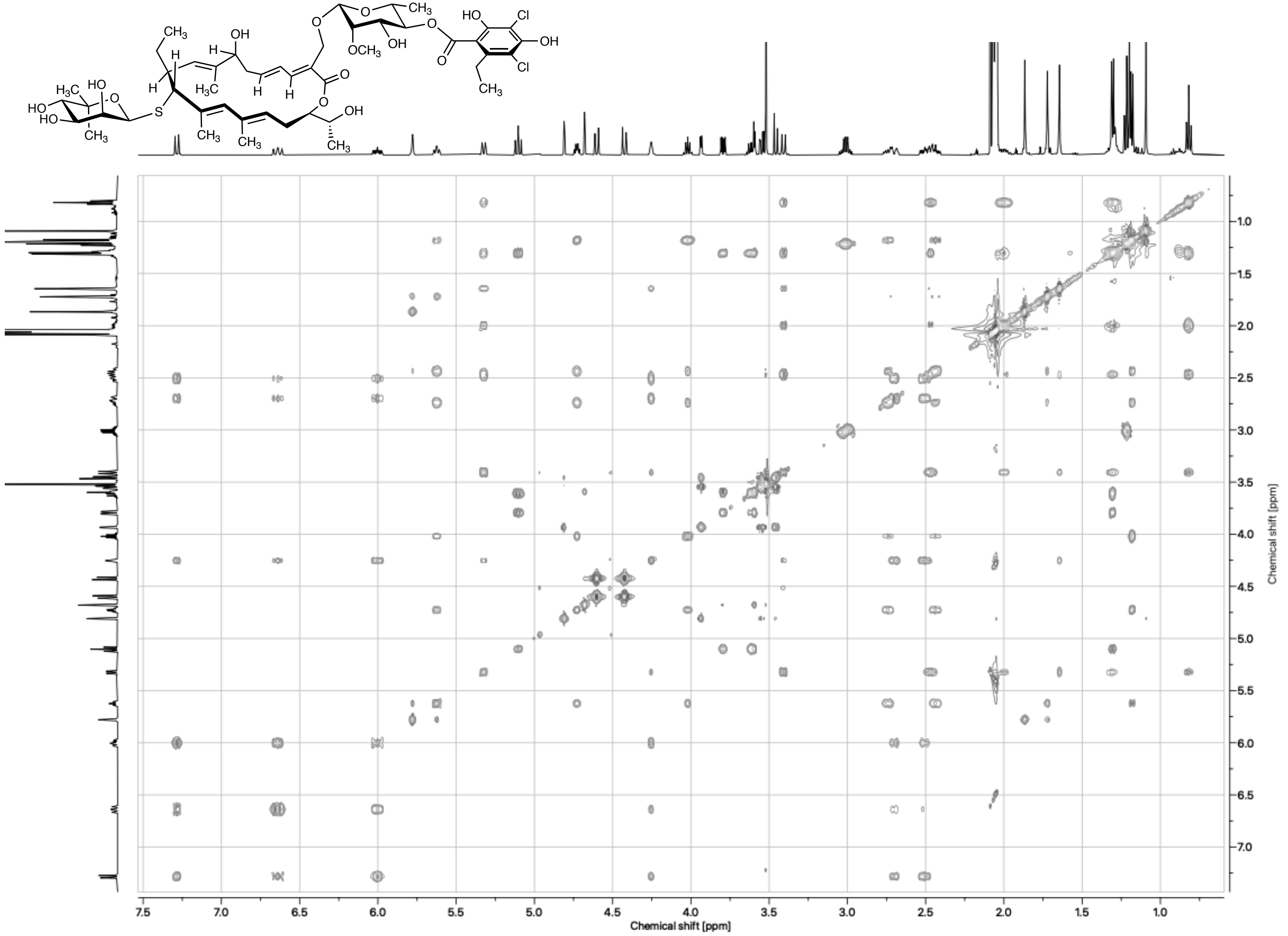

Figure 151: TOCSY spectrum of 11-desnoviosyl-11-thio-(4"-desbutyryl)-β-D-noviosyl fidaxomicin (S-OP1118, 18e-C(11)) in acetone-d_6

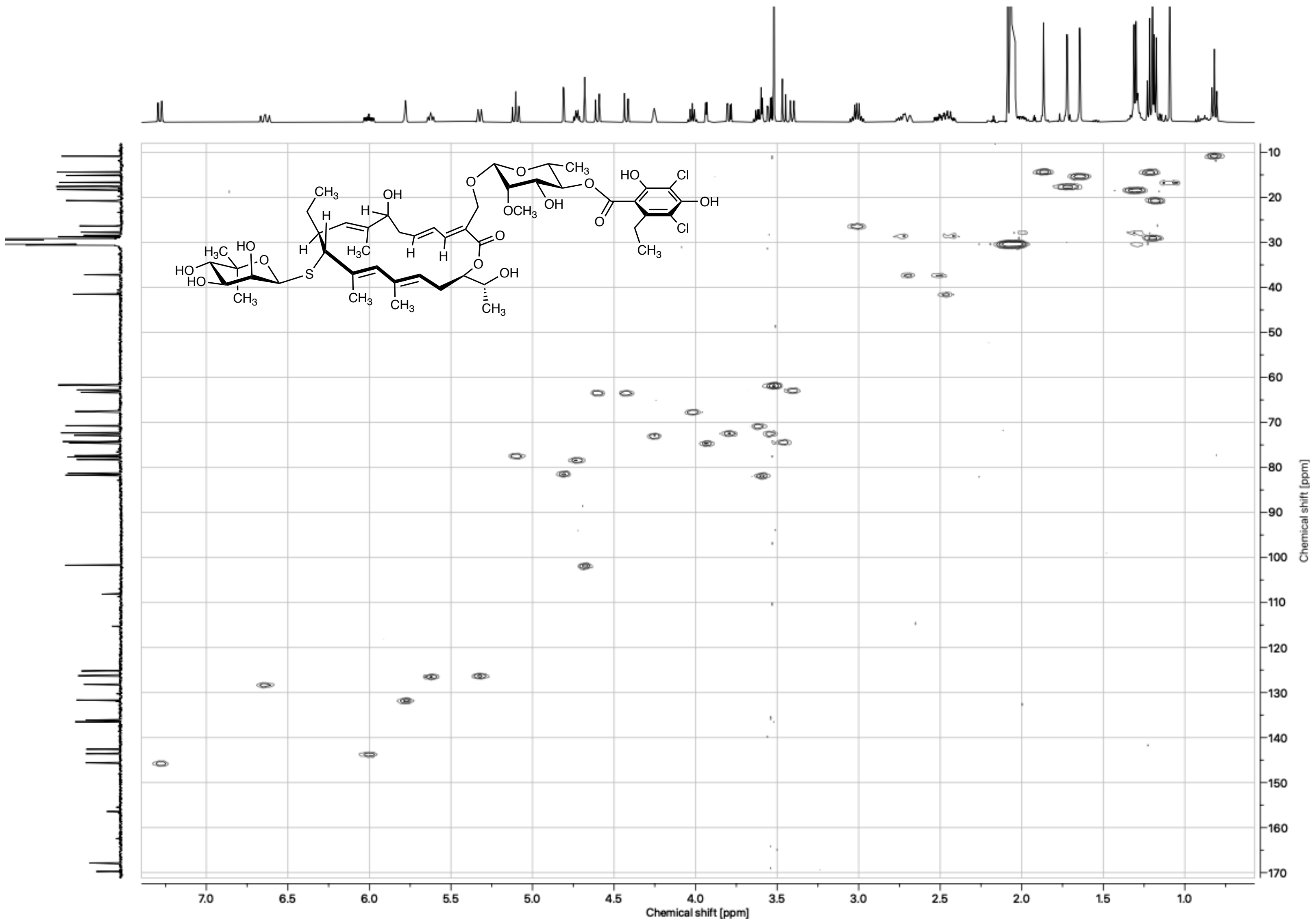

Figure 152: HSQC spectrum of 11-desnoviosyl-11-thio-(4”-desbutyryl)-β-D-noviosyl fidaxomicin (S-OP1118, 18e-C(11)) in acetone-d_6

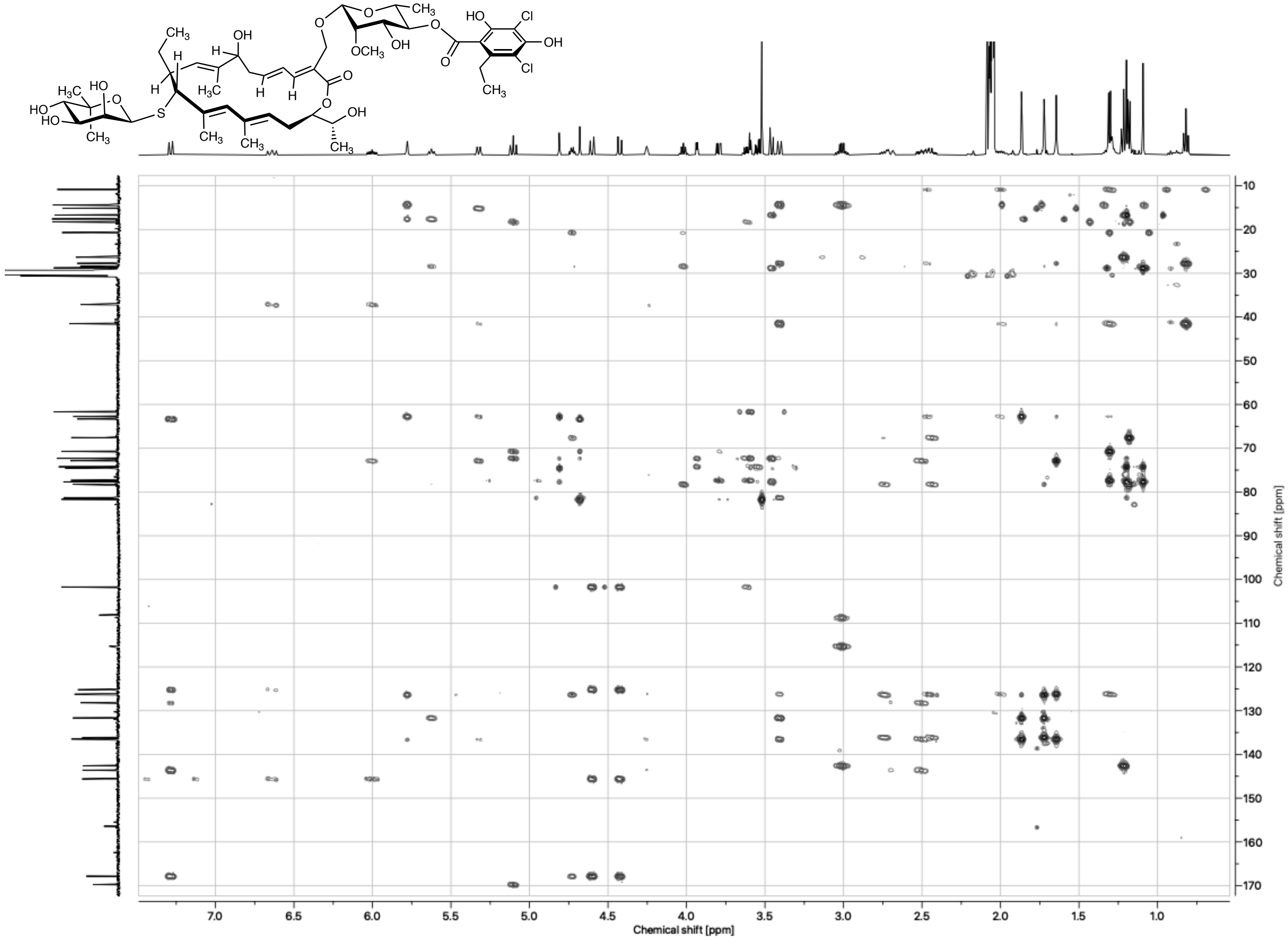

Figure 153: HMBC spectrum of 11-desnoviosyl-11-thio-(4”-desbutyryl)-β-D-noviosyl fidaxomicin (S-OP1118, 18e-C(11)) in acetone-d_6

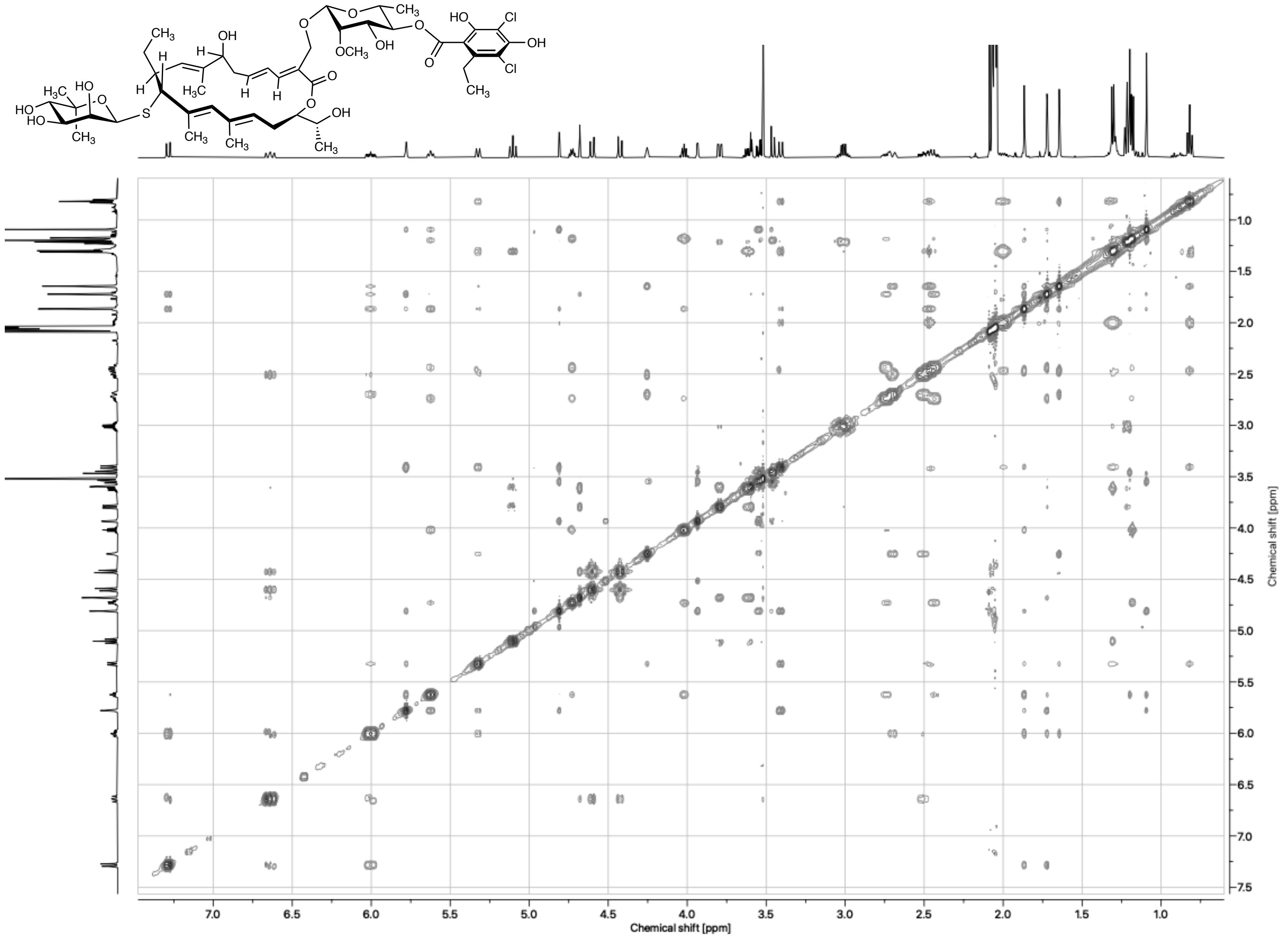

Figure 154: NOESY spectrum of 11-desnoviosyl-11-thio-(4”-desbutyryl)-β-D-noviosyl fidaxomicin (S-OP1118, 18e-C(11)) in acetone-d_6

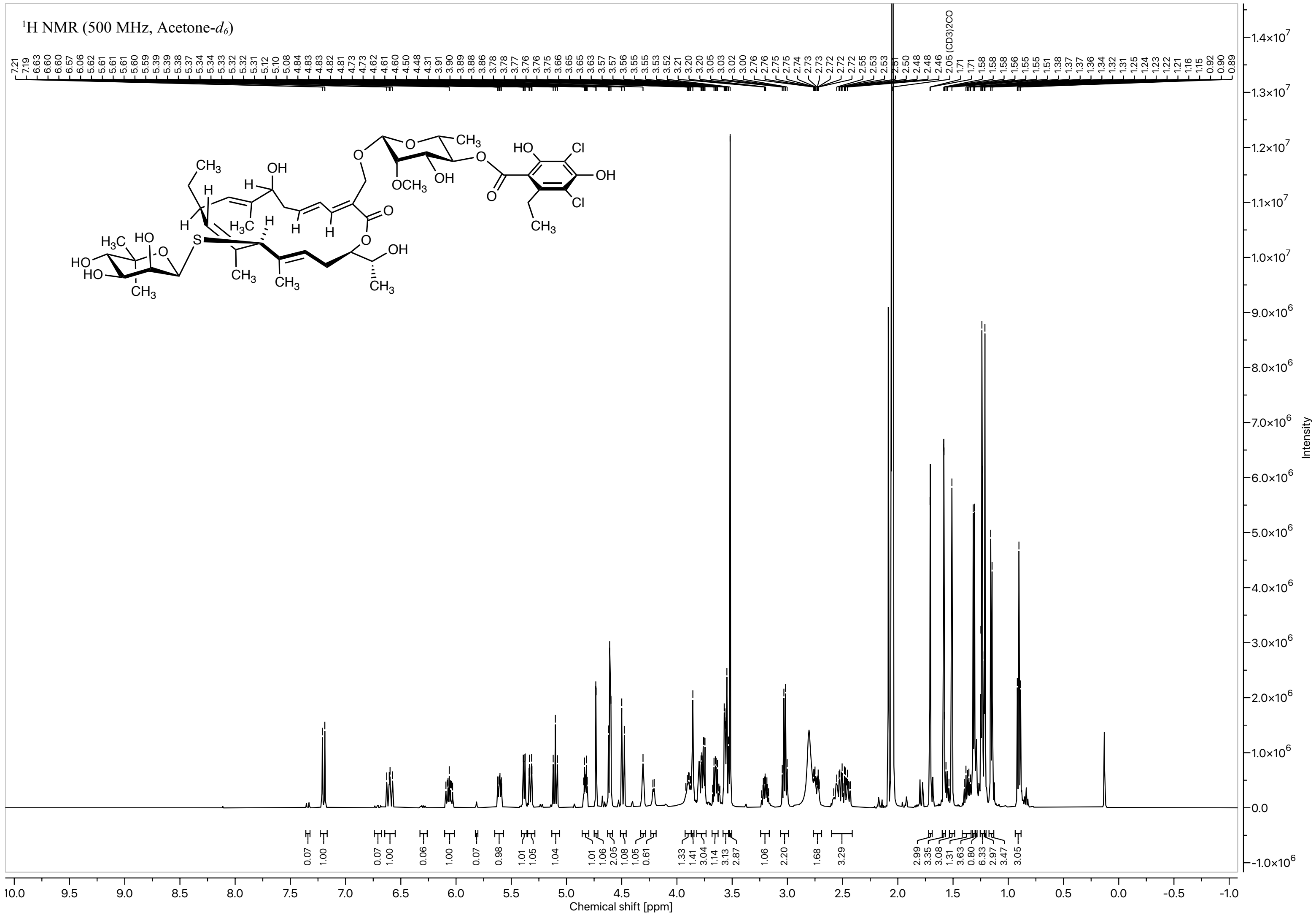

Figure 155: ^{1}H NMR spectrum of 11-desnoviosyl-13-thio-(4”-desbutyryl)-β-D-noviosyl fidaxomicin (18e-C(13)) in acetone-d_6

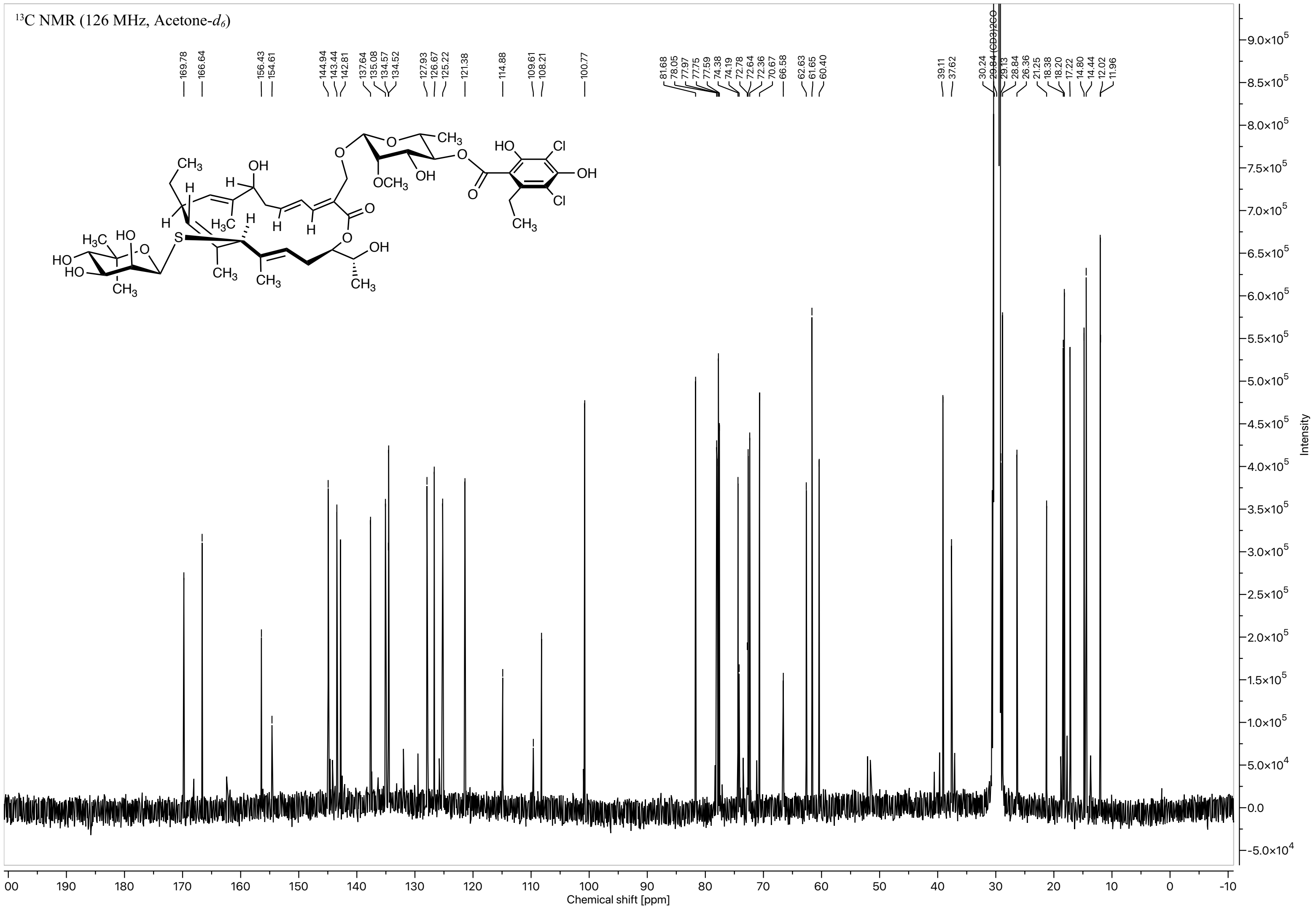

Figure 156: ^{13}C NMR spectrum of 11-desnoviosyl-13-thio-(4”-desbutyryl)-β-D-noviosyl fidaxomicin (18e-C(13)) in acetone-d_6

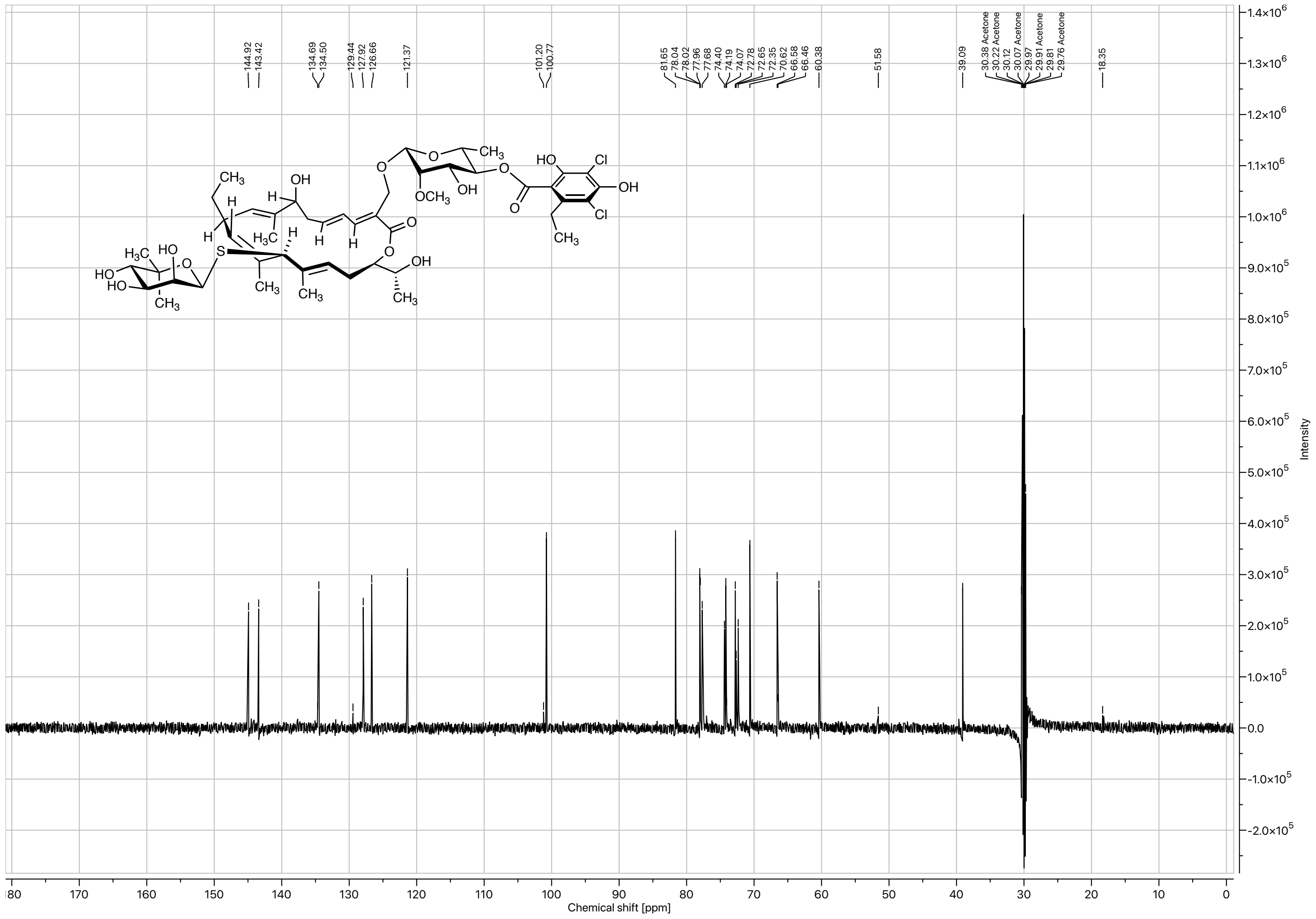

Figure 157: DEPT90 spectrum of 11-desnoviosyl-13-thio-(4”-desbutyryl)-β-D-noviosyl fidaxomicin (18e-C(13)) in acetone-d_6

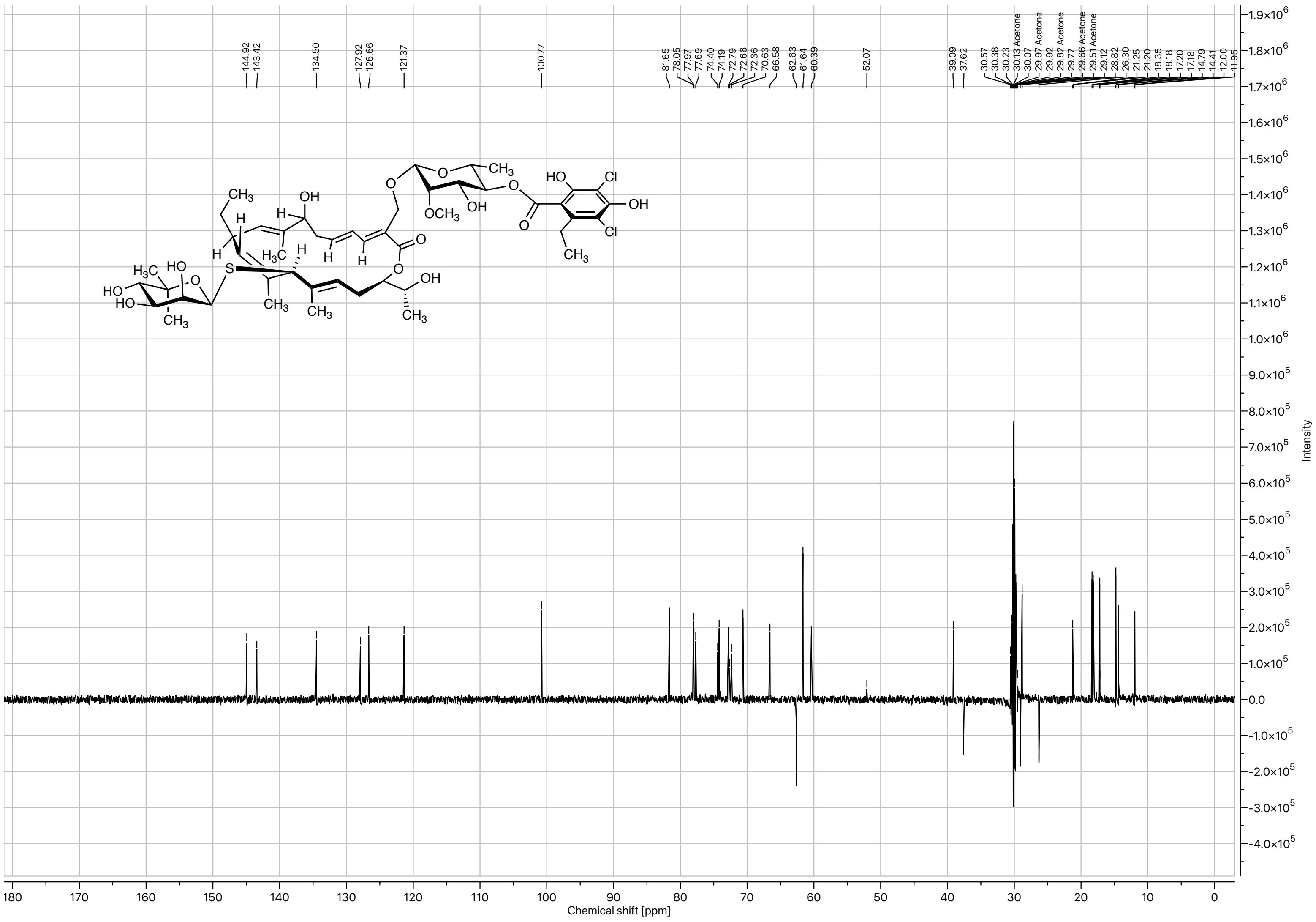

Figure 158: DEPT135 spectrum of 11-desnoviosyl-13-thio-(4”-desbutyryl)-β-D-noviosyl fidaxomicin (18e-C(13)) in acetone-d_6

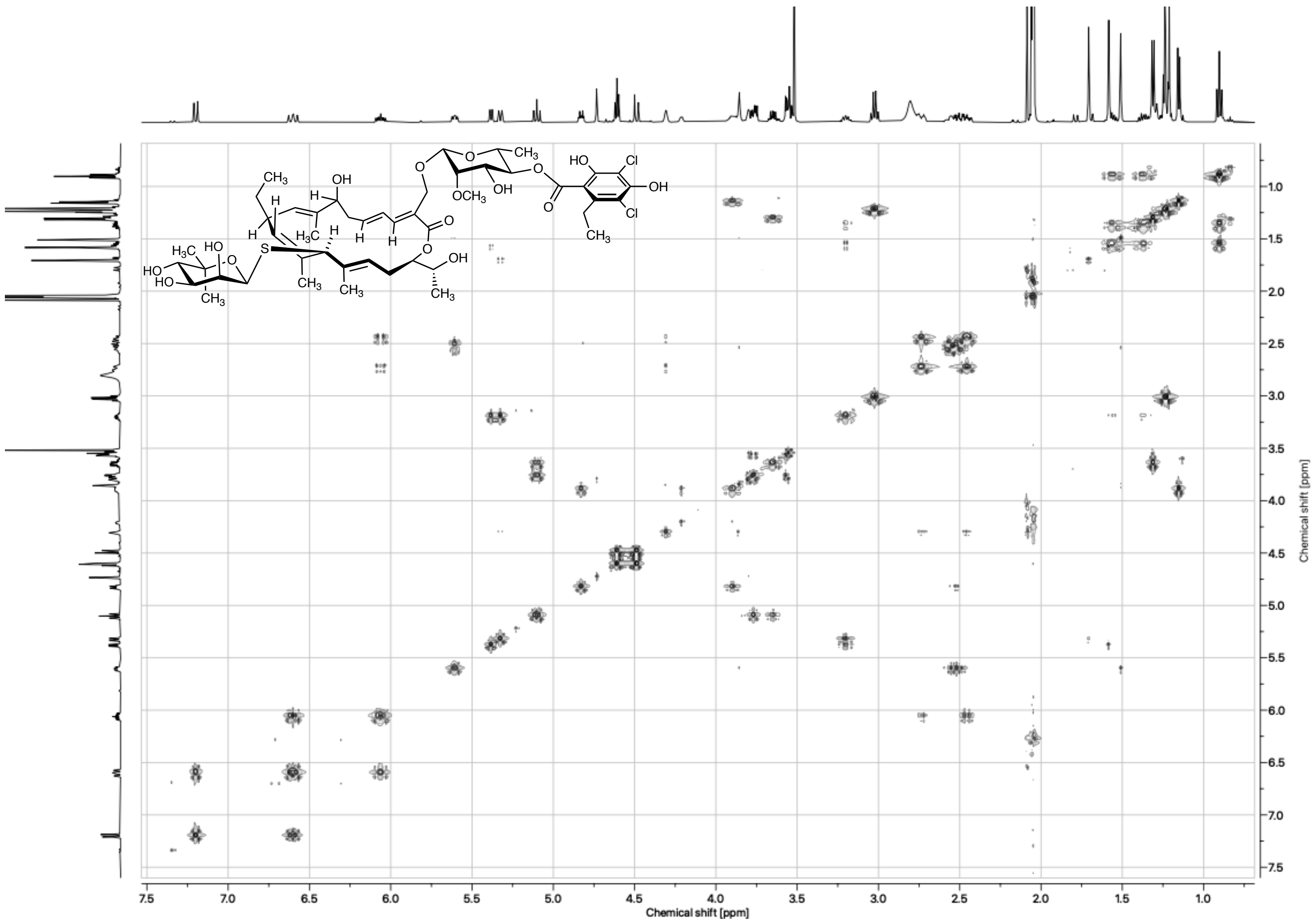

Figure 159: COSY spectrum of 11-desnoviosyl-13-thio-(4”-desbutyryl)-β-D-noviosyl fidaxomicin (18e-C(13)) in acetone-d_6

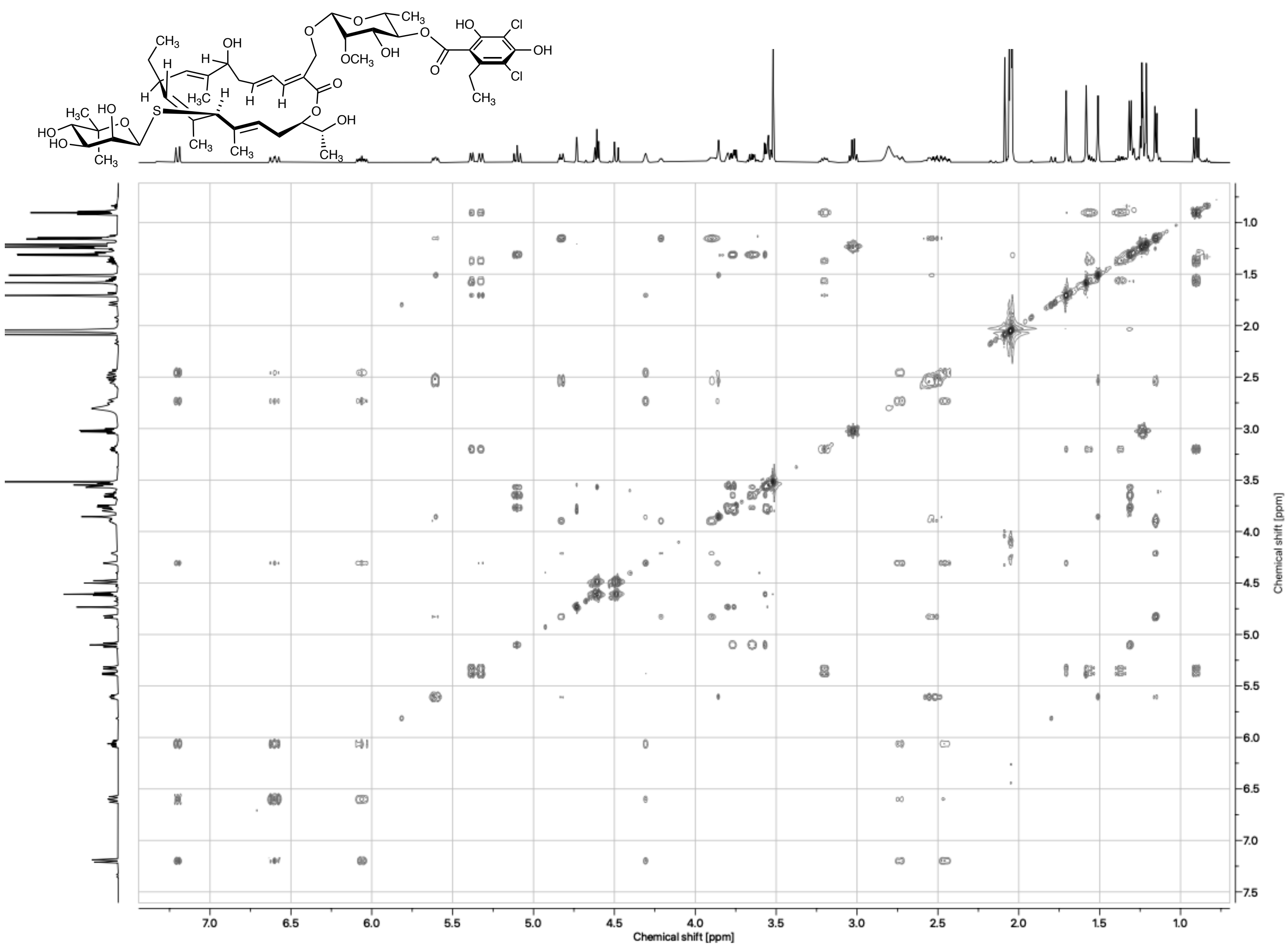

Figure 160: TOCSY spectrum of 11-desnoviosyl-13-thio-(4''-desbutyryl)-β-D-noviosyl fidaxomicin (18e-C(13)) in acetone-d_6

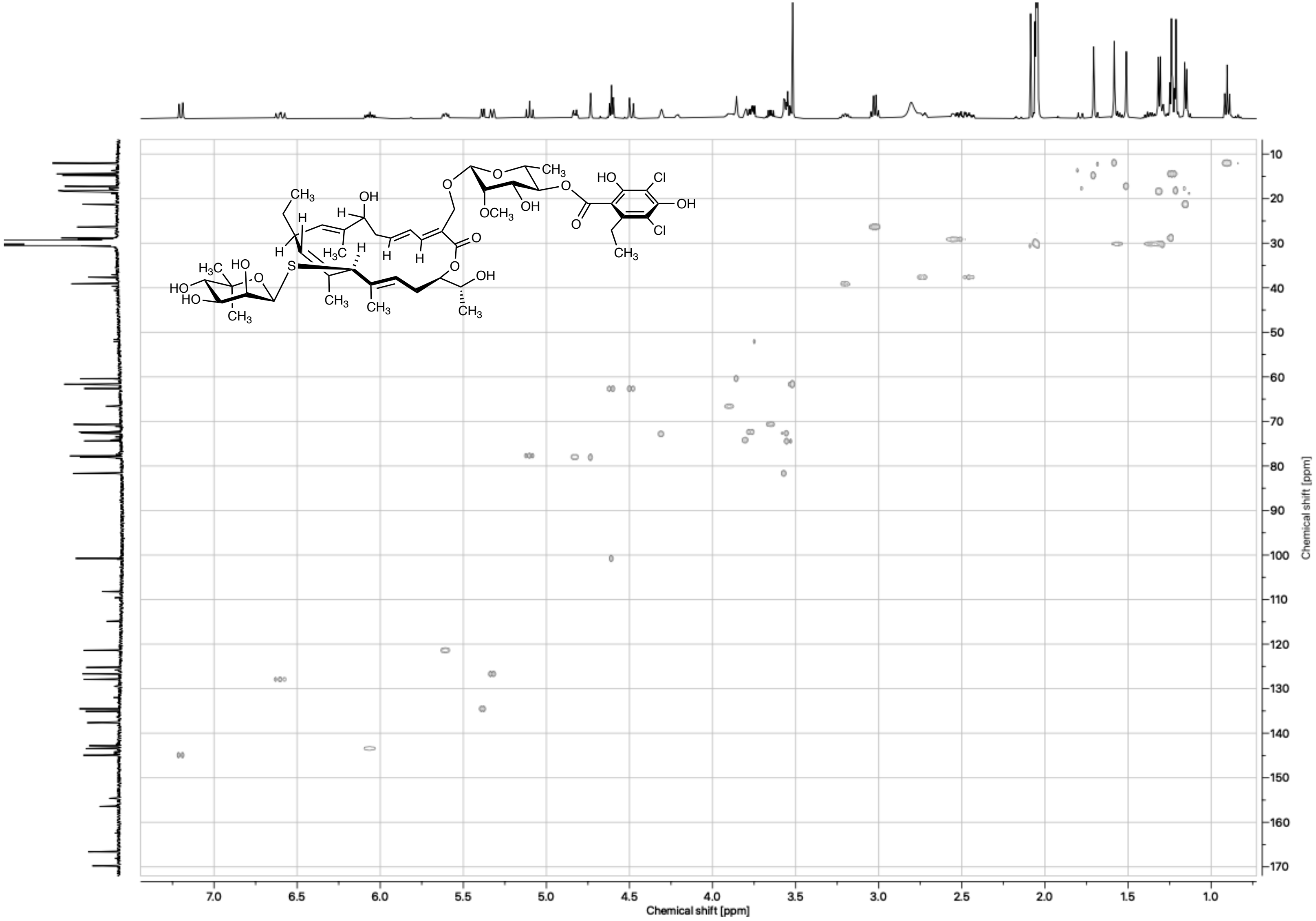

Figure 161: HSQC spectrum of 11-desnoviosyl-13-thio-(4”-desbutyryl)-β-D-noviosyl fidaxomicin (18e-C(13)) in acetone-d_6

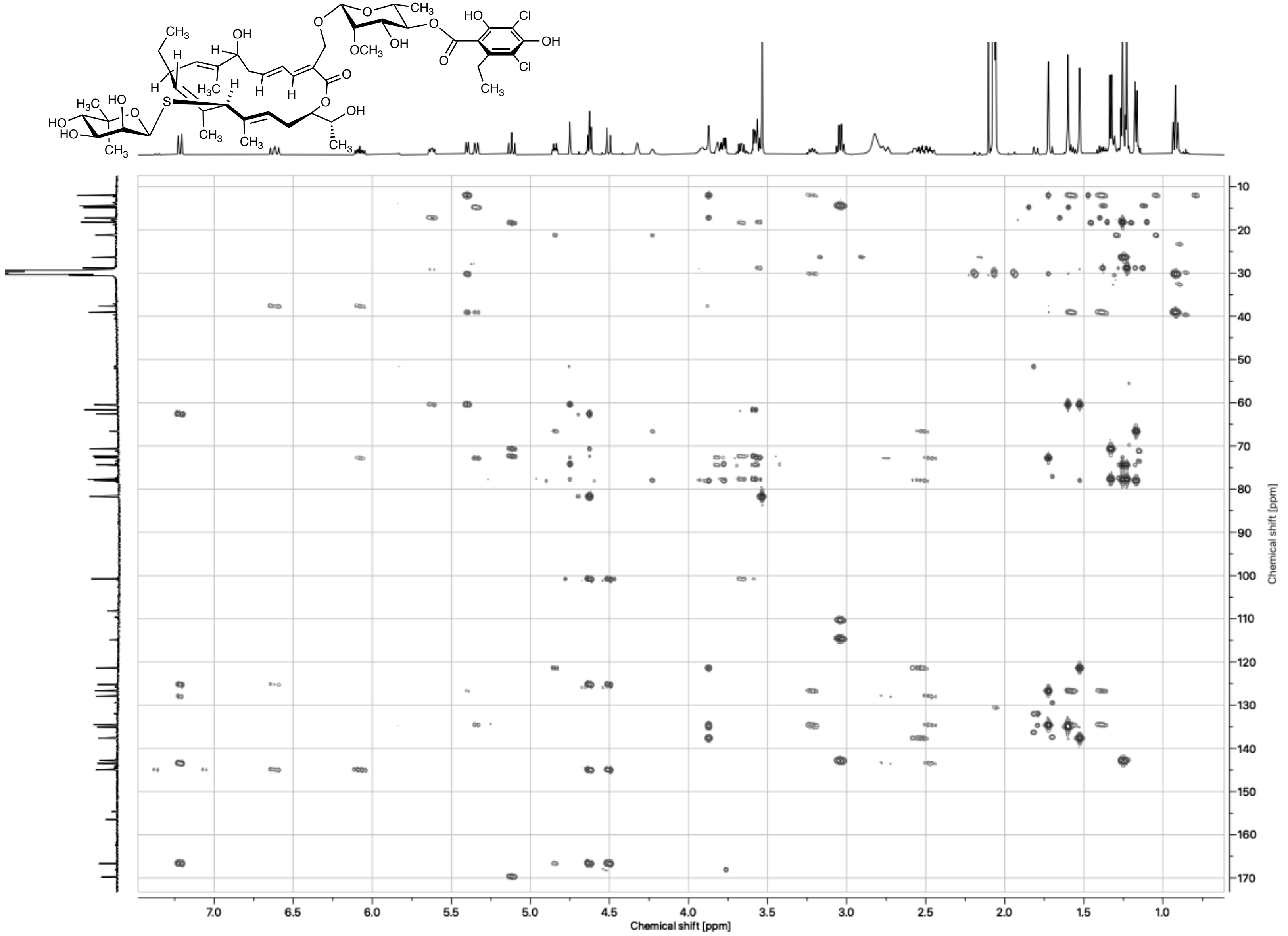

Figure 162: HMBC spectrum of 11-desnoviosyl-13-thio-(4”-desbutyryl)-β-D-noviosyl fidaxomicin (18e-C(13)) in acetone-d_6

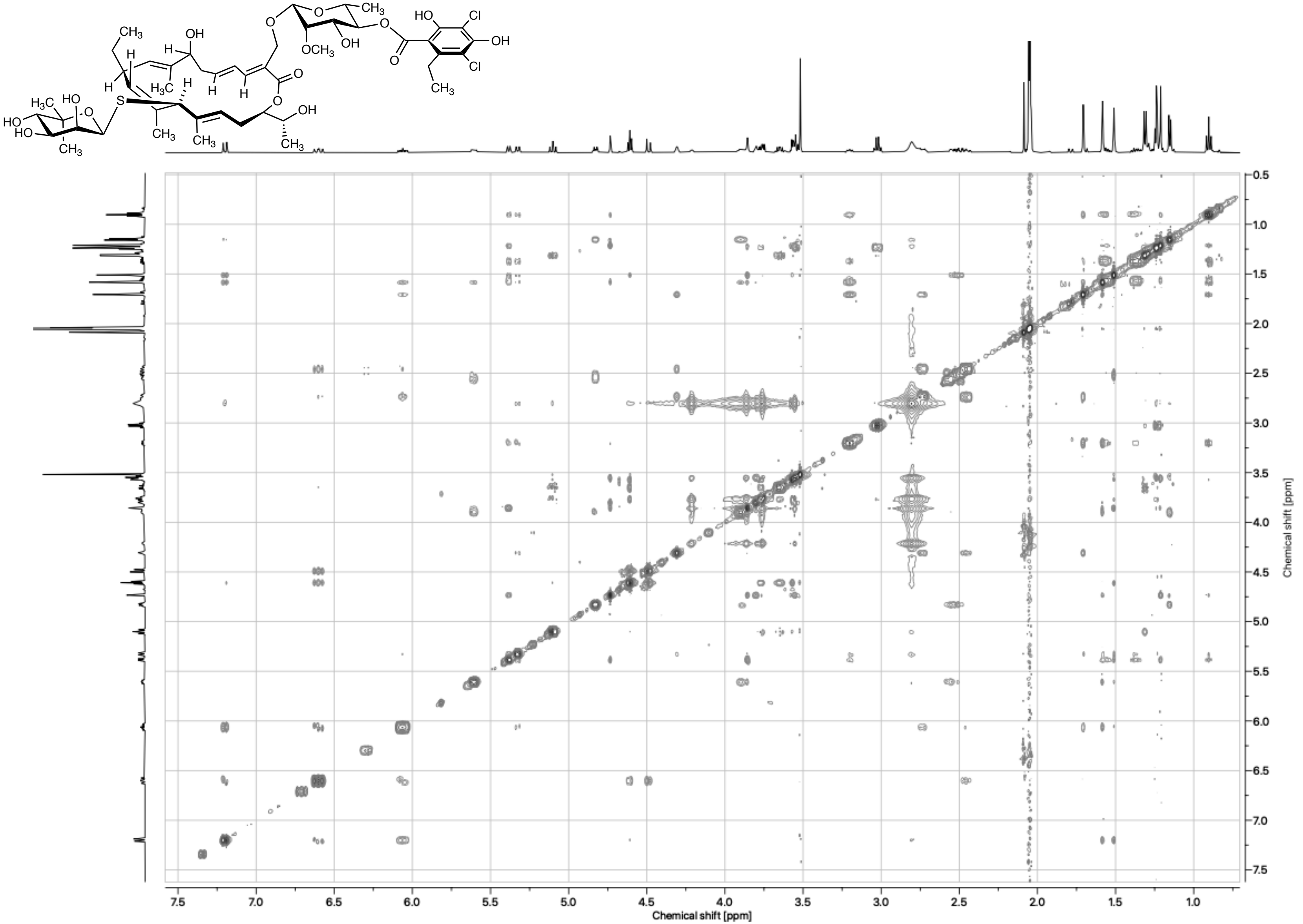

Figure 163: NOESY spectrum of 11-desnoviosyl-13-thio-(4”-desbutyryl)-β-D-noviosyl fidaxomicin (18e-C(13)) in acetone-d_6

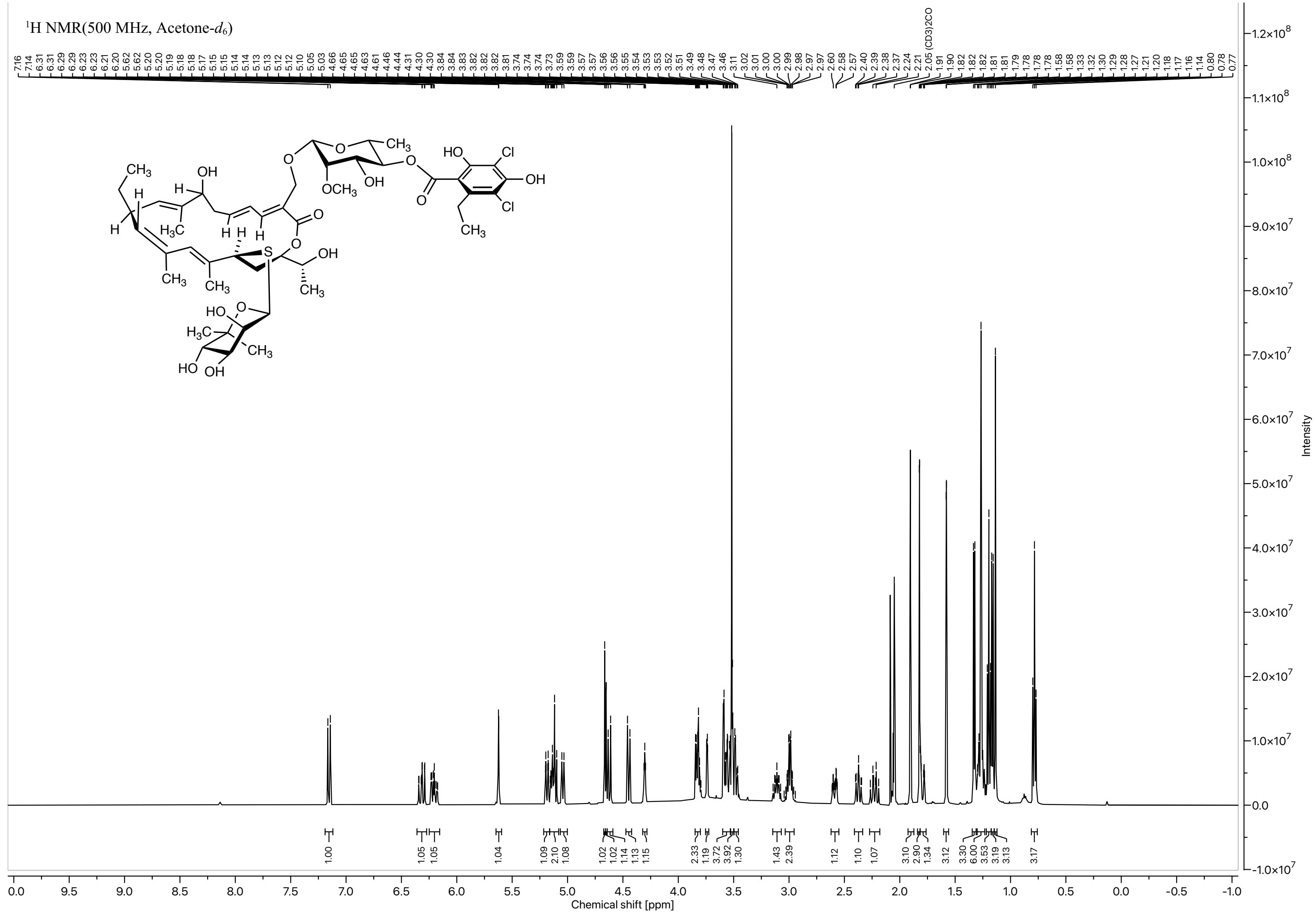

Figure 164: ^{1}H NMR spectrum of 11-desnoviosyl-15-thio-(4”-desbutyryl)-β-D-noviosyl fidaxomicin (18e-C(15)) in acetone-d_6

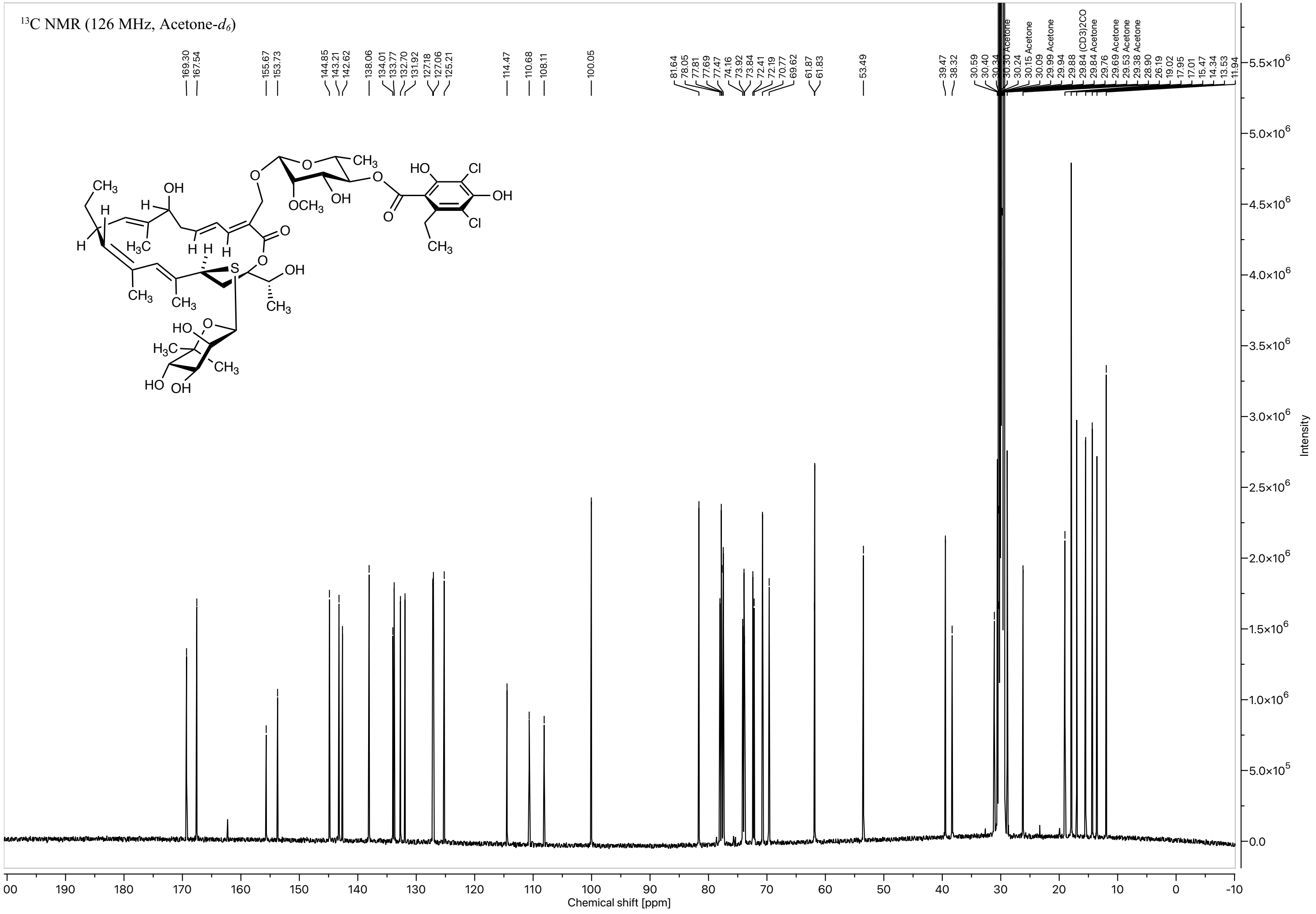

Figure 165: ^{13}C NMR spectrum of 11-desnoviosyl-15-thio-(4”-desbutyryl)-β-D-noviosyl fidaxomicin (18e-C(15)) in acetone-d_6

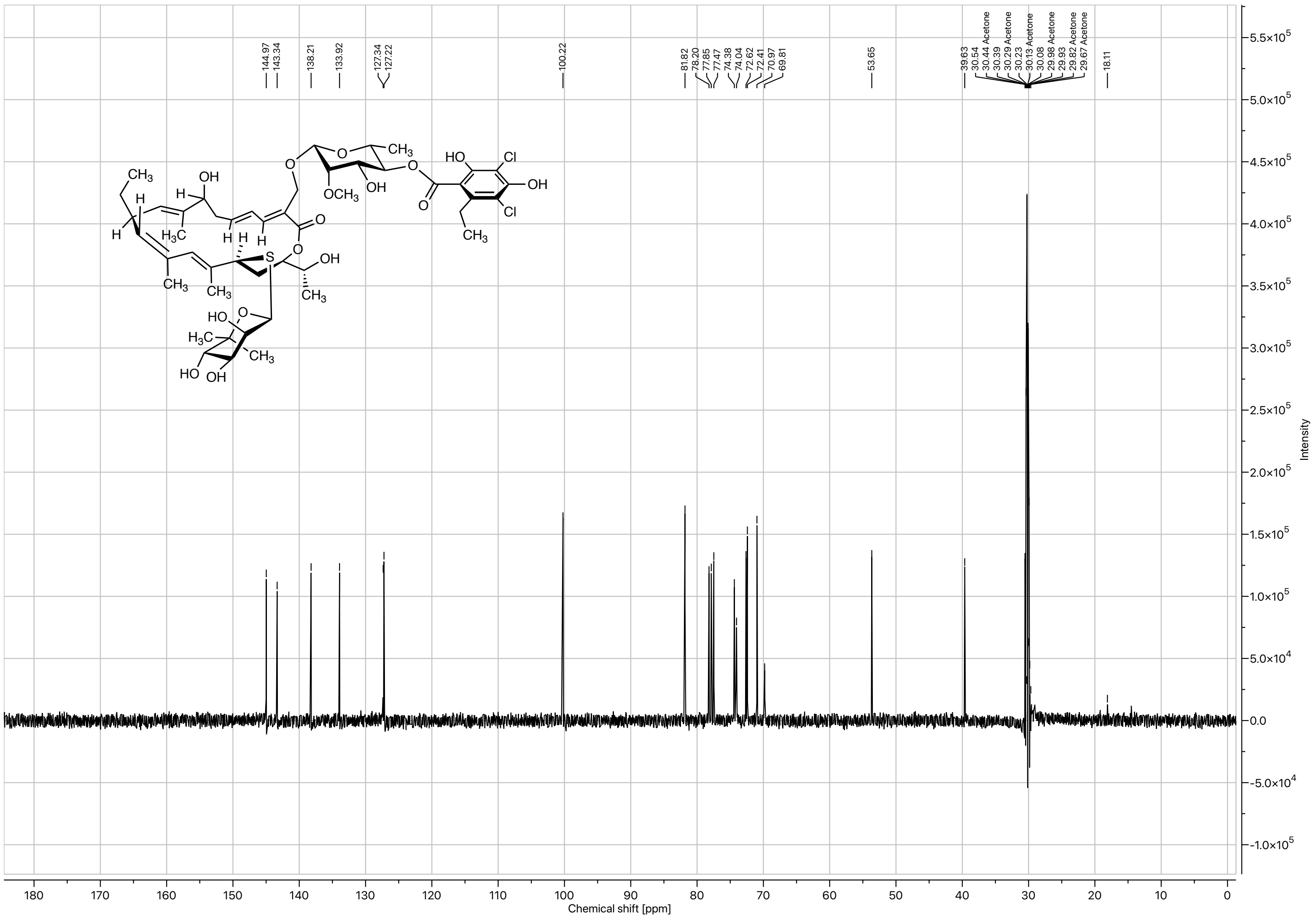

Figure 166: DEPT90 spectrum of 11-desnoviosyl-15-thio-(4”-desbutyryl)-β-D-noviosyl fidaxomicin (18e-C(15)) in acetone-d_6

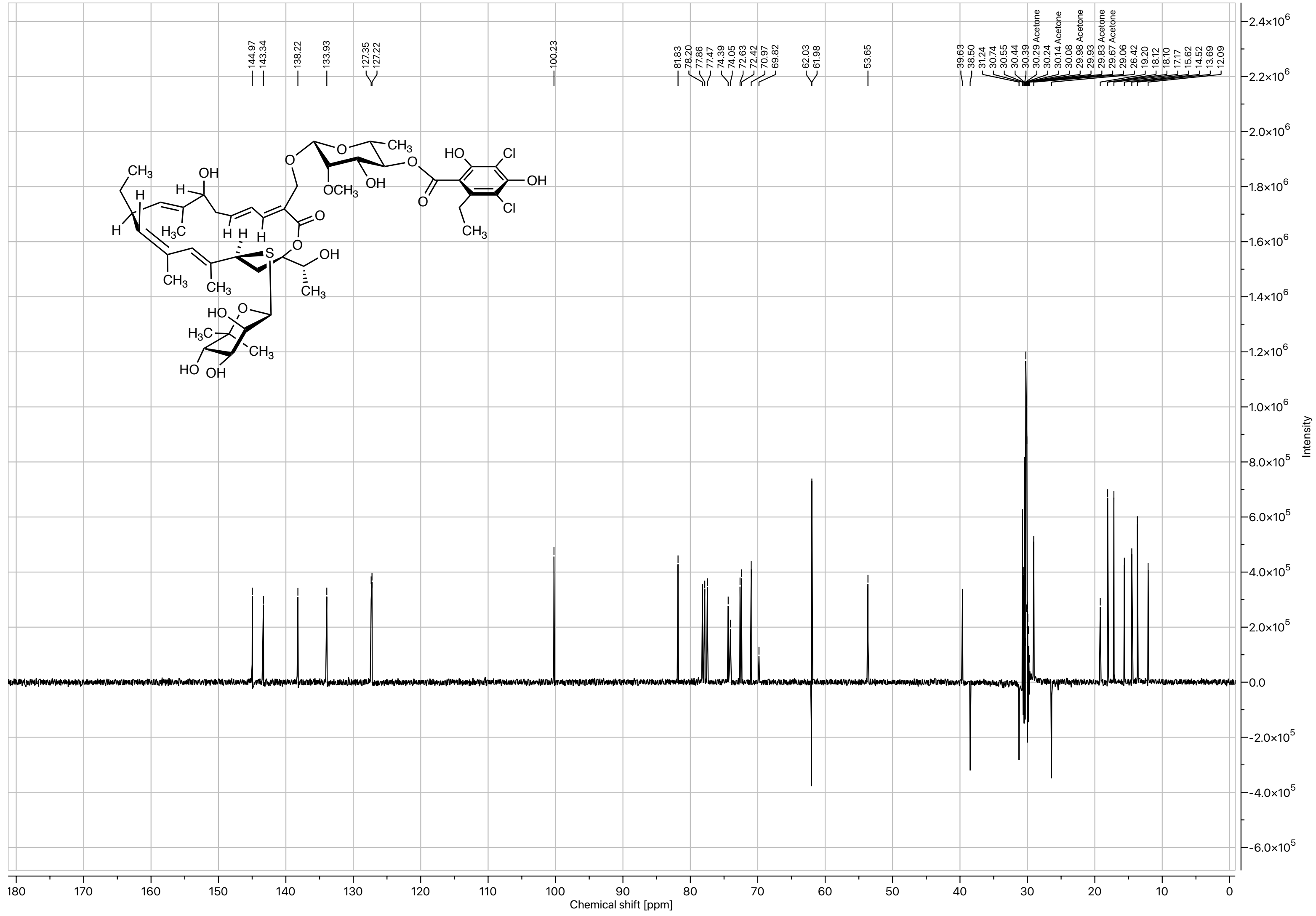

Figure 167: DEPT135 spectrum of 11-desnoviosyl-15-thio-(4”-desbutyryl)-β-D-noviosyl fidaxomicin (18e-C(15)) in acetone-d_6

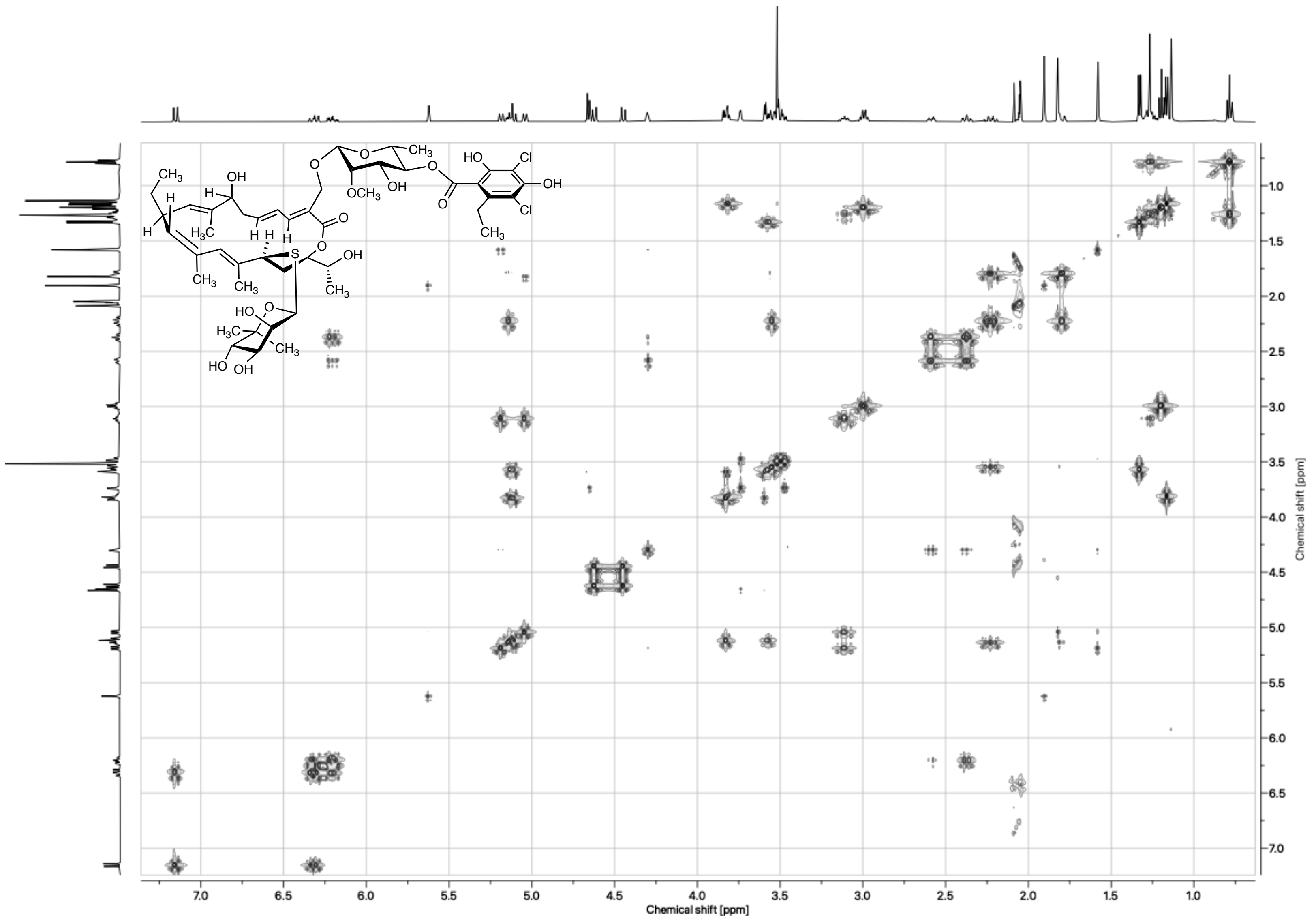

Figure 168: COSY spectrum of 11-desnoviosyl-15-thio-(4”-desbutyryl)-β-D-noviosyl fidaxomicin (18e-C(15)) in acetone-d_6

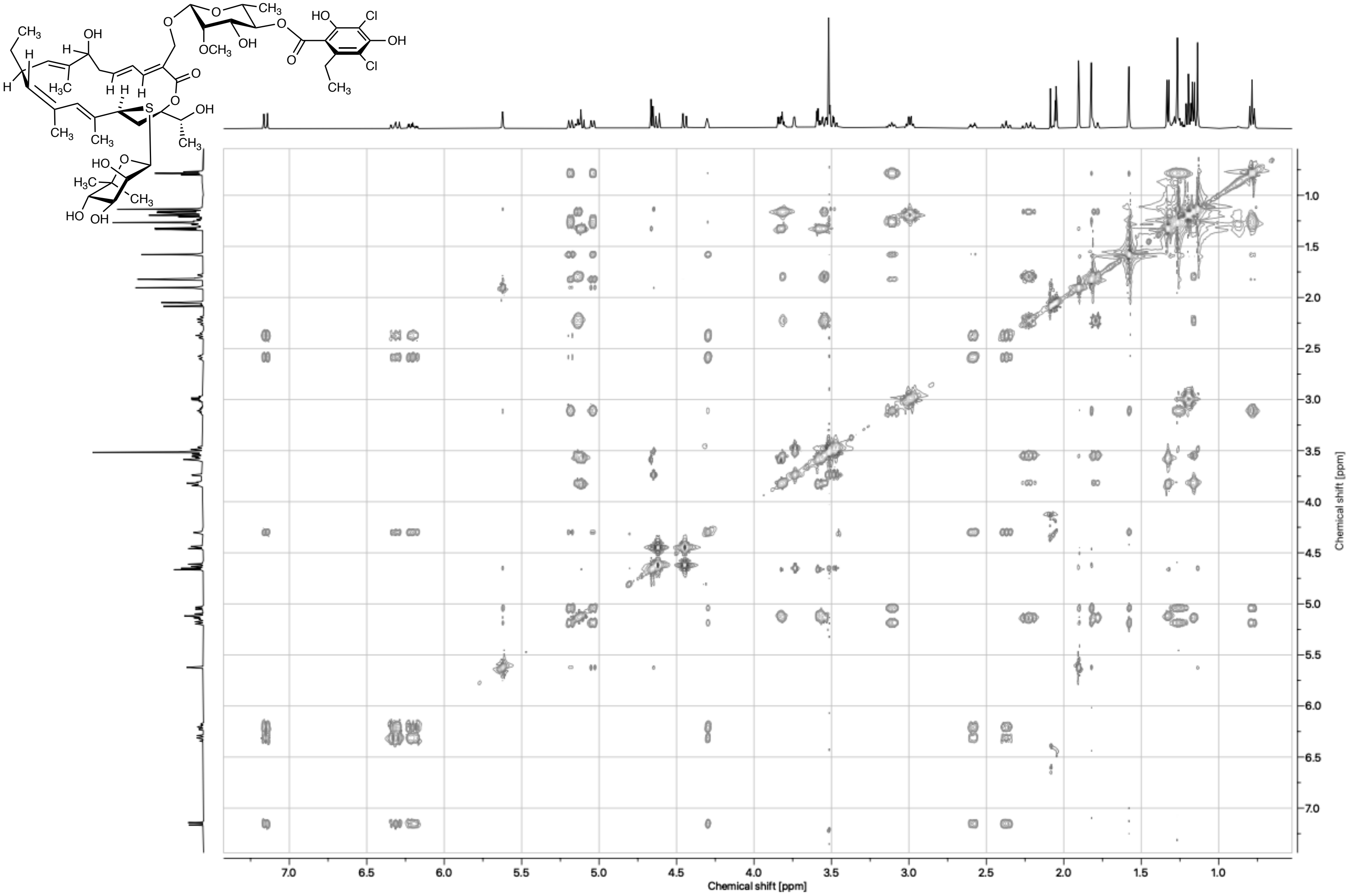

Figure 169: TOCSY spectrum of 11-desnoviosyl-15-thio-(4”-desbutyryl)-β-D-noviosyl fidaxomicin (18e-C(15)) in acetone-d_6

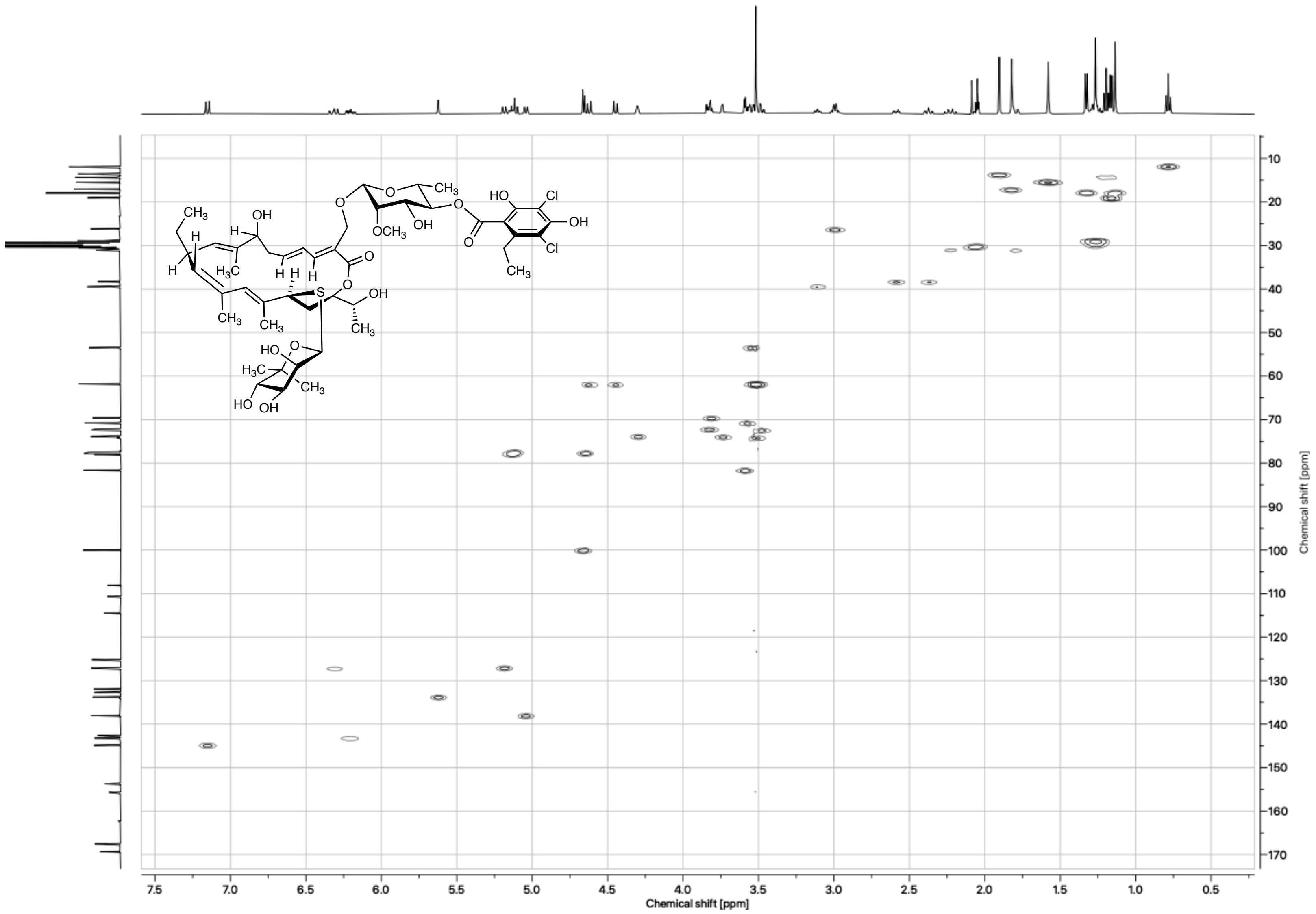

Figure 170: HSQC spectrum of 11-desnoviosyl-15-thio-(4”-desbutyryl)-β-D-noviosyl fidaxomicin (18e-C(15)) in acetone-d_6

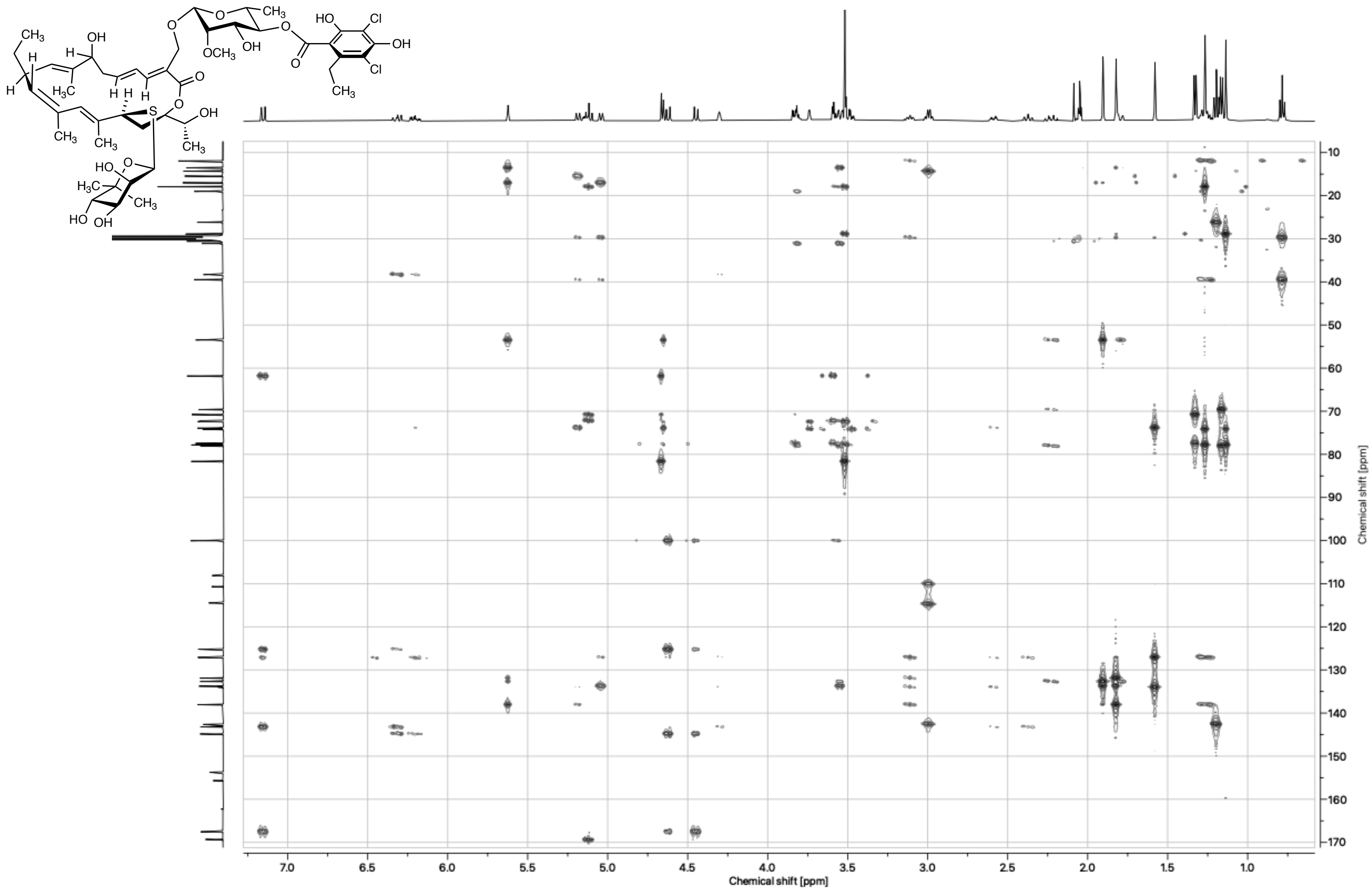

Figure 171: HMBC spectrum of 11-desnoviosyl-15-thio-(4”-desbutyryl)-β-D-noviosyl fidaxomicin (18e-C(15)) in acetone-d_6

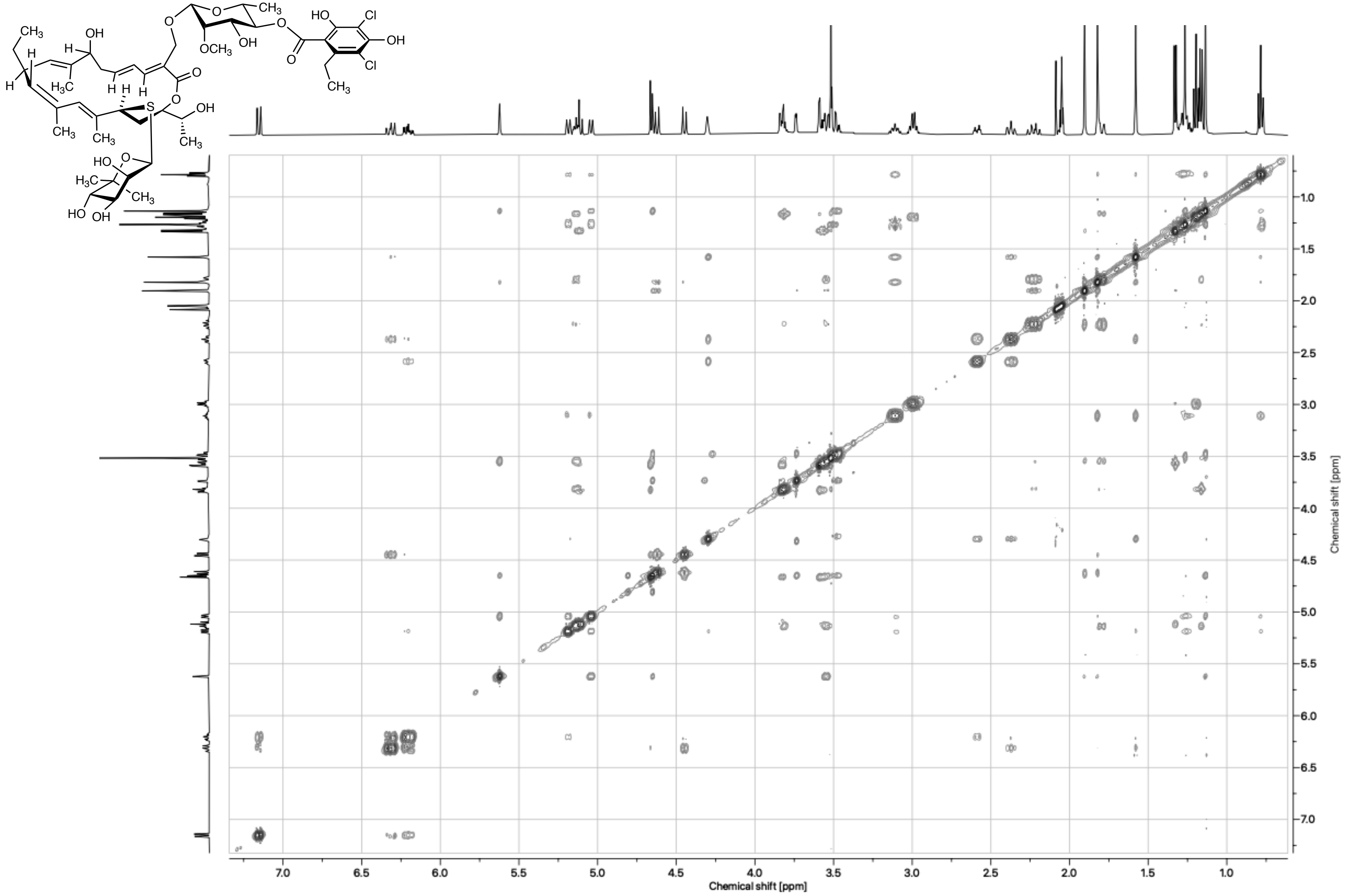

Figure 172: NOESY spectrum of 11-desnoviosyl-15-thio-(4”-desbutyryl)-β-D-noviosyl fidaxomicin (18e-C(15)) in acetone-d_6

Spectral data for 4’’-*O*-acyl-*S*-Fdx derivatives

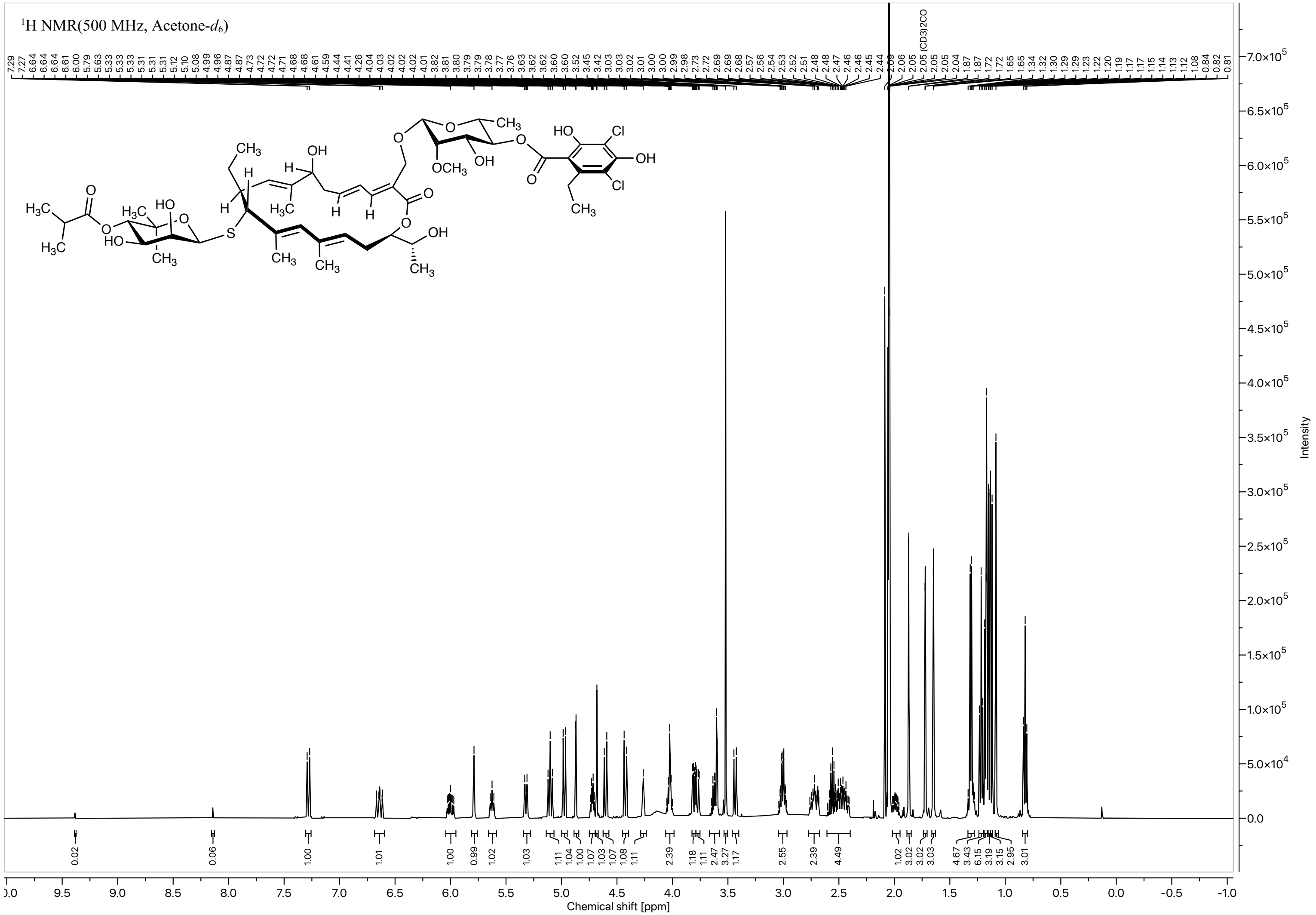

Figure 173: ^{1}H NMR spectrum of 11-desnoviosyl-11-thio-β-D-noviosyl fidaxomicin (S-Fdx, 3a-C(11)) in acetone-d_6

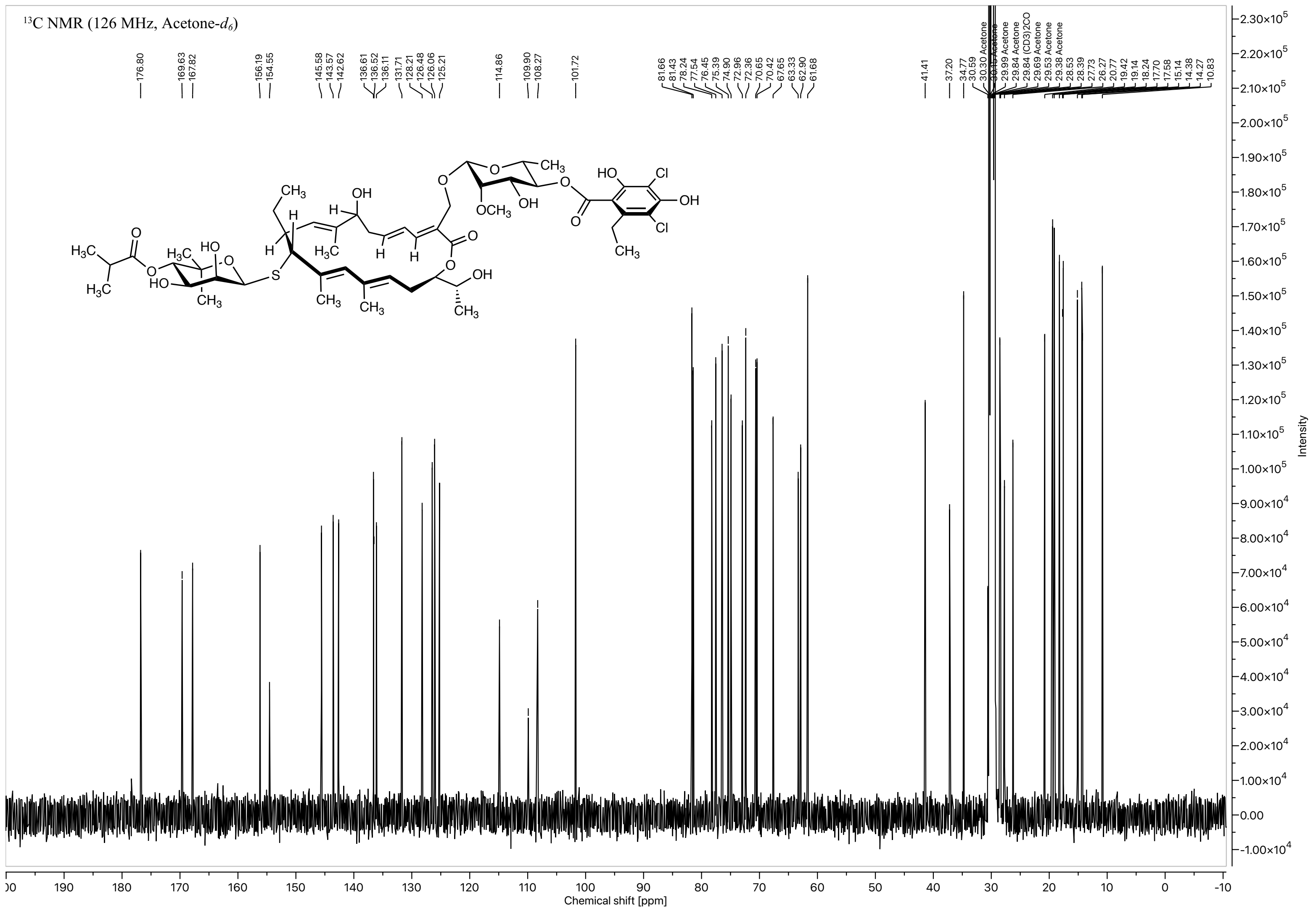

Figure 174: ^{13}C NMR spectrum of 11-desnoviosyl-11-thio-β-D-noviosyl fidaxomicin (S-Fdx, 3a-C(11)) in acetone-d_6

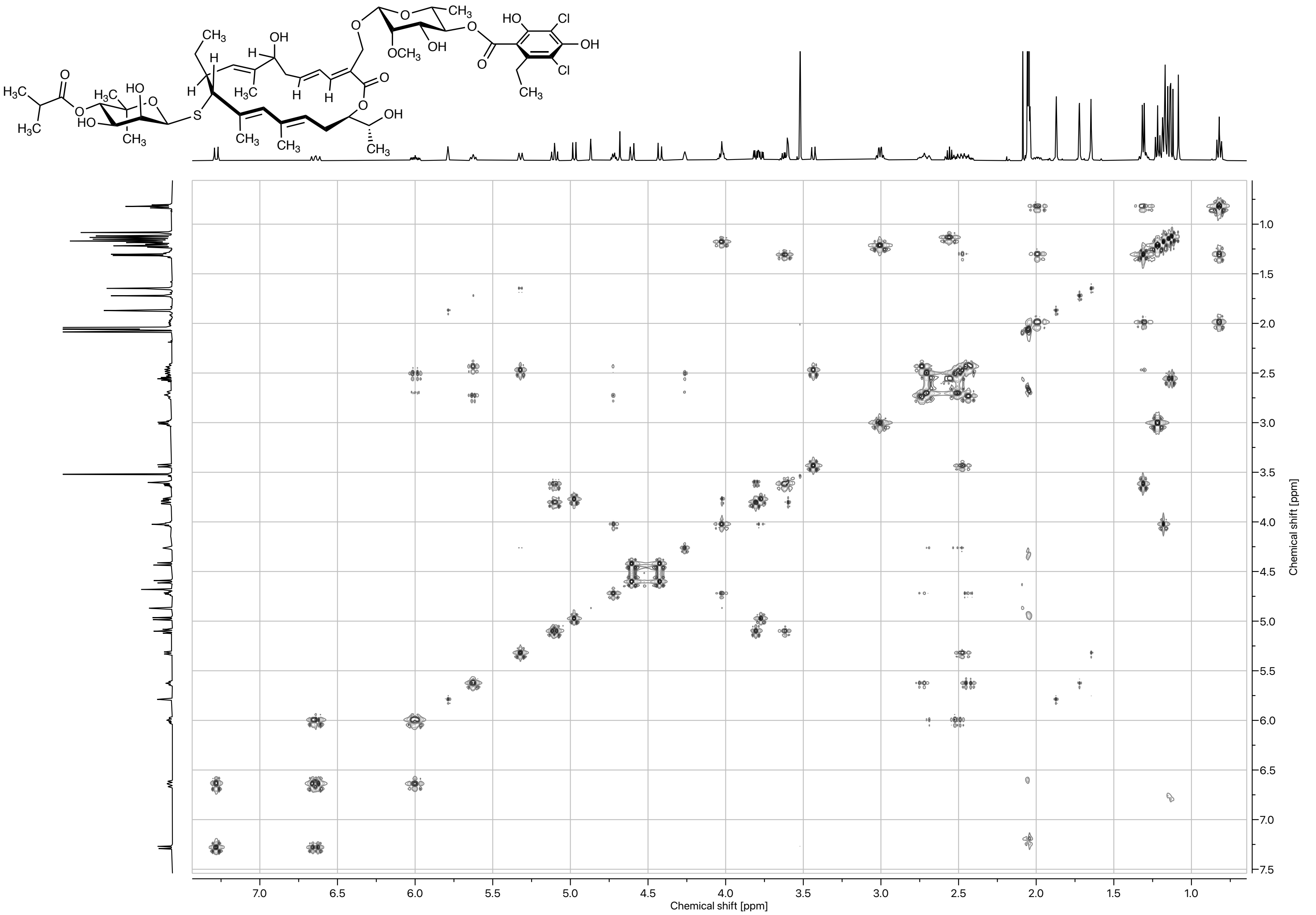

Figure 175: COSY spectrum of 11-desnoviosyl-11-thio-β-D-noviosyl fidaxomicin (S-Fdx, 3a-C(11)) in acetone-d_6

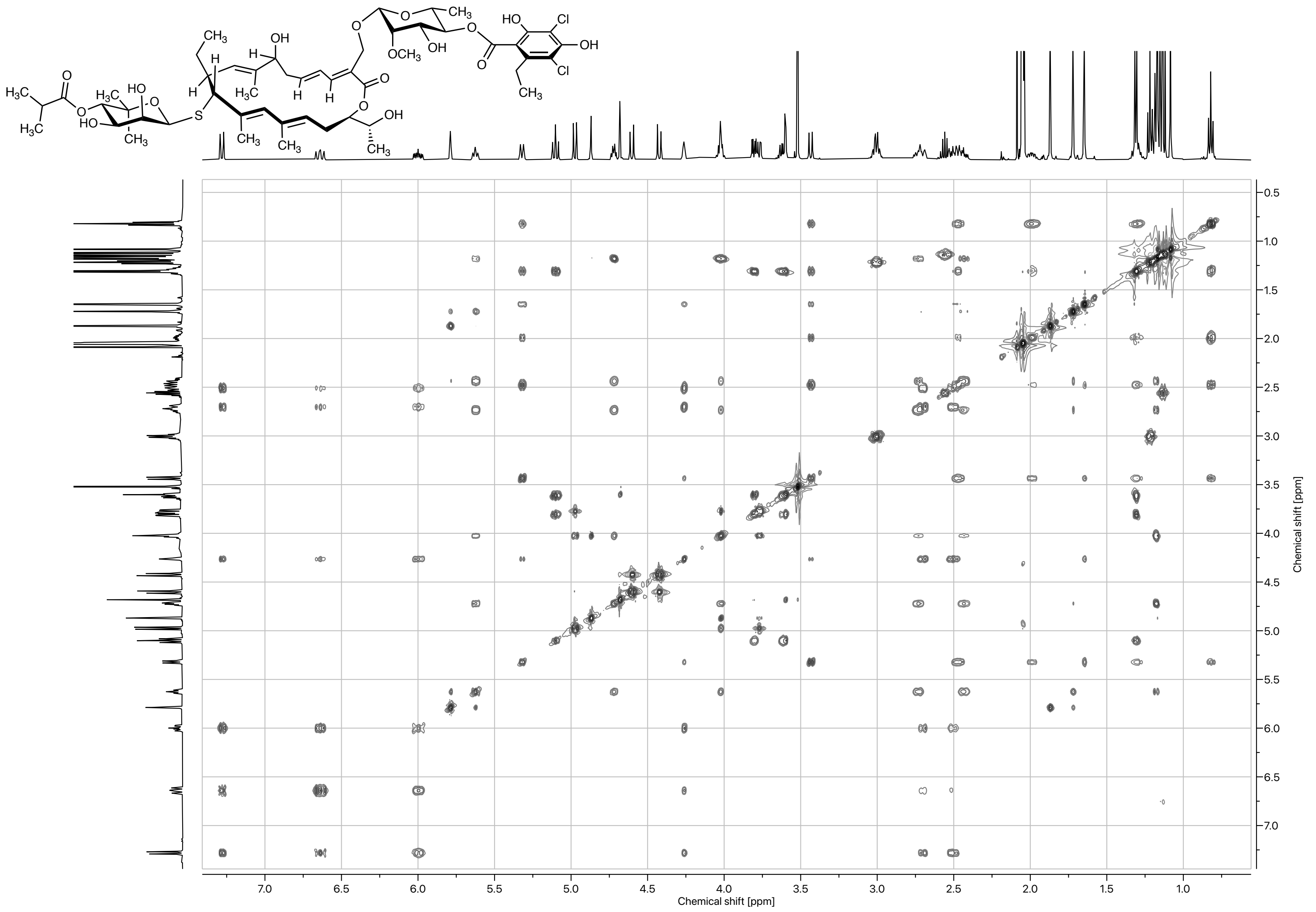

Figure 176: TOCSY spectrum of 11-desnoviosyl-11-thio-β-D-noviosyl fidaxomicin (S-Fdx, 3a-C(11)) in acetone-d_6

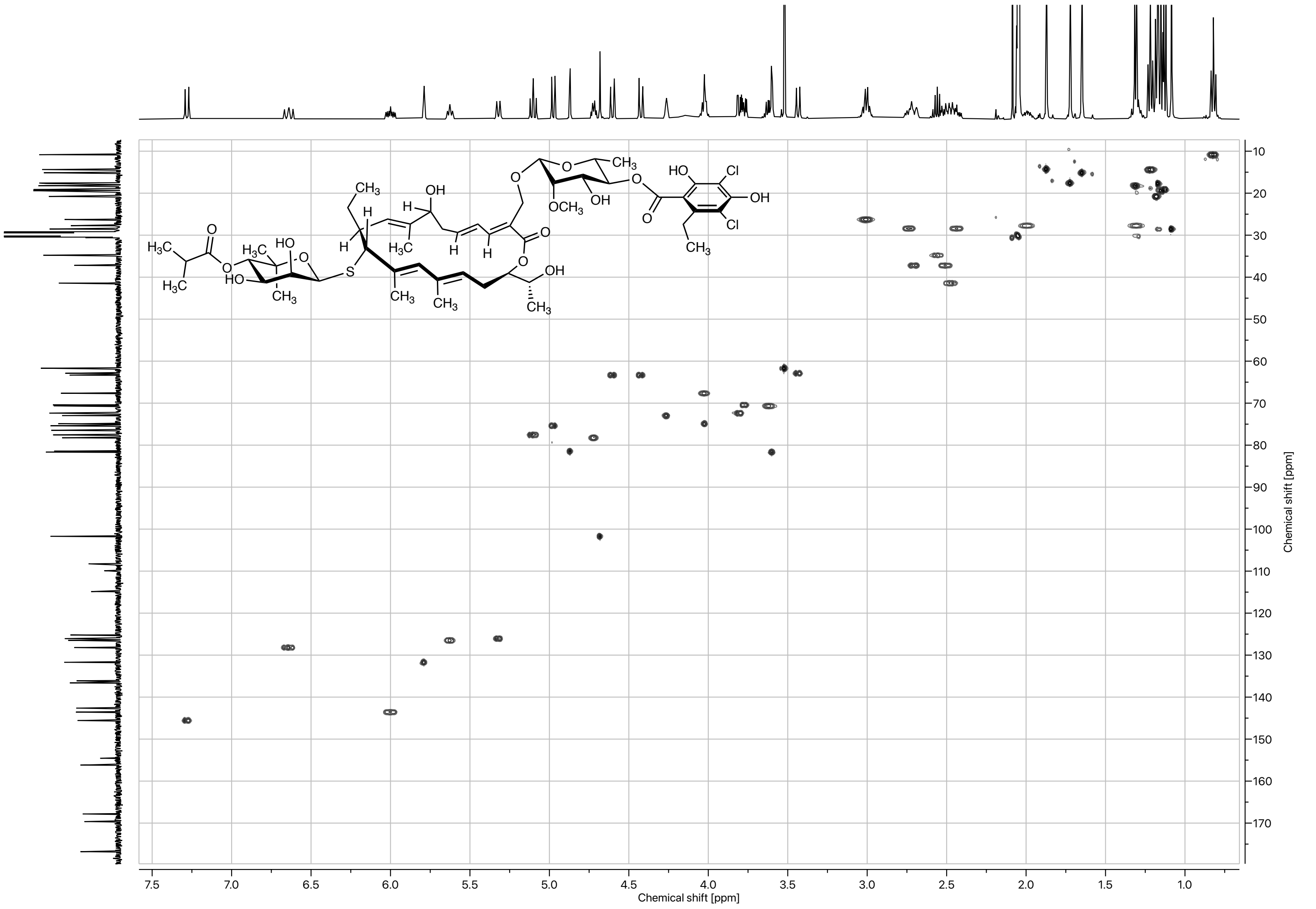

Figure 177: HSQC spectrum of 11-desnoviosyl-11-thio-β-D-noviosyl fidaxomicin (S-Fdx, 3a-C(11)) in acetone-d_6

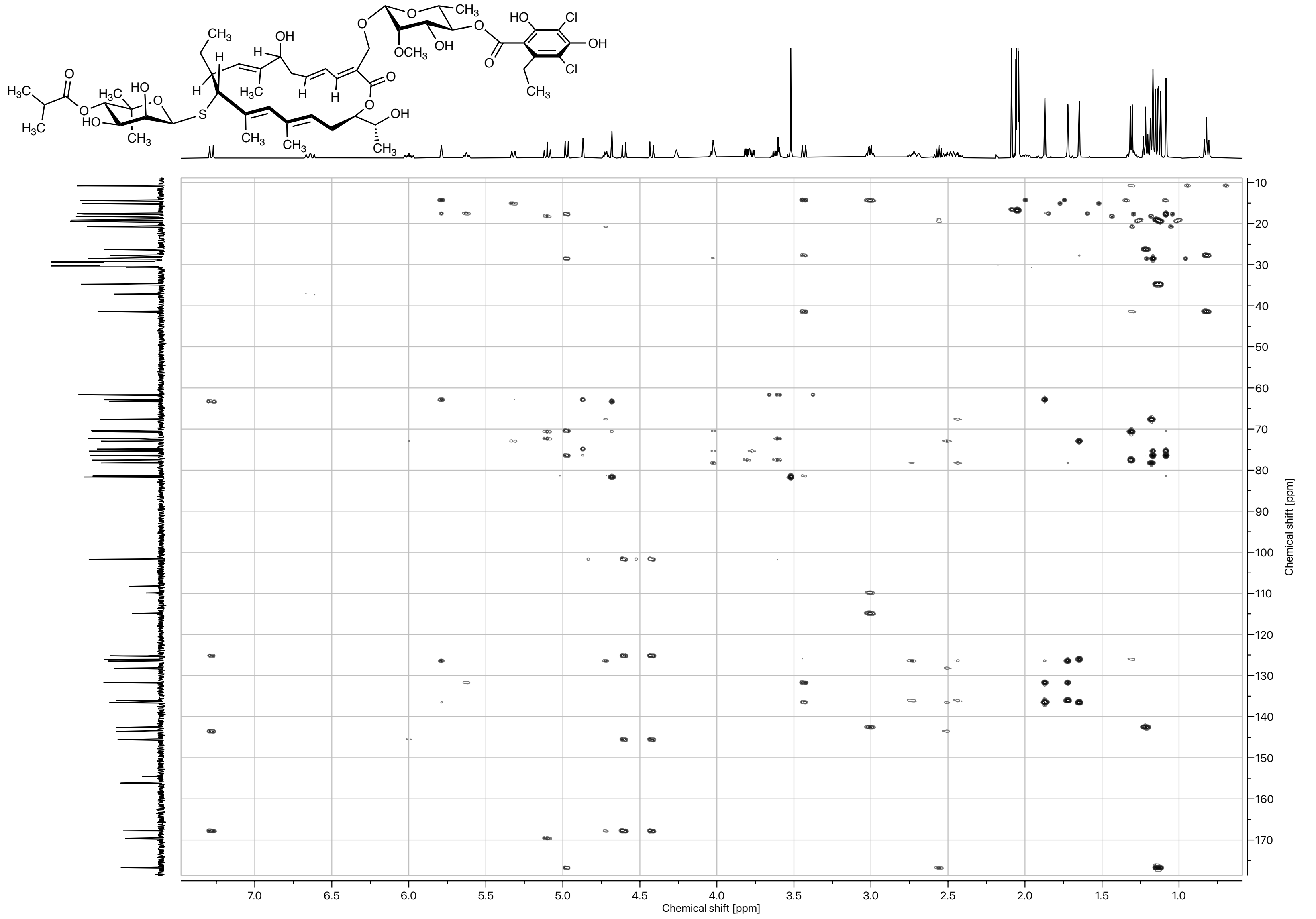

Figure 178: HMBC spectrum of 11-desnoviosyl-11-thio-β-D-noviosyl fidaxomicin (S-Fdx, 3a-C(11)) in acetone-d_6

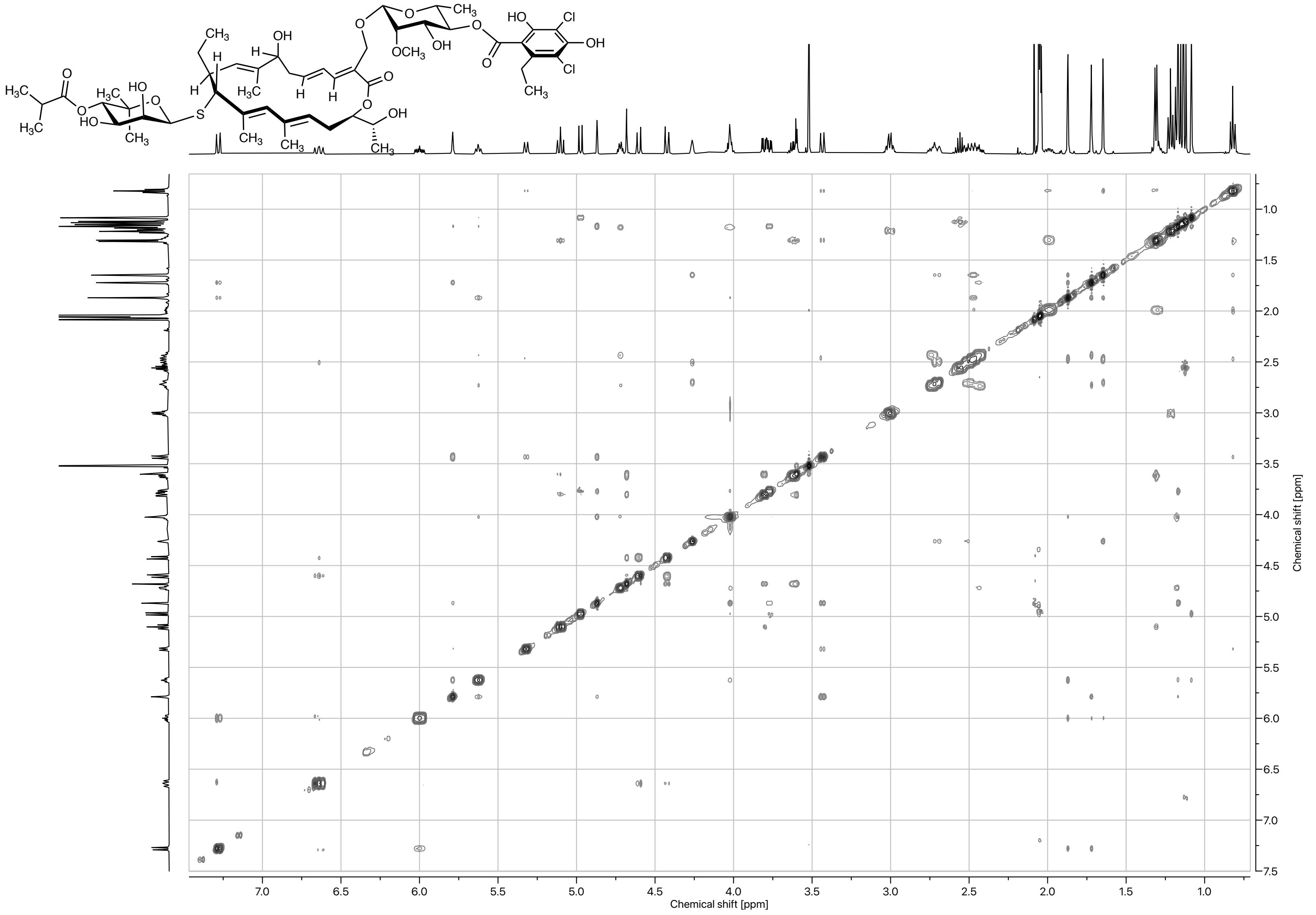

Figure 179: NOESY spectrum of 11-desnoviosyl-11-thio-β-D-noviosyl fidaxomicin (S-Fdx, 3a-C(11)) in acetone-d_6

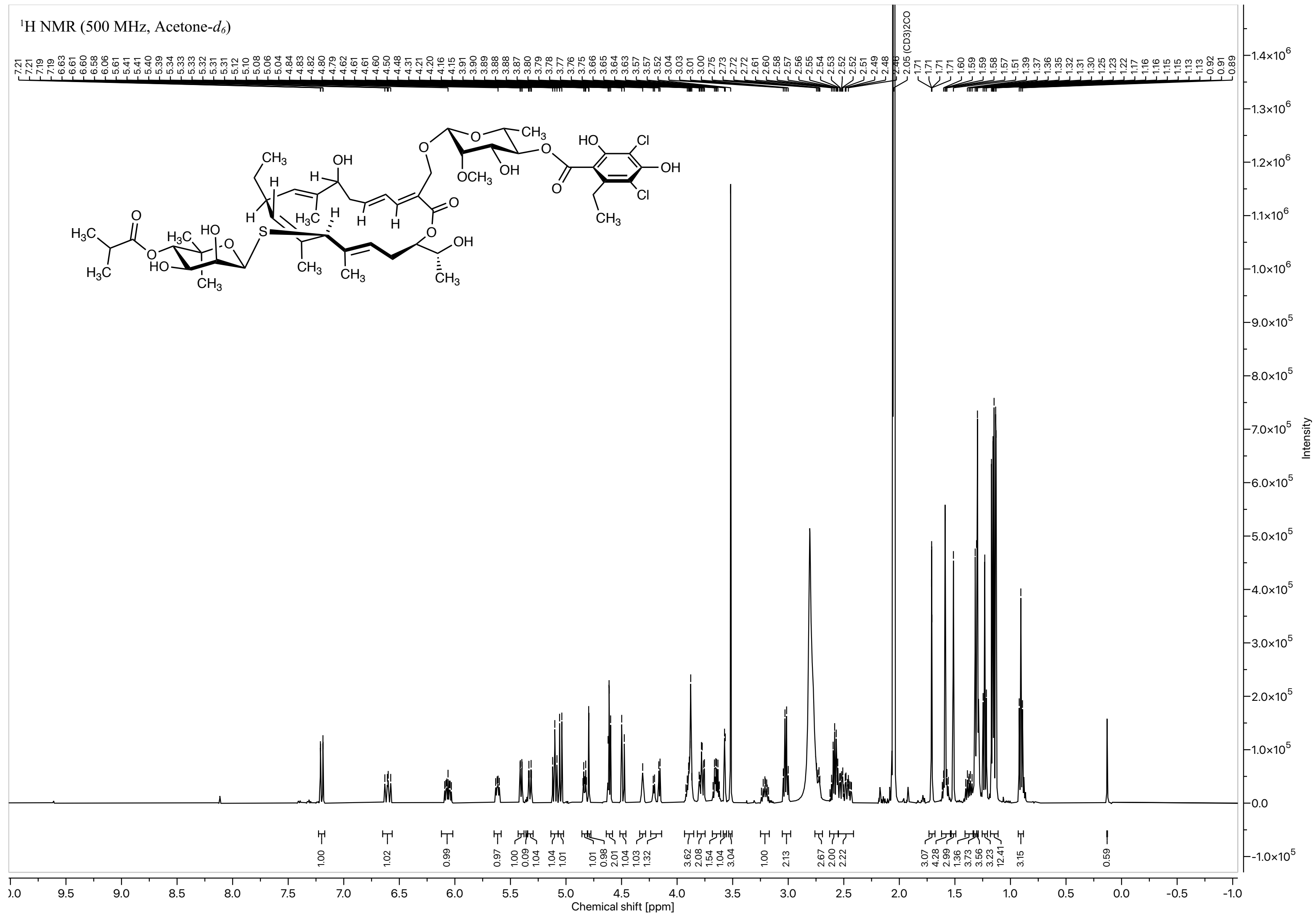

Figure 180: ^{1}H NMR spectrum of spectrum of 11-desnoviosyl-13-thio-β-D-noviosyl fidaxomicin (3a-C(13)) in acetone-d_6

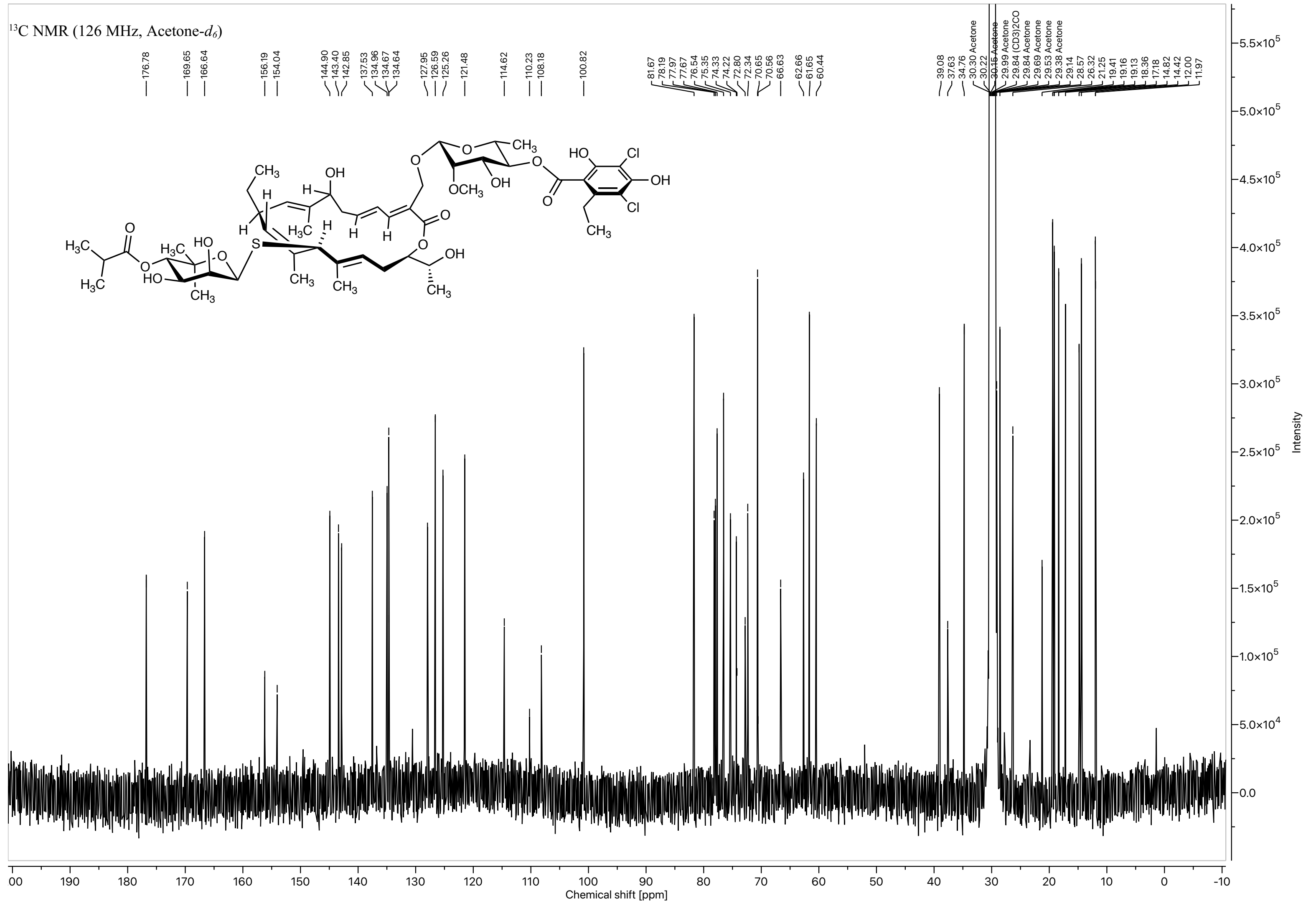

Figure 181: ^{13}C NMR spectrum of spectrum of 11-desnoviosyl-13-thio-β-D-noviosyl fidaxomicin (3a-C(13)) in acetone-d_6

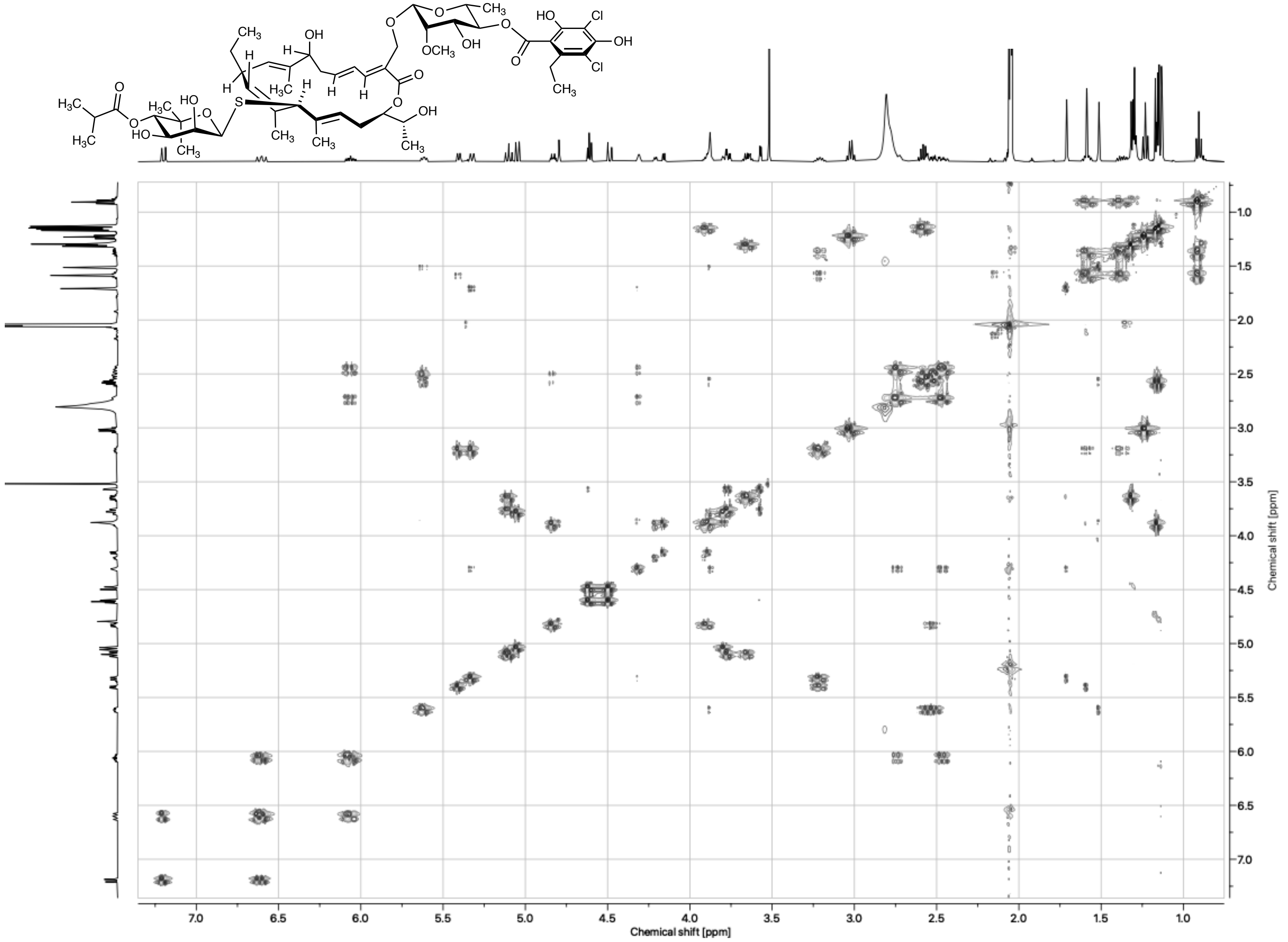

Figure 182: COSY spectrum of spectrum of 11-desnoviosyl-13-thio-β-D-noviosyl fidaxomicin (3a-C(13)) in acetone-d_6

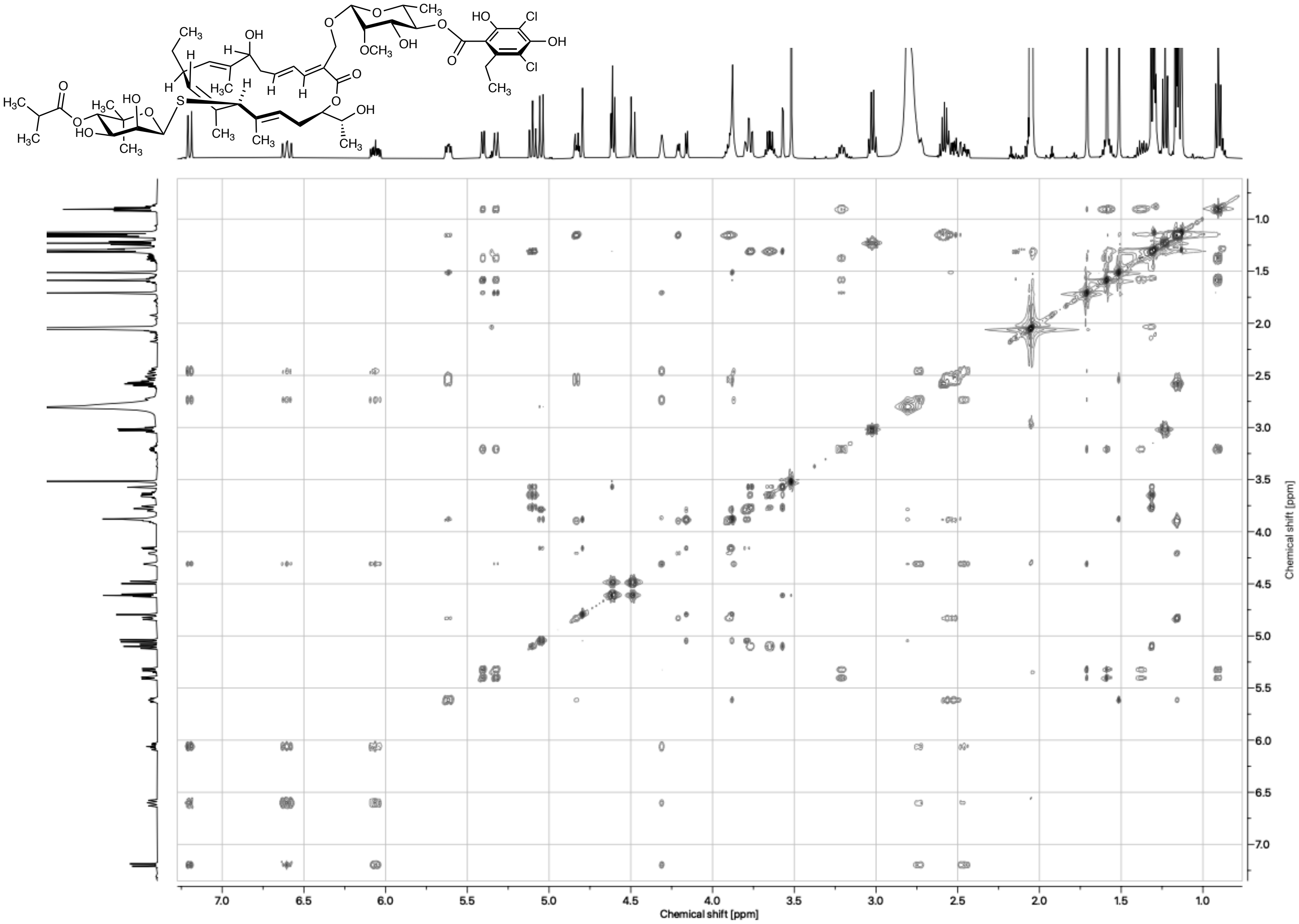

Figure 183: TOCSY spectrum of spectrum of 11-desnoviosyl-13-thio-β-D-noviosyl fidaxomicin (3a-C(13)) in acetone-d_6

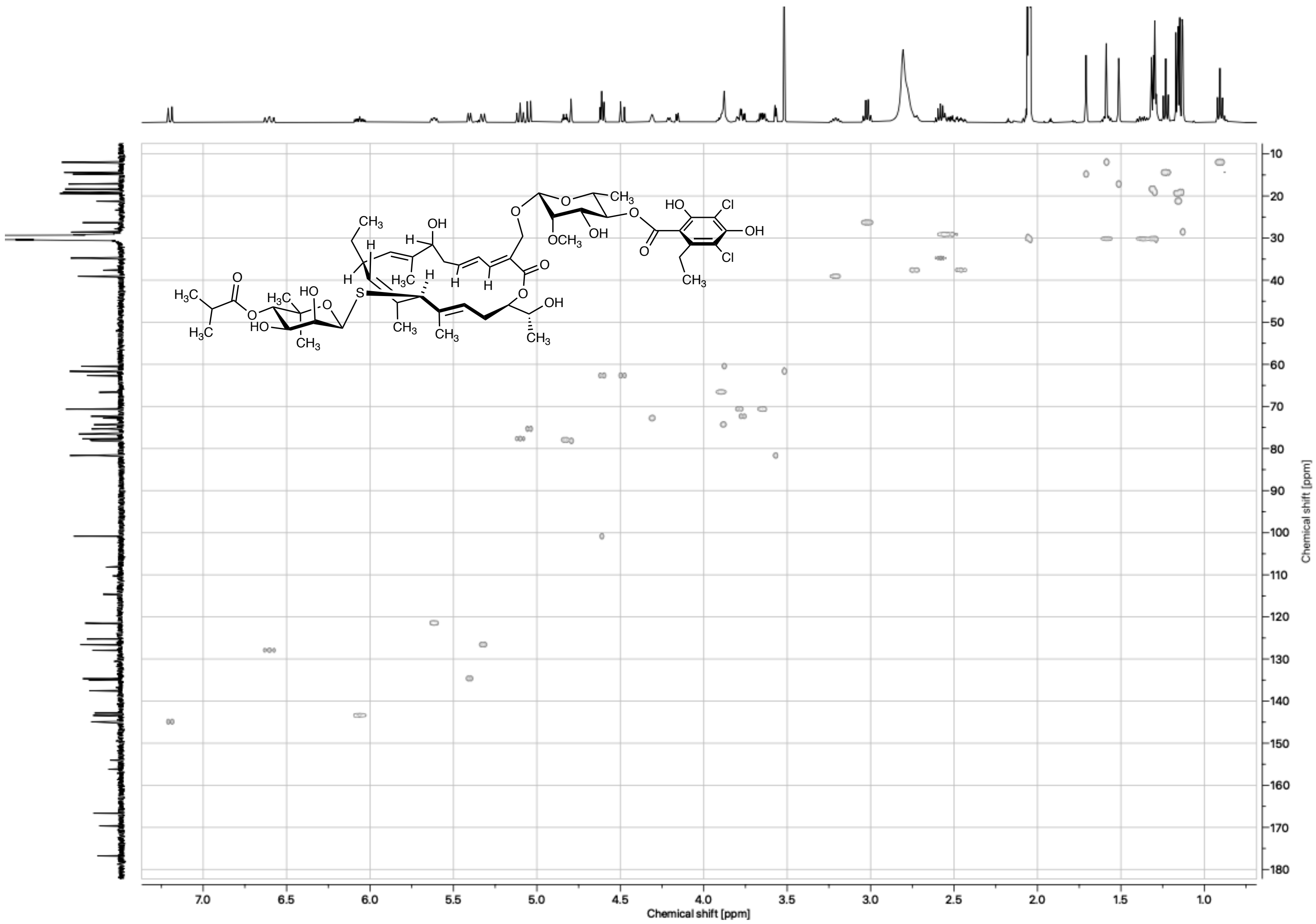

Figure 184: HSQC spectrum of spectrum of 11-desnoviosyl-13-thio-β-D-noviosyl fidaxomicin (3a-C(13)) in acetone-d_6

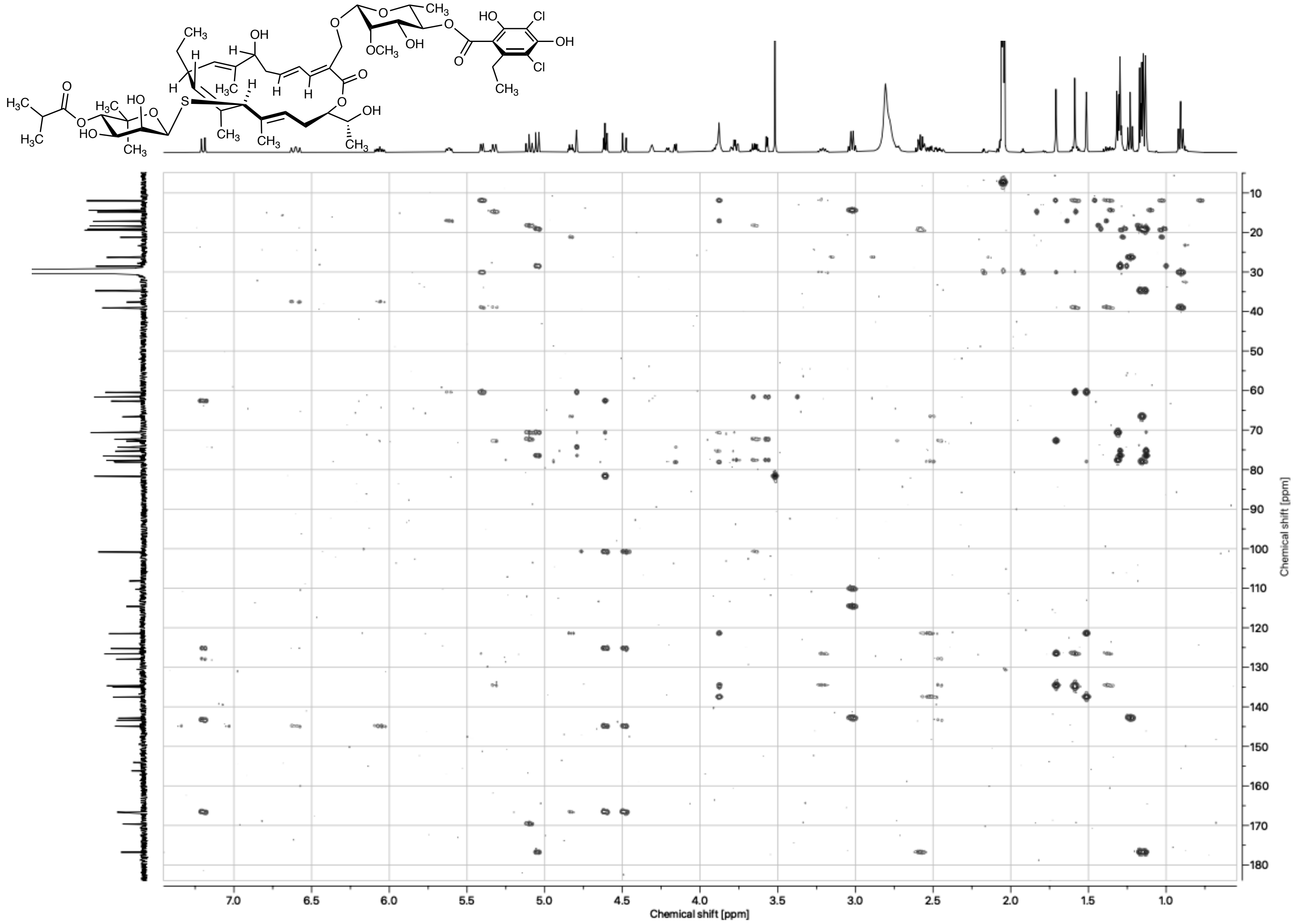

Figure 185: HMBC spectrum of spectrum of 11-desnoviosyl-13-thio-β-D-noviosyl fidaxomicin (3a-C(13)) in acetone-d_6

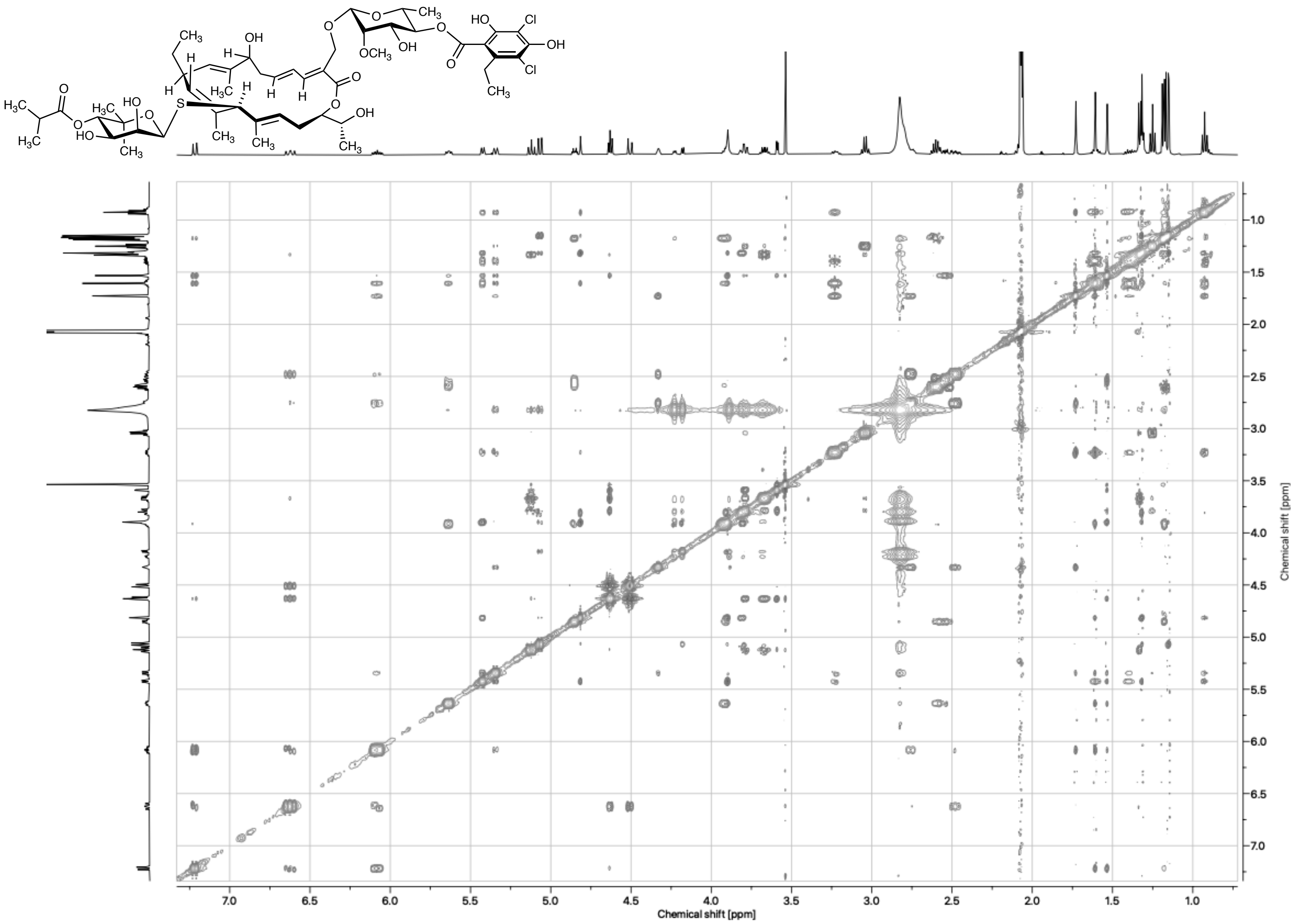

Figure 186: NOESY spectrum of spectrum of 11-desnoviosyl-13-thio-β-D-noviosyl fidaxomicin (3a-C(13)) in acetone-d_6

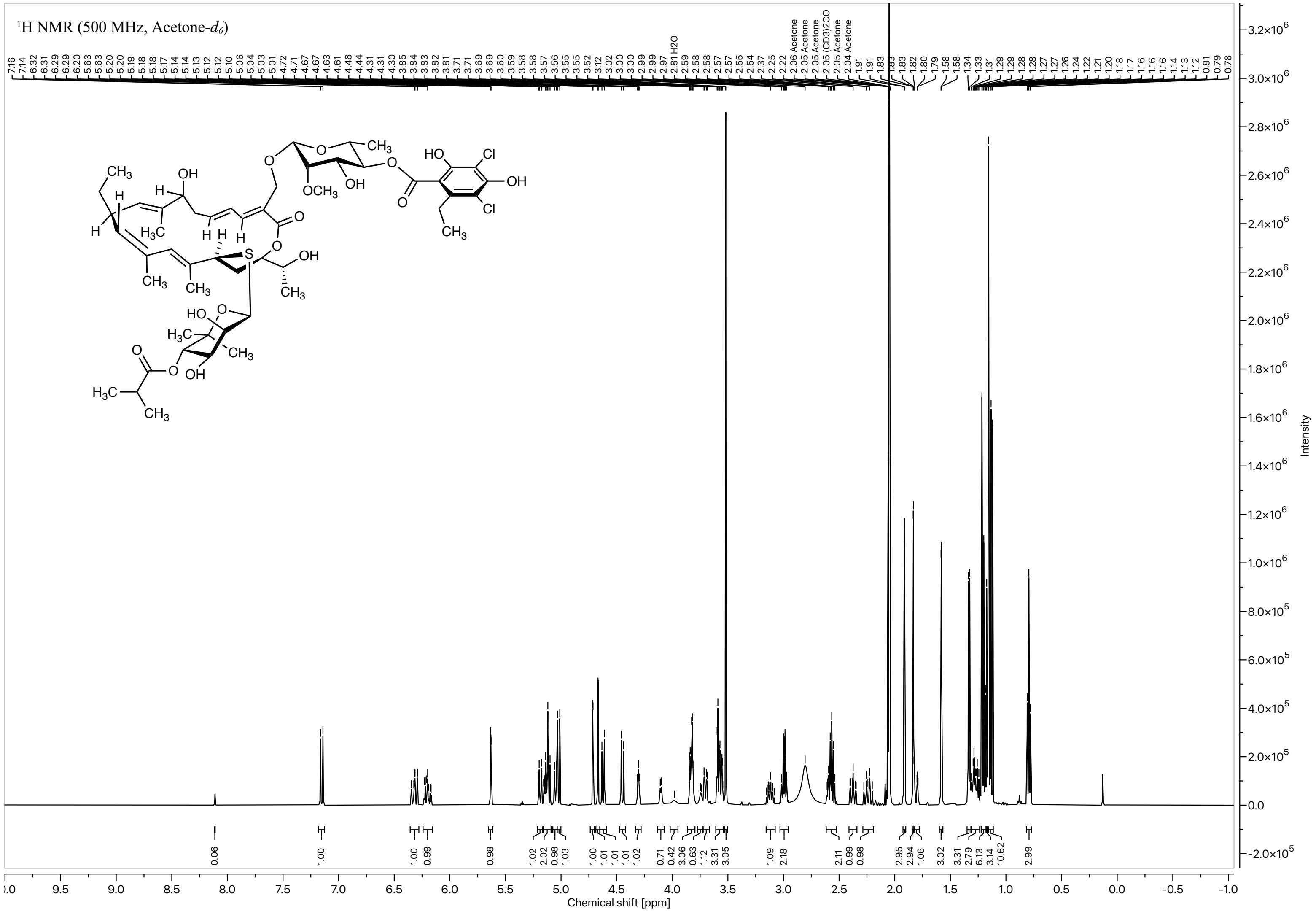

Figure 187: ^{1}H NMR spectrum of 11-desnoviosyl-15-thio-β-D-noviosyl fidaxomicin (3a-C(15)) in acetone-d_6

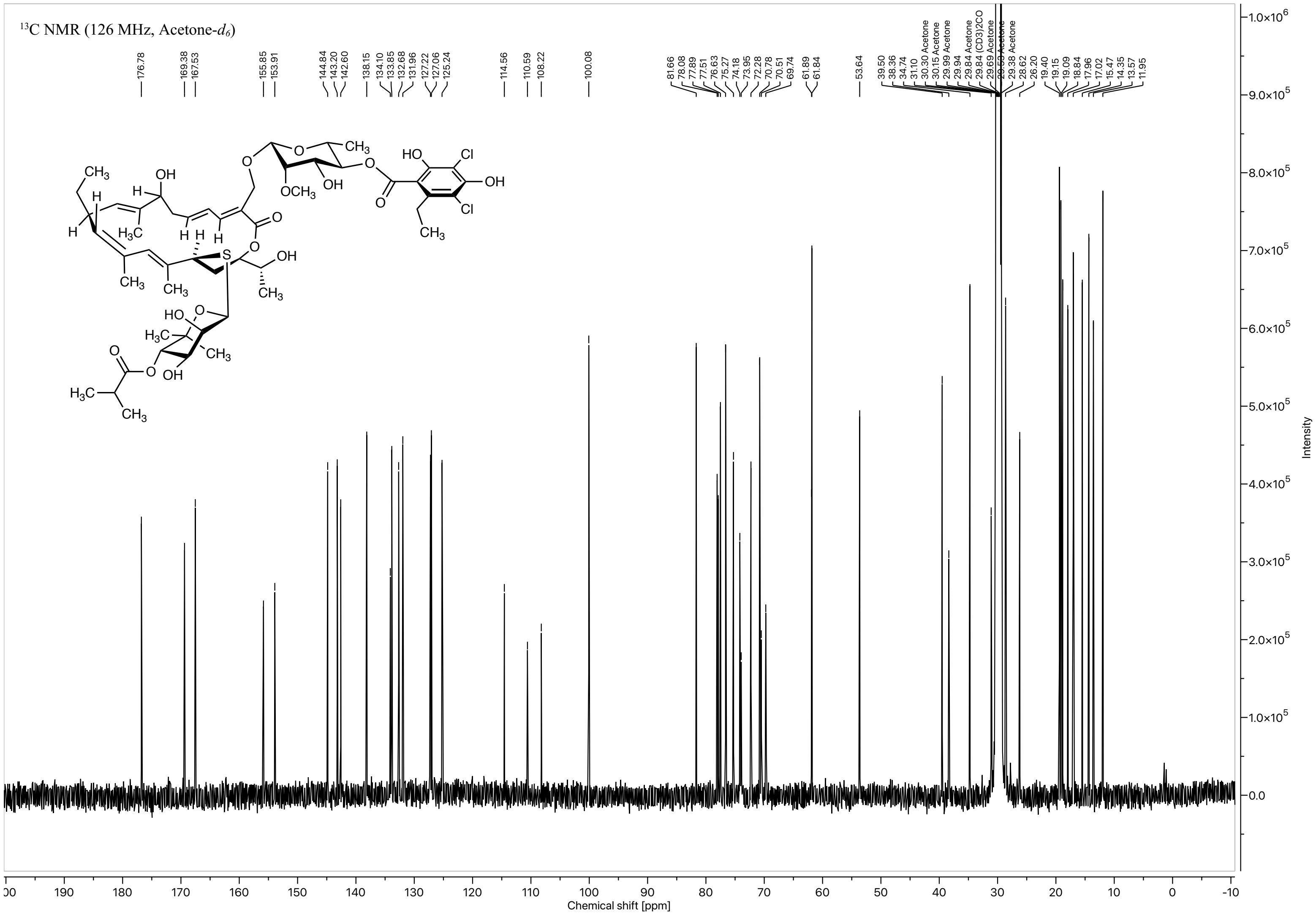

Figure 188: ^{13}C NMR spectrum of 11-desnoviosyl-15-thio-β-D-noviosyl fidaxomicin (3a-C(15)) in acetone-d_6

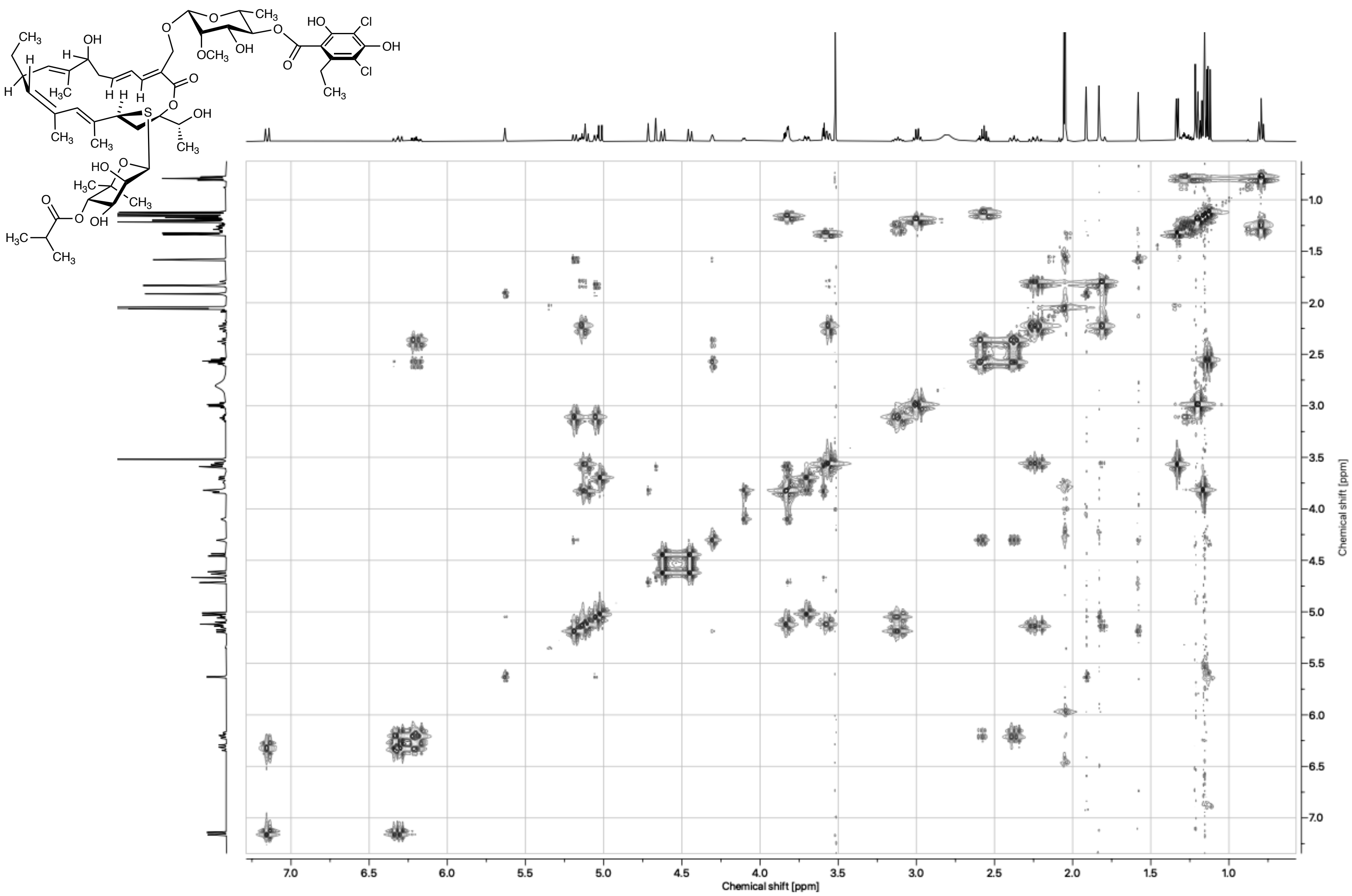

Figure 189: COSY spectrum of 11-desnoviosyl-15-thio-β-D-noviosyl fidaxomicin (3a-C(15)) in acetone-d_6

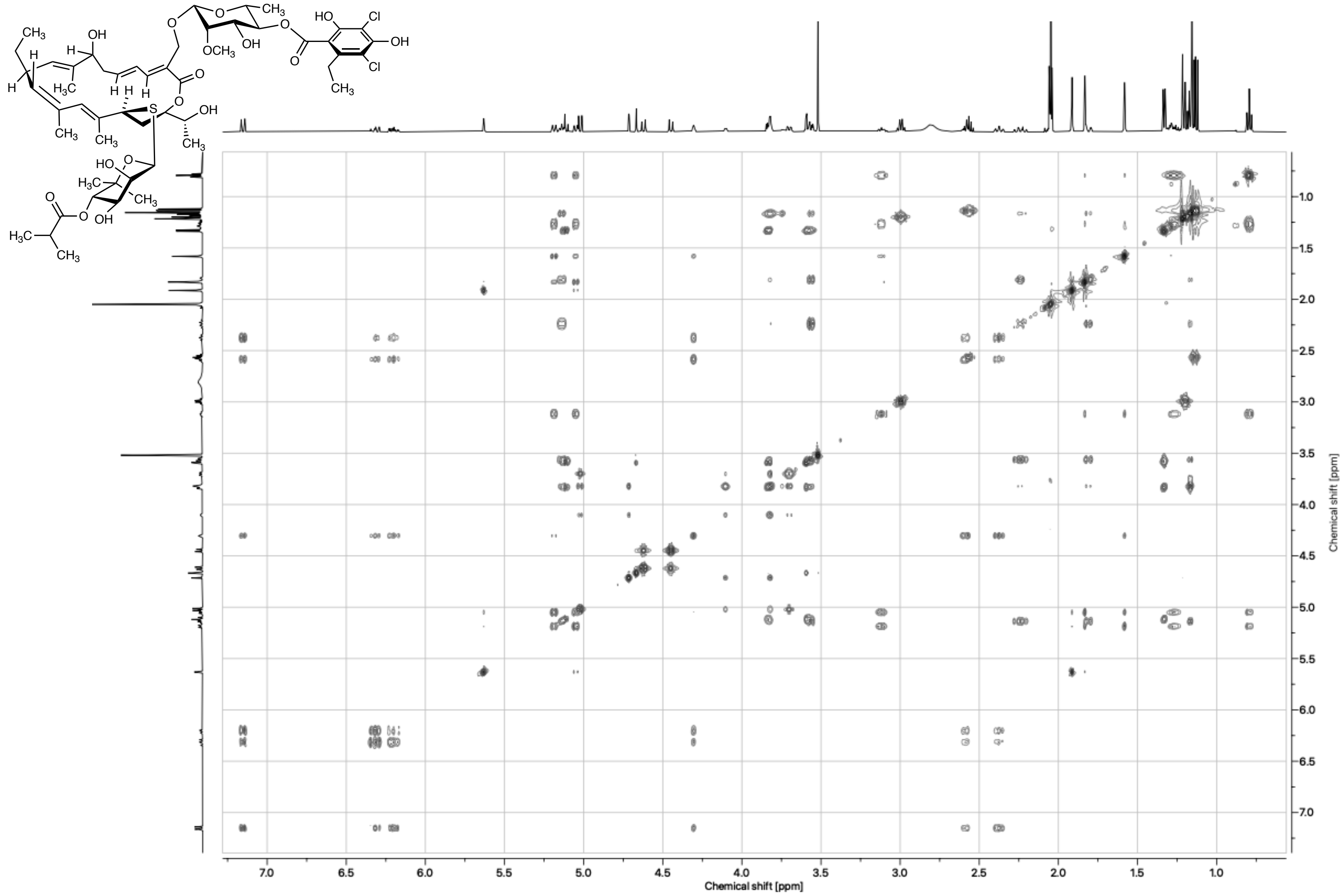

Figure 190: TOCSY spectrum of 11-desnoviosyl-15-thio-β-D-noviosyl fidaxomicin (3a-C(15)) in acetone-d_6

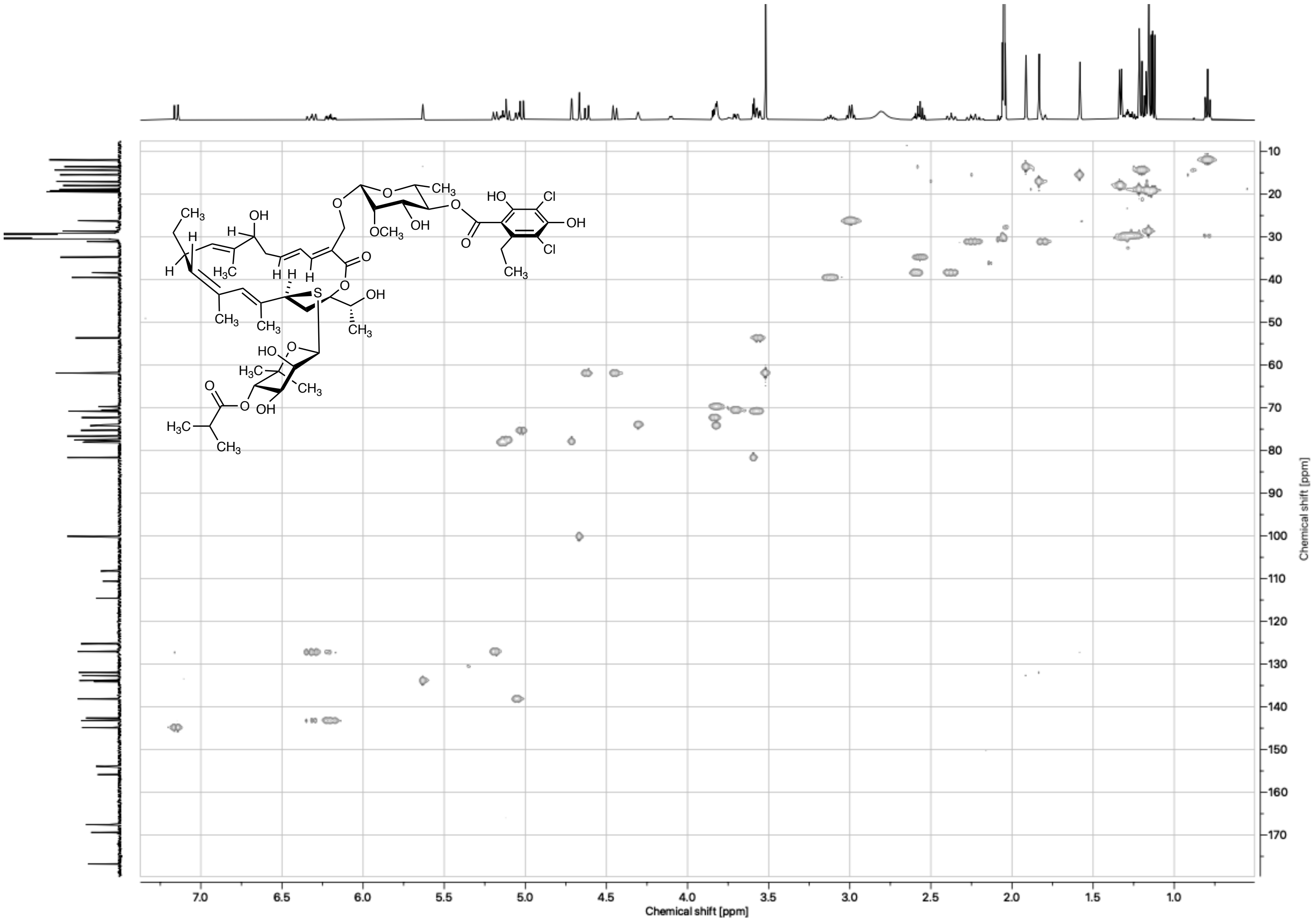

Figure 191: HSQC spectrum of 11-desnoviosyl-15-thio-β-D-noviosyl fidaxomicin (3a-C(15)) in acetone-d_6

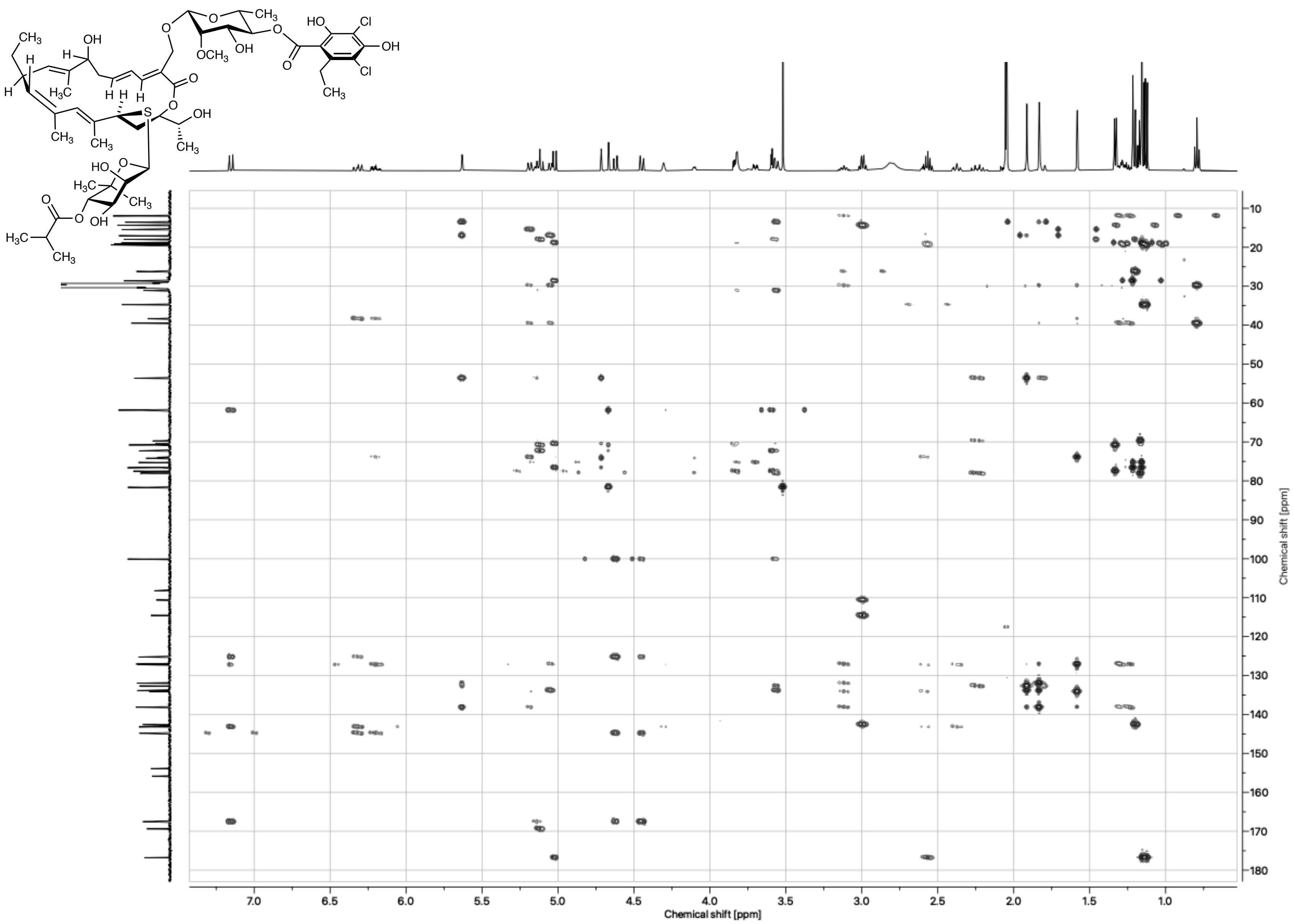

Figure 192: HMBC spectrum of 11-desnoviosyl-15-thio-β-D-noviosyl fidaxomicin (3a-C(15)) in acetone-d_6

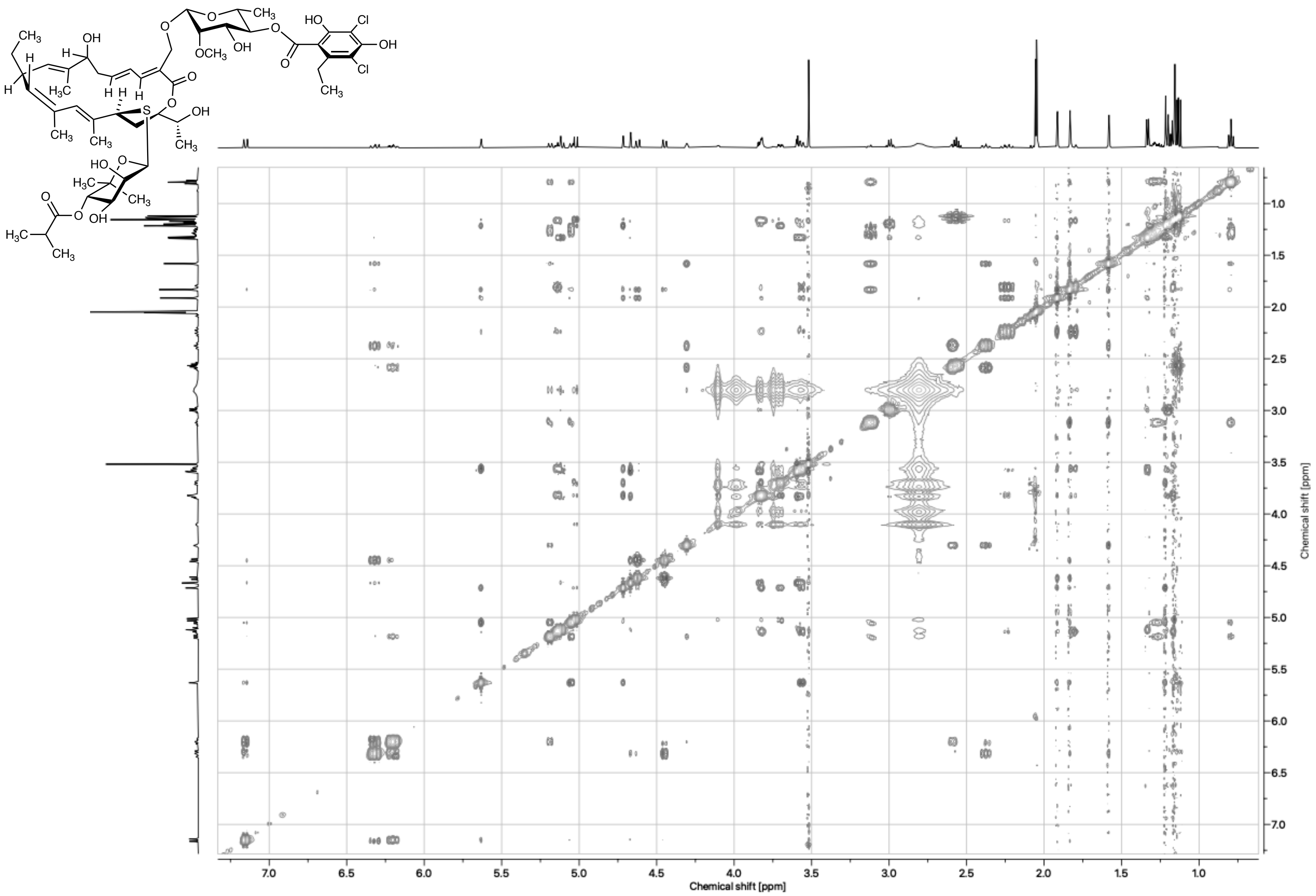

Figure 193: NOESY spectrum of 11-desnoviosyl-15-thio-β-D-noviosyl fidaxomicin (3a-C(15)) in acetone-d_6

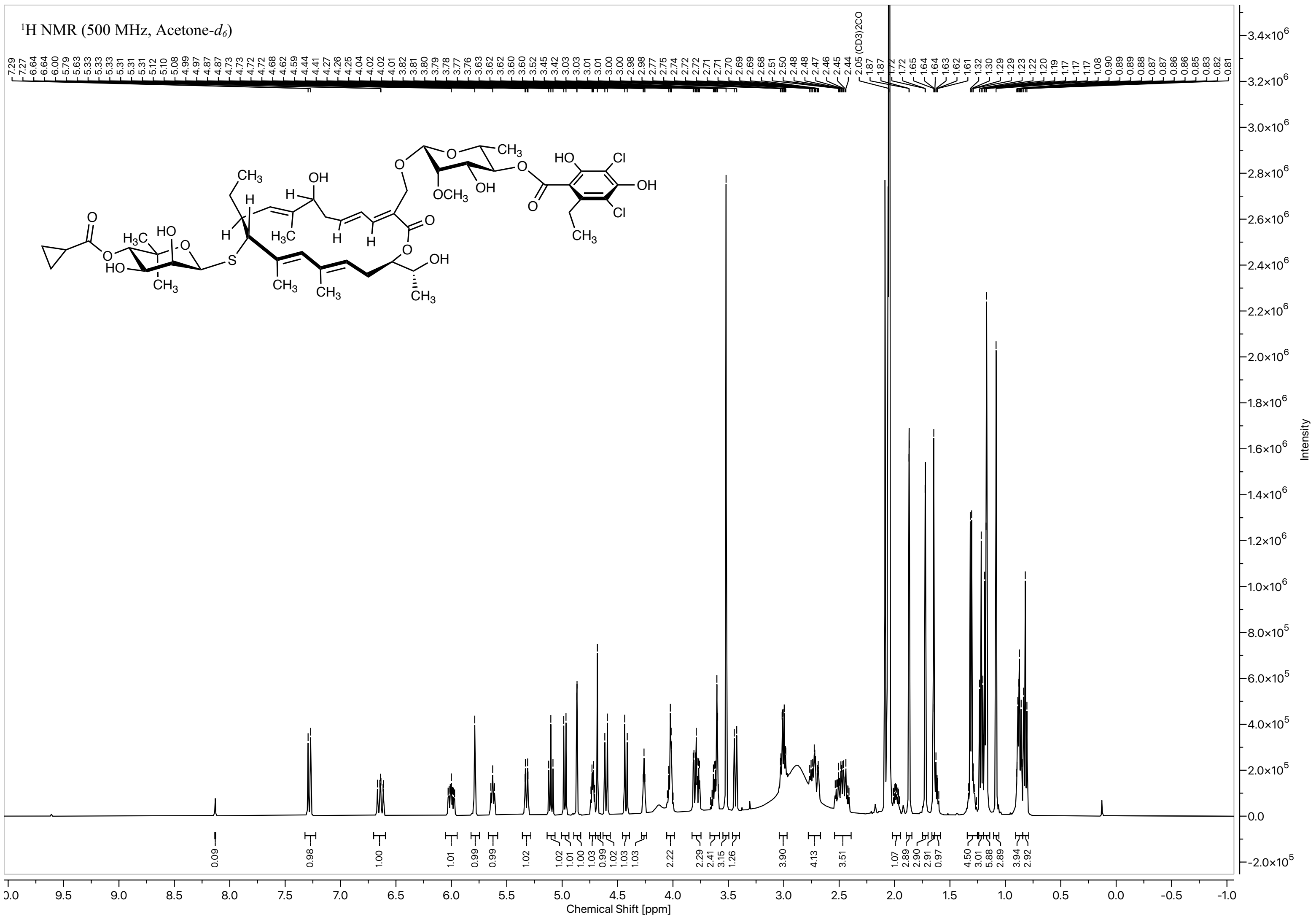

Figure 194: ^{1}H NMR spectrum of 11-desnoviosyl-11-4"-O-cyclopropanoyl-thio-β-D-noviosyl fidaxomicin (3b-C(11)) in acetone-d_6

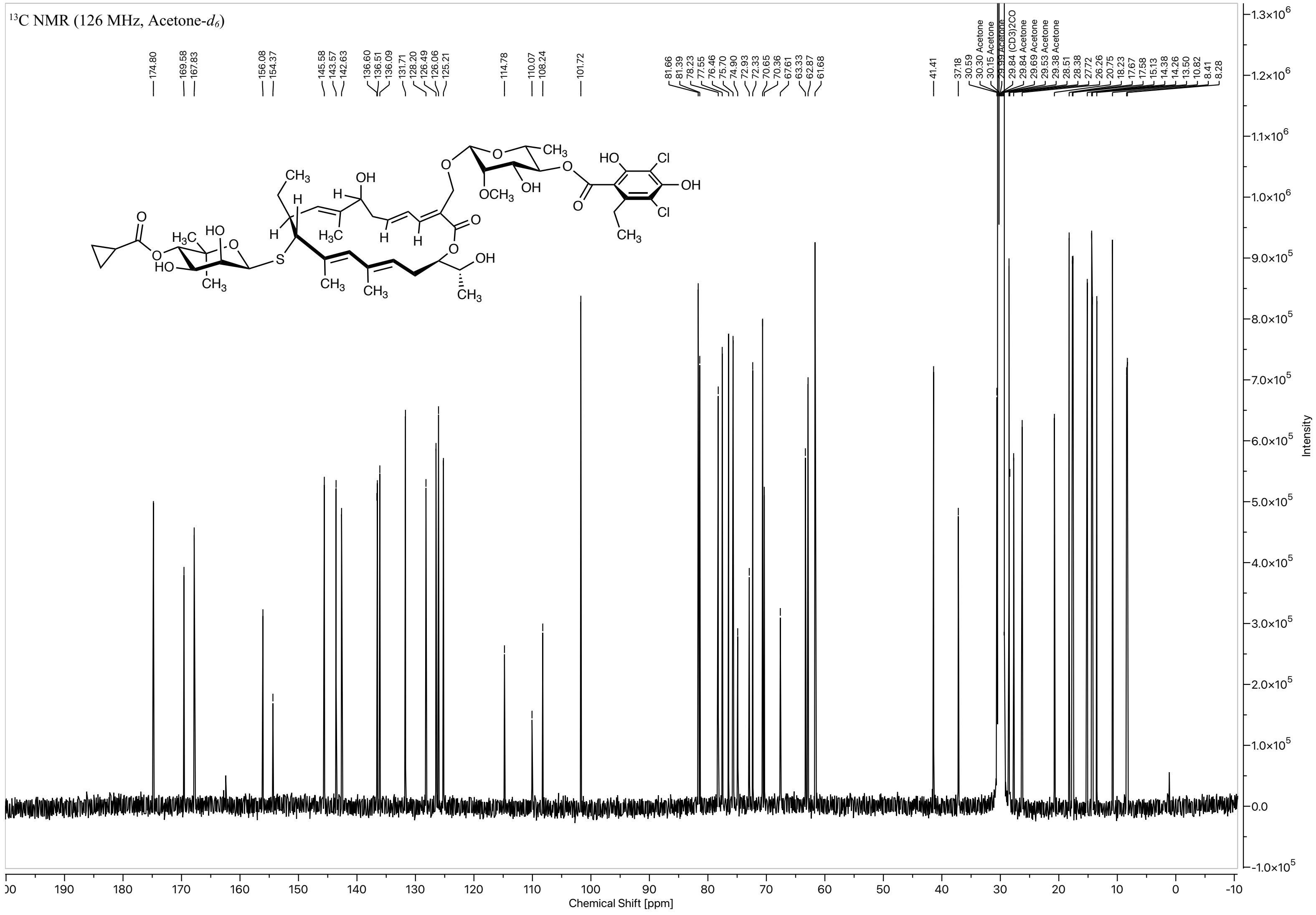

Figure 195: 13C NMR spectrum of 11-desnoviosyl-11-4”-O-cyclopropanoyl-thio-β-D-noviosyl fidaxomicin (3b-C(11)) in acetone-d_6

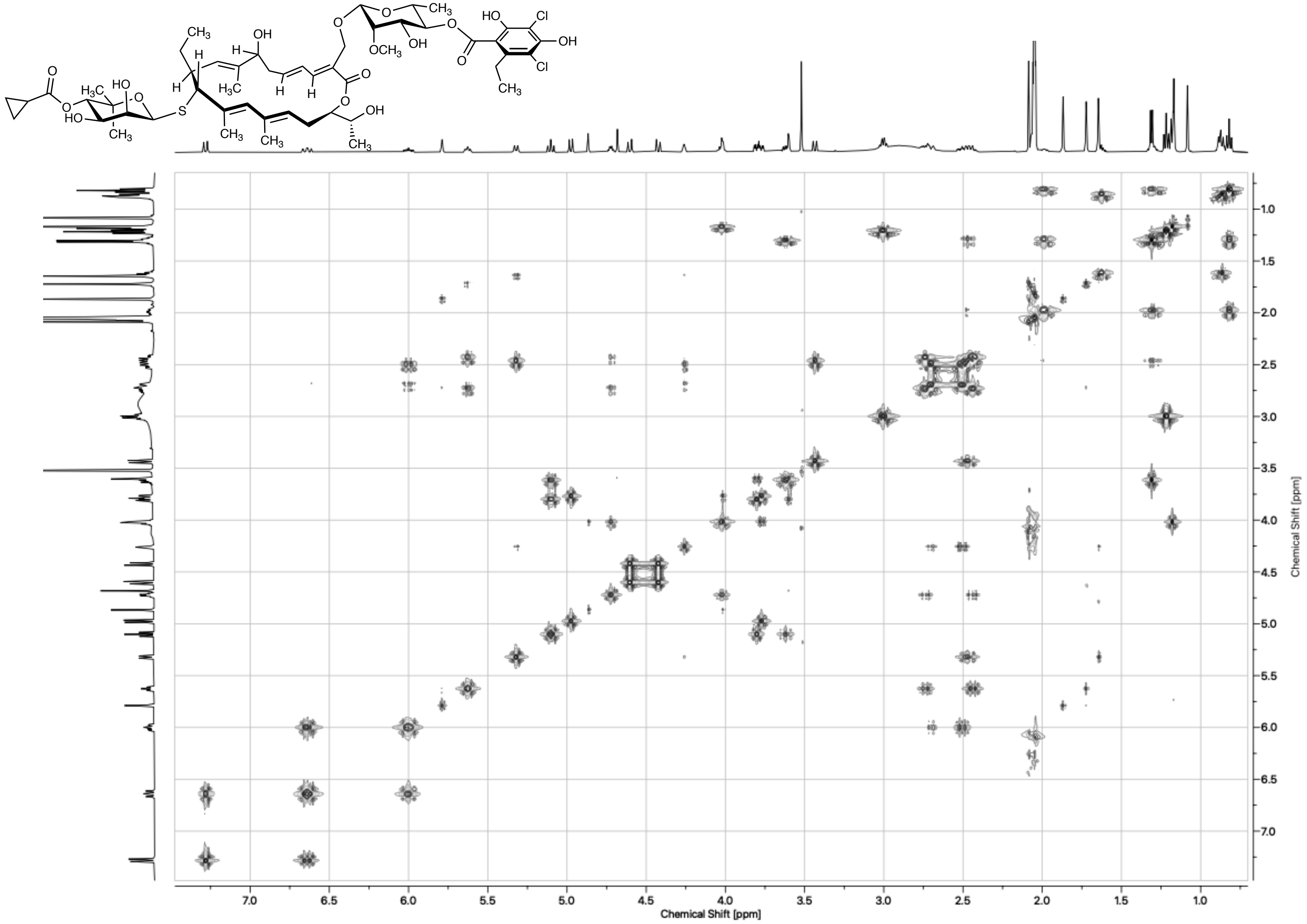

Figure 196: COSY spectrum of 11-desnoviosyl-11-4”-O-cyclopropanoyl-thio-β-D-noviosyl fidaxomicin (3b-C(11)) in acetone-d_6

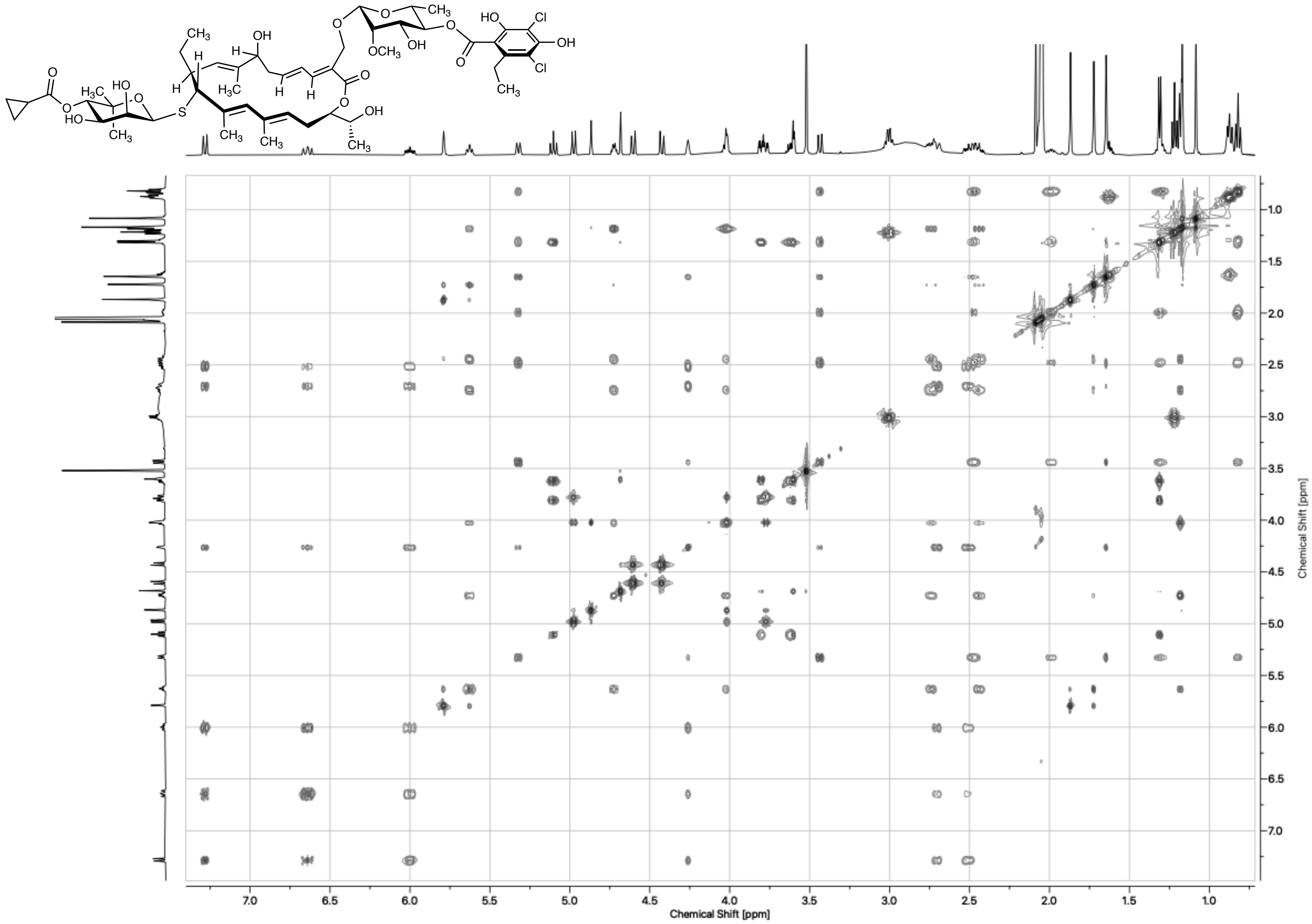

Figure 197: TOCSY spectrum of 11-desnoviosyl-11-4”-O-cyclopropanoyl-thio-β-D-noviosyl fidaxomicin (3b-C(11)) in acetone-d_6

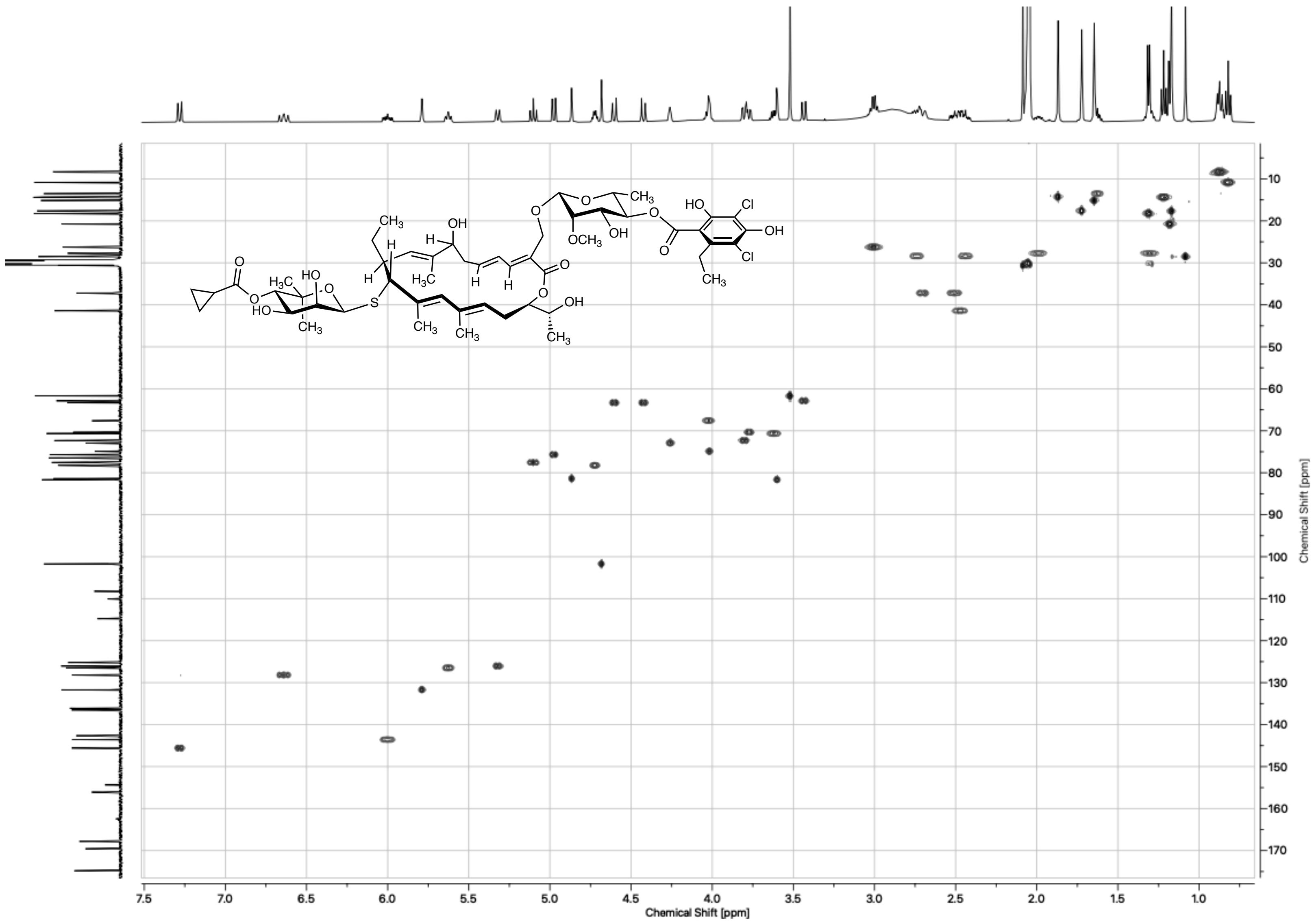

Figure 198: HSQC spectrum of 11-desnoviosyl-11-4”-O-cyclopropanoyl-thio-β-D-noviosyl fidaxomicin (3b-C(11)) in acetone-d_6

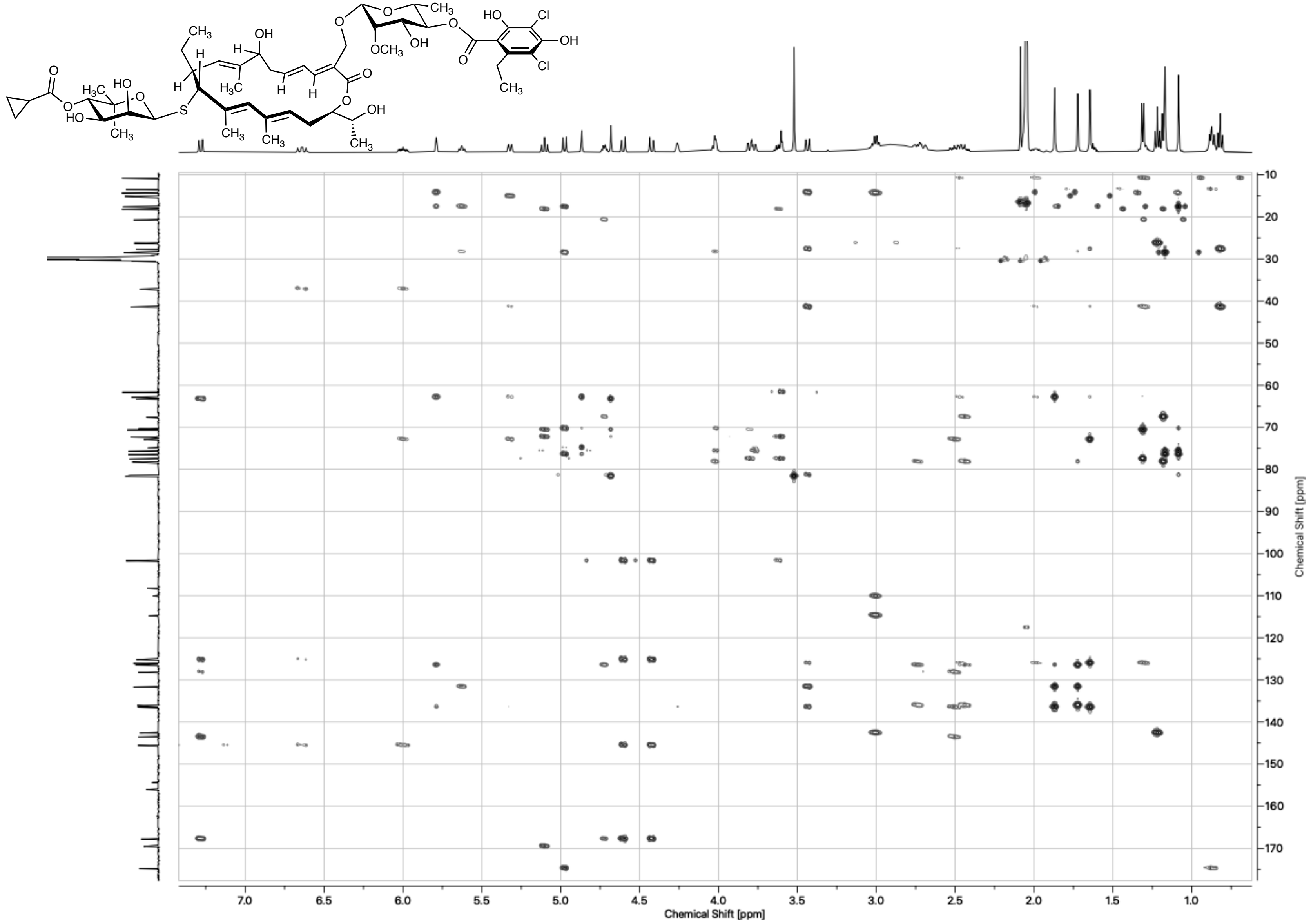

Figure 199: HMBC spectrum of 11-desnoviosyl-11-4''-O-cyclopropanoyl-thio-β-D-noviosyl fidaxomicin (3b-C(11)) in acetone-d_6

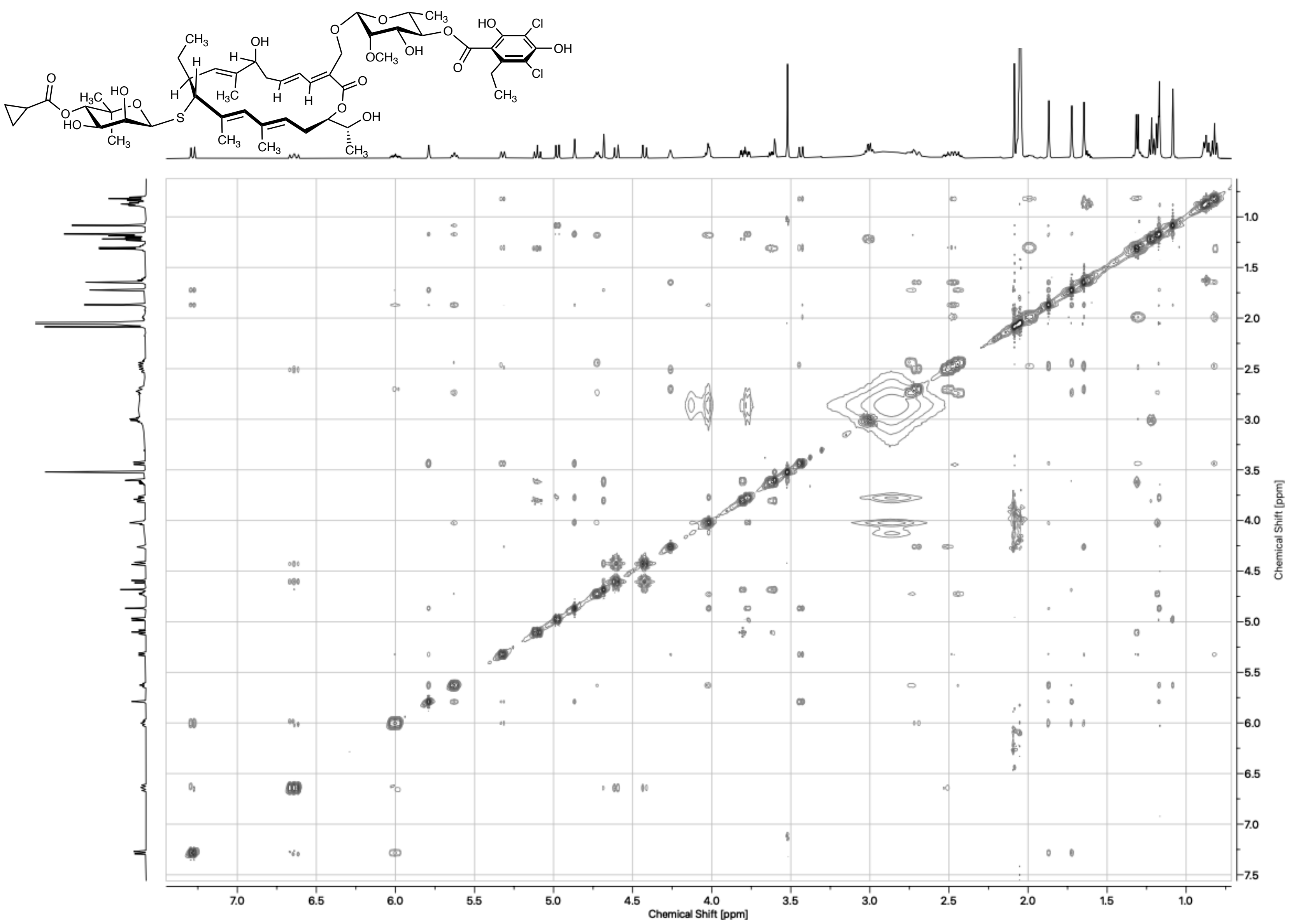

Figure 200: NOESY spectrum of 11-desnoviosyl-11-4”-O-cyclopropanoyl-thio-β-D-noviosyl fidaxomicin (3b-C(11)) in acetone-d_6

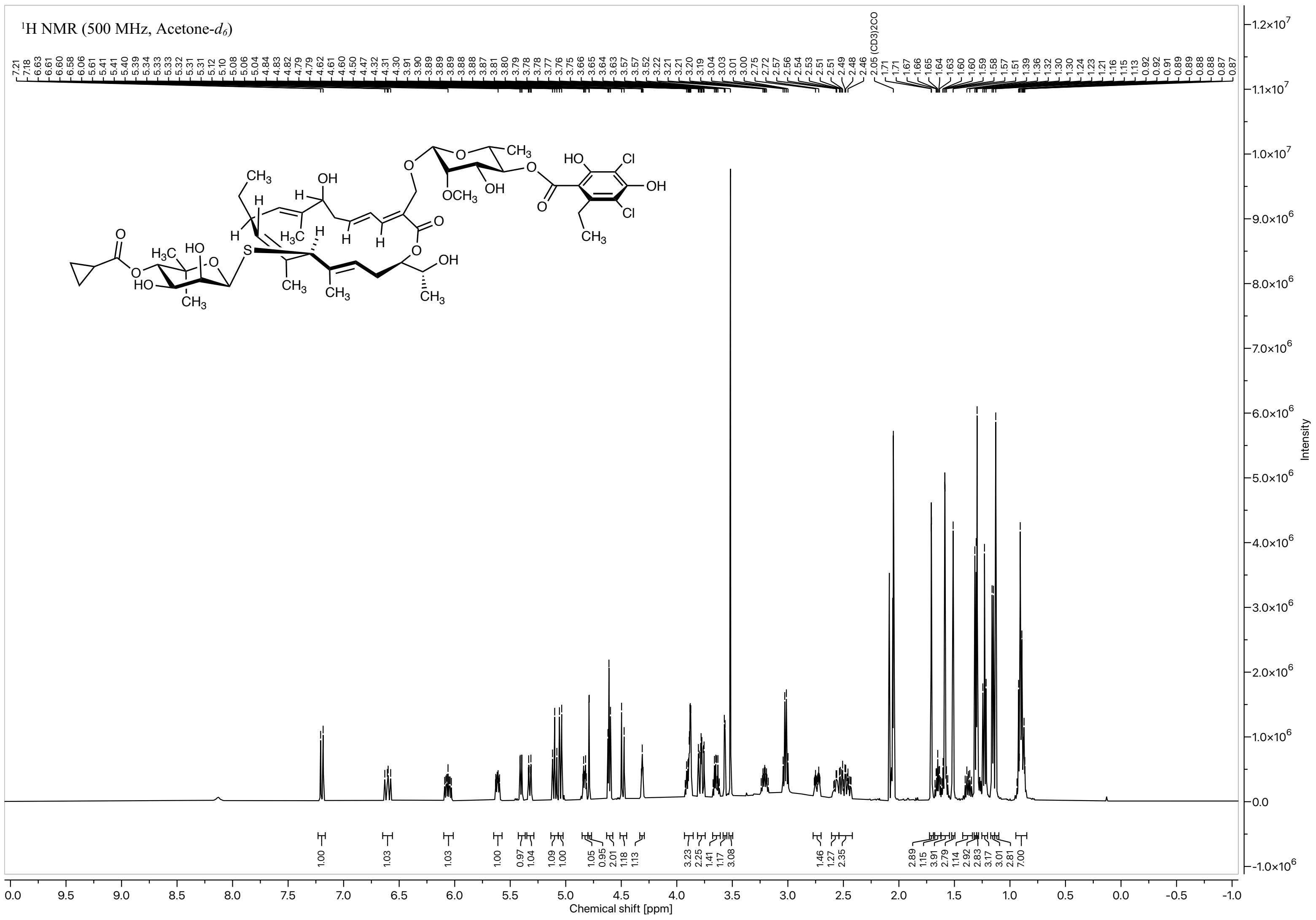

Figure 201: ^{1}H NMR spectrum of 11-desnoviosyl-13-4”-O-cyclopropanoyl-thio-β-D-noviosyl fidaxomicin (3b-C(13)) in acetone-d_6

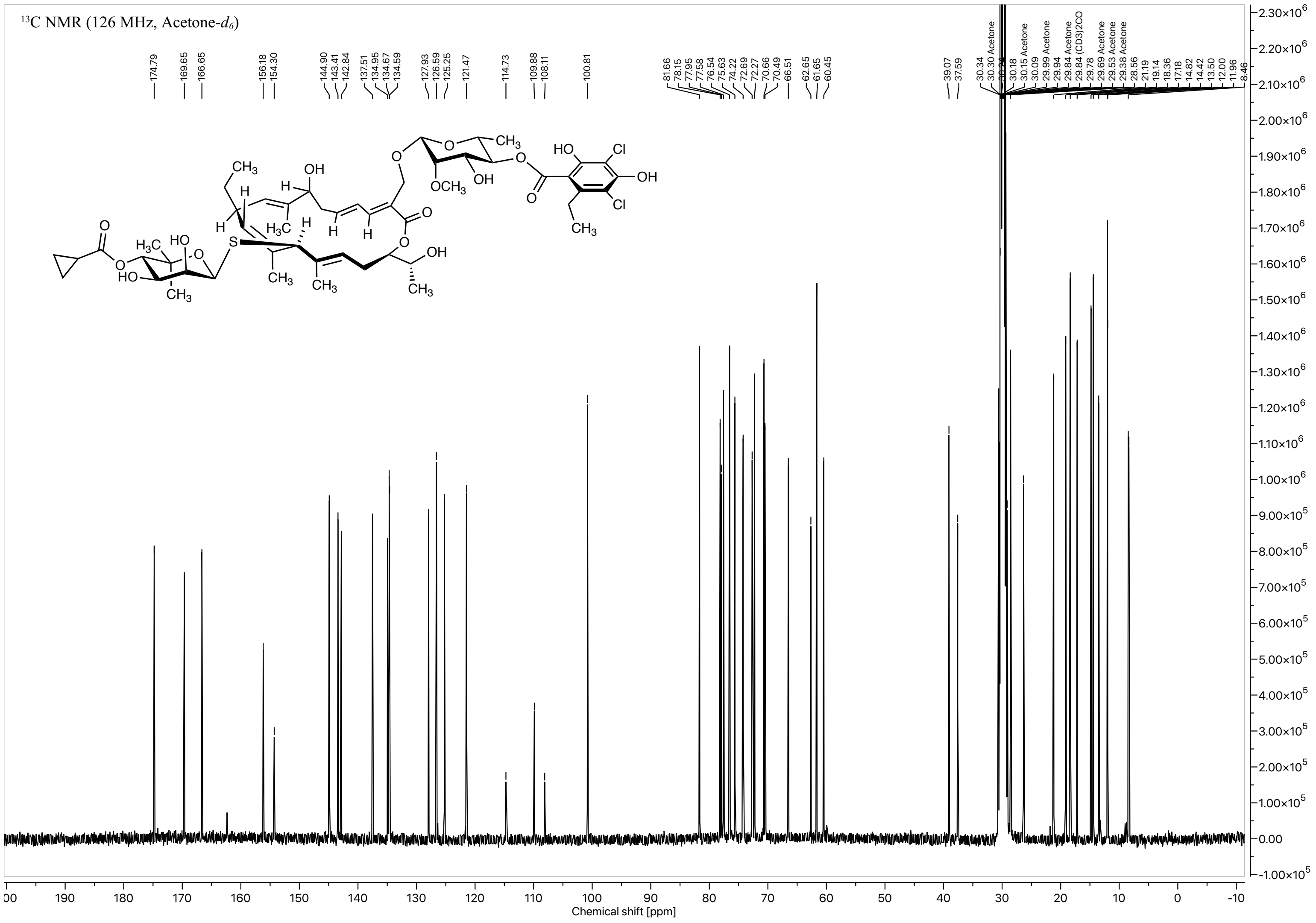

Figure 202: ^{13}C NMR spectrum of 11-desnoviosyl-13-4”-O-cyclopropanoyl-thio-β-D-noviosyl fidaxomicin (3b-C(13)) in acetone-d_6

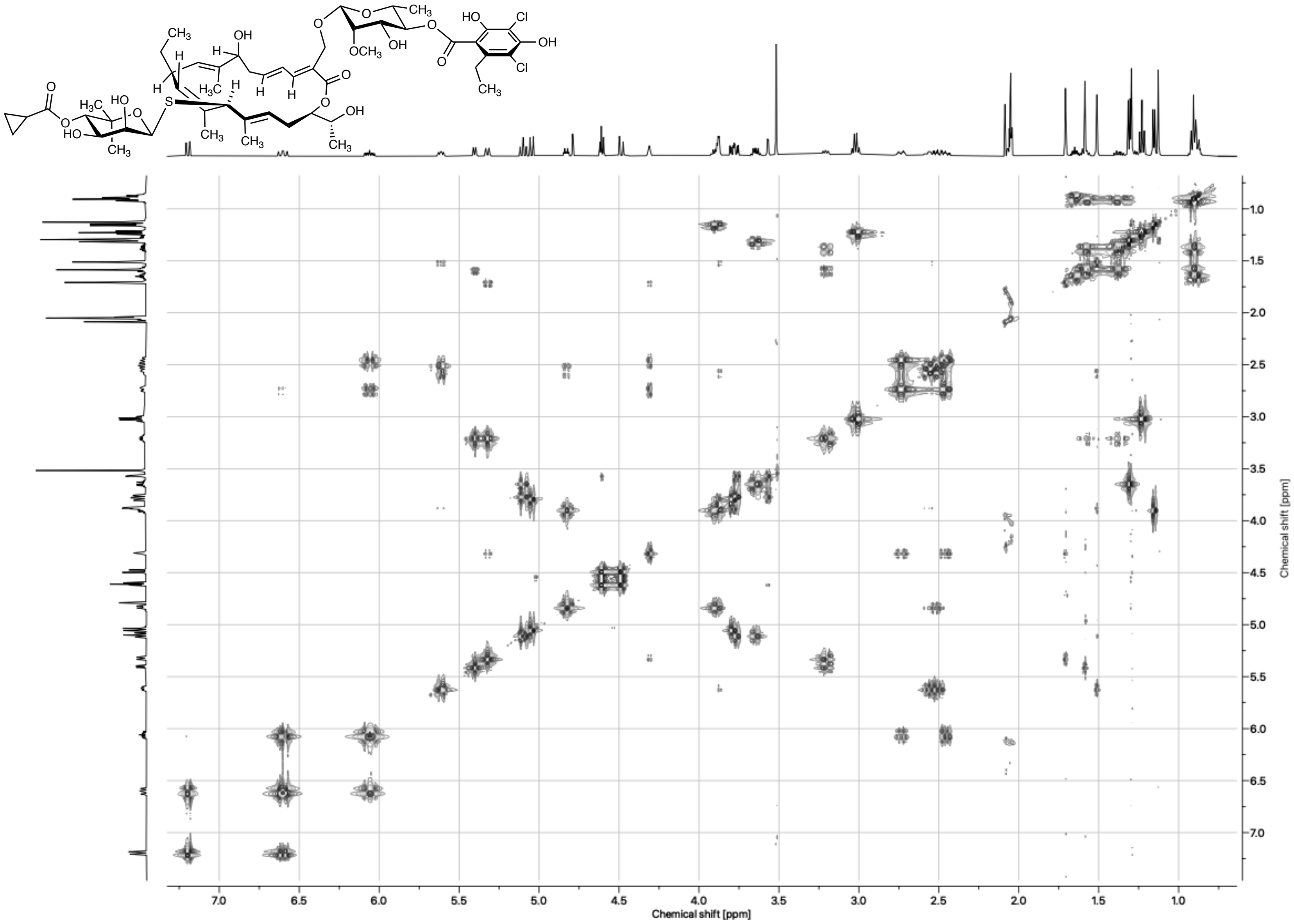

Figure 203: COSY spectrum of 11-desnoviosyl-13-4”-O-cyclopropanoyl-thio-β-D-noviosyl fidaxomicin (3b-C(13)) in acetone-d_6

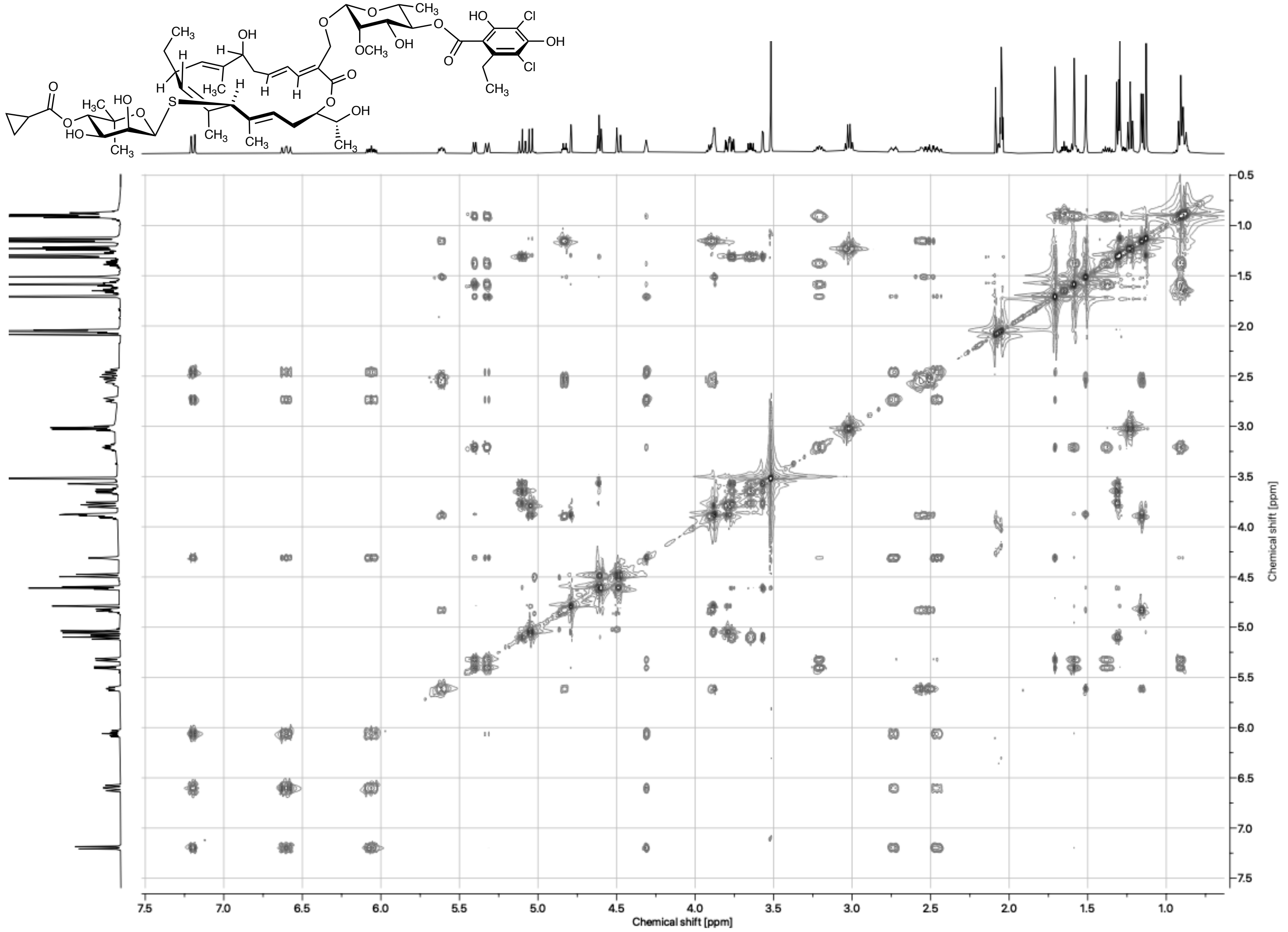

Figure 204: TOCSY spectrum of 11-desnoviosyl-13-4”-O-cyclopropanoyl-thio-β-D-noviosyl fidaxomicin (3b-C(13)) in acetone-d_6

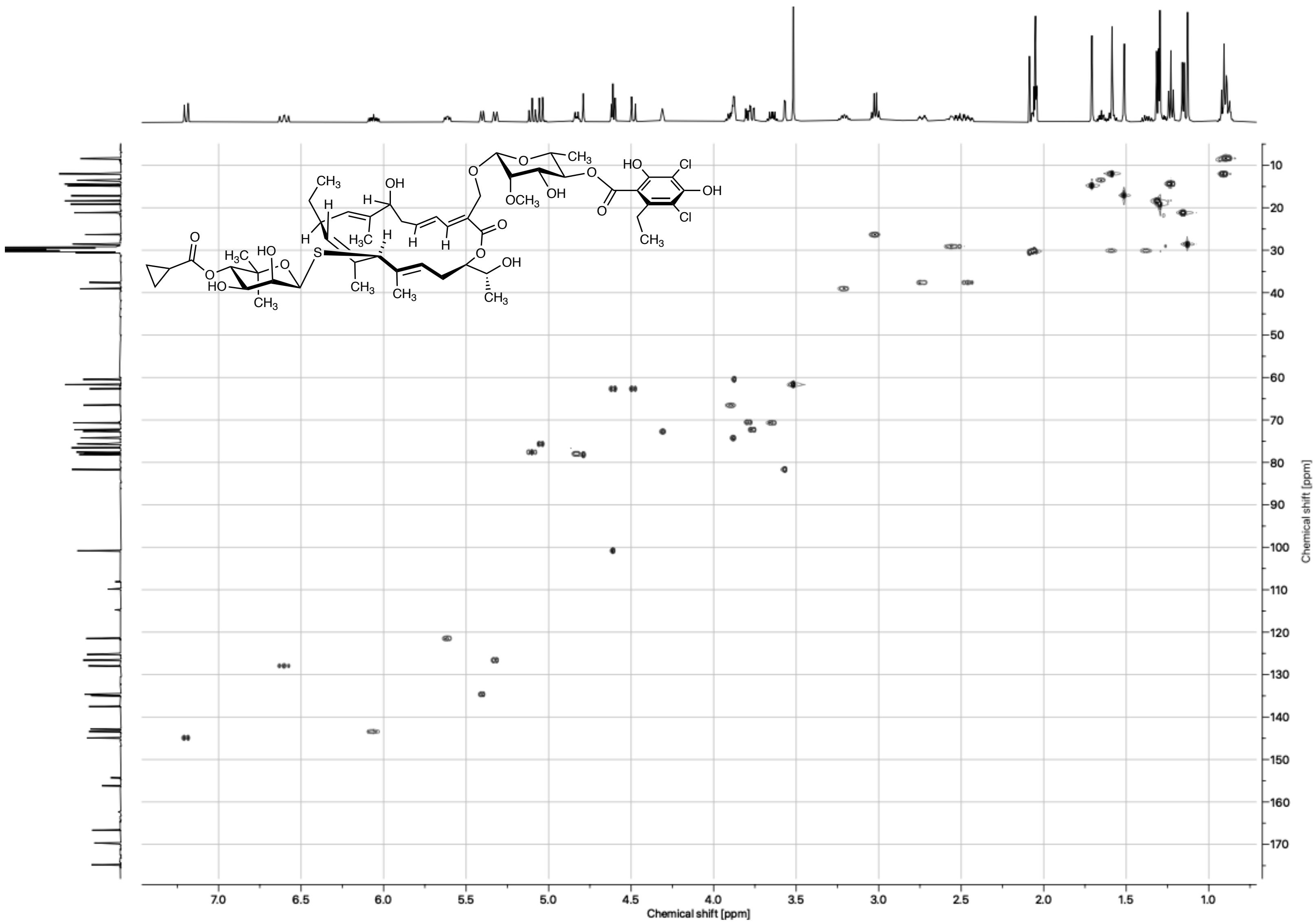

Figure 205: HSQC spectrum of 11-desnoviosyl-13-4”-O-cyclopropanoyl-thio-β-D-noviosyl fidaxomicin (3b-C(13)) in acetone-d_6

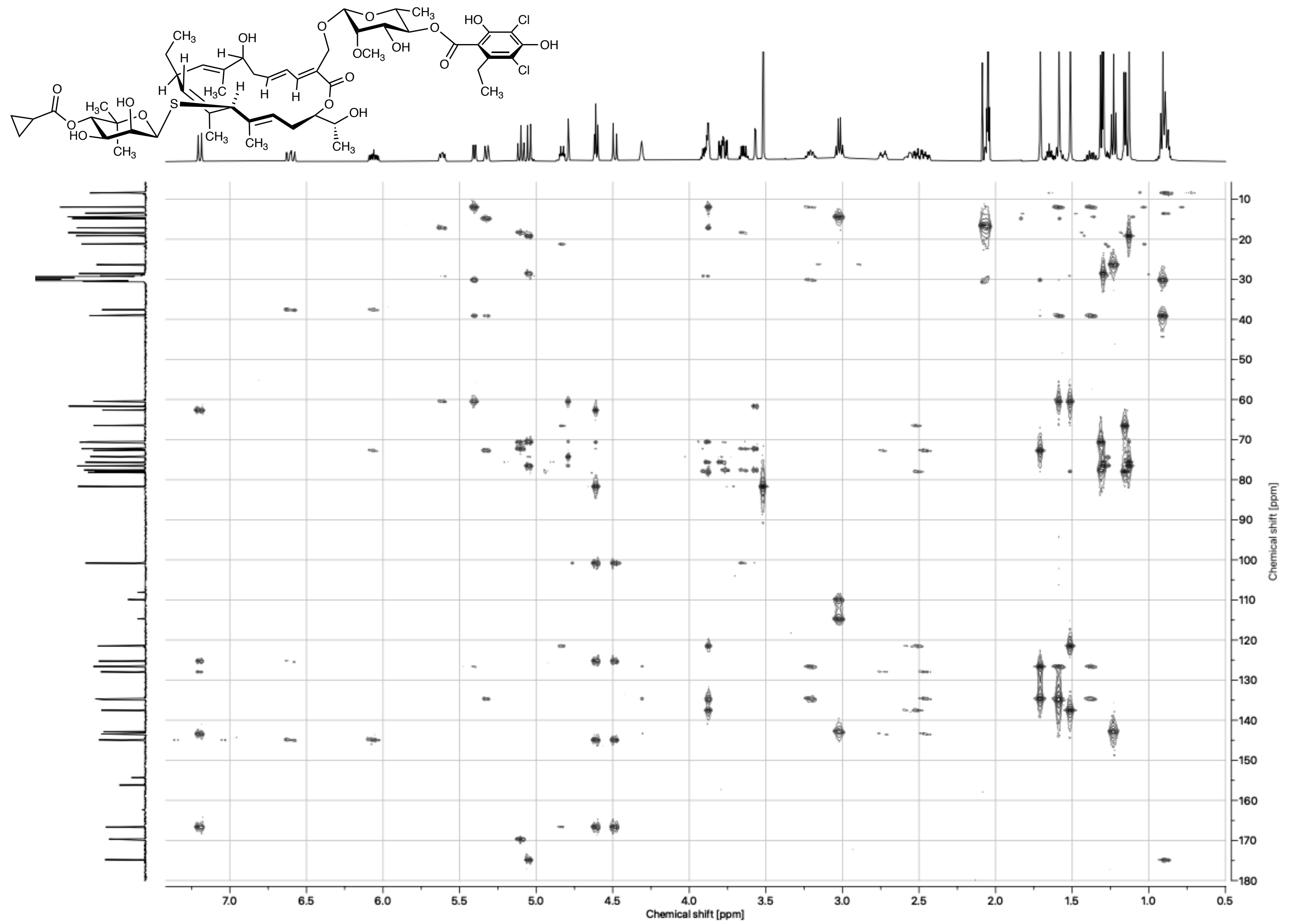

Figure 206: HMBC spectrum of 11-desnoviosyl-13-4”-O-cyclopropanoyl-thio-β-D-noviosyl fidaxomicin (3b-C(13)) in acetone-d_6

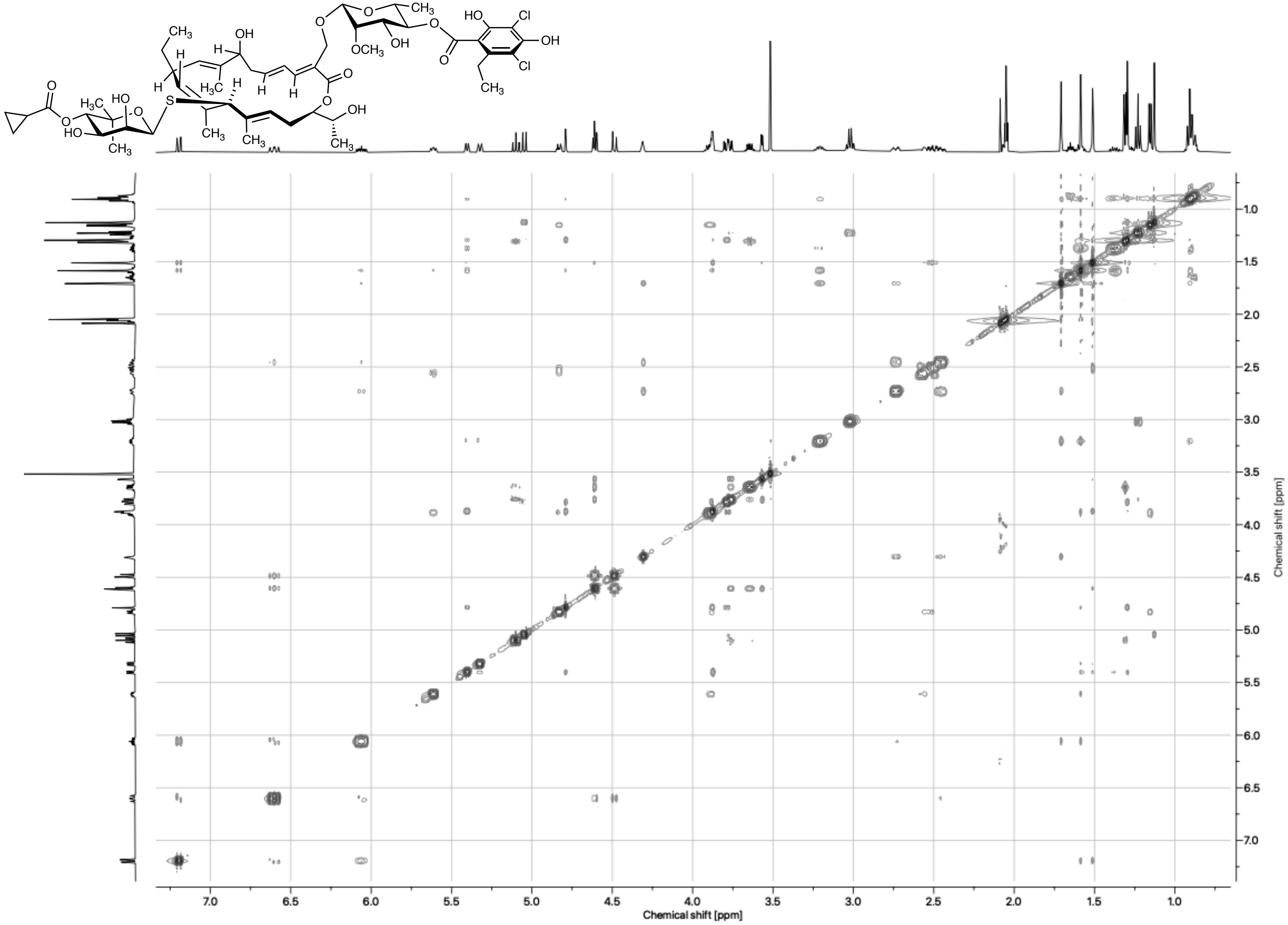

Figure 207: NOESY spectrum of 11-desnoviosyl-13-4”-O-cyclopropanoyl-thio-β-D-noviosyl fidaxomicin (3b-C(13)) in acetone-d_6

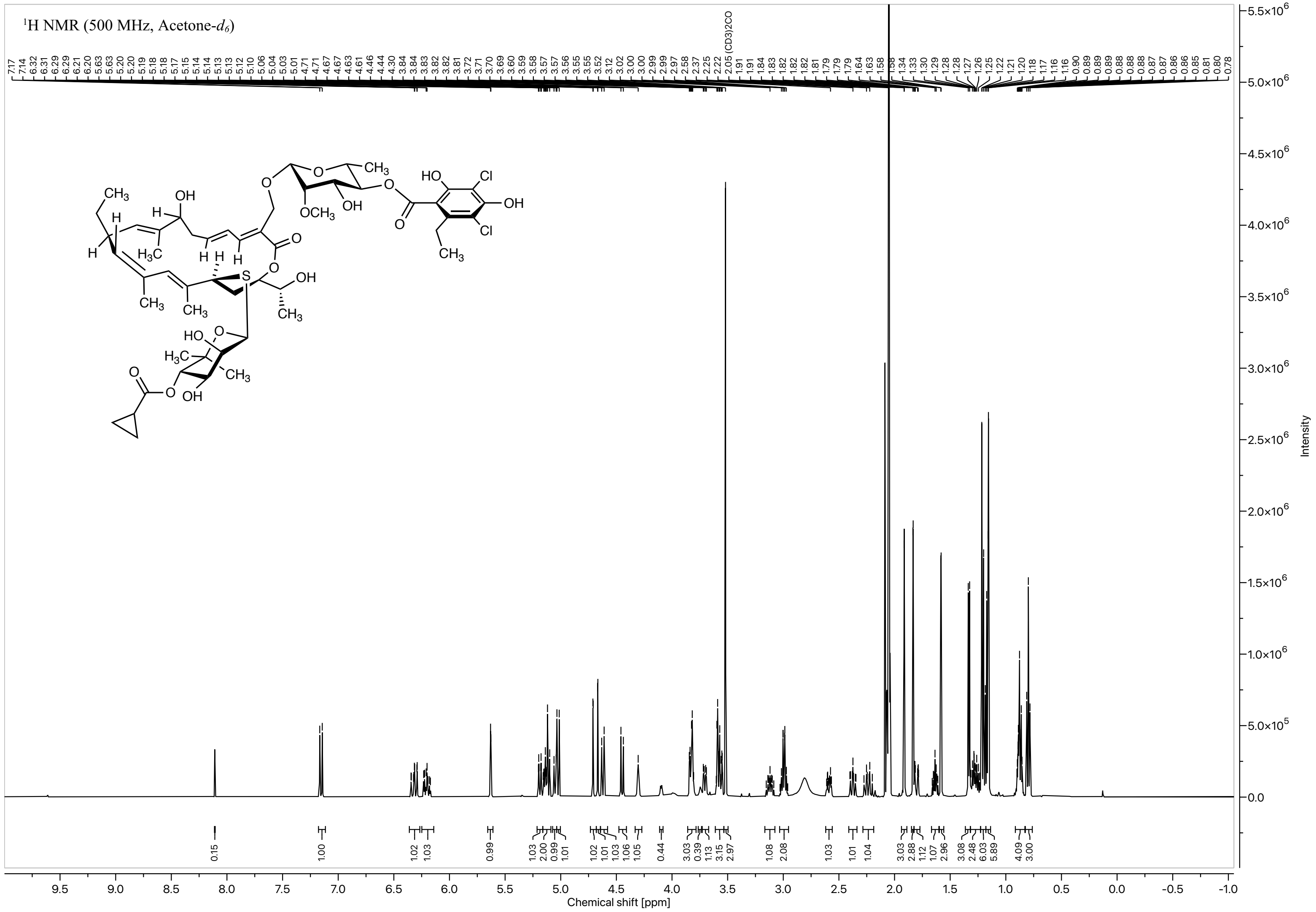

Figure 208: ^{1}H NMR spectrum of 11-desnoviosyl-15-4”-O-cyclopropanoyl-thio-β-D-noviosyl fidaxomicin (3b-C(15)) in acetone-d_6

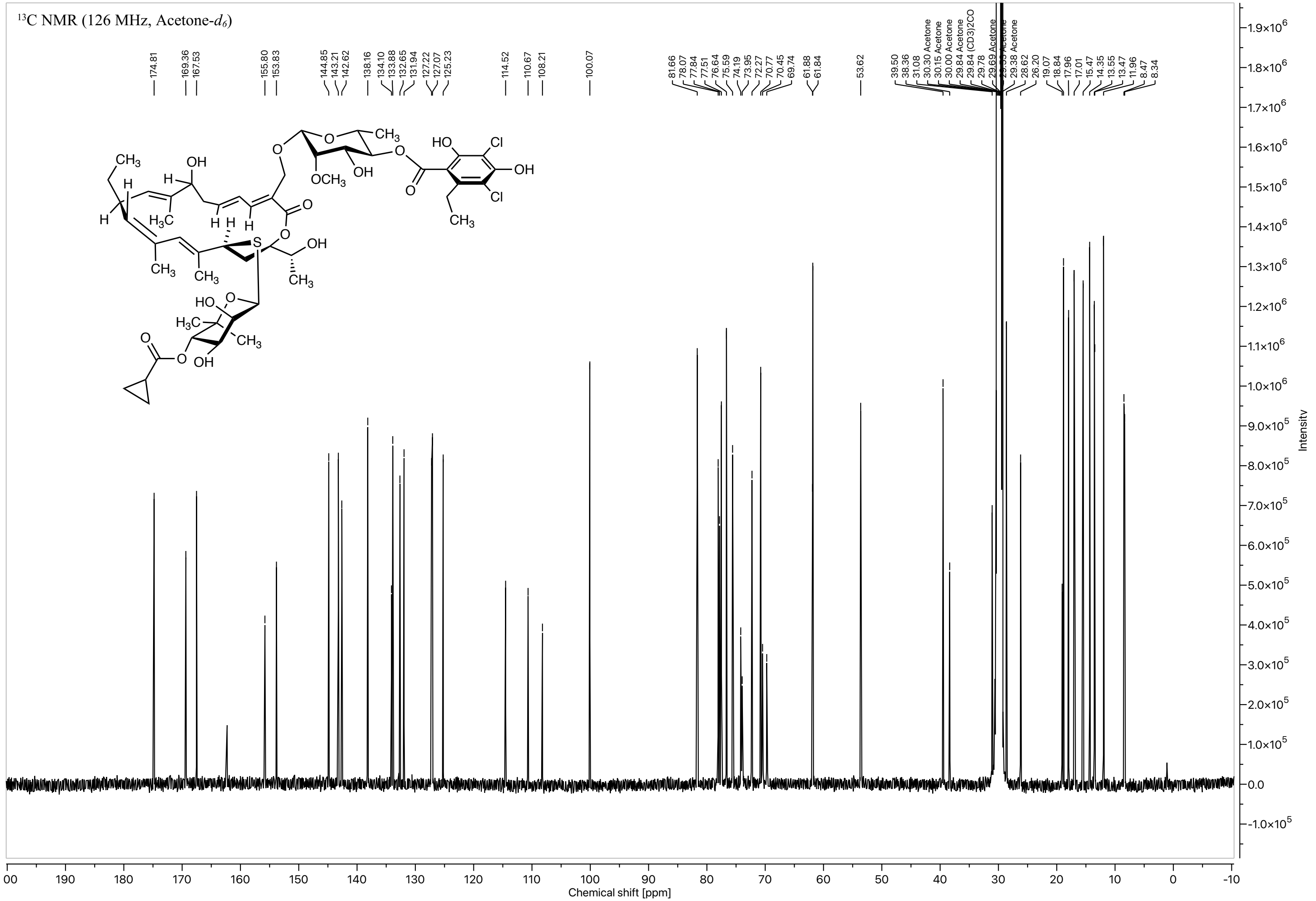

Figure 209: ^{13}C NMR spectrum of 11-desnoviosyl-15-4”-O-cyclopropanoyl-thio-β-D-noviosyl fidaxomicin (3b-C(15)) in acetone-d_6

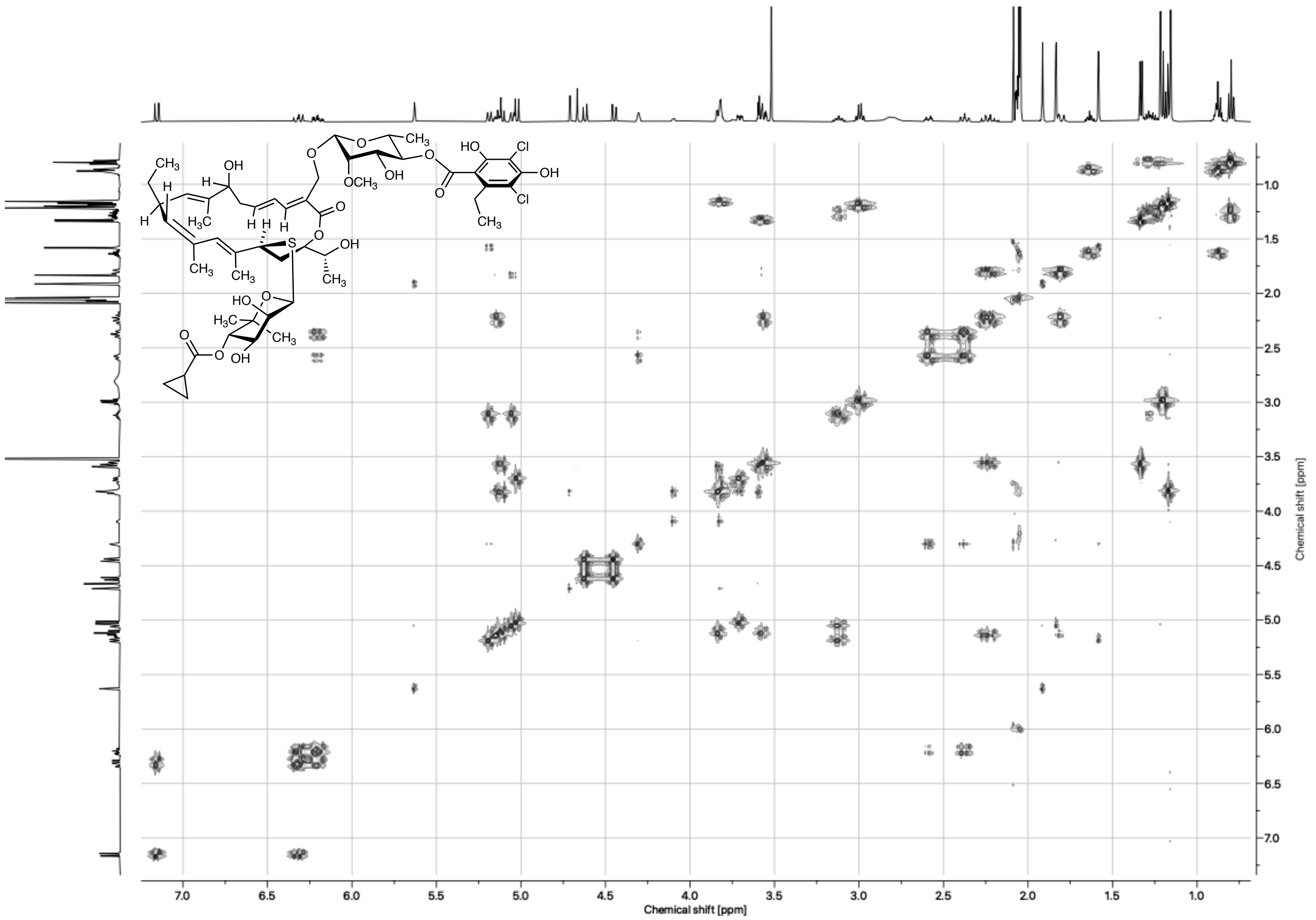

Figure 210: COSY spectrum of 11-desnoviosyl-15-4”-O-cyclopropanoyl-thio-β-D-noviosyl fidaxomicin (3b-C(15)) in acetone-d_6

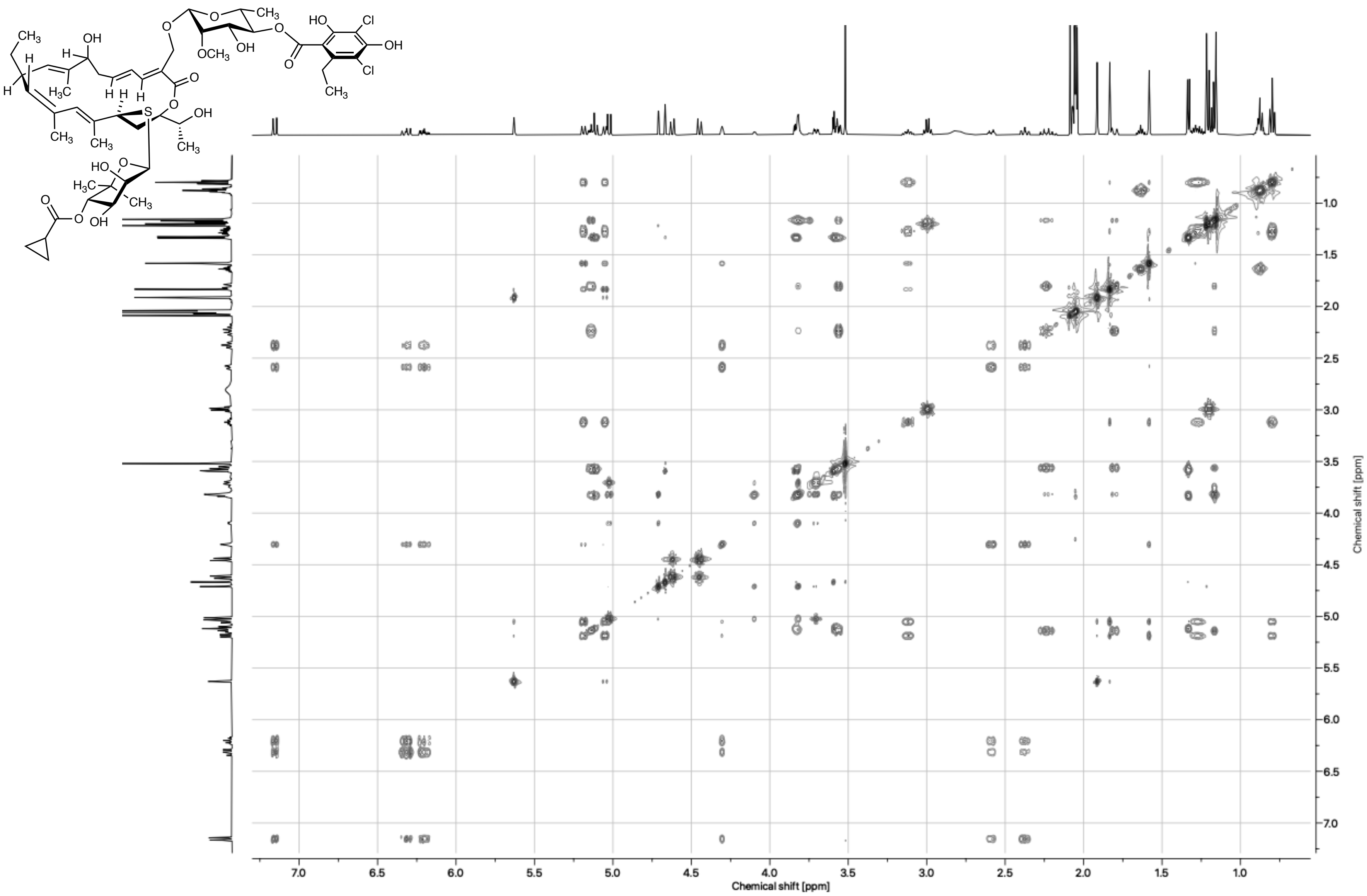

Figure 211: TOCSY spectrum of 11-desnoviosyl-15-4”-O-cyclopropanoyl-thio-β-D-noviosyl fidaxomicin (3b-C(15)) in acetone-d_6

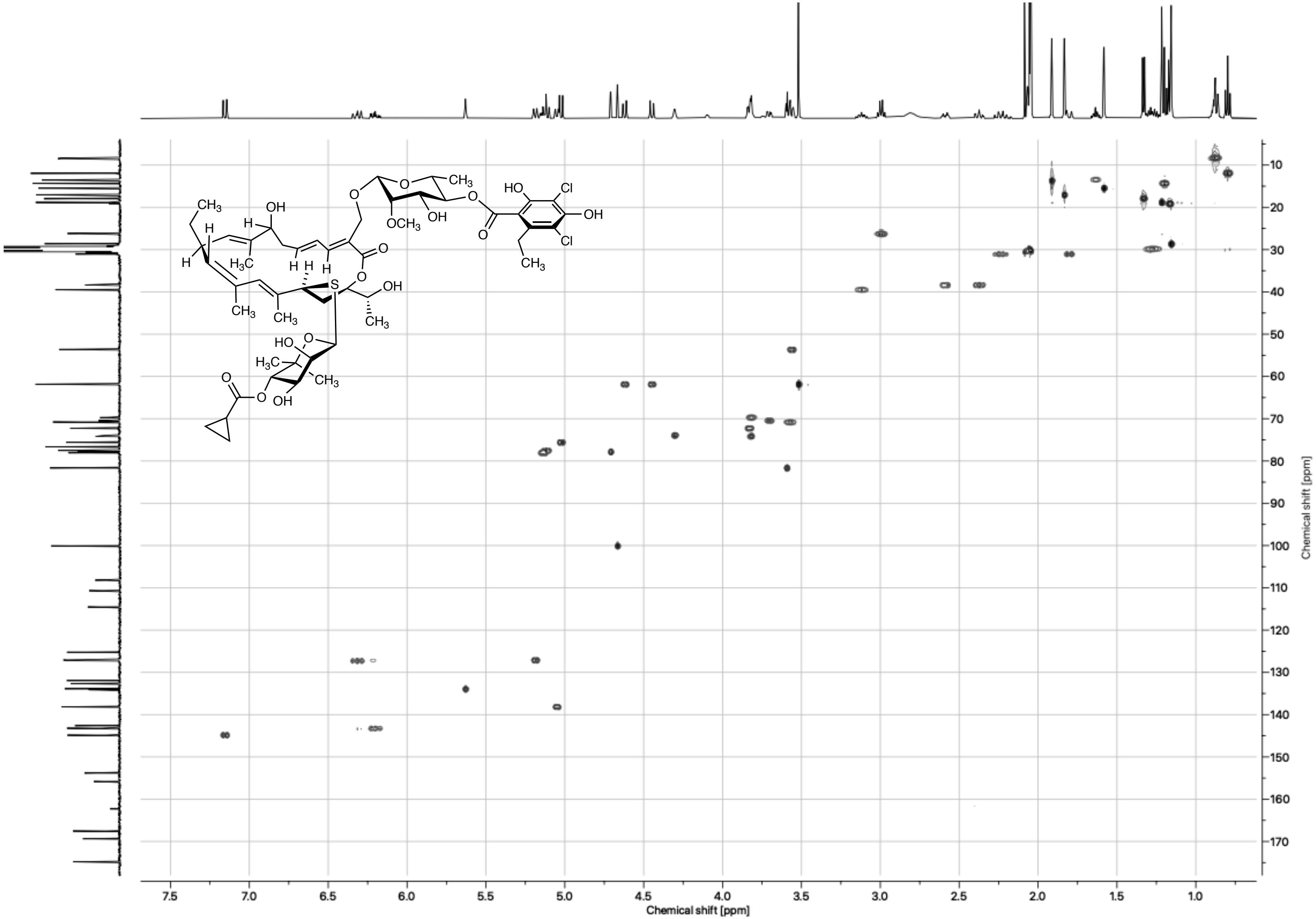

Figure 212: HSQC spectrum of 11-desnoviosyl-15-4”-O-cyclopropanoyl-thio-β-D-noviosyl fidaxomicin (3b-C(15)) in acetone-d_6

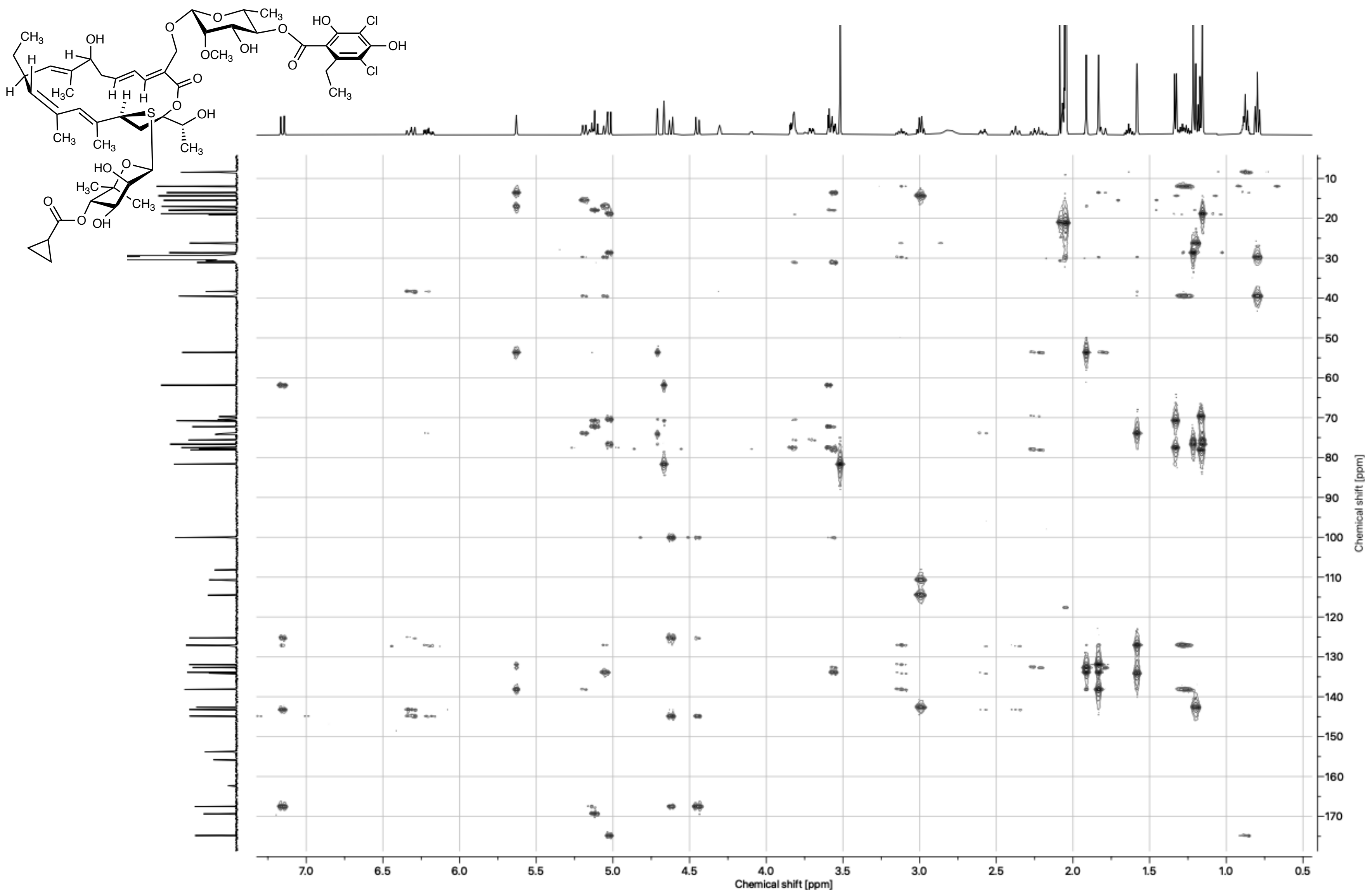

Figure 213: HMBC spectrum of 11-desnoviosyl-15-4''-O-cyclopropanoyl-thio-β-D-noviosyl fidaxomicin (3b-C(15)) in acetone-d_6

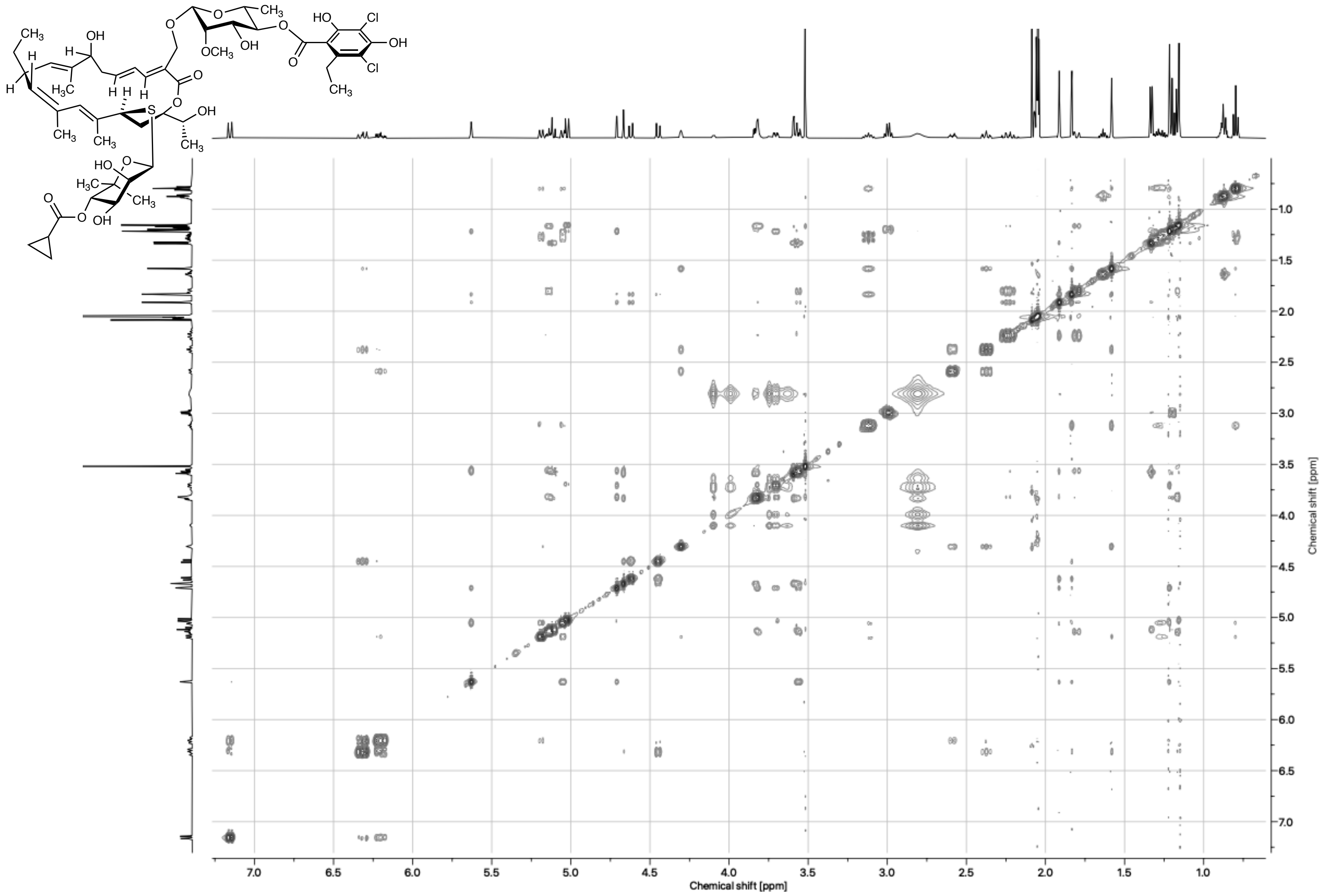

Figure 214: NOESY spectrum of 11-desnoviosyl-15-4”-O-cyclopropanoyl-thio-β-D-noviosyl fidaxomicin (3b-C(15)) in acetone-d_6

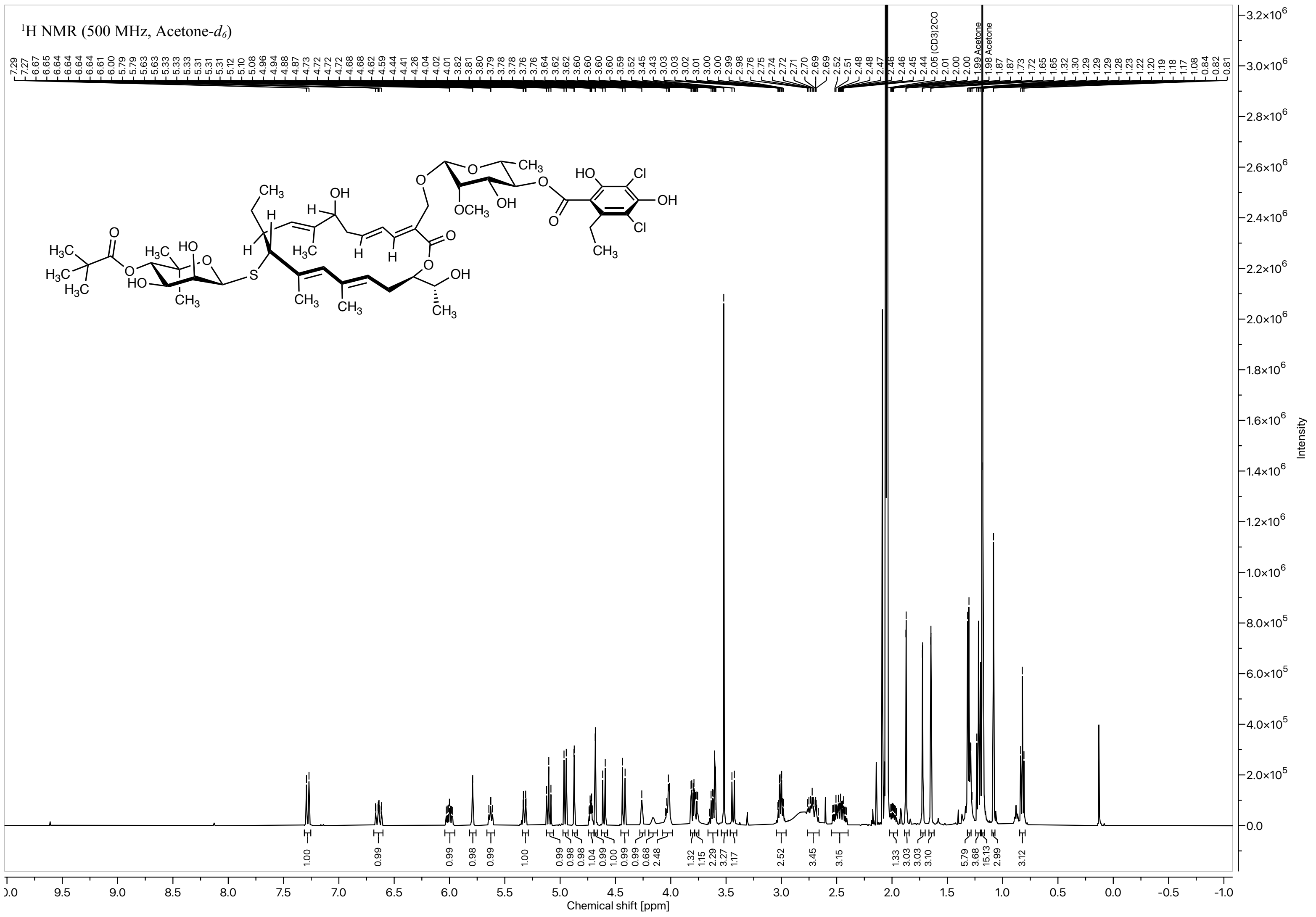

Figure 215: ^{1}H NMR spectrum of 11-desnoviosyl-11-4”-O-pivaloyl-thio-β-D-noviosyl fidaxomicin (3c-C(11)) in acetone-d_6

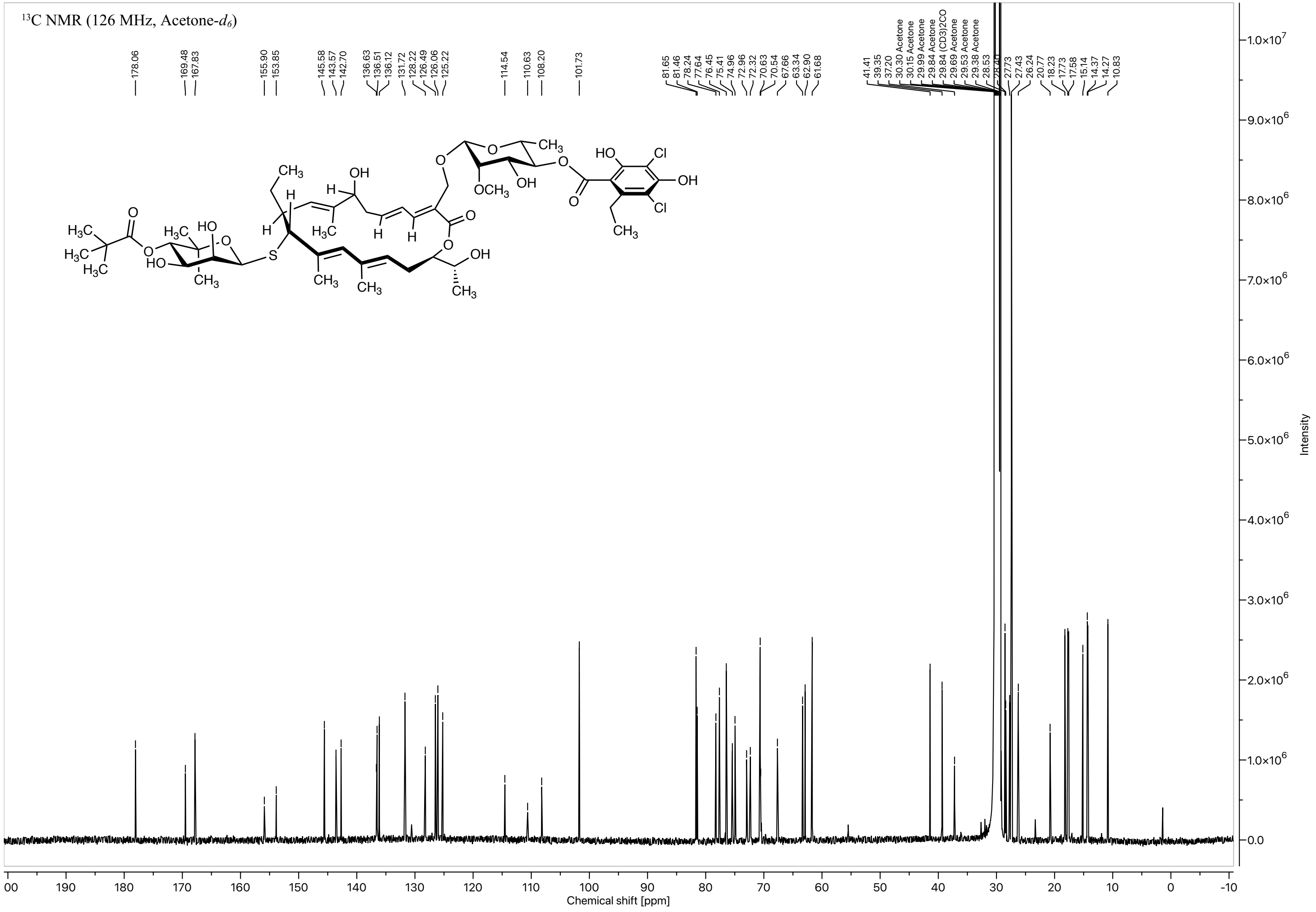

Figure 216: ^{13}C NMR spectrum of 11-desnoviosyl-11-4”-O-pivaloyl-thio-β-D-noviosyl fidaxomicin (3c-C(11)) in acetone-d_6

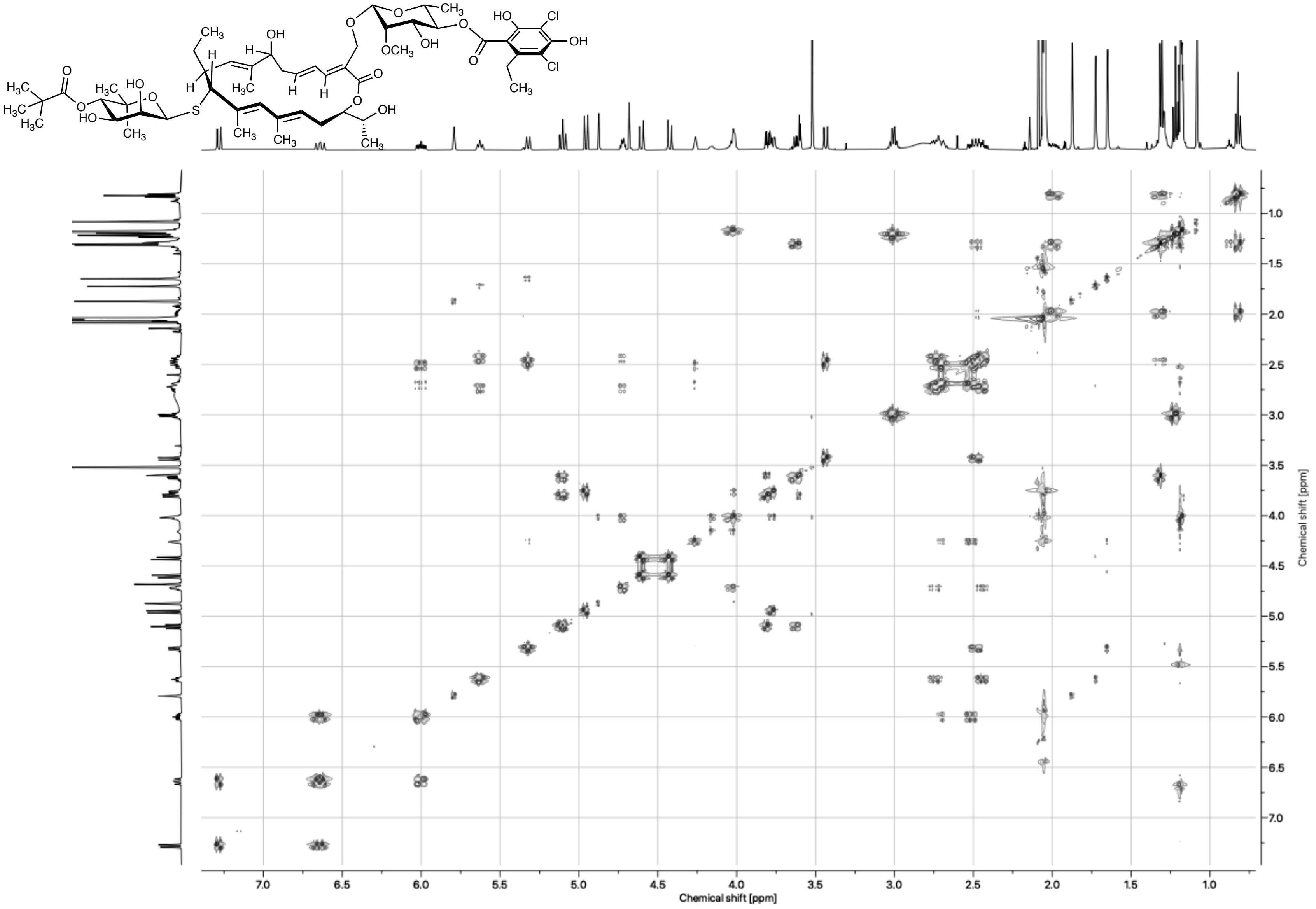

Figure 217: COSY spectrum of 11-desnoviosyl-11-4”-O-pivaloyl-thio-β-D-noviosyl fidaxomicin (3c-C(11)) in acetone-d_6

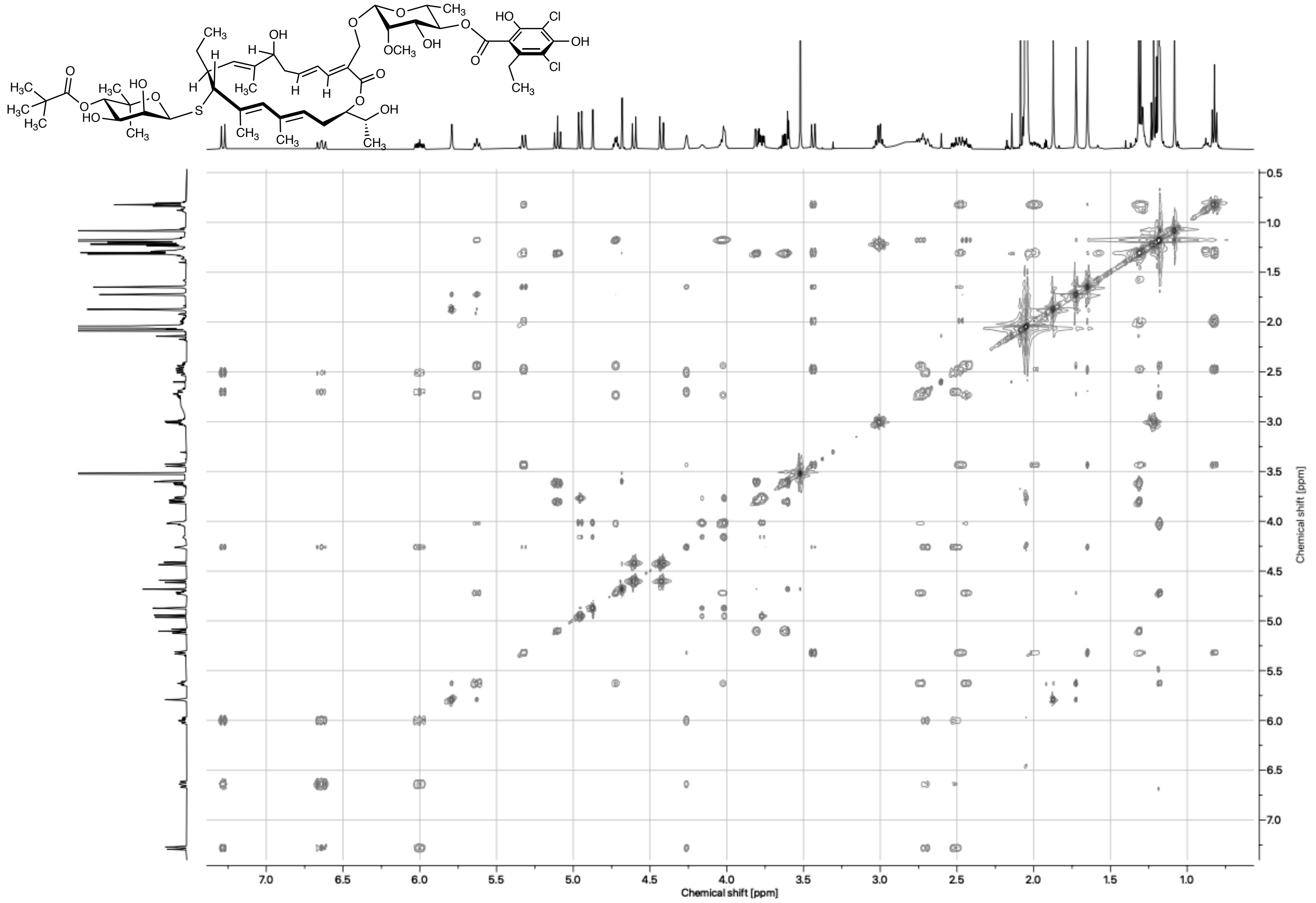

Figure 218: TOCSY spectrum of 11-desnoviosyl-11-4”-O-pivaloyl-thio-β-D-noviosyl fidaxomicin (3c-C(11)) in acetone-d_6

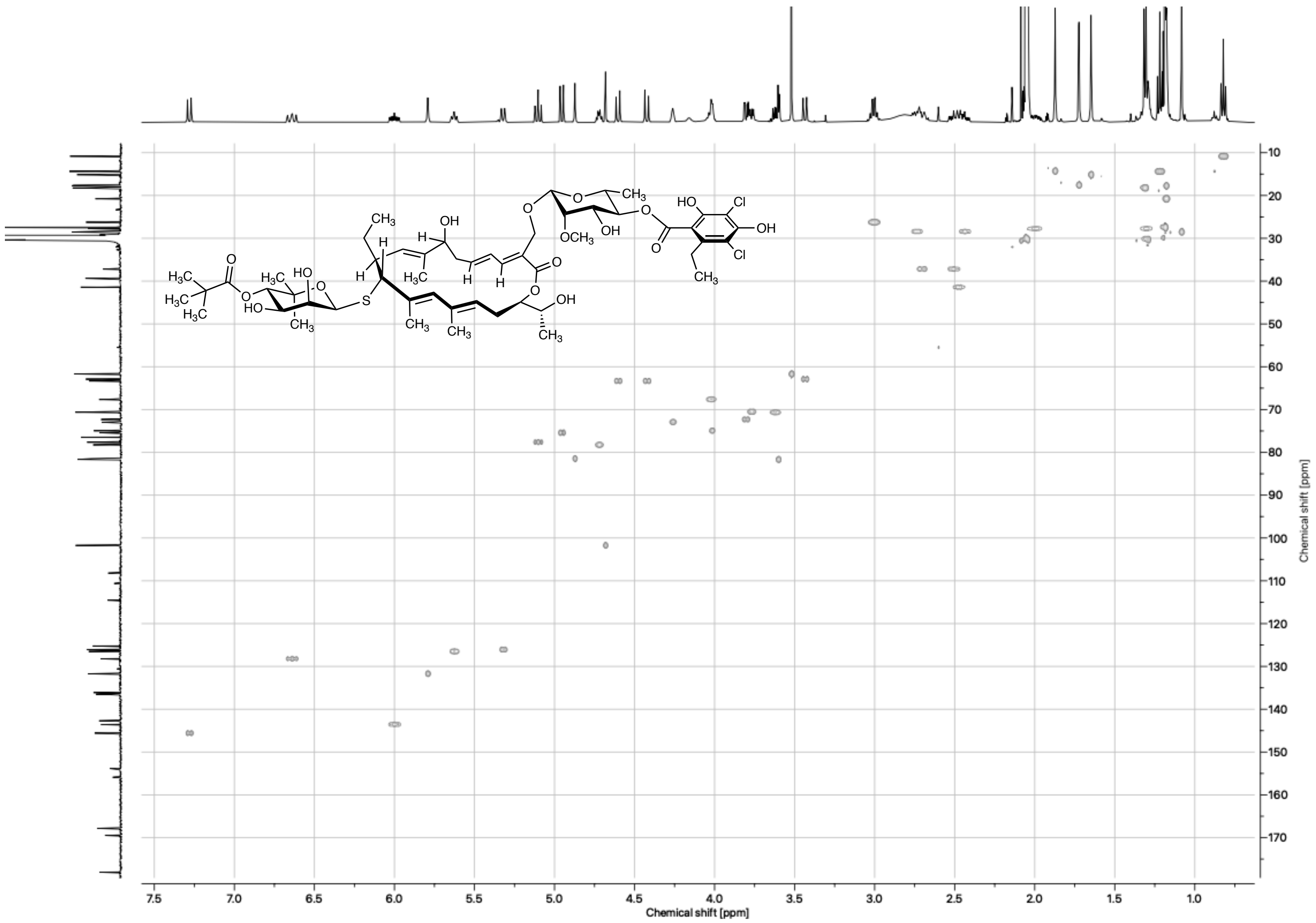

Figure 219: HSQC spectrum of 11-desnoviosyl-11-4''-O-pivaloyl-thio-β-D-noviosyl fidaxomicin (3c-C(11)) in acetone-d_6

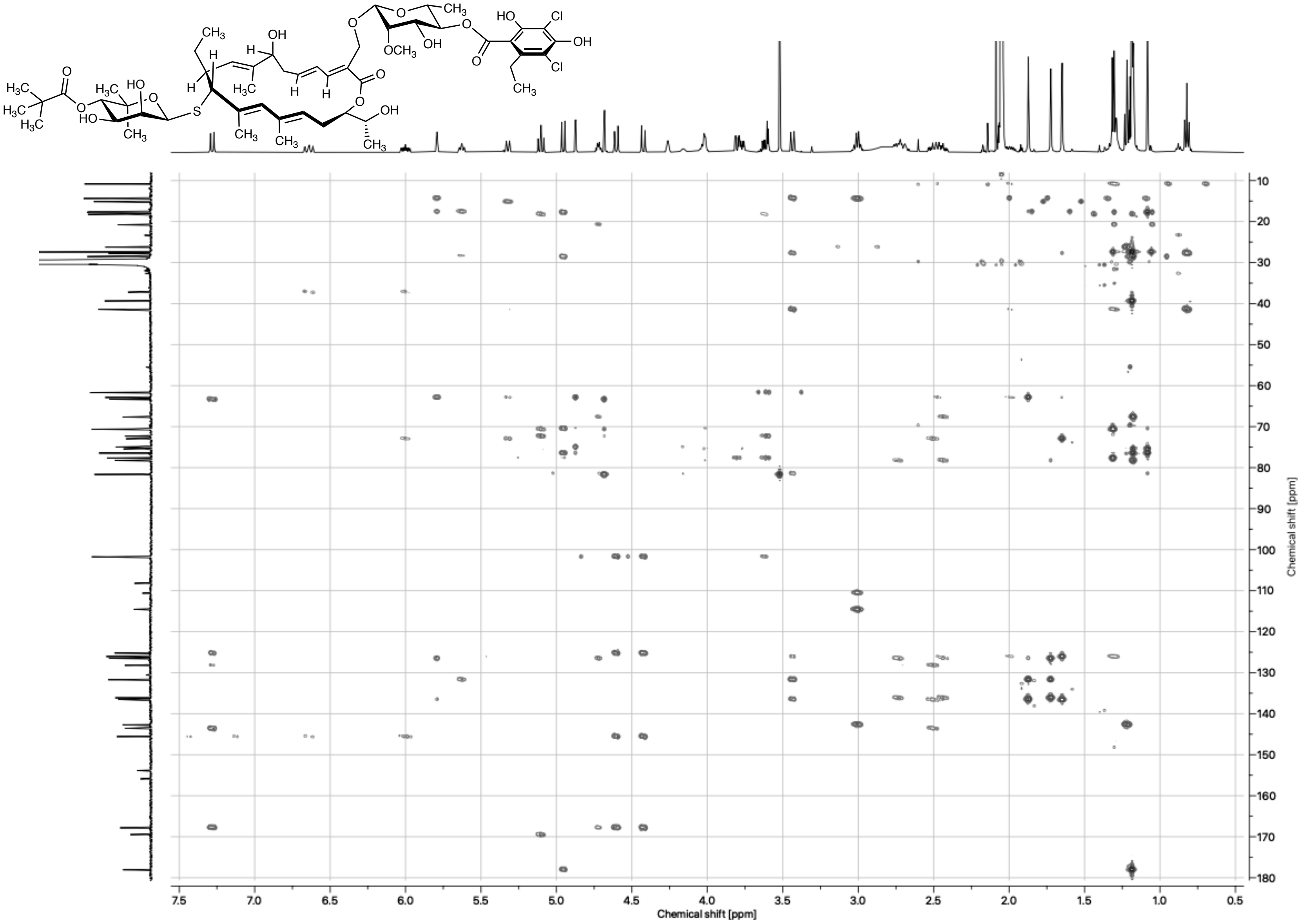

Figure 220: HMBC spectrum of 11-desnoviosyl-11-4”-O-pivaloyl-thio-β-D-noviosyl fidaxomicin (3c-C(11)) in acetone-d_6

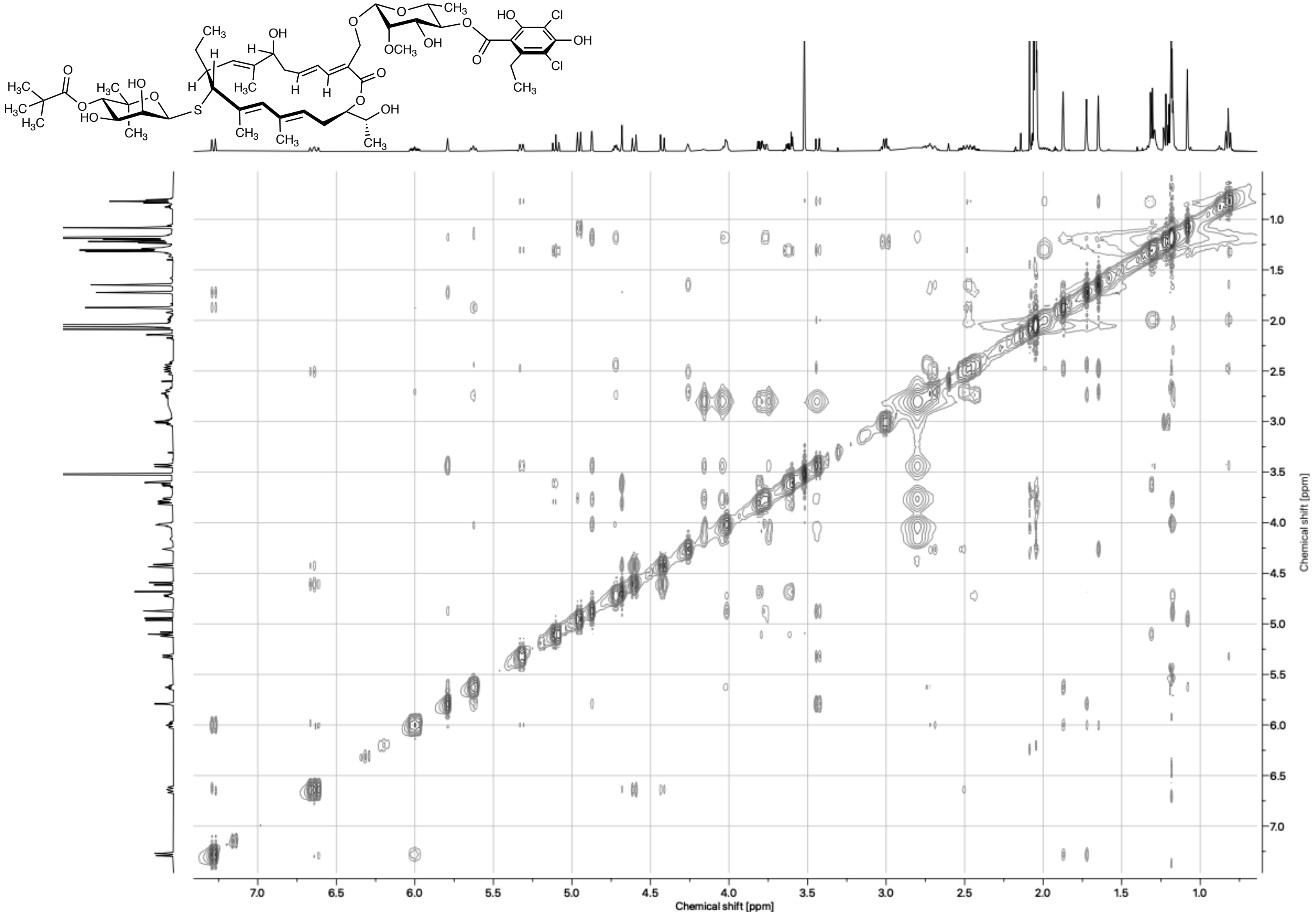

Figure 221: NOESY spectrum of 11-desnoviosyl-11-4”-O-pivaloyl-thio-β-D-noviosyl fidaxomicin (3c-C(11)) in acetone-d_6

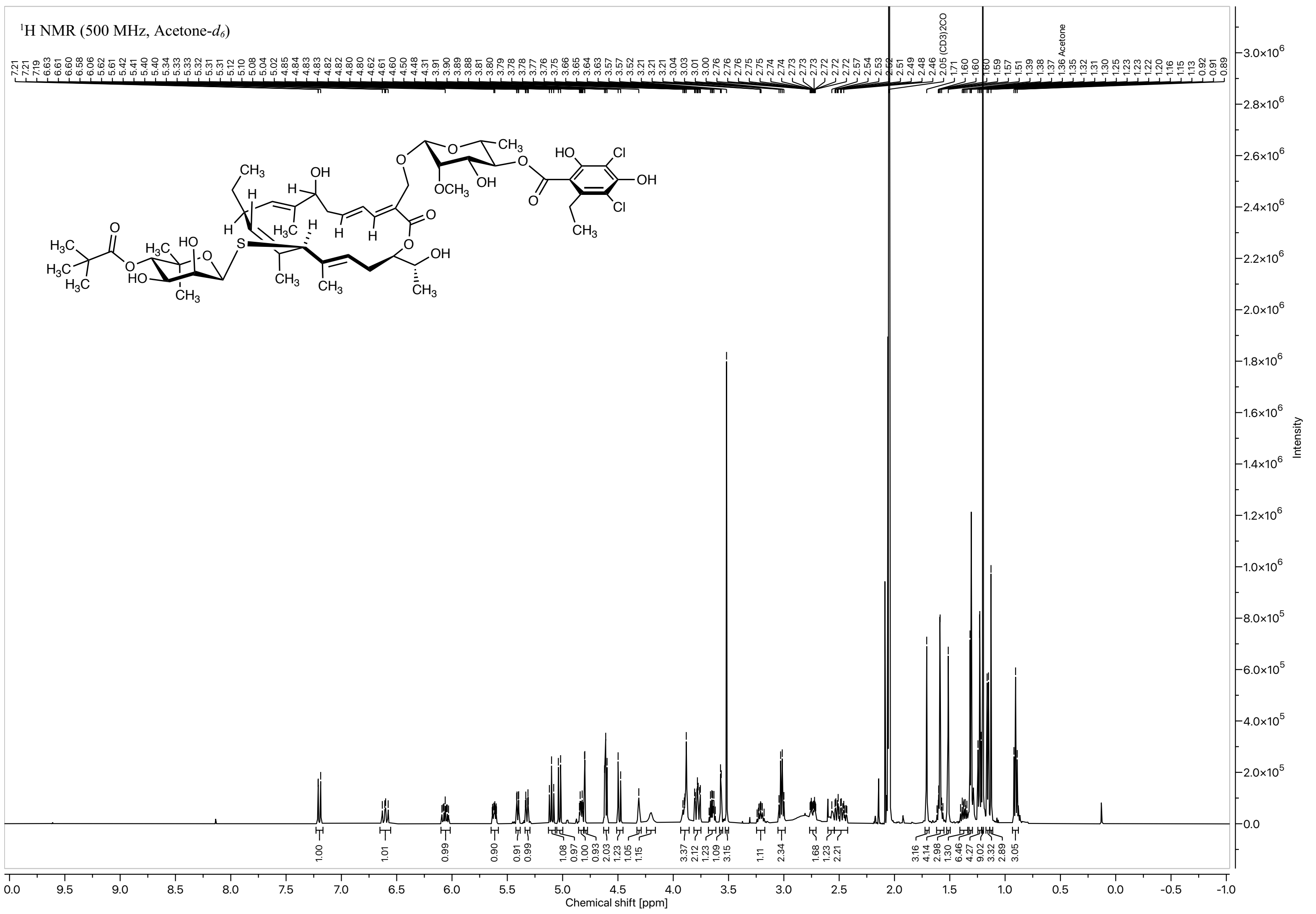

Figure 222: ^{1}H NMR spectrum of 11-desnoviosyl-13-4”-O-pivaloyl-thio-β-D-noviosyl fidaxomicin (3c-C(13)) in acetone-d_6

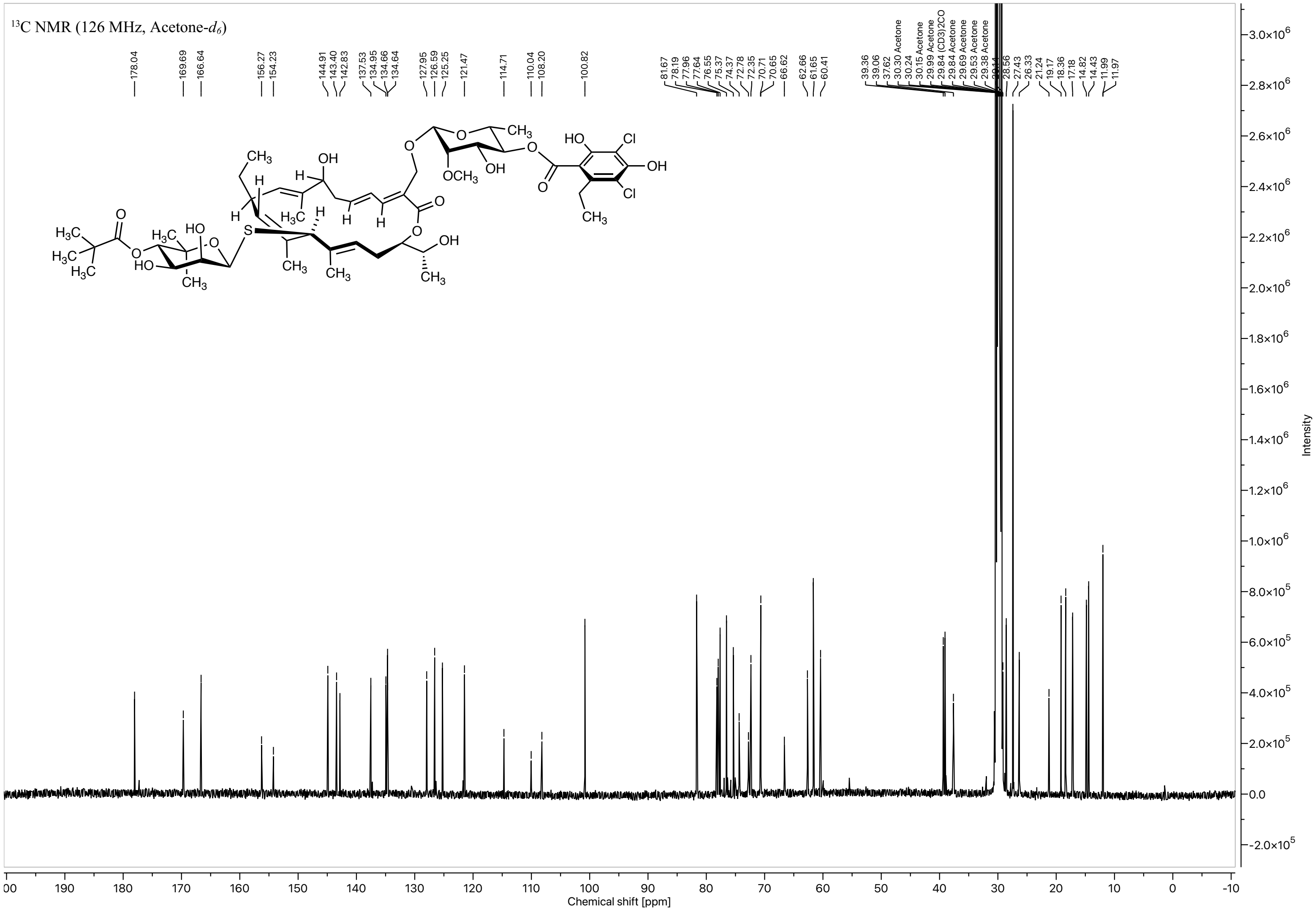

Figure 223: ^{13}C NMR spectrum of 11-desnoviosyl-13-4”-O-pivaloyl-thio-β-D-noviosyl fidaxomicin (3c-C(13)) in acetone-d_6

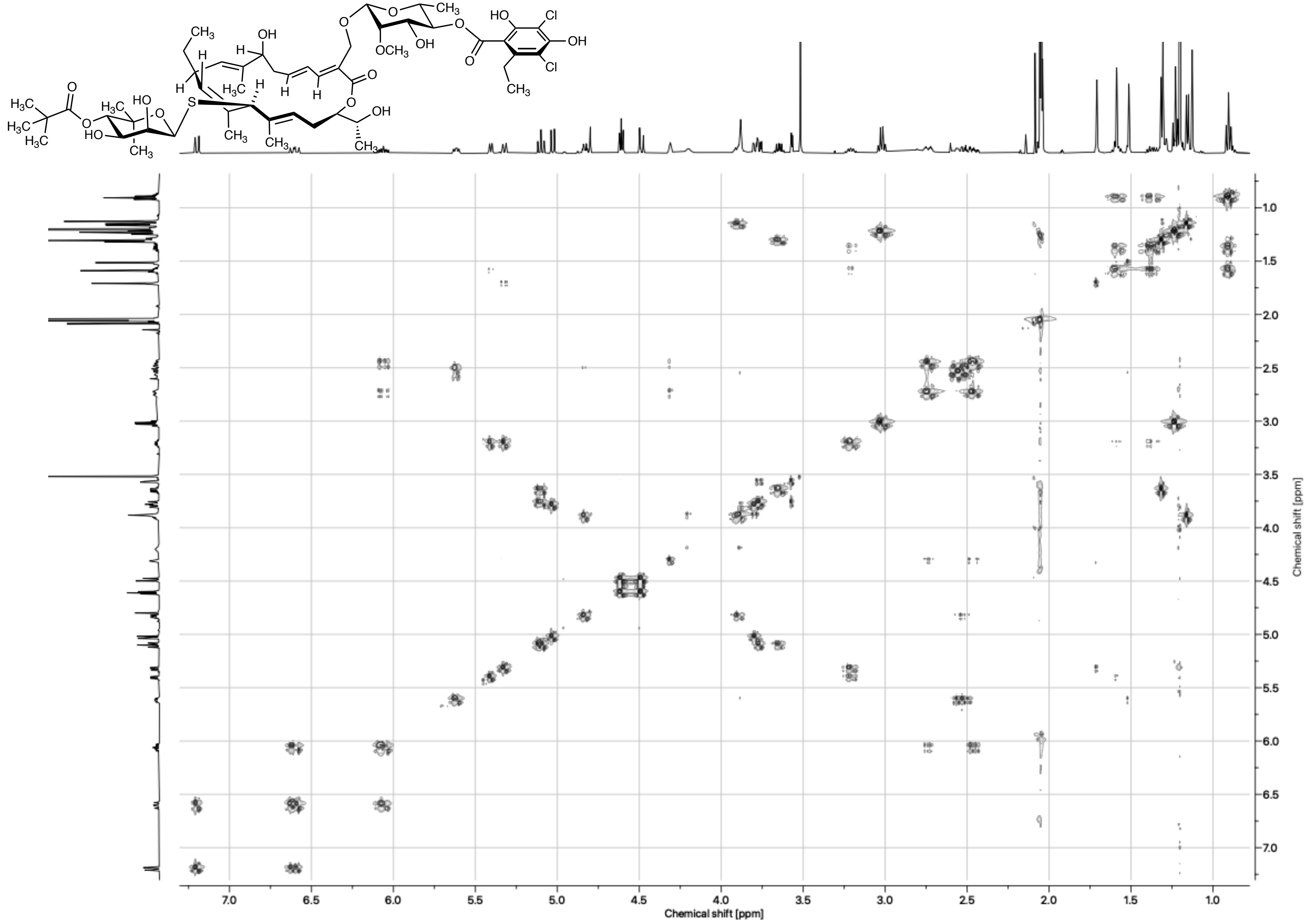

Figure 224: COSY spectrum of 11-desnoviosyl-13-4’’-O-pivaloyl-thio-β-D-noviosyl fidaxomicin (3c-C(13)) in acetone-d_6

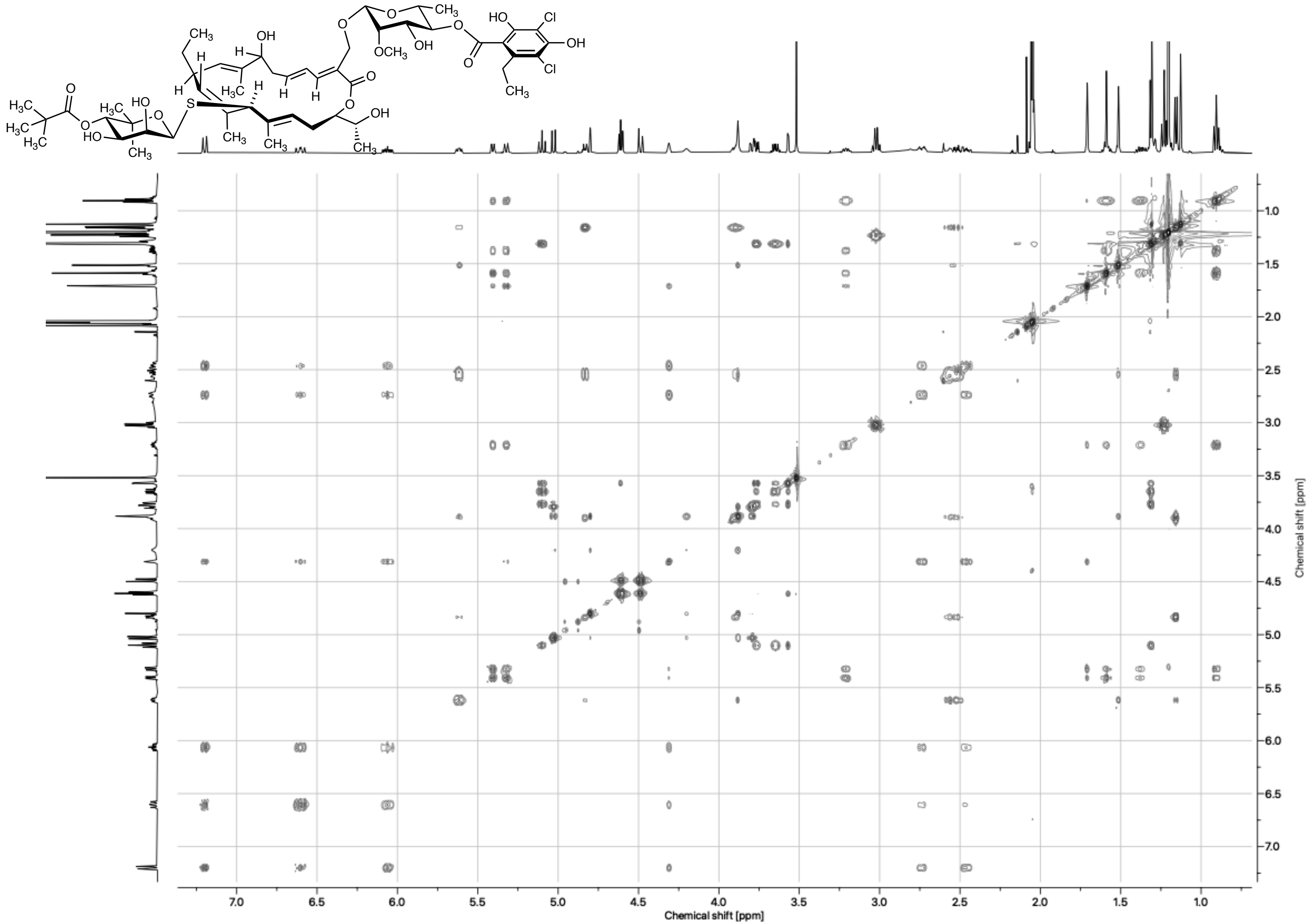

Figure 225: TOCSY spectrum of 11-desnoviosyl-13-4''-O-pivaloyl-thio-β-D-noviosyl fidaxomicin (3c-C(13)) in acetone-d_6

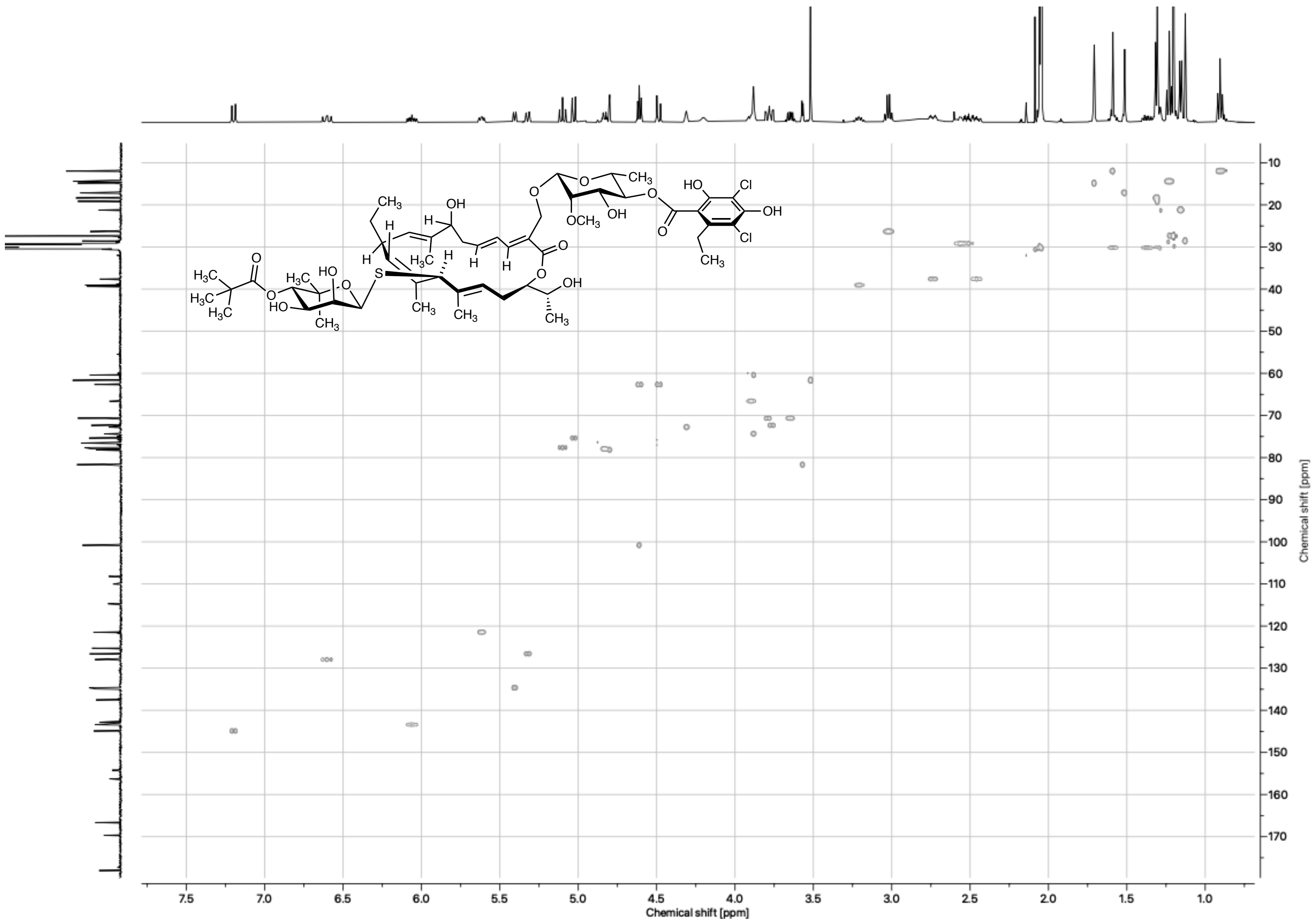

Figure 226: HSQC spectrum of 11-desnoviosyl-13-4''-O-pivaloyl-thio-β-D-noviosyl fidaxomicin (3c-C(13)) in acetone-d_6

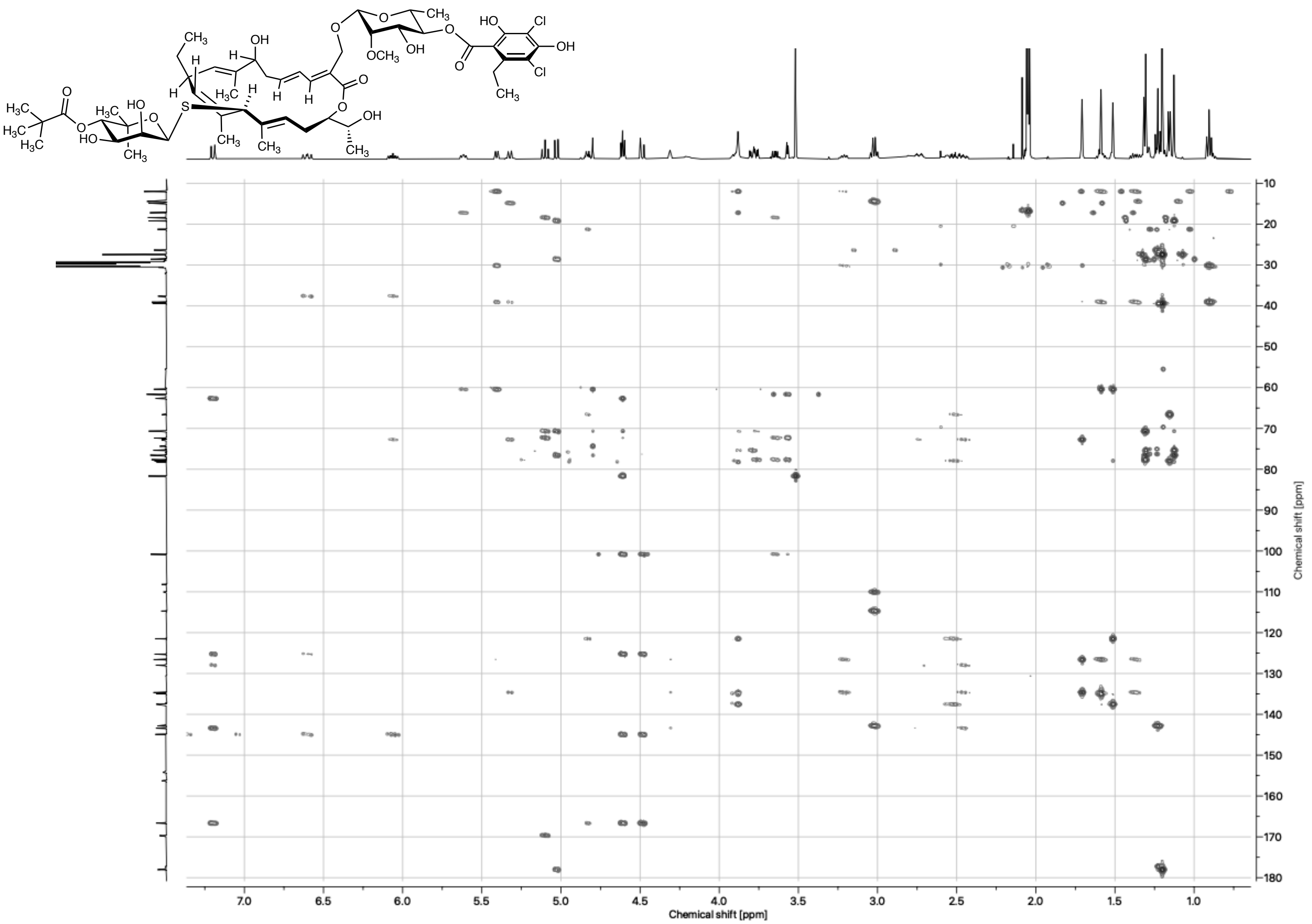

Figure 227: HMBC spectrum of 11-desnoviosyl-13-4”-O-pivaloyl-thio-β-D-noviosyl fidaxomicin (3c-C(13)) in acetone-d_6

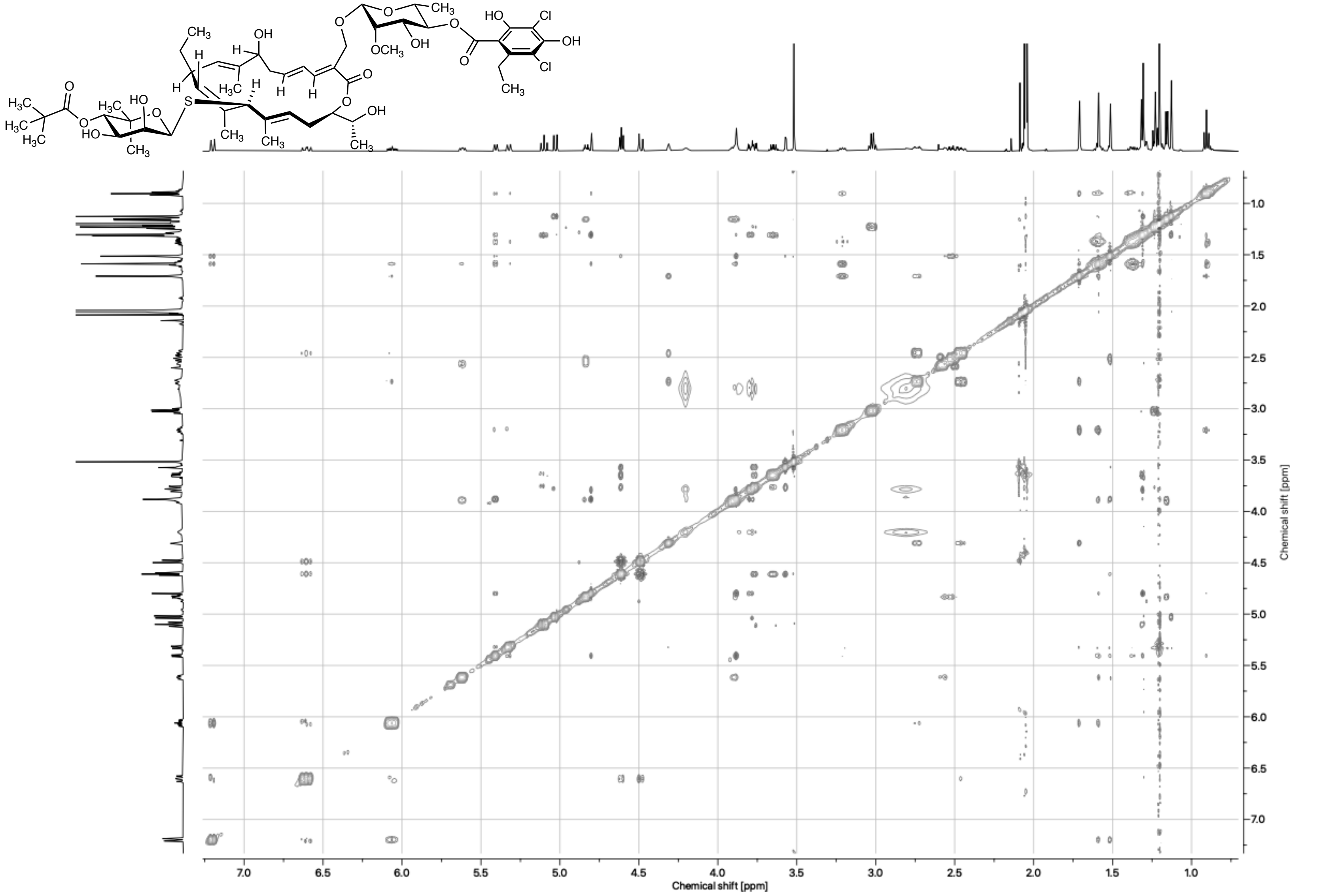

Figure 228: NOESY spectrum of 11-desnoviosyl-13-4”-O-pivaloyl-thio-β-D-noviosyl fidaxomicin (3c-C(13)) in acetone-d_6

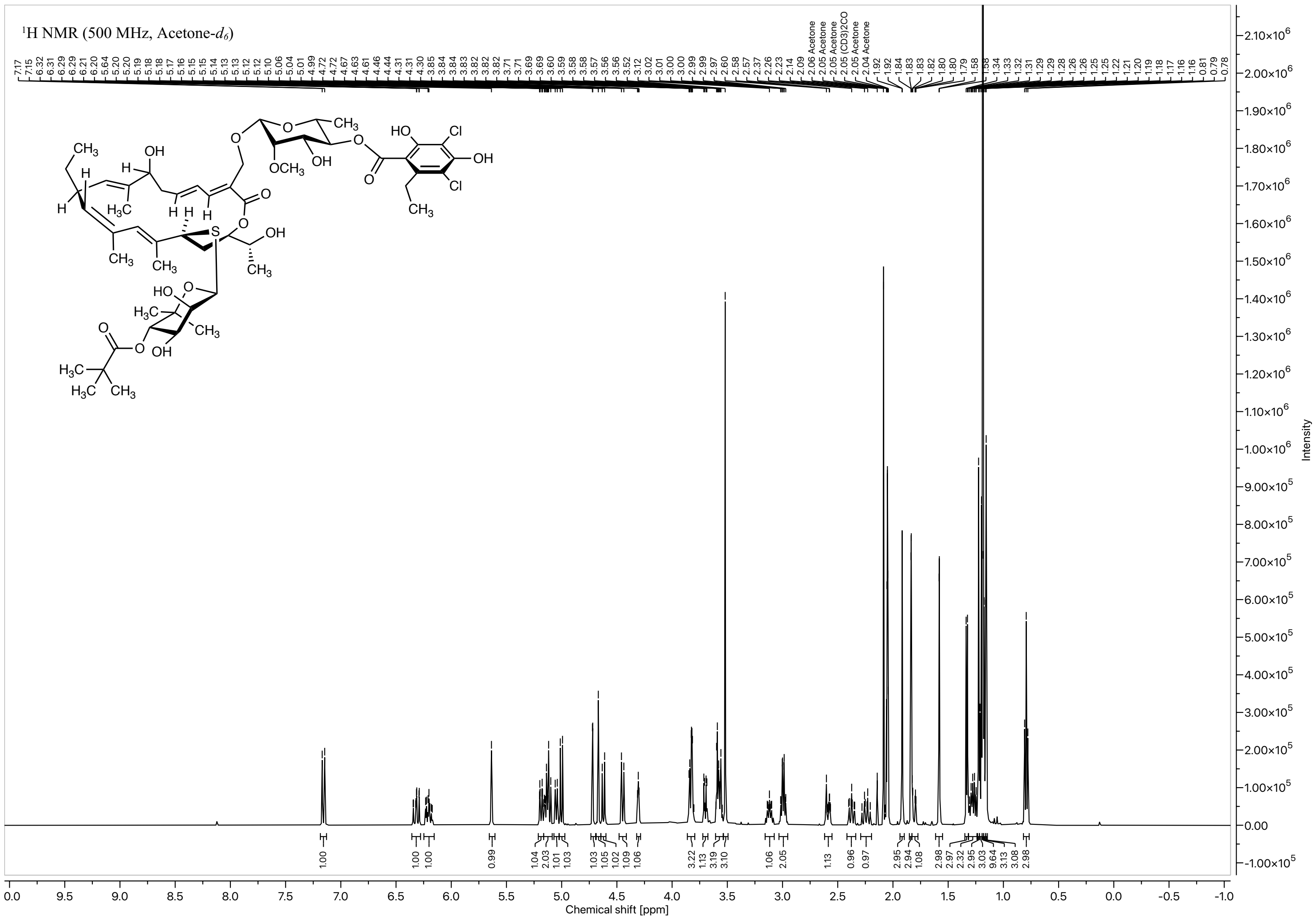

Figure 229: ^{1}H NMR spectrum of 11-desnoviosyl-15-4”-O-pivaloyl-thio-β-D-noviosyl fidaxomicin (3c-C(15)) in acetone-d_6

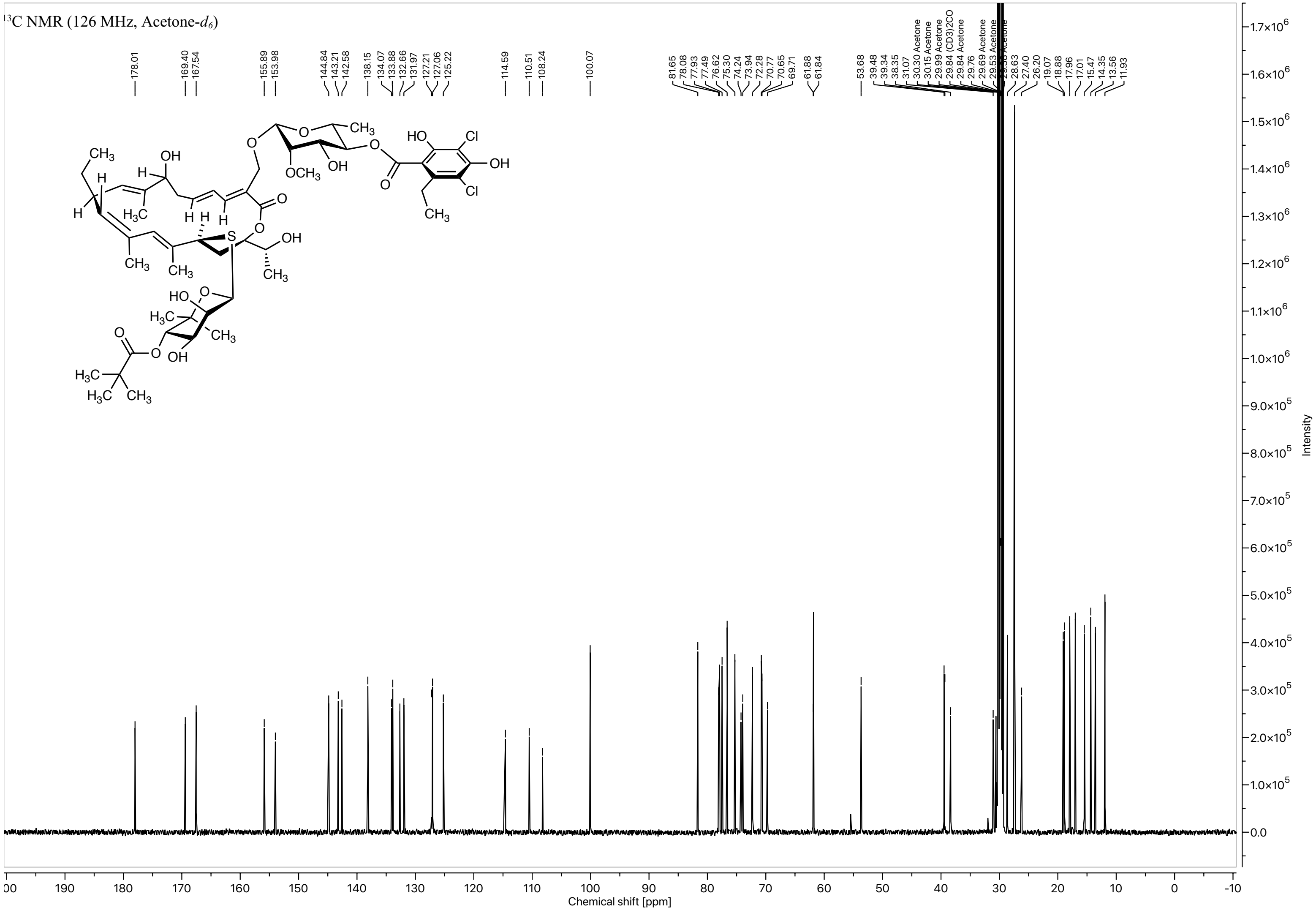

Figure 230: ^{13}C NMR spectrum of 11-desnoviosyl-15-4''-O-pivaloyl-thio-β-D-noviosyl fidaxomicin (3c-C(15)) in acetone-d_6

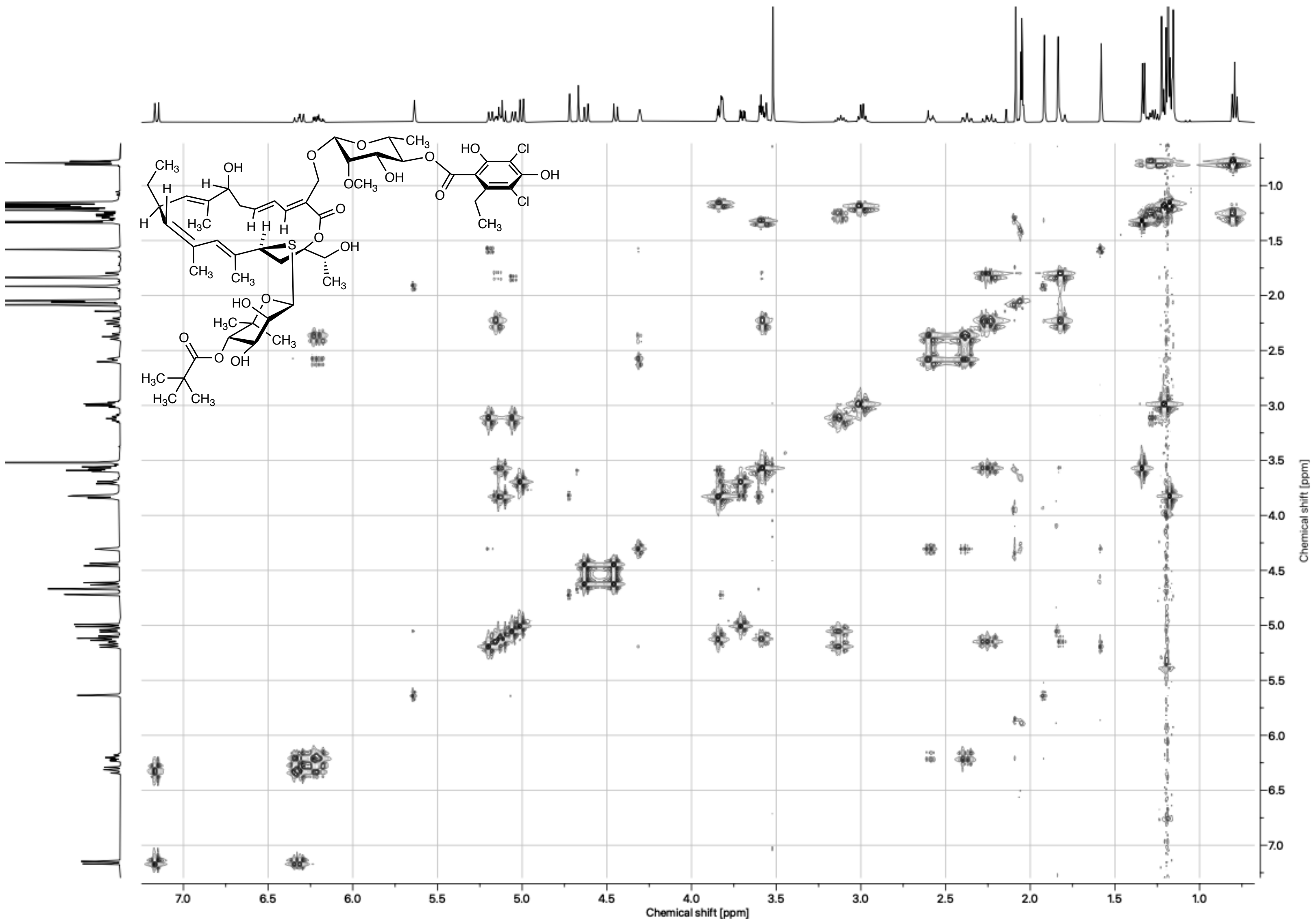

Figure 231: COSY spectrum of 11-desnoviosyl-15-4”-O-pivaloyl-thio-β-D-noviosyl fidaxomicin (3c-C(15)) in acetone-d_6

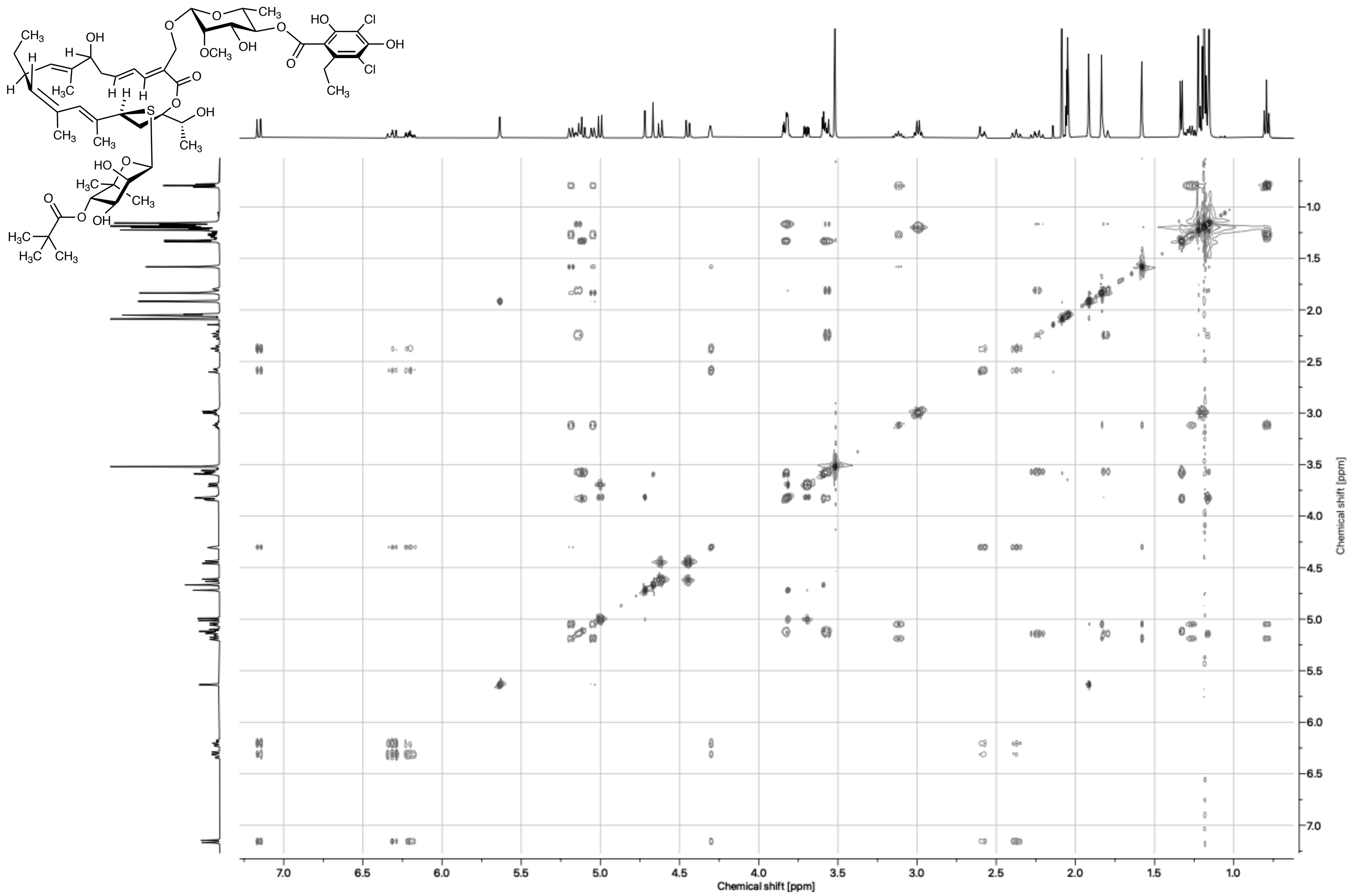

Figure 232: TOCSY spectrum of 11-desnoviosyl-15-4”-O-pivaloyl-thio-β-D-noviosyl fidaxomicin (3c-C(15)) in acetone-d_6

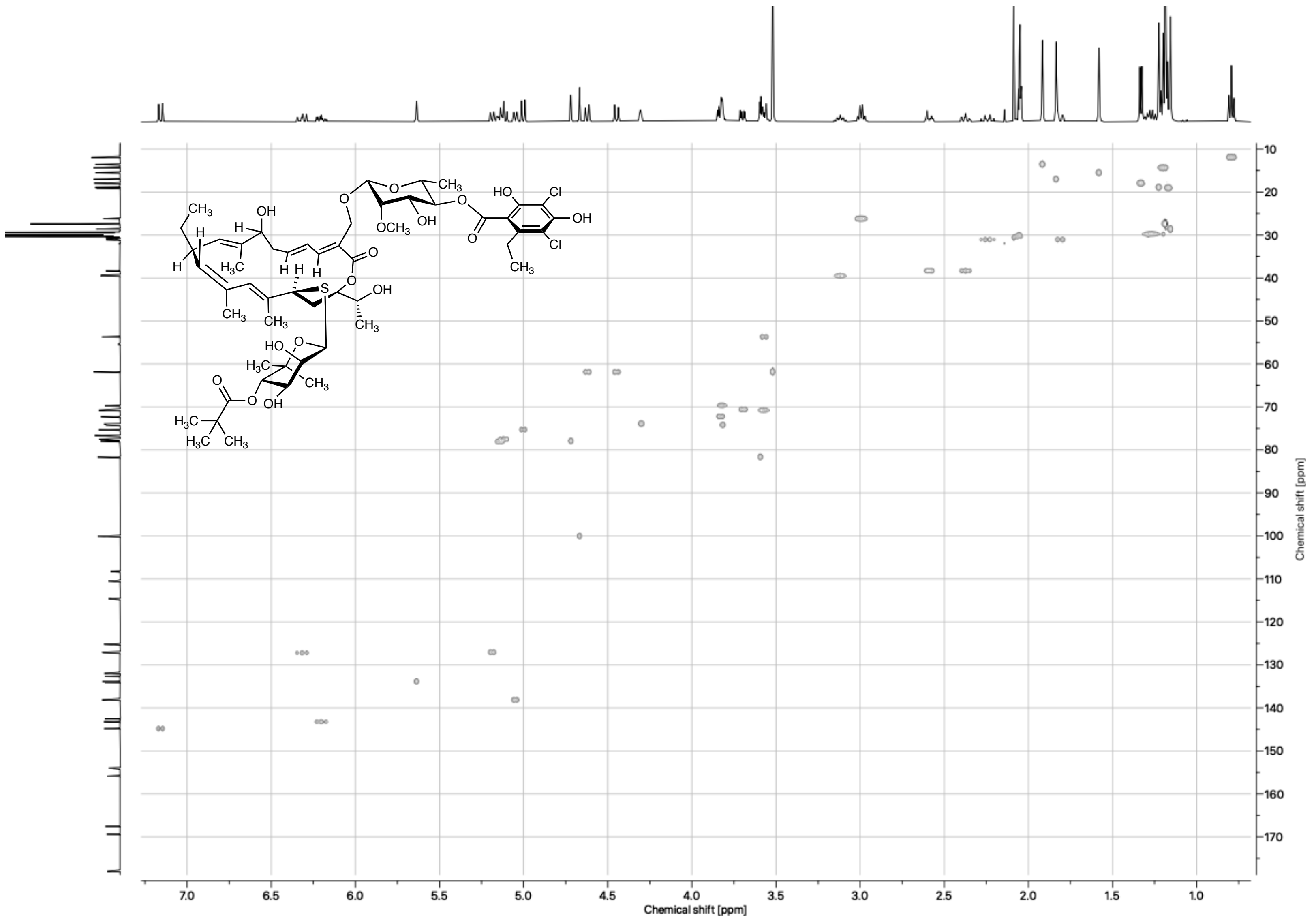

Figure 233: HSQC spectrum of 11-desnoviosyl-15-4”-O-pivaloyl-thio-β-D-noviosyl fidaxomicin (3c-C(15)) in acetone-d_6

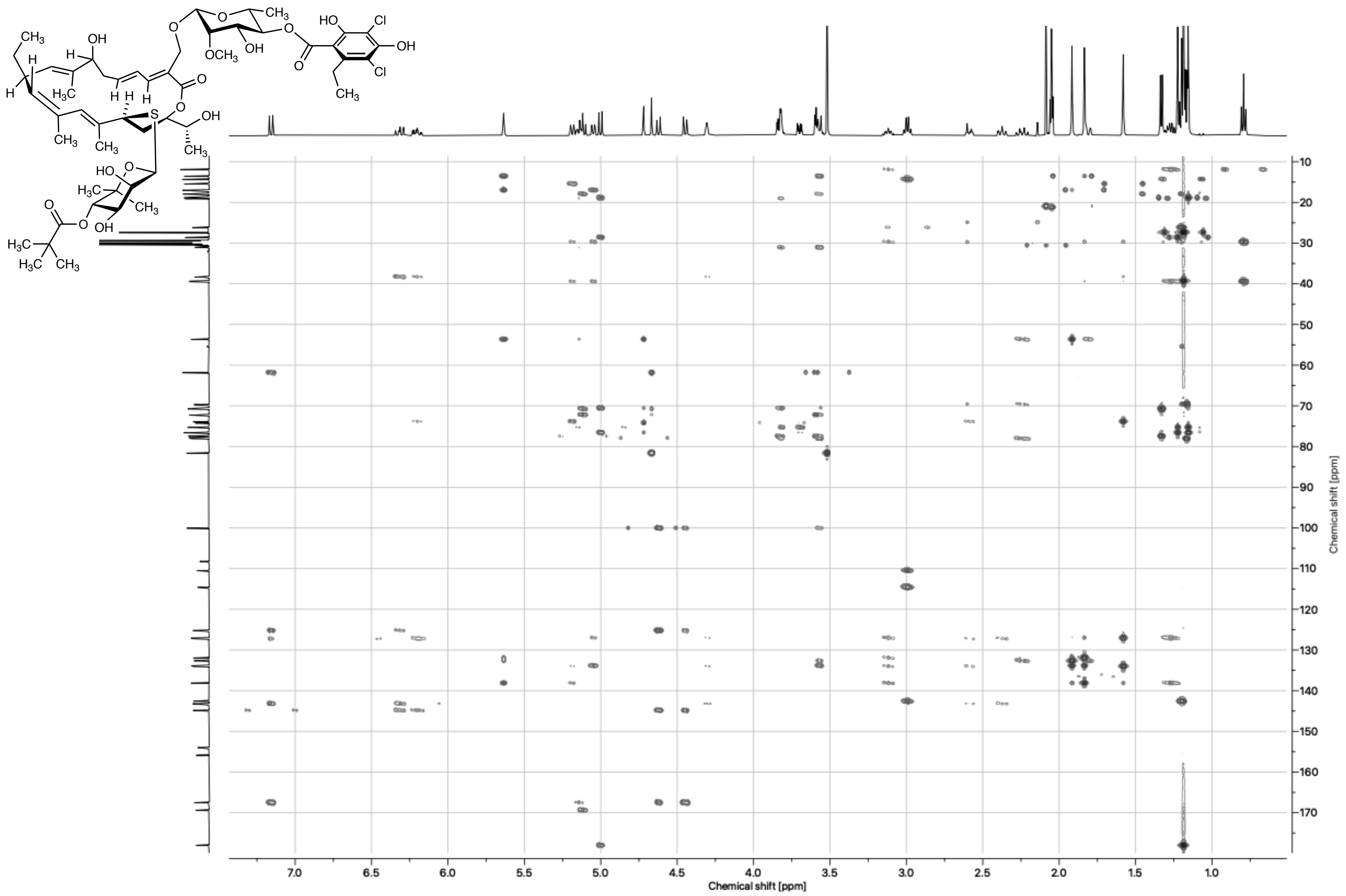

Figure 234: HMBC spectrum of 11-desnoviosyl-15-4”-O-pivaloyl-thio-β-D-noviosyl fidaxomicin (3c-C(15)) in acetone-d_6

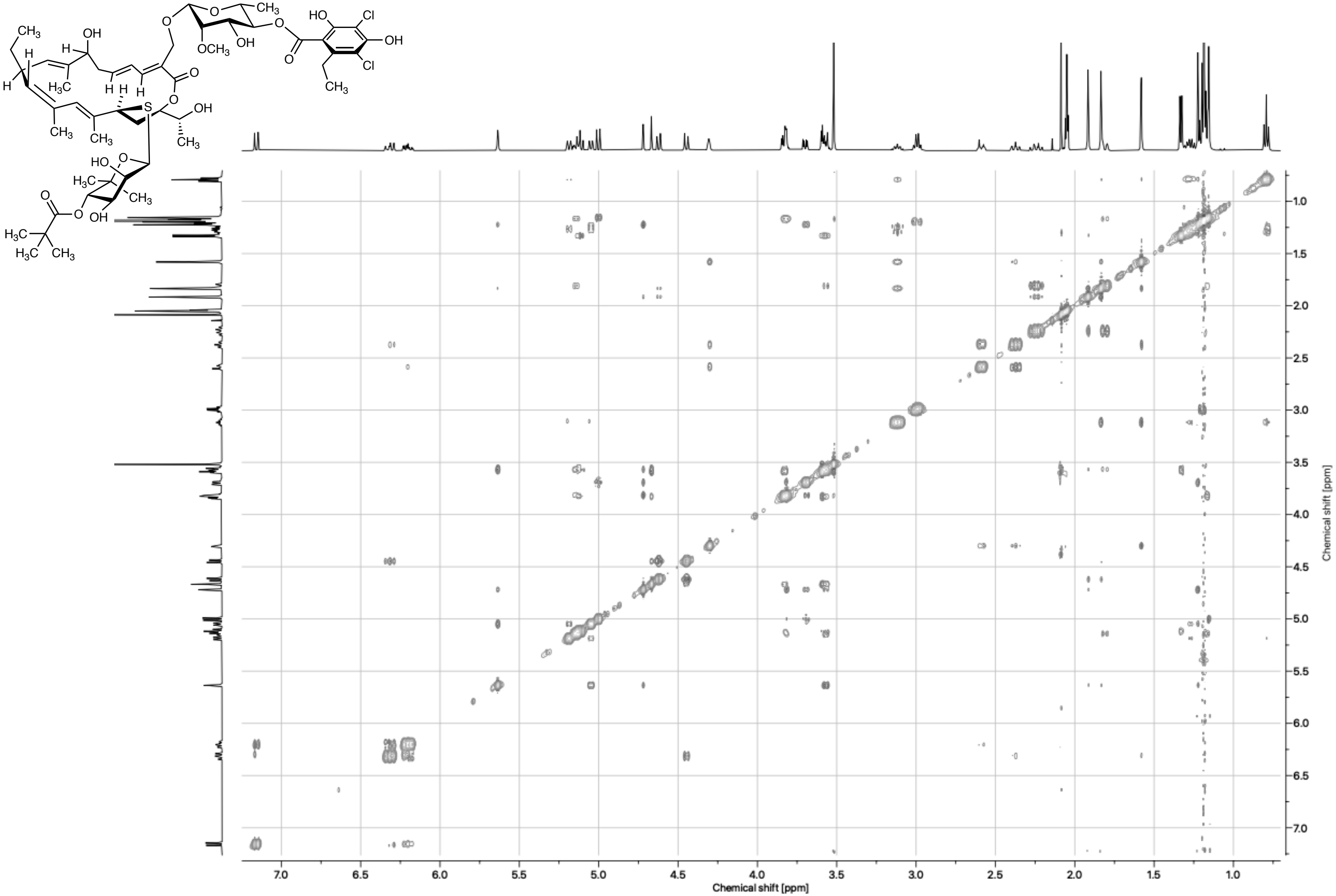

Figure 235: NOESY spectrum of 11-desnoviosyl-15-4”-O-pivaloyl-thio-β-D-noviosyl fidaxomicin (3c-C(15)) in acetone-d_6

Spectral data for noviose derivatives

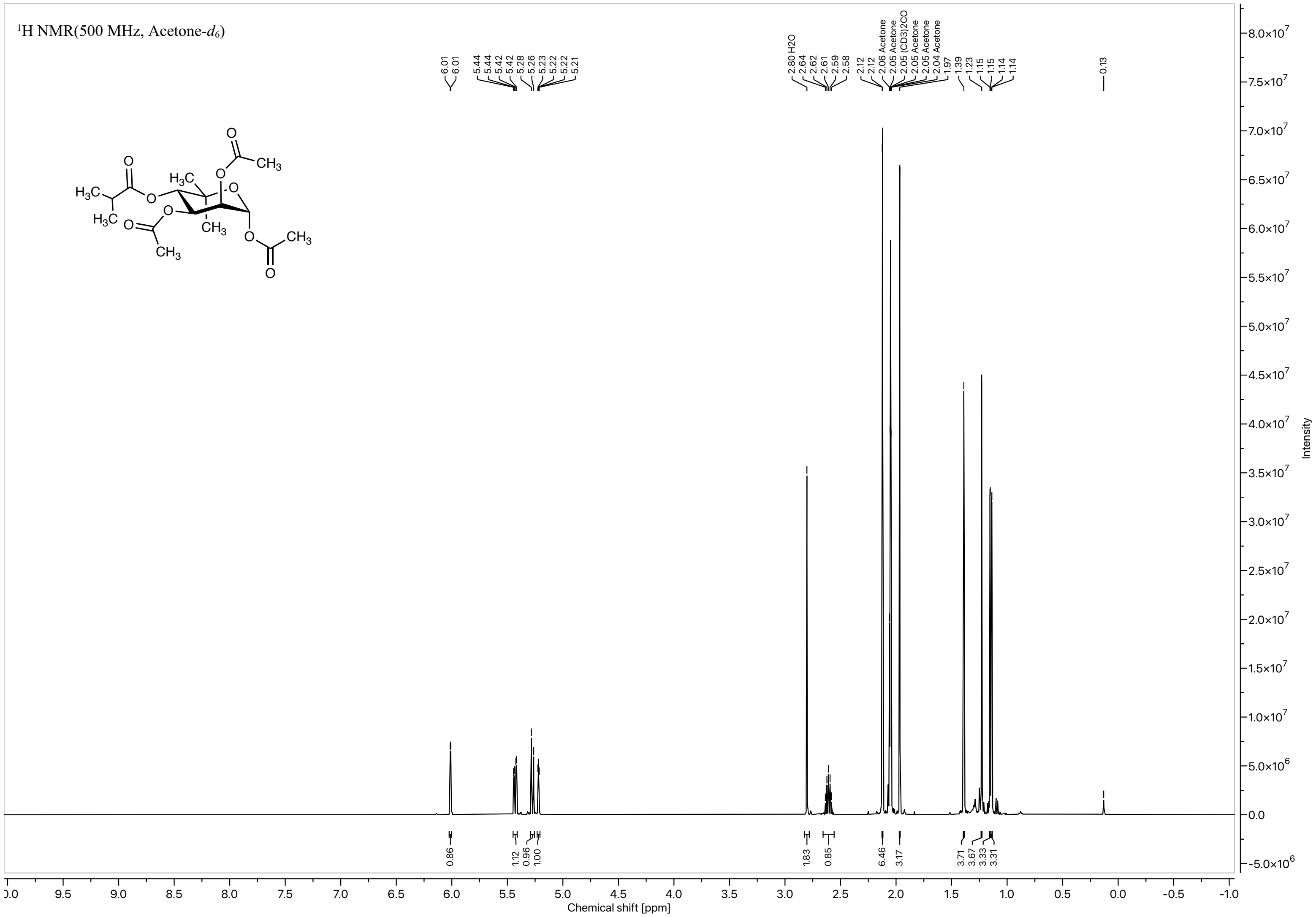

Figure 236: ¹H NMR spectrum of 1,2,3-tri-*O*-acetyl-4-*O*-demethyl-4-*O*-isobutyryl-α-D-noviose (8-α) in acetone-d_6

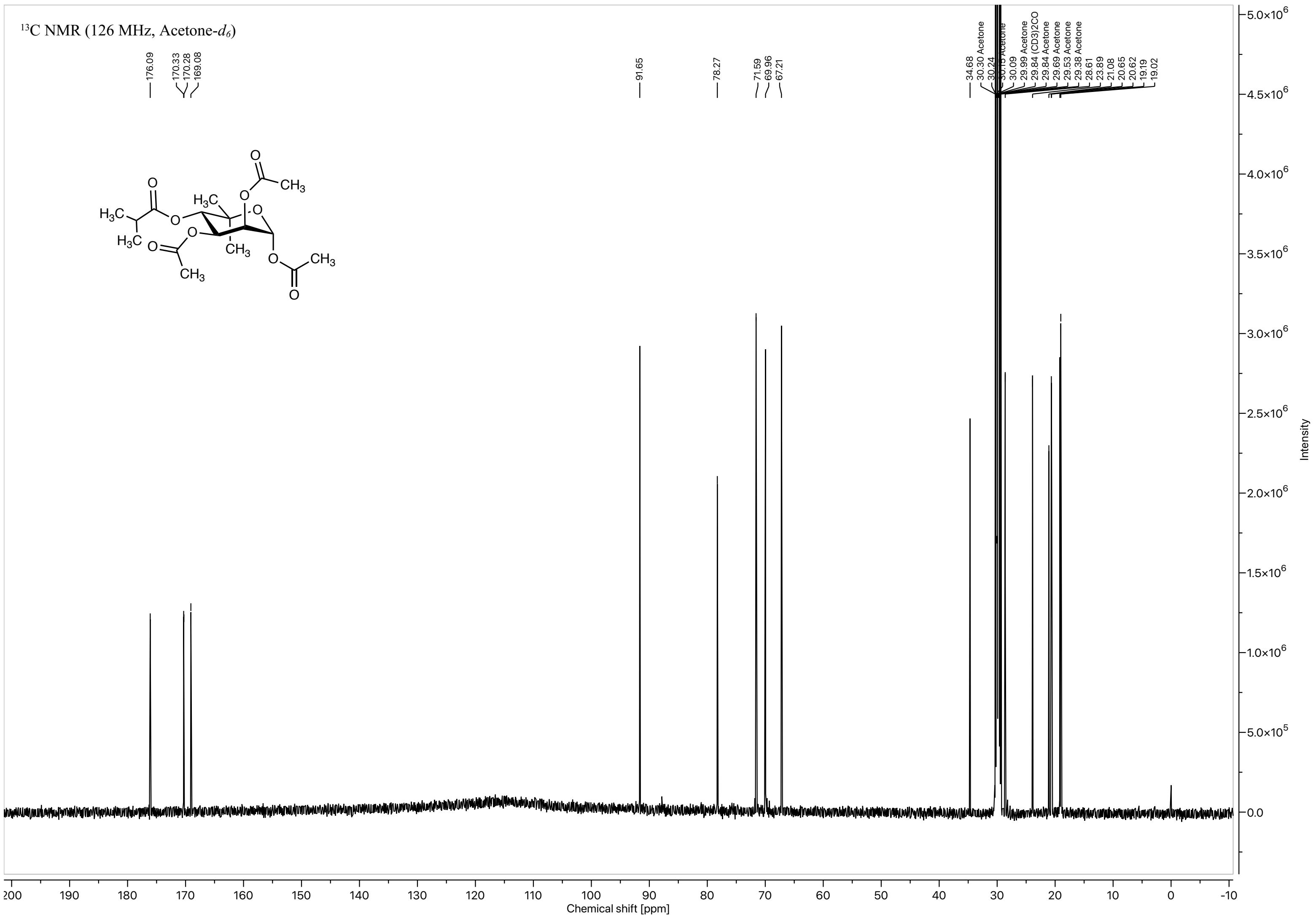

Figure 237: ^{13}C NMR spectrum of 1,2,3-tri-*O*-acetyl-4-*O*-demethyl-4-*O*-isobutyryl-α-D-noviose (8-α) in acetone-d_6

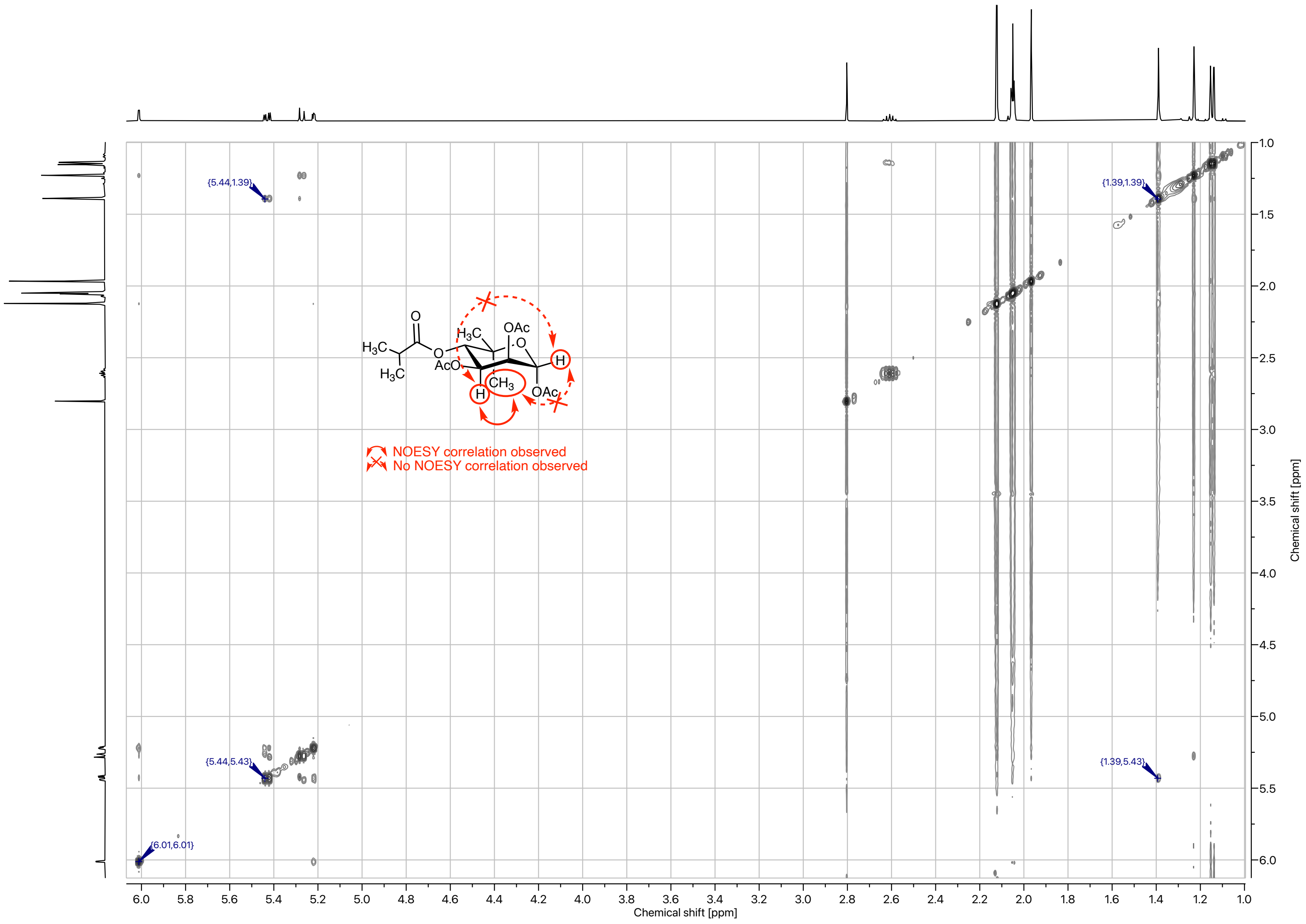

Figure 238: NOESY spectrum of 1,2,3-tri-*O*-acetyl-4-*O*-demethyl-4-*O*-isobutyryl-α-D-noviose (8-α) in acetone-d_6

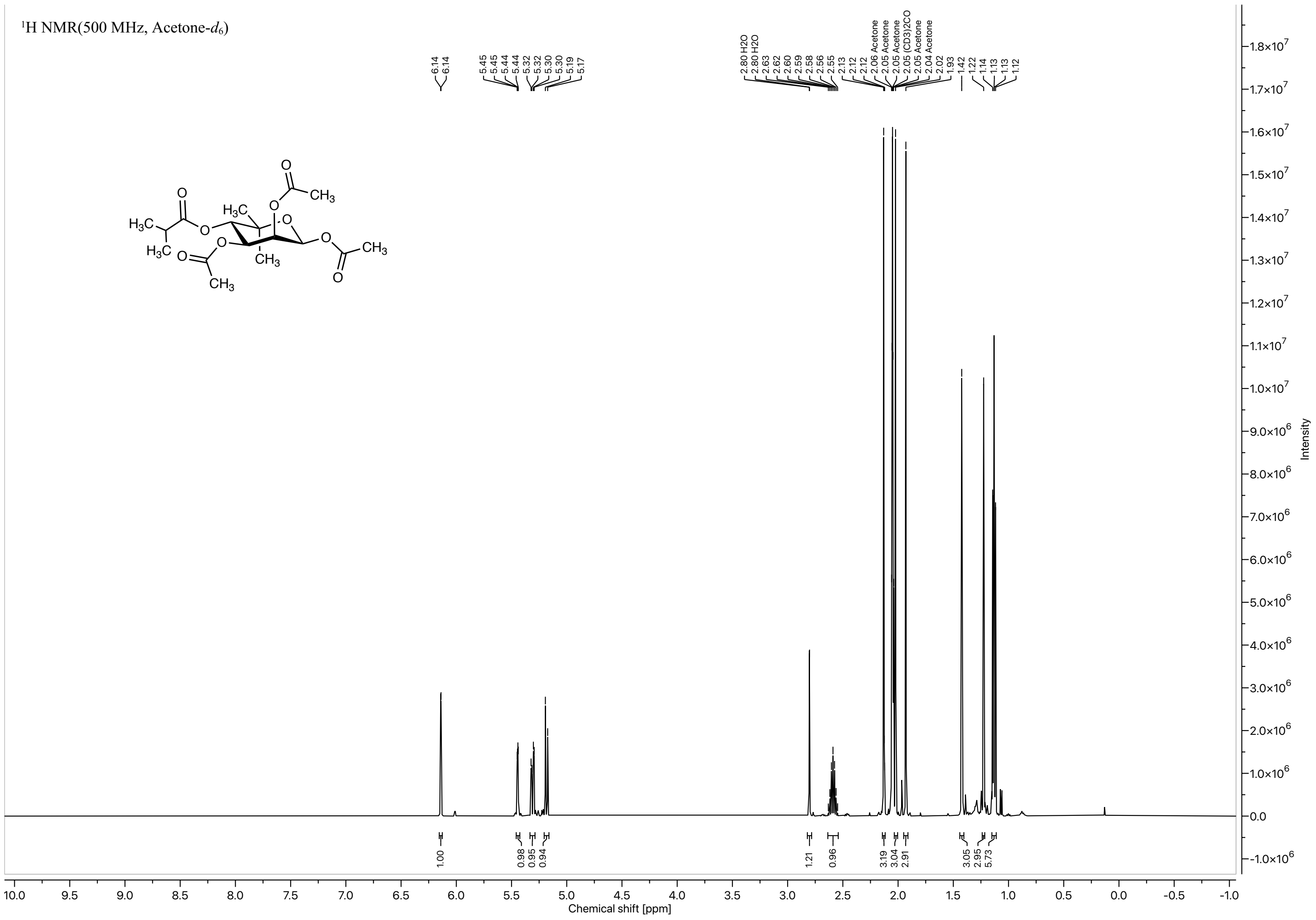

Figure 239: ^{1}H NMR spectrum of 1,2,3-tri-*O*-acetyl-4-*O*-demethyl-4-*O*-isobutyryl-β-D-noviose (8-β) in acetone-d_6

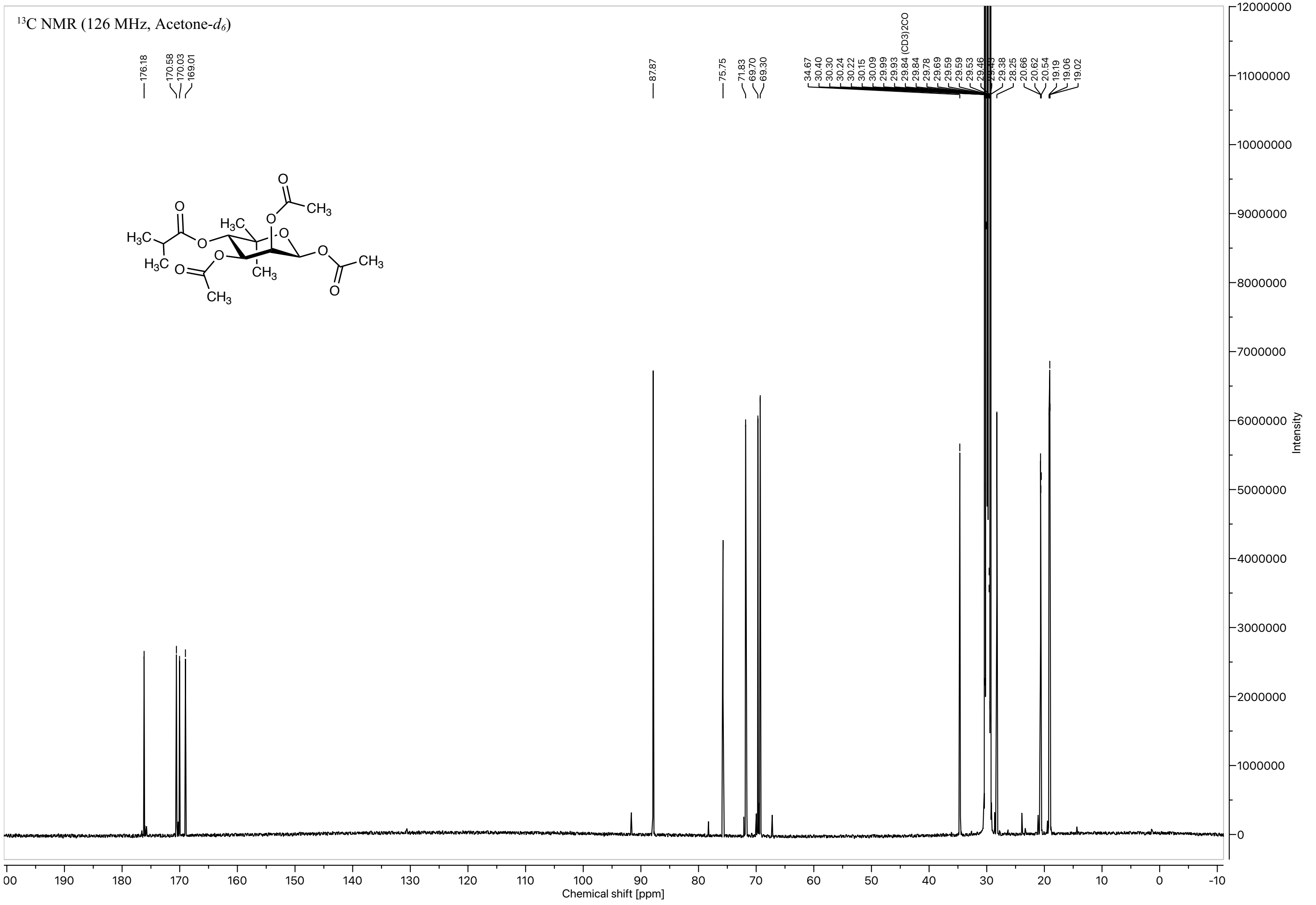

Figure 240: ^{13}C NMR spectrum of 1,2,3-tri-*O*-acetyl-4-*O*-demethyl-4-*O*-isobutyryl-β-D-noviose (8-β) in acetone-*d*$_6$

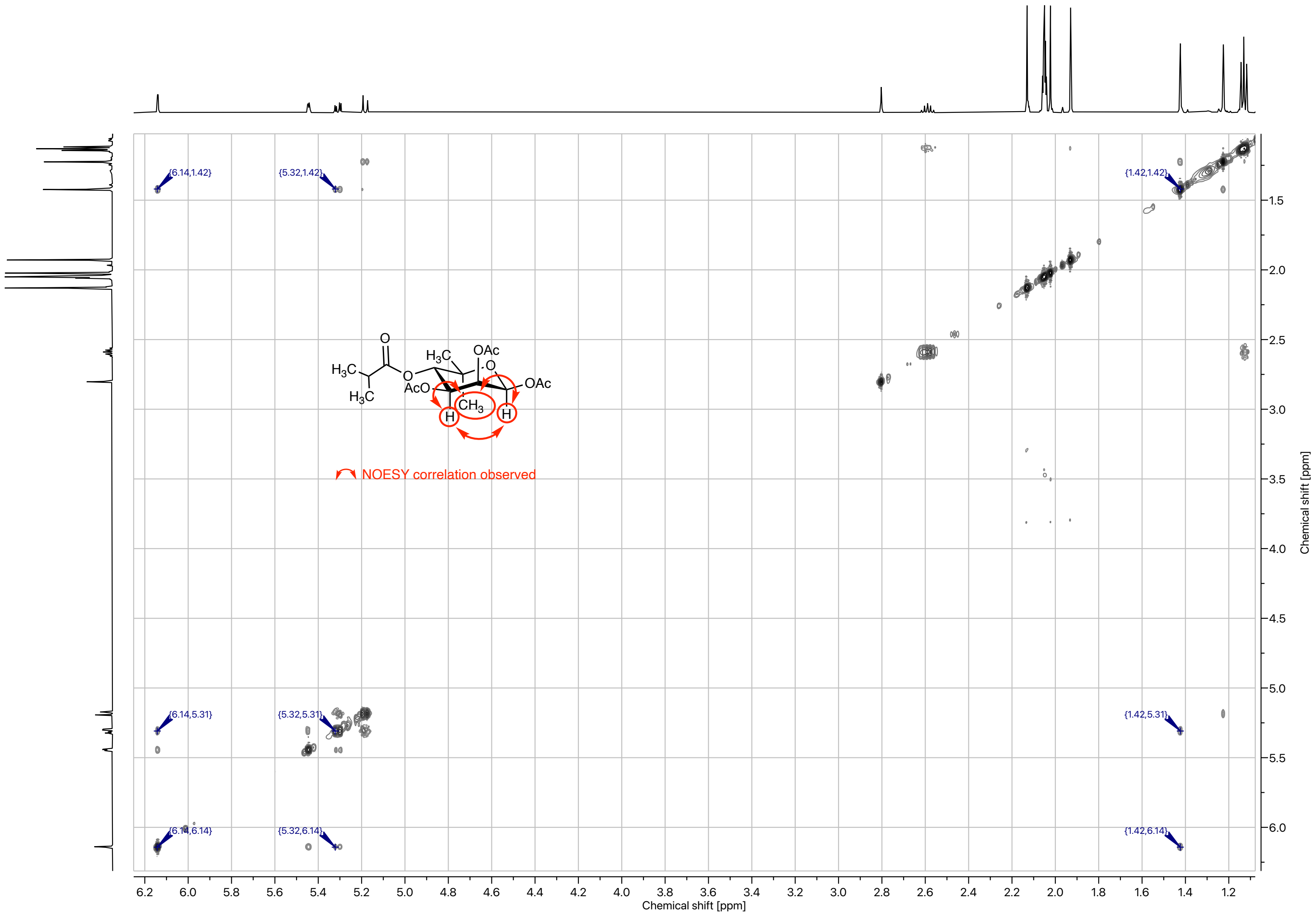

Figure 241: NOESY spectrum of 1,2,3-tri-*O*-acetyl-4-*O*-demethyl-4-*O*-isobutyryl-β-D-noviose (8-β) in acetone-d_6

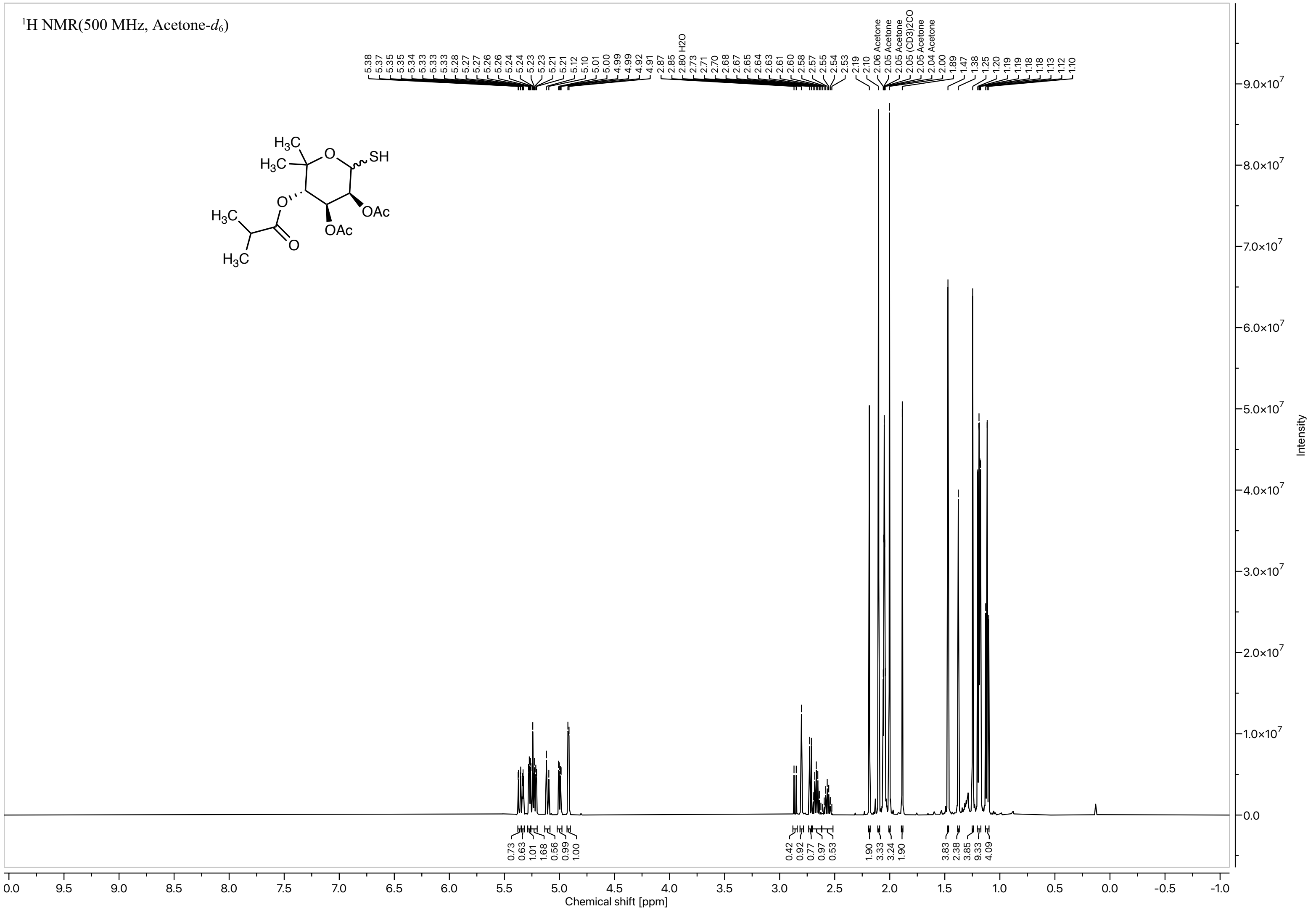

Figure 242: ^{1}H NMR spectrum of 2,3-di-*O*-acetyl-4-*O*-demethyl-4-*O*-isobutyryl-1-thio-D-noviose (9, mixture of anomers) in acetone-d_6

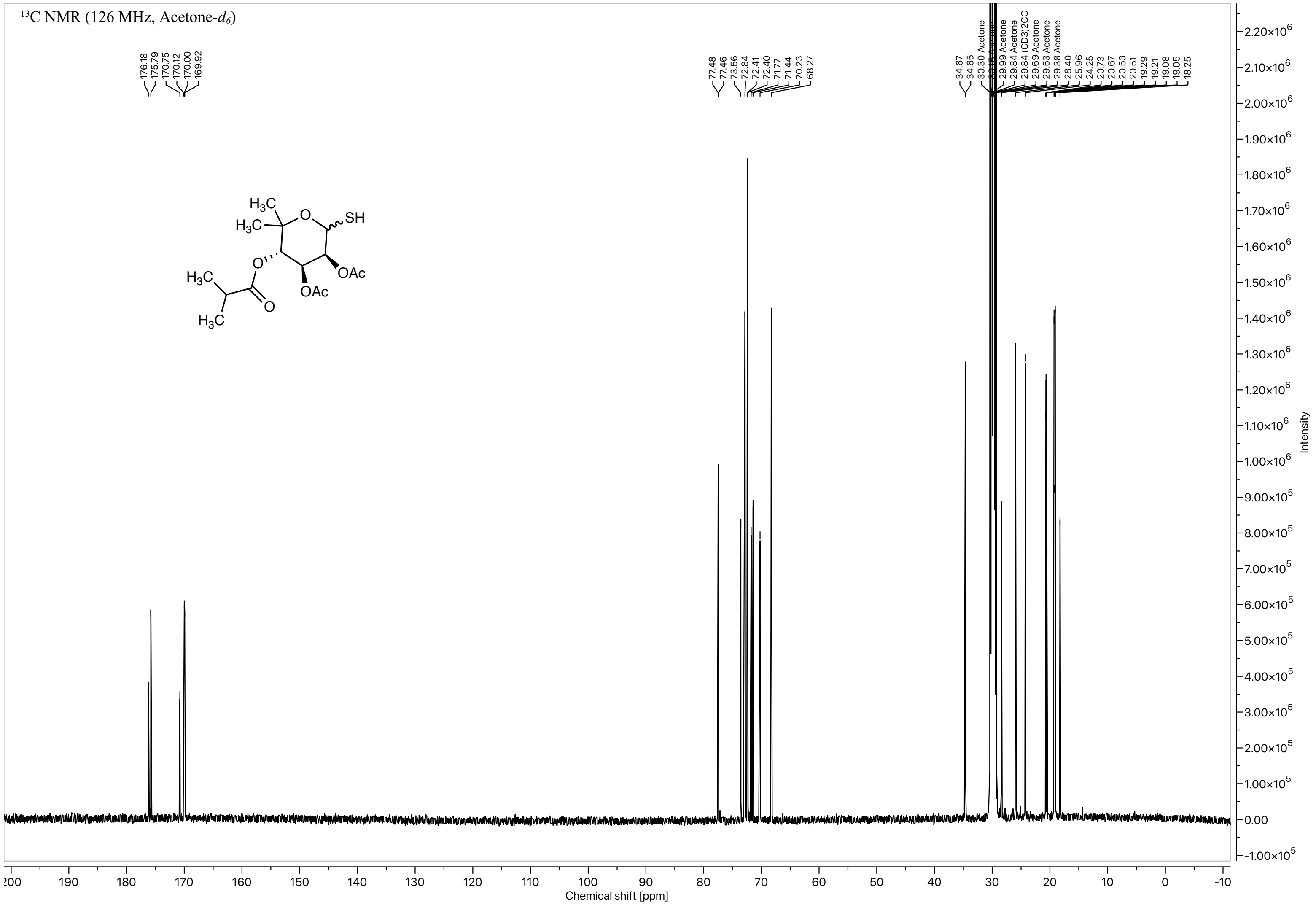

Figure 243: ^{13}C NMR spectrum of 2,3-di-*O*-acetyl-4-*O*-demethyl-4-*O*-isobutyryl-1-thio-D-noviose (9, mixture of anomers) in acetone-d_6

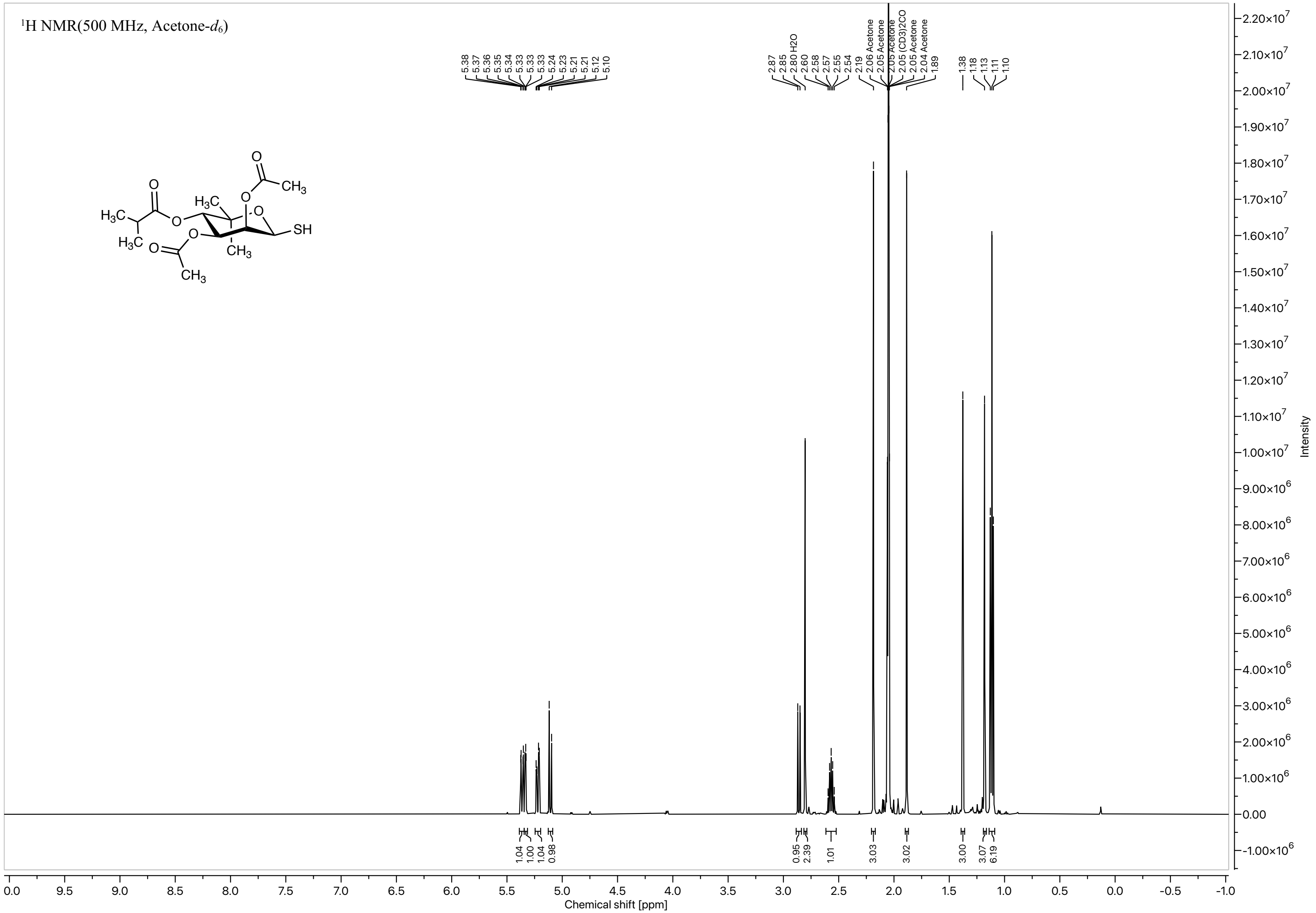

Figure 244: ^{1}H NMR spectrum of 2,3-di-*O*-acetyl-4-*O*-demethyl-4-*O*-isobutyryl-1-thio-β–D-noviose (9-β) in acetone-d_6

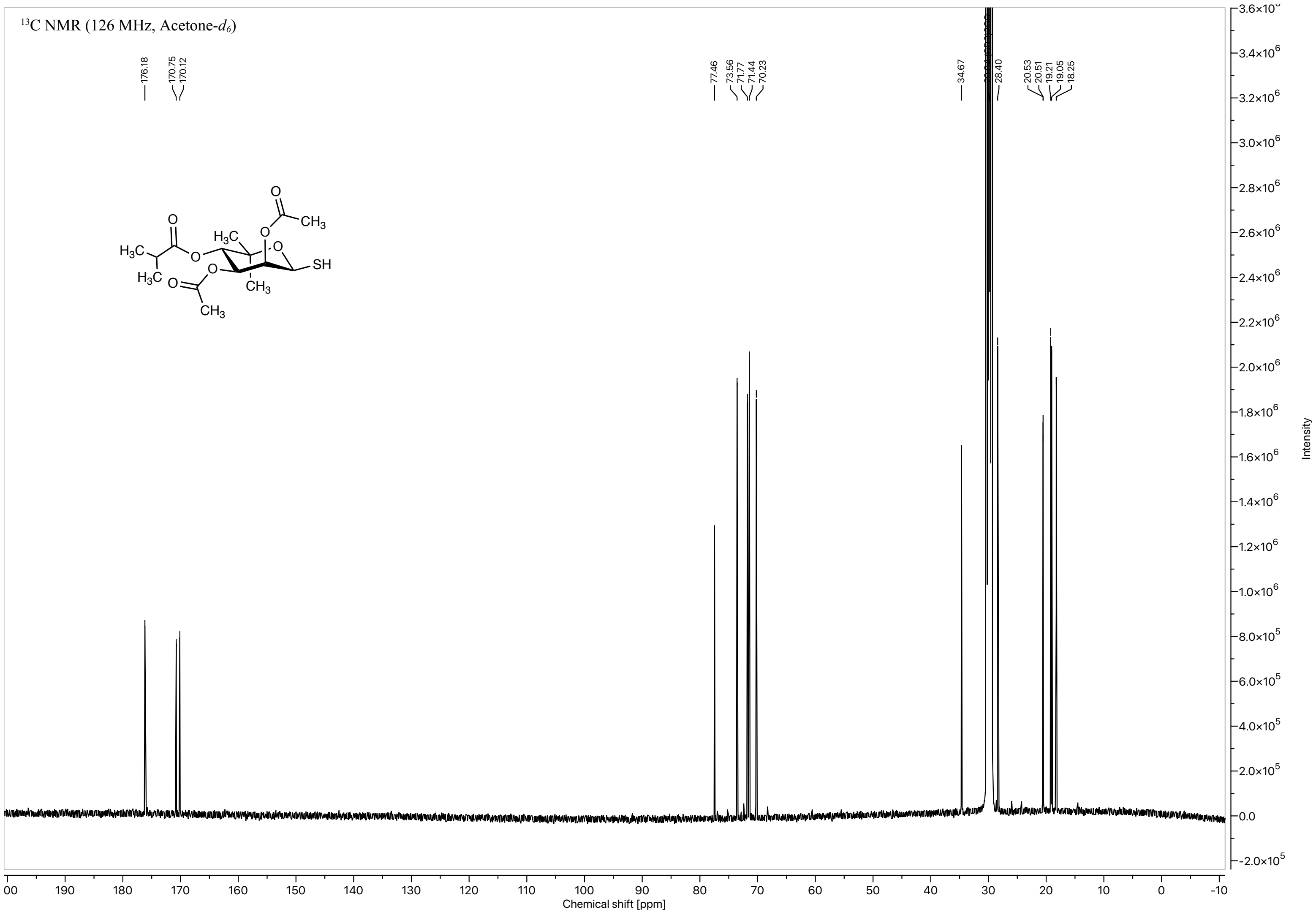

Figure 245: ^{13}C NMR spectrum of 2,3-di-*O*-acetyl-4-*O*-demethyl-4-*O*-isobutyryl-1-thio-β–D-noviose (9-β) in acetone-d_6

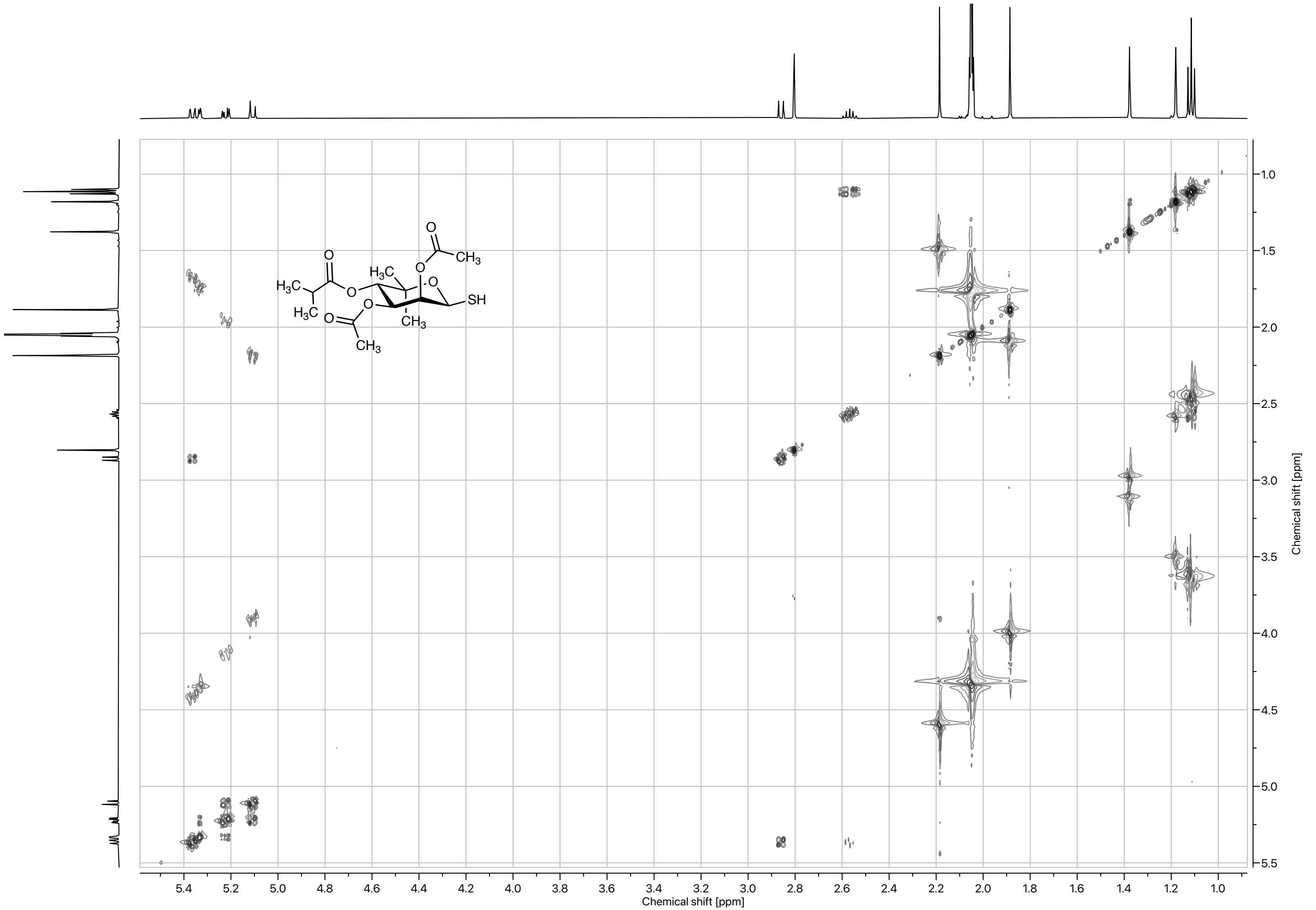

Figure 246: COSY spectrum of 2,3-di-*O*-acetyl-4-*O*-demethyl-4-*O*-isobutyryl-1-thio-β–D-noviose (9-β) in acetone-d_6

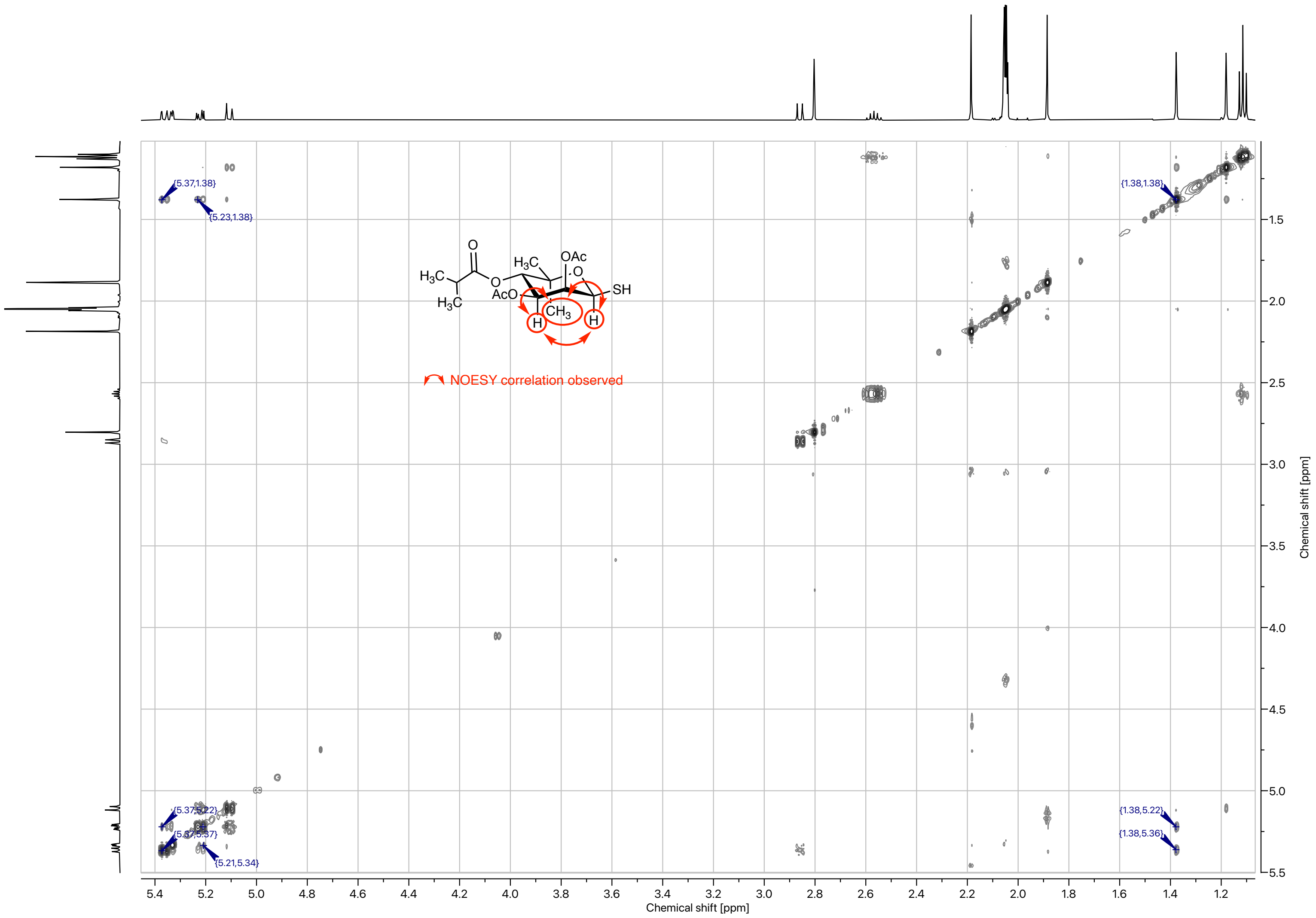

Figure 247: NOESY spectrum of 2,3-di-*O*-acetyl-4-*O*-demethyl-4-*O*-isobutyryl-1-thio-β–D-noviose (9-β) in acetone-d_6

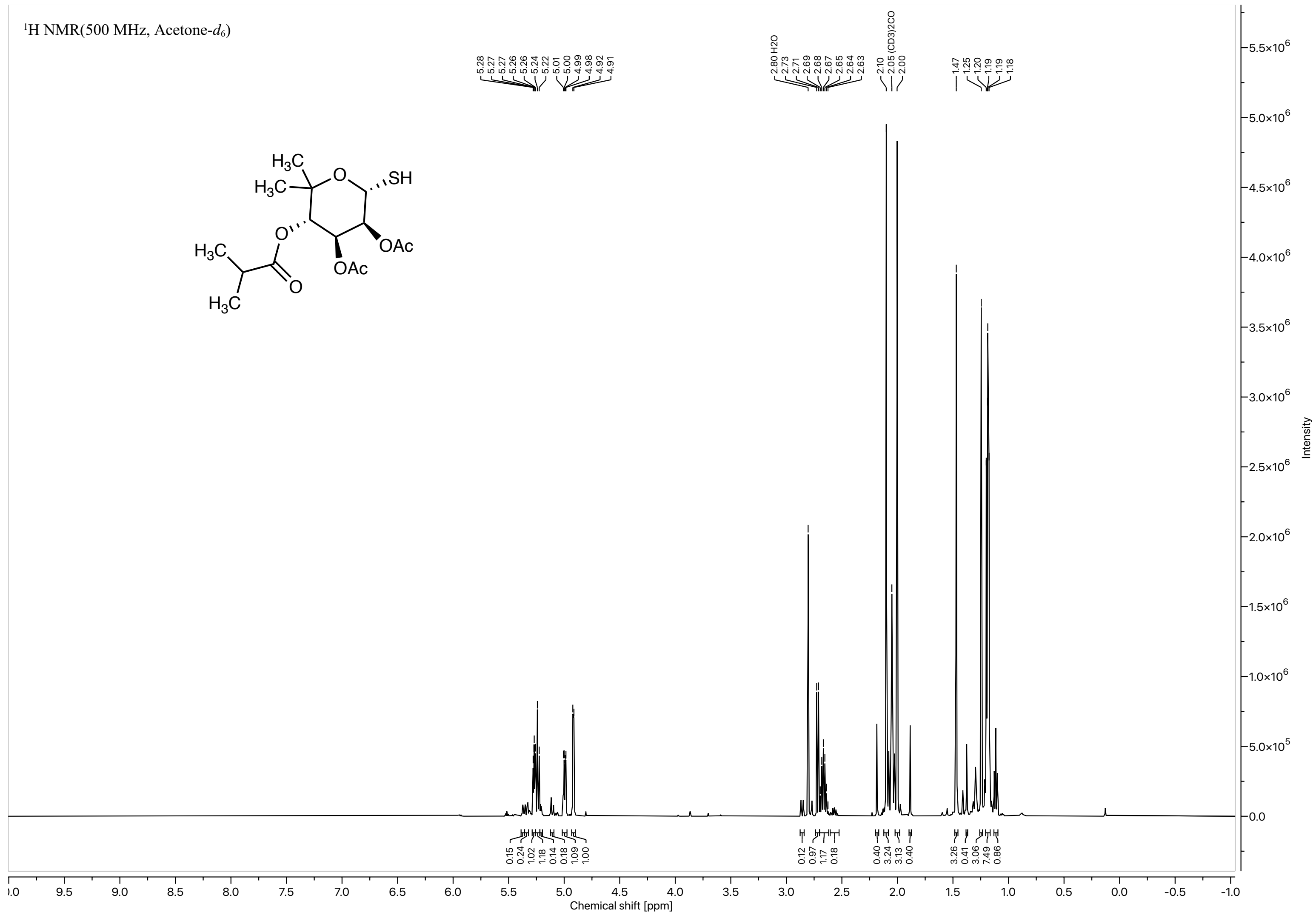

Figure 248: ^{1}H NMR spectrum of 2,3-di-*O*-acetyl-4-*O*-demethyl-4-*O*-isobutyryl-1-thio-α–D-noviose (9-α) in acetone-d_6

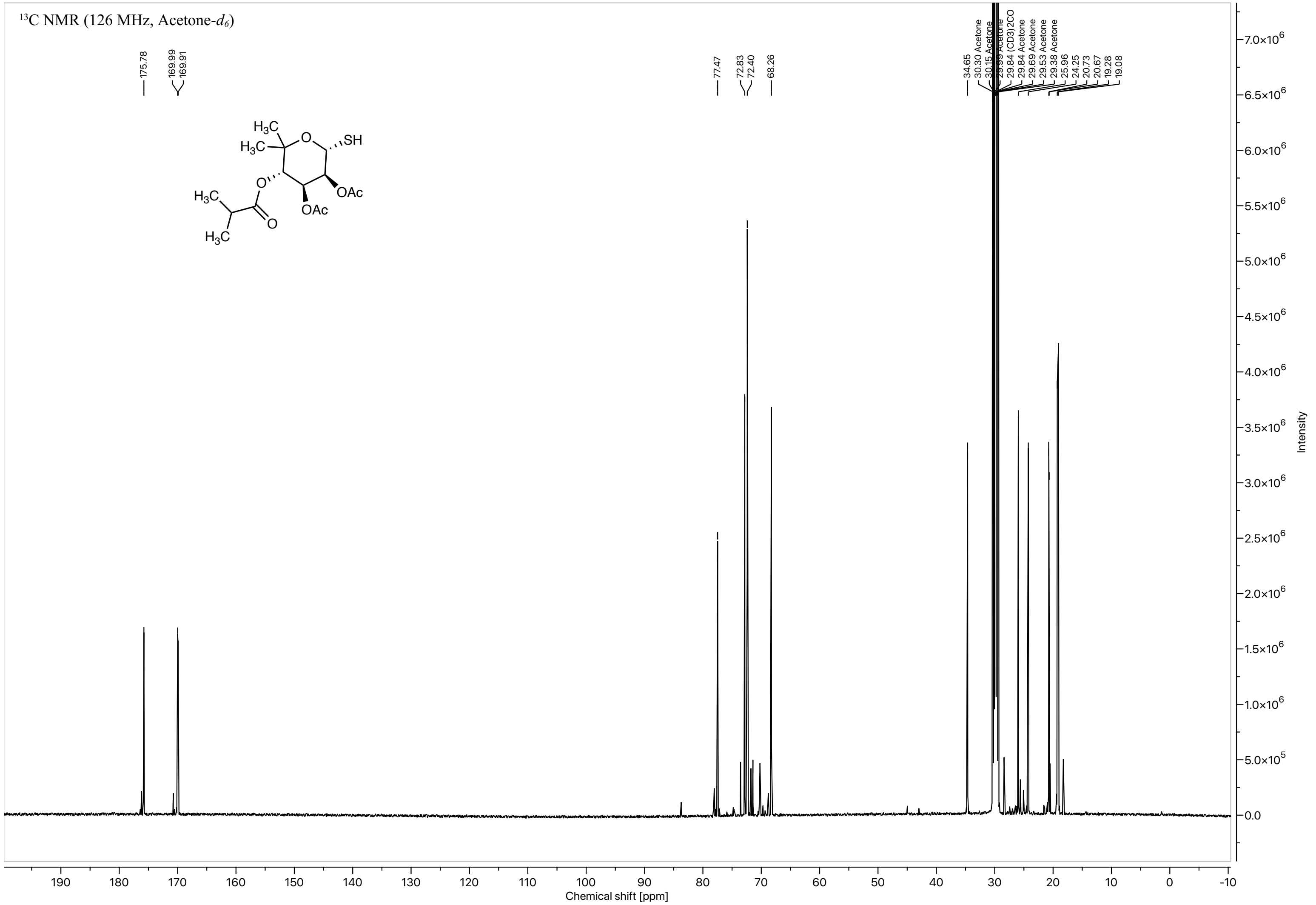

Figure 249: ^{13}C NMR spectrum of 2,3-di-*O*-acetyl-4-*O*-demethyl-4-*O*-isobutyryl-1-thio-α−D-noviose (9-α) in acetone-d_6

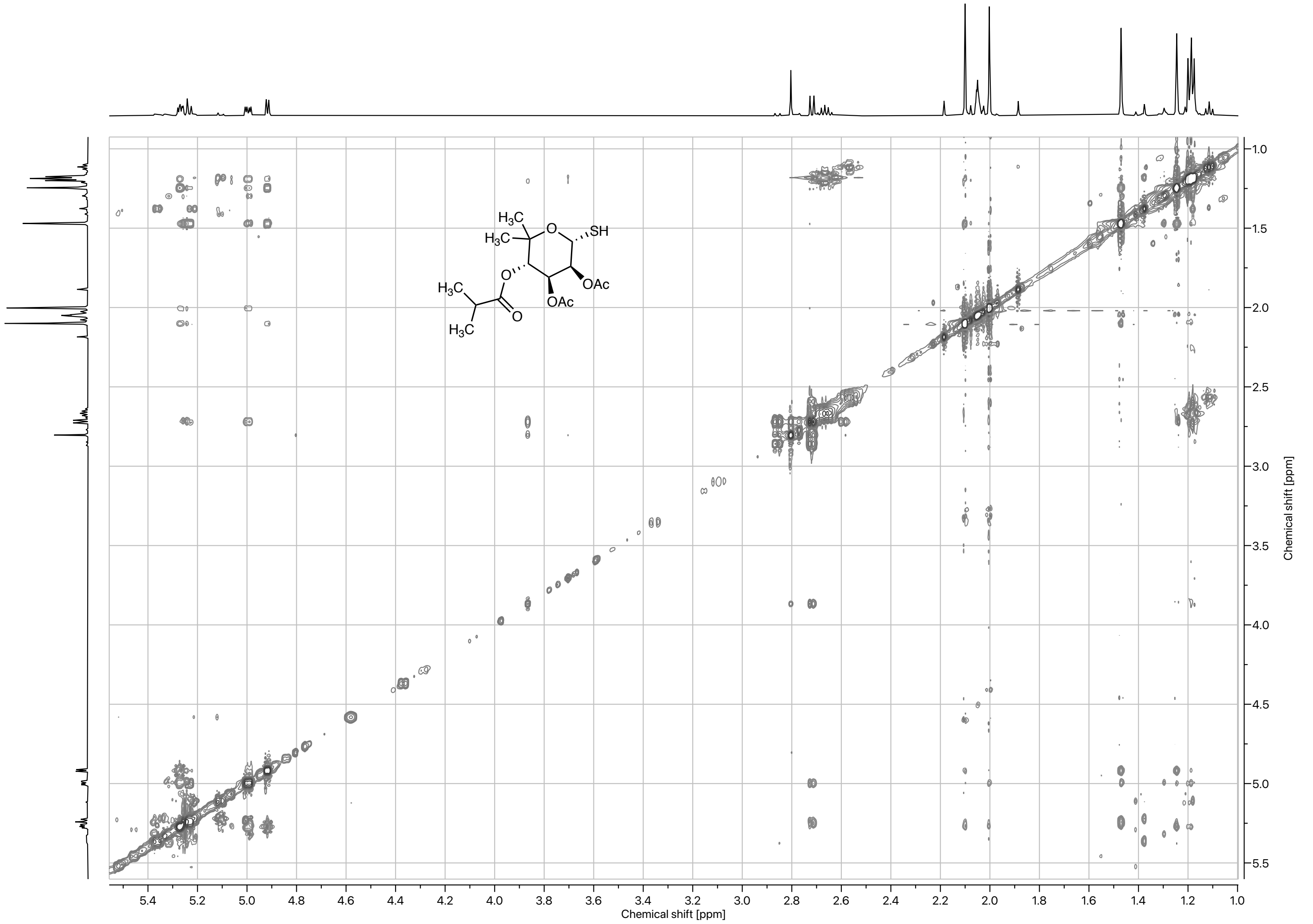

Figure 250: NOESY spectrum of 2,3-di-*O*-acetyl-4-*O*-demethyl-4-*O*-isobutyryl-1-thio-α–D-noviose (9-α) in acetone-d_6

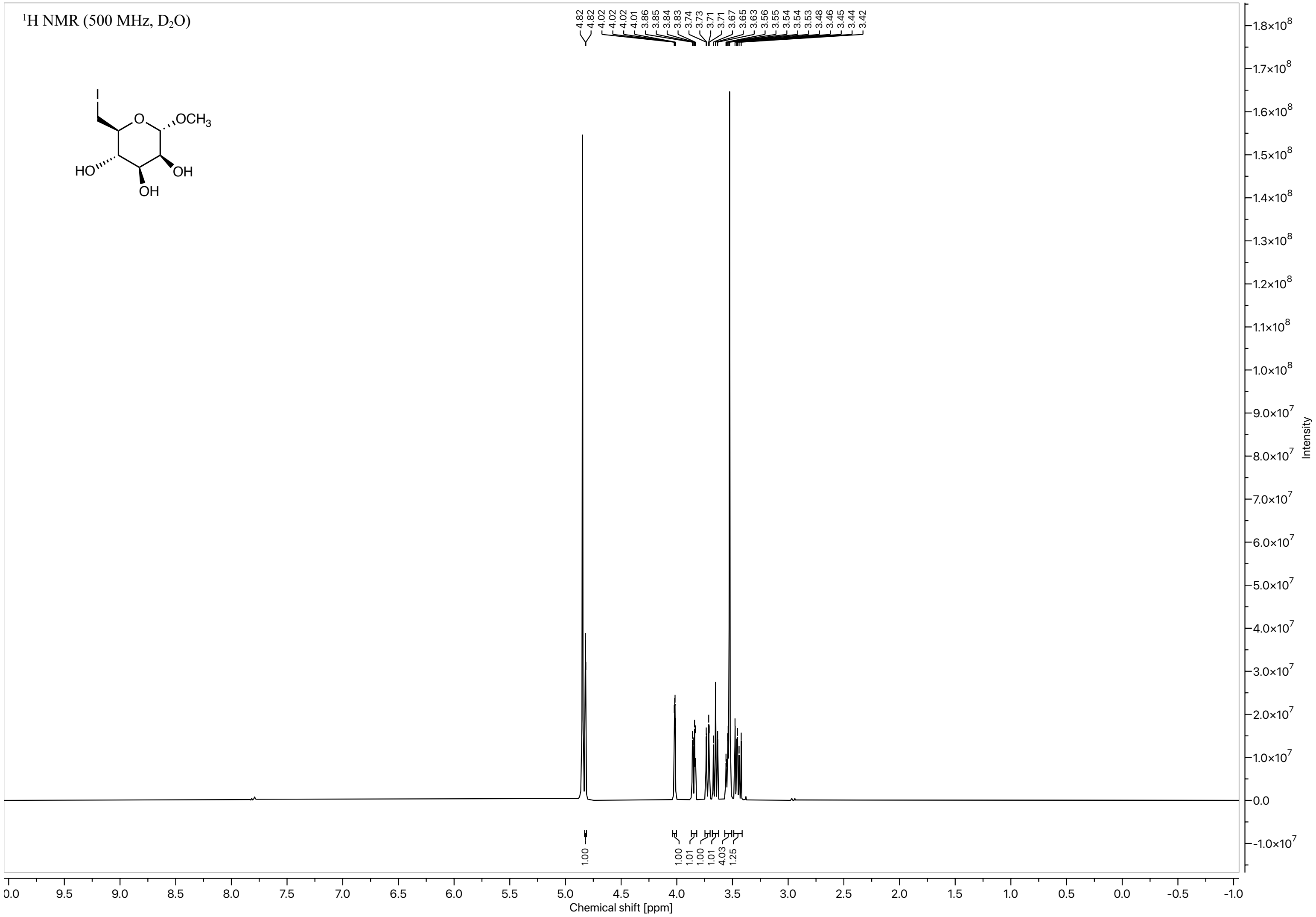

Figure 251: ¹H NMR spectrum of methyl 6-deoxy-6-iodo-α-D-mannopyranoside (10-I) in D_2O

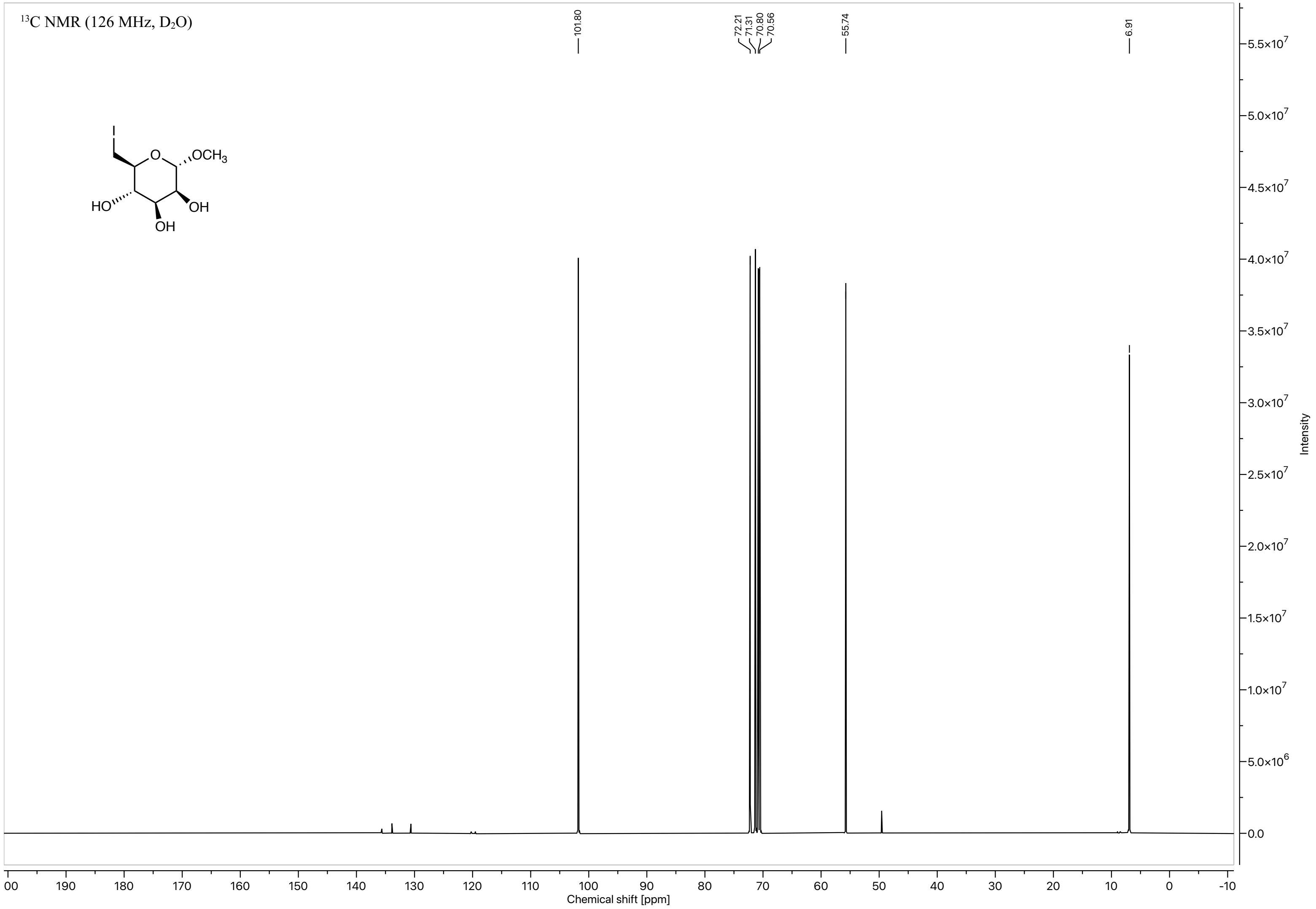

Figure 252: ^{13}C NMR spectrum of methyl 6-deoxy-6-iodo-α-D-mannopyranoside (10-I) in D_2O

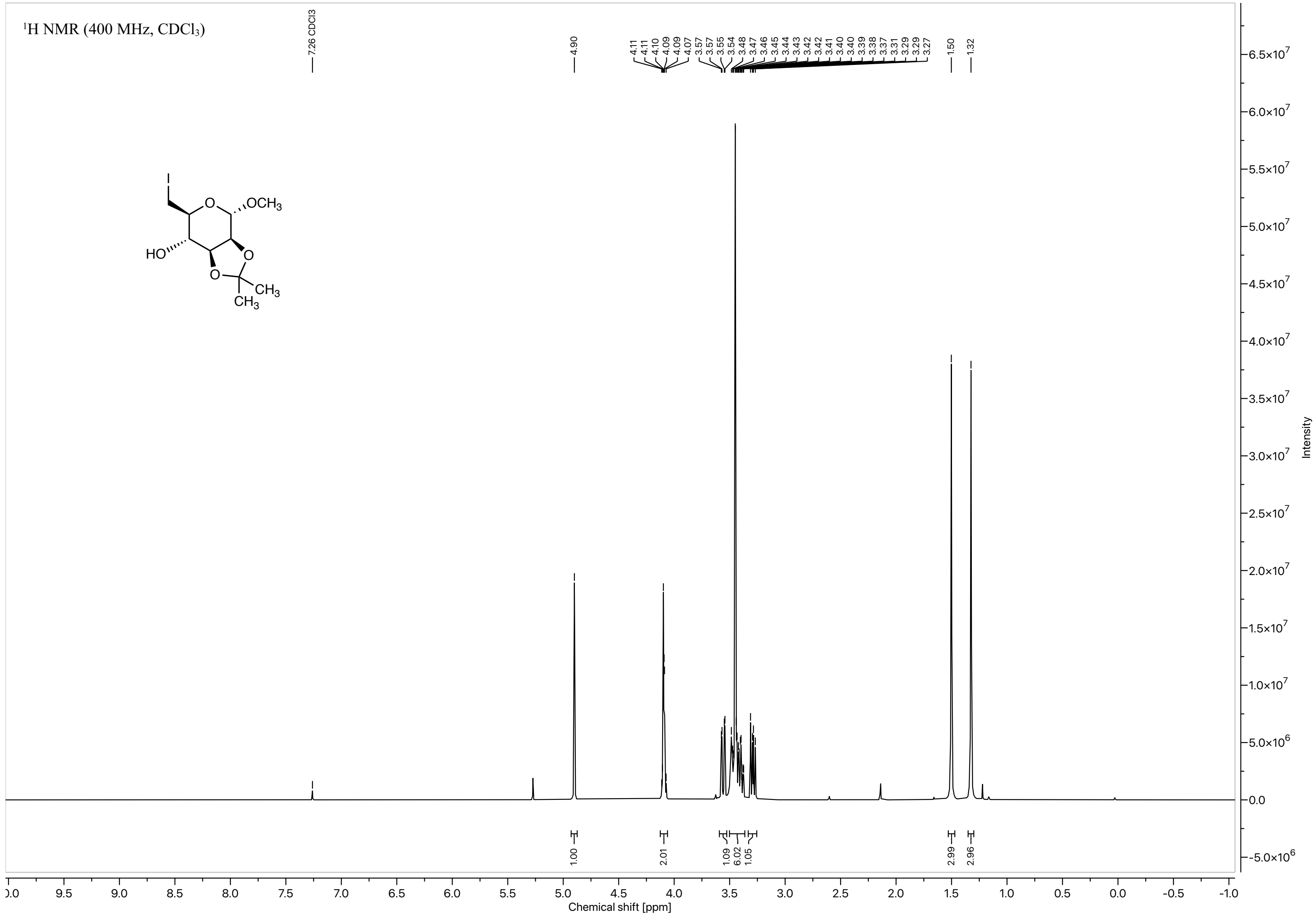

Figure 253: ^{1}H NMR spectrum of methyl 6-deoxy-6-iodo-2,3-O-isoproylidene-α-D-mannopyranoside (10) in $CDCl_3$

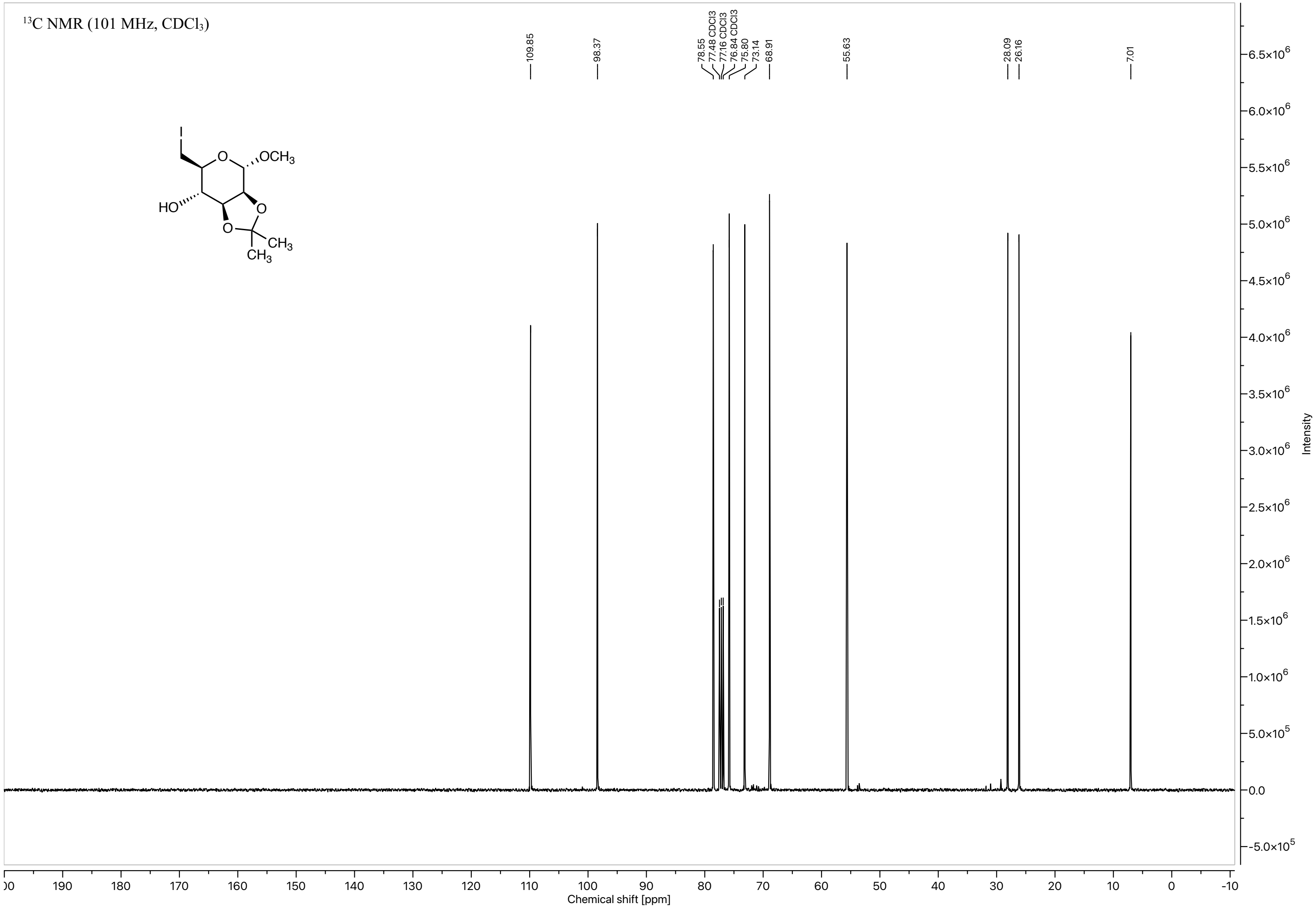

Figure 254: ^{13}C NMR spectrum of methyl 6-deoxy-6-iodo-2,3-O-isoproylidene-α-D-mannopyranoside (10) in $CDCl_3$

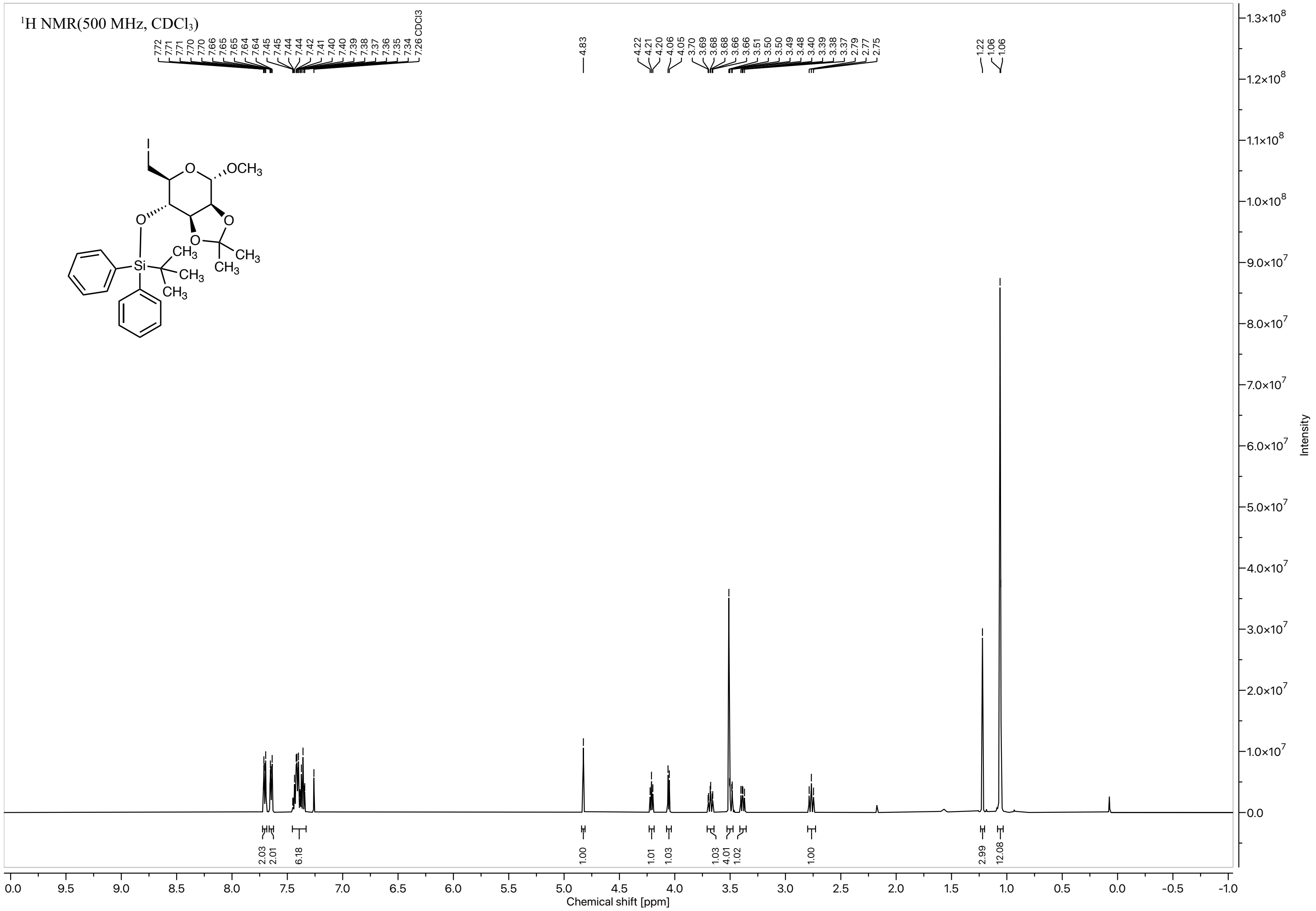

Figure 255: ^{1}H NMR spectrum of methyl 4-*O*-*tert*-butyldiphenylsilyloxy-6-deoxy-6-iodo-2,3-*O*-isoproylidene-α-D-mannopyranoside (11-I) in $CDCl_3$

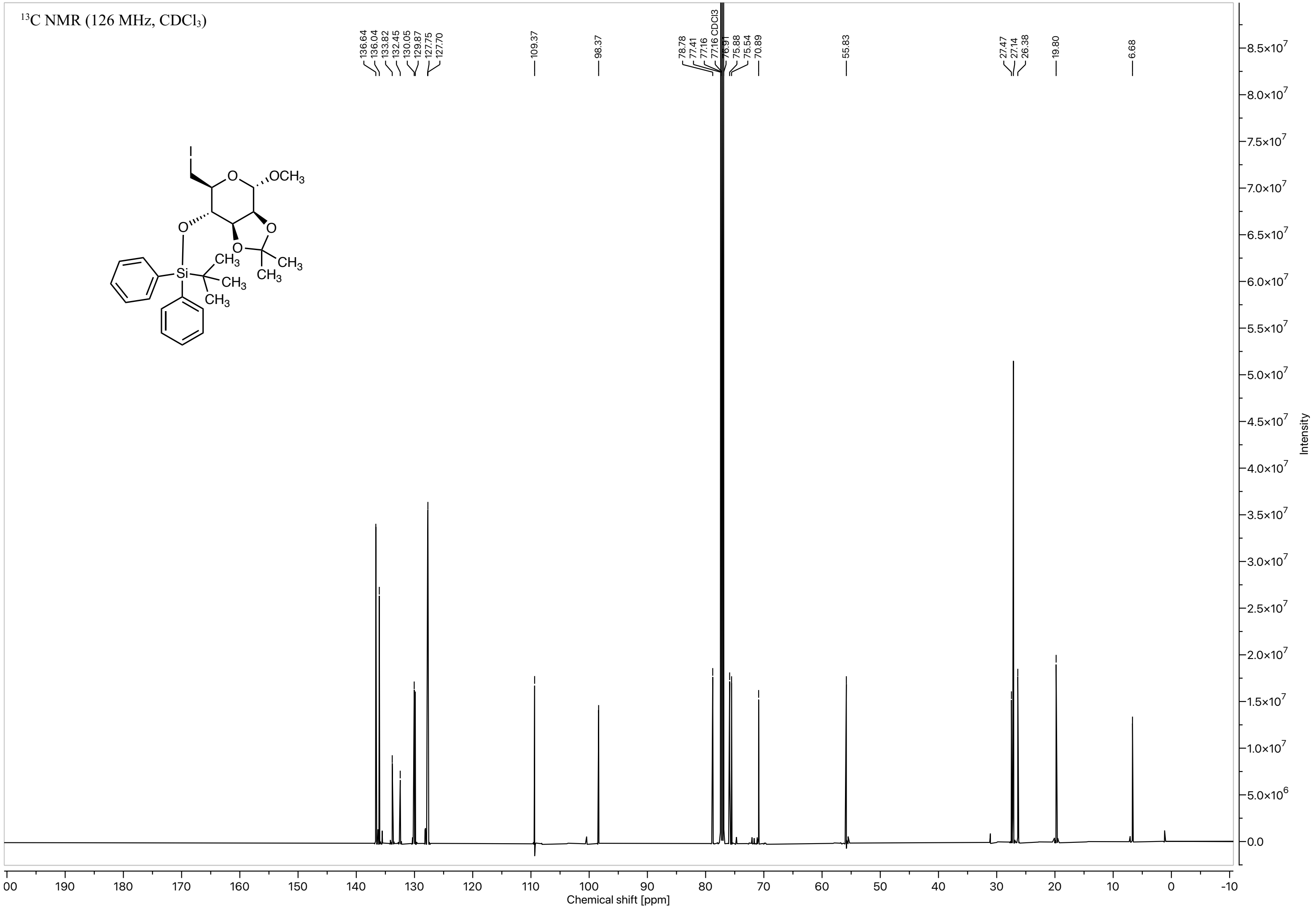

Figure 256: ^{13}C NMR spectrum of methyl 4-*O*-*tert*-butyldiphenylsilyloxy-6-deoxy-6-iodo-2,3-*O*-isoproylidene-α-D-mannopyranoside (11-I) in $CDCl_3$

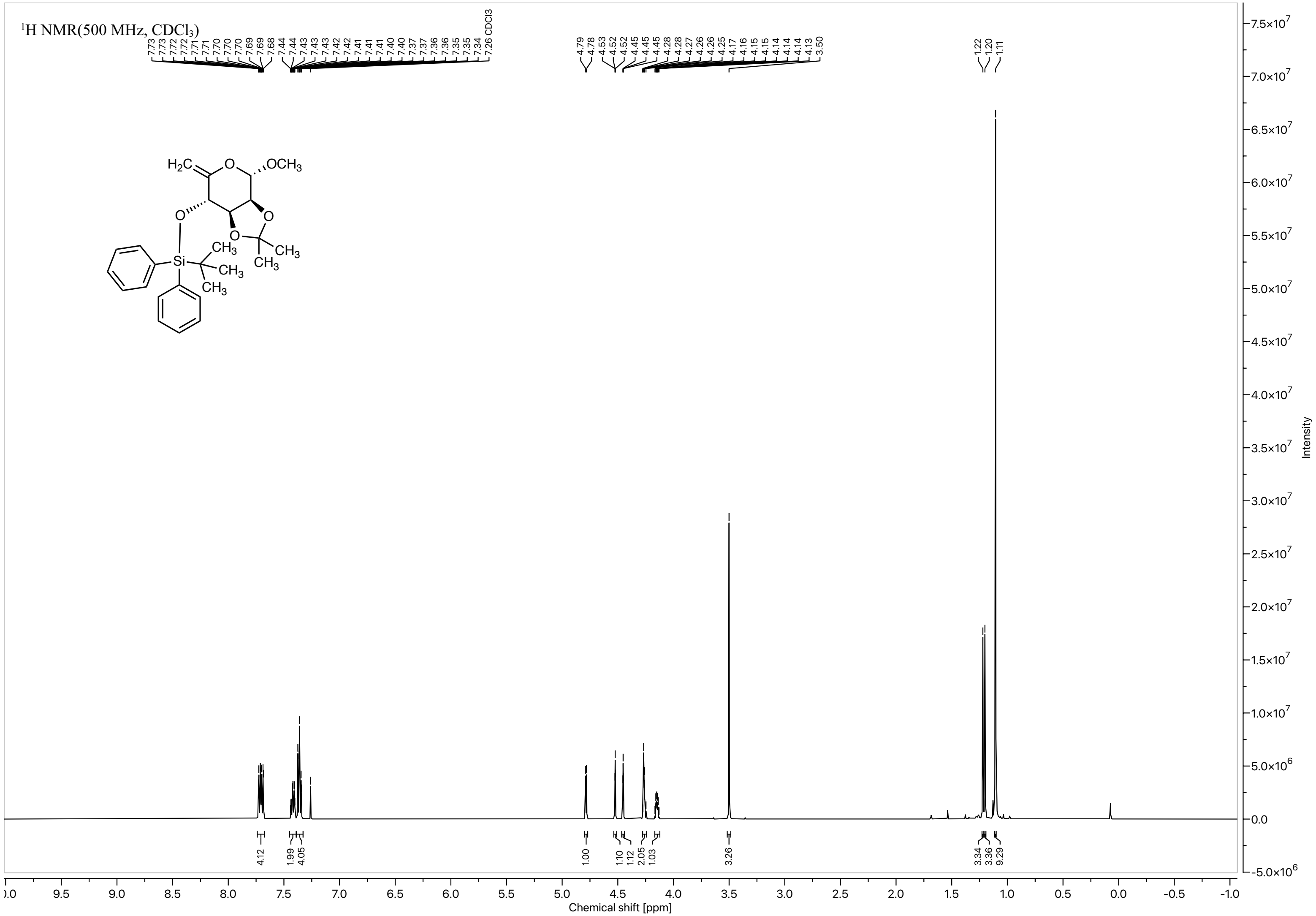

Figure 257: ^{1}H NMR spectrum of methyl 4-*O*-*tert*-butyldiphenylsilyloxy-6-deoxy-2,3-*O*-isoproylidene-α-D-manno-hex-5-enopyranoside (11) in $CDCl_3$

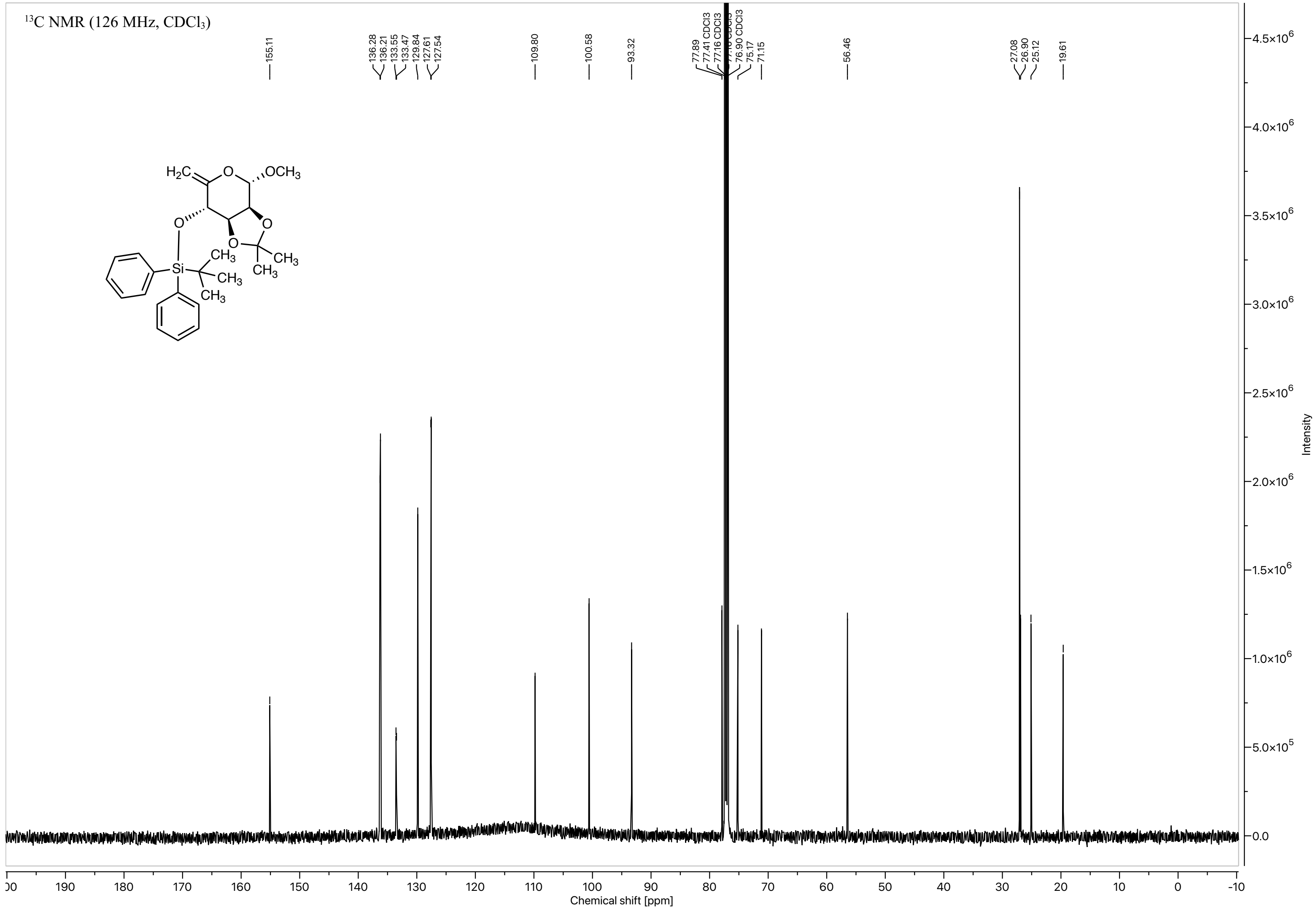

Figure 258: ^{13}C NMR spectrum of methyl 4-*O*-*tert*-butyldiphenylsilyloxy-6-deoxy-2,3-*O*-isoproylidene-α-D-manno-hex-5-enopyranoside (11) in $CDCl_3$

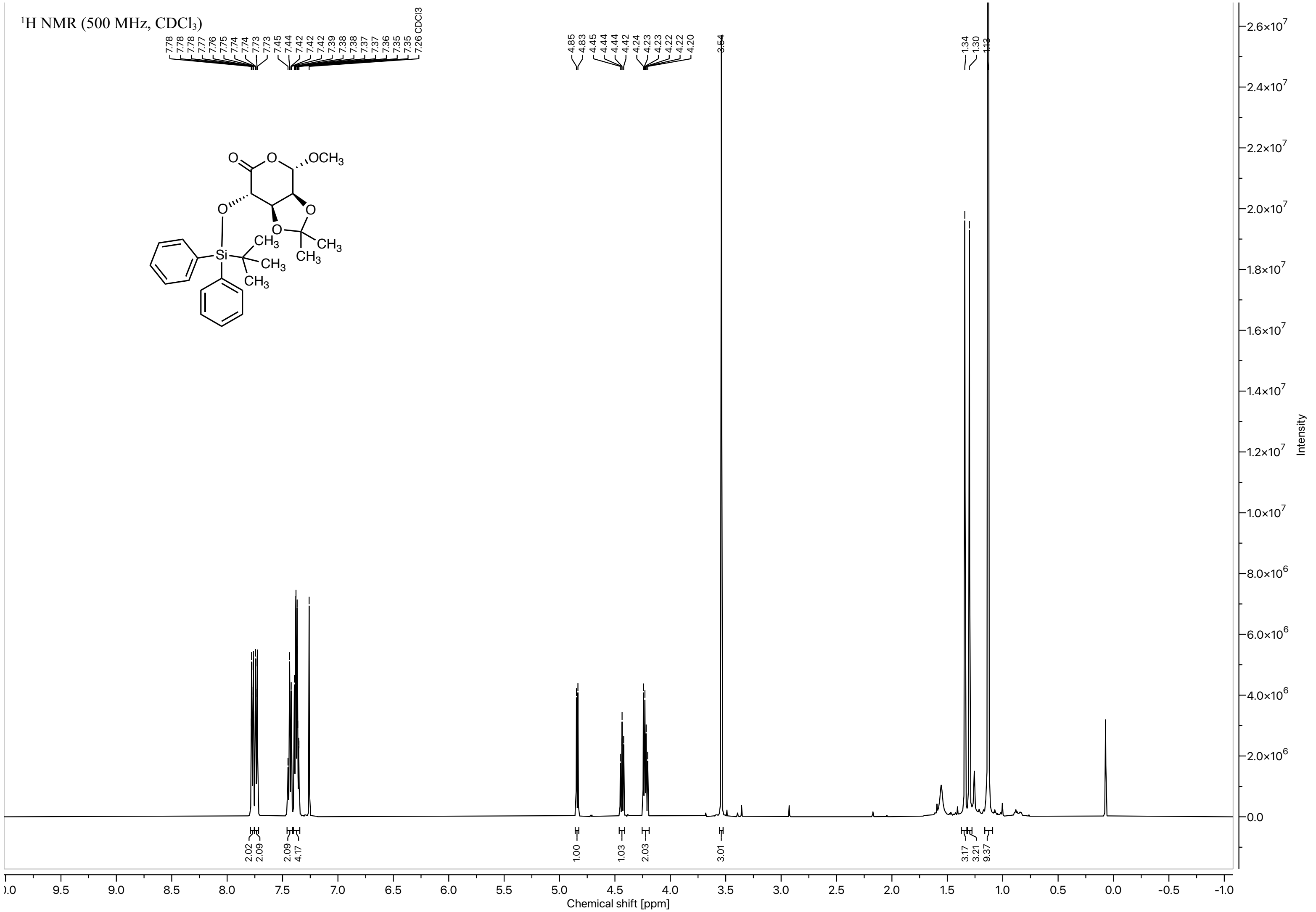

Figure 259: ^{1}H NMR spectrum of (4S,7S,7aR)-7-((tert-butyldiphenylsilyl)oxy)-4-methoxy-2,2-dimethyltetrahydro-6H-[1,3]dioxolo[4,5-c]pyran-6-one (12) in $CDCl_3$

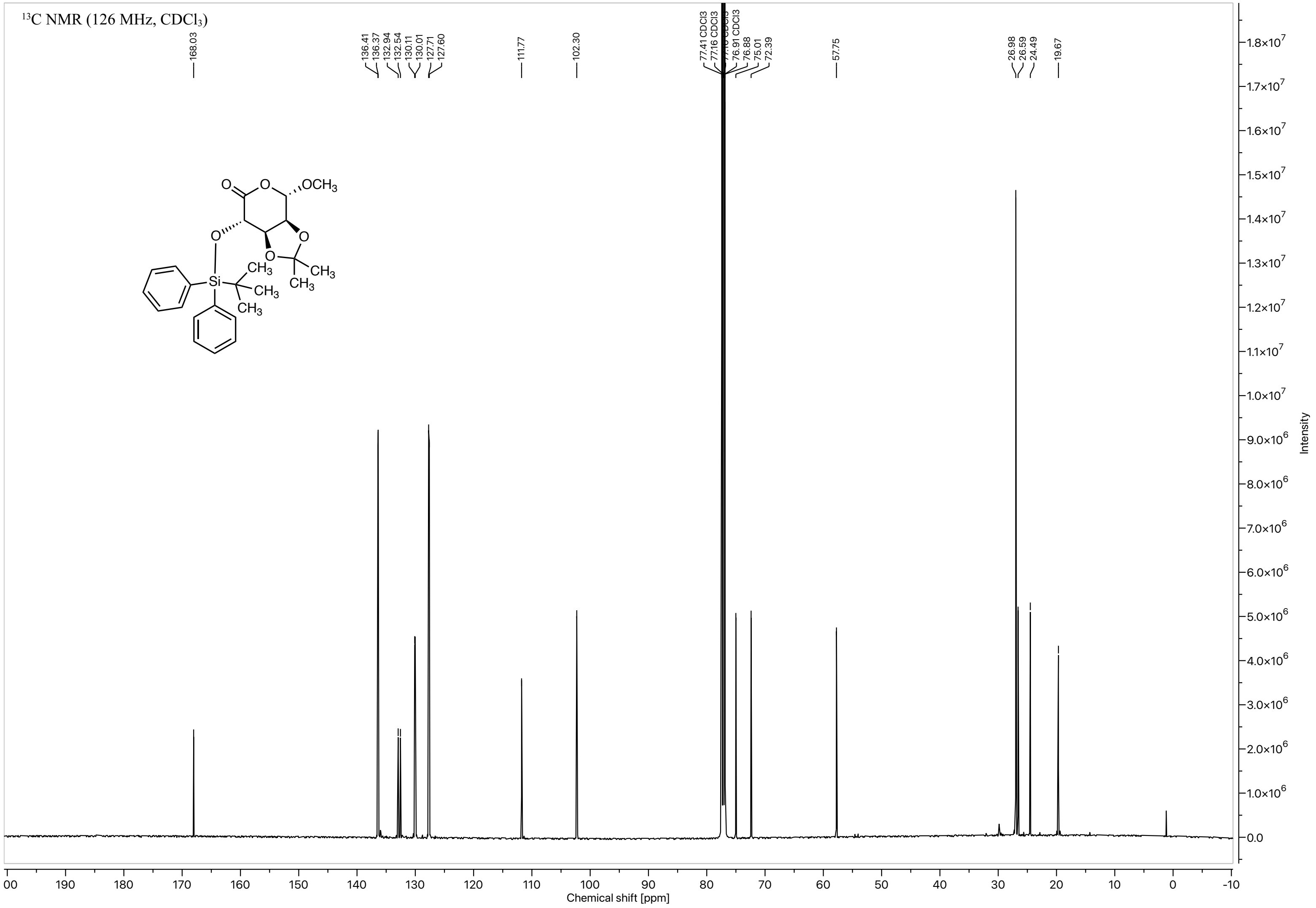

Figure 260: ^{13}C NMR spectrum of (4S,7S,7aR)-7-((tert-butyldiphenylsilyl)oxy)-4-methoxy-2,2-dimethyltetrahydro-6H-[1,3]dioxolo[4,5-c]pyran-6-one (12) in $CDCl_3$

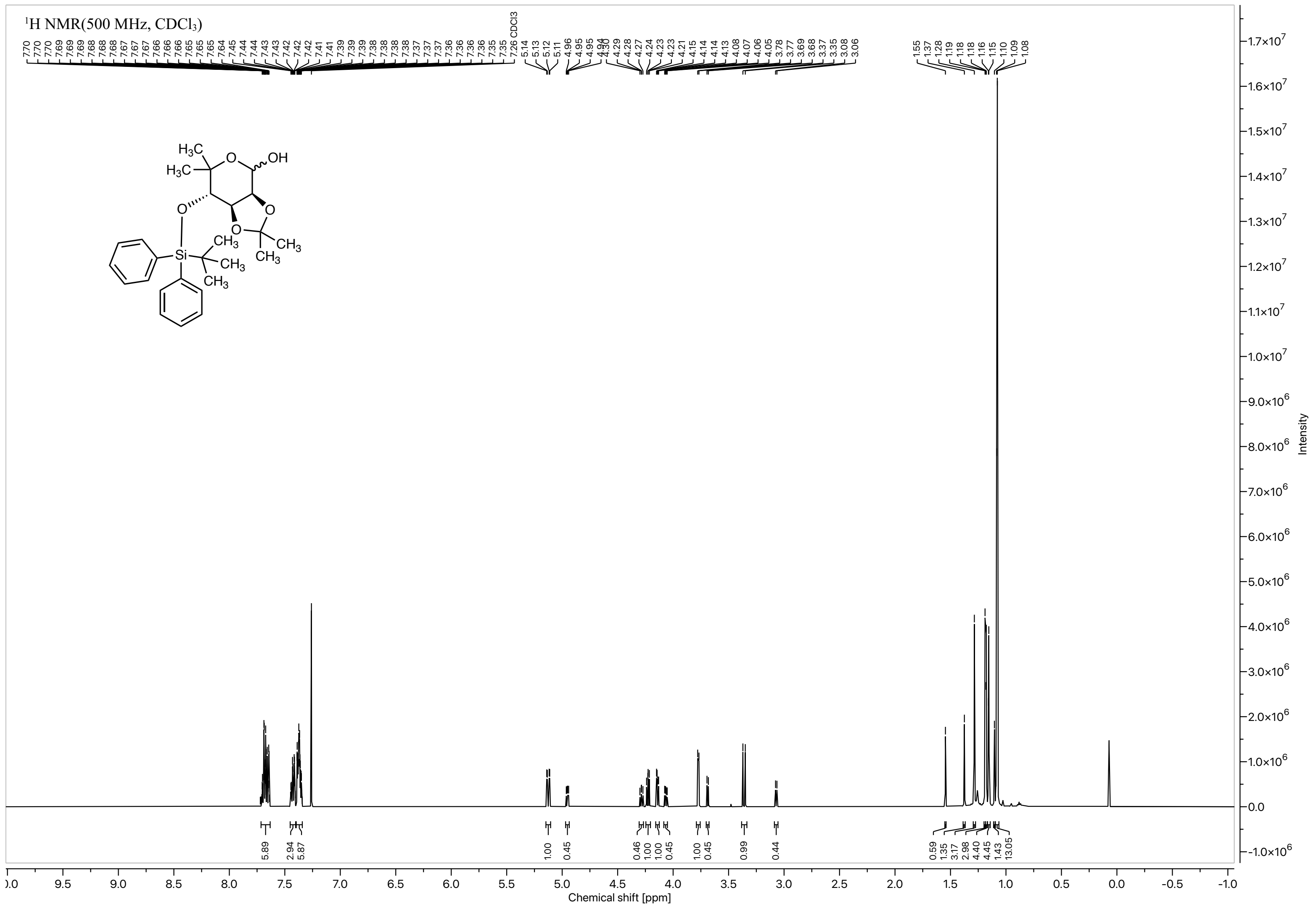

Figure 261: ^{1}H NMR spectrum of (7S,7aR)-7-((tert-butyldiphenylsilyl)oxy)-2,2,6,6-tetramethyltetrahydro-4H-[1,3]dioxolo[4,5-c]pyran-4-ol (13-I) in $CDCl_3$

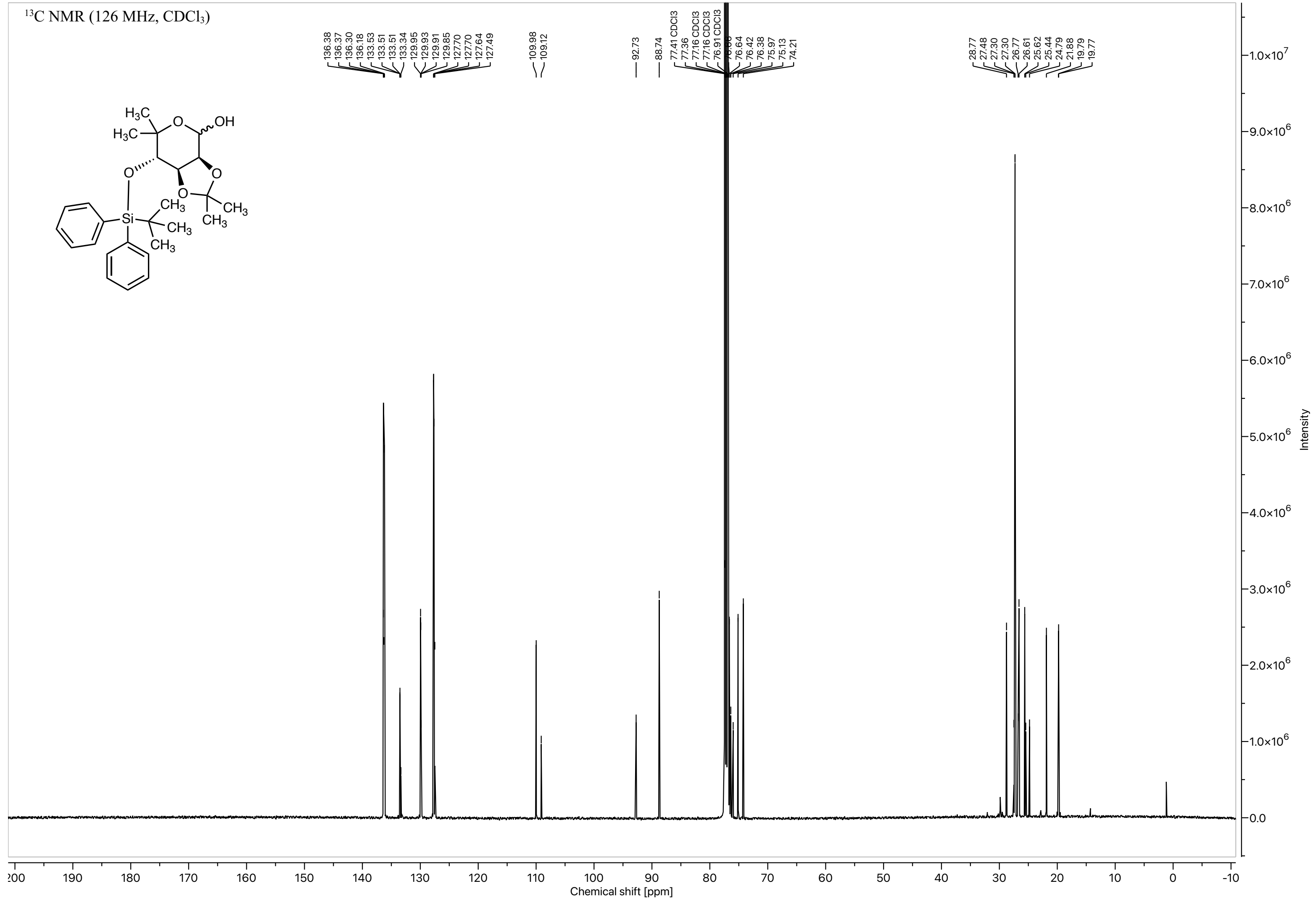

Figure 262: ^{13}C NMR spectrum of (7S,7aR)-7-((tert-butyldiphenylsilyl)oxy)-2,2,6,6-tetramethyltetrahydro-4H-[1,3]dioxolo[4,5-c]pyran-4-ol (13-I) in $CDCl_3$

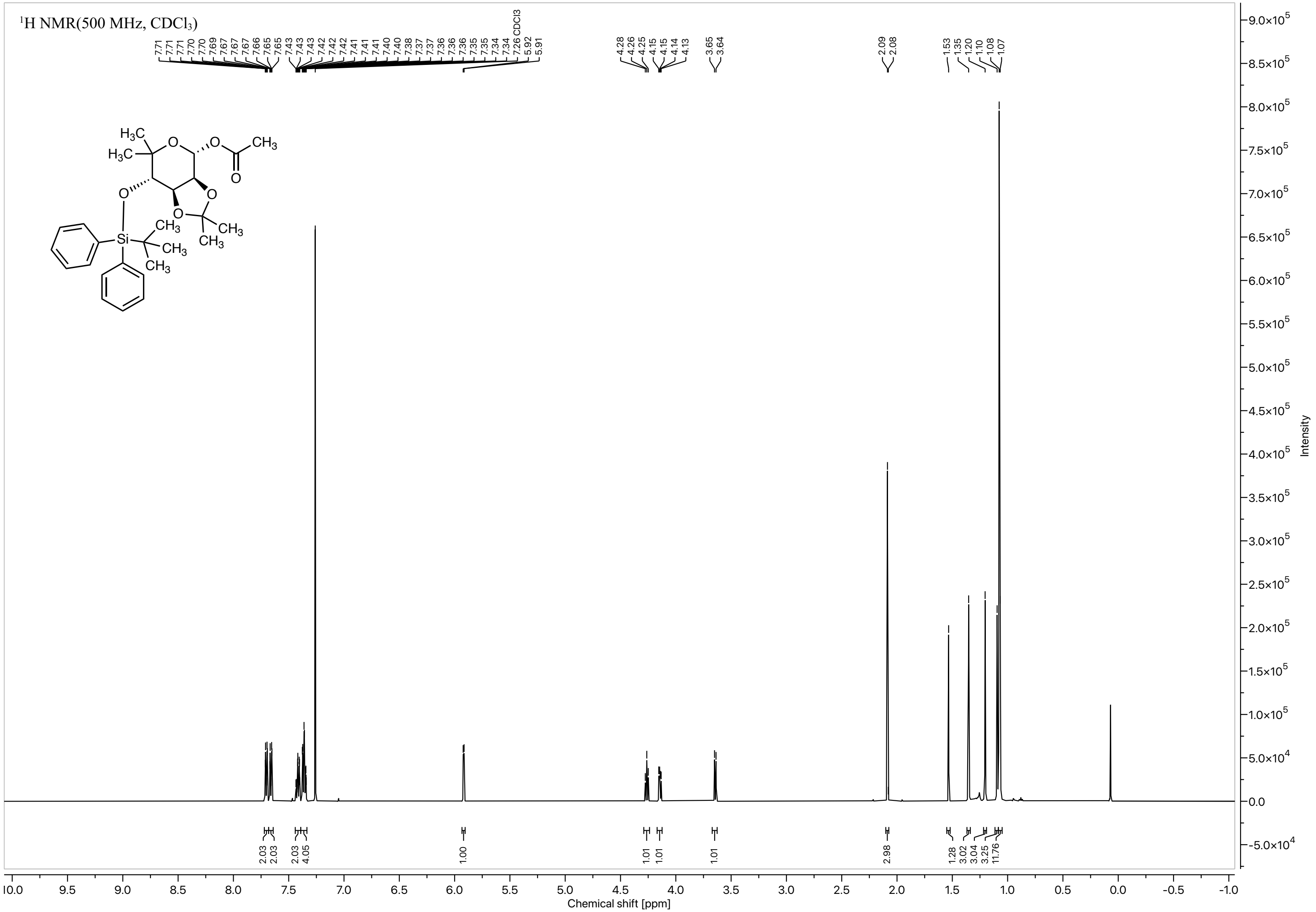

Figure 263: ¹H NMR spectrum of (3aS,4R,7S,7aR)-7-((tert-butyldiphenylsilyl)oxy)-2,2,6,6-tetramethyltetrahydro-4H-[1,3]dioxolo[4,5-c]pyran-4-yl acetate (13-α) in $CDCl_3$

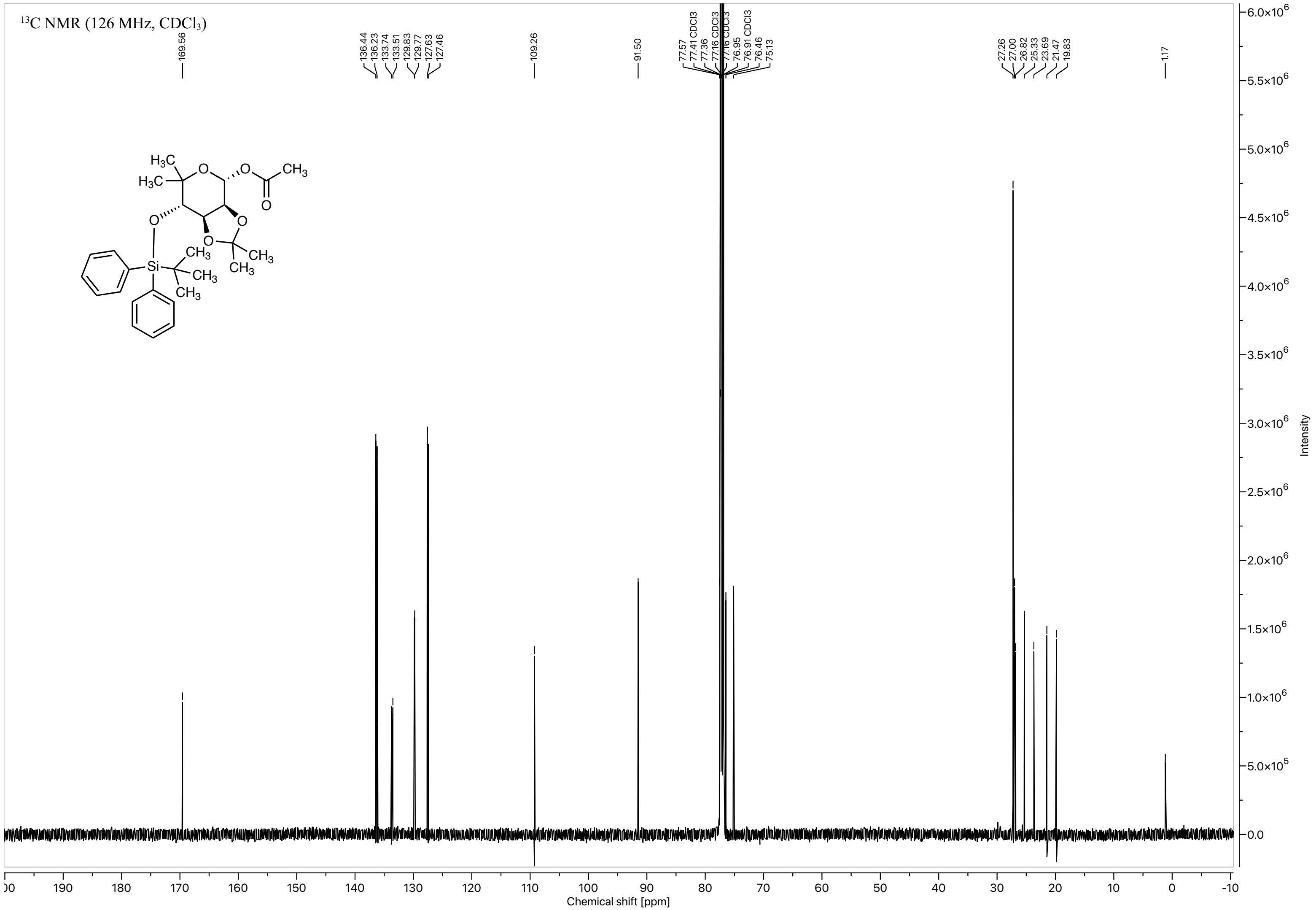

Figure 264: ^{13}C NMR spectrum of (3aS,4R,7S,7aR)-7-((tert-butyldiphenylsilyl)oxy)-2,2,6,6-tetramethyltetrahydro-4H-[1,3]dioxolo[4,5-c]pyran-4-yl acetate (13-α) in $CDCl_3$

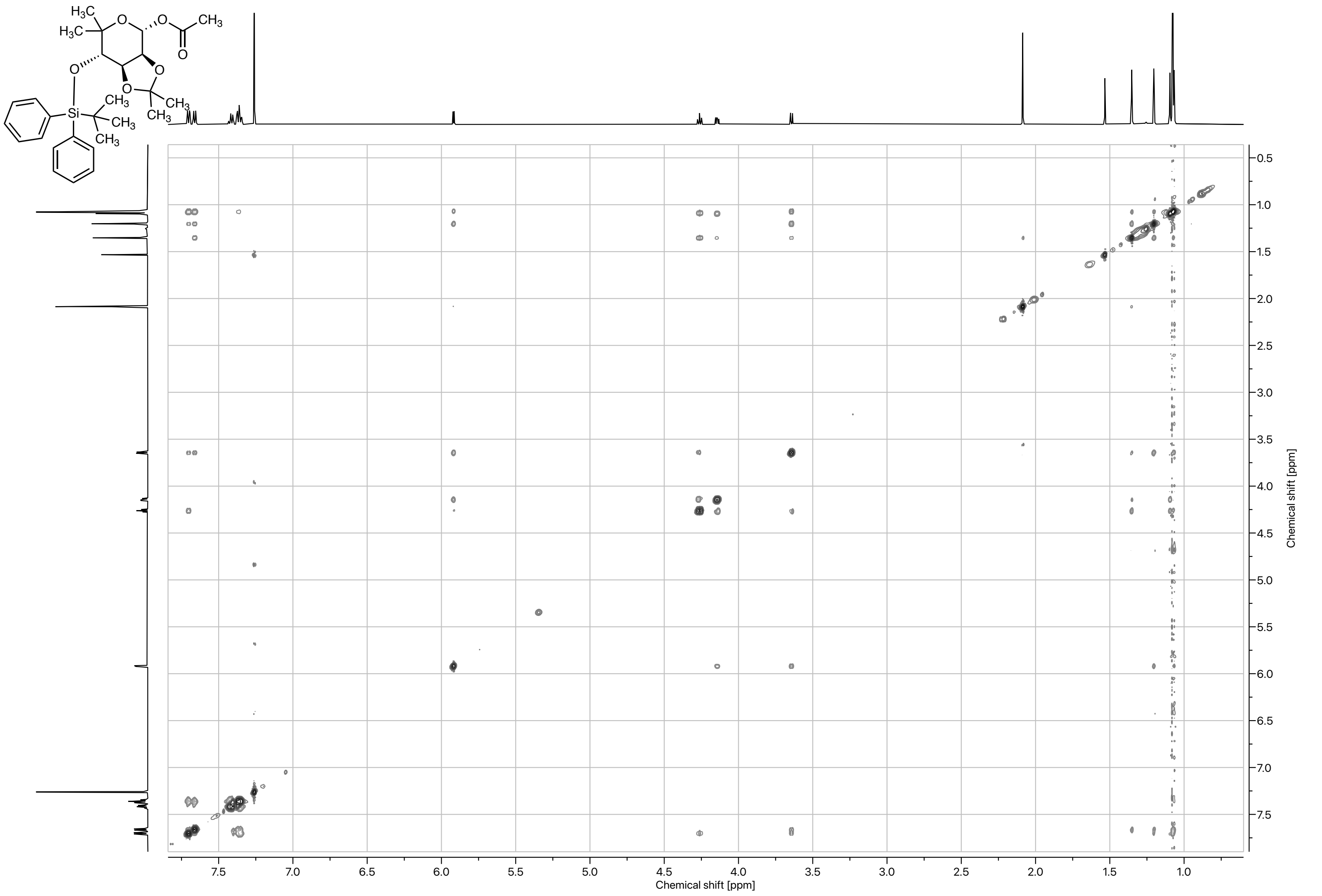

Figure 265: NOESY spectrum of (3aS,4R,7S,7aR)-7-((tert-butyldiphenylsilyl)oxy)-2,2,6,6-tetramethyltetrahydro-4H-[1,3]dioxolo[4,5-c]pyran-4-yl acetate (13-α) in $CDCl_3$

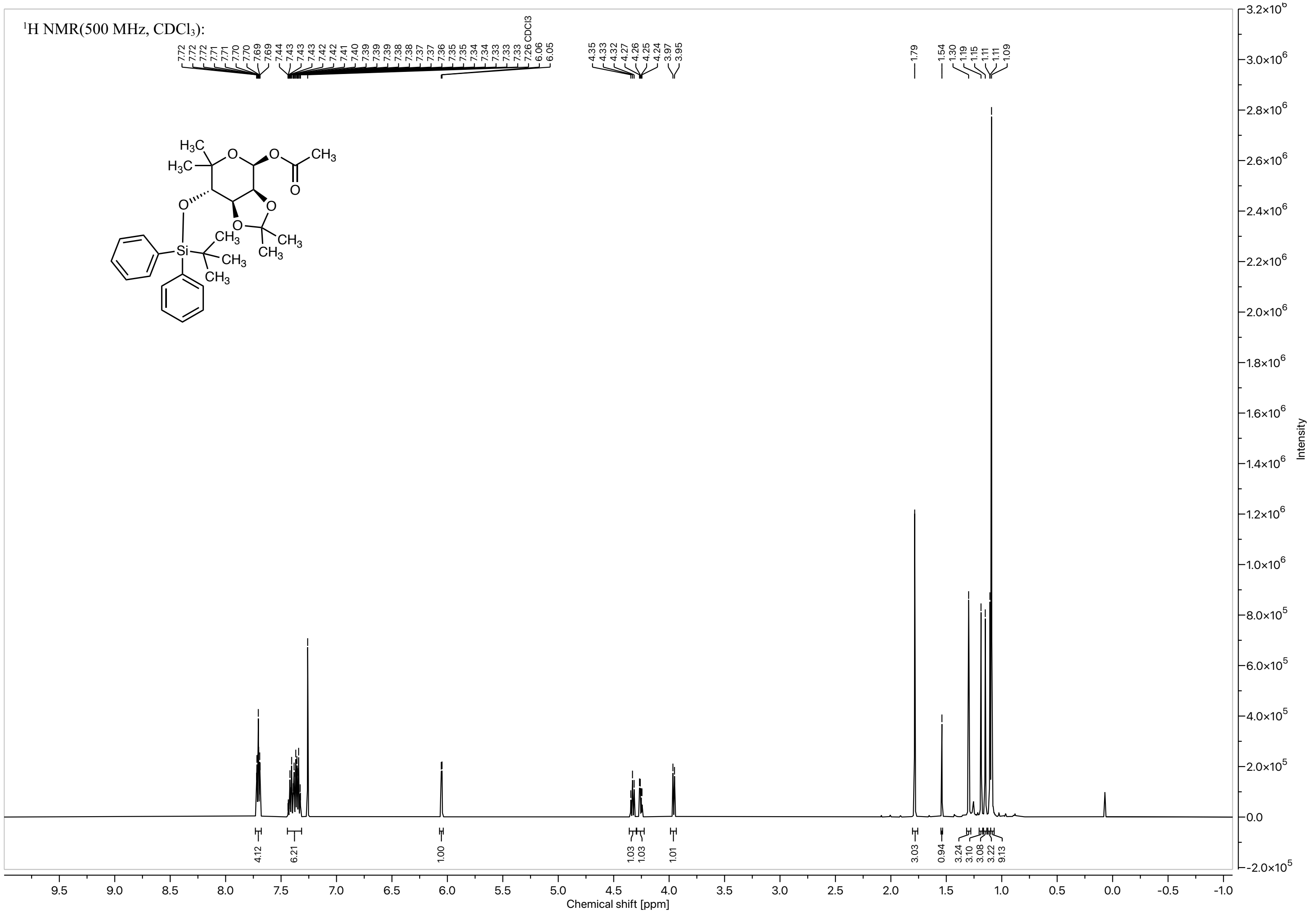

Figure 266: ^{1}H NMR spectrum of (3aS,4S,7S,7aR)-7-((tert-butyldiphenylsilyl)oxy)-2,2,6,6-tetramethyltetrahydro-4H-[1,3]dioxolo[4,5-c]pyran-4-yl acetate in (13-β) $CDCl_3$

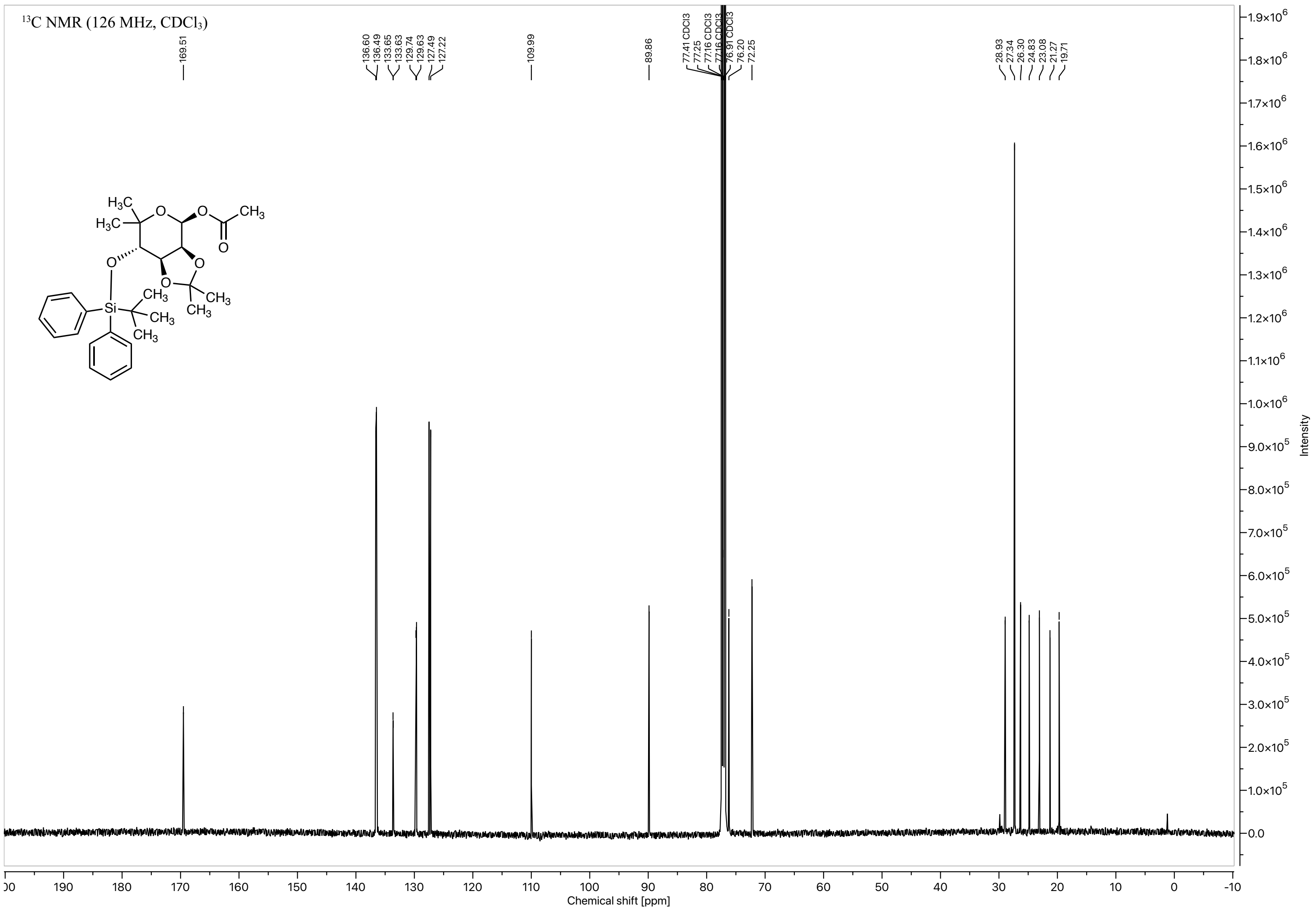

Figure 267: ^{13}C spectrum of (3aS,4S,7S,7aR)-7-((tert-butyldiphenylsilyl)oxy)-2,2,6,6-tetramethyltetrahydro-4H-[1,3]dioxolo[4,5-c]pyran-4-yl acetate (13-β) in $CDCl_3$

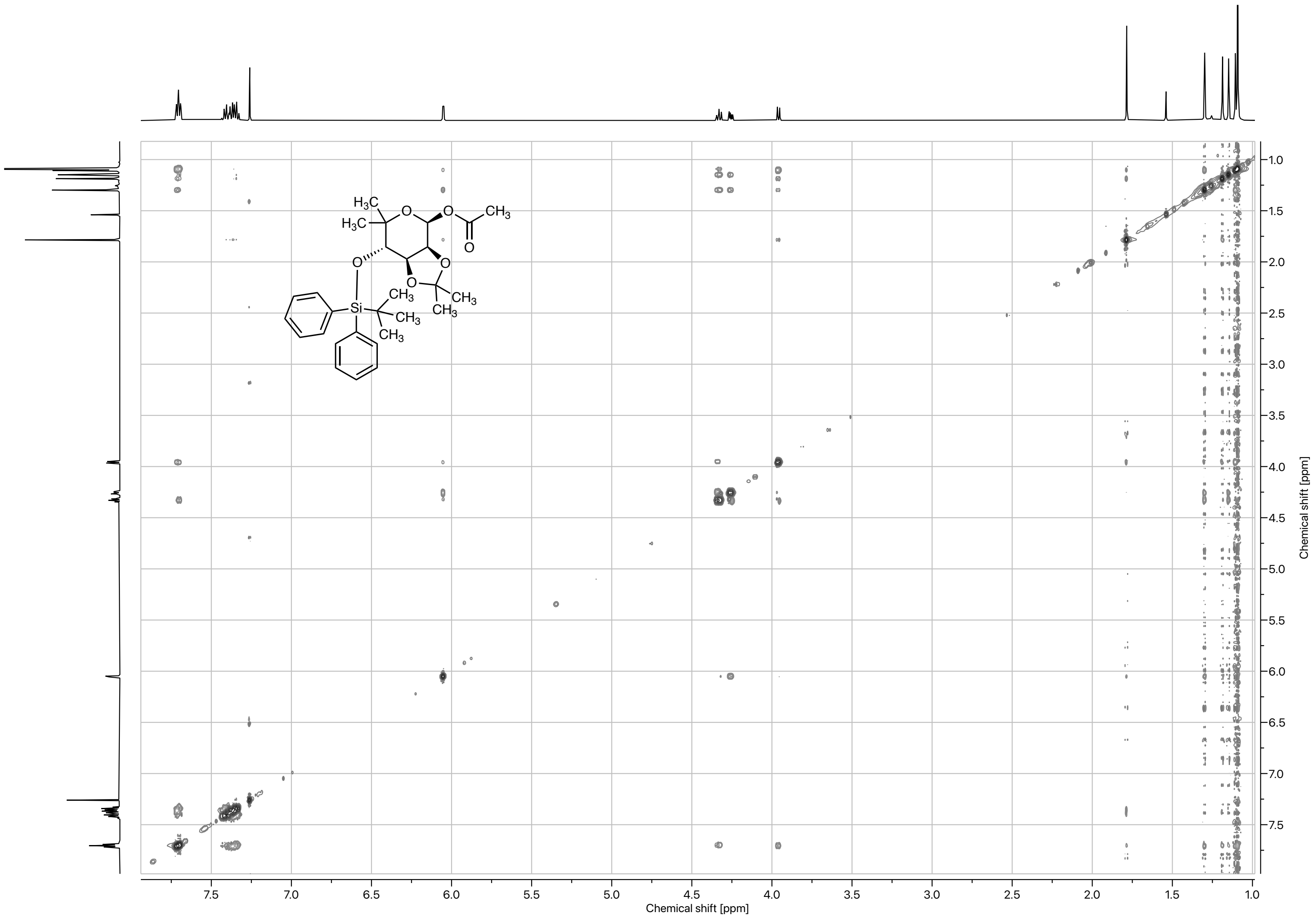

Figure 268: NOESY spectrum of (3aS,4S,7S,7aR)-7-((tert-butyldiphenylsilyl)oxy)-2,2,6,6-tetramethyltetrahydro-4H-[1,3]dioxolo[4,5-c]pyran-4-yl acetate (13-β) in $CDCl_3$

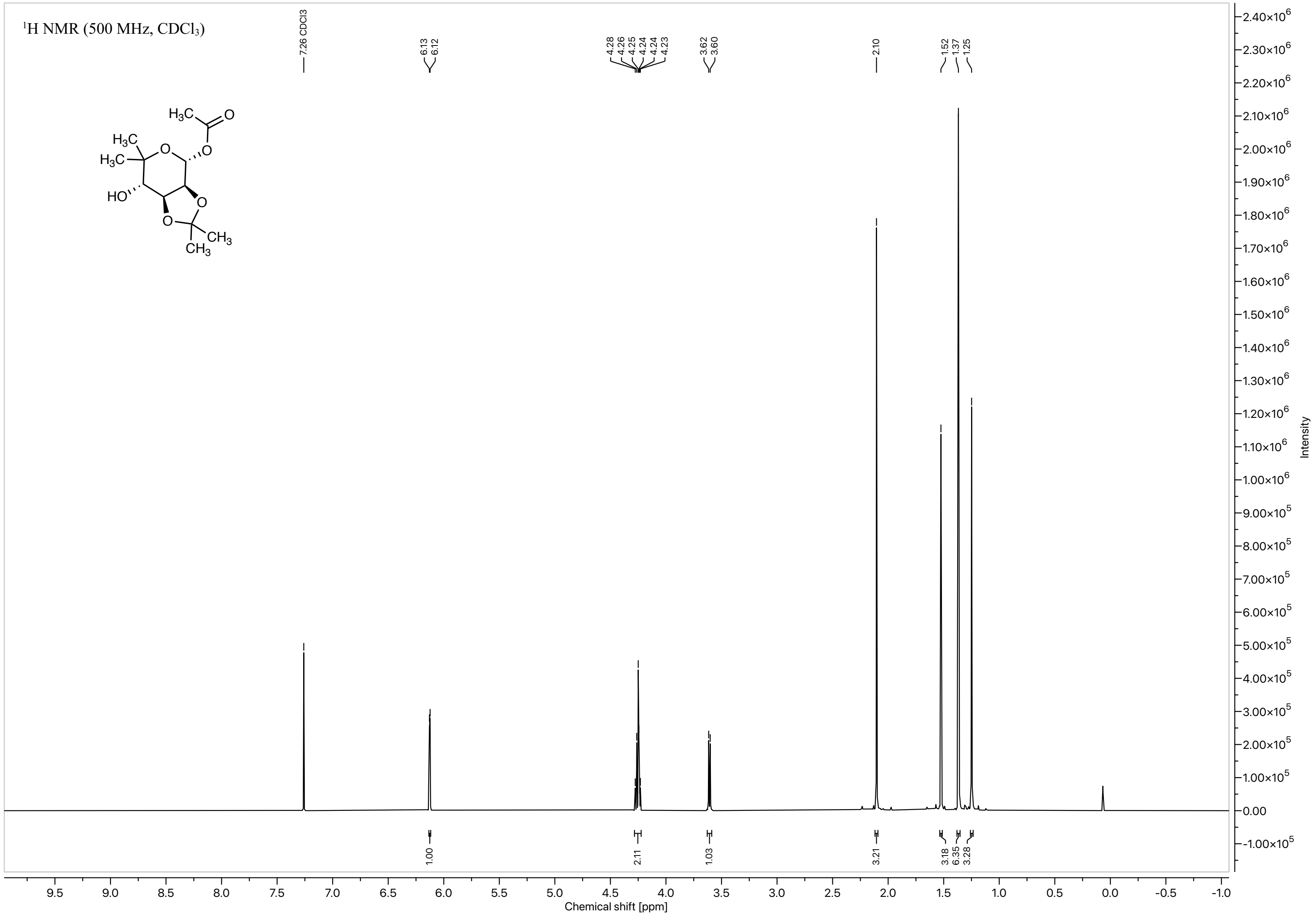

Figure 269: ^{1}H NMR spectrum of (3aS,4R,7S,7aS)-7-hydroxy-2,2,6,6-tetramethyltetrahydro-4H-[1,3]dioxolo[4,5-c]pyran-4-yl acetate (14) in $CDCl_3$

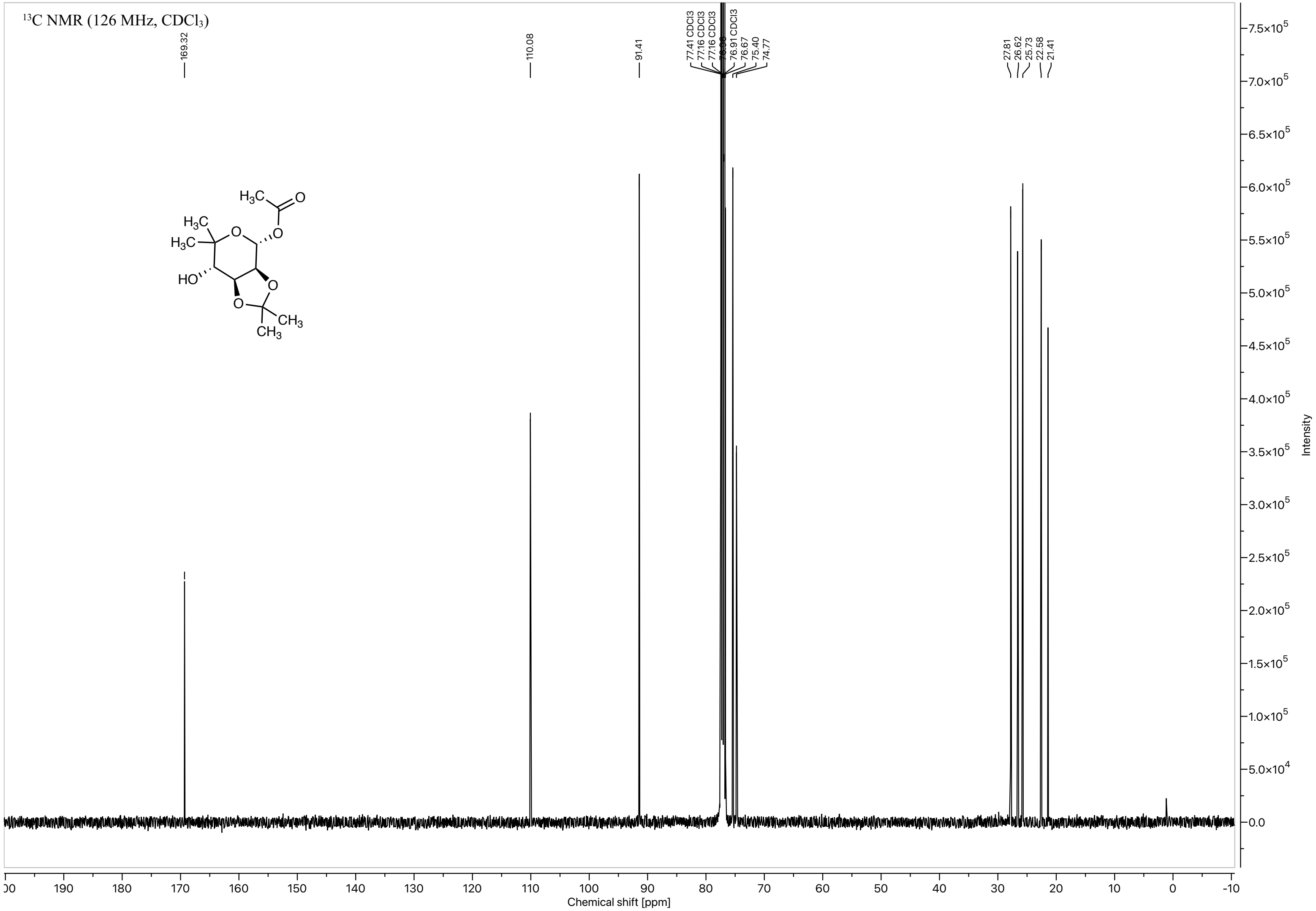

Figure 270: ^{13}C NMR spectrum of (3aS,4R,7S,7aS)-7-hydroxy-2,2,6,6-tetramethyltetrahydro-4H-[1,3]dioxolo[4,5-c]pyran-4-yl acetate (14) in $CDCl_3$

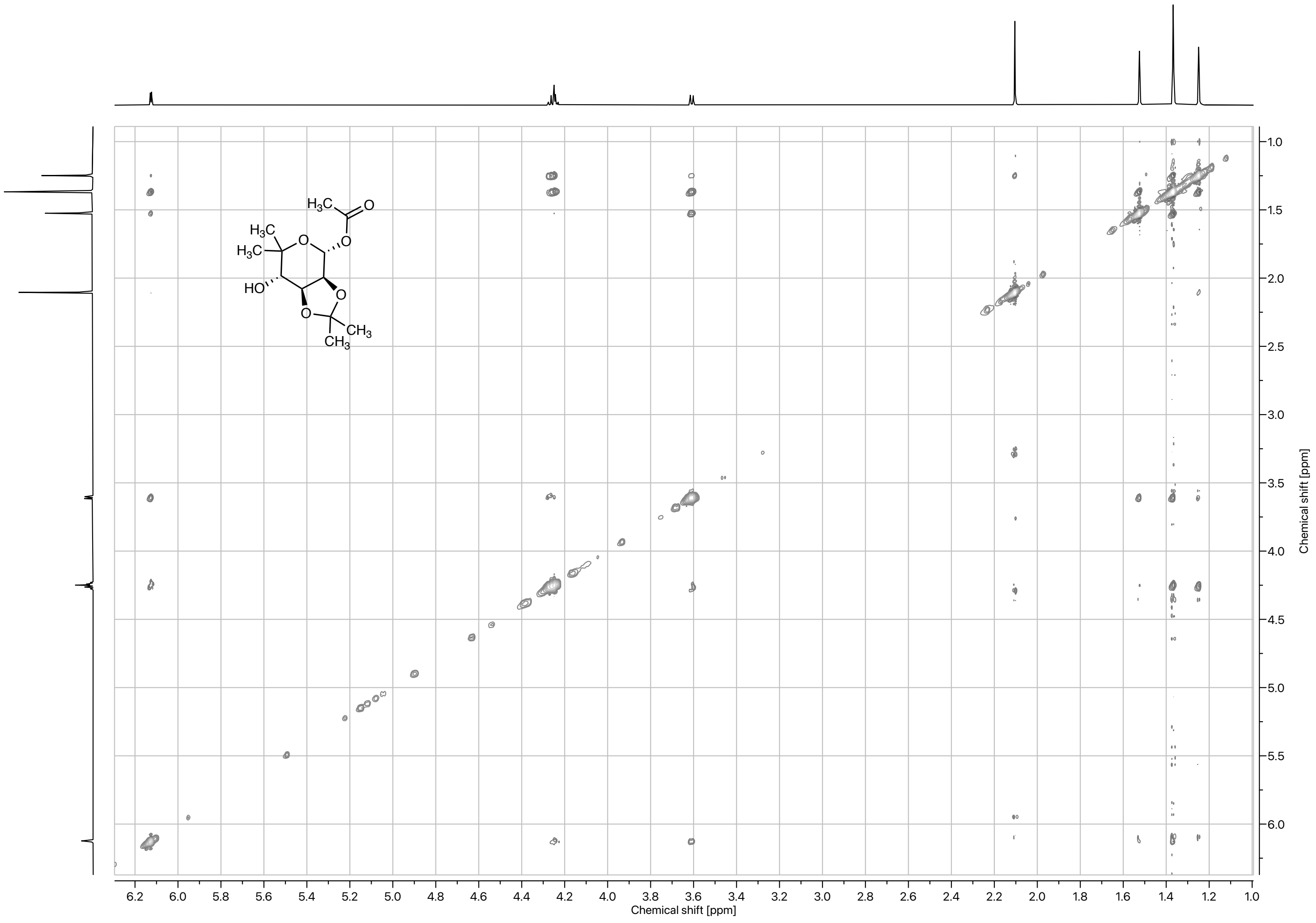

Figure 271: NOESY spectrum of (3aS,4R,7S,7aS)-7-hydroxy-2,2,6,6-tetramethyltetrahydro-4H-[1,3]dioxolo[4,5-c]pyran-4-yl acetate (14) in $CDCl_3$

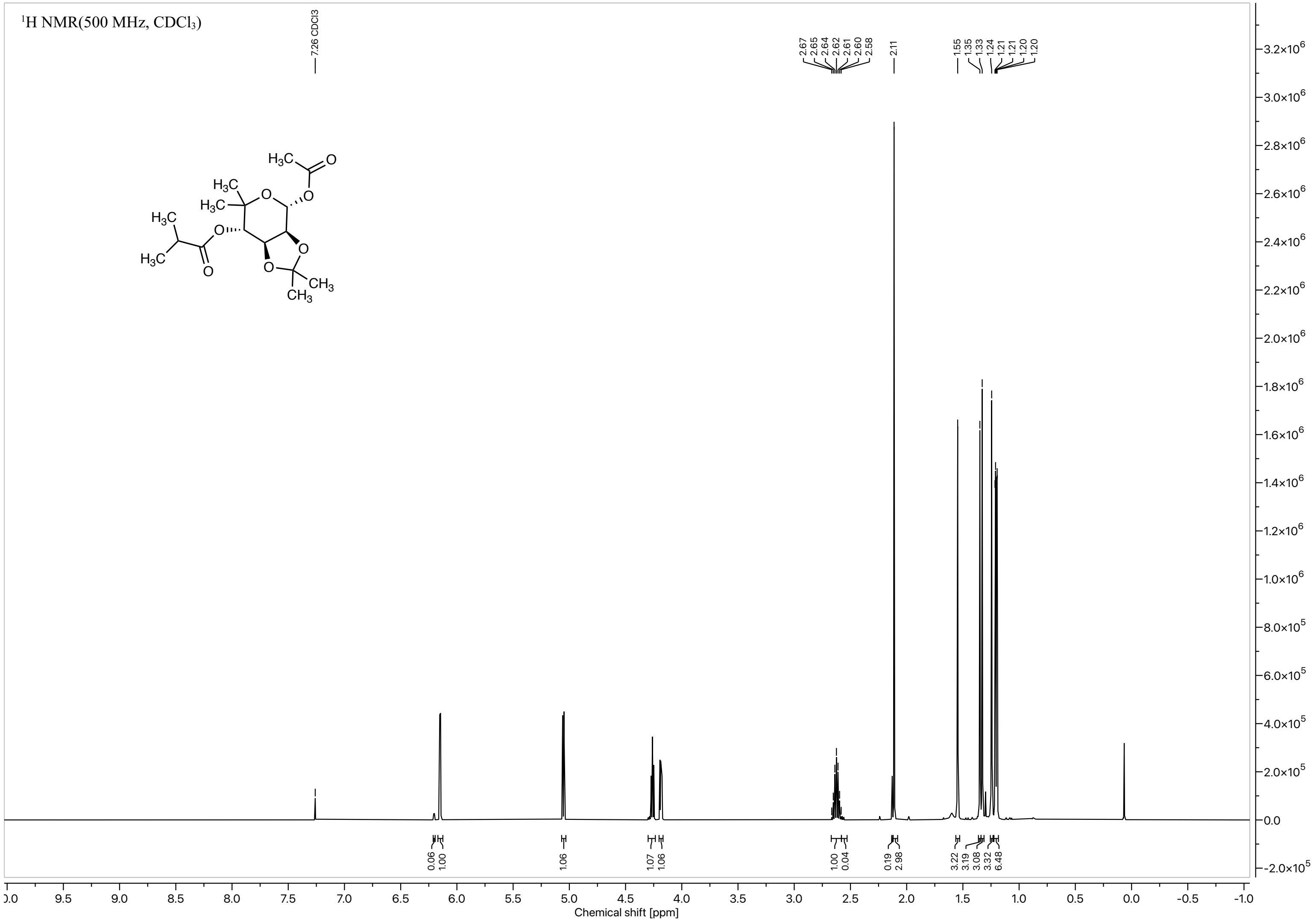

Figure 272: ^{1}H NMR spectrum of (3aS,4S,7S,7aS)-4-acetoxy-2,2,6,6-tetramethyltetrahydro-4H-[1,3]dioxolo[4,5-c]pyran-7-yl isobutyrate (15a) in $CDCl_3$

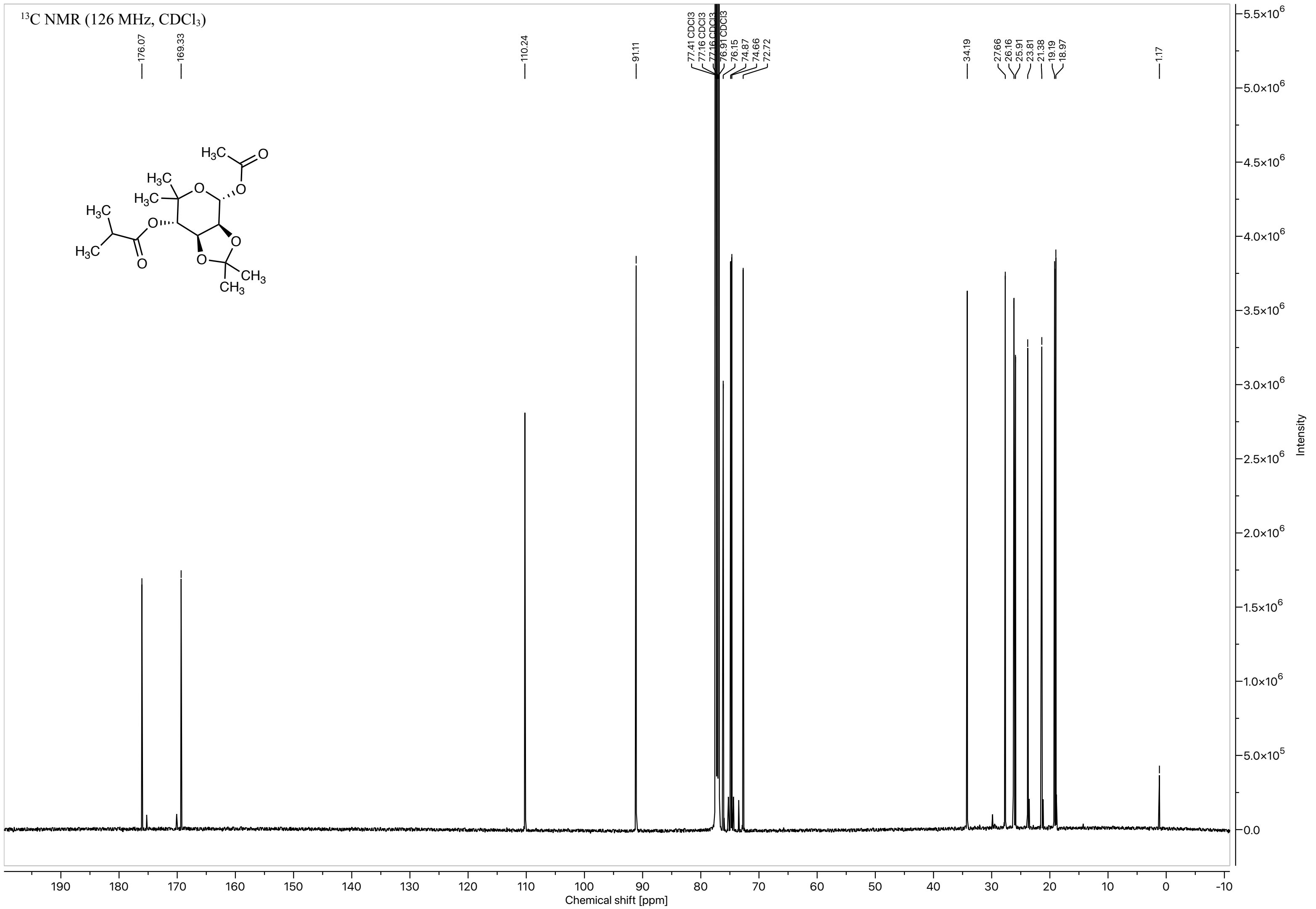

Figure 273: ^{13}C NMR spectrum of (3aS,4S,7S,7aS)-4-acetoxy-2,2,6,6-tetramethyltetrahydro-4H-[1,3]dioxolo[4,5-c]pyran-7-yl isobutyrate (15a) in $CDCl_3$

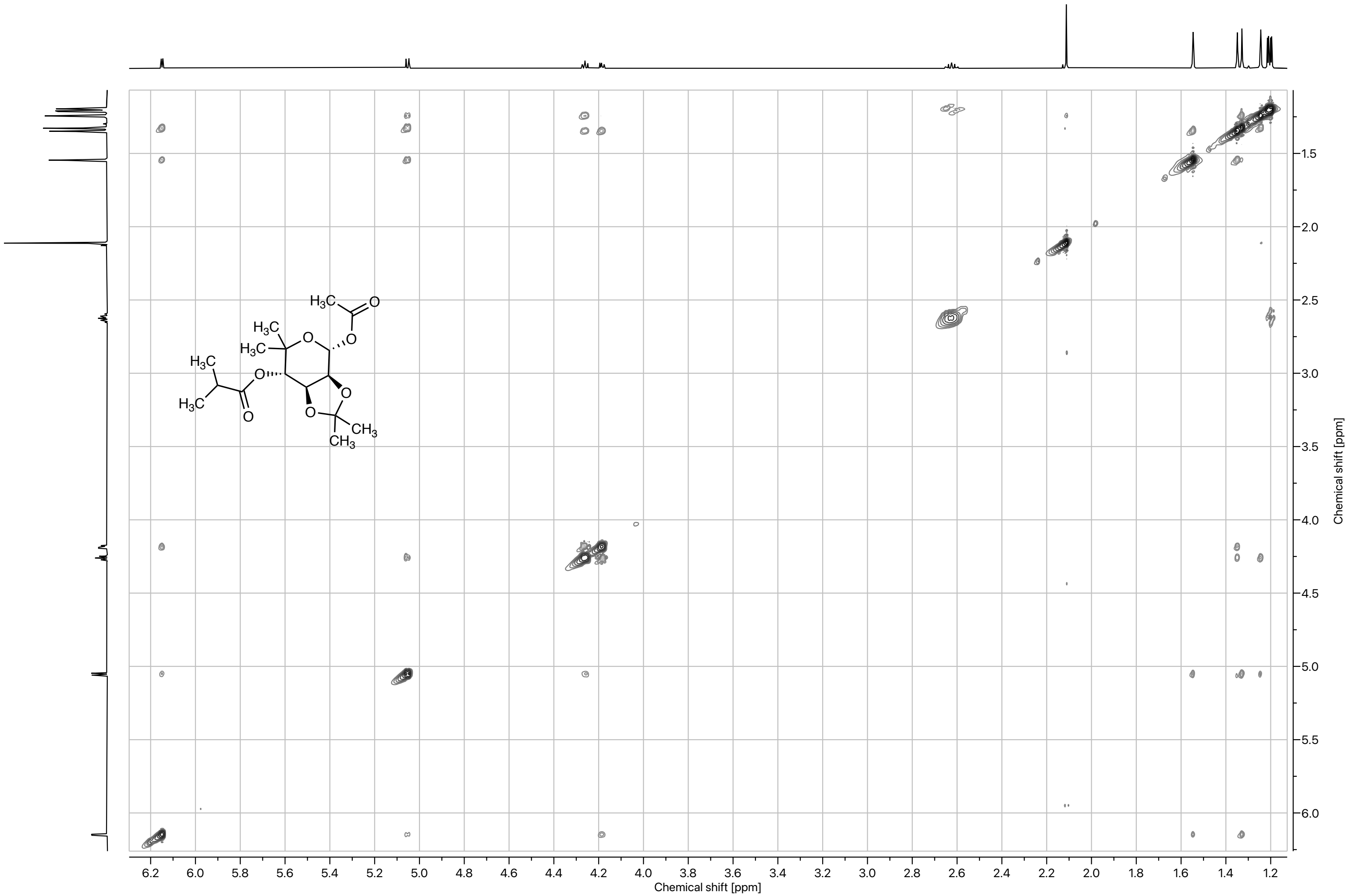

Figure 274: NOESY spectrum of (3aS,4S,7S,7aS)-4-acetoxy-2,2,6,6-tetramethyltetrahydro-4H-[1,3]dioxolo[4,5-c]pyran-7-yl isobutyrate (15a) in CDCl$_3$

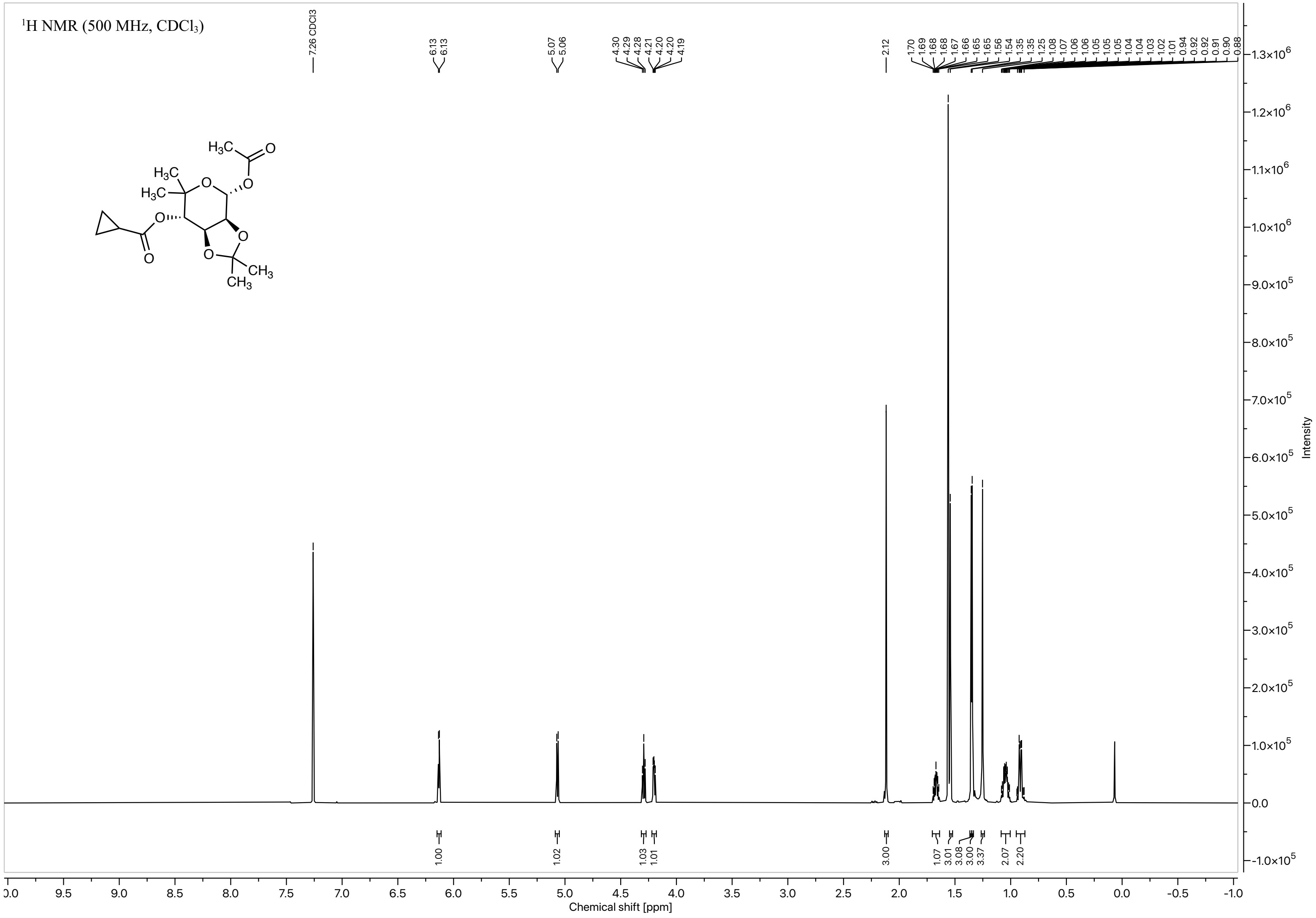

Figure 275: ^{1}H NMR spectrum of (3aS,4R,7S,7aS)-4-Acetoxy-2,2,6,6-tetramethyltetrahydro-4H-[1,3]dioxolo[4,5-c]pyran-7-yl cyclopropanecarboxylate (15b) in $CDCl_3$

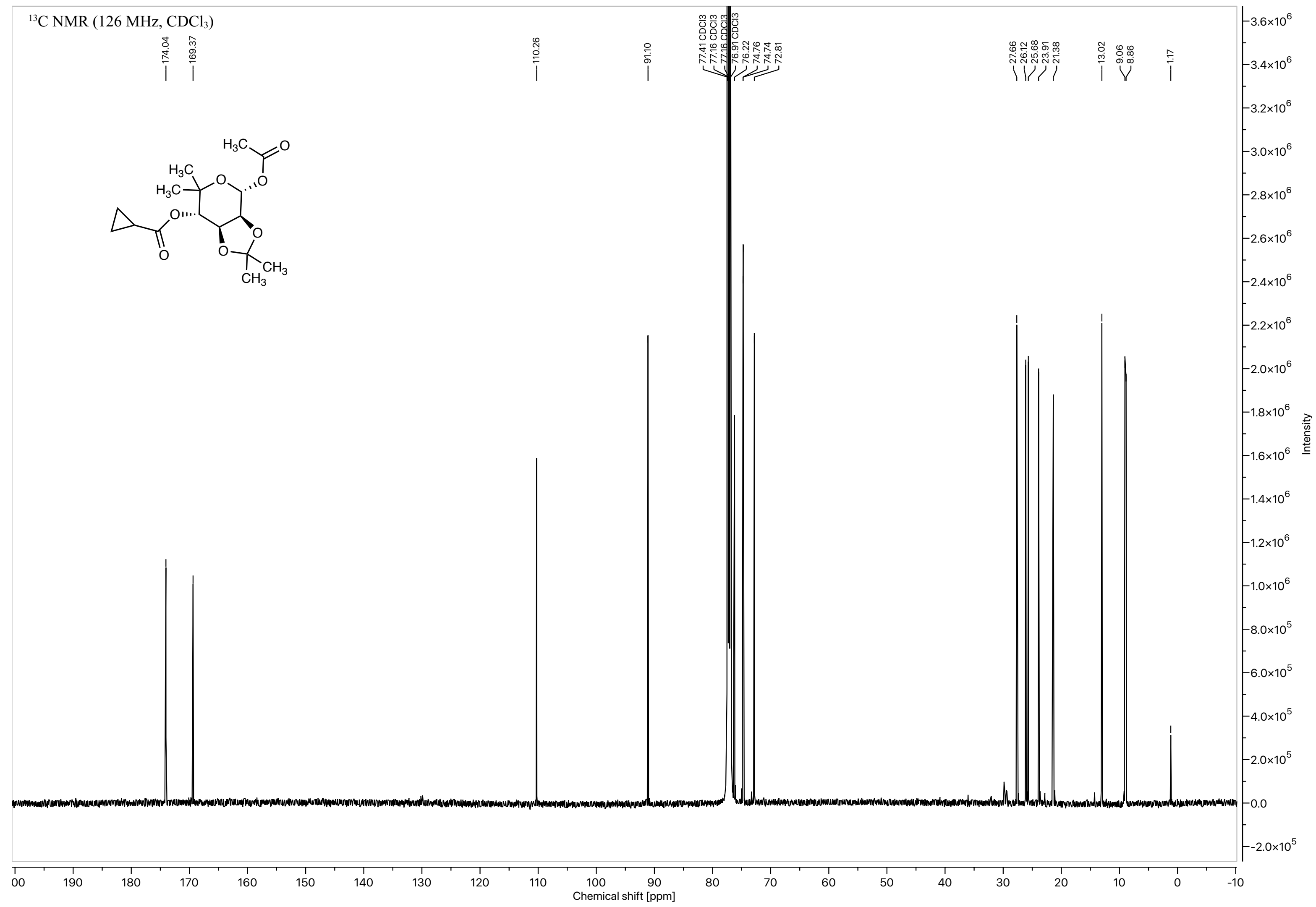

Figure 276: ^{13}C NMR spectrum of (3aS,4R,7S,7aS)-4-Acetoxy-2,2,6,6-tetramethyltetrahydro-4H-[1,3]dioxolo[4,5-c]pyran-7-yl cyclopropanecarboxylate (15b) in $CDCl_3$

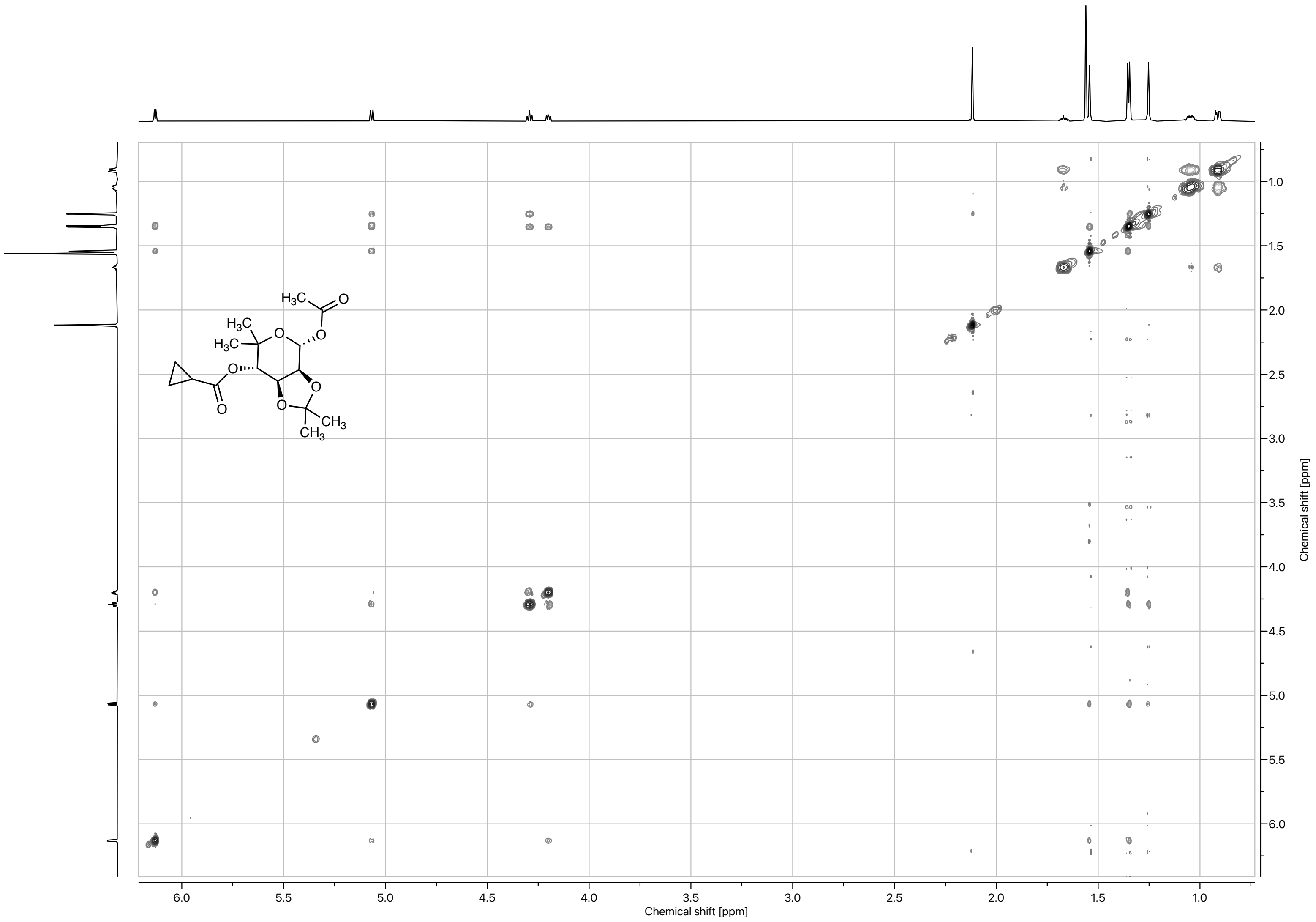

Figure 277: NOESY spectrum of (3aS,4R,7S,7aS)-4-Acetoxy-2,2,6,6-tetramethyltetrahydro-4H-[1,3]dioxolo[4,5-c]pyran-7-yl cyclopropanecarboxylate (15b) in $CDCl_3$

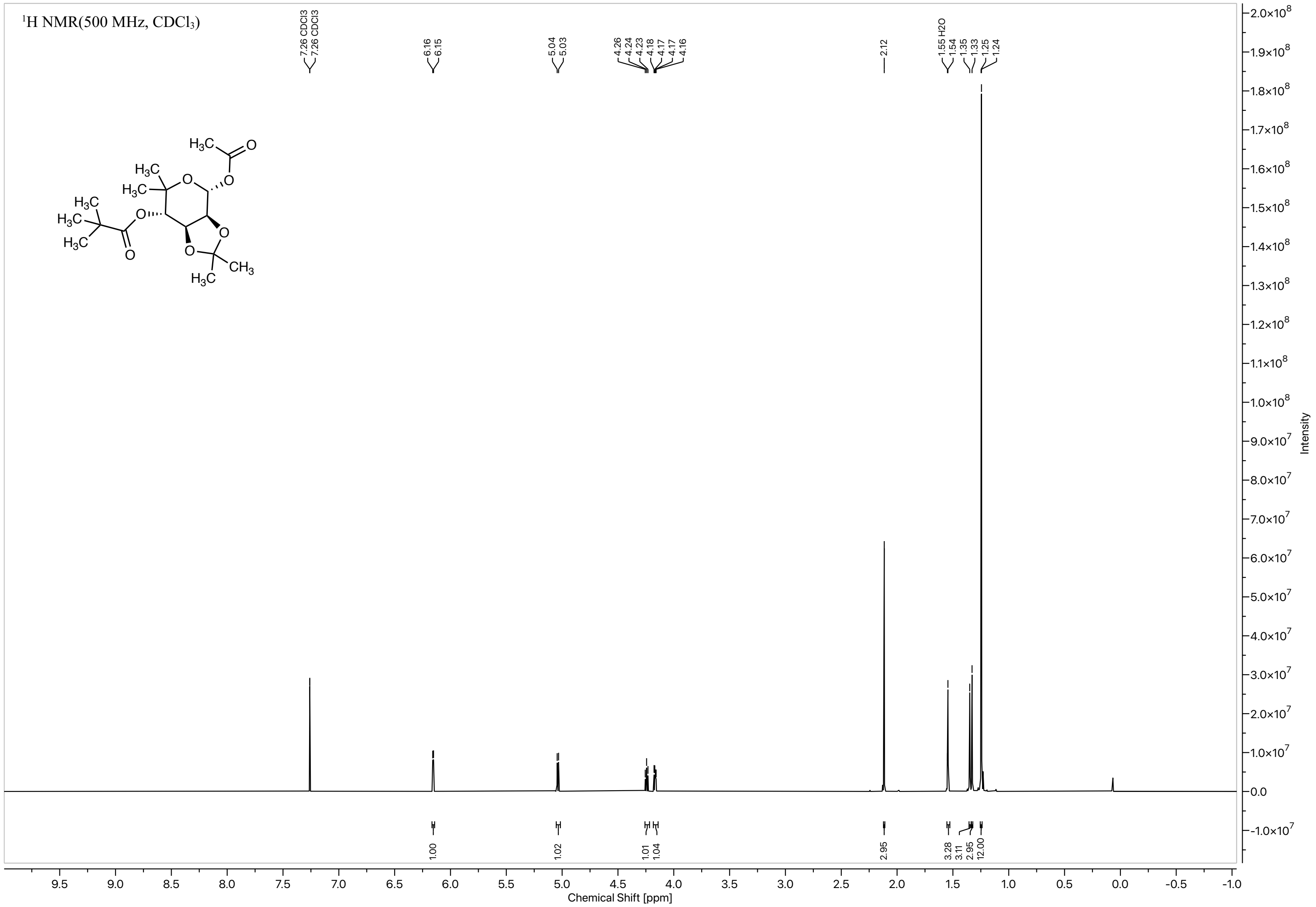

Figure 278: ¹H NMR spectrum of (3aS,4R,7S,7aS)-4-acetoxy-2,2,6,6-tetramethyltetrahydro-4H-[1,3]dioxolo[4,5-c]pyran-7-yl pivalate (15c) in $CDCl_3$

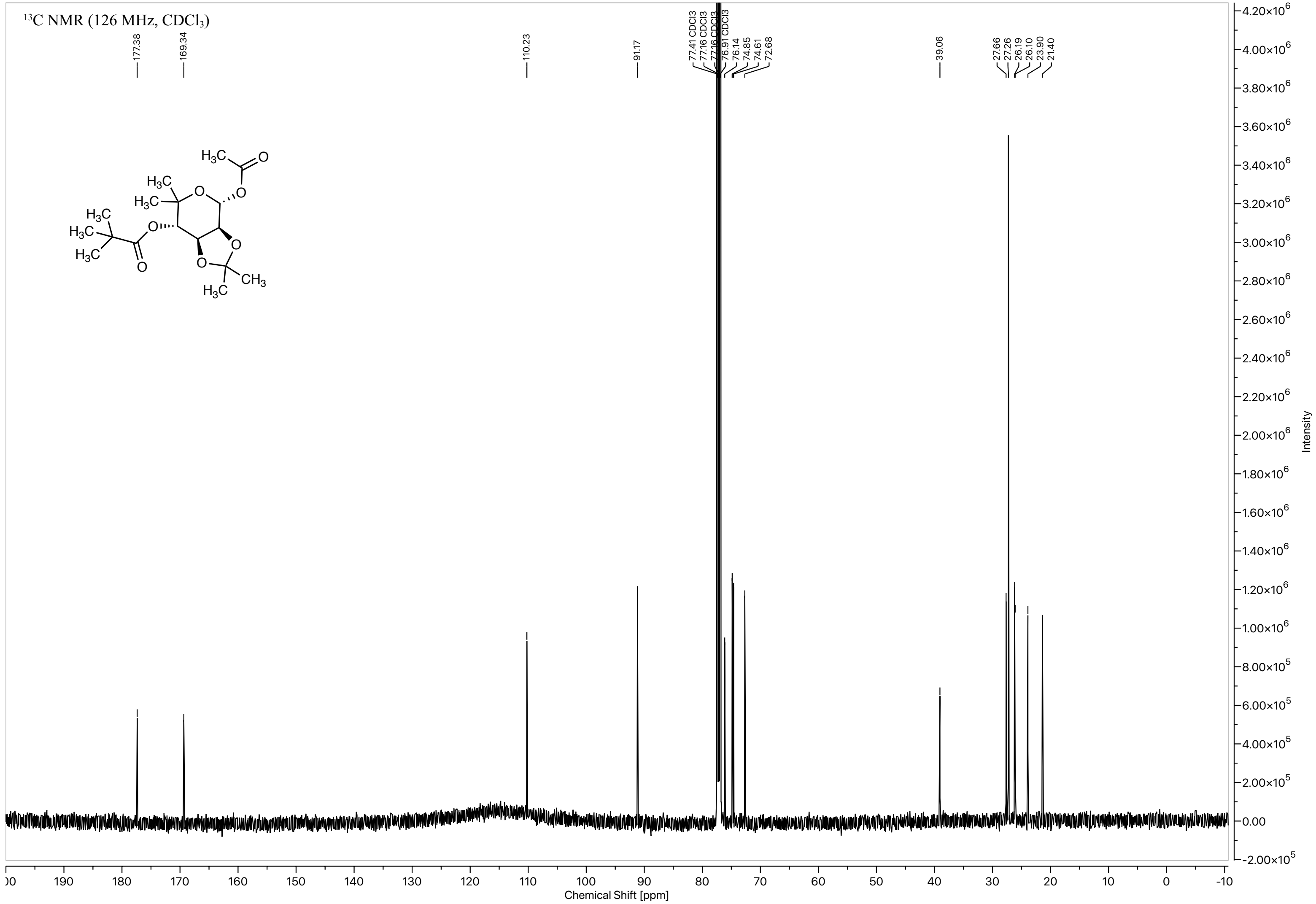

Figure 279: ^{13}C NMR spectrum of (3aS,4R,7S,7aS)-4-acetoxy-2,2,6,6-tetramethyltetrahydro-4H-[1,3]dioxolo[4,5-c]pyran-7-yl pivalate (15c) in CDCl$_3$

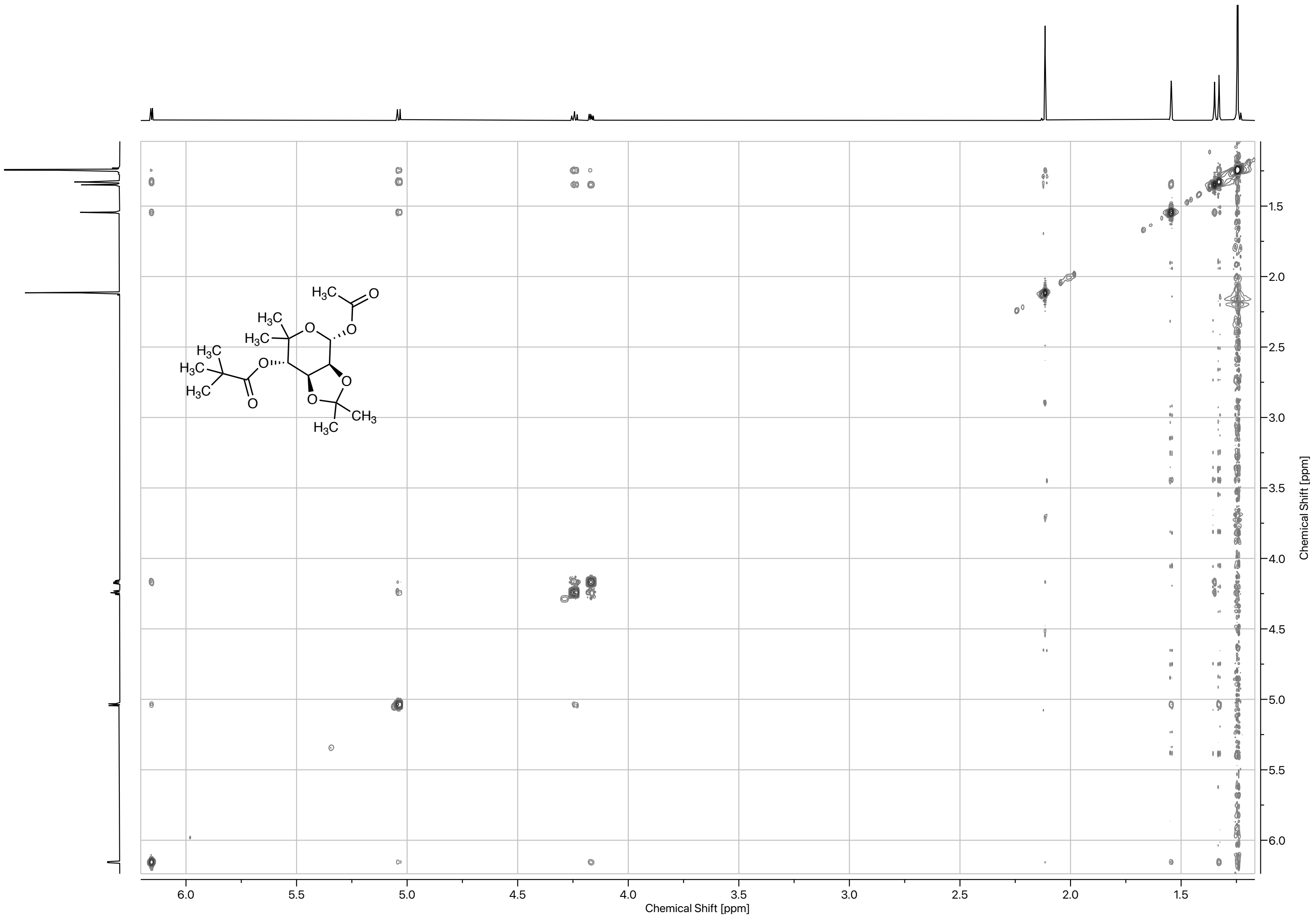

Figure 280: NOESY spectrum of (3aS,4R,7S,7aS)-4-acetoxy-2,2,6,6-tetramethyltetrahydro-4H-[1,3]dioxolo[4,5-c]pyran-7-yl pivalate (15c) in $CDCl_3$

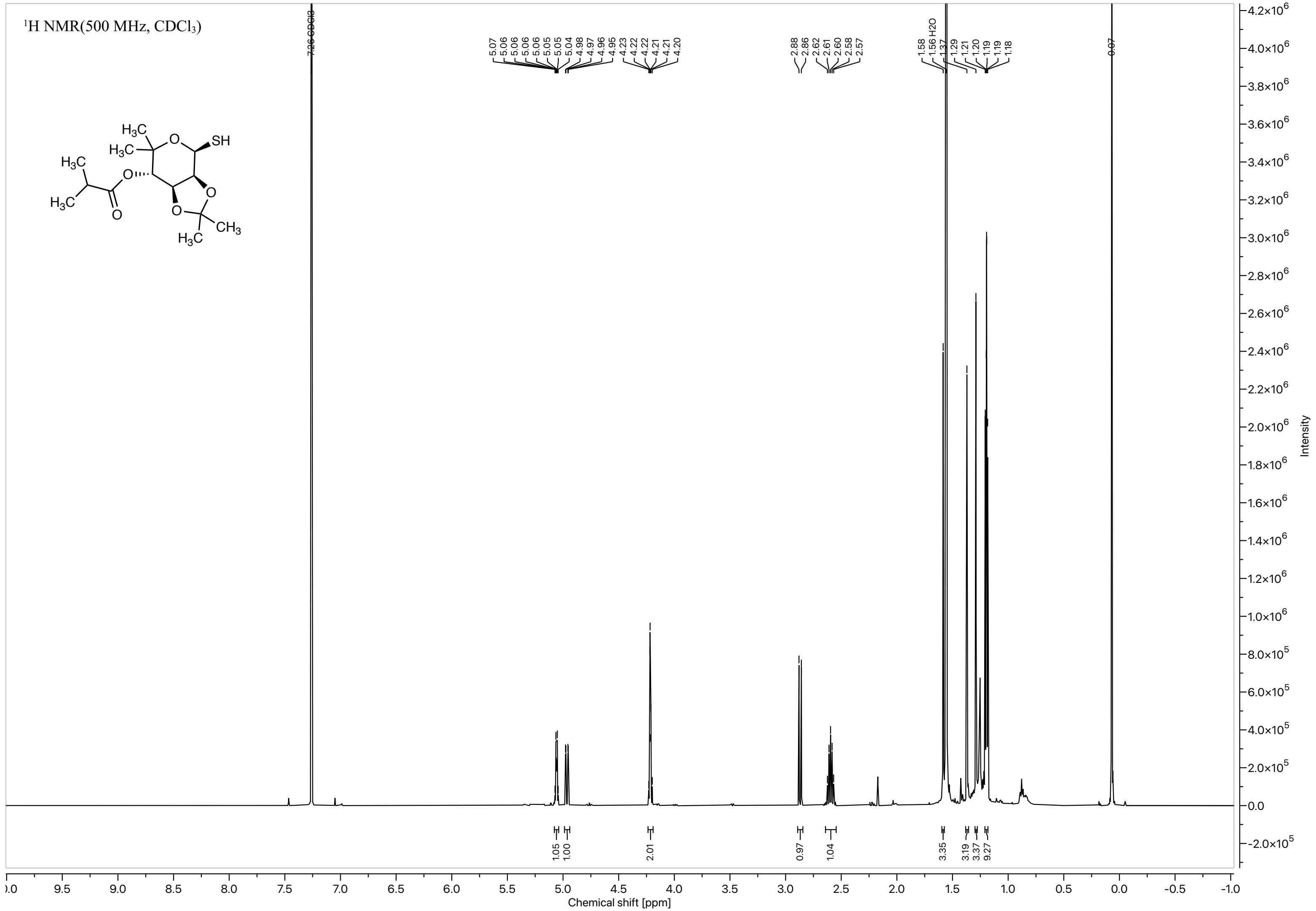

Figure 281: ^{1}H NMR spectrum of (3aS,4S,7S,7aS)-4-mercapto-2,2,6,6-tetramethyltetrahydro-4H-[1,3]dioxolo[4,5-c]pyran-7-yl isobutyrate (17a) in $CDCl_3$

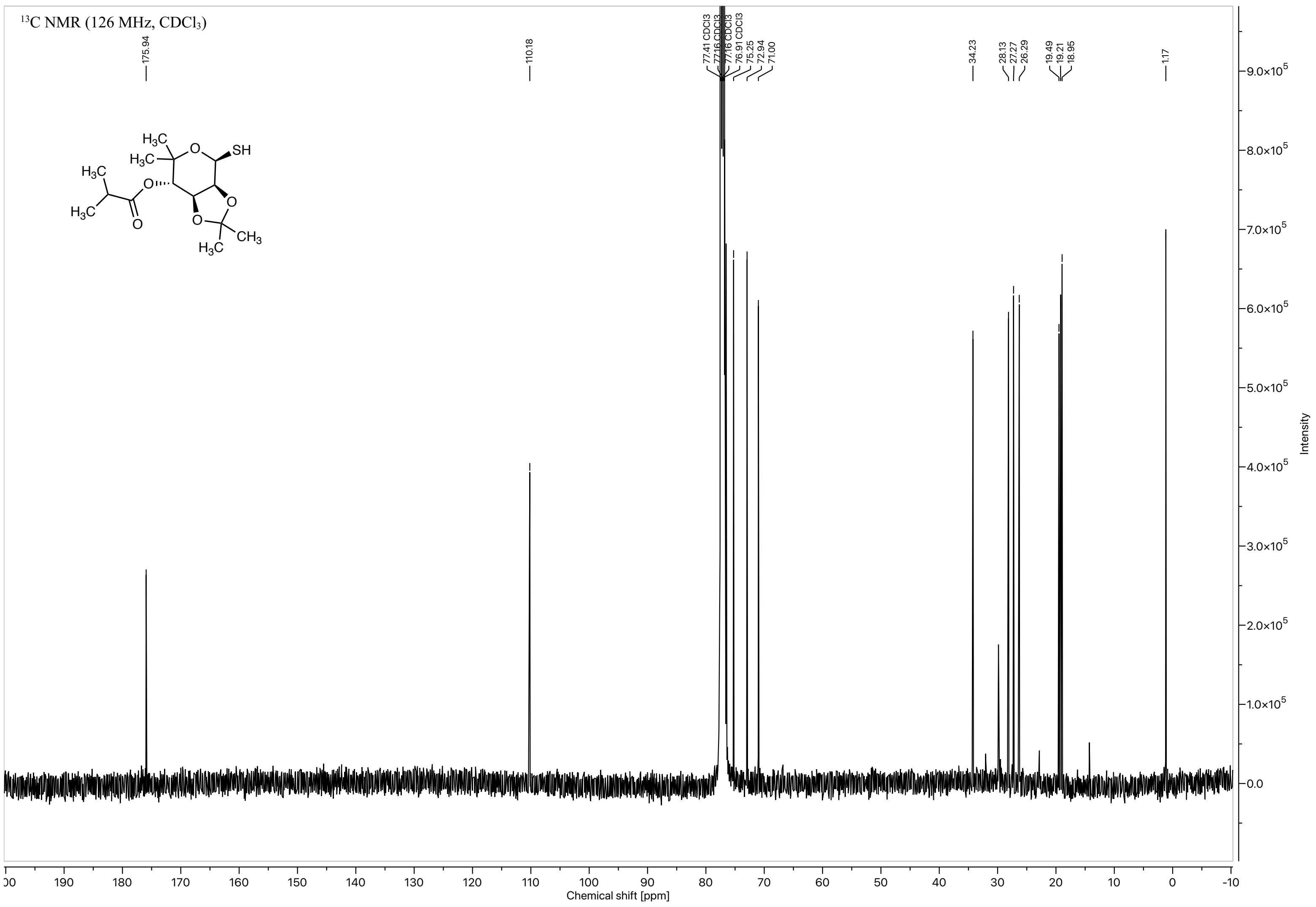

Figure 282: ^{13}C NMR spectrum of (3aS,4S,7S,7aS)-4-mercapto-2,2,6,6-tetramethyltetrahydro-4H-[1,3]dioxolo[4,5-c]pyran-7-yl isobutyrate (17a) in $CDCl_3$

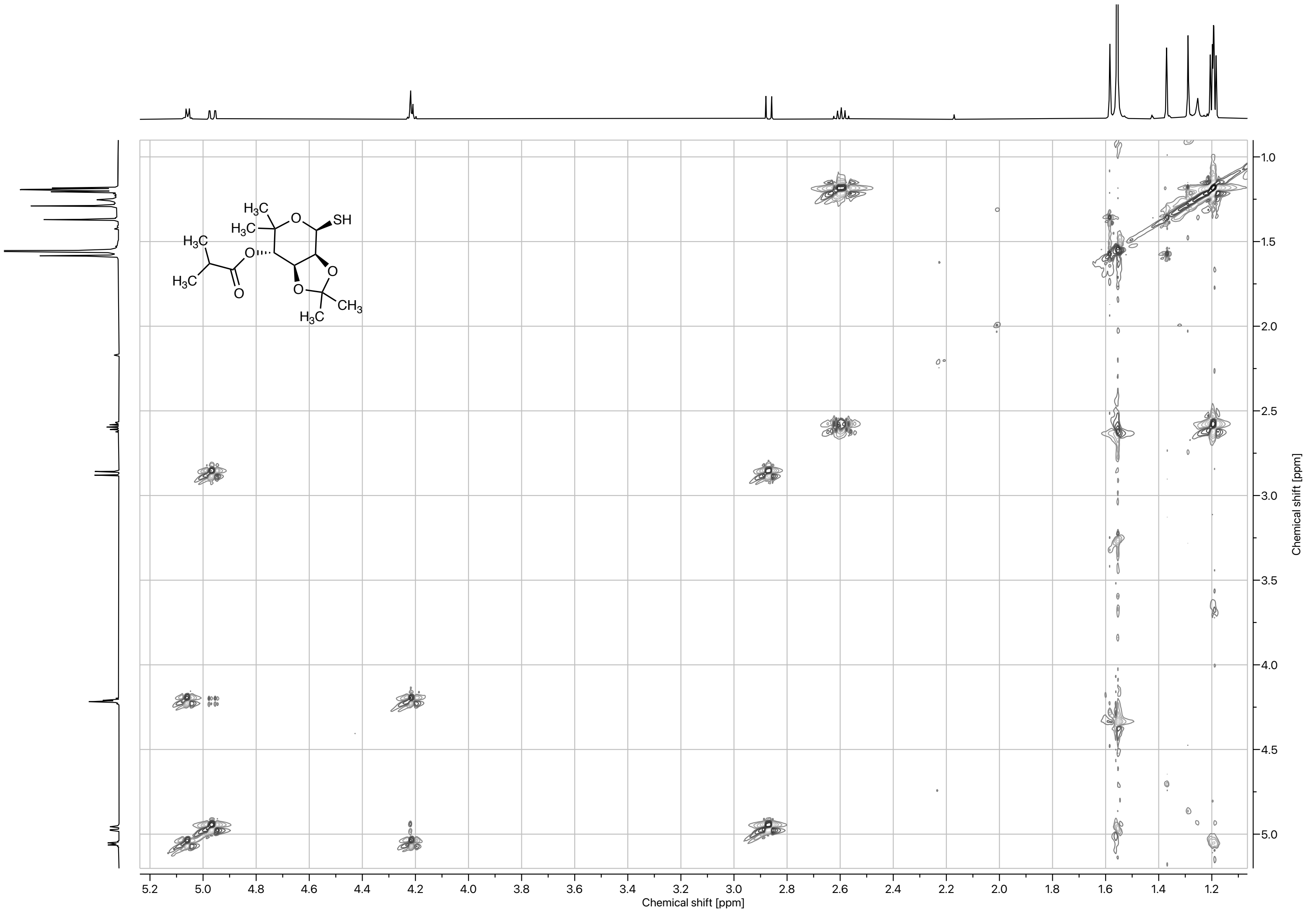

Figure 283: COSY spectrum of (3aS,4S,7S,7aS)-4-mercapto-2,2,6,6-tetramethyltetrahydro-4H-[1,3]dioxolo[4,5-c]pyran-7-yl isobutyrate (17a) in $CDCl_3$

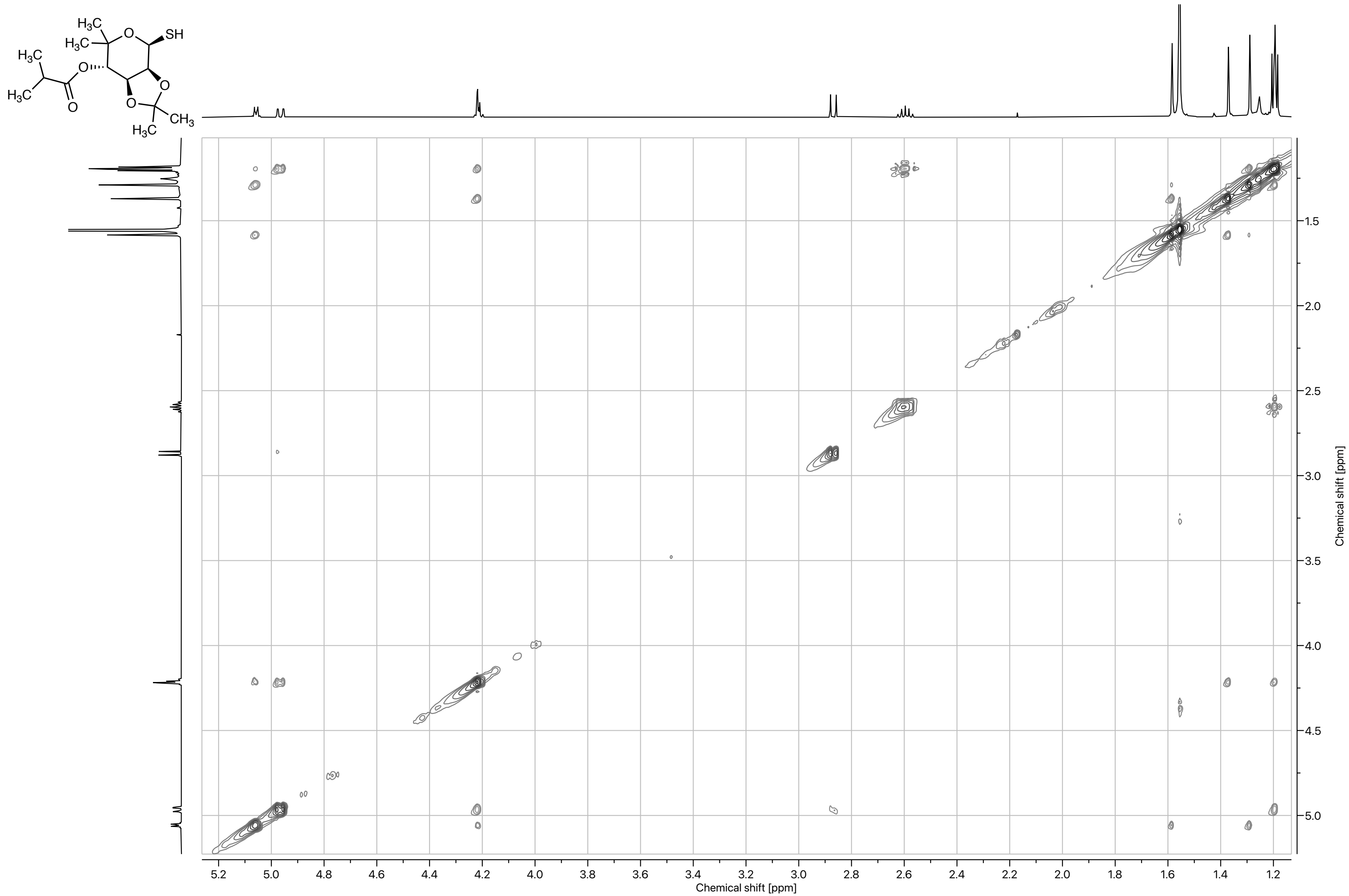

Figure 284: NOESY spectrum of (3aS,4S,7S,7aS)-4-mercapto-2,2,6,6-tetramethyltetrahydro-4H-[1,3]dioxolo[4,5-c]pyran-7-yl isobutyrate (17a) in $CDCl_3$

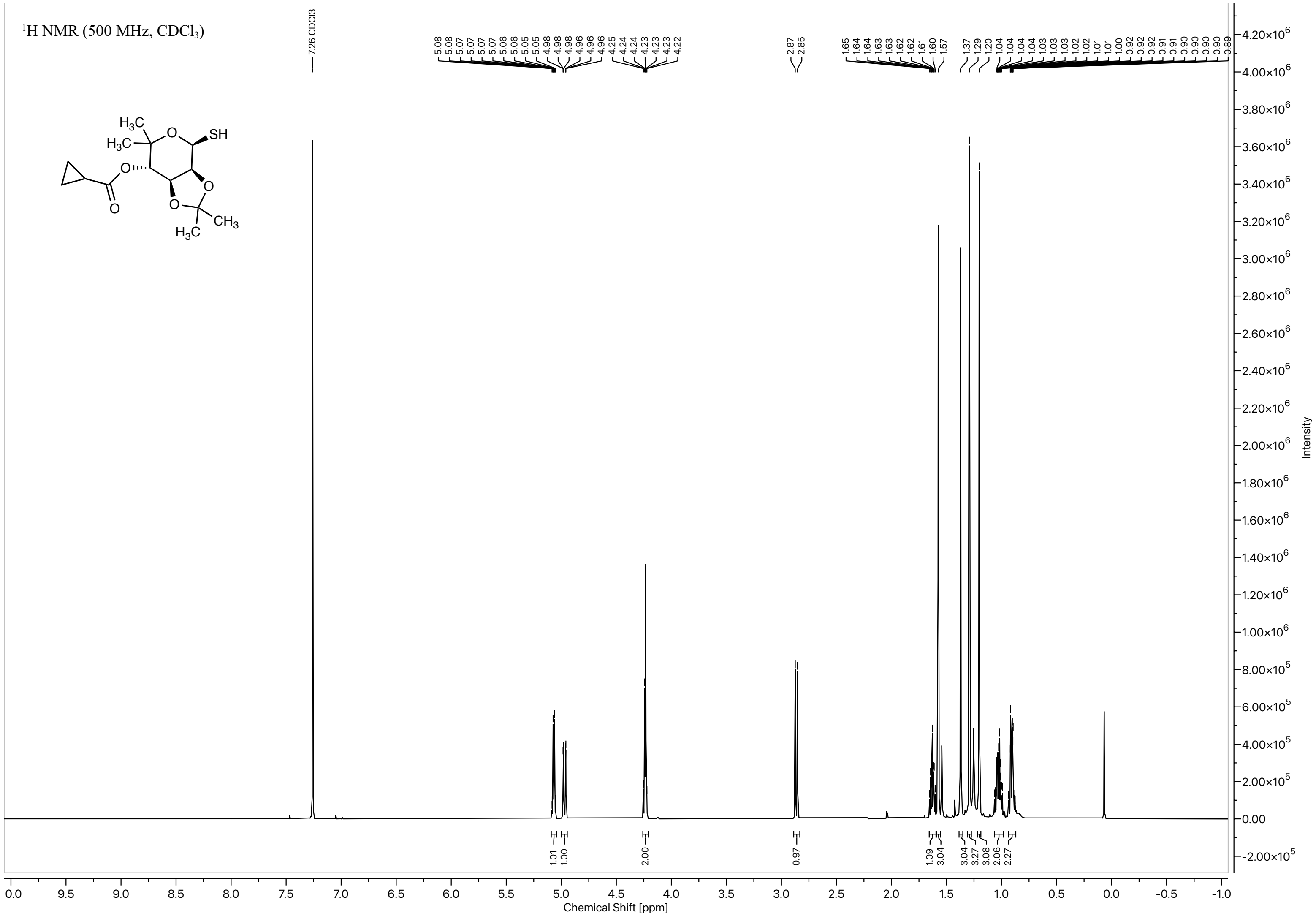

Figure 285: ¹H NMR spectrum of (3aS,4S,7S,7aS)-4-mercapto-2,2,6,6-tetramethyltetrahydro-4H-[1,3]dioxolo[4,5-c]pyran-7-yl cyclopropanecarboxylate (17b) in $CDCl_3$

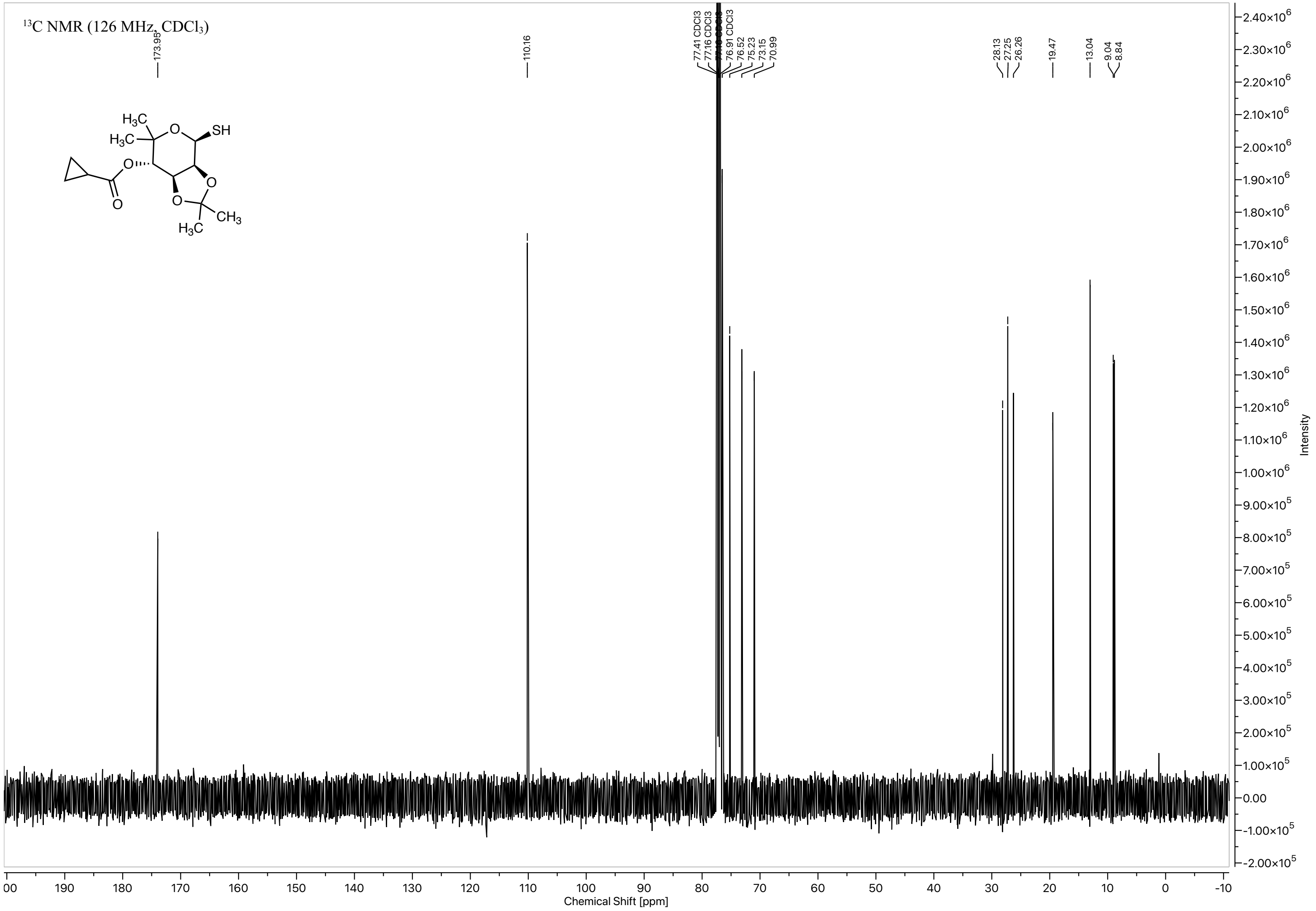

Figure 286: ^{13}C NMR spectrum of (3aS,4S,7S,7aS)-4-mercapto-2,2,6,6-tetramethyltetrahydro-4H-[1,3]dioxolo[4,5-c]pyran-7-yl cyclopropanecarboxylate (17b) in $CDCl_3$

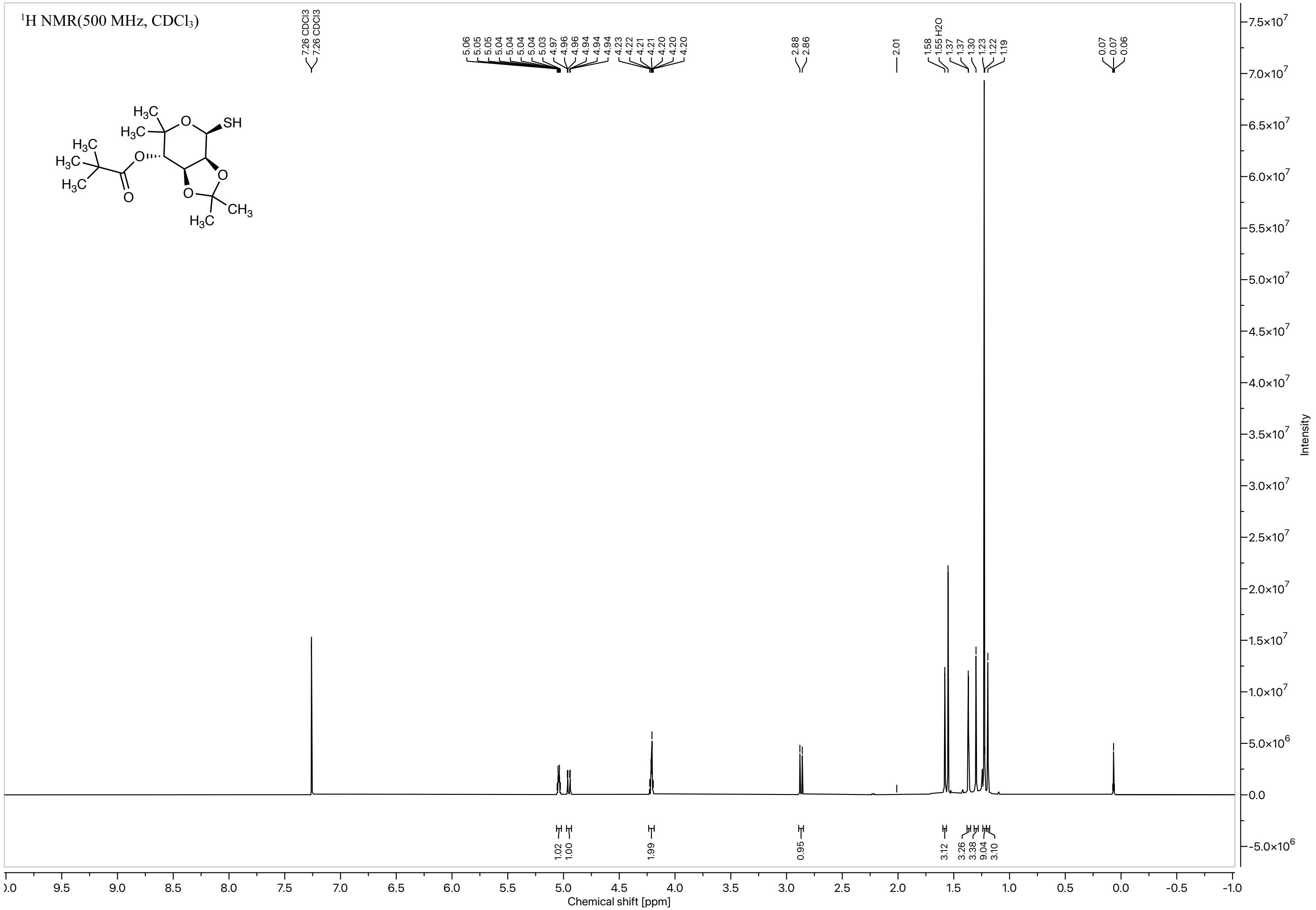

Figure 287: ^{1}H NMR spectrum of (3aS,4S,7S,7aS)-4-mercapto-2,2,6,6-tetramethyltetrahydro-4H-[1,3]dioxolo[4,5-c]pyran-7-yl pivalate (17c) in $CDCl_3$

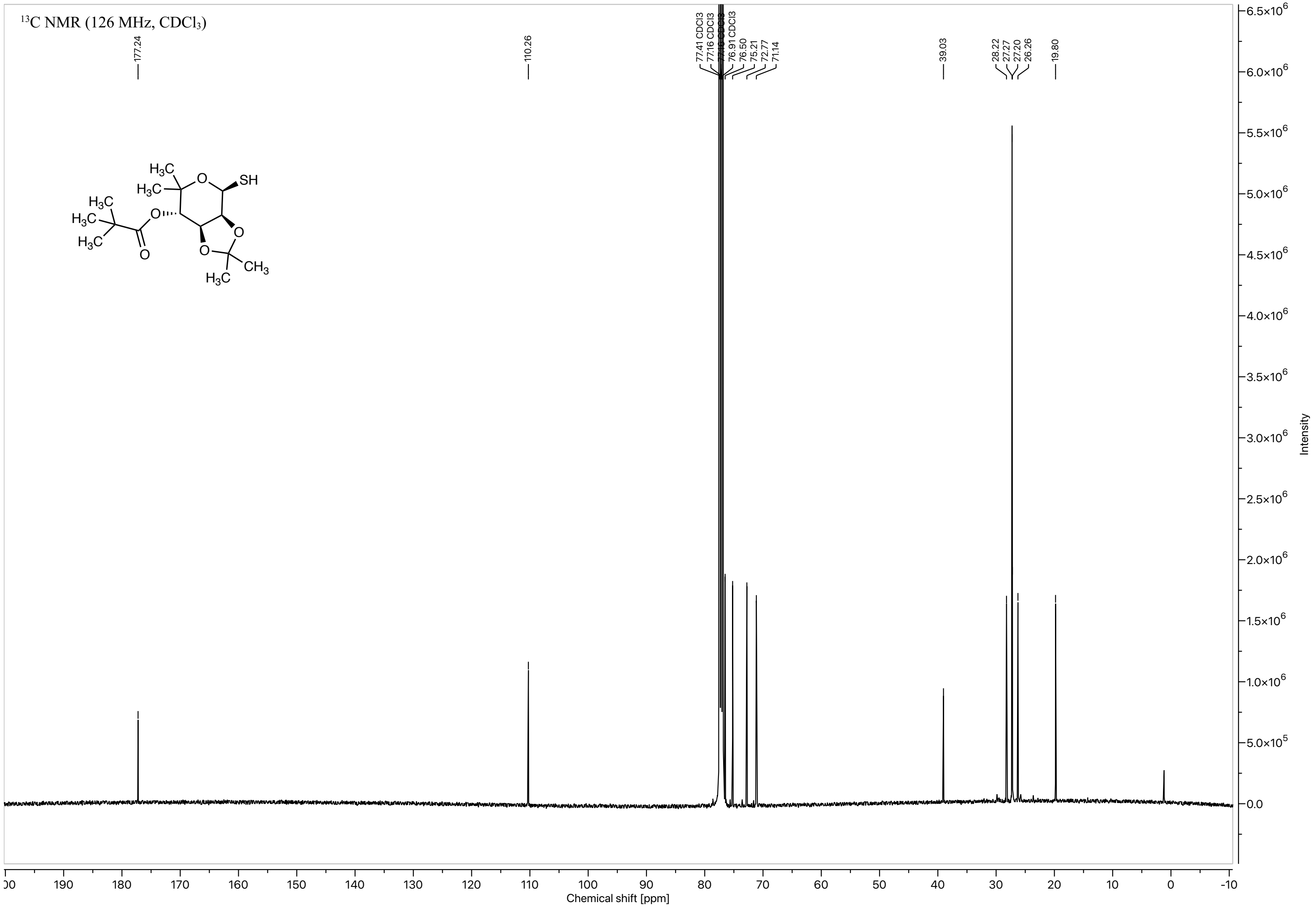

Figure 288: ^{13}C NMR spectrum of (3aS,4S,7S,7aS)-4-mercapto-2,2,6,6-tetramethyltetrahydro-4H-[1,3]dioxolo[4,5-c]pyran-7-yl pivalate (17c) in $CDCl_3$

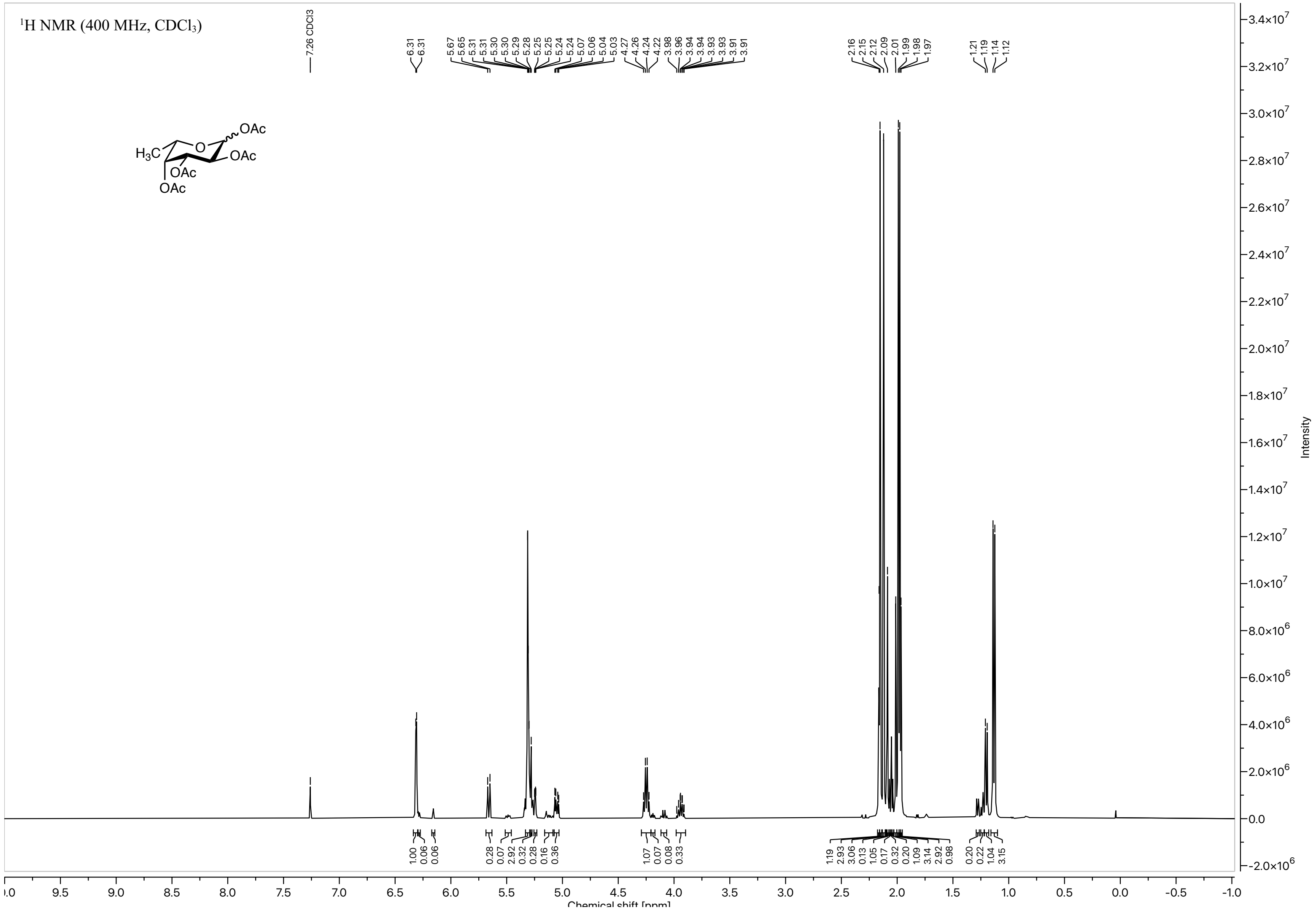

Figure 289: ^{1}H NMR spectrum of 1,2,3,4-tetra-*O*-acetyl-α/β-L-fucopyranose in $CDCl_3$

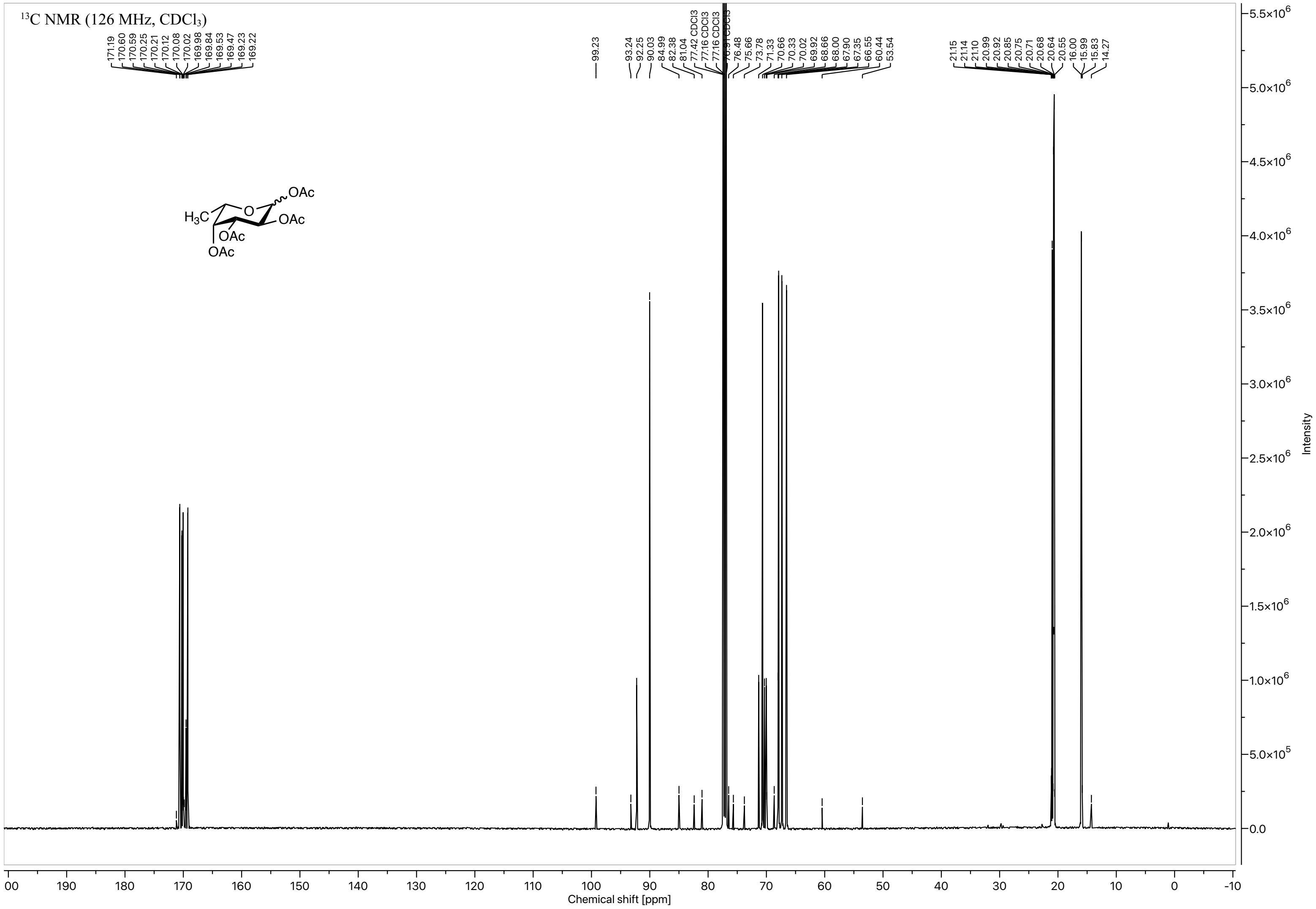

Figure 290: ^{13}C NMR spectrum of 1,2,3,4-tetra-*O*-acetyl-α/β-L-fucopyranose in CDCl$_3$

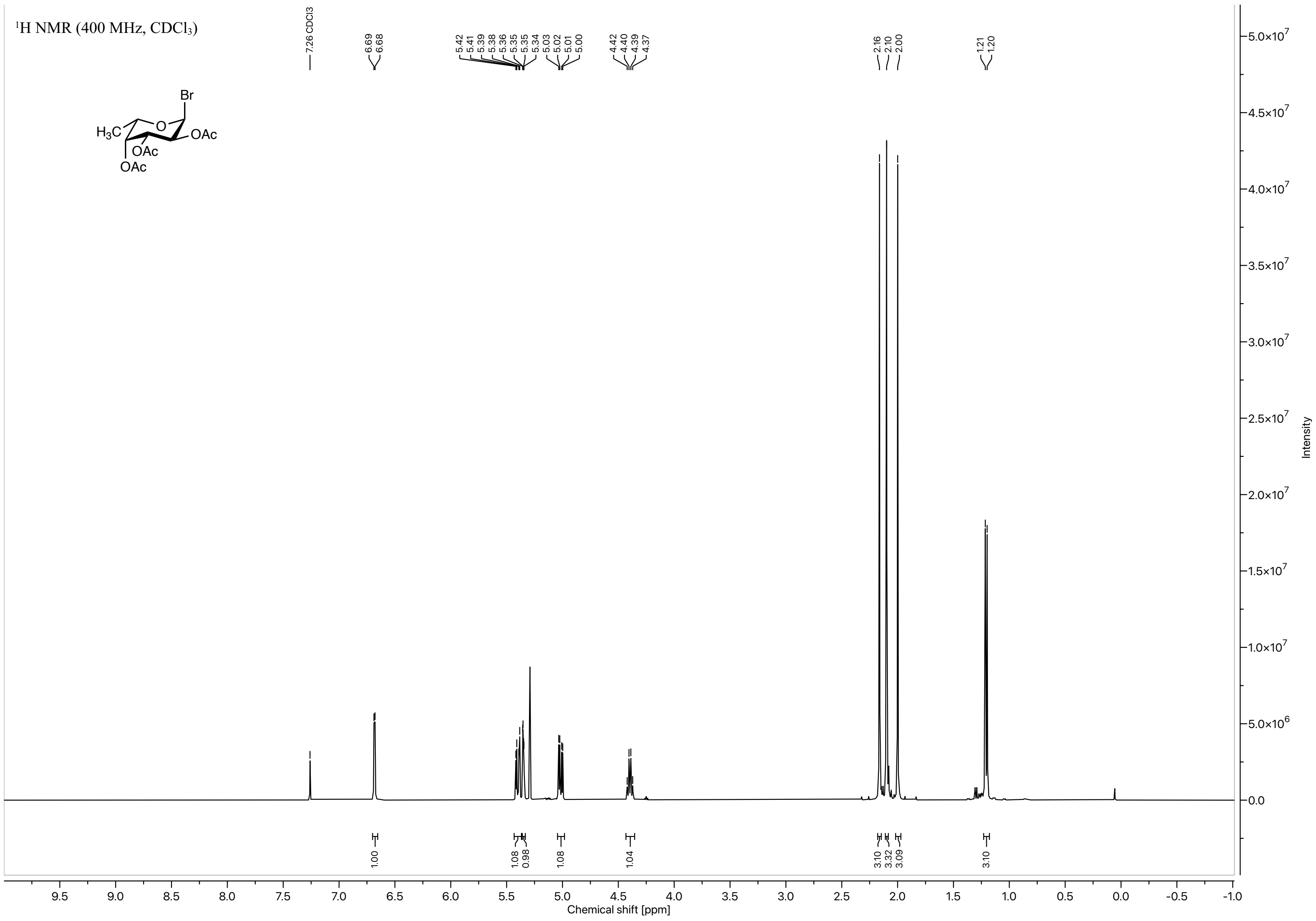

Figure 291: ^{1}H NMR spectrum of 2,3,4-tri-*O*-acetyl-α-L-fucopyranosyl bromide in $CDCl_3$

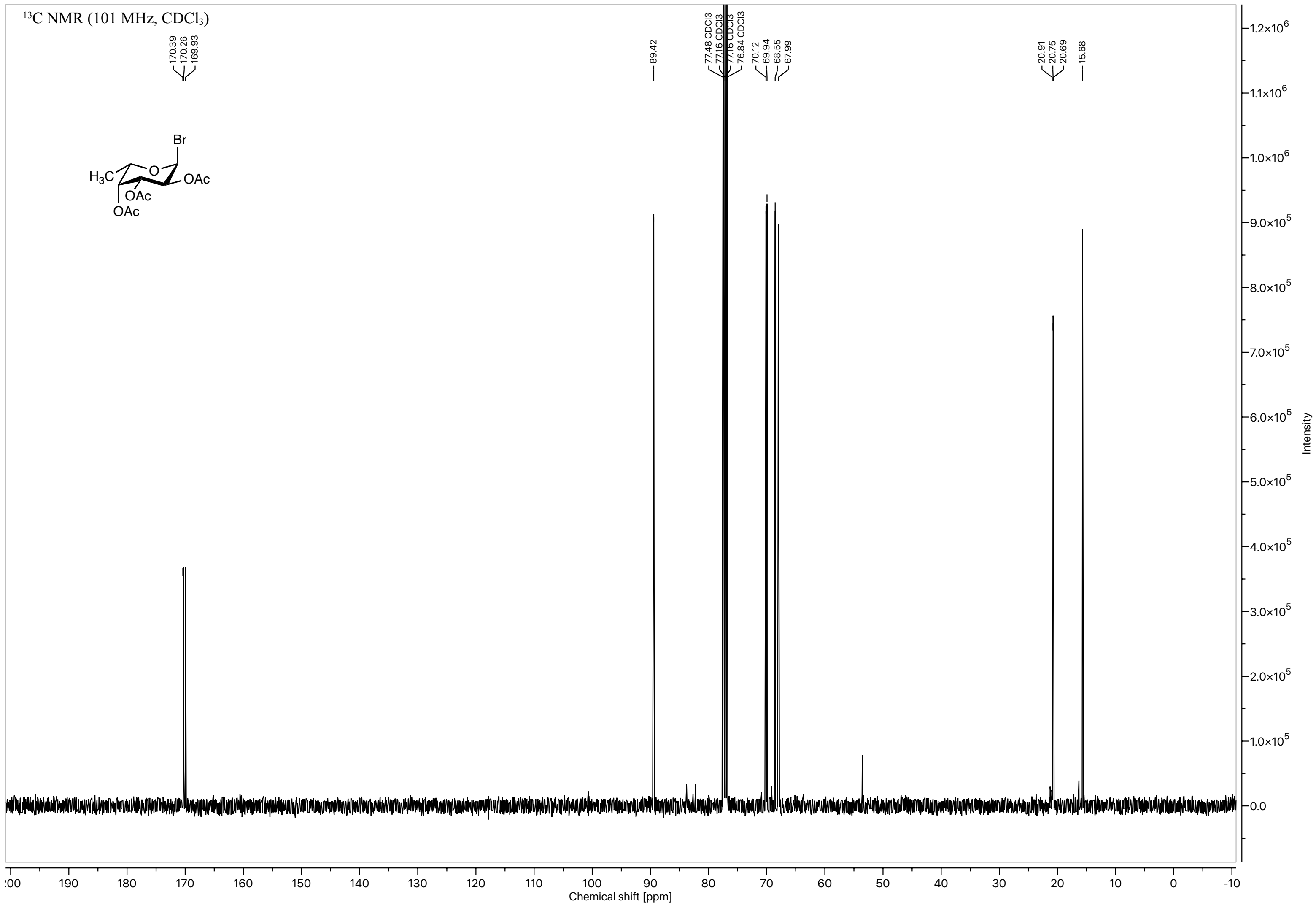

Figure 292: ^{13}C NMR spectrum of 2,3,4-tri-*O*-acetyl-α-L-fucopyranosyl bromide in $CDCl_3$

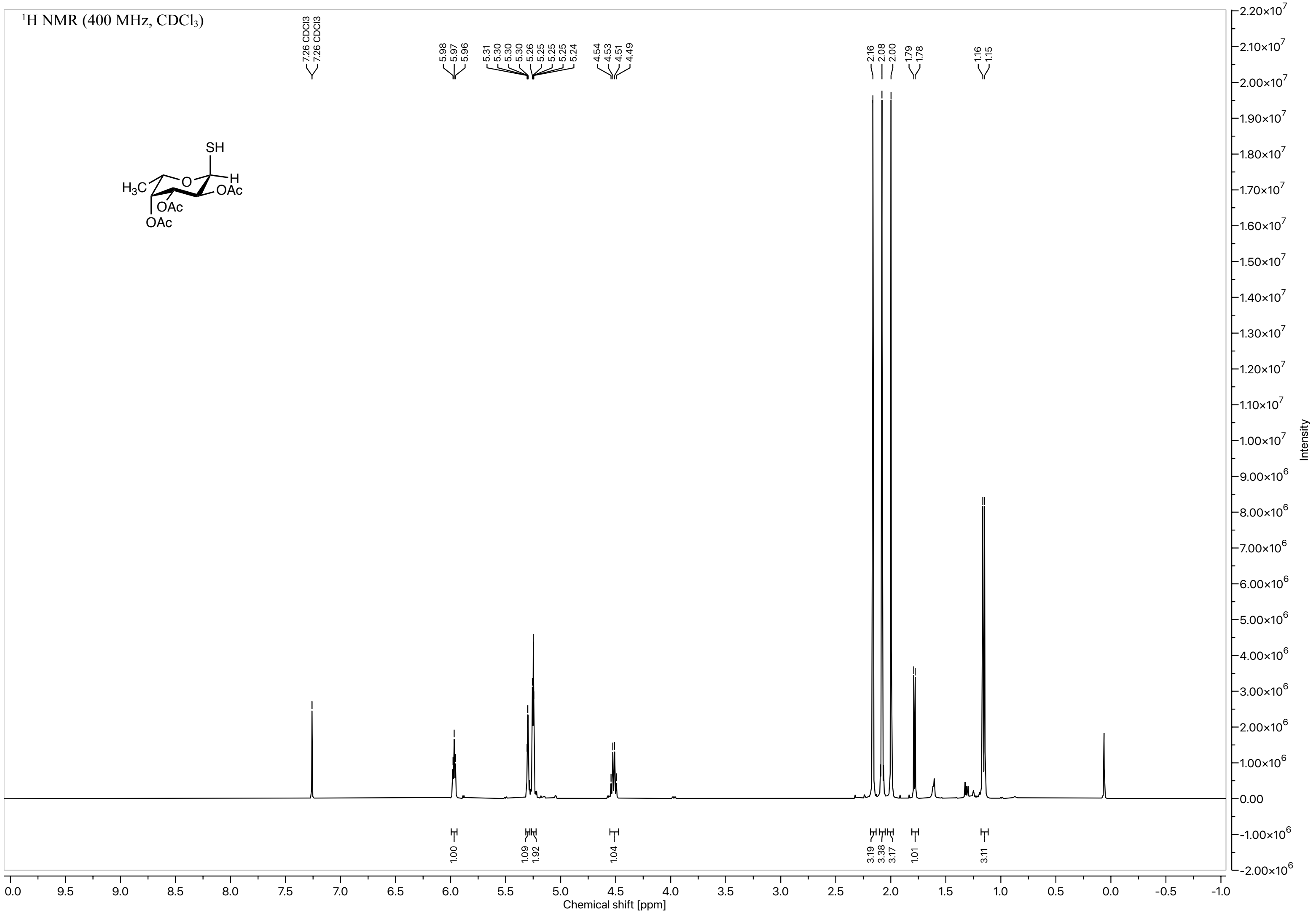

Figure 293: ^{1}H NMR spectrum of 2,3,4-tri-*O*-acetyl-1-thio-α-L-fucopyranose (6c) in $CDCl_3$

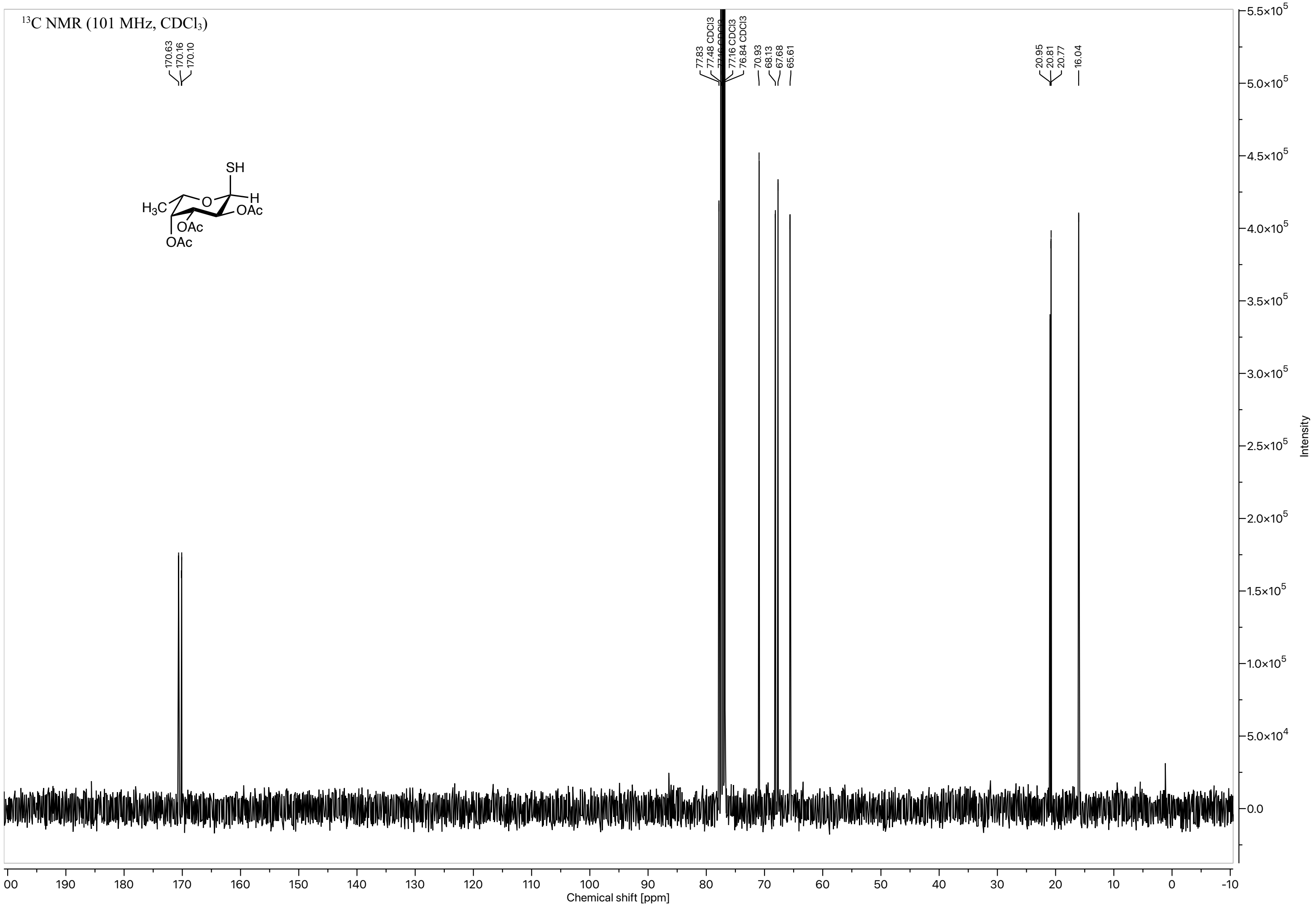

Figure 294: ^{13}C NMR spectrum of 2,3,4-tri-*O*-acetyl-1-thio-α-L-fucopyranose (6c) in $CDCl_3$

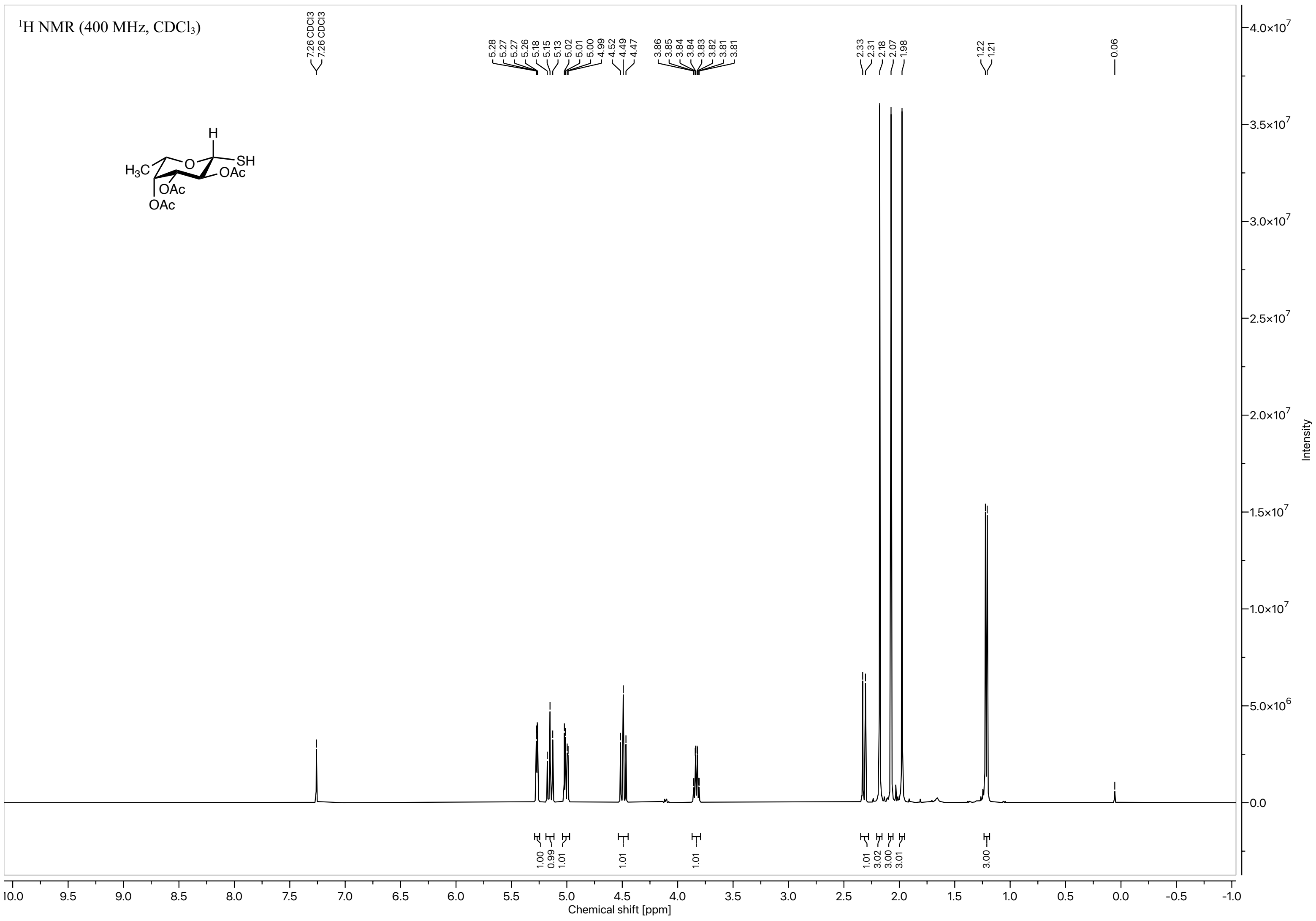

Figure 295: ^{1}H NMR spectrum of 2,3,4-tri-*O*-acetyl-1-thio-β-L-fucopyranose (6d) in $CDCl_3$

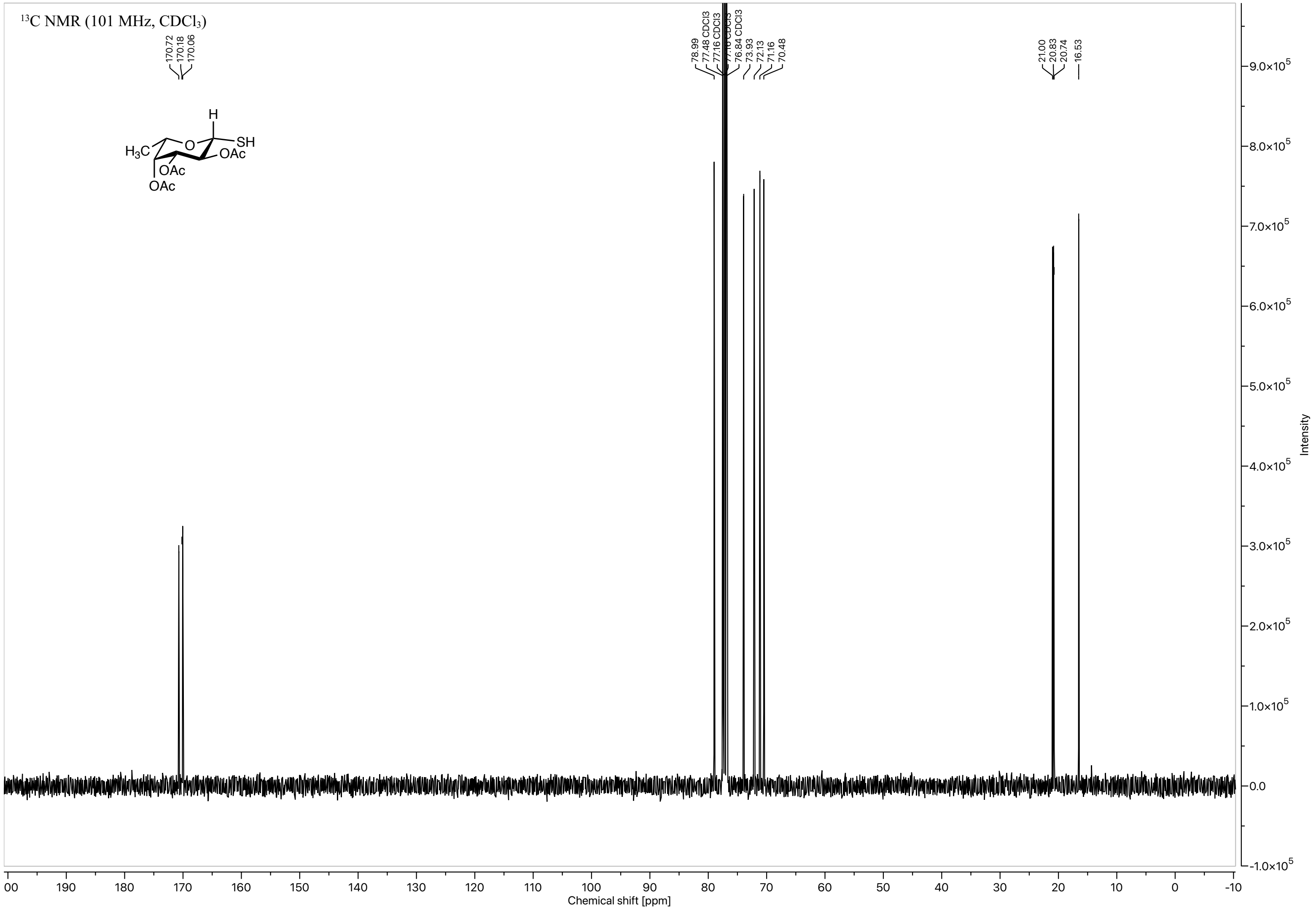

Figure 296: ^{13}C NMR spectrum of 2,3,4-tri-*O*-acetyl-1-thio-β-L-fucopyranose (6d) in $CDCl_3$

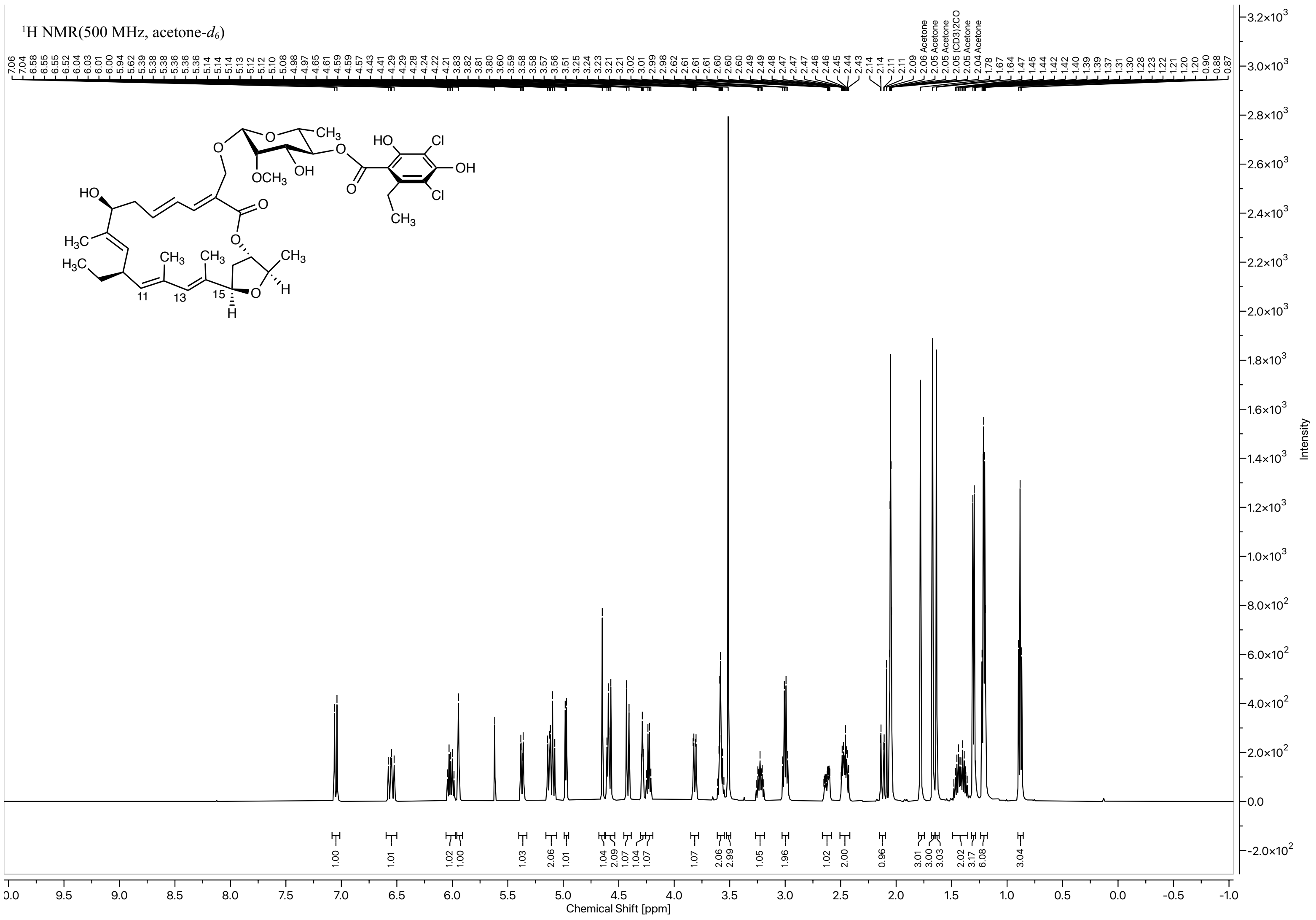

Figure 297: ^{1}H NMR spectrum of 15*R*-THF Fdx (2a) in acetone-d_6

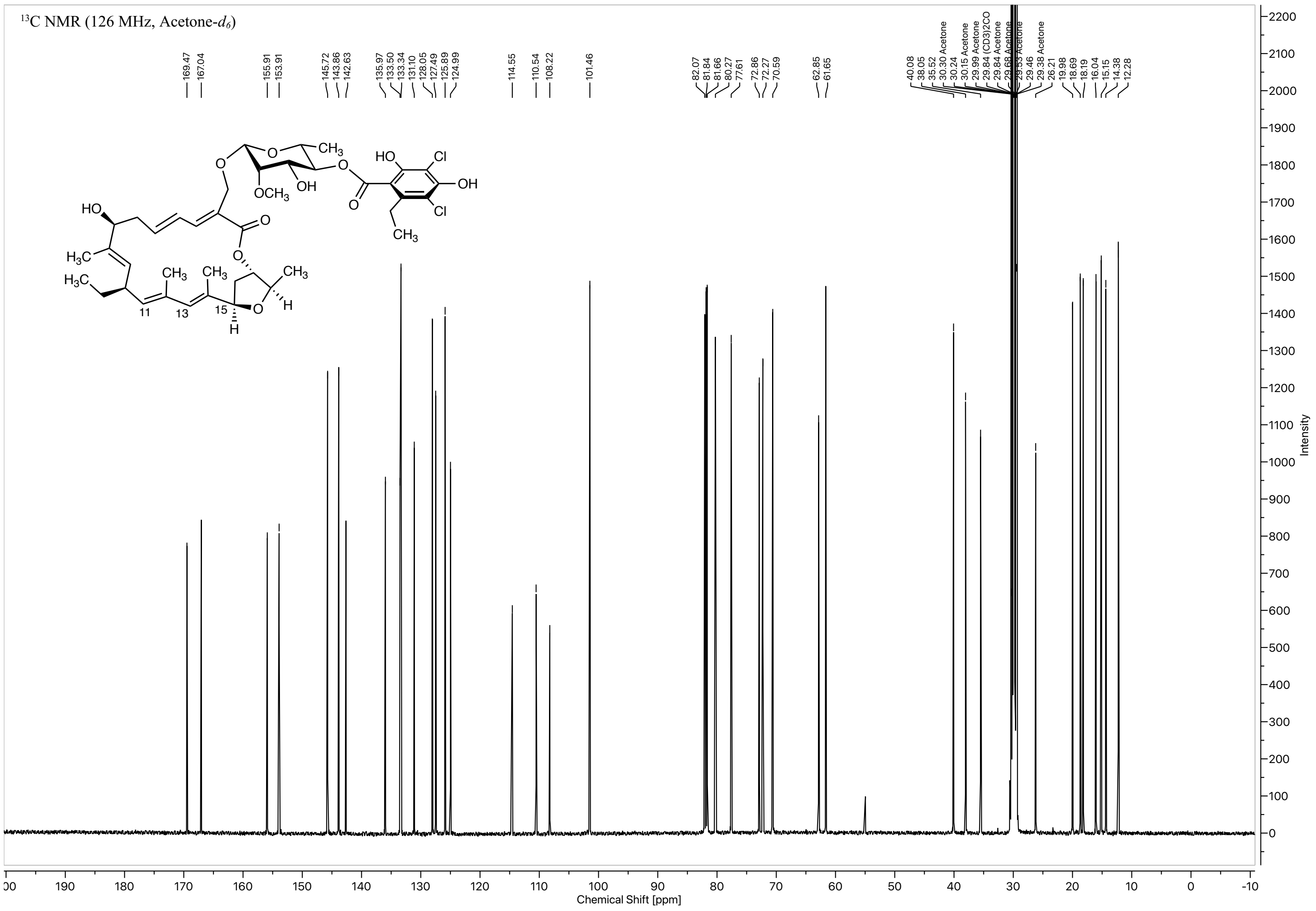

Figure 298: ^{13}C NMR spectrum of 15*R*-THF Fdx (2a) in acetone-d_6

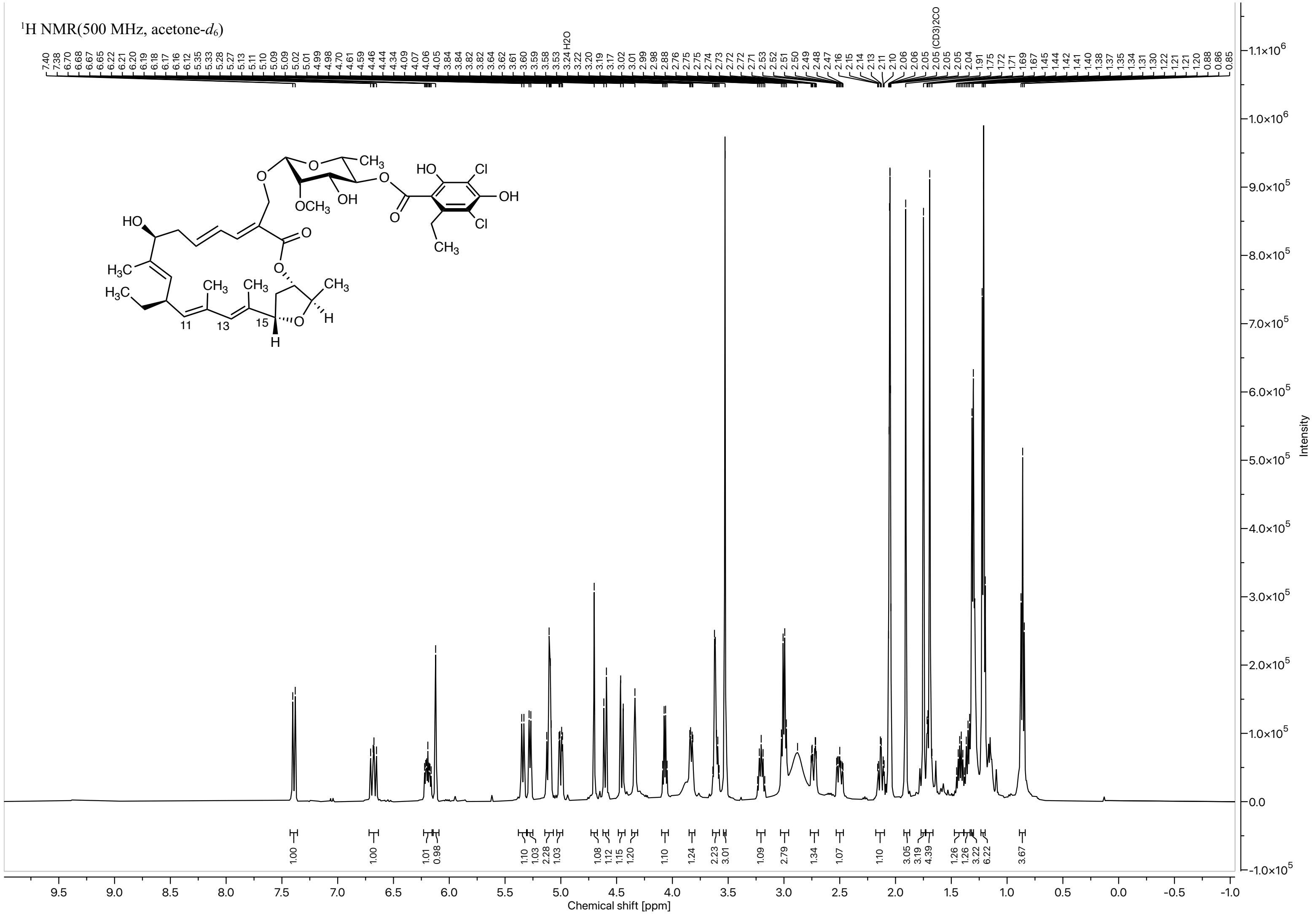

Figure 299: ^{1}H NMR spectrum of 15*S*-THF Fdx (2b) in acetone-d$_6$

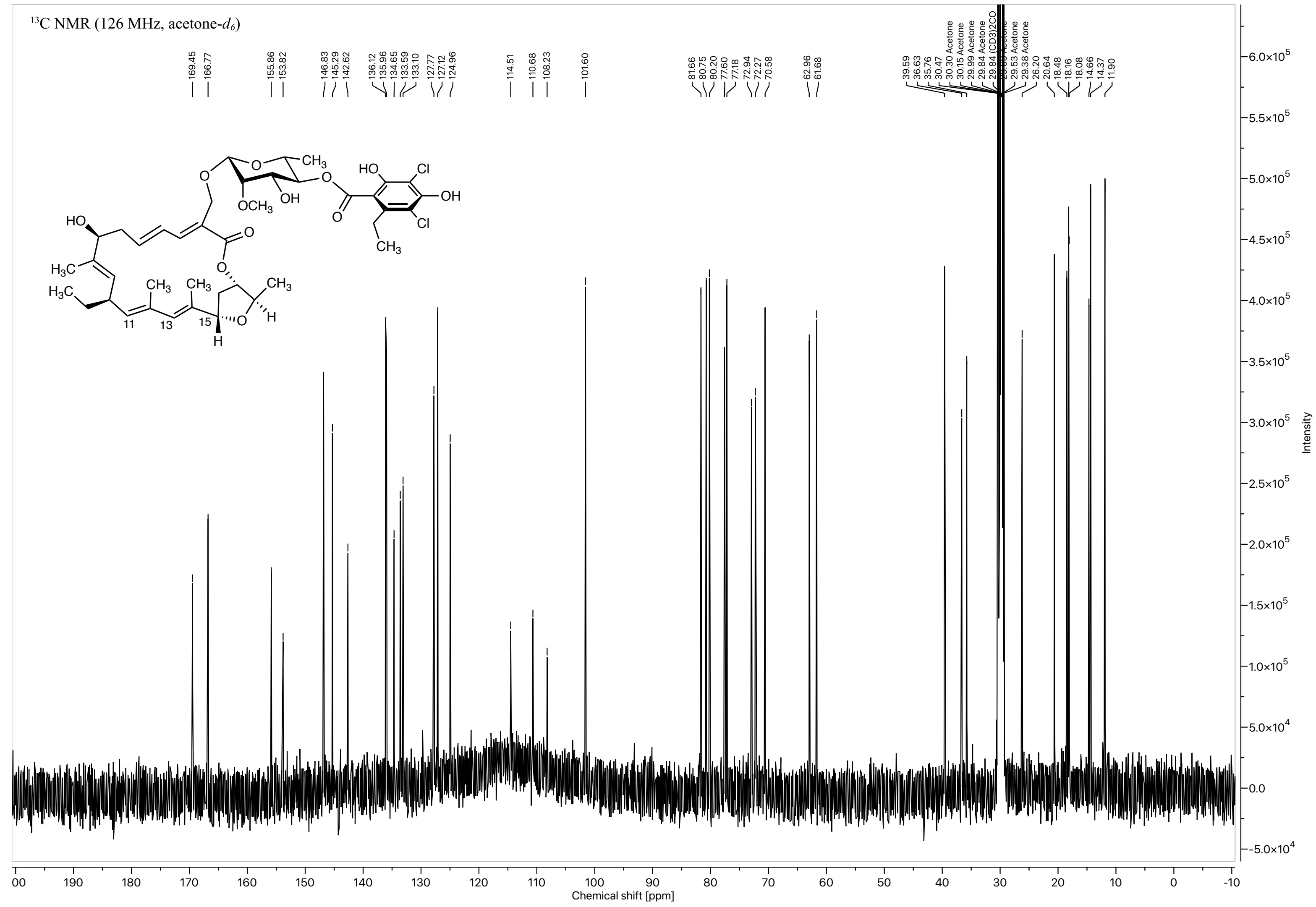

Figure 300: ^{13}C NMR spectrum of 15*S*-THF Fdx (2b) in acetone-d$_6$
