# Supplementary material for: Formal Single Atom Editing of the Glycosylated Natural Product Fidaxomicin Improves Acid Stability and Retains Antibiotic Activity
Source: JACS Au. 2024 May 21;4(6):2267–80. doi: 10.1021/jacsau.4c00206 (PMC11200244; doi:10.1021/jacsau.4c00206)
Supplement: Supplementary file 2 — au4c00206_si_002.pdf [file au4c00206_si_002.pdf]

Supplementary Information DP4+

Formal Single Atom Editing of the Glycosylated Natural Product Fidaxomicin Improves Acid Stability and Retains Antibiotic Activity

Isabella Ferrara[a], Gleb A. Chesnokov[a], Silvia Dittmann[b], Olivier Blacque[a], Susanne Sievers[b], and Karl Gademann*[a]

[a]Department of Chemistry, University of Zurich, Winterthurerstrasse 190, 8057 Zürich, Switzerland.

[b]Department for Microbial Physiology and Molecular Biology, Institute of Microbiology, Center for Functional Genomics of Microbes, University of Greifswald, Felix-Hausdorff-Strasse 8, 17489 Greifswald, Germany.

*Correspondence to Karl Gademann. E-Mail: karl.gademann@chem.uzh.ch

DP4+ Calculations

The absolute configuration for the individual constitutional isomers was assigned based on the coupling constants in ^{1}H NMR spectra and correlations in NOESY spectra. The determined stereoconfiguration was corroborated by DP4+ calculations following the protocol by Grimblat, Zanardi and Sarotti.[1]

Computational details

DFT calculations were carried out using the ORCA 5.0.1 (for **5b-C(11)**, **5b-C(13)**, **5b-C(15)**) or the ORCA 5.0.3 (for **18e-C(11)** (full molecule and simplified molecule), **18e-C(13)**, **18e-C(15)**) package[2–5] following the DP4+ protocol developed by Grimblat, Zanardi and Sarotti[1] as an improved method to the previously reported DP4 method by Smith and Goodman.[6] Initial input files were generated in Avogadro 1.2.0[7,8] starting out from the fidaxomicin structure (PDB ID: FI8[9]; rcsb.org[10]) and performing an energy minimization using the MMFF94 forcefield after modifying the structures. Following, a preoptimization of the geometry was performed with xtb version 6.5.0[11,12] including implicit solvation (solvent: acetone)[13]. Then a conformational search was performed with crest version 2.12[14,15] using the semi-empirical GFN2-xTB method[11] and implicit solvation (solvent: acetone)[13] (Note: For 11-desnoviosyl-xy-thio-(4''-desbutyryl)-β-D-noviosyl fidaxomicin structures, the GC *z*-matrix crossing was not performed (--nocross flag)). All conformers within 2 kcal/mol from the lowest energy conformer were reoptimized at the B3LYP/6-31G(d) level of theory (Note: Gaussian-style B3LYP/G functional was used here and further). All conformers within 2 kcal/mol from the global minimum were used for further analysis and the Boltzmann factors per conformer calculated ($e^{-\frac{E_i}{RT}}$). ^{1}H and ^{13}C magnetic shielding tensors (σ) were computed using gauge including atomic orbital (GIAO) single-point NMR calculations at the B3LYP/6-31G(d) level of theory with the CPCM[16,17] implicit solvation model (solvent: acetone).[1] Furthermore, the resolution of identity (RI) approximation was used in order to speed up the calculations (ORCA keywords RIJCOSX[18] and AutoAux[19] used). Hs connected to heteroatoms and the dummy methyl group to simplify the rhamnose-resorcinol moiety were excluded for further analysis. Boltzmann averaged shielding tensors for nucleus n ($\sigma_{\text{avrg}}^{\text{n}}$) weighting the conformers according to their Boltzmann factors were calculated following equation 1:

$$\sigma_{\text{avrg}}^{\text{n}} = \frac{\Sigma_i \sigma_i^{\text{n}} * e^{-\frac{E_i}{RT}}}{\Sigma_i e^{-\frac{E_i}{RT}}}$$

Where σ_i and E_i are the shielding tensor and the relative energy of the ith conformer, respectively, R is the ideal gas constant (8.3145 J/K·mol), and T is the temperature (298.15 K).[1] The same level of theory (CPCM, solvent: acetone/B3LYP/6-31G(d)) was used to compute the shielding tensors for tetramethylsilane (TMS) (σ_C(TMS): 190.0976; σ_H(TMS): 32.148) as a reference to calculate the Boltzmann averaged unscaled NMR chemical shifts per nucleus. DP4+ probabilities were calculated from the unscaled Boltzmann averaged NMR chemical shifts using the Excel spreadsheet provided by Grimblat, Zanardi and Sarotti.[1]

Computational results

The following data refers if not indicated differently to unscaled data (unscaled computed NMR chemical shifts δ or Δδ obtained from ($\delta_{\text{comp\_unscaled}}-\delta_{\text{exp}}$).

For methyl groups (Me), the processed computed ^{1}H chemical shifts (i.e.: Comp. δ; σ Boltz. avrg.; Computed avrg. δ; Δδ) refer to the averaged data including all three H nuclei per Me whereas the raw computed σ for H-nuclei for each conformer (i.e.: Conformers, shielding tensor σ) refer to one of the H-nuclei of the Methyl group only (For simplicity the other two H-nuclei data sets are not shown). As stated in the individual data sets, H20a (H_{Re}) and H20b

(H_{Si}) could not be unambiguously assigned and thus, the experimental chemical shift could be the other way round, than shown in the tables. However, as this stereocenter is relatively far away from the C(11)-, C(13)-, and C(15)-position, it does not significantly affect the results.

11-Desnoviosyl-11-*p*-tolylsulfide fidaxomicin (**5b-C(11)**)

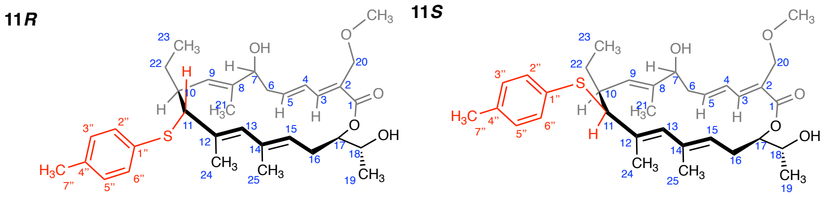

*Table 1: Experimental vs. computed ^{1}H chemical shifts and DP4+ results for simplified 11-desnoviosyl-11-p-tolylsulfide fidaxomicin **5b-C(11)** (PCM, solvent: acetone/B3LYP/6-31G(d))*

H-atom	Exp. δ, ppm	11*R*		11S	
		Comp. δ, ppm	Δδ, ppm	Comp. δ, ppm	Δδ, ppm
H(3)	7.21	7.25	0.04	7.37	0.16
H(4)	6.62	6.75	0.13	6.82	0.20
H(5)	5.98	6.45	0.47	6.37	0.39
H(6a, H_{Si})	2.50	2.51	0.01	2.42	-0.08
H(6b, H_{Re})	2.71	2.81	0.10	2.92	0.21
H(7)	4.27	4.30	0.03	4.25	-0.02
H(9)	5.34	5.23	-0.11	5.58	0.24
H(10)	2.61	2.80	0.19	3.11	0.50
H(11)	3.50	3.54	0.04	3.96	0.46
H(13)	5.31	4.93	-0.38	6.20	0.89
H(15)	5.22	5.00	-0.22	5.39	0.17
H(16a, H_{Si})	2.61	1.93	-0.68	2.14	-0.47
H(16b, H_{Re})	2.34	2.63	0.29	2.73	0.39
H(17)	4.69	4.49	-0.20	4.66	-0.03
H(18)	3.97	3.68	-0.29	3.75	-0.22
H(19)-Me	1.16	0.96	-0.20	0.99	-0.17
H(20a, H_{Re})	4.58[a]	4.24	-0.34	4.21	-0.37
H(20b, H_{Si})	4.40[a]	4.04	-0.36	4.14	-0.26
H(21)-Me	1.68	1.73	0.05	1.70	0.02
H(22a, H_{Re})	2.15	2.36	0.21	1.78	-0.37
H(22b, H_{Si})	1.46	1.26	-0.20	1.66	0.20
H(23)-Me	0.89	0.88	-0.01	0.95	0.06
H(24)-Me	1.84	1.99	0.15	1.92	0.08
H(25)-Me	1.56	1.87	0.31	1.87	0.31
H(2")	7.30	7.38	0.08	7.11	-0.19
H(3")	7.10	7.20	0.10	7.09	-0.01
H(5")	7.10	7.16	0.06	7.14	0.04
H(6")	7.30	7.32	0.02	7.20	-0.10
H(7")-Me	2.28	2.25	-0.03	2.19	-0.09
**DP4+ (^{1}H data)**		**100.00%**		**0.00%**	
**DP4+ (all data)**		**100.00%**		**0.00%**	

[a]H20a, H_{Re} and H20b, H_{Si} could not be unambiguously assigned and thus, the experimental chemical shift could be the other way round.

11*R*

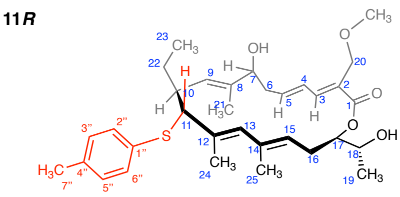

11*S*

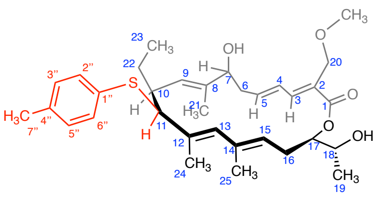

Table 2: Experimental vs. computed ^{13}*C chemical shifts and DP4+ results for simplified 11-desnoviosyl-11-p-tolylsulfide fidaxomicin* ***5b-C(11)*** *(PCM, solvent: acetone/B3LYP/6-31G(d))*

C-atom	Exp. δ, ppm	11*R*		11S	
		Comp. δ, ppm	Δδ, ppm	Comp. δ, ppm	Δδ, ppm
C(1)	167.6	163.8	-3.8	163.7	-3.9
C(2)	125.2	119.5	-5.7	119.7	-5.5
C(3)	145.5	143.5	-2.0	143.8	-1.7
C(4)	128.1	124.0	-4.1	124.1	-4.0
C(5)	143.6	144.0	0.4	143.6	0.0
C(6)	37.2	38.8	1.6	39.1	1.9
C(7)	72.9	73.3	0.4	73.3	0.4
C(8)	136.7	134.8	-1.9	134.7	-2.0
C(9)	126.0	122.6	-3.4	120.6	-5.4
C(10)	41.2	42.3	1.1	44.8	3.6
C(11)	66.7	78.1	11.4	70.6	3.9
C(12)	134.0	130.7	-3.3	132.8	-1.2
C(13)	133.4	128.6	-4.8	127.1	-6.3
C(14)	135.9	131.9	-4.0	131.2	-4.7
C(15)	125.2	128.1	2.9	127.2	2.0
C(16)	28.3	33.2	4.9	33.4	5.1
C(17)	78.0	83.8	5.8	84.5	6.5
C(18)	67.5	69.1	1.6	69.1	1.6
C(19)	20.7	17.5	-3.2	17.6	-3.1
C(20)	63.3	64.3	1.0	64.3	1.0
C(21)	15.1	16.2	1.1	15.2	0.1
C(22)	27.8	29.0	1.2	31.1	3.3
C(23)	10.8	12.7	1.9	12.8	2.0
C(24)	13.7	14.9	1.2	20.4	6.7
C(25)	17.4	16.8	-0.6	17.3	-0.1
C(1")	132.9	129.4	-3.5	133.2	0.3
C(2")	135.0	130.0	-5.0	122.0	-13.0
C(3")	130.1	123.1	-7.0	123.3	-6.8
C(4")	138.1	133.9	-4.2	130.5	-7.6
C(5")	130.1	123.0	-7.1	122.8	-7.3
C(6")	135.0	130.2	-4.8	122.7	-12.3
C(7")	21.1	21.0	-0.1	20.8	-0.3
**DP4+ (^{13}C data)**		**100.00%**		**0.00%**	
**DP4+ (all data)**		**100.00%**		**0.00%**	

*Table 3: DP4+ results for **11R**: Computed σ for H-nuclei for each conformer and the averaged σ (conformers weighted based on Boltzmann factors) for simplified 11-desnoviosyl-11R-p-tolylsulfide fidaxomicin **5b-C(11)**. Computed chemical shifts δ were calculated using TMS as a reference (σ_H(TMS): 32.148). (PCM, solvent: acetone/B3LYP/6-31G(d)). Total amount of conformers: 37.*

**H-atom**	**Exp. δ, ppm**	**Conformers, shielding tensor σ**															**σ Boltz. avrg.**	**Comp. avrg. δ, ppm**	**Δδ, ppm**
		**384**	**185**	**117**	**385**	**102**	**388**	**478**	**…**	**353**	**306**	**392**	**344**	**69**	**295**	**154**			
H(3)	7.21	24.97	24.95	24.89	24.88	24.89	24.86	24.86	…	25.07	24.65	24.76	24.76	24.77	24.91	24.97	24.90	7.25	0.04
H(4)	6.62	25.55	25.48	25.39	25.53	25.39	25.51	25.37	…	25.39	25.10	25.43	25.45	25.26	25.40	25.46	25.40	6.75	0.13
H(5)	5.98	25.57	25.76	25.62	25.55	25.62	25.58	25.55	…	25.93	25.76	25.74	25.78	25.82	25.63	25.86	25.70	6.45	0.47
H(6a, H_{Si})	2.50	29.56	29.71	29.56	29.54	29.56	29.55	29.52	…	29.76	29.73	29.71	29.73	29.72	29.77	29.33	29.64	2.51	0.01
H(6b, H_{Re})	2.71	29.50	29.19	29.44	29.46	29.45	29.45	29.46	…	29.22	29.14	29.20	29.21	29.21	29.36	29.58	29.34	2.81	0.10
H(7)	4.27	27.75	27.96	27.73	27.73	27.73	27.74	27.72	…	27.98	27.88	28.00	28.01	28.01	27.74	28.20	27.84	4.30	0.03
H(9)	5.34	26.82	27.07	26.84	26.85	26.84	26.86	26.77	…	27.08	26.90	27.10	27.13	27.08	26.25	26.83	26.92	5.23	-0.11
H(10)	2.61	29.43	29.32	29.31	29.37	29.30	29.39	29.43	…	29.32	29.47	29.25	29.25	29.23	29.07	29.33	29.35	2.80	0.19
H(11)	3.50	28.65	28.59	28.53	28.63	28.49	28.66	28.62	…	28.58	28.69	28.59	28.62	28.56	28.23	28.55	28.60	3.54	0.04
H(13)	5.31	27.17	27.31	27.20	27.20	27.18	27.20	27.11	…	27.32	27.29	26.09	26.12	26.06	27.12	27.14	27.21	4.93	-0.38
H(15)	5.22	27.11	27.23	27.16	27.14	27.15	27.14	27.03	…	27.25	26.54	26.53	26.55	26.49	27.13	27.14	27.15	5.00	-0.22
H(16a, H_{Si})	2.61	30.23	30.25	30.25	30.23	30.26	30.22	30.23	…	30.28	29.76	29.99	29.99	29.99	30.25	30.23	30.22	1.93	-0.68
H(16b, H_{Re})	2.34	29.50	29.53	29.53	29.50	29.54	29.49	29.50	…	29.30	29.72	29.32	29.32	29.33	29.54	29.54	29.51	2.63	0.29
H(17)	4.69	27.66	27.62	27.65	27.68	27.65	27.66	27.71	…	27.75	27.69	27.47	27.46	27.44	27.63	27.74	27.66	4.49	-0.20
H(18)	3.97	28.48	28.46	28.49	28.48	28.49	28.47	28.51	…	27.89	28.42	28.36	28.35	28.37	28.48	28.53	28.47	3.68	-0.29
H(19)-Me	1.16	31.29	31.13	31.34	31.10	31.34	31.27	31.13	…	31.22	31.23	31.21	31.19	31.29	31.15	31.13	31.18	0.96	-0.20
H(20a, H_{Re})	4.58[a]	27.66	28.17	27.71	28.09	27.71	28.07	27.71	…	27.78	28.04	28.05	28.01	27.71	27.73	27.72	27.91	4.24	-0.34
H(20b, H_{Si})	4.40[a]	28.24	28.33	28.16	28.23	28.15	28.22	28.18	…	28.25	27.72	28.20	28.19	28.09	28.16	28.22	28.11	4.04	-0.36
H(21)-Me	1.68	30.78	30.79	30.51	30.76	30.02	30.04	30.05	…	30.38	30.74	30.37	30.02	30.01	30.72	30.10	30.42	1.73	0.05
H(22a, H_{Re})	2.15	29.67	29.88	29.83	29.68	29.85	29.66	29.65	…	29.90	29.53	30.01	30.01	30.02	30.71	29.82	29.79	2.36	0.21
H(22b, H_{Si})	1.46	30.93	30.87	30.93	30.94	30.93	30.94	30.90	…	30.88	30.90	31.46	31.51	31.47	30.01	30.88	30.89	1.26	-0.20
H(23)-Me	0.89	31.44	31.19	31.22	31.21	31.22	31.23	31.20	…	31.44	31.14	31.34	31.35	31.34	31.28	31.19	31.27	0.88	-0.01
H(24)-Me	1.84	30.28	30.19	30.16	30.22	30.00	30.31	29.88	…	30.30	30.14	30.72	30.73	30.19	30.17	30.21	30.16	1.99	0.15
H(25)-Me	1.56	30.11	30.41	30.41	30.27	30.17	30.11	30.12	…	30.27	31.31	30.22	30.23	29.96	30.32	30.21	30.28	1.87	0.31
H(2'')	7.30	24.67	24.66	24.70	24.95	24.67	24.94	24.92	…	24.96	24.69	24.62	24.82	24.63	24.59	24.67	24.77	7.38	0.08
H(3'')	7.10	24.87	24.86	24.93	25.09	24.87	25.08	25.07	…	25.10	24.96	24.89	24.99	24.92	24.84	24.88	24.95	7.20	0.10
H(5'')	7.10	25.05	25.09	25.00	24.84	25.07	24.84	24.85	…	24.88	24.93	25.06	24.96	25.03	25.08	25.05	24.99	7.16	0.06
H(6'')	7.30	24.91	24.96	24.89	24.65	24.93	24.65	24.64	…	24.68	24.95	24.88	24.67	24.86	24.93	24.93	24.83	7.32	0.02
H(7'')-Me	2.28	30.15	30.27	30.08	29.70	30.22	29.68	29.68	…	29.74	29.72	29.86	29.89	29.99	29.71	29.70	29.90	2.25	-0.03
Energy (kJ/mol)		0.00	0.39	0.60	0.68	0.68	0.79	0.79	…	7.02	7.17	7.20	7.51	7.53	7.90	8.31	**DP4+ (^{1}H data)**		
Boltzmann factor		0.08	0.07	0.06	0.06	0.06	0.06	0.06	…	0.00	0.00	0.00	0.00	0.00	0.00	0.00	100.00%		

[a]H20a, H_{Re} and H20b, H_{Si} could not be unambiguously assigned and thus, the experimental chemical shift could be the other way round.

*Table 4: DP4+ results for **11R**: Computed σ for C-nuclei for each conformer and the averaged σ (conformers weighted based on Boltzmann factors) for simplified 11-desnoviosyl-11R-p-tolylsulfide fidaxomicin **5b-C(11)**. Computed chemical shifts δ were calculated using TMS as a reference (σ_C(TMS): 190.0976). (PCM, solvent: acetone/B3LYP/6-31G(d)). Total amount of conformers: 37.*

**C-atom**	***Exp. δ, ppm***	***Conformers, shielding tensor σ***															***σ Boltz. avrg.***	***Comp. avrg. δ, ppm***	***Δδ, ppm***
		**384**	**185**	**117**	**385**	**102**	**388**	**478**	**…**	**353**	**306**	**392**	**344**	**69**	**295**	**154**			
C(1)	167.6	26.3	26.4	25.4	26.4	25.4	26.6	25.3	…	27.9	26.7	26.6	26.8	25.7	25.4	25.1	26.3	163.8	-3.8
C(2)	125.2	71.7	69.9	70.7	71.3	70.6	71.5	70.4	…	68.2	71.0	69.9	70.0	69.4	70.4	69.8	70.6	119.5	-5.7
C(3)	145.5	47.6	46.6	45.9	45.3	45.9	45.1	45.9	…	49.0	47.1	46.4	46.3	46.7	46.2	44.8	46.6	143.5	-2.0
C(4)	128.1	68.9	64.8	67.0	67.9	67.0	67.2	67.4	…	64.3	64.4	64.3	64.0	64.0	66.5	68.7	66.1	124.0	-4.1
C(5)	143.6	45.0	47.9	44.5	43.3	44.5	43.7	44.2	…	50.3	47.6	47.4	47.7	48.2	44.7	44.7	46.1	144.0	0.4
C(6)	37.2	152.0	151.0	152.0	152.0	151.9	151.9	151.9	…	151.4	151.1	150.9	151.0	151.0	149.4	151.0	151.3	38.8	1.6
C(7)	72.9	116.0	117.5	115.9	115.9	116.0	115.9	116.0	…	117.7	117.4	117.4	117.6	117.4	117.1	117.7	116.8	73.3	0.4
C(8)	136.7	54.3	55.8	54.4	54.5	54.3	54.3	54.3	…	55.7	55.8	56.2	56.2	56.2	59.3	56.1	55.3	134.8	-1.9
C(9)	126.0	67.4	67.5	67.6	67.3	67.6	67.4	67.6	…	67.6	68.6	67.4	67.5	67.5	66.7	66.1	67.5	122.6	-3.4
C(10)	41.2	147.7	147.8	147.9	147.9	147.9	147.8	147.5	…	147.8	147.0	142.9	142.9	142.8	151.0	147.6	147.8	42.3	1.1
C(11)	66.7	111.2	112.8	112.5	111.2	112.8	111.0	111.2	…	112.8	112.0	111.9	111.5	111.9	116.8	113.1	112.0	78.1	11.4
C(12)	134.0	59.3	59.9	60.0	59.2	60.1	59.2	59.3	…	60.4	56.6	55.5	55.7	55.6	60.1	59.1	59.4	130.7	-3.3
C(13)	133.4	61.8	60.9	60.6	61.9	60.5	61.9	61.7	…	60.8	65.4	62.5	62.5	62.5	60.3	61.0	61.5	128.6	-4.8
C(14)	135.9	57.6	58.5	58.3	58.1	58.3	58.3	57.3	…	58.2	58.6	57.9	58.0	57.8	58.4	57.9	58.2	131.9	-4.0
C(15)	125.2	62.4	61.7	61.6	61.9	61.5	61.7	62.6	…	62.0	69.0	61.3	61.3	61.3	61.3	62.5	62.0	128.1	2.9
C(16)	28.3	156.7	156.9	156.7	156.7	156.7	156.8	156.5	…	163.4	157.1	156.6	156.7	156.6	156.8	156.5	156.9	33.2	4.9
C(17)	78.0	106.5	106.3	106.3	106.4	106.3	106.4	106.1	…	111.0	103.5	105.8	105.9	106.1	106.3	105.4	106.3	83.8	5.8
C(18)	67.5	120.9	121.0	121.0	121.1	121.0	121.0	121.0	…	122.2	121.2	121.0	121.0	120.9	121.0	121.5	121.0	69.1	1.6
C(19)	20.7	172.6	172.6	172.7	172.7	172.7	172.6	172.8	…	172.9	172.8	172.5	172.5	172.5	172.7	172.8	172.6	17.5	-3.2
C(20)	63.3	126.4	125.3	126.0	125.4	126.0	125.0	126.0	…	126.3	124.8	125.7	125.4	126.2	126.1	125.8	125.8	64.3	1.0
C(21)	15.1	173.9	173.8	173.9	173.9	173.9	173.9	174.0	…	173.8	174.4	173.9	174.0	173.9	174.7	172.3	173.9	16.2	1.1
C(22)	27.8	161.2	161.2	161.2	161.1	161.2	161.0	161.1	…	161.2	160.3	161.3	161.3	161.3	161.2	161.7	161.1	29.0	1.2
C(23)	10.8	177.4	177.2	177.3	177.4	177.3	177.4	177.3	…	177.2	177.6	177.3	177.3	177.3	182.1	177.3	177.4	12.7	1.9
C(24)	13.7	174.8	175.2	175.6	175.3	175.7	175.2	174.7	…	175.2	174.8	173.5	173.4	173.5	176.1	174.9	175.2	14.9	1.2
C(25)	17.4	173.3	173.3	173.4	173.3	173.4	173.3	173.3	…	173.2	171.8	172.9	172.9	172.8	173.5	173.3	173.3	16.8	-0.6
C(1'')	132.9	60.6	61.2	61.3	60.2	61.4	60.2	60.9	…	61.2	60.4	56.6	56.7	56.6	61.5	61.3	60.7	129.4	-3.5
C(2'')	135.0	60.5	60.3	60.3	59.7	60.4	59.7	59.5	…	58.9	60.0	64.5	64.3	64.4	60.3	60.4	60.1	130.0	-5.0
C(3'')	130.1	66.8	67.1	66.9	67.1	67.1	67.2	67.1	…	67.1	66.4	66.5	67.2	66.5	67.1	66.9	67.0	123.1	-7.0
C(4'')	138.1	56.1	56.0	56.0	56.3	56.0	56.2	56.1	…	56.1	55.9	57.9	57.8	57.9	56.0	56.0	56.2	133.9	-4.2
C(5'')	130.1	67.2	67.1	67.4	66.8	67.1	66.8	66.9	…	67.2	67.7	67.0	66.3	67.0	67.0	67.2	67.1	123.0	-7.1
C(6'')	135.0	59.6	58.9	58.9	60.7	58.9	60.6	60.3	…	60.3	59.4	64.8	64.2	64.7	59.0	59.0	59.9	130.2	-4.8
C(7'')	21.1	169.1	169.1	169.1	169.1	169.1	169.1	169.1	…	169.1	169.2	169.2	169.2	169.2	169.1	169.1	169.1	21.0	-0.1
Energy (kJ/mol)		0.00	0.39	0.60	0.68	0.68	0.79	0.79	…	7.02	7.17	7.20	7.51	7.53	7.90	8.31	**DP4+ (^{13}C data)**		
Boltzmann factor		0.08	0.07	0.06	0.06	0.06	0.06	0.06	…	0.00	0.00	0.00	0.00	0.00	0.00	0.00	100.00%		

*Table 5: DP4+ results for **11S**: Computed σ for H-nuclei for each conformer and the averaged σ (conformers weighted based on Boltzmann factors) for simplified 11-desnoviosyl-11S-p-tolylsulfide fidaxomicin **5b-C(11)**. Computed chemical shifts δ were calculated using TMS as a reference (σ_H(TMS): 32.148). (PCM, solvent: acetone/B3LYP/6-31G(d)). Total amount of conformers: 63.*

H-atom	***Exp. δ, ppm***	***Conformers, shielding tensor σ***															***σ Boltz. avrg.***	***Comp. avrg. δ, ppm***	***Δδ, ppm***
		**42**	**63**	**5**	**8**	**75**	**160**	**44**	**…**	**73**	**53**	**94**	**191**	**79**	**88**	**68**			
H(3)	7.21	24.93	24.83	24.77	24.80	24.71	24.75	24.53	…	24.60	24.65	24.73	24.74	24.62	24.64	24.62	24.77	7.37	0.16
H(4)	6.62	25.46	25.45	25.29	25.19	25.27	25.15	25.11	…	25.38	25.28	25.37	25.28	25.39	25.39	25.39	25.33	6.82	0.20
H(5)	5.98	25.82	25.80	25.87	25.90	25.85	25.90	25.67	…	25.67	25.70	25.62	25.64	25.63	25.63	25.62	25.77	6.37	0.39
H(6a, H_{Si})	2.50	29.74	29.72	29.74	29.73	29.73	29.73	29.77	…	29.67	29.92	29.83	29.81	29.92	29.94	29.92	29.72	2.42	-0.08
H(6b, H_{Re})	2.71	29.21	29.19	29.19	29.20	29.19	29.21	29.15	…	29.36	29.29	29.32	29.33	29.29	29.31	29.28	29.23	2.92	0.21
H(7)	4.27	27.93	27.92	27.92	27.92	27.92	27.93	27.91	…	27.79	27.60	27.78	27.78	27.58	27.69	27.58	27.89	4.25	-0.02
H(9)	5.34	26.75	26.76	26.76	26.74	26.76	26.76	26.27	…	26.37	26.11	26.49	26.51	26.07	26.01	26.06	26.57	5.58	0.24
H(10)	2.61	29.01	28.99	28.99	29.00	29.06	29.05	29.35	…	29.07	29.14	28.90	28.89	29.15	29.19	29.14	29.03	3.11	0.50
H(11)	3.50	28.33	28.33	28.32	28.34	28.12	28.17	28.31	…	27.86	27.67	28.13	28.16	27.66	27.55	27.64	28.19	3.96	0.46
H(13)	5.31	25.85	25.82	25.82	25.84	25.84	25.82	26.92	…	26.07	26.32	25.67	25.70	26.31	26.33	26.30	25.95	6.20	0.89
H(15)	5.22	26.72	26.68	26.69	26.71	26.55	26.56	26.53	…	26.69	27.29	26.62	26.64	27.29	27.24	27.29	26.76	5.39	0.17
H(16a, H_{Si})	2.61	30.03	30.03	30.03	30.04	30.03	30.04	29.80	…	29.91	30.10	30.02	30.03	30.10	30.08	30.09	30.01	2.14	-0.47
H(16b, H_{Re})	2.34	29.41	29.41	29.41	29.40	29.44	29.42	29.75	…	29.37	29.49	29.38	29.37	29.49	29.49	29.49	29.42	2.73	0.39
H(17)	4.69	27.48	27.50	27.48	27.54	27.36	27.44	27.58	…	27.37	27.52	27.48	27.54	27.47	27.46	27.47	27.49	4.66	-0.03
H(18)	3.97	28.41	28.41	28.41	28.42	28.37	28.38	28.40	…	28.40	28.48	28.41	28.41	28.48	28.47	28.48	28.40	3.75	-0.22
H(19)-Me	1.16	31.11	31.09	31.30	31.10	31.09	31.22	31.08	…	31.32	31.12	31.31	31.08	31.14	31.17	31.18	31.16	0.99	-0.17
H(20a, H_{Re})	4.58[a]	28.18	28.16	27.72	28.05	27.69	28.01	27.91	…	27.73	27.98	27.71	28.04	27.77	27.77	27.77	27.94	4.21	-0.37
H(20, H_{Si})	4.40[a]	28.15	28.27	28.09	27.72	28.05	27.68	27.65	…	28.05	27.73	28.09	27.73	28.06	28.05	28.06	28.01	4.14	-0.26
H(21)-Me	1.68	30.07	30.79	30.79	30.06	30.08	30.80	30.78	…	30.85	30.53	30.90	30.48	30.78	30.13	30.09	30.45	1.70	0.02
H(22a, H_{Re})	2.15	30.55	30.54	30.53	30.55	30.26	30.26	30.16	…	30.29	30.27	30.16	30.18	30.28	30.37	30.28	30.37	1.78	-0.37
H(22b, H_{Si})	1.46	30.17	30.19	30.19	30.18	30.13	30.13	30.69	…	30.74	30.50	30.80	30.79	30.50	30.74	30.50	30.49	1.66	0.20
H(23)-Me	0.89	31.32	31.31	31.31	31.31	31.12	31.26	30.65	…	31.12	31.13	31.21	31.25	31.16	31.08	31.16	31.20	0.95	0.06
H(24)-Me	1.84	29.82	30.66	30.66	29.65	30.94	30.95	30.61	…	30.42	30.50	30.68	30.67	30.46	30.44	30.46	30.22	1.92	0.08
H(25)-Me	1.56	30.05	30.19	30.19	30.03	30.13	30.29	31.21	…	30.08	30.71	30.17	30.17	30.23	30.22	30.23	30.27	1.87	0.31
H(2")	7.30	25.34	25.29	25.29	25.32	24.71	24.73	24.56	…	25.06	24.79	24.95	24.95	24.86	24.85	24.88	25.04	7.11	-0.19
H(3")	7.10	25.14	25.13	25.13	25.13	25.02	25.04	24.88	…	25.11	24.92	24.98	24.98	25.02	25.03	25.04	25.05	7.09	-0.01
H(5")	7.10	25.00	25.00	25.01	25.00	24.93	24.92	25.04	…	25.00	25.09	25.11	25.11	24.99	24.97	24.99	25.01	7.14	0.04
H(6")	7.30	25.01	24.99	25.01	25.00	24.71	24.70	24.94	…	25.09	24.90	25.21	25.23	24.83	24.80	24.83	24.95	7.20	-0.10
H(7")-Me	2.28	30.37	29.78	30.34	30.37	30.06	29.73	30.08	…	29.88	29.73	30.35	29.73	29.71	29.73	30.32	29.95	2.19	-0.09
Energy (kJ/mol)		0.00	0.22	1.01	1.22	1.36	1.51	1.71	…	7.29	7.49	7.82	7.94	8.04	8.09	8.10	**DP4+ (^{1}H data)**		
Boltzmann factor		0.06	0.06	0.04	0.04	0.04	0.03	0.03	…	0.00	0.00	0.00	0.00	0.00	0.00	0.00	0.00%		

[a]H20a, H_{Re} and H20b, H_{Si} could not be unambiguously assigned and thus, the experimental chemical shift could be the other way round.

Table 6: DP4+ results for ***11S****: Computed σ for C-nuclei for each conformer and the averaged σ (conformers weighted based on Boltzmann factors) for simplified 11-desnoviosyl-11S-p-tolylsulfide fidaxomicin* ***5b-C(11)****. Computed chemical shifts δ were calculated using TMS as a reference (σ_C(TMS): 190.0976). (PCM, solvent: acetone/B3LYP/6-31G(d)). Total amount of conformers: 63.*

**C-atom**	***Exp. δ, ppm***	***Conformers, shielding tensor σ***															***σ Boltz. avrg.***	***Comp. avrg. δ, ppm***	***Δδ, ppm***
		**42**	**63**	**5**	**8**	**75**	**160**	**44**	**…**	**73**	**53**	**94**	**191**	**79**	**88**	**68**			
C(1)	167.6	26.6	26.5	25.9	26.7	25.8	26.6	27.0	…	26.0	26.9	25.7	26.4	26.2	26.1	26.2	26.4	163.7	-3.9
C(2)	125.2	69.9	70.2	69.7	70.0	69.7	70.0	71.1	…	71.2	71.8	70.8	71.0	71.7	71.5	71.7	70.4	119.7	-5.5
C(3)	145.5	48.0	45.7	46.1	46.5	45.9	46.4	46.6	…	44.9	45.7	45.5	45.6	44.3	44.7	44.3	46.3	143.8	-1.7
C(4)	128.1	66.2	65.6	64.9	64.9	65.0	64.8	65.6	…	67.8	67.4	67.1	67.3	67.4	67.1	67.4	66.0	124.1	-4.0
C(5)	143.6	47.3	47.0	47.5	47.6	47.3	47.6	46.4	…	44.0	43.8	44.1	44.0	43.5	43.9	43.5	46.5	143.6	0.0
C(6)	37.2	150.9	150.8	150.9	150.8	150.9	150.7	150.6	…	152.1	148.8	149.4	149.5	148.9	149.0	148.9	151.0	39.1	1.9
C(7)	72.9	117.0	117.0	117.1	117.0	117.0	117.1	117.0	…	115.6	116.7	116.6	116.8	116.6	116.4	116.6	116.8	73.3	0.4
C(8)	136.7	53.8	54.0	53.9	53.8	54.2	54.2	57.2	…	55.3	57.7	59.4	59.3	57.7	59.7	57.7	55.4	134.7	-2.0
C(9)	126.0	70.9	70.8	70.9	70.9	70.6	70.6	67.1	…	70.1	70.3	68.3	68.2	70.2	68.5	70.2	69.5	120.6	-5.4
C(10)	41.2	146.3	146.3	146.4	146.3	146.7	146.6	142.9	…	139.0	141.3	146.6	146.7	141.2	140.8	141.3	145.3	44.8	3.6
C(11)	66.7	116.6	116.2	116.4	116.3	113.5	113.1	118.3	…	129.7	119.9	123.3	123.1	119.9	126.1	120.2	119.5	70.6	3.9
C(12)	134.0	59.4	59.4	59.4	59.3	60.2	60.0	55.3	…	51.7	53.8	58.6	58.6	53.9	53.5	54.0	57.3	132.8	-1.2
C(13)	133.4	62.5	62.3	62.2	62.4	61.9	62.0	62.9	…	64.0	63.0	62.2	62.5	62.8	63.1	62.7	63.0	127.1	-6.3
C(14)	135.9	58.8	58.8	58.8	58.8	58.7	58.5	59.7	…	59.6	60.1	58.5	58.4	60.1	60.2	60.1	58.9	131.2	-4.7
C(15)	125.2	60.9	60.9	60.7	61.0	61.3	61.6	66.6	…	67.3	67.3	61.3	61.5	67.3	67.5	67.3	62.9	127.2	2.0
C(16)	28.3	156.8	156.7	156.8	156.7	156.8	156.6	157.2	…	156.0	156.3	156.6	156.5	156.4	156.4	156.4	156.7	33.4	5.1
C(17)	78.0	105.6	105.5	105.5	105.7	105.2	105.4	103.5	…	105.4	105.8	105.8	105.9	105.6	105.7	105.7	105.6	84.5	6.5
C(18)	67.5	121.1	121.2	121.2	121.1	121.2	121.1	120.3	…	120.5	120.5	121.1	121.1	120.5	120.5	120.5	121.0	69.1	1.6
C(19)	20.7	172.5	172.6	172.5	172.5	172.5	172.5	172.7	…	172.5	172.6	172.5	172.6	172.5	172.6	172.5	172.5	17.6	-3.1
C(20)	63.3	125.4	125.2	126.4	125.7	126.5	125.5	124.9	…	126.4	125.6	126.4	125.8	126.7	126.6	126.7	125.8	64.3	1.0
C(21)	15.1	174.6	174.6	174.6	174.6	174.8	174.7	175.7	…	175.7	174.6	175.3	175.2	174.6	175.7	174.6	174.9	15.2	0.1
C(22)	27.8	157.9	157.8	157.8	157.8	157.4	157.4	160.8	…	160.7	159.1	160.2	160.1	159.2	161.1	159.2	159.0	31.1	3.3
C(23)	10.8	177.0	177.0	177.0	177.0	176.8	176.8	177.5	…	177.7	176.7	177.6	177.7	176.7	177.5	176.7	177.3	12.8	2.0
C(24)	13.7	169.3	169.4	169.5	169.3	169.2	169.1	173.0	…	170.5	170.8	169.4	169.4	170.8	170.9	170.8	169.7	20.4	6.7
C(25)	17.4	172.8	172.9	172.9	172.8	172.9	172.7	173.5	…	172.4	172.5	172.7	172.7	172.5	172.4	172.5	172.7	17.3	-0.1
C(1")	132.9	55.7	55.6	55.7	55.6	60.0	59.8	61.4	…	55.4	60.1	55.9	55.8	60.0	60.2	59.9	56.9	133.2	0.3
C(2")	135.0	71.5	71.2	71.3	71.4	62.1	62.0	59.7	…	73.8	65.1	68.9	68.9	65.1	65.3	65.2	68.1	122.0	-13.0
C(3")	130.1	66.4	66.4	66.5	66.4	67.3	67.3	66.7	…	66.5	67.2	67.9	67.8	66.9	66.8	66.9	66.8	123.3	-6.8
C(4")	138.1	60.8	60.7	60.7	60.8	57.1	57.1	55.8	…	61.7	58.3	60.6	60.6	58.3	58.4	58.4	59.6	130.5	-7.6
C(5")	130.1	67.8	67.9	67.9	67.9	66.6	66.6	67.5	…	67.6	67.0	66.4	66.4	67.3	67.3	67.3	67.3	122.8	-7.3
C(6")	135.0	69.3	69.1	69.1	69.1	63.5	63.2	59.1	…	70.5	64.6	71.2	71.3	64.3	64.4	64.4	67.4	122.7	-12.3
C(7")	21.1	169.4	169.4	169.4	169.4	169.1	169.1	169.1	…	169.5	169.3	169.4	169.4	169.3	169.3	169.3	169.3	20.8	-0.3
Energy (kJ/mol)		0.00	0.22	1.01	1.22	1.36	1.51	1.71	…	7.29	7.49	7.82	7.94	8.04	8.09	8.10	**DP4+ (^{13}C data)**		
Boltzmann factor		0.06	0.06	0.04	0.04	0.04	0.03	0.03	…	0.00	0.00	0.00	0.00	0.00	0.00	0.00	0.00%		

11-Desnoviosyl-13-*p*-tolylsulfide fidaxomicin (**5b-C(13)**)

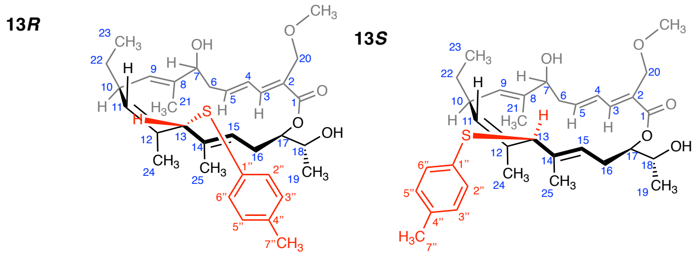

*Table 7: Experimental vs. computed ^{1}H chemical shifts and DP4+ results for simplified 11-desnoviosyl-13-p-tolylsulfide fidaxomicin **5b-C(13)** (PCM, solvent: acetone/B3LYP/6-31G(d))*

H-atom	Exp. δ, ppm	13*R*		13S	
		Comp. δ, ppm	Δδ, ppm	Comp. δ, ppm	Δδ, ppm
H(3)	7.20	7.25	0.05	7.31	0.11
H(4)	6.58	6.71	0.13	6.75	0.17
H(5)	6.02	6.39	0.37	6.36	0.34
H(6a, H_{Si})	2.42	2.34	-0.08	2.32	-0.10
H(6b, H_{Re})	2.69	2.92	0.23	2.88	0.19
H(7)	4.25	4.24	-0.01	4.19	-0.06
H(9)	5.22	5.33	0.11	5.21	-0.01
H(10)	2.87	3.39	0.52	3.19	0.32
H(11)	4.96	5.72	0.76	5.39	0.43
H(13)	4.13	4.14	0.01	4.19	0.06
H(15)	5.84	5.43	-0.41	6.04	0.20
H(16a, H_{Si})	2.61	2.07	-0.54	2.39	-0.22
H(16b, H_{Re})	2.53	2.02	-0.51	2.52	-0.01
H(17)	4.84	4.50	-0.34	4.62	-0.22
H(18)	3.93	3.88	-0.05	4.04	0.11
H(19)-Me	1.17	0.99	-0.18	1.07	-0.10
H(20a, H_{Re})	4.61[a]	4.15	-0.46	4.21	-0.40
H(20b, H_{Si})	4.48[a]	4.10	-0.38	4.21	-0.27
H(21)-Me	1.64	1.75	0.11	1.68	0.04
H(22a, H_{Re})	1.09	1.59	0.50	1.31	0.23
H(22b, H_{Si})	1.09	1.48	0.40	1.30	0.22
H(23)-Me	0.52	0.96	0.44	0.64	0.12
H(24)-Me	1.59	1.81	0.22	1.74	0.15
H(25)-Me	1.53	1.51	-0.02	1.54	0.01
H(2")	7.27	7.34	0.07	7.28	0.01
H(3")	7.12	7.17	0.05	7.16	0.04
H(5")	7.12	7.18	0.06	7.17	0.05
H(6")	7.27	7.35	0.08	7.29	0.02
H(7")-Me	2.28	2.24	-0.04	2.22	-0.06
**DP4+ (^{1}H data)**		**0.00%**		**100.00%**	
**DP4+ (all data)**		**0.00%**		**100.00%**	

[a]H20a, H_{Re} and H20b, H_{Si} could not be unambiguously assigned and thus, the experimental chemical shift could be the other way round.

13*R*

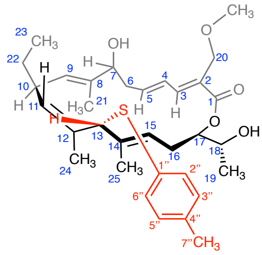

13*S*

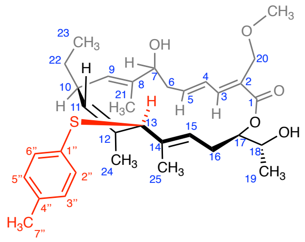

*Table 8: Experimental vs. computed ^{13}C chemical shifts and DP4+ results for simplified 11-desnoviosyl-13-p-tolylsulfide fidaxomicin **5b-C(13)** (PCM, solvent: acetone/B3LYP/6-31G(d))*

C-atom	Exp. δ, ppm	13*R*		13S	
		Comp. δ, ppm	Δδ, ppm	Comp. δ, ppm	Δδ, ppm
C(1)	166.7	159.9	-6.8	160.6	-6.1
C(2)	125.2	120.3	-4.9	120.1	-5.1
C(3)	145.0	141.4	-3.6	141.6	-3.4
C(4)	127.9	123.6	-4.3	123.5	-4.4
C(5)	143.5	142.6	-0.9	142.9	-0.6
C(6)	37.6	38.9	1.3	38.9	1.3
C(7)	72.8	72.8	0.0	72.7	-0.1
C(8)	133.9	130.5	-3.4	130.4	-3.5
C(9)	127.1	122.1	-5.0	122.2	-4.9
C(10)	39.1	40.7	1.6	40.3	1.2
C(11)	135.0	128.8	-6.2	130.8	-4.2
C(12)	133.3	131.5	-1.8	131.3	-2.0
C(13)	66.9	72.9	6.0	74.6	7.7
C(14)	137.2	134.3	-2.9	134.7	-2.5
C(15)	121.5	120.7	-0.8	119.2	-2.3
C(16)	29.1	30.9	1.8	31.3	2.2
C(17)	77.9	77.5	-0.4	79.1	1.2
C(18)	66.7	66.6	-0.1	67.2	0.5
C(19)	21.2	20.9	-0.3	20.6	-0.6
C(20)	62.6	64.1	1.5	64.0	1.4
C(21)	14.6	15.1	0.5	14.9	0.3
C(22)	29.7	31.4	1.7	31.3	1.6
C(23)	11.7	12.9	1.2	12.8	1.1
C(24)	12.0	17.8	5.8	12.7	0.7
C(25)	17.1	11.6	-5.5	16.7	-0.4
C(1'')	132.7	129.9	-2.8	130.9	-1.8
C(2'')	134.3	128.4	-5.9	126.4	-7.9
C(3'')	130.3	123.1	-7.2	122.9	-7.4
C(4'')	137.9	133.6	-4.3	132.7	-5.2
C(5'')	130.3	123.1	-7.2	123.3	-7.0
C(6'')	134.3	128.3	-6.0	126.0	-8.3
C(7'')	21.0	21.0	0.0	20.9	-0.1
**DP4+ (^{13}C data)**		**0.52%**		**99.48%**	
**DP4+ (all data)**		**0.00%**		**100.00%**	

*Table 9: DP4+ results for **13R**: Computed σ for H-nuclei for each conformer and the averaged σ (conformers weighted based on Boltzmann factors) for simplified 11-desnoviosyl-13R-p-tolylsulfide fidaxomicin **5b-C(13)**. Computed chemical shifts δ were calculated using TMS as a reference (σ_H(TMS): 32.148). (PCM, solvent: acetone/B3LYP/6-31G(d)). Total amount of conformers: 92.*

H-atom	***Exp. δ, ppm***	***Conformers, shielding tensor σ***															***σ Boltz. avrg.***	***Comp. avrg. δ, ppm***	***Δδ, ppm***
		**308**	**423**	**349**	**404**	**392**	**408**	**286**	**…**	**36**	**201**	**20**	**74**	**113**	**398**	**61**			
H(3)	7.20	24.96	24.97	24.90	24.96	24.91	24.96	24.90	…	24.82	24.94	24.84	24.77	24.75	24.88	24.77	24.90	7.25	0.05
H(4)	6.58	25.54	25.53	25.56	25.53	25.55	25.53	25.55	…	25.35	25.31	25.28	25.41	25.35	25.23	25.33	25.44	6.71	0.13
H(5)	6.02	25.75	25.75	25.75	25.75	25.76	25.75	25.76	…	25.77	25.84	25.81	25.56	25.47	25.83	25.60	25.75	6.39	0.37
H(6a, H_{Si})	2.42	29.85	29.84	29.85	29.84	29.85	29.84	29.85	…	29.83	29.72	29.85	29.67	29.43	29.70	29.68	29.81	2.34	-0.08
H(6b, H_{Re})	2.69	29.19	29.17	29.19	29.18	29.19	29.18	29.19	…	29.16	29.26	29.17	29.40	29.52	29.27	29.41	29.23	2.92	0.23
H(7)	4.25	27.97	27.90	27.97	27.91	27.97	27.91	27.97	…	27.88	27.95	27.89	27.77	27.82	27.95	27.78	27.91	4.24	-0.01
H(9)	5.22	26.80	26.88	26.84	26.92	26.83	26.92	26.82	…	26.86	26.69	26.91	26.69	26.66	26.66	26.72	26.82	5.33	0.11
H(10)	2.87	28.76	28.78	28.77	28.78	28.76	28.78	28.76	…	28.71	28.70	28.76	28.63	28.78	28.70	28.65	28.76	3.39	0.52
H(11)	4.96	26.54	26.51	26.51	26.48	26.52	26.48	26.54	…	25.96	26.39	26.00	25.89	25.99	26.35	25.92	26.43	5.72	0.76
H(13)	4.13	28.01	28.06	28.00	28.07	28.08	28.07	28.04	…	27.91	28.15	27.90	27.85	27.86	28.10	27.85	28.01	4.14	0.01
H(15)	5.84	26.78	26.79	26.76	26.81	26.85	26.82	26.80	…	26.12	27.12	26.15	26.07	26.22	27.10	26.09	26.72	5.43	-0.41
H(16a, H_{Si})	2.61	30.13	30.10	30.18	30.17	30.19	30.17	30.17	…	29.85	29.78	29.83	29.85	29.43	29.76	29.88	30.08	2.07	-0.54
H(16b, H_{Re})	2.53	30.17	30.16	30.13	30.15	30.17	30.15	30.15	…	29.71	30.40	29.72	29.68	29.86	30.42	29.69	30.12	2.02	-0.51
H(17)	4.84	27.66	27.69	27.67	27.67	27.66	27.67	27.67	…	27.57	27.37	27.66	27.50	27.38	27.39	27.53	27.65	4.50	-0.34
H(18)	3.93	28.31	28.31	28.30	28.31	28.32	28.31	28.31	…	28.18	28.35	28.17	28.16	28.14	28.36	28.15	28.27	3.88	-0.05
H(19)-Me	1.17	30.96	31.54	31.53	31.53	30.97	30.96	30.96	…	30.92	31.34	30.86	30.95	31.30	31.26	31.46	31.16	0.99	-0.18
H(20a, H_{Re})	4.61[a]	28.23	28.23	28.12	28.21	28.12	28.21	28.10	…	27.83	28.19	27.98	27.81	27.99	27.80	27.97	28.00	4.15	-0.46
H(20b, H_{Si})	4.48[a]	28.28	28.30	28.23	28.22	28.23	28.22	28.22	…	28.06	27.72	27.75	28.04	27.80	28.11	27.74	28.05	4.10	-0.38
H(21)-Me	1.64	30.79	30.41	29.99	29.96	29.98	30.76	29.99	…	29.91	30.84	29.93	30.80	30.90	30.47	30.44	30.40	1.75	0.11
H(22a, H_{Re})	1.09	30.58	30.53	30.59	30.54	30.58	30.54	30.59	…	30.48	30.55	30.53	30.59	30.69	30.53	30.60	30.56	1.59	0.50
H(22b, H_{Si})	1.09	30.65	30.67	30.66	30.68	30.66	30.68	30.66	…	30.62	30.63	30.66	30.68	30.75	30.63	30.70	30.67	1.48	0.40
H(23)-Me	0.52	31.02	31.17	31.29	31.18	31.28	31.28	31.04	…	31.11	31.12	31.23	31.31	31.38	31.27	31.33	31.19	0.96	0.44
H(24)-Me	1.59	30.08	30.46	30.08	30.42	30.44	30.42	30.45	…	30.58	30.05	29.97	30.27	30.12	30.51	30.28	30.33	1.81	0.22
H(25)-Me	1.53	30.76	30.31	30.88	30.79	30.89	30.79	30.30	…	30.54	30.24	30.73	30.48	30.79	30.44	30.63	30.63	1.51	-0.02
H(2'')	7.27	24.96	24.60	24.61	24.97	24.97	24.97	24.60	…	25.02	24.92	25.06	25.02	25.04	24.93	25.05	24.81	7.34	0.07
H(3'')	7.12	25.09	24.86	24.89	25.07	25.09	25.08	24.86	…	25.05	24.98	25.03	25.00	24.97	25.03	25.02	24.98	7.17	0.05
H(5'')	7.12	24.82	25.06	25.05	24.86	24.84	24.86	25.07	…	25.00	24.93	25.04	25.06	25.12	24.87	25.06	24.96	7.18	0.06
H(6'')	7.27	24.58	24.95	24.94	24.60	24.59	24.60	24.96	…	25.01	24.65	25.10	25.09	25.30	24.63	25.12	24.80	7.35	0.08
H(7'')-Me	2.28	30.26	30.10	29.70	29.89	29.70	29.88	29.70	…	30.28	30.20	29.73	29.73	29.78	29.70	29.74	29.91	2.24	-0.04
Energy (kJ/mol)		0.00	0.46	1.00	1.01	1.01	1.01	1.04	…	7.94	8.00	8.06	8.17	8.19	8.23	8.36	**DP4+ (^{1}H data)**		
Boltzmann factor		0.05	0.04	0.04	0.04	0.04	0.04	0.04	…	0.00	0.00	0.00	0.00	0.00	0.00	0.00	0.00%		

[a]H20a, H_{Re} and H20b, H_{Si} could not be unambiguously assigned and thus, the experimental chemical shift could be the other way round.

Table 10: DP4+ results for ***13R****: Computed σ for C-nuclei for each conformer and the averaged σ (conformers weighted based on Boltzmann factors) for simplified 11-desnoviosyl-13R-p-tolylsulfide fidaxomicin* ***5b-C(13)****. Computed chemical shifts δ were calculated using TMS as a reference (σ_C(TMS): 190.0976). (PCM, solvent: acetone/B3LYP/6-31G(d)). Total amount of conformers: 92.*

C-atom	***Exp. δ, ppm***	***Conformers, shielding tensor σ***															***σ Boltz. avrg.***	***Comp. avrg. δ, ppm***	***Δδ, ppm***
		**308**	**423**	**349**	**404**	**392**	**408**	**286**	**…**	**36**	**201**	**20**	**74**	**113**	**398**	**61**			
C(1)	166.7	30.7	30.7	30.9	30.8	30.8	30.8	30.8	…	30.0	25.6	30.8	29.9	26.1	26.5	30.7	30.2	159.9	-6.8
C(2)	125.2	69.8	69.9	69.8	69.9	69.8	69.9	69.8	…	69.5	69.2	69.7	70.7	71.2	70.1	70.8	69.8	120.3	-4.9
C(3)	145.0	49.1	48.9	48.6	49.5	48.6	49.4	48.5	…	48.2	48.2	49.5	47.3	46.3	47.0	48.3	48.7	141.4	-3.6
C(4)	127.9	66.6	66.6	66.3	66.8	66.2	66.8	66.1	…	65.8	65.3	65.6	68.0	69.1	65.8	67.5	66.5	123.6	-4.3
C(5)	143.5	47.9	47.8	47.3	47.6	47.4	47.6	47.4	…	47.8	47.9	48.5	44.9	44.9	47.0	45.4	47.5	142.6	-0.9
C(6)	37.6	151.1	150.9	151.2	151.1	151.2	151.0	151.1	…	151.0	151.3	151.2	152.6	152.2	151.4	152.5	151.2	38.9	1.3
C(7)	72.8	117.4	117.5	117.5	117.4	117.4	117.4	117.5	…	117.3	116.9	117.6	115.7	117.4	116.9	115.8	117.2	72.8	0.0
C(8)	133.9	60.7	58.4	60.5	58.3	60.6	58.3	60.5	…	58.6	60.8	58.5	59.3	59.4	61.0	59.1	59.6	130.5	-3.4
C(9)	127.1	67.4	68.7	67.5	68.8	67.5	68.8	67.5	…	68.4	66.8	68.6	66.8	68.7	66.6	66.8	68.0	122.1	-5.0
C(10)	145.0	149.1	149.4	149.1	149.4	149.1	149.4	149.1	…	149.9	148.9	150.0	149.7	149.4	149.0	149.7	149.4	40.7	1.6
C(11)	135.0	61.7	61.4	61.5	61.2	61.5	61.2	61.6	…	60.2	60.1	60.4	60.5	61.6	60.0	60.6	61.3	128.8	-6.2
C(12)	133.3	57.5	59.8	57.7	59.8	57.5	59.8	57.6	…	58.8	58.0	59.0	57.4	59.8	58.0	57.4	58.6	131.5	-1.8
C(13)	66.9	116.8	117.0	117.4	117.4	116.9	117.4	116.9	…	115.9	117.3	117.0	116.5	119.0	117.8	117.1	117.2	72.9	6.0
C(14)	137.2	55.9	56.0	55.7	56.0	56.0	56.0	55.9	…	54.0	55.8	53.9	54.2	55.0	55.8	54.0	55.8	134.3	-2.9
C(15)	121.5	69.2	69.3	69.4	69.4	69.4	69.4	69.3	…	69.8	70.2	69.9	69.6	70.4	70.4	69.7	69.4	120.7	-0.8
C(16)	29.1	159.2	159.3	159.3	159.3	159.2	159.3	159.3	…	159.1	156.7	159.8	158.9	156.4	156.5	159.2	159.1	30.9	1.8
C(17)	77.9	112.9	112.8	113.1	113.2	113.1	113.2	112.8	…	112.9	108.1	112.5	113.2	106.2	107.8	113.3	112.6	77.5	-0.4
C(18)	66.7	123.8	123.8	123.8	123.8	123.8	123.8	123.8	…	123.8	119.9	123.7	123.7	120.4	120.1	123.7	123.5	66.6	-0.1
C(19)	21.2	169.1	169.1	169.3	169.2	169.3	169.2	169.2	…	169.1	170.4	169.1	169.1	170.2	170.5	169.2	169.2	20.9	-0.3
C(20)	62.6	125.3	125.3	125.5	125.6	125.5	125.6	125.5	…	126.6	126.2	125.7	126.5	126.3	126.2	125.9	126.0	64.1	1.5
C(21)	14.6	175.2	174.7	175.2	174.6	175.1	174.6	175.1	…	174.7	174.9	174.7	175.3	175.7	174.8	175.3	175.0	15.1	0.5
C(22)	29.7	158.6	159.0	158.4	159.0	158.4	159.0	158.5	…	158.9	158.6	158.8	158.1	158.4	158.7	157.9	158.7	31.4	1.7
C(23)	11.7	176.9	177.5	176.9	177.5	176.9	177.5	176.9	…	177.5	176.8	177.5	176.6	176.7	176.9	176.6	177.2	12.9	1.2
C(24)	12.0	172.4	172.3	172.2	172.1	172.2	172.1	172.3	…	172.8	171.7	172.9	173.0	173.3	171.7	173.0	172.3	17.8	5.8
C(25)	17.1	178.8	178.9	178.6	178.6	178.6	178.6	178.6	…	176.6	178.5	176.6	176.8	177.0	178.3	176.7	178.5	11.6	-5.5
C(1")	132.7	60.6	60.5	60.9	60.6	60.7	60.6	60.6	…	55.5	60.7	55.6	55.4	55.9	60.8	55.5	60.2	129.9	-2.8
C(2")	134.3	60.9	60.8	60.8	60.7	60.5	60.6	60.8	…	69.9	59.9	68.7	68.8	69.9	60.0	69.0	61.7	128.4	-5.9
C(3")	130.3	67.2	66.7	66.7	67.3	67.2	67.3	66.7	…	66.4	67.6	67.9	67.7	67.6	67.4	67.7	67.0	123.1	-7.2
C(4")	137.9	56.1	56.0	56.0	56.0	56.0	56.0	56.0	…	59.5	55.6	60.0	60.1	61.0	55.7	60.3	56.5	133.6	-4.3
C(5")	130.3	66.8	67.3	67.3	66.8	66.7	66.8	67.3	…	67.9	66.6	66.6	66.4	66.4	66.8	66.5	67.0	123.1	-7.2
C(6")	134.3	61.0	60.8	60.9	60.7	60.8	60.7	60.8	…	67.9	60.2	70.6	70.7	72.3	60.5	71.0	61.8	128.3	-6.0
C(7")	21.0	169.1	169.1	169.1	169.1	169.1	169.1	169.1	…	169.4	169.1	169.4	169.4	169.5	169.0	169.4	169.1	21.0	0.0
Energy (kJ/mol)		0.00	0.46	1.00	1.01	1.01	1.01	1.04	…	7.94	8.00	8.06	8.17	8.19	8.23	8.36	**DP4+ (^{13}C data)**		
Boltzmann factor		0.05	0.04	0.04	0.04	0.04	0.04	0.04	…	0.00	0.00	0.00	0.00	0.00	0.00	0.00	0.52%		

*Table 11: DP4+ results for **13S**: Computed σ for H-nuclei for each conformer and the averaged σ (conformers weighted based on Boltzmann factors) for simplified 11-desnoviosyl-13S-p-tolylsulfide fidaxomicin **5b-C(13)**. Computed chemical shifts δ were calculated using TMS as a reference (σ_H(TMS): 32.148). (PCM, solvent: acetone/B3LYP/6-31G(d)). Total amount of conformers: 101.*

H-atom	***Exp. δ, ppm***	***Conformers, shielding tensor σ***															***σ Boltz. avrg.***	***Comp. avrg. δ, ppm***	***Δδ, ppm***
		**270**	**175**	**255**	**193**	**274**	**96**	**256**	**…**	**181**	**133**	**305**	**287**	**307**	**294**	**304**			
H(3)	7.20	24.90	24.89	24.80	24.85	24.79	24.82	24.79	…	24.71	24.80	24.86	24.76	24.86	24.76	24.86	24.84	7.31	0.11
H(4)	6.58	25.36	25.36	25.42	25.35	25.42	25.33	25.41	…	25.51	25.34	25.30	25.42	25.29	25.42	25.30	25.39	6.75	0.17
H(5)	6.02	25.91	25.90	25.83	25.89	25.83	25.87	25.83	…	25.73	25.82	25.87	25.62	25.87	25.62	25.87	25.79	6.36	0.34
H(6a, H_{Si})	2.42	29.83	29.83	29.90	29.91	29.89	29.91	29.89	…	29.80	29.86	29.89	29.69	29.89	29.69	29.89	29.82	2.32	-0.10
H(6b, H_{Re})	2.69	29.21	29.22	29.24	29.24	29.24	29.24	29.24	…	29.21	29.18	29.21	29.37	29.21	29.37	29.21	29.27	2.88	0.19
H(7)	4.25	27.95	27.99	28.02	28.04	28.02	28.04	28.02	…	27.96	27.90	28.00	27.73	28.00	27.73	28.00	27.96	4.19	-0.06
H(9)	5.22	27.00	26.91	26.98	27.02	26.99	27.03	26.99	…	26.93	26.93	26.90	26.88	26.90	26.88	26.90	26.94	5.21	-0.01
H(10)	2.87	28.80	28.81	29.11	29.13	29.09	29.12	29.09	…	28.87	28.85	28.87	28.79	28.87	28.79	28.87	28.96	3.19	0.32
H(11)	4.96	26.59	26.64	26.99	27.01	27.03	27.09	27.03	…	26.85	26.51	26.47	26.51	26.47	26.51	26.47	26.76	5.39	0.43
H(13)	4.13	27.83	27.59	27.96	27.99	28.02	28.09	28.03	…	27.97	27.96	27.92	27.97	27.91	27.98	27.92	27.96	4.19	0.06
H(15)	5.84	27.27	27.11	26.18	26.19	26.22	26.26	26.23	…	25.53	25.63	25.80	25.59	25.80	25.59	25.79	26.10	6.04	0.20
H(16a, H_{Si})	2.61	30.33	30.43	29.72	29.70	29.71	29.70	29.70	…	29.87	29.67	29.65	29.71	29.65	29.71	29.65	29.76	2.39	-0.22
H(16b, H_{Re})	2.53	29.77	29.82	29.59	29.62	29.59	29.61	29.59	…	29.17	29.59	29.69	29.50	29.70	29.49	29.69	29.63	2.52	-0.01
H(17)	4.84	27.62	27.59	27.57	27.65	27.57	27.63	27.58	…	27.40	27.42	27.52	27.51	27.52	27.51	27.52	27.53	4.62	-0.22
H(18)	3.93	28.59	28.61	28.05	28.05	28.05	28.05	28.05	…	28.45	27.90	27.95	28.10	27.95	28.10	27.94	28.10	4.04	0.11
H(19)-Me	1.17	31.17	31.18	31.44	30.82	31.44	30.82	31.44	…	31.41	31.07	30.92	31.46	30.92	31.46	30.91	31.08	1.07	-0.10
H(20a, H_{Re})	4.61[a]	28.01	28.00	27.85	27.97	27.84	27.97	27.84	…	28.16	27.81	27.93	27.80	27.92	27.80	27.92	27.94	4.21	-0.40
H(20b, H_{Si})	4.48[a]	27.80	27.79	28.05	27.78	28.05	27.77	28.05	…	28.28	28.00	27.74	28.02	27.74	28.03	27.74	27.94	4.21	-0.27
H(21)-Me	1.64	30.47	30.47	30.48	30.86	30.47	30.12	30.86	…	30.46	30.75	30.42	30.49	30.04	30.49	30.42	30.47	1.68	0.04
H(22a, H_{Re})	1.09	30.64	30.70	30.97	30.99	30.98	30.98	30.98	…	30.73	30.53	30.67	30.53	30.66	30.54	30.67	30.83	1.31	0.23
H(22b, H_{Si})	1.09	30.71	30.68	30.94	30.95	30.93	30.96	30.93	…	30.68	30.76	30.72	30.83	30.72	30.83	30.72	30.85	1.30	0.22
H(23)-Me	0.52	31.33	31.05	31.67	32.80	31.64	31.84	31.64	…	31.20	31.30	31.17	31.19	31.16	31.19	31.17	31.50	0.64	0.12
H(24)-Me	1.59	30.40	30.25	30.42	30.07	30.05	30.43	30.05	…	29.93	30.72	30.32	30.21	30.55	30.21	30.55	30.41	1.74	0.15
H(25)-Me	1.53	30.40	30.42	30.77	30.83	30.75	30.82	30.74	…	30.15	30.83	30.40	30.63	30.40	30.18	30.40	30.61	1.54	0.01
H(2")	7.27	24.96	24.90	24.61	24.81	24.82	24.60	24.85	…	25.01	25.04	25.04	24.99	25.04	25.00	25.05	24.87	7.28	0.01
H(3")	7.12	25.06	25.04	24.86	25.02	25.00	24.83	25.04	…	24.99	25.04	25.00	25.01	25.00	25.04	25.02	24.99	7.16	0.04
H(5")	7.12	24.85	24.88	25.00	24.85	24.86	25.04	24.83	…	25.05	25.08	25.13	25.09	25.14	25.06	25.11	24.97	7.17	0.05
H(6")	7.27	24.61	24.61	24.80	24.61	24.61	24.85	24.59	…	25.02	25.22	25.32	25.21	25.34	25.19	25.32	24.86	7.29	0.02
H(7")-Me	2.28	29.72	30.13	30.00	30.12	30.04	29.84	29.84	…	29.73	30.13	30.35	29.73	29.77	29.74	30.26	29.93	2.22	-0.06
Energy (kJ/mol)		0.00	0.23	1.44	1.54	1.71	1.79	1.79	…	7.96	8.15	8.27	8.29	8.29	8.32	8.32	**DP4+ (^{1}H data)**		
Boltzmann factor		0.07	0.06	0.04	0.04	0.03	0.03	0.03	…	0.00	0.00	0.00	0.00	0.00	0.00	0.00	100.00%		

[a]H20a, H_{Re} and H20b, H_{Si} could not be unambiguously assigned and thus, the experimental chemical shift could be the other way round.

*Table 12: DP4+ results for **13S**: Computed σ for C-nuclei for each conformer and the averaged σ (conformers weighted based on Boltzmann factors) for simplified 11-desnoviosyl-13S-p-tolylsulfide fidaxomicin **5b-C(13)**. Computed chemical shifts δ were calculated using TMS as a reference (σ_C(TMS): 190.0976). (PCM, solvent: acetone/B3LYP/6-31G(d)). Total amount of conformers: 101.*

**C-atom**	***Exp. δ, ppm***	***Conformers, shielding tensor σ***															***σ Boltz. avrg.***	***Comp. avrg. δ, ppm***	***Δδ, ppm***
		**270**	**175**	**255**	**193**	**274**	**96**	**256**	**…**	**181**	**133**	**305**	**287**	**307**	**294**	**304**			
C(1)	166.7	26.7	26.7	30.2	30.7	30.2	30.7	30.1	…	29.9	30.3	30.8	30.1	30.9	30.1	30.8	29.5	160.6	-6.1
C(2)	125.2	70.3	70.4	69.9	70.0	69.9	69.8	69.9	…	69.8	69.8	69.6	70.9	69.6	70.9	69.6	70.0	120.1	-5.1
C(3)	145.0	47.2	47.1	48.0	49.4	47.9	49.5	48.0	…	47.7	48.3	50.0	47.2	50.0	47.2	50.0	48.5	141.6	-3.4
C(4)	127.9	65.6	65.7	66.5	66.0	66.4	65.8	66.3	…	66.2	66.0	65.6	68.4	65.6	68.4	65.6	66.6	123.5	-4.4
C(5)	143.5	46.9	46.8	47.1	47.8	47.1	48.1	47.2	…	47.6	47.8	48.6	44.7	48.7	44.7	48.6	47.2	142.9	-0.6
C(6)	37.6	150.8	150.8	151.2	151.3	151.3	151.3	151.3	…	151.2	151.1	151.2	152.5	151.2	152.5	151.2	151.2	38.9	1.3
C(7)	72.8	117.2	117.3	117.4	117.7	117.4	117.7	117.4	…	117.2	117.5	117.6	115.8	117.6	115.8	117.7	117.4	72.7	-0.1
C(8)	133.9	57.5	60.3	60.6	60.6	60.6	60.8	60.5	…	55.8	58.4	60.4	57.5	60.3	57.5	60.3	59.7	130.4	-3.5
C(9)	127.1	68.6	67.8	67.1	67.2	67.1	67.1	67.1	…	66.5	68.8	67.5	69.4	67.5	69.4	67.5	67.9	122.2	-4.9
C(10)	145.0	149.6	149.6	149.5	149.5	149.5	149.7	149.5	…	149.4	150.3	149.8	150.6	149.8	150.6	149.8	149.8	40.3	1.2
C(11)	135.0	59.8	60.1	58.8	59.0	59.2	59.3	59.2	…	61.8	60.0	59.7	60.1	59.5	60.1	59.6	59.3	130.8	-4.2
C(12)	133.3	62.1	59.8	58.5	58.7	58.3	58.0	58.2	…	62.2	58.7	57.0	58.6	57.1	58.6	57.1	58.8	131.3	-2.0
C(13)	66.9	110.0	111.3	117.0	117.0	116.2	116.1	116.1	…	122.3	116.4	116.9	115.5	117.2	115.4	117.1	115.5	74.6	7.7
C(14)	137.2	58.3	58.3	54.7	54.3	54.5	54.2	54.4	…	58.0	55.3	54.4	54.7	54.5	54.6	54.4	55.4	134.7	-2.5
C(15)	121.5	65.9	65.3	71.9	71.8	72.1	72.4	72.1	…	69.1	70.8	72.2	71.0	72.2	70.9	72.2	70.9	119.2	-2.3
C(16)	29.1	157.3	157.3	159.1	159.7	159.1	159.8	159.1	…	159.8	159.4	160.6	159.2	160.6	159.2	160.6	158.8	31.3	2.2
C(17)	77.9	105.7	105.7	112.6	112.2	112.6	112.2	112.5	…	114.3	113.3	112.5	113.0	112.5	112.9	112.4	111.0	79.1	1.2
C(18)	66.7	120.4	120.4	123.7	123.7	123.7	123.7	123.7	…	123.5	123.6	123.8	123.6	123.8	123.6	123.8	122.9	67.2	0.5
C(19)	21.2	172.6	172.6	169.0	168.9	169.0	168.9	169.0	…	169.8	168.2	170.7	169.2	170.7	169.2	170.7	169.5	20.6	-0.6
C(20)	62.6	126.5	126.5	126.8	125.8	126.8	125.8	126.7	…	125.0	126.5	125.5	126.6	125.5	126.6	125.5	126.1	64.0	1.4
C(21)	14.6	174.8	175.1	175.4	175.5	175.4	175.6	175.4	…	175.0	174.7	175.4	174.6	175.4	174.6	175.4	175.2	14.9	0.3
C(22)	29.7	159.2	159.0	158.8	158.7	158.8	158.7	158.8	…	158.6	159.2	158.6	159.0	158.6	159.0	158.6	158.8	31.3	1.6
C(23)	11.7	177.4	177.5	177.5	177.6	177.4	177.4	177.4	…	176.8	177.6	176.9	177.7	176.9	177.7	176.9	177.3	12.8	1.1
C(24)	12.0	174.9	174.8	178.9	179.1	178.8	178.6	178.7	…	172.1	176.7	176.7	176.8	176.6	176.8	176.7	177.4	12.7	0.7
C(25)	17.1	174.7	174.8	173.2	173.0	173.2	173.2	173.2	…	173.4	173.3	173.4	173.1	173.4	173.1	173.4	173.4	16.7	-0.4
C(1'')	132.7	61.7	61.8	61.0	61.1	60.7	60.8	60.6	…	55.1	55.7	55.9	55.5	56.0	55.5	55.9	59.2	130.9	-1.8
C(2'')	134.3	60.2	60.8	61.3	61.3	61.1	60.8	61.2	…	69.2	68.9	69.1	68.4	69.2	68.3	69.2	63.7	126.4	-7.9
C(3'')	130.3	67.0	67.1	66.6	67.0	67.1	66.7	67.0	…	67.7	67.8	67.8	67.9	67.8	67.9	67.8	67.2	122.9	-7.4
C(4'')	137.9	55.8	55.9	56.1	56.1	56.1	55.9	56.2	…	60.2	60.2	60.4	59.9	60.5	59.8	60.4	57.4	132.7	-5.2
C(5'')	130.3	66.9	66.9	67.0	66.6	66.6	66.9	66.7	…	66.6	66.6	66.5	66.5	66.5	66.5	66.6	66.8	123.3	-7.0
C(6'')	134.3	60.0	60.3	61.2	61.3	61.3	60.7	61.3	…	70.8	70.6	71.2	70.3	71.3	70.2	71.2	64.1	126.0	-8.3
C(7'')	21.0	169.2	169.1	169.1	169.1	169.1	169.1	169.1	…	169.4	169.4	169.5	169.4	169.5	169.3	169.4	169.2	20.9	-0.1
Energy (kJ/mol)		0.00	0.23	1.44	1.54	1.71	1.79	1.79	…	7.96	8.15	8.27	8.29	8.29	8.32	8.32	**DP4+ (^{13}C data)**		
Boltzmann factor		0.07	0.06	0.04	0.04	0.03	0.03	0.03	…	0.00	0.00	0.00	0.00	0.00	0.00	0.00	99.48%		

11-Desnoviosyl-15-*p*-tolylsulfide fidaxomicin (**5b-C(15)**)

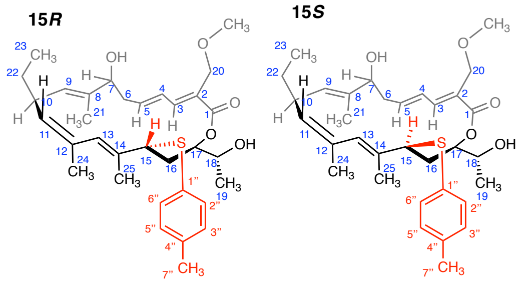

Table 13: Experimental vs. computed ^{1}H chemical shifts and DP4+ results for simplified 11-desnoviosyl-15-p-tolylsulfide fidaxomicin ***5b-C(15)*** *(PCM, solvent: acetone/B3LYP/6-31G(d))*

H-atom	Exp. δ, ppm	15*R*		15S	
		Comp. δ, ppm	Δδ, ppm	Comp. δ, ppm	Δδ, ppm
H(3)	7.04	7.14	0.10	7.02	-0.02
H(4)	6.30	6.64	0.34	6.29	-0.01
H(5)	6.14	6.34	0.20	6.64	0.50
H(6a, H_{Si})	2.55	2.37	-0.18	2.69	0.14
H(6b, H_{Re})	2.37	2.76	0.39	2.40	0.03
H(7)	4.26	4.25	-0.01	4.32	0.06
H(9)	5.12	5.24	0.12	5.32	0.20
H(10)	2.99	3.25	0.26	3.13	0.14
H(11)	4.61	5.22	0.61	4.87	0.26
H(13)	5.06	5.41	0.35	5.18	0.12
H(15)	3.65	4.30	0.65	4.02	0.37
H(16a, H_{Si})	2.29	2.25	-0.04	2.15	-0.14
H(16b, H_{Re})	1.96	2.20	0.24	1.45	-0.51
H(17)	5.11	4.67	-0.44	5.16	0.05
H(18)	3.83	4.16	0.33	3.63	-0.20
H(19)-Me	1.15	1.07	-0.08	1.13	-0.02
H(20a, H_{Re})	4.42[a]	4.12	-0.30	3.92	-0.50
H(20b, H_{Si})	4.59[a]	4.15	-0.44	4.58	-0.01
H(21)-Me	1.55	1.66	0.11	1.51	-0.04
H(22a, H_{Re})	1.22	1.47	0.25	1.30	0.08
H(22b, H_{Si})	1.22	1.44	0.22	1.34	0.12
H(23)-Me	0.76	0.84	0.08	0.76	0.00
H(24)-Me	1.63	1.46	-0.17	1.74	0.11
H(25)-Me	1.86	1.67	-0.19	1.89	0.03
H(2")	7.22	7.45	0.23	7.36	0.14
H(3")	7.10	7.23	0.13	7.20	0.10
H(5")	7.10	7.19	0.09	7.21	0.11
H(6")	7.22	7.38	0.16	7.44	0.22
H(7")-Me	2.28	2.24	-0.04	2.25	-0.03
**DP4+ (^{1}H data)**		**0.00%**		**100.00%**	
**DP4+ (all data)**		**0.00%**		**100.00%**	

[a]H20a, H_{Re} and H20b, H_{Si} could not be unambiguously assigned and thus, the experimental chemical shift could be the other way round.

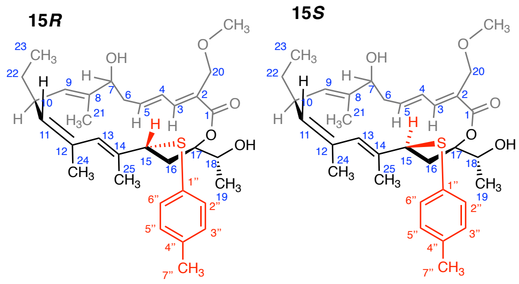

*Table 14: Experimental vs. computed ^{13}C chemical shifts and DP4+ results for simplified 11-desnoviosyl-15-p-tolylsulfide fidaxomicin **5b-C(15)** (PCM, solvent: acetone/B3LYP/6-31G(d))*

C-atom	Exp. δ, ppm	15*R*		15S	
		Comp. δ, ppm	Δδ, ppm	Comp. δ, ppm	Δδ, ppm
C(1)	167.6	159.7	-7.9	162.61	-4.99
C(2)	125.2	120.1	-5.1	118.96	-6.24
C(3)	144.8	141.2	-3.6	142.01	-2.79
C(4)	127.0	123.1	-3.9	119.18	-7.82
C(5)	143.1	143.2	0.1	144.95	1.85
C(6)	38.4	38.7	0.3	37.78	-0.62
C(7)	73.7	73.5	-0.2	74.05	0.35
C(8)	133.8	130.0	-3.8	132.09	-1.71
C(9)	126.9	123.1	-3.8	121.54	-5.36
C(10)	39.4	41.0	1.6	40.32	0.92
C(11)	137.2	130.7	-6.5	134.59	-2.61
C(12)	131.9	127.3	-4.6	128.51	-3.39
C(13)	134.2	127.0	-7.2	129.76	-4.44
C(14)	131.3	132.6	1.3	129.54	-1.76
C(15)	60.1	65.2	5.1	67.48	7.38
C(16)	31.1	32.9	1.8	32.08	0.98
C(17)	77.9	76.3	-1.6	76.28	-1.62
C(18)	69.7	65.9	-3.8	68.01	-1.69
C(19)	19.1	21.2	2.1	17.65	-1.45
C(20)	61.9	64.3	2.4	64.94	3.04
C(21)	15.4	15.2	-0.2	15.53	0.13
C(22)	29.6	31.3	1.7	31.14	1.54
C(23)	11.9	13.0	1.1	12.59	0.69
C(24)	17.0	18.4	1.4	17.14	0.14
C(25)	13.9	15.2	1.3	15.39	1.49
C(1")	132.1	128.7	-3.4	128.46	-3.64
C(2")	135.4	129.4	-6.0	129.77	-5.63
C(3")	130.1	123.1	-7.0	122.74	-7.36
C(4")	138.4	134.1	-4.3	134.33	-4.07
C(5")	130.1	122.9	-7.2	123.16	-6.94
C(6")	135.4	129.2	-6.2	129.82	-5.58
C(7")	21.1	21.0	-0.1	21.00	-0.10
**DP4+ (^{13}C data)**		**99.98%**		**0.02%**	
**DP4+ (all data)**		**0.00%**		**100.00%**	

*Table 15: DP4+ results for **15R**: Computed σ for H-nuclei for each conformer and the averaged σ (conformers weighted based on Boltzmann factors) for simplified 11-desnoviosyl-15R-p-tolylsulfide fidaxomicin **5b-C(15)**. Computed chemical shifts δ were calculated using TMS as a reference (σ_H(TMS): 32.148). (PCM, solvent: acetone/B3LYP/6-31G(d)). Total amount of conformers: 98.*

**H-atom**	**Exp. δ, ppm**	**Conformers, shielding tensor σ**															**σ Boltz. avrg.**	**Comp. avrg. δ, ppm**	**Δδ, ppm**
		**434**	**344**	**329**	**320**	**66**	**299**	**167**	**…**	**290**	**284**	**317**	**61**	**386**	**45**	**346**			
H(3)	7.04	25.10	25.09	25.06	25.05	25.03	25.05	25.00	…	24.98	24.85	24.92	24.76	24.91	24.79	24.97	25.01	7.14	0.10
H(4)	6.30	25.56	25.56	25.57	25.59	25.37	25.58	25.41	…	25.36	25.75	25.79	25.54	25.43	25.52	25.47	25.51	6.64	0.34
H(5)	6.14	25.85	25.84	25.85	25.89	25.96	25.88	25.92	…	25.86	25.42	25.45	25.50	25.91	25.49	26.01	25.81	6.34	0.20
H(6a, H_{Si})	2.55	29.89	29.88	29.88	29.89	29.89	29.87	29.89	…	29.85	29.36	29.61	29.36	30.00	29.37	29.89	29.77	2.37	-0.18
H(6b, H_{Re})	2.37	29.27	29.25	29.25	29.26	29.26	29.24	29.26	…	29.22	29.81	29.71	29.83	29.43	29.81	29.30	29.39	2.76	0.39
H(7)	4.26	27.99	27.94	27.98	27.99	27.99	27.93	27.98	…	27.94	27.72	27.74	27.76	27.73	27.77	27.98	27.90	4.25	-0.01
H(9)	5.12	26.99	27.07	27.01	27.01	26.99	27.04	26.96	…	26.85	26.79	26.66	26.81	26.65	26.79	27.00	26.91	5.24	0.12
H(10)	2.99	28.94	28.95	28.92	28.94	28.92	28.89	28.91	…	28.70	28.86	28.99	28.96	28.87	28.97	28.96	28.90	3.25	0.26
H(11)	4.61	27.00	27.04	26.99	27.05	26.96	26.83	26.93	…	26.64	27.01	27.11	27.06	26.87	27.08	27.00	26.93	5.22	0.61
H(13)	5.06	26.69	26.66	26.69	26.68	26.73	26.80	26.71	…	25.85	26.09	27.21	27.27	27.02	27.26	26.96	26.74	5.41	0.35
H(15)	3.65	27.78	27.76	27.78	27.76	27.83	27.93	27.81	…	27.23	27.94	28.20	28.28	27.96	28.24	27.82	27.85	4.30	0.65
H(16a, H_{Si})	2.29	29.86	29.88	29.87	29.89	29.87	29.79	29.87	…	29.87	30.05	29.97	29.98	29.79	30.00	29.90	29.90	2.25	-0.04
H(16b, H_{Re})	1.96	29.97	29.95	29.99	30.01	30.01	29.97	29.96	…	29.95	29.54	29.63	29.68	30.05	29.67	30.01	29.95	2.20	0.24
H(17)	5.11	27.45	27.48	27.48	27.48	27.49	27.48	27.50	…	27.33	27.95	27.73	27.72	27.45	27.73	27.40	27.48	4.67	-0.44
H(18)	3.83	28.02	28.01	28.01	28.01	28.02	28.04	28.02	…	27.69	28.69	27.90	27.89	28.11	27.90	27.85	27.99	4.16	0.33
H(19)-Me	1.15	30.94	30.89	31.47	31.47	31.48	31.47	30.90	…	30.98	31.15	30.80	30.56	30.90	30.54	31.30	31.08	1.07	-0.08
H(20a, H_{Re})	4.42[a]	28.22	28.23	28.13	28.04	27.95	28.04	27.82	…	27.82	28.29	28.34	28.08	27.79	28.03	27.77	28.03	4.12	-0.30
H(20b, H_{Si})	4.59[a]	28.19	28.19	28.21	28.20	27.75	28.20	28.01	…	28.02	27.82	27.90	28.13	27.97	28.12	27.99	27.99	4.15	-0.44
H(21)-Me	1.55	30.47	30.47	30.47	30.47	30.04	30.80	30.04	…	30.80	30.86	30.28	30.74	30.61	30.26	30.62	30.49	1.66	0.11
H(22a, H_{Re})	1.22	30.69	30.73	30.69	30.71	30.68	30.64	30.67	…	30.57	30.67	30.74	30.75	30.67	30.75	30.73	30.68	1.47	0.25
H(22b, H_{Si})	1.22	30.74	30.75	30.74	30.75	30.73	30.69	30.72	…	30.63	30.68	30.67	30.71	30.66	30.71	30.61	30.71	1.44	0.22
H(23)-Me	0.76	31.23	31.39	31.22	31.23	31.48	31.21	31.48	…	31.15	31.28	31.22	31.28	31.21	31.28	31.21	31.31	0.84	0.08
H(24)-Me	1.63	30.26	30.65	30.88	30.65	30.65	30.21	30.89	…	30.47	30.60	31.25	30.40	31.06	31.50	30.39	30.69	1.46	-0.17
H(25)-Me	1.86	30.56	30.59	30.73	30.74	30.38	30.73	30.31	…	30.24	30.35	30.46	30.49	30.40	30.49	30.11	30.48	1.67	-0.19
H(2'')	7.22	24.80	24.62	24.59	24.62	24.61	24.57	24.77	…	25.17	24.45	24.46	24.48	24.52	25.04	24.97	24.70	7.45	0.23
H(3'')	7.10	25.04	24.89	24.87	24.90	24.91	24.86	24.96	…	24.98	24.82	24.81	24.84	24.84	25.01	25.02	24.92	7.23	0.13
H(5'')	7.10	24.84	25.01	25.02	24.99	24.97	25.04	24.91	…	25.05	24.89	25.03	25.01	24.98	24.85	24.87	24.96	7.19	0.09
H(6'')	7.22	24.57	24.80	24.81	24.78	24.78	24.88	24.61	…	24.76	24.76	25.05	25.04	24.99	24.49	24.55	24.77	7.38	0.16
H(7'')-Me	2.28	30.23	29.70	30.08	30.00	30.14	29.93	29.71	…	29.75	29.63	30.14	30.01	29.95	29.69	29.98	29.91	2.24	-0.04
Energy (kJ/mol)		0.00	0.70	0.92	0.96	1.38	1.53	1.54	…	8.03	8.03	8.05	8.12	8.27	8.28	8.31	**DP4+ (^{1}H data)**		
Boltzmann factor		0.06	0.05	0.04	0.04	0.03	0.03	0.03	…	0.00	0.00	0.00	0.00	0.00	0.00	0.00	0.00%		

[a]H20a, H_{Re} and H20b, H_{Si} could not be unambiguously assigned and thus, the experimental chemical shift could be the other way round.

*Table 16: DP4+ results for **15R**: Computed σ for C-nuclei for each conformer and the averaged σ (conformers weighted based on Boltzmann factors) for simplified 11-desnoviosyl-15R-p-tolylsulfide fidaxomicin **5b-C(15)**. Computed chemical shifts δ were calculated using TMS as a reference (σ_C(TMS): 190.0976). (PCM, solvent: acetone/B3LYP/6-31G(d)). Total amount of conformers: 98.*

C-atom	Exp. δ, ppm	Conformers, shielding tensor σ															σ Boltz. avrg.	Comp. avrg. δ, ppm	Δδ, ppm
		434	344	329	320	66	299	167	…	290	284	317	61	386	45	346			
C(1)	167.6	30.7	30.7	30.6	30.8	30.7	30.9	30.3	…	30.4	29.7	29.4	30.0	30.1	29.8	30.3	30.4	159.7	-7.9
C(2)	125.2	69.7	69.7	69.8	69.7	69.4	69.7	69.5	…	69.4	72.0	71.5	69.7	71.0	69.7	69.9	70.0	120.1	-5.1
C(3)	144.8	49.5	49.4	48.9	48.8	49.7	48.8	48.7	…	48.6	48.2	48.4	47.3	48.2	47.4	48.8	48.9	141.2	-3.6
C(4)	127.0	66.6	66.6	66.5	66.0	65.6	66.0	66.1	…	66.0	68.3	69.2	66.4	67.6	66.6	67.5	67.0	123.1	-3.9
C(5)	143.1	47.8	47.9	47.2	47.6	48.3	47.6	47.9	…	47.9	46.5	46.2	46.4	45.2	46.3	47.1	46.9	143.2	0.1
C(6)	38.4	151.1	151.1	151.1	151.1	151.0	151.0	151.1	…	150.8	151.7	148.8	151.6	149.6	151.5	151.7	151.4	38.7	0.3
C(7)	73.7	117.3	117.3	117.3	117.4	117.4	117.3	117.3	…	117.0	114.8	116.2	114.7	116.3	115.0	117.3	116.6	73.5	-0.2
C(8)	133.8	62.2	59.9	62.2	62.2	62.1	59.7	62.2	…	61.8	55.4	59.0	55.5	60.4	55.3	60.8	60.1	130.0	-3.8
C(9)	126.9	67.3	68.1	67.3	67.4	67.4	68.1	67.3	…	67.2	66.2	65.9	66.1	65.5	66.3	66.0	67.0	123.1	-3.8
C(10)	39.4	149.1	149.3	149.1	149.1	149.0	149.2	149.0	…	148.7	148.9	149.0	149.0	149.0	149.1	149.3	149.1	41.0	1.6
C(11)	137.2	59.1	59.3	59.2	59.2	59.1	59.2	59.1	…	57.8	59.2	59.8	59.9	59.5	59.9	61.6	59.4	130.7	-6.5
C(12)	131.9	61.7	63.6	61.8	61.7	61.8	63.7	61.8	…	61.5	64.5	65.1	64.6	64.8	64.7	63.3	62.8	127.3	-4.6
C(13)	134.2	62.5	62.5	62.8	62.5	62.9	63.5	62.8	…	61.3	62.1	63.9	63.7	64.0	63.7	64.7	63.1	127.0	-7.2
C(14)	131.3	57.1	57.0	57.1	57.1	57.2	57.0	57.2	…	58.1	56.4	57.7	58.1	58.7	58.0	58.1	57.5	132.6	1.3
C(15)	60.1	125.1	125.4	125.0	125.3	124.8	123.6	124.7	…	133.0	124.4	125.4	125.3	122.9	125.6	122.8	124.8	65.2	5.1
C(16)	31.1	157.4	157.5	157.4	157.4	157.3	156.8	157.3	…	157.5	159.5	160.5	160.8	155.3	160.8	155.8	157.2	32.9	1.8
C(17)	77.9	113.8	113.5	113.6	113.6	113.8	113.8	113.7	…	114.6	113.7	112.1	112.3	114.0	112.2	114.0	113.8	76.3	-1.6
C(18)	69.7	124.5	124.5	124.5	124.5	124.5	124.5	124.5	…	124.1	124.5	124.2	124.3	123.9	124.4	123.9	124.2	65.9	-3.8
C(19)	19.1	169.0	168.9	168.9	168.9	168.9	169.0	168.9	…	168.1	169.8	169.4	169.1	169.1	169.0	168.0	168.9	21.2	2.1
C(20)	61.9	125.2	125.2	125.4	125.1	125.9	125.1	126.7	…	126.5	126.6	126.6	126.5	126.3	126.4	126.5	125.8	64.3	2.4
C(21)	15.4	174.9	174.6	174.9	174.9	174.9	174.6	174.9	…	174.7	174.6	174.6	174.7	175.0	174.7	175.3	174.9	15.2	-0.2
C(22)	29.6	159.4	159.6	159.3	159.3	159.3	159.5	159.3	…	159.3	157.4	157.4	157.5	157.8	157.5	158.1	158.8	31.3	1.7
C(23)	11.9	177.0	177.3	177.1	177.1	177.0	177.3	177.0	…	176.8	177.0	176.8	176.9	176.8	176.9	177.5	177.1	13.0	1.1
C(24)	17.0	171.6	171.6	171.5	171.6	171.5	171.4	171.5	…	171.4	171.9	172.4	172.4	172.1	172.5	170.8	171.7	18.4	1.4
C(25)	13.9	175.2	175.3	175.0	175.1	175.1	175.0	174.9	…	174.4	173.3	174.9	175.1	174.7	175.0	174.0	174.9	15.2	1.3
C(1'')	132.1	61.9	61.9	61.8	61.9	61.9	61.6	61.9	…	58.2	61.7	60.9	60.9	61.1	61.0	61.4	61.4	128.7	-3.4
C(2'')	135.4	60.8	60.5	60.2	60.3	60.2	60.0	60.7	…	69.9	60.1	59.6	59.6	59.7	59.7	59.9	60.7	129.4	-6.0
C(3'')	130.1	67.4	66.7	66.8	66.7	66.6	66.7	67.7	…	67.1	66.2	66.8	66.7	66.7	67.6	67.5	67.0	123.1	-7.0
C(4'')	138.4	55.7	56.0	55.7	55.8	55.7	55.7	55.7	…	61.1	55.6	55.5	55.5	55.6	55.5	55.7	56.0	134.1	-4.3
C(5'')	130.1	66.9	67.3	67.6	67.6	67.7	67.3	66.6	…	66.6	67.1	67.6	67.7	67.6	66.8	66.8	67.2	122.9	-7.2
C(6'')	135.4	60.3	61.2	60.9	60.9	60.7	60.6	60.1	…	71.6	60.5	59.6	59.6	59.5	59.7	59.9	60.9	129.2	-6.2
C(7'')	21.1	169.1	169.1	169.1	169.1	169.1	169.1	169.1	…	169.5	169.1	169.1	169.1	169.0	169.1	169.1	169.1	21.0	-0.1
**Energy (kJ/mol)**		0.00	0.70	0.92	0.96	1.38	1.53	1.54	…	8.03	8.03	8.05	8.12	8.27	8.28	8.31	**DP4+ (^{13}C data)**		
**Boltzmann factor**		0.06	0.05	0.04	0.04	0.03	0.03	0.03	…	0.00	0.00	0.00	0.00	0.00	0.00	0.00	99.98%		

*Table 17: DP4+ results for **15S**: Computed σ for H-nuclei for each conformer and the averaged σ (conformers weighted based on Boltzmann factors) for simplified 11-desnoviosyl-15S-p-tolylsulfide fidaxomicin **5b-C(15)**. Computed chemical shifts δ were calculated using TMS as a reference (σ_H(TMS): 32.148). (PCM, solvent: acetone/B3LYP/6-31G(d)).*

H-atom	***Exp. δ, ppm***	***Conformers, shielding tensor σ***					***σ Boltz. avrg.***	***Comp. avrg. δ, ppm***	***Δδ, ppm***
		**93**	**129**	**90**	**55**	**257**			
H(3)	7.04	25.17	25.07	25.07	24.99	25.23	25.13	7.02	-0.02
H(4)	6.30	25.89	25.84	25.84	25.75	25.81	25.86	6.29	-0.01
H(5)	6.14	25.54	25.45	25.45	25.51	25.85	25.51	6.64	0.50
H(6a, H_{Si})	2.55	29.47	29.42	29.43	29.44	29.59	29.46	2.69	0.14
H(6b, H_{Re})	2.37	29.78	29.71	29.71	29.78	29.66	29.75	2.40	0.03
H(7)	4.26	27.85	27.77	27.80	27.81	28.00	27.83	4.32	0.06
H(9)	5.12	26.87	26.77	26.67	27.04	27.15	26.83	5.32	0.20
H(10)	2.99	29.08	28.90	28.88	29.11	29.14	29.01	3.13	0.14
H(11)	4.61	27.54	26.81	26.88	27.46	27.50	27.28	4.87	0.26
H(13)	5.06	27.36	26.32	26.27	27.36	27.32	26.97	5.18	0.12
H(15)	3.65	28.24	27.92	27.95	28.33	28.19	28.13	4.02	0.37
H(16a, H_{Si})	2.29	30.01	30.00	29.99	30.02	30.01	30.00	2.15	-0.14
H(16b, H_{Re})	1.96	30.62	30.92	30.90	29.96	30.64	30.70	1.45	-0.51
H(17)	5.11	26.80	27.28	27.29	27.11	26.78	26.99	5.16	0.05
H(18)	3.83	28.40	28.73	28.72	28.46	28.42	28.52	3.63	-0.20
H(19)-Me	1.15	30.93	31.01	30.99	30.80	31.09	31.02	1.13	-0.02
H(20a, H_{Re})	4.42[a]	28.24	28.21	28.21	28.28	28.23	28.23	3.92	-0.50
H(20b, H_{Si})	4.59[a]	27.56	27.56	27.56	27.63	27.58	27.57	4.58	-0.01
H(21)-Me	1.55	30.85	30.78	30.88	30.93	30.90	30.64	1.51	-0.04
H(22a, H_{Re})	1.22	30.94	30.70	30.67	31.01	31.00	30.85	1.30	0.08
H(22b, H_{Si})	1.22	30.84	30.72	30.71	31.02	30.93	30.80	1.34	0.12
H(23)-Me	0.76	31.54	31.42	31.18	31.34	31.49	31.39	0.76	0.00
H(24)-Me	1.63	30.58	30.15	30.48	30.50	30.55	30.41	1.74	0.11
H(25)-Me	1.86	30.28	29.98	29.97	30.34	30.16	30.26	1.89	0.03
H(2'')	7.22	24.99	24.50	24.48	25.01	24.58	24.79	7.36	0.14
H(3'')	7.10	25.05	24.79	24.78	25.11	24.88	24.95	7.20	0.10
H(5'')	7.10	24.89	25.03	25.03	24.87	25.08	24.94	7.21	0.11
H(6'')	7.22	24.58	24.90	24.89	24.61	25.00	24.71	7.44	0.22
H(7'')-Me	2.28	29.70	30.22	30.24	29.81	29.94	29.90	2.25	-0.03
Energy (kJ/mol)		0.00	2.59	2.86	6.25	7.13	**DP4+ (^{1}H data)**		
Boltzmann factor		0.55	0.19	0.18	0.04	0.03	100.00%		

[a]H20a, H_{Re} and H20b, H_{Si} could not be unambiguously assigned and thus, the experimental chemical shift could be the other way round.

*Table 18: DP4+ results for **15S**: Computed σ for C-nuclei for each conformer and the averaged σ (conformers weighted based on Boltzmann factors) for simplified 11-desnoviosyl-15S-p-tolylsulfide fidaxomicin **5b-C(15)**. Computed chemical shifts δ were calculated using TMS as a reference (σ_C(TMS): 190.0976). (PCM, solvent: acetone/B3LYP/6-31G(d)).*

C-atom	Exp. δ, ppm	Conformers, shielding tensor σ					σ Boltz. avrg.	Comp. avrg. δ, ppm	Δδ, ppm
		93	129	90	55	257			
C(1)	167.6	27.5	27.3	27.4	28.6	27.3	27.48	162.61	-4.99
C(2)	125.2	71.2	71.0	71.1	72.1	70.3	71.14	118.96	-6.24
C(3)	144.8	48.1	48.2	48.2	47.4	48.6	48.09	142.01	-2.79
C(4)	127.0	71.0	71.0	71.0	69.9	70.4	70.92	119.18	-7.82
C(5)	143.1	45.0	45.1	45.0	45.9	48.0	45.15	144.95	1.85
C(6)	38.4	152.4	152.4	152.4	151.9	150.7	152.31	37.78	-0.62
C(7)	73.7	116.1	116.0	116.1	115.6	115.5	116.05	74.05	0.35
C(8)	133.8	58.9	55.7	58.5	56.5	56.5	58.01	132.09	-1.71
C(9)	126.9	68.3	69.1	68.3	69.5	69.2	68.55	121.54	-5.36
C(10)	39.4	149.9	149.7	149.6	150.0	149.9	149.78	40.32	0.92
C(11)	137.2	56.2	54.3	54.7	55.3	56.0	55.51	134.59	-2.61
C(12)	131.9	61.1	63.2	60.6	63.5	63.9	61.59	128.51	-3.39
C(13)	134.2	61.1	59.0	59.2	60.6	61.5	60.33	129.76	-4.44
C(14)	131.3	60.7	60.4	60.5	60.7	59.7	60.56	129.54	-1.76
C(15)	60.1	122.1	123.6	123.4	121.6	122.2	122.62	67.48	7.38
C(16)	31.1	158.2	157.9	158.0	155.9	157.9	158.02	32.08	0.98
C(17)	77.9	114.1	113.4	113.5	114.0	113.4	113.82	76.28	-1.62
C(18)	69.7	122.1	122.4	122.3	119.6	122.1	122.09	68.01	-1.69
C(19)	19.1	172.5	172.6	172.6	170.5	172.5	172.44	17.65	-1.45
C(20)	61.9	125.1	125.0	125.0	126.9	125.0	125.15	64.94	3.04
C(21)	15.4	174.7	174.4	174.6	174.3	174.4	174.57	15.53	0.13
C(22)	29.6	158.9	159.3	158.6	159.6	159.2	158.96	31.14	1.54
C(23)	11.9	177.7	177.3	177.3	177.6	177.2	177.50	12.59	0.69
C(24)	17.0	172.9	173.1	172.8	173.2	173.3	172.96	17.14	0.14
C(25)	13.9	175.2	173.9	174.0	175.3	174.8	174.71	15.39	1.49
C(1'')	132.1	61.4	61.9	62.0	61.6	61.2	61.63	128.46	-3.64
C(2'')	135.4	59.8	61.3	60.9	60.2	60.5	60.33	129.77	-5.63
C(3'')	130.1	67.8	66.7	66.7	67.3	66.8	67.35	122.74	-7.36
C(4'')	138.4	55.6	56.0	55.9	55.9	56.0	55.76	134.33	-4.07
C(5'')	130.1	66.9	67.0	67.0	67.1	67.4	66.94	123.16	-6.94
C(6'')	135.4	59.8	61.1	60.8	60.2	60.7	60.28	129.82	-5.58
C(7'')	21.1	169.1	169.1	169.1	169.2	169.1	169.10	21.00	-0.10
**Energy (kJ/mol)**		0.00	2.59	2.86	6.25	7.13	**DP4+ (^{13}C data)**		
**Boltzmann factor**		0.55	0.19	0.18	0.04	0.03	0.02%		

11-Desnoviosyl-11-thio-(4”-desbutyryl)-β-D-noviosyl fidaxomicin (*S*-OP1118, **18e-C(11)**)

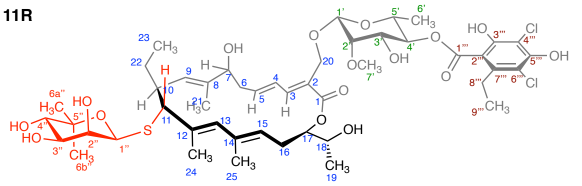

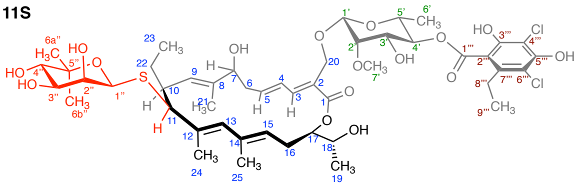

Table 19: Experimental vs. computed ^{1}H chemical shifts and DP4+ results for 11-desnoviosyl-11-thio-(4”-desbutyryl)-β-D-noviosyl fidaxomicin ***18e-C(11)*** *(PCM, solvent: acetone/B3LYP/6-31G(d))*

H-atom	Exp. δ, ppm	11*R*		11S	
		Comp. δ, ppm	Δδ, ppm	Comp. δ, ppm	Δδ, ppm
H(3)	7.28	7.56	0.28	7.35	0.07
H(4)	6.64	6.74	0.10	6.74	0.10
H(5)	6.00	6.44	0.44	6.41	0.41
H(6a, H_{Si})	2.47	2.39	-0.08	2.84	0.37
H(6b, H_{Re})	2.73	2.92	0.20	2.57	-0.16
H(7)	4.25	4.27	0.03	3.95	-0.30
H(9)	5.32	5.33	0.01	5.43	0.11
H(10)	2.47	2.65	0.18	3.10	0.63
H(11)	3.41	3.62	0.21	3.91	0.50
H(13)	5.78	5.80	0.02	6.33	0.55
H(15)	5.63	6.02	0.40	5.60	-0.03
H(16a, H_{Si})	2.47	2.53	0.06	2.23	-0.24
H(16b, H_{Re})	2.73	2.65	-0.07	2.71	-0.02
H(17)	4.73	4.61	-0.12	4.76	0.03
H(18)	3.99	4.16	0.17	3.74	-0.25
H(19)-Me	1.18	1.15	-0.03	1.02	-0.16
H(20a, H_{Re})	4.60[a]	4.54	-0.06	4.66	0.06
H(20b, H_{Si})	4.42[a]	4.38	-0.04	4.29	-0.13
H(21)-Me	1.64	1.71	0.07	1.77	0.13
H(22a, H_{Re})	1.99	2.16	0.17	1.73	-0.26
H(22b, H_{Si})	1.32	1.24	-0.08	1.52	0.21
H(23)-Me	0.82	0.89	0.07	0.98	0.16
H(24)-Me	1.87	1.93	0.06	1.93	0.06
H(25)-Me	1.72	1.46	-0.26	1.89	0.17
H(1")	4.81	4.89	0.08	4.74	-0.07
H(2")	3.93	3.87	-0.06	3.81	-0.12
H(3")	3.55	3.44	-0.11	3.37	-0.18
H(4")	3.46	3.40	-0.06	3.45	-0.01
H(6"eq)-Me	1.22	1.32	0.10	1.25	0.03
H(6"ax)-Me	1.09	1.05	-0.04	1.05	-0.04
H(1')	4.68	4.56	-0.12	4.80	0.12
H(2')	3.62	3.42	-0.20	3.53	-0.09
H(3')	3.79	3.69	-0.10	3.69	-0.10
H(4')	5.10	5.05	-0.05	5.06	-0.04
H(5')	3.62	3.75	0.14	3.69	0.07
H(6')-Me	1.31	1.29	-0.02	1.17	-0.14
H(7')-Me	3.52	3.61	0.09	3.64	0.12
H(8''')	3.01	3.25	0.24	3.23	0.22
H(9''')	1.22	1.26	0.05	1.25	0.03
**DP4+ (^{1}H data)**		**100.00**		**0.00**	
**DP4+ (all data)**		**100.00**		**0.00**	

[a]H20a, H_{Re} and H20b, H_{Si} could not be unambiguously assigned and thus, the experimental chemical shift could be the other way round.

11R

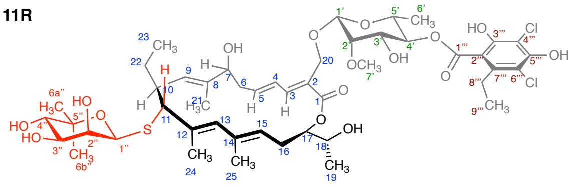

11S

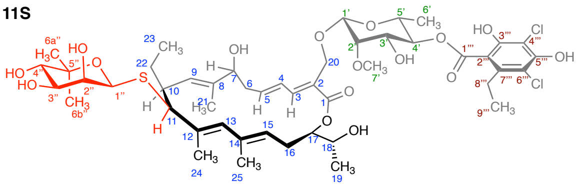

Table 20: Experimental vs. computed ^{13}C chemical shifts and DP4+ results for 11-desnoviosyl-11-thio-(4”-desbutyryl)-β-D-noviosyl fidaxomicin ***18e-C(11)*** *(PCM, solvent: acetone/B3LYP/6-31G(d))*

C-atom	Exp. δ, ppm	11*R*		11S	
		Comp. δ, ppm	Δδ, ppm	Comp. δ, ppm	Δδ, ppm
C(1)	167.8	159.5	-8.3	164.2	-3.6
C(2)	125.2	116.9	-8.3	120.4	-4.8
C(3)	145.6	143.7	-1.9	145.0	-0.6
C(4)	128.2	123.4	-4.8	120.5	-7.7
C(5)	143.6	143.8	0.2	146.4	2.8
C(6)	37.2	39.1	1.9	38.5	1.3
C(7)	72.9	73.4	0.5	72.9	0.0
C(8)	136.5	134.0	-2.5	134.2	-2.3
C(9)	126.2	120.7	-5.5	121.5	-4.7
C(10)	41.5	42.7	1.2	43.0	1.5
C(11)	62.8	69.8	7.0	63.9	1.1
C(12)	136.5	134.0	-2.5	130.7	-5.8
C(13)	131.7	126.7	-5.0	127.2	-4.5
C(14)	136.1	131.7	-4.4	131.8	-4.3
C(15)	126.3	121.9	-4.4	128.0	1.7
C(16)	28.4	29.7	1.3	33.6	5.2
C(17)	78.3	77.8	-0.5	84.4	6.1
C(18)	67.6	66.4	-1.2	68.5	0.9
C(19)	20.7	21.3	0.6	17.7	-3.0
C(20)	63.3	58.9	-4.4	64.8	1.5
C(21)	15.1	15.6	0.5	16.8	1.7
C(22)	27.7	30.2	2.5	30.3	2.6
C(23)	10.9	12.6	1.7	12.4	1.5
C(24)	14.3	15.9	1.6	20.9	6.6
C(25)	17.6	17.4	-0.2	17.5	-0.1
C(1'')	81.3	85.7	4.4	83.0	1.7
C(2'')	74.5	74.5	0.0	73.0	-1.5
C(3'')	72.3	71.1	-1.2	71.1	-1.2
C(4'')	74.2	73.8	-0.4	74.1	-0.1
C(5'')	77.7	78.2	0.5	77.8	0.1
C(6''eq)	28.8	28.4	-0.4	28.5	-0.3
C(6''ax)	16.7	15.6	-1.1	16.5	-0.2
C(1')	101.7	97.4	-4.3	104.0	2.3
C(2')	81.7	79.8	-1.9	80.4	-1.3
C(3')	72.3	70.4	-1.9	71.2	-1.1
C(4')	77.3	76.2	-1.1	75.6	-1.7
C(5')	70.7	69.5	-1.2	69.3	-1.4
C(6')	18.3	17.8	-0.5	17.6	-0.7
C(7')	61.7	59.3	-2.4	59.3	-2.4
C(1''')	169.8	165.1	-4.7	165.0	-4.8
C(2''')	108.7	103.0	-5.7	102.9	-5.8
C(3''')	156.4	152.3	-4.1	152.5	-3.9
C(4''')	108.1	107.3	-0.8	107.4	-0.7
C(5''')	155.5	145.5	-10.0	145.4	-10.1
C(6''')	115.3	115.5	0.2	115.5	0.2
C(7''')	142.6	141.8	-0.8	141.7	-0.9
C(8''')	26.3	28.3	2.0	28.4	2.1
C(9''')	14.4	14.5	0.1	14.6	0.2
**DP4+ (^{13}C data)**		**99.99**		**0.01**	
**DP4+ (all data)**		**100.00**		**0.00**	

*Table 21: DP4+ results for **11R**: Computed σ for H-nuclei for each conformer and the averaged σ (conformers weighted based on Boltzmann factors) for 11-desnoviosyl-11R-thio-(4"-desbutyryl)-β-D-noviosyl fidaxomicin **18e-C(11)**. Computed chemical shifts δ were calculated using TMS as a reference (σ_H(TMS): 32.148). (PCM, solvent: acetone/B3LYP/6-31G(d))*

H-atom	***Exp. δ, ppm***	***Conformers, shielding tensor σ***							***σ Boltz. avrg.***	***Computed avrg. δ, ppm***	***Δδ, ppm***
		**397**	**385**	**379**	**61**	**1**	**333**	**15**			
H(3)	7.28	24.58	24.61	24.51	24.65	24.66	24.49	24.64	24.59	7.56	0.28
H(4)	6.64	25.42	25.47	25.45	25.26	25.22	25.44	25.26	25.41	6.74	0.10
H(5)	6.00	25.74	25.72	25.57	25.75	25.77	25.56	25.75	25.71	6.44	0.44
H(6a, H_{Si})	2.47	29.80	29.78	29.66	29.72	29.73	29.65	29.72	29.76	2.39	-0.08
H(6b, H_{Re})	2.73	29.22	29.18	29.41	29.15	29.14	29.40	29.15	29.23	2.92	0.20
H(7)	4.25	27.94	27.83	27.79	27.87	27.87	27.80	27.88	27.87	4.27	0.03
H(9)	5.32	26.82	26.74	26.90	26.85	26.84	26.90	26.86	26.82	5.33	0.01
H(10)	2.47	29.51	29.46	29.48	29.53	29.55	29.51	29.53	29.50	2.65	0.18
H(11)	3.41	28.53	28.50	28.54	28.57	28.59	28.59	28.63	28.53	3.62	0.21
H(13)	5.78	26.34	26.35	26.30	26.41	26.40	26.31	26.42	26.35	5.80	0.02
H(15)	5.63	26.12	26.13	26.09	26.18	26.19	26.06	26.16	26.12	6.02	0.40
H(16a, H_{Si})	2.47	29.61	29.61	29.60	29.63	29.66	29.61	29.63	29.61	2.53	0.06
H(16b, H_{Re})	2.73	29.49	29.50	29.47	29.55	29.57	29.45	29.53	29.50	2.65	-0.07
H(17)	4.73	27.52	27.53	27.53	27.63	27.63	27.51	27.61	27.54	4.61	-0.12
H(18)	3.99	27.98	27.99	27.97	28.00	28.00	27.94	27.98	27.98	4.16	0.17
H(19)-Me	1.18	30.79	30.82	30.82	30.76	30.75	31.38	30.75	31.00	1.15	-0.03
H(20a, H_{Re})	4.60[a]	27.56	27.61	27.54	27.86	27.76	27.53	27.86	27.61	4.54	-0.06
H(20b, H_{Si})	4.42[a]	27.81	27.83	27.78	27.50	27.55	27.77	27.50	27.77	4.38	-0.04
H(21)-Me	1.64	30.79	30.73	30.87	30.06	30.07	30.07	30.07	30.44	1.71	0.07
H(22a, H_{Re})	1.99	29.99	29.96	29.98	30.00	29.98	30.06	30.05	29.98	2.16	0.17
H(22b, H_{Si})	1.32	30.91	30.87	30.97	30.91	30.91	30.98	30.94	30.91	1.24	-0.08
H(23)-Me	0.82	31.20	31.13	31.28	31.22	31.22	31.30	31.24	31.26	0.89	0.07
H(24)-Me	1.87	30.57	30.54	30.48	30.58	30.62	29.93	30.57	30.21	1.93	0.06
H(25)-Me	1.72	30.93	30.97	30.79	31.10	30.51	30.69	30.49	30.69	1.46	-0.26
H(1")	4.81	27.25	27.25	27.23	27.25	27.25	27.38	27.43	27.25	4.89	0.08
H(2")	3.93	28.29	28.29	28.29	28.32	28.31	28.06	28.08	28.28	3.87	-0.06
H(3")	3.55	28.71	28.72	28.71	28.74	28.75	28.62	28.64	28.71	3.44	-0.11
H(4")	3.46	28.76	28.76	28.76	28.78	28.78	28.57	28.58	28.75	3.40	-0.06
H(6"eq)-Me	1.22	31.09	31.09	31.08	30.66	30.65	31.01	30.62	30.83	1.32	0.10
H(6"ax)-Me	1.09	30.78	30.79	30.77	30.80	30.79	30.77	31.10	31.10	1.05	-0.04
H(1')	4.68	27.63	27.57	27.63	27.47	27.47	27.63	27.46	27.59	4.56	-0.12
H(2')	3.62	28.73	28.80	28.72	28.63	28.59	28.72	28.63	28.73	3.42	-0.20
H(3')	3.79	28.41	28.57	28.38	28.47	28.38	28.39	28.47	28.45	3.69	-0.10
H(4')	5.10	27.15	27.04	27.15	27.02	27.00	27.16	27.02	27.10	5.05	-0.05
H(5')	3.62	28.46	28.29	28.46	28.32	28.39	28.47	28.32	28.40	3.75	0.14
H(6')-Me	1.31	30.67	30.73	30.63	30.79	30.81	30.99	30.79	30.86	1.29	-0.02
H(7')-Me	3.52	28.18	28.86	28.17	28.61	28.40	28.50	28.43	28.54	3.61	0.09
H(8''')	3.01	28.89	28.85	28.99	28.86	28.99	28.99	28.86	28.90	3.25	0.24
H(9''')	1.22	30.82	30.95	30.92	30.87	30.81	30.83	30.87	30.88	1.26	0.05
Energy (kJ/mol)		0.00	0.89	2.88	4.52	4.90	5.98	7.68	**DP4+ (^{1}H data)**		
Boltzmann factor		0.41	0.29	0.13	0.07	0.06	0.04	0.02	**100.00**		

[a]H20a, H_{Re} and H20b, H_{Si} could not be unambiguously assigned and thus, the experimental chemical shift could be the other way round.

Table 22: DP4+ results for ***11R****: Computed σ for C-nuclei for each conformer and the averaged σ (conformers weighted based on Boltzmann factors) for 11-desnoviosyl-11R-thio-(4"-desbutyryl)-β-*D*-noviosyl fidaxomicin* ***18e-C(11)****. Computed chemical shifts δ were calculated using TMS as a reference (σ_C(TMS): 190.0976). (PCM, solvent: acetone/B3LYP/6-31G(d))*

C-atom	*Exp. δ, ppm*	***Conformers, shielding tensor σ***							*σ Boltz. avrg.*	*Computed avrg. δ, ppm*	*Δδ, ppm*
		**397**	**385**	**379**	**61**	**1**	**333**	**15**			
C(1)	167.8	30.7	30.7	30.7	30.4	30.4	30.8	30.4	30.6	159.5	-8.3
C(2)	125.2	73.7	72.4	75.5	70.9	70.9	75.6	71.0	73.2	116.9	-8.3
C(3)	145.6	46.2	47.2	44.8	47.3	47.3	44.7	47.2	46.4	143.7	-1.9
C(4)	128.2	66.3	66.3	69.5	65.3	65.2	69.4	65.4	66.7	123.4	-4.8
C(5)	143.6	46.7	46.9	43.6	46.6	46.9	43.6	46.5	46.2	143.8	0.2
C(6)	37.2	150.6	150.8	152.5	151.0	150.9	152.4	150.9	151.0	39.1	1.9
C(7)	72.9	116.8	116.9	115.5	117.0	117.1	115.5	117.0	116.7	73.4	0.5
C(8)	136.5	56.2	56.3	55.6	56.3	56.3	55.5	56.2	56.1	134.0	-2.5
C(9)	126.2	69.2	69.1	70.4	69.3	69.3	70.4	69.3	69.4	120.7	-5.5
C(10)	41.5	147.4	147.4	147.5	147.3	147.5	147.5	147.5	147.4	42.7	1.2
C(11)	62.8	120.4	120.4	119.9	120.4	120.1	120.0	120.2	120.3	69.8	7.0
C(12)	136.5	56.1	56.1	56.3	55.8	55.6	56.1	55.9	56.1	134.0	-2.5
C(13)	131.7	63.4	63.5	63.4	63.4	63.4	63.4	63.4	63.4	126.7	-5.0
C(14)	136.1	58.5	58.3	58.5	57.8	57.8	58.5	57.9	58.3	131.7	-4.4
C(15)	126.3	68.1	68.2	68.3	68.4	68.5	68.3	68.4	68.2	121.9	-4.4
C(16)	28.4	160.3	160.3	160.2	161.0	161.1	160.2	161.0	160.4	29.7	1.3
C(17)	78.3	112.5	112.3	112.6	111.5	111.4	112.7	111.5	112.3	77.8	-0.5
C(18)	67.6	123.7	123.6	123.7	123.6	123.6	123.6	123.5	123.7	66.4	-1.2
C(19)	20.7	168.8	168.8	168.9	168.8	168.8	168.9	168.8	168.8	21.3	0.6
C(20)	63.3	131.9	131.4	131.3	128.3	128.5	131.2	128.3	131.2	58.9	-4.4
C(21)	15.1	174.5	174.5	174.5	174.6	174.7	174.5	174.6	174.5	15.6	0.5
C(22)	27.7	160.0	160.1	159.3	159.8	159.9	159.3	160.0	159.9	30.2	2.5
C(23)	10.9	177.5	177.4	177.7	177.5	177.5	177.8	177.5	177.5	12.6	1.7
C(24)	14.3	174.2	174.4	174.0	174.1	174.4	173.8	174.1	174.2	15.9	1.6
C(25)	17.6	172.8	172.5	172.5	172.9	173.1	172.5	173.0	172.7	17.4	-0.2
C(1")	81.3	104.6	104.5	104.4	104.6	104.0	103.4	103.3	104.4	85.7	4.4
C(2")	74.5	115.6	115.5	115.7	115.7	115.7	115.9	115.7	115.6	74.5	0.0
C(3")	72.3	118.9	118.9	118.9	119.1	119.0	119.9	119.9	119.0	71.1	-1.2
C(4")	74.2	116.3	116.3	116.2	116.2	116.1	117.1	117.1	116.3	73.8	-0.4
C(5")	77.7	111.9	111.8	111.9	111.8	111.8	112.6	112.5	111.9	78.2	0.5
C(6"eq)	28.8	161.7	161.6	161.6	161.6	161.6	161.7	161.6	161.7	28.4	-0.4
C(6"ax)	16.7	174.5	174.5	174.6	174.7	174.5	174.5	174.4	174.5	15.6	-1.1
C(1')	101.7	93.7	92.6	93.8	88.3	88.6	94.0	88.3	92.7	97.4	-4.3
C(2')	81.7	110.2	110.2	110.3	110.3	110.4	110.3	110.3	110.2	79.8	-1.9
C(3')	72.3	120.4	119.0	120.0	118.7	119.5	120.0	118.7	119.7	70.4	-1.9
C(4')	77.3	113.6	114.5	113.3	114.3	114.4	113.3	114.3	113.9	76.2	-1.1
C(5')	70.7	120.5	120.8	120.5	120.8	120.6	120.6	120.9	120.6	69.5	-1.2
C(6')	18.3	172.2	172.3	172.0	172.5	172.8	171.8	172.5	172.3	17.8	-0.5
C(7')	61.7	130.7	130.8	130.9	131.0	131.0	130.9	131.0	130.8	59.3	-2.4
C(1''')	169.8	24.8	24.9	25.2	25.1	25.3	25.2	25.1	25.0	165.1	-4.7
C(2''')	108.7	86.8	87.7	86.7	87.3	86.7	86.6	87.3	87.1	103.0	-5.7
C(3''')	156.4	38.1	37.0	38.4	37.4	38.4	38.5	37.4	37.8	152.3	-4.1
C(4''')	108.1	82.8	82.7	82.9	82.6	82.8	82.9	82.6	82.8	107.3	-0.8
C(5''')	155.5	44.6	44.7	44.7	44.7	44.7	44.7	44.7	44.6	145.5	-10.0
C(6''')	115.3	74.5	74.8	74.5	74.5	74.6	74.5	74.5	74.6	115.5	0.2
C(7''')	142.6	48.4	47.7	48.8	48.2	48.8	48.8	48.2	48.3	141.8	-0.8
C(8''')	26.3	161.9	162.0	161.6	161.7	161.5	161.7	161.7	161.8	28.3	2.0
C(9''')	14.4	175.7	175.5	175.8	175.5	175.6	175.8	175.6	175.6	14.5	0.1
Energy (kJ/mol)		0.00	0.89	2.88	4.52	4.90	5.98	7.68	**DP4+ (^{13}C data)**		
Boltzmann factor		0.41	0.29	0.13	0.07	0.06	0.04	0.02	**99.99**		

*Table 23: DP4+ results for **11S**: Computed σ for H-nuclei for each conformer and the averaged σ (conformers weighted based on Boltzmann factors) for 11-desnoviosyl-11S-thio-(4''-desbutyryl)-β-D-noviosyl fidaxomicin **18e-C(11)**. Computed chemical shifts δ were calculated using TMS as a reference (σ_H(TMS): 32.148). (PCM, solvent: acetone/B3LYP/6-31G(d)). Total amount of conformers: 33.*

**H-atom**	**Exp. δ, ppm**	**Conformers, shielding tensor σ**														**σ Boltz. avrg.**	**Comp. avrg. δ, ppm**	**Δδ, ppm**
		**212**	**209**	**261**	**262**	**260**	**263**	…	**83**	**130**	**33**	**163**	**121**	**129**	**96**			
H(3)	7.28	24.78	24.78	24.76	24.75	24.75	24.75	…	25.07	24.99	24.99	24.98	24.99	24.99	25.00	24.80	7.35	0.07
H(4)	6.64	25.36	25.37	25.37	25.36	25.38	25.36	…	25.71	25.65	25.65	25.65	25.65	25.65	25.65	25.41	6.74	0.10
H(5)	6.00	25.76	25.74	25.74	25.75	25.71	25.75	…	25.70	25.67	25.67	25.68	25.67	25.67	25.68	25.73	6.41	0.41
H(6a, H_{Si})	2.47	29.25	29.26	29.29	29.29	29.30	29.29	…	29.49	29.49	29.49	29.49	29.49	29.49	29.49	29.31	2.84	0.37
H(6b, H_{Re})	2.73	29.58	29.59	29.59	29.58	29.60	29.58	…	29.56	29.57	29.56	29.56	29.56	29.56	29.56	29.58	2.57	-0.16
H(7)	4.25	28.25	28.27	28.25	28.24	28.26	28.24	…	27.92	27.93	27.93	27.93	27.93	27.93	27.93	28.20	3.95	-0.30
H(9)	5.32	26.73	26.72	26.68	26.69	26.66	26.69	…	26.86	26.88	26.88	26.87	26.88	26.88	26.88	26.72	5.43	0.11
H(10)	2.47	29.01	29.01	29.01	29.03	29.00	29.03	…	29.26	29.26	29.26	29.26	29.26	29.26	29.26	29.05	3.10	0.63
H(11)	3.41	28.33	28.32	28.16	28.16	28.15	28.16	…	28.48	28.46	28.46	28.45	28.45	28.46	28.45	28.24	3.91	0.50
H(13)	5.78	25.77	25.76	25.73	25.74	25.70	25.74	…	26.14	26.20	26.19	26.19	26.19	26.19	26.19	25.82	6.33	0.55
H(15)	5.63	26.50	26.49	26.50	26.50	26.48	26.50	…	26.80	26.84	26.84	26.84	26.84	26.84	26.84	26.55	5.60	-0.03
H(16a, H_{Si})	2.47	29.94	29.95	29.95	29.94	29.96	29.94	…	30.15	29.54	29.54	29.55	29.55	29.55	29.55	29.92	2.23	-0,24
H(16b, H_{Re})	2.73	29.43	29.43	29.40	29.41	29.39	29.41	…	29.63	29.68	29.68	29.68	29.68	29.68	29.68	29.44	2.71	-0,02
H(17)	4.73	27.39	27.44	27.41	27.39	27.48	27.39	…	26.71	27.27	27.28	27.27	27.28	27.27	27.27	27.38	4.76	0.03
H(18)	3.99	28.42	28.44	28.42	28.41	28.44	28.41	…	28.25	28.34	28.34	28.34	28.34	28.34	28.34	28.41	3.74	-0.25
H(19)-Me	1.18	31.08	31.07	31.08	31.07	31.07	31.30	…	30.87	31.33	30.92	30.67	30.69	30.69	31.34	31.13	1.02	-0.16
H(20a, H_{Re})	4.60[a]	27.46	27.43	27.47	27.51	27.44	27.51	…	27.52	27.58	27.58	27.56	27.58	27.59	27.59	27.48	4.66	0.06
H(20b, H_{Si})	4.42[a]	27.91	27.95	27.90	27.86	27.94	27.85	…	27.62	27.65	27.64	27.63	27.64	27.64	27.64	27.86	4.29	-0.13
H(21)-Me	1.64	30.16	30.66	30.16	30.68	30.16	30.68	…	30.29	30.83	30.28	30.29	30.93	30.28	30.84	30.38	1.77	0.13
H(22a, H_{Re})	1.99	30.50	30.50	30.34	30.35	30.33	30.36	…	30.52	30.52	30.52	30.52	30.52	30.52	30.52	30.41	1.73	-0.26
H(22b, H_{Si})	1.32	30.37	30.36	30.83	30.84	30.83	30.84	…	30.24	30.24	30.24	30.24	30.24	30.24	30.24	30.62	1.52	0.21
H(23)-Me	0.82	31.26	31.26	31.15	31.16	31.15	31.16	…	31.24	31.43	31.30	31.43	31.43	31.43	31.30	31.17	0.98	0.16
H(24)-Me	1.87	29.89	29.88	29.83	30.84	30.83	29.83	…	29.98	29.95	29.96	29.97	30.88	30.88	30.88	30.22	1.93	0.06
H(25)-Me	1.72	30.14	30.13	30.11	30.12	30.09	30.12	…	30.32	30.61	30.16	30.62	30.16	30.32	30.16	30.26	1.89	0.17
H(1'')	4.81	27.40	27.40	27.34	27.37	27.33	27.37	…	27.46	27.46	27.45	27.44	27.45	27.45	27.45	27.41	4.74	-0.07
H(2'')	3.93	28.41	28.38	28.37	28.40	28.33	28.40	…	28.37	28.38	28.38	28.38	28.38	28.38	28.38	28.34	3.81	-0.12
H(3'')	3.55	28.80	28.78	28.78	28.81	28.75	28.81	…	28.77	28.78	28.78	28.78	28.78	28.78	28.78	28.78	3.37	-0.18
H(4'')	3.46	28.74	28.73	28.72	28.74	28.71	28.74	…	28.73	28.74	28.73	28.73	28.73	28.73	28.73	28.70	3.45	-0.01
H(6''eq)-Me	1.22	31.13	31.12	31.12	31.12	31.11	31.12	…	30.64	30.65	31.14	30.98	30.98	30.98	30.65	30.90	1.25	0.03
H(6''ax)-Me	1.09	31.54	31.53	31.52	30.99	30.97	30.99	…	31.00	31.59	30.79	31.60	30.79	30.79	31.00	31.10	1.05	-0.04
H(1')	4.68	27.30	27.34	27.29	27.25	27.34	27.25	…	27.62	27.67	27.68	27.69	27.68	27.67	27.68	27.34	4.80	0.12
H(2')	3.62	28.60	28.59	28.60	28.61	28.59	28.61	…	28.79	28.75	28.76	28.78	28.76	28.76	28.77	28.62	3.53	-0.09
H(3')	3.79	28.46	28.42	28.45	28.47	28.42	28.47	…	28.45	28.45	28.49	28.51	28.49	28.45	28.49	28.45	3.69	-0.10
H(4')	5.1	27.08	27.09	27.08	27.11	27.09	27.11	…	27.02	27.03	27.03	27.06	27.03	27.03	27.03	27.09	5.06	-0.04
H(5')	3.62	28.44	28.45	28.44	28.44	28.46	28.44		28.55	28.56	28.52	28.51	28.52	28.55	28.51	28.46	3.69	0.07

H(6')-Me	1.31	31.12	30.90	30.93	30.90	30.90	31.10		30.87	31.02	31.08	30.85	30.85	30.85	31.08	30.98	1.17	-0.14
H(7')-Me	3.52	28.18	28.18	28.49	28.49	28.50	28.19		28.09	28.16	28.17	28.90	28.17	28.17	28.17	28.51	3.64	0.12
H(8''')	3.01	28.87	28.95	28.87	28.85	28.94	28.84	…	29.00	28.99	29.04	28.98	29.03	28.99	29.04	28.92	3.23	0.22
H(9''')	1.22	30.92	30.84	30.87	30.85	30.84	30.85	…	30.88	30.85	31.00	30.87	30.86	30.85	31.00	30.90	1.25	0.03
Energy (kJ/mol)		0.00	0.27	0.69	1.00	1.03	1.10	…	8.19	8.19	8.20	8.21	8.24	8.24	8.27	**DP4+ (^{1}H data)**		
Boltzmann factor		0.15	0.14	0.12	0.10	0.10	0.10	…	0.01	0.01	0.01	0.01	0.01	0.01	0.01	**0.00**		

[a]H20a, H_{Re} and H20b, H_{Si} could not be unambiguously assigned and thus, the experimental chemical shift could be the other way round.

*Table 24: DP4+ results for **11S**: Computed σ for C-nuclei for each conformer and the averaged σ (conformers weighted based on Boltzmann factors) for 11-desnoviosyl-11S-thio-(4''-desbutyryl)-β-D-noviosyl fidaxomicin **18e-C(11)**. Computed chemical shifts δ were calculated using TMS as a reference (σ_C(TMS): 190.0976). (PCM, solvent: acetone/B3LYP/6-31G(d)). Total amount of conformers: 33.*

C-atom	***Exp. δ, ppm***	***Conformers, shielding tensor σ***														***σ Boltz. avrg.***	***Comp. avrg. δ, ppm***	***Δδ, ppm***
		**212**	**209**	**261**	**262**	**260**	**263**	…	**83**	**130**	**33**	**163**	**121**	**129**	**96**			
C(1)	167.8	25.5	25.6	25.5	25.4	25.7	25.4	…	27.7	28.2	28.2	28.2	28.2	28.2	28.2	25.9	164.2	-3.6
C(2)	125.2	69.4	69.4	69.5	69.6	69.4	69.6	…	70.0	70.9	70.9	71.0	70.9	70.9	70.8	69.7	120.4	-4.8
C(3)	145.6	44.8	44.7	44.8	44.8	44.8	44.7	…	47.6	47.8	47.8	47.5	47.8	47.8	47.9	45.1	145.0	-0.6
C(4)	128.2	69.7	69.8	69.6	69.5	69.6	69.5	…	70.0	69.8	69.8	69.9	69.8	69.8	69.8	69.6	120.5	-7.7
C(5)	143.6	42.9	42.7	43.1	43.1	43.1	43.1	…	48.6	48.7	48.7	48.7	48.7	48.7	48.7	43.7	146.4	2.8
C(6)	37.2	151.9	151.7	151.7	151.9	151.4	151.9	…	150.6	150.6	150.7	150.7	150.7	150.7	150.7	151.6	38.5	1.3
C(7)	72.9	117.2	117.1	117.6	117.6	117.4	117.5	…	116.1	115.9	115.9	115.9	115.9	115.9	115.9	117.2	72.9	0.0
C(8)	136.5	54.2	54.2	57.4	57.4	57.3	57.3	…	52.8	52.8	52.9	52.9	52.9	52.9	52.9	55.9	134.2	-2.3
C(9)	126.2	69.3	69.3	67.6	67.5	67.5	67.5	…	71.2	71.2	71.2	71.2	71.2	71.2	71.2	68.6	121.5	-4.7
C(10)	41.5	146.9	146.9	147.2	147.3	147.2	147.3	…	148.1	148.1	148.1	148.1	148.1	148.1	148.1	147.1	43.0	1.5
C(11)	62.8	122.6	122.4	129.0	128.8	129.2	128.8	…	123.0	122.9	123.0	123.3	123.1	123.0	123.1	126.2	63.9	1.1
C(12)	136.5	59.9	59.8	59.2	59.3	59.1	59.3	…	59.8	60.7	60.6	60.6	60.6	60.6	60.6	59.4	130.7	-5.8
C(13)	131.7	62.9	62.9	62.9	62.9	62.8	62.9	…	63.5	63.2	63.2	63.3	63.3	63.3	63.3	62.9	127.2	-4.5
C(14)	136.1	58.7	58.6	58.4	58.6	58.2	58.6	…	56.5	56.9	57.0	57.0	57.0	56.9	57.0	58.3	131.8	-4.3
C(15)	126.3	61.4	61.5	61.9	61.6	62.1	61.6	…	64.5	63.1	63.1	63.2	63.1	63.1	63.1	62.1	128.0	1.7
C(16)	28.4	156.6	156.5	156.5	156.5	156.3	156.5	…	157.4	155.3	155.3	155.3	155.2	155.2	155.3	156.5	33.6	5.2
C(17)	78.3	104.6	104.4	104.5	104.5	104.5	104.5	…	113.9	112.7	112.7	112.8	112.7	112.7	112.7	105.7	84.4	6.1
C(18)	67.6	121.7	121.8	121.7	121.7	121.8	121.7	…	121.9	120.4	120.4	120.4	120.4	120.4	120.4	121.6	68.5	0.9
C(19)	20.7	172.7	172.7	172.7	172.6	172.7	172.6	…	172.4	169.1	169.1	169.0	169.1	169.1	169.0	172.4	17.7	-3.0
C(20)	63.3	124.4	123.9	124.5	125.1	123.9	125.1	…	129.9	131.0	130.9	130.9	130.9	130.9	130.9	125.3	64.8	1.5
C(21)	15.1	172.2	172.2	173.5	173.5	173.6	173.4	…	175.1	175.0	175.1	175.1	175.1	175.0	175.1	173.3	16.8	1.7
C(22)	27.7	158.3	158.3	161.2	161.1	161.2	161.1	…	157.3	157.2	157.3	157.3	157.3	157.3	157.3	159.8	30.3	2.6
C(23)	10.9	176.8	176.8	178.2	178.2	178.2	178.2	…	176.9	176.9	176.9	176.9	176.9	176.9	176.9	177.7	12.4	1.5
C(24)	14.3	169.0	169.0	169.1	169.0	169.1	169.0	…	169.2	169.3	169.2	169.2	169.2	169.2	169.2	169.2	20.9	6.6
C(25)	17.6	172.7	172.7	172.6	172.6	172.5	172.6	…	172.2	172.6	172.5	172.5	172.5	172.5	172.5	172.6	17.5	-0.1
C(1'')	81.3	107.4	107.3	107.1	107.1	107.1	107.1	…	107.6	107.8	107.9	108.2	107.9	107.9	108.0	107.1	83.0	1.7
C(2'')	74.5	117.2	117.1	117.0	117.0	117.0	117.0	…	117.3	117.4	117.4	117.5	117.4	117.4	117.4	117.1	73.0	-1.5

C(3'')	72.3	118.8	118.8	118.8	118.8	118.8	118.8	…	118.8	118.8	118.8	118.7	118.8	118.8	118.8	119.0	71.1	-1.2
C(4'')	74.2	115.9	115.8	115.9	115.9	115.9	115.9	…	115.9	115.8	115.8	115.7	115.7	115.8	115.7	116.0	74.1	-0.1
C(5'')	77.7	112.2	112.2	112.1	112.1	112.1	112.1	…	112.3	112.3	112.3	112.2	112.3	112.3	112.3	112.3	77.8	0.1
C(6''eq)	28.8	161.7	161.7	161.5	161.5	161.5	161.5	…	161.8	161.8	161.8	161.8	161.8	161.8	161.8	161.6	28.5	-0.3
C(6''ax)	16.7	173.6	173.6	173.6	173.6	173.6	173.6	…	173.7	173.7	173.7	173.7	173.7	173.7	173.7	173.6	16.5	-0.2
C(1')	101.7	85.0	84.4	85.1	85.6	84.5	85.6	…	92.3	92.4	92.4	92.7	92.4	92.3	92.3	86.1	104.0	2.3
C(2')	81.7	109.6	109.7	109.6	109.6	109.7	109.6	…	108.9	110.0	110.0	109.9	110.0	110.0	109.9	109.7	80.4	-1.3
C(3')	72.3	118.8	118.9	118.8	118.6	119.0	118.6	…	119.7	120.0	119.7	119.3	119.7	119.9	119.6	118.9	71.2	-1.1
C(4')	77.3	114.6	114.4	114.5	114.4	114.4	114.3	…	114.4	114.4	114.6	114.4	114.6	114.5	114.6	114.5	75.6	-1.7
C(5')	70.7	120.9	120.6	120.9	121.0	120.6	121.0	…	120.7	120.7	120.9	120.9	120.9	120.7	120.9	120.8	69.3	-1.4
C(6')	18.3	172.5	172.6	172.4	172.3	172.6	172.3	…	172.9	172.8	172.8	172.7	172.8	172.8	172.8	172.5	17.6	-0.7
C(7')	61.7	130.8	130.9	130.8	130.8	130.9	130.8	…	129.7	130.8	130.7	130.7	130.8	130.8	130.7	130.8	59.3	-2.4
C(1''')	169.8	25.1	25.2	25.1	25.1	25.2	25.1	…	25.1	25.1	25.2	25.3	25.2	25.1	25.2	25.1	165.0	-4.8
C(2''')	108.7	87.3	86.9	87.3	87.3	86.9	87.3	…	87.0	86.9	86.8	86.9	86.8	86.9	86.8	87.2	102.9	-5.8
C(3''')	156.4	37.5	38.0	37.5	37.3	37.9	37.3	…	38.1	38.1	38.2	37.2	38.2	38.1	38.3	37.6	152.5	-3.9
C(4''')	108.1	82.7	82.8	82.7	82.7	82.8	82.7	…	82.8	82.8	82.9	82.7	82.9	82.8	82.9	82.7	107.4	-0.7
C(5''')	155.5	44.7	44.8	44.7	44.7	44.8	44.7	…	44.7	44.7	44.7	44.7	44.7	44.7	44.7	44.7	145.4	-10.1
C(6''')	115.3	74.7	74.6	74.7	74.6	74.6	74.6	…	74.6	74.6	74.7	74.4	74.7	74.6	74.7	74.6	115.5	0.2
C(7''')	142.6	48.2	48.5	48.2	48.2	48.5	48.2	…	48.6	48.7	48.7	49.7	48.7	48.7	48.7	48.4	141.7	-0.9
C(8''')	26.3	161.7	161.6	161.7	161.7	161.6	161.7	…	161.7	161.7	161.6	161.2	161.6	161.7	161.6	161.7	28.4	2.1
C(9''')	14.4	175.4	175.5	175.4	175.5	175.5	175.5	…	175.6	175.7	175.6	175.7	175.6	175.7	175.6	175.5	14.6	0.2
Energy (kJ/mol)		0.00	0.27	0.69	1.00	1.03	1.10	…	8.19	8.19	8.20	8.21	8.24	8.24	8.27	**DP4+ (^{13}C data)**		
Boltzmann factor		0.15	0.14	0.12	0.10	0.10	0.10	…	0.01	0.01	0.01	0.01	0.01	0.01	0.01	**0.01**		

11-Desnoviosyl-11-thio-(4”-desbutyryl)-β-D-noviosyl fidaxomicin simplified (**18e-C(11)S**)

11*R*

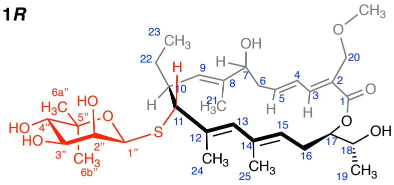

11*S*

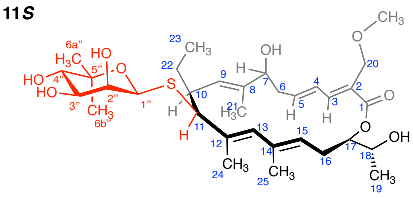

Table 25: Experimental vs. computed ^{1}H chemical shifts and DP4+ results for simplified 11-desnoviosyl-11-thio-(4”-desbutyryl)-β-D-noviosyl fidaxomicin ***18e-C(11)S*** *(PCM, solvent: acetone/B3LYP/6-31G(d))*

H-atom	Exp. δ, ppm	11*R*		11S	
		Comp. δ, ppm	Δδ, ppm	Comp. δ, ppm	Δδ, ppm
H(3)	7.28	7.36	0.08	7.38	0.10
H(4)	6.64	6.84	0.20	6.85	0.21
H(5)	6.00	6.39	0.39	6.29	0.29
H(6a, H_{Si})	2.47	2.46	-0.01	2.40	-0.07
H(6b, H_{Re})	2.73	2.89	0.16	2.93	0.21
H(7)	4.25	4.24	-0.01	4.19	-0.06
H(9)	5.32	5.21	-0.11	5.31	-0.01
H(10)	2.47	2.69	0.22	3.04	0.57
H(11)	3.41	3.43	0.02	3.84	0.43
H(13)	5.78	5.81	0.03	6.19	0.41
H(15)	5.63	5.60	-0.03	5.50	-0.12
H(16a, H_{Si})	2.47	2.18	-0.29	2.16	-0.31
H(16b, H_{Re})	2.73	2.75	0.03	2.76	0.03
H(17)	4.73	4.61	-0.12	4.76	0.03
H(18)	3.99	3.81	-0.18	3.81	-0.18
H(19)-Me	1.18	1.03	-0.15	1.01	-0.17
H(20a, H_{Re})	4.60[a]	4.15	-0.45	4.32	-0.28
H(20b, H_{Si})	4.42[a]	4.18	-0.24	4.18	-0.24
H(21)-Me	1.64	1.71	0.07	1.67	0.03
H(22a, H_{Re})	1.99	2.14	0.15	1.69	-0.30
H(22b, H_{Si})	1.32	1.14	-0.18	1.63	0.32
H(23)-Me	0.82	0.85	0.03	0.94	0.12
H(24)-Me	1.87	1.89	0.02	1.98	0.11
H(25)-Me	1.72	1.88	0.16	1.93	0.21
H(1")	4.81	4.90	0.09	4.71	-0.10
H(2")	3.93	3.83	-0.10	3.79	-0.14
H(3")	3.55	3.42	-0.13	3.40	-0.15
H(4")	3.46	3.42	-0.04	3.45	-0.01
H(6"eq)-Me	1.22	1.24	0.03	1.25	0.04
H(6"ax)-Me	1.09	1.10	0.01	1.06	-0.03
**DP4+ (^{1}H data)**		**100.00%**		**0.00%**	
**DP4+ (all data)**		**100.00%**		**0.00%**	

[a]H20a, H_{Re} and H20b, H_{Si} could not be unambiguously assigned and thus, the experimental chemical shift could be the other way round.

11***R***

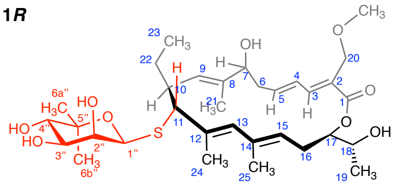

11***S***

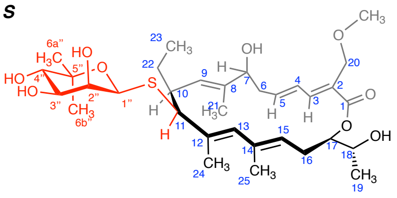

*Table 26: Experimental vs. computed ¹³C chemical shifts and DP4+ results for simplified 11-desnoviosyl-11-thio-(4"-desbutyryl)-β-D-noviosyl fidaxomicin **18e-C(11)S** (PCM, solvent: acetone/B3LYP/6-31G(d))*

C-atom	Exp. δ, ppm	11*R*		11S	
		Comp. δ, ppm	Δδ, ppm	Comp. δ, ppm	Δδ, ppm
C(1)	167.8	163.4	-4.4	164.2	-3.6
C(2)	125.2	120.0	-5.2	120.2	-5.0
C(3)	145.6	143.5	-2.1	144.1	-1.5
C(4)	128.2	124.8	-3.4	124.9	-3.3
C(5)	143.6	143.1	-0.5	143.0	-0.6
C(6)	37.2	38.9	1.7	39.2	2.0
C(7)	72.9	73.0	0.1	73.0	0.1
C(8)	136.5	134.7	-1.8	135.1	-1.4
C(9)	126.2	122.3	-3.9	119.8	-6.4
C(10)	41.5	44.0	2.5	43.2	1.7
C(11)	62.8	69.9	7.1	65.7	2.9
C(12)	136.5	134.2	-2.3	130.6	-5.9
C(13)	131.7	127.2	-4.5	127.0	-4.7
C(14)	136.1	132.3	-3.8	131.3	-4.8
C(15)	126.3	127.2	0.9	128.6	2.3
C(16)	28.4	33.0	4.6	33.0	4.6
C(17)	78.3	83.4	5.1	84.7	6.4
C(18)	67.6	68.8	1.2	69.0	1.4
C(19)	20.7	18.1	-2.6	17.7	-3.0
C(20)	63.3	64.5	1.2	63.7	0.4
C(21)	15.1	16.0	0.9	15.1	0.0
C(22)	27.7	28.9	1.2	31.3	3.6
C(23)	10.9	12.6	1.7	12.7	1.8
C(24)	14.3	16.4	2.1	21.1	6.8
C(25)	17.6	17.2	-0.4	17.4	-0.2
C(1")	81.3	85.8	4.5	82.7	1.4
C(2")	74.5	73.7	-0.8	72.9	-1.6
C(3")	72.3	71.1	-1.2	71.1	-1.2
C(4")	74.2	73.9	-0.3	74.1	-0.1
C(5")	77.7	77.8	0.1	77.8	0.1
C(6"eq)	28.8	28.5	-0.3	28.5	-0.3
C(6"ax)	16.7	16.4	-0.3	16.5	-0.2
**DP4+ (¹³C data)**		**99.99%**		**0.01%**	
**DP4+ (all data)**		**100.00%**		**0.00%**	

*Table 27: DP4+ results for **11R**: Computed σ for H-nuclei for each conformer and the averaged σ (conformers weighted based on Boltzmann factors) for simplified 11-desnoviosyl-11R-thio-(4”-desbutyryl)-β-D-noviosyl fidaxomicin **18e-C(11)S**. Computed chemical shifts δ were calculated using TMS as a reference (σ_C(TMS): 190.0976; σ_H(TMS): 32.148). (PCM, solvent: acetone/B3LYP/6-31G(d))*

H-atom	***Exp. δ, ppm***	***Conformers, shielding tensor σ***																									***σ Boltz. avrg.***	***Comp. avrg. δ, ppm***	***Δδ, ppm***
		**73**	**282**	**18**	**25**	**305**	**13**	**281**	**36**	**57**	**34**	**56**	**267**	**70**	**234**	**46**	**68**	**300**	**14**	**247**	**226**	**217**	**222**	**167**	**279**	**280**			
H(3)	7.28	24.8	24.9	24.8	24.8	24.8	24.8	24.8	24.8	24.6	24.8	24.7	24.8	24.8	24.8	24.7	24.8	24.8	24.8	24.8	24.9	24.8	24.8	24.8	24.7	24.7	24.8	7.36	0.08
H(4)	6.64	25.3	25.4	25.2	25.3	25.5	25.2	25.5	25.2	25.4	25.1	25.4	25.4	25.3	25.5	25.3	25.3	25.3	25.2	25.1	25.6	25.5	25.5	25.4	25.5	25.3	25.3	6.84	0.20
H(5)	6.00	25.6	25.8	25.8	25.9	25.8	25.8	25.7	25.8	25.8	26.0	25.8	25.6	25.6	25.8	25.9	25.6	25.8	25.9	26.0	25.7	25.8	25.7	25.8	25.8	25.9	25.8	6.39	0.39
H(6a, H_{Si})	2.47	29.5	29.7	29.7	29.7	29.8	29.7	29.8	29.7	29.8	29.8	29.8	29.6	29.6	29.8	29.8	29.5	29.7	29.7	29.8	29.8	29.8	29.8	29.3	29.8	29.8	29.7	2.46	-0.01
H(6b, H_{Re})	2.73	29.4	29.2	29.2	29.2	29.2	29.2	29.2	29.2	29.2	29.2	29.2	29.5	29.5	29.2	29.2	29.5	29.2	29.2	29.2	29.2	29.2	29.2	29.6	29.2	29.2	29.3	2.89	0.16
H(7)	4.25	27.7	28.0	28.0	28.0	27.9	28.0	27.9	28.0	27.9	27.9	27.9	27.8	27.8	27.9	27.9	27.8	28.0	28.0	27.9	27.9	27.9	27.9	28.2	27.9	27.9	27.9	4.24	-0.01
H(9)	5.32	26.8	27.0	27.1	27.0	26.8	27.1	26.8	27.1	26.8	26.8	26.8	26.4	26.8	26.9	26.8	26.9	27.0	27.0	26.8	27.0	27.0	27.0	26.9	26.9	26.5	26.9	5.21	-0.11
H(10)	2.47	29.6	29.4	29.6	29.4	29.6	29.5	29.3	29.5	29.3	29.5	29.3	29.2	29.4	29.5	29.5	29.4	29.4	29.4	29.5	29.1	29.1	29.1	29.4	29.5	29.1	29.5	2.69	0.22
H(11)	3.41	28.6	28.9	28.6	28.9	28.6	28.6	29.1	28.8	29.1	28.6	29.1	28.3	28.8	28.6	28.6	28.7	28.8	28.8	28.6	29.1	29.1	29.1	28.4	28.6	28.1	28.7	3.43	0.02
H(13)	5.78	26.3	26.3	26.4	26.3	26.4	26.4	26.3	26.6	26.3	26.4	26.3	26.2	26.2	26.4	26.4	26.2	26.3	26.3	26.4	26.3	26.3	26.3	26.2	26.4	26.3	26.3	5.81	0.03
H(15)	5.63	26.6	26.6	26.7	26.6	26.2	26.6	26.7	26.6	26.7	26.2	26.7	26.5	26.6	26.2	26.1	26.6	26.6	26.7	26.2	26.6	26.5	26.6	26.5	26.2	26.1	26.6	5.60	-0.03
H(16a, H_{Si})	2.47	30.0	30.0	30.0	30.0	29.6	30.0	30.0	30.0	30.0	29.7	30.0	30.0	30.0	29.6	29.7	30.0	30.0	30.0	29.7	29.9	29.9	29.9	30.0	29.7	29.7	30.0	2.18	-0.29
H(16b, H_{Re})	2.73	29.3	29.4	29.4	29.4	29.5	29.4	29.4	29.4	29.4	29.6	29.4	29.3	29.3	29.5	29.5	29.3	29.4	29.4	29.6	29.4	29.4	29.4	29.4	29.5	29.5	29.4	2.75	0.03
H(17)	4.73	27.6	27.5	27.5	27.5	27.6	27.6	27.4	27.5	27.4	27.6	27.4	27.5	27.5	27.6	27.6	27.5	27.5	27.5	27.5	27.4	27.4	27.4	27.6	27.6	27.6	27.5	4.61	-0.12
H(18)	3.99	28.4	28.4	28.4	28.4	28.0	28.4	28.4	28.4	28.4	28.0	28.4	28.4	28.4	28.0	28.0	28.4	28.4	28.4	27.8	28.4	28.4	28.4	28.4	28.0	28.0	28.3	3.81	-0.18
H(19)-Me	1.18	31.1	31.3	31.1	31.1	30.8	31.1	31.1	31.1	31.1	30.8	31.3	31.3	31.1	31.4	30.8	31.1	31.1	31.1	30.8	31.1	31.1	31.2	31.3	30.8	30.8	31.1	1.03	-0.15
H(20a, H_{Re})	4.60[a]	28.0	28.1	28.0	27.7	28.2	28.0	28.2	28.0	27.7	28.0	27.7	27.7	28.1	28.2	27.8	28.1	27.7	28.0	28.0	28.2	27.7	28.2	27.7	28.1	27.8	28.0	4.15	-0.45
H(20b, H_{Si})	4.42[a]	27.7	28.3	27.7	28.1	28.2	27.7	28.3	27.7	28.1	27.7	28.1	28.1	27.7	28.2	28.1	27.7	28.1	27.7	27.6	28.2	28.2	28.3	28.2	28.2	28.1	28.0	4.18	-0.24
H(21)-Me	1.64	30.1	30.0	30.1	30.8	30.5	30.1	30.5	30.1	30.7	30.1	30.5	30.8	30.6	30.8	30.5	30.5	30.4	30.4	30.8	30.7	30.5	30.1	30.0	30.5	30.1	30.4	1.71	0.07
H(22a, H_{Re})	1.99	29.9	30.0	30.0	30.0	30.0	30.1	30.0	30.1	30.0	30.0	30.0	30.9	30.0	30.1	30.0	30.0	30.0	30.0	30.0	29.8	29.8	29.8	30.1	30.0	30.6	30.0	2.14	0.15
H(22b, H_{Si})	1.32	31.1	31.0	31.0	31.0	30.9	31.1	31.0	31.1	31.0	30.9	31.0	30.2	31.1	31.0	30.9	31.1	31.0	31.0	30.9	31.2	31.2	31.2	31.0	31.0	30.1	31.0	1.14	-0.18
H(23)-Me	0.82	31.3	31.4	31.3	31.4	31.4	31.3	31.2	31.2	31.3	31.2	31.3	31.5	31.3	31.2	31.2	31.3	31.2	31.2	31.2	31.2	31.3	31.3	31.3	31.2	31.4	31.3	0.85	0.03
H(24)-Me	1.87	30.3	29.9	30.4	30.8	30.1	30.3	30.2	30.2	30.3	30.1	30.2	30.4	29.8	30.6	30.0	29.8	30.8	29.9	30.1	30.1	30.9	30.1	30.3	30.6	30.0	30.3	1.89	0.02
H(25)-Me	1.72	30.0	30.3	30.0	30.0	30.5	30.0	30.1	30.3	30.1	30.9	30.6	30.2	30.0	30.9	31.0	30.3	30.3	30.3	31.1	30.4	30.4	30.5	30.3	30.5	30.9	30.3	1.88	0.16
H(1'')	4.81	27.2	27.1	27.3	27.1	27.3	27.5	27.3	27.6	27.3	27.3	27.3	27.4	27.3	27.4	27.3	27.3	27.3	27.3	27.3	27.3	27.3	27.2	27.3	27.4	27.2	27.3	4.90	0.09
H(2'')	3.93	28.3	28.4	28.3	28.4	28.3	28.1	28.4	28.3	28.4	28.3	28.4	28.1	28.2	28.1	28.3	28.2	28.1	28.2	28.3	28.5	28.5	28.5	28.4	28.1	28.3	28.3	3.83	-0.10
H(3'')	3.55	28.7	28.8	28.7	28.8	28.7	28.6	28.8	28.6	28.8	28.7	28.8	28.6	28.7	28.6	28.7	28.7	28.7	28.7	28.7	28.8	28.8	28.7	28.7	28.6	28.7	28.7	3.42	-0.13
H(4'')	3.46	28.8	28.7	28.8	28.7	28.8	28.6	28.7	28.5	28.7	28.8	28.7	28.6	28.5	28.6	28.8	28.5	28.5	28.5	28.8	28.7	28.7	28.7	28.8	28.6	28.8	28.7	3.42	-0.04
H(6''eq)-Me	1.22	31.2	30.6	31.2	30.6	30.8	31.1	31.1	31.1	30.6	30.8	31.1	31.0	30.6	31.0	30.7	30.6	31.0	30.6	30.8	31.1	30.9	30.8	31.0	30.6	30.7	30.9	1.24	0.03
H(6''ax)-Me	1.09	30.7	30.9	30.7	30.9	31.1	30.8	30.7	31.0	30.9	31.5	31.4	30.7	30.9	31.5	31.4	30.9	31.4	30.9	31.4	30.9	30.5	30.5	30.8	31.1	31.4	31.1	1.10	0.01
Energy (kJ/mol)		0.00	0.29	0.48	1.09	2.71	2.73	4.29	4.72	4.93	4.98	5.14	5.36	5.55	5.60	5.74	6.01	6.04	6.33	6.50	6.95	7.36	7.50	8.14	8.26	8.30	**DP4+ (^{1}H data)**		
Boltzmann factor		0.17	0.15	0.14	0.11	0.06	0.06	0.03	0.03	0.02	0.02	0.02	0.02	0.02	0.02	0.02	0.02	0.02	0.01	0.01	0.01	0.01	0.01	0.01	0.01	0.01	100.00%		

[a]H20a, H_{Re} and H20b, H_{Si} could not be unambiguously assigned and thus, the experimental chemical shift could be the other way round.

*Table 28: DP4+ results for **11R**: Computed σ for C-nuclei for each conformer and the averaged σ (conformers weighted based on Boltzmann factors) for simplified 11-desnoviosyl-11R-thio-(4"-desbutyryl)-β-D-noviosyl fidaxomicin **18e-C(11)S**. Computed chemical shifts δ were calculated using TMS as reference (σ_C(TMS): 190.0976; σ_H(TMS): 32.148). (PCM, solvent: acetone/B3LYP/6-31G(d))*

C-atom	Exp. δ, ppm	Conformers, shielding tensor σ																									σ Boltz. avrg.	Comp. avrg. δ, ppm	Δδ, ppm
		73	282	18	25	305	13	281	36	57	34	56	267	70	234	46	68	300	14	247	226	217	222	167	279	280			
C(1)	167.8	26.1	26.3	26.6	25.5	30.2	26.6	26.3	26.5	26.0	30.4	25.6	25.5	26.0	30.2	29.2	26.0	25.6	26.3	30.4	26.3	26.4	26.2	25.1	30.1	29.2	26.7	163.4	-4.4
C(2)	125.2	71.2	69.7	69.7	69.0	69.7	69.5	70.8	69.6	70.4	70.5	70.3	70.8	71.0	69.7	70.0	70.9	69.1	69.6	70.4	70.2	71.5	70.7	69.9	70.0	69.7	70.1	120.0	-5.2
C(3)	145.6	45.6	46.4	47.2	46.6	47.7	47.3	45.2	47.4	44.9	49.9	45.1	45.5	45.7	47.6	47.3	45.7	46.5	47.2	49.9	47.8	47.0	45.3	44.3	47.1	47.3	46.6	143.5	-2.1
C(4)	128.2	67.1	64.5	63.7	64.0	65.9	63.6	66.9	63.8	66.2	65.2	66.2	66.6	67.3	66.0	65.5	67.4	64.0	63.9	65.1	67.3	67.4	66.9	68.8	65.9	65.3	65.2	124.8	-3.4
C(5)	143.6	43.9	47.9	48.3	48.4	47.8	48.5	45.6	48.6	45.9	48.9	45.9	44.3	44.2	47.8	47.6	44.2	48.2	48.4	49.0	45.9	46.5	45.4	44.2	46.8	47.8	47.0	143.1	-0.5
C(6)	37.2	152.0	151.0	151.1	151.0	150.9	151.0	150.6	151.0	150.7	151.1	150.7	152.1	151.9	150.9	151.1	151.9	151.0	150.9	151.0	150.7	150.8	150.7	151.3	151.0	151.3	151.2	38.9	1.7
C(7)	72.9	116.0	117.3	117.6	117.3	117.1	117.6	117.2	117.6	117.3	117.1	117.2	115.9	116.0	117.1	116.9	115.9	117.4	117.3	117.1	117.4	117.4	117.4	117.8	117.0	116.7	117.1	73.0	0.1
C(8)	136.5	53.9	55.9	55.6	55.8	56.3	55.5	55.5	55.5	55.5	56.1	55.5	55.1	54.2	56.2	56.2	54.4	55.8	56.1	56.1	56.5	56.5	56.6	55.7	56.2	57.4	55.4	134.7	-1.8
C(9)	126.2	67.7	67.4	67.6	67.4	69.4	67.9	68.2	67.6	68.0	69.3	68.2	67.7	67.4	69.3	69.2	67.3	67.3	67.2	69.4	66.4	66.4	66.4	66.6	69.1	68.8	67.8	122.3	-3.9
C(10)	41.5	147.2	144.8	147.1	144.7	147.5	147.0	141.6	147.8	142.1	147.3	141.8	151.1	144.9	147.7	147.3	145.0	144.9	144.9	147.3	147.1	147.1	147.0	146.4	147.7	150.7	146.1	44.0	2.5
C(11)	62.8	117.5	119.9	118.1	120.0	120.0	119.2	129.3	120.8	129.5	120.2	129.1	122.5	120.0	120.4	120.1	121.1	119.7	121.4	120.5	124.1	124.0	124.3	121.2	120.4	125.7	120.2	69.9	7.1
C(12)	136.5	56.6	55.9	56.4	55.7	55.8	57.2	51.1	59.0	51.0	55.8	51.2	57.4	56.0	56.0	55.9	56.0	55.9	55.9	55.9	54.7	54.8	54.4	57.3	56.0	55.8	55.9	134.2	-2.3
C(13)	131.7	62.2	62.9	62.5	62.9	62.9	61.7	66.1	60.1	66.1	63.2	66.2	61.5	62.3	62.8	63.3	62.2	62.6	62.7	63.1	66.2	66.3	66.4	60.2	63.2	63.3	62.9	127.2	-4.5
C(14)	136.1	57.1	58.2	57.3	58.0	57.6	57.2	59.0	58.1	59.0	57.7	59.0	57.6	57.9	57.8	58.1	57.9	58.2	58.1	57.8	59.3	59.3	59.3	57.3	57.8	58.2	57.8	132.3	-3.8
C(15)	126.3	61.3	61.1	61.8	61.2	68.3	61.5	67.0	60.6	66.9	68.7	66.9	60.6	61.1	67.9	68.8	61.2	60.9	61.4	68.3	65.6	65.5	65.6	60.9	68.3	68.5	62.9	127.2	0.9
C(16)	28.4	156.5	156.5	156.7	156.5	161.2	156.6	156.0	156.6	156.1	160.7	156.1	156.6	156.5	161.2	160.2	156.5	156.6	156.6	160.8	155.9	155.8	155.8	156.2	160.8	160.1	157.1	33.0	4.6
C(17)	78.3	106.1	105.6	106.0	105.5	111.7	106.0	105.7	106.0	105.7	112.1	105.8	106.0	106.1	111.6	112.4	106.1	105.5	105.8	112.8	105.5	105.5	105.5	105.0	111.8	112.3	106.7	83.4	5.1
C(18)	67.6	121.0	121.1	121.0	121.1	123.6	121.0	120.7	120.9	120.7	123.6	120.6	121.1	121.0	123.4	123.7	121.0	121.1	121.0	123.7	120.5	120.5	120.6	121.5	123.5	123.7	121.3	68.8	1.2
C(19)	20.7	172.6	172.5	172.5	172.5	168.8	172.5	172.5	172.5	172.5	168.8	172.5	172.5	172.5	168.7	168.8	172.5	172.5	172.5	167.9	172.5	172.5	172.5	172.7	168.8	168.8	172.0	18.1	-2.6
C(20)	63.3	125.7	125.2	125.7	126.2	125.3	125.8	125.2	125.4	126.5	124.1	126.4	126.4	125.6	125.4	126.3	125.6	126.4	125.5	124.0	125.2	126.5	125.2	126.0	126.0	126.3	125.6	64.5	1.2
C(21)	15.1	174.0	173.8	173.9	173.8	174.7	173.9	174.3	173.8	174.4	174.6	174.3	174.9	174.0	174.6	174.6	174.0	173.8	173.9	174.6	174.3	174.3	174.4	172.4	174.5	175.2	174.1	16.0	0.9
C(22)	27.7	161.0	161.7	161.3	161.7	159.8	161.3	161.9	161.4	161.8	159.9	161.9	161.1	161.5	160.1	159.8	161.5	161.6	161.6	159.8	161.7	161.7	161.5	161.9	160.3	162.0	161.2	28.9	1.2
C(23)	10.9	177.5	177.2	177.3	177.2	177.5	177.3	177.2	177.4	177.3	177.5	177.2	182.5	177.4	177.5	177.5	177.3	177.3	177.2	177.5	177.5	177.5	177.5	177.3	177.5	182.1	177.5	12.6	1.7
C(24)	14.3	175.0	173.9	174.2	173.9	174.4	174.4	168.8	174.9	168.8	174.3	168.9	175.4	174.1	174.5	174.2	174.0	173.9	173.6	174.1	167.4	167.3	167.2	174.7	174.6	175.0	173.7	16.4	2.1
C(25)	17.6	173.0	173.2	173.1	173.2	172.9	173.2	172.1	173.2	172.1	173.0	172.0	173.1	173.1	172.8	172.5	173.0	173.2	172.9	173.0	171.4	171.3	171.4	173.2	172.8	172.3	172.9	17.2	-0.4
C(1'')	81.3	103.7	104.2	104.1	104.3	104.3	104.4	104.9	104.4	105.5	104.3	104.9	103.6	103.4	103.4	104.3	104.5	103.2	104.7	104.5	107.3	107.3	107.4	106.7	103.3	105.0	104.3	85.8	4.5
C(2'')	74.5	115.5	117.5	115.5	117.6	115.7	115.6	117.7	117.3	117.9	115.7	117.7	115.4	117.4	115.8	115.7	117.6	117.3	117.6	115.7	116.3	116.3	116.6	115.5	115.8	115.7	116.4	73.7	-0.8
C(3'')	72.3	118.9	118.8	118.9	118.8	118.9	119.8	118.9	119.1	118.8	118.9	118.8	119.7	119.6	119.8	118.9	119.5	119.7	119.5	119.0	118.9	118.9	118.9	119.0	119.9	118.9	119.0	71.1	-1.2
C(4'')	74.2	116.2	115.9	116.2	116.0	116.1	117.1	115.9	117.2	115.8	116.1	115.8	117.0	116.5	117.0	116.1	116.4	116.6	116.4	116.1	116.2	116.2	116.2	116.2	117.1	116.1	116.2	73.9	-0.3
C(5'')	77.7	112.1	112.3	112.2	112.3	111.9	113.0	112.3	112.5	112.2	111.9	112.3	113.0	112.7	112.6	111.9	112.5	112.8	112.6	111.8	112.0	112.0	112.0	112.5	112.5	111.8	112.3	77.8	0.1
C(6''eq)	28.8	161.8	161.6	161.8	161.5	161.6	161.6	161.6	161.7	161.7	161.6	161.6	161.7	161.4	161.6	161.6	161.5	161.4	161.6	161.8	161.2	161.2	161.0	161.6	161.7	161.7	161.6	28.5	-0.3
C(6''ax)	16.7	174.0	173.2	174.0	173.2	174.4	173.7	173.5	173.7	173.4	174.3	173.4	173.7	173.3	174.3	174.4	173.2	173.4	173.2	174.4	172.9	172.8	172.9	173.3	174.3	174.4	173.7	16.4	-0.3
**Energy (kJ/mol)**	0.00	0.29	0.48	1.09	2.71	2.73	4.29	4.72	4.93	4.98	5.14	5.36	5.55	5.60	5.74	6.01	6.04	6.33	6.50	6.95	7.36	7.50	8.14	8.26	8.30	**DP4+ (^{13}C data)**			
**Boltzmann factor**	0.17	0.15	0.14	0.11	0.06	0.06	0.03	0.03	0.02	0.02	0.02	0.02	0.02	0.02	0.02	0.02	0.02	0.01	0.01	0.01	0.01	0.01	0.01	0.01	0.01	99.99%			

Table 29: DP4+ results for ***11S****: Computed σ for H-nuclei for each conformer and the averaged σ (conformers weighted based on Boltzmann factors) for simplified 11-desnoviosyl-11S-thio-(4”-desbutyryl)-β-*D*-noviosyl fidaxomicin* ***18e-C(11)S****. Computed chemical shifts δ were calculated using TMS as a reference (σ_C(TMS): 190.0976; σ_H(TMS): 32.148). (PCM, solvent: acetone/B3LYP/6-31G(d))*

H-atom	***Exp. δ, ppm***	***Conformers, shielding tensor σ***										***σ Boltz.avrg.***	***Computed avrg. δ, ppm***	***Δδ, ppm***
		**26**	**43**	**75**	**14**	**22**	**51**	**36**	**29**	**78**	**34**			
H(3)	7.28	24.76	24.79	24.76	24.78	24.80	24.82	24.78	24.67	24.68	24.78	24.77	7.38	0.10
H(4)	6.64	25.33	25.23	25.34	25.34	25.23	25.21	25.35	25.31	25.23	25.35	25.30	6.85	0.21
H(5)	6.00	25.85	25.90	25.86	25.86	25.91	25.94	25.87	25.63	25.87	25.87	25.86	6.29	0.29
H(6a, H_{Si})	2.47	29.75	29.76	29.77	29.76	29.76	29.78	29.77	29.46	29.84	29.77	29.75	2.40	-0.07
H(6b, H_{Re})	2.73	29.20	29.21	29.22	29.20	29.21	29.24	29.22	29.45	29.21	29.22	29.22	2.93	0.21
H(7)	4.25	27.93	27.94	28.03	27.93	27.94	28.05	28.03	27.82	27.96	28.03	27.96	4.19	-0.06
H(9)	5.32	26.87	26.90	26.81	26.88	26.90	26.83	26.82	26.54	26.49	26.82	26.84	5.31	-0.01
H(10)	2.47	29.11	29.10	29.09	29.10	29.09	29.08	29.09	29.35	29.28	29.09	29.11	3.04	0.57
H(11)	3.41	28.37	28.45	28.17	28.30	28.34	28.14	28.11	28.42	28.16	28.11	28.31	3.84	0.43
H(13)	5.78	25.94	25.92	25.91	26.00	26.00	25.99	25.97	26.33	26.28	25.97	25.96	6.19	0.41
H(15)	5.63	26.62	26.64	26.62	26.67	26.69	26.69	26.66	26.65	26.83	26.66	26.64	5.50	-0.12
H(16a, H_{Si})	2.47	29.98	29.99	29.98	30.00	30.01	30.01	30.00	30.03	29.96	30.00	29.99	2.16	-0.31
H(16b, H_{Re})	2.73	29.41	29.39	29.40	29.42	29.41	29.40	29.41	29.06	29.39	29.41	29.39	2.76	0.03
H(17)	4.73	27.35	27.40	27.35	27.37	27.43	27.43	27.38	27.82	27.33	27.38	27.38	4.76	0.03
H(18)	3.99	28.35	28.36	28.35	28.36	28.37	28.37	28.36	27.89	28.39	28.36	28.34	3.81	-0.18
H(19)-Me	1.18	31.09	31.19	31.09	31.10	31.20	31.09	31.10	30.89	31.09	31.10	31.14	1.01	-0.17
H(20a, H_{Re})	4.60[a]	27.72	28.02	27.72	27.73	28.04	28.02	27.73	28.04	27.94	27.73	27.83	4.32	-0.28
H(20b, H_{Si})	4.42[a]	28.10	27.72	28.10	28.12	27.74	27.72	28.12	27.79	27.75	28.12	27.97	4.18	-0.24
H(21)-Me	1.64	30.10	30.09	30.85	30.09	30.09	30.87	30.85	30.89	30.83	30.85	30.48	1.67	0.03
H(22a, H_{Re})	1.99	30.53	30.54	30.33	30.50	30.51	30.30	30.30	30.54	30.39	30.30	30.46	1.69	-0.30
H(22b, H_{Si})	1.32	30.33	30.36	30.85	30.30	30.31	30.84	30.83	30.45	30.82	30.84	30.52	1.63	0.32
H(23)-Me	0.82	31.25	31.25	31.30	31.24	31.24	30.83	31.13	31.23	31.14	31.13	31.20	0.94	0.12
H(24)-Me	1.87	29.85	29.86	29.89	29.86	29.88	29.92	29.81	30.32	30.56	30.84	30.17	1.98	0.11
H(25)-Me	1.72	30.24	30.24	30.08	30.25	30.25	30.24	30.25	30.41	30.67	30.09	30.22	1.93	0.21
H(1'')	4.81	27.42	27.46	27.36	27.58	27.59	27.52	27.51	27.37	27.51	27.51	27.44	4.71	-0.10
H(2'')	3.93	28.42	28.42	28.39	28.18	28.18	28.15	28.15	28.42	28.20	28.15	28.36	3.79	-0.14
H(3'')	3.55	28.77	28.77	28.75	28.69	28.68	28.67	28.67	28.74	28.67	28.67	28.75	3.40	-0.15
H(4'')	3.46	28.73	28.74	28.72	28.57	28.56	28.55	28.56	28.72	28.54	28.55	28.69	3.45	-0.01
H(6''eq)-Me	1.22	31.14	30.62	31.13	31.06	30.61	30.61	31.05	31.13	31.05	31.05	30.90	1.25	0.04
H(6''ax)-Me	1.09	30.98	30.97	30.74	30.99	30.98	30.98	31.54	31.52	30.74	30.75	31.09	1.06	-0.03
Energy (kJ/mol)		0.00	0.88	0.91	5.00	5.15	5.46	5.74	5.75	5.76	5.76	**DP4+ (^{1}H data)**		
Boltzmann factor		0.32	0.22	0.22	0.04	0.04	0.04	0.03	0.03	0.03	0.03	0.00%		

[a]H20a, H_{Re} and H20b, H_{Si} could not be unambiguously assigned and thus, the experimental chemical shift could be the other way round.

*Table 30: DP4+ results for **11S**: Computed σ for C-nuclei for each conformer and the averaged σ (conformers weighted based on the Boltzmann factors) for simplified 11-desnoviosyl-11S-thio-(4"-desbutyryl)-β-D-noviosyl fidaxomicin **18e-C(11)S**. Computed chemical shifts δ were calculated using TMS as a reference (σ_C(TMS): 190.0976; σ_H(TMS): 32.148). (PCM, solvent: acetone/B3LYP/6-31G(d))*

C-atom	***Exp. δ, ppm***	***Conformers, shielding tensor σ***										***σ Boltz.avrg.***	***Comp. avrg. δ, ppm***	***Δδ, ppm***
		**26**	**43**	**75**	**14**	**22**	**51**	**36**	**29**	**78**	**34**			
C(1)	167.8	25.5	26.4	25.5	25.6	26.4	26.5	25.6	27.6	26.9	25.6	25.9	164.2	-3.6
C(2)	125.2	69.8	70.0	69.7	69.8	70.0	70.0	69.7	70.1	70.7	69.7	69.9	120.2	-5.0
C(3)	145.6	45.8	46.4	45.9	45.8	46.4	46.9	45.9	46.0	46.7	45.9	46.0	144.1	-1.5
C(4)	128.2	65.2	64.9	65.1	65.3	64.9	64.6	65.1	67.1	65.8	65.2	65.2	124.9	-3.3
C(5)	143.6	47.0	47.4	47.1	47.0	47.4	47.8	47.1	46.8	46.8	47.1	47.1	143.0	-0.6
C(6)	37.2	151.0	150.9	151.1	151.0	150.9	151.1	151.1	148.9	150.8	151.1	150.9	39.2	2.0
C(7)	72.9	117.1	117.2	117.2	117.1	117.2	117.2	117.2	116.5	117.1	117.2	117.1	73.0	0.1
C(8)	136.5	54.0	54.1	56.8	54.1	54.1	56.8	56.9	54.2	56.6	56.9	55.0	135.1	-1.4
C(9)	126.2	70.8	70.7	69.4	70.7	70.8	69.4	69.3	70.8	69.0	69.3	70.2	119.8	-6.4
C(10)	41.5	147.1	146.9	147.6	147.1	147.0	147.4	147.6	142.1	140.6	147.6	146.9	43.2	1.7
C(11)	62.8	122.3	121.2	129.1	122.0	121.5	128.5	128.8	122.4	129.6	128.8	124.4	65.7	2.9
C(12)	136.5	60.3	59.6	59.7	60.2	59.8	59.4	59.6	54.0	53.5	59.6	59.5	130.6	-5.9
C(13)	131.7	63.1	63.2	63.1	63.2	63.2	63.2	63.1	63.2	63.2	63.1	63.1	127.0	-4.7
C(14)	136.1	58.8	58.7	58.6	58.8	58.7	58.5	58.6	59.9	59.9	58.6	58.8	131.3	-4.8
C(15)	126.3	60.9	61.2	61.3	61.0	61.3	61.7	61.5	65.8	67.0	61.4	61.5	128.6	2.3
C(16)	28.4	157.0	156.8	156.9	157.0	156.9	156.8	156.9	163.3	156.3	156.9	157.1	33.0	4.6
C(17)	78.3	105.4	105.7	105.4	105.4	105.7	105.7	105.5	102.0	105.5	105.5	105.4	84.7	6.4
C(18)	67.6	121.2	121.0	121.1	121.2	121.1	121.0	121.1	121.5	120.4	121.1	121.1	69.0	1.4
C(19)	20.7	172.4	172.4	172.4	172.5	172.5	172.4	172.4	169.7	172.5	172.4	172.4	17.7	-3.0
C(20)	63.3	126.7	125.8	126.7	126.7	125.8	125.6	126.7	126.5	125.5	126.7	126.4	63.7	0.4
C(21)	15.1	174.8	174.7	175.4	174.8	174.7	175.3	175.4	175.1	175.8	175.4	175.0	15.1	0.0
C(22)	27.7	157.7	157.6	160.8	157.7	157.6	160.7	160.8	158.7	161.3	160.8	158.8	31.3	3.6
C(23)	10.9	176.9	177.0	178.2	176.9	176.9	178.1	178.2	177.2	178.1	178.2	177.4	12.7	1.8
C(24)	14.3	169.0	168.9	169.0	169.0	169.0	168.9	169.0	169.3	169.7	169.0	169.0	21.1	6.8
C(25)	17.6	172.8	172.7	172.7	172.8	172.7	172.6	172.6	172.1	172.4	172.6	172.7	17.4	-0.2
C(1'')	81.3	108.0	106.7	107.7	107.0	106.1	106.1	106.7	108.3	107.2	106.7	107.4	82.7	1.4
C(2'')	74.5	117.4	117.2	117.3	117.1	117.0	116.9	117.0	116.8	116.8	117.0	117.2	72.9	-1.6
C(3'')	72.3	118.8	118.8	118.8	119.8	119.8	119.9	119.8	118.7	119.7	119.8	119.0	71.1	-1.2
C(4'')	74.2	115.8	115.9	115.9	116.4	116.6	116.6	116.4	115.8	116.5	116.4	116.0	74.1	-0.1
C(5'')	77.7	112.2	112.3	112.1	112.9	112.9	112.7	112.7	112.3	112.7	112.7	112.3	77.8	0.1
C(6''eq)	28.8	161.7	161.7	161.5	161.6	161.6	161.4	161.4	161.8	161.4	161.4	161.6	28.5	-0.3
C(6''ax)	16.7	173.5	173.6	173.6	173.6	173.7	173.7	173.7	173.3	173.7	173.7	173.6	16.5	-0.2
Energy (kJ/mol)		0.00	0.88	0.91	5.00	5.15	5.46	5.74	5.75	5.76	5.76	**DP4+ (^{13}C data)**		
Boltzmann factor		0.32	0.22	0.22	0.04	0.04	0.04	0.03	0.03	0.03	0.03	0.01%		

11-Desnoviosyl-13-thio-(4”-desbutyryl)-β-D-noviosyl fidaxomicin simplified (**18e-C(13)**)

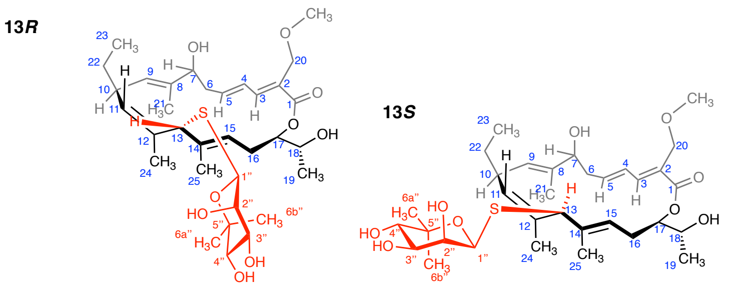

Table 31: Experimental vs. computed ^{1}H chemical shifts and DP4+ results for simplified 11-desnoviosyl-13-thio-(4”-desbutyryl)-β-D-noviosyl fidaxomicin ***18e-C(13)*** *(PCM, solvent: acetone/B3LYP/6-31G(d))*

H-atom	Exp. δ, ppm	13*R*		13S	
		Comp. δ, ppm	Δδ, ppm	Comp. δ, ppm	Δδ, ppm
H(3)	7.20	7.28	0.08	7.34	0.14
H(4)	6.60	6.73	0.13	6.73	0.13
H(5)	6.06	6.41	0.35	6.43	0.37
H(6a, H_{Si})	2.51	2.36	-0.14	2.36	-0.14
H(6b, H_{Re})	2.74	2.89	0.16	2.88	0.14
H(7)	4.31	4.24	-0.07	4.26	-0.05
H(9)	5.33	5.33	0.00	5.23	-0.10
H(10)	3.20	3.38	0.18	3.28	0.08
H(11)	5.38	5.53	0.15	5.53	0.15
H(13)	3.86	3.96	0.10	3.86	0.00
H(15)	5.61	5.98	0.38	5.72	0.11
H(16a, H_{Si})	2.51	2.32	-0.19	2.46	-0.04
H(16b, H_{Re})	2.51	2.50	0.00	2.49	-0.02
H(17)	4.83	4.67	-0.16	4.64	-0.19
H(18)	3.88	4.01	0.13	4.02	0.14
H(19)-Me	1.15	1.05	-0.10	1.06	-0.09
H(20a, H_{Re})	4.49[a]	4.17	-0.32	4.20	-0.29
H(20b, H_{Si})	4.62[a]	4.14	-0.48	4.13	-0.49
H(21)-Me	1.71	1.73	0.02	1.71	0.00
H(22a, H_{Re})	1.55	1.54	-0.01	1.59	0.04
H(22b, H_{Si})	1.37	1.46	0.09	1.43	0.06
H(23)-Me	0.90	0.95	0.05	0.93	0.03
H(24)-Me	1.58	1.80	0.22	1.73	0.15
H(25)-Me	1.51	1.50	-0.01	1.51	0.00
H(1")	4.73	4.60	-0.13	4.74	0.01
H(2")	3.78	3.70	-0.08	3.74	-0.04
H(3")	3.56	3.38	-0.18	3.43	-0.13
H(4")	3.56	3.46	-0.10	3.47	-0.09
H(6"eq)-Me	1.24	1.28	0.04	1.25	0.01
H(6"ax)-Me	1.21	1.15	-0.06	1.17	-0.04
**DP4+ (^{1}H data)**		**0.00%**		**100.00%**	
**DP4+ (all data)**		**0.00%**		**100.00%**	

[a]H20a, H_{Re} and H20b, H_{Si} could not be unambiguously assigned and thus, the experimental chemical shift could be the other way round.

13*R*

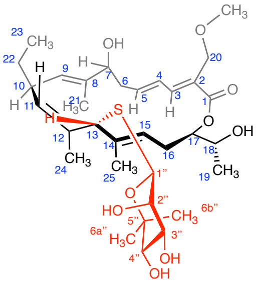

13*S*

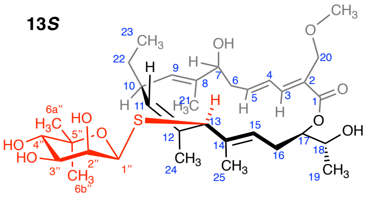

Table 32: Experimental vs. computed ^{13}C chemical shifts and DP4+ results for simplified 11-desnoviosyl-13-thio-(4"-desbutyryl)-β-D-noviosyl fidaxomicin ***18e-C(13)*** *(PCM, solvent: acetone/B3LYP/6-31G(d))*

C-atom	Exp. δ, ppm	13*R*		13S	
		Comp. δ, ppm	Δδ, ppm	Comp. δ, ppm	Δδ, ppm
C(1)	166.6	160.0	-6.6	161.1	-5.5
C(2)	125.2	120.1	-5.1	120.2	-5.0
C(3)	144.9	141.5	-3.4	141.7	-3.2
C(4)	127.9	123.4	-4.5	123.3	-4.6
C(5)	143.4	142.9	-0.5	143.1	-0.3
C(6)	37.6	38.7	1.1	38.8	1.2
C(7)	72.8	73.0	0.2	72.8	0.0
C(8)	134.6	130.4	-4.2	131.6	-3.0
C(9)	126.7	122.1	-4.6	121.5	-5.2
C(10)	39.1	40.7	1.6	40.4	1.3
C(11)	134.5	128.5	-6.0	131.5	-3.0
C(12)	135.1	131.9	-3.2	130.6	-4.5
C(13)	60.4	63.8	3.4	66.0	5.6
C(14)	137.6	134.0	-3.6	135.1	-2.5
C(15)	121.4	122.9	1.5	118.7	-2.7
C(16)	29.1	31.4	2.3	31.1	2.0
C(17)	78	77.5	-0.5	79.7	1.7
C(18)	66.6	66.7	0.1	67.5	0.9
C(19)	21.3	21.0	-0.3	20.3	-1.0
C(20)	62.6	64.2	1.6	64.0	1.4
C(21)	14.8	15.0	0.2	15.1	0.3
C(22)	30.2	31.4	1.2	31.8	1.6
C(23)	12	13.0	1.0	12.8	0.8
C(24)	12	17.7	5.7	12.4	0.4
C(25)	17.2	11.4	-5.8	17.0	-0.2
C(1")	78.1	82.5	4.4	83.0	4.9
C(2")	74.2	73.2	-1.0	73.2	-1.0
C(3")	72.6	71.0	-1.6	71.1	-1.5
C(4")	74.4	74.1	-0.3	74.1	-0.3
C(5")	77.8	77.7	-0.1	77.7	-0.1
C(6"eq)	28.8	28.5	-0.3	28.5	-0.3
C(6"ax)	18.2	17.0	-1.2	17.3	-0.9
**DP4+ (^{13}C data)**		**0.00%**		**100.00%**	
**DP4+ (all data)**		**0.00%**		**100.00%**	

*Table 33: DP4+ results for **13R**: Computed σ for H-nuclei for each conformer and the averaged σ (conformers weighted based on Boltzmann factors) for simplified 11-desnoviosyl-13R-thio-(4"-desbutyryl)-β-D-noviosyl fidaxomicin **18e-C(13)**. Computed chemical shifts δ were calculated using TMS as a reference (σ_C(TMS): 190.0976; σ_H(TMS): 32.148). (PCM, solvent: acetone/B3LYP/6-31G(d)). Total amount of conformers: 53.*

**H-atom**	**Exp. δ, ppm**	**Conformers, shielding tensor σ**															**σ Boltz. avrg.**	**Comp. avrg. δ, ppm**	**Δδ, ppm**
		**200**	**300**	**253**	**62**	**56**	**101**	**60**	…	**128**	**96**	**193**	**140**	**237**	**132**	**211**			
H(3)	7.20	24.93	24.96	24.88	24.84	24.86	24.84	24.87	…	24.85	24.88	24.82	24.82	24.86	24.82	24.86	24.87	7.28	0.08
H(4)	6.60	25.54	25.54	25.56	25.37	25.35	25.36	25.31	…	25.37	25.35	25.41	25.35	25.36	25.35	25.36	25.42	6.73	0.13
H(5)	6.06	25.74	25.75	25.75	25.82	25.84	25.82	25.86	…	25.83	25.85	25.64	25.66	25.83	25.66	25.83	25.74	6.41	0.35
H(6a, H_{Si})	2.51	29.85	29.86	29.86	29.86	29.86	29.84	29.84	…	29.86	29.86	29.67	29.67	29.84	29.67	29.84	29.79	2.36	-0.14
H(6b, H_{Re})	2.74	29.19	29.20	29.19	29.19	29.19	29.18	29.18	…	29.20	29.20	29.38	29.38	29.18	29.38	29.18	29.26	2.89	0.16
H(7)	4.31	27.97	27.98	27.97	27.97	27.98	27.90	27.91	…	27.98	27.99	27.73	27.73	27.91	27.73	27.91	27.91	4.24	-0.07
H(9)	5.33	26.83	26.85	26.85	26.81	26.83	26.90	26.91	…	26.83	26.84	26.84	26.85	26.91	26.85	26.91	26.82	5.33	0.00
H(10)	3.20	28.78	28.78	28.79	28.77	28.78	28.78	28.79	…	28.78	28.79	28.73	28.74	28.78	28.74	28.78	28.77	3.38	0.18
H(11)	5.38	26.64	26.64	26.65	26.62	26.63	26.58	26.57	…	26.68	26.65	26.51	26.55	26.57	26.55	26.57	26.62	5.53	0.15
H(13)	3.86	28.20	28.19	28.20	28.19	28.20	28.20	28.19	…	28.20	28.15	28.10	28.15	28.13	28.14	28.13	28.18	3.96	0.10
H(15)	5.61	26.16	26.15	26.16	26.14	26.16	26.14	26.15	…	26.19	26.16	26.12	26.14	26.13	26.14	26.13	26.16	5.98	0.38
H(16a, H_{Si})	2.51	29.84	29.87	29.87	29.84	29.91	29.85	29.91	…	29.83	29.89	29.86	29.93	29.83	29.93	29.83	29.83	2.32	-0.19
H(16b, H_{Re})	2.51	29.63	29.63	29.65	29.63	29.64	29.62	29.64	…	29.67	29.69	29.60	29.60	29.66	29.60	29.66	29.65	2.50	0.00
H(17)	4.83	27.50	27.51	27.55	27.53	27.51	27.53	27.51	…	27.40	27.39	27.50	27.47	27.39	27.47	27.39	27.48	4.67	-0.16
H(18)	3.88	28.17	28.17	28.17	28.17	28.17	28.17	28.17	…	27.95	27.95	28.16	28.16	27.96	28.16	27.96	28.14	4.01	0.13
H(19)-Me	1.15	30.94	30.88	30.89	31.47	30.90	30.96	30.91	…	31.28	30.93	30.96	30.88	31.08	31.45	30.99	31.09	1.05	-0.10
H(20a, H_{Re})	4.49[a]	28.22	28.21	28.05	27.82	27.97	27.82	27.97	…	27.82	27.96	27.80	27.99	27.82	27.99	27.82	27.98	4.17	-0.32
H(20b, H_{Si})	4.62[a]	28.25	28.19	28.21	28.04	27.76	28.05	27.75	…	28.04	27.76	28.05	27.73	28.04	27.73	28.04	28.00	4.14	-0.48
H(21)-Me	1.71	30.00	30.81	30.01	30.80	30.40	29.97	30.40	…	30.80	30.41	30.78	30.47	29.97	29.93	30.76	30.42	1.73	0.02
H(22a, H_{Re})	1.55	30.61	30.61	30.62	30.60	30.61	30.55	30.56	…	30.61	30.63	30.55	30.54	30.56	30.54	30.56	30.60	1.54	-0.01
H(22b, H_{Si})	1.37	30.67	30.68	30.68	30.66	30.67	30.69	30.70	…	30.66	30.68	30.75	30.76	30.71	30.76	30.71	30.69	1.46	0.09
H(23)-Me	0.90	31.33	31.33	31.09	31.08	31.15	31.29	31.13	…	31.07	31.33	31.18	31.32	31.29	31.19	31.13	31.20	0.95	0.05
H(24)-Me	1.58	30.42	30.52	30.52	30.41	30.42	30.06	30.42	…	30.54	30.53	30.46	30.37	30.51	30.37	30.43	30.35	1.80	0.22
H(25)-Me	1.51	30.49	30.90	30.79	30.45	30.74	30.80	30.52	…	30.85	30.92	30.68	30.74	30.62	30.68	30.53	30.65	1.50	-0.01
H(1")	4.73	27.50	27.52	27.53	27.51	27.52	27.52	27.53	…	27.66	27.70	27.70	27.71	27.71	27.71	27.71	27.54	4.60	-0.13
H(2")	3.78	28.49	28.50	28.51	28.50	28.50	28.51	28.51	…	28.28	28.26	28.26	28.27	28.26	28.27	28.26	28.45	3.70	-0.08
H(3")	3.56	28.78	28.78	28.79	28.78	28.79	28.79	28.79	…	28.69	28.70	28.70	28.70	28.71	28.70	28.71	28.76	3.38	-0.18
H(4")	3.56	28.72	28.73	28.73	28.72	28.73	28.73	28.74	…	28.54	28.55	28.55	28.55	28.56	28.55	28.56	28.69	3.46	-0.10
H(6"eq)-Me	1.24	31.10	30.61	30.91	31.10	30.61	30.90	31.11	…	30.93	31.04	30.58	31.02	31.03	30.58	30.92	30.87	1.28	0.04
H(6"ax)-Me	1.21	30.70	30.93	31.35	30.93	30.93	30.71	31.34	…	31.42	30.72	30.72	31.36	30.73	30.93	31.41	30.99	1.15	-0.06
**Energy (kJ/mol)**		0.00	0.75	1.24	1.39	1.68	1.88	2.16	…	7.99	8.07	8.10	8.34	8.34	8.34	8.35	**DP4+ (^{1}H data)**		
**Boltzmann factor**		0.11	0.08	0.07	0.06	0.05	0.05	0.05	…	0.004	0.004	0.004	0.004	0.004	0.004	0.004	0.00%		

[a]H20a, H_{Re} and H20b, H_{Si} could not be unambiguously assigned and thus, the experimental chemical shift could be the other way round.

*Table 34: DP4+ results for **13R**: Computed σ for C-nuclei for each conformer and the averaged σ (conformers weighted based on Boltzmann factors) for simplified 11-desnoviosyl-13R-thio-(4”-desbutyryl)-β-D-noviosyl fidaxomicin **18e-C(13)**. Computed chemical shifts δ were calculated using TMS as a reference (σ_C(TMS): 190.0976; σ_H(TMS): 32.148). (PCM, solvent: acetone/B3LYP/6-31G(d)). Total amount of conformers: 53.*

C-atom	***Exp. δ, ppm***	***Conformers, shielding tensor σ***															***σ Boltz. avrg.***	***Comp. avrg. δ, ppm***	***Δδ, ppm***
		**200**	**300**	**253**	**62**	**56**	**101**	**60**	…	**128**	**96**	**193**	**140**	**237**	**132**	**211**			
C(1)	166.6	30.7	30.7	30.7	30.1	30.7	30.1	30.8	…	30.2	30.8	29.9	30.7	30.2	30.7	30.2	30.1	160.0	-6.6
C(2)	125.2	69.8	69.7	69.7	69.6	69.4	69.6	69.5	…	69.6	69.4	70.8	70.8	69.6	70.8	69.6	70.0	120.1	-5.1
C(3)	144.9	49.1	49.9	48.4	48.2	49.4	48.2	49.4	…	48.2	49.3	47.4	48.1	48.2	48.0	48.2	48.6	141.5	-3.4
C(4)	127.9	66.6	66.8	66.1	65.9	65.4	65.9	65.5	…	65.9	65.5	68.1	67.9	65.9	68.0	65.9	66.7	123.4	-4.5
C(5)	143.4	47.8	47.8	47.3	47.9	48.3	48.0	48.5	…	47.9	48.2	45.3	45.5	48.0	45.5	48.0	47.2	142.9	-0.5
C(6)	37.6	151.1	151.1	151.2	151.1	151.1	151.0	150.9	…	151.1	151.1	152.5	152.4	151.0	152.4	151.0	151.4	38.7	1.1
C(7)	72.8	117.4	117.4	117.5	117.4	117.5	117.3	117.4	…	117.4	117.5	115.8	115.8	117.3	115.8	117.3	117.1	73.0	0.2
C(8)	134.6	60.5	60.6	60.4	60.4	60.4	58.2	58.3	…	60.5	60.4	57.3	57.3	58.2	57.4	58.2	59.7	130.4	-4.2
C(9)	126.7	67.5	67.5	67.5	67.5	67.5	68.9	68.9	…	67.5	67.5	69.4	69.3	68.9	69.3	68.9	68.0	122.1	-4.6
C(10)	39.1	149.2	149.2	149.2	149.2	149.2	149.6	149.6	…	149.2	149.2	149.9	149.9	149.6	149.9	149.6	149.4	40.7	1.6
C(11)	134.5	61.8	61.7	61.8	61.7	61.6	61.2	61.1	…	61.8	61.6	60.8	60.9	61.1	60.9	61.1	61.5	128.5	-6.0
C(12)	135.1	57.7	57.6	57.7	57.6	57.6	59.7	59.7	…	57.6	57.7	59.4	59.2	59.8	59.2	59.8	58.2	131.9	-3.2
C(13)	60.4	126.2	126.2	126.2	126.2	126.3	126.6	127.0	…	125.7	126.0	126.4	126.4	126.4	126.5	126.4	126.3	63.8	3.4
C(14)	137.6	55.8	56.0	55.9	56.0	56.0	56.3	56.4	…	55.4	56.2	56.7	56.4	56.6	56.4	56.6	56.1	134.0	-3.6
C(15)	121.4	67.2	67.2	67.1	67.2	67.3	67.1	67.2	…	67.3	66.7	66.7	67.1	66.5	67.1	66.4	67.2	122.9	1.5
C(16)	29.1	158.9	159.0	159.2	159.0	158.9	159.0	158.9	…	159.1	159.1	158.9	158.7	159.1	158.7	159.1	158.7	31.4	2.3
C(17)	78	112.9	113.1	112.6	113.0	113.2	113.1	113.3	…	113.8	113.8	113.4	113.8	114.0	113.8	114.0	112.6	77.5	-0.5
C(18)	66.6	123.7	123.7	123.7	123.7	123.7	123.7	123.7	…	123.6	123.6	123.6	123.6	123.6	123.6	123.6	123.4	66.7	0.1
C(19)	21.3	169.1	169.1	169.1	169.1	169.2	169.1	169.2	…	168.2	168.2	169.2	169.3	168.2	169.3	168.2	169.1	21.0	-0.3
C(20)	62.6	125.2	125.3	125.3	126.6	126.0	126.6	125.8	…	126.6	126.0	126.5	125.9	126.6	125.9	126.6	125.9	64.2	1.6
C(21)	14.8	175.2	175.2	175.2	175.2	175.2	174.6	174.6	…	175.2	175.2	174.6	174.6	174.6	174.6	174.6	175.1	15.0	0.2
C(22)	30.2	158.7	158.7	158.7	158.7	158.7	159.1	159.1	…	158.7	158.7	158.9	158.9	159.1	158.9	159.1	158.7	31.4	1.2
C(23)	12	177.0	177.0	177.0	176.9	176.9	177.6	177.6	…	176.9	176.9	177.7	177.7	177.5	177.7	177.5	177.1	13.0	1.0
C(24)	12	172.5	172.4	172.4	172.4	172.4	172.3	172.2	…	172.6	172.4	172.1	172.1	172.2	172.1	172.2	172.4	17.7	5.7
C(25)	17.2	178.6	178.7	178.5	178.6	178.7	178.6	178.7	…	178.5	178.7	178.8	178.8	178.8	178.7	178.8	178.7	11.4	-5.8
C(1")	78.1	107.7	107.8	107.7	107.7	107.9	107.7	108.1	…	106.4	107.0	106.9	106.8	107.0	106.8	107.0	107.6	82.5	4.4
C(2")	74.2	116.9	117.0	116.9	117.0	117.0	117.0	117.1	…	116.7	116.7	116.6	116.7	116.6	116.7	116.6	116.9	73.2	-1.0
C(3")	72.6	118.9	118.9	118.9	118.9	118.9	118.9	118.9	…	119.9	119.8	119.7	119.8	119.8	119.8	119.8	119.1	71.0	-1.6
C(4")	74.4	115.8	115.8	115.8	115.8	115.8	115.8	115.9	…	116.7	116.5	116.5	116.5	116.5	116.5	116.5	116.0	74.1	-0.3
C(5")	77.8	112.3	112.3	112.3	112.2	112.3	112.3	112.3	…	112.9	112.9	112.9	113.0	112.9	113.0	112.9	112.4	77.7	-0.1
Energy (kJ/mol)		0.00	0.75	1.24	1.39	1.68	1.88	2.16	…	7.99	8.07	8.10	8.34	8.34	8.34	8.35	**DP4+ (^{13}C data)**		
Boltzmann factor		0.11	0.08	0.07	0.06	0.05	0.05	0.05	…	0.004	0.004	0.004	0.004	0.004	0.004	0.004	0.00%		

*Table 35: DP4+ results for **13S**: Computed σ for H-nuclei for each conformer and the averaged σ (conformers weighted based on Boltzmann factors) for simplified 11-desnoviosyl-13S-thio-(4”-desbutyryl)-β-D-noviosyl fidaxomicin **18e-C(13)**. Computed chemical shifts δ were calculated using TMS as a reference (σ_C(TMS): 190.0976; σ_H(TMS): 32.148). (PCM, solvent: acetone/B3LYP/6-31G(d)). Total amount of conformers: 46.*

H-atom	***Exp. δ, ppm***	***Conformers, shielding tensor σ***															***σ Boltz. avrg.***	***Comp. avrg. δ, ppm***	***Δδ, ppm***
		**197**	**327**	**425**	**74**	**37**	**55**	**46**	…	**393**	**377**	**198**	**268**	**174**	**34**	**132**			
H(3)	7.2	24.80	24.88	24.85	24.76	24.77	24.77	24.79	…	24.91	24.84	24.83	24.73	24.75	24.74	24.79	24.81	7.34	0.14
H(4)	6.6	25.36	25.55	25.54	25.34	25.36	25.35	25.39	…	25.56	25.31	25.39	25.41	25.43	25.31	25.38	25.41	6.73	0.13
H(5)	6.06	25.82	25.68	25.69	25.79	25.78	25.80	25.80	…	25.72	25.91	25.81	25.58	25.63	25.50	25.80	25.72	6.43	0.37
H(6a, H_{Si})	2.51	29.81	29.85	29.86	29.86	29.86	29.88	29.89	…	29.88	29.84	29.88	29.97	29.69	29.43	29.86	29.78	2.36	-0.14
H(6b, H_{Re})	2.74	29.20	29.21	29.20	29.19	29.19	29.21	29.21	…	29.23	29.22	29.21	29.34	29.38	29.53	29.20	29.27	2.88	0.14
H(7)	4.31	27.92	27.89	27.90	27.89	27.89	27.97	27.98	…	27.98	28.01	27.98	27.74	27.73	27.77	27.90	27.88	4.26	-0.05
H(9)	5.33	26.96	26.98	26.99	26.94	26.97	26.86	26.92	…	26.91	26.96	26.86	26.61	26.90	26.85	26.94	26.92	5.23	-0.10
H(10)	3.2	28.80	28.90	28.87	28.85	28.87	28.88	28.89	…	28.94	28.90	28.89	28.80	28.79	28.93	28.86	28.86	3.28	0.08
H(11)	5.38	26.79	26.60	26.60	26.57	26.60	26.58	26.60	…	26.62	26.76	26.43	26.54	26.51	26.53	26.54	26.62	5.53	0.15
H(13)	3.86	28.04	28.43	28.43	28.40	28.42	28.41	28.42	…	28.39	27.75	27.41	28.37	28.18	28.22	28.22	28.29	3.86	0.00
H(15)	5.61	26.56	26.40	26.41	26.38	26.43	26.38	26.40	…	26.41	26.59	25.69	26.35	26.32	26.57	26.34	26.43	5.72	0.11
H(16a, H_{Si})	2.51	29.93	29.62	29.70	29.69	29.74	29.72	29.76	…	29.62	29.84	29.74	29.73	29.71	29.35	29.68	29.69	2.46	-0.04
H(16b, H_{Re})	2.51	29.71	29.62	29.61	29.60	29.63	29.62	29.63	…	29.68	29.64	29.57	29.61	29.60	29.80	29.68	29.66	2.49	-0.02
H(17)	4.83	27.25	27.66	27.64	27.65	27.65	27.63	27.63	…	27.48	27.27	27.56	27.59	27.59	27.35	27.47	27.51	4.64	-0.19
H(18)	3.88	28.39	28.10	28.10	28.09	28.10	28.11	28.11	…	27.90	28.37	28.19	28.10	28.09	28.09	27.90	28.13	4.02	0.14
H(19)-Me	1.15	31.32	31.45	30.86	31.47	30.82	31.46	31.44	…	31.05	31.25	31.48	30.89	31.45	31.01	31.25	31.09	1.06	-0.09
H(20a, H_{Re})	4.49[a]	27.73	28.21	28.22	27.82	27.99	27.83	27.99	…	28.22	27.96	27.84	27.83	27.81	27.99	27.83	27.95	4.20	-0.29
H(20b, H_{Si})	4.62[a]	28.02	28.23	28.23	28.03	27.79	28.03	27.79	…	28.20	27.76	28.06	28.03	28.09	27.79	28.08	28.02	4.13	-0.49
H(21)-Me	1.71	30.05	30.74	30.44	29.99	30.75	30.79	30.80	…	30.80	30.05	30.42	29.97	29.97	30.17	30.45	30.44	1.71	0.00
H(22a, H_{Re})	1.55	30.68	30.46	30.46	30.45	30.46	30.62	30.64	…	30.64	30.76	30.61	30.61	30.45	30.56	30.47	30.55	1.59	0.04
H(22b, H_{Si})	1.37	30.69	30.70	30.71	30.69	30.71	30.70	30.71	…	30.73	30.73	30.68	30.68	30.75	30.75	30.70	30.71	1.43	0.06
H(23)-Me	0.9	31.20	31.30	31.30	31.29	31.29	31.13	31.01	…	31.04	31.19	31.03	31.12	31.33	31.36	31.31	31.22	0.93	0.03
H(24)-Me	1.58	30.15	30.41	30.53	30.49	30.32	30.51	30.33	…	30.43	30.15	30.29	30.28	30.28	30.56	30.33	30.42	1.73	0.15
H(25)-Me	1.51	30.52	30.89	30.90	30.77	30.60	30.39	30.89	…	30.53	29.99	30.73	30.35	30.80	30.38	30.83	30.63	1.51	0.00
H(1'')	4.73	27.34	27.46	27.45	27.44	27.44	27.31	27.30	…	27.50	27.46	27.44	27.29	27.55	27.57	27.57	27.41	4.74	0.01
H(2'')	3.78	28.50	28.45	28.45	28.44	28.44	28.44	28.45	…	28.23	28.10	28.27	28.43	28.18	28.24	28.20	28.41	3.74	-0.04
H(3'')	3.56	28.75	28.72	28.72	28.72	28.72	28.73	28.73	…	28.66	28.65	28.64	28.72	28.64	28.67	28.65	28.72	3.43	-0.13
H(4'')	3.56	28.73	28.72	28.72	28.72	28.71	28.71	28.71	…	28.54	28.62	28.54	28.70	28.53	28.54	28.54	28.68	3.47	-0.09
H(6''eq)-Me	1.24	31.15	31.14	30.97	31.14	30.63	30.96	30.62	…	30.94	31.01	30.88	30.94	31.05	30.94	31.06	30.90	1.25	0.01
H(6''ax)-Me	1.21	30.64	31.39	30.91	30.90	30.91	30.57	30.90	…	30.61	31.58	31.42	30.56	30.64	30.93	30.65	30.98	1.17	-0.04
Energy (kJ/mol)		0.00	0.62	1.56	2.18	2.29	2.50	2.76	…	7.27	7.31	7.72	7.79	8.04	8.12	8.16	**DP4+ (^{1}H data)**		
Boltzmann factor		0.12	0.10	0.07	0.05	0.05	0.05	0.04	…	0.01	0.01	0.01	0.01	0.005	0.005	0.005	100.00%		

[a]H20a, H_{Re} and H20b, H_{Si} could not be unambiguously assigned and thus, the experimental chemical shift could be the other way round.

*Table 36: DP4+ results for **13S**: Computed σ for C-nuclei for each conformer and the averaged σ (conformers weighted based on Boltzmann factors) for simplified 11-desnoviosyl-13S-thio-(4”-desbutyryl)-β-D-noviosyl fidaxomicin **18e-C(13)**. Computed chemical shifts δ were calculated using TMS as a reference (σ_C(TMS): 190.0976; σ_H(TMS): 32.148). (PCM, solvent: acetone/B3LYP/6-31G(d)). Total amount of conformers: 46.*

**C-atom**	***Exp. δ, ppm***	***Conformers, shielding tensor σ***															***σ Boltz. avrg.***	***Comp. avrg. δ, ppm***	***Δδ, ppm***
		**197**	**327**	**425**	**74**	**37**	**55**	**46**	…	**393**	**377**	**198**	**268**	**174**	**34**	**132**			
C(1)	166.6	26.0	30.6	30.6	30.3	30.6	30.3	30.6	…	30.7	26.7	30.1	30.0	29.9	26.2	30.1	29.0	161.1	-5.5
C(2)	125.2	70.3	69.6	69.9	69.9	69.4	69.8	69.3	…	69.5	70.4	69.4	70.9	70.7	70.7	69.7	69.9	120.2	-5.0
C(3)	144.9	46.5	49.6	49.4	48.4	49.2	48.4	49.4	…	49.7	47.4	48.5	47.5	47.2	46.9	48.0	48.4	141.7	-3.2
C(4)	127.9	66.0	67.1	67.1	66.1	65.8	66.0	65.8	…	67.0	65.5	65.9	67.7	68.3	68.6	66.1	66.8	123.3	-4.6
C(5)	143.4	46.3	47.8	47.2	47.7	48.0	47.7	47.9	…	47.8	47.0	47.8	44.9	44.7	45.6	47.4	47.0	143.1	-0.3
C(6)	37.6	150.9	151.4	151.3	151.2	151.2	151.3	151.4	…	151.4	151.0	151.1	149.4	152.7	151.6	151.2	151.3	38.8	1.2
C(7)	72.8	117.1	117.6	117.5	117.4	117.7	117.5	117.6	…	117.7	117.4	117.5	116.7	115.8	117.4	117.4	117.3	72.8	0.0
C(8)	134.6	57.5	58.4	58.4	58.3	58.3	60.0	60.1	…	60.2	60.4	60.4	62.6	57.4	57.2	58.3	58.5	131.6	-3.0
C(9)	126.7	68.5	68.9	68.9	68.8	68.9	67.1	67.1	…	67.2	68.1	67.2	66.4	69.3	70.9	68.7	68.6	121.5	-5.2
C(10)	39.1	149.3	150.0	149.9	149.9	150.0	149.2	149.3	…	149.2	149.7	150.0	149.2	150.3	150.0	150.0	149.7	40.4	1.3
C(11)	134.5	60.3	58.0	58.0	57.8	57.9	59.4	59.7	…	59.4	60.2	60.4	59.1	56.8	57.3	56.9	58.6	131.5	-3.0
C(12)	135.1	61.6	59.4	59.3	59.3	59.2	56.6	56.3	…	56.9	59.4	55.3	56.8	59.9	61.1	60.0	59.5	130.6	-4.5
C(13)	60.4	121.0	124.8	124.8	124.8	124.8	124.2	124.1	…	123.8	117.1	128.1	124.1	125.1	124.2	125.3	124.1	66.0	5.6
C(14)	137.6	57.7	54.0	54.2	54.4	54.3	54.5	54.2	…	54.1	57.1	50.8	54.6	54.7	54.8	54.9	55.0	135.1	-2.5
C(15)	121.4	66.9	72.5	72.5	72.4	72.6	72.0	72.0	…	71.8	65.9	70.2	72.0	72.1	73.5	71.8	71.4	118.7	-2.7
C(16)	29.1	157.3	160.5	160.2	159.8	160.2	159.5	159.9	…	160.2	156.5	159.4	159.2	159.3	156.0	159.4	159.0	31.1	2.0
C(17)	78	105.6	112.0	112.3	112.3	112.3	112.3	112.2	…	112.7	105.5	112.6	112.6	112.9	106.2	113.4	110.4	79.7	1.7
C(18)	66.6	120.4	123.9	123.9	123.9	123.9	123.8	123.8	…	123.8	120.3	123.7	123.7	123.8	120.0	123.7	122.6	67.5	0.9
C(19)	21.3	172.6	169.0	169.1	169.1	169.1	169.1	169.1	…	168.1	172.5	169.1	169.1	169.1	170.3	168.1	169.8	20.3	-1.0
C(20)	62.6	126.8	125.4	125.4	126.3	126.3	126.4	126.3	…	125.4	126.2	126.5	126.5	126.6	126.0	126.7	126.1	64.0	1.4
C(21)	14.8	174.9	174.9	174.7	174.7	174.8	175.4	175.4	…	175.5	175.0	175.3	175.2	174.7	175.1	174.7	175.0	15.1	0.3
C(22)	30.2	158.0	158.1	158.2	158.2	158.2	158.6	158.5	…	158.5	159.0	158.5	158.3	158.0	158.3	158.4	158.3	31.8	1.6
C(23)	12	177.3	177.5	177.5	177.5	177.5	176.5	176.5	…	176.5	177.3	176.8	176.4	177.6	177.6	177.5	177.2	12.8	0.8
C(24)	12	176.4	178.0	177.9	178.0	177.9	177.6	177.7	…	177.8	175.0	176.8	177.7	178.6	178.6	178.5	177.7	12.4	0.4
C(25)	17.2	173.4	172.7	172.9	172.9	173.0	173.2	173.1	…	172.9	173.2	173.3	173.3	173.2	173.2	173.3	173.1	17.0	-0.2
C(1")	78.1	107.8	107.0	107.1	107.1	107.3	106.4	106.5	…	105.0	103.5	109.1	106.4	107.2	106.4	107.3	107.1	83.0	4.9
C(2")	74.2	117.1	116.8	117.0	116.9	117.1	117.0	117.2	…	116.6	115.7	113.6	117.0	116.6	116.5	116.6	116.9	73.2	-1.0
C(3")	72.6	118.8	118.8	118.7	118.8	118.7	118.8	118.8	…	119.8	119.9	119.3	118.8	119.8	119.8	119.8	119.0	71.1	-1.5
C(4")	74.4	115.9	115.9	115.8	115.8	115.8	115.9	115.8	…	116.6	117.0	117.2	115.9	116.6	116.6	116.6	116.0	74.1	-0.3
C(5")	77.8	112.1	112.4	112.3	112.4	112.3	112.3	112.3	…	113.0	113.0	111.7	112.3	113.0	112.8	113.0	112.4	77.7	-0.1
Energy (kJ/mol)		0.00	0.62	1.56	2.18	2.29	2.50	2.76	…	7.27	7.31	7.72	7.79	8.04	8.12	8.16	**DP4+ (^{13}C data)**		
Boltzmann factor		0.12	0.10	0.07	0.05	0.05	0.05	0.04	…	0.01	0.01	0.01	0.01	0.005	0.005	0.005	100.00%		

11-Desnoviosyl-15-thio-(4”-desbutyryl)-β-D-noviosyl fidaxomicin simplified (**18e-C(15)**)

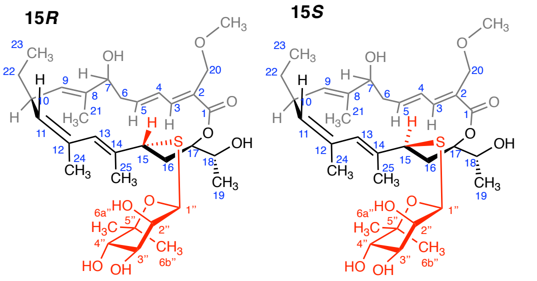

Table 37: Experimental vs. computed [1]*H chemical shifts and DP4+ results for simplified 11-desnoviosyl-15-thio-(4”-desbutyryl)-β-D-noviosyl fidaxomicin* ***18e-C(15)*** *(PCM, solvent: acetone/B3LYP/6-31G(d))*

**H-atom**	**Exp. δ, ppm**	**15*R***		**15S**	
		**Comp. δ, ppm**	**Δδ, ppm**	**Comp. δ, ppm**	**Δδ, ppm**
H(3)	7.15	7.18	0.03	7.00	-0.15
H(4)	6.32	6.69	0.37	6.30	-0.02
H(5)	6.20	6.24	0.04	6.65	0.45
H(6a, H_{Si})	2.59	2.30	-0.29	2.75	0.16
H(6b, H_{Re})	2.37	2.85	0.48	2.37	0.00
H(7)	4.30	4.23	-0.07	4.38	0.08
H(9)	5.19	5.14	-0.05	5.31	0.12
H(10)	3.11	3.34	0.23	3.25	0.14
H(11)	5.04	5.24	0.20	5.14	0.10
H(13)	5.62	6.08	0.46	5.53	-0.09
H(15)	3.56	5.03	1.47	3.60	0.04
H(16b, H_{Re})	1.79	2.32	0.54	1.60	-0.19
H(16a, H_{Si})	2.23	2.69	0.46	2.33	0.10
H(17)	5.14	4.62	-0.51	5.09	-0.04
H(18)	3.82	4.23	0.41	3.83	0.01
H(19)-Me	1.16	1.09	-0.07	1.05	-0.11
H(20a, H_{Re})	4.45[a]	4.24	-0.21	4.02	-0.43
H(20b, H_{Si})	4.62[a]	4.24	-0.38	4.51	-0.11
H(21)-Me	1.58	1.68	0.10	1.57	-0.01
H(22a, H_{Re})	1.27	1.51	0.24	1.37	0.10
H(22b, H_{Si})	1.27	1.50	0.23	1.38	0.11
H(23)-Me	0.78	0.92	0.14	0.85	0.07
H(24)-Me	1.82	1.74	-0.08	1.87	0.05
H(25)-Me	1.90	1.62	-0.28	1.91	0.01
H(1")	4.65	4.82	0.17	4.65	0.00
H(2")	3.74	3.91	0.17	3.62	-0.12
H(3")	3.48	3.40	-0.08	3.35	-0.13
H(4")	3.52	3.37	-0.15	3.43	-0.09
H(6"eq)-Me	1.27	1.24	-0.03	1.27	0.00
H(6"ax)-Me	1.14	1.13	-0.01	1.15	0.01
**DP4+ (^{1}H data)**		**0.00%**		**100.00%**	
**DP4+ (all data)**		**0.00%**		**100.00%**	

[a]H20a, H_{Re} and H20b, H_{Si} could not be unambiguously assigned and thus, the experimental chemical shift could be the other way round.

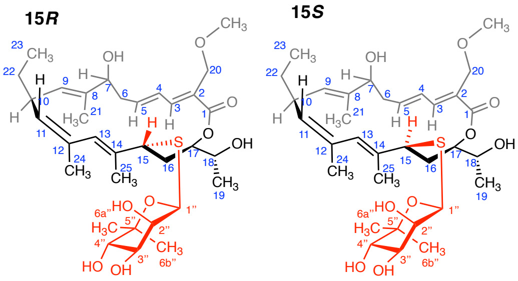

*Table 38: Experimental vs. computed ^{13}C chemical shifts and DP4+ results for simplified 11-desnoviosyl-15-thio-(4”-desbutyryl)-β-D-noviosyl fidaxomicin **18e-C(15)** (PCM, solvent: acetone/B3LYP/6-31G(d))*

C-atom	Exp. δ, ppm	15*R*		15S	
		Comp. δ, ppm	Δδ, ppm	Comp. δ, ppm	Δδ, ppm
C(1)	167.5	159.8	-7.7	161.1	-6.4
C(2)	125.2	120.3	-4.9	120.5	-4.7
C(3)	144.9	140.9	-4.0	140.7	-4.2
C(4)	127.2	123.3	-3.9	120.3	-6.9
C(5)	143.2	142.1	-1.1	143.7	0.5
C(6)	38.3	38.6	0.3	38.0	-0.3
C(7)	73.8	73.2	-0.6	74.8	1.0
C(8)	133.8	131.2	-2.6	131.5	-2.3
C(9)	127.1	123.2	-3.9	122.0	-5.1
C(10)	39.5	41.2	1.7	40.5	1.0
C(11)	138.1	130.4	-7.7	134.7	-3.4
C(12)	131.9	126.3	-5.6	129.2	-2.7
C(13)	134.0	126.6	-7.4	130.0	-4.0
C(14)	132.7	133.3	0.6	130.3	-2.4
C(15)	53.5	58.0	4.5	60.6	7.1
C(16)	31.1	36.1	5.0	30.8	-0.3
C(17)	78.1	76.3	-1.8	76.5	-1.6
C(18)	69.6	66.5	-3.1	68.4	-1.2
C(19)	19.0	20.7	1.7	17.5	-1.5
C(20)	61.9	64.2	2.3	65.2	3.3
C(21)	15.5	15.1	-0.4	15.6	0.1
C(22)	29.8	31.6	1.8	31.7	1.9
C(23)	11.9	13.0	1.1	12.9	1.0
C(24)	17.0	18.7	1.7	17.1	0.1
C(25)	13.5	16.2	2.7	15.5	2.0
C(1")	77.7	82.8	5.1	82.7	5.0
C(2")	73.9	76.2	2.3	73.4	-0.5
C(3")	72.4	71.3	-1.1	71.2	-1.2
C(4")	74.2	73.8	-0.4	74.1	-0.1
C(5")	77.8	78.3	0.5	77.7	-0.1
C(6"eq)	28.9	28.6	-0.3	28.6	-0.3
C(6"ax)	18.0	16.1	-1.9	17.2	-0.8
**DP4+ (^{13}C data)**		**0.08%**		**99.92%**	
**DP4+ (all data)**		**0.00%**		**100.00%**	

*Table 39: DP4+ results for **15R**: Computed σ for H-nuclei for each conformer and the averaged σ (conformers weighted based on Boltzmann factors) for simplified 11-desnoviosyl-15R-thio-(4"-desbutyryl)-β-D-noviosyl fidaxomicin **18e-C(15)**. Computed chemical shifts δ were calculated using TMS as a reference (σ_C(TMS): 190.0976; σ_H(TMS): 32.148). (PCM, solvent: acetone/B3LYP/6-31G(d)). Total amount of conformers: 25.*

H-atom	***Exp. δ, ppm***	***Conformers, shielding tensor σ***															***σ Boltz. avrg.***	***Comp. avrg. δ, ppm***	***Δδ, ppm***
		**169**	**43**	**183**	**155**	**54**	**53**	**235**	…	**65**	**89**	**61**	**268**	**133**	**315**	**197**			
H(3)	7.15	24.97	24.98	24.95	24.98	24.98	25.01	24.93	…	24.94	24.99	25.02	24.85	24.96	24.90	24.96	24.97	7.18	0.03
H(4)	6.32	25.44	25.44	25.56	25.45	25.45	25.40	25.57	…	25.78	25.39	25.31	25.40	25.41	25.40	25.39	25.46	6.69	0.37
H(5)	6.20	25.97	25.95	25.96	25.98	25.96	25.90	25.97	…	25.44	25.85	25.89	25.99	25.69	25.93	25.68	25.91	6.24	0.04
H(6a, H_{Si})	2.59	29.88	29.87	29.88	29.89	29.88	29.87	29.89	…	29.37	29.86	29.86	29.54	29.69	29.69	29.68	29.84	2.30	-0.29
H(6b, H_{Re})	2.37	29.26	29.27	29.36	29.26	29.27	29.22	29.38	…	29.81	29.22	29.22	29.27	29.44	29.55	29.43	29.30	2.85	0.48
H(7)	4.30	27.93	27.92	27.93	27.96	27.95	27.91	27.96	…	27.77	27.95	27.92	27.92	27.77	27.81	27.73	27.92	4.23	-0.07
H(9)	5.19	27.06	27.02	27.18	27.03	26.99	27.00	27.15	…	26.73	26.88	26.96	26.79	26.76	26.82	26.84	27.01	5.14	-0.05
H(10)	3.11	28.82	28.81	28.87	28.84	28.83	28.73	28.89	…	28.92	28.73	28.75	28.85	28.70	28.81	28.72	28.81	3.34	0.23
H(11)	5.04	26.99	26.93	27.04	27.06	26.98	26.57	27.12	…	27.12	26.68	26.71	27.10	26.66	27.06	26.67	26.91	5.24	0.20
H(13)	5.62	26.04	26.03	26.07	26.03	26.02	26.16	26.07	…	26.30	26.02	26.04	26.02	26.01	26.11	26.01	26.07	6.08	0.46
H(15)	3.56	26.70	26.68	26.71	26.69	26.67	27.87	26.72	…	28.18	27.88	27.93	26.69	27.88	28.08	27.89	27.12	5.03	1.47
H(16b, H_{Re})	1.79	29.93	29.88	29.64	29.93	29.87	30.08	29.62	…	29.25	29.87	29.93	29.87	29.93	29.73	29.94	29.83	2.32	0.54
H(16a, H_{Si})	2.23	29.24	29.24	29.31	29.24	29.24	30.06	29.29	…	29.74	29.87	29.88	29.23	29.86	29.78	29.87	29.46	2.69	0.46
H(17)	5.14	27.44	27.48	27.70	27.43	27.48	27.59	27.69	…	27.77	27.51	27.51	27.48	27.49	27.55	27.48	27.53	4.62	-0.51
H(18)	3.82	27.93	27.93	27.91	27.92	27.92	27.95	27.90	…	27.92	27.95	27.95	27.92	27.92	27.98	27.93	27.92	4.23	0.41
H(19)-Me	1.16	31.42	31.45	31.41	30.91	30.97	30.86	31.41	…	30.87	30.89	30.87	30.89	31.48	31.49	30.86	31.06	1.09	-0.07
H(20a, H_{Re})	4.45[a]	27.95	27.76	28.04	27.95	27.76	27.98	28.03	…	28.26	27.83	27.95	27.82	27.98	27.95	27.98	27.91	4.24	-0.21
H(20b, H_{Si})	4.62[a]	27.77	27.98	27.98	27.77	27.99	27.78	27.98	…	27.78	28.02	27.75	27.97	27.75	27.72	27.74	27.90	4.24	-0.38
H(21)-Me	1.58	30.13	30.13	30.57	30.53	30.11	30.77	30.16	…	30.20	29.94	29.94	30.17	29.92	30.74	29.92	30.47	1.68	0.10
H(22a, H_{Re})	1.27	30.66	30.64	30.66	30.72	30.70	30.51	30.71	…	30.74	30.58	30.51	30.74	30.61	30.74	30.50	30.63	1.51	0.24
H(22b, H_{Si})	1.27	30.66	30.64	30.68	30.59	30.57	30.65	30.63	…	30.69	30.65	30.64	30.66	30.71	30.68	30.67	30.64	1.50	0.23
H(23)-Me	0.78	31.35	31.19	31.21	31.20	31.08	31.32	31.34	…	31.12	31.15	31.18	31.19	31.32	31.06	31.21	31.23	0.92	0.14
H(24)-Me	1.82	30.59	30.58	30.63	30.54	30.08	30.59	30.13	…	30.74	30.02	30.44	30.09	30.58	30.58	30.41	30.41	1.74	-0.08
H(25)-Me	1.90	30.67	30.40	30.64	30.64	30.51	30.58	30.45	…	30.40	30.58	30.76	30.33	30.67	30.56	30.70	30.52	1.62	-0.28
H(1'')	4.65	27.45	27.45	27.45	27.45	27.44	27.19	27.45	…	26.70	27.26	27.27	27.46	27.28	26.92	27.29	27.33	4.82	0.17
H(2'')	3.74	28.14	28.14	28.13	28.14	28.14	28.34	28.13	…	28.46	28.25	28.25	28.13	28.24	28.46	28.24	28.24	3.91	0.17
H(3'')	3.48	28.78	28.77	28.77	28.78	28.77	28.73	28.77	…	28.71	28.63	28.63	28.77	28.62	28.70	28.62	28.75	3.40	-0.08
H(4'')	3.52	28.82	28.82	28.81	28.82	28.82	28.82	28.81	…	28.71	28.53	28.54	28.82	28.54	28.72	28.54	28.78	3.37	-0.15
H(6''eq)-Me	1.27	31.16	31.16	31.17	31.16	30.99	30.96	31.17	…	31.07	30.83	31.02	31.16	30.83	30.58	31.02	30.91	1.24	-0.03
H(6''ax)-Me	1.14	30.68	30.97	30.67	31.52	30.97	30.75	30.67	…	31.22	31.28	30.95	31.53	31.28	31.22	30.96	31.02	1.13	-0.01
Energy (kJ/mol)		0.00	0.08	0.72	1.37	1.40	1.45	1.93	…	7.31	7.51	7.65	8.15	8.18	8.23	7.31	**DP4+ (^{1}H data)**		
Boltzmann factor		0.14	0.13	0.10	0.08	0.08	0.08	0.06	…	0.01	0.01	0.01	0.01	0.01	0.005	0.01	0.00%		

[a]H20a, H_{Re} and H20b, H_{Si} could not be unambiguously assigned and thus, the experimental chemical shift could be the other way round.

*Table 40: DP4+ results for **15R**: Computed σ for C-nuclei for each conformer and the averaged σ (conformers weighted based on Boltzmann factors) for simplified 11-desnoviosyl-15R-thio-(4”-desbutyryl)-β-D-noviosyl fidaxomicin **18e-C(15)**. Computed chemical shifts δ were calculated using TMS as a reference (σ_C(TMS): 190.0976; σ_H(TMS): 32.148). (PCM, solvent: acetone/B3LYP/6-31G(d)). Total amount of conformers: 25.*

**C-atom**	**Exp. δ, ppm**	**Conformers, shielding tensor σ**															**σ Boltz. avrg.**	**Comp. avrg. δ, ppm**	**Δδ, ppm**
		**169**	**43**	**183**	**155**	**54**	**53**	**235**	…	**65**	**89**	**61**	**268**	**133**	**315**	**197**			
C(1)	167.5	30.2	30.3	30.6	30.3	30.3	30.6	30.8	…	29.3	30.2	30.8	30.2	30.5	29.9	30.5	30.3	159.8	-7.7
C(2)	125.2	69.9	69.7	69.5	70.0	69.8	69.4	69.6	…	71.1	69.6	69.7	71.3	70.7	71.0	70.8	69.8	120.3	-4.9
C(3)	144.9	49.3	49.2	49.7	49.3	49.1	49.5	49.7	…	48.7	48.6	49.8	47.2	48.2	48.2	48.2	49.2	140.9	-4.0
C(4)	127.2	67.3	67.3	66.0	67.3	67.4	65.8	65.7	…	68.7	66.2	65.6	70.0	67.6	67.0	67.7	66.8	123.3	-3.9
C(5)	143.2	47.0	47.4	51.7	46.9	47.3	48.0	52.2	…	46.7	47.6	48.3	43.4	44.9	45.0	45.0	48.0	142.1	-1.1
C(6)	38.3	151.4	151.6	152.2	151.5	151.6	151.1	152.1	…	151.7	151.0	150.9	150.3	152.3	153.3	152.2	151.5	38.6	0.3
C(7)	73.8	117.5	117.1	116.0	117.5	117.2	117.3	115.7	…	115.3	117.1	117.1	116.8	115.4	116.1	115.4	116.9	73.2	-0.6
C(8)	133.8	57.5	57.6	58.1	60.4	60.4	59.4	60.9	…	58.4	62.0	59.6	60.6	61.1	59.6	58.7	58.9	131.2	-2.6
C(9)	127.1	66.6	66.6	67.0	66.5	66.4	68.2	67.0	…	65.7	67.2	67.9	60.7	67.2	65.7	68.3	66.9	123.2	-3.9
C(10)	39.5	148.7	148.7	148.9	149.0	149.0	148.9	149.0	…	149.2	148.8	149.0	148.1	149.1	149.1	149.3	148.9	41.2	1.7
C(11)	138.1	59.8	59.6	59.5	61.2	61.1	58.5	60.9	…	61.4	58.3	58.7	61.1	58.3	61.3	58.5	59.7	130.4	-7.7
C(12)	131.9	64.9	64.9	65.0	63.0	62.9	63.0	63.2	…	62.8	61.2	63.2	62.6	60.8	62.7	62.8	63.8	126.3	-5.6
C(13)	134.0	63.3	63.3	62.7	63.7	63.7	64.0	62.9	…	65.2	63.5	63.4	61.9	63.5	65.3	63.3	63.5	126.6	-7.4
C(14)	132.7	57.0	57.1	57.4	56.9	57.0	55.5	57.2	…	55.6	56.6	56.5	57.5	56.5	56.0	56.6	56.8	133.3	0.6
C(15)	53.5	131.9	131.8	132.2	131.7	131.5	129.7	131.8	…	132.9	133.8	133.7	132.5	133.7	131.6	133.7	132.1	58.0	4.5
C(16)	31.1	152.7	152.9	154.2	152.6	152.9	157.1	154.6	…	154.7	155.5	155.1	153.6	155.4	151.0	155.3	154.0	36.1	5.0
C(17)	78.1	114.5	114.3	112.3	114.6	114.3	113.5	112.3	…	112.5	113.8	114.0	114.7	114.1	114.1	114.2	113.8	76.3	-1.8
C(18)	69.6	123.0	123.1	123.2	123.0	123.1	124.6	123.3	…	124.7	124.5	124.5	123.2	124.5	124.0	124.5	123.6	66.5	-3.1
C(19)	19.0	169.7	169.7	169.3	169.7	169.7	168.9	169.4	…	169.4	169.0	169.0	169.6	169.0	169.1	169.0	169.4	20.7	1.7
C(20)	61.9	126.0	126.3	124.5	126.0	126.3	126.3	124.4	…	126.5	126.5	125.7	125.7	126.3	126.4	126.3	125.9	64.2	2.3
C(21)	15.5	175.1	175.1	175.2	175.2	175.2	174.6	175.2	…	174.7	174.8	174.4	173.4	175.0	174.9	174.6	175.0	15.1	-0.4
C(22)	29.8	158.2	158.2	158.4	158.5	158.5	159.1	158.9	…	157.4	159.1	159.5	159.8	158.8	158.4	159.4	158.5	31.6	1.8
C(23)	11.9	176.8	176.8	176.9	177.4	177.4	177.2	177.4	…	177.6	176.9	177.2	177.2	176.9	177.5	177.3	177.1	13.0	1.1
C(24)	17.0	171.6	171.6	172.1	170.7	170.6	171.1	171.4	…	170.9	171.1	171.1	171.8	171.2	171.1	171.2	171.4	18.7	1.7
C(25)	13.5	173.7	173.6	173.7	173.5	173.3	174.6	173.2	…	172.7	174.9	175.1	173.9	174.8	172.9	174.9	173.9	16.2	2.7
C(1")	77.7	107.5	107.5	107.4	107.5	107.4	104.7	107.2	…	106.6	107.5	107.2	107.3	107.3	105.9	107.2	107.3	82.8	5.1
C(2")	73.9	112.2	112.3	112.4	112.3	112.3	115.8	112.4	…	116.5	117.1	117.1	112.3	117.0	116.8	117.0	113.9	76.2	2.3
C(3")	72.4	118.8	118.8	118.7	118.8	118.8	118.9	118.8	…	118.8	119.6	119.7	118.9	119.6	118.9	119.6	118.8	71.3	-1.1
C(4")	74.2	116.5	116.4	116.4	116.4	116.4	116.1	116.4	…	115.9	116.5	116.5	116.4	116.4	116.0	116.4	116.3	73.8	-0.4
C(5")	77.8	111.7	111.7	111.7	111.7	111.7	112.1	111.7	…	111.8	112.5	112.6	111.8	112.6	112.0	112.6	111.8	78.3	0.5
C(6"eq)	28.9	161.4	161.4	161.4	161.4	161.4	161.5	161.4	…	161.1	161.7	161.7	161.4	161.7	161.6	161.7	161.5	28.6	-0.3
C(6"ax)	18.0	174.3	174.2	174.2	174.3	174.2	174.3	174.2	…	173.6	173.5	173.5	174.3	173.4	172.7	173.4	174.0	16.1	-1.9
Energy (kJ/mol)		0.00	0.08	0.72	1.37	1.40	1.45	1.93	…	7.31	7.51	7.65	8.15	8.18	8.23	7.31	**DP4+ (^{13}C data)**		
Boltzmann factor		0.14	0.13	0.10	0.08	0.08	0.08	0.06	…	0.01	0.01	0.01	0.01	0.01	0.005	0.01	0.08%		

*Table 41: DP4+ results for **15S**: Computed σ for H-nuclei for each conformer and the averaged σ (conformers weighted based on Boltzmann factors) for simplified 11-desnoviosyl-15S-thio-(4"-desbutyryl)-β-D-noviosyl fidaxomicin **18e-C(15)**. Computed chemical shifts δ were calculated using TMS as a reference (σ_C(TMS): 190.0976; σ_H(TMS): 32.148). (PCM, solvent: acetone/B3LYP/6-31G(d)).*

H-atom	***Exp. δ, ppm***	***Conformers, shielding tensor σ***										***σ Boltz. avrg.***	***Comp. avrg. δ, ppm***	***Δδ, ppm***
		**264**	**48**	**160**	**140**	**253**	**137**	**150**	**19**	**146**	**21**			
H(3)	7.15	25.23	25.08	25.22	24.86	25.19	25.03	25.03	24.93	25.03	24.93	25.15	7.00	-0.15
H(4)	6.32	25.89	25.85	25.89	25.66	25.82	25.70	25.71	25.70	25.71	25.72	25.85	6.30	-0.02
H(5)	6.20	25.54	25.46	25.53	25.35	25.44	25.42	25.42	25.45	25.42	25.44	25.50	6.65	0.45
H(6a, H_{Si})	2.59	29.38	29.44	29.38	29.38	29.42	29.40	29.40	29.41	29.40	29.41	29.40	2.75	0.16
H(6b, H_{Re})	2.37	29.81	29.74	29.81	29.68	29.72	29.72	29.73	29.74	29.73	29.75	29.78	2.37	0.00
H(7)	4.30	27.76	27.80	27.76	27.77	27.77	27.76	27.79	27.78	27.79	27.80	27.77	4.38	0.08
H(9)	5.19	26.88	26.72	26.88	26.83	26.82	26.90	26.82	26.89	26.82	26.82	26.84	5.31	0.12
H(10)	3.11	28.89	28.92	28.89	28.92	28.93	28.93	28.91	28.93	28.91	28.91	28.90	3.25	0.14
H(11)	5.04	27.05	26.95	27.04	26.89	26.87	26.92	26.98	26.92	26.97	26.97	27.01	5.14	0.10
H(13)	5.62	26.63	26.56	26.63	26.67	26.65	26.70	26.64	26.70	26.63	26.64	26.62	5.53	-0.09
H(15)	3.56	28.63	28.37	28.56	28.55	28.35	28.56	28.56	28.56	28.55	28.56	28.55	3.60	0.04
H(16b, H_{Re})	1.79	30.61	30.73	30.62	29.98	30.73	30.03	30.02	30.03	30.02	30.02	30.55	1.60	-0.19
H(16a, H_{Si})	2.23	29.70	30.00	29.70	30.06	30.01	30.02	30.01	30.03	30.01	30.02	29.82	2.33	0.10
H(17)	5.14	27.13	26.83	27.12	27.14	26.81	27.14	27.13	27.14	27.13	27.14	27.06	5.09	-0.04
H(18)	3.82	28.22	28.45	28.22	28.48	28.45	28.48	28.48	28.49	28.48	28.49	28.32	3.83	0.01
H(19)-Me	1.16	31.24	30.98	31.21	30.86	31.09	30.80	30.80	31.01	31.01	30.78	31.10	1.05	-0.11
H(20a, H_{Re})	4.45[a]	28.10	28.20	28.10	28.24	28.10	28.15	28.16	28.23	28.15	28.24	28.13	4.02	-0.43
H(20b, H_{Si})	4.62[a]	27.57	27.54	27.59	28.19	27.97	28.11	28.10	27.60	28.10	27.60	27.64	4.51	-0.11
H(21)-Me	1.58	30.11	30.81	30.11	30.67	30.79	30.12	30.87	30.70	30.11	30.87	30.58	1.57	-0.01
H(22a, H_{Re})	1.27	30.78	30.76	30.79	30.72	30.76	30.74	30.76	30.74	30.76	30.76	30.77	1.37	0.10
H(22b, H_{Si})	1.27	30.77	30.75	30.78	30.79	30.79	30.82	30.77	30.82	30.76	30.76	30.77	1.38	0.11
H(23)-Me	0.78	31.40	31.39	31.24	31.25	31.45	31.26	31.25	31.45	31.25	31.20	31.29	0.85	0.07
H(24)-Me	1.82	30.29	30.45	30.33	30.25	30.35	30.20	30.22	30.34	30.49	30.49	30.28	1.87	0.05
H(25)-Me	1.90	29.96	30.55	30.16	30.09	30.60	30.06	30.20	30.07	30.07	30.23	30.24	1.91	0.01
H(1")	4.65	27.47	27.46	27.63	27.52	27.69	27.54	27.50	27.55	27.51	27.51	27.50	4.65	0.00
H(2")	3.74	28.55	28.56	28.31	28.56	28.33	28.57	28.56	28.57	28.57	28.57	28.53	3.62	-0.12
H(3")	3.48	28.81	28.79	28.71	28.81	28.73	28.82	28.81	28.82	28.81	28.81	28.79	3.35	-0.13
H(4")	3.52	28.74	28.73	28.56	28.74	28.56	28.75	28.74	28.75	28.74	28.74	28.71	3.43	-0.09
H(6"eq)-Me	1.27	31.12	30.59	30.60	31.11	30.89	31.12	30.91	31.12	31.11	31.11	30.88	1.27	0.00
H(6"ax)-Me	1.14	31.37	30.69	30.72	30.94	30.94	31.39	30.91	31.39	30.68	30.91	31.00	1.15	0.01
Energy (kJ/mol)		0.00	2.30	4.52	6.64	7.24	7.49	7.68	7.71	7.72	7.94	**DP4+ (^{1}H data)**		
Boltzmann factor		0.53	0.21	0.08	0.04	0.03	0.03	0.02	0.02	0.02	0.02	100.00%		

[a]H20a, H_{Re} and H20b, H_{Si} could not be unambiguously assigned and thus, the experimental chemical shift could be the other way round.

*Table 42: DP4+ results for **15S**: Computed σ for C-nuclei for each conformer and the averaged σ (conformers weighted based on the Boltzmann factors) for simplified 11-desnoviosyl-15S-thio-(4”-desbutyryl)-β-D-noviosyl fidaxomicin **18e-C(15)**. Computed chemical shifts δ were calculated using TMS as a reference (σ_C(TMS): 190.0976; σ_H(TMS): 32.148). (PCM, solvent: acetone/B3LYP/6-31G(d)).*

C-atom	Exp. δ, ppm	Conformers, shielding tensor σ										σ Boltz. avrg.	Comp. avrg. δ, ppm	Δδ, ppm
		264	48	160	140	253	137	150	19	146	21			
C(1)	167.5	29.8	27.2	29.8	28.5	27.3	28.4	28.4	28.4	28.4	28.4	29.0	161.1	-6.4
C(2)	125.2	68.6	71.2	68.7	71.6	70.4	70.3	70.4	72.0	70.3	72.1	69.6	120.5	-4.7
C(3)	144.9	50.4	47.9	50.4	44.5	49.3	48.2	48.0	47.3	48.1	47.2	49.3	140.7	-4.2
C(4)	127.2	69.4	71.0	69.3	69.7	70.8	69.6	69.5	69.9	69.5	69.7	69.8	120.3	-6.9
C(5)	143.2	47.5	44.7	47.5	43.7	44.7	45.3	45.0	45.7	45.1	45.5	46.4	143.7	0.5
C(6)	38.3	152.0	152.4	152.1	151.9	152.4	151.8	151.8	151.8	151.8	151.8	152.1	38.0	-0.3
C(7)	73.8	114.9	116.1	114.8	115.9	116.0	115.6	115.7	115.6	115.8	115.8	115.3	74.8	1.0
C(8)	133.8	59.1	58.6	59.2	56.0	55.7	56.0	58.9	56.1	58.9	58.9	58.6	131.5	-2.3
C(9)	127.1	67.9	68.1	67.8	69.1	69.1	69.3	68.2	69.4	68.3	68.3	68.1	122.0	-5.1
C(10)	39.5	149.7	149.6	149.6	149.8	149.7	149.8	149.6	149.8	149.6	149.6	149.6	40.5	1.0
C(11)	138.1	55.6	55.3	55.5	55.1	54.4	54.4	54.8	54.5	54.8	54.7	55.4	134.7	-3.4
C(12)	131.9	60.6	60.7	60.6	62.3	63.5	63.4	60.8	63.3	60.8	60.8	60.9	129.2	-2.7
C(13)	134.0	60.3	60.2	60.1	59.7	59.5	59.6	59.8	59.6	59.8	59.8	60.1	130.0	-4.0
C(14)	132.7	59.4	60.1	59.7	60.5	60.4	60.4	60.5	60.4	60.5	60.5	59.8	130.3	-2.4
C(15)	53.5	128.8	131.3	129.0	129.1	130.6	129.6	129.7	129.7	129.7	129.8	129.5	60.6	7.1
C(16)	31.1	161.0	157.3	161.0	155.4	157.6	155.4	155.4	155.3	155.4	155.3	159.3	30.8	-0.3
C(17)	78.1	113.5	113.8	113.4	113.7	114.0	114.1	114.2	114.1	114.2	114.1	113.6	76.5	-1.6
C(18)	69.6	122.0	122.1	122.0	120.4	122.3	119.7	119.6	119.7	119.6	119.6	121.7	68.4	-1.2
C(19)	19.0	173.2	172.6	173.2	170.2	172.3	170.4	170.5	170.5	170.5	170.5	172.6	17.5	-1.5
C(20)	61.9	124.6	125.1	124.7	125.0	123.7	125.3	125.3	126.8	125.3	126.8	124.9	65.2	3.3
C(21)	15.5	174.6	174.6	174.6	174.1	174.4	174.2	174.5	174.2	174.5	174.5	174.5	15.6	0.1
C(22)	29.8	158.3	158.5	158.3	159.1	159.2	159.3	158.5	159.3	158.6	158.6	158.4	31.7	1.9
C(23)	11.9	177.2	177.3	177.2	177.4	177.4	177.4	177.1	177.4	177.1	177.1	177.2	12.9	1.0
C(24)	17.0	173.0	172.8	173.1	173.5	173.2	173.1	172.9	173.1	172.9	172.9	173.0	17.1	0.1
C(25)	13.5	174.5	174.6	174.7	174.7	175.0	174.8	174.7	174.8	174.7	174.8	174.6	15.5	2.0
C(1")	77.7	107.3	107.9	106.7	107.7	106.6	107.5	107.5	107.5	107.5	107.5	107.4	82.7	5.0
C(2")	73.9	116.7	116.9	116.6	116.7	116.4	116.6	116.6	116.5	116.5	116.6	116.7	73.4	-0.5
C(3")	72.4	118.7	118.9	119.8	118.7	119.7	118.7	118.8	118.8	118.8	118.8	118.9	71.2	-1.2
C(4")	74.2	115.9	115.9	116.7	115.8	116.7	115.9	115.9	115.9	115.9	116.0	116.0	74.1	-0.1
C(5")	77.8	112.3	112.3	112.9	112.3	112.9	112.4	112.4	112.4	112.3	112.4	112.4	77.7	-0.1
C(6"eq)	28.9	161.5	161.5	161.5	161.5	161.4	161.5	161.5	161.5	161.5	161.5	161.5	28.6	-0.3
C(6"ax)	18.0	172.8	172.9	173.1	172.7	172.9	172.7	172.8	172.7	172.8	172.8	172.9	17.2	-0.8
**Energy (kJ/mol)**		0.00	2.30	4.52	6.64	7.24	7.49	7.68	7.71	7.72	7.94	**DP4+ (^{13}C data)**		
**Boltzmann factor**		0.53	0.21	0.08	0.04	0.03	0.03	0.02	0.02	0.02	0.02	99.92%		

References

(1) Grimblat, N.; Zanardi, M. M.; Sarotti, A. M. Beyond DP4: An Improved Probability for the Stereochemical Assignment of Isomeric Compounds Using Quantum Chemical Calculations of NMR Shifts. *J. Org. Chem.* **2015**, *80* (24), 12526–12534. https://doi.org/10.1021/acs.joc.5b02396.

(2) Neese, F. The ORCA Program System. *WIREs Comput. Mol. Sci.* **2012**, *2* (1), 73–78. https://doi.org/10.1002/wcms.81.

(3) Neese, F. Software Update: The ORCA Program System, Version 4.0. *WIREs Comput. Mol. Sci.* **2018**, *8* (1), e1327. https://doi.org/10.1002/wcms.1327.

(4) Neese, F. Software Update: The ORCA Program System—Version 5.0. *WIREs Comput. Mol. Sci.* **2022**, *12* (5), e1606. https://doi.org/10.1002/wcms.1606.

(5) Neese, F.; Wennmohs, F.; Becker, U.; Riplinger, C. The ORCA Quantum Chemistry Program Package. *J. Chem. Phys.* **2020**, *152* (22), 224108. https://doi.org/10.1063/5.0004608.

(6) Smith, S. G.; Goodman, J. M. Assigning Stereochemistry to Single Diastereoisomers by GIAO NMR Calculation: The DP4 Probability. *J. Am. Chem. Soc.* **2010**, *132* (37), 12946–12959. https://doi.org/10.1021/ja105035r.

(7) Avogadro: An Open-Source Molecular Builder and Visualization Tool. Version 1.2.0. http://avogadro.cc/.

(8) Hanwell, M. D.; Curtis, D. E.; Lonie, D. C.; Vandermeersch, T.; Zurek, E.; Hutchison, G. R. Avogadro: An Advanced Semantic Chemical Editor, Visualization, and Analysis Platform. *J. Cheminformatics* **2012**, *4* (1), 17. https://doi.org/10.1186/1758-2946-4-17.

(9) Cao, X.; Boyaci, H.; Chen, J.; Bao, Y.; Landick, R.; Campbell, E. A. Basis of Narrow-Spectrum Activity of Fidaxomicin on Clostridioides Difficile. *Nature* **2022**, *604* (7906), 541–545. https://doi.org/10.1038/s41586-022-04545-z.

(10) Berman, H. M.; Westbrook, J.; Feng, Z.; Gilliland, G.; Bhat, T. N.; Weissig, H.; Shindyalov, I. N.; Bourne, P. E. The Protein Data Bank. *Nucleic Acids Res.* **2000**, *28* (1), 235–242. https://doi.org/10.1093/nar/28.1.235.

(11) Bannwarth, C.; Ehlert, S.; Grimme, S. GFN2-xTB—An Accurate and Broadly Parametrized Self-Consistent Tight-Binding Quantum Chemical Method with Multipole Electrostatics and Density-Dependent Dispersion Contributions. *J. Chem. Theory Comput.* **2019**, *15* (3), 1652–1671. https://doi.org/10.1021/acs.jctc.8b01176.

(12) Bannwarth, C.; Caldeweyher, E.; Ehlert, S.; Hansen, A.; Pracht, P.; Seibert, J.; Spicher, S.; Grimme, S. Extended Tight-binding Quantum Chemistry Methods. *WIREs Comput. Mol. Sci.* **2021**, *11* (2), e1493. https://doi.org/10.1002/wcms.1493.

(13) Ehlert, S.; Stahn, M.; Spicher, S.; Grimme, S. Robust and Efficient Implicit Solvation Model for Fast Semiempirical Methods. *J. Chem. Theory Comput.* **2021**, *17* (7), 4250–4261. https://doi.org/10.1021/acs.jctc.1c00471.

(14) Pracht, P.; Bohle, F.; Grimme, S. Automated Exploration of the Low-Energy Chemical Space with Fast Quantum Chemical Methods. *Phys. Chem. Chem. Phys.* **2020**, *22* (14), 7169–7192. https://doi.org/10.1039/C9CP06869D.

(15) Grimme, S. Exploration of Chemical Compound, Conformer, and Reaction Space with Meta-Dynamics Simulations Based on Tight-Binding Quantum Chemical Calculations. *J. Chem. Theory Comput.* **2019**, *15* (5), 2847–2862. https://doi.org/10.1021/acs.jctc.9b00143.

(16) Barone, V.; Cossi, M. Quantum Calculation of Molecular Energies and Energy Gradients in Solution by a Conductor Solvent Model. *J. Phys. Chem. A* **1998**, *102* (11), 1995–2001. https://doi.org/10.1021/jp9716997.

(17) Tomasi, J.; Mennucci, B.; Cammi, R. Quantum Mechanical Continuum Solvation Models. *Chem. Rev.* **2005**, *105* (8), 2999–3093. https://doi.org/10.1021/cr9904009.

(18) Weigend, F. Accurate Coulomb-Fitting Basis Sets for H to Rn. *Phys. Chem. Chem. Phys.* **2006**, *8* (9), 1057–1065. https://doi.org/10.1039/b515623h.
(19) Stoychev, G. L.; Auer, A. A.; Neese, F. Automatic Generation of Auxiliary Basis Sets. *J. Chem. Theory Comput.* **2017**, *13* (2), 554–562. https://doi.org/10.1021/acs.jctc.6b01041.
