# Supplementary material for: Formal Single Atom Editing of the Glycosylated Natural Product Fidaxomicin Improves Acid Stability and Retains Antibiotic Activity
Source: JACS Au. 2024 May 21;4(6):2267–80. doi: 10.1021/jacsau.4c00206 (PMC11200244; doi:10.1021/jacsau.4c00206)

checkCIF/PLATON report

Structure factors have been supplied for datablock(s) 15b

THIS REPORT IS FOR GUIDANCE ONLY. IF USED AS PART OF A REVIEW PROCEDURE FOR PUBLICATION, IT SHOULD NOT REPLACE THE EXPERTISE OF AN EXPERIENCED CRYSTALLOGRAPHIC REFEREE.

No syntax errors found. CIF dictionary Interpreting this report

Datablock: 15b

```
Bond precision:   C-C = 0.0025 A          Wavelength=1.54184

Cell:              a=5.6653(1)     b=8.5363(1)         c=17.7724(1)
                   alpha=90        beta=92.874(1)      gamma=90
Temperature:       160 K

                 Calculated                  Reported
Volume           858.405(19)                 858.405(19)
Space group      P 21                        P 1 21 1
Hall group       P 2yb                       P 2yb
Moiety formula   C16 H24 O7                  C16 H24 O7
Sum formula      C16 H24 O7                  C16 H24 O7
Mr               328.35                      328.35
Dx,g cm-3        1.270                       1.270
Z                2                           2
Mu (mm-1)        0.835                       0.835
F000             352.0                       352.0
F000'            353.23
h,k,lmax         7,10,22                     7,10,22
Nref             3495[ 1870]                 3489
Tmin,Tmax        0.878,0.943                 0.875,0.947
Tmin'            0.839

Correction method= # Reported T Limits: Tmin=0.875 Tmax=0.947
AbsCorr = ANALYTICAL

Data completeness= 1.87/1.00       Theta(max)= 74.441

R(reflections)= 0.0267( 3446)                          wR2(reflections)=
                                                       0.0715( 3489)
S = 1.043                 Npar= 214
```

The following ALERTS were generated. Each ALERT has the format
test-name_ALERT_alert-type_alert-level.
Click on the hyperlinks for more details of the test.

Alert level G

```
PLAT066_ALERT_1_G Predicted and Reported Tmin&Tmax Range Identical          ? Check
PLAT143_ALERT_4_G s.u. on c - Axis Small or Missing .............     0.00010 Ang.
PLAT153_ALERT_1_G The s.u.'s on the Cell Axes   are Equal ..(Note)     0.0001 Ang.
PLAT398_ALERT_2_G Deviating  C-O-C   Angle From 120 for O4       .      105.0 Degree
PLAT398_ALERT_2_G Deviating  C-O-C   Angle From 120 for O5       .      108.1 Degree
PLAT969_ALERT_5_G The 'Henn et al.' R-Factor-gap value ...........       3.51 Note
              Predicted wR2: Based on SigI**2  2.04 or SHELX Weight  7.07
PLAT978_ALERT_2_G Number C-C Bonds with Positive Residual Density.          7 Info
PLAT992_ALERT_5_G Repd & Actual _reflns_number_gt Values Differ by          2 Check
```

0 **ALERT level A** = Most likely a serious problem - resolve or explain
0 **ALERT level B** = A potentially serious problem, consider carefully
0 **ALERT level C** = Check. Ensure it is not caused by an omission or oversight
8 **ALERT level G** = General information/check it is not something unexpected

2 ALERT type 1 CIF construction/syntax error, inconsistent or missing data
3 ALERT type 2 Indicator that the structure model may be wrong or deficient
0 ALERT type 3 Indicator that the structure quality may be low
1 ALERT type 4 Improvement, methodology, query or suggestion
2 ALERT type 5 Informative message, check

It is advisable to attempt to resolve as many as possible of the alerts in all categories. Often the minor alerts point to easily fixed oversights, errors and omissions in your CIF or refinement strategy, so attention to these fine details can be worthwhile. In order to resolve some of the more serious problems it may be necessary to carry out additional measurements or structure refinements. However, the purpose of your study may justify the reported deviations and the more serious of these should normally be commented upon in the discussion or experimental section of a paper or in the "special_details" fields of the CIF. checkCIF was carefully designed to identify outliers and unusual parameters, but every test has its limitations and alerts that are not important in a particular case may appear. Conversely, the absence of alerts does not guarantee there are no aspects of the results needing attention. It is up to the individual to critically assess their own results and, if necessary, seek expert advice.

Publication of your CIF in IUCr journals

A basic structural check has been run on your CIF. These basic checks will be run on all CIFs submitted for publication in IUCr journals (*Acta Crystallographica*, *Journal of Applied Crystallography*, *Journal of Synchrotron Radiation*); however, if you intend to submit to *Acta Crystallographica Section C* or *E* or *IUCrData*, you should make sure that full publication checks are run on the final version of your CIF prior to submission.

Publication of your CIF in other journals

Please refer to the *Notes for Authors* of the relevant journal for any special instructions relating to CIF submission.

PLATON version of 06/01/2024; check.def file version of 05/01/2024

Datablock 15b - ellipsoid plot

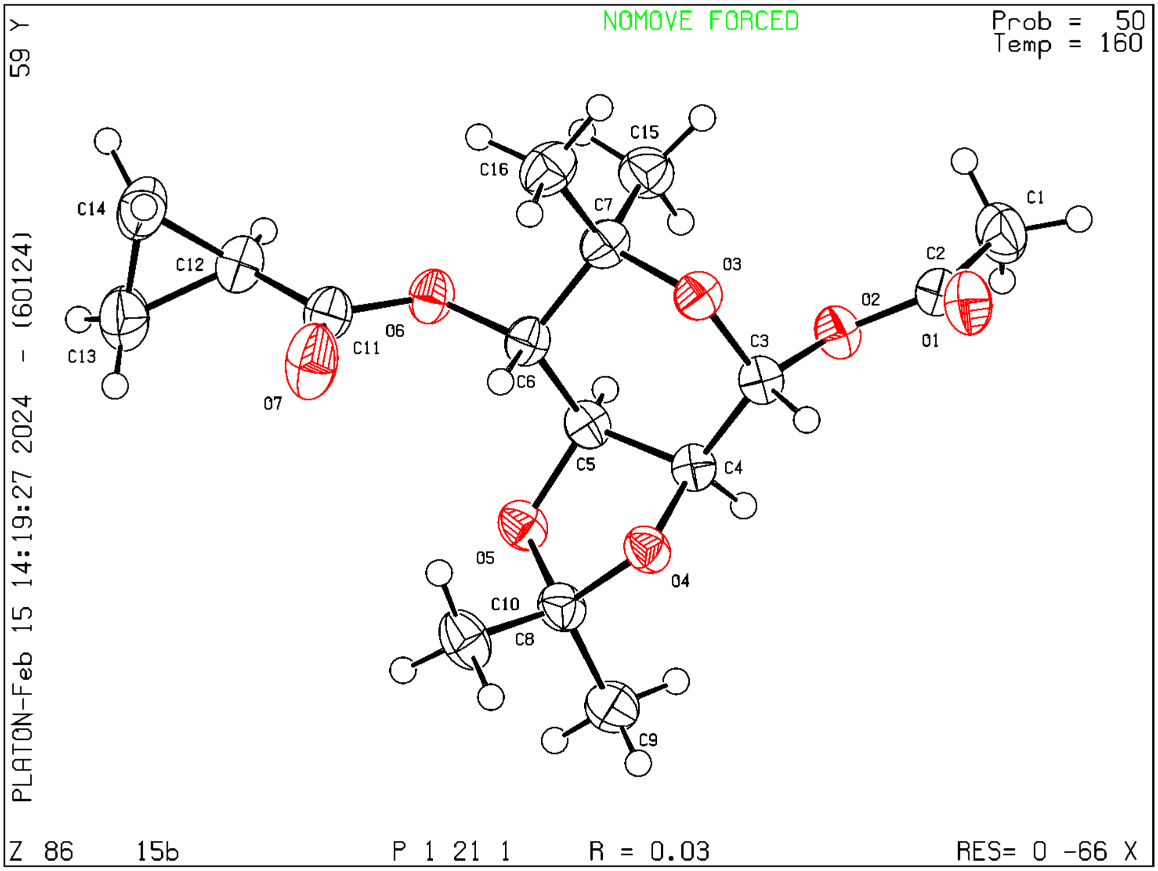

Supplement: Supplementary file 8 — au4c00206_si_008.pdf [file au4c00206_si_008.pdf]
