# Supplementary material for: Formal Single Atom Editing of the Glycosylated Natural Product Fidaxomicin Improves Acid Stability and Retains Antibiotic Activity
Source: JACS Au. 2024 May 21;4(6):2267–80. doi: 10.1021/jacsau.4c00206 (PMC11200244; doi:10.1021/jacsau.4c00206)

checkCIF/PLATON report

Structure factors have been supplied for datablock(s) 17a

THIS REPORT IS FOR GUIDANCE ONLY. IF USED AS PART OF A REVIEW PROCEDURE FOR PUBLICATION, IT SHOULD NOT REPLACE THE EXPERTISE OF AN EXPERIENCED CRYSTALLOGRAPHIC REFEREE.

No syntax errors found. CIF dictionary Interpreting this report

Datablock: 17a

Bond precision: C-C = 0.0039 A Wavelength=1.54184

Cell:	a=8.1344(2)	b=5.4669(1)	c=18.7103(4)
	alpha=90	beta=98.803(2)	gamma=90
Temperature:	160 K		

	Calculated	Reported
Volume	822.25(3)	822.24(3)
Space group	P 21	P 1 21 1
Hall group	P 2yb	P 2yb
Moiety formula	C14 H24 O5 S	C14 H24 O5 S
Sum formula	C14 H24 O5 S	C14 H24 O5 S
Mr	304.39	304.39
Dx,g cm-3	1.230	1.229
Z	2	2
Mu (mm-1)	1.889	1.889
F000	328.0	328.0
F000’	329.64	
h,k,lmax	10,6,23	10,6,23
Nref	3346[1858]	3338
Tmin,Tmax	0.893,0.963	0.742,0.971
Tmin’	0.635	

Correction method= # Reported T Limits: Tmin=0.742 Tmax=0.971
AbsCorr = ANALYTICAL

Data completeness= 1.80/1.00 Theta(max)= 74.341

R(reflections)= 0.0310(3222) wR2(reflections)= 0.0822(3338)

S = 1.045 Npar= 210

The following ALERTS were generated. Each ALERT has the format
test-name_ALERT_alert-type_alert-level.
Click on the hyperlinks for more details of the test.

Alert level C

```
PLAT220_ALERT_2_C NonSolvent   Resd 1  C   Ueq(max)/Ueq(min) Range        3.2 Ratio
PLAT222_ALERT_3_C NonSolvent Resd 1  H   Uiso(max)/Uiso(min) Range        4.1 Ratio
PLAT242_ALERT_2_C Low    'MainMol' Ueq as Compared to Neighbors of        C12 Check
```

Alert level G

```
PLAT003_ALERT_2_G Number of Uiso or Uij Restrained non-H Atoms ...           6 Report
PLAT178_ALERT_4_G The CIF-Embedded .res File Contains SIMU Records           1 Report
PLAT188_ALERT_3_G A Non-default SIMU Restraint Value has been used      0.0100 Report
PLAT301_ALERT_3_G Main Residue  Disorder ..............(Resd    1)         10% Note
PLAT398_ALERT_2_G Deviating  C-O-C   Angle From 120 for O2       .       106.8 Degree
PLAT398_ALERT_2_G Deviating  C-O-C   Angle From 120 for O3       .       108.8 Degree
PLAT860_ALERT_3_G Number of Least-Squares Restraints ............           91 Note
PLAT912_ALERT_4_G Missing # of FCF Reflections Above STh/L=  0.600           1 Note
PLAT969_ALERT_5_G The 'Henn et al.' R-Factor-gap value ..........         2.77 Note
              Predicted wR2: Based on SigI**2  2.97 or SHELX Weight  8.13
PLAT978_ALERT_2_G Number C-C Bonds with Positive Residual Density.           3 Info
```

0 **ALERT level A** = Most likely a serious problem - resolve or explain
0 **ALERT level B** = A potentially serious problem, consider carefully
3 **ALERT level C** = Check. Ensure it is not caused by an omission or oversight
10 **ALERT level G** = General information/check it is not something unexpected

0 ALERT type 1 CIF construction/syntax error, inconsistent or missing data
6 ALERT type 2 Indicator that the structure model may be wrong or deficient
4 ALERT type 3 Indicator that the structure quality may be low
2 ALERT type 4 Improvement, methodology, query or suggestion
1 ALERT type 5 Informative message, check

It is advisable to attempt to resolve as many as possible of the alerts in all categories. Often the minor alerts point to easily fixed oversights, errors and omissions in your CIF or refinement strategy, so attention to these fine details can be worthwhile. In order to resolve some of the more serious problems it may be necessary to carry out additional measurements or structure refinements. However, the purpose of your study may justify the reported deviations and the more serious of these should normally be commented upon in the discussion or experimental section of a paper or in the "special_details" fields of the CIF. checkCIF was carefully designed to identify outliers and unusual parameters, but every test has its limitations and alerts that are not important in a particular case may appear. Conversely, the absence of alerts does not guarantee there are no aspects of the results needing attention. It is up to the individual to critically assess their own results and, if necessary, seek expert advice.

Publication of your CIF in IUCr journals

A basic structural check has been run on your CIF. These basic checks will be run on all CIFs submitted for publication in IUCr journals (*Acta Crystallographica*, *Journal of Applied Crystallography*, *Journal of Synchrotron Radiation*); however, if you intend to submit to *Acta Crystallographica Section C* or *E* or *IUCrData*, you should make sure that full publication checks are run on the final version of your CIF prior to submission.

Publication of your CIF in other journals

Please refer to the *Notes for Authors* of the relevant journal for any special instructions relating to CIF submission.

PLATON version of 06/01/2024; check.def file version of 05/01/2024

Datablock 17a - ellipsoid plot

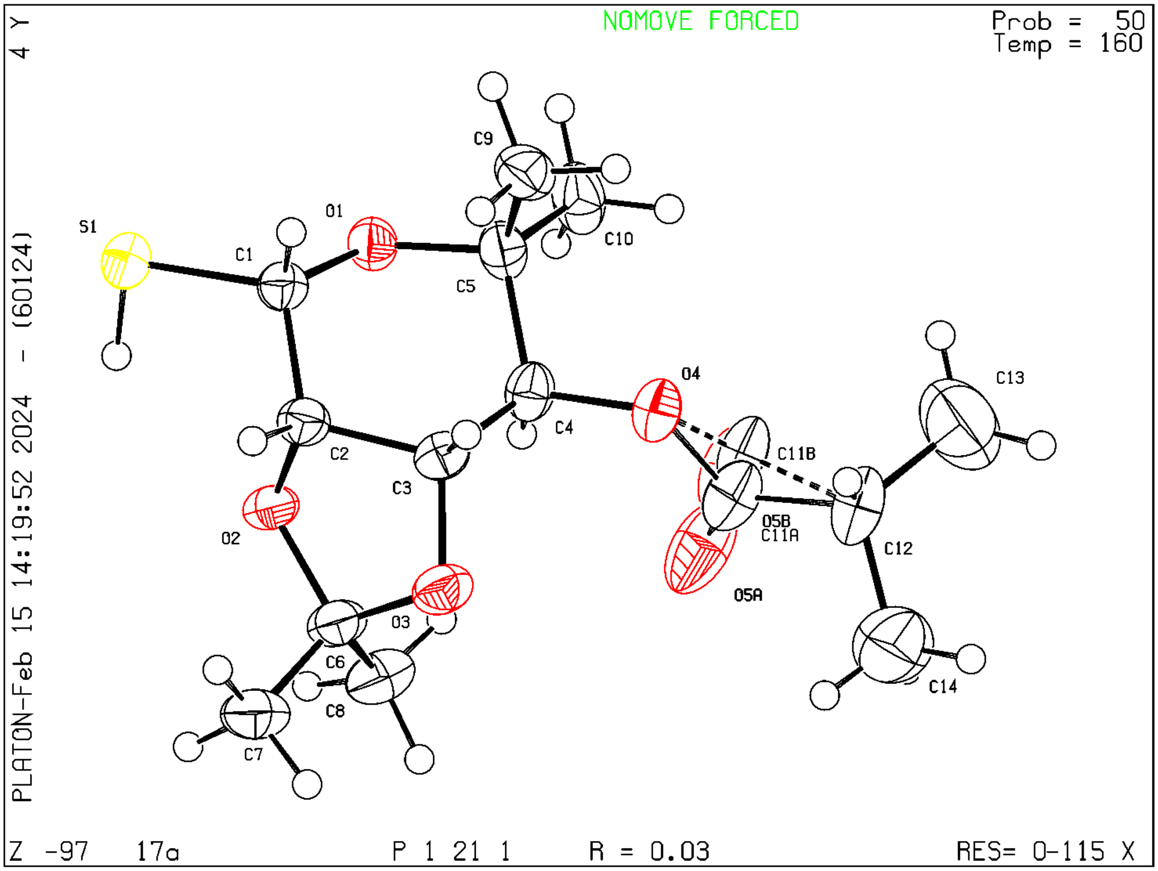

Supplement: Supplementary file 10 — au4c00206_si_010.pdf [file au4c00206_si_010.pdf]
