# Supplementary material for: Formal Single Atom Editing of the Glycosylated Natural Product Fidaxomicin Improves Acid Stability and Retains Antibiotic Activity
Source: JACS Au. 2024 May 21;4(6):2267–80. doi: 10.1021/jacsau.4c00206 (PMC11200244; doi:10.1021/jacsau.4c00206)

checkCIF/PLATON report

Structure factors have been supplied for datablock(s) 17c

THIS REPORT IS FOR GUIDANCE ONLY. IF USED AS PART OF A REVIEW PROCEDURE FOR PUBLICATION, IT SHOULD NOT REPLACE THE EXPERTISE OF AN EXPERIENCED CRYSTALLOGRAPHIC REFEREE.

No syntax errors found. CIF dictionary Interpreting this report

Datablock: 17c

```
Bond precision:   C-C = 0.0133 A          Wavelength=1.54184

Cell:            a=16.1776(9)    b=10.9250(5)       c=21.0953(14)
                 alpha=90        beta=109.647(7)    gamma=90
Temperature:     160 K

                Calculated                  Reported
Volume          3511.3(4)                   3511.3(4)
Space group     C 2                         C 1 2 1
Hall group      C 2y                        C 2y
Moiety formula  C15 H26 O5 S                C15 H26 O5 S
Sum formula     C15 H26 O5 S                C15 H26 O5 S
Mr              318.42                      318.42
Dx,g cm-3       1.205                       1.205
Z               8                           8
Mu (mm-1)       1.790                       1.790
F000            1376.0                      1376.0
F000'           1382.69
h,k,lmax        19,13,25                    19,13,25
Nref            6431[ 3398]                 6365
Tmin,Tmax       0.842,0.931                 0.722,0.939
Tmin'           0.674

Correction method= # Reported T Limits: Tmin=0.722 Tmax=0.939
AbsCorr = ANALYTICAL

Data completeness= 1.87/0.99       Theta(max)= 68.248

R(reflections)= 0.1027( 4023)                       wR2(reflections)=
                                                    0.3082( 6365)
S = 1.132                  Npar= 399
```

The following ALERTS were generated. Each ALERT has the format
test-name_ALERT_alert-type_alert-level.
Click on the hyperlinks for more details of the test.

Alert level B

```
PLAT340_ALERT_3_B Low Bond Precision on  C-C Bonds ..............    0.01333 Ang.
```

Author Response: That value reflects the medium quality of the platelet, and its weak diffraction. The large thermal parameters of the atoms in the model probably result from a twinning issue along the smallest direction but a cut was not possible. We considered that the structure deserves to be reported as characterization proof.

```
PLAT413_ALERT_2_B Short Inter XH3 .. XHn     H8B      ..H8B      .       1.91 Ang.
                                                  -x,y,1-z  =      2_556 Check
```

Alert level C

```
PLAT084_ALERT_3_C High wR2 Value (i.e. > 0.25) ..................       0.31 Report
PLAT094_ALERT_2_C Ratio of Maximum / Minimum Residual Density ....       2.97 Report
PLAT242_ALERT_2_C Low    'MainMol' Ueq as Compared to Neighbors of        C10 Check
PLAT242_ALERT_2_C Low    'MainMol' Ueq as Compared to Neighbors of        C21 Check
PLAT242_ALERT_2_C Low    'MainMol' Ueq as Compared to Neighbors of        C25 Check
PLAT260_ALERT_2_C Large Average Ueq of Residue Including        S2      0.108 Check
PLAT420_ALERT_2_C D-H Bond Without Acceptor  S1       --H1A      .     Please Check
PLAT420_ALERT_2_C D-H Bond Without Acceptor  S2       --H2       .     Please Check
PLAT906_ALERT_3_C Large K Value in the Analysis of Variance ......      3.434 Check
```

Alert level G

```
PLAT072_ALERT_2_G SHELXL First  Parameter in WGHT  Unusually Large       0.20 Report
PLAT398_ALERT_2_G Deviating  C-O-C   Angle From 120 for O2       .      107.6 Degree
PLAT398_ALERT_2_G Deviating  C-O-C   Angle From 120 for O3       .      109.4 Degree
PLAT398_ALERT_2_G Deviating  C-O-C   Angle From 120 for O7       .      107.5 Degree
PLAT398_ALERT_2_G Deviating  C-O-C   Angle From 120 for O8       .      109.5 Degree
PLAT933_ALERT_2_G Number of HKL-OMIT Records in Embedded .res File          1 Note
               -3  5  3,
PLAT969_ALERT_5_G The 'Henn et al.' R-Factor-gap value ...........       5.72 Note
              Predicted wR2: Based on SigI**2  5.39 or SHELX Weight 28.13
PLAT978_ALERT_2_G Number C-C Bonds with Positive Residual Density.          1 Info
```

0 **ALERT level A** = Most likely a serious problem - resolve or explain
2 **ALERT level B** = A potentially serious problem, consider carefully
9 **ALERT level C** = Check. Ensure it is not caused by an omission or oversight
8 **ALERT level G** = General information/check it is not something unexpected

0 ALERT type 1 CIF construction/syntax error, inconsistent or missing data
15 ALERT type 2 Indicator that the structure model may be wrong or deficient
3 ALERT type 3 Indicator that the structure quality may be low
0 ALERT type 4 Improvement, methodology, query or suggestion
1 ALERT type 5 Informative message, check

It is advisable to attempt to resolve as many as possible of the alerts in all categories. Often the minor alerts point to easily fixed oversights, errors and omissions in your CIF or refinement strategy, so attention to these fine details can be worthwhile. In order to resolve some of the more serious problems it may be necessary to carry out additional measurements or structure refinements. However, the purpose of your study may justify the reported deviations and the more serious of these should normally be commented upon in the discussion or experimental section of a paper or in the "special_details" fields of the CIF. checkCIF was carefully designed to identify outliers and unusual parameters, but every test has its limitations and alerts that are not important in a particular case may appear. Conversely, the absence of alerts does not guarantee there are no aspects of the results needing attention. It is up to the individual to critically assess their own results and, if necessary, seek expert advice.

Publication of your CIF in IUCr journals

A basic structural check has been run on your CIF. These basic checks will be run on all CIFs submitted for publication in IUCr journals (*Acta Crystallographica*, *Journal of Applied Crystallography*, *Journal of Synchrotron Radiation*); however, if you intend to submit to *Acta Crystallographica Section C* or *E* or *IUCrData*, you should make sure that full publication checks are run on the final version of your CIF prior to submission.

Publication of your CIF in other journals

Please refer to the *Notes for Authors* of the relevant journal for any special instructions relating to CIF submission.

PLATON version of 06/01/2024; check.def file version of 05/01/2024

Datablock 17c - ellipsoid plot

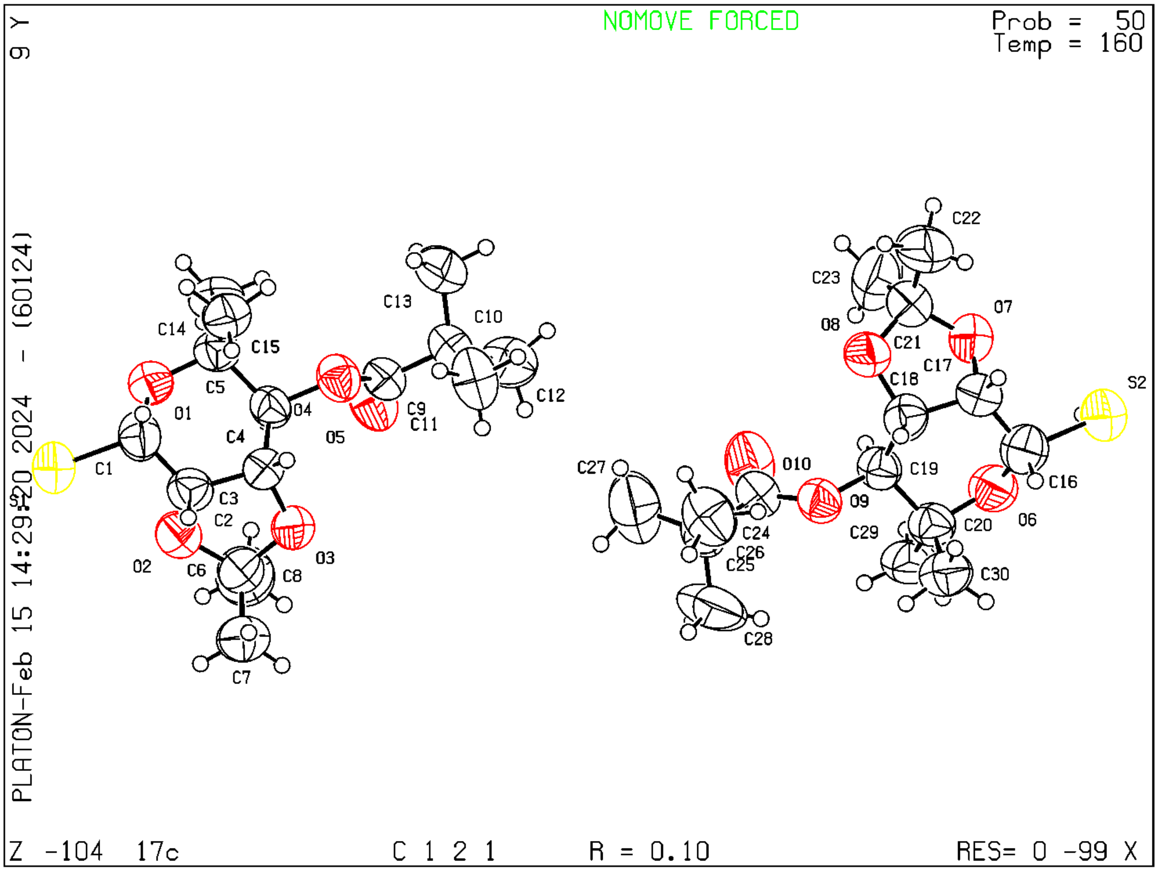

Supplement: Supplementary file 14 — au4c00206_si_014.pdf [file au4c00206_si_014.pdf]
